# Transcriptomic profiling and pathway analysis of cultured human lung microvascular endothelial cells following ionizing radiation exposure

**DOI:** 10.1038/s41598-021-03636-7

**Published:** 2021-12-20

**Authors:** Roxane M. Bouten, Clifton L. Dalgard, Anthony R. Soltis, John E. Slaven, Regina M. Day

**Affiliations:** 1 Department of Pharmacology and Molecular Therapeutics, Uniformed Services University of the Health Sciences, Bethesda, MD 20814 USA; 2 The American Genome Center, Uniformed Services University of the Health Sciences, Bethesda, MD 20814 USA; 3 Department of Anatomy, Physiology and Genetics, Uniformed Services University of the Health Sciences, Bethesda, MD 20814 USA; 4 Collaborative Health Initiative Research Program, Uniformed Services University of the Health Sciences, Bethesda, MD 20814 USA; 5 Henry M. Jackson Foundation for the Advancement of Military Medicine, Bethesda, MD 20817 USA

**Keywords:** Cell biology, Molecular biology

## Abstract

The vascular system is sensitive to radiation injury, and vascular damage is believed to play a key role in delayed tissue injury such as pulmonary fibrosis. However, the response of endothelial cells to radiation is not completely understood. We examined the response of primary human lung microvascular endothelial cells (HLMVEC) to 10 Gy (1.15 Gy/min) X-irradiation. HLMVEC underwent senescence (80–85%) with no significant necrosis or apoptosis. Targeted RT-qPCR showed increased expression of genes *CDKN1A* and *MDM2* (10–120 min). Western blotting showed upregulation of p2/waf1, MDM2, ATM, and Akt phosphorylation (15 min–72 h). Low levels of apoptosis at 24–72 h were identified using nuclear morphology. To identify novel pathway regulation, RNA-seq was performed on mRNA using time points from 2 to 24 h post-irradiation. Gene ontology and pathway analysis revealed increased cell cycle inhibition, DNA damage response, pro- and anti- apoptosis, and pro-senescence gene expression. Based on published literature on inflammation and endothelial-to-mesenchymal transition (EndMT) pathway genes, we identified increased expression of pro-inflammatory genes and EndMT-associated genes by 24 h. Together our data reveal a time course of integrated gene expression and protein activation leading from early DNA damage response and cell cycle arrest to senescence, pro-inflammatory gene expression, and endothelial-to-mesenchymal transition.

## Introduction

Exposure to thoracic irradiation, accidental or from clinical therapy, can result in Radiation Induced Lung Injury (RILI)^1–3^. Two clinical stages of RILI have been documented in human and animal models^2,4,5^. First, radiation pneumonitis, a pulmonary inflammatory response, develops, usually within ~ 4–12 weeks of exposure^2,4^. At about six to twelve months later, pulmonary fibrosis can develop, characterized by fibroblast proliferation, excessive extracellular matrix deposition, and the irreversible loss of normal alveolar architecture^2,4,6–10^. Additionally, radiation can result in pleural effusions in humans and in animal models^10^. Currently, there are no agents approved by the US Food and Drug Administration (FDA) to prevent the development of radiation pneumonitis or the subsequent fibrosis^1,11^.

Changes in the lung vasculature are shown to precede and contribute to RILI, resulting in vascular dysfunction through a variety of mechanisms^3,12,13^. Studies have suggested that microvascular endothelium, which is plentiful in the pulmonary system, is more sensitive to radiation than the endothelium of larger vessels, as the vessel walls of the microvasculature consist only of the layer of endothelial cells (ECs) and a basal lamina^14^. Within a few weeks of irradiation, changes to the endothelium in vivo leads to loss of barrier function, with narrowing and obliteration of capillaries, swelling and hypoplasia in the endothelial layer, and hypoxia in the surrounding tissue^9,15,16^. Blood flow in irradiated tissues is significantly reduced, correlating with reduced microvascular density and diminished oxygen perfusion of the tissues^9,16,17^. In vivo studies demonstrated that radiation microvascular endothelial barrier damage results in increased permeability, fluid extravasation, and lung edema^10,15^.

Chronic inflammation is believed to be a critical contributor to radiation pneumonitis and fibrotic remodeling^5,12,18^. A potential mechanism of increased inflammation is radiation-induced endothelial barrier permeability that allows the extravasation of a variety of inflammatory cells and factors as well as other macromolecules that can contribute to tissue injury. Additionally, the endothelium itself is a key modulator of inflammatory response. Radiation induces increased cytokine secretion in ECs, leading to a pro-inflammatory transcriptional program^19^. Because of the tissue hypoxia and the increased inflammatory response as a result of vascular damage, it has been suggested that mitigating damage to microvascular ECs could provide significant protection for underlying tissues from radiation damage^12,20^.

Investigations into the molecular effects of radiation have been performed to understand the mechanisms underlying the biological damage of radiation to ECs. Radiation initially induces single and double stranded DNA breaks and oxidation of biomolecules from the generation of ROS and RNS (Huang et al., 2017)^3,21^. Apoptosis, DNA damage response, cell cycle alterations, accelerated senescence, and secretory pathway (pro-inflammatory) activation are a few of the signaling pathways demonstrated to be induced in ECs by radiation and the persistent changes in oxidative stress^15,22–24^.

Recently, endothelial-to-mesenchymal transition (EndMT) was shown to precede lung fibrosis in animal models as well as patients with fibrotic lung disease^5,25,26^. EndMT is characterized by the loss of normal EC characteristics, especially loss of cell–cell junctions and increased cell motility^26^. Mesenchymal cell markers including alpha-smooth muscle actin (α-SMA), fibroblast-specific protein-1 (FSP-1), and vimentin are upregulated while standard EC markers, such as platelet endothelial cell adhesion molecule (PECAM-1) and vascular endothelial (VE)-cadherin, are reduced following irradiation^5,26,27^. Inflammatory pathways such as TGF-β and TNF-α were also demonstrated to be involved in this process^15,26,28,29^.

The time course of radiation-induced changes in total gene expression is not known in lung microvascular ECs. Here we have addressed this gap of knowledge by examining the change in transcription in cultured human lung microvascular ECs in an early time course following exposure to X-ray irradiation. The RNA-seq time course provides evidence that cascades of gene expression occur in pathways for DNA damage response, cell cycle effects, apoptosis, accelerated senescence, inflammation, and EndMT. Together these findings provide insight into the sequence of cellular events following acute radiation exposure of lung microvascular ECs.

## Methods

### Reagents

Chemicals and reagents were purchased from MilliporeSigma (St. Louis, MO, USA) except where indicated.

### Cell culture and irradiation

Human lung microvascular endothelial cells (HLMVEC) were purchased from Cell Applications (San Diego, CA, USA), cultured on plates treated with endothelial cell attachment factor in Microvascular Endothelial Cell Growth Medium (Cell Applications) in a humidified environment of 5% CO_2_/95% air at 37 °C, according to the manufacturer’s instructions. Cells were used within seven passages for all experiments. Cells were irradiated at 70–90% confluence using an RS2000 Biological Irradiator (Rad Source Technologies, Alpharetta, GA, USA) at a dose rate of 1.15 Gy/min (160 kV, 25 mA) for a total dose of 10 Gy as previously described^24,30^.

### Senescence-associated beta-galactosidase (SA-β-gal) assay and nuclear morphology

Cells were irradiated at 70–90% confluence and assayed at 24, 48, and 72 h post-irradiation in triplicate. Dishes were washed twice with phosphate buffered saline (PBS), and fixed with 3.7% formaldehyde in PBS for 5 min at room temperature, washed twice more with PBS, treated with X-gal solution [1 mg/ml 5-bromo-4-chloro-3-indoyl β-galactopyranoside, 150 mM NaCl, 2 mM MgCl2, 5 mM K3Fe(CN)_6_, 5 mM K_4_Fe(CN)_6_, citric acid/sodium phosphate buffer (pH 6)], and incubated at 37 °C for 20 h without CO_2_. Cells were then washed with PBS, treated for 2 min with methanol, and air dried. At least 100 cells were scored in three random fields for expression of β-galactosidase; all cells in each field were scored. Imaging was performed on an Olympus IX73 fluorescence microscope (Olympus, Center Valley, PA) using × 10 magnification at 488 nm or using phase contrast. For nuclear morphology analysis, cells were grown on coverslips and irradiated at 70–90% confluence. At the required time points, cells were washed with PBS and fixed for 5 min in buffered formalin at room temp. Cells were then washed again with PBS, and then mounted on slides in Prolong Gold antifade reagent with DAPI (Invitrogen, Eugene, OR, USA). Cells were imaged as for SA-β-gal. All nuclei were analyzed in 5 random images, to allow counting of at least 100 nuclei per slide, with three slides per time point.

### Lactate dehydrogenase assay

HLMVEC were grown to 70–90% confluence and 5000–6000 cells per sample were irradiated or sham-irradiated, and assayed using the Invitrogen CyQUANT LDH Cytotoxicity Assay Kit (Thermo Fisher Scientific, Eugene, OR, USA) according to the manufacturer’s product information sheet. Plates were read using a Cytation 5 imaging plate reader (BioTek, Winooski, VT, USA). Assays were performed in triplicate.

### Quantitative PCR analysis

HLMVEC were irradiated at 70–90% confluence, and total RNA was isolated from HLMVEC using the RNeasy Mini Kit with on-column DNase digestion (Qiagen, Valencia, CA, USA) according to manufacturer’s protocol. RNA was quantified spectroscopically (ND-1000 Spectrophotometer, Nano-Drop, Wilmington, DE, USA) and 1.0 µg was reverse transcribed using iScript cDNA synthesis kit (Bio-Rad, Hercules, CA, USA), according to the manufacturer’s protocol. Complementary DNA (cDNA) was diluted tenfold with nuclease-free water and 2 µl was used in each 20 µl RT-qPCR reaction. RT-qPCRs were performed with technical triplicates using 6 μM of each primer and 10 µl of iTaq™ Universal SYBR Green Supermix (Bio-Rad), on a CFX96 Touch Real-Time PCR Detection System (Bio-Rad). Primers for qRT-PCR were designed using NCBI /Primer-BLAST and purchased from Integrated DNA Technologies (Coralville, IA, USA). Forward and reverse primer sequences are shown in Table 1. Relative gene expression to the reference genes was calculated using the ΔΔCq method using CFX Maestro software, 2.0 (Bio-Rad)^31,32^.

**Table 1:** Primers for qPCR.

HGNC gene symbol	Forward Primer	Reverse Primer
ACTA2	5′-TATCCCCGGGACTAAGACGGG-3′	5′-CAGAGCCCAGAGCCATTGTC-3′
ATM	5′-AGTGGGACCATTGCACTTCC-3′	5′-CAAGGCTGCGCTTACACATC-3′
BBC3	5′-GACGACCTCAACGCACAGTA-3′	5′-TAATTGGGCTCCATCTCGGG-3′
BCL2A1	5′-AAATTGCCCCGGATGTGGATA-3′	5′-TGGGCCACTGACTCTACCAG-3′
CCL2	5′-GATCTCAGTGCAGAGGCTCG-3′	5′-TTTGCTTGTCCAGGTGGTCC-3′
CCND2	5′-GTGCTGGGGAAGTTGAAGTG-3′	5′-GATCATCGACGGTGGGTACA-3′
CDKN1A	5′-ACTCTCAGGGTCGAAAACGG-3′	5′-GATGTAGAGCGGGCCTTTGA-3′
CDKN2B	5′-CAACGGAGTCAACCGTTTCG-3′	5′-ACATCGGCGATCTAGGTTCC-3′
E2F1	5′-CCGGGGAATGAAGGTGAACA-3′	5′-GAGCAAAAGGGCCGAAAGTG-3′
ESPL1	5′-TCCTGCTGCTACGGATTGTC-3′	5′-CGAGATGCTTCAGGCTCGAT-3′
FAS	5′-CTGTGACCCTTGCACCAAATG-3′	5′-GACAAAGCCACCCCAAGTTAG-3′
FLT1	5′-TCACTCAGCGCATGGCAATA-3′	5′-CTCTCCTTCCGTCGGCATTT-3′
GAPDH	5′-AGCCACATCGCTCAGACAC-3′	5′-GCCCAATACGACCAAATCC-3′
HEY1	5′-GGCTCTAGGTTCCATGTCCC-3′	5′-CCTTGCTCCATTACCTGCTTC-3′
HIPK2	5′-TCCCCGTTGCCATGAACC-3′	5′-ACCCAGTCATGTCCCAGTTG-3′
ICAM1	5′-ACCCCGTTGCCTAAAAAGGA-3′	5′-GGGTAAGGTTCTTGCCCACT-3′
IGF1R	5′-CCGATGTGTGAGAAGACCAC-3′	5′-GTGGCAGCACTCATTGTTCT-3′
IGFBP3	5′-TGCTAGTGAGTCGGAGGAAGA-3′	5′-CAACTTTGTAGCGCTGGCTG -3′
IL1A	5′-GGGAGTCATTTCATTGGCGT-3′	5′-TGGAGTGGGCCATAGCTTACA-3′
LIF	5′-CCTCTGAAGTGCAGCCCATA-3′	5′-GTTGTGACATGGGTGGCGTA-3′
MDM2	5′-TGGTGAACGACAAAGAAAACG-3′	5′-GTAACTTGATATACACCAGCATCAA-3′
MTOR	5′-CAAATGTGTGCAGTTCCTGCC-3′	5′-CAAAGGACACCAACATTCCCA-3′
ORC1	5′-CATACCCTCACGAAGGTGCC-3′	5′-CAGCAGAAACATGCAGCCTC-3′
PIK3CA	5′-GAGGTTTGGCCTGCTTTTGG-3′	5′-GGTCGCCTCATTTGCTCAAC-3′
PIK3CB	5′-GATGCCCTTCTGAACTGGCT-3′	5′-GTCAATGTGGAAGAGCTGGC-3′
PIK3CD	5′-CTTCCTCCACCTCTTTGCCC-3′	5′-TCCTCTGTTTTCCCCAGTGC-3′
PIK3CG	5′-TGATCTGCGCCAAGACATGC-3′	5′-ATTGTCGTGGCGTCTTTCAC-3′
RAD9A	5′-AGCCCTTTTCCCAGAGTTACA-3′	5′-GCAGCATTTTTCCACCGTCTT-3′
RASSF5	5′-TAAGCGGATACACAAGGACGG-3′	5′-GTTCAGGGATGGAGAAGGCAT-3′
RPS6KB1	5′-GATTTATTGGCAGCCCACG-3′	5′-GCTTCCCCACTCATTGTCAC-3′
SESN1	5′-GGCGTACACGGCCCCTTT-3′	5′-GGATGAATCTGCTTGGTCCCT-3′
SIRT1	5′-GCAGATTAGTAGGCGGCTTG-3′	5′-TCTGGCATGTCCCACTATCAC-3′
TP53	5′-GACACGCTTCCCTGGATTG-3′	5′-TCAGGAAGTAGTTTCCATAGGT-3′
TRAF1	5′-CCTTGAGGTCACCCAGACAC-3′	5′-CTGGCTTGTGTGGTTCAACG-3′
TUBULIN	5′-CTCCATCCTCACCCACAC-3′	5′-CAGGGTCACATTTCACCATCT-3′
VIM	5′-GGACCAGCTAACCAACGACA-3′	5′-AAGGTCAAGACGTGCCAGAG-3′

### Western blotting

HLMVEC were irradiated at 70–90% confluence. Cells were lysed in RIPA buffer (1% NP-40, 0.1% SDS, 0.1% Na-deoxycholate, 10% glycerol, 0.137 M NaCl, 20 mM Tris pH [8.0]) (Thermo Fisher Scientific), protease (#A32953, Thermo Fisher) and phosphatase (#A32957, Thermo Fisher Scientific) inhibitor cocktails for 20 min at 4 °C, vortexed, rotated at 4 °C for 20 min, then centrifuged at 15,000 × *g* for 15 min. Protein concentrations were determined using the BCA protein assay (MilliporeSigma). Clarified lysates were boiled in SDS sample buffer containing 100 mM DTT for 5 min prior to resolution by sodium dodecyl sulfate–polyacrylamide gel electrophoresis (Criterion TGX precast, Bio-Rad). Proteins were transferred to nitrocellulose membranes (MilliporeSigma). Proteins were identified using primary antibodies: anti-p53 (Santa Cruz Biotechnology [Santa Cruz, CA, USA] #sc-256; 1:1000; or Cell Signaling [Danvers, MA, USA] #2527; 1:1000), anti-insulin-like growth factor 1 receptor (IGF1R; Cell Signaling #3027; 1:500), anti-phospho-IGF1R Tyr980 (Cell Signaling #4568; 1:500), anti-β-actin (Sigma #AC-15; 1:5,000), anti-mouse double minute 2 homolog (MDM2; Cell Signaling #86934; 1:1000), anti-ataxia telangiectasia mutated (ATM, Cell Signaling #4267; 1:1000), anti-phospho-ATM Ser1981 (Cell Signaling 13050; 1:1000), anti-p21 (waf1) (Cell Signaling #2947; 1:1000), anti-phospho-AKT Ser473 (Cell Signaling 4060; 1:1000), anti-AKT (Cell Signaling #2920; 1:1000), and anti-cleaved caspase 3 (Cell Signaling #9661; 1:500). Anti-mouse and anti-rabbit secondary antibodies conjugated to IRDye680 or IRDye800 (LI-COR, Lincoln, NE, USA; 1:10,000) were used to probe primary antibodies. Western blot protein bands were detected and quantified using the Odyssey system (LI-COR). For quantification, samples were normalized to β-actin.

### Transcriptome profiling by RNA sequencing

HLMVEC were irradiated at 70–90% confluence. Total RNA was isolated from cells using the RNeasy Mini Kit with on-column DNase digestion (Qiagen) according to manufacturer’s protocol. RNA was quantified spectroscopically (ND-1000 Spectrophotometer, Nano-Drop, Wilmington, DE, USA). The total RNA integrity was assessed using automated capillary electrophoresis with a Fragment Analyzer (Roche, Pleasanton, CA, USA). For all samples with an RNA quality indicator (RQI) > 8.0, a total of > 75 ng RNA was used as the input for library preparation using the TruSeq Stranded mRNA Library Preparation Kit (Illumina, San Diego, CA, USA). The sequencing libraries were quantified by Real-Time PCR on a Roche LightCycler 480 Instrument II using a KAPA Library Quantification Kit for NGS (Kapa, Wilmington, MA, USA). The size distribution was assessed by automated capillary-based gel electrophoresis with a Fragment Analyzer to confirm absence of free adapters or adapter dimers. The sequencing libraries were pooled and sequenced on a NovaSeq 6000 Sequencer (Illumina) using a NovaSeq 6000 SP Reagent Kit (300 cycles) within one flowcell lane using an XP workflow with 101 + 8 + 8 + 101 cycle parameters with paired-end reads of 75 bp in length. Raw sequencing reads were demuxed using bcl2fastq2 (v2.20) and aligned to the human reference genome (hg38) with MapSplice (v2.2.2)^33^. Gene-level quantification was performed with HTSeq (v0.9.1)^34^ against GENCODE (v28) basic gene annotations. Read alignment statistics and sample quality features were calculated with Samtools and RSeQC^35–37^. Sequencing quality was verified by manual inspection of sample-wise characteristics: total reads, mapping percentages, pairing percentages, transcript integrity number (TIN), 5′ to 3′ gene body read coverage slopes, and ribosomal RNA content^38^. The transcript abundance quantitation data were deposited in the NCBI Gene Expression Omnibus (GSE179810). Time-series differential expression analysis was performed with DESeq2 (v1.16.1)^39^ on raw gene counts using a likelihood-ratio test (LRT) framework, whereby a full model including stimulation time information was compared against a reduced model including only an intercept term. We defined significant time series differentially expressed genes (DEGs) as those with an LRT False Discovery Rate (FDR) q-value < 0.05, an absolute fold change > 1.5 (i.e. |log2 (fold-change)|> 0.585) at one or more time points compared to controls, and mean transcripts per million (TPM) ≥ 1 across samples.

### Gene ontology, pathway enrichment analysis, and heat map construction

Gene Ontology (GO) and Kyoto Encyclopedia of Genes and Genomes (KEGG) pathway analyses were performed using the Database for Annotation, Visualization, Integrated Discovery (DAVID), version 6.8. with the medium classification stringency, an enrichment threshold of 0.05, and the Bonferroni method of adjustment for multiple testing (Laboratory of Human Retrovirology and Immunoinformatics, Frederick, MD, USA^40^; https://david.ncifcrf.gov/)^41^, g:Profiler (https://biit.cs.ut.ee/gprofiler/)^42^; and Gene Ontology enRIchment anaLysis and visuaLizAtion tool (GOrilla), version 4.1 (http://cbl-gorilla.cs.technion. ac.il/)^43,44^*.* Heatmaps were generated using ClustVis software (https://biit.cs.ut.ee/clustvis/)^45^. Venn diagrams were constructed using Venny, version 2.1 (Juan Carlos Oliveros, BioInfoGP Service, Centro Nacional de Biotecnologia, Madrid, Spain; https://bioinfogp.cnb.csic.es/tools/venny/). The summary figure for pathway interconnections was generated using Metascape (https://metascape.org^46^).

### Statistics

Statistical analyses of assays were performed using Graphpad Prism 7 (San Diego, CA, USA) or Excel. For RNA-seq and qPCR analysis, one way ANOVA with a post-test analysis was used for comparing multiple data sets. For western blot analysis, two-way ANOVA with either Tukey’s or Sidak’s post-hoc tests for multiple comparisons.

## Results

### Accelerated senescence observed in endothelial cells after 10 Gy X-irradiation

**Figure 1:**
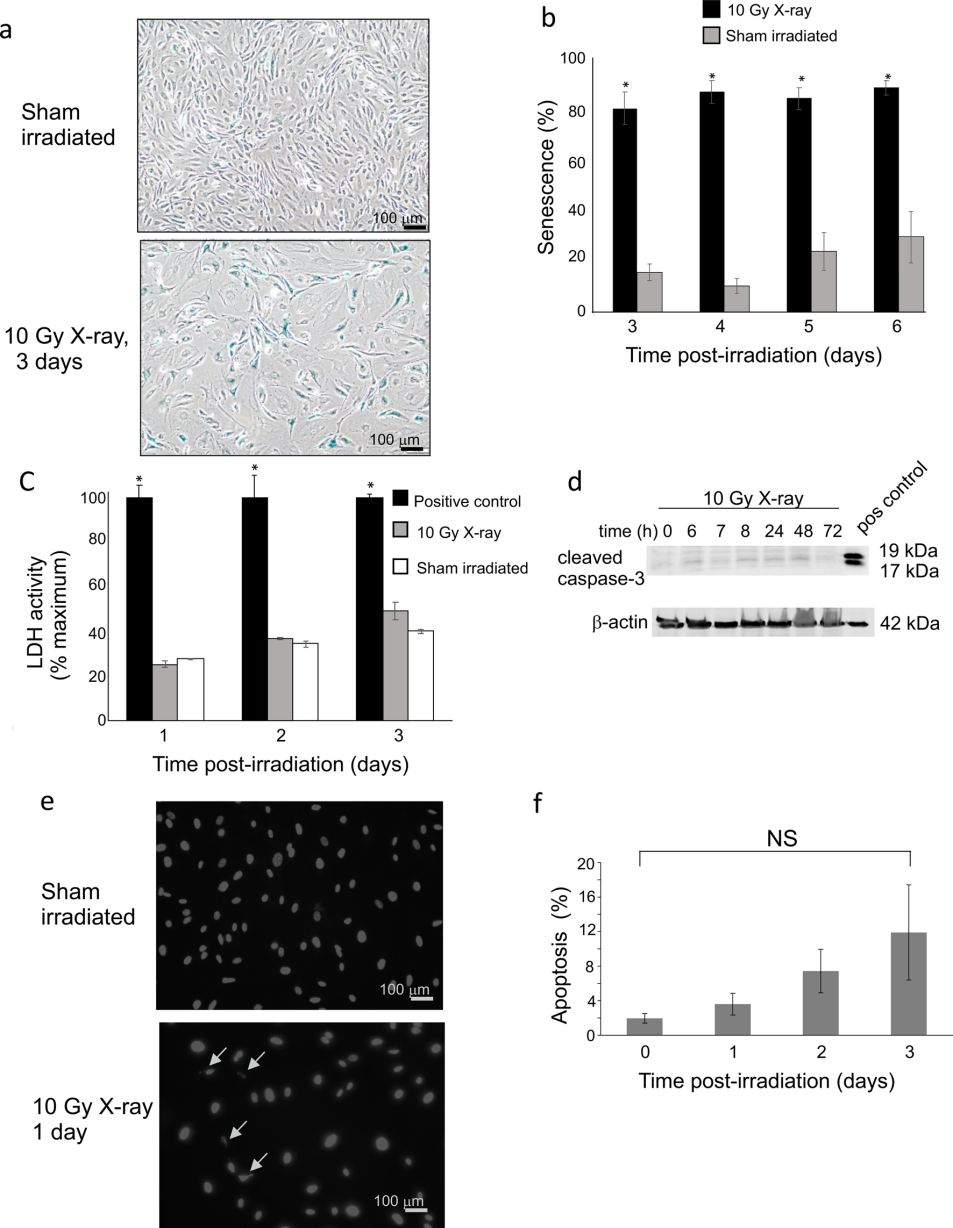
10 Gy X-irradiation induces accelerated senescence, but not necrosis or apoptosis in HMLVEC. HLMVEC were grown to 70% confluence and sham irradiated or X-irradiated at 10 Gy. (**a**) Cells were stained for SA-β-gal activity at indicated times post-irradiation, and scored for SA-β-gal staining and senescent morphology. Representative images of SA-β-gal staining and cell morphology in control and irradiated HMLVEC. (**b**) All cells were counted in three random fields per dish (minimum 100 cells per field). The graph indicates means $\pm$ SE *n* = 6; * *p* < 0.05 from sham irradiated. (**c**) LDH released into the medium was measured at 24, 48, and 72 h post-irradiation. Cell death by necrosis is expressed as a percentage of LDH in the medium of irradiated or sham irradiated cells divided by the LDH released in medium of positive control treated cells. Bars indicate mean $\pm$ standard error, *n* = 3. * *p* < 0.05 from sham irradiated. (**d**) HLMVEC were grown to 70% confluence and X-irradiated at 10 Gy. Cell lysates were prepared at the indicated time points, and western blots were performed for cleaved caspase 3 as an indication of apoptotic signaling. A positive control is provided for cleaved caspase 3 (pos control). (**e**) Cells stained with DAPI to visualize nuclear morphology to identify apoptotic nuclei. (**f**) Nuclei were scored from random fields to determine percentage of apoptotic nuclei; graph shows average of percent apoptosis ± SEM; NS = not significant.

We previously reported that the primary response of human pulmonary artery endothelial cells (HPAEC) to 10 Gy X-irradiation was accelerated senescence^24,30^. However, it was postulated that ECs from large vessels have different responses to radiation compared with microvascular ECs^14^. Therefore, we investigated the effects of radiation on human lung microvascular endothelial cells (HLMVEC) exposed to 10 Gy X-irradiation. Accelerated senescence, necrosis, and apoptosis were measured. Senescence was significantly increased 24–72 h (Fig. 1a–f). Senescent cells displayed characteristic “fried egg” morphology with expression of senescence-associated beta galactosidase (Fig. 1a,b). Cells did not display significant necrosis, as indicated by lactate dehydrogenase release in the medium in response to radiation from 24, 48, and 72 h (Fig. 1c). We were unable to detect significant apoptosis using western blotting for cleaved caspase-3 (Fig. 1d). We next performed nuclear morphology analysis to detect low levels of apoptosis occurring over the time course (Fig. 1e,f). Nuclear blebbing is consistent with late apoptotic events. We observed a trend toward 3.6–11.9% apoptosis at 24–72 h post-irradiation, respectively, although these levels did not reach significance due to variation between experiments (Fig. 1f).

**Figure 2:**
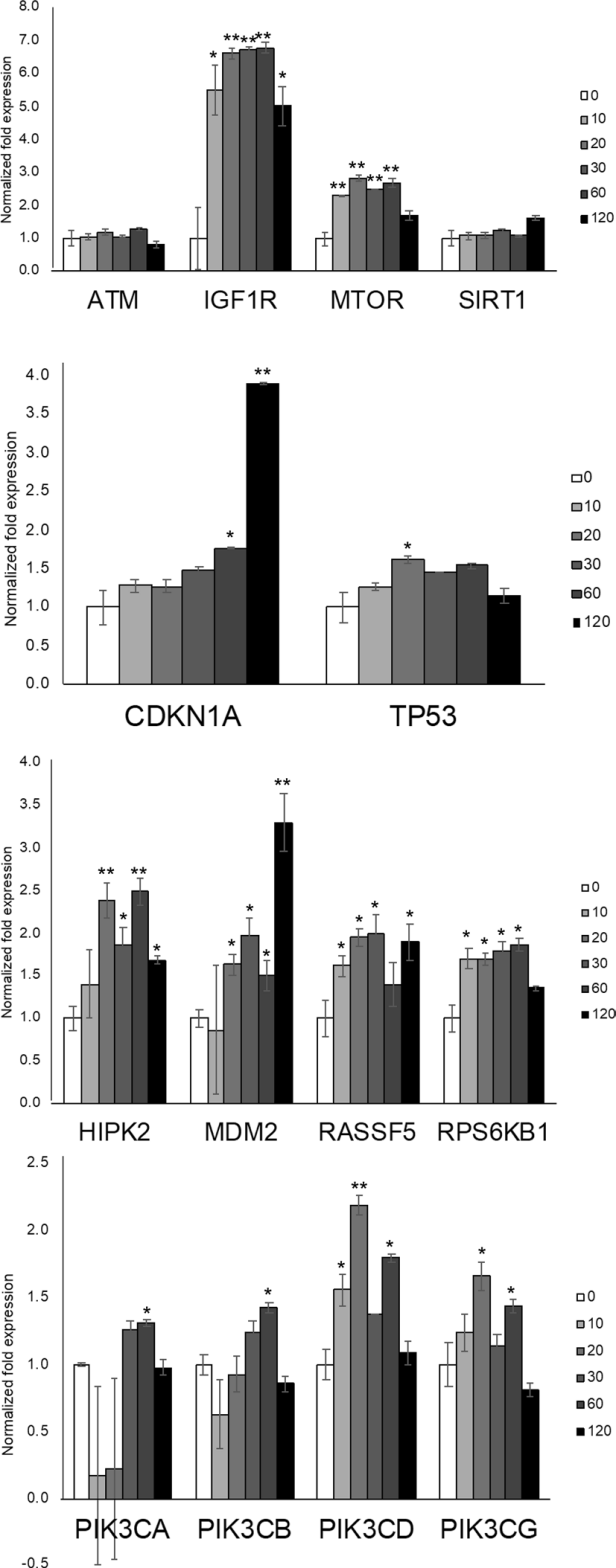
Gene expression changes in irradiated HLMVEC. HLMVEC were grown to 70% confluence and exposed to 10 Gy X-ray irradiation. RNA was obtained at the indicated time points. mRNA levels in irradiated HLMVEC were assessed by RT-qPCR at indicated time points post-irradiation. Graph represents means, ± SEM from n = 3 independent experiments. *p* < 0.05 is indicated by *, *p* < 0.01 is indicated by **.

Our laboratory and others demonstrated the regulation of a variety of signaling pathways in vitro response to radiation, including pathways regulating growth regulation, DNA repair, and cell death^24,30,47^. We investigated alterations in HLMVEC mRNA changes in an early time course (10–120 min) following 10 Gy X-ray irradiation exposure (Fig. 2). The most rapid change was in *IGF1R* mRNA, which increased significantly at all time points tested with maximum expression (~ 5–6.5-fold, *p* < 0.05) from 10 min through 2 h post-irradiation. Cyclin dependent kinase inhibitor 1A (*CDKN1A*) increased by fourfold, and murine double minute 2 (*MDM2*), an E3 ubiquitin ligase regulator of p53, increased ~ threefold, both at 2 h (both *p* < 0.05). Phosphatidylinositol 3-kinase (*PI3K*) proteins integrate cell growth in response to stress. The *PIK3CD* isoform of *PI3K* was increased at 20 min post-irradiation (*p* < 0.05). We also observed trends toward increased expression in several stress response and survival genes: homeodomain interacting protein kinase-2 (*HIPK2*), mammalian target of rapamycin (*MTOR*), Ras association domain family member (*RASSF5*), and ribosomal protein S6 kinase B1 (*RPS6KB1*)^48,49^. Interestingly, we observed no significant changes gene expression for genes encoding proteins associated with DNA repair, stress response, or cell growth regulation: the DNA damage response serine/threonine kinase ataxia-telangiectasia mutated (*ATM*) gene; the transcription factor tumor protein p53 (*TP53*), which regulates senescence-associated genes; and sirtuin 1 (*SIRT1*), which is activated by oxidative stress and DNA damage to regulate survival.

**Figure 3:**
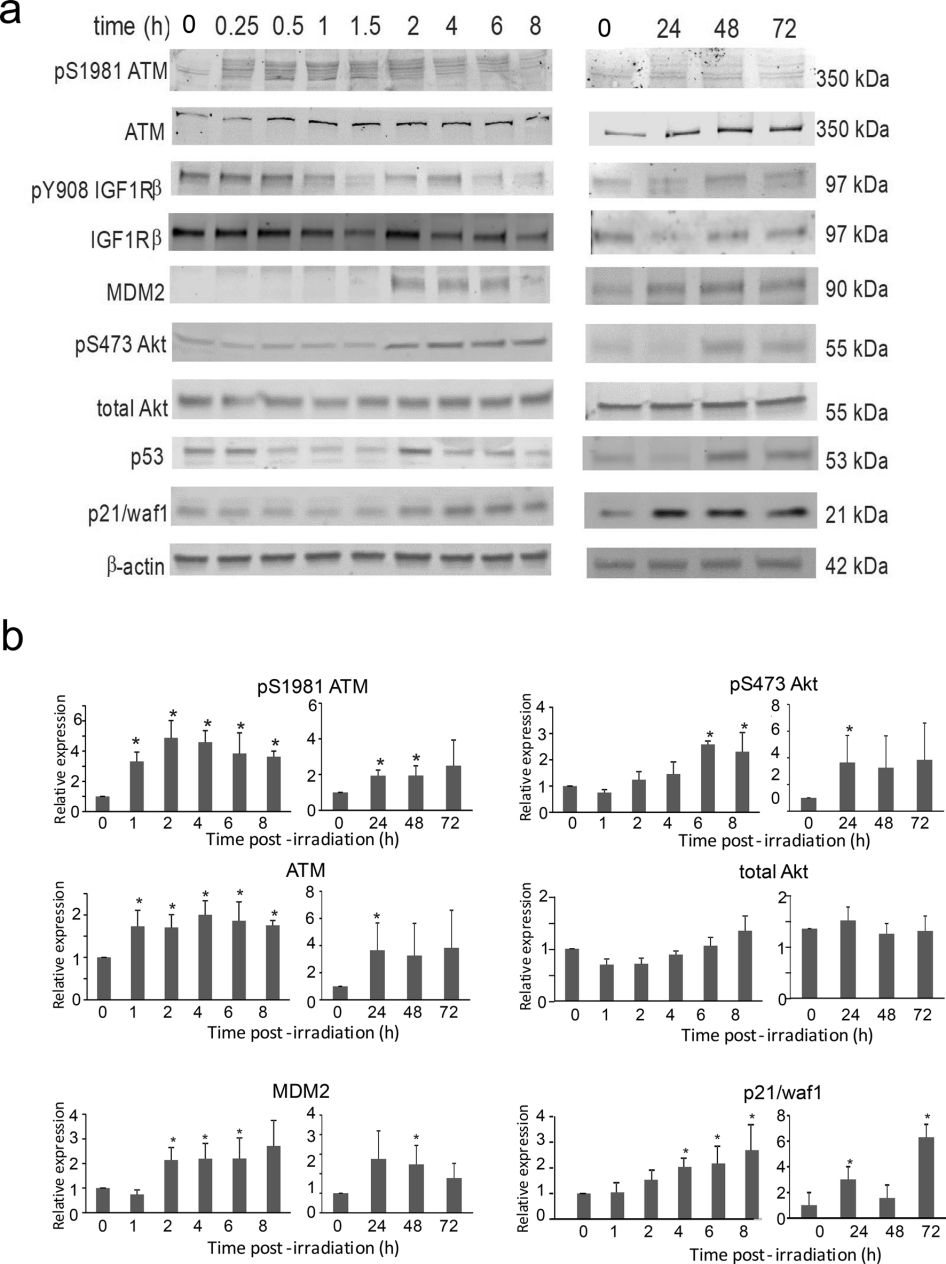
Changes in protein levels and phosphorylation in irradiated HLMVEC. HLMVEC were grown to 70% confluence and exposed to 10 Gy X-ray irradiation. Protein lysates were prepared at the indicated time points and used for western blotting for the indicated proteins and phospho-proteins. (**a**) Representative blots are shown from 3 independent experiments for (from top to bottom) pS1981 ATM, total ATM, pY908 IGF1Rβ, total IGF1Rβ, MDM2, pS473 Akt, total Akt, p53, p21/waf1, and β-actin. (**b**) Densitometry was performed for pS1981 ATM, total ATM, pS473 Akt, total Akt, MDM2, and p21/waf1. Graphs indicate means, normalized to β-actin protein levels, ± SEM from 3 independent experiments. * indicates *p* < 0.05.

Western blotting was performed to examine the regulation of selected proteins by radiation in a time course from 15 min through 72 h (Fig. 3). Consistent with the RT-qPCR findings, we observed an upregulation of p21/ waf1, (gene *CDKN1A*) and MDM2 within 2 h post-irradiation that was sustained through 72 h. Phosphorylated Akt, often a surrogate of PI3K activation, was increased at 2 h post-irradiation, consistent with the time point for upregulation of PI3KCD mRNA. Total and phosphorylated ATM were increased within 15 min, consistent with the role of ATM in DNA damage response. Increase in phosphorylated ATM was sustained through 4 h, but the increase in total ATM was maintained through 72 h. The level of p53 and IGF1R displayed trends of reduced levels at early time points, that returned to basal levels ~ 2 h post-irradiation. We unable to observe significant increases in IGF1R phosphorylation, but this may be because cells were not synchronized prior to irradiation.

### Genome-wide transcriptional responses to 10 Gy X-irradiation

Targeted gene expression analysis provides information regarding known genes with predicted functions in biological processes. To discover previously unidentified gene regulation, and to expand known pathways in primary HLMVEC in response to ionizing radiation, we used comprehensive transcriptome profiling by RNA sequencing (RNA-seq). Gene expression profiles from sham-irradiated (control) HLMVEC were compared with irradiated HLMVEC at 2, 4, 6, 8, and 24 h. Comparative differential expression analysis identified differential gene expression of 9,151 transcripts over all time points, with a q-value < 0.05 (Supplemental Table S1). In order to interpret the functional relationship of candidate DEG in response to ionizing radiation, we filtered for coding transcripts that were differentially expressed in excess of 1.5-fold as compared to the control group. Based on these criteria, we observed 3,581 unique significant DEGs in the irradiated cells irradiated vs the control cells, irrespective of time point (Supplemental Table S1).

**Figure 4:**
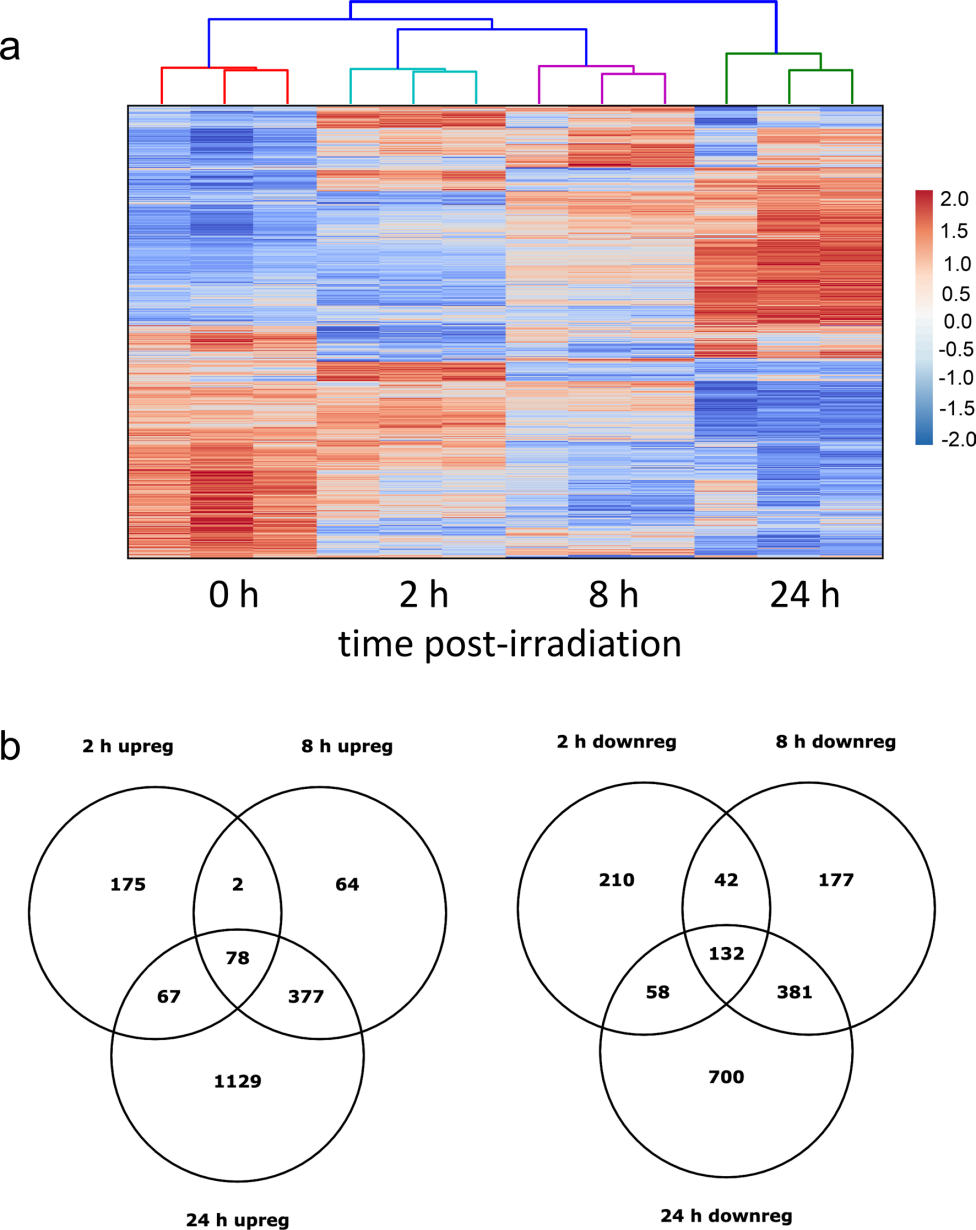
Clustering and distribution of differentially expressed genes (DEG). HLMVEC were grown to 70% confluence and exposed to 10 Gy X-ray irradiation. RNA was prepared at 2, 4, 6, 8 and 24 h post-irradiation and used for RNA-seq. (**a**) Heat map indicates distinct gene expression patterns at each time point post-irradiation. Controls are non-irradiated samples. Included are genes differentially regulated over the 24 h time course, q < 0.05. (**b**) Venn diagrams illustrate overlap of the number of DEG expressed at each time point for upregulated or downregulated genes, q < 0.05, fold change > 1.5. Data was generated using RNA from three independent experiments.

The differences in gene expression patterns post-irradiation compared to controls were very robust at 2, 8 and 24 h, indicating significant gene expression activity at these time points (Fig. 4a). Because of this we focused on these three time points for our initial analysis to gain an overall picture of changes in gene expression, and later considered all of the time points in targeted biological pathway analysis. Two Venn diagrams of the data from 2, 8 and 24 h illustrate the overlap of the differentially expressed genes up- and down-regulated in response to 10 Gy irradiation (Fig. 4b). At 2 and 8 h post-irradiation, downregulated genes outnumbered upregulated genes, with 442 and 732 genes downregulated at 2 and 8 h respectively and 322 and 521 upregulated, respectively. At 24 h post-irradiation the number of DEGs upregulated vs downregulated were reversed with 1,651 genes upregulated and 1,271 genes downregulated. mRNA expression levels of selected genes were analyzed using real-time qPCR. We selected genes (*ACTA2, BBC3, BCL2A1, CCNE2, E2F1, ESPL1, FAS, IGFBP3, ORC1, SESMN1,* and *VIM*) that were highly regulated in our sequencing data and represented in the six biological pathways we identified as affected by 10 Gy X-irradiation (cell cycle, apoptosis, DNA damage, inflammatory response, senescence, and EndMT). The results from RT-PCR correlated well in the direction of regulation with the sequencing-derived mRNA expression levels. Overall, the RNA-seq results were in good agreement with the qPCR, although there were a few instances in which the results diverged (Supplemental Fig. S1). The 2 h time point provided most of the differences, with expression measured using qPCR higher than RNA-seq. In many of these cases, the qPCR expression is also more variable than the RNA-seq results. The other three times points do not show significant differences suggesting the issue was in the samples or execution of the qPCR at one time point.

### Gene ontology and pathway analysis post-irradiation

**Figure 5:**
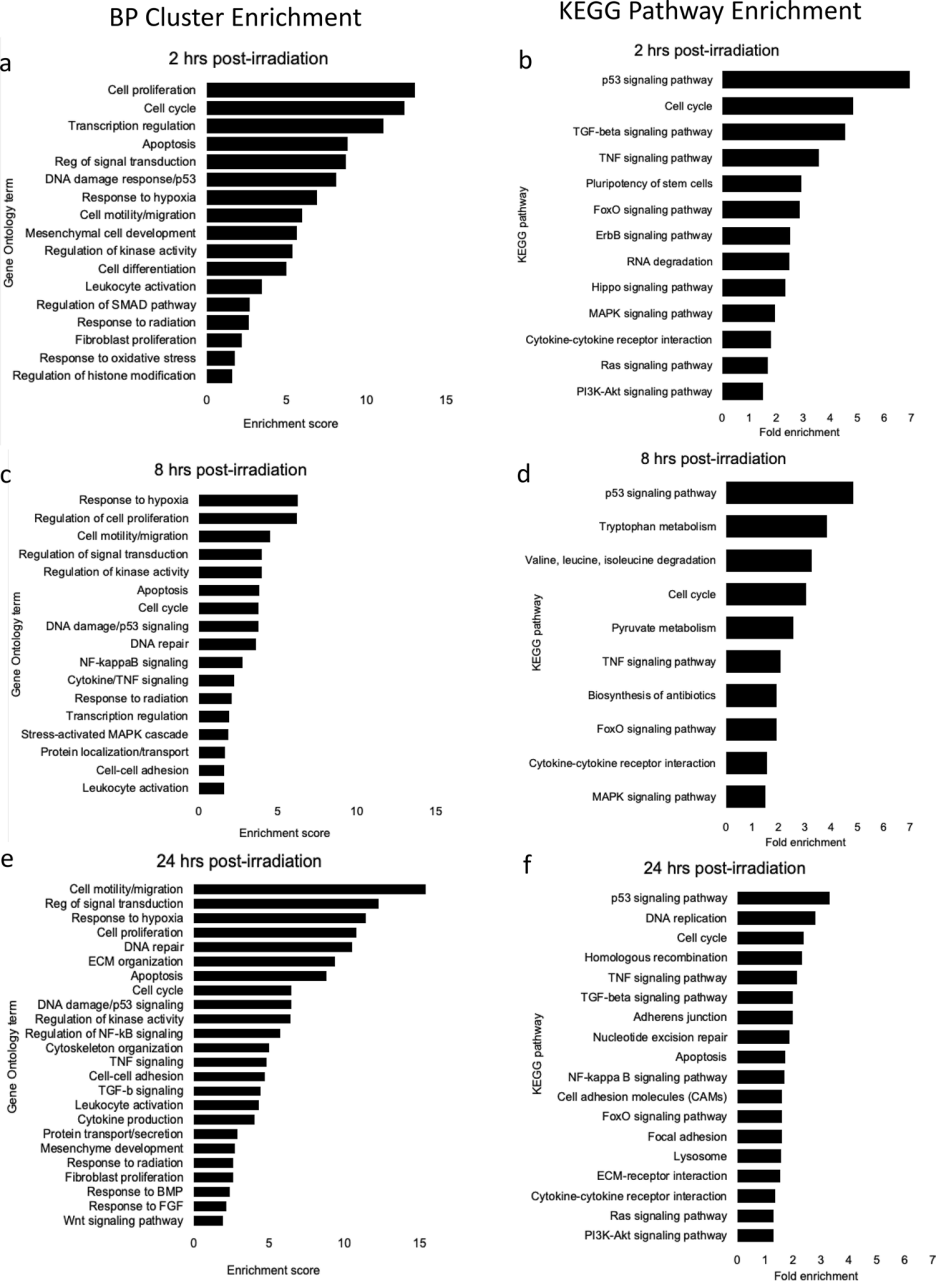
GO term cluster and KEGG pathway enrichment analyses of differentially expressed genes in HLMVEC following 10 Gy X-irradiation. HLMVEC were grown to 70% confluence and exposed to 10 Gy X-ray irradiation. RNA was prepared and used for RNA-seq. (**a**,**b**) 2 h post-irradiation. (**c**,**d**) 8 h post-irradiation. (**e**,**f**) 24 h post-irradiation. DAVID (https://david.ncifcrf.gov) software was used for functional annotations. DEG > 1.5-fold, q < 0.05. The enrichment is shown for both up- and down-regulated pathways. Data was generated using RNA from three independent samples.

To identify enrichment of pertinent gene ontology (GO) term clusters, we analyzed 2, 8 and 24 h gene sets using DAVID (v 6.8)^40,41^. Analyses were focused on GO terms and KEGG pathways relevant to cellular response pathways and to the endothelium. The biological processes (BP) graphs show clusters with enrichment scores greater than 1.6 and the y-axis is labeled with the term having the highest fold enrichment in that cluster (Fig. 5). For the KEGG pathway graphs we included all of the relevant to cell biology; these pathways were visualized using DAVID (Supplementary Figs. S2-S4). GOrilla (v 4.1)^43,44^ was used to identify biological processes enriched (*p* ≤ 10^–5^) in our data, with a focus on categories relevant to cell biology with cluster enrichment scores greater than 1.6, labeling the graph with terms having the highest fold enrichment in the cluster. The pathways with the most enrichment for downregulated pathways are shown in Fig. S5, and the pathways with the most enrichment for upregulation are shown in Fig. S6. DAVID analysis showed that at 2 h post-irradiation, the largest changes (both up- and down-regulation) in BP terms were cell cycle, apoptosis, and DNA damage terms, as well as the related terms, cell proliferation and transcription regulation (Fig. 5a). Consistent with these, we observed enriched KEGG pathways for p53, TGF-β, and TNF signaling, as well as cell cycle at 2 h (Fig. 5b). These pathways, as well as FoxO, and Hippo, suggested the regulation of genes associated with cellular senescence and apoptosis. GO terms related to hypoxia, cell migration, and mesenchymal cell development were also increased. Remarkably, analysis of down-regulated GO term clusters enriched showed that all major down-regulations at 2 h and the majority of the clusters at 8 and 24 h involve cell cycle terms (Supplementary Figs. S5). The GOrilla GO term analysis agreed with the DAVID GO clustering and KEGG pathway analysis, with the most enriched terms associated with cell cycle events (spindle checkpoint, sister chromatid segregation, and metaphase/anaphase transition) and signal transduction by p53 class mediator, and transcription regulation. GOrilla analysis also revealed enrichment in terms related to the TGF-β signaling pathway, positive regulation of cell migration, and response to hypoxia.

At 8 h post-irradiation, DAVID analyses showed that the most enriched BP cluster was response to hypoxia, followed by terms related to regulation of proliferation, motility, and migration, suggesting a continuation of the response seen at 2 h post-irradiation (Fig. 5c). Clusters of gene regulation terms for apoptosis, cell cycle, and DNA damage were present but to a lesser degree. At 8 h, the most enriched KEGG pathway was p53 signaling followed by the cell cycle and tumor necrosis factor (TNF) and FoxO signaling pathways (Fig. 5d). GOrilla analysis identified enrichment of terms associated with hypoxia response, RNA processing, kinase activity, and cell cycle phase transition. The clusters, pathways, and terms enriched at 8 h suggest that the cells responded to radiation by arresting the cell cycle, possibly through p53 signaling, that may be coordinated with DNA repair and the induction of accelerated senescence through FoxO and TNF signaling pathways.

At 24 h post-irradiation, there was an increase in the numbers of regulated genes (2,922 genes > 1.5-fold [q ≤ 0.05]) compared with prior time points, and with higher enrichment scores than at 8 h. In addition to hypoxia, cell proliferation and cell cycle terms that were observed at other times, DAVID analysis of genes regulated at 24 h indicated that cell motility and migration, extracellular matrix (ECM) organization, cytoskeleton organization, and protein secretion processes were more prominent. The increased alterations in genes affecting structure, movement, and the exterior of the cell suggested a new phase of cellular response at 24 h (Fig. 5e). KEGG analysis showed that at 24 h, p53 signaling, DNA replication, cell cycle, and homologous recombination were most enriched, however TNF and TGF-ß pathways were also significant (Fig. 5f). GOrilla analysis also showed regulation of cell migration and adhesion, DNA damage response, replication and repair, focal adhesion assembly, and NF-kappaB transcription factor activity.

**Figure 6:**
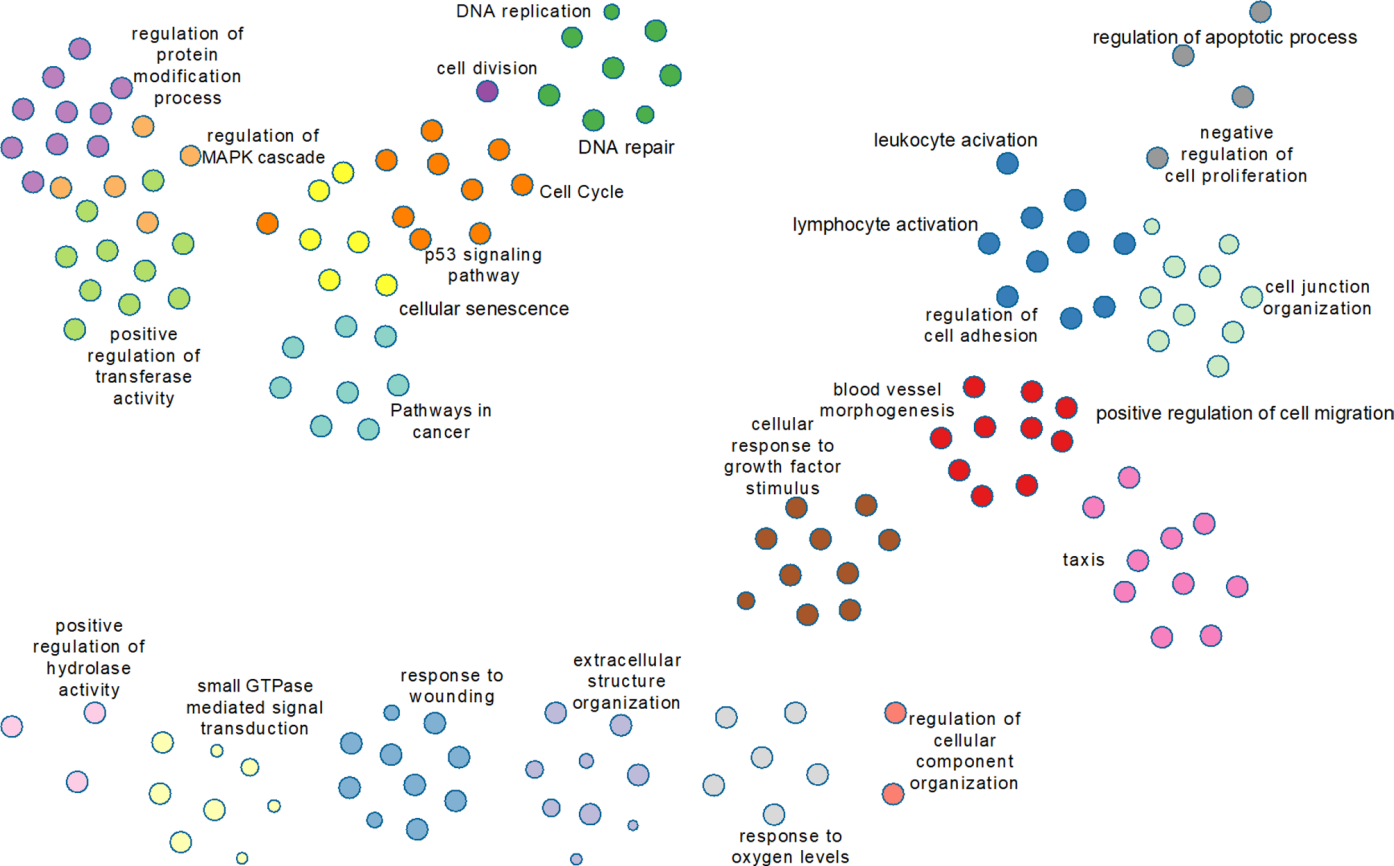
Visualization of clustered GO terms. Metascape (https://metascape.org^46^) was used to create an image of clustered GO terms present in the 1000 genes with the lowest q-value regardless of time point, Clusters, represented by colored circles, are grouped to aid in visualization of the relationships among the 1000 genes with the lowest q-value (q $\le$ 2.61E-37).

Metascape (https://metascape.org^46^) was used to create an image of clustered GO terms present in the 1000 genes with the lowest q-value regardless of time point (Fig. 6). This image allows us to visualize the important gene functions that are regulated in HLMVEC in response to 10 Gy irradiation.

### Focused analysis of RNA-seq after 10 Gy X-irradiation

To further analyze the DEGs, we used g:Profiler, web based software that performs functional enrichment analysis, and a literature search to identify pathways in the 3000 genes with the lowest q-value (≤ 1.18E-14). We identified four significant pathways activated following 10 Gy X-irradiation: cell cycle regulation; apoptosis; DNA damage response; and cellular senescence (Supplemental Table S2). Based on literature reports, we also found significant regulation of genes related to two additional processes: inflammation and endothelial-to-mesenchymal transition (EndMT)^50^. Focused heat maps were constructed using ClustVis that included genes regulated within each pathway to determine overall pathway activation or suppression over the experimental time course^45^.

### Cell cycle regulation

**Figure 7:**
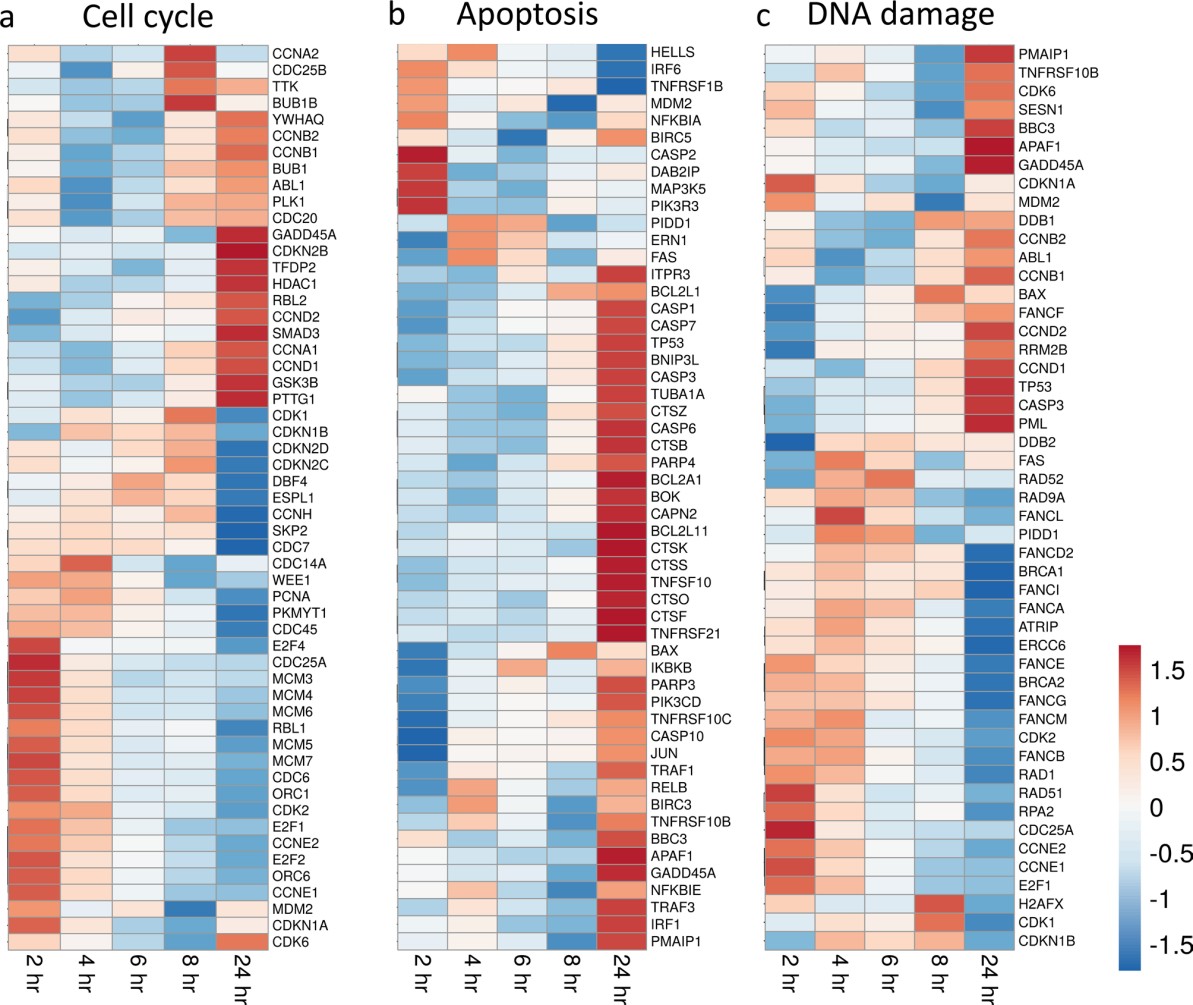

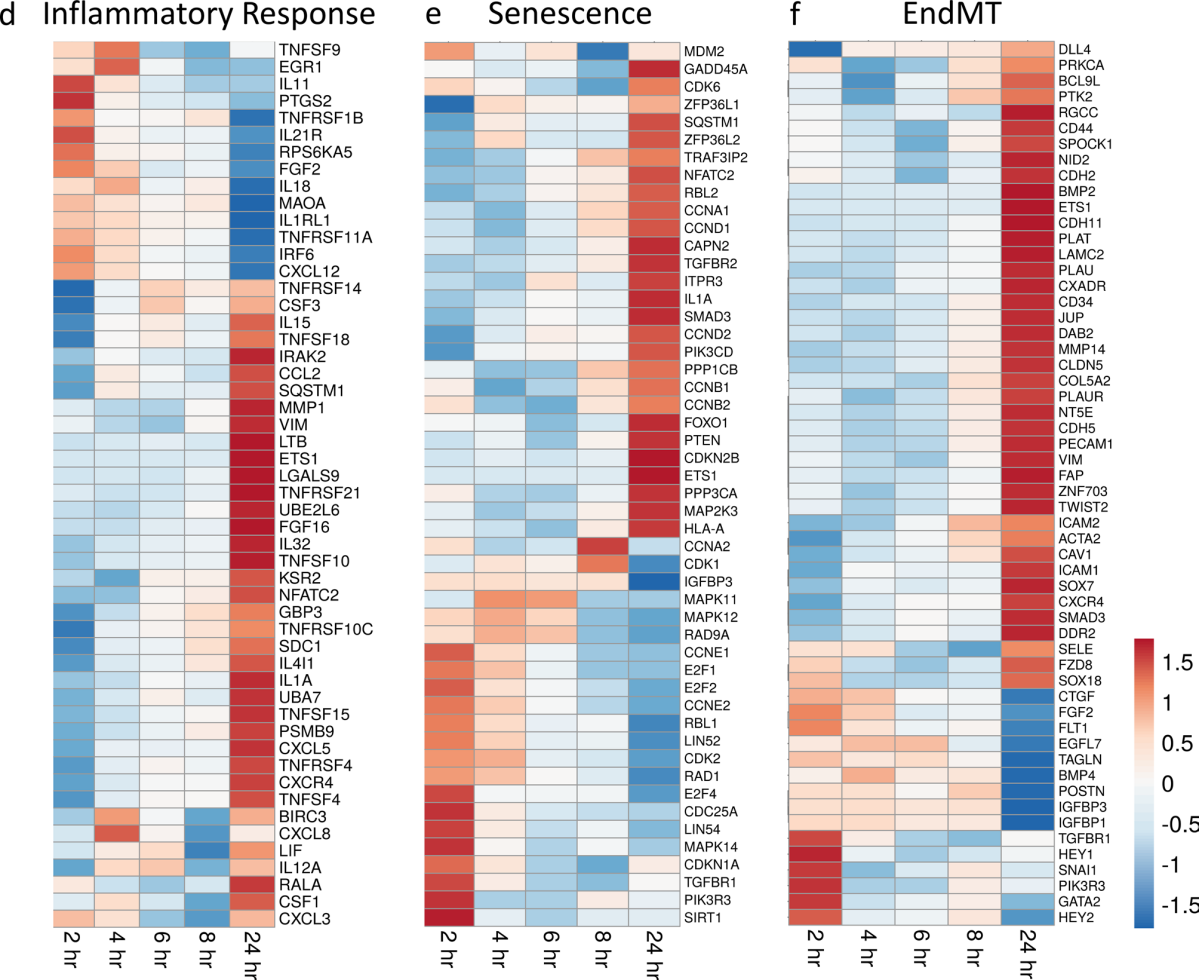
Heatmap of biological pathway gene expression in HLMVEC following radiation exposure. RNA-seq was used to identify gene expression changes in HLMVEC following exposure to 10 Gy X-ray irradiation. ClustVis (https://biit.cs.ut.ee/clustvis) was used to generate heat maps. Rows are centered; unit variance scaling is applied to rows. Rows and columns are clustered using correlation distance and average linkage. Set of 3000 genes with lowest q-value was submitted to g:Profiler (https://biit.cs.ut.ee/gprofiler/) to identify pathways. Pathway heat maps indicate changes in gene expression compared to control at the post-irradiation time points indicated. (**a**) Cell cycle pathway, (**b**) Apoptosis pathway, (**c**) DNA damage pathway, (**d**) Inflammatory response pathway, (**e**) Senescence pathway, and (**f**) Endothelial to Mesenchymal Transition pathway.

Radiation has a broad effect on the cell cycle in a wide variety of cell types^21^. Analysis of HLMVEC gene expression changes following radiation exposure identified 54 genes with roles in cell cycle regulation (Fig. 7a). Nine cyclin genes (*CCNA1*, *CCNA2*, *CCNB1*, *CCNB2*, *CCND1*, *CCND2*, *CCNE1*, *CCNE2*, and *CCNH*) displayed distinct expression patterns in response to ionizing radiation. *CCNA2*, that promotes transition through S/G_2_ and G_2_/M, decreased 1.7-fold at 24 h. *CCNB1* and *CCNB2*, that regulate progression through the G_2_/M transition, decreased at 2–6 h post-irradiation, but returned to near basal levels at 24 h. In contrast *CCND1* and *CCND2*, that regulate G_1_ phase transition, were elevated over full the time course, upregulated 1.9- and 4.5-fold at 24 h post-irradiation. *CCNE1* and *CCNE2*, that regulate G_1_/S phase transition, were increased at 2 h (1.5- and 1.8-fold respectively), but then were downregulated by 24 h (2.4- and 3.9-fold respectively). Three cyclin-dependent kinases (CDKs) *CDK1* and *CDK2* were downregulated 1.6- and 1.5-fold at 24 h. Cyclin-dependent kinase inhibitors *CDKN1A* and CDKN2B, were upregulated over the entire time course, and increased 4.2- and 4.7-fold, respectively, at 24 h. *CDKN1B*, *CDKN2C*, and *CDKN2D*, that control G_1_ progression, were downregulated over the time course, decreasing 1.6- ,2.6-, and 2.5-fold, respectively, by 24 h post-irradiation.

E2F genes encode transcription activators that control transcription of cell cycle genes^51^. In particular, G_1_-S phase transition requires E2F transcription factor activation. *E2F1* and *E2F2* transcriptional activators were initially upregulated (1.4- and 1.7-fold, respectively) and then downregulated (2.9-fold and 3.8-fold respectively) at 24 h. *E2F8*, that regulates G_1_ to S phase progression, was downregulated 3.2-fold by 24 h. Interestingly, the repressive E2F family member *E2F4*, which suppresses cyclin D expression, was downregulated 1.4-fold, correlating with the increased expression of cyclin D gene expression.

Two ORC subunit genes, *ORC1* and *ORC6*, were downregulated 2.7- and 2.1-fold, respectively, at 24 h post-irradiation. Expression of *MCM* complex genes 3–7 also decreased through 24 h in a similar pattern. Additionally, our further investigation of the data showed that *MCM2*, *MCM8*, and *MCM10* that also participate in DNA replication, although not identified in the program analysis, were also suppressed following X-irradiation.

### Regulation of apoptosis

Pathway analysis showed that both pro- and anti-apoptotic proteins involved in the initiation of the extrinsic and intrinsic apoptotic pathways were regulated, with an early wave of apoptotic gene regulation at 2 h, and a second wave of regulation at 24 h post-irradiation (Fig. 7b). The extrinsic pathway-related death receptors *TNFRSF10B*, *TNFRSF21* and *TNFRSF4* were increased 1.7-, 3.7-, and 10.7-fold respectively, at 24 h. However, the anti-apoptotic *TNFRSF10C*, a decoy death receptor that does not contain a death domain, had early and sustained expression, and was increased 2- to 3.3-fold from 4 to 24 h. Genes encoding TNF receptor ligands were also upregulated inclugin *TNFSF4*, *TNFSF9*, *TNFSF10*, *TNFSF12*, *TNFSF13*, *TNFSF15*, and *TNFSF18* (3.6-, 4.0-, 4.2-, 1.5-, 1.5-, 44.0-, and 2.3-fold respectively). *LTB*, encoding a cytokine that binds a TNF receptor 3 (*TNFRSF3*) was upregulated 26.5-fold. Genes encoding anti-apoptotic proteins acting to counteract extrinsic apoptotic pathway signaling were also increased, including TNF Receptor Associated Factor 1 (*TRAF1*), a negative regulator of apoptosis signaling by TNFR superfamily members, increased 7.2-fold by 24 h.

The Bcl2 family of proteins are pro- or anti-apoptotic, depending upon their effects on cytochrome *c* release from the mitochondrion. Genes encoding pro-apoptotic members of the Bcl-2 family were upregulated, including: *BBC3*, *BCL2L11*, *BCL2* Interacting Protein 3 Like (*BNIP3L*), and *BOK* (5.2-, 6.9-, 3.2- and twofold, respectively) at 24 h. However, at the same time, the anti-apoptotic *BCL2A1* gene was upregulated 3.5-fold. The transcript for *BCL2L1* was upregulated 2- and 2.1-fold at 8 h and 24 h, respectively, although alternative splicing can result in either a pro- or anti-apoptotic activity for this transcript.

We also observed mixed regulation of proteins required for downstream apoptotic signaling. Apoptotic protease activating factor 1 (*APAF1*) required for caspase activation, was increased 1.6-fold at 24 h. The gene for Jun proto-oncogene, AP-1 transcription factor subunit (*JUN*), also involved in caspase activation, was downregulated at 2 h, but increased 2.2-fold at 24 h. Cathepsins, lysosomal proteolytic enzymes that can directly or indirectly activate caspases, were also upregulated by radiation^52^: *CTSK*, *CTSO*, and *CTSS* were upregulated 3-, 2.2-, and 4.1-fold, respectively, at 24 h. Caspases 1, 3, 6, 7, and 10 were upregulated at 24 h post-irradiation (4.6-, 1.9-, 1.6-, 2.6-, and 1.5-fold, respectively). Caspase 2 was upregulated 1.3-fold at 2 h post-irradiation, but was subsequently downregulated. Our findings of mixed regulation of apoptotic genes is consistent with our finding of only very low levels of apoptosis in HLMVEC in response to radiation.

### Regulation of DNA damage response

GO analysis identified 48 genes associated with DNA damage that were regulated > 1.5-fold (Fig. 7c). Cyclins and their related kinases and kinase inhibitors make up 25% of the DNA damage response genes; these were described in Cell cycle regulation section, above. The data set also includes genes encoding DNA damage response and repair enzymes.

Radiation increased mRNA levels for genes in the excision repair pathway including DNA damage binding protein 1 (*DDB1*), its partner *DDB2*, and excision repair cross-complementing rodent repair deficiency (*ERCC6*), all critical for DNA excision repair, upregulated 1.6-, 2.2-, and 3.3-fold, respectively, throughout the time course. Growth arrest and DNA damage inducible 45A (*GADD45A*), an early DNA damage response gene with multiple functions in cell cycle and survival, was increased 3.4-fold at 24 h.

Interestingly, genes encoding proteins for homologous recombination repair and long-patch excision repair were downregulated. Homologous recombination repair genes that were downregulated included radiation 51 (*RAD51*) and *RAD52*, both reduced 1.5-fold at 24 h. Breast cancer 1 (*BRCA1*) and *BRCA2*, that also contribute homologous recombination repair, were downregulated 1.5- and 1.8-fold at 24 h. *RAD1* and *RAD9A*, involved in long-patch base excision repair, were downregulated 2.5- and 1.6-fold. Additionally, 9 members of the Fanconi anemia complementation group, involved in intra-strand crosslinking repair, were downregulated from 1.3- to 2.8-fold at later time points. These data suggest that HMLVEC utilized only specific pathways for DNA repair.

### Radiation-induced inflammatory response

Inflammation is a critical in vivo response to radiation exposure^13,53^. The normal function of the immune-inflammatory response is to prevent infection, instigate removal of dead or damaged cells, and initiate normal tissue repair^13^. However, chronic activation of inflammation post-irradiation can lead to an amplification of initial radiation damage to areas outside of the radiation field, leading to fibrosis and tissue necrosis^13,53^. We identified inflammation-related 52 genes with altered expression. Of these, 32 genes were upregulated > two-fold, and 17 were upregulated > four-fold at 24 h (Fig. 7d). These genes included transcription factors, interleukins (ILs), and chemokine receptors and ligands. Tumor necrosis factor receptors and ligands were discussed in the section on apoptosis (see above).

Transcription factors *ETS1* and *NFATC2* that regulate cytokine expression were upregulated 3.3- and 7.2-fold at 24 h. We identified 19 interleukin (IL) genes, targets of *ETS1* and *NFATC2*, that were significantly regulated from 2 to 6 h post-irradiation. Interleukin 1A (*IL1A*), a potent pro-inflammatory cytokine, was upregulated 8.5-fold at 24 h. T cell regulatory cytokine LIF was upregulated 2.6-fold at 2 h and 4.2-fold at 24 h. *CSF1*, macrophage colony-stimulating factor, was also upregulated 3.9-fold at 24 h. Chemoattractants for immune cells were also increased. *CXCR4*, that mediates migration of leukocytes, was increased 8.9-fold at 24 h. *CXCL12*, that mediates migration of lymphocytes and macrophages, was upregulated early, but was steadily reduced to 9.5-fold decrease at 24 h. *CCL2*, a monocyte, T cell, and dendritic cell attractant, was upregulated sixfold at 24 h.

### Senescence related gene expression induced by radiation

GO pathway analysis identified 50 genes involved in senescence (Fig. 7e). As for apoptosis where both pro- and anti-apoptotic genes were regulated, our data set for senescence also showed both pro- and anti-senescence gene regulation. Senescence pathway genes overlap with cell cycle regulation genes and DNA damage genes, previously discussed (see above sections). Interestingly, inflammation-related genes involved in senescence are some of the most upregulated in our data set; the inflammatory genes regulated by radiation were discussed in the prior section.

Insulin-like growth factor (IGF) signaling has been demonstrated to play a major role in age-related senescence as well as accelerated senescence^54^. Although immediate activation of the *IGF1* receptor is associated with the induction of senescence in cultured cells following biological stress, the IGF-binding proteins (IGFBPs) that affect the half-lives of IGFs and alter their interactions with cell surface receptors are associated with both pro- and anti-senescent activities^24,54^. The anti-senescence protein *IGFBP1*^55^ was downregulated 19-fold. *IGFBP3*, that can mediate proliferation, apoptosis, or senescence^56,57^, was also highly downregulated (> 15-fold) at 24 h. In contrast, *IGFBP6*, important in the progression of senescence, was upregulated 1.7-fold at 24 h. *IGFBP7*, that is secreted by senescent cells and can induce senescence in neighboring cells^58^, was also upregulated 1.7-fold at 24 h.

### Radiation regulation of endothelial-to-mesenchymal transition

EndMT is the process in which ECs lose their normal characteristics and exhibit a mesenchymal-like phenotype, including loss of cell–cell junctions, increased migration, and increased invasive capacity^50,59^. EndMT is not currently described as a distinct pathway in GO terms, so we conducted a literature search to identify genes associated with EndMT^60,61^. We found 64 genes in our data set that were regulated > 1.5-fold in at least one time point (Fig. 7f). Forty-four of the genes were upregulated at 24 h, 28 of them > twofold. Twenty-two genes were downregulated at 24 h, seven > twofold.

Consistent with mesenchymal transition, EC markers were suppressed and mesenchymal (MC) markers were upregulated within 24 h post-irradiation. EC markers *FLT1* was downregulated 2.8-fold at 24 h. Other markers, *PECAM1*, an intercellular junction adhesion molecule and *CDH5*, a cadherin that controls cohesion and organization of intercellular junctions, were upregulated 2.2-fold at 24 h suggesting radiation effects cell–cell adhesion. Several genes encoding cell surface proteins associated with recruitment of inflammatory cells, that were associated with EndMT in other systems, were also increased: *ICAM1* and *SELE* (2.1- and 6.2-fold respectively). The mesenchymal cell surface markers *ACTA2*, *VIM*, *CDH2* and *CDH11*^62^ were upregulated 5.4-, 6.1-, 2.3- and 4.6-fold, respectively, at 24 h. Surprisingly, although we observed significant upregulation of mesenchymal markers, we also observed simultaneous upregulation of endothelial markers *PECAM1* and *CDH5* at 24 h post-irradiation (as stated above).

Cytokines that contribute to EndMT and intracellular downstream signaling molecules were upregulated following HLMVEC irradiation. *BMP2* was upregulated 11.7-fold at 24 h post-irradiation. Interestingly, *BMP4*, shown to inhibit epithelial-to-mesenchymal transition^63^, was downregulated 4.4-fold at 24 h post-irradiation. *FGF2*, *HEY1*, and *HEY2* family members are negative regulators of EndMT were downregulated at 24 h. *TWIST2*, *FZD8*, *SPOCK1*, *DAB2*, *SMAD1* and *SMAD3*, signaling molecules that positively regulate EndMT^64^, were upregulated 2-, 2.2-, 1.7-, 2.5-, 4- and 3.3-fold at 24 h. Additionally, EndMT signaling protein *SNAI1*, and its stabilizer *DDR2*, were upregulated 1.1- and 1.84-fold at 24 h. *SOX7* and *SOX18*, transcription factors involved in regulating EndMT gene expression^65^, were increased 3.1- and 1.9-fold at 24 h post-irradiation. At 24 h post-irradiation, *DLL4*, a negative regulator of EndMT signaling, was downregulated 2.7-fold.

ECM proteins and proteases that degrade the extracellular matrix (ECM) were altered by radiation in HMLVECs. *COL5A2*, a protein characteristic of mesenchymal ECM secretion, was upregulated 1.9-fold at 24 h. Nine matrix metalloproteinase (MMP) genes were regulated > 1.5-fold: Collagenases *MMP1*, *MMP2* and *MMP19*, stromelysins *MMP10* and *MMP11*, and membrane anchored *MMP14* and *MMP15* were upregulated^66,67^. At this same time point matrilysin *MMP7* and membrane anchored *MMP16* were downregulated (1.5- to 2.5-fold). *PLAU*, *PLAT*, and *FAP*, encoding secreted serine proteases that degrade the ECM, release of growth factors and cytokines, and tissue cell migration, were upregulated 16.6-, 8.8-, 2.4-fold fold at 24 h.

## Discussion

Radiation injury to the vasculature is a key factor in delayed radiation damage to tissues. Damage to the endothelium can result in reduced blood flow and lower oxygen perfusion of the tissues^9,16,17^. Additionally, increased permeability of the microvascular endothelial barrier results in increased fluid extravasation, tissue edema, and increased inflammation of the tissues^10,15^. Thus, microvascular damage is considered to be a critical component for persistent radiation tissue damage and for the expansion of injury outside of the original field of radiation exposure. Here we examined an early time course of gene expression changes in cultured primary HLMVEC to gain insight into the pathways activated by acute radiation exposure. An overall summary of our GO analysis shows the regulation of KEGG and Wiki pathways, including cell cycle, apoptosis, DNA damage, and senescence (Fig. 7). We perused the literature for genes that have roles in EndMT and the inflammatory response, and interrogated our RNA-seq data for relevant gene regulation of those pathways. The time course of gene expression shows the coordination of these complex, yet interdependent responses to X-irradiation.

Regulation of genes regulating the cell cycle was notable within 2 h following irradiation. The majority of the genes identified in the cell cycle pathway were cyclin-related genes, origin recognition complex (ORC) subunit genes, and minichromosome maintenance complex components (MCM). Cyclins, cell division cycle genes, cyclin dependent kinases, and cyclin dependent kinase inhibitors cooperate to regulate the cell cycle progression and transitions. ORC protein subunits are required for assembly of the pre-replication complex for initiation of DNA replication during S phase of the cell cycle and serves as a platform at the origin of replication for the assembly of initiation factors and MCM proteins. MCMs control the cell cycle by regulating DNA replication^68^. The data show a complex pattern of regulation of cell cycle controlling genes, and overall suggest a downregulation of genes required for DNA replication and for S, G_2_, and M phase progression, and at the same time an increase in expression of genes encoding proteins involved in early G_1_ progression.

DNA damage response for the repair of single- and double-stranded breaks is critical for cell survival following radiation exposure. Multiple pathways of DNA repair have been recognized, including non-homologous end joining, homologous recombination, base excision repair, nucleotide excision repair, mismatch repair, the Fanconi anemia pathway, which corrects DNA intra-strand crosslinks, and DNA demethylating enzymes^47,69^. DNA repair activities are closely coordinated with cell cycle regulation, which is hypothesized to allow the time for DNA repair enzyme activity and to prevent the proliferation of cells with mutations or chromosomal aberrations^70^. We observed the activation of DNA damage response protein ATM within 15–30 min post-irradiation, with other DNA damage response genes upregulated 2–24 h post-irradiation, consistent with rapid and sustained DNA damage response.

Following the interruption of the cell cycle in the presence of DNA damage, cells may undergo a variety of responses including necrosis, apoptosis, or senescence. We did not observe significant necrosis in our biological assays, and we detected only low levels of apoptosis. However, we observed the activation of a significant number of pro- and anti-apoptotic genes with early regulation of both pathways at 2 h, followed by a second wave of activation of genes in both pathways at 24 h including genes for both the intrinsic and extrinsic apoptotic pathways^71^. The extrinsic pathway is usually triggered by activation of the death receptor family, including Fas receptor, and tumor necrosis factors activated by soluble or membrane-bound ligands^72,73^. In contrast, in the intrinsic apoptotic pathway various stimuli trigger the release of cytochrome *c* from the mitochondria, a process that can be regulated by pro- and anti-apoptotic members of the Bcl-2 protein family. In both apoptotic pathways, caspase proteases are activated, leading to DNA cleavage and disintegration of cellular structures ^74^. The mixed gene regulation that we observed for pro- and anti-apoptotic genes in both the intrinsic and extrinsic apoptosis pathways suggests that cells may be receiving multiple conflicting signals for survival and programmed death. This is in agreement with our findings of low levels of apoptosis from 24 to 72 h post-irradiation. Ultimately, the integrated response to gene expression changes in the apoptosis pathway appears to result in HLMVEC cell survival.

Our results indicate several groups of senescence-related genes are highly regulated in response to 10 Gy X-irradiation. Normal cells undergo an ageing process that induces irreversible replicative arrest^54,75^. Genotoxic stress and other stressors can cause the cell to permanently exit the cell cycle, resulting in accelerated senescence^54,75,76^. An increasing body of evidence suggests that induction of cellular senescence by ionizing radiation may be a driver of the pathogenesis of RILI^77^. The senescent phenotype is characterized by a variety of physiological alterations including changes in the production of extracellular matrix proteins, cell–cell connectivity, resistance to apoptosis, and the secretion of pro-inflammatory cytokines and factors^78^. Senescence signaling pathways overlap with cell cycle regulation and DNA damage response, since response to genotoxic stress, such as is induced by radiation exposure, can lead to DNA damage response that induces a pause of the cell cycle as an initial step in DNA repair, and failed DNA repair can result in senescence^54^. Insulin-like growth factor (IGF) signaling has been demonstrated to play a major role in age-related senescence as well as accelerated senescence^54^, although we did not observe IGFR activation in the HLMVEC.

The use of GO and KEGG for the analysis of RNA-seq data is limited by the molecular functions, biological processes, and cellular components that have been annotated. Because EndMT is not currently identified in GO terms, we used published findings to identify EndMT associated genes. EndMT process plays a role in organ development and in adult wound healing^79^. Dysregulation of EndMT is associated with the secretion of abnormal ECM in fibrotic organ diseases and in tumor microenvironments where it is believed to contribute to cancer progression and metastasis^59,79,80^. Signaling transduction cascades that contribute to EndMT include those induced by TGF-β family members, Notch, and Wnt ligands, oxidative stress, and inflammation^79^. MMPs are important in a variety of developmental processes including mediation of cell–cell adhesion, tissue remodeling, cell migration, and proliferation^81^. Our identification of EndMT in the biological response to radiation in HLMVEC may indicate that these cells can play a role in fibrotic remodeling following radiation exposure. Further investigation of EndMT may help to determine whether this pathway can be inhibited or modulated to reduce delayed tissue injuries following radiation exposure.

Together, our data indicate a complex and integrated regulation of biological processes leading mostly to cellular senescence, a low levels of apoptosis, increased inflammation, and EndMT. Further investigation of these pathways may lead to the identification of targets for prevention, mitigation and treatment of radiation injury to normal tissues.

## Supplementary Information

Supplementary Information 1.

Supplementary Information 2.

Supplementary Information 3.
