# Supplementary material for: Transcriptomic profiling and pathway analysis of cultured human lung microvascular endothelial cells following ionizing radiation exposure
Source: Sci Rep. 2021 Dec 20;11:24214. doi: 10.1038/s41598-021-03636-7 (PMC8688546; doi:10.1038/s41598-021-03636-7)
Supplement: Supplementary file 1 — Supplementary Information 1. [file 41598_2021_3636_MOESM1_ESM.pdf]

Table S1. List of gene exprssion changes by time point post-irradiation: 2 h, 4 h, 6 h, 8 h , and

Gene	l2fc_2hr	l2fc_4hr	l2fc_6hr	l2fc_8hr	l2fc_24hr	LRT_fdr
MAMDC2	0.369	0.543	0.937	1.402	2.945	0
PLA2G4C	0.007	0.712	1.364	1.767	4.326	4.6e-311
BMP2	0.568	0.699	0.78	0.88	3.549	8.73e-316
PLAU	0.655	0.743	1.531	1.733	4.057	3.97E-296
CX3CL1	-0.005	1.347	1.105	0.319	4.73	1.02E-291
PCDH12	-0.206	0.335	1.142	1.527	3.511	5.50E-290
STC1	-0.366	-0.237	0.43	0.582	3.786	2.13E-275
TNFSF15	-0.336	0.56	1.691	2.056	5.46	4.85E-267
BTG2	2.559	1.562	1.677	1.436	2.108	8.18E-258
NRG1	-1.283	0.805	0.16	-0.112	-0.891	2.73E-256
ATF3	3.62	2.856	2.38	1.628	2.592	4.81E-256
CCNE2	0.858	0.194	-0.632	-1.459	-1.955	1.16E-254
CDCP1	0.112	0.138	0.556	1.101	3.544	4.06E-251
CDH11	0.058	0.173	0.157	0.536	2.215	1.80E-250
PLAT	0.286	0.133	0.307	0.919	3.139	7.03E-245
TP53INP1	1.753	1.398	1.291	1.006	1.758	6.40E-244
TRIM22	0.443	0.963	1.104	1.164	1.584	1.85E-227
LTB	-0.335	-0.08	0.314	-0.081	4.73	1.65E-221
CCND2	-0.489	0.35	0.967	0.829	2.169	5.20E-221
CTNNBIP1	0.119	-0.039	0.184	0.581	1.712	3.19E-220
GAS6	0.212	0.162	0.22	0.401	1.647	6.90E-216
LPXN	0.37	0.031	-0.064	0.242	2.073	3.17E-214
SIPA1L3	0.216	0.016	0.728	1.071	1.93	9.87E-213
ADAM19	0.123	-0.165	0.126	0.406	1.891	2.17E-212
IL32	0.221	0.52	0.757	0.823	2.378	3.45E-209
OGFRL1	0.278	0.267	0.522	0.645	1.988	5.09E-209
TRAF1	-0.272	1.614	1.3	0.337	2.844	1.10E-202
FST	-0.01	0.992	0.195	-0.086	-2.006	5.53E-201
VWCE	1.286	2.246	2.343	2.015	2.606	1.35E-199
CYTL1	0.082	0.555	0.97	1.053	2.515	5.66E-198
SLC7A7	-0.255	0.158	0.641	1.058	1.959	1.41E-197
MYO1B	0.224	0.134	0.365	0.68	1.833	1.53E-193
KCNMA1	0.192	0.096	0.56	0.77	2.841	4.13E-190
E2F1	0.526	0.049	-0.852	-1.497	-1.559	7.89E-190
SULF2	0.454	0.35	0.465	0.883	2.26	1.29E-188
NDST1	-0.012	-0.02	0.228	0.587	1.632	1.64E-187

HES2	0.677	0.433	0.892	1.227	1.993	1.74E-187
CTSS	0.063	0.339	0.41	0.72	2.041	3.62E-185
RHBDF2	-0.181	0.245	0.795	0.85	1.64	5.35E-185
AOX1	0.055	-0.018	0.191	0.903	2.374	1.89E-182
IGFBP1	-0.049	-0.109	-0.561	-0.532	-4.25	4.58E-182
STK32B	0.282	0.447	0.995	1.338	2.053	8.89E-182
ITPKB	-0.629	0.174	0.603	0.705	1.95	1.22E-181
DDX58	-0.224	0.658	0.692	0.925	1.947	6.32E-181
FAM210B	0.483	0.452	0.59	0.883	1.646	1.31E-179
TMEM158	-0.113	-0.334	-0.387	-0.091	2.609	6.93E-177
MYH16	-0.003	0.839	2.054	2.735	5.406	1.82E-176
POU2F2	-1.159	0.959	1.321	0.955	2.419	1.39E-175
MDM2	2.096	1.75	1.922	1.397	1.909	2.57E-175
PBXIP1	0.055	0.118	0.243	0.647	1.783	2.63E-175
F11R	0.169	0.554	0.812	1.127	1.723	2.88E-175
ALPK3	0.008	-0.302	-0.061	0.022	1.504	2.65E-172
TP53I11	-0.038	0.22	0.543	0.704	1.946	7.64E-172
CMBL	0.433	0.866	1.417	1.776	2.558	8.48E-166
MAP4K4	0.172	0.051	0.164	0.331	1.109	3.39E-163
ZNF367	1.046	0.002	-0.52	-0.583	-0.774	5.37E-163
SH3RF3	0.328	-0.077	0.538	0.976	1.946	2.65E-162
CD82	-0.048	0.18	0.808	1.349	3.282	3.60E-162
SIRPB2	-0.019	-0.338	0.235	0.647	3.373	7.12E-161
SLC7A11	0.084	0.386	-0.171	-0.287	-1.792	7.72E-161
GADD45A	1.233	1.076	1.153	0.88	1.778	9.50E-161
TNFRSF21	0.384	0.236	0.274	0.495	1.893	3.40E-160
JUP	0.184	0.078	0.302	0.906	2.285	1.31E-159
E2F2	0.741	-0.278	-0.795	-1.422	-1.942	1.42E-158
SPATA18	0.565	1.447	1.726	1.843	2.242	2.58E-158
HIC1	0.286	0.31	1.019	0.828	3.179	1.56E-157
TRIB1	-0.843	0.745	0.889	0.694	2.001	1.14E-156
SESN1	1.545	1.359	1.31	1.108	1.605	2.08E-155
OTOGL	0.078	0.005	-0.538	-0.531	-3.275	7.91E-155
DYSF	0.24	0.03	0.414	0.887	2.234	1.04E-154
PLEKHG1	-0.43	0.427	0.799	0.907	1.855	7.49E-153
CHST1	-0.124	-0.236	0.611	0.782	2.274	4.68E-151
IGFBP3	-0.076	-0.008	-0.263	-0.119	-3.927	1.71E-150
RALA	0.354	-0.169	-0.301	-0.111	1.055	7.14E-150

RGMB	-0.363	-0.128	0.069	0.185	1.313	1.29E-147
NINJ1	0.313	0.499	0.799	1.086	2.05	2.68E-147
KIAA1551	-0.032	0.243	0.049	-0.106	1.406	8.19E-147
CCDC85C	-0.226	-0.548	0.141	0.641	1.661	1.56E-146
SH3BP5	-0.003	0.143	0.476	0.766	1.887	1.98E-146
ARL4C	0.44	0.465	0.664	1.157	3.398	5.32E-146
FRMD6	0.095	0.573	0.785	0.866	1.963	6.69E-145
KITLG	0.994	1.049	0.781	1.048	2.583	9.34E-145
MPP4	-0.096	0.034	0.699	0.924	2.993	3.37E-143
ADAMTS1	0.866	0.112	-0.507	-0.557	-1.86	3.56E-143
CYFIP2	0.353	1.565	1.803	2.174	2.523	3.17E-142
APOL3	0.073	0.762	1.47	1.259	2.578	4.83E-142
CHST2	0.223	-0.286	-0.089	0.359	1.297	2.33E-141
CDKN1A	2.376	2.121	1.81	1.717	2.078	3.72E-141
ETS1	0.681	0.683	0.691	0.738	1.723	2.37E-140
WDR76	0.624	0.02	-0.594	-0.789	-1.588	3.11E-140
ALDH3A1	1.637	2.573	2.971	3.609	3.866	1.16E-138
GALNT6	0.195	0.077	0.296	0.705	2.073	1.76E-138
EPS8L2	0.103	1.353	1.842	1.662	2.574	3.22E-137
ITGA2	0.069	0.913	1.015	1.131	1.776	5.77E-137
RP11-497D6	0.142	0.014	-0.192	-0.167	2.524	2.39E-136
BTG1	0.84	0.379	0.293	0.48	1.304	3.31E-136
PNMA2	-0.564	0	0.949	1.353	2.416	1.00E-135
SMAD1	-0.095	0.06	0.252	0.553	1.985	1.34E-134
CDC25A	0.825	-0.142	-0.686	-0.819	-0.874	1.52E-134
CSF1	0.517	1.239	0.384	-0.155	1.945	2.62E-134
IL1A	0.258	0.519	1.256	1.02	3.087	3.61E-134
INSIG1	0.824	-0.269	-0.567	-0.514	0.749	5.49E-133
RP11-54A9.1	-0.061	0.059	0.495	0.545	1.184	1.49E-131
PARP14	0.006	0.602	0.871	0.889	1.905	3.07E-131
TMEM98	0.053	0.022	0.007	0.215	1.001	8.61E-131
SESN2	1.87	1.147	1.305	0.831	1.33	8.93E-130
TNS3	-0.077	0.098	0.594	0.855	1.453	7.43E-127
ARHGAP22	-0.196	0.042	0.707	0.936	1.485	1.75E-125
SVIL	0.142	0.166	0.452	0.523	1.116	5.20E-124
PLA2G4A	0.372	0.097	-0.555	-0.901	-3.052	1.07E-123
CCNB1	-0.946	-1.877	-1.593	-0.76	-0.194	2.59E-123
DFNA5	0.362	0.39	0.699	1.155	1.718	2.93E-123

ZFAND5	0.532	0.318	0.173	0.139	0.893	3.16E-123
TCF19	0.259	0.103	-0.02	-0.157	-1.266	1.09E-122
TMEM217	0.485	1.866	1.801	1.525	3.171	1.10E-122
MFAP2	0.123	0.059	-0.018	0.312	1.671	1.56E-122
IRF1	0.798	1.008	0.237	0.132	1.986	2.10E-122
ERGIC1	0.188	-0.108	0.077	0.416	1.245	5.24E-122
GDF15	1.882	2.126	1.776	1.622	1.959	1.34E-121
BNIP3L	0.211	0.322	0.589	0.994	1.679	1.68E-121
RBFOX2	0.175	-0.116	0.083	0.366	0.921	6.20E-121
PLK2	1.436	1.23	1.115	0.827	1.691	4.22E-120
LOX	0.386	0.044	-0.175	-0.033	1.452	6.15E-120
SLC40A1	0.789	1.33	0.341	-0.448	-1.44	4.88E-119
LDLR	0.724	-0.516	-1.141	-1.126	0.122	1.74E-118
SOX7	0.015	0.482	0.322	0.495	1.643	4.76E-118
PIK3CD	-0.223	0.604	0.742	0.654	1.672	6.77E-118
TNFSF4	-0.075	0.605	0.874	0.867	1.852	1.49E-117
RHOF	0.189	-0.193	0.182	0.558	1.74	4.37E-117
RPGR	0.196	-0.009	-0.227	-0.473	-1.627	5.56E-117
PDGFC	0.439	0.797	0.61	1.008	1.615	9.70E-117
DOCK4	0.513	-0.032	0.456	0.526	1.115	6.51E-116
CEACAM1	0.568	1.604	1.948	2.11	3.632	2.54E-115
PHLDB2	-0.267	0.03	0.289	0.339	1.172	3.48E-115
DLG1	-0.188	0.12	0.399	0.617	1.209	2.50E-114
PPM1D	1.464	0.785	0.634	0.457	0.875	4.00E-114
PTGS2	2.054	0.236	-0.45	-0.748	-1.219	6.74E-114
LPIN1	-0.093	0.117	0.277	0.393	1.217	2.51E-113
OCIAD2	0.1	-0.18	-0.274	-0.271	1.085	3.87E-113
UBE2H	0.233	0.218	0.311	0.568	1.503	4.96E-113
ARL6IP1	-0.483	-0.86	-1.032	-0.546	0.143	8.12E-113
SMAD3	0.35	0.642	0.889	0.778	1.727	1.04E-112
IL1RL1	0.252	0.076	-0.4	-0.477	-2.633	2.60E-112
MYLK	0.217	0.019	0.18	0.634	1.599	3.82E-112
MGLL	0.244	-0.027	0.191	0.697	1.597	7.88E-112
CDC6	0.76	0.119	-0.487	-0.553	-1.115	1.05E-111
TNFRSF4	-0.87	0.619	1.209	0.905	3.413	1.77E-111
ANGPTL2	0.51	0.495	0.778	1.348	2.288	7.93E-111
POLE	0.123	0.021	-0.303	-0.845	-1.109	2.46E-110
CCNG2	0.178	0.22	0.281	0.637	1.587	5.24E-110

FHL2	0.51	0.622	0.945	1.254	1.669	4.26E-109
IRF6	0.73	-0.035	-0.809	-0.98	-2.681	4.66E-109
DOCK10	0.169	-0.059	0.209	0.214	1.118	2.09E-108
TMEM132A	-0.039	0.063	0.042	0.189	1.147	2.17E-108
TRAK1	0.071	0.338	0.482	0.542	1.106	3.75E-108
PTPN22	0.706	-0.201	0.213	0.722	2.914	1.28E-107
RHOBTB1	0.599	0.108	0.226	0.455	1.512	1.28E-107
YBX3	0.388	0.594	0.724	0.847	1.229	1.54E-107
ORC1	0.767	0.053	-0.699	-0.621	-1.408	2.39E-107
LRP8	-0.039	-0.134	-0.124	-0.539	-1.258	3.14E-106
IRAK2	0.414	0.909	0.684	0.616	1.847	7.55E-106
TNIP1	0.056	0.607	0.744	0.912	1.663	7.75E-106
DRAM1	0.496	0.827	0.768	0.823	1.238	1.02E-105
ANTXR1	0.09	-0.036	-0.121	0.217	1.25	1.14E-105
ART4	0.528	0.534	-0.303	-0.792	-1.85	1.69E-105
HMGCS1	0.873	-0.2	-0.699	-0.745	0.01	4.16E-105
DBNDD1	-0.077	0.051	0.362	0.818	1.428	4.68E-105
RP1-152L7.5	-0.968	0.024	0.395	0.125	1.534	6.37E-105
ADAMTS7	0.108	0.434	0.565	0.478	1.518	6.72E-105
PGF	0.632	-0.047	0.252	0.376	1.5	1.35E-104
BACE2	0.221	0.038	0.011	0.355	1.299	2.87E-104
AKR1C3	-0.194	-0.106	-0.129	-0.071	-1.132	1.64E-103
MMP14	0.146	0.245	0.376	0.702	1.439	2.93E-103
CORO1C	0.142	-0.088	0.044	0.331	0.819	1.18E-102
DAB2	0.268	0.172	0.338	0.612	1.317	1.38E-102
BMP4	-0.653	-0.086	-0.581	-0.489	-2.128	2.91E-102
PSRC1	-2.334	-2.493	-1.695	-1.101	-1.16	4.18E-102
PFKFB3	0.578	0.342	0.268	0.367	1.338	9.18E-102
KSR2	-0.12	-0.429	0.466	0.455	1.32	3.17E-101
MAP1LC3B	0.264	0.194	0.224	0.437	1.018	2.04E-100
MAP3K6	0.603	0.056	-0.2	-0.605	-1.164	3.01E-100
JUN	-1.228	0.261	0.284	0.323	1.119	9.67E-100
RP11-889L3.	-0.542	0.056	0.596	0.357	2.121	2.20E-99
AFAP1L1	0.078	-0.027	0.406	0.609	1.373	8.40E-99
SLCO2A1	0.228	-0.284	-0.858	-0.886	-2.54	9.91E-99
RELB	0.213	1.85	1.101	0.575	1.811	1.85E-98
ANXA3	-0.092	-0.152	0.126	0.308	1.646	4.00E-98
PHLDA3	1.657	1.824	1.633	1.737	2.151	1.07E-97

NMT2	0.158	-0.019	0.005	0.325	0.959	1.13E-97
APOL1	0.258	0.363	0.68	1.093	2.229	2.19E-97
DGCR8	-0.006	-0.183	-0.344	-0.892	-0.676	2.76E-97
NEK7	-0.206	-0.11	-0.366	-0.493	-1.339	3.55E-97
SLC7A14	-0.304	0.4	0.96	1.256	1.953	4.61E-97
NTM	0.169	0.084	0.03	0.206	1.797	7.40E-97
ANXA4	0.317	0.313	0.435	0.697	1.369	2.92E-96
SP6	-0.823	0.075	0.58	0.633	1.357	8.67E-96
PLD1	-0.245	-0.034	0.621	0.941	1.649	1.11E-95
FDXR	1.392	1.6	1.637	1.488	1.646	1.39E-95
TSPAN18	0.209	-0.289	-0.172	0.31	1.56	1.64E-95
CDKN2B	0.673	0.852	0.679	0.885	2.224	1.18E-94
RAD54L	0.082	0.037	-0.253	-0.813	-1.474	2.88E-94
TMTC1	-0.116	0.005	-0.344	-0.348	-2.268	4.39E-94
AURKA	-1.134	-1.789	-1.506	-0.661	-0.442	4.51E-94
ELMOD1	0.324	-0.695	-1.255	-1.812	-3.173	4.53E-94
LGALS9	0.146	0.208	0.164	0.546	2.621	1.03E-93
CCDC68	0.76	-0.109	-0.758	-0.811	-2.356	1.36E-93
MB21D2	0.074	0.117	0.406	1.078	1.866	1.47E-93
CABLES1	1.364	-0.244	0.391	1.104	1.559	1.50E-93
SOX4	0.683	0.271	0.876	1.285	2.219	1.62E-93
CSGALNAC	-0.022	0.069	0.567	1.121	2.116	2.29E-93
ZWINT	-0.02	0.035	-0.062	-0.099	-1.051	3.36E-93
ATP2B4	0.128	-0.038	0.296	0.595	1.38	4.84E-93
CXCR4	-0.328	0.669	1.207	1.198	3.156	5.31E-93
BLOC1S5	-0.022	-0.038	-0.407	-0.659	-1.557	7.65E-93
ANTXR2	0.274	-0.188	-0.218	0.104	0.944	1.31E-92
MYO5C	-0.311	0.146	0.819	1.03	2.442	2.11E-92
MEDAG	0.283	-0.837	-0.137	0.662	1.424	2.52E-92
ZSWIM4	0.084	0.963	0.62	0.437	1.582	4.84E-92
ERCC6	-0.397	-0.15	-0.389	-0.599	-1.726	7.18E-92
CLCN4	0.168	-0.231	-0.382	-0.091	-1.42	8.06E-92
SRSF3	0.243	-0.046	-0.274	-0.483	-0.537	9.97E-92
APOBEC3C	0.493	0.593	0.921	1.546	2.745	4.02E-91
GRK5	0.219	-0.353	-0.168	0.153	1.055	4.37E-91
OAS2	-0.03	-0.014	0.611	1.101	2.157	5.54E-91
MICA	0.167	-0.057	-0.214	-0.356	-0.921	9.80E-91
SHISA3	-0.536	-0.115	-0.549	-0.51	-2.817	1.36E-90

FAM83D	-1.6	-1.591	-1.24	-0.48	-0.391	1.53E-90
CCNE1	0.606	-0.068	-0.611	-1.211	-1.268	2.21E-90
P2RX4	-0.07	0.236	0.44	0.657	1.701	5.87E-90
DTX3L	-0.033	0.646	0.8	0.997	1.612	7.43E-90
ICAM1	0.116	1.137	0.956	0.963	2.611	1.57E-89
BBC3	2.09	1.701	1.827	1.627	2.389	1.64E-89
ABLIM1	0.218	0.456	0.647	0.97	2.007	1.82E-89
PASK	-0.279	-0.099	-0.396	-1.129	-1.688	2.62E-89
EFNB2	0.45	-0.129	-0.769	-0.593	-1.098	3.23E-89
GLTSCR2	0.068	0.175	0.208	0.409	1.08	9.39E-89
PAQR4	0.249	-0.134	-0.571	-0.691	-1.473	1.27E-88
MCAM	0.024	0.118	0.204	0.549	1.409	1.45E-88
MARCKSL1	0.14	-0.124	-0.278	0.189	1.162	2.34E-88
WSCD1	-0.243	0.107	0.607	0.952	1.849	2.43E-88
DDB2	0.652	1.191	1.211	1.142	1.125	8.19E-88
SIRPA	0.312	0.017	-0.076	0.243	1.271	9.26E-88
MASTL	0.569	0.3	-0.191	-0.237	-0.518	1.16E-87
FAM111B	0.663	0.258	-0.43	-0.596	-1.919	1.64E-87
SULT1B1	-0.058	-0.026	-0.08	0.071	-0.979	2.41E-87
MSI2	0.337	0.046	0.298	0.512	0.996	2.55E-87
MERTK	-0.278	0.599	1.139	1.158	1.778	3.81E-87
FAM49A	0.129	0.218	0.322	0.559	1.357	4.60E-87
ARVCF	-0.848	0.088	0.475	0.123	1.517	7.92E-87
DSN1	-0.115	-0.02	-0.225	-0.403	-1.165	8.38E-87
ALDH7A1	0.341	0.283	0.313	0.736	1.669	8.74E-87
CREB3L1	0.315	0.176	0.615	0.731	2.932	1.90E-86
RNF24	0.285	0.209	0.247	0.222	1.207	2.56E-86
IPMK	0.504	0.704	0.522	0.506	1.434	9.59E-86
PLEKHO1	0.524	-0.004	-0.1	0.027	1.137	2.58E-85
CTD-2562J1	-0.03	0.213	0.756	1.061	1.981	2.76E-85
FILIP1L	0.351	0.216	0.332	0.792	1.486	3.17E-85
RRM2B	0.606	1.02	1.008	1.006	1.278	3.18E-85
DDX11	-0.215	-0.142	-0.22	-0.887	-1.496	3.23E-85
HOMER3	-0.178	-0.149	-0.096	0.053	0.898	7.10E-85
TGIF1	-0.874	0.148	0.228	0.229	1.106	1.55E-84
ELMO1	0.226	0.224	0.332	0.87	1.701	1.72E-84
DKK3	0.291	0.028	-0.058	0.337	1.337	2.73E-84
SH3D19	-0.157	-0.09	-0.018	0.161	0.699	1.56E-83

POLD3	0.195	0.124	-0.097	-0.326	-1.068	1.56E-83
KIAA1211L	0.242	0.367	0.736	1.026	1.729	1.92E-83
TFPI2	0.063	0.032	-0.106	0.105	0.846	2.38E-83
USP1	0.462	0.05	-0.223	-0.058	-0.837	3.33E-83
DTL	0.992	0.022	-0.66	-0.57	-0.726	3.61E-83
NT5E	0.158	0.006	0.086	0.525	1.245	4.84E-83
GPC1	0.398	0.736	0.852	1.225	1.991	6.94E-83
BCL2L11	0.161	0.661	0.383	0.283	2.781	7.08E-83
SLC35B1	0.155	0.031	-0.126	-0.132	-0.9	1.02E-82
HMGCR	0.423	-0.344	-0.706	-0.597	-0.022	1.03E-82
DCBLD2	0.088	-0.138	-0.405	-0.191	0.771	1.06E-82
GMNN	0.042	-0.272	-0.614	-0.711	-1.376	1.40E-82
RALGPS2	-0.023	-0.234	-0.248	-0.034	0.905	1.59E-82
CCBE1	0.256	-0.075	0.149	0.532	1.33	2.11E-82
FEZ1	0.235	0.263	0.412	0.748	1.148	2.55E-82
EXO1	0.828	0.299	-0.235	-0.317	-1.226	9.09E-82
SASH1	0.273	0.153	0.71	0.967	1.299	1.10E-81
CDCA7	0.051	-0.43	-1.028	-1.225	-0.901	1.77E-81
CAPN2	0.179	0.065	0.218	0.549	1.275	1.04E-80
PIK3IP1	0.104	-0.252	0.055	0.247	2.488	1.08E-80
CCNG1	0.545	0.765	0.763	0.696	1.176	1.50E-80
ATP8B1	0.028	0.087	0.388	0.571	1.539	1.54E-80
AIM1	-0.665	-0.09	0.326	0.559	1.254	1.57E-80
RP11-284F2	-0.191	0.218	1.643	2.175	2.852	1.76E-80
SGIP1	-0.089	0.243	0.643	0.337	2.55	2.53E-80
UNG	0.557	-0.172	-1.008	-0.914	-0.561	2.53E-80
FGL2	0.517	0.063	-0.45	-0.081	-1.989	3.99E-80
OPTN	0.123	0.482	0.535	0.642	1.289	5.56E-80
ZBTB16	0.387	0.264	-0.145	-0.764	-1.942	7.68E-80
DGKA	0.067	0.597	0.871	0.66	0.567	1.00E-79
RSPO3	-0.481	0.44	0.023	-0.186	-2.299	1.03E-79
HJURP	-1.468	-0.936	-1.087	-1.073	-1.598	1.79E-79
RNF121	0.02	-0.098	-0.298	-0.361	-1.091	1.98E-79
STEAP1	-0.406	-0.443	-0.49	-0.449	-1.557	2.32E-79
SARAF	-0.007	0.018	0.086	0.262	0.837	2.82E-79
LIMS1	0.447	0.079	0.132	0.582	1.293	5.11E-79
NF2	-0.097	-0.061	0.187	0.61	1.085	5.19E-79
ABCG1	0.263	0.253	0.827	1.352	2.384	7.04E-79

BHLHE40	0.496	0.399	0.886	1.074	2.47	1.17E-78
FRMD4A	0.3	0.092	0.514	0.618	0.932	1.28E-78
NBAS	0.208	0.101	0.246	0.664	1.396	2.48E-78
HMMR	-0.802	-1.148	-1.149	-0.857	0.041	5.31E-78
MTSS1	0.021	-0.143	0.488	0.484	1.801	9.09E-78
CASP7	-0.045	0.311	0.608	0.652	1.356	9.09E-78
GBP3	-0.246	0.092	0.457	0.628	0.973	1.19E-77
AC007743.1	0.09	0.314	0.075	-0.248	-1.269	2.77E-77
LAMA4	0.159	0.001	0.113	0.39	1.147	4.02E-77
PLK1	-1.146	-2.204	-1.641	-0.69	-0.644	6.28E-77
PECAM1	0.173	-0.033	-0.028	0.422	1.117	1.02E-76
PER2	0.251	-0.658	-1.325	-1.948	-1.226	1.24E-76
CITED4	-0.441	-0.019	0.082	0.381	2.006	3.26E-76
SERTAD1	1.176	0.234	0.247	0.268	-0.032	9.42E-76
PHACTR1	0.161	-0.515	0.498	0.771	1.209	9.56E-76
ATAD5	0.411	0.168	-0.272	-0.683	-1.088	1.03E-75
OSBPL3	-0.219	0.512	0.773	0.575	1.157	1.61E-75
NFATC2	0.762	0.812	1.642	1.81	2.853	3.94E-75
MCM8	0.003	0.135	-0.051	-0.321	-1.125	4.97E-75
RUSC2	0.683	0.617	0.821	0.684	1.034	1.12E-74
DNAJC9	0.279	0.073	-0.148	-0.178	-0.727	1.61E-74
TACC2	0.689	-0.202	0.336	0.762	1.203	2.14E-74
ACTA2	0.589	1.186	1.511	1.996	2.44	2.99E-74
PALD1	-0.374	-0.136	1.057	1.139	2.584	4.18E-74
THSD1	1.018	0.853	1.087	1.096	1.193	4.27E-74
NFKB2	-0.042	1.151	0.724	0.124	0.806	4.50E-74
SNX22	-0.607	-0.641	-1.339	-1.926	-3.518	8.87E-74
RBBP8	0.346	0.337	0.023	-0.097	-1.094	8.99E-74
ZNF703	0.299	-0.096	0.079	0.434	1.358	9.07E-74
TSPAN13	0.345	-0.102	-0.665	-0.899	-1.207	1.20E-73
FAP	0.212	-0.016	0.023	0.274	1.235	2.53E-73
RP11-424C2	0.839	-0.244	-0.883	-0.791	-0.73	2.93E-73
SPSB1	1.258	0.072	0.674	1.086	2.131	4.07E-73
FBXO5	-0.232	-0.27	-0.377	-0.295	-1.259	4.45E-73
SYT9	0.179	0.002	0.231	0.669	1.559	5.08E-73
NCOR2	0.422	-0.222	0.449	0.736	1.195	6.67E-73
FZD8	0.611	-0.308	-0.472	-0.175	1.151	6.97E-73
TRIM16	-0.157	-0.238	0.344	0.552	1.244	6.98E-73

ASPM	-1.222	-1.564	-1.409	-1.062	-0.734	8.06E-73
UGCG	-0.467	0.386	0.518	0.288	1.175	1.06E-72
MCM10	0.614	0.088	-0.49	-0.451	-1.244	1.09E-72
TBC1D9	0.115	0.053	0.165	0.509	1.24	1.26E-72
E2F8	-0.132	-0.264	-0.268	-0.319	-1.677	1.26E-72
CDC45	0.187	0.107	-0.297	-0.526	-1.255	1.34E-72
MEF2A	0.861	0.234	0.312	0.221	0.503	1.67E-72
PDE1C	-0.379	0.029	0.517	0.73	0.825	2.15E-72
NYNRIN	0.037	-0.103	0.266	0.513	1.143	2.22E-72
CNTNAP1	-0.324	0.168	0.606	0.501	1.532	2.80E-72
NTN4	0.172	0.378	0.647	1.131	1.541	3.68E-72
KIAA1462	-0.055	-0.089	0.177	0.31	0.998	3.99E-72
RAD51AP1	-0.125	0.033	-0.18	-0.298	-1.318	4.75E-72
TMEM63B	-0.215	0.111	0.349	0.295	0.96	5.35E-72
NFE2L3	0.255	0.303	0.337	0.754	1.557	5.82E-72
QPCT	0.263	0.104	0.035	0.325	1.094	6.51E-72
CXADR	-0.415	-0.526	-0.075	-0.033	1.303	7.64E-72
CXorf36	0.001	-0.042	0.558	0.681	3.101	1.09E-71
LRRC17	0.302	-0.288	-0.725	-0.767	1.759	2.61E-71
F2RL1	-0.211	0.569	0.316	0.324	1.43	3.39E-71
PERP	0.252	0.177	0.2	0.565	1.213	4.18E-71
UBE2C	-0.752	-1.399	-1.25	-0.934	-0.683	4.18E-71
ASAP1	0.307	0.128	0.45	0.472	0.939	4.83E-71
RTN4	0.32	0.052	-0.03	0.307	1.081	8.23E-71
LAMC2	0.301	0.138	0.436	0.755	2.528	1.42E-70
UBE2L6	0.151	0.088	0.325	0.744	2.148	1.45E-70
PSTPIP2	0.364	1.105	1.009	1.036	1.341	1.86E-70
MAST4	0.197	0.482	0.53	0.432	1.169	1.97E-70
TNFRSF10C	0.313	1.014	1.167	1.334	1.729	4.73E-70
ANKRD1	0.002	-0.047	-0.117	-0.043	-1.014	6.69E-70
NRIP1	0.547	0.468	0.507	0.491	1.679	7.18E-70
PRSS23	0.248	0.005	-0.131	0.153	0.844	9.17E-70
ZNF207	0.087	0.027	-0.1	-0.37	-0.384	9.29E-70
RAB15	-0.012	0.105	0.099	-0.139	0.81	1.27E-69
TNFAIP8L1	-0.668	0.079	0.005	0.121	1.21	1.40E-69
CDC20	-0.911	-1.817	-1.549	-0.721	-0.673	2.01E-69
IGBP1	0.178	0.152	0.254	0.518	0.955	2.28E-69
COL25A1	0.506	0.183	-0.004	0.004	-1.935	2.32E-69

BASP1	0.244	-0.103	-0.1	0.275	0.95	2.51E-69
XPC	0.726	0.986	0.91	0.898	1.323	6.37E-69
ADGRL2	-0.226	-0.134	-0.696	-0.797	-1.406	3.34E-68
ESPL1	-0.963	-0.673	-0.507	-0.599	-1.448	6.14E-68
MGP	0.029	0.004	-0.088	-0.144	-1.331	6.27E-68
SAMD9L	-0.235	0.187	0.544	0.467	1.076	1.06E-67
CELF2	0.097	0.138	0.533	0.933	2.34	1.15E-67
KLHL24	1.142	0.752	0.601	0.849	1.932	1.15E-67
UTRN	0.183	0.039	0.185	0.392	1.231	1.64E-67
DAP	0.149	-0.011	-0.002	0.307	0.852	1.73E-67
CALCRL	0.182	-0.036	-0.566	-0.544	-0.501	1.92E-67
SERPIND1	0.176	-0.089	-0.155	0.411	2.693	2.23E-67
PLOD2	0.051	0.145	0.131	0.387	1.01	2.39E-67
GULP1	-0.262	-0.265	-0.671	-0.942	-1.418	2.62E-67
RIN2	-0.001	0.388	0.499	0.438	0.976	2.85E-67
RASSF2	-0.695	-0.323	0.102	0.015	0.8	4.22E-67
KRT80	0.517	0.456	0.85	1.027	1.128	5.63E-67
NR2F1	-0.389	-0.33	-0.666	-0.717	-1.919	7.98E-67
SLC16A7	0.363	-0.124	-0.235	-0.465	-1.635	9.63E-67
DHRS7	0.14	0.013	0.068	0.243	1.238	1.45E-66
SLBP	0.431	-0.127	-0.457	-0.472	-0.392	2.17E-66
CXCL12	0.749	0.03	-0.782	-1	-3.244	2.27E-66
KNTC1	-0.269	0.153	0.057	-0.28	-0.871	2.32E-66
FANCA	-0.387	0.035	-0.073	-0.766	-1.489	2.39E-66
OSGIN2	0.675	0.046	-0.477	-0.44	-0.946	3.80E-66
CD93	0.329	-0.055	-0.064	0.358	1.162	4.34E-66
BIRC3	0.633	2.139	1.267	0.358	2.021	5.89E-66
RAP2B	0.568	0.249	0.35	0.606	1.129	7.09E-66
UBALD2	-0.054	-0.001	0.268	0.596	1.333	1.21E-65
SLC3A2	0.141	0.018	-0.143	-0.194	-1.137	1.28E-65
STARD13	-0.085	0.055	0.342	0.281	0.789	2.19E-65
GAS2L3	-1.475	-1.35	-0.892	-0.564	0.117	3.39E-65
KRT18	0.144	0.055	0.283	0.841	1.452	3.50E-65
NR1D2	1.366	0.218	-0.069	0.577	1.054	3.95E-65
KLF6	-0.668	0.278	0.005	-0.258	0.26	4.57E-65
APBB2	0.378	-0.142	0.247	0.43	0.925	4.73E-65
LFNG	1.201	-0.621	-0.234	-0.285	0.611	5.23E-65
CCNF	-1.291	-0.895	-0.695	-0.403	-0.903	5.32E-65

PREX2	-0.167	-0.036	-0.225	-0.483	-1.229	6.12E-65
UBTD1	-0.194	-0.384	-0.177	0.103	1.084	7.20E-65
NFKBIA	1.116	0.659	0.216	0.081	0.853	1.35E-64
FNDC5	0.135	0.167	0.266	0.241	3.437	1.85E-64
FYN	0.224	0.026	0.256	0.505	0.992	1.87E-64
ORC6	0.22	-0.2	-0.572	-0.876	-1.053	2.93E-64
FGF2	1.11	0.858	0.306	0.435	-0.171	4.74E-64
TIGAR	0.865	0.987	0.819	0.767	0.984	7.17E-64
AK3	0.062	0.168	0.086	0.177	0.802	7.72E-64
CLDN5	-0.447	-0.406	-0.143	0.22	1.07	9.64E-64
MARCKS	0.192	0.011	0.075	0.644	1.241	1.29E-63
ALDH3A2	0.546	0.73	0.832	1.145	0.739	2.47E-63
USP53	-0.302	0.047	0.05	0.052	0.765	2.90E-63
ENC1	1.274	-0.042	0.222	0.274	1.128	3.08E-63
FREM3	0.033	-0.273	-0.822	-1.49	-3.147	5.52E-63
UHRF1	0.826	-0.163	-0.763	-0.764	-0.698	5.58E-63
STOM	0.761	0.881	0.723	0.797	0.711	5.73E-63
TRAM2	-0.154	-0.212	0.151	0.288	0.758	6.86E-63
CEP85	-0.428	-0.336	-0.426	-0.768	-1.183	7.05E-63
TSTA3	-0.105	-0.01	0.066	0.021	0.779	7.93E-63
EXOC6	-0.512	-0.412	-0.816	-0.525	-0.99	2.29E-62
ARHGAP29	-0.107	0.067	0.365	0.533	0.47	2.53E-62
RP5-1039K5	-0.032	0.08	0.243	0.507	1.443	2.79E-62
CD34	-0.042	0.042	0.015	0.261	0.73	3.28E-62
SAMD1	0.579	-0.223	-0.827	-1.082	-0.565	3.28E-62
FGFR3	0.696	0.003	-0.765	-1.193	-2.527	3.43E-62
IDS	0.256	0.085	0.061	0.084	0.842	3.71E-62
C10orf10	-1.602	0.34	-0.073	-0.113	1.967	6.11E-62
EFNB1	0.534	0.379	0.315	0.615	1.415	6.22E-62
CDH13	0.127	0.06	-0.007	0.434	1.13	6.37E-62
DDIT4L	0.369	0.586	0.699	0.788	2.917	7.34E-62
APLN	-1.27	-0.018	0.865	1.041	2.279	1.18E-61
HTR1B	-0.507	-0.147	-0.553	-0.526	-2.017	1.45E-61
RFC3	0.258	0.165	-0.461	-0.432	-0.841	1.60E-61
FGFR1	0.059	-0.094	-0.128	0.015	0.787	2.61E-61
MCTP1	-0.035	0.126	-0.424	-0.728	-1.369	3.25E-61
SRPRB	0.149	-0.157	-0.249	-0.24	-0.797	4.26E-61
GRASP	0.984	-0.013	-0.085	-0.238	-0.314	1.08E-60

EGLN1	0.209	0.192	0.133	0.507	1.049	1.48E-60
ADD3	0.071	0.175	0.409	0.655	1.583	1.72E-60
CCNJL	-0.431	-0.053	0.181	0.552	1.086	2.72E-60
ARHGEF28	-0.381	0.012	0.256	0.062	0.415	2.72E-60
INSC	0.355	0.046	0.658	1.137	2.4	2.75E-60
FKBP1A	0.268	0.074	-0.003	0.245	0.799	2.99E-60
AGRN	0.041	0.265	0.372	0.447	1.275	6.28E-60
CERK	0.232	-0.28	0.123	0.406	0.647	8.28E-60
CMTM7	0.198	-0.246	0.108	0.319	0.893	8.30E-60
TMEM154	0.3	-0.229	-0.202	0.074	1.685	8.72E-60
TIMP2	0.188	0.02	0.065	0.453	1.358	9.59E-60
CDC20P	-0.866	-1.927	-1.653	-0.776	-0.657	9.74E-60
RNF19B	0.929	0.654	0.618	0.698	1.33	1.10E-59
FAM46A	1.02	0.669	0.152	0.079	0.076	1.50E-59
IDI1	0.31	-0.401	-0.877	-1.05	-0.374	1.68E-59
MAML3	0.767	-0.493	0.556	0.888	1.245	1.97E-59
CCND1	0.182	0.036	0.269	0.607	0.925	2.17E-59
PHF19	-0.433	-0.584	-0.518	-0.445	-1.105	2.28E-59
SQLE	0.314	-0.437	-0.735	-0.559	0.162	2.29E-59
MYCBPAP	-0.587	0.441	1.538	0.932	2.55	2.47E-59
HELLS	0.074	0.357	-0.262	-0.368	-0.982	2.54E-59
MSMO1	0.227	-0.503	-0.949	-1.014	-0.138	2.69E-59
NEBL	0.379	-0.052	-0.416	-0.616	-1.344	3.13E-59
CTSK	0.175	0.331	0.277	-0.022	1.581	3.76E-59
INPP4B	-0.789	0.465	0.148	0.616	1.703	6.47E-59
EPHB2	0.226	-0.199	-0.071	0.444	1.161	7.20E-59
TAP1	0.328	0.938	0.922	0.902	1.261	7.24E-59
RP11-536O1	0.026	-0.356	-0.06	0.2	2.021	9.54E-59
SYNE1	0.117	0.355	0.565	0.783	1.117	1.06E-58
TSKU	1.372	0.639	0.733	0.778	1.301	1.07E-58
WNK4	0.031	0.064	0.551	0.494	1.331	1.14E-58
BRIP1	0.281	0.33	-0.149	-0.477	-0.777	1.94E-58
CDH5	0.296	0.123	0.162	0.555	1.134	3.49E-58
ZNF618	0.114	-0.319	0.018	0.319	0.883	3.79E-58
FAM129B	0.17	-0.088	0.123	0.515	1.03	3.87E-58
CTGF	0.08	0.015	-0.302	-0.351	-0.983	6.56E-58
FGF16	-0.12	-0.09	0.293	0.444	2.89	7.73E-58
NEDD4L	0.666	-0.074	-0.007	0.296	1.058	1.04E-57

RFC5	0.083	0.035	-0.212	-0.299	-0.96	1.56E-57
ACAT2	0.214	-0.227	-0.528	-0.654	-0.796	1.70E-57
KALRN	-0.187	0.035	0.494	0.52	1.866	1.71E-57
STEAP2	-0.994	-0.513	-0.775	-1.012	-1.371	1.94E-57
TNFSF9	2.12	2.236	1.847	1.801	1.995	1.96E-57
AEN	0.911	0.583	0.709	0.44	0.787	2.13E-57
TNFRSF14	-0.287	0.599	1.079	0.845	1.149	2.24E-57
SLC9A3R2	0.371	-0.15	-0.443	-0.413	-0.862	2.29E-57
PLPP3	0.867	-0.009	-0.445	0.031	-0.144	2.41E-57
PODXL	0.579	-0.047	-0.282	-0.009	1.023	2.67E-57
COL17A1	0.015	0.148	0.705	0.634	2.376	4.37E-57
ANGPTL4	0.035	0.031	0.983	1.277	1.391	4.46E-57
TMCC3	-0.115	0.009	0.286	0.41	1.335	5.22E-57
UNC13B	0.042	0.175	0.472	0.576	0.636	5.75E-57
GINS3	0.522	-0.1	-0.488	-0.332	-0.939	6.16E-57
PHLDA1	-0.402	0.16	0.256	0.121	0.948	6.32E-57
JUNB	1.358	0.862	0.048	-0.017	1.115	7.01E-57
RAB33B	0.246	0.295	0.571	0.517	1.06	7.21E-57
PCNA	0.474	0.599	0.35	-0.004	-0.305	9.01E-57
ETS2	0.338	0.42	0.334	0.359	1.136	9.03E-57
PLXNB2	0.536	0.99	0.963	0.561	0.882	1.51E-56
EXOSC9	0.132	0.066	-0.195	-0.371	-0.824	1.92E-56
MMP1	0.188	0.008	-0.019	0.382	1.212	2.12E-56
CSDAP1	0.394	0.396	0.644	0.859	1.131	2.38E-56
MCC	0.235	0.077	0.231	0.639	1.098	2.50E-56
PDCD1LG2	-0.207	0.191	0.421	0.708	0.816	3.19E-56
SLC9A7	-0.089	-0.139	0.319	0.503	1.439	3.31E-56
SAP30L	0.08	0.138	0.095	0.235	0.914	5.53E-56
CLMN	0.233	0.034	0.915	0.878	2.073	8.52E-56
IER5	1.026	0.629	0.739	0.848	1.374	8.61E-56
CCDC69	0.074	0.18	0.398	0.674	1.399	1.36E-55
DUSP6	-1.143	-0.118	-0.237	-0.331	-0.209	1.42E-55
FEN1	0.472	0.167	-0.181	-0.136	-0.956	1.46E-55
NPAS2	-0.016	-0.149	0.269	0.488	1.254	1.62E-55
FLT1	0.478	-0.075	-0.503	-0.318	-1.467	1.78E-55
MPST	-0.161	-0.188	-0.049	0.196	0.804	2.05E-55
KIF18B	-1.02	-0.798	-0.54	-0.776	-1.499	2.22E-55
DKK1	0.409	0.302	-0.019	0.051	-0.889	2.38E-55

SGO2	-0.893	-1.135	-1.112	-0.569	-0.783	3.04E-55
TMEM37	0.087	-0.65	-0.977	-1.057	-2.891	3.20E-55
WDR26	0.019	0.148	0.181	0.211	0.648	4.41E-55
CTSB	0.246	0.054	0.004	0.405	0.971	5.72E-55
MTMR10	-0.234	0.049	-0.201	-0.403	-1.054	6.34E-55
GPSM2	-1.081	-1.468	-0.869	-0.259	0.083	6.44E-55
TBC1D4	-0.03	-0.1	-0.386	-0.706	-1.055	1.02E-54
TSPAN14	0.154	-0.282	-0.013	0.22	0.671	1.12E-54
NOP56	-0.272	-0.183	-0.225	-0.555	-0.906	1.20E-54
HN1	0.103	-0.278	-0.373	-0.107	0.412	1.48E-54
USP37	0.492	0.152	-0.257	-0.59	-0.725	1.65E-54
HEY2	1.175	-0.446	-0.329	0.153	-1.524	1.69E-54
LTF	-0.264	-0.692	0.203	0.506	1.675	2.35E-54
SMAD9	1.256	-0.21	-0.574	-0.431	-1.351	2.54E-54
IL4I1	-0.032	0.639	0.856	1.265	2.175	2.55E-54
TBC1D5	0.488	0.061	0.224	0.778	1.184	2.55E-54
DLL4	-1.421	-0.079	-0.042	0.022	0.448	2.57E-54
ADGRA2	0.06	-0.12	-0.154	0.031	0.941	2.63E-54
FAM89B	0.201	-0.127	-0.163	-0.141	0.683	2.69E-54
DEPDC1	-1.194	-1.64	-1.389	-1.112	-0.643	3.17E-54
TIMELESS	0.006	0.037	-0.07	-0.116	-0.819	3.89E-54
SH2D2A	0.23	0.444	0.93	1.309	2.47	3.98E-54
CAV1	-0.221	0.049	0.249	0.554	1.076	3.98E-54
KIF18A	-0.895	-1.408	-1.215	-0.465	-0.355	4.41E-54
BUB1	-0.659	-1.189	-1.047	-0.361	-0.228	4.73E-54
CENPA	-1.959	-1.654	-1.115	-0.623	-0.843	6.40E-54
HIVEP2	1.96	1.702	0.902	0.484	2.073	6.42E-54
EIF3F	0.069	0.195	0.225	0.348	0.965	6.42E-54
CD274	-1.229	0.046	-0.009	-0.447	-0.201	1.37E-53
PMEPA1	0.753	-0.171	-0.357	0.388	1.39	1.98E-53
FAR2	0.325	-0.064	-0.103	0.334	1.364	2.59E-53
SLC9A1	0.528	0.908	0.867	0.988	1.394	3.18E-53
DUSP5	-0.042	0.678	0.517	0.374	1.027	4.03E-53
GAL3ST4	0.518	1.024	1.342	1.784	2.303	5.13E-53
CCNB2	-0.564	-1.02	-1.08	-0.587	-0.315	5.17E-53
GLT25D1	0.094	0.126	0.092	0.153	0.754	6.05E-53
YPEL2	-0.29	0.484	0.069	0.117	0.939	6.97E-53
CASP3	0.098	0.271	0.344	0.542	0.909	7.52E-53

MCMBP	0.427	-0.055	-0.527	-0.328	-0.316	7.61E-53
STAP2	-0.138	0.39	0.919	0.727	2.356	8.58E-53
SPC25	-0.399	-0.313	-0.249	0.081	-1.426	9.51E-53
CDC42EP3	-0.473	0.29	0.381	0.389	0.739	1.18E-52
SCARB2	0.215	0.098	0.003	0.342	0.891	1.35E-52
SDC1	0.418	1.19	1.271	1.696	2.248	1.38E-52
TSC22D1	0.426	0.323	0.412	0.657	0.92	1.42E-52
CRYAB	0.306	-0.092	-0.288	-0.169	-2.12	1.96E-52
BST2	-0.116	-0.166	-0.036	0.178	0.692	2.11E-52
CENPE	-0.984	-0.571	-0.628	-0.143	0.129	2.59E-52
FMNL2	-0.129	-0.029	0.554	0.346	1.16	3.48E-52
PRNP	0.231	-0.056	-0.202	0.094	0.722	3.95E-52
BARD1	0.126	-0.195	-0.416	-0.46	-1.042	3.95E-52
NCAPH2	0.168	0.123	-0.031	-0.157	-0.885	4.97E-52
EPHX4	-0.132	0.173	0.561	1.26	3.134	5.05E-52
CDH2	0.466	0.151	-0.159	0.274	1.23	5.36E-52
QSOX1	0.141	0.004	0.017	0.372	1.101	1.04E-51
SLC5A6	-0.172	-0.183	-0.271	-0.485	-0.862	1.11E-51
PRKX	0.036	0.421	0.445	0.414	0.862	1.14E-51
BDNF	-0.059	0.212	-0.424	-0.182	-1.452	1.24E-51
DEPDC1B	-0.96	-1.083	-0.913	-0.667	-1.498	1.26E-51
PTPRM	0.035	-0.061	0.003	-0.068	1.037	1.34E-51
SOD2	0.192	0.439	0.226	0.268	1.227	1.45E-51
MEX3B	0.362	0.14	0	0.334	2.127	1.57E-51
GLS	-0.044	-0.021	0.104	0.202	0.655	1.73E-51
PIF1	-2.384	-2.239	-1.326	-1.384	-1.354	1.73E-51
SSBP3	0.385	-0.165	-0.046	0.356	0.937	2.37E-51
PPM1F	-0.125	-0.307	0.148	0.351	0.705	2.38E-51
DGAT2	-0.424	-0.484	0.36	0.764	1.905	2.42E-51
STX3	0.803	0.147	0.071	0.138	0.383	2.63E-51
SAV1	1.106	0.424	0.011	0.147	0.294	3.06E-51
ITGA6	0.201	0.076	0.084	0.503	1.102	3.11E-51
TMEM200A	0.357	0.131	-0.28	0.032	1.126	4.02E-51
SLC7A2	0.976	1.278	0.64	0.136	0.018	4.29E-51
CRY2	0.095	0.069	-0.529	-1.183	-0.82	6.50E-51
PRRG1	0.132	0.229	-0.032	0.036	0.86	6.58E-51
DDIT4	-0.479	-0.155	0.372	0.501	0.767	6.93E-51
HERC4	-0.32	0.002	0.306	0.4	0.433	7.74E-51

BMF	0.498	0.602	0.679	0.186	2.142	8.36E-51
TRIM21	0.096	0.51	0.669	0.986	1.25	8.98E-51
RNF41	-0.048	-0.01	-0.306	-0.261	-0.926	9.63E-51
ATF6B	-0.153	0.008	0.061	0.315	0.697	1.23E-50
PHLPP2	0.676	0.131	-0.148	-0.558	-0.074	1.29E-50
PINK1	-0.115	-0.013	0.153	0.294	0.742	1.49E-50
ARHGEF7	-0.352	0.084	0.18	0.124	0.596	1.50E-50
BOK	0.194	0.013	0.223	0.45	0.971	1.54E-50
GINS2	-0.074	-0.23	-0.534	-1.023	-1.288	1.54E-50
MBNL2	0.516	0.113	0.342	0.786	1.131	2.01E-50
OAF	0.188	-0.079	-0.109	0.231	0.931	2.10E-50
RP11-286H1	0.083	0.208	-0.447	-1.183	-2.932	2.32E-50
SLC27A3	-0.576	-0.145	-0.219	-0.718	-1.36	3.04E-50
DBN1	0.031	-0.173	-0.069	0.178	0.76	3.11E-50
EDA2R	0.797	1.272	1.287	0.679	1.219	3.25E-50
CASP1	-0.067	0.361	0.956	1.02	2.191	3.31E-50
GJA5	0.594	-0.655	-1.596	-1.354	-3.065	3.31E-50
RETSAT	0.161	0.335	0.43	0.515	0.687	3.34E-50
KLF13	1.157	-0.084	0.337	0.692	0.809	3.85E-50
SYNC	0.228	0.473	0.854	0.938	1.418	3.94E-50
RP11-443P1	-0.247	0.396	0.09	-0.6	-1.784	4.73E-50
SH2D3C	-0.561	-0.118	0.344	0.472	0.804	5.79E-50
SRSF7	0.137	-0.192	-0.332	-0.614	-0.664	6.18E-50
ATOH8	1.326	-0.402	-0.24	0.105	-0.534	6.25E-50
WDR62	-0.331	-0.274	-0.222	-0.579	-1.37	6.85E-50
DMD	0.066	0.016	0.188	0.235	-1.128	7.01E-50
SNED1	0.2	0.06	0.196	0.311	1.189	8.59E-50
KIF20A	-0.925	-1.674	-1.54	-0.572	-0.283	8.60E-50
RFC4	0.195	-0.11	-0.26	-0.356	-0.847	9.90E-50
ARRB1	0.059	0.096	0.555	0.71	1.405	1.06E-49
PPP2R3A	0.228	0.221	0.241	0.293	1.361	1.20E-49
EIF2B2	0.388	-0.03	-0.023	0.386	0.934	1.21E-49
PTPRU	0.314	0.07	-0.001	0.205	1.254	1.33E-49
AC008268.3	-0.777	-0.067	0.228	-1.157	-2.107	1.66E-49
SLC12A2	-0.039	0.159	-0.077	-0.244	0.727	2.04E-49
ID2	1.777	-0.006	0.227	0.29	-1.252	2.08E-49
SMURF2	0.464	-0.067	0.166	0.121	0.963	2.18E-49
SEMA7A	0.305	0.378	-0.005	-0.397	1.962	2.22E-49

PNRC1	0.345	0.705	0.454	0.546	1.443	2.44E-49
ST6GAL1	-0.1	-0.004	0.228	0.395	1.104	3.02E-49
PARD3B	-0.142	-0.177	0.229	0.306	0.952	3.02E-49
CHMP1B	0.488	0.282	0.164	0.441	0.86	4.03E-49
GCHFR	-0.169	-0.015	0.109	-0.09	2.012	4.21E-49
ARSD	0.347	0.109	0.155	0.541	1.156	6.20E-49
SEPT11	0.327	0.121	0.065	0.377	1.07	6.69E-49
CAMKK2	-0.046	0.088	0.243	0.296	0.683	6.94E-49
MAFG	0.337	0.219	0.015	-0.326	-0.376	7.85E-49
CCL2	-0.31	1.245	0.886	0.285	2.573	8.58E-49
JAG2	0.454	-0.321	-0.637	-0.732	-1.002	9.08E-49
SNX9	0.343	0.156	0.171	0.357	0.932	1.03E-48
ZSWIM5	0.38	-0.215	-0.832	-0.925	-1.594	1.03E-48
SPHK1	0.656	0.354	0.268	0.052	1.037	1.04E-48
NUAK2	0.625	1.12	0.146	-0.099	2.459	1.06E-48
NLRC3	0.547	-0.32	0.53	0.836	1.718	1.27E-48
EXTL3	0.059	-0.169	-0.064	0.319	0.911	1.49E-48
CCNA2	-0.504	-0.78	-0.736	-0.267	-0.755	1.76E-48
ROBO4	-0.093	-0.147	0.1	0.415	0.791	1.86E-48
PRADC1	-0.246	-0.393	-0.494	-0.651	-1.355	2.26E-48
ST3GAL5	0.317	-0.282	0.102	0.475	1.286	2.41E-48
FUCA1	0.148	0.56	0.735	0.95	1.594	2.81E-48
TENM3	0.152	-0.008	0.201	0.677	1.38	2.92E-48
NLK	-0.222	0.043	0.156	0.101	0.648	3.19E-48
CTXN1	-0.253	-0.156	-0.074	0.138	1.696	3.39E-48
SCD5	-0.035	-0.274	-0.179	0.121	0.747	3.56E-48
KIAA1324L	0.099	0.182	0.198	0.509	0.953	4.38E-48
SRSF1	0.232	-0.044	-0.204	-0.147	-0.354	5.92E-48
KIAA0319L	-0.026	0.091	0.159	0.383	0.746	6.02E-48
CDK2	0.229	0.169	-0.208	-0.292	-0.532	7.60E-48
TRAF3IP2	-0.336	-0.164	0.344	0.873	1.162	9.36E-48
MTCL1	0.633	-0.193	-0.262	-0.597	-0.804	1.10E-47
CLDN14	1.198	1.063	1.359	1.657	2.87	1.25E-47
PXDN	0.305	0.186	0.124	0.512	1.358	1.51E-47
TMEM100	0.238	-0.713	-1.758	-2.047	-3.216	2.04E-47
ARHGAP26	0.212	-0.104	0.254	0.315	1.202	2.27E-47
SMG6	-0.002	0.26	0.254	0.466	1.089	2.53E-47
FBXL7	-0.4	-0.191	-0.139	-0.043	0.831	2.84E-47

SNCA	0.028	-0.162	-0.281	-0.138	-0.969	2.92E-47
DPF1	-0.029	0.066	-0.332	-0.648	-1.749	3.39E-47
RUNX1T1	0.886	-0.443	-0.598	-0.424	-0.423	3.61E-47
GPRC5B	-0.108	0.48	0.368	0.514	1.36	3.85E-47
TNFRSF10B	0.268	0.646	0.424	0.098	0.79	3.93E-47
TTK	-0.795	-0.904	-0.851	-0.191	-0.319	4.28E-47
NID2	0.549	0.329	0.038	0.325	1.658	5.60E-47
DOCK6	0.138	-0.026	0.256	0.11	0.821	6.20E-47
ADGRF5	0.018	0.04	-0.008	0.298	1.765	6.44E-47
NPR1	-0.123	-0.069	-0.271	-0.252	-2.008	7.77E-47
RPS6KA5	0.836	-0.031	-0.089	-0.295	-1.319	1.24E-46
BST1	-0.425	-0.122	0.082	0.53	0.952	1.68E-46
VSIR	0.12	0.081	0.26	0.822	1.173	1.81E-46
EPB41L3	0.409	0.219	0.236	0.735	1.416	1.85E-46
TNFAIP8L3	0.953	-0.172	-0.419	-0.276	0.101	2.06E-46
TGFBR2	-0.134	-0.081	0.064	0.292	0.815	2.14E-46
SLC35F2	-0.066	-0.268	-0.127	-0.077	0.518	2.34E-46
TBX18	-0.688	-0.212	-0.663	-0.743	-1.089	2.41E-46
CDKN2C	-0.494	-0.742	-0.661	-0.237	-1.402	2.99E-46
TFAP2A	0.985	0.717	-0.376	-0.773	-2.085	3.66E-46
GJC1	0.656	0.231	-0.03	-0.214	-0.065	4.34E-46
RPS6KA3	0.206	-0.079	-0.017	0.225	0.699	4.58E-46
RRM2	0.481	0.348	0.207	0.341	-0.84	4.75E-46
PTPRE	-0.112	0.004	0.378	0.315	0.876	5.61E-46
MMP10	0.42	0.171	-0.061	0.226	0.935	6.15E-46
DNA2	-0.079	0.118	-0.191	-0.358	-1.222	6.22E-46
MEX3D	0.257	0.07	-0.317	0.028	0.957	7.16E-46
TMEM97	0.172	-0.113	-0.48	-0.52	-0.649	7.88E-46
DHCR24	0.205	-0.324	-0.75	-0.869	-0.496	8.12E-46
EVA1A	-0.47	-0.329	0.11	0.462	0.956	8.16E-46
C4orf46	0.072	-0.367	-0.539	-0.408	-0.811	8.49E-46
TRA2B	-0.035	-0.148	-0.276	-0.351	-0.8	8.71E-46
GALNT1	0.068	0.228	0.279	0.596	0.861	1.02E-45
C1QTNF1	0.223	-0.201	-0.212	0.24	1.377	1.28E-45
SNPH	-0.699	-0.745	-0.138	0.193	0.449	1.41E-45
NEK2	-0.927	-1.25	-1.247	-0.428	-0.03	1.42E-45
TM7SF3	-0.006	-0.011	0.153	0.403	0.658	1.62E-45
ZC3H7B	0.04	0.144	0.199	0.335	0.898	1.66E-45

DMTN	-0.035	-0.03	0.033	0.342	1.464	1.70E-45
SQSTM1	0.163	0.662	0.472	0.484	1.064	1.96E-45
UBE2J1	0.399	0.023	-0.008	0.155	0.599	2.01E-45
LMO7	-0.061	-0.054	-0.616	-0.605	-1.076	2.17E-45
RP11-874J12	-0.865	0.032	0.63	0.299	1.653	2.19E-45
GATA2	0.703	-0.471	-0.245	-0.038	-0.682	2.21E-45
CDC42BPA	0.243	-0.022	0.246	0.512	0.99	2.33E-45
ATP2B1	0.254	0.017	-0.591	-0.666	-0.738	2.69E-45
PDE4B	-0.5	0.32	-0.073	-0.26	0.318	2.74E-45
CHAF1A	0.453	0.004	-0.464	-0.548	-0.718	2.76E-45
CES2	0.419	0.687	0.776	0.872	0.87	2.85E-45
LIPG	0.625	-0.232	-0.804	-0.685	-2.105	2.87E-45
FAM117A	0.223	-0.319	-0.633	-0.608	-1.603	2.92E-45
CASP8AP2	0.241	0.054	-0.27	-0.614	-0.495	3.06E-45
TMEM255B	-0.004	-0.041	0.079	0.114	0.618	3.15E-45
TNFAIP1	0.195	0.381	0.304	0.468	0.963	4.43E-45
RNF44	-0.034	0.128	0.348	0.355	0.947	4.50E-45
PTK2	0.209	-0.065	0.171	0.521	0.688	4.53E-45
PCDH7	0.269	-0.415	-1.112	-1.081	-2.279	4.72E-45
DPYSL2	0.338	-0.101	0.249	0.824	1.377	4.87E-45
PDE3A	-0.427	0.181	0.074	-0.047	-0.83	5.19E-45
H1F0	-0.194	-0.798	-0.386	0.026	-0.898	5.35E-45
NIPAL3	0.127	0.252	0.483	0.81	0.777	5.68E-45
CTD-2568A1	0.059	-0.091	-0.341	-0.488	-0.654	5.81E-45
AURKB	-0.801	-0.663	-0.378	-0.245	-1.125	5.85E-45
CHMP4B	0.306	0.198	0.197	0.585	1.01	5.90E-45
PDLIM1	0.293	0.379	0.476	1.022	1.408	6.18E-45
RP11-284F2	-0.367	0.085	1.277	1.595	2.456	6.46E-45
SLC44A1	0.311	0.005	-0.259	-0.036	-0.656	6.74E-45
PARP9	0.05	0.219	0.478	0.856	1.242	6.80E-45
MAGI1	0.087	0.139	-0.181	-0.399	0.93	6.98E-45
SLX4	-0.573	-0.117	-0.083	-0.311	-1.023	7.02E-45
UBA7	-0.206	0.146	0.505	0.205	1.343	8.07E-45
FJX1	-0.721	-0.461	-0.252	-0.062	0.507	8.39E-45
ICAM2	-0.259	-0.171	0.195	0.628	0.803	9.20E-45
RCN1	0.222	0.256	0.216	0.533	0.858	1.07E-44
SPAAR	-0.504	-0.486	-0.434	-0.221	-1.254	1.12E-44
CDYL	0.084	-0.001	0.094	0.426	1.031	1.22E-44

INPP5D	-0.19	-0.44	0.236	0.686	0.79	1.22E-44
ZC3H12C	0.981	0.48	0.379	0.326	0.853	1.33E-44
RBMX	0.136	-0.005	-0.107	-0.148	-0.432	1.35E-44
MAOA	0.196	-0.101	-0.418	-0.229	-2.023	1.37E-44
HSD17B2	-0.284	-0.296	-0.018	0.389	0.725	2.14E-44
RASSF8	0.627	0.169	0.059	0.384	0.847	2.46E-44
FIG4	0.044	0.226	0.391	0.755	0.998	2.60E-44
DOT1L	0.019	0.025	-0.152	-0.531	-0.83	3.25E-44
NREP	-0.151	-0.184	0.131	0.269	1.014	3.30E-44
TIPIN	0.266	0.014	-0.34	-0.488	-1.044	3.40E-44
CDCA8	-0.947	-1.042	-0.95	-0.391	-0.772	3.67E-44
SSFA2	0.034	-0.139	-0.078	0.03	0.552	4.03E-44
CDC7	-0.231	-0.2	-0.22	-0.412	-1.261	4.07E-44
FBXO32	1.149	0.772	1.066	1.284	2.27	4.21E-44
EIF4B	0.253	0.185	0.248	0.516	0.74	4.29E-44
WTIP	0.586	-0.004	-0.264	-0.518	-0.281	4.40E-44
FRMD5	-0.344	0.029	0.249	0.405	1.079	5.45E-44
RNPEPL1	0.165	-0.062	-0.005	0.171	0.77	5.68E-44
STX6	0.333	0.405	0.471	0.641	1.15	6.92E-44
ACSS1	-0.199	0.474	1.176	0.905	1.943	7.24E-44
DUSP4	0.297	0.264	0.425	0.453	1.051	7.56E-44
ABC7-42404	0.603	-0.071	-0.739	-0.629	-2.629	7.92E-44
BMX	-0.058	-0.167	0.032	0.407	0.937	7.95E-44
EPHA4	0.198	0.74	0.852	0.657	1.331	8.88E-44
NRGN	0.013	-0.011	-0.019	0.192	1.132	1.03E-43
MARCH2	-0.152	-0.069	0.082	0.115	0.767	1.26E-43
E2F7	0.809	0.559	0.455	0.028	0.696	1.42E-43
CYP51A1	0.347	-0.243	-0.67	-0.547	-0.189	1.55E-43
CDYL2	0.722	-0.021	0.098	0.235	0.786	1.91E-43
CXCL5	0.026	0.853	0.856	0.919	2.548	2.32E-43
SYTL4	-0.043	0.001	-0.359	-0.617	-0.834	2.32E-43
SPRY1	-0.167	0.079	-0.368	-0.897	-1.087	2.32E-43
TPP1	0.203	0.17	0.194	0.461	1.02	2.47E-43
PCBD1	-0.11	-0.115	0.079	0.277	0.772	3.24E-43
RMI1	-0.062	-0.008	-0.265	-0.431	-0.957	3.40E-43
ZMAT3	0.888	1.07	0.796	0.848	1.082	3.46E-43
SMC5	0.259	0.419	0.172	-0.114	-0.293	3.50E-43
SOX12	-0.165	-0.431	0.108	0.41	1.062	3.84E-43

ADIPOR2	0.128	-0.141	-0.478	-0.638	-1.048	4.40E-43
MN1	1.756	0.574	0.789	0.872	1.431	4.62E-43
SH3RF2	-0.073	-0.595	-0.431	0.17	1.509	4.63E-43
NR4A1	1.999	1.172	0.879	0.446	0.388	5.41E-43
SNN	0.348	0.008	-0.373	-0.346	0.774	6.18E-43
RASA3	0.365	-0.243	0.124	0.542	0.752	6.54E-43
C16orf74	-0.152	-0.299	0.043	0.199	0.887	6.60E-43
JAG1	0.705	0.194	-0.098	-0.256	0.865	6.68E-43
SIAE	0.164	0.015	0.072	0.327	0.915	6.74E-43
IRS2	-0.258	0.288	-0.266	-0.453	-1.526	8.55E-43
ADTRP	0.117	-0.138	0.346	0.313	1.375	8.83E-43
KIF23	-0.755	-0.908	-0.539	-0.208	-0.649	9.95E-43
OSBP2	0.258	0.093	0.285	0.644	1.411	1.04E-42
SLC30A1	0.992	0.397	0.383	0.328	0.642	1.05E-42
KIT	-0.925	0.31	1.18	1.24	2.72	1.08E-42
TNFRSF1B	0.105	-0.373	-0.357	-0.217	-1.109	1.11E-42
NES	-0.221	-0.074	0.098	-0.086	0.725	1.15E-42
SEMA6D	-0.516	0.779	0.337	-0.125	-0.603	1.16E-42
TNS1	0.397	-0.193	-0.014	0.192	0.772	1.28E-42
PARP12	0.03	-0.067	0.245	0.405	1.034	1.64E-42
TMEM25	-0.387	-0.101	0.122	0.326	0.827	1.66E-42
RUNX1	-0.042	-0.137	0.082	0.174	0.686	1.87E-42
CD163L1	0.077	-0.104	0.134	0.241	0.96	1.89E-42
RP1-140K8.5	-0.375	-0.179	0.379	0.305	1.009	1.94E-42
DUSP23	-0.259	-0.363	-0.35	-0.341	-1.218	2.03E-42
SOX13	0.6	-0.428	-0.058	-0.124	-0.452	2.26E-42
COL4A5	0.176	0.167	0.305	0.37	0.909	2.32E-42
CD276	0.242	0.019	-0.028	0.322	1.025	3.11E-42
CHAF1B	0.553	0.225	-0.276	-0.371	-0.819	3.39E-42
RAB29	-0.185	-0.005	0.241	0.413	0.758	3.47E-42
SSR1	0.19	0.007	-0.114	-0.012	-0.525	3.76E-42
ZGRF1	-0.009	0.182	-0.308	-0.76	-0.948	3.82E-42
SYNM	0.713	0.756	0.141	-0.364	0.069	4.79E-42
NR3C1	1.286	0.026	-0.094	0.355	0.108	4.86E-42
SLFN5	0.438	0.627	0.46	0.558	1.117	5.13E-42
DAPK3	0.345	0.303	0.263	0.483	1.007	5.21E-42
SCD	0.525	-0.157	-0.66	-0.657	0.098	5.80E-42
ADAMTSL1	-0.185	-0.262	-0.365	-0.339	0.733	6.00E-42

SEMA6B	-0.319	-0.226	0.096	0.541	1.058	6.40E-42
ACKR1	0.06	0.113	0.025	-0.488	-1.457	7.79E-42
TMEM30A	0.224	0.28	0.16	0.418	0.708	7.88E-42
MXRA7	0.083	0.029	0.057	0.202	0.656	8.58E-42
TOX	-0.502	-0.015	-0.426	-0.202	-1.606	8.79E-42
PEG10	0.205	-0.143	-0.183	0.132	1.196	9.50E-42
ISCU	0.212	0.546	0.689	0.611	0.833	9.62E-42
PKMYT1	0.144	0.15	-0.198	-0.356	-1.116	9.69E-42
DONSON	0.179	0.171	-0.054	-0.11	-0.676	1.37E-41
SMURF1	0.622	0.239	0.117	0.062	0.664	1.40E-41
MAPK14	0.147	-0.232	-0.432	-0.257	-0.446	1.57E-41
ADA	0.308	0.087	0.096	0.224	1	1.58E-41
RP11-597D1	-0.336	0.528	0.81	0.709	1.223	1.69E-41
GFOD1	0.346	-0.311	-0.484	-0.665	-0.604	1.94E-41
DUSP1	-0.314	0.536	0.553	0.4	0.576	2.09E-41
PDPN	0.125	-0.125	-0.175	-0.062	1.16	2.31E-41
CYB561	0.256	-0.259	-0.229	-0.364	-0.512	2.37E-41
GALNT10	0.258	0.021	0.002	0.29	0.801	2.44E-41
SLC12A7	-0.209	0.38	0.168	-0.169	0.497	2.49E-41
ERGIC3	0.042	0.011	0.034	0.237	0.442	2.53E-41
APAF1	0.385	0.292	0.25	0.253	0.704	2.90E-41
DYRK1B	-0.03	0.048	0.204	0.398	1.005	3.10E-41
AIF1L	-0.207	-0.154	0.174	0.365	0.839	3.42E-41
HRH1	0.512	0.166	0.451	0.597	0.973	3.78E-41
PLEKHA6	-0.018	0.344	1	0.988	1.227	5.01E-41
IFFO2	0.486	-0.062	0.265	0.562	0.904	5.98E-41
LXN	-0.184	-0.173	-0.068	-0.055	1.103	6.32E-41
ADM	-0.582	-0.092	-0.895	-1.161	-1.681	6.89E-41
ARHGAP5	0.4	0.206	0.092	0.208	0.921	7.10E-41
HSPA4L	0.554	1.116	1.236	1.591	1.511	7.21E-41
ZNFX1	0.795	0.448	0.508	0.479	0.982	7.21E-41
HRCT1	0.128	0.052	-0.188	0.013	-1.53	7.80E-41
MTMR4	-0.756	-0.178	-0.197	-0.364	-0.709	8.97E-41
REV3L	0.489	0.807	0.705	0.693	0.997	8.99E-41
DYRK3	0.735	0.179	0.465	0.803	1.196	1.01E-40
KIAA1549	-0.001	0.223	0.191	0.23	0.92	1.02E-40
MGAT4B	0.14	0.123	0.019	0.127	0.711	1.24E-40
KIAA1715	-0.035	0.188	-0.076	-0.29	-1.045	1.32E-40

KNSTRN	-0.835	-0.878	-0.691	-0.312	-0.398	1.44E-40
HIF3A	-0.636	-0.41	0.117	-0.272	-1.101	1.44E-40
PIR	0.167	0.016	0.008	0.283	0.742	1.84E-40
PSMC3IP	0.288	0.108	-0.188	-0.582	-0.825	1.88E-40
TMCO6	-0.504	-0.111	-0.082	-0.608	-1.05	1.88E-40
ACVRL1	-0.056	-0.077	0.133	0.284	0.512	2.07E-40
SRGAP1	0.406	0.379	0.706	0.667	1.094	2.48E-40
SNX33	-0.166	0.062	0.065	0.321	0.783	2.54E-40
IQCJ-SCHIP	1.065	0.489	0.649	0.771	1.589	2.87E-40
RASGRF2	0.033	-0.176	-0.357	0.04	0.742	2.94E-40
PARP10	-0.464	0.406	0.833	0.528	1.018	3.49E-40
BIN1	0.177	-0.158	-0.006	0.347	0.774	3.52E-40
PEBP1	0.162	-0.005	-0.007	0.328	0.781	4.30E-40
SPIN4	0.068	-0.393	-0.456	-0.249	-1.399	4.33E-40
FDFT1	0.44	-0.235	-0.618	-0.497	0.134	4.43E-40
C9orf142	-0.25	-0.138	-0.189	-0.597	-1.128	4.58E-40
CDC14A	-0.01	0.315	-0.54	-0.833	-0.401	5.58E-40
OTUD3	0.22	0.039	0.024	-0.66	-0.758	5.61E-40
CST3	0.103	-0.058	-0.056	0.255	0.728	5.70E-40
ACSL3	0.189	-0.192	-0.257	0.047	0.664	6.02E-40
KDM5B	0.148	0.171	0.151	0.348	0.955	6.13E-40
MAP3K2	0.45	0.347	0.109	-0.216	-0.392	6.26E-40
RGS3	-0.308	-0.04	-0.065	-0.027	0.762	6.86E-40
ITFG1	0.194	0.13	-0.007	0.4	0.698	6.95E-40
TRIM8	0.338	0.084	0.236	0.55	0.968	7.67E-40
TMEM65	0.243	-0.016	0.047	0.318	0.725	7.74E-40
FLRT2	-0.037	0.087	0.064	0.028	1.017	8.75E-40
SPTBN2	0.289	-0.066	-0.067	-0.212	0.928	8.80E-40
EGFL7	-0.533	-0.363	-0.366	-0.746	-1.174	9.46E-40
LRP10	0.15	0.202	0.222	0.674	1.054	1.08E-39
WDFY4	-0.084	-0.241	0.059	0.612	0.886	1.10E-39
RP11-398C1	0.207	0.049	-0.263	-0.473	-0.759	1.45E-39
ARHGAP11A	-0.789	-0.752	-0.652	-0.558	-1.044	1.48E-39
SCRN1	0.161	-0.02	0.113	0.518	0.956	1.55E-39
RP11-680F8	-0.302	0.376	0.809	0.599	1.927	1.62E-39
NEK6	0.269	-0.352	0.101	0.342	0.359	1.63E-39
SH3BGRL2	-0.231	-0.028	-0.015	0.13	-0.561	1.67E-39
KDM4B	-0.12	0.375	0.419	0.55	0.922	1.86E-39

HDAC1	0.241	-0.041	-0.027	0.089	0.612	1.94E-39
EXOC3L1	0.318	-0.166	-0.396	-0.485	-1.692	1.96E-39
NIPSNAP1	0.267	0.02	-0.09	0.307	1.008	2.07E-39
GAB1	-0.063	0.358	-0.22	-0.634	0.647	2.16E-39
G2E3	-0.935	-0.835	-0.799	-0.53	-0.297	2.22E-39
RTEL1	-0.336	0.08	0.051	-0.445	-1.129	2.29E-39
ZSWIM6	0.335	0.157	0.778	0.549	1.343	2.76E-39
CRY1	0.172	0.01	-0.333	-0.647	-0.284	2.78E-39
SSH2	-0.481	-0.093	-0.131	-0.132	0.493	2.83E-39
ERF	0.514	-0.383	-0.435	-0.348	-0.338	2.87E-39
EIF1AD	0.235	0.105	-0.05	-0.162	-0.586	3.02E-39
CTC1	-0.598	0.252	0.449	0.145	0.214	3.73E-39
PTCHD4	-0.282	1.055	0.919	1.001	1.729	3.89E-39
ASB9	0.052	-0.173	-0.111	0.176	1.475	3.99E-39
APOD	0.28	0.737	0.982	1.319	1.961	4.04E-39
DSE	0.179	0.237	0.129	0.102	1.276	4.04E-39
RECQL4	-0.203	0.018	-0.203	-0.836	-1.196	4.05E-39
MEGF6	-0.614	0.001	0.473	0.203	1.079	4.19E-39
GRB14	-0.087	-0.325	-0.433	-0.839	-1.284	4.37E-39
IER3	0.046	0.276	0.236	0.233	0.7	4.56E-39
YPEL1	-0.52	0.145	0.273	0.618	1.859	4.58E-39
STK17B	-0.041	-0.438	-0.676	-0.506	-1.057	4.67E-39
LAPTM5	0.259	0.103	0.092	0.5	0.972	4.68E-39
DPF3	-0.523	0.143	0.774	0.584	1.058	4.78E-39
FAM69B	0.003	-0.429	-0.615	-0.7	-0.418	5.13E-39
ID1	0.955	-0.361	0.003	0.206	-1.865	5.48E-39
BCL6B	-0.728	0.208	0.147	-0.443	0.602	5.94E-39
SUGCT	0.311	0.352	0.736	1.19	2.108	6.30E-39
ARMC9	-0.09	-0.25	0.006	0.326	1.212	6.46E-39
F2RL3	0.74	0.467	1.161	1.294	2.249	6.57E-39
IL7R	-0.084	0.726	0.134	-0.198	0.027	6.91E-39
SGCG	0.169	0.13	-0.553	-0.355	-2.599	7.57E-39
CCRL2	-0.507	-0.069	0.603	0.868	0.551	7.77E-39
AP000695.4	0.243	-0.076	0.555	0.737	1.467	8.28E-39
RP11-359E1	0.215	0.942	0.986	1.261	2.138	8.89E-39
RP11-304F1	-0.24	-0.11	-0.111	-0.89	-1.9	9.27E-39
NUP85	0.083	0.062	-0.087	-0.242	-0.58	9.70E-39
ZMYND19	0.002	-0.053	-0.509	-0.783	-0.594	1.04E-38

CTC-316M18	0.286	0.013	0.137	0.646	1.432	1.05E-38
PTTG1IP	0.048	-0.065	-0.123	0.162	0.498	1.06E-38
ERMP1	0.132	0.009	0.06	0.386	1	1.07E-38
TNKS1BP1	0.019	0.104	0.21	0.248	0.773	1.11E-38
NCAPG	-0.272	-0.472	-0.527	-0.167	-0.776	1.15E-38
PCYOX1	0.174	0.128	0.16	0.597	1.112	1.23E-38
MMP19	-0.467	0.251	0.474	0.276	0.904	1.46E-38
SELE	1.765	1.732	0.2	-0.316	2.629	1.52E-38
RND1	1.821	1.587	-0.099	-0.025	2.075	1.67E-38
PCDH10	0.083	-0.004	-0.427	-0.253	-1.174	1.76E-38
ETV4	-0.376	-0.225	0.041	0.236	0.642	1.81E-38
RP11-168P8	0.226	-0.652	-0.891	-0.769	-2.313	1.81E-38
ARNTL2	0.066	0.138	0.236	0.305	0.669	1.92E-38
DNMBP	-0.288	-0.032	0.124	0.139	0.429	1.96E-38
IFIH1	-0.001	1.033	0.909	1.054	2.07	2.14E-38
SC5D	0.491	-0.064	-0.516	-0.461	-0.386	2.15E-38
SFRP1	0.265	0.104	0.026	0.42	1.077	2.40E-38
TRAF5	-0.223	-0.132	0.339	-0.058	1.319	2.50E-38
LTK	0.004	-0.015	-0.238	-0.722	-2.437	2.50E-38
VEGFC	0.388	0.288	-0.477	-0.23	-0.057	2.51E-38
PDE2A	0.297	0.034	0.369	0.489	1.312	2.56E-38
APOL2	-0.065	0.348	0.702	0.382	0.711	2.56E-38
MAP4	0.078	0.041	0.075	0.097	0.588	2.71E-38
EIF1	0.468	0.153	0.035	0.167	-0.107	3.29E-38
AUP1	-0.082	0.029	-0.015	-0.257	-0.571	3.90E-38
KLF15	-1.112	-0.349	-0.996	-1.356	-2.67	3.99E-38
TSPAN5	0.109	-0.253	-0.127	0.181	0.675	4.29E-38
MARCH4	0.118	-0.093	0.291	0.831	0.963	4.41E-38
AC073130.1	-0.638	0.144	0.545	0.668	-0.086	4.43E-38
FUCA2	0.147	0.15	0.127	0.413	0.785	4.57E-38
LMBRD2	-0.106	0.152	-0.095	-0.209	-0.928	5.09E-38
UCK2	0.323	-0.089	0	0.284	0.725	5.14E-38
HBP1	0.315	0.236	0.424	0.571	0.863	5.41E-38
CDK6	0.442	0.3	0.078	-0.058	0.605	5.86E-38
CLIP2	-0.315	0.078	0.381	0.293	0.801	7.21E-38
TMEM233	0.187	-0.365	0.084	0.355	1.226	8.38E-38
EPHB4	0.563	-0.33	-0.539	-0.193	-0.342	9.01E-38
CMTM8	0.307	-0.335	-1.062	-1.088	-1.162	9.19E-38

CLSPN	0.572	0.267	-0.054	-0.213	-0.579	9.35E-38
KIF22	-0.431	-0.428	-0.438	-0.329	-1	9.45E-38
STC2	-0.192	-0.235	0.001	0.203	-0.725	9.67E-38
ZBTB2	0.7	-0.392	-0.57	-0.386	0.169	9.88E-38
PLXNB1	0.346	0.593	0.606	0.185	1.155	1.01E-37
RND3	0.433	-0.094	-0.233	-0.146	-0.323	1.03E-37
CDCA3	-1.161	-1.101	-0.764	-0.838	-1.505	1.07E-37
EIF3H	0.14	0.085	0.089	0.3	0.516	1.12E-37
SHE	0.419	-0.261	-0.332	-0.193	-0.765	1.50E-37
CCDC130	-0.434	0.158	-0.004	-0.771	-0.857	1.57E-37
ZNF185	0.345	0.061	0.211	0.699	1.148	1.67E-37
SRRT	0.108	-0.069	-0.164	-0.385	-0.6	1.76E-37
RNF149	-0.066	-0.011	0.205	0.258	0.756	2.22E-37
GTSE1	-0.835	-0.892	-0.61	-0.281	-0.583	2.61E-37
GINS1	0.054	0.009	-0.194	-0.277	-0.966	2.95E-37
HAUS5	-0.329	0.051	-0.032	-0.552	-1.133	2.99E-37
MKI67	-0.9	-0.761	-0.542	-0.411	-1.14	3.01E-37
ASAP2	0.431	0.143	0.329	0.539	0.715	3.35E-37
PPP1R15B	0.187	0.135	-0.133	-0.237	-0.6	3.84E-37
RAB3D	-0.242	-0.138	0.233	0.592	0.754	4.26E-37
RAB30	0.857	0.125	-0.009	0.216	0.069	4.45E-37
TPST2	0.003	-0.076	-0.066	-0.069	0.733	4.49E-37
RP11-137N2	0.216	-0.224	-0.365	-0.554	-0.677	4.57E-37
PRIM1	0.136	0.113	-0.158	-0.173	-1.21	4.63E-37
BCS1L	-0.31	-0.075	-0.02	-0.453	-0.997	5.46E-37
ZFPM1	1.234	0.696	0.768	0.419	0.972	5.69E-37
MAVS	-0.292	0.052	0.247	0.185	0.356	6.26E-37
MPZL2	-0.235	-0.164	-0.157	0.085	0.611	6.49E-37
KIAA1549L	0.081	-0.4	-0.101	0.29	0.778	8.10E-37
SAPCD2	-0.751	-1.356	-0.97	-0.404	-0.287	8.93E-37
KBTBD2	0.596	0.042	-0.022	0.035	-0.114	8.96E-37
CENPF	-0.779	-1.039	-0.728	-0.337	-0.306	9.15E-37
RHOB	0.867	0.173	-0.235	-0.044	0.259	9.35E-37
ARHGAP33	-0.794	-0.391	-0.362	-1.205	-1.926	1.20E-36
TONSL	-0.021	-0.133	-0.444	-0.929	-1.104	1.27E-36
AKNA	-0.232	0.041	0.203	0.23	0.797	1.77E-36
RAD9A	-0.424	-0.21	-0.281	-1.161	-1.309	1.78E-36
ICOSLG	0.307	1.197	1.432	1.036	1.96	1.79E-36

FAM208A	-0.282	-0.03	0.244	0.422	0.259	1.79E-36
PRR11	-0.274	-0.665	-0.62	-0.139	0.361	2.02E-36
RP11-322E1	-0.784	-0.217	0.617	0.539	1.779	2.18E-36
BTN3A3	-0.099	-0.045	0.426	0.438	1.147	2.18E-36
TSEN54	-0.142	-0.069	-0.299	-0.563	-1.035	2.21E-36
BACH1	0.23	0.279	0.275	0.53	0.998	2.66E-36
EMP1	0.387	-0.205	-0.33	-0.078	-0.645	2.75E-36
SPRED1	0.574	-0.081	-0.025	0.163	0.714	2.90E-36
S1PR3	0.04	0.495	0.506	0.543	0.9	2.91E-36
NOP58	0.327	0.044	-0.195	-0.101	-0.4	3.10E-36
DHRS9	0.097	-0.059	-0.31	-0.249	-1.136	3.21E-36
CXCL2	0.752	0.74	-0.022	-1.092	0.756	3.55E-36
ERN1	-0.352	0.484	0.365	-0.057	0.072	3.69E-36
PPFIBP1	0.028	0.369	0.403	0.406	0.594	4.54E-36
PMAIP1	0.575	0.715	0.526	0.14	1.243	4.79E-36
CLDN1	0.386	1.205	1.394	1.628	2.315	5.83E-36
WDR90	-0.436	-0.002	-0.128	-1.049	-1.35	6.54E-36
MET	0.285	-0.06	-0.208	0.175	0.799	6.78E-36
TMEM123	0.311	0.1	-0.176	0.055	-0.844	7.48E-36
SRSF10	0.033	-0.107	-0.098	-0.319	-0.497	7.59E-36
JMJD1C	-0.291	0.081	0.2	0.133	0.752	7.63E-36
FMNL3	0.104	0.113	0.347	0.042	0.842	7.79E-36
FLNB	0.041	-0.069	0.132	0.253	0.924	7.81E-36
CSNK1G1	0.013	0.306	0.23	0.186	0.66	7.85E-36
BRCA2	0.082	0.052	-0.186	-0.309	-0.823	9.25E-36
EHD1	0.487	0.232	0.477	0.945	1.364	9.51E-36
RBM26	-0.365	0.079	0.073	-0.161	-0.497	9.84E-36
ASTN2	0.496	1.05	1.878	1.812	2.098	1.11E-35
KCTD15	0.528	-0.124	-0.16	-0.266	-0.223	1.12E-35
ATAD2	0.436	0.068	-0.403	-0.426	-0.712	1.19E-35
AHR	0.896	0.441	-0.216	-0.138	-0.401	1.23E-35
PER1	-0.223	-0.209	-0.479	-0.751	-1.218	1.32E-35
GATA6	0.883	-0.232	-0.178	0.214	-0.203	1.33E-35
KRI1	-0.507	-0.052	-0.031	-0.279	-0.976	1.36E-35
DDX39A	-0.067	-0.172	-0.265	-0.243	-0.716	1.61E-35
TMPO	-0.03	-0.304	-0.429	-0.068	-0.865	1.73E-35
WDR48	0.19	0.491	0.451	0.542	0.89	1.76E-35
COTL1	0.19	0.072	0.033	0.349	0.795	1.79E-35

KIF3C	0.196	-0.203	-0.152	0.222	0.885	1.87E-35
RP11-150O1	0.047	-0.28	0.353	0.419	1.943	1.96E-35
PTPRF	-0.01	-0.035	0.161	0.444	1.002	1.97E-35
CDCA2	-1.145	-1.069	-0.889	-0.214	-0.512	1.99E-35
DGKD	-0.156	0.281	-0.06	-0.624	-0.365	2.13E-35
TNFSF18	-0.978	0.252	0.425	0.086	1.224	2.15E-35
CSRP1	0.261	-0.012	0.031	0.362	0.884	2.16E-35
NR2C2AP	0.14	-0.088	-0.495	-0.731	-0.974	2.29E-35
CTHRC1	0.171	-0.192	-0.497	-0.653	0.568	2.34E-35
RP11-874J1:	-0.222	0.251	0.399	0.418	1.91	2.48E-35
FIGNL1	-0.127	0.023	-0.113	-0.363	-0.64	2.59E-35
RGCC	0.21	-0.079	0.149	-0.071	1.078	2.83E-35
FANCI	0.084	0.21	0.135	0.229	-0.602	3.31E-35
DENND5B	0.469	-0.34	-0.5	-0.204	0.556	3.36E-35
PLCG1	-0.131	-0.174	-0.057	-0.218	0.389	3.79E-35
MYCT1	0.449	0.291	0.276	0.558	0.704	3.96E-35
SERPINB1	0.294	0.082	0.217	0.634	0.935	4.05E-35
BTBD3	0.236	0.021	-0.344	-0.352	-0.804	4.13E-35
NOG	1.557	-0.278	0.083	0.402	2.14	4.34E-35
FAS	0.871	1.51	1.348	0.91	1.263	4.60E-35
ITGA3	0.182	0.099	0.272	0.64	1.027	4.69E-35
STARD10	-0.102	0.268	0.645	0.615	1.224	4.75E-35
SOX18	0.578	-0.377	-0.394	-0.286	0.904	4.75E-35
CHSY3	0.528	-0.019	-0.811	-0.99	-0.906	4.91E-35
RNF145	0.21	0.032	-0.428	-0.48	-0.24	4.97E-35
FBXO38	0.112	0.212	0.329	0.458	0.657	5.42E-35
TCF7L2	1.162	0.07	0.158	0.365	0.462	5.76E-35
JARID2	0.141	-0.257	0.198	0.427	0.84	6.49E-35
AC002055.4	-0.087	0.301	0.542	0.653	0.676	6.49E-35
ARHGAP21	1.061	0.392	0.242	0.292	0.537	6.95E-35
C8orf4	1.822	0.212	-0.262	-0.414	0.8	8.18E-35
CREBL2	0.128	0.193	0.363	0.63	0.681	9.16E-35
PHTF2	-0.121	-0.064	-0.322	-0.5	-0.727	9.22E-35
GAS6-AS1	0.679	1.774	2.215	1.635	1.896	1.18E-34
C2CD2	-0.364	0.098	0.473	0.18	0.891	1.21E-34
NUF2	-0.68	-0.773	-0.557	-0.27	-0.916	1.30E-34
CCNA1	-0.14	-0.322	-0.009	0.526	0.932	1.35E-34
SORT1	0.141	0.02	0.063	0.39	1.3	1.51E-34

BORA	-1.358	-1.147	-0.977	-0.544	-0.653	1.55E-34
LRIG1	-0.347	0.164	0.292	0.147	1.086	1.61E-34
DCUN1D3	0.22	0.412	0.309	0.23	0.874	1.66E-34
LDB2	0.135	-0.506	-0.703	-0.576	-1.139	1.74E-34
ADAMTS4	-0.305	-0.036	0.202	0.095	0.764	1.78E-34
BCL2L13	0.25	0.105	0.209	0.486	0.668	1.84E-34
MAPK12	-0.209	-0.107	-0.207	-0.7	-0.784	1.93E-34
COL4A6	0.231	0.088	0.158	0.355	0.871	1.99E-34
WASF3	-0.626	-0.376	-0.343	-0.424	-0.715	2.01E-34
TGFBRAP1	-0.059	0.174	0.395	0.518	0.9	2.04E-34
EFNA1	-0.686	0.125	0.259	0.204	1.464	2.12E-34
RAC2	0.224	-0.23	-0.044	0.452	0.962	2.19E-34
GPR161	0.155	0.01	-0.046	0.046	0.77	2.78E-34
RGP1	-0.375	0.011	0.38	0.276	0.18	2.80E-34
SEMA3A	0.084	0.453	0.33	0.53	1.548	3.40E-34
RHOC	0.063	0.032	0.057	0.293	0.647	3.63E-34
TLE1	0.507	-0.297	-0.149	0.193	0.467	3.70E-34
LIMS2	0.11	-0.183	-0.152	-0.162	-0.837	3.91E-34
NUP58	-0.017	0.041	-0.314	-0.314	-0.514	3.99E-34
LGALS1	0.074	-0.11	0.007	0.171	0.491	4.34E-34
PRKACB	0.315	-0.048	-0.38	-0.202	-0.292	4.34E-34
ACSL5	0.102	0.007	0.153	0.39	0.939	4.38E-34
DLGAP5	-0.492	-0.965	-1.025	-0.297	-0.153	4.46E-34
WWTR1	0.143	-0.275	-0.419	-0.454	-0.452	4.76E-34
GPX3	0.041	0.074	0.243	0.648	0.833	5.07E-34
GAMT	-0.091	0.056	0.269	0.552	1.049	5.63E-34
TCAIM	-0.024	0.252	0.481	0.605	0.664	6.27E-34
ZNF358	-0.106	0.024	0.003	0.221	0.892	6.40E-34
SCARF1	-0.641	-0.245	0.109	-0.008	0.412	6.86E-34
MCM6	0.583	0.067	-0.575	-0.527	-0.765	6.87E-34
GAS6-AS2	0.104	0.36	0.769	0.779	2.108	6.97E-34
CALM1	0.028	-0.051	-0.008	0.163	0.506	7.02E-34
BBS10	-0.922	-0.319	-0.217	-0.265	-0.592	7.04E-34
MYEF2	-0.643	-0.191	0.266	0.167	0.7	7.17E-34
LIMCH1	0.126	-0.23	-0.917	-1.293	-1.819	7.77E-34
FAT4	0.15	-0.067	-0.507	-0.446	-1.112	8.32E-34
FAM89A	0.016	-0.059	0.168	0.034	1.396	9.62E-34
TAPBP	0.108	0.284	0.223	0.401	0.868	1.00E-33

ITPR3	-0.115	-0.192	0.305	-0.011	0.712	1.00E-33
HEXB	0.132	0.059	0.012	0.235	0.575	1.01E-33
MFSD12	-0.023	0.001	-0.008	-0.079	0.666	1.03E-33
ZMIZ1	0.595	-0.398	0.169	0.603	0.598	1.03E-33
FRMD4B	0.69	-0.081	-0.046	0.159	0.578	1.11E-33
FZD1	0.633	-0.265	-0.73	-0.378	-0.701	1.14E-33
RPL3	0.062	0.134	0.17	0.305	0.511	1.22E-33
FKBP1C	0.238	-0.082	-0.103	0.159	0.681	1.24E-33
PHF10	0.146	0.094	-0.091	-0.302	0.481	1.24E-33
GALNT7	0.35	0.216	0.117	0.401	0.914	1.28E-33
CMTM3	0.147	-0.113	0.06	0.179	0.642	1.41E-33
YIPF5	0.5	-0.033	-0.324	-0.231	-0.435	1.55E-33
FAM126A	-0.111	-0.004	-0.029	0.056	0.514	1.56E-33
PHEX	0.036	-0.209	0.041	0.335	1.335	1.59E-33
HECA	0.868	0.201	0.192	0.366	0.667	1.60E-33
PAXIP1	0.17	0.054	-0.113	-0.413	-0.636	1.62E-33
LAMP3	-0.031	0.198	0.261	0.722	1.102	1.69E-33
ROBO3	-0.437	0.126	0.23	-0.26	-1.134	1.70E-33
ZDHHC23	0.5	0.217	0.037	-0.354	-1.159	1.70E-33
MMS22L	0.301	0.144	-0.256	-0.328	-0.551	1.77E-33
KIAA1217	-0.157	0.106	0.211	0.209	1.757	1.89E-33
ITM2B	0.235	0.138	0.019	0.271	0.674	1.98E-33
NAV2	-0.125	-0.26	0.164	0.136	0.675	2.04E-33
BCL2L1	0.49	0.528	0.68	1.035	1.091	2.05E-33
NCR3LG1	0.463	0.266	0.116	-0.306	-0.507	2.16E-33
TDG	0.377	0.138	-0.113	-0.231	-0.455	2.18E-33
CDKN1B	-0.677	-0.178	-0.237	-0.16	-0.719	2.24E-33
ADAM17	0.212	0.197	0.179	0.317	0.63	2.30E-33
GCNT1	-0.037	-0.472	-0.528	-0.191	-0.959	2.35E-33
HIST1H2AC	0.585	0.819	1.414	1.663	1.625	2.37E-33
DENND6B	-0.253	0.025	0.249	-0.263	1.624	2.37E-33
REEP3	0.015	0.015	0.019	0.029	0.695	3.00E-33
ATRX	0.033	0.123	0.182	0.111	0.632	3.55E-33
IGHMBP2	-0.308	0.14	-0.089	-0.423	-0.677	3.77E-33
ZNF438	-0.103	-0.086	0.376	0.567	0.713	3.80E-33
CDCA5	0.181	0.002	-0.125	-0.185	-0.731	3.89E-33
DUSP16	0.357	0.537	0.558	0.617	1.276	4.04E-33
SPOCD1	-0.213	-0.14	0.222	0.047	0.814	4.08E-33

NADSYN1	-0.062	0.413	0.536	0.239	0.666	4.10E-33
LAMC1	0.317	0.22	0.154	0.476	1.129	4.14E-33
NUAK1	0.228	0.112	-0.33	-0.491	-0.658	4.30E-33
DOCK1	0.35	0.115	0.116	0.444	0.83	4.56E-33
LYL1	0.336	-0.681	-0.293	0.155	-0.114	4.58E-33
PCDH1	0.7	-0.096	-0.185	0.183	0.621	4.76E-33
WASF2	0.07	0.2	0.223	0.355	0.827	4.93E-33
DERA	0.075	0.06	-0.158	-0.26	-0.743	5.40E-33
USP54	-0.155	-0.026	-0.145	0.078	0.796	5.46E-33
HMG20A	0.057	0.119	0.15	0.493	0.836	6.05E-33
TGM2	0.148	-0.123	0.058	0.701	1.255	7.54E-33
DNAJB11	0.303	0.142	-0.068	0.169	-0.56	7.63E-33
FAM171A1	0.21	0.015	-0.043	0.348	0.835	7.83E-33
PDGFRL	0.056	0.087	0.302	0.665	1.186	7.90E-33
JADE1	-0.478	-0.296	-0.237	-0.202	-0.882	8.06E-33
MSH6	0.714	-0.05	-0.582	-0.441	-0.294	8.13E-33
RRP15	-0.209	-0.047	-0.097	-0.172	-0.835	9.43E-33
ARHGEF2	-0.481	0.051	0.246	0.253	0.487	9.49E-33
PCNT	-0.278	0.079	0.056	-0.504	-0.784	1.01E-32
CCHCR1	-0.155	-0.087	-0.079	-0.265	-1.019	1.08E-32
SNHG1	-0.081	-0.127	-0.187	-1.043	-1.406	1.09E-32
CLCN5	-0.017	0.046	-0.253	-0.396	-1.017	1.18E-32
NDC80	-0.505	-0.85	-0.606	0.003	-0.522	1.19E-32
PLK3	1.089	0.695	0.764	0.265	0.64	1.21E-32
PALLD	-0.095	0.102	0.056	0.023	0.499	1.25E-32
PRAG1	1.403	0.76	0.785	0.785	0.911	1.27E-32
SH3BGRL	0.153	0.003	0.067	0.206	0.641	1.28E-32
ATRIP	-0.064	0.133	-0.112	-0.277	-0.86	1.34E-32
RASAL2	0.045	-0.096	-0.154	-0.121	0.627	1.44E-32
DHX34	-0.691	-0.195	-0.085	-0.461	-0.648	1.45E-32
ZNF532	0.355	0.048	0.085	0.23	0.61	1.66E-32
RAI14	0.276	-0.099	0.249	0.499	0.603	1.79E-32
CC2D1B	-0.247	0.114	0.09	-0.194	-0.552	1.86E-32
AKAP2	-0.247	0.151	0.31	0.254	1.103	2.14E-32
SRC	0.19	0.317	0.218	0.434	0.894	2.26E-32
TRIM44	0.194	0.029	0.155	0.214	0.594	2.36E-32
RIBC2	-0.222	0.055	0.234	-0.308	-2.195	2.36E-32
SUSD6	0.434	0.442	0.399	0.086	0.738	2.40E-32

PDE4D	0.821	0.971	0.666	0.53	0.906	2.43E-32
PLEKHA8	-0.25	0.207	0.29	0.207	0.625	2.47E-32
MMP15	0.168	-0.217	-0.107	-0.059	1.242	2.65E-32
RP4-564F22	-0.37	-0.259	-0.34	-0.976	-1.101	2.70E-32
SYNGAP1	-0.965	-0.131	0.19	-0.736	-0.913	2.74E-32
RAD1	0.078	-0.003	-0.23	-0.342	-0.658	2.76E-32
ABCA3	-0.18	-0.116	0.324	0.644	1.043	2.91E-32
KIAA1522	0.018	0.172	0.387	0.291	1.097	3.03E-32
PRSS12	0.139	0.155	0.266	0.554	1.093	3.38E-32
KIF15	-0.67	-0.46	-0.327	-0.156	-0.99	3.81E-32
IFNAR1	-0.003	0.183	0.172	0.368	0.589	4.06E-32
TIE1	-0.063	-0.112	-0.025	0.033	0.523	4.06E-32
SDC4	0.537	0.487	0.064	-0.038	0.641	4.32E-32
BTN3A1	-0.133	-0.027	0.468	0.413	1.023	4.37E-32
CALD1	0.345	0.024	0.017	0.274	0.953	4.91E-32
SYNPO	0.211	-0.05	0.289	0.265	0.507	4.96E-32
EML1	0.588	-0.204	-0.421	0.043	0.186	5.13E-32
APCDD1L	0.363	-0.027	-0.248	0.193	0.916	5.81E-32
CHST3	1.008	-0.221	-0.188	-0.088	-0.158	5.81E-32
REXO2	0.171	0.035	0.022	0.265	0.545	6.00E-32
MON1B	-0.346	0.091	0.239	0.279	0.431	6.25E-32
ZNF335	-0.115	0.1	-0.081	-0.629	-0.822	6.54E-32
ENOX1	0.275	-0.216	-0.453	-0.331	0.93	6.74E-32
EIF2AK4	0.151	0.071	0.215	0.505	0.793	7.31E-32
TSPAN15	0.042	-0.37	-0.571	-0.467	-0.255	7.45E-32
HBEGF	0.67	0.57	0.385	0.402	0.754	7.60E-32
LYN	0.498	-0.175	-0.656	-0.328	-0.164	7.60E-32
MBP	-0.024	0.244	1.004	1.693	2.13	9.10E-32
BTN3A2	-0.127	0.033	0.173	0.345	0.815	9.12E-32
TCERG1	-0.119	-0.056	-0.13	-0.33	-0.528	9.94E-32
RP11-255G1	-0.178	0.067	0.612	0.837	2.458	1.06E-31
SMIM15	0.057	-0.054	0.056	0.391	0.686	1.10E-31
CBLN2	-0.007	0.669	0.518	0.543	-0.42	1.13E-31
MYRIP	0.555	-0.285	0.38	1.101	1.258	1.15E-31
SCHIP1	-0.085	0.85	1.535	1.281	2.235	1.24E-31
INCENP	-0.743	-0.777	-0.564	-0.203	-0.812	1.25E-31
FOXC2	0.786	-0.23	-0.175	0.249	-0.417	1.26E-31
TMEM140	0.102	-0.034	0.192	0.572	1.78	1.37E-31

CIRBP	-0.251	0.087	-0.107	-0.598	-0.923	1.41E-31
TCF3	0.111	-0.138	-0.211	0.017	0.519	1.46E-31
VARS2	-0.454	-0.161	0.005	-0.653	-0.979	1.54E-31
CYP26A1	-2.08	-0.601	-0.947	-1.879	-2.427	1.59E-31
PSMB9	0.065	0.433	0.738	1.047	1.989	1.61E-31
SERPINB2	0.593	0.117	-0.355	-0.679	-1.505	1.73E-31
PRKAR1B	0.272	0.019	0.549	1.018	1.218	1.75E-31
MMD	0.238	0.12	-0.42	-0.767	-0.83	1.75E-31
MME	0.055	0.024	0.078	0.262	0.847	1.81E-31
TBC1D22B	0.212	0.547	0.44	0.484	0.904	2.17E-31
RNF144B	-0.482	-0.213	-0.411	-0.303	-1.147	2.37E-31
ARHGAP10	0.114	0.323	0.191	0.478	0.988	2.48E-31
SHROOM2	0.163	0.034	0.114	0.478	1.172	2.60E-31
VIM	0.342	0.131	0.061	0.442	1.098	2.80E-31
TUB	-0.131	-0.281	-0.649	-1.038	-1.371	2.88E-31
PLEK2	0.594	-0.15	-0.334	-0.098	0.448	3.07E-31
MGAT5	0.148	-0.187	-0.037	0.384	0.662	3.27E-31
KCTD12	0.321	-0.1	-0.376	-0.168	-1.187	3.39E-31
KHNYN	0.265	0.305	0.531	0.395	0.655	3.42E-31
ORAI3	0.17	0.361	0.505	0.685	1.511	3.66E-31
TLE4	0.549	0.668	0.468	0.558	0.988	4.12E-31
BNIP2	0.35	0.019	0.062	0.313	0.547	4.12E-31
SEMA3D	0.041	0.042	-0.263	-0.199	-1.06	4.22E-31
TACC1	-0.101	-0.131	0.014	0.07	0.485	4.23E-31
NR3C2	-0.363	-0.083	0.141	0.615	0.985	4.48E-31
CBFB	0.6	-0.106	-0.314	-0.18	-0.159	4.49E-31
BCL9L	0.22	-0.204	0.229	0.47	0.817	4.64E-31
RP11-800A3	0.059	-0.104	0.335	0.735	0.875	4.85E-31
TNS2	-0.638	-0.314	-0.003	-0.323	-0.673	5.54E-31
RMND5B	-0.317	0.071	0.358	0.395	0.41	5.58E-31
GTPBP3	-0.446	-0.154	-0.02	-0.418	-0.969	6.61E-31
CADM4	0.185	0.613	0.567	0.73	2.118	6.67E-31
TES	0.755	0.181	0.073	0.412	0.028	7.70E-31
CEP152	-0.289	0.092	0.019	-0.661	-1.049	7.70E-31
UNC5B	1.224	-0.184	-0.838	-0.761	0.022	8.12E-31
AIG1	-0.026	0.087	0.443	0.52	1.054	8.25E-31
C3orf52	1.307	0.868	0.766	0.377	0.613	8.31E-31
ATP8A1	-0.246	-0.037	-0.451	-0.635	-1.667	8.62E-31

CAPG	0.289	0.191	0.12	0.546	1.146	9.16E-31
FOXN2	0.239	0.089	-0.3	-0.311	-0.506	9.52E-31
ASCC3	0.318	0.859	0.795	1.138	1.211	9.55E-31
GOLPH3L	-0.114	0.286	0.302	0.574	0.905	1.00E-30
HSPA13	0.17	-0.027	-0.292	-0.264	-0.638	1.01E-30
AXL	0.1	0.095	0.328	0.672	0.779	1.05E-30
CD44	0.327	0.121	-0.009	0.348	0.817	1.06E-30
PARP4	0.233	-0.003	0.207	0.588	0.924	1.07E-30
PAWR	0.017	0.194	0.371	0.479	0.287	1.08E-30
RARB	0.705	0.361	-0.02	0.754	1.708	1.33E-30
TNFRSF11A	0.151	-0.005	-0.228	-0.326	-1.091	1.33E-30
RIN3	0.294	0.224	0.214	0.464	1.107	1.35E-30
AL035209.2	-1.265	-0.16	-0.179	-1.56	-1.093	1.37E-30
FANCG	-0.08	-0.08	-0.213	-0.408	-0.943	1.42E-30
CHTF18	-0.621	-0.03	-0.195	-0.953	-1.406	1.45E-30
BRPF1	0.353	0.122	-0.041	-0.279	-0.269	1.49E-30
SEMA4B	0.08	0.011	0.428	0.624	1.096	1.68E-30
CASC4	0.1	0.065	-0.022	0.2	0.534	1.92E-30
SPRED3	-0.784	-0.077	0.037	-0.323	-0.262	2.02E-30
EFNA5	0.454	-0.217	-0.24	-0.126	-0.42	2.02E-30
PCGF5	0.046	0.06	0.029	-0.011	-0.532	2.02E-30
CTDSPL2	0.376	0.126	-0.197	-0.117	-0.319	2.06E-30
AC005041.1(	0.283	0.116	0.125	0.647	1.052	2.24E-30
RP11-420G6	-0.127	-0.402	0.281	0.76	1.268	2.27E-30
GNA12	0.263	-0.031	-0.043	0.34	0.808	2.50E-30
INHBA	0.145	0.28	0.016	0.068	-0.599	2.55E-30
LYNX1	0.408	0.854	1.077	1.456	1.792	2.62E-30
COL12A1	0.208	0.318	0.459	0.326	1.487	2.72E-30
RNF38	0.577	0.236	0.15	0.189	0.581	2.81E-30
PANX1	0.459	0.627	0.443	0.5	1.079	2.82E-30
RP11-612L3.	0.256	0.177	0.291	0.568	0.733	2.82E-30
SMAD6	1.094	-0.3	-0.888	-0.984	-2.077	2.94E-30
VAT1	0.22	0.016	0.034	0.524	1.095	3.20E-30
CAMTA2	-0.373	-0.044	0.104	-0.249	-0.601	3.45E-30
PSMB8	0.078	0.171	0.13	0.436	0.928	3.61E-30
ANKIB1	-0.158	0.096	0.182	0.192	0.455	3.94E-30
SPAG9	0.21	0.224	-0.067	-0.365	-0.36	3.94E-30
TMEM106C	-0.469	-0.082	0.111	-0.176	-1.107	4.01E-30

GRAP	0.492	-0.197	-0.082	0.196	0.716	4.08E-30
BBS2	-0.066	0.195	0.503	0.675	0.834	4.10E-30
TUBA1A	0.18	-0.151	-0.214	0.164	0.686	4.25E-30
MAP2K3	0.474	0.289	0.361	0.606	1.049	5.27E-30
GPX4	-0.113	-0.098	-0.064	-0.021	0.41	5.30E-30
CXCL6	-0.159	0.539	0.144	-0.236	0.882	6.34E-30
BSDC1	0.074	0.414	0.363	0.239	0.572	7.66E-30
LRRC1	0.306	0.111	-0.385	-0.487	-0.906	7.91E-30
PAQR8	-0.242	-0.139	-0.002	0.182	0.656	8.06E-30
ACER2	0.526	1.143	0.89	0.476	1.048	8.41E-30
HSPA12B	-0.33	-0.309	0.119	-0.044	0.791	1.01E-29
SRSF2	-0.001	-0.057	-0.263	-0.633	-0.529	1.01E-29
AP000695.6	0.295	0.075	0.699	0.873	1.46	1.03E-29
ZFYVE1	0.525	0.536	0.394	0.428	0.958	1.04E-29
SDK1	0.09	-0.1	0.049	0.064	-1.101	1.05E-29
FHL1	0.226	-0.004	-0.049	0.292	0.798	1.09E-29
RP5-1158H2	0.432	0.207	0.199	0.482	-0.716	1.12E-29
FSTL1	0.273	0.073	-0.06	0.281	0.662	1.15E-29
SDCCAG8	-0.142	0.017	0.224	0.303	0.885	1.32E-29
IL4R	0.389	0.24	0.391	0.371	0.641	1.41E-29
ANKH	0.691	0.1	-0.371	-0.27	-0.232	1.45E-29
SYNGR2	0.006	-0.091	-0.104	0.192	0.488	1.48E-29
RP11-492E3	0.176	-0.185	0.315	0.569	0.197	1.58E-29
ACVR1B	-0.096	0.101	0.028	-0.172	-0.664	1.69E-29
ARMC8	0.04	-0.042	-0.235	-0.179	-0.559	1.73E-29
SLC4A8	-0.382	-0.417	0.111	0.182	0.797	1.75E-29
ULK1	0.266	0.511	0.074	-0.266	0.414	1.81E-29
MEF2D	0.442	0.193	0.289	0.39	1.023	1.89E-29
AC092066.1	0.182	0.227	0.601	0.412	0.934	1.89E-29
SKA1	-0.001	-0.218	-0.249	0.039	-0.785	1.89E-29
OTUD4	0.035	0.441	0.327	0.151	0.442	2.05E-29
PML	-0.006	0.198	0.288	0.391	0.86	2.12E-29
FANCD2	-0.135	0.114	0.084	-0.014	-0.573	2.24E-29
MAFF	-0.234	0.491	0.46	0.233	0.669	2.25E-29
C16orf52	-0.061	-0.06	-0.137	-0.535	-0.962	2.39E-29
CCDC41	-0.447	0.254	0.24	-0.273	-0.879	2.53E-29
CLN6	0.077	-0.068	-0.258	-0.213	-0.984	2.66E-29
PDE7B	-0.799	-0.346	-0.117	0.059	0.247	2.77E-29

RNMT	0.143	0.279	0.262	0.242	0.708	3.09E-29
RAVER2	0.547	0.033	-0.263	-0.344	-0.426	3.30E-29
AC007250.3	0.119	0.032	0.122	0.344	0.693	3.34E-29
TRIM46	-1.143	-0.261	0.056	-0.51	-0.239	3.38E-29
EIF3L	0.234	0.169	0.228	0.429	0.684	3.68E-29
RFX5	-0.276	0.099	0.271	0.314	0.456	3.74E-29
CBLN3	-1.016	0.169	0.677	-0.219	0.94	3.91E-29
S100A16	0.084	-0.097	-0.088	0.209	0.471	3.97E-29
SLC4A7	-0.32	0.004	0.094	-0.077	0.529	4.04E-29
FADS3	-0.208	0.268	0.546	0.285	0.861	4.08E-29
PRKAG2	0.139	0.243	0.474	0.494	0.841	4.20E-29
TMEM201	-0.209	-0.285	-0.467	-0.577	-1.031	4.20E-29
BIRC5	-0.528	-0.629	-0.731	-0.558	-0.465	4.25E-29
ATF7IP	0.267	-0.085	-0.28	-0.038	0.541	4.27E-29
MVD	0.43	-0.169	-0.504	-0.637	-0.292	4.35E-29
SAMSN1	0.683	0.067	-0.346	-0.106	-0.463	4.35E-29
RFX2	1.24	0.035	0.165	0.46	-0.401	4.36E-29
NAB2	0.463	-0.014	-0.442	-0.619	-0.786	4.73E-29
ST13	0.291	0.151	0.106	0.49	0.711	4.90E-29
SH3PXD2B	0.019	-0.425	-0.166	0.039	0.677	5.18E-29
PYCARD	0	-0.24	-0.109	0.192	1.122	5.42E-29
HMGB2	-0.331	-0.69	-0.632	-0.279	-0.707	5.46E-29
NFXL1	-0.105	0.161	0.078	-0.302	-0.704	5.86E-29
MEX3A	0.034	-0.039	-0.123	0.082	0.948	5.90E-29
ANPEP	0.351	0.052	-0.012	0.408	1.049	6.31E-29
C16orf59	-0.268	-0.145	-0.284	-0.689	-1.097	6.31E-29
ATP6AP2	0.14	0.1	0.056	0.379	0.58	6.47E-29
EMG1	0.143	-0.001	0.044	0.019	-0.686	6.74E-29
MANF	0.228	-0.212	-0.188	-0.007	-0.496	6.77E-29
SNRPA1	-0.065	0.094	0.037	-0.086	-0.719	7.97E-29
TRIM56	-0.319	0.163	0.449	0.023	0.282	8.03E-29
ABHD3	-0.236	0.041	-0.021	-0.85	-1.361	8.39E-29
MMS19	-0.014	-0.003	0.125	0.112	0.533	8.50E-29
MEGF8	-0.113	0.072	0.12	0.117	0.534	9.16E-29
NDOR1	-0.418	-0.011	-0.132	-0.721	-0.805	9.77E-29
C19orf66	-0.307	0.282	0.505	0.265	0.743	1.00E-28
RPL13A	-0.067	0.008	0.128	0.134	0.657	1.04E-28
KANK3	0.032	0.28	0.628	0.315	1.169	1.06E-28

ANKRD26P1	-0.221	1.201	1.843	1.259	2.191	1.13E-28
CLK2	-0.455	0.07	0	-0.827	-0.949	1.13E-28
RBCK1	-0.2	-0.012	0.139	0.019	0.527	1.14E-28
PIM3	-0.669	-0.045	-0.521	-0.716	-0.328	1.16E-28
FLOT2	-0.101	-0.114	0.106	0.519	0.818	1.18E-28
SNCAIP	-0.496	-0.474	-0.06	-0.354	1.364	1.19E-28
XRCC3	-0.344	-0.056	-0.22	-0.628	-1.034	1.29E-28
FBXL2	-0.315	-0.503	-0.084	-0.13	0.597	1.35E-28
TTLL4	-0.485	-0.233	-0.159	-0.432	-0.653	1.44E-28
NECAP2	0.11	0.135	0.171	0.415	0.77	1.66E-28
STK38L	0.554	-0.089	-0.306	-0.175	0.344	1.70E-28
ATG16L2	-0.548	0.874	1.135	0.066	0.179	1.74E-28
NOX4	0.175	0.117	0.201	0.227	0.757	1.76E-28
SH3GLB1	0.186	-0.07	-0.091	0.129	0.449	2.01E-28
DACH1	-0.26	-0.148	-1.007	-1.05	0.805	2.02E-28
VPS13C	-0.295	0.133	0.378	0.186	0.599	2.02E-28
RANBP6	-0.141	-0.141	-0.007	0.321	0.353	2.09E-28
TCF7L1	-0.026	-0.128	0.234	0.387	0.811	2.36E-28
TRAF3	0.167	0.429	0.202	0.116	0.686	2.38E-28
GLYCTK	-0.407	0.113	-0.003	-0.335	-1.037	2.52E-28
HAPLN1	-0.023	-0.107	-0.429	-0.198	-1.23	2.52E-28
ZFP90	-0.267	0.421	0.47	-0.003	0.544	2.53E-28
RP4-794H19	0.268	0.394	0.778	0.538	2.215	2.69E-28
EZH2	-0.138	-0.136	-0.261	-0.737	-1.018	2.71E-28
GLIS2	0.915	0.204	0.141	0.096	0.69	2.78E-28
FAM167B	-0.273	-0.424	0.472	0.337	1.785	2.97E-28
ACSF2	-0.51	-0.137	0.035	0.235	1.153	2.97E-28
PAM	0.315	0.204	0.1	0.458	0.867	3.01E-28
POLE2	0.016	-0.076	-0.215	-0.403	-1.025	3.05E-28
IGFBP7	0.219	0.076	0.001	0.32	0.735	3.06E-28
TMEM171	0.093	-0.09	-0.029	0.071	-1.208	3.15E-28
PIK3C2B	-0.728	-0.577	-0.018	0.008	0.27	3.18E-28
CD99	0.106	-0.056	-0.053	0.22	0.514	3.26E-28
CDS2	0.103	0.107	0.186	0.319	0.58	3.39E-28
DPY19L3	-0.002	0.054	-0.037	-0.287	-0.711	3.56E-28
CTPS1	0.198	0.215	0.067	0.038	-0.339	3.75E-28
GALNT15	-0.241	0.044	1.002	1.283	1.395	3.76E-28
ANKRD12	0.639	0.267	0.017	-0.134	-0.047	3.90E-28

WNT9A	-0.125	0.45	0.945	0.235	1.874	3.96E-28
PHF1	-0.058	0.403	0.264	-0.325	-0.575	4.20E-28
TRPM3	-0.785	-0.124	-0.496	-1.301	-1.536	4.33E-28
FOXO1	0.586	0.567	0.406	0.49	0.888	4.40E-28
ZNF71	0.175	0.33	0.051	0.288	0.965	4.47E-28
MARVELD1	0.066	0.075	0.085	0.258	0.617	4.55E-28
VPS37B	0.558	0.264	0.336	0.553	0.893	4.60E-28
CHD7	0.379	-0.046	-0.219	-0.578	0.718	4.67E-28
LAMB3	-0.039	0.174	0.222	0.601	1.339	4.72E-28
CNTROB	-0.433	-0.147	-0.029	-0.306	-0.734	6.33E-28
MB21D1	0.511	0.163	-0.315	-0.495	-0.455	6.47E-28
PABPC1	0.118	0.192	0.174	0.25	0.524	6.57E-28
LYPD1	0.107	-0.335	-0.249	0.111	0.701	6.61E-28
AKAP1	-0.162	0.028	-0.027	-0.167	-0.567	6.82E-28
AJUBA	0.012	0.181	0.547	0.603	0.517	7.01E-28
RFC2	0.286	0.034	-0.355	-0.179	-0.7	7.37E-28
NAP1L1	0.283	0.283	0.238	0.42	0.569	7.52E-28
SLC25A29	-0.816	-0.315	-0.18	-1.007	-0.816	8.10E-28
CALCOCO1	-0.203	0.324	0.457	0.173	0.527	9.21E-28
HMGA1	0.129	0.007	0.081	0.47	0.947	9.79E-28
OSGIN1	0.146	0.337	-0.261	-0.281	-1.217	1.00E-27
CREB3L2	0.006	-0.104	0.061	0.201	0.759	1.01E-27
SLC10A7	0.013	0.036	-0.121	-0.231	-0.834	1.11E-27
PIK3R3	0.795	-0.05	-0.054	0.308	0.147	1.22E-27
ADAM15	-0.047	-0.053	-0.012	0.225	0.656	1.23E-27
TMEM173	0.237	0.369	0.548	0.608	0.411	1.25E-27
RFLNB	0.163	0.139	-0.121	-0.407	-0.58	1.26E-27
KRT17	0.138	0.682	1.19	1.62	2.149	1.28E-27
RP11-541M1	-0.502	-0.172	0.547	0.634	1.54	1.29E-27
PBK	-0.401	-0.532	-0.512	-0.236	-0.891	1.31E-27
VEGFB	-0.02	-0.037	0.165	0.19	0.677	1.34E-27
FADS2	0.32	-0.144	-0.477	-0.432	0.47	1.37E-27
MLLT3	-0.137	-0.415	-0.104	0.306	0.942	1.45E-27
SKIL	0.737	0.197	0.457	0.421	0.337	1.45E-27
NETO2	0.097	-0.059	-0.204	-0.182	0.458	1.60E-27
HAUS3	-0.16	0.016	-0.162	-0.376	-0.936	1.63E-27
MYLK2	-0.044	-0.404	0.19	0.522	1.666	1.67E-27
FAM20C	0.406	-0.216	-0.231	0.076	0.581	1.67E-27

BMP1	-0.032	0.157	0.181	0.044	0.53	1.67E-27
BRIX1	0.157	-0.019	-0.289	-0.162	-0.558	1.71E-27
DOLPP1	-0.091	-0.016	-0.252	-0.224	-0.876	1.83E-27
UBE2T	-0.454	-0.529	-0.534	-0.942	-1.523	1.91E-27
ARHGEF39	-1.641	-1.278	-0.793	-0.828	-1.656	1.92E-27
FGF5	-0.048	0.47	0.41	0.658	0.636	1.97E-27
CLEC14A	0.124	-0.277	-0.589	-0.172	-0.754	2.03E-27
GIMAP8	-0.477	0.157	-0.083	0.01	-0.945	2.04E-27
FANCE	-0.069	-0.247	-0.371	-0.569	-1.044	2.09E-27
FAM53C	0.472	0.159	0.008	0.104	0.488	2.11E-27
DDX60	-0.097	0.009	0.407	0.641	1.054	2.15E-27
LURAP1L	-0.905	-0.049	0.119	0.358	1.22	2.20E-27
FCHO2	0.538	0.711	0.647	0.472	0.657	2.35E-27
CYLD	0.231	0.638	0.385	0.312	0.682	2.50E-27
RASSF4	-0.337	0.192	0.641	0.636	1.271	2.55E-27
MIS12	-0.206	-0.071	-0.116	-0.6	-0.931	2.61E-27
ZNF467	-0.55	-0.833	-0.506	-0.71	-1.783	2.69E-27
RBMS2	0.036	0.047	0.3	0.443	0.364	2.72E-27
COPS8	0.282	0.121	-0.097	0.02	-0.485	2.74E-27
PPP1R3B	-0.003	0.089	0.214	0.439	0.87	2.87E-27
EPHA5	-0.11	-0.142	-0.61	-0.831	-1.448	2.91E-27
RSRC2	0.235	0.049	-0.125	-0.446	-0.388	2.97E-27
C15orf52	-0.728	-0.046	0.719	0.24	0.401	3.20E-27
FAM50B	-0.451	-0.764	-0.116	0.513	0.356	3.24E-27
EEF1E1	-0.286	-0.207	-0.338	-0.915	-1.538	3.28E-27
PELI1	0.1	0.025	0.174	0.28	0.762	3.32E-27
HNRNPDL	0.047	0.034	-0.152	-0.737	-0.757	3.46E-27
ANKRD33B	0.768	0.583	0.178	0.097	0.178	3.61E-27
AL162151.1	0.118	0.089	0.217	0.346	0.511	3.70E-27
NLRP1	0.066	0.58	0.713	0.264	0.313	3.70E-27
LTA4H	0.17	0.226	0.316	0.768	0.909	3.76E-27
ENTPD5	0.003	0.158	0.279	0.14	-0.403	3.92E-27
CDIP1	0.458	0.81	0.896	1.123	1.118	4.14E-27
HELZ2	0.048	0.617	0.524	0.125	0.675	4.15E-27
BANP	-1.222	-0.23	-0.154	-0.333	-0.376	4.24E-27
AC005363.9	-0.346	-0.095	-0.147	-1.146	-1.473	4.48E-27
MEX3C	0.391	0.095	0.006	0.444	0.841	4.51E-27
GCH1	0.896	0.433	-0.13	-0.283	-0.335	4.54E-27

SLC17A9	-0.357	-0.315	-0.298	-0.995	-1.034	5.07E-27
RP11-255M2	0.044	-0.102	0.114	0.401	0.807	5.17E-27
SERPINB8	0.685	0.115	-0.125	0.103	0.137	5.33E-27
NEK9	0.13	0.102	0.26	0.399	0.562	5.35E-27
FOXP1	-0.017	-0.137	-0.075	0.295	0.633	5.87E-27
EEF1A1P5	0.147	0.144	0.219	0.308	0.585	6.47E-27
KB-176G8.1	0.267	0.306	0.961	0.704	1.863	7.60E-27
EFL1	0.305	0.169	0.437	1.012	1.085	7.73E-27
PLPP4	0.402	-0.043	-0.388	-0.603	-0.441	7.74E-27
SLCO4A1	-0.133	-0.32	-0.147	-0.371	-1.34	8.55E-27
PTS	-0.127	-0.038	-0.061	-0.532	-1.092	8.79E-27
PAN2	-0.713	-0.145	0.318	-0.334	-0.711	8.86E-27
E2F4	0.194	-0.21	-0.217	-0.217	-0.534	9.01E-27
ABCC3	-0.056	-0.073	0.346	0.19	0.827	9.03E-27
RYBP	0.728	0.094	-0.131	0.064	0.329	1.01E-26
SLC25A19	-0.341	-0.297	-0.267	-0.446	-1.105	1.15E-26
TRMT2A	-0.351	0.101	-0.003	-0.326	-0.692	1.17E-26
RP1-222E13	-0.283	-0.177	-0.264	-0.494	-0.928	1.18E-26
PITPNA	0.334	0.004	-0.106	0.196	0.673	1.21E-26
MT-TP	-0.372	0.02	0.363	-1.328	-1.284	1.22E-26
H2AFX	-0.526	-0.741	-0.723	-0.366	-0.877	1.23E-26
YES1	0.109	-0.164	-0.134	0.157	0.376	1.26E-26
UBE2S	-0.442	-0.657	-0.909	-0.886	-0.253	1.30E-26
TANK	-0.228	0.166	0.318	0.239	0.799	1.34E-26
ELF4	0.74	-0.002	0.014	0.238	0.497	1.37E-26
GCKR	-0.444	0.474	0.699	0.109	-0.244	1.39E-26
POLQ	-0.622	-0.219	-0.261	-0.638	-0.935	1.51E-26
ATP13A3	0.489	0.013	-0.401	-0.33	-0.333	1.53E-26
CSF3	-1.789	-0.501	0.25	-0.337	0.392	1.54E-26
RP11-382A2	0.366	0.065	-0.21	-0.268	0.005	1.56E-26
PREB	-0.133	-0.035	-0.026	-0.046	-0.559	1.64E-26
CCDC71L	-0.66	-0.101	-0.39	-0.358	-0.684	1.70E-26
TPCN2	-0.293	0.279	0.003	-0.445	-0.711	1.71E-26
GDPD5	-0.397	-0.264	0.11	-0.189	0.648	2.31E-26
RDX	0.287	0.01	-0.346	-0.301	-0.267	2.34E-26
CRTAP	0.021	0.004	0.035	0.117	0.549	2.36E-26
LZTS3	-0.263	0.557	0.785	0.535	1.064	2.37E-26
TRIB3	-0.296	-0.026	0.328	0.67	0.834	2.43E-26

RRAD	0.893	1.526	1.087	0.278	2.465	2.56E-26
MCM4	0.538	0.034	-0.495	-0.496	-0.635	2.57E-26
NRCAM	0.273	0.074	-0.265	-0.043	-1.036	2.63E-26
SDF2L1	-0.041	-0.304	-0.4	-0.368	-0.989	2.68E-26
RP11-475N2	0.174	-0.28	-0.202	-0.41	-0.81	2.77E-26
RP11-356J5.	-0.015	-0.288	-0.359	-0.507	-1.278	2.95E-26
RSAD1	-0.498	-0.193	-0.049	-0.236	0.183	2.98E-26
PLXNA1	-0.128	-0.202	-0.214	-0.102	0.427	3.08E-26
CTSF	-0.063	-0.001	-0.077	0.067	0.745	3.14E-26
IL11	1.145	0.329	-0.282	-0.618	-0.61	3.16E-26
FBLN5	0.389	0.053	-0.248	0.067	-1.244	3.16E-26
NABP1	-0.734	0.409	0.403	-0.574	-0.652	3.23E-26
MEF2C	0.719	-0.131	-0.135	0.178	-0.092	3.24E-26
TIFA	0.173	0.366	-0.242	-0.447	0.645	3.31E-26
ATG3	-0.097	-0.136	-0.207	-0.241	-0.68	3.43E-26
PLCXD2	-0.577	0.46	0.762	0.359	1.074	3.52E-26
IFNGR2	-0.015	0.106	0.05	0.087	0.667	3.56E-26
EPT1	-0.013	0.031	-0.376	-0.631	-0.577	3.68E-26
EIF5	0.398	0.12	0.033	0.105	-0.062	3.76E-26
PCNX2	0.025	0.269	0.479	0.251	0.92	3.84E-26
ZFP36L1	-0.386	0.272	0.159	0.134	0.379	3.96E-26
SMIM13	0.318	-0.082	-0.192	-0.263	-0.323	3.96E-26
GNPDA1	-0.117	-0.026	0.194	0.477	0.499	4.19E-26
ZNF641	-0.002	0.055	-0.145	-0.474	-1.016	4.27E-26
CENPN	0.086	-0.005	-0.071	0.022	-0.494	4.33E-26
KIFC1	-0.573	-0.574	-0.404	-0.169	-0.901	4.33E-26
GBX2	-0.658	-0.588	-0.528	-0.241	-1.071	4.48E-26
VIMP	0.207	-0.087	-0.18	-0.105	-0.416	4.81E-26
CTSO	0.271	0.342	0.219	0.537	1.147	4.85E-26
EIF4EBP2	0.372	0.216	0.119	0.166	0.468	4.91E-26
NEIL3	-0.687	-0.615	-0.403	-0.273	-1.181	5.10E-26
KDM7A	1.15	0.19	0.109	-0.299	0.648	5.18E-26
GCC2	0.332	0.519	0.474	0.474	0.754	5.25E-26
NRP2	0.092	-0.027	-0.086	0.206	0.774	5.30E-26
SLC45A3	-0.011	-0.621	-0.067	0.323	0.395	5.31E-26
RNF130	0.07	0.01	0.019	0.211	0.548	5.37E-26
MIR126	-1.12	-0.38	-0.499	-1.52	-1.718	5.51E-26
EIF5A2	0.158	-0.163	-0.53	-0.791	-0.592	5.52E-26

KDM3A	0.799	0.198	-0.1	-0.131	0.264	5.62E-26
ZNF365	0.714	-0.391	-0.132	0.241	0.65	5.74E-26
ESYT2	0.313	0.094	0.008	0.156	0.587	5.87E-26
FAXDC2	-0.137	0.136	0.526	0.903	2.254	5.91E-26
CASC5	-0.542	-0.492	-0.411	-0.147	-0.728	6.03E-26
PRKCE	0.645	-0.121	-0.038	0.431	0.564	6.12E-26
AC018766.5	-0.591	-0.16	0.018	-0.996	-1.358	6.22E-26
SH3PXD2A	-0.4	-0.417	0.424	0.183	0.842	6.41E-26
EXOSC8	-0.199	-0.132	-0.218	-0.353	-0.908	6.42E-26
GIMAP6	-0.308	-0.01	0.138	0.358	0.439	7.47E-26
PGLS	0.083	0.072	0.086	0.369	0.813	7.76E-26
SHANK1	-0.36	-0.064	0.234	0.037	0.855	7.87E-26
LNX2	0.637	-0.116	-0.136	0.064	0.007	8.19E-26
HOXB4	-0.992	-0.264	-0.176	-0.244	-0.091	8.33E-26
CHML	0.202	0.138	-0.084	0.056	-0.546	9.05E-26
CUTA	0.01	-0.158	-0.09	0.175	0.605	9.25E-26
SPNS2	0.111	-0.335	-0.327	-0.343	0.201	9.84E-26
EXTL2	0.049	0.036	-0.208	-0.19	-0.561	1.15E-25
NCOA7	0.119	0.073	-0.462	-0.479	-0.388	1.27E-25
RFWD2	-0.008	0.055	0.078	0.358	0.475	1.30E-25
RNF122	0.44	0.493	0.208	0.481	1.307	1.32E-25
ARL6IP6	0.02	-0.18	-0.397	-0.529	-0.496	1.33E-25
PFDN1	0.089	-0.073	-0.084	0.063	0.409	1.34E-25
RGL1	0.349	0.493	0.521	0.458	1.199	1.35E-25
FANCB	-0.122	-0.093	-0.481	-0.77	-1.134	1.42E-25
HOXA9	0.537	-0.132	0.035	0.051	-0.25	1.44E-25
PANK3	0.452	0.158	-0.026	-0.106	0.184	1.50E-25
TSPYL2	-0.155	0.019	0.002	-0.632	-0.747	1.56E-25
ARAP1	-0.178	0.264	0.472	0.338	0.424	1.59E-25
STOML1	-0.242	-0.179	-0.15	-0.057	-0.967	1.59E-25
EPG5	-0.058	0.177	0.32	0.41	0.613	1.67E-25
ZNF385A	0.36	0.612	0.81	0.941	1.253	1.77E-25
PTP4A1	0.659	0.472	0.286	0.307	0.285	1.80E-25
PRDX2	0.211	0.078	0.035	0.439	0.752	1.82E-25
ADRB2	0.056	0.603	0.74	0.669	0.233	1.84E-25
LUZP1	0.598	0.146	0.199	0.442	0.827	1.95E-25
MLXIP	0.286	0.132	0.214	0.174	0.688	1.95E-25
NHSL2	-0.361	-0.427	0.128	0.261	0.317	2.10E-25

ZSCAN30	-0.539	-0.021	0.556	0.054	0.301	2.29E-25
FAM111A	-0.642	0.036	0.19	-0.322	-0.796	2.50E-25
PTGIR	-0.541	0.431	0.656	0.523	1.817	2.59E-25
TMCC1	0.009	0.041	0.299	0.471	0.895	2.82E-25
YPEL5	0.054	0.204	0.212	0.076	0.795	2.83E-25
SLC2A8	0.047	-0.188	-0.383	-0.482	-0.772	2.87E-25
GNAI2	0.214	0.039	0.042	0.44	0.886	3.10E-25
RP11-244O1	0.063	-0.545	0.314	0.982	1.175	3.11E-25
PTGR1	0.156	0.257	0.189	0.563	0.629	3.18E-25
ADAMTS9	-0.612	-0.227	0.215	0.286	-0.131	3.22E-25
SLC8B1	-0.105	0.039	0.156	0.136	0.589	3.25E-25
NHSL1	-0.056	-0.232	-0.254	-0.426	0.367	3.25E-25
CEP170	-0.076	0.033	0.074	0.252	0.606	3.31E-25
APBA2	0.265	-0.06	0.038	0.234	0.806	3.43E-25
TGFBR3	-0.024	-0.12	-0.261	-0.265	-0.864	4.09E-25
SPECC1	0.115	0.045	-0.416	-0.48	0.278	4.19E-25
PLXNA4	0.059	-0.201	-0.551	-0.071	-1.044	4.28E-25
PPP1R14B	-0.105	-0.149	-0.141	-0.187	0.449	4.31E-25
SLC2A6	-0.015	0.361	0.146	0.041	0.609	4.45E-25
AFG3L1P	-0.258	0.064	0.065	-0.875	-1.008	4.79E-25
CMIP	0.406	-0.159	0.087	0.517	0.775	5.06E-25
ZNF654	0.821	0.518	0.358	0.004	0.347	5.07E-25
ZNF76	-0.338	0.101	0.248	-0.04	-0.427	5.31E-25
PI4KAP1	-0.255	0.482	0.229	-0.75	-0.686	5.36E-25
ZNF561	0.087	0.645	0.754	0.331	0.82	5.47E-25
ZNF436	0.98	0.601	0.324	0.27	0.66	5.48E-25
TRAC	0.368	0.547	1.039	1.497	2.403	5.50E-25
CEBPG	0.502	-0.094	-0.147	0.141	-0.065	5.56E-25
EXOC4	0.49	0.016	-0.018	0.511	1.027	5.68E-25
PPM1M	-0.436	-0.143	0.35	0.197	0.453	6.09E-25
PSD3	0.309	-0.015	-0.115	0.209	0.866	6.30E-25
KIF14	-0.347	-0.883	-0.643	-0.322	-0.382	6.59E-25
SLC35D1	0.297	0.171	-0.066	-0.419	-0.553	7.25E-25
ARPC1B	0.203	-0.081	-0.107	0.133	0.439	7.28E-25
SNHG15	-0.37	-0.162	-0.191	-0.944	-1.189	7.54E-25
TBC1D2	0.626	0.592	0.875	1.188	0.72	7.90E-25
REST	1.058	0.249	0.364	0.661	0.69	7.91E-25
TOPBP1	0.151	-0.045	-0.223	-0.204	-0.376	8.02E-25

ULK3	-0.26	0.031	-0.067	-0.614	-0.726	8.02E-25
LIMK2	0.154	0.124	0.266	0.024	0.475	8.13E-25
ZNF827	0.229	-0.055	0.358	0.385	0.653	8.53E-25
MCM3	0.445	-0.035	-0.519	-0.464	-0.506	8.53E-25
TGFBR1	0.838	0.422	0.084	0.017	0.349	8.54E-25
GDE1	-0.056	-0.062	-0.026	0.156	0.486	8.57E-25
EXOC3L2	1.195	-0.159	-0.253	0.059	-1.092	8.58E-25
ABR	0.239	-0.07	0.072	0.479	0.795	1.04E-24
SIRT1	0.708	0.084	-0.089	0.055	0.058	1.07E-24
ING4	-0.258	-0.011	0.178	0.102	0.748	1.08E-24
IPPK	0.56	0.206	0.119	0.008	-0.37	1.08E-24
ERRFI1	0.307	0.139	0.177	0.051	0.675	1.09E-24
MYO19	0.005	-0.048	-0.074	-0.444	-0.438	1.11E-24
CREB5	0.057	0.277	0.297	0.52	1.584	1.15E-24
ATHL1	-0.406	0.083	0.055	-0.937	-0.9	1.16E-24
RP3-399L15.	-0.586	-0.182	0.298	0.467	1.635	1.20E-24
RSPH3	0.246	0.463	0.485	0.803	1.382	1.29E-24
MFHAS1	0.868	0.084	0.148	0.225	0.65	1.38E-24
KLF3	0.742	0.289	0.264	0.288	0.647	1.40E-24
CERCAM	0.035	-0.031	0.077	0.263	0.665	1.42E-24
C1orf74	0.597	0.135	-0.223	-0.195	-0.74	1.55E-24
EFHD2	-0.186	-0.205	0.044	0.367	0.503	1.56E-24
FAM129A	0.26	0.57	0.669	1.187	1.469	1.60E-24
GLRB	0.088	0.193	0.287	0.259	1.06	1.60E-24
LMO4	0.153	-0.429	-0.482	-0.259	-0.469	1.63E-24
NUMB	0.092	0.039	0.042	0.221	0.586	1.64E-24
RP11-142E9	0.468	0.129	-0.448	-0.751	-1.237	1.67E-24
SDC2	0.275	-0.05	-0.423	-0.335	-0.891	1.76E-24
HMOX1	0.681	0.397	0.486	0.897	1.125	1.78E-24
RAB31	0.476	-0.112	-0.386	-0.136	0.371	1.87E-24
HSF2	0.42	0.077	-0.315	-0.259	-0.384	1.93E-24
MESDC1	0.118	-0.146	-0.14	0.055	0.584	2.03E-24
APOBEC3B	0.041	0.346	0.545	1.108	1.076	2.08E-24
MTURN	0.466	0.346	0.131	-0.469	-0.864	2.14E-24
IQCE	0.288	-0.149	0.017	0.157	0.695	2.17E-24
RACGAP1	-0.562	-0.862	-0.751	-0.19	-0.543	2.30E-24
TNFAIP3	0.097	0.656	0.204	-0.293	0.533	2.32E-24
ZBTB8A	1.181	0.172	0.122	0.19	0.376	2.34E-24

C14orf80	-0.018	-0.05	-0.066	-0.187	-0.915	2.48E-24
MRPS27	0.097	-0.063	0.087	0.278	0.391	2.51E-24
PCDHGB7	0.2	0.28	0.024	0.534	1.588	2.78E-24
KIAA0556	-0.318	-0.032	0.23	0.079	0.459	2.78E-24
PLCL2	0.525	0.696	0.534	0.673	1.406	2.84E-24
PLCXD1	-0.263	-0.128	-0.233	-1.002	-0.733	2.88E-24
ZFP36L2	-0.183	0.393	0.014	-0.018	0.7	2.91E-24
SFXN3	-0.222	-0.193	0.044	0.031	0.359	2.93E-24
FNIP2	0.645	0.057	-0.035	0.08	-0.069	3.01E-24
RP11-22B23	-0.6	-0.114	-0.109	-1.094	-1.576	3.02E-24
RAD18	0.147	0.101	-0.2	-0.217	-0.546	3.12E-24
MAMDC4	-0.266	0.773	1.037	-0.068	-0.283	3.18E-24
NBR1	0.275	0.16	0.308	0.717	0.828	3.36E-24
TROAP	-1.432	-1.086	-0.705	-1.037	-1.062	3.44E-24
SLC38A2	0.201	-0.037	-0.263	-0.435	-0.795	3.50E-24
NHLRC2	0.162	0.187	0.165	0.001	-0.393	3.53E-24
TMEM35	0.3	-0.021	0.086	0.231	1.109	3.55E-24
DLGAP4	0.203	0.176	0.345	0.562	0.959	3.55E-24
DNAJB4	-0.258	-0.025	-0.469	-0.31	-1.078	3.75E-24
MMP11	-0.026	-0.142	-0.091	-0.092	1.352	3.78E-24
AGTRAP	-0.142	-0.105	0.154	0.38	0.494	3.80E-24
BNC2	-0.035	0.028	0.118	0.315	0.6	3.89E-24
DSTN	0.215	0.03	-0.08	-0.119	-0.468	3.97E-24
VKORC1L1	0.025	-0.192	-0.101	0.05	0.484	3.98E-24
CDT1	0.617	0.004	-0.305	-0.336	-0.293	4.08E-24
ADGRG1	0.314	0.063	0.086	0.449	0.788	4.44E-24
SBDSP	-0.021	-0.087	-0.164	-0.377	-0.761	4.67E-24
B4GAT1	-0.079	0.088	0.093	0.592	0.734	4.76E-24
SYNE3	0.489	0.092	0.642	0.31	-0.158	5.00E-24
GPX1	-0.053	-0.031	0.09	0.2	0.417	5.12E-24
DIAPH2	0.521	0.065	0.292	0.701	0.76	5.21E-24
AMIGO2	0.762	0.633	0.335	0.067	1.026	5.30E-24
ANKRD18A	-0.079	-0.08	-0.012	-0.499	-1.258	5.48E-24
C21orf58	-1.153	-0.349	-0.187	-0.577	-0.891	5.61E-24
FAM107B	0.066	0.052	0.052	0.201	-0.58	5.66E-24
DBF4	-0.809	-0.754	-0.654	-0.706	-1.007	5.67E-24
RABL2B	-0.28	0.184	0.55	0.291	0.671	5.75E-24
FOXF1	0.599	0.296	0.475	0.859	1.731	5.87E-24

KLF4	1.329	0.253	0.052	0.057	0.149	5.88E-24
KCNJ2	1.253	0.974	1.622	1.421	1.33	6.59E-24
ST3GAL1	0.592	-0.068	0.047	0.091	-0.183	6.67E-24
RHOJ	-0.234	-0.187	0.01	-0.161	0.472	7.00E-24
ZNF704	0.537	-0.07	-0.141	-0.212	-0.468	7.29E-24
C3orf58	0.266	0.02	-0.346	-0.3	-0.352	7.46E-24
GMPPB	0.096	-0.002	0.017	0	-0.608	7.96E-24
MANSC1	-0.043	-0.023	0.069	0.464	0.543	8.01E-24
PM20D2	0.058	0.308	0.353	0.46	0.799	8.29E-24
TFDP2	0.182	0.011	-0.198	0.043	0.655	8.41E-24
ZBTB38	0.33	0.259	0.41	0.743	0.834	8.61E-24
RP4-794I6.4	0.735	1.217	1.446	1.092	1.279	8.67E-24
MAP3K3	0.095	-0.101	0.046	0.21	0.546	8.83E-24
C1RL	-0.094	0.079	0.273	0.58	0.744	8.97E-24
CRTAC1	0.187	0.114	0.493	0.649	1.941	9.62E-24
TMEM248	0.251	0.059	-0.036	0.261	0.544	9.65E-24
LRRC4	0.88	-0.734	-1.215	-1.267	-1.871	9.66E-24
ASPH	0.141	-0.056	-0.13	0.13	0.469	9.87E-24
HLX	0.211	0.548	-0.217	0.054	1.107	1.02E-23
STIL	-0.09	-0.393	-0.449	-0.292	-0.71	1.04E-23
CH17-472G2	-1.201	-0.063	0.108	-0.774	0.299	1.09E-23
FAM213A	-0.053	-0.092	0.099	0.314	0.648	1.13E-23
COG5	0.266	0.064	0.138	0.397	0.619	1.25E-23
PLVAP	0.296	-0.07	-0.275	0.245	-1.466	1.26E-23
PRDM15	-0.77	0.311	0.241	-0.239	-0.347	1.35E-23
RNF19A	0.549	0.169	0.199	0.173	0.554	1.36E-23
MSRA	0.101	0.116	0.108	0.489	0.959	1.38E-23
CARD11	0.217	-0.074	-0.078	0.261	0.76	1.41E-23
XRCC2	0.249	-0.028	-0.37	-0.787	-0.903	1.45E-23
SP100	0.152	0.021	0.064	0.264	0.497	1.48E-23
TMEM164	0.482	0.114	-0.228	-0.259	-0.553	1.57E-23
TBC1D8	0.192	0.089	0.17	-0.275	-0.545	1.61E-23
HLA-A	0.24	0.136	0.034	0.398	0.799	1.65E-23
CPNE3	0.27	0.114	0.149	0.585	0.83	1.70E-23
TLR4	0.157	-0.181	-0.468	-0.378	-0.612	1.72E-23
SULF1	-0.15	0.033	-0.016	0.324	-1.044	1.76E-23
RINL	-0.078	0.159	0.741	1.032	0.969	1.78E-23
ZNF692	-1.151	-0.08	0.164	-0.886	-0.686	1.83E-23

ZBTB48	-0.799	-0.01	0.002	-0.569	-0.687	1.97E-23
TAB2	0.378	0.408	0.285	0.466	0.739	1.98E-23
ATG4D	0.082	0.236	-0.123	-0.308	-0.556	2.03E-23
NFKBIE	0.458	0.669	0.246	0.033	0.738	2.05E-23
FLOT1	0.105	-0.185	-0.282	-0.086	-0.617	2.05E-23
HIVEP1	0.564	0.825	0.501	0.224	0.283	2.10E-23
HDAC9	-0.055	0.766	0.563	0.599	0.986	2.11E-23
VWA5A	-0.256	-0.241	0.024	0.26	0.948	2.11E-23
CALHM2	0.341	-0.386	-0.279	-0.03	-0.514	2.14E-23
MPZL1	0.11	-0.057	-0.23	-0.024	0.409	2.17E-23
GRINA	0.067	0.029	-0.011	0.261	0.763	2.29E-23
PPARA	-0.36	0.142	0.311	0.31	0.844	2.44E-23
PRPF40B	-0.584	-0.169	-0.165	-0.518	-1.36	2.47E-23
FHL3	0.098	0.038	0.171	0.268	0.725	2.49E-23
SPATA7	-0.476	0.183	0.009	-0.224	-0.713	2.50E-23
HPCAL1	0.226	-0.166	-0.208	0.248	0.436	2.58E-23
SDCBP2	0.136	-0.047	0.167	0.403	1.239	2.67E-23
GM2A	0.218	0.099	0.129	0.554	0.69	2.73E-23
ELMO3	-0.041	-0.156	-0.098	-1.035	-1.227	2.77E-23
SLITRK4	-0.183	0.006	0.029	0.066	0.749	2.78E-23
HEY1	1.199	-0.237	-0.721	-0.516	-0.154	2.78E-23
PRKAB1	-0.011	0.218	0.477	0.387	0.828	2.81E-23
VAPA	0.162	-0.184	-0.284	-0.197	-0.327	3.03E-23
ADAMTS18	-0.388	-0.065	0.617	0.693	2.15	3.17E-23
RBM38	0.335	0.36	0.354	0.428	0.928	3.28E-23
MMP16	0.051	-0.239	-0.466	-0.527	-0.617	3.28E-23
DNASE1L1	0.046	-0.091	0.006	0.256	0.538	3.36E-23
TIMM10	-0.437	-0.506	-0.459	-0.626	-1.018	3.44E-23
EDEM1	0.262	0.103	0.093	0.07	0.527	3.56E-23
TAF1C	-0.332	-0.148	-0.224	-0.937	-0.977	3.57E-23
PWWP2A	0.093	0.116	0.109	0.327	0.728	3.96E-23
HMGN2P5	-0.05	-0.177	-0.06	-0.052	-0.799	4.01E-23
VOPP1	0.116	0.024	-0.022	0.239	0.54	4.06E-23
AES	0.056	-0.07	-0.048	0.091	0.414	4.27E-23
GCLM	0.324	0.135	-0.109	0.046	-0.675	4.29E-23
ABCB6	-0.359	0.263	0.276	0.205	-0.934	4.29E-23
EGR1	-0.777	-0.185	-1.158	-1.788	-1.733	4.54E-23
HNRPA1L-2	0.251	0.458	0.373	0.81	0.67	4.62E-23

PRDM1	0.43	0.761	0.415	0.06	1.151	4.89E-23
AHNAK2	-0.195	-0.216	0.52	0.31	0.673	4.99E-23
DCP1A	0.468	0.085	-0.059	-0.161	-0.186	5.01E-23
BCAT1	0.326	-0.256	-0.379	-0.093	0.268	5.06E-23
FAM193B	-0.69	0.048	-0.025	-0.904	-0.801	5.19E-23
DPYSL3	0.571	0.037	-0.382	-0.339	-0.649	5.30E-23
NUDT4	-0.08	-0.082	-0.513	-0.459	-0.064	5.77E-23
PLEKHJ1	-0.634	-0.342	-0.42	-0.747	-0.69	5.77E-23
CHST11	0.331	-0.166	-0.251	-0.037	0.639	5.87E-23
KRT7	0.655	0.023	-0.243	0.074	0.971	5.88E-23
TNFSF10	-0.457	-0.028	0.174	0.223	2.081	5.95E-23
NOL6	0.15	-0.011	-0.103	-0.175	-0.678	5.95E-23
FAM63A	-0.308	0.132	0.447	0.6	0.581	6.28E-23
SLFN11	0.103	-0.247	-0.182	-0.32	-0.49	6.67E-23
STK11IP	0.115	0.088	-0.003	-0.177	-0.753	6.68E-23
SNX5	0.035	0.072	-0.051	-0.137	-0.372	6.81E-23
TTC3	0.089	0.072	0.07	-0.027	0.385	7.28E-23
CXCL1	0.05	0.265	-0.182	-0.633	-0.194	7.34E-23
ZNRF1	0.234	0.301	0.16	0.267	0.78	7.48E-23
PTPN14	0.185	0.03	0.122	0.289	0.681	7.69E-23
CENPO	0.039	-0.297	-0.427	-0.331	-0.595	7.86E-23
SHB	0.683	-0.082	0.048	0.45	0.779	7.97E-23
CCDC112	0.056	0.045	0.179	0.422	0.976	8.16E-23
FNDC3B	0.392	0.051	0.177	0.422	0.824	8.38E-23
SEMA4C	-0.572	-0.055	-0.489	-0.584	-0.835	8.82E-23
ANKRD11	0.124	-0.026	0.153	0.167	0.427	9.04E-23
PMP22	0.273	-0.246	-0.411	-0.187	-0.308	9.25E-23
SLC30A7	0.122	0.347	0.061	-0.185	-0.299	9.34E-23
PARVB	0.282	-0.006	0.022	0.379	0.714	1.02E-22
RPS6KA2	0.248	-0.044	-0.154	0.13	0.523	1.05E-22
SREBF2	0.304	-0.072	-0.337	-0.38	0.162	1.08E-22
GMDS	0.515	0.202	0.094	0.575	0.86	1.15E-22
UBA6	0.104	0.226	0.272	0.375	0.591	1.16E-22
ANK3	0.327	-0.149	0.201	0.63	0.888	1.21E-22
PLK4	-0.164	-0.31	-0.24	-0.328	-0.873	1.25E-22
KLHL4	0.297	-0.14	-0.597	-0.565	0.098	1.26E-22
KIF2C	-0.606	-0.824	-0.687	-0.305	-0.517	1.27E-22
BEND3	0.923	0.056	-0.311	-0.192	0.644	1.39E-22

CRYL1	0.231	-0.175	-0.221	0.097	0.546	1.39E-22
ELMSAN1	-0.454	-0.169	0.139	0.076	0.185	1.39E-22
ELL2	0.458	0.415	0.159	0.193	0.535	1.42E-22
KIAA0922	-0.094	-0.103	0.008	0.157	0.842	1.43E-22
IQCC	-0.412	-0.131	-0.701	-0.657	-1.428	1.45E-22
PTTG1	-0.427	-0.578	-0.465	-0.255	0.147	1.50E-22
ZMYM2	0.124	0.018	-0.067	0.038	0.56	1.54E-22
SUV39H1	-0.119	-0.24	-0.398	-0.133	-0.825	1.59E-22
SKI	0.779	0.02	0.032	0.209	0.326	1.67E-22
ZNF512	-0.588	-0.128	0.077	-0.034	0.15	1.68E-22
BTBD10	0.042	0.177	0.167	0.437	0.49	1.75E-22
CEP95	-0.139	0.248	0.223	-0.438	-0.628	1.80E-22
DIXDC1	0.385	-0.359	-0.584	-0.272	-0.682	1.80E-22
ELP2	0.096	0.072	0.14	0.327	0.525	1.95E-22
MIPOL1	0.131	-0.148	-0.133	-0.338	-0.658	1.97E-22
SLC25A28	-0.004	0.046	-0.081	-0.503	-0.9	1.98E-22
HMOX2	0.302	-0.145	-0.1	0.361	0.658	2.05E-22
MZF1	-0.592	0.268	0.598	-0.096	-0.205	2.08E-22
TMEM38B	-0.144	-0.009	-0.121	-0.563	-1.235	2.08E-22
EBI3	-0.021	0.987	0.849	1.106	1.859	2.13E-22
GPD2	0.236	0.007	-0.218	-0.174	-0.571	2.22E-22
ZNF79	0.916	0.904	0.852	0.288	0.593	2.32E-22
UAP1	0.354	-0.129	-0.04	0.329	0.309	2.33E-22
ALS2CL	-0.578	0.049	0.79	0.267	-0.055	2.34E-22
LRRC58	0.482	0.093	-0.172	-0.021	-0.286	2.36E-22
RNF138	0.154	-0.123	-0.331	-0.474	-0.388	2.40E-22
RPS6KL1	-0.524	0.079	0.572	0.157	0.433	2.42E-22
PAPLN	-0.637	0.344	0.739	0.154	0.54	2.45E-22
RFWD3	0.192	-0.204	-0.35	-0.306	-0.508	2.45E-22
AC116366.4	-0.484	0.165	0.753	-0.039	1.366	2.53E-22
CCIN	0.231	0.071	-0.051	0.56	1.266	2.55E-22
RP11-295D4	-0.791	-0.166	-0.119	-1.341	-1.215	2.60E-22
PLCL1	-0.328	-0.002	-0.897	-0.49	-0.63	2.66E-22
SLC15A3	-0.048	0.262	0.319	0.746	1.613	2.80E-22
TINAGL1	0.317	0.074	-0.059	0.143	-0.402	2.85E-22
YRDC	0.205	-0.155	-0.342	-0.39	-0.595	2.91E-22
NASP	0.31	0.156	-0.118	-0.144	-0.504	2.92E-22
LRRC20	-0.051	-0.356	-0.056	0.423	1.003	2.99E-22

SOGA1	-0.421	-0.301	-0.193	-0.114	0.362	3.09E-22
ZNF121	-0.066	0.279	0.173	-0.697	-0.578	3.10E-22
RP11-504P2	-1.09	0.171	0.581	-0.38	-0.511	3.22E-22
CCP110	0.032	0.054	-0.055	-0.117	-0.611	3.48E-22
APMAP	0.175	0.073	0.02	0.354	0.549	3.56E-22
LYST	-0.789	0.064	0.201	-0.07	0.22	3.56E-22
WDR59	-0.176	0.217	0.365	0.14	0.371	3.67E-22
RAB11FIP5	-0.055	-0.026	0.179	0.37	0.615	3.72E-22
KSR1	-0.514	-0.079	0.336	0.155	0.294	3.90E-22
TDRD10	0.247	0.056	-0.101	-0.609	-1.201	3.95E-22
RP11-363E7	0.559	1.142	1.11	0.591	1.112	3.97E-22
AK1	0.18	0.128	0.269	0.569	0.859	4.14E-22
NCEH1	0.024	0.2	0.214	0.513	0.628	4.20E-22
UGT2A1	-0.147	-0.036	-0.392	-0.284	-1.689	4.50E-22
PLEKHA1	0.097	0.335	0.237	0.118	0.613	4.51E-22
HMGXB4	0.496	0.021	-0.081	-0.067	-0.119	4.62E-22
WASH3P	-0.47	0.153	0.244	-0.572	-0.734	4.83E-22
SHROOM3	0.664	0.15	0.655	0.891	0.976	4.94E-22
KMT2B	-0.324	-0.067	-0.125	-0.674	-0.597	5.00E-22
LAPTM4A	0.119	-0.089	-0.083	0.187	0.402	5.11E-22
ZKSCAN1	-0.118	0.283	0.238	0.124	0.483	5.34E-22
CDKN2D	-0.131	-0.501	-0.056	0.151	-1.294	5.42E-22
PAIP1	0.12	0.007	-0.175	0.093	0.597	5.52E-22
ST3GAL4	-0.171	-0.219	-0.024	-0.198	0.443	5.63E-22
PVR	0.335	0.255	0.239	0.328	0.572	5.69E-22
TNFRSF10D	0.516	0.476	0.235	0.338	-0.285	5.76E-22
RP11-334L9.	0.358	-0.274	-0.362	-0.125	-0.712	6.07E-22
PRPSAP1	-0.105	-0.098	-0.006	0.244	0.46	6.09E-22
RP11-245J9.	0.529	0.446	0.418	-1.049	-1.163	6.24E-22
GBP2	0.546	0.259	0.091	0.46	1.89	6.40E-22
FSTL3	0.62	0.269	0.345	0.394	0.45	6.46E-22
GREM1	0.331	0.127	-0.251	-0.248	0.729	6.53E-22
HACD2	0.068	0	-0.228	-0.217	-0.579	6.56E-22
DHRS3	0.279	-0.049	-0.07	0.182	1.121	6.70E-22
STAU2	0.026	0.104	0.228	0.517	0.726	7.31E-22
CXXC1	-0.194	-0.112	-0.155	-0.317	-0.651	8.08E-22
FSCN1	0.299	-0.065	-0.138	0.295	0.947	8.12E-22
KDR	0.353	-0.13	-0.06	0.181	0.544	8.26E-22

FBXO22	0.427	0.686	0.822	0.456	0.648	8.34E-22
NDFIP1	0.064	-0.036	-0.076	0.086	0.454	8.40E-22
HYLS1	-0.927	-0.851	-0.667	-0.322	-0.246	8.55E-22
PCTP	0.112	-0.21	-0.528	-0.497	-1.004	8.65E-22
DLC1	0.711	0.301	0.355	0.493	0.678	8.84E-22
FTX	-0.364	0.177	0.152	-0.597	-0.557	9.06E-22
RAD52	-0.9	0.006	0.162	-0.537	-0.59	9.19E-22
CDK19	-0.188	0.251	0.308	0.414	0.783	9.24E-22
SNX7	0.042	0.091	0.135	0.416	0.595	9.29E-22
HSDL2	0.343	0.335	0.439	0.689	0.766	9.34E-22
CLDN15	-0.507	-0.035	0.408	-0.566	-0.745	9.81E-22
RP11-181C3	-0.546	-0.526	-0.387	-0.499	-1.825	9.93E-22
EFHC1	-0.527	-0.087	0.485	-0.037	0.835	1.00E-21
ZFAND2B	-0.079	-0.09	-0.207	-0.382	-0.781	1.00E-21
C8orf33	0.17	0.031	-0.14	-0.206	-0.385	1.03E-21
TCF4	0.397	0.103	-0.019	0.015	0.535	1.08E-21
ASPHD2	-0.288	-0.138	0.019	0.266	0.748	1.12E-21
TBL1X	0.155	-0.189	-0.058	0.27	0.709	1.14E-21
CEP68	-0.122	-0.008	0.063	0.133	0.631	1.17E-21
ANKRD18B	0.212	0.012	-0.235	-0.313	-1.149	1.17E-21
EIF2S3	0.202	0.257	0.291	0.319	0.446	1.18E-21
POLL	-0.497	0.199	0.186	-0.212	-0.334	1.20E-21
IQCK	0.007	0.268	0.495	0.387	1.287	1.30E-21
PCMTD1	0.148	0.306	0.294	0.134	0.747	1.30E-21
CARD8	-0.6	-0.225	0.28	-0.075	0.093	1.33E-21
NUP35	-0.182	-0.414	-0.583	-0.324	-0.868	1.40E-21
RAB5B	0.122	0.039	0.143	0.254	0.613	1.41E-21
GLIPR2	0.279	0.13	0.022	0.077	0.705	1.43E-21
MIR22HG	-0.155	0.097	-0.061	-0.969	-0.886	1.43E-21
RBL1	0.429	0.244	0.032	0.082	-0.307	1.45E-21
MPG	-0.102	-0.151	-0.058	0.151	0.499	1.46E-21
HDAC6	-0.185	0.072	0.207	-0.041	-0.596	1.46E-21
CACHD1	0.158	-0.094	-0.647	-0.589	-0.74	1.46E-21
ALDH2	0.196	0.12	0.003	0.41	0.772	1.57E-21
AC002480.6	0.116	0.235	0.285	0.398	0.616	1.59E-21
EVC	-0.189	-0.147	0.125	-0.036	0.561	1.59E-21
PPM1A	0.558	0.34	0.156	0.238	0.383	1.59E-21
SNHG12	-0.081	0.113	0.097	-0.971	-1.125	1.59E-21

SKA3	-0.468	-0.413	-0.156	-0.045	-0.724	1.63E-21
ST6GALNAC	0.006	-0.116	-0.278	-0.322	-0.662	1.68E-21
MFSD2A	-0.369	-0.355	-0.825	-0.959	-1.121	1.72E-21
TRIM25	0.067	0.016	0.044	0.321	0.584	1.74E-21
JUND	0.272	0.199	0.034	0.208	0.964	1.77E-21
CKAP2	-0.454	-0.631	-0.476	-0.006	-0.02	1.77E-21
CPEB2	0.548	0.384	0.114	-0.098	0.072	1.79E-21
CUL9	-0.643	-0.087	0.156	-0.407	-0.477	1.79E-21
STAG3L5P-F	-0.802	0.001	0.159	-0.909	-0.928	1.86E-21
IFIT3	1.107	0.061	0.436	1.181	1.64	1.91E-21
KAT7	0.126	-0.079	-0.334	-0.224	-0.8	1.91E-21
UBXN2B	-0.338	0.02	0.233	0.379	0.771	1.95E-21
ECE1	0.27	0.083	0.039	0.477	0.903	1.98E-21
CREG1	0.159	0.229	0.128	0.394	0.824	2.06E-21
MPHOSPH9	-0.362	-0.231	-0.094	-0.211	-0.724	2.11E-21
HOXD1	0.183	0.438	-0.053	-0.094	-0.635	2.20E-21
POP5	-0.252	-0.128	-0.201	-0.235	-0.946	2.25E-21
TUBGCP6	-0.634	0.079	0.198	-0.648	-0.763	2.28E-21
RILPL1	-0.102	-0.272	0.107	0.178	0.545	2.32E-21
AP001053.1	-0.469	0.278	1.061	0.974	1.37	2.47E-21
TMEM79	-0.699	0.064	-0.106	-0.696	-0.832	2.53E-21
CSRNP2	0.369	0.18	-0.119	-0.122	0.242	2.55E-21
DENND4B	-0.418	-0.193	-0.047	-0.48	-0.618	2.63E-21
RAI2	0.502	0.022	-0.307	-0.325	-0.744	2.75E-21
CREM	-0.092	0.319	0.62	0.592	0.69	2.83E-21
MBNL1-AS1	-0.425	-0.515	-0.476	-0.848	-1.533	2.83E-21
MAGEH1	0.153	-0.101	-0.293	-0.095	-0.649	2.89E-21
FAM167A	0.49	0.272	0.868	1.615	0.866	2.91E-21
CDK14	0.445	-0.141	-0.264	-0.275	-0.082	2.93E-21
TNFAIP2	0.053	0.436	0.267	-0.212	0.44	2.96E-21
PSME1	0.123	0.09	0.18	0.236	0.59	3.02E-21
LHFPL2	0.529	0.015	-0.316	-0.297	0.325	3.05E-21
CYBA	-0.107	-0.145	-0.104	-0.043	0.456	3.07E-21
TJP1	0.473	0.12	0.249	0.472	0.719	3.23E-21
TACC3	-0.378	-0.644	-0.525	-0.318	-0.699	3.66E-21
SPOCK1	0.332	0.159	-0.02	0.379	0.801	3.76E-21
SLC22A23	0.364	0.661	1.375	1.269	0.883	3.85E-21
ILVBL	-0.116	-0.121	-0.295	-0.141	-0.721	3.88E-21

DOPEY2	0.074	0.317	0.474	0.822	1.071	4.12E-21
CLIC4	0.434	0.11	-0.121	0.073	0.515	4.24E-21
ATAD3B	-0.179	0.002	-0.231	-0.739	-0.666	4.24E-21
LIN54	0.41	0.053	-0.195	-0.052	-0.295	4.47E-21
CD47	-0.264	-0.037	0.17	0.201	0.642	4.48E-21
NR5A2	-0.879	-0.122	-0.765	-0.587	-0.643	4.51E-21
ARMC10	-0.125	-0.118	-0.001	-0.038	0.609	4.64E-21
RELT	0.084	-0.032	-0.365	-0.883	-0.491	4.88E-21
SNX19	0.005	0.024	0.109	0.309	0.406	5.07E-21
TMEM175	-0.306	0.013	0.023	-0.248	-0.803	5.07E-21
C1orf21	0.29	0.073	-0.188	0.149	0.917	5.15E-21
APLF	-0.173	0.825	1.102	0.951	1.11	5.33E-21
HNRNPC	0.443	0.146	-0.013	0.325	0.662	5.42E-21
PLEKHA2	0.714	0.001	-0.004	0.315	0.289	5.54E-21
ALDH1A1	0.125	0.056	-0.023	0.54	-0.718	5.56E-21
NAV3	0.08	0.06	-0.501	-0.355	0.037	5.57E-21
SLC4A11	-0.154	0.532	0.478	-0.225	0.414	5.71E-21
FBLIM1	0.294	-0.212	-0.046	0.294	0.385	5.87E-21
VLDLR	0.549	0.269	-0.184	0.268	1.224	5.93E-21
C9orf114	-0.384	-0.118	-0.184	-0.397	-0.678	5.98E-21
MALL	0.395	-0.206	-0.424	-0.171	-0.635	6.06E-21
CDR2	-0.044	-0.2	-0.214	0.087	0.463	6.28E-21
SLC35E2B	-0.431	-0.018	0.125	-0.077	0.257	6.28E-21
KISS1	-0.318	0.002	0.247	1.108	1.859	6.29E-21
EPS8	0.287	0.339	-0.004	0.023	-0.722	6.32E-21
IL27RA	0.196	0.063	0.032	0.472	0.769	6.41E-21
RHPN1	-1.097	-0.37	0.388	-0.055	0.773	6.46E-21
ID3	-0.221	-0.391	-0.401	-0.179	-1.209	6.46E-21
TJAP1	-0.223	0.301	0.102	-0.385	-0.421	6.48E-21
ZBTB18	-0.622	-0.151	-0.087	-0.245	-0.422	7.00E-21
C2CD2L	-0.26	-0.032	-0.126	-0.449	-0.556	7.15E-21
KLHL21	0.527	0.294	0.271	0.285	0.6	7.46E-21
HECTD2	-0.054	0.118	0.003	-0.539	-0.747	7.55E-21
TSPAN3	0.194	-0.025	-0.13	0.138	0.361	7.56E-21
POLH	0.533	0.628	0.621	0.578	0.553	7.70E-21
PXN	0.16	-0.18	0.034	0.196	0.461	7.83E-21
CASP6	0.081	-0.071	-0.07	0.248	0.687	7.84E-21
TMEM237	-0.198	-0.13	0.016	0.033	0.653	7.95E-21

LINC00205	-0.439	0.04	0.333	0.073	0.221	8.03E-21
FAM73B	-0.478	0.085	-0.129	-0.653	-0.684	8.45E-21
COL5A2	0.165	0.155	0.097	0.521	0.914	8.48E-21
EEF1A1	0.11	0.109	0.195	0.261	0.502	8.48E-21
BCAR3	-0.206	-0.11	-0.062	0.265	0.516	8.59E-21
GRB10	0.399	-0.194	-0.386	-0.126	0.198	8.59E-21
PCDH9	-0.139	-0.191	-0.977	-0.716	-0.725	8.72E-21
NMNAT1	-0.27	0.047	0.277	0.421	0.456	8.74E-21
CNKSR3	0.523	0.712	-0.058	-0.116	0.63	8.75E-21
ARG2	-0.304	-0.034	0.276	0.522	0.878	9.01E-21
RELL1	-0.087	0.183	0.182	0.173	0.658	9.07E-21
ABLIM3	0.009	-0.126	0.273	0.214	0.556	9.35E-21
CAB39	0.334	0.393	0.157	0.445	0.782	9.79E-21
BUB1B	-0.606	-0.783	-0.771	-0.304	-0.575	1.00E-20
MTUS1	-0.349	0.057	0.097	0.187	-0.163	1.02E-20
TWF2	0.268	0.04	0.007	0.455	0.844	1.03E-20
ZNF710	-0.079	-0.216	0.121	0.292	0.607	1.04E-20
RASA1	-0.199	0.041	0.003	0.204	0.531	1.04E-20
WBP1L	-0.052	0.144	0.134	0.263	0.465	1.06E-20
FAM131A	-0.668	-0.29	-0.072	-0.07	0.155	1.06E-20
EED	0.183	0.165	-0.05	-0.153	-0.37	1.07E-20
MAPKBP1	-0.496	0.142	0.439	-0.101	0.436	1.08E-20
NEURL4	-0.654	-0.14	-0.114	-0.713	-0.742	1.08E-20
TBC1D16	0.31	-0.133	-0.157	-0.123	0.512	1.09E-20
NDE1	-0.486	-0.574	-0.336	0.08	0.28	1.16E-20
WLS	0.26	0.03	-0.162	0.124	0.512	1.18E-20
ERAP2	-0.089	0.415	0.889	0.322	1.076	1.20E-20
EHD4	0.338	-0.156	-0.082	0.356	0.687	1.20E-20
UNKL	-0.079	0.372	0.183	0.009	-0.459	1.21E-20
RNF115	0.137	-0.09	-0.168	-0.228	-0.35	1.22E-20
RMND5A	0.336	0.128	0.073	0.296	0.627	1.26E-20
MXD3	-1.364	-1.093	-0.508	-0.476	-1.281	1.28E-20
MTMR2	0.106	-0.036	-0.143	-0.082	-0.489	1.39E-20
STARD4	0.203	-0.103	-0.297	-0.69	-0.564	1.39E-20
RNF10	0.166	0.068	0.08	0.344	0.607	1.40E-20
PAPD5	0.692	0.443	0.473	0.58	0.809	1.42E-20
PRKCA	0.484	0.01	0.109	0.498	0.695	1.52E-20
RP6-99M1.2	-1.032	0.2	0.403	-0.724	-0.388	1.52E-20

S100A10	0.175	0.03	0.092	0.256	0.493	1.55E-20
TOB1	0.566	0.519	0.303	0.448	0.939	1.59E-20
CFAP97	0.059	0.156	0.358	0.484	0.653	1.68E-20
NCS1	0.429	0.086	-0.089	0.129	0.685	1.72E-20
LINS1	-0.068	0.207	-0.074	-0.51	-0.752	1.76E-20
FYCO1	-0.29	0.117	0.438	0.306	0.285	1.80E-20
KDSR	0.417	0.153	-0.037	0.049	0.307	1.87E-20
HERC2P2	-1.062	-0.202	0.08	-0.937	-0.873	1.88E-20
KLHL6	0.408	-0.056	0.177	0.442	1.119	1.92E-20
SFXN5	0.03	0.43	0.198	-0.22	-0.179	1.92E-20
JRK	-0.557	-0.195	-0.074	-0.543	-0.763	1.96E-20
IL18R1	0.281	0.206	-0.164	-0.582	-0.603	1.99E-20
ATP9A	0.335	0.148	0.156	0.234	0.768	2.03E-20
RUSC1	0.157	0.22	-0.049	-0.182	-0.34	2.03E-20
TTLL5	0.127	-0.076	0.113	0.48	0.724	2.12E-20
AC019178.1	0.411	0.017	-0.066	0.305	0.611	2.12E-20
GGA3	-0.395	0.007	-0.093	-0.457	-0.443	2.12E-20
PRPS1	0.196	-0.062	-0.255	-0.131	-0.483	2.19E-20
TNIP2	-0.077	0.215	0.088	0.036	0.507	2.21E-20
RP11-296E7	1.028	0.366	0.245	0.293	0.532	2.34E-20
STAT2	-0.388	0.041	0.141	-0.332	-0.486	2.35E-20
AC068831.1	-1.251	-0.793	-0.544	-1.205	-1.571	2.36E-20
HECW2	0.315	0.232	0.336	0.219	0.651	2.47E-20
HMGCL	0.185	0.239	0.375	0.744	0.659	2.49E-20
ZNF33B	-0.173	-0.074	0.345	0.313	0.533	2.52E-20
PITPNC1	0.142	-0.044	-0.068	0.158	1.078	2.63E-20
AMH	-0.33	0.016	-0.482	-1.649	-1.757	2.66E-20
GLIPR1	0.13	0.153	0.096	0.115	0.545	2.83E-20
BRWD3	-0.349	0.15	0.16	-0.352	-0.602	2.83E-20
YWHAQ	0.278	0.103	-0.003	0.28	0.446	2.85E-20
SPTBN5	-0.609	-0.293	0.148	-0.775	0.548	2.88E-20
CHN1	0.14	-0.101	-0.039	0.003	0.815	2.92E-20
CLDN7	-0.571	-0.174	0.194	-0.083	0.823	2.94E-20
CTSZ	0.222	0.065	0.014	0.463	0.755	2.95E-20
CENPP	-0.236	0.154	0.16	0.244	-0.647	2.98E-20
TMEM41A	0.117	0.078	-0.206	-0.179	-0.513	3.10E-20
LDLRAD3	0.325	-0.094	0.029	0.327	0.63	3.12E-20
CEACAM19	-0.353	0.227	0.85	0.09	0.919	3.17E-20

RNF168	0.133	-0.06	-0.064	-0.093	-0.517	3.27E-20
RBL2	-0.215	-0.142	0.09	0.169	0.443	3.35E-20
MLLT1	0.375	-0.232	-0.286	-0.191	-0.485	3.36E-20
GBP1	0.239	0.367	0.266	0.454	0.9	3.39E-20
PGM2L1	0.328	0.367	0.166	0.398	0.822	3.45E-20
TPM1	0.436	-0.047	-0.275	-0.208	0.065	3.47E-20
KPNA2	-0.265	-0.661	-0.721	0.018	0.187	3.78E-20
TMEM64	0.515	0.101	-0.078	0.098	0.293	3.81E-20
NRARP	0.472	-0.237	-0.024	0.282	0.665	3.90E-20
FICD	-0.047	0.094	-0.277	-0.431	-0.819	3.91E-20
HHEX	0.627	0.135	-0.096	-0.117	-0.137	4.46E-20
NECAP1	0.105	0.245	-0.035	-0.059	-0.481	4.77E-20
PPP2R5B	-0.546	0.257	0.231	-0.053	0.109	4.82E-20
ANAPC15	-0.012	-0.025	-0.131	-0.212	-0.59	4.85E-20
RBM12B	-0.302	0.139	-0.021	-0.345	-0.533	5.08E-20
TNC	0.512	0.138	-0.034	-0.016	1.301	5.12E-20
SUMF1	0.153	0.109	0.093	0.435	0.743	5.13E-20
NT5DC3	-0.012	-0.197	0.086	-0.672	-0.784	5.13E-20
MCFD2	0.074	-0.101	-0.074	0.146	0.399	5.30E-20
RNF207	-0.575	0.576	1.127	0.147	1.303	5.31E-20
SIN3B	-0.27	0.115	0.333	0.059	0.33	6.27E-20
AC093509.1	-0.173	-0.182	-0.046	-0.137	-0.958	6.35E-20
C11orf71	-0.232	0.051	-0.006	-0.292	-1.258	6.56E-20
PHKG2	-0.212	-0.038	-0.243	-0.57	-0.686	6.60E-20
PTEN	0.157	0.241	0.099	0.303	0.6	6.80E-20
CIT	-0.663	-0.603	-0.414	-0.393	-0.273	6.90E-20
SLC24A1	-0.441	-0.24	0.187	0.194	0.401	6.92E-20
PLD3	0.167	-0.008	-0.056	0.404	0.738	6.98E-20
CAT	0.133	-0.078	-0.146	0.382	0.651	6.98E-20
RP11-793E1	0.434	-0.113	-0.428	-0.311	-1.228	7.06E-20
RACK1	0.181	0.035	0.056	0.235	0.422	7.09E-20
MGA	-0.582	-0.078	0.113	-0.006	0.173	7.15E-20
DNAJC3	0.401	0.028	-0.116	-0.006	-0.387	7.21E-20
NOP16	0.02	-0.142	-0.306	-0.161	-0.614	7.28E-20
CENPL	-0.587	-0.583	-0.497	-0.597	-1.02	7.32E-20
ZBTB1	0.354	0.093	0.28	0.428	0.657	8.15E-20
GRAMD1A	-0.191	-0.057	0.097	-0.043	0.492	8.21E-20
ERAP1	0.315	0.158	0.082	0.467	0.798	8.50E-20

EXD2	-0.006	0.034	0.261	0.474	0.62	8.77E-20
CTB-50E14.5	0.676	0.248	0.138	0.212	0.751	8.97E-20
RQCD1	0.267	0.044	-0.092	-0.056	-0.324	9.01E-20
CTD-2228K2	-0.896	0.313	0.5	-0.552	-0.705	9.03E-20
ACOT11	0.536	-0.113	-0.307	-0.139	-0.657	9.22E-20
FAM83G	0.683	0.227	0.777	0.802	0.627	9.29E-20
LIMK1	0.152	-0.134	-0.032	0.294	0.669	9.73E-20
ZBTB40	-0.281	0.336	0.369	-0.18	-0.088	9.82E-20
FXR1	0.157	0.185	0.147	0.422	0.631	9.84E-20
GOLGA2	0.04	0.098	0.038	-0.357	-0.42	9.87E-20
LRBA	0.387	0.13	0.084	0.549	0.833	1.00E-19
APLP2	0.354	0.165	0.053	0.478	0.846	1.01E-19
TEX30	-0.374	-0.062	-0.173	-1.019	-1.398	1.05E-19
RP11-22B23	-0.609	-0.052	-0.102	-1.107	-1.765	1.05E-19
PDE4DIP	-0.197	0.197	0.212	0.29	0.478	1.10E-19
KCMF1	0.368	-0.064	-0.173	-0.189	-0.195	1.12E-19
VGLL4	-0.02	0.424	0.161	-0.222	0.257	1.15E-19
XXyac-R12D	0.236	0.224	0.205	0.552	0.791	1.20E-19
PRDM2	0.465	0.137	-0.046	-0.213	-0.042	1.20E-19
WEE1	-0.493	-0.498	-0.554	-0.643	-0.618	1.25E-19
FAM63B	-0.179	0.168	0.412	0.565	0.42	1.26E-19
LOXL1	0.088	0.022	0.045	0.229	0.935	1.28E-19
SYNE2	-0.225	0.147	-0.187	-0.296	-0.966	1.28E-19
SMAD7	1.419	-0.189	0.102	0.287	-0.718	1.29E-19
TWIST2	0.002	-0.308	-0.21	0.097	0.991	1.32E-19
AC005264.2	-0.242	0.906	1.478	1.136	0.997	1.34E-19
ATP13A2	0.044	-0.115	0.006	0.31	0.536	1.38E-19
CABYR	0.359	0.873	0.859	0.954	1.769	1.39E-19
CTC-259J15	0.383	0.161	0.238	0.613	0.799	1.45E-19
TWSG1	0.103	0.113	-0.051	0.097	0.442	1.53E-19
SNORD86	-0.457	-0.421	-0.266	-1.518	-1.325	1.58E-19
CH507-42P1	-0.168	0.453	0.225	0.244	1.068	1.65E-19
RAPGEF2	0.338	0.169	0.185	0.34	0.613	1.67E-19
AD000090.2	-0.739	0.122	0.034	-0.753	-0.96	1.72E-19
SLC16A4	0.093	0.026	-0.025	0.427	0.931	1.73E-19
AP000347.2	-0.556	0.158	0.508	-0.865	-0.613	1.73E-19
MSX1	0.501	0.363	-0.182	-0.319	0.573	1.75E-19
ROR1	0.448	0.121	0.182	0.339	0.89	1.90E-19

GSAP	-0.403	-0.2	0.679	0.402	1.388	1.91E-19
MBD4	0.142	-0.07	-0.067	-0.301	-0.406	1.95E-19
ARID4B	0.119	0.397	0.046	0.081	0.454	1.97E-19
HMGA1P2	0.231	0.098	0.171	0.529	1.018	1.98E-19
RPL4	0.2	0.119	0.072	0.248	0.395	1.98E-19
MADD	0.231	0.092	-0.024	-0.071	-0.324	2.01E-19
CTD-2270P1	-0.857	0.042	0.124	-0.876	-0.919	2.05E-19
CCSER2	0.851	0.469	0.056	0.13	0.071	2.18E-19
IFRD1	-0.095	-0.134	-0.213	-0.537	-0.507	2.19E-19
TEP1	-0.06	0.156	0.277	-0.165	0.453	2.27E-19
HADHB	0.268	0.17	0.132	0.483	0.678	2.30E-19
LRPAP1	0.163	0.241	0.122	0.489	0.736	2.32E-19
NCOR1	0.057	-0.011	0.068	0.323	0.605	2.33E-19
CENPJ	-0.146	-0.027	-0.126	-0.572	-0.755	2.46E-19
THAP5	-0.097	0.082	-0.145	-0.368	-0.65	2.66E-19
HYI	-0.679	-0.185	0.191	-0.014	0.557	2.72E-19
RP11-322E1	-0.559	-0.142	0.348	0.249	1.574	2.75E-19
NAA16	-0.054	-0.055	-0.134	-0.579	-0.586	2.75E-19
ACTN1	0.4	0.061	0.043	0.49	0.996	2.88E-19
POLE3	0.097	-0.076	-0.17	-0.144	-0.346	2.89E-19
ABCA6	-0.239	0.101	0.069	-0.253	-0.664	2.91E-19
THSD4	0.439	0.093	-0.292	-0.051	0.595	2.97E-19
PDLIM7	0.169	-0.155	-0.034	-0.054	0.335	3.01E-19
LPP	0.448	0.051	0.021	0.268	0.687	3.05E-19
CDC25B	-0.455	-0.642	-0.403	-0.207	-0.433	3.25E-19
ZDHHC9	0.174	0.094	0.083	0.335	0.693	3.57E-19
ALCAM	0.113	0.106	-0.106	0.162	-0.617	3.75E-19
VAMP5	-0.238	-0.26	-0.114	-0.109	0.47	3.81E-19
RNF219	0.244	-0.144	-0.306	-0.12	-0.42	3.97E-19
RP5-857K21	-0.331	-0.333	0.028	-1.491	-1.356	4.15E-19
CTD-2119F7	0.258	0.244	0.257	0.657	0.439	4.38E-19
TRMT1L	-0.413	0.182	0.055	0.424	0.132	4.44E-19
IKBKB	-0.246	0.115	0.393	0.069	0.376	4.45E-19
USP49	0.254	0.656	0.532	-0.321	-0.154	4.45E-19
SPDL1	-0.195	-0.425	-0.282	0.037	-0.24	4.49E-19
PSMG1	0.101	-0.063	-0.245	-0.1	-0.539	4.51E-19
TTYH3	0.236	0.032	-0.014	0.249	0.707	4.58E-19
CROT	-0.057	0.643	0.603	0.592	0.671	4.64E-19

ZBTB21	0.403	0.72	0.465	0.356	0.362	4.64E-19
GLB1	0.25	0.097	-0.02	0.466	0.705	4.66E-19
PHLDB1	0.579	0.168	0.268	0.138	0.469	4.70E-19
TOR3A	-0.093	-0.085	-0.188	-0.175	-0.722	4.70E-19
EI24	0.256	0.294	0.313	0.472	0.472	4.72E-19
KATNAL1	0.278	0.066	0.076	0.003	-0.349	4.83E-19
NLRC5	0.072	0.043	0.322	0.14	0.833	4.88E-19
VPS35	0.27	0.017	0.025	0.459	0.623	4.99E-19
FAM160A2	-0.574	-0.066	0.055	-0.527	-0.478	5.15E-19
MIR1304	-0.378	0.071	0.032	-1.143	-1.197	5.16E-19
RPS6KB2	-0.103	0.023	-0.121	-0.397	-0.547	5.19E-19
AGAP1	0.548	0.003	0.06	0.465	0.583	5.21E-19
RP4-591C20	-0.897	-0.043	-0.189	-0.547	-0.803	5.29E-19
NFKBID	-0.118	0.032	-0.163	-1.01	-1.057	5.38E-19
MTHFR	-0.621	-0.098	0.086	-0.04	-0.175	5.49E-19
BMP6	0.148	0.073	-0.165	0.09	-0.717	5.49E-19
FBF1	-0.765	0.097	0.236	-0.37	-0.544	5.51E-19
RP11-95D17	0.116	0.067	0.162	0.705	1.117	5.56E-19
CCDC92	0.361	0.228	0.286	0.355	0.72	5.79E-19
PLTP	0.366	0.239	0.083	0.454	0.999	5.88E-19
ITGAV	0.42	0.232	-0.148	0.001	-0.57	5.89E-19
GIMAP2	-0.802	-0.168	0.101	0.16	0.218	5.92E-19
RP11-513I15	-0.522	0.074	0.764	0.943	0.804	6.01E-19
FOXRED1	-0.045	-0.005	-0.061	-0.211	-0.681	6.01E-19
FAM26E	-0.002	-0.311	-0.65	-0.425	-0.327	6.08E-19
SLC26A6	-0.488	-0.271	-0.261	-0.999	-1.061	6.17E-19
HSPA5	0.499	0.032	-0.26	0.011	-0.539	6.28E-19
ZNF219	-0.224	0.374	0.716	0.486	1.365	6.42E-19
MCM7	0.471	0.088	-0.221	-0.182	-0.443	6.48E-19
CENPT	-0.472	-0.071	-0.077	-0.633	-0.723	6.49E-19
MT-RNR1	-0.832	-0.299	0.191	-1.281	-0.985	6.59E-19
ITGB8	-0.633	0.507	0.404	0.26	0.449	6.64E-19
MDC1	0.007	-0.106	-0.215	-0.411	-0.508	6.78E-19
MAP1LC3B2	0.108	0.045	0.077	0.287	0.69	6.96E-19
PLAGL1	-0.165	0.249	0.244	-0.268	-0.342	7.01E-19
SYDE1	-0.395	-0.135	-0.352	-0.663	-0.493	7.09E-19
CYB561D2	0.001	-0.151	-0.108	0.006	-0.694	7.15E-19
PALMD	0.169	-0.041	-0.045	0.237	-0.455	7.25E-19

DENND3	-0.097	0.238	0.583	0.333	0.271	7.27E-19
KIAA1671	0.132	0.269	0.408	0.524	0.8	7.32E-19
TYK2	-0.157	0.054	0.061	-0.289	-0.503	7.37E-19
TOP3A	-0.058	-0.051	-0.215	-0.303	-0.626	7.74E-19
DCAF15	-0.206	-0.142	-0.282	-0.543	-0.766	7.94E-19
RHBDD3	-0.165	0.076	-0.184	-0.344	-0.775	8.24E-19
UVSSA	-0.367	0.023	0.245	-0.445	-0.702	8.27E-19
C11orf91	0.233	0.719	0.73	-0.298	-0.85	8.36E-19
CACNG8	-0.361	-0.238	-0.001	-0.937	-1.149	8.65E-19
IL21R	1.534	0.253	-0.048	0.011	-1.148	8.67E-19
PREX1	0.345	-0.273	-0.469	-0.25	-0.686	8.78E-19
LSM11	0.449	0.025	-0.068	0.029	-0.285	8.87E-19
KHDRBS3	0.024	0.026	0.161	0.591	0.704	8.95E-19
PRR5L	-0.335	-0.382	-0.205	-0.385	0.888	9.06E-19
LPCAT3	0.079	0.123	-0.012	0.191	-0.612	9.13E-19
CDK17	-0.12	-0.028	0	0.065	0.349	9.30E-19
NMI	0.168	0.07	0.156	0.578	0.672	9.62E-19
MFSD6	0.216	0.496	0.382	0.614	1.45	9.69E-19
KIAA0430	-0.026	0.116	0.246	0.338	0.408	9.82E-19
UBE2SP1	-0.221	-0.65	-0.937	-0.703	-0.598	9.90E-19
ZBED1	0.435	0.196	-0.213	0.094	0.582	9.96E-19
TOP2A	-0.618	-0.903	-0.728	-0.06	-0.241	1.02E-18
UBXN1	0.182	0.185	0.2	0.41	0.593	1.03E-18
JADE2	-0.278	-0.168	0.102	0.162	0.382	1.03E-18
LRRFIP1	0.285	-0.103	0.055	0.429	0.535	1.05E-18
SKP2	-0.319	-0.282	-0.331	-0.302	-0.812	1.06E-18
WDR45	-0.187	0.04	0.324	0.206	0.664	1.07E-18
DCK	-0.013	-0.172	-0.312	-0.507	-0.704	1.07E-18
FBXL17	0.288	0.022	0.203	0.442	0.916	1.09E-18
CARD6	0.435	0.353	0.334	0.656	0.762	1.10E-18
SPATS2	0.136	0.033	0.056	0.284	0.486	1.10E-18
TMTC4	-0.123	-0.059	0.131	0.142	0.743	1.12E-18
ALG6	-0.122	0.046	0.045	0.081	-0.598	1.12E-18
CABIN1	-0.06	-0.258	0.057	0.233	0.392	1.15E-18
MYEOV	-0.52	0.083	0.268	0.144	0.874	1.17E-18
MFSD3	-0.698	-0.14	-0.184	-0.296	0.606	1.17E-18
CAPZA2	-0.23	-0.255	-0.383	-0.755	-1.019	1.20E-18
RF02271	-0.804	0.102	0.064	-0.994	-0.975	1.21E-18

TUBGCP5	0.151	0.073	0.026	-0.05	-0.534	1.22E-18
DZIP1L	-0.239	-0.02	-0.127	-0.85	-1.026	1.23E-18
ZNF142	0.009	0.036	-0.056	-0.309	-0.548	1.25E-18
UBE2R2	0.278	-0.002	-0.034	0.296	0.685	1.26E-18
ARL6IP5	-0.125	-0.183	-0.148	0.083	0.335	1.26E-18
ULBP2	0.365	0.223	-0.049	0.062	-0.767	1.27E-18
GIT2	-0.099	-0.086	0.116	0.288	0.508	1.30E-18
CXCL3	0.966	0.711	-0.194	-0.468	0.981	1.31E-18
RGS17	-0.448	-0.141	0.339	-0.051	1.074	1.38E-18
EPB41L1	0.192	-0.15	-0.028	0.437	0.776	1.40E-18
TNKS	-0.106	0.21	0.149	0.263	0.624	1.42E-18
MICAL2	0.463	-0.059	0.103	0.525	0.731	1.50E-18
DHX40	0.104	0.238	0.255	0.446	0.501	1.58E-18
HTRA1	0.344	0.055	-0.093	0.315	0.654	1.59E-18
GDI1	-0.065	-0.018	-0.028	-0.301	-0.419	1.62E-18
BCOR	0.966	-0.26	0.188	0.352	0.517	1.71E-18
CCDC150	-0.6	-0.055	-0.106	-0.927	-1.418	1.73E-18
RP11-540B6	-0.402	0.155	0.129	-0.772	-0.932	1.78E-18
ABL1	0.452	-0.128	0.075	0.424	0.59	1.79E-18
SEC11C	-0.147	-0.138	-0.085	-0.175	-1.15	1.81E-18
THBS1	0.212	0.033	-0.255	-0.194	-0.64	1.83E-18
DBF4B	-0.861	-0.517	-0.437	-0.804	-0.94	1.84E-18
TSC22D4	-0.676	-0.149	-0.024	-0.145	0.043	1.98E-18
PGPEP1	-0.753	0.03	0.427	0.037	0.308	2.00E-18
EZH1	-0.589	0.059	0.333	-0.41	-0.48	2.09E-18
CASP10	-0.109	0.349	0.312	0.334	0.577	2.13E-18
NDUFC2	0.005	-0.181	-0.276	-0.279	-0.76	2.16E-18
GALC	0.011	0.059	-0.137	0.182	-0.629	2.23E-18
CLDN11	0.059	-0.04	0.268	0.48	0.834	2.34E-18
NOA1	-0.021	-0.148	-0.026	0.162	0.558	2.35E-18
HNRNPA1P7	0.222	0.219	0.265	0.624	0.475	2.40E-18
RP11-456N1	-1.062	-0.407	0.398	-0.358	1.187	2.54E-18
C3orf67	0.687	1.4	1.49	1.296	1.099	2.67E-18
GMPPA	0.119	0.041	-0.058	0.051	-0.529	2.69E-18
HK2	-0.655	-0.375	-0.519	-0.586	-1.128	2.71E-18
BLM	0.383	0.254	-0.006	0.013	-0.539	2.73E-18
PPP3CA	0.244	-0.113	-0.152	0.092	0.781	2.81E-18
MEIS1	-0.57	-0.072	-0.369	-0.39	-0.796	2.82E-18

SREBF1	0.096	-0.033	-0.165	-0.147	0.465	2.88E-18
C14orf1	0.218	-0.109	-0.336	-0.234	-0.318	2.91E-18
VPS50	-0.03	0.184	0.366	0.524	0.419	3.14E-18
CLIC3	0.484	-0.038	-0.39	-0.193	-1.018	3.14E-18
UPP1	-0.237	-0.014	0.033	-0.152	-0.599	3.18E-18
EVA1C	0.239	-0.196	0.127	0.237	-0.386	3.19E-18
FOXJ3	0.172	0.046	0.086	0.094	0.461	3.22E-18
MBD6	-0.355	-0.015	-0.103	-0.462	-0.433	3.36E-18
BTG3	0.004	0.096	-0.217	-0.581	-0.539	3.39E-18
NOL12	-0.554	-0.181	-0.117	-0.699	-0.982	3.39E-18
SGCE	-0.023	-0.174	-0.177	0.099	0.391	3.52E-18
RP11-110C1	0.185	0.022	0.021	0.211	0.328	3.58E-18
BAX	0.229	0.339	0.427	0.561	0.477	3.62E-18
MAP3K12	-0.865	-0.317	-0.059	-0.528	-0.128	3.74E-18
FRMD8	0.421	0.058	0.077	0.01	0.305	3.84E-18
PPP1R12B	-0.731	0.02	0.198	-0.598	-0.696	3.89E-18
SZT2	-0.287	0.111	0.16	-0.41	-0.514	3.99E-18
AIFM2	0.23	0.27	0.136	0.402	-0.521	4.07E-18
HAUS2	0.143	0.062	-0.047	-0.133	-0.412	4.18E-18
IL18BP	-0.086	0.037	-0.003	-0.415	-0.64	4.19E-18
HMGA2	-0.127	-0.11	0.17	0.394	0.057	4.27E-18
SNORA73A	-0.256	0.097	0.088	-0.904	-1.395	4.33E-18
DCLRE1A	-0.301	-0.235	-0.195	-0.113	-0.774	4.49E-18
LRRC8C	0.327	0.028	-0.235	-0.033	-0.207	4.50E-18
AP2B1	0.299	0.102	0.094	0.456	0.864	4.54E-18
HAGH	-0.166	-0.098	-0.011	0.282	0.44	4.57E-18
AC017048.3	-0.158	-0.179	0.203	0.476	0.351	4.59E-18
ZDHHC13	-0.512	-0.269	-0.022	-0.046	0.38	4.64E-18
LIN52	-0.1	-0.239	-0.467	-0.45	-0.744	4.66E-18
GVINP1	-0.311	-0.054	0	-0.137	1.114	4.71E-18
SNORA61	-0.226	0.053	0.027	-1.161	-1.147	4.74E-18
SIPA1L2	0.565	0.643	0.239	0.407	0.737	4.80E-18
HEXA	0.188	0.09	0.067	0.33	0.46	4.85E-18
ATP6V0B	-0.148	-0.111	-0.158	-0.389	-0.704	4.97E-18
SLC25A13	0.281	-0.035	0.021	0.395	0.625	4.99E-18
MRI1	-0.479	-0.065	-0.065	-0.655	-0.644	5.04E-18
SLC26A11	-0.032	0.075	-0.031	-0.445	-0.526	5.08E-18
GRSF1	0.144	0.003	-0.042	0.217	0.463	5.10E-18

ZEB2	0.536	-0.278	-0.29	-0.166	-0.317	5.14E-18
TRIM5	0.481	0.572	0.583	0.763	0.977	5.23E-18
SLC43A3	-0.124	-0.083	0.17	0.387	0.432	5.49E-18
SCN5A	-0.082	0.153	0.318	0.447	0.535	5.64E-18
AC069513.3	-0.114	0.195	0.196	-0.89	-0.744	5.64E-18
SLF1	0.047	-0.224	-0.513	-0.48	-0.81	5.69E-18
KLF12	0.18	-0.051	0.345	0.294	0.624	5.70E-18
LTB4R	-0.522	0.214	0.367	-0.65	-0.727	5.79E-18
DUSP8	0.16	0.711	0.757	0.09	1.221	5.89E-18
MIR17HG	-0.748	-0.332	-0.407	-1.57	-1.362	5.89E-18
RP11-54A4.2	-0.886	-0.026	0.426	-0.527	-1.072	6.32E-18
THOC6	-0.135	-0.277	0.014	-0.042	-0.725	6.44E-18
BCL10	-0.072	0.125	0.17	0.218	0.507	6.89E-18
KANK1	0.306	-0.313	0.073	0.327	0.313	6.89E-18
GPR37	0.83	0.095	0.014	0.265	0.153	6.97E-18
MT-ATP6	-0.453	-0.342	0.265	-1.205	-1.1	7.07E-18
B3GNT5	-0.137	0.12	0.03	0.063	0.643	7.08E-18
RGS2	-0.841	-0.599	-0.481	-0.422	0.672	7.09E-18
ZNF337	-0.029	0.53	0.69	-0.109	0.107	7.56E-18
FAT1	0.318	0.12	0.191	0.27	1.14	7.61E-18
IL17RA	0.134	-0.186	-0.255	-0.257	-0.581	7.82E-18
SAMD9	0.374	0.304	0.429	0.83	0.996	7.92E-18
MAZ	-0.04	-0.242	-0.263	-0.288	-0.461	7.96E-18
FAM161A	-0.351	-0.141	-0.111	-0.481	-1.311	7.96E-18
CRELD2	0.17	-0.085	-0.263	-0.032	-0.691	8.17E-18
TCIRG1	-0.264	0.146	0.046	-0.486	-0.606	8.28E-18
DCP1B	0.72	0.792	0.897	0.851	0.928	8.31E-18
MLLT10	-0.532	-0.16	0.06	-0.184	-0.236	8.49E-18
NUSAP1	-0.384	-0.357	-0.372	-0.147	-0.588	8.57E-18
LZTFL1	0.181	0.047	0.077	0.408	0.818	8.69E-18
TMEM163	0.161	-0.244	0.024	0.198	1.082	8.95E-18
AP000640.9	-0.047	-0.216	-0.293	-0.493	0.383	9.19E-18
CCDC102A	-0.346	0.102	0.144	0.082	0.858	9.29E-18
NUP54	0.155	0.011	-0.189	-0.099	-0.367	9.32E-18
WDFY1	0.198	-0.018	-0.042	0.262	0.54	9.34E-18
LINC00338	-0.854	-0.045	0.156	-0.578	-0.499	9.47E-18
SLC35E2	-0.513	0.065	0.156	-0.199	0.218	9.89E-18
TIMM21	-0.04	-0.011	-0.046	-0.106	-0.616	9.89E-18

MINK1	-0.211	0.122	0.317	0	0.355	1.01E-17
MGST2	-0.045	-0.156	-0.086	0.136	0.483	1.02E-17
FAM64A	-0.647	-0.906	-0.943	-0.436	-0.39	1.02E-17
FAM189A2	-0.361	0.024	0.37	-0.175	0.742	1.03E-17
RGS12	-0.022	0.082	0.227	0.14	0.518	1.03E-17
DHODH	-0.054	-0.033	-0.137	-0.235	-0.894	1.04E-17
UFM1	0.183	0.127	-0.034	-0.338	-0.39	1.05E-17
C11orf95	0.09	0.065	-0.254	-0.503	0.321	1.06E-17
TNPO2	-0.372	-0.084	-0.182	-0.573	-0.491	1.07E-17
AC004383.4	-1.754	-0.275	0.089	-0.781	-0.507	1.07E-17
NPEPPS	0.213	0.117	0.157	0.377	0.614	1.09E-17
CENPU	0.127	0.13	-0.019	-0.35	-0.709	1.09E-17
TAGLN	0.268	0.042	0.149	-0.09	-1.029	1.11E-17
UBTF	0.375	-0.227	-0.221	0.07	0.332	1.13E-17
RGS4	-0.379	-0.102	0.009	0.5	0.376	1.16E-17
CASKIN2	0.478	-0.037	-0.066	-0.044	-0.527	1.16E-17
AK2	0.07	-0.059	-0.202	-0.176	-0.333	1.17E-17
SAT1	0.15	0.565	0.196	-0.582	-0.45	1.17E-17
TXNRD1	0.378	0.149	-0.216	-0.095	-0.664	1.17E-17
SFI1	-0.385	-0.2	-0.035	-0.62	-0.755	1.18E-17
SKAP2	0.191	0.105	0.081	0.531	0.678	1.19E-17
TRIM47	0.415	0.508	0.322	0.324	0.901	1.20E-17
PSAP	0.269	0.083	-0.014	0.406	0.705	1.23E-17
ADGRL1	0.349	-0.028	-0.413	-0.627	-0.937	1.24E-17
GIGYF1	-0.31	0.133	0.093	-0.721	-0.598	1.25E-17
ELOVL6	0.104	-0.287	-0.361	-0.247	-0.87	1.25E-17
SPAG5	-0.351	-0.506	-0.489	-0.255	-0.655	1.26E-17
TOE1	-0.008	-0.199	-0.255	-0.41	-0.847	1.27E-17
SPATA33	-0.23	-0.104	-0.125	-0.535	-0.774	1.28E-17
CARD10	-0.096	0.065	0.304	0.391	0.617	1.29E-17
SGK1	0.285	0.012	-0.16	-0.251	-0.668	1.31E-17
PDGFD	0.017	-0.018	0.198	0.587	1.206	1.34E-17
POLR3K	-0.22	-0.305	-0.479	-0.49	-0.704	1.34E-17
GATA3	-0.016	-0.298	-0.483	-0.128	-1.134	1.35E-17
AC087590.3	-0.199	0.04	0.073	-0.97	-1.215	1.35E-17
MCCC2	0.316	0.121	0.215	0.427	0.911	1.38E-17
CTPS2	-0.064	-0.181	0.072	0.406	0.662	1.42E-17
RALGAPB	-0.017	0.287	0.225	0.096	0.341	1.42E-17

CACNG6	0.325	-0.254	-0.424	-0.317	-1.123	1.42E-17
TBC1D2B	0.058	-0.085	0.163	0.137	0.356	1.48E-17
RP11-77I22.:	0.21	0.103	-0.022	0.065	-0.774	1.52E-17
DAB2IP	0.55	-0.14	-0.063	0.09	0.222	1.55E-17
HOXA10	0.178	-0.518	-0.322	0.096	0.033	1.57E-17
MAT2A	-0.304	-0.214	-0.17	-0.676	-0.461	1.57E-17
DPH2	0.036	-0.004	-0.057	-0.16	-0.434	1.65E-17
MT-ND2	-0.42	-0.305	0.325	-1.188	-1.099	1.77E-17
EBAG9	-0.031	-0.004	0.037	-0.048	-0.752	1.78E-17
ADRA1B	0.417	-0.525	-0.711	-0.283	-0.769	1.79E-17
DAGLA	-0.335	0.214	0.361	0.304	0.589	1.80E-17
CENPI	-0.693	-0.341	-0.177	-0.247	-0.461	1.83E-17
C9orf37	-0.779	-0.185	-0.252	-0.722	-0.936	1.83E-17
NATD1	-0.019	0.172	-0.083	0.088	0.805	1.87E-17
PSME4	0.188	0.354	0.276	0.501	0.584	1.87E-17
SLC47A1	0.107	0.453	0.59	0.755	0.406	1.90E-17
KIAA0895L	-0.296	0.002	-0.022	-1.026	-0.833	1.90E-17
NAV1	0.299	-0.301	0.023	0.23	0.405	1.91E-17
CEP55	-0.165	-0.452	-0.52	0.06	0.013	1.91E-17
NR2F6	-0.239	-0.415	-0.109	0.18	0.585	1.95E-17
MT-ND6	-0.458	-0.108	0.317	-1.218	-1.093	1.95E-17
ANLN	-0.33	-0.606	-0.621	-0.194	-0.346	1.99E-17
LYRM7	-0.423	-0.36	-0.217	-0.331	-0.752	2.02E-17
NF1	-0.268	0.221	0.102	-0.002	0.362	2.06E-17
CERS6	0.242	0.071	-0.231	-0.165	-0.71	2.09E-17
AGER	-0.639	0.045	0.124	-0.924	-1.043	2.10E-17
ENOSF1	-0.715	-0.322	-0.15	-0.769	-0.91	2.14E-17
EOGT	0.298	0.112	0.085	0.175	0.38	2.20E-17
RP11-676M6	0.277	0.051	0.041	0.289	0.689	2.26E-17
EFCAB14	0.052	0.06	0.052	0.173	0.461	2.27E-17
TP53I3	0.077	0.376	0.409	0.265	0.397	2.27E-17
GDF6	0.053	0.672	0.721	0.611	1.424	2.30E-17
ABCA5	-0.313	0.471	0.547	0.164	-0.163	2.30E-17
GLUL	0.506	0.142	-0.19	0.123	0.512	2.40E-17
BTBD1	0.224	0.074	0.006	0.19	0.463	2.41E-17
PRPF3	-0.233	0.099	0.105	-0.292	-0.385	2.46E-17
RP11-112J1.	-0.032	-0.201	-0.075	0.126	0.883	2.49E-17
OAS3	0.171	0.472	0.562	0.817	0.948	2.52E-17

FAM160B1	0.354	0.116	0.087	0.096	0.523	2.54E-17
RP5-857K21	-0.434	-0.405	0.256	-1.174	-1.151	2.59E-17
TMEM51	0.095	-0.083	-0.091	-0.134	0.812	2.65E-17
DHRSX	-0.078	0.054	0.037	0.203	0.664	2.67E-17
NOC3L	-0.348	0.08	-0.087	0.019	-0.423	2.67E-17
ANKRD26	-0.389	0.148	0.131	-0.141	-0.31	2.72E-17
CYP1A1	0.244	-0.383	-0.528	-0.712	-1.625	2.74E-17
DHRS1	-0.728	0.015	0.118	-0.12	0.529	2.79E-17
C5orf24	0.017	-0.117	-0.026	0.177	0.432	2.89E-17
ZFP36	-0.229	0.228	-0.301	-0.394	-0.81	2.89E-17
PEAR1	-0.184	0.093	0.474	0.217	0.49	2.92E-17
FCGRT	-0.08	-0.102	-0.042	0.173	0.483	2.96E-17
LOXL2	0.296	0.087	-0.011	0.466	0.927	3.04E-17
C22orf29	-0.072	0.032	0.116	-0.051	-0.399	3.15E-17
AC011325.1	-0.252	0.174	0.982	0.737	1.401	3.23E-17
ZSCAN2	0.125	0.284	0.172	0.568	1.064	3.25E-17
SEC14L2	0.289	0.403	0.448	0.545	0.791	3.28E-17
ZMIZ2	-0.261	0.009	0.276	0.041	0.38	3.31E-17
BCL2A1	-0.106	-0.257	0.089	0.339	1.801	3.35E-17
FLT4	-0.071	-0.217	-0.075	0.065	0.988	3.45E-17
GNB4	0.652	-0.017	-0.184	0.085	-0.025	3.53E-17
ITPRIP	-0.161	-0.045	-0.035	0.052	0.494	3.54E-17
MBD1	-0.14	0.068	-0.023	-0.256	-0.358	3.54E-17
GIMAP4	-0.308	-0.26	-0.033	0.447	0.125	3.83E-17
BCKDHB	-0.138	0.004	0.124	0.377	0.599	3.91E-17
COL27A1	-0.424	0.049	0.426	-0.377	0.581	3.92E-17
DNAJC6	0.476	-0.075	-0.248	-0.349	-0.731	3.93E-17
MID2	-0.282	0.047	0.088	0.294	0.605	3.97E-17
NEK1	0.054	0.218	0.449	0.713	0.45	3.99E-17
GABBR1	-0.403	0.114	0.259	-0.686	-0.713	3.99E-17
JMJD7	-0.434	-0.045	0.063	-0.511	-1.173	4.00E-17
MCM5	0.449	0.034	-0.409	-0.269	-0.797	4.04E-17
PKIA	0.038	0	0.165	0.241	0.626	4.07E-17
CMSS1	-0.098	-0.01	0.011	-0.041	-0.8	4.13E-17
ADAT2	-0.431	-0.029	0.027	-0.636	-0.957	4.29E-17
RPGRIP1L	-0.366	-0.118	0.03	0.227	0.49	4.32E-17
ZFYVE9	0.123	-0.178	0.025	0.358	0.59	4.45E-17
AGPAT3	0.129	0.009	0.125	0.363	0.59	4.50E-17

CKAP2L	-0.569	-0.37	-0.132	0.171	-0.184	4.52E-17
CDK10	-0.595	-0.101	0.02	-0.735	-0.752	4.58E-17
MARK4	-0.003	0.08	0.013	0.096	0.586	4.68E-17
GPATCH11	0.06	0.065	-0.183	-0.131	-0.583	4.85E-17
LEMD3	0.365	0.132	-0.039	-0.094	-0.133	4.93E-17
CCNH	-0.108	-0.048	-0.111	0.008	-0.469	4.96E-17
PLAUR	0.124	-0.07	0.018	0.291	0.634	4.98E-17
TELO2	-0.088	-0.051	-0.24	-0.548	-0.674	4.98E-17
YPEL3	-0.11	0.317	0.373	0.135	0.899	5.03E-17
ZNF862	-0.613	0.208	0.504	-0.202	-0.001	5.17E-17
NECAB3	-0.138	0.008	-0.001	-0.542	-0.65	5.17E-17
TBC1D22A	0.283	-0.018	-0.049	0.458	0.575	5.23E-17
CCDC136	-0.197	0.756	0.909	0.713	1.046	5.29E-17
HEATR5B	-0.411	0.05	0.186	0.013	0.023	5.29E-17
STAT1	0.085	0.001	0.087	0.517	0.725	5.44E-17
CCNI	0.309	0.23	0.204	0.456	0.586	5.47E-17
BCAM	-0.016	0.213	0.262	0.34	0.817	5.88E-17
KIAA1109	-0.404	-0.029	0.248	0.202	0.408	5.92E-17
FBXL3	0.528	0.184	0.018	0.252	0.327	5.97E-17
P4HA3	0.192	0.068	0.028	0.248	0.876	6.07E-17
IQGAP3	-0.915	-0.467	0.089	-0.318	-0.474	6.11E-17
KIAA1524	-0.317	-0.259	-0.411	-0.212	-0.559	6.18E-17
MAF1	0.072	0.007	-0.026	0.239	0.4	6.30E-17
RAP2A	0.334	0.129	0.028	0.279	0.478	6.40E-17
ZDHHC3	0.029	-0.034	-0.058	0.101	0.396	6.65E-17
FKBP14	0.169	-0.005	-0.252	-0.159	-0.369	6.71E-17
THEM6	0.225	-0.298	-0.502	-0.261	-0.647	6.86E-17
CFAP54	-0.074	0.144	0.443	0.275	0.997	7.16E-17
PBX2	-0.527	-0.068	-0.012	-0.378	-0.334	7.25E-17
VHL	0.02	0.144	0.084	0.167	0.482	7.32E-17
ZNF512B	-0.398	-0.076	-0.02	-0.234	-0.435	7.36E-17
RP11-45M22	-0.635	-0.185	-0.01	-1.223	-1.107	7.41E-17
SMYD4	-0.398	-0.136	-0.283	-0.595	-0.681	7.47E-17
KDM6B	-0.446	0.253	0.061	-0.269	0.349	7.60E-17
GPCPD1	0.009	0.007	-0.054	-0.342	-0.742	7.60E-17
FGFRL1	0.496	0.033	-0.338	-0.33	-0.226	7.69E-17
ELP5	-0.236	-0.154	-0.244	-0.245	-0.516	7.80E-17
MEIS2	-0.369	0.009	-0.681	-0.646	-0.808	8.12E-17

AHI1	-0.165	0.061	0.054	-0.364	-0.746	8.40E-17
POLR2G	-0.122	-0.104	-0.034	-0.2	-0.602	8.57E-17
PXDC1	0.349	-0.175	-0.207	-0.05	-0.073	8.65E-17
FAM171A2	0.196	0.083	-0.363	-0.12	0.805	8.67E-17
CBX2	0.183	0.04	-0.419	-0.623	-0.339	8.87E-17
MIR34AHG	0.885	0.856	1.141	-0.114	0.358	8.93E-17
RP13-88F20	0.338	-0.077	-0.056	0.38	0.777	9.04E-17
ZNF7	-0.125	0.205	0.092	-0.505	-0.435	9.04E-17
RALGAPA2	-0.257	-0.141	0.05	0.03	0.59	9.07E-17
ATP6V0E2	-0.222	-0.068	0.118	0.471	0.554	9.30E-17
RP13-138P1	0.078	-0.261	0.103	0.598	1.197	9.33E-17
CWC25	-0.042	0.144	-0.096	-0.202	-0.501	9.42E-17
APBB1	0.126	0.106	0.214	0.454	0.717	9.67E-17
TCTN3	0.133	0.061	0.094	0.433	0.695	9.83E-17
DPY19L1	0.337	-0.041	-0.31	-0.127	-0.278	9.85E-17
TLR3	0.574	0.664	0.71	1.038	1.41	9.96E-17
NOP2	-0.14	0.003	-0.03	-0.136	-0.516	1.02E-16
HIRIP3	0.058	-0.002	-0.032	0.248	-0.794	1.02E-16
MT-ND4	-0.545	-0.378	0.307	-1.169	-0.918	1.02E-16
SAMHD1	0.184	0.117	0.142	0.518	0.858	1.03E-16
RNF103	0.52	0.299	0.015	-0.026	0.399	1.03E-16
CISD2	-0.064	-0.136	-0.161	-0.396	-0.815	1.06E-16
LINC00340	-0.412	0.322	0.06	0.119	1.39	1.08E-16
RBM15B	0.172	-0.023	-0.037	0.32	0.64	1.10E-16
SQRDL	0.222	0.138	0.254	0.682	0.668	1.11E-16
TRIM41	-0.307	-0.033	0.084	-0.384	-0.6	1.11E-16
GNB3	-0.621	-0.345	-0.145	-1.261	-1.067	1.15E-16
GPR162	-0.423	-0.128	0.369	0.256	0.837	1.16E-16
CLASRP	-0.194	-0.026	-0.071	-0.625	-0.685	1.18E-16
WIPI2	0.21	0.209	0.198	0.428	0.726	1.19E-16
ARHGAP18	-0.013	-0.11	-0.41	-0.33	-0.464	1.19E-16
ECT2	-0.298	-0.473	-0.322	-0.087	-0.314	1.21E-16
PLCD3	0.554	0.273	0.265	0.416	0.685	1.22E-16
GSTA4	-0.04	-0.132	-0.219	-0.204	-0.839	1.23E-16
C22orf46	-0.547	0.011	0.469	-0.207	-0.804	1.25E-16
RP11-456J2(	-1.11	-1.759	-1.338	-1.29	-0.819	1.27E-16
STIM2	0.002	-0.225	-0.248	-0.259	-0.663	1.29E-16
TLL1	0.608	0.014	-0.402	-0.464	-0.784	1.32E-16

C16orf45	-0.148	-0.217	-0.219	0.054	0.509	1.33E-16
HNRNPH1	0.018	0.101	0.155	-0.181	-0.347	1.33E-16
MAPKAP1	0.21	0.018	-0.008	0.286	0.426	1.34E-16
PHKA1	0.027	-0.103	-0.156	-0.145	-0.752	1.34E-16
TCEANC2	-0.832	-0.164	0.036	-0.309	-0.429	1.35E-16
UVRAG	-0.029	-0.094	0.07	0.14	0.428	1.36E-16
TYRO3	0.387	0.029	-0.252	-0.095	-0.336	1.37E-16
REPS2	0.396	0.143	0.256	0.706	0.91	1.38E-16
PPP1R16B	-0.176	-0.208	-0.303	-0.398	0.44	1.38E-16
SNHG3	-0.156	-0.126	0.024	-0.88	-1.03	1.38E-16
RP13-331H1	-0.431	-1.026	-1.111	-0.53	-0.034	1.40E-16
IFT172	-0.497	-0.161	0.191	0.098	0.053	1.42E-16
SMEK1	0.374	0.353	0.315	0.167	0.107	1.43E-16
PSMD2	0.402	0.224	0.21	0.641	0.9	1.45E-16
EPS8L1	-0.348	-0.179	-0.085	-0.397	0.582	1.45E-16
FAM212B	1.646	1.532	1.434	1.13	1.716	1.47E-16
HEMK1	-0.543	0.23	0.542	-0.136	-0.314	1.47E-16
THAP7	-0.254	-0.155	-0.316	-0.176	-0.876	1.50E-16
AP1G2	-0.55	0.089	0.472	-0.028	0.05	1.51E-16
ZNF646	-0.077	0.126	-0.062	-0.422	-0.447	1.52E-16
ZNF217	0.331	0.217	0.233	0.329	0.67	1.53E-16
SEPT9	0.207	-0.141	-0.027	0.493	0.652	1.53E-16
DNASE1	-0.697	-0.088	0.306	-0.472	-0.277	1.54E-16
RP5-1042K1	-0.607	0.088	0.14	-0.902	-0.668	1.61E-16
ADAMTS6	-0.637	0.02	-0.235	-0.807	-0.75	1.62E-16
CPA4	-0.055	0.71	0.918	0.955	0.631	1.66E-16
PLEKHM1	-0.169	0.139	0.218	0.414	0.507	1.66E-16
PDCL	0.395	0.074	0.16	0.451	0.661	1.71E-16
PPOX	-0.956	-0.102	0.084	-0.209	-0.217	1.74E-16
DERL2	-0.001	-0.057	-0.09	-0.171	-0.574	1.74E-16
PHF21A	-0.436	-0.002	0.164	-0.015	0.429	1.75E-16
SRRM2	-0.248	0.02	0.144	-0.459	-0.386	1.75E-16
METRNL	0.813	0.055	0.05	0.435	0.329	1.77E-16
CYTH3	0.412	0.101	0.073	0.304	0.586	1.82E-16
KIAA0753	-0.577	0.129	0.149	-0.055	-0.016	1.83E-16
SLC44A4	0.061	0.22	0.313	0.438	1.168	1.88E-16
SEPT6	0.292	-0.038	-0.048	0.301	0.855	1.89E-16
MT-CO3	-0.481	-0.348	0.264	-1.187	-1.035	1.90E-16

DLGAP1	0.156	-0.211	-0.335	0.308	0.798	1.92E-16
RASAL3	-0.108	-0.053	0.186	0.066	0.609	1.93E-16
LAMP1	0.291	0.128	-0.003	0.448	0.708	1.94E-16
FAM122B	-0.323	-0.037	0.05	-0.277	-0.495	1.96E-16
EEF2	0.383	0.255	0.243	0.61	0.923	1.97E-16
KLHL8	0.236	-0.051	-0.218	-0.157	-0.539	2.00E-16
SH3BP5L	-0.052	0.144	0.112	0.268	0.589	2.02E-16
TYMS	0.32	0.44	0.386	0.601	0.305	2.02E-16
RASSF9	1.096	0.352	0.629	0.863	1.016	2.04E-16
GSK3B	0.301	0.177	0.169	0.422	0.762	2.05E-16
DNAJB6	0.175	0.045	0.052	0.324	0.645	2.06E-16
SIPA1L1	0.101	0.101	0.261	0.228	0.629	2.11E-16
TICRR	-0.664	-0.547	-0.584	-0.873	-0.968	2.12E-16
PHF13	0.305	0.16	-0.337	-0.211	-0.092	2.14E-16
PDSS1	0.15	0.009	-0.242	-0.351	-0.773	2.14E-16
GSTT2B	-0.803	-0.03	0.144	-0.564	-0.96	2.14E-16
GOLM1	0.16	0.021	-0.088	0.196	0.602	2.16E-16
STK24	0.457	0.013	-0.1	0.146	0.115	2.16E-16
THAP12	0.154	0.078	-0.137	-0.172	-0.348	2.18E-16
TCF25	0.048	0.089	0.033	-0.231	-0.343	2.19E-16
ZNF37B	-0.681	0.017	0.177	-0.752	-0.568	2.21E-16
RRAGA	0.266	-0.039	-0.008	0.561	0.659	2.23E-16
TRIM32	0.631	0.408	0.337	0.478	0.767	2.33E-16
CTD-2263F2	-0.175	0.185	0.796	0.601	1.705	2.34E-16
CYSTM1	0.044	0.044	0.124	0.218	0.638	2.34E-16
DLEU2	-0.552	-0.445	-0.232	-0.765	-1.357	2.40E-16
RPH3AL	0.059	-0.224	0.317	0.821	0.691	2.41E-16
GTF2I	-0.001	-0.179	-0.03	0.144	0.605	2.43E-16
LANCL1	0.189	0.136	-0.043	0.293	0.544	2.50E-16
RHBDF1	-0.21	0.17	0.228	-0.143	0.461	2.54E-16
RRBP1	0.349	0.069	0.052	0.307	0.694	2.55E-16
YAP1	0.378	-0.111	-0.31	-0.192	-0.381	2.58E-16
TIMM17A	0.173	-0.021	-0.095	-0.075	-0.452	2.68E-16
TRAF4	0.474	0.464	0.41	-0.138	0.512	2.71E-16
TMEM106A	-0.39	0.078	0.16	0.066	-0.598	2.71E-16
PCDHGA4	0.427	0.376	0.062	0.478	1.436	2.72E-16
TMEM33	0.007	0.023	-0.221	-0.148	-0.426	2.72E-16
AHDC1	-0.324	0.211	0.025	-0.301	0.418	2.73E-16

LINC00346	-0.428	0.07	0.442	0.532	0.522	2.82E-16
MFGE8	0.176	0.123	0.069	0.294	0.665	2.87E-16
EFR3A	0.169	0.028	0.003	0.29	0.515	2.88E-16
ACSL4	0.462	0.169	0.143	0.532	0.775	2.90E-16
TOMM34	0.115	-0.063	-0.026	0.308	0.66	2.91E-16
ATP10D	0.094	0.386	0.08	-0.065	-0.051	2.98E-16
MTMR9L	-0.712	-0.217	0.319	-0.375	0.673	3.03E-16
ACYP1	-0.224	-0.199	-0.009	-0.147	-0.812	3.06E-16
MAPK11	-0.242	0.172	0.156	-0.327	-0.329	3.07E-16
C7orf43	-0.375	0.177	-0.13	-0.725	-0.47	3.08E-16
RCBTB1	0.138	0.329	0.302	0.185	0.477	3.20E-16
MMAB	0.114	-0.139	-0.396	-0.536	-0.382	3.22E-16
TXNDC5	0.161	-0.051	-0.265	0.081	-0.56	3.23E-16
TIMM23B	-0.003	0.091	-0.043	-0.635	-0.671	3.23E-16
AACS	0.371	0.115	0.056	0.208	0.676	3.28E-16
DNMT3B	-0.979	-0.722	-0.62	-0.747	-1.208	3.30E-16
ZNF248	-0.819	0.033	-0.058	-0.622	-0.493	3.31E-16
RIOK3	0.502	0.332	0.257	0.214	0.254	3.34E-16
TST	-0.053	-0.034	0.078	0.401	0.679	3.53E-16
FAHD2A	-0.127	0.151	-0.009	-0.031	-0.56	3.55E-16
ATP6AP1	0.138	0.03	-0.051	0.342	0.61	3.57E-16
PPP1R15A	0.428	0.406	0.213	0.222	0.18	3.65E-16
ZNF384	-0.133	0.099	0.055	-0.244	-0.421	3.66E-16
ZBTB4	0.138	0.043	0.195	0.409	0.749	3.76E-16
CYGB	-0.149	-0.006	0.896	0.947	1.658	3.81E-16
BRCA1	0.028	0.14	0.021	0.027	-0.579	3.85E-16
RP11-39C10	0.487	-0.164	-0.289	-0.036	-0.585	3.86E-16
TPD52L2	0.2	-0.03	-0.049	0.219	0.52	4.07E-16
LRRC14	-0.227	0.048	-0.145	-0.4	-0.398	4.12E-16
USP13	0.075	-0.432	-0.565	-0.117	-0.085	4.21E-16
HIBADH	0.252	-0.039	-0.093	0.222	0.451	4.23E-16
CHAC2	-0.483	-0.186	-0.255	-0.527	-0.89	4.33E-16
DARS	0.321	0.137	0.227	0.661	0.647	4.38E-16
PEX6	-0.128	0.065	0.398	0.348	0.477	4.38E-16
YY1	0.337	0.024	-0.047	0.219	0.585	4.39E-16
SGMS1	0.463	-0.096	-0.029	0.05	-0.041	4.53E-16
TOB2	0.525	0.274	-0.029	0.172	0.486	4.60E-16
CDK1	-0.58	-0.475	-0.509	-0.371	-0.709	4.60E-16

RAD51C	0.041	0.182	0.261	0.323	-0.352	4.68E-16
ATXN1	-0.318	-0.41	-0.517	-0.282	0.196	4.72E-16
GBGT1	-0.244	0.126	0.364	0.336	0.384	4.81E-16
PTPRG	0.365	0.033	-0.16	0.032	-0.522	4.99E-16
SH2D5	-0.533	0.085	0.616	0.551	0.815	5.14E-16
CAST	0.347	0.224	0.2	0.517	0.71	5.39E-16
GBAP1	-0.819	0.095	0.43	-0.429	-0.398	5.39E-16
FOCAD	0.328	0.01	0.073	0.379	0.693	5.70E-16
SLC18B1	-0.186	-0.341	-0.164	0.089	0.604	5.76E-16
SUV39H2	-0.209	-0.139	-0.279	-0.425	-0.779	5.77E-16
RP11-904M1	-0.396	0.271	0.079	-1.1	-0.93	5.78E-16
RABGGTB	0.06	0.084	0.085	-0.33	-0.691	6.02E-16
ANP32E	0.151	-0.093	-0.239	0.08	-0.757	6.02E-16
PLXND1	0.401	-0.225	-0.297	-0.166	0.149	6.03E-16
DLG4	-0.57	-0.16	-0.145	-0.619	-0.474	6.13E-16
RP11-221N1	-0.525	0.156	0.553	0.462	0.705	6.15E-16
GJA1	0.313	0.066	-0.101	0.157	0.374	6.25E-16
LDHB	0.303	0.042	-0.033	0.42	0.539	6.38E-16
MT-CO2	-0.447	-0.328	0.264	-1.175	-0.944	6.49E-16
ACOT2	0.096	0.209	0.245	0.47	1.11	6.55E-16
MT-CO1	-0.551	-0.382	0.271	-1.163	-0.901	6.55E-16
TSPAN9	0.231	0.001	-0.135	0.165	0.495	6.56E-16
NRK	0.122	-0.07	0.074	0.364	0.696	6.78E-16
SOCS6	0.178	-0.165	-0.305	-0.057	0.372	7.02E-16
RP11-452L6.	-0.813	0.124	0.175	-0.136	-0.474	7.02E-16
RP11-419C5	-0.243	-0.043	0.004	-1.311	-0.842	7.05E-16
SPRYD7	0.192	0	-0.18	-0.104	-0.454	7.10E-16
CD109	0.278	0.044	-0.087	0.176	0.703	7.11E-16
HIF1A	0.219	0.084	0.114	0.442	0.776	7.15E-16
AC034193.5	-0.086	0.027	-0.161	-0.703	-1.088	7.16E-16
CTD-2571E1	-1.068	-2.063	-1.417	-0.8	-0.816	7.19E-16
ZNF839	-0.732	-0.079	0.149	-0.254	-0.488	7.24E-16
CLIP4	-0.089	0.118	0.359	0.372	0.515	7.35E-16
CASP2	0.422	-0.032	-0.217	-0.069	-0.066	7.37E-16
ZNF362	0.32	-0.178	-0.183	0.056	0.572	7.39E-16
CSAD	-0.459	0.02	0.23	-0.752	-0.724	7.42E-16
GGA1	-0.244	0.016	0.151	-0.088	-0.373	7.49E-16
TUFT1	0.776	0.258	0.014	0.276	0.917	7.63E-16

RAPGEF1	0.49	0.094	-0.043	0.038	0.294	7.70E-16
VPS39	0.069	0.208	0.166	0.219	0.463	7.77E-16
MTHFSD	-0.586	-0.001	0.162	-0.199	-0.274	7.94E-16
ARRDC2	0.437	0.307	0.002	-0.377	-0.122	8.02E-16
PLSCR4	0.058	0.172	-0.043	0.056	-0.525	8.13E-16
PDE10A	0.302	0.005	-0.514	-0.571	-0.099	8.18E-16
RBBP6	0.384	0.226	0.16	0.033	-0.121	8.25E-16
P4HA2	0.282	0.214	0.156	0.614	0.78	8.29E-16
KLHL42	0.245	0.056	0.087	-0.027	0.503	8.42E-16
DIP2A	-0.454	0.043	0.127	-0.207	0.034	8.54E-16
C1orf52	0.008	0.379	0.163	-0.286	-0.336	8.54E-16
ITGB5	0.263	-0.011	-0.07	0.368	0.627	8.76E-16
MT-TQ	-0.189	-0.013	0.297	-1.278	-1.155	8.78E-16
EXT1	0.264	-0.138	-0.34	-0.028	0.319	8.80E-16
IPO7	0.325	0.244	0.189	0.365	0.558	8.83E-16
PHLDA2	0.227	-0.024	-0.254	-0.149	0.584	8.94E-16
UBXN7	0.247	0.308	0.129	0.121	0.54	8.94E-16
FOXA1	0.291	-0.29	-0.461	-0.436	1.001	9.02E-16
HHIPL1	0.219	0.146	0.492	0.708	1.269	9.20E-16
GIMAP7	-0.414	-0.168	-0.436	-0.073	-0.777	9.28E-16
PDGFA	0.523	0.332	0.554	0.584	0.337	9.45E-16
IGF2BP3	-0.227	-0.316	-0.383	-0.42	-0.306	9.45E-16
TMEM209	0.227	0.11	-0.063	-0.106	-0.507	9.65E-16
LSS	0.232	-0.166	-0.362	-0.461	-0.166	9.71E-16
MAP3K5	0.412	-0.123	-0.204	0.073	0.009	9.77E-16
LRRC32	0.606	-0.123	-0.049	0.281	-0.065	9.77E-16
NLGN1	0.291	0.011	0.241	0.604	0.923	9.82E-16
RP11-117P2	-0.479	0.025	0.442	-0.021	0.463	1.00E-15
ZNF702P	-0.368	-0.029	0.44	0.319	0.398	1.01E-15
DERL3	-0.194	-0.573	-0.567	-0.977	-1.947	1.03E-15
PMS1	-0.466	-0.011	0.125	-0.087	-0.468	1.07E-15
MGST1	0.042	-0.01	0.009	-0.068	-0.97	1.07E-15
CD200	-0.108	-0.143	-0.163	0.183	1.263	1.09E-15
LAMA3	-0.028	0.448	0.646	0.53	0.999	1.11E-15
EPOR	-0.313	-0.217	0.102	0.318	0.596	1.11E-15
DDR2	-0.259	-0.089	0.141	0.004	0.877	1.17E-15
MT-RNR2	-0.797	-0.373	0.247	-1.301	-0.714	1.20E-15
UBE2G1	0.207	-0.024	-0.216	0.074	0.442	1.22E-15

ARSJ	-0.368	-0.208	-0.246	-0.044	0.163	1.24E-15
UCP2	-0.179	0.376	0.55	1.131	0.682	1.26E-15
ICE2	-0.281	0.016	0.076	-0.06	-0.456	1.26E-15
CTD-2574D2	-0.576	0.073	0.159	-1.158	-0.726	1.26E-15
RP5-857K21	-0.513	-0.441	0.203	-1.346	-1.169	1.26E-15
PSEN1	0.145	0.3	0.179	0.311	0.51	1.33E-15
INPP5K	0.3	-0.012	-0.187	-0.047	-0.447	1.33E-15
MUTYH	-0.48	-0.075	-0.042	-0.444	-0.869	1.35E-15
ZNF521	0.344	0.045	0.176	0.625	0.794	1.36E-15
LARP1B	0.299	0.22	0.027	-0.081	-0.337	1.36E-15
PHC3	-0.036	0.197	0.093	-0.247	-0.333	1.38E-15
STAT6	-0.06	0.033	0.198	0.225	0.401	1.41E-15
SS18L1	0.205	0.198	0.062	-0.633	-0.63	1.41E-15
USP21	-0.544	-0.154	-0.032	-0.35	-0.478	1.42E-15
RPA2	0.199	0.025	-0.217	-0.114	-0.448	1.45E-15
FAM134C	-0.046	0.251	0.23	0.181	0.534	1.46E-15
ST3GAL4-AS	-0.839	-0.363	0.355	0.032	0.478	1.46E-15
NPHP4	-0.039	-0.211	-0.137	-0.419	-0.69	1.46E-15
KANK2	0.211	0.11	0.441	0.102	0.279	1.47E-15
POP7	-0.007	-0.225	-0.196	-0.158	-0.581	1.48E-15
SHROOM1	-0.781	-0.172	-0.02	-0.525	-0.165	1.50E-15
ZNF274	0.007	0.007	0.418	0.337	0.409	1.53E-15
ARHGAP4	-0.157	-0.424	-0.132	-0.403	0.331	1.53E-15
MAPK8IP3	-0.647	-0.067	-0.058	-1.02	-0.61	1.54E-15
NOTCH2	0.069	0.064	0.04	0.196	0.655	1.56E-15
DOK3	-0.435	-0.039	-0.273	-1.384	-0.82	1.58E-15
DHCR7	0.347	-0.147	-0.56	-0.407	-0.191	1.61E-15
ARL13B	0.203	0.117	-0.403	-0.353	-0.509	1.62E-15
RECQL5	-0.614	0.057	0.271	-0.205	-0.229	1.65E-15
DDX1	0.389	0.118	0.099	0.532	0.694	1.68E-15
GOLGA1	-0.329	-0.086	0.098	-0.219	-0.586	1.69E-15
TCAF1	0.362	-0.096	-0.247	-0.322	-0.295	1.74E-15
STX1A	-0.077	-0.353	-0.145	-0.539	-0.85	1.78E-15
RP11-417L14	-0.204	-0.847	-0.671	-0.097	0.152	1.79E-15
CRIP1	-0.062	-0.175	-0.216	-0.32	-1.095	1.80E-15
VGLL3	0.641	0.136	0.227	0.537	0.306	1.83E-15
GOLPH3	0.45	0.103	-0.078	0.135	0.2	1.83E-15
PIDD1	-0.072	0.509	0.455	-0.274	-0.081	1.89E-15

ISG20	0.137	-0.042	0.156	0.458	1.412	1.94E-15
SLC43A2	0.28	-0.274	-0.319	0.074	0.446	1.96E-15
TRIM59	-0.771	-0.695	-0.419	-0.285	-0.429	2.01E-15
BMPR2	0.554	0.221	-0.314	-0.184	-0.365	2.04E-15
PLPP1	0.27	0.177	-0.091	-0.045	-0.621	2.07E-15
C5orf42	-0.438	0.226	0.16	-0.28	-0.316	2.16E-15
PLEKHM1P	-0.175	0.141	0.081	-0.923	-0.699	2.16E-15
FGF12	-0.173	-0.248	-0.94	-0.944	-1.232	2.18E-15
SLC26A2	0.344	-0.139	-0.146	0.076	-0.234	2.23E-15
GEN1	-0.275	-0.186	-0.19	-0.535	-0.67	2.26E-15
TOP2B	0.297	0.14	0.116	0.541	0.821	2.33E-15
CDR2L	0.312	0.097	0.022	0.164	0.806	2.33E-15
HDGF	0.149	0.025	-0.011	0.311	0.562	2.33E-15
RP11-371A2	0.127	0.063	0.077	0.207	0.351	2.33E-15
SUGT1P	0.286	0.007	0.05	-0.275	-1.128	2.33E-15
FBXW2	0.049	0.128	0.204	0.2	0.391	2.39E-15
RP11-802E1	0.063	0.385	0.387	-0.833	-0.256	2.42E-15
MAP7D1	0.191	-0.011	-0.05	0.223	0.575	2.44E-15
SIK2	0.142	0.123	-0.067	0.053	0.575	2.44E-15
BAMBI	0.659	-0.199	-0.411	-0.047	-0.51	2.44E-15
FAM60A	0.125	-0.025	0.013	0.215	0.577	2.47E-15
ARIH1	0.209	0.264	0.007	0.147	0.523	2.48E-15
PNKP	-0.363	-0.081	0.003	-0.523	-0.66	2.54E-15
FAM118A	-0.408	-0.034	0.11	-0.713	-0.544	2.55E-15
PAK4	0.098	-0.258	-0.202	0.278	0.582	2.60E-15
FGD5	0.085	-0.224	-0.011	0.291	0.495	2.62E-15
CRAT	0.059	0.12	0.148	0.434	0.466	2.68E-15
STAT3	0.427	0.339	0.334	0.604	0.836	2.69E-15
SUGP2	-0.468	-0.077	-0.041	-0.812	-0.645	2.74E-15
USP46	0.384	0.354	0.231	0.193	0.644	2.82E-15
GPR153	-0.303	-0.061	0.046	-0.111	0.732	2.88E-15
MBTPS1	0.146	0.039	0.016	0.229	0.41	2.88E-15
ABI2	-0.126	-0.153	0.083	0.079	0.345	3.00E-15
KIAA1324	0.237	0.417	0.678	0.982	0.825	3.02E-15
ABCA7	-1.134	-0.403	0.316	-0.201	-0.054	3.17E-15
RFX3	-0.473	0.116	0.334	0.285	0.745	3.18E-15
ERP29	0.2	0.096	0.057	0.436	0.541	3.29E-15
ARID1B	0.061	-0.065	-0.197	-0.006	0.456	3.31E-15

FAM43A	-0.434	0.004	0.094	0.165	0.346	3.33E-15
LTB4R2	-0.681	0.097	0.223	-0.792	-0.671	3.33E-15
HERPUD2	0.4	0.231	0.065	0.302	0.501	3.34E-15
TIMM8A	-0.163	-0.042	0.022	-0.305	-0.769	3.38E-15
GPT2	-0.186	-0.076	-0.017	0.311	0.486	3.48E-15
POSTN	0.212	0.188	-0.047	0.307	-1.011	3.50E-15
ZHX1	0.3	0.115	-0.094	0.098	-0.367	3.51E-15
MIS18BP1	-0.34	-0.446	-0.342	-0.109	-0.128	3.58E-15
GSDMC	-0.129	-0.234	0.263	0.317	0.885	3.62E-15
NPIPB4	-0.56	-0.404	-0.051	-1.197	-0.896	3.62E-15
ANKS1A	-0.8	-0.465	-0.047	-0.021	-0.145	3.64E-15
PPP1R18	-0.102	0.024	0.115	0.235	0.395	3.71E-15
AKAP17A	-0.261	0.157	0.073	-0.614	-0.509	3.73E-15
IL17D	-0.038	0.052	0.069	0.136	0.879	3.78E-15
C11orf49	0.145	-0.021	-0.106	0.279	0.578	3.79E-15
ZNF664	0.287	0.251	0.136	0.223	0.513	3.93E-15
WDR66	0.398	0.195	0.617	0.66	1.361	4.00E-15
MR1	0.075	0.36	0.422	0.722	0.663	4.00E-15
SGO1	-0.674	-0.504	-0.4	-0.065	-0.708	4.09E-15
CLSTN1	0.204	0.095	-0.009	0.42	0.749	4.10E-15
TMEM198B	-0.593	0.328	0.549	-0.189	0.203	4.10E-15
PTGFRN	0.403	0.121	0.13	0.417	0.837	4.11E-15
GFOD2	-0.173	-0.125	-0.115	0.039	0.363	4.11E-15
CD83	0.441	1.738	0.952	-0.086	0.831	4.14E-15
NOS3	-0.045	-0.427	-0.23	-0.04	-0.627	4.17E-15
SNORD30	-0.335	-0.188	-0.34	-1.272	-1.659	4.25E-15
SLC36A1	-0.099	0.09	0.001	-0.084	-0.533	4.27E-15
MT-ND1	-0.49	-0.347	0.34	-1.158	-1.04	4.28E-15
GNPTAB	0.374	0.081	-0.041	0.152	0.364	4.30E-15
DHRS13	-0.661	-0.393	-0.189	-0.089	-1.123	4.33E-15
PEBP1P2	0.204	-0.3	-0.154	0.327	0.643	4.44E-15
QARS	0.125	0.127	0.128	0.268	0.495	4.47E-15
SENP6	0.189	0.342	0.228	0.286	0.493	4.54E-15
TCN2	0.204	0.018	0.066	0.525	0.687	4.58E-15
PPP1R13L	0.108	-0.064	0.372	0.508	0.551	4.62E-15
CLEC11A	-0.513	-0.361	-0.101	-0.266	0.438	4.64E-15
CYB5R1	0.079	0.182	0.094	0.165	-0.381	4.65E-15
CROCCP2	-0.404	0.127	0.115	-0.739	-0.663	4.65E-15

LIG3	0.217	-0.067	0.017	0.173	-0.473	4.66E-15
RP11-473M2	-0.852	0.2	0.52	-0.262	0.065	4.67E-15
APP	0.343	0.124	-0.006	0.451	0.804	4.68E-15
TMED1	0.102	0.045	-0.089	0.224	-0.432	4.71E-15
RP11-384K6	-0.122	0.244	0.203	-0.792	-0.857	4.74E-15
PTHLH	0.532	-0.31	-0.598	-0.591	-0.368	4.75E-15
NIF3L1	-0.238	-0.397	-0.112	0.075	-0.718	4.79E-15
KIF20B	-0.452	-0.584	-0.621	-0.243	-0.209	4.81E-15
RBM14	0.334	0.189	-0.38	-0.621	-0.225	4.86E-15
LIF	1.374	1.709	1.827	0.972	2.039	4.91E-15
HS1BP3	-0.098	0.206	0.242	0.421	0.513	4.95E-15
SMAD5	0.288	0.219	0.227	0.261	0.527	5.03E-15
ZMYM3	-0.464	-0.215	0.016	0.054	-0.127	5.18E-15
TRNP1	0.435	-0.003	-0.241	-0.221	-0.18	5.20E-15
TMBIM6	0.238	0.032	-0.038	0.299	0.48	5.24E-15
FLNC	0.377	0.293	0.364	0.49	0.893	5.26E-15
RP11-152N1	-0.682	-0.261	-0.367	-1.027	-1.291	5.26E-15
RP11-345J4.	-0.25	-0.125	-0.183	-0.862	-0.65	5.56E-15
PIGW	0.191	0.024	-0.235	-0.263	-0.333	5.69E-15
H1FX	-0.062	-0.572	-0.552	-0.105	-0.347	5.85E-15
PPP1CB	0.227	0.117	0.125	0.36	0.44	5.95E-15
RF00099	-0.076	-0.208	-0.158	-1.062	-1.465	5.97E-15
SDR39U1	-0.398	0.062	0.063	-0.324	-0.577	6.00E-15
EMC3	-0.089	-0.101	-0.101	-0.111	-0.491	6.01E-15
SNORA8	-0.416	0.064	0.11	-0.945	-0.927	6.09E-15
IRAK1	0.071	-0.024	-0.016	0.101	0.413	6.13E-15
NEK10	-0.215	0.309	1.004	0.306	1.473	6.14E-15
FBXO43	-1.212	-0.722	-0.287	-0.309	-1.363	6.42E-15
CTD-2258A2	0.468	-0.176	0.169	0.134	1.423	6.67E-15
PIGZ	-0.52	-0.047	-0.15	-0.266	0.733	6.68E-15
MT1X	0.107	-0.03	-0.177	-0.171	-0.807	7.00E-15
QKI	0.371	0.043	-0.218	-0.082	0.165	7.33E-15
SP3	0.447	0.114	0.017	0.148	0.051	7.40E-15
HID1	-0.426	-0.089	0.197	0.256	0.501	7.50E-15
FUOM	-0.079	-0.163	-0.213	-0.299	-0.755	7.61E-15
USP18	0.083	-0.18	-0.142	0.049	-0.493	7.77E-15
TPGS1	0.058	0.261	0.142	0.46	1.2	7.79E-15
TTF2	-0.203	-0.086	-0.08	-0.152	-0.574	7.96E-15

ZFPM2	-0.37	0.2	-0.385	-0.538	-0.385	8.00E-15
ABHD8	-0.323	0.208	0.182	0.567	0.929	8.03E-15
RP11-10K16	-0.811	-0.033	0.207	0.252	0.995	8.16E-15
MARK3	-0.077	0.079	0.163	0.166	0.412	8.17E-15
PPP1R35	-0.972	-0.467	-0.193	-0.313	-0.067	8.18E-15
AAMDC	0.156	0.022	0.138	0.36	0.648	8.30E-15
ESCO2	0.04	0.241	-0.114	-0.044	-0.504	8.68E-15
WDR11	0.146	0.175	0.219	0.255	0.459	8.71E-15
TOP3B	-0.485	0.078	0.069	-0.327	-0.447	8.76E-15
HNRNPH3	0.164	0.085	0.174	0.354	-0.286	8.90E-15
CTNS	-0.266	0.116	-0.099	-0.145	-0.491	8.90E-15
FAM108C1	0.386	-0.086	-0.025	0.198	0.716	8.94E-15
ALDH9A1	0.334	0.084	0.003	0.398	0.575	9.00E-15
NFIX	0.102	-0.223	-0.014	0.436	0.798	9.13E-15
ANKS6	-0.033	-0.015	0.293	0.097	0.49	9.28E-15
DCLRE1B	0.339	0.018	-0.151	-0.032	-0.512	9.35E-15
STK35	0.437	0.024	-0.081	-0.061	0.031	9.40E-15
SLMAP	-0.045	0.132	0.06	0.207	0.488	9.43E-15
PANK2	0.206	0.049	0.092	0.069	0.463	9.54E-15
FBXL6	-0.597	0.193	0.041	-0.784	-0.606	9.91E-15
TMOD3	0.287	0.126	0.052	0.415	0.658	9.97E-15
GPATCH2L	-0.119	0.438	0.265	-0.14	0.104	1.02E-14
ABCC10	-0.32	0.02	0.102	-0.329	-0.381	1.02E-14
LBR	-0.384	-0.475	-0.464	-0.268	-0.339	1.03E-14
PARP3	-0.116	0.155	0.246	0.119	0.546	1.06E-14
FAM109A	0.655	0.103	0.094	0.127	0.419	1.06E-14
LIMD2	0.162	-0.071	-0.113	-0.281	0.373	1.06E-14
FKBP11	0	-0.094	-0.13	-0.041	-0.411	1.06E-14
PLS3	0.535	0.219	0.078	0.494	0.857	1.07E-14
KPNA4	0.258	0.034	-0.076	0.237	0.474	1.07E-14
BRI3BP	0.002	-0.044	-0.262	-0.281	-0.567	1.07E-14
ZNF596	-0.01	0.483	0.535	-0.15	-0.769	1.07E-14
NOL4L	-0.186	-0.422	0.097	0.136	0.227	1.11E-14
FAM127C	0.361	0.039	0.03	0.446	0.688	1.12E-14
UBAP1	0.148	0.109	0.035	0.262	0.477	1.14E-14
RAP1A	0.362	0.025	-0.16	-0.077	-0.114	1.15E-14
PMM1	-0.376	-0.147	-0.009	-0.521	-0.673	1.15E-14
PNMAL1	0.032	-0.302	0.194	0.708	0.551	1.16E-14

HMG20B	-0.339	-0.262	-0.263	-0.304	0.188	1.16E-14
SEMA3F	0.025	-0.164	-0.02	0.252	0.421	1.18E-14
VASN	0.864	-0.072	-0.467	-0.708	-0.894	1.23E-14
ANO6	0.256	0.13	-0.022	0.12	0.568	1.39E-14
DSCC1	0.001	-0.059	-0.084	-0.163	-1.186	1.43E-14
LARS	0.231	0.164	0.202	0.521	0.562	1.48E-14
CREB1	0.446	0.446	0.321	0.186	0.386	1.48E-14
SMARCE1	0.193	0.02	-0.067	0.111	0.451	1.53E-14
PLA2R1	-0.099	0.033	0.273	0.049	0.461	1.54E-14
CMC2	-0.315	-0.307	-0.234	-0.578	-1.172	1.54E-14
SLC25A25	-0.21	-0.102	-0.267	-0.372	-0.726	1.57E-14
SSH1	0.031	0.396	0.331	0.018	0.34	1.58E-14
IRF2	0.086	0.607	0.518	0.548	0.936	1.59E-14
CBLB	-0.315	-0.097	-0.519	-0.451	-0.691	1.62E-14
RP11-392P7	-0.271	-0.289	-0.205	-0.078	-0.871	1.62E-14
CD55	0.4	0.194	-0.067	0.168	0.509	1.63E-14
TAF7	0.07	0	0.046	0.504	0.465	1.63E-14
TNK2	-0.725	-0.497	-0.092	-0.532	-0.172	1.64E-14
ZNF473	-0.566	-0.103	-0.437	-0.574	-0.713	1.66E-14
TMCO3	0.153	-0.02	-0.085	0.152	0.353	1.67E-14
ZNF343	-0.12	0.298	0.074	-0.377	-0.417	1.68E-14
TLDC1	-0.052	0.042	0.209	0.48	0.573	1.73E-14
PTPN4	0.051	-0.223	-0.27	-0.212	0.406	1.74E-14
PLSCR1	0.079	-0.05	-0.242	-0.196	-0.453	1.75E-14
FGD6	0.339	0.307	0.163	0.261	0.659	1.79E-14
N6AMT1	-0.468	-0.105	0.015	-0.386	-0.719	1.79E-14
COL8A1	0.283	0.009	-0.186	0.216	0.673	1.81E-14
PIGK	0.094	0.061	0.064	0.474	0.535	1.85E-14
PPP2R5A	-0.012	-0.011	-0.012	0.229	0.492	1.92E-14
PGM3	0.363	0.058	0.068	0.211	0.368	1.92E-14
CLSTN3	-0.075	0.241	0.375	0.336	0.422	1.94E-14
SH2B3	0.22	-0.144	0.048	0.238	0.447	1.97E-14
MYO1D	0.414	0.12	-0.223	0.106	0.833	2.00E-14
RCCD1	-0.697	-0.533	-0.106	-0.324	0.045	2.00E-14
SCDP1	0.322	-0.482	-0.778	-0.63	-0.092	2.00E-14
CYCS	0.025	0.001	-0.01	-0.273	-0.694	2.03E-14
WASHC5	0.221	0.017	0.016	0.322	0.478	2.12E-14
MLH3	-0.351	-0.131	0.242	0.192	0.283	2.13E-14

PHC2	0.119	-0.039	-0.059	0.173	0.485	2.15E-14
SREK1	-0.052	0.108	0.104	-0.431	-0.462	2.15E-14
FAM45B	0.091	-0.083	-0.116	0.108	0.624	2.18E-14
BRPF3	0.418	0.099	-0.119	-0.124	-0.019	2.18E-14
MAP2	0.133	0.017	-0.416	-0.429	-1.004	2.25E-14
CTB-36O1.5	-0.635	-0.584	0.018	-1.401	-1.391	2.26E-14
NPFFR2	-0.08	-0.19	-0.338	0.422	0.809	2.33E-14
CTC-539A10	-0.282	-0.272	-0.215	-0.121	-0.875	2.39E-14
SAMD4A	0.501	0.792	0.856	0.254	0.952	2.40E-14
CTSC	0.117	-0.129	-0.303	-0.041	-0.357	2.40E-14
SLC1A5	0.271	-0.11	-0.375	-0.173	-0.635	2.40E-14
GTPBP2	-0.588	-0.06	0.055	-0.395	-0.277	2.43E-14
RP11-89A12	0.213	-0.074	-0.19	-0.07	-0.518	2.44E-14
LCAT	0.291	0.25	0.336	-0.768	-0.274	2.47E-14
PLIN3	0.08	-0.238	-0.251	0.243	0.565	2.50E-14
AGAP6	-0.469	0.048	0.025	-0.941	-0.847	2.50E-14
FOXK1	0.093	0.104	0.351	0.326	0.461	2.51E-14
MRM1	-0.888	-0.045	-0.319	-0.663	-0.725	2.51E-14
ILK	0.089	-0.037	0.029	0.297	0.417	2.64E-14
RP11-506B1	-1.767	-0.527	0.253	-0.688	-0.422	2.68E-14
GNS	0.232	0.077	-0.033	0.352	0.653	2.74E-14
NCAPD3	0.011	-0.066	-0.251	-0.097	-0.552	2.74E-14
NUDT3	0.285	-0.081	-0.128	0.086	0.368	2.80E-14
ZMYM1	-0.672	-0.225	-0.169	-0.174	-0.308	2.81E-14
CENPK	-0.386	0.001	0.024	-0.225	-0.669	2.82E-14
FRY	-0.451	-0.28	-0.328	-0.228	-0.603	2.83E-14
SPC24	-0.48	-0.35	-0.103	0.079	-0.668	2.95E-14
FAM13A	-0.566	-0.006	-0.055	-0.297	-0.695	2.98E-14
NRROS	0.095	-0.349	0.161	0.687	0.984	3.13E-14
NPM3	-0.08	-0.176	-0.26	-0.421	-0.573	3.13E-14
CLU	0.282	0.048	-0.088	0.378	0.711	3.36E-14
DYNC1I2	0.354	0.162	0.094	0.577	0.653	3.37E-14
RP11-632K2	-0.57	0.244	0.281	-0.565	-0.414	3.38E-14
KLF11	0.812	0.14	0.16	0.503	0.632	3.48E-14
SPG11	-0.013	0.019	0.254	0.482	0.2	3.48E-14
SIRT7	-0.512	-0.045	-0.141	-0.621	-0.596	3.48E-14
CEP170B	0.091	0.331	0.24	-0.203	-0.064	3.53E-14
KLHL17	-0.884	-0.37	-0.226	-1.043	-0.758	3.54E-14

NFE2L2	0.64	0.239	0.035	0.214	0.072	3.57E-14
CAPRIN2	0.161	-0.25	0.04	-0.661	-0.707	3.57E-14
RP11-567G2	0.388	-0.042	0.113	0.594	0.795	3.62E-14
CAPN7	-0.042	0.183	0.141	0.119	-0.324	3.62E-14
ACVR1	0.296	-0.19	-0.323	-0.043	0.052	3.63E-14
ARFGAP1	-0.094	0.212	0.077	-0.349	-0.308	3.63E-14
ANKMY1	-0.205	-0.223	-0.097	-0.472	-0.875	3.72E-14
FBP1	-0.234	-0.209	-0.454	0.155	1.062	3.97E-14
EXT2	0.216	0.07	-0.052	0.342	0.5	3.98E-14
ABC12-4924	-0.653	0.27	0.402	-0.531	-0.541	3.99E-14
RP11-343L1	0.373	-0.084	0.084	0.52	0.814	4.00E-14
MKNK2	-0.166	0.013	0.04	0.066	0.547	4.01E-14
RP11-379H1	-0.214	-0.171	0.237	0.515	0.537	4.10E-14
PRKCH	0.365	-0.152	0.09	0.424	0.517	4.28E-14
MRTO4	0.193	-0.08	-0.244	-0.02	-0.368	4.29E-14
CHST7	-0.218	-0.179	-0.124	0.106	0.796	4.33E-14
DDX55	-0.234	-0.059	-0.078	-0.477	-0.653	4.39E-14
AGBL5	-0.003	0.123	0.113	0.426	0.532	4.44E-14
FRRS1	0.126	-0.047	-0.255	-0.551	-1.267	4.48E-14
RP11-274B2	-0.208	0.169	-0.031	-0.965	-0.671	4.56E-14
TCTEX1D1	-0.619	0.195	0.362	-0.246	0.133	4.65E-14
PPTC7	0.161	-0.01	-0.335	-0.26	-0.163	4.74E-14
SFXN2	-0.502	-0.089	0.05	-0.238	-0.639	4.74E-14
KANSL2	0.159	-0.029	-0.215	-0.36	-0.441	4.98E-14
TCEA3	-0.207	0.577	0.906	1.035	1.63	5.01E-14
OXA1L	-0.087	-0.011	0.107	0.164	0.355	5.01E-14
NQO2	-0.151	-0.091	-0.051	-0.207	-0.73	5.01E-14
METTL4	-0.426	-0.028	0.049	-0.058	-0.474	5.08E-14
TSPAN2	-0.137	0.109	0.02	-0.105	-1.071	5.14E-14
GYPE	0.887	0.007	-0.036	-0.15	-0.333	5.19E-14
RP1-45P21.3	-0.079	0.31	0.351	-0.57	-0.532	5.20E-14
KCNJ12	0.348	-0.416	-0.114	0.32	0.906	5.21E-14
GPR3	-0.58	-0.411	-0.73	-1.15	-1.06	5.22E-14
ESM1	0.076	0.45	0.043	0.076	-0.287	5.30E-14
GSN	0.352	0.269	0.273	0.788	0.807	5.32E-14
GRN	0.249	0.083	-0.027	0.442	0.807	5.36E-14
C1orf174	-0.293	-0.313	-0.273	-0.547	-0.65	5.46E-14
CBX1	0.288	-0.016	-0.109	0.218	0.522	5.49E-14

CHCHD8	-0.14	-0.17	-0.113	-0.262	-0.589	5.62E-14
INTS10	0.043	-0.02	-0.021	0.07	-0.408	5.68E-14
MT-ND4L	-0.524	-0.415	0.284	-1.23	-0.947	5.72E-14
CLEC1B	-0.456	-0.306	-0.594	-0.929	-2.082	5.73E-14
AC124789.1	-0.407	-0.038	0.158	0.092	-0.715	5.76E-14
AUNIP	0.238	-0.288	-0.612	-0.425	-0.808	5.77E-14
CCDC74B	0.067	-0.165	-0.509	-0.547	-1.476	5.85E-14
NRM	0.024	-0.074	-0.07	0.114	-0.566	5.87E-14
IRF3	-0.271	-0.021	0.031	-0.194	-0.597	5.89E-14
TSPAN8	-0.146	0.128	-0.101	0.065	-1.36	5.89E-14
SPTLC2	0.092	-0.19	-0.037	0.118	0.344	5.90E-14
SPATA20	-0.317	-0.076	0.074	-0.192	0.361	6.09E-14
ZNF37A	0.049	0.44	0.433	0.135	0.398	6.14E-14
RP11-320A1	-0.298	-0.495	-0.505	-0.91	-1.11	6.14E-14
PFAS	-0.079	-0.039	-0.133	-0.165	-0.594	6.52E-14
HYPK	0.063	-0.069	-0.08	-0.118	-0.461	6.56E-14
UTP15	-0.061	-0.053	-0.324	-0.279	-0.625	6.58E-14
FSTL5	-0.254	-0.067	0.317	0.226	0.673	6.62E-14
STRAP	0.289	0.199	0.003	0.17	-0.337	6.68E-14
ASF1A	-0.036	-0.228	-0.439	-0.298	-0.615	6.74E-14
FRAT2	-0.426	0.035	-0.434	0.009	0.479	6.82E-14
KIAA1370	0.335	0.363	0.294	0.206	0.587	6.95E-14
MT-ND5	-0.507	-0.364	0.361	-1.062	-0.65	6.98E-14
GEMIN2	-0.604	-0.26	-0.174	-0.561	-0.966	7.17E-14
CCDC15	-0.48	0.221	0.273	0.352	-0.126	7.25E-14
ATP6V0A2	-0.049	0.049	-0.102	-0.288	-0.347	7.26E-14
AC009336.1	-1.103	-0.459	-0.059	-1.132	-1.203	7.26E-14
GGCX	0.008	0.072	0.055	0.203	0.386	7.30E-14
SUPT7L	-0.093	0.317	0.33	-0.089	-0.249	7.31E-14
C1QTNF6	-0.153	-0.422	-0.13	0.214	-0.36	7.31E-14
ASAH1	-0.032	-0.018	-0.063	0.154	0.493	7.33E-14
CFAP20	0.062	-0.124	-0.314	-0.193	-0.433	7.48E-14
CDCA4	0.003	-0.41	-0.426	-0.184	-0.426	7.49E-14
RBM3	0.026	-0.063	-0.094	-0.247	-0.481	7.62E-14
CD2AP	0.231	0.208	0.085	0.382	0.627	8.05E-14
CPTP	-0.388	-0.076	0.065	0.284	0.526	8.11E-14
ZNF366	-1.302	-0.708	-0.363	0.03	0.161	8.18E-14
SNORD22	-0.057	0.226	-0.06	-0.954	-1.317	8.22E-14

CNOT6L	0.153	0.229	-0.161	-0.213	-0.373	8.47E-14
THBS3	-0.528	-0.052	0.247	-0.183	0.266	8.62E-14
SPRY4	-0.195	-0.018	0.247	-0.045	0.513	8.79E-14
STARD8	0.344	-0.399	-0.079	-0.372	-0.033	8.83E-14
CCDC149	0.259	0.062	0.181	0.384	0.985	9.08E-14
MAP4K3	0.344	0.176	0.071	0.167	0.484	9.20E-14
SNORD83B	-0.3	0.194	0.01	-1.038	-1.164	9.24E-14
ENAH	0.399	-0.081	-0.255	-0.093	-0.023	9.29E-14
EIF2S3L	0.248	0.27	0.327	0.409	0.546	9.36E-14
STAG3L5P	-0.797	-0.073	0.209	-0.877	-0.646	9.41E-14
SLC1A4	0.263	0.024	0.002	0.243	-0.429	9.53E-14
MAP1A	0.034	0.291	0.223	0.334	-1.013	9.54E-14
TRIM38	-0.038	0.269	0.404	0.248	0.448	9.64E-14
ZNF333	-0.296	0.071	-0.085	-0.719	-0.819	9.69E-14
PVT1	-0.344	0.433	0.658	0.083	0.413	9.71E-14
ABL2	0.595	0.101	0.307	0.317	0.485	9.75E-14
MT-CYB	-0.487	-0.373	0.315	-1.132	-0.911	9.77E-14
CAP2	0.303	-0.035	0.087	0.474	0.804	9.79E-14
RRP8	-0.493	-0.189	-0.118	-0.32	-0.541	1.02E-13
GPATCH4	0.207	0.1	0.102	0.113	-0.33	1.04E-13
PPFIA1	-0.376	0.062	-0.063	-0.579	-0.517	1.06E-13
ATP6V0A1	0.007	0.01	0.261	0.429	0.327	1.08E-13
ZNF700	-0.352	0.08	-0.162	-0.676	-0.639	1.09E-13
ATP11C	0.11	0.023	-0.077	0.009	0.418	1.11E-13
MGEA5	0.308	0.213	0.256	0.045	0.443	1.14E-13
ERMARD	-0.204	0.038	0.132	0.075	-0.533	1.15E-13
TEK	0.286	-0.084	-0.407	-0.04	0.191	1.17E-13
SLF2	-0.123	0.045	0.097	-0.119	-0.356	1.17E-13
SUOX	-0.419	-0.002	-0.022	0.118	-0.222	1.18E-13
TNIK	0.177	-0.197	-0.305	-0.103	-0.561	1.18E-13
ACACA	0.168	-0.182	-0.285	-0.408	0.059	1.19E-13
TEF	0.197	0.085	-0.402	-0.952	-0.021	1.19E-13
SPSB2	-1.018	-0.281	-0.451	-0.694	-0.95	1.21E-13
PLXNA3	-0.268	0.019	0.112	-0.563	-0.381	1.23E-13
TINF2	0.048	0.076	0.186	0.409	0.164	1.25E-13
TAOK2	-0.256	-0.014	-0.06	-0.326	-0.398	1.28E-13
DCHS1	0.181	-0.073	-0.174	-0.026	0.503	1.33E-13
RP4-751H13	-1.167	-0.276	-0.231	-0.796	-0.973	1.37E-13

SH3D21	-0.219	0.207	0.22	-0.54	-0.663	1.38E-13
ALMS1	-0.047	0.091	-0.067	-0.251	-0.734	1.39E-13
CPQ	0.111	0.039	-0.08	0.29	0.601	1.40E-13
PAFAH2	-0.57	-0.188	0.047	0.289	-0.067	1.40E-13
DCUN1D5	0.048	-0.038	-0.125	0.064	-0.362	1.42E-13
RP11-1024P	-0.519	-0.148	0.15	-0.229	0.61	1.45E-13
SLC12A4	0.184	0.329	0.298	-0.132	0.017	1.45E-13
LRRC8B	0.104	0.202	-0.353	-0.459	-0.1	1.46E-13
KCTD7	-0.237	-0.011	0.039	-0.402	-0.619	1.48E-13
TTC12	0.205	0.318	0.65	0.948	1.095	1.49E-13
ZNF324B	-0.031	0.238	-0.385	-0.44	-0.737	1.50E-13
LMBR1	0.081	0.095	0.084	0.239	0.452	1.52E-13
MIB2	-0.286	0.114	0.416	0.039	0.605	1.55E-13
FGD4	0.447	0.422	0.486	0.534	0.269	1.60E-13
NXF1	-0.049	0.181	0.029	-0.475	-0.243	1.61E-13
LARP6	0.434	0.112	0.085	0.151	0.729	1.62E-13
QTRT1	-0.147	0.12	0.114	-0.386	-0.513	1.64E-13
MXD1	0.223	0.418	0.351	0.058	-0.171	1.68E-13
PSKH1	-0.016	0.02	-0.066	0.141	0.465	1.69E-13
RP11-323P1	0.171	0.234	0.007	-0.945	-1.028	1.72E-13
AC133109.1	0.412	0.888	0.722	0.78	1.933	1.73E-13
POLG2	-0.706	-0.004	0.225	-0.38	-0.59	1.73E-13
RP4-539M6.	0.083	-0.167	-0.172	0.076	0.897	1.75E-13
SUFU	-0.121	-0.171	-0.053	0.295	0.545	1.76E-13
TRMU	-0.226	-0.015	-0.135	-0.405	-0.602	1.78E-13
RP11-111F1	0.299	0.198	0.351	0.605	0.75	1.80E-13
HMGN2	-0.201	-0.097	-0.072	-0.251	-0.81	1.80E-13
EP400NL	-0.384	0.07	0.08	-0.684	-0.661	1.81E-13
MYD88	0.123	-0.019	-0.08	0.179	0.455	1.83E-13
DCTN2	0.165	0.143	0.149	0.448	0.453	1.83E-13
EVL	-0.23	0.002	-0.151	-0.51	-0.047	1.89E-13
RP11-381E2	-0.628	-0.385	-0.505	-1.437	-1.356	1.89E-13
RAB10	0.296	0.056	0.011	0.328	0.571	1.94E-13
AK9	-0.137	0.011	0.435	0.58	0.988	1.95E-13
SEPSECS	-0.092	0.317	0.01	-0.199	-0.493	1.95E-13
TSPO	0.04	-0.096	-0.074	0.188	0.481	1.97E-13
SAMD10	-0.851	-0.114	0.058	0.076	0.544	1.99E-13
RP11-278C7	0.163	0.125	0.16	0.385	0.489	1.99E-13

CTD-2568P8	0.219	-0.084	-0.23	0.038	-0.424	1.99E-13
SDHAP1	-0.199	0.135	0.044	-0.92	-0.793	2.05E-13
KB-1572G7.2	-0.454	0.363	0.36	-0.68	-0.686	2.07E-13
SLC35D2	0.032	-0.228	0.156	0.231	0.693	2.11E-13
BCL3	0.676	0.497	0.152	-0.028	0.486	2.14E-13
RP11-25K19	-0.248	-0.152	-0.315	-0.129	-0.942	2.15E-13
RP6-218J18.	0.013	-0.069	-0.117	-0.509	-0.567	2.16E-13
UBR7	0.195	0.017	-0.239	-0.08	-0.515	2.19E-13
MMP2	0.347	0.075	-0.067	0.378	0.715	2.20E-13
GPRC5A	0.319	0.09	0.436	0.758	0.347	2.26E-13
CCDC14	-0.585	-0.033	0.077	-0.714	-0.758	2.26E-13
ACSL1	0.408	0.172	0.115	0.253	0.628	2.27E-13
CTD-2033D1	-0.331	0.065	0.109	-0.628	-0.689	2.28E-13
NBL1	0.156	-0.037	-0.073	0.14	0.525	2.32E-13
FBXO28	0.092	0.274	0.117	0.278	0.5	2.32E-13
SOCS4	0.481	0.165	0.145	-0.247	-0.062	2.32E-13
ZNF507	0.107	0.051	-0.11	-0.14	-0.482	2.34E-13
PNMA1	0.251	-0.2	-0.138	0.369	0.346	2.39E-13
RAB2A	0.414	0.002	-0.23	0.02	0.158	2.44E-13
KIAA0040	0.002	0.195	-0.033	-0.08	0.493	2.45E-13
ABI3BP	0.219	0.102	0.09	0.363	0.841	2.46E-13
LPCAT2	-0.004	-0.144	-0.291	-0.233	-0.377	2.56E-13
LMBR1L	-0.33	0.145	0.2	-0.139	0.444	2.58E-13
DUSP3	0.245	0.035	0.104	0.391	0.447	2.59E-13
LDOC1	0.076	-0.005	0.049	0.446	0.699	2.63E-13
AP1S1	0.151	-0.011	0.069	0.282	0.394	2.63E-13
SLC25A24	0.395	-0.026	-0.141	0.203	0.114	2.63E-13
CHKB	-0.578	0.184	0.315	-0.544	-0.328	2.63E-13
C16orf72	0.447	0.142	-0.008	0.266	0.386	2.64E-13
POLA2	0.198	0.227	0.023	0.133	-0.497	2.70E-13
CTNNB1	0.284	0.039	-0.109	0.035	0.37	2.74E-13
FAM76B	0.081	0.122	-0.102	-0.627	-0.512	2.76E-13
MEAF6	-0.056	-0.095	0.011	0.127	0.416	2.78E-13
DENND1A	0.419	0.015	0.35	0.738	0.377	2.78E-13
RP3-412A9.1	-0.177	-0.105	-0.212	-0.345	-0.713	2.81E-13
ALPL	0.79	0.689	0.236	0.292	-1.034	2.81E-13
MTERF3	-0.285	-0.121	-0.292	-0.306	-0.596	2.82E-13
ANKRD36C	-0.767	-0.144	0.154	-0.965	-1.632	2.82E-13

TPX2	-0.31	-0.7	-0.582	-0.013	-0.1	2.83E-13
TMEM131	0.117	0.353	0.336	0.509	0.571	2.85E-13
RAC1	0.356	0.052	-0.055	0.23	0.47	2.93E-13
ZNF761	-0.125	0.31	0.227	-0.307	-0.42	2.99E-13
ZNF778	0.041	0.113	-0.018	-0.508	-0.476	2.99E-13
DNAJA3	-0.142	-0.004	-0.069	0.013	-0.463	3.02E-13
DNAJC10	0.209	0.154	0.089	0.325	0.396	3.05E-13
SCRIB	0.098	0.336	0.415	0.461	0.515	3.06E-13
HOXB2	-0.737	-0.278	-0.454	-0.535	-0.392	3.06E-13
PILRB	-0.778	-0.16	0.08	-0.926	-0.743	3.09E-13
ELF1	0.581	0.093	0.017	0.316	0.246	3.15E-13
UBTD2	0.16	-0.036	-0.036	0.138	0.477	3.16E-13
SLC35E4	-0.05	0.029	0.026	0.079	0.651	3.17E-13
ADNP	0.216	0.038	-0.056	0.199	0.411	3.19E-13
B3GALT6	-0.157	-0.051	0.101	0.361	0.543	3.20E-13
TOR2A	-0.213	0.148	-0.013	-0.258	-0.558	3.23E-13
MSH5	-0.719	-0.29	0.258	-0.781	-1.145	3.31E-13
STYXL1	-0.049	0.052	0.261	0.392	0.525	3.33E-13
CLEC4GP1	-1.853	-0.906	-0.02	-0.564	-0.337	3.34E-13
TMEM187	-0.089	-0.065	0.143	0.57	0.686	3.39E-13
CTA-384D8.3	0.161	0.169	-0.04	-0.542	-1.074	3.58E-13
THUMPD3	-0.308	-0.119	0.063	0.166	-0.381	3.62E-13
HDX	-0.489	0.386	0.033	0.024	0.505	3.65E-13
IDH1	0.325	-0.107	-0.238	0.163	0.46	3.70E-13
CYP2R1	-0.841	-0.162	0.176	-0.227	-0.486	3.80E-13
BRD3	0.16	0.124	-0.039	0.129	0.457	3.85E-13
RANBP10	-0.135	0.335	0.134	0.064	0.32	3.92E-13
C6orf62	0.241	0.357	0.256	0.256	0.218	3.92E-13
INTS7	0.354	0.066	-0.231	0.002	-0.138	3.94E-13
MTMR12	0.142	-0.03	-0.078	0.137	0.463	4.00E-13
BTBD6	0.017	-0.137	-0.079	-0.122	-0.398	4.05E-13
RIC1	0.06	0.353	0.065	-0.113	0.048	4.06E-13
ENGASE	-0.643	-0.018	-0.153	-0.686	-0.445	4.07E-13
PTPN9	0.231	-0.022	-0.142	0.187	0.401	4.10E-13
PPIL1	-0.032	-0.247	-0.167	-0.09	-0.653	4.14E-13
AC010084.1	-0.089	0.028	-0.146	-0.031	-1.366	4.17E-13
TRIM2	0.206	0.011	-0.013	0.154	0.726	4.21E-13
UBE3B	0.027	0.039	0.218	0.242	0.376	4.25E-13

GRAMD1B	0.098	0.139	0.393	0.138	1.067	4.27E-13
DGKQ	-0.637	-0.073	-0.174	-0.822	-0.431	4.27E-13
LTBP2	0.343	0.006	-0.209	0.084	0.688	4.28E-13
PGM2	0.154	-0.035	-0.044	0.364	0.542	4.28E-13
SLC25A40	-0.171	0.016	-0.198	-0.409	-0.536	4.28E-13
ZER1	0.205	0.198	0.382	0.709	0.777	4.34E-13
ZSCAN16	-0.743	0.015	0.232	0.246	0.776	4.35E-13
RP11-370B1	0.528	0.031	-0.232	-0.005	0.374	4.35E-13
MCCC1	-0.14	-0.008	0.107	0.422	0.073	4.36E-13
BICD2	-0.105	-0.057	-0.172	-0.561	-0.498	4.36E-13
PABPC3	0.111	0.035	0.109	0.219	0.445	4.37E-13
FAM217B	-0.129	0.165	-0.138	-0.062	-0.506	4.37E-13
PIGN	-0.037	-0.054	-0.016	0.248	0.354	4.42E-13
PDE8A	0.367	0.131	-0.11	-0.242	0.066	4.44E-13
TUBG2	-0.423	0.041	0.002	-0.236	-0.48	4.44E-13
CTD-2224J9	-0.271	0.216	-0.139	-0.411	-0.695	4.46E-13
NAT9	-0.411	0.091	0.12	-0.331	-0.19	4.48E-13
SMARCD3	0.012	-0.269	-0.374	-0.807	-0.987	4.54E-13
WDR37	0.405	0.012	-0.043	0.088	0.271	4.61E-13
LPAR1	-0.629	0.13	0.996	0.823	0.248	4.69E-13
PARD6A	-0.523	-0.277	0.493	1.021	0.875	4.81E-13
HIST1H2BK	0.37	0.367	0.382	0.945	0.936	4.82E-13
HLA-B	0.251	0.328	0.166	0.524	0.662	4.90E-13
RUBCN	-0.266	0.183	0.088	-0.36	-0.235	5.00E-13
HDAC5	0.358	0.404	0.348	0.472	0.716	5.07E-13
KIF1B	0.481	0.272	0.05	0.186	0.383	5.10E-13
A4GALT	0.211	-0.009	0.025	0.26	0.674	5.11E-13
SLC25A6	0.184	0.085	0.063	0.316	0.524	5.13E-13
PRSS53	-0.573	-0.181	0.012	-0.909	-0.764	5.13E-13
CADPS2	0.616	0.087	-0.111	0.115	-0.292	5.15E-13
C9orf72	0.128	-0.138	-0.219	-0.744	-0.893	5.18E-13
AC026271.5	0.176	0.038	-0.109	0.239	0.397	5.19E-13
RELL2	-0.538	0.181	0.287	-0.255	-0.441	5.19E-13
TP53	0.15	0.235	0.221	0.448	0.695	5.22E-13
C8orf58	-0.309	-0.143	-0.133	-0.313	-0.577	5.22E-13
STK11	-0.043	0.173	0.214	0.155	0.425	5.23E-13
SH3BP4	0.741	0.099	0.038	0.287	0.273	5.25E-13
NNT	0.099	-0.021	-0.048	0.042	0.387	5.27E-13

AC002116.8	-0.572	0.08	-0.227	-1.101	-1.203	5.29E-13
CNPY4	0.042	0.011	0.041	0.344	0.5	5.33E-13
PLPP5	-0.1	-0.04	0.001	-0.281	-0.538	5.37E-13
SCAMP5	-0.146	-0.137	0.154	0.5	0.709	5.38E-13
CYTH1	0.272	0.349	0.272	0.087	0.45	5.48E-13
DDX11L2	-0.522	0.417	0.214	-0.132	-0.583	5.53E-13
PCDHGC3	0.197	0.018	-0.004	0.312	0.729	5.55E-13
NBPF8P	-0.507	0.03	0.356	-0.6	-0.688	5.57E-13
TRIM66	-0.521	-0.011	0.418	-0.402	-0.317	5.59E-13
BTD	0.183	0.126	0.1	0.389	0.637	5.74E-13
MIR3064	-0.722	0.074	0.151	-1.01	-0.726	5.75E-13
MED20	0.186	0.255	0.19	0.407	-0.021	5.78E-13
ANKRD13D	-0.725	-0.226	-0.14	-0.729	-0.47	5.81E-13
MDM4	-0.451	-0.059	0.178	-0.702	-0.82	5.82E-13
EXOSC2	-0.071	-0.137	-0.223	-0.231	-0.606	5.93E-13
SPR	0.067	0.016	0.125	0.415	0.634	5.96E-13
TMED8	0.095	0.146	-0.201	-0.201	-0.348	6.02E-13
KCNK6	-0.301	0.038	0.226	-0.494	-0.486	6.10E-13
CTDSP2	0.164	0	-0.06	0.176	0.441	6.17E-13
CXCL8	-0.937	0.012	-0.628	-1.346	-0.567	6.18E-13
UBFD1	0.319	0.075	0.05	0.192	0.567	6.21E-13
NFRKB	-0.303	-0.037	0.028	-0.134	-0.331	6.21E-13
RP3-368A4.6	-0.67	0.062	0.124	-1.051	-0.864	6.42E-13
BTAF1	-0.219	0.28	0.209	-0.468	-0.381	6.46E-13
MOB3A	-0.025	0.134	0.373	0.631	0.627	6.50E-13
RP11-231C1	-0.293	-0.039	0.17	-0.952	-0.678	6.50E-13
MRPS28	-0.018	-0.175	-0.249	-0.297	-0.725	6.63E-13
BNC1	-0.235	0.109	0.163	0.338	0.319	6.69E-13
TLR1	-0.137	0.228	0.277	0.618	0.944	6.81E-13
D2HGDH	-0.572	-0.086	-0.007	-0.487	-0.643	6.90E-13
TRPV2	-0.047	-0.265	-0.033	0.386	0.879	7.07E-13
HDAC7	-0.196	-0.26	-0.107	-0.281	-0.473	7.12E-13
CDKN3	-0.814	-0.906	-0.796	-0.854	-1.017	7.18E-13
SNCG	-0.076	-0.169	-0.057	0.179	0.474	7.22E-13
SCFD2	0.529	-0.099	-0.268	0.074	-0.333	7.25E-13
SNORD36A	-0.476	-0.004	-0.132	-1.297	-1.03	7.27E-13
AMBRA1	0.207	0.236	0.13	0.312	0.646	7.31E-13
METTL17	-0.259	-0.082	-0.104	-0.479	-0.743	7.36E-13

NFATC1	0.786	0.188	0.089	0.23	0.165	7.52E-13
RNF215	-0.636	-0.295	-0.083	-0.536	-0.52	7.55E-13
CISD1	0.043	-0.054	0.092	0.119	0.473	7.57E-13
VPS26A	0.073	0.04	-0.063	0.195	0.322	7.67E-13
RP11-115D1	0.095	-0.456	-0.218	-0.599	-1.654	7.81E-13
IFRD2	-0.051	-0.077	-0.179	-0.193	-0.36	7.83E-13
LOXL4	0.289	0.517	0.638	0.98	1.34	7.92E-13
DNAJA1	0.235	0.245	0.221	0.52	0.728	7.96E-13
RBM5	-0.373	0.029	0.142	-0.406	-0.353	7.97E-13
SMARCA1	0.312	0.262	0.146	0.565	1.012	8.05E-13
RP11-448G1	-0.036	-0.002	0.2	-1.029	-0.812	8.07E-13
NDRG3	-0.06	-0.092	0.079	0.35	0.373	8.10E-13
HAUS8	-0.05	-0.329	-0.359	-0.266	-0.743	8.17E-13
RUFY1	0.255	0.044	0.11	0.305	0.559	8.29E-13
PTPRS	-0.116	0.256	0.124	0.078	0.894	8.31E-13
DUS3L	-0.006	-0.048	-0.119	-0.368	-0.628	8.39E-13
RP11-657G2	0.407	-0.314	-0.623	-0.594	-0.31	8.42E-13
PPRC1	-0.017	-0.21	-0.234	-0.457	-0.391	8.42E-13
RNF11	0.263	0.157	0.009	0.04	0.379	8.50E-13
C1GALT1C1	-0.05	-0.108	0.048	0.267	0.447	8.62E-13
SLC37A1	0.13	0.371	0.567	0.438	1.126	8.70E-13
SLC25A4	0.145	0.243	0.124	0.502	0.644	8.70E-13
SLC37A4	-0.393	0.041	0	-0.057	-0.515	8.94E-13
HERC2P4	-0.538	-0.067	-0.175	-1.102	-1.029	9.25E-13
RP11-100G1	1.229	1.584	1.696	1.303	1.669	9.33E-13
WIPF1	0.15	0.163	0.18	0.41	0.643	9.38E-13
AP2A2	0.065	-0.094	-0.078	0.191	0.556	9.60E-13
RPP30	0.122	0.102	-0.069	-0.069	-0.418	9.62E-13
AC004987.9	0.303	-0.197	-0.07	0.391	0.762	9.82E-13
BSG	0.194	0.036	-0.056	0.388	0.622	9.90E-13
ITGA10	-0.025	0.108	0.085	-0.38	-0.239	9.90E-13
ALDH1A3	0.698	0.03	-0.396	-0.152	0.111	9.95E-13
AC093838.4	-0.331	0.117	0.02	-0.949	-0.578	1.01E-12
AC009299.5	0.068	-0.04	-0.052	0.138	0.595	1.03E-12
TGFB2	-0.35	0.329	-0.016	0.154	-0.387	1.04E-12
IMP4	0.029	-0.161	-0.169	-0.176	-0.49	1.04E-12
MPP3	-0.163	-0.112	0.132	-0.647	-0.891	1.04E-12
DDX51	-0.141	-0.108	-0.125	-0.372	-0.546	1.05E-12

UBLCP1	0.158	-0.018	0.087	0.387	0.385	1.06E-12
REPIN1	-0.322	-0.125	-0.178	-0.122	0.24	1.06E-12
PHF5A	0.002	-0.141	-0.139	-0.186	-0.549	1.07E-12
MUS81	-0.454	-0.043	-0.052	-0.48	-0.569	1.07E-12
SLC30A3	0.16	-0.095	-0.493	-0.663	-1.691	1.08E-12
RP11-1H8.3	0.346	-0.135	-0.157	0.308	0.837	1.10E-12
ARID4A	-0.062	0.268	0.004	-0.113	0.347	1.10E-12
FAM196B	0.126	-0.36	0.088	0.493	0.184	1.10E-12
NME7	0.068	-0.136	-0.054	0.336	0.571	1.11E-12
CTD-2552B1	0.408	-0.221	-0.161	-0.814	-0.283	1.11E-12
PATZ1	-0.35	-0.407	-0.38	-0.014	0.255	1.13E-12
SMAP1	0.591	0.053	-0.211	0.001	-0.033	1.13E-12
IFITM2	0.178	-0.025	0.045	0.471	0.592	1.14E-12
ASMTL	0.378	0.05	-0.221	-0.073	-0.446	1.14E-12
PRMT2	0.149	0.057	0.122	0.265	0.339	1.15E-12
RP11-395L1	0.186	0.156	0.154	0.293	-0.528	1.15E-12
RHOD	-0.183	0.368	0.166	0.682	1.413	1.17E-12
DEPDC5	-0.346	0.081	-0.027	-0.342	-0.52	1.17E-12
ARHGEF40	0.123	0.508	0.498	0.103	0.779	1.19E-12
CCDC142	-0.688	-0.104	0.128	-0.435	-0.614	1.20E-12
FANCF	-0.555	-0.014	0.164	0.405	0.551	1.22E-12
NDRG1	0.732	0.179	0.009	0.338	0.445	1.23E-12
NT5DC2	0.243	-0.043	-0.19	-0.144	-0.381	1.23E-12
WASH7P	-0.35	0.078	0.041	-0.529	-0.704	1.23E-12
RP5-857K21	-0.467	-0.571	-0.055	-1.517	-1.035	1.23E-12
ZNF260	0.392	0.283	0.128	0.153	0.626	1.24E-12
REEP4	-0.519	-0.549	-0.576	-0.447	-0.556	1.26E-12
SERINC5	0.209	0.612	0.349	0.03	0.503	1.27E-12
RP11-147L1	0.111	0.208	0.22	0.318	0.523	1.28E-12
MXD4	-0.083	-0.096	0.084	0.252	0.438	1.28E-12
RP11-27I1.2	-0.744	-0.097	0.28	0.217	0.217	1.32E-12
RAI1	-0.162	-0.183	-0.086	0.002	0.391	1.33E-12
GDF7	-0.637	0.563	0.428	-0.006	0.22	1.34E-12
ODF2	-0.172	-0.181	-0.196	-0.409	-0.478	1.35E-12
MAK16	0.003	0.029	-0.125	-0.017	-0.45	1.36E-12
POLR3F	0.194	0.129	-0.078	-0.063	-0.42	1.38E-12
RNPEP	0.214	0.075	0.058	0.382	0.681	1.43E-12
SYT11	0.043	-0.008	0.054	0.514	0.396	1.44E-12

ZCCHC14	0.565	0.172	0.232	0.242	0.362	1.44E-12
RP11-289I1(	0.013	-0.103	-0.069	0.147	0.629	1.45E-12
FASN	0.397	-0.214	-0.533	-0.537	-0.104	1.45E-12
TBC1D24	-0.266	0.036	-0.026	-0.307	-0.41	1.45E-12
RP11-566E1	0.135	-0.36	-0.258	-0.091	0.818	1.47E-12
ETNK2	0.106	0.198	0.297	0.684	0.578	1.49E-12
HDAC2	0.29	0.093	-0.027	0.306	0.509	1.49E-12
RFTN1	0.547	-0.038	-0.105	0.35	0.302	1.50E-12
ASF1B	0.27	0.157	-0.131	-0.007	-0.541	1.50E-12
OAZ2	0.126	0.038	0.031	0.33	0.417	1.51E-12
MAN2A2	-0.442	-0.079	-0.04	-0.419	0.048	1.52E-12
C17orf80	-0.293	-0.001	-0.048	-0.19	-0.507	1.56E-12
SNAPC4	-0.501	-0.007	-0.101	-0.663	-0.109	1.57E-12
IL12A	0.544	1.478	1.564	0.608	1.6	1.59E-12
HOOK2	-0.565	-0.18	0.177	-0.359	-0.35	1.59E-12
C9orf91	0.693	0.173	-0.044	0.026	-0.084	1.60E-12
RHOT2	-0.274	-0.015	-0.115	-0.529	-0.522	1.62E-12
ZNF496	0.242	-0.013	-0.043	0.009	0.344	1.66E-12
MTIF2	-0.047	0.089	0.221	0.402	-0.062	1.66E-12
SBNO2	-0.007	0.242	0.29	0.013	0.405	1.68E-12
RNASET2	0.057	-0.125	-0.06	0.235	0.703	1.70E-12
RP5-890O3.(	-0.584	-0.032	0.212	-1.099	-0.93	1.71E-12
MAN2B1	0.095	0.232	0.139	0.273	0.627	1.73E-12
EVI2A	-0.727	-1.177	-1.096	-0.968	0.366	1.73E-12
SLC35G2	-0.098	0.064	-0.083	-0.207	-0.685	1.73E-12
C20orf194	-0.176	0.007	0.27	0.065	0.352	1.74E-12
PTBP1	0.38	0.051	-0.292	-0.237	0.01	1.81E-12
FUS	0.378	0.031	-0.185	-0.195	-0.181	1.85E-12
AP5B1	0.393	0.103	-0.065	-0.181	-0.184	1.85E-12
MND1	-0.329	0.067	-0.163	-0.385	-1.267	1.86E-12
SNORA6	-0.316	-0.173	-0.083	-1.079	-1.361	1.86E-12
ATG7	0.173	0.138	0.226	0.533	0.525	1.89E-12
ZGLP1	-0.345	0.259	0.291	-0.908	-0.905	1.90E-12
GGA2	-0.136	-0.148	-0.208	-0.533	-0.303	1.96E-12
TMEM144	-0.097	0.67	0.904	0.123	0.714	2.00E-12
ATXN7L3B	0.162	0.07	-0.088	0.286	0.423	2.00E-12
KBTBD8	0.191	0.143	0.102	-0.153	-0.686	2.00E-12
TMEM128	-0.109	-0.043	-0.077	0.002	-0.646	2.03E-12

POC5	-0.429	-0.394	-0.345	-0.298	-0.962	2.04E-12
ARHGEF3	0.606	0.484	0.312	0.434	0.831	2.05E-12
LYPLA2P1	0.284	0.066	0.211	0.499	0.652	2.05E-12
SRPK2	-0.006	-0.002	-0.125	0.138	0.395	2.06E-12
ACAD10	-0.601	-0.043	0.343	-0.069	0.036	2.06E-12
CBARP	-0.757	-0.109	-0.152	-0.823	-0.108	2.06E-12
DYNC1I2P1	0.41	0.155	0.107	0.617	0.681	2.07E-12
ITGBL1	-0.007	-0.039	-0.002	0.288	0.531	2.08E-12
EVI2B	0.048	-0.951	-0.827	-0.152	-0.247	2.09E-12
ACADSB	-0.103	0.156	0.049	0.342	0.461	2.10E-12
RP11-331F4	-0.885	0.004	0.161	-1.023	-0.57	2.11E-12
RABGAP1L	0.556	0.104	0.139	0.551	0.713	2.12E-12
CTB-89H12.4	-0.255	-0.037	0.281	-0.831	-0.646	2.12E-12
LASP1	0.195	-0.033	0.027	0.454	0.651	2.13E-12
EIF2D	0.09	0.076	0.172	0.307	0.407	2.15E-12
HP1BP3	-0.373	-0.109	0.13	-0.295	0.167	2.20E-12
DHX35	0.032	-0.064	-0.151	-0.272	-0.58	2.20E-12
F2R	0.037	-0.157	-0.478	-0.293	-0.195	2.21E-12
GUF1	0.024	0.152	0.129	0.187	-0.338	2.21E-12
ITSN2	0.165	0.303	0.271	0.087	0.395	2.27E-12
LY6G5B	0.189	0.158	0.239	-0.905	-0.493	2.27E-12
TFB1M	-0.416	0.019	0.121	0.181	-0.393	2.29E-12
ORC2	-0.338	-0.107	0.091	0.103	-0.039	2.34E-12
UEVLD	-0.099	0.042	0.097	0.325	0.375	2.39E-12
SPCS3	0.137	-0.025	-0.16	-0.188	-0.425	2.39E-12
WASHC1	-0.395	0.09	0.118	-0.661	-0.663	2.41E-12
CHSY1	0.326	0.007	-0.262	-0.167	-0.175	2.46E-12
POLR3H	0.043	-0.384	-0.31	-0.102	-0.279	2.47E-12
CDC42EP4	0.698	0.032	0.225	0.61	0.427	2.51E-12
PLP2	0.089	-0.065	0.018	0.351	0.35	2.51E-12
MRPL20	-0.015	-0.08	-0.106	-0.143	-0.445	2.53E-12
JMJD4	-0.408	0.059	-0.134	-0.097	-0.459	2.53E-12
ATXN7L2	-0.124	-0.02	-0.277	-0.689	-0.805	2.54E-12
INF2	-0.13	0.031	0.238	0.093	0.391	2.55E-12
TRMT6	0.225	0.142	0.062	-0.014	-0.469	2.55E-12
PIGO	-0.203	-0.067	-0.045	-0.008	-0.559	2.59E-12
ANXA1	0.181	0.035	0.068	0.509	0.354	2.66E-12
PNN	-0.177	-0.09	-0.012	-0.532	-0.584	2.70E-12

BMP2K	-0.025	-0.124	0.065	0.094	0.421	2.76E-12
QRICH2	-0.675	-0.02	0.412	-0.116	0.707	2.86E-12
NFATC2IP	-0.263	-0.011	0.098	-0.392	-0.451	2.87E-12
WDR27	-0.818	-0.158	0.38	-0.424	-0.051	2.88E-12
MYL5	-0.275	0.315	0.366	-0.435	-0.466	2.89E-12
MEG3	-0.45	0.013	0.208	-0.875	-0.415	2.94E-12
BCL2L2	-0.384	0.066	-0.039	-0.73	-0.561	3.02E-12
ALG10	-0.425	-0.09	-0.146	-0.169	-0.805	3.02E-12
SRPX2	0.283	0.071	0.074	0.307	0.462	3.04E-12
NSD1	0.005	0.234	0.29	0.382	0.491	3.07E-12
SPEN	0.372	0.044	-0.044	-0.184	-0.061	3.08E-12
USP40	0.083	0.021	0.199	0.469	0.258	3.10E-12
ATE1	0.136	0.025	0.04	0.301	0.503	3.14E-12
NSUN5P2	-1.028	-0.175	0.045	-0.649	-0.512	3.16E-12
RP11-335I12	0.378	-0.135	-0.057	0.118	0.279	3.17E-12
SARM1	-0.193	-0.102	0.285	0.037	0.448	3.23E-12
AGAP4	-0.523	-0.074	0.038	-0.932	-0.495	3.27E-12
SECTM1	0.012	0.229	0.512	0.961	1.437	3.36E-12
DPP9	0.101	-0.046	0.107	0.276	0.432	3.50E-12
INTS6	0.435	0.21	-0.012	-0.198	0.057	3.51E-12
CTB-25B13.1	0.529	0.249	0.563	1.249	1.267	3.55E-12
IFT81	-0.267	0.003	0.219	0.344	0.719	3.55E-12
RP5-882C2.2	0.304	0.358	0.221	-0.875	-1.164	3.55E-12
SYF2	0.259	0.216	0.211	0.466	0.57	3.61E-12
NUMA1	-0.028	-0.205	-0.03	0.02	0.35	3.61E-12
SLC16A1	0.276	0.085	-0.052	0.258	0.45	3.66E-12
RP11-396K3	-0.152	0.35	0.106	0.512	1.004	3.67E-12
NPPA-AS1	-0.384	0.08	-0.052	-1.264	-1.082	3.67E-12
TP73-AS1	-0.439	0.161	0.234	-0.407	-0.136	3.68E-12
AC090937.2	0.008	-0.162	-0.23	-0.269	-0.366	3.68E-12
CREBZF	-0.221	0.069	0.055	-0.772	-0.643	3.70E-12
EXOC2	0.146	-0.127	0.033	0.511	0.414	3.72E-12
GID4	0.165	0.346	0.227	0.302	0.612	3.74E-12
RNF169	-0.147	0.198	0.281	0.221	0.561	3.74E-12
CAPN11	-0.022	-0.194	0.59	0.249	1.021	3.79E-12
RP3-508I15.2	-0.231	0.089	0.093	-0.977	-1.16	3.79E-12
TRIM37	-0.035	0.384	0.268	0.234	0.235	3.80E-12
ZNF18	-0.39	-0.011	-0.178	-0.499	-0.872	3.80E-12

RP11-1212A	-0.801	-0.18	-0.205	-1.192	-0.577	3.82E-12
C17orf51	-0.115	-0.258	-0.078	-0.028	0.413	3.87E-12
C4orf48	-0.305	-0.184	-0.163	-0.207	0.702	3.90E-12
FMN2	0.212	-0.032	-0.663	-0.491	-1.45	3.90E-12
IFNGR1	-0.051	0.258	0.107	-0.061	0.376	3.94E-12
KLHL7	-0.111	0.12	0.112	0.24	0.473	3.95E-12
CTD-3099C6	-0.255	0.053	0.079	-0.816	-0.811	4.06E-12
KLHL23	0.32	0.133	-0.439	-0.561	-0.021	4.11E-12
NDC1	-0.283	-0.265	-0.185	0.019	-0.478	4.14E-12
SNORD28	0.02	-0.141	-0.067	-0.976	-1.261	4.19E-12
HTRA3	0.383	0.151	-0.038	0.121	1.054	4.28E-12
GABARAPL1	0.442	0.482	0.252	0.341	0.291	4.28E-12
ABCC5	-0.745	-0.233	-0.05	-0.641	-0.272	4.28E-12
SEC61B	0.015	-0.057	-0.04	-0.162	-0.47	4.29E-12
XXbac-B444I	-0.542	-0.135	-0.083	-1.206	-1.556	4.29E-12
PYCR2	0.117	0.017	-0.019	0.109	-0.446	4.31E-12
CTU2	0.061	-0.182	-0.271	-0.086	-0.598	4.35E-12
FRMD3	0.769	0.125	-0.075	0.078	0.479	4.42E-12
PRICKLE2	0.844	-0.157	-0.34	-0.122	0.015	4.42E-12
HNRNPAB	0.393	0.107	-0.201	-0.028	-0.155	4.42E-12
ANKRA2	0.396	0.76	0.692	0.174	0.326	4.43E-12
INPP5B	-0.138	0.099	0.115	-0.125	-0.43	4.46E-12
SRPX	0.206	0.059	-0.062	0.363	0.487	4.50E-12
SP110	0.293	0.245	0.334	0.652	0.885	4.51E-12
PTER	0.212	0.174	0.014	0.718	0.808	4.55E-12
ICA1	0.06	0.213	0.503	0.439	0.347	4.55E-12
VPS4B	0.373	0.279	0.213	0.464	0.62	4.56E-12
BTBD2	0.054	-0.098	-0.082	0.27	0.585	4.56E-12
APPL2	0.601	0.002	-0.001	0.018	0.202	4.60E-12
FAM91A1	0.155	0.295	0.238	0.165	0.442	4.62E-12
MAPKAPK3	0.027	-0.098	-0.103	0.138	0.372	4.66E-12
SLC20A2	0.51	0.035	-0.186	0.101	0.11	4.67E-12
TXLNG2P	-0.599	-0.06	0.153	-0.864	-0.628	4.69E-12
AARS2	-0.154	0.098	0.149	-0.059	-0.384	4.70E-12
SCML1	-0.149	-0.2	-0.201	-0.639	-0.754	4.70E-12
CRIP2	0.322	-0.049	-0.295	-0.119	-0.426	4.73E-12
FASTK	-0.311	0.016	-0.017	-0.285	-0.425	4.75E-12
TMEM57	0.411	0.08	-0.167	-0.043	0.165	4.78E-12

RP11-500C1	0.151	0.709	0.306	-0.091	0.89	4.79E-12
UHRF2	-0.421	-0.032	0.125	-0.136	-0.095	4.79E-12
FAM198B	0.3	0.233	0.11	0.285	0.499	4.86E-12
GRPEL2	-0.075	0.333	0.487	0.006	0.319	4.88E-12
MKRN1	0.307	0.14	0.098	0.465	0.665	4.90E-12
ANXA2P2	0.348	0.056	0.038	0.558	0.672	4.92E-12
EFEMP1	0.17	0.04	-0.02	0.421	0.506	4.94E-12
ZNF75A	-0.495	0.062	0.333	-0.149	-0.189	4.94E-12
DHTKD1	-0.071	0.104	0.227	0.527	0.409	5.01E-12
RP11-262A1	0.377	-0.238	-0.259	-0.616	-1.35	5.06E-12
BOLA3	-0.293	-0.198	-0.263	-0.586	-0.904	5.35E-12
RFX7	0.161	0.37	0.226	0.076	0.438	5.41E-12
ALG14	0.077	-0.102	-0.117	-0.201	-0.743	5.49E-12
DUS1L	-0.179	0.045	-0.042	-0.378	-0.479	5.51E-12
RP11-203I2.	-0.94	0.007	0.024	-0.597	-0.755	5.52E-12
ETFDH	0.205	0.16	0.294	0.549	0.157	5.78E-12
MICALL1	0.256	0.137	0.128	0.426	0.678	5.83E-12
PLEKHG2	-0.358	-0.192	-0.227	-0.68	-0.422	5.83E-12
CCM2L	-0.105	-0.521	0.194	0.177	0.055	5.85E-12
CCDC88C	-0.413	-0.093	0.404	0.128	0.145	5.95E-12
MAML1	0.317	-0.058	-0.155	-0.007	0.332	5.96E-12
PHKB	0.102	0.074	0.148	0.265	0.382	5.98E-12
NR2F2	-0.004	-0.293	-0.212	0.193	-0.517	6.02E-12
FCHSD1	-0.278	0.145	0.161	-0.419	-0.375	6.05E-12
FIGN	-0.479	-0.424	-0.372	-0.35	-0.737	6.07E-12
AKTIP	-0.207	0.056	0.142	0.326	0.403	6.16E-12
PMCH	-0.609	-0.178	-0.388	-0.971	-1.55	6.16E-12
SNORD110	-0.224	0.065	-0.149	-1.257	-1.151	6.21E-12
CCNT2	-0.119	0.1	0.052	-0.643	-0.443	6.25E-12
LENG8	-0.521	-0.015	0.11	-0.827	-0.505	6.26E-12
FURIN	0.423	-0.026	-0.361	-0.145	-0.245	6.29E-12
PCSK1	-0.04	-0.133	-0.149	0.657	1.032	6.32E-12
PNO1	-0.015	-0.117	-0.162	-0.204	-0.523	6.32E-12
ECH1	0.213	-0.074	-0.057	0.334	0.522	6.52E-12
TRMT13	-0.357	-0.026	0.149	-0.221	-0.571	6.55E-12
RP11-1277A	-0.103	-0.282	-0.161	-1.187	-1.069	6.58E-12
ABHD17B	0.469	0.207	-0.021	0.016	0.38	6.63E-12
PATL1	0.385	0.248	-0.059	-0.01	-0.081	6.67E-12

MT-ATP8	-0.437	-0.348	0.234	-1.192	-1.082	6.72E-12
PLEKHG4	-0.446	-0.317	0.148	-0.439	0.24	6.77E-12
ZNF202	-0.25	0.061	0.092	-0.499	-0.499	6.83E-12
TUSC2	-0.039	0.105	0.21	0.245	0.463	6.93E-12
ZNF267	-0.249	0.099	-0.041	-0.078	0.434	6.99E-12
CD58	-0.008	0.057	-0.154	-0.893	-0.828	7.01E-12
PPA1	0.14	0.103	0.1	0.272	0.496	7.15E-12
PPP1R3E	-0.562	-0.153	-0.119	-0.757	-0.935	7.17E-12
TMEM138	-0.429	-0.248	-0.129	-0.324	-0.561	7.19E-12
KCTD13	-0.494	-0.056	0.067	-0.851	-0.346	7.22E-12
SPPL3	0.075	0.133	0.199	0.426	0.657	7.33E-12
TMX1	0.092	0.072	-0.063	-0.018	-0.363	7.39E-12
ZBTB33	0.253	0.133	-0.058	0.298	0.482	7.56E-12
PALM	0.367	-0.218	0.326	0.704	1.309	7.62E-12
MCOLN1	-0.037	0.042	-0.242	-0.157	-0.57	7.62E-12
PPIP5K2	-0.089	0.06	0.05	-0.06	-0.36	7.63E-12
RP11-18C24	-0.445	-0.074	0.329	0.259	0.486	7.67E-12
TMEM50B	-0.087	-0.233	-0.189	-0.698	-0.655	7.76E-12
RAPGEF4	0.615	-0.109	-0.261	-0.182	0.287	7.87E-12
VPS25	0.031	-0.111	0.044	0.176	-0.333	7.89E-12
WSB1	-0.572	0.096	0.391	-0.47	-0.295	8.04E-12
RALGDS	-0.182	0.225	0.406	0.015	0.78	8.10E-12
ANKLE1	-0.702	-0.278	-0.317	-0.815	-0.716	8.11E-12
SCRN3	0.068	0.011	0.126	0.478	0.339	8.12E-12
ALDOC	0.193	0.066	-0.218	0.048	-0.852	8.15E-12
PIP5K1C	0.088	-0.07	0.033	0.134	0.519	8.23E-12
PSMB10	0.199	-0.004	0.106	0.265	0.675	8.24E-12
NAPG	0.322	0.161	0.056	0.118	-0.122	8.24E-12
URI1	0.209	0.04	0.097	0.227	0.425	8.37E-12
RP11-389C8	-1.242	-0.537	-0.081	0.239	0.202	8.45E-12
SLC7A5	-0.155	-0.339	0.023	0.008	-0.643	8.47E-12
CTD-3252C9	-0.231	-0.046	0.163	-0.882	-0.755	8.49E-12
HSPB1	0.172	-0.047	-0.115	0.206	0.562	8.51E-12
RB1CC1	0.257	0.314	0.243	0.405	0.594	8.59E-12
LRP12	0.517	0.291	-0.177	0.016	0.238	8.67E-12
HDDC2	0.055	0.008	0.356	0.126	1.181	8.68E-12
FAM127A	0.179	0.038	-0.022	0.278	0.517	8.70E-12
MUM1	-0.464	-0.092	0.013	-0.514	-0.365	8.75E-12

ARL10	-0.569	-0.113	0.102	-0.345	-0.065	8.76E-12
TBCCD1	-0.35	0.015	0.026	-0.235	-0.523	9.05E-12
OFD1	-0.052	0.529	0.562	0.128	0.169	9.07E-12
AHRR	0.526	0.087	0.573	0.306	-0.045	9.14E-12
RP11-97E23	0.342	-0.351	-0.698	-0.577	-0.11	9.24E-12
SRSF6	0.237	0.014	-0.041	-0.509	-0.153	9.38E-12
ZBTB25	-0.53	0.081	0.176	-0.406	-0.6	9.42E-12
RP13-122B2	-0.216	0.06	-0.09	-0.685	-0.672	9.46E-12
KIAA0907	-0.296	-0.028	0.16	-0.497	-0.559	9.53E-12
CASP4	-0.035	0.042	0.099	0.205	0.332	9.60E-12
SLC6A8	0.407	-0.003	-0.269	-0.218	0.024	9.84E-12
APBB3	-0.206	0.278	0.426	-0.408	-0.622	9.86E-12
RP11-126F1	-0.597	-1.353	-1.51	-1.068	-0.755	9.86E-12
GSR	0.299	0.077	-0.187	0.036	-0.478	9.90E-12
ZNF318	-0.331	0.061	-0.154	-0.277	-0.278	9.92E-12
MAPKAPK2	0.217	0.033	-0.036	0.175	0.473	1.00E-11
TM9SF3	0.364	0.056	-0.184	0.058	-0.08	1.03E-11
MDN1	-0.345	-0.12	0.047	-0.445	-0.578	1.05E-11
NT5C	-0.016	0.071	-0.303	-0.353	-0.785	1.06E-11
ZCCHC6	-0.093	0.26	0.188	0.026	0.414	1.08E-11
EPB41L5	-0.217	-0.013	-0.356	-0.576	-0.023	1.09E-11
MT1E	0.071	-0.17	0.125	0.352	0.301	1.10E-11
TMEM194A	-0.216	-0.06	-0.211	-0.463	-0.503	1.10E-11
AK4	0.444	0.118	0.108	0.33	0.672	1.11E-11
SERPINE1	0.42	0.19	0.129	0.511	0.576	1.11E-11
MIR5047	-0.656	0.261	0.236	-0.811	-0.561	1.11E-11
LIMA1	0.256	0.122	0.004	0.397	0.589	1.12E-11
MORC2	-0.349	-0.031	0.122	0.223	0.226	1.12E-11
SCNN1D	-0.707	0.359	0.899	0.102	0.667	1.13E-11
EHBP1L1	-0.041	-0.012	0.339	0.241	0.404	1.13E-11
CXorf40B	0.105	0.045	-0.061	-0.025	-0.45	1.13E-11
RAD51	0.573	0.08	-0.372	-0.194	-0.586	1.13E-11
RP11-197N1	-0.396	0.084	-0.033	-0.877	-0.752	1.13E-11
DNAH1	-0.332	0.089	0.006	-0.192	0.787	1.14E-11
CEP44	-0.668	-0.179	-0.174	-0.484	-0.654	1.14E-11
AC097711.1	-0.222	-0.906	-0.697	0.002	-0.051	1.15E-11
CLDND1	-0.187	0.106	0.009	-0.23	-0.542	1.15E-11
CXXC5	0.396	-0.328	-0.023	0.069	0.6	1.16E-11

EIF4E	0.12	-0.225	-0.317	-0.269	-0.329	1.16E-11
PCF11	0.251	0.003	0.123	-0.309	-0.342	1.16E-11
POLM	-0.391	-0.16	-0.022	-0.076	-0.399	1.16E-11
MYZAP	0.256	-0.415	-0.021	0.114	0.748	1.17E-11
FAM114A1	0.41	0.172	0.179	0.394	0.625	1.17E-11
MYADM	0.594	0.224	-0.045	0.087	-0.304	1.20E-11
C14orf179	-0.011	-0.113	0.022	0.195	0.668	1.21E-11
PLOD1	0.217	0.029	-0.084	0.381	0.673	1.22E-11
ZNF84	-0.192	0.07	0.076	-0.444	0.43	1.22E-11
SLC38A1	0.195	-0.083	0.017	0.305	0.331	1.25E-11
L2HGDH	0.007	0.002	0.033	-0.144	-0.738	1.25E-11
ST5	0.056	-0.345	0.181	0.621	0.602	1.27E-11
GNE	0.605	0.323	0.133	0.219	0.277	1.27E-11
CCNL1	-0.307	0.12	0.011	-0.757	-0.584	1.27E-11
ZC3H7A	0.157	0.143	0.22	0.172	0.37	1.28E-11
FNBP4	-0.172	-0.014	0.107	-0.486	-0.413	1.28E-11
FBXW11	0.072	0.144	0.02	0.22	0.511	1.32E-11
RBMS1	0.342	0.022	-0.002	0.069	0.301	1.32E-11
LACC1	0.105	0.789	0.507	-0.018	0.469	1.34E-11
LUC7L	-0.217	0.129	0.039	-0.509	-0.335	1.39E-11
STRIP2	-0.268	-0.003	-0.165	-0.843	-0.445	1.42E-11
AKT2	-0.358	-0.099	-0.014	-0.389	-0.446	1.42E-11
SEPT10	0.207	0.087	0.073	0.328	0.362	1.45E-11
FOSL1	0.669	0.275	0.329	0.362	0.188	1.47E-11
EEF2K	-0.11	-0.406	-0.03	0.114	0.064	1.47E-11
TESK1	-0.035	0.056	-0.154	-0.531	-0.436	1.47E-11
BBS9	0.492	0.137	0.088	0.754	0.609	1.49E-11
CYP20A1	0.135	0.117	0.033	0.071	-0.449	1.51E-11
GPR146	0.047	-0.35	0.008	0.171	1.13	1.52E-11
NRN1	-0.262	-0.129	-0.127	-0.149	-0.571	1.52E-11
RTKN2	-0.557	-0.36	-0.295	-0.661	-0.889	1.52E-11
MAN1A1	0.151	0.115	0.005	0.101	0.611	1.54E-11
TMEM63A	-0.33	-0.084	0.222	0.017	0.202	1.54E-11
SEPN1	0.102	0.001	-0.051	0.198	0.521	1.55E-11
RCN3	0.23	0.107	0.012	0.442	0.616	1.60E-11
GAA	0.113	0.13	0.07	0.302	0.633	1.62E-11
GNPDA2	0.12	0.129	-0.175	-0.153	-0.355	1.62E-11
ITGB1	0.323	0.153	-0.008	0.401	0.532	1.64E-11

AFF4	0.48	0.245	0.125	0.193	0.379	1.64E-11
GSE1	0.486	-0.08	-0.265	-0.211	0.082	1.64E-11
TRIB2	0.129	-0.089	-0.243	-0.078	-0.675	1.65E-11
ZNF234	-0.21	0.151	0.019	-0.437	-0.527	1.66E-11
RP11-673E1	0.914	0.01	-0.05	-0.046	-0.312	1.68E-11
TBC1D9B	-0.069	-0.084	-0.064	0.052	0.331	1.70E-11
ARHGAP12	0.069	-0.05	0.133	0.236	0.49	1.72E-11
AC093673.5	-0.1	-0.286	0.022	0.24	0.488	1.73E-11
SENP7	-0.757	-0.248	-0.032	-0.485	-0.541	1.74E-11
HOMER1	0.348	-0.207	-0.373	-0.311	-0.535	1.76E-11
EME1	-0.529	-0.176	-0.137	-0.573	-0.822	1.76E-11
SH2B1	-0.448	-0.03	0.016	-0.52	-0.383	1.77E-11
NKTR	-0.255	-0.047	0.02	-0.796	-0.439	1.77E-11
RASIP1	-0.235	-0.065	0.139	0.257	0.442	1.79E-11
TBX20	0.907	0.068	-0.022	0.315	-0.147	1.81E-11
DOCK8	0.472	0.51	0.45	0.558	1.419	1.82E-11
RYK	0.018	-0.041	0.011	0.058	0.349	1.82E-11
POM121C	0.368	0.117	-0.049	-0.153	-0.129	1.84E-11
KRT19	0.397	-0.285	-0.313	0.225	0.607	1.85E-11
JAK3	-0.161	0.71	0.891	0.303	0.518	1.87E-11
ABHD10	-0.18	-0.12	-0.268	-0.103	-0.472	1.91E-11
PTPRK	0.286	0.062	0.014	0.064	0.591	1.92E-11
C5orf45	-0.867	-0.206	0.122	-0.528	-0.456	1.92E-11
SMARCC1	0.411	0.126	-0.05	0.282	0.66	1.93E-11
MT-ND3	-0.48	-0.356	0.247	-1.202	-0.935	1.93E-11
ARHGAP32	-0.722	-0.114	-0.052	-0.355	0.028	1.94E-11
XRN2	0.363	0.152	0.121	0.45	0.576	2.00E-11
FOXC1	0.611	-0.145	-0.348	0.05	0.069	2.02E-11
MAGOHB	-0.211	-0.079	-0.119	-0.453	-0.888	2.04E-11
ANKLE2	-0.028	0.05	-0.043	-0.364	-0.309	2.06E-11
CDC25C	-0.447	-0.739	-0.488	0.079	-0.071	2.07E-11
TXNRD2	-0.423	-0.063	0.24	0.084	0.134	2.09E-11
PIAS3	0.085	0.125	-0.017	-0.161	-0.332	2.09E-11
GEMIN6	-0.49	-0.282	-0.136	-0.209	-0.778	2.09E-11
AC024560.3	-0.229	-0.05	0.083	-0.643	-0.698	2.14E-11
DCTPP1	0.031	-0.231	-0.457	-0.289	-0.253	2.18E-11
CDH4	-0.01	-0.122	-0.165	0.168	0.718	2.22E-11
RSF1	0.186	0.233	0.187	0.215	0.526	2.22E-11

GNAI1	0.597	0.015	-0.165	0.258	0.431	2.23E-11
SDF4	0.174	0.066	-0.022	0.356	0.49	2.25E-11
GEMIN8	-0.056	-0.177	0.163	0.359	0.603	2.26E-11
CENPQ	0.016	0.067	-0.305	-0.465	-0.786	2.27E-11
ACAA1	-0.09	0.017	-0.023	0.01	0.339	2.28E-11
C6orf1	-0.377	-0.208	-0.072	0.121	-0.321	2.31E-11
NUDCD3	0.124	-0.173	-0.026	0.315	0.375	2.34E-11
PABPC1L	-0.734	-0.317	0.063	-0.99	-0.447	2.35E-11
TMEM200B	0.31	-0.278	-0.824	-1.003	-0.752	2.36E-11
PRMT3	-0.119	-0.148	-0.039	0.109	-0.591	2.38E-11
GNPTG	0.082	0.089	0.023	0.062	0.385	2.39E-11
GTF2IP1	0.322	0.072	0.278	0.331	0.928	2.44E-11
CACNB3	-0.295	-0.087	-0.099	-0.392	0.03	2.47E-11
PIM1	0.396	-0.156	-0.404	-0.142	-0.502	2.51E-11
MST1	-0.834	-0.018	0.237	-0.764	-0.713	2.52E-11
13-Sep	-0.405	-0.11	-0.205	-1.042	-1.132	2.55E-11
SOX17	0.504	0.327	0.128	-0.006	0.441	2.60E-11
RP11-18B3.2	-1.056	-1.274	-1.359	-1.162	-0.635	2.64E-11
SNORA40	-0.133	0.124	0.125	-0.623	-0.845	2.73E-11
ANKRD36	-0.755	-0.193	0.016	-0.977	-0.911	2.73E-11
STMN1	-0.212	-0.066	0.091	0.212	0.353	2.75E-11
RP11-884K1	-0.006	-0.083	-0.135	-0.293	-0.81	2.75E-11
RP11-420L9.	0.22	0.287	0.276	0.465	0.639	2.76E-11
RP11-334C1	-0.524	0.085	0.467	-0.363	-0.789	2.78E-11
ZNF697	0.143	0.386	0.323	0.313	0.601	2.79E-11
ATP1B1	0.208	0.402	0.217	0.441	0.571	2.79E-11
C5	-0.409	-0.146	0.254	0.099	-0.488	2.79E-11
MAP3K11	0.001	0.218	0.16	0.224	0.567	2.80E-11
FBN2	0.207	0.087	0.02	0.272	0.759	2.84E-11
RP11-274B2	-0.295	0.05	-0.096	-0.916	-0.665	2.86E-11
MAN2B2	0.093	0.078	-0.049	0.29	0.625	2.88E-11
KHDRBS1	0.337	0.134	-0.003	0.511	0.727	3.01E-11
GHITM	0.248	0.135	0.103	0.398	0.289	3.01E-11
IL15	-0.049	0.746	0.866	0.542	1.459	3.03E-11
NFKB1	0.108	0.584	0.117	0.226	0.543	3.03E-11
KIF11	-0.166	-0.343	-0.482	0.06	-0.575	3.03E-11
TNFSF12	-0.17	-0.19	-0.121	0.108	0.559	3.04E-11
TPRG1L	0.21	0.042	0.107	0.432	0.539	3.04E-11

USPL1	-0.245	0.092	-0.025	-0.422	-0.456	3.05E-11
COQ5	0.035	-0.119	0.025	0.438	0.215	3.07E-11
RP11-392E2	-0.127	-0.008	-0.171	-0.625	-1.065	3.09E-11
TAF4B	0.857	1.061	0.939	0.585	0.693	3.10E-11
FN3K	-0.253	-0.288	-0.078	0.352	0.929	3.16E-11
MDFIC	0.094	-0.199	-0.398	-0.262	0.196	3.20E-11
SSBP2	-0.205	-0.44	-0.573	-0.537	-1.062	3.20E-11
RP11-243J1	-0.618	-0.449	-0.354	-0.533	-1.218	3.21E-11
RP11-137L1	0.009	0.496	0.849	0.592	0.22	3.26E-11
EPAS1	0.381	0.042	-0.242	-0.129	-0.431	3.29E-11
RP11-119K6	0.406	0.342	-0.1	-0.859	-0.782	3.30E-11
NCAPG2	-0.009	0.024	-0.121	0.032	-0.466	3.36E-11
EPB41L2	0.052	0.153	0.174	0.095	0.432	3.37E-11
TNFRSF10A	0.323	0.393	0.485	0.299	0.424	3.39E-11
UACA	-0.187	-0.132	-0.311	-0.873	-0.307	3.39E-11
ETV1	0.118	-0.315	0.278	0.394	0.881	3.42E-11
CCL7	-0.073	1.164	1.108	0.46	1.672	3.47E-11
MANBA	-0.017	-0.083	-0.001	0.316	0.793	3.47E-11
IKBIP	-0.002	0.196	0.238	0.384	0.487	3.47E-11
TUBGCP3	0.184	-0.162	-0.218	-0.012	-0.394	3.51E-11
SLK	0.535	0.134	0.065	0.398	0.507	3.56E-11
FERMT1	0.443	0.122	0.044	0.629	0.875	3.61E-11
ZFYVE27	-0.173	0.056	-0.164	-0.559	-0.48	3.63E-11
C1orf159	-0.336	0.04	-0.085	-0.479	-0.634	3.63E-11
RASA2	-0.667	-0.023	0.131	-0.384	-0.28	3.64E-11
SMAGP	-0.031	-0.403	-0.247	0.163	-0.102	3.67E-11
ENKUR	0.823	0.423	0.308	0.133	-0.752	3.74E-11
HDHD2	0.074	0.008	0.092	0.407	0.453	3.77E-11
FAM57A	0.148	-0.114	-0.375	-0.335	-0.282	3.77E-11
HOXB3	-0.833	-0.325	-0.035	-0.682	-0.102	3.78E-11
USP35	-0.355	0.17	0.143	-0.547	-0.486	3.84E-11
CPNE8	-0.052	0.285	0.516	0.421	0.435	3.85E-11
MRPS25	-0.357	-0.037	0.041	-0.472	-0.482	3.85E-11
UBE2D1	0.277	0.213	0.035	0.149	0.564	3.89E-11
FER	0.246	-0.038	0.118	0.273	0.527	3.92E-11
TNFRSF6B	-0.182	0.124	0.358	0.313	1.088	3.93E-11
SS18L2	-0.655	-0.314	-0.382	-0.725	-0.918	3.98E-11
MAP2K5	0.066	0.164	0.327	0.62	0.566	4.02E-11

SIVA1	-0.312	-0.194	-0.24	-0.517	-0.775	4.02E-11
RBM25	-0.02	0.04	-0.013	-0.33	-0.227	4.14E-11
CTD-2530H1	0.144	0.072	0.094	0.373	0.533	4.19E-11
RP11-268J1	-0.091	0.241	0.349	-0.684	-0.596	4.19E-11
RASSF7	-0.71	-0.053	-0.09	-0.411	-0.359	4.20E-11
C19orf47	0.152	-0.009	-0.178	-0.105	-0.557	4.20E-11
RP11-677M1	-0.454	0.088	0.195	-0.722	-1.169	4.20E-11
ZNF792	-0.558	-0.09	0.296	0.41	0.451	4.24E-11
MRPL51	-0.101	-0.137	-0.074	-0.083	-0.442	4.27E-11
BIRC2	0.422	0.219	0.053	-0.153	-0.324	4.37E-11
TRIM45	-0.688	-0.145	-0.048	-0.861	-1.053	4.37E-11
SNORD45B	-0.027	0.356	0.371	-0.78	-0.816	4.40E-11
C11orf80	-0.311	0.007	0.407	0.167	0.607	4.42E-11
RPAP2	-0.343	0.171	0.124	0.103	0.212	4.43E-11
ASCC1	0.203	0.035	0.092	0.479	0.192	4.43E-11
RP3-393P12	0.106	0.13	0.212	0.216	0.801	4.47E-11
PIP4K2A	0.164	-0.164	-0.021	0.123	0.393	4.48E-11
RP11-206L1	-0.394	-0.012	0.135	-0.767	-0.817	4.52E-11
RP11-644F5	-0.736	-0.014	0.247	0.164	-0.113	4.54E-11
GOLGA8A	-0.433	0.049	0.235	-0.702	-0.34	4.54E-11
RP5-1100H1	0.063	-0.083	-0.532	-0.97	-1.312	4.56E-11
RP11-378A1	0.687	0.246	0.369	0.651	1.424	4.57E-11
RP11-123M2	0.389	-0.096	-0.061	-0.035	0.819	4.58E-11
GATS	-0.604	0.217	0.267	0.143	0.835	4.59E-11
MBOAT1	-0.241	0.075	0.094	-0.682	-1.08	4.65E-11
AQP3	0.172	0.175	0.349	0.653	-0.981	4.66E-11
TBC1D23	0.331	0.312	0.122	0.343	0.487	4.68E-11
SLC25A17	0.079	0.091	-0.171	-0.106	-0.379	4.71E-11
E4F1	-0.339	-0.042	-0.15	-0.541	-0.473	4.75E-11
PRKAG1	0.172	0.047	0.215	0.433	0.223	4.76E-11
RTTN	-0.06	0.092	-0.133	-0.207	-0.435	4.76E-11
SNORA18	-0.119	0.037	0.095	-0.8	-0.867	4.77E-11
UHRF1BP1L	0.48	0.264	0.01	0.07	-0.038	4.79E-11
NEXN	0.43	0.202	-0.035	0.087	-0.523	4.81E-11
HAUS6	-0.137	-0.109	-0.266	-0.308	-0.432	4.93E-11
TMEM184B	0.207	-0.077	-0.064	0.164	0.464	4.95E-11
AASS	-0.633	-0.179	-0.198	-0.48	-0.649	4.97E-11
TGS1	0.569	0.207	-0.036	0.146	0.089	4.99E-11

TJP2	0.534	0.038	0.151	0.542	1.003	5.00E-11
MYO10	0.304	0.02	0.151	0.263	0.431	5.02E-11
MIS18A	-0.095	-0.176	-0.32	-0.115	-0.606	5.04E-11
RP11-569G1	0.24	0.11	0.33	0.505	0.57	5.06E-11
DXO	-0.288	0.103	0.038	-0.261	-0.43	5.13E-11
AL449209.1	-1.407	-0.456	-0.134	-0.481	-0.397	5.22E-11
CASP9	-0.193	0.163	0.16	0.541	0.265	5.23E-11
ZNF281	0.355	0.194	0.126	0.253	0.58	5.25E-11
AC002056.3	0.077	0.017	-0.142	0.116	-0.677	5.26E-11
SOS2	0.38	0.22	0.163	0.124	0.508	5.27E-11
NBEAL2	-0.533	-0.356	0.282	-0.315	-0.148	5.28E-11
TMEM120A	-0.343	0.137	0.059	0.089	-0.347	5.28E-11
RP11-242D8	-0.734	-0.076	0.086	-0.762	-0.771	5.28E-11
OSBPL5	-0.157	-0.017	0.352	0.224	0.424	5.30E-11
BCL7A	0.526	0.033	0.099	0.259	0.632	5.33E-11
INO80C	-0.121	0.071	0.324	0.161	0.554	5.58E-11
NEDD9	0.619	0.197	-0.015	-0.06	0.242	5.62E-11
PIGH	-0.203	-0.008	0.084	-0.18	-0.733	5.62E-11
CDH24	-0.093	-0.097	-0.143	-0.546	-0.398	5.67E-11
VPS8	-0.02	0.117	0.244	0.353	0.372	5.77E-11
VASH1	-0.764	-0.66	-0.252	-0.849	-0.334	5.85E-11
WBSCR22	0.17	0.024	-0.157	0.039	-0.327	5.88E-11
ANXA2	0.328	0.096	0.037	0.534	0.649	5.91E-11
BMS1P1	-0.416	0.006	0.425	-0.687	-0.743	5.96E-11
FAM127B	0.186	0.125	0.176	0.498	0.644	5.99E-11
ITGB1P1	0.326	0.115	-0.01	0.433	0.542	6.06E-11
GBA	0.168	0.116	0.07	0.495	0.535	6.13E-11
BEX4	0.308	0.249	0.121	0.512	0.662	6.29E-11
C21orf91	0.37	0.206	-0.205	-0.366	-0.317	6.32E-11
ZNF117	-0.387	0.062	0.153	-0.784	-0.562	6.33E-11
SUN2	-0.075	-0.254	-0.369	0.01	0.299	6.34E-11
CSGALNAC	0.352	0.106	-0.05	-0.032	-0.012	6.47E-11
CEP290	-0.127	0.252	0.323	0.278	0.132	6.50E-11
CDC42EP5	-0.142	-0.248	-0.096	0.104	0.4	6.60E-11
CTD-2509G1	0.534	0.042	-0.314	-0.112	-0.047	6.62E-11
GLI4	-0.715	0.025	0.164	-0.484	-0.705	6.62E-11
IRF2BPL	0.328	0.361	0.465	0.692	0.742	6.64E-11
CTDSPL	-0.175	-0.231	-0.187	-0.05	0.338	6.66E-11

CTA-360L10	-0.559	-0.139	0.605	0.248	1.152	6.71E-11
TAX1BP3	0.251	0.122	0.11	0.261	0.355	6.92E-11
PLEKHF1	-0.493	0.261	0.319	0.636	0.867	6.98E-11
YTHDF3	0.399	0.149	0.009	0.168	0.305	7.00E-11
RP11-291L1!	0.014	-0.281	-0.202	-0.136	-0.51	7.02E-11
NT5C2	0.119	0.041	0.063	0.07	0.364	7.04E-11
VRK1	0.056	0.057	-0.275	-0.045	-0.388	7.05E-11
AGPAT4	-0.307	-0.106	0.17	-0.07	0.479	7.12E-11
SNORD5	-0.556	0.014	-0.005	-1.042	-1.029	7.14E-11
GNG12	0.346	-0.002	-0.103	0.134	0.236	7.23E-11
PRRC2B	0.073	-0.036	0.014	0.107	0.423	7.26E-11
SLC9A8	-0.059	0.162	0.116	-0.238	-0.337	7.28E-11
RNASEH2B	-0.066	0.031	-0.029	-0.323	-0.46	7.30E-11
RAB6B	-0.088	-0.355	0.158	0.193	0.926	7.32E-11
CCL14	-0.462	0.042	0.052	-0.04	0.549	7.34E-11
MCUR1	0.273	0.032	-0.115	-0.13	-0.334	7.38E-11
CCDC81	-0.054	0.013	0.588	0.373	1.415	7.39E-11
HCFC2	-0.218	-0.019	-0.135	-0.363	-0.523	7.40E-11
CDPF1	-0.588	-0.124	-0.188	-0.401	-0.687	7.40E-11
FYTTD1	0.217	0.064	-0.078	0.175	0.44	7.41E-11
PHYKPL	-0.385	0.17	0.366	-0.17	-0.036	7.45E-11
MT-TS1	-0.478	-0.023	0.364	-1.031	-0.909	7.46E-11
ANXA6	0.397	0.048	-0.02	0.455	0.773	7.47E-11
C14orf79	-0.835	-0.096	-0.019	-0.679	-0.255	7.56E-11
FKBP5	0.461	0.118	-0.189	-0.065	-0.389	7.61E-11
SDC3	0.199	0.244	0.21	0.536	0.636	7.66E-11
BCAR1	-0.426	-0.002	0.062	0.235	0.264	7.66E-11
CTB-175E5.4	-0.507	0.06	0.011	-0.527	-0.472	7.66E-11
ZRANB1	0.405	0.204	0.016	0.057	0.288	7.85E-11
UQCC1	-0.064	0.308	0.398	0.256	0.258	7.85E-11
EIF2AK1	0.303	0.079	-0.013	0.298	0.457	8.08E-11
NCK2	0.162	-0.046	-0.203	0.029	0.575	8.22E-11
PGD	0.397	0.19	0.077	0.526	0.757	8.28E-11
ALG5	-0.134	-0.179	-0.172	-0.353	-0.802	8.30E-11
NCKAP1	0.32	0.102	-0.006	0.323	0.542	8.39E-11
SLC25A21-A	-0.192	0.135	-0.179	-0.209	-1.429	8.42E-11
LATS2	0.424	0.174	0.28	0.374	0.441	8.56E-11
RBM39	0.091	0.123	0.099	-0.342	-0.315	8.63E-11

ELMOD3	-0.573	0.091	0.142	-0.383	-0.281	8.68E-11
RP11-274B2	-0.176	0.073	0.036	-0.75	-0.665	8.70E-11
NPTN-IT1	-0.414	-0.107	-0.109	-1.238	-0.837	8.75E-11
EFNA4	-0.1	-0.126	-0.285	-0.13	0.481	8.80E-11
TAF5	0.149	-0.041	-0.298	-0.22	-0.57	8.80E-11
INTS6L	-0.148	0.177	0.102	-0.741	-0.796	8.98E-11
ZBTB47	-0.42	-0.214	-0.034	-0.502	-0.186	9.02E-11
NPC2	-0.14	-0.152	-0.018	0.015	0.41	9.17E-11
RP1-167F1.1	0.102	0.069	0.212	0.606	1.193	9.26E-11
CDAN1	-0.37	-0.086	-0.199	-0.523	-0.425	9.30E-11
HADHA	0.325	0.201	0.148	0.542	0.753	9.32E-11
TMEM87B	0.04	0.105	0.157	0.146	0.425	9.33E-11
ARFGEF1	0.045	0.165	0.056	0.301	0.404	9.38E-11
PAGR1	-0.25	-0.353	-0.239	-0.047	-0.973	9.43E-11
GPR173	-0.344	-0.123	-0.054	-0.772	-0.482	9.65E-11
PACS1	0.321	-0.081	-0.207	0.013	0.396	9.71E-11
GGT5	-0.713	-0.279	0.032	0.086	-0.592	9.76E-11
TRAPPC5	-0.36	-0.055	-0.062	-0.215	0.962	9.78E-11
PAPSS2	0.402	0.155	-0.132	0.146	-1.133	9.78E-11
SLC35E3	0.119	0.437	0.485	0.284	0.524	9.98E-11
ARID5B	0.585	0.355	-0.02	0.096	0.552	1.00E-10
BAZ2B	-0.094	0.139	0.124	0.022	0.448	1.00E-10
MCM2	0.487	0.072	-0.443	-0.373	-0.851	1.01E-10
APLP1	0.264	0.528	0.481	0.905	0.726	1.04E-10
H2AFY	0.173	0.076	0.071	0.331	0.434	1.04E-10
ZCCHC24	0.179	-0.127	0.169	0.27	0.687	1.05E-10
SEC31A	0.493	0.214	0.18	0.559	0.816	1.06E-10
PPP1R16A	-0.656	-0.085	-0.046	-0.339	-0.28	1.06E-10
DDIT3	-0.854	-0.038	-0.089	-0.597	-0.385	1.06E-10
CAMK1	-0.117	0.12	0.124	0.309	0.425	1.07E-10
AC137932.6	-0.052	-0.073	0.858	1.04	0.927	1.08E-10
CHCHD7	-0.148	0.052	-0.132	-0.426	-0.674	1.08E-10
RP3-425C14	0.618	-0.133	-0.197	-0.102	0.878	1.09E-10
IQGAP1	0.357	0.176	0.111	0.397	0.698	1.09E-10
PMPCB	0.246	0.079	0.067	0.27	0.434	1.09E-10
BRICD5	-0.631	-0.283	0.005	-1.078	-0.629	1.10E-10
BTBD11	0.683	0.005	-0.23	0.024	0.819	1.15E-10
NLGN2	-0.214	0.105	0.057	-0.34	-0.253	1.15E-10

ZFAND2A	0.003	0.03	-0.046	-0.439	-0.617	1.16E-10
MIIP	-0.759	-0.358	-0.252	-0.491	-0.561	1.17E-10
LMCD1	0.153	-0.486	-0.405	-0.157	0.417	1.18E-10
SLC43A1	-0.576	-0.296	-0.423	-0.716	-0.481	1.18E-10
PSTPIP1	-0.114	-0.412	0.221	0.185	0.906	1.19E-10
KCNN4	0.334	0.065	0.567	0.869	1.165	1.20E-10
RGL3	-0.328	0.071	0.471	-0.45	-0.587	1.21E-10
MZT1	-0.468	-0.473	-0.502	-0.799	-0.876	1.21E-10
SLC35G1	0.093	0.115	-0.411	-0.495	-0.895	1.21E-10
FOPNL	-0.159	-0.251	-0.311	-0.426	-0.656	1.22E-10
MAGI3	-0.327	-0.149	-0.455	-0.453	-0.671	1.22E-10
SCARA3	0.078	-0.081	-0.132	0.215	0.507	1.23E-10
PPAT	0.009	-0.186	-0.339	-0.31	-0.394	1.24E-10
SLC25A22	-0.031	-0.183	-0.341	-0.441	-0.564	1.24E-10
RPL21	-0.109	-0.072	0.059	-0.637	-0.775	1.24E-10
ANKRD28	-0.004	0.097	0.34	0.339	0.297	1.25E-10
ARHGEF17	-0.232	-0.011	0.105	-0.347	0.096	1.25E-10
RAB42	0.058	-0.354	-0.41	-0.208	1.026	1.27E-10
FAM171B	0.204	0.266	0.159	0.13	0.618	1.28E-10
CREBRF	-0.125	0.484	0.206	-0.044	0.601	1.28E-10
GS1-44D20.1	0.334	-0.084	-0.147	-0.029	-0.032	1.28E-10
LPCAT1	0	-0.177	-0.291	-0.37	-0.131	1.28E-10
WIPI1	0.347	0.034	-0.034	0.083	0.293	1.31E-10
TPM4	0.335	0.036	-0.119	0.028	0.151	1.31E-10
JMY	0.101	0.265	0.345	0.148	0.615	1.33E-10
STX16	-0.36	0.058	0.24	-0.392	-0.286	1.34E-10
GLTP	0.269	0.133	0.104	0.408	0.346	1.35E-10
SLC23A3	-0.598	0.174	0.788	-0.402	-0.184	1.36E-10
MTMR1	0.091	0.269	0.281	0.303	0.384	1.37E-10
ADNP2	0.472	0.034	-0.059	0.141	0.315	1.38E-10
PORCN	0.118	0.002	-0.151	-0.277	-0.42	1.38E-10
CDC40	0.231	0.189	0.112	0.478	0.468	1.40E-10
NFKBIZ	0.182	0.354	0.25	-0.798	0.237	1.40E-10
HMGB3	0.251	-0.153	-0.362	-0.07	0.076	1.40E-10
ACP6	-0.882	-0.04	0.07	-0.574	-0.419	1.41E-10
PKM	0.362	0.117	0.089	0.569	0.833	1.42E-10
THADA	0.231	-0.076	0.25	0.446	0.269	1.42E-10
PPIP5K1	-0.307	0.046	0.188	-0.24	-0.466	1.42E-10

GALNT18	0.115	0.051	0.182	0.331	0.962	1.43E-10
HYAL3	0.087	0.118	0.349	0.47	0.803	1.43E-10
POU4F1	-0.29	-0.177	-0.275	-0.829	-0.411	1.44E-10
GLCCI1	0.052	0.39	0.235	0.232	0.891	1.45E-10
NSUN5P1	-1.025	-0.406	-0.098	-0.95	-0.668	1.45E-10
DCTD	0.128	0.064	0.066	0.202	0.325	1.46E-10
NUP155	0.325	0.144	-0.013	0.223	-0.357	1.47E-10
HIBCH	0.037	-0.034	0.086	0.308	0.459	1.48E-10
PKD1P6	-0.527	-0.336	-0.311	-1.226	-0.558	1.48E-10
RP11-172H2	0.153	-0.042	0.082	0.251	0.738	1.50E-10
PEA15	0.216	0.004	-0.023	0.233	0.389	1.51E-10
HSPA1A	-0.925	-0.313	-0.185	0.122	0.077	1.51E-10
GPRIN1	0.568	0.184	-0.161	0.106	0.493	1.53E-10
MAP1S	0.223	-0.094	0.208	0.49	0.639	1.54E-10
STX12	0.092	0.161	0.167	0.205	0.434	1.56E-10
AC004381.6	-0.639	0.071	0.088	-0.132	-0.528	1.57E-10
RP11-354P1	0.245	-0.075	-0.111	-0.533	0.632	1.58E-10
NCRNA0010	-0.671	-0.066	0.252	-0.717	-0.366	1.58E-10
IGFBP6	0.181	0.072	0.037	0.221	0.759	1.59E-10
MYH10	0.139	-0.049	-0.15	0.212	0.692	1.59E-10
PFKP	0.351	-0.024	-0.142	0.247	0.59	1.59E-10
MAN2C1	-0.328	-0.023	0.062	-0.594	-0.493	1.59E-10
MTHFD2	0.356	0.084	-0.135	0.058	-0.143	1.63E-10
ZCCHC9	-0.343	-0.069	-0.011	-0.345	-0.68	1.66E-10
TRPT1	-0.553	-0.116	0.109	-0.146	-0.263	1.68E-10
RORA	0.357	0.115	-0.029	-0.473	-0.282	1.71E-10
KDM5D	-0.389	0.03	0.107	-0.4	-0.466	1.71E-10
TMEM54	0.143	0.116	0.116	0.281	0.519	1.73E-10
MRPL17	-0.125	-0.34	-0.261	-0.146	-0.294	1.73E-10
SLC44A5	-0.176	-0.197	-0.143	-0.469	0.256	1.77E-10
ELK3	0.33	0.079	0.188	0.495	0.395	1.80E-10
ILKAP	0.09	-0.1	-0.183	-0.21	-0.363	1.81E-10
PCYT2	-0.023	-0.288	-0.444	-0.475	-0.268	1.82E-10
AC097639.4	-0.036	-0.438	0.034	0.559	0.967	1.84E-10
ZNF696	-0.691	0.2	0.014	-0.152	-0.11	1.86E-10
CORO1B	-0.042	0.091	0.07	0.428	0.538	1.87E-10
BTN2A2	0.063	0.409	0.305	0.161	0.579	1.90E-10
GTF2IRD2B	0.524	0.199	0.591	0.686	1.386	1.92E-10

TNPO1	0.148	0.117	-0.087	-0.016	-0.333	1.94E-10
CPEB4	0.604	0.322	0.076	0.096	0.226	1.95E-10
COL18A1	0.313	0.08	-0.065	0.287	0.656	1.98E-10
RNF111	0.413	0.236	0.018	0.005	0.329	1.98E-10
ACO1	0.27	0.132	0.104	0.585	0.791	2.00E-10
DENND5A	0.132	0.087	0.049	0.163	0.42	2.00E-10
CEP72	-0.249	0.062	-0.169	-0.323	-0.922	2.02E-10
PRKD1	-0.135	-0.204	-0.372	-0.749	-0.338	2.04E-10
RP11-568G1	0.289	0.099	0.056	0.384	0.346	2.07E-10
ANKRD49	-0.292	0.099	0.043	-0.465	-0.678	2.13E-10
ADCK3	0.262	0.752	0.67	0.666	0.689	2.14E-10
MAPK1	0.247	0.05	-0.003	0.286	0.485	2.14E-10
LRRC27	-0.846	-0.139	-0.043	-0.644	0.039	2.14E-10
SEC31B	-0.798	-0.096	0.196	-0.731	-0.069	2.14E-10
HYAL1	0.054	0.094	0.243	0.277	0.467	2.17E-10
MIER1	-0.051	-0.034	-0.169	-0.311	-0.437	2.19E-10
MTFR2	-0.273	-0.285	-0.43	-0.16	-0.759	2.19E-10
GHRLOS	-0.561	0.05	0.125	-1.33	-0.929	2.22E-10
RAP1GDS1	0.175	-0.048	-0.053	0.269	0.4	2.25E-10
DUSP12	0.067	-0.143	-0.16	-0.324	-0.445	2.28E-10
RP11-157P1	-0.655	-0.441	0.311	-0.529	-0.811	2.28E-10
NEIL1	-0.867	-0.204	0.045	-0.703	-0.593	2.30E-10
CHFR	-0.016	0.079	-0.107	-0.37	-0.184	2.31E-10
KIAA1147	0.218	0.2	0.083	-0.328	0.539	2.33E-10
MEGF9	0.052	0.253	-0.043	-0.064	0.377	2.33E-10
RP11-488L1[illegible]	-0.361	0.082	0.28	-0.789	-0.328	2.34E-10
ADCK5	-0.565	-0.032	-0.138	-0.739	-0.705	2.36E-10
EIF3LP2	0.314	0.047	0.154	0.514	0.707	2.37E-10
ERCC6L	0.051	-0.252	-0.292	0.11	-0.621	2.37E-10
ZNF276	-0.159	-0.038	0.255	-0.397	-0.484	2.38E-10
RP11-386C2	-0.715	-0.641	-0.565	-0.622	-1.028	2.41E-10
ZNF783	-0.427	-0.111	0.093	-0.363	-0.544	2.42E-10
ZNF528	-0.235	0.081	0.304	0.126	0.59	2.46E-10
CYB5D2	0.12	0.135	0.258	0.543	0.315	2.46E-10
PHGDH	0.062	-0.015	0.119	0.706	0.739	2.47E-10
DUSP14	0.146	0.268	0.324	0.504	0.579	2.47E-10
CTBP2	0.14	-0.009	0.131	0.306	0.509	2.47E-10
MIRLET7BH[illegible]	-0.611	-0.278	-0.395	-0.962	-1.009	2.48E-10

RP11-186B7	-0.111	-0.052	-0.088	-0.562	-0.607	2.50E-10
MOB1B	0.31	0.326	0.096	0.033	0.385	2.51E-10
CLCN6	-0.325	-0.043	-0.07	-0.685	-0.313	2.51E-10
EML3	-0.121	-0.018	0.017	-0.267	-0.465	2.52E-10
PKDCC	-0.28	-0.217	-0.582	-1.144	-1.083	2.52E-10
ACOT9	0.146	0.144	0.246	0.203	0.384	2.56E-10
TARS2	-0.047	0.094	0.263	0.393	-0.162	2.59E-10
FPGT	-0.515	-0.092	-0.066	-0.193	-0.684	2.61E-10
HAS3	-0.287	0.111	-0.12	-0.508	-0.916	2.66E-10
PHF7	-0.457	0.066	0.235	0.168	-0.69	2.67E-10
BCLAF1	0.432	0.136	-0.018	0.18	-0.074	2.69E-10
CCDC59	0.022	0.017	-0.223	-0.373	-0.571	2.70E-10
CHID1	0.063	-0.012	0.023	0.36	0.472	2.73E-10
VSIG2	-0.962	-0.328	0.25	-0.894	-0.406	2.74E-10
PRICKLE4	-0.37	0.663	0.892	-0.084	0.177	2.75E-10
TAOK3	0.187	0.125	0.224	0.448	0.529	2.78E-10
EDN1	-0.164	-0.061	-0.113	-0.213	-0.541	2.81E-10
EIF6	0.248	0.063	0.075	0.385	0.503	2.83E-10
ST13P5	0.106	-0.043	-0.018	0.329	0.637	2.85E-10
BCR	0.42	-0.155	-0.085	0.212	0.166	2.88E-10
UBE2SP2	-0.313	-0.687	-0.955	-0.84	-0.797	2.88E-10
HNRNPLL	0.06	-0.031	-0.169	0.113	0.414	2.89E-10
RP11-253E3	0.247	-0.366	0.073	-0.283	-0.083	2.90E-10
ATAD2B	-0.485	0.041	-0.172	-0.534	-0.462	2.92E-10
STK39	0.395	0.004	-0.327	0.162	0.254	2.95E-10
PTPN21	0.328	0.162	0.183	0.166	0.441	2.97E-10
EHMT2	-0.408	-0.244	-0.119	-0.026	0.071	2.97E-10
USP20	-0.393	0.119	0.224	0.007	0.066	3.02E-10
LY6K	0.099	0.033	-0.017	0.05	-0.422	3.05E-10
RP11-568K1	-0.132	-0.212	-0.006	0.162	1.007	3.06E-10
PACSIN2	0.38	-0.056	-0.187	0.071	0.494	3.06E-10
TMEM44	-0.21	-0.179	0.253	0.01	0.425	3.10E-10
SHISA5	0.07	-0.025	0	0.339	0.423	3.11E-10
TRIAP1	0.342	0.581	0.613	0.401	0.415	3.11E-10
HSPA14	0.01	0.001	-0.203	-0.464	-0.803	3.12E-10
MPC1	-0.304	-0.165	-0.158	-0.494	-0.837	3.12E-10
ARGLU1	-0.36	0.005	-0.029	-0.896	-0.502	3.14E-10
PRR22	-0.154	0.385	-0.087	-0.782	-1.034	3.14E-10

RHOBTB3	0.367	-0.021	-0.222	-0.051	0.106	3.15E-10
RIN1	-0.77	-0.603	-0.155	-0.288	-0.623	3.17E-10
TK2	0.01	-0.179	0.089	0.21	0.357	3.23E-10
METTL23	-0.628	-0.223	0.079	0.043	-0.106	3.23E-10
ME3	-0.154	-0.049	0.023	-0.456	0.224	3.29E-10
WWP1	0.417	0.076	-0.062	0.056	0.36	3.37E-10
IGF2BP1	0.114	-0.151	-0.116	-0.222	-0.334	3.37E-10
ATF6	0.266	0.157	0.02	0.411	0.501	3.40E-10
ALDH1A2	0.207	0.017	0.153	0.51	0.614	3.44E-10
AP1M1	0.194	0.002	0.082	0.483	0.423	3.54E-10
LPIN3	-0.545	0.145	0.357	-0.312	-0.046	3.54E-10
PPT1	0.212	0.105	0.024	0.438	0.222	3.55E-10
SPHK2	-0.49	0.03	-0.001	-0.06	-0.489	3.55E-10
DNMT3A	-0.166	0.131	0.212	-0.019	0.354	3.57E-10
EDRF1	0.003	0.053	-0.002	-0.406	-0.404	3.57E-10
XIAP	0.416	0.078	0.179	0.34	0.515	3.58E-10
NEDD4	0.289	0.142	-0.051	0.158	0.432	3.59E-10
SMC4	-0.163	-0.321	-0.252	-0.111	-0.394	3.60E-10
IMP3	-0.33	-0.248	-0.074	0.181	0.175	3.61E-10
ARSA	-0.258	-0.091	0.035	-0.016	0.335	3.64E-10
GATAD2A	0.4	-0.136	-0.129	0.1	0.226	3.64E-10
DNASE2	0.204	0.069	0.028	0.504	0.506	3.66E-10
TMEM127	0.216	0.193	0.034	0.248	0.543	3.70E-10
PPARD	0.138	-0.082	0.107	0.485	0.24	3.84E-10
HIPK3	0.384	0.205	0.162	-0.007	0.25	3.85E-10
ASTE1	0.22	0.077	-0.227	-0.133	-0.62	3.87E-10
TM4SF18	-0.487	-0.257	-0.091	-0.092	0.218	3.92E-10
IFI16	0.203	0.087	0.071	0.478	0.543	3.93E-10
C16orf62	0.391	0.131	0.061	0.43	0.595	3.94E-10
PRDX6	0.175	0.047	0.061	0.261	0.411	3.96E-10
CSNK2A2	0.15	0.118	0.05	0.281	0.458	3.98E-10
RP11-87M1.	0.22	0.099	0.077	0.326	0.384	4.00E-10
PEAK1	0.065	0.094	0.301	-0.07	0.433	4.01E-10
GNB5	0.161	0.035	0.106	0.281	0.603	4.05E-10
BRI3	0.01	-0.15	0.023	0.102	0.35	4.06E-10
CYB561D1	-0.237	0	-0.004	-0.352	-0.448	4.11E-10
SH3GLB2	-0.52	-0.056	0.153	-0.195	0.054	4.14E-10
DHFRP1	0.105	0.147	-0.016	0.067	-0.338	4.14E-10

STRA13	-0.434	-0.272	-0.197	-0.483	-0.716	4.23E-10
APBA1	0.012	-0.326	-0.487	-0.33	-0.799	4.25E-10
SCAND2	-0.324	0.141	0.359	-0.334	-0.41	4.29E-10
CLNS1A	0.222	0.099	0.05	0.301	0.337	4.30E-10
SIAH1	0.487	0.093	-0.075	0.081	0.384	4.33E-10
RP11-274B2	-0.065	0.337	0.072	-0.922	-0.622	4.34E-10
IGF2R	0.159	0.163	0.135	0.482	0.664	4.43E-10
BRD8	-0.477	-0.171	0.017	-0.3	-0.341	4.44E-10
TRIP4	0.336	0.235	0.262	0.585	0.379	4.45E-10
EPC2	0.56	0.27	0.208	0.219	0.293	4.48E-10
RP11-958N2	-0.523	-0.131	-0.117	-0.937	-0.772	4.56E-10
PGBD4	-0.385	0.034	-0.106	-0.492	-0.8	4.68E-10
LRRC3	0.256	0.408	0.127	-0.044	0.574	4.69E-10
OS9	0.202	0.123	0.061	0.403	0.594	4.71E-10
MRPL24	0.141	-0.11	-0.173	-0.013	-0.361	4.72E-10
FKBP9	0.279	0.095	-0.01	0.342	0.542	4.78E-10
HGSNAT	0.236	0.155	0.098	0.301	0.411	4.81E-10
FLVCR1	0.013	-0.106	-0.291	-0.555	-0.424	4.83E-10
RP11-2B6.2	-0.593	0.089	-0.232	-1.057	-1.014	4.84E-10
PIBF1	-0.013	0.053	0.13	0.456	0.489	4.87E-10
RP5-1057J7.	-0.353	-0.016	0.154	-1.043	-0.816	4.88E-10
C3orf70	0.455	0.033	-0.209	-0.344	-0.638	4.92E-10
AC004945.2	-0.208	0.047	0.041	-0.254	-0.489	4.95E-10
LAG3	-0.311	-0.354	-0.307	-1.081	-1.342	4.96E-10
DGKH	-0.031	0.243	0.264	-0.443	-0.019	5.00E-10
NIPAL2	-0.204	-0.208	0.042	-0.126	0.535	5.09E-10
B3GALNT2	0.035	0.049	-0.015	0.018	0.451	5.13E-10
F8A1	0.025	0.115	0.311	0.78	0.829	5.14E-10
ZBTB10	0.409	0.247	-0.09	0.138	0.459	5.15E-10
SLC19A2	0.37	0.075	-0.024	-0.181	-0.163	5.16E-10
MORF4L1	0.267	0.05	0.027	0.307	0.328	5.18E-10
SNORD2	-0.153	0.041	0.066	-1.007	-1.117	5.19E-10
HSPB8	0.257	0.458	0.287	0.463	-0.229	5.20E-10
ZNF397	-0.661	-0.065	0.028	-0.414	-0.647	5.20E-10
ZFYVE28	-0.308	-0.234	0.53	0.419	-0.378	5.36E-10
KIF24	0.02	0.06	-0.195	-0.092	-0.726	5.36E-10
CDC42SE1	-0.031	0.114	0.07	-0.447	-0.103	5.51E-10
CLPB	0.161	0.182	0.144	0.467	-0.2	5.51E-10

FBXL5	-0.397	-0.055	0.002	-0.039	-0.2	5.54E-10
WDTC1	0.029	0.08	0.12	0.388	0.42	5.55E-10
RP11-469E1	0.365	-0.127	-0.224	-0.08	0.239	5.55E-10
NFAT5	-0.343	0.081	0.215	-0.435	0.09	5.61E-10
SNRPA	0.246	-0.109	-0.288	0.026	-0.365	5.63E-10
PPP2R5E	0.136	0.113	0.04	0.309	0.562	5.68E-10
CDKL5	-0.287	0.713	0.432	0.401	0.41	5.80E-10
TCEAL4	0.146	0.08	0.033	0.249	0.353	5.82E-10
C1orf216	-0.105	-0.144	-0.212	-0.259	-0.395	5.83E-10
IFFO1	-0.881	-0.398	-0.155	-0.634	-0.274	5.84E-10
ERLIN2	0.035	0.011	0.107	0.421	0.21	5.85E-10
STAG1	0.166	-0.175	-0.253	0.066	-0.36	5.85E-10
EMILIN2	-0.332	0.285	0.44	0.117	-0.281	6.01E-10
PPP5C	0.157	-0.083	-0.22	0.057	-0.483	6.02E-10
KMT2D	-0.118	-0.032	-0.102	-0.479	-0.472	6.03E-10
UAP1L1	-0.02	-0.057	-0.023	-0.323	-0.489	6.10E-10
TMEM231	-0.171	-0.421	-0.44	-0.263	0.363	6.12E-10
LRR1	-0.173	-0.185	-0.23	-0.244	-0.798	6.18E-10
CTC-308K20	-0.569	0.533	0.84	0.135	0.003	6.29E-10
SEC23A	0.468	0.135	-0.057	0.303	0.464	6.36E-10
GALNS	0.177	0.071	0.069	0.411	0.634	6.37E-10
SNX1	0.128	0.126	0.097	0.286	0.354	6.41E-10
PSAT1	0.174	0.017	0.248	0.712	0.454	6.43E-10
CAPN5	0.34	-0.269	-0.402	-0.208	-0.078	6.43E-10
ZBTB34	0.398	0.193	0.036	-0.373	-0.073	6.59E-10
SNORA16A	-0.23	0.083	0.119	-0.66	-1.172	6.59E-10
TBKBP1	-0.5	-0.101	0.153	0.014	-0.136	6.62E-10
OAS1	-0.157	-0.386	0.231	0.482	0.979	6.66E-10
RP1-34B21.6	-0.625	0.079	0.225	-0.646	-0.558	6.73E-10
PCSK5	0.201	0.172	-0.045	0.175	0.584	6.75E-10
MRPL45	-0.056	0.107	0.121	0.293	0.4	6.75E-10
RP11-1100L3	-0.021	-0.198	0.053	0.193	0.815	6.86E-10
EIF3D	0.399	0.178	0.179	0.536	0.54	6.88E-10
TNRC18	-0.043	0.048	0.136	0.213	0.492	6.89E-10
ARHGEF19	0.099	0.039	-0.094	-0.41	-0.596	6.95E-10
GOT1	0.211	0.002	0.053	0.547	0.402	6.99E-10
TMEM208	-0.073	-0.181	-0.228	-0.29	-0.619	7.15E-10
RP11-58E21	0.396	0.108	0.244	0.892	1.195	7.16E-10

TP53BP1	-0.071	0.09	0.212	0.278	0.373	7.25E-10
ZNF841	-0.212	0.067	0.114	-0.688	-0.464	7.38E-10
HYAL2	0.055	-0.203	-0.391	-0.026	-0.316	7.46E-10
RP11-66N24	-0.552	0.109	0.44	-0.512	0.126	7.52E-10
LYSMD1	-0.098	0.235	0.041	-0.389	-0.716	7.55E-10
TRIM39	0.25	0.392	0.316	-0.078	-0.094	7.60E-10
FRS2	0.521	0.382	0.409	0.119	0.344	7.61E-10
RP5-1099D1	0.292	0.062	-0.187	-0.082	-0.655	7.65E-10
TAF1B	0.089	0.012	0.199	0.442	0.358	7.70E-10
RP11-309I15	0.368	0.142	0.203	0.441	0.586	7.72E-10
SLC8A1	0.049	-0.317	0.046	0.146	0.698	7.75E-10
CARM1	0.155	-0.076	-0.135	0.151	0.482	7.77E-10
TMEM47	0.254	0.342	-0.136	-0.143	-0.022	7.94E-10
EXOG	-0.185	0.002	-0.112	-0.375	-0.459	7.95E-10
AC073130.3	-1.19	-0.459	-0.019	-0.488	0.192	7.99E-10
SUCLG2	0.363	0.148	0.012	0.368	0.455	8.14E-10
DUSP22	-0.196	0.061	0.222	0.237	0.421	8.20E-10
RP11-564D1	-1.093	-0.096	0.299	-0.045	0.269	8.20E-10
RP11-479G2	0.419	0.147	0.024	0.443	0.73	8.25E-10
MRPS34	0.134	-0.061	-0.066	0.262	0.378	8.38E-10
IARS2	0.174	0.025	-0.045	0.298	0.335	8.53E-10
SNORA32	-0.348	0.197	0.062	-0.796	-1.006	8.61E-10
OTUD5	0.129	0.174	0.095	0.21	0.552	8.69E-10
RASGRP3	0.081	-0.12	-0.218	-0.657	0.012	8.73E-10
CACUL1	0.044	0.104	0.084	0.102	0.459	8.80E-10
DDOST	0.163	0.021	-0.042	0.316	0.386	8.81E-10
CEP131	-0.43	-0.119	-0.208	-0.554	-0.528	8.81E-10
DYRK2	0.409	-0.033	-0.139	-0.225	0.052	8.87E-10
ANKRD52	-0.224	-0.201	-0.254	-0.367	-0.428	8.99E-10
RP1-278C19	-0.521	-0.534	-0.485	-0.839	-1.481	9.06E-10
MRAS	0.29	-0.103	-0.245	-0.061	0.761	9.07E-10
COPS7A	0.173	0.02	-0.005	0.168	-0.407	9.10E-10
SMARCAL1	-0.104	-0.107	0.176	0.551	0.351	9.14E-10
GBA2	-0.485	-0.273	0.167	-0.376	0.005	9.16E-10
MPC2	-0.154	-0.067	-0.163	-0.37	-0.598	9.16E-10
RP1-102E24	-0.444	0.274	0.3	-0.775	-0.783	9.23E-10
LRCH1	0.285	0.043	-0.033	0.007	0.381	9.24E-10
C15orf54	-0.496	-0.051	-0.082	-0.207	-0.134	9.25E-10

HABP4	0.389	-0.132	-0.358	-0.129	-0.043	9.26E-10
RAB3GAP1	0.283	0.329	0.216	0.477	0.619	9.33E-10
TTC13	-0.425	-0.016	0.09	-0.212	-0.286	9.33E-10
RPL13	-0.132	0.016	0.05	-0.043	0.436	9.51E-10
RP11-539G1	0.462	0.342	-0.202	-0.133	-0.822	9.55E-10
HOXB9	-0.045	0.004	-0.139	-0.048	0.837	9.59E-10
TOX4	0.37	0.267	0.225	0.5	0.532	9.62E-10
RP11-159F2	0.184	0.273	0.283	0.38	0.644	9.71E-10
MAPK6	0.339	0.093	0.031	0.144	0.343	9.90E-10
RNF187	0.217	-0.092	-0.114	0.289	0.475	9.95E-10
PACRGL	-0.221	0.039	-0.086	-0.142	-0.577	9.96E-10
RP1-274L14	0.381	-0.176	-0.361	0.137	0.014	1.01E-09
MED30	-0.165	-0.134	-0.346	-0.401	-0.791	1.01E-09
RBBP4	0.357	0.093	-0.078	0.055	0.118	1.02E-09
STEAP1B	-0.18	-0.384	-0.239	0.034	-0.264	1.02E-09
TNFRSF12A	0.075	-0.076	-0.105	-0.197	-0.337	1.02E-09
VAMP1	0.144	0.264	0.328	-0.395	-0.486	1.02E-09
RRP9	0.164	-0.155	-0.361	-0.081	-0.614	1.02E-09
CDK5RAP2	-0.221	-0.144	-0.045	0.119	-0.399	1.05E-09
CTD-3092A1	-0.509	0.148	0.146	-0.36	-0.37	1.06E-09
RP11-159D1	-0.077	-0.061	0.266	-0.849	-0.467	1.06E-09
PRPF39	-0.176	0.112	-0.023	-0.767	-0.401	1.08E-09
SASS6	-0.237	0.059	-0.267	-0.194	-0.614	1.08E-09
RBPMS	0.067	-0.006	-0.173	-0.076	0.416	1.09E-09
IFNAR2	0.22	-0.043	0.013	0.023	0.405	1.09E-09
CDKN2A	-0.111	-0.098	-0.141	-0.044	0.675	1.10E-09
PARGP1	-0.616	-0.117	-0.093	-0.99	-0.593	1.10E-09
TULP4	0.057	0.082	-0.064	-0.042	0.479	1.11E-09
DUSP18	-0.183	0.238	0.168	0.261	0.45	1.11E-09
SLC16A13	-0.727	0.024	0.037	0.05	-0.161	1.11E-09
FAM195B	-0.026	-0.085	-0.114	-0.048	-0.41	1.11E-09
FBXL18	-0.001	0.273	0.364	0.519	0.7	1.13E-09
WDR24	-0.191	0.327	0.174	0.021	-0.432	1.13E-09
SH3KBP1	0.434	-0.058	-0.407	-0.116	-0.419	1.14E-09
DDI2	-0.05	-0.06	-0.021	0.125	0.382	1.15E-09
PICALM	0.562	0.212	-0.012	0.188	0.245	1.15E-09
PCED1A	-0.421	0.138	0.208	-0.379	-0.294	1.16E-09
HES1	0.026	0.061	0.706	0.57	0.977	1.17E-09

PSPC1	0.202	-0.1	-0.236	0.161	0.341	1.17E-09
ORC5	-0.399	-0.215	-0.205	0.003	-0.41	1.17E-09
ZNF300	-0.226	0.348	0.35	-0.486	-0.495	1.21E-09
GPR143	0.514	0.263	0.439	0.551	1.469	1.23E-09
AMOTL1	0.3	0.132	0.061	0.324	0.565	1.23E-09
PAPOLA	0.348	0.133	0.086	0.298	0.43	1.23E-09
CTC-205M6.	-0.801	-0.297	-0.078	0.02	0.344	1.24E-09
DOPEY1	-0.512	-0.015	-0.222	-0.369	-0.516	1.24E-09
TBC1D1	0.206	0.115	0.072	0.303	0.392	1.26E-09
ZNF302	-0.488	0.069	0.231	-0.074	-0.131	1.26E-09
ENTHD2	-0.24	0.227	0.183	-0.427	-0.3	1.27E-09
TFAP4	-0.337	-0.35	-0.229	0.081	-0.891	1.27E-09
AC133644.2	0.208	0.561	0.16	0.597	1.762	1.28E-09
CCDC127	-0.376	-0.18	-0.015	0.062	-0.048	1.28E-09
ANKRD23	-0.748	-0.038	-0.035	-1.098	-1.027	1.29E-09
RP11-34P13	-0.317	-0.004	-0.061	-0.418	-0.638	1.30E-09
MSTO1	-0.129	-0.175	-0.087	-0.186	-0.674	1.31E-09
MGRN1	0.237	0.488	0.27	0.229	0.545	1.33E-09
MRC1	0.188	-0.048	0.146	0.507	0.987	1.34E-09
ORAI1	0.055	-0.207	-0.182	0.325	0.326	1.34E-09
RP11-27M15	-0.451	-0.044	0.1	-1.184	-0.409	1.36E-09
CYFIP1	0.376	0.206	0.165	0.531	0.747	1.37E-09
EME2	-0.695	0.041	0.129	-0.708	-0.665	1.37E-09
STK36	-0.665	-0.143	0.082	-0.424	-0.291	1.39E-09
SYNGR3	0.157	0.229	-0.135	0.032	1.051	1.41E-09
RP4-794H19	-0.296	-0.262	0.396	0.537	1.136	1.44E-09
COPS6	0.111	-0.036	-0.04	0.292	0.33	1.45E-09
CA12	0.256	-0.021	-0.177	-0.065	-0.889	1.46E-09
CD27-AS1	-0.554	-0.149	0.256	-0.121	-0.279	1.49E-09
ZFP62	-0.423	0.152	0.066	-0.226	-0.3	1.49E-09
PIK3CA	0.2	0.097	-0.079	0.071	0.421	1.50E-09
RP1-224A6.4	-0.357	-0.258	-0.172	-0.556	-0.864	1.52E-09
GLMP	0.011	0.008	0.011	0.234	0.344	1.53E-09
TMEM2	0.339	0.278	-0.165	-0.099	-0.158	1.53E-09
C1orf27	-0.073	0.076	-0.076	-0.426	-0.604	1.53E-09
SYTL3	0.572	-0.174	0.328	0.409	1.059	1.54E-09
PDE4A	0.353	-0.02	0.067	0.164	-0.294	1.54E-09
FRA10AC1	-0.011	0.192	0.303	0.408	0.021	1.55E-09

TRABD	-0.427	-0.147	-0.127	-0.389	-0.426	1.55E-09
SGK494	-0.872	-0.027	0.134	-0.696	-0.627	1.55E-09
C7orf61	-0.723	-0.687	-0.087	0.145	0.961	1.56E-09
TROVE2	0.4	0.274	0.051	0.138	0.231	1.56E-09
C16orf87	0.54	0.099	-0.108	0.003	0.045	1.57E-09
TLE3	0.383	-0.045	-0.063	-0.023	-0.249	1.57E-09
ZNF266	-0.316	0.206	0.169	-0.505	-0.375	1.59E-09
LONRF1	0.125	0.305	-0.115	-0.622	-0.308	1.60E-09
INPP5E	-0.278	-0.081	-0.11	-0.629	-0.317	1.60E-09
TTC17	0.011	0.145	0.147	0.097	0.342	1.61E-09
ABTB1	-0.348	0.285	0.267	-0.026	0.358	1.65E-09
IPO8	0.089	0.092	0.147	0.48	0.207	1.66E-09
NRBP2	-0.535	-0.022	0.116	-0.546	0.033	1.66E-09
RP13-104F2	0.268	0.372	0.245	-0.612	-0.311	1.67E-09
RNF214	-0.544	-0.139	-0.021	-0.072	-0.201	1.68E-09
RP11-752G1	-0.549	0.008	-0.016	-0.982	-0.986	1.68E-09
HDHD3	-0.326	0.341	0.134	-0.169	-0.419	1.69E-09
ACKR4	-0.026	0.82	0.805	1.012	1.344	1.72E-09
SCPEP1	0.129	0.185	0.053	0.414	0.429	1.72E-09
CDV3	0.132	0.123	0.139	0.382	0.497	1.75E-09
SAMD14	-0.15	0.417	0.272	0.042	0.39	1.77E-09
LRRC71	-0.44	-0.05	1.112	0.617	0.367	1.78E-09
FKBP7	0.03	-0.082	-0.178	-0.038	0.359	1.79E-09
TTC1	0.198	0.103	0.171	0.434	0.238	1.83E-09
SMCO4	0.149	-0.181	-0.386	-0.478	-0.589	1.85E-09
RP11-1055B	-0.159	0.106	-0.346	-1.148	-1.004	1.85E-09
CNOT6	0.229	0.254	0.108	0.182	0.489	1.86E-09
PDCL3	0.331	0.116	-0.079	0.186	-0.283	1.87E-09
SLC25A35	-0.525	-0.096	-0.111	-0.368	-0.687	1.88E-09
SNORD104	-0.276	0.199	0.293	-0.897	-0.954	1.89E-09
PSMD11	0.337	0.141	-0.069	0.173	-0.279	1.90E-09
TRMT1	-0.186	0.052	0.126	-0.264	-0.405	1.90E-09
DCP2	-0.406	-0.147	-0.073	-0.298	-0.339	1.92E-09
DIS3	0.503	0.286	0.096	0.265	0.378	2.00E-09
ZNF767	-0.311	0.066	0.334	-0.664	-0.274	2.01E-09
MED13	0.439	0.193	-0.003	0.04	0.223	2.02E-09
THEM4	0.019	-0.269	-0.21	-0.438	-0.378	2.02E-09
MARVELD2	0.373	0.367	-0.13	-0.207	-0.595	2.02E-09

HSD17B14	-0.327	-0.015	-0.086	0.285	0.538	2.04E-09
BCAP31	0.183	0.002	-0.028	0.365	0.422	2.04E-09
RP11-686G2	-0.777	-1.093	-1.158	-0.972	-0.329	2.04E-09
PEX3	-0.004	-0.097	-0.229	-0.065	-0.502	2.05E-09
OSBPL7	-0.564	-0.088	0.351	-0.293	-0.159	2.08E-09
FBXO45	0.33	0.046	-0.242	-0.076	-0.213	2.08E-09
RP11-599J14	-0.366	0.463	0.325	-0.446	-1.018	2.09E-09
SLC5A3	-0.465	0.034	-0.284	-0.63	-0.204	2.10E-09
RP11-473M2	-0.415	0.219	0.115	-0.268	-0.444	2.10E-09
RNASEH1	0.149	-0.023	-0.113	-0.094	-0.45	2.12E-09
CTD-2619J1	-0.55	0.191	0.036	-0.196	-0.433	2.13E-09
INTS2	-0.182	0.162	0.09	-0.005	-0.362	2.16E-09
PCDHGB5	0.348	0.07	-0.081	0.085	0.704	2.18E-09
HLA-C	0.199	0.135	0.015	0.401	0.435	2.18E-09
GCLC	0.403	0.137	-0.093	0.047	-0.073	2.18E-09
ING5	-0.065	-0.003	-0.039	-0.271	-0.471	2.18E-09
THUMPD1	0.338	0.18	0.005	0.133	0.038	2.19E-09
ZNF134	0.384	0.259	0.218	0.335	0.597	2.20E-09
RP4-717I23.3	-0.431	0.051	0.407	-0.434	-0.501	2.24E-09
IDUA	-0.464	-0.069	0.149	-0.428	0.35	2.26E-09
RP11-478B9	-0.5	-0.985	-0.775	-0.275	-0.772	2.27E-09
KIF1BP	0.092	0.091	0.075	0.446	0.501	2.29E-09
COMMD8	-0.356	-0.221	-0.186	-0.577	-0.98	2.31E-09
RP11-631N1	-0.012	-0.026	0.556	-0.457	-0.324	2.33E-09
HES4	-0.561	-0.079	-0.273	-0.776	0.687	2.34E-09
GLDC	-0.097	-0.051	0.207	0.535	0.73	2.35E-09
ANKZF1	-0.543	0.153	0.337	-0.237	-0.171	2.35E-09
MPP5	0.463	0.168	0.106	0.416	0.366	2.36E-09
LANCL2	0.478	0.053	-0.164	0.164	-0.042	2.36E-09
SRBD1	0.19	0.023	0.166	0.516	-0.224	2.37E-09
PHKA2	-0.33	-0.022	-0.063	-0.437	-0.523	2.38E-09
TNKS2	0.105	0.163	0.076	0.154	0.391	2.40E-09
ATXN7L1	-0.527	-0.103	0.182	0.062	0.037	2.40E-09
STT3B	0.354	0.146	-0.064	0.182	-0.245	2.40E-09
GPATCH1	0.24	0.245	0.102	0.103	-0.403	2.41E-09
RP11-446H1	-0.573	0.072	0.047	-0.029	-0.527	2.42E-09
SETMAR	0.144	-0.048	-0.287	-0.376	-0.565	2.43E-09
CCDC94	0.539	0.083	-0.023	0.445	0.074	2.44E-09

GABPB1-AS	-0.427	0.008	0.432	-0.687	-0.255	2.44E-09
LRRC8A	0.409	-0.104	-0.049	0.167	0.178	2.46E-09
AMPD2	-0.439	-0.202	-0.091	-0.352	-0.363	2.47E-09
AC000123.2	-0.362	0.027	-0.08	-0.947	-0.483	2.47E-09
TAF6L	-0.116	-0.073	-0.229	-0.252	-0.63	2.47E-09
DNHD1	-0.395	0.263	0.479	-0.485	-0.238	2.48E-09
KDM4A	-0.044	0.211	0.224	0.392	0.438	2.49E-09
EPB41	0.212	0.101	0.336	0.444	0.515	2.50E-09
TMEM50A	0.134	0.072	-0.014	0.197	0.367	2.50E-09
SNORD88B	-0.117	-0.295	-0.241	-1.158	-1.353	2.50E-09
RP11-134N1	0.159	0.115	0.028	0.569	0.867	2.52E-09
UNC119	-0.445	-0.145	-0.091	-0.631	-0.147	2.53E-09
ANO10	0.279	0.075	0.042	0.407	0.56	2.54E-09
RICTOR	-0.099	0.351	0.185	-0.204	0.126	2.54E-09
DOCK5	0.066	0.066	0.421	0.079	0.42	2.58E-09
ABC7-42389	-0.42	0.146	0.054	-0.676	-0.618	2.58E-09
RP11-415I12	0.078	-0.014	0.079	0.191	0.446	2.59E-09
ZEB1	-0.403	-0.249	0.169	-0.122	0.308	2.59E-09
KIAA0513	0.147	0.155	-0.052	-0.008	-0.773	2.59E-09
BCAS3	0.673	0.17	0.14	0.626	0.877	2.62E-09
TTLL3	-0.522	-0.133	0.202	-0.714	-0.347	2.63E-09
RP11-156P1	-0.221	-0.079	0.117	0.228	0.688	2.64E-09
PHAX	0.224	0.133	0.078	0.445	0.601	2.64E-09
SGSM2	-0.472	-0.082	0.005	-0.662	-0.149	2.65E-09
RNF217	0.575	0.283	0.388	0.193	0.525	2.69E-09
MAFK	0.529	0.218	0.37	0.052	0.45	2.72E-09
PAK3	-0.191	0.106	0.543	0.393	1.094	2.77E-09
ZC3H12A	0.091	0.235	-0.069	-0.592	0.064	2.78E-09
ABT1	0.003	-0.102	-0.209	-0.084	-0.389	2.80E-09
GNG5P2	0.018	-0.238	-0.199	-0.341	-0.584	2.81E-09
RP3-510D11	-0.085	0.625	0.763	0.562	1.068	2.82E-09
FBXL20	0.265	0.06	0.027	-0.138	0.48	2.83E-09
GGNBP2	0.405	0.035	0.086	0.159	0.105	2.83E-09
SIX4	0.142	0.023	-0.257	-0.095	0.582	2.85E-09
MEG9	-0.26	0.142	0.243	-0.871	-0.445	2.85E-09
PTX3	-0.379	-0.117	-0.305	-0.003	-0.494	2.85E-09
GLYR1	0.222	-0.004	0.046	0.214	0.415	2.88E-09
SNX18	0.346	0.076	0.027	0.453	0.361	2.88E-09

NUDT5	0.076	-0.004	0.074	0.144	0.334	2.88E-09
CPNE2	0.138	-0.026	-0.011	0.308	0.454	2.89E-09
MLX	-0.369	-0.207	-0.081	-0.134	-0.453	2.92E-09
TMEM39B	0.146	-0.311	-0.245	-0.06	-0.351	2.93E-09
RP11-467J12	-0.016	0.701	1.28	0.591	0.788	2.95E-09
ANP32A	0.245	-0.208	-0.38	-0.028	-0.258	2.96E-09
NANP	0.453	0.017	-0.058	-0.061	-0.15	2.98E-09
C10orf25	-0.38	-0.248	0.083	0.495	0.736	2.99E-09
NCAPH	0.006	-0.315	-0.394	0.085	-0.597	2.99E-09
TMEM204	0.22	-0.352	-0.143	0.244	0.115	3.00E-09
CPD	0.256	0.218	0.06	0.39	0.63	3.03E-09
FUT1	0.111	0.219	-0.046	-0.369	-0.32	3.04E-09
FAM234B	-0.289	0.151	0.122	0.088	-0.396	3.07E-09
RP11-307P2	-0.808	-0.83	-0.749	-0.819	-1.077	3.07E-09
ITGB3	0.498	0.175	-0.076	0.048	0.252	3.08E-09
BAG5	0.345	0.089	0.022	0.171	0.301	3.09E-09
SGK3	0.301	-0.063	-0.365	-0.096	-0.052	3.09E-09
UBALD1	0.135	0.215	0.17	-0.144	0.539	3.11E-09
NUS1	0.382	0.188	0.007	0.099	0.067	3.13E-09
SRGAP2	-0.106	0.153	0.219	0.109	0.398	3.16E-09
NAXD	-0.261	-0.005	-0.09	-0.451	-0.375	3.17E-09
CCDC85B	-0.069	-0.429	-0.459	-0.286	0.206	3.18E-09
KMT5B	0.485	0.204	0.149	0.089	0.181	3.25E-09
MIER3	0.372	0.209	0.011	-0.229	-0.149	3.25E-09
ACBD4	-0.932	-0.094	0.048	-0.31	-0.241	3.26E-09
ZSCAN22	0.268	0.196	0.129	-0.167	-0.474	3.26E-09
FBXO44	-0.415	0.116	0.257	-0.042	0.28	3.27E-09
P3H2	0.458	0.411	0.299	0.551	0.822	3.28E-09
PTCD2	-0.509	-0.081	0.079	-0.193	-0.369	3.32E-09
BICD1	-0.541	-0.043	0.08	-0.138	-0.014	3.33E-09
FH	0.205	0.139	0.052	0.326	-0.239	3.34E-09
GPATCH2	0.089	0.189	-0.137	-0.274	-0.382	3.34E-09
TBCK	-0.143	0.044	0.02	0.161	0.448	3.35E-09
ELFN2	0.524	0.055	0.386	0.72	0.89	3.36E-09
NR1D1	-0.828	0.081	0.294	0.379	0.148	3.36E-09
TMCO4	0.037	-0.124	0.327	0.589	0.773	3.38E-09
RP11-144N1	0.065	-0.506	-0.469	-0.055	-0.963	3.39E-09
MPV17L2	0.118	-0.125	-0.188	-0.069	-0.632	3.41E-09

SMOX	0.574	0.203	-0.036	0.055	0.391	3.44E-09
PDZD8	0.341	0.066	-0.159	-0.032	0.076	3.44E-09
AGO4	0.332	-0.007	0.092	-0.453	-0.133	3.44E-09
RHBDD1	0.204	0.23	0.293	0.508	-0.129	3.46E-09
MTPN	0.351	0.129	0.012	0.238	0.387	3.48E-09
FARS2	0.612	-0.062	-0.102	0.516	0.298	3.48E-09
ABCC4	0.337	0.018	-0.228	-0.028	-0.006	3.48E-09
REPS1	0.318	-0.086	-0.244	0.169	0.348	3.50E-09
ADPRH	0.406	0.283	0.384	0.66	0.975	3.52E-09
SELPLG	-0.263	-0.388	0.409	0.007	1.041	3.53E-09
RUVBL1	0.314	0.1	0.045	0.599	0.468	3.57E-09
SFMBT1	0.53	0.058	-0.332	-0.387	-0.51	3.59E-09
RGS20	-0.047	0.301	0.625	0.407	0.63	3.60E-09
RP11-133L1	0.392	-0.173	-0.236	-0.028	-0.335	3.62E-09
KCTD1	0.049	0.106	0.066	0.373	0.678	3.63E-09
BLVRB	0.05	0.028	0.078	0.475	-0.004	3.63E-09
PSMG3	-0.145	-0.035	-0.141	-0.405	-0.537	3.63E-09
CXorf40A	-0.263	-0.046	-0.183	-0.256	-0.582	3.69E-09
RF00019	-0.567	-0.019	0.066	-1.036	-0.756	3.72E-09
ATL2	-0.296	-0.217	-0.315	-0.457	-0.571	3.73E-09
TEAD4	0.181	-0.153	-0.101	0.065	-0.439	3.82E-09
MZT2A	-0.256	-0.005	0.052	0.058	0.521	3.83E-09
BHLHB9	0.037	-0.148	0.018	0.531	0.476	3.86E-09
CEP78	0.041	0.001	-0.127	-0.022	-0.444	3.92E-09
NAA40	-0.352	-0.016	-0.071	-0.278	-0.269	3.93E-09
THBD	0.17	-0.042	0.205	0.389	0.514	4.02E-09
COPB1	0.363	0.126	0.06	0.405	0.537	4.03E-09
EIF2S1	0.349	0.152	-0.002	0.271	-0.037	4.03E-09
RP11-462L8	0.281	-0.564	-0.168	0.183	1.192	4.14E-09
FAM107A	0.104	0.144	0.225	0.68	0.418	4.14E-09
ZNF789	-0.439	-0.098	-0.012	-0.815	-0.799	4.17E-09
C1orf115	-0.48	0.005	0.212	-0.236	-0.214	4.23E-09
VPS16	-0.054	0.12	0.003	-0.068	-0.351	4.23E-09
ZNF26	0.021	0.085	-0.103	-0.518	-0.387	4.26E-09
FTSJ1	0.152	0.008	-0.001	0.066	-0.333	4.37E-09
TRIO	0.087	0.032	0.132	0.019	0.389	4.38E-09
CCDC66	-0.433	0.005	0.084	-0.355	-0.376	4.39E-09
ZNF691	-0.432	0.145	0.037	-0.344	-0.528	4.44E-09

SYVN1	-0.282	-0.168	0.113	-0.126	-0.326	4.45E-09
TSC22D2	0.283	-0.026	0.018	0.039	0.404	4.46E-09
RP11-231C1	-0.413	-0.165	-0.054	-0.779	-0.547	4.46E-09
RP3-331H24	-0.022	0.131	0.217	-0.654	-0.892	4.49E-09
LDHBP	0.339	-0.084	-0.081	0.356	0.511	4.54E-09
MSANTD2	-0.206	-0.094	-0.036	-0.673	-0.541	4.57E-09
NIN	-0.156	0.05	-0.083	-0.56	-0.193	4.58E-09
RP11-410F1	0.451	0.086	0.095	0.566	0.882	4.63E-09
PRSS3	0.014	-0.118	-0.217	-0.43	-0.019	4.63E-09
AC009948.3	-0.563	0.08	0.202	-0.494	-0.14	4.69E-09
CECR5	0.107	-0.179	-0.253	-0.01	-0.445	4.69E-09
RAPGEF3	-0.331	-0.037	0.323	-0.316	-0.288	4.73E-09
KDM6A	0.354	0.396	0.414	0.196	0.474	4.75E-09
MED31	-0.029	0.11	-0.013	-0.531	-0.929	4.77E-09
VEZF1	0.019	0.154	0.209	0.243	0.435	4.82E-09
GK5	-0.123	0.095	0.085	-0.361	-0.491	4.86E-09
RP11-458F8	-0.18	0.6	0.93	0.156	0.006	4.89E-09
STON1	-0.354	-0.006	-0.155	-0.096	-0.772	4.89E-09
RAPGEF5	0.097	0.054	0.221	0.265	0.422	4.92E-09
CPT1C	-0.625	-0.281	-0.041	-0.307	-0.22	4.94E-09
CCDC167	-0.133	-0.144	-0.027	-0.227	-0.616	4.95E-09
CTA-215D11	-1.018	-0.191	0.296	-0.872	-0.761	4.95E-09
CTNND1	0.313	0.01	-0.028	0.335	0.568	4.99E-09
C12orf4	0.024	0.053	-0.001	-0.049	-0.416	5.01E-09
LGR4	-0.107	-0.305	-0.519	-0.185	0.214	5.05E-09
BRF2	0.356	0.148	-0.058	-0.125	-0.361	5.05E-09
ATP5D	0.019	-0.042	0.038	0.231	0.428	5.07E-09
CHD2	0.204	0.291	0.356	0.009	0.273	5.07E-09
MIR100HG	-0.354	-0.346	-0.211	-0.778	-0.627	5.13E-09
MRPL16	0.073	-0.022	0.019	0.344	-0.057	5.14E-09
USP28	-0.136	-0.058	-0.08	-0.022	0.351	5.18E-09
PHF20	0.008	0.129	0.247	0.361	0.471	5.20E-09
CSRNP1	-0.083	0.167	-0.02	-0.059	0.457	5.21E-09
RP11-480P3	0.258	0.175	0.213	0.532	0.775	5.24E-09
ZNF354A	-0.485	0.269	0.266	-0.078	0.307	5.30E-09
P4HTM	-0.103	-0.009	0.117	0.102	0.458	5.33E-09
ZMAT2	0.308	0.176	0.181	0.481	0.257	5.38E-09
MYO5A	0.378	0.108	0.073	0.301	0.564	5.42E-09

SNORD14E	-0.599	-0.513	-0.381	-1.672	-1.059	5.42E-09
RP5-907D15	-0.137	-0.303	0.049	-0.055	0.659	5.45E-09
NACC2	0.478	0.207	0.233	0.261	0.516	5.46E-09
PARD3	0.441	0.074	0.042	0.281	0.434	5.46E-09
TGOLN2	0.131	0.081	0.007	0.316	0.433	5.48E-09
RP4-800G7.2	-0.874	-0.216	0.147	-0.556	-0.402	5.49E-09
CUL4B	0.16	0.166	0.193	0.494	0.352	5.50E-09
GTF2H4	-0.114	-0.019	0.074	-0.096	-0.411	5.52E-09
MLLT11	0.234	0.17	-0.121	0.112	-0.403	5.53E-09
GSDMB	-0.652	0.249	0.594	-0.41	-0.07	5.54E-09
PI4K2A	0.219	0.093	-0.048	0.282	0.601	5.55E-09
RP11-264B1	-0.183	0.101	0.26	-0.8	-0.575	5.59E-09
SEMA3G	0.457	-0.114	-0.217	-0.304	-0.581	5.67E-09
ZNF655	-0.181	0.05	0.328	-0.247	-0.322	5.68E-09
SAMD5	0.01	0.247	0.251	0.408	0.626	5.69E-09
MAP3K14	-0.144	-0.171	-0.249	-0.702	-0.357	5.73E-09
ANKRD35	-0.682	-0.812	-0.667	-0.605	-1.376	5.73E-09
MOB3C	0.207	0.414	0.154	-0.269	0.139	5.74E-09
RDH11	0.045	-0.115	-0.366	-0.228	-0.16	5.74E-09
PLEKHO2	0.255	0.23	0.074	0.283	0.569	5.83E-09
APEX2	0.085	0.07	-0.23	-0.065	-0.537	5.97E-09
FAN1	-0.211	0.136	0.125	-0.18	-0.376	6.02E-09
CENPM	-0.018	-0.072	-0.166	0.027	-0.686	6.08E-09
C12orf60	-0.784	-0.03	-0.172	-0.627	-1.061	6.13E-09
RNASEL	-0.186	0.231	0.327	0.785	0.888	6.14E-09
RP3-380B8.1	0.289	-0.123	-0.099	0.561	0.773	6.17E-09
TNFRSF25	-0.382	-0.064	0.476	-0.392	0.321	6.18E-09
ATG13	0.399	0.179	0.073	0.323	0.48	6.23E-09
RP5-1050D4	-0.843	-0.122	-0.02	-0.804	-0.861	6.23E-09
PTPN12	0.335	0.059	0.113	0.281	0.267	6.27E-09
PKP4	-0.245	-0.303	-0.328	-0.37	-0.385	6.35E-09
KIF13B	0.394	0.104	0.031	0.12	0.389	6.37E-09
TUSC1	0.505	0.226	-0.172	0.493	0.521	6.44E-09
ZC3HAV1	0.452	0.48	0.473	0.807	0.82	6.47E-09
KIFC2	-0.536	0.148	0.291	-0.625	-0.333	6.47E-09
ZBTB14	-0.257	-0.037	-0.196	-0.494	-0.444	6.50E-09
C11orf84	0.145	-0.022	-0.351	-0.361	-0.195	6.52E-09
CEP112	0.279	0.053	-0.003	0.171	0.623	6.53E-09

RP11-443N2	0.41	0.074	0.018	-0.085	-0.38	6.53E-09
SLC25A15	0.253	0.056	-0.051	0.159	-0.351	6.54E-09
IL13RA1	0.359	0.125	-0.112	0.173	-0.125	6.55E-09
TOMM22	0.028	-0.097	-0.164	-0.198	-0.335	6.62E-09
PEX2	-0.211	-0.311	-0.237	-0.392	-0.837	6.62E-09
VILL	-0.469	0.114	0.146	-0.298	-0.54	6.63E-09
AC004158.2	-0.527	-0.09	-0.11	-0.879	-1.159	6.64E-09
SNORD14C	-0.575	-0.396	-0.354	-1.193	-1.521	6.66E-09
ZDHHC1	-0.127	-0.12	0.063	-0.062	0.679	6.75E-09
C1orf112	-0.278	0.061	-0.012	-0.008	-0.498	6.75E-09
FARP2	0.255	0.026	-0.139	-0.088	0.375	6.97E-09
SNORA52	-0.382	-0.031	-0.275	-1.095	-1.314	7.09E-09
FBXW8	0.262	0.026	0.195	0.233	0.601	7.11E-09
WDR91	0.069	0.162	0.025	-0.258	-0.467	7.11E-09
GNG5	-0.051	-0.094	-0.143	-0.355	-0.429	7.12E-09
CCNL2	-0.454	0.205	0.195	-0.716	-0.548	7.20E-09
EAF2	0.133	-0.109	-0.067	-0.521	-1.294	7.27E-09
AL450992.2	0.039	0.383	0.719	0.975	1.39	7.36E-09
HOXA11	0.214	-0.22	0.059	0.469	-0.076	7.41E-09
ABI1	0.123	0.127	0.094	0.274	0.423	7.43E-09
RP11-736I24	-1.045	-0.65	-0.231	-0.847	-1.033	7.52E-09
DLX1	0.252	-0.597	-0.752	-0.78	-0.656	7.58E-09
CAPN1	0.285	0.104	0.101	0.519	0.709	7.59E-09
MRPL55	-0.164	-0.07	-0.137	-0.114	-0.531	7.68E-09
MMRN2	0.161	-0.048	-0.103	0.369	0.487	7.71E-09
AASDH	-0.4	0.131	0.201	0.049	-0.318	7.74E-09
SATB2	-0.556	0.065	0.042	-0.287	-0.398	7.76E-09
AC012667.1	0.299	-0.042	0.299	0.608	1.157	7.77E-09
MIGA1	-0.019	0.156	0.033	0.051	-0.339	7.77E-09
USP7	0.36	0.099	-0.056	0.049	-0.006	7.79E-09
TIMM17B	0.045	-0.132	-0.074	-0.019	-0.536	7.82E-09
AL162151.3	0.03	0.013	0.099	0.125	0.404	7.87E-09
VCPKMT	-0.128	0.004	-0.076	-0.588	-0.823	7.95E-09
FAM72B	-0.919	-0.531	-0.441	-0.726	-0.984	7.95E-09
SRM	0.175	-0.076	-0.2	-0.109	-0.428	7.97E-09
CCDC82	0.041	0.142	0.036	-0.331	-0.259	8.01E-09
AC125238.2	0.181	-0.217	-0.344	-0.264	-0.441	8.13E-09
RP11-159D1	-0.265	-0.18	0.166	-0.472	-0.611	8.23E-09

NPHP3-ACA	-0.766	0.093	0.584	-0.363	-0.196	8.24E-09
CCDC17	-0.363	0.088	0.049	-0.874	-1.079	8.30E-09
NAP1L1P1	0.251	0.175	0.231	0.447	0.556	8.31E-09
ACAP3	-0.398	-0.13	-0.117	-0.933	-0.429	8.33E-09
XAF1	-0.38	-0.134	0.588	0.037	0.485	8.39E-09
TANC2	0.07	0.034	-0.434	-0.323	-0.002	8.40E-09
CDC34	0.008	0.027	0.068	0.24	0.414	8.46E-09
NUCB1	0.251	0.109	0.041	0.482	0.59	8.73E-09
RFX1	-0.384	-0.059	0.255	0.314	0.494	8.73E-09
NISCH	-0.444	-0.109	-0.032	-0.527	-0.08	8.79E-09
RP11-284E2	0.319	0.008	-0.075	0.255	0.398	8.82E-09
TRPC1	-0.191	0.468	0.311	-0.252	-0.153	8.85E-09
HIC2	0.139	0.076	0.052	-0.507	-0.415	8.86E-09
MARS2	-0.527	-0.266	-0.505	-0.439	-0.844	9.03E-09
WARS	0.31	0.08	0.007	0.387	0.554	9.08E-09
MIR3176	-0.57	-0.121	-0.356	-1.576	-1.001	9.14E-09
CLK1	0.041	0.204	0.227	-0.537	-0.394	9.16E-09
HUS1	-0.04	-0.09	0.009	-0.062	-0.422	9.19E-09
TYSND1	-0.826	-0.595	-0.428	-0.456	-0.364	9.26E-09
RP11-43N16	-0.428	0.145	-0.075	-0.892	-0.892	9.27E-09
POMZP3	0.537	0.346	0.304	0.47	0.127	9.39E-09
MVP	0.297	0.051	0.061	0.498	0.78	9.43E-09
HOXA-AS4	0.151	-0.502	-0.331	-0.273	-0.644	9.43E-09
CTSH	-0.552	-0.508	-0.538	-0.261	0.383	9.54E-09
FAM213B	-0.064	0.009	0.041	0.292	0.349	9.57E-09
DNAAF5	0.135	-0.081	0.055	0.463	0.49	9.58E-09
IP6K2	0.003	0.167	0.197	-0.022	0.407	9.62E-09
INO80E	-0.165	-0.187	-0.114	-0.149	-0.497	9.68E-09
TRAPPC12	0.188	-0.124	0.061	0.333	0.344	9.79E-09
SDCCAG3	-0.273	-0.078	-0.045	-0.311	-0.396	9.89E-09
TTPAL	0.06	0.303	0.281	0.399	0.087	9.93E-09
PDK3	0.241	0.112	-0.089	0.361	0.937	1.01E-08
AC016747.2	-0.7	0.092	0.326	-0.649	-0.426	1.01E-08
TRIML2	0.269	0.609	0.733	0.391	0.627	1.02E-08
SRP72	0.327	0.011	-0.137	0.151	0.048	1.02E-08
MYBL1	0.181	0.185	0.155	0.012	-0.441	1.02E-08
DPP4	0.4	-0.011	-0.063	0.295	0.749	1.03E-08
RCAN3	0.713	0.039	0.217	0.517	0.421	1.04E-08

GNPAT	0.058	0.063	0.132	0.24	0.418	1.04E-08
EYA3	-0.119	-0.055	-0.067	-0.213	-0.336	1.04E-08
LSM2	-0.281	-0.242	-0.086	-0.337	-0.836	1.04E-08
GTF2B	0.457	0.246	0.091	0.037	-0.088	1.05E-08
TP53BP2	0.203	0.255	0.075	0.126	0.481	1.06E-08
VSIG1	0.382	0.402	0.463	0.797	0.227	1.06E-08
KMT5A	0.024	-0.323	-0.242	-0.059	-0.022	1.06E-08
FCN3	0.308	-0.031	-0.264	0.109	-0.763	1.06E-08
KLF2	-0.044	0.29	0.546	0.452	0.382	1.08E-08
CHAC1	-0.212	-0.102	0.53	0.6	0.44	1.10E-08
AHCY	0.34	0.178	0.132	0.465	0.503	1.11E-08
HECTD4	-0.463	-0.035	0.04	-0.347	-0.118	1.11E-08
RP11-467L1:	0.138	-0.027	0.049	0.559	0.565	1.12E-08
CSNK1G3	0.256	0.233	0.115	0.195	0.41	1.13E-08
ERVK3-1	0.079	-0.033	-0.301	-0.504	-0.626	1.15E-08
SNORD35B	-0.694	-0.323	-0.164	-1.209	-1.237	1.15E-08
PRPF38B	-0.213	0.041	0.094	-0.383	-0.426	1.16E-08
MC1R	-0.144	0.193	0.287	-0.495	-0.697	1.16E-08
ICK	-0.142	-0.184	-0.568	-0.288	-0.035	1.17E-08
MAP3K7CL	-0.336	-0.194	-0.063	-0.303	0.581	1.18E-08
FDXACB1	-0.169	-0.157	0.163	0.296	-0.835	1.18E-08
RCBTB2	0.438	0.127	0.097	0.002	0.438	1.19E-08
RBPJ	0.265	0.257	0.169	0.385	0.499	1.20E-08
NICN1	-0.667	-0.197	0.208	-0.217	0.138	1.20E-08
SNORD96A	-0.295	0.055	0.09	-0.748	-0.956	1.20E-08
DECR1	0.052	0.015	-0.007	0.15	0.416	1.21E-08
ZNF251	-0.775	-0.271	0.062	-0.567	-0.068	1.22E-08
PIK3C2A	0.116	0.058	-0.092	-0.097	-0.401	1.23E-08
NCOA4	0.442	0.203	0.119	0.487	0.551	1.24E-08
SLC1A1	0.069	-0.149	-0.377	-0.226	-0.603	1.24E-08
CRYGS	-0.339	-0.087	0.189	-1.063	-0.894	1.25E-08
RP11-706O1	0.362	0.408	0.398	-0.118	0.452	1.26E-08
TRMT44	-0.509	0.091	-0.071	-0.313	-0.315	1.26E-08
SCYL2	-0.015	0.2	0.029	0.15	0.359	1.27E-08
PPP2R3B	-0.566	-0.004	0.176	-0.074	-0.172	1.27E-08
MAD2L1	-0.577	-0.549	-0.569	-0.729	-0.826	1.27E-08
RP11-527N2	-0.167	-0.017	0.245	0.47	1.488	1.28E-08
RP11-793H1	-0.19	0.047	0.142	-0.844	-0.65	1.28E-08

KCTD20	0.211	0.057	0.089	0.365	0.38	1.30E-08
WHAMMP2	-0.485	-0.093	-0.218	-0.451	-0.715	1.30E-08
CTD-2267D1	0.136	0.236	0.328	0.772	0.766	1.31E-08
STK10	0.193	0.112	0.178	0.367	0.616	1.31E-08
MTMR14	0.023	0.267	0.326	0.37	0.409	1.31E-08
VWA8	0.288	0.094	0.256	0.54	0.521	1.32E-08
B3GNTL1	-0.135	-0.17	0.349	0.288	0.5	1.32E-08
NUDT9	0.126	0.046	0.124	0.382	0.391	1.32E-08
FBXL19-AS1	-0.633	0.008	0.294	-0.458	-0.519	1.33E-08
SYAP1	0.297	0.147	0.062	0.443	0.414	1.34E-08
KRAS	0.491	0.163	-0.029	0.204	0.261	1.34E-08
LSM10	-0.383	-0.449	-0.247	-0.237	-0.536	1.34E-08
MLF2	0.204	0.098	0.056	0.339	0.495	1.35E-08
PTP4A3	0.267	-0.165	0.338	0.687	-0.175	1.35E-08
SKA2	-0.042	-0.125	-0.255	-0.177	-0.342	1.35E-08
NUCB2	0.209	0.024	0.014	0.396	0.113	1.36E-08
METAP1D	-0.19	-0.017	0.306	0.087	-0.429	1.37E-08
HMGA1P3	0.13	-0.176	0.066	0.426	0.665	1.39E-08
PGM5P2	-0.87	-0.234	0.582	-0.263	0.093	1.40E-08
RP11-131L2:	0.493	0.764	0.278	0.173	-0.449	1.40E-08
GSG2	-0.006	-0.348	-0.384	-0.086	-0.543	1.41E-08
ZNF444	-0.14	0.111	0.153	0.147	0.531	1.44E-08
GATB	0.257	0.228	0.242	0.602	0.503	1.46E-08
E2F6	0.043	-0.044	-0.215	-0.412	-0.448	1.46E-08
COX17	-0.202	-0.093	-0.012	-0.541	-0.789	1.47E-08
PODXL2	-0.088	0.031	-0.353	-0.132	1.029	1.49E-08
FZR1	-0.36	-0.261	-0.287	-0.202	0.058	1.49E-08
CTC-425O23	-0.845	-0.049	0.463	-0.693	-0.396	1.49E-08
ZNRD1	-0.445	-0.111	-0.089	-0.28	-0.67	1.49E-08
USP22	0.182	-0.054	-0.11	0.146	0.431	1.50E-08
TMEM136	-0.447	-0.03	0.01	-0.638	-0.349	1.50E-08
UBR1	0.073	0.321	0.26	0.423	0.404	1.53E-08
ZNF557	-0.163	0.077	-0.064	-0.359	-0.477	1.54E-08
SUSD5	0.361	0.009	-0.266	0.031	0.104	1.55E-08
TTC31	-0.124	0.012	0.036	-0.228	-0.387	1.55E-08
RP11-693N9	-0.546	0.41	0.286	-0.615	0.27	1.56E-08
AP5M1	0.013	0.132	0.108	0.146	0.342	1.57E-08
PJA2	0.492	0.154	0.185	0.54	0.528	1.58E-08

OLA1	0.058	-0.002	0.022	0.154	0.396	1.58E-08
SLC46A1	-0.548	-0.171	0.017	0.04	0.213	1.58E-08
STRADB	-0.24	-0.007	0.077	-0.002	0.433	1.62E-08
NAB1	-0.08	0.076	0.062	-0.334	-0.14	1.63E-08
TUBB6	0.453	0.23	0.198	0.677	0.465	1.66E-08
DPYD	0.445	0.04	0.053	0.447	0.539	1.67E-08
LTBP4	-0.036	0.181	0.455	0.213	0.326	1.67E-08
BUD13	0.166	-0.047	-0.106	-0.064	-0.405	1.67E-08
AC004166.7	-0.534	0.114	0.007	-0.286	-0.503	1.67E-08
IKZF4	0.069	-0.085	0.015	-0.397	-0.668	1.68E-08
TBCEL	0.247	0.343	0.26	0.247	0.475	1.71E-08
CEP104	-0.05	0.084	0.086	0.212	0.35	1.71E-08
MICALL2	-0.367	0.107	0.166	-0.497	0.198	1.71E-08
S100A11	0.304	0.046	-0.003	0.292	0.386	1.72E-08
RMND1	-0.122	-0.139	-0.009	0.222	0.391	1.75E-08
SUPT20H	-0.365	-0.115	-0.046	-0.218	-0.169	1.75E-08
SNHG7	0.058	-0.037	-0.063	-0.336	-0.339	1.76E-08
MAP2K1	0.316	0.12	0.023	0.418	0.446	1.77E-08
TARBP1	-0.076	-0.134	0.098	0.02	0.412	1.78E-08
SBF2	0.348	-0.031	0.053	0.398	0.36	1.79E-08
TRIM11	-0.132	0.048	-0.133	-0.507	-0.253	1.80E-08
PCMTD2	-0.072	0.307	0.375	0.218	0.355	1.81E-08
CTTNBP2NL	0.17	0.167	0.07	0.228	0.551	1.83E-08
PGS1	-0.364	-0.063	0.126	-0.081	0.206	1.83E-08
INSIG2	-0.414	-0.076	0.014	-0.052	0.042	1.84E-08
GDPD3	-0.439	-0.121	0.286	-0.71	-0.559	1.84E-08
RP11-705B1	0.155	-0.104	0.098	0.357	0.514	1.90E-08
PROSER2	0.615	-0.007	-0.059	0.218	0.422	1.91E-08
ITGA5	0.433	0.029	-0.271	0.044	-0.23	1.92E-08
ZDHHC6	-0.141	0.082	0.078	-0.258	-0.391	1.92E-08
CCDC125	0.052	0.014	0.541	0.705	0.886	1.95E-08
FBRSL1	-0.115	0.043	-0.027	-0.327	0.28	1.95E-08
CPSF1	-0.308	-0.033	-0.05	-0.396	-0.307	1.95E-08
TMEM161B	-0.134	0.204	0.063	-0.437	-0.551	1.95E-08
GSTCD	-0.199	-0.08	-0.004	-0.102	-0.556	1.95E-08
RP5-862P8.2	0.79	0.651	0.315	0.001	-0.004	1.96E-08
PRICKLE1	0.122	-0.641	-0.672	-0.414	-0.521	1.96E-08
NCRNA0010	-0.702	-0.114	0.181	-0.708	-0.357	1.97E-08

UBR4	-0.084	0.129	0.046	-0.385	-0.149	1.99E-08
FUK	-0.684	-0.124	0.015	-0.331	-0.323	1.99E-08
MOCS2	-0.196	0.021	0.119	-0.173	-0.619	1.99E-08
CNST	0.165	0.138	0.299	0.226	0.526	2.00E-08
ATP6V1G1	-0.168	-0.088	-0.161	-0.542	-0.711	2.01E-08
RP11-49I11.	-0.056	0.184	0.714	0.887	1.443	2.02E-08
KIAA2013	0.035	-0.105	-0.165	0.217	0.411	2.02E-08
CORO7	-0.39	-0.167	0.045	-0.12	0.45	2.03E-08
YWHAB	0.304	0.082	-0.005	0.312	0.371	2.04E-08
MYSM1	-0.358	0.134	0.26	-0.456	-0.145	2.04E-08
MTRF1	-0.564	0.044	0.048	-0.347	-0.654	2.04E-08
RP11-163O1	-0.232	0.044	0.008	-0.721	-0.725	2.04E-08
SNORD43	-0.314	-0.005	0.024	-0.996	-1.287	2.04E-08
RALBP1	0.23	0.081	0.056	0.376	0.421	2.05E-08
ATXN10	0.379	0.067	-0.075	0.316	0.404	2.07E-08
TBK1	0.378	0.261	0.164	0.257	0.3	2.08E-08
TUBE1	-0.564	-0.229	-0.008	-0.348	-0.703	2.09E-08
MDM1	-0.471	-0.084	-0.061	-0.392	-0.564	2.11E-08
CAMKK1	0.057	-0.344	0.209	-0.332	-0.539	2.13E-08
NBEAL1	-0.592	-0.017	0.168	-0.127	-0.169	2.15E-08
FAHD1	0.194	0.074	0.008	0.399	0.434	2.16E-08
TAF1D	-0.12	0.094	0.061	-0.573	-0.537	2.16E-08
MAP3K10	-0.389	0.193	0.368	-0.004	0.161	2.17E-08
SPATS2L	0.463	-0.057	-0.204	0.11	0.116	2.17E-08
GPR137B	0.278	0.366	0.236	0.348	0.805	2.18E-08
CTU1	-0.375	-0.022	-0.065	-0.195	0.526	2.20E-08
EIF2S2	0.234	0.103	0.027	0.341	0.195	2.21E-08
MTBP	-0.16	-0.12	-0.266	-0.266	-0.661	2.21E-08
RP11-58B17	-0.234	-0.062	0.127	-0.777	-0.581	2.22E-08
HLA-E	0.141	0.169	0.064	0.32	0.323	2.23E-08
FAM219B	-0.499	0.003	0.036	-0.608	-0.289	2.24E-08
DNAJC19	-0.507	-0.323	-0.145	-0.67	-0.755	2.25E-08
SLC25A43	0.209	0.12	0.186	0.344	0.587	2.26E-08
LMF1	0.024	0.084	0.078	0.653	0.427	2.27E-08
UXS1	0.199	0.106	0.072	0.36	0.387	2.27E-08
ZCCHC2	-0.306	0.121	0.028	-0.204	-0.374	2.27E-08
C9orf69	0.055	0.192	0.179	0.33	0.467	2.30E-08
RP11-21M24	-0.472	-0.041	-0.007	-0.517	0.061	2.31E-08

NBPF9	-0.4	0.013	0.224	-0.333	-0.091	2.31E-08
TBC1D30	0.534	-0.13	-0.223	0.03	0.06	2.32E-08
IBA57	0.474	0.214	0.08	0.022	0.068	2.33E-08
NT5DC1	0.219	0.175	0.184	0.448	0.47	2.35E-08
RP11-99E15	-1.804	-0.994	-0.885	-0.987	-1.356	2.35E-08
RAB1A	0.375	0.208	0.127	0.397	0.399	2.36E-08
MIDN	-0.125	0.079	0.161	0.154	0.334	2.36E-08
TMED7	0.089	-0.093	-0.184	-0.15	-0.331	2.36E-08
YTHDF2	0.281	0.058	-0.024	0.237	0.395	2.37E-08
RP11-496N1	-0.035	0.173	0.09	-0.788	-0.978	2.37E-08
C2orf66	-0.999	-0.066	-0.097	-1.311	-0.727	2.38E-08
DUT	-0.054	0.039	0.039	-0.143	-0.414	2.39E-08
BDH2	-0.216	0.148	0.115	0.019	0.464	2.40E-08
PTBP3	0.406	0.283	0.219	0.377	0.349	2.47E-08
AC074117.1(	-0.553	0.066	0.143	-0.354	-0.469	2.52E-08
RP11-392P7	-0.343	0.01	0.278	-0.132	-0.488	2.52E-08
NDUFV2P1	-0.188	-0.227	-0.215	-0.272	-0.615	2.53E-08
DDX60L	-0.414	0.022	0.186	-0.349	0.098	2.54E-08
KIAA0114	-0.299	-0.151	-0.099	-0.19	-0.489	2.54E-08
OGT	-0.368	0.08	0.179	-0.492	0.116	2.55E-08
MIR221	-0.908	0.284	0.158	-0.964	-0.447	2.55E-08
MRPL40	-0.005	-0.072	-0.046	0.01	-0.431	2.56E-08
SPRED2	0.377	0.241	0.313	0.439	0.688	2.57E-08
B3GNT9	0.437	-0.13	-0.243	0.017	0.299	2.59E-08
VPS41	0.277	0.16	0.126	0.304	0.486	2.64E-08
ASGR1	-0.573	0.015	0.407	0.122	0.526	2.67E-08
SLC6A6	0.405	0.254	0.278	0.391	0.441	2.68E-08
ZFP64	0.086	0.025	0.245	0.432	0.576	2.71E-08
SCAI	-0.528	-0.024	0.228	-0.347	0.08	2.72E-08
BCAS4	-0.061	0.066	0.091	0.421	1.11	2.73E-08
SLC16A5	0.434	0.248	0.356	-0.014	-0.126	2.73E-08
SCARNA12	-0.407	-0.035	-0.429	-1.162	-1.249	2.75E-08
STARD9	0.076	0.228	0.497	-0.247	0.132	2.76E-08
CYTH2	0.126	0.297	0.223	-0.445	0.068	2.76E-08
BID	0.165	0.01	-0.031	0.095	0.366	2.77E-08
RP11-440L1	-0.436	-0.163	0.314	-0.492	-0.81	2.77E-08
RIPK1	0.488	0.302	0.185	0.285	0.394	2.80E-08
PCLAF	-0.208	0.055	0.083	-0.209	-0.69	2.80E-08

P3H3	-0.015	-0.073	-0.143	0.095	0.398	2.82E-08
FGGY	0.364	-0.03	-0.063	0.491	0.783	2.84E-08
RP11-569H1	0.543	-0.051	-0.089	0.077	0.33	2.84E-08
DNAAF2	-0.588	-0.481	-0.241	-0.104	0.055	2.93E-08
RP5-961K14	0.077	-0.345	-0.448	-0.419	-0.893	2.94E-08
GPR135	-0.442	-0.11	-0.031	-1.044	-0.976	2.95E-08
RP11-645C2	-0.578	-0.16	0.213	-0.738	-0.414	2.96E-08
HPDL	0.196	-0.441	-0.705	-0.232	-1.13	2.96E-08
CHTF8	-0.068	0.001	0.068	0.338	0.355	3.07E-08
PDSS2	0.313	0.001	0.266	0.527	0.407	3.11E-08
PSMD12	0.492	0.24	0.107	0.325	0.001	3.11E-08
DCUN1D4	-0.102	0.012	-0.013	-0.439	-0.167	3.14E-08
USP33	0.088	-0.014	-0.234	-0.212	-0.35	3.16E-08
PRPS2	0.387	0.139	-0.089	0.042	-0.241	3.18E-08
CCZ1	0.25	-0.012	0.163	0.398	0.428	3.19E-08
LIN7B	0.016	-0.422	-0.135	-0.868	-1.097	3.21E-08
C19orf33	-0.173	-0.139	0.046	0.11	0.439	3.22E-08
KRT8	-0.296	-0.028	0.072	-0.004	0.381	3.24E-08
SNORA73B	0.059	0.181	0.172	-0.578	-1.016	3.24E-08
RP11-706O1	0.404	0.26	0.271	-0.309	0.079	3.26E-08
CAMK4	0.057	-0.151	-0.277	-0.098	-0.462	3.26E-08
MCAT	-0.096	0.049	0.058	0.401	0.48	3.37E-08
CPPED1	0.347	0.028	-0.117	0.285	0.349	3.38E-08
SH3YL1	-0.453	0.165	0.334	-0.204	-0.28	3.38E-08
INPPL1	-0.102	-0.05	-0.099	-0.464	-0.219	3.39E-08
SNORA71C	-1.216	-0.359	-0.38	-1.127	-1.457	3.41E-08
AC022182.2	0.41	0.047	-0.254	-0.013	-0.395	3.42E-08
ZNF600	0.044	0.552	0.552	0.161	0.003	3.45E-08
ARL4A	0.142	-0.067	-0.168	-0.384	-0.381	3.45E-08
ZBED4	0.386	0.063	-0.146	0.027	0.364	3.46E-08
KDM1A	0.209	-0.002	-0.113	0.245	0.375	3.47E-08
C2orf44	-0.281	0.037	-0.086	-0.07	-0.345	3.55E-08
ARHGAP19	-0.095	-0.22	-0.215	0.048	-0.54	3.57E-08
RAPGEFL1	-0.241	0.081	-0.019	-0.907	-0.362	3.59E-08
SNORD101	-0.028	0.201	0.373	-0.774	-0.916	3.61E-08
GIMAP1	-0.386	0.005	0.016	0.381	0.064	3.64E-08
RP5-1074L1.	-0.799	-0.501	-0.217	-1.174	-1.149	3.66E-08
ITPKC	0.021	0.407	0.123	0.056	0.091	3.72E-08

IFI6	0.002	-0.162	-0.15	0.038	0.546	3.74E-08
ANO8	-0.804	-0.345	0.161	-0.53	-0.339	3.74E-08
UBE2M	0.033	-0.052	-0.151	-0.078	-0.376	3.77E-08
ZNF740	0.139	0.308	0.309	0.247	0.389	3.78E-08
POLI	-0.535	-0.001	0.254	-0.225	-0.083	3.78E-08
ZNF205	-0.384	0.119	0.197	-0.007	0.035	3.82E-08
PLEKHH3	-0.41	0.123	0.021	-0.407	-0.426	3.83E-08
C6orf120	-0.324	-0.119	0.02	0.136	-0.094	3.85E-08
AVEN	0.314	0.026	0.046	0.291	0.475	3.93E-08
GPR107	0.189	0.112	0.072	0.24	0.389	3.93E-08
RNF31	-0.372	-0.187	-0.151	-0.384	-0.407	3.93E-08
NUDT19	0.255	-0.026	-0.101	0.293	0.519	3.96E-08
RP11-570P1	-0.012	-0.394	-0.438	-0.147	-0.51	3.97E-08
HIST1H3E	0.719	0.915	1.46	1.163	0.967	3.98E-08
USP9Y	-0.237	0.212	0.382	0.004	0.226	3.98E-08
RGS10	-0.214	-0.216	-0.162	-0.261	0.448	4.02E-08
IFT57	0.232	0.053	0.064	0.189	0.549	4.03E-08
TSC22D3	0.422	0.025	-0.173	-0.198	0.149	4.03E-08
DNAJC27	-0.509	-0.195	-0.201	-0.718	-0.801	4.03E-08
SNORD14B	-0.194	0.181	0.147	-0.72	-1.163	4.04E-08
ATG14	0.465	0.258	0.19	0.119	0.351	4.08E-08
NACAD	0.004	0.49	0.639	0.653	-0.12	4.09E-08
GSTP1	0.211	0.123	0.093	0.379	0.394	4.15E-08
SRSF11	-0.112	0.017	0.022	-0.422	-0.308	4.17E-08
PAIP2	0.101	0.205	0.158	0.17	0.471	4.19E-08
IL1R1	0.411	0.07	-0.186	-0.517	0.259	4.21E-08
NAPB	-0.317	0.098	0.064	-0.622	-0.628	4.26E-08
ANGEL1	-0.451	-0.002	0.043	-0.138	0.002	4.31E-08
VPS51	0.128	0.096	0.059	0.311	0.458	4.38E-08
ST3GAL6	-0.116	-0.197	-0.222	-0.191	0.456	4.44E-08
PCGF1	-0.133	0.129	0.114	-0.232	-0.463	4.48E-08
ZNF546	-1.089	-0.034	-0.034	-0.264	-0.587	4.48E-08
RP11-762H8	-0.562	0.141	0.198	-0.82	-0.95	4.48E-08
PHF14	0.236	0.413	0.291	0.569	0.422	4.49E-08
CLP1	0.588	0.327	0.211	0.512	0.296	4.54E-08
HMBOX1	-0.313	0.119	0.116	-0.494	-0.371	4.55E-08
CCDC137	-0.008	-0.012	-0.251	-0.379	-0.284	4.57E-08
WDR70	0.267	0.101	0.125	0.477	0.397	4.59E-08

DGKE	-0.587	-0.208	-0.1	-0.744	-0.633	4.60E-08
LPCAT4	-0.173	-0.377	0.05	0.069	-0.226	4.61E-08
CDA	0.3	0.03	0.464	0.721	1.366	4.62E-08
WDR4	-0.199	-0.093	-0.173	-0.311	-0.5	4.62E-08
PAM16	-0.455	-0.275	-0.11	-0.8	-0.82	4.62E-08
WNK1	0.159	0.04	-0.021	0.092	0.392	4.65E-08
C14orf159	0.068	-0.13	0.18	0.108	0.394	4.66E-08
AC005154.6	-0.579	-0.144	0.038	-0.635	-0.705	4.66E-08
WNT2B	-0.156	0.231	0.354	-0.517	-0.317	4.67E-08
CCNY	0.329	-0.024	0.009	0.273	0.352	4.71E-08
SUZ12P	-0.281	-0.174	-0.149	-0.772	-0.706	4.71E-08
RGS5	0.083	-0.09	-0.165	-0.097	-0.863	4.71E-08
BTBD7	0.325	-0.012	-0.085	0.007	-0.084	4.74E-08
MID1	-0.263	-0.534	-0.304	-0.056	0.029	4.77E-08
RP11-538I12	-0.409	-0.352	-0.006	0.731	1.041	4.83E-08
ECM1	0.043	0.149	0.161	0.2	0.651	4.83E-08
CRTC1	-0.195	0.151	0.434	0.183	0.419	4.83E-08
RP11-455B3	0.414	-0.016	0.038	-0.117	1.222	4.86E-08
NFIL3	0.276	0.178	-0.223	0.041	-0.344	4.88E-08
MYC	-0.499	-0.006	-0.173	0.007	0.054	4.89E-08
CTIF	-0.266	-0.381	-0.252	-0.34	-0.101	4.95E-08
HERC2P3	-1.208	-0.461	-0.229	-1.234	-1.028	4.97E-08
TRAPPC4	0.08	-0.078	-0.083	0.028	-0.365	4.98E-08
KLHL13	0.426	0.014	-0.341	-0.148	-0.573	5.00E-08
ATP7A	0.35	-0.031	-0.237	0.088	-0.018	5.13E-08
SNHG4	-0.54	-0.256	-0.035	-0.888	-0.649	5.14E-08
RP11-81A1.6	-0.687	0.272	0.322	-0.551	-0.005	5.20E-08
CCDC91	0.378	-0.036	-0.159	0.158	0.364	5.21E-08
RP11-158L1:	-0.094	-0.033	-0.018	-0.836	-0.704	5.26E-08
RP11-296I10	-0.383	-0.106	0.095	-0.997	-0.549	5.33E-08
TSHZ1	0.212	-0.366	-0.269	0.154	0.306	5.34E-08
RP11-864I4.	-0.588	-0.172	-0.182	-0.743	-0.613	5.43E-08
RP11-214O1	0.217	-0.07	0.11	0.348	0.39	5.45E-08
PLCB1	0.071	0.102	0.001	0.348	0.485	5.47E-08
FLYWCH1	-0.064	0.183	0.29	0.31	0.447	5.47E-08
PI4KAP2	-0.157	0.473	0.469	-0.411	-0.257	5.47E-08
ZNF449	-0.731	-0.057	-0.034	-0.66	-0.416	5.53E-08
RP11-252A2	-0.37	0.048	0.261	-0.619	-0.551	5.62E-08

ZKSCAN3	-0.549	0.077	0.132	-0.107	-0.152	5.65E-08
OXR1	0.016	0.063	-0.219	-0.168	-0.39	5.65E-08
TRMT10A	-0.019	0.094	0.223	0.496	-0.148	5.67E-08
KIAA0355	0.463	-0.086	0.15	0.006	0.288	5.68E-08
LTBP1	0.301	0.11	-0.169	0.187	-0.481	5.68E-08
SNORA77B	-0.501	-0.391	-0.391	-1.165	-1.529	5.68E-08
ETAA1	-0.268	0.079	0.235	-0.116	-0.366	5.71E-08
ZNF514	-0.785	0.051	0.132	-0.498	-0.391	5.71E-08
TPBG	0.02	0.08	-0.01	0.013	0.385	5.72E-08
GPX8	-0.068	-0.183	-0.248	-0.317	-0.508	5.73E-08
DNASE1L2	-0.604	-0.155	0.141	-0.717	-1.187	5.73E-08
PKD1	-0.357	-0.347	-0.111	-0.81	-0.247	5.85E-08
AC006026.1(	-0.371	-0.063	-0.111	-0.758	-0.883	5.85E-08
JAK1	0.636	0.179	-0.086	0.138	0.24	5.91E-08
NAT1	-0.032	0.192	-0.006	-0.063	-0.529	5.93E-08
AGAP3	-0.405	-0.308	-0.135	-0.336	-0.211	5.97E-08
ARHGDIG	0.152	-0.19	-0.167	-0.524	-1.031	5.97E-08
RF00056	-0.739	-0.109	-0.389	-1.136	-1.211	5.98E-08
DMTF1	-0.054	0.162	0.298	-0.436	-0.266	5.99E-08
SNORD14A	0.001	0.277	0.156	-0.629	-1.149	6.07E-08
RNF123	-0.059	0.139	0.261	0.363	0.386	6.08E-08
DBR1	0.472	0.003	-0.07	0.216	0.259	6.13E-08
CRAMP1	0.001	0.191	0.068	-0.373	-0.151	6.15E-08
CCDC85A	0.256	-0.31	-0.451	-0.076	-0.157	6.34E-08
ROGDI	-0.385	-0.12	-0.199	-0.661	-0.171	6.41E-08
SNORD102	-0.343	-0.049	0.034	-0.817	-1.212	6.47E-08
SLC22A31	-0.119	-0.109	-0.213	-0.612	-1.353	6.47E-08
EP300	0.391	0.216	0.075	-0.021	0.187	6.51E-08
ANXA2P3	0.323	-0.157	-0.26	0.38	0.588	6.56E-08
DIEXF	0.558	0.098	0.255	0.324	0.221	6.63E-08
KCNAB3	-0.295	0.114	0.264	-0.793	-1.09	6.63E-08
H3F3C	0.169	-0.3	-0.356	-0.157	-0.301	6.68E-08
PARP2	0.036	0.045	-0.153	-0.054	-0.344	6.77E-08
DYNC1LI1	0.475	0.16	-0.097	0.233	-0.03	6.80E-08
ZDHHC12	-0.016	-0.027	-0.07	0.378	-0.32	6.85E-08
TIMP3	-0.582	0.552	0.69	0.237	0.785	6.87E-08
NRBP1	0.295	0.076	-0.053	0.298	0.454	6.93E-08
ARRDC3	-0.285	0.028	-0.328	-0.274	-0.311	6.93E-08

RP11-438L7.	0.412	0.14	0.14	0.603	0.801	6.94E-08
THEMIS2	-0.11	-0.253	-0.241	-0.086	0.561	7.16E-08
DTYMK	-0.197	-0.185	-0.178	-0.083	-0.426	7.20E-08
MSANTD3	0.13	-0.007	0.069	0.143	0.37	7.22E-08
CLEC2B	-0.837	-0.204	-0.3	-0.802	-1.006	7.25E-08
AF131215.2	-0.239	-0.162	0.15	-0.122	0.529	7.28E-08
THAP3	-0.605	-0.194	-0.321	-0.343	-0.201	7.28E-08
SERPINB9	0.525	0.107	0.102	0.342	0.207	7.40E-08
ENTPD7	0.468	0.062	-0.055	0.123	0.141	7.48E-08
SPAG4	-0.089	-0.011	0.01	-0.373	-0.698	7.51E-08
BCL9	0.497	0.094	-0.126	0.06	0.131	7.65E-08
TLE2	-0.073	-0.008	0.106	0.028	0.428	7.68E-08
DCUN1D2	-0.136	-0.183	-0.16	-0.677	-0.514	7.72E-08
CTD-2027G2	-1.026	-0.996	-0.512	-0.933	-0.78	7.72E-08
EMC9	-0.738	-0.422	-0.379	-0.47	-0.503	7.85E-08
ACTR3B	0.058	0.312	0.215	0.159	-0.381	7.90E-08
WFS1	0.194	0.125	0.159	0.467	0.573	7.91E-08
TUBG1	0.168	0.088	-0.016	0.345	-0.394	7.91E-08
DLAT	0.324	-0.037	-0.122	0.265	-0.145	7.94E-08
UCKL1	-0.239	-0.063	-0.175	-0.446	-0.209	8.08E-08
STAMBPL1	0.302	-0.116	0.469	0.547	-0.599	8.10E-08
RP11-513I15	-0.052	-0.151	-0.123	-0.276	-0.841	8.12E-08
UBC	0.648	0.228	0.229	0.629	0.658	8.16E-08
RP11-368I7.2	-0.093	-0.21	-0.205	-0.323	-0.709	8.20E-08
CD81	0.293	0.068	-0.072	0.345	0.442	8.21E-08
HCN2	0.31	0.016	-0.136	-0.144	-0.82	8.29E-08
PRKCDBP	-0.102	-0.371	-0.461	-0.154	0.211	8.38E-08
RPN2	0.261	0.074	-0.042	0.368	0.425	8.40E-08
ZNF500	-0.201	0.205	-0.017	-0.42	-0.208	8.45E-08
PRKD2	-0.341	-0.044	0.082	0.015	0.194	8.55E-08
C5orf34	-0.528	-0.125	-0.346	-0.166	-0.776	8.56E-08
TMC6	-0.16	-0.251	-0.02	-0.162	-0.457	8.65E-08
MIER2	0.242	-0.065	-0.019	-0.018	-0.34	8.74E-08
SNX29	0.445	0.127	0.251	0.563	0.598	8.76E-08
RP11-139E1	0.094	0.092	0.239	0.375	0.718	8.77E-08
SLC39A14	0.231	-0.03	-0.041	0.109	0.323	8.79E-08
PANK4	-0.092	0.035	-0.004	-0.153	-0.433	8.81E-08
NGLY1	0.077	-0.026	0.147	0.408	0.215	8.84E-08

GRAMD1C	-0.112	-0.125	0.322	0.239	0.364	8.88E-08
ZNF529	0.066	0.442	0.336	-0.096	-0.106	8.91E-08
ENG	0.425	-0.008	-0.268	0.063	-0.434	8.93E-08
ATP5A1	0.297	0.079	-0.001	0.419	0.346	9.01E-08
NAPEPLD	-0.547	-0.202	0.039	-0.081	-0.225	9.02E-08
TTC32	0.199	0.163	-0.024	-0.316	-0.872	9.04E-08
RP11-304L1	-0.644	-0.422	-0.202	-0.914	-0.905	9.11E-08
PARP6	-0.494	-0.117	-0.013	-0.655	-0.332	9.14E-08
HK1	0.341	-0.032	0.031	0.537	0.545	9.16E-08
PRRT3	0.22	-0.348	0.119	0.377	0.674	9.20E-08
KAZALD1	0.385	-0.214	-0.363	-0.338	-0.293	9.20E-08
ZNF513	-0.289	0.115	-0.093	-0.528	-0.502	9.24E-08
PLXNB3	-0.371	0.148	0.556	0.052	0.355	9.26E-08
RP4-702J19.	0.044	-0.046	-0.083	-0.282	-0.519	9.26E-08
RP4-756G23	0.267	0.502	0.667	0.642	1.404	9.39E-08
CIC	0.03	0.309	0.32	0.119	0.35	9.39E-08
RP11-235E1	-0.317	-0.008	0.427	-0.282	0.45	9.49E-08
CCDC144B	-0.773	-0.16	0.095	-0.932	-0.749	9.62E-08
TLK2	0.439	0.116	0.005	0.096	0.293	9.71E-08
RP11-336K2	-1.438	-0.305	0.004	-0.808	-0.128	9.71E-08
ZNF587	-0.194	0.026	0.128	-0.401	0.231	9.76E-08
RAC3	-0.142	-0.243	-0.343	-0.281	0.248	9.79E-08
AC108488.4	-0.859	-0.2	-0.394	-0.902	-0.956	9.85E-08
CHRNB1	-0.225	0.088	0.015	0.289	0.497	9.94E-08
ZNF485	-0.889	-0.105	0.22	-0.247	-0.586	9.98E-08
TMEM160	-0.209	0.043	-0.061	0.062	0.775	1.00E-07
ZFHX3	0	-0.074	0.117	0.098	0.437	1.00E-07
BAZ1A	0.283	0.365	0.04	0.125	0.423	1.00E-07
ABCE1	0.286	0.032	-0.019	0.343	0.17	1.00E-07
MIR3916	-0.421	-0.003	0.04	-0.997	-0.506	1.01E-07
ST13P3	0.279	0.66	0.33	0.886	0.957	1.02E-07
MPDZ	0.149	0.025	0.22	0.205	0.439	1.02E-07
LARP7	0.312	0.167	0.146	0.456	0.197	1.02E-07
N4BP2	0.568	-0.123	0.025	0.162	0.15	1.02E-07
ARL5B	0.396	0.092	-0.12	-0.098	0.149	1.02E-07
LAMA5	-0.383	-0.282	0.268	-0.065	-0.013	1.02E-07
NIFK	-0.079	-0.062	-0.148	-0.124	-0.406	1.02E-07
JKAMP	-0.207	-0.222	-0.236	-0.453	-0.66	1.02E-07

MICE	-0.78	-0.112	-0.169	-1.001	-1.211	1.02E-07
SCN8A	-0.528	-0.332	0.074	-0.257	-0.054	1.03E-07
MTCP1	-0.242	0.333	0.149	-0.691	-0.466	1.03E-07
RP11-249C2	-0.358	0.558	1.071	0.443	1.089	1.05E-07
HNRNPCL1	0.446	-0.049	-0.118	0.232	0.594	1.05E-07
HEXIM1	0.642	0.144	-0.148	0.122	0.094	1.05E-07
SMYD3	0.33	0.055	-0.286	0.232	0.422	1.07E-07
UBE2J2	0.126	0.113	0.02	0.155	0.369	1.07E-07
OSBPL10	0.299	-0.017	-0.052	0.312	0.329	1.07E-07
RCE1	-0.226	-0.086	-0.154	-0.534	-0.493	1.07E-07
NDUFA12	-0.13	-0.15	-0.111	-0.213	-0.544	1.07E-07
CCDC9	0.273	0.085	0.019	-0.362	-0.085	1.08E-07
TRDMT1	-0.061	-0.232	-0.104	-0.331	-0.815	1.08E-07
C15orf39	0.1	0.133	0.193	0.311	0.555	1.11E-07
EIF2B3	0.306	0.001	-0.084	0.329	-0.146	1.11E-07
RP11-793I11	-0.093	-0.293	-0.245	-0.405	-0.648	1.11E-07
HOXD4	-0.828	-0.11	-0.046	-0.702	-0.668	1.12E-07
CEBPZOS	-0.115	-0.044	0.031	0.135	0.35	1.14E-07
DMPK	-0.125	0.16	0.263	-0.603	-0.067	1.14E-07
RP11-263K1	-0.324	0.046	-0.275	-1.286	-0.958	1.15E-07
ZNF445	-0.098	0.045	0.015	-0.403	-0.26	1.16E-07
C19orf25	-0.079	0.044	0.009	-0.25	-0.345	1.16E-07
RP11-345I19	-0.558	-0.779	-0.651	-0.352	-0.031	1.17E-07
XG	0.054	-0.17	0.114	0.189	1.068	1.20E-07
ZNF252P	-0.361	0.025	0.35	0.189	0.233	1.20E-07
TAZ	-0.48	-0.065	0.216	-0.243	-0.085	1.20E-07
RP11-147L1:	-0.286	0.024	0.22	-0.001	0.924	1.21E-07
TTC28	0.394	0.126	0.341	0.4	0.374	1.21E-07
APPL1	0.404	0.072	0.035	0.263	0.08	1.21E-07
KIAA0195	-0.281	-0.08	-0.134	-0.307	-0.34	1.21E-07
PPM1K	-0.874	-0.334	-0.356	-0.524	-0.487	1.21E-07
KLRG1	-0.165	-0.796	-0.764	-1.175	-1.477	1.21E-07
CEP295	-0.465	-0.108	-0.162	-0.394	-0.592	1.22E-07
UBE2F	0.131	-0.031	-0.048	-0.007	0.356	1.25E-07
C3orf18	-0.628	0.279	0.465	0.055	0.108	1.25E-07
IGF1R	0.164	0.14	-0.117	-0.161	0.327	1.26E-07
EIF4G2	0.335	0.099	-0.056	0.118	0.216	1.27E-07
ADM2	0.479	0.562	0.861	0.986	1.413	1.28E-07

ZFR	0.261	0.07	0.067	0.304	0.448	1.28E-07
NXT1	0.016	-0.32	-0.312	-0.216	-0.463	1.28E-07
SHOC2	0.379	0.202	0.025	0.185	0.297	1.29E-07
SMIM14	0.108	-0.048	0.091	0.378	0.132	1.29E-07
TSPAN33	0.715	0.291	-0.106	-0.034	-0.928	1.31E-07
AKAP12	0.446	0.194	0.299	0.2	0.537	1.32E-07
TMBIM1	0.34	0.138	0.119	0.348	0.386	1.33E-07
PYCRL	-0.155	-0.225	-0.064	0.038	-0.509	1.33E-07
PGM1	0.416	-0.1	-0.054	0.31	0.534	1.35E-07
NBPF11	-0.167	0.356	0.232	-0.488	-0.306	1.35E-07
PPP1R12C	-0.31	0.035	0.029	-0.417	-0.27	1.36E-07
L3HYPDH	-0.451	-0.052	0.101	-0.457	-0.66	1.36E-07
AC083873.4	0.223	0.219	-0.044	0.569	0.737	1.37E-07
ZNF346	-0.234	0.045	-0.196	-0.264	-0.384	1.37E-07
GALK1	-0.112	-0.092	-0.115	0.174	0.371	1.38E-07
NCKIPSD	-0.304	-0.059	-0.069	-0.16	-0.581	1.40E-07
PIK3CB	0.139	-0.011	0.111	0.387	0.397	1.41E-07
ERO1A	0.145	0.293	0.11	0.303	0.335	1.41E-07
CCNT1	0.004	0.101	-0.172	-0.345	-0.273	1.41E-07
ERBIN	0.353	0.379	0.176	0.174	0.094	1.42E-07
TMUB2	-0.345	-0.011	0.055	-0.131	-0.192	1.42E-07
PSMC3	0.4	0.226	0.099	0.539	-0.01	1.43E-07
SNORD14D	-0.778	-0.495	-0.579	-1.267	-1.467	1.43E-07
FBLN7	0.331	0.297	0.139	0.554	1.103	1.44E-07
AC005519.4	-0.513	-0.115	0.098	-0.864	-0.64	1.44E-07
NDUFAF4	-0.117	-0.218	-0.284	-0.377	-0.655	1.44E-07
PROB1	0.193	-0.112	-0.289	-0.381	0.318	1.45E-07
RBM6	-0.39	-0.129	0.019	-0.472	-0.222	1.45E-07
SCARF2	0.024	-0.077	-0.251	-0.73	-0.164	1.46E-07
BAG3	0.382	-0.051	-0.089	0.414	0.483	1.47E-07
ERC1	0.214	0.119	0.084	0.221	0.453	1.48E-07
PKN1	0.186	-0.031	-0.088	0.281	0.352	1.48E-07
TP53TG1	-0.054	0.133	0.547	0.383	0.655	1.49E-07
PLA2G15	0.023	-0.011	-0.134	0.22	0.358	1.49E-07
TGFA	0.673	0.254	-0.504	-0.12	0.318	1.49E-07
RP3-323B6.2	0.529	-0.473	-0.017	0.287	0.452	1.50E-07
RDH10	0.023	0.062	-0.25	-0.413	-0.375	1.51E-07
OPN3	0.155	0.039	0.043	0.313	0.427	1.53E-07

ZSCAN31	-0.476	-0.382	-0.675	-0.804	-0.106	1.53E-07
MTRNR2L8	-0.839	-0.512	-0.276	-1.538	-0.947	1.54E-07
SULT1E1	-0.53	-0.295	-0.105	-0.29	-1.54	1.54E-07
EZR	0.134	0.034	0.051	0.331	0.481	1.56E-07
SIMC1	0.291	-0.096	-0.355	-0.352	-0.287	1.56E-07
RP11-1334A	-0.203	-0.566	0.552	-0.231	0.087	1.57E-07
FANCM	-0.19	-0.157	-0.382	-0.341	-0.53	1.57E-07
ABRACL	-0.083	-0.076	-0.174	-0.66	-0.624	1.58E-07
CNOT2	0.322	0.166	0.081	0.245	0.321	1.59E-07
RP11-501M7	0.25	0.111	0.119	0.551	0.737	1.60E-07
SAMM50	0.227	0.152	0.121	0.498	0.33	1.60E-07
FAM168B	0.41	0.101	-0.088	0.141	0.209	1.60E-07
RP4-548D19	-0.302	0.109	-0.064	-0.986	-0.934	1.60E-07
SLU7	0.535	0.395	0.258	0.545	0.533	1.61E-07
CH507-9B2.9	1.071	0.21	0.685	0.332	0.249	1.61E-07
NDRG4	0.087	-0.157	-0.554	-0.546	-0.22	1.61E-07
FAM124B	0.058	-0.067	0.102	0.299	0.491	1.63E-07
BHLHE41	-0.398	0.12	-0.318	-0.284	-1.249	1.63E-07
PRRX1	-0.313	-0.171	0.072	0.233	0.8	1.64E-07
SEH1L	0.352	0.031	-0.026	0.215	-0.236	1.64E-07
THSD7A	0.16	-0.23	-0.16	-0.364	-0.252	1.65E-07
BOD1L1	0.387	0.293	0.261	0.226	0.42	1.66E-07
KMT5C	-0.36	0.213	0.012	-0.601	-0.371	1.66E-07
MBLAC2	-0.448	-0.26	-0.339	-0.216	-0.647	1.67E-07
MIR647	-0.852	-0.166	-0.242	-1.225	-0.962	1.69E-07
UBN2	0.001	0.359	0.311	-0.311	0.061	1.71E-07
NARFL	-0.34	0.009	-0.067	-0.072	-0.406	1.71E-07
LL21NC02-2	0.129	0.434	0.318	0.59	1.303	1.72E-07
SNHG9	-0.25	-0.213	-0.051	-0.426	-0.975	1.73E-07
AC011526.1	-0.076	0.03	0.153	-0.15	0.534	1.74E-07
ZBTB5	0.348	0.276	0.036	-0.034	-0.054	1.74E-07
RERE	0.179	-0.093	0.037	0.117	0.326	1.76E-07
TUBA4A	0.167	0.092	0.03	0.528	-0.269	1.76E-07
NDUFAF7	-0.434	0.115	0.195	-0.18	-0.323	1.76E-07
MYO6	0.432	0.398	0.344	0.523	0.671	1.77E-07
NCSTN	0.258	0.128	0.009	0.351	0.487	1.77E-07
CAMLG	-0.081	0.056	0.124	0.107	0.405	1.78E-07
ITGB3BP	-0.452	0.035	0.159	-0.427	-0.822	1.78E-07

ZFAND3	0.396	0.223	0.096	0.419	0.605	1.79E-07
FOXO3	0.343	0.14	0.093	0.371	0.533	1.79E-07
CACNB1	-0.268	-0.142	-0.09	-0.792	-0.645	1.79E-07
RP11-466P2	-0.367	-0.064	-0.052	-0.093	-1.082	1.79E-07
RP4-553F4.6	0.409	0.427	0.461	0.637	1.628	1.80E-07
CUEDC1	-0.13	0	0.196	0.005	0.391	1.80E-07
ADRM1	0.296	0.025	-0.144	0.131	-0.373	1.81E-07
CARD16	-0.17	0.104	0.343	0.184	0.496	1.83E-07
LA16c-60H5.	0.273	-0.036	-0.194	-0.321	-1.26	1.83E-07
BRE	0.363	0.069	-0.021	0.478	0.461	1.87E-07
METTL8	-0.048	0.068	0.343	0.304	0.37	1.87E-07
ARF6	0.311	0.102	0.148	0.444	0.304	1.87E-07
AC008070.1	0.486	-0.008	-0.05	-0.697	-0.934	1.89E-07
IFITM3	0.189	0.014	0.032	0.445	0.447	1.92E-07
AC100748.2	-0.578	-0.232	-0.086	-0.193	-0.878	1.92E-07
YWHAZ	0.4	0.102	-0.035	0.271	0.28	1.93E-07
CTD-2574D2	-0.552	0.024	0.211	-0.835	-0.573	1.94E-07
CTSA	0.133	0.04	-0.044	0.261	0.481	1.98E-07
ZFAT	0.332	0.209	0.254	0.766	0.46	1.98E-07
XXbac-BPG2	-0.973	-0.05	-0.016	-0.536	-0.759	1.98E-07
LEMD2	-0.241	-0.041	0.012	-0.302	-0.39	1.99E-07
LZTR1	-0.375	0.064	0.149	-0.286	-0.182	2.04E-07
NFATC4	-0.479	-0.088	0.063	-0.558	-0.063	2.05E-07
ARF4	0.362	0.082	-0.058	0.122	0.077	2.06E-07
FNBP1L	0.11	0.143	0.054	0.131	0.464	2.07E-07
TTC21B	0.031	0.088	-0.165	-0.288	-0.346	2.07E-07
RP11-211G2	-0.316	-0.064	0.922	0.192	1.119	2.08E-07
TLR6	0.014	0.028	0.067	0.272	1.045	2.09E-07
AC084018.1	-0.24	-0.066	0.095	-0.782	-0.54	2.11E-07
TAGLN2	0.331	0.064	-0.003	0.426	0.44	2.12E-07
DST	-0.192	-0.092	0.252	-0.185	0.404	2.13E-07
ZNF550	-0.262	0.22	0.124	-0.56	-0.37	2.13E-07
SEMA3B	0.325	0.699	1.031	0.161	-0.174	2.16E-07
SLC46A3	0.188	0.162	0.094	0.454	1.08	2.18E-07
DDX18	0.33	0.138	0.053	0.33	-0.016	2.18E-07
PDCD4	0.452	0.116	0.066	0.443	0.556	2.19E-07
ADARB1	0.152	0.295	0.363	0.194	0.308	2.19E-07
PRR14	-0.214	0.022	-0.029	-0.308	-0.395	2.19E-07

ZNF484	0.241	0.224	0.01	-0.221	-0.398	2.19E-07
TCF7	0.332	0.071	-0.457	-0.45	-0.69	2.21E-07
ALG10B	-0.319	0.103	-0.079	-0.339	-0.56	2.23E-07
MVK	0.05	-0.357	-0.591	-0.396	-0.03	2.25E-07
GPC2	-0.782	-0.165	-0.299	-0.896	-0.1	2.25E-07
TRIOBP	0.165	0.059	0.174	0.369	0.137	2.26E-07
RP11-320M2	-0.918	0.121	-0.058	-0.516	-1.066	2.26E-07
MSL3	-0.095	-0.033	0.081	-0.015	0.335	2.28E-07
C17orf49	0.175	0.07	0.041	0.083	-0.833	2.29E-07
RP11-572O1	-0.629	-0.601	-0.731	-1.627	-0.963	2.30E-07
LIG1	0.266	0.424	0.349	0.389	0.012	2.31E-07
KIF26A	0.045	-0.193	-0.017	-0.432	-0.539	2.35E-07
TRIM7	-0.251	-0.191	0.068	0.167	0.385	2.36E-07
RP11-228M1	-0.591	-0.164	-0.166	-0.782	-0.934	2.36E-07
NANS	0.347	0.065	-0.074	0.319	0.405	2.37E-07
SNORA25	-0.236	-0.045	0.091	-0.827	-0.925	2.37E-07
CCDC6	0.112	0.087	0.115	0.418	0.425	2.39E-07
FADD	-0.071	-0.073	0.043	0.387	0.227	2.40E-07
SNORA62	-0.205	0.336	0.242	-0.725	-0.896	2.42E-07
C9orf172	-0.358	0.279	0.547	-0.204	0.012	2.43E-07
CCT4	0.286	0.017	-0.036	0.324	0.218	2.44E-07
STEAP3	0.334	-0.127	-0.138	0.079	0.261	2.46E-07
HHIP	0.232	0.134	-0.147	0.128	-0.422	2.46E-07
LRRC37BP1	0.314	0.118	0.167	0.049	0.451	2.47E-07
TRIM4	-0.126	0.156	0.03	0.205	0.419	2.47E-07
BRCC3	0.228	0.046	0.12	0.38	0.379	2.47E-07
SNORD45A	-0.321	0.147	0.075	-0.914	-0.939	2.48E-07
SH3GL1	0.371	-0.043	-0.006	0.308	0.409	2.49E-07
HOXA5	-0.108	-0.292	-0.063	-0.071	0.439	2.50E-07
RP11-436J2(	0.413	0.19	0.077	0.415	0.006	2.51E-07
FAM216A	-0.233	-0.294	-0.104	-0.161	-0.572	2.52E-07
REEP2	-0.022	0.25	0.261	0.415	1.14	2.54E-07
RP11-142G7	-0.088	-0.027	0.134	0.21	0.467	2.54E-07
TSPYL1	0.245	0.111	0.021	0.48	0.607	2.55E-07
WASHC2C	0.161	0.208	0.026	0.368	0.438	2.56E-07
TTC33	-0.13	0.167	0.07	-0.149	-0.387	2.61E-07
NPIPB11	-0.322	-0.104	0.046	-0.962	-0.669	2.61E-07
SNORA70	-0.203	-0.078	0.07	-0.811	-0.903	2.61E-07

ARSK	0.096	0.02	-0.201	-0.114	-0.363	2.63E-07
LCA5	0.668	0.629	0.374	0.436	0.527	2.64E-07
CMTM4	0.445	0.336	0.27	-0.129	0.129	2.64E-07
RP11-288G3	0.234	-0.501	-0.365	0.137	0.301	2.66E-07
RIOK1	0.09	0.044	-0.118	-0.032	-0.388	2.66E-07
OXLD1	-0.573	-0.023	-0.086	-0.377	-0.464	2.66E-07
RP11-359J1	-1.026	-0.229	-0.049	-0.945	-1.082	2.66E-07
TTC7B	0.244	-0.028	-0.101	0.188	0.379	2.67E-07
PLAGL2	0.383	0.13	-0.045	-0.053	0.126	2.67E-07
DYM	0.364	0.031	0.074	0.349	0.387	2.68E-07
CKAP5	-0.191	-0.391	-0.321	0.069	0.229	2.69E-07
VPS13A	-0.3	0.197	0.325	-0.167	0.002	2.72E-07
POMP	-0.096	-0.092	-0.098	-0.31	-0.673	2.74E-07
RP11-9G1.3	-0.307	-0.164	-0.232	-0.565	-1.277	2.74E-07
PREPL	0.103	0.054	0.132	0.456	0.153	2.75E-07
TAX1BP1	0.329	0.239	0.131	0.479	0.338	2.79E-07
RGS11	-0.4	0.116	0.35	-0.477	-0.072	2.81E-07
CCDC189	-0.452	-0.147	-0.37	-1.062	-0.902	2.82E-07
FAM102B	0.538	-0.374	-0.536	-0.169	-0.213	2.84E-07
PDIA3	0.366	0.206	0.054	0.485	0.479	2.86E-07
PDS5A	0.173	0.086	-0.003	0.25	0.383	2.89E-07
FASTKD3	-0.264	-0.14	-0.185	-0.624	-0.743	2.89E-07
CITED2	0.208	0.273	0.266	0.614	0.413	2.90E-07
RP11-274B2	-0.157	0.302	0.138	-0.879	-0.542	2.91E-07
HPS5	0.089	0.162	-0.016	0.03	-0.424	2.92E-07
SNORD99	0.074	0.033	0.095	-0.874	-0.972	2.92E-07
OSTM1	0.247	0.227	0.21	0.437	0.35	2.94E-07
NYAP1	-0.934	0.014	0.344	-0.114	0.172	2.94E-07
MELK	-0.045	-0.047	-0.2	0.055	-0.417	2.94E-07
MYH9	0.492	0.125	0.061	0.316	0.72	2.95E-07
RP11-2C24.	-0.598	-0.297	-0.497	-1.019	-1.056	2.97E-07
SOS1	0.033	0.165	0.137	0.061	0.351	2.98E-07
NBPF25P	-0.449	-0.156	0.071	-0.48	-0.31	2.98E-07
USP16	0.354	0.154	0.154	0.203	-0.021	3.00E-07
LUC7L3	-0.439	-0.028	0.126	-0.496	-0.266	3.01E-07
GNA13	0.361	0.091	-0.059	0.083	0.198	3.08E-07
CTB-55O6.1	-0.483	-0.01	0.582	0.025	0.115	3.09E-07
TTC14	-0.662	0.019	0.08	-0.593	-0.265	3.10E-07

ACVR2B	0.043	0.343	-0.011	-0.468	-0.197	3.17E-07
HSP90AA1	0.401	0.197	0.122	0.605	0.585	3.19E-07
NTHL1	-0.132	-0.397	-0.15	0.208	0.213	3.20E-07
RP5-1182A1	-0.582	-0.01	0.075	-0.847	-0.83	3.20E-07
N4BP1	0.254	0.101	0.079	0.207	0.468	3.22E-07
MRFAP1	0.302	0.15	0.105	0.457	0.383	3.22E-07
RP11-206L1(	0.107	0.048	-0.089	-0.489	-0.322	3.27E-07
ZBTB7B	0.132	0.377	0.37	0.03	0.041	3.28E-07
ZEB1-AS1	-0.771	-0.282	0.03	-0.357	-0.407	3.28E-07
YARS2	0.105	0.07	-0.05	0.229	0.419	3.29E-07
A1BG-AS1	-0.159	0.351	0.267	-0.157	-0.259	3.29E-07
RELA	0.133	0.171	0.196	0.328	0.429	3.32E-07
EPHA2	0.416	0.265	0.229	0.113	0.377	3.37E-07
ZNF275	0.406	0.137	-0.005	-0.09	0.098	3.38E-07
RAB23	0.403	0.094	-0.113	-0.049	-0.033	3.38E-07
TICAM1	0.518	0.253	-0.031	0.172	0.59	3.39E-07
ALDH16A1	0.004	0.042	-0.06	0.004	-0.521	3.40E-07
NUDT12	-0.175	0.144	0.251	0.169	-0.329	3.42E-07
DSTYK	0.455	0.247	0.079	0.248	0.367	3.43E-07
DMAP1	-0.135	0.011	0.021	0.016	-0.369	3.45E-07
RABGGTA	0.114	0.437	0.377	0.332	0.146	3.46E-07
EEF1D	0.144	0.149	0.122	0.216	0.328	3.47E-07
MPZL3	-0.011	-0.129	0.116	0.108	-0.434	3.47E-07
ADAR	0.161	0.09	0.077	0.292	0.474	3.50E-07
WHAMML1	-0.438	-0.057	-0.03	-0.304	-0.599	3.50E-07
AMZ2	0.009	0.116	0.279	0.326	0.135	3.51E-07
AC006465.4	-0.758	-0.953	-1.104	-0.54	-0.371	3.51E-07
CLTB	0.215	0.07	-0.019	0.238	0.467	3.52E-07
FAM53B	-0.146	-0.246	-0.175	0.134	0.332	3.54E-07
SOCS2	0.067	0.49	0.17	-0.027	0.791	3.55E-07
CTB-193M12	-0.517	-0.719	-0.627	-0.215	-0.11	3.56E-07
NXT2	0.067	0.112	-0.063	-0.152	-0.658	3.56E-07
ZNF331	-0.578	0.01	0.255	-0.421	-0.418	3.58E-07
TCP11L2	-0.072	0.405	0.45	0.194	0.929	3.60E-07
RP11-298J2:	-0.521	0.278	0.518	-0.249	-0.1	3.61E-07
FAM110D	-0.552	-0.216	-0.031	0.237	0.618	3.62E-07
FAM168A	-0.028	0.148	0.256	0.377	0.354	3.63E-07
SLC41A2	0.289	0.537	0.394	0.381	0.564	3.64E-07

EXOC8	0.452	0.285	0.207	0.184	0.043	3.64E-07
NAP1L5	0.376	0.115	0.117	0.46	-0.22	3.64E-07
KCTD5	0.445	0.138	-0.215	0.109	-0.025	3.66E-07
AC136007.2	-0.396	0.021	0.223	-0.463	-0.382	3.66E-07
CBL	0.116	0.18	0.126	0.083	0.426	3.68E-07
XPO6	0.147	-0.106	-0.193	-0.061	-0.395	3.68E-07
KCTD10	0.372	0.092	0.016	0.137	0.173	3.71E-07
CCDC34	-0.07	0.033	0.078	0.52	0.003	3.71E-07
SLC39A6	-0.09	0.152	0.08	0.116	0.361	3.75E-07
FTO	0.429	0.004	0.028	0.426	0.354	3.77E-07
ZNF579	0.103	0.262	0.211	0.056	0.72	3.80E-07
MYO1E	0.532	0.461	0.16	0.101	0.501	3.84E-07
LRIG3	-0.486	-0.034	-0.193	-0.257	0.137	3.87E-07
C1orf109	-0.367	-0.18	-0.177	-0.282	-0.555	3.90E-07
IMPA1	0.005	-0.062	-0.291	-0.304	-0.379	3.93E-07
RP11-1152H	0.323	0.231	-0.07	-0.656	-1.096	3.94E-07
RP11-555K1	-0.554	-0.062	-0.012	-0.632	-0.682	3.95E-07
SORBS1	0.504	0.174	0.178	0.15	0.626	3.96E-07
SPIN3	-0.677	0.138	0.145	-0.367	0.1	3.96E-07
TAF10	-0.16	-0.052	-0.157	-0.149	0.451	3.99E-07
DHX29	0.232	0.075	0.06	0.5	0.31	3.99E-07
CCDC191	-0.741	0.1	0.481	-0.015	0.301	3.99E-07
CCDC84	-0.251	0.046	0.129	-0.582	-0.545	4.08E-07
PCCB	0.302	0.069	0.123	0.495	0.244	4.09E-07
TRMT10B	-0.353	0.059	0.011	-0.454	-0.491	4.11E-07
PYROXD1	0.172	-0.038	-0.064	0.339	-0.264	4.13E-07
ANXA5	0.428	0.174	0.067	0.489	0.469	4.17E-07
RP11-566K1	-0.345	-0.015	-0.024	0.287	0.263	4.19E-07
RP11-932O9	-1.306	-0.798	-0.66	-1.064	-1.235	4.19E-07
ANGPT2	0.408	0.112	0.193	0.557	0.403	4.21E-07
RP11-395P1	-0.481	-0.234	0.268	-0.625	-0.422	4.21E-07
RP11-180M1	-0.911	0.076	0.282	-0.776	-0.847	4.21E-07
DCAF4	0.078	-0.074	0.203	0.222	0.501	4.23E-07
ZNF277	-0.276	0.014	0.109	0.022	0.417	4.23E-07
NBPF15	-0.376	-0.218	0.186	-0.431	-0.06	4.23E-07
CMTR2	-0.373	0.057	0.026	-0.082	-0.16	4.23E-07
ANKS3	-0.597	-0.056	0.036	-0.511	-0.425	4.23E-07
PELI3	0.154	0.541	0.034	-0.397	0.036	4.26E-07

NDUFAF5	-0.282	-0.165	-0.145	-0.707	-0.529	4.30E-07
CAPN10	-0.515	-0.062	-0.056	-0.447	-0.276	4.32E-07
RBAK	0.053	0.346	0.109	-0.076	-0.111	4.33E-07
ANKRD34A	-0.8	0.15	0.27	0.262	-0.05	4.34E-07
PDIA6	0.28	0.032	-0.075	0.339	0.065	4.35E-07
XPNPEP1	0.19	0.081	0.025	0.208	0.392	4.38E-07
CAMSAP1	0.325	0.004	-0.067	0.02	0.228	4.39E-07
FAAP100	-0.312	-0.145	-0.112	-0.328	-0.406	4.40E-07
MIR3653	-0.371	0.35	0.292	-0.669	-0.735	4.41E-07
PPT2	0.03	0.166	0.065	0.114	-0.35	4.42E-07
TYRO3P	0.589	-0.063	-0.291	-0.075	-0.182	4.45E-07
HMGA1L6	-0.128	-0.464	-0.058	0.336	0.749	4.46E-07
RP11-732A1	-0.807	-0.098	0.251	-0.643	-0.17	4.47E-07
GIN1	-0.609	-0.073	-0.021	-0.159	-0.573	4.49E-07
PDIA2	-0.097	-0.173	-0.522	-1.21	-0.776	4.50E-07
GS1-309P15	0.284	-0.046	-0.402	-0.24	-0.307	4.54E-07
DDX5	-0.097	0.18	0.165	-0.365	-0.216	4.57E-07
SEC61G	-0.286	-0.167	-0.038	-0.601	-0.998	4.59E-07
CUEDC2	0.096	0.065	0.049	0.28	0.463	4.61E-07
RP5-1086D1	-0.953	-0.666	-0.214	-0.705	-0.386	4.63E-07
CTC-308K20	0.091	-0.007	0.03	0.214	0.392	4.66E-07
C19orf44	-0.727	0.107	0.135	-0.045	-0.125	4.67E-07
DTX2	-0.416	0.263	0.106	0.201	0.26	4.70E-07
HOXB6	-0.424	-0.36	-0.24	-0.424	-0.347	4.70E-07
RTN3	0.173	0.052	-0.027	0.25	0.352	4.71E-07
FARP1	0.027	0.036	0.102	0.142	0.323	4.72E-07
METTL7A	-0.515	-0.55	-0.44	-0.092	-0.512	4.74E-07
RBBP7	0.182	0.07	0.015	0.314	0.4	4.79E-07
RP11-215C7	0.365	-0.02	-0.176	0.212	0.184	4.79E-07
CHMP3	0.342	0.143	0.104	0.298	0.309	4.81E-07
CTNNA1	0.482	0.208	0.078	0.499	0.679	4.83E-07
CASK	0.188	0.068	0.11	0.328	0.57	4.83E-07
TMEM80	-0.568	-0.274	-0.135	-0.184	-0.374	4.83E-07
ASNSD1	-0.129	-0.034	-0.12	-0.336	-0.596	4.88E-07
GOT2	0.296	0.081	0.026	0.443	0.523	4.93E-07
ZNF780B	-0.647	-0.08	0.059	-0.352	-0.171	4.93E-07
RAB5A	0.418	0.157	-0.034	0.121	0.207	4.95E-07
SH3TC2	-0.151	-0.176	0.307	0.064	-0.47	5.01E-07

CCDC152	0.149	-0.009	-0.047	0.336	-0.693	5.04E-07
ZNF684	-0.37	-0.243	-0.477	-0.639	-0.92	5.04E-07
MMP7	0.055	-0.042	-0.594	-0.389	-1.345	5.06E-07
KIF21A	0.204	0.431	0.476	0.218	0.186	5.07E-07
ITPR1	0.115	0.021	-0.325	-0.239	-0.414	5.07E-07
EPB41L4A	0.39	0.013	-0.153	-0.049	0.197	5.12E-07
G3BP1	0.466	0.062	0.001	0.394	0.264	5.15E-07
ANAPC10	-0.143	-0.03	-0.012	-0.279	-0.759	5.15E-07
PRPF4	0.098	0.083	-0.1	-0.002	-0.392	5.17E-07
TSNARE1	-0.268	0.052	0.561	0.478	0.568	5.19E-07
TUBA1C	0.059	-0.225	-0.326	0.009	-0.241	5.20E-07
ENTPD1	0.333	-0.055	-0.052	-0.169	0.951	5.21E-07
GUK1	-0.177	-0.138	-0.177	-0.409	-0.352	5.21E-07
OSBPL6	-0.284	-0.481	-0.597	-0.472	-0.171	5.25E-07
PAFAH1B3	-0.162	-0.153	-0.215	0.162	0.354	5.28E-07
PDLIM4	0.235	0.026	-0.003	0.317	0.441	5.29E-07
LYRM1	-0.306	0.078	0.472	0.323	0.386	5.30E-07
CIR1	0.307	0.357	0.166	0.587	0.509	5.31E-07
THAP4	0.138	-0.172	-0.218	0.158	0.34	5.39E-07
SPTSSA	-0.203	-0.161	-0.177	-0.449	-0.553	5.39E-07
DND1	-0.193	-0.096	0.05	-1.073	-0.854	5.40E-07
TAGLN2P1	0.377	0.022	0.053	0.418	0.448	5.41E-07
RP11-108M1	0.084	-0.029	0.166	0.349	0.524	5.43E-07
TMED2	-0.05	-0.077	-0.128	-0.335	-0.454	5.45E-07
FKBPL	-0.344	0.002	-0.162	0.167	-0.627	5.48E-07
TGFB1	0.442	0.078	-0.187	-0.017	0.194	5.50E-07
AMZ2P1	-0.53	-0.031	-0.06	-0.642	-0.766	5.51E-07
PUS1	-0.355	-0.16	-0.081	-0.291	-0.419	5.52E-07
RP5-1070A1	0.118	-0.186	-0.269	-0.273	-0.612	5.53E-07
METAP2	0.452	0.198	0.076	0.406	0.252	5.55E-07
HIST1H2BD	0.147	0.264	0.759	1.123	1.246	5.56E-07
CYB5RL	-0.368	-0.071	0.176	-0.016	-0.392	5.61E-07
SLC35C2	0.155	0.283	0.348	0.098	0.299	5.63E-07
DIS3L	-0.024	-0.236	0.091	0.336	0.059	5.69E-07
PRKY	0.014	0.286	0.544	-0.365	0.065	5.70E-07
CYP2S1	-0.306	-0.111	0.2	0.362	0.044	5.70E-07
FAM65A	0.341	0.158	-0.023	-0.041	0.142	5.72E-07
TBP	-0.015	-0.048	-0.044	-0.301	-0.398	5.78E-07

PAQR6	-0.16	0.424	0.107	-0.619	-0.87	5.78E-07
CHRNA5	0.162	0.12	-0.252	-0.143	-0.512	5.82E-07
SLCO3A1	0.27	0.303	0.463	0.613	0.893	5.85E-07
RARG	0.33	-0.183	-0.176	0.097	-0.207	5.85E-07
ZC3HAV1L	-0.637	-0.225	-0.127	-0.413	-0.217	5.85E-07
GLRX	-0.067	-0.496	-0.384	-0.276	-0.289	5.85E-07
PIGF	-0.225	-0.107	-0.075	-0.349	-0.649	5.88E-07
ARID3B	-0.171	0.184	0.019	-0.433	-0.438	5.89E-07
KRTCAP2	0.048	-0.111	-0.125	-0.162	-0.569	5.94E-07
RP11-121L1(	0.099	-0.242	-0.13	0.069	-0.352	5.97E-07
RP4-758J18.	-0.164	-0.066	0.191	-0.601	-0.793	5.98E-07
LSM6	-0.213	-0.21	-0.101	-0.381	-0.818	6.06E-07
NUB1	0.286	0.201	0.152	0.406	0.47	6.10E-07
SNRNP48	-0.033	-0.024	-0.192	-0.37	-0.352	6.17E-07
ZNF280B	-0.483	-0.057	-0.377	-0.479	-0.907	6.17E-07
HYOU1	0.288	0.029	-0.149	0.105	-0.406	6.22E-07
SCAMP1	0.105	-0.046	-0.011	0.142	0.341	6.23E-07
DPYSL4	0.108	0.364	0.18	0.16	0.859	6.26E-07
PCNP	0.169	0.228	0.219	0.199	0.375	6.26E-07
PAPD7	-0.137	-0.299	-0.275	-0.524	-0.369	6.27E-07
GLG1	0.261	0.073	-0.063	0.295	0.485	6.28E-07
BROX	0.296	0.329	0.235	0.349	0.317	6.29E-07
RP11-122K1	-0.746	-0.005	-0.077	-0.96	-0.786	6.29E-07
CTSL	0.255	0.063	0.005	0.425	0.307	6.31E-07
GTF2E1	-0.371	-0.023	-0.125	0.132	0.126	6.32E-07
HCG11	0.259	0.318	0.468	0.79	0.626	6.39E-07
ZPR1	0.329	0.149	0.046	0.146	0.109	6.45E-07
MRPL18	-0.19	-0.19	-0.037	-0.069	-0.417	6.45E-07
PSMC2	0.385	0.22	0.046	0.38	0.059	6.48E-07
RP11-809O1	-0.434	0.271	0.753	0.552	0.647	6.49E-07
MYNN	-0.134	0.025	0.046	-0.293	-0.467	6.52E-07
CNTNAP3	-0.493	-0.2	0.008	-0.354	-0.016	6.61E-07
LRRC45	-0.735	-0.266	-0.057	-0.351	-0.366	6.65E-07
FBXL8	-0.393	0.264	0.282	-0.508	-0.178	6.67E-07
TERF2IP	0.245	0.166	-0.098	0.263	0.406	6.68E-07
VIP	-0.049	0.786	1.26	0.29	0.451	6.69E-07
USE1	0.036	0.174	0.164	0.003	-0.332	6.69E-07
LMF2	-0.113	0.064	-0.055	-0.181	-0.348	6.70E-07

SCLT1	-0.327	-0.406	-0.298	0.067	-0.061	6.79E-07
LARP4	-0.05	-0.009	-0.234	-0.209	-0.471	6.81E-07
PWP1	0.3	0.101	0.043	0.384	0.27	6.82E-07
PXK	0.364	0.01	-0.043	0.112	0.32	6.86E-07
MORC3	0.261	0.287	0.19	0.186	0.403	6.91E-07
GS1-251I9.4	0.058	-0.002	-0.201	-0.029	-0.384	6.91E-07
MED14	0.147	0.133	0.132	0.356	0.443	6.94E-07
MALAT1	-0.375	0.173	0.389	-0.571	-0.353	6.94E-07
NDUFS2	0.134	-0.011	0.035	0.341	0.237	6.95E-07
SEMA3C	0.09	0.345	0.199	0.403	-0.057	7.08E-07
SNRPD1	0.003	-0.232	-0.224	-0.181	-0.425	7.10E-07
POLR2H	-0.387	-0.118	0.016	-0.34	-0.39	7.11E-07
GOLT1B	-0.102	-0.015	-0.055	-0.576	-0.615	7.13E-07
EPN1	0.142	-0.02	-0.051	0.175	0.392	7.17E-07
TMEM185A	0.031	-0.005	0.072	0.176	0.501	7.26E-07
CTSD	0.224	0.175	0.059	0.539	0.634	7.33E-07
RP11-85K15	0.404	0.023	0.284	0.42	0.9	7.37E-07
ZNF211	-0.033	0.225	0.11	-0.273	-0.326	7.40E-07
AC139713.1	0.339	-0.32	-0.652	-0.472	-0.937	7.40E-07
LRP5L	-0.403	-0.201	-0.071	-0.939	-0.791	7.44E-07
PRKAB2	0.104	0.347	0.227	-0.168	-0.112	7.57E-07
RP1-39G22.7	-0.501	-0.216	-0.193	-0.45	-1.145	7.57E-07
ARC	0.638	0.405	0.592	0.415	0.665	7.61E-07
USHBP1	-0.163	0.271	0.425	-0.046	0.132	7.61E-07
SENP1	0.126	0.082	0.059	0.042	-0.352	7.62E-07
CPSF3	0.196	0.144	0.126	0.487	0.158	7.67E-07
PANX2	0.001	0.098	-0.053	-0.067	-0.758	7.67E-07
POLD1	0.044	0	-0.187	-0.209	-0.432	7.70E-07
RLIM	0.366	0.088	-0.05	0.159	0.058	7.86E-07
OMA1	-0.137	-0.153	0.157	0.264	-0.492	7.86E-07
SPSB3	-0.357	-0.019	-0.007	-0.23	0.202	7.88E-07
SUSD1	0.343	0.121	0.128	0.356	0.458	7.90E-07
C1orf233	-0.493	-0.402	-0.214	-0.136	0.673	7.93E-07
NUP37	-0.141	-0.253	-0.233	-0.171	-0.457	8.00E-07
ZNF530	0.135	-0.303	-0.193	-0.353	-0.781	8.02E-07
XXbac-BPG2	-0.323	-0.104	0.282	-0.084	0.436	8.03E-07
RP11-21K12	-0.521	0.048	0.116	-0.87	-0.814	8.17E-07
ZNF587B	-0.19	0.065	-0.068	-0.202	-0.601	8.24E-07

WDR82	0.347	-0.008	-0.03	0.266	0.334	8.26E-07
MECP2	0.258	0.03	0.086	0.148	0.362	8.27E-07
PRRC1	0.548	0.171	0.12	0.338	0.447	8.37E-07
P4HB	0.21	0.077	-0.027	0.373	0.416	8.47E-07
RSU1	0.402	0.046	-0.102	0.25	0.345	8.50E-07
MTF2	-0.035	-0.153	-0.368	-0.251	-0.168	8.58E-07
CTA-390C10	-0.498	-0.077	0.435	-0.103	0.612	8.61E-07
TMEM68	0.047	0.472	0.434	-0.128	0.089	8.69E-07
SETD4	-0.471	0.046	0.13	-0.377	-0.389	8.69E-07
RP11-428C1	0.417	0.501	0.289	-0.013	-0.361	8.71E-07
FAM84B	-0.314	0.1	-0.267	-0.334	-0.401	8.74E-07
CEP350	-0.145	0.064	-0.068	-0.378	-0.437	8.77E-07
ABCB1	0.051	0.453	0.074	0.27	-0.597	8.77E-07
AC007246.3	-0.372	-0.122	0.086	0.058	0.875	8.83E-07
RBM15	0.147	-0.019	-0.036	0	-0.414	8.83E-07
TARBP2	-0.258	-0.29	-0.243	-0.317	-0.414	8.99E-07
CFAP44	-0.473	0.181	0.395	-0.479	-0.183	9.01E-07
TECPR2	-0.044	0.188	0.194	0.236	0.415	9.03E-07
ZNF133	-0.059	0.332	0.227	-0.207	-0.238	9.03E-07
AMD1	-0.137	-0.291	-0.34	-0.352	-0.368	9.05E-07
TUBA8	0.211	-0.257	-0.292	-0.083	-0.448	9.05E-07
POLR2K	-0.098	0.044	0.081	-0.295	-0.444	9.09E-07
RP13-136L2.	0.337	-0.032	-0.129	0.181	0.021	9.10E-07
GPX7	0.106	-0.09	-0.183	-0.128	-0.437	9.16E-07
KDM3B	0.07	0.039	0.022	0.175	0.441	9.26E-07
CTD-3222D1	-0.402	-0.367	-0.301	-1.205	-0.97	9.27E-07
SPATA5	-0.112	0.252	-0.031	-0.012	-0.345	9.29E-07
CCNK	0.431	0.36	0.282	0.344	0.38	9.31E-07
VCPIP1	0.46	0.394	0.129	0.318	0.384	9.46E-07
PTK2B	0.15	0.131	0.417	0.597	0.322	9.49E-07
USP4	0.089	0.093	0.24	0.369	0.214	9.57E-07
RP11-416K2	0.204	-0.153	-0.115	-0.008	-0.477	9.63E-07
RP11-772E1	-0.137	-0.341	-0.211	-0.744	-0.796	9.68E-07
ITGB1BP2	-0.523	0.008	-0.014	-0.975	-1.203	9.70E-07
MRPS7	-0.067	-0.107	-0.147	-0.042	-0.334	9.73E-07
RP11-152F1	-0.593	0.008	0.002	-0.847	-0.73	9.76E-07
DHRS12	-0.029	0.084	0.054	0.031	0.941	9.79E-07
MSH2	0.409	0.105	-0.162	0.062	-0.232	9.82E-07

RPP25L	-0.405	-0.22	-0.161	0.165	-0.314	9.82E-07
TDRKH	-0.087	-0.016	0.064	0.441	0.491	9.94E-07
EIF2B5	-0.072	-0.037	-0.009	-0.023	-0.343	9.95E-07
GAPDH	0.307	0.099	0.069	0.482	0.459	1.01E-06
RP11-473N1	0.37	-0.109	-0.114	0.197	-0.231	1.01E-06
CUTC	-0.104	-0.108	-0.003	0.126	-0.428	1.01E-06
SWT1	0.05	0.484	0.748	0.519	0.569	1.02E-06
SMAD4	0.271	0.179	0.108	0.152	0.348	1.02E-06
GALT	-0.598	-0.092	0.053	-0.311	-0.144	1.02E-06
DISC1	0.391	-0.206	0.198	0.238	0.483	1.03E-06
AC007238.1	0.383	0.122	0.132	0.579	0.408	1.03E-06
CTD-2324A2	0.289	-0.01	0.013	0.427	0.379	1.03E-06
ULBP1	0.503	0.92	0.93	1.013	0.342	1.03E-06
MIR3191	0.909	1.228	1.391	0.48	0.84	1.04E-06
ARID3A	0.053	0.153	0.198	0.223	0.573	1.04E-06
CTD-2017F1	-0.469	-0.158	0.072	-0.983	-1.014	1.04E-06
PRPF40A	0.362	0.252	0.08	0.311	0.306	1.06E-06
SP2	0.505	0.119	0.093	0.185	-0.012	1.06E-06
RP5-854E16	-0.049	-0.106	0.013	-0.388	-0.488	1.06E-06
PPCDC	-0.322	-0.044	0.054	-0.218	-0.564	1.06E-06
MTFP1	-0.17	-0.162	-0.111	-0.08	-0.61	1.06E-06
RP11-303E1	-0.452	-0.011	0.24	-0.809	-0.913	1.06E-06
ZNF189	-0.269	0.005	-0.227	-0.479	-0.238	1.07E-06
DHH	0.072	-0.306	-0.484	-0.122	-0.392	1.07E-06
PSEN2	0.139	0.013	0.098	0.394	0.613	1.08E-06
TLK1	0.176	0.046	0.056	0.157	0.349	1.09E-06
AGPS	0.412	-0.032	-0.163	0.145	0.159	1.10E-06
PINLYP	-0.855	0.241	0.416	-0.328	-0.214	1.10E-06
NORAD	0.365	0.302	0.191	0.355	0.461	1.12E-06
LY6E	0.051	-0.014	-0.016	0.287	0.437	1.12E-06
CPNE7	-0.311	-0.073	0.045	-0.716	0.045	1.12E-06
FAM45A	-0.069	-0.028	-0.113	0.07	0.461	1.13E-06
CARS2	-0.127	-0.089	-0.102	-0.392	-0.334	1.13E-06
OSGEPL1	-0.535	-0.356	-0.032	0.164	-0.378	1.13E-06
SMO	0.004	0.067	-0.605	-0.842	-0.569	1.13E-06
RP11-298C3	0.139	-0.116	-0.165	0.013	-0.355	1.14E-06
ZNF777	0.391	-0.02	-0.105	0.201	0.465	1.15E-06
FBXO33	0.514	0.39	0.275	0.295	0.309	1.15E-06

ARHGAP27	-0.028	-0.02	0.253	-0.385	0.094	1.15E-06
IKBKE	0.091	-0.036	0.057	-0.001	0.337	1.16E-06
SNX2	0.327	0.231	0.102	0.514	0.43	1.17E-06
SDSL	0.151	0.009	0.11	0.599	0.247	1.17E-06
TMEM42	-0.712	-0.208	0.091	-0.281	-0.123	1.17E-06
ACD	0.04	-0.074	-0.333	-0.308	-0.39	1.17E-06
DUX4L50	0.717	1.317	1.246	0.812	1.544	1.18E-06
KB-431C1.4	-0.163	0.592	0.916	0.54	0.926	1.18E-06
SGSH	-0.075	0.053	-0.092	-0.034	-0.33	1.18E-06
CKS2	-1.135	-0.916	-0.563	-0.96	-0.798	1.18E-06
RP11-566E1	0.233	-0.127	-0.205	-0.146	0.75	1.20E-06
AK5	0.208	-0.055	0.115	0.612	0.206	1.20E-06
PTBP2	-0.21	-0.007	-0.047	-0.683	-0.449	1.20E-06
AHCYL1	0.332	0.151	0.091	0.306	0.384	1.21E-06
DDRGK1	0.006	-0.002	-0.031	0.106	-0.328	1.21E-06
DPAGT1	-0.189	-0.212	-0.213	-0.251	-0.364	1.21E-06
DARS2	0.06	-0.045	0.021	0.483	0.192	1.22E-06
ACAD11	-0.654	0.042	0.41	-0.489	-0.013	1.22E-06
CSDE1	0.338	0.134	0.079	0.299	0.393	1.23E-06
C16orf13	-0.353	-0.153	-0.1	-0.28	0.098	1.23E-06
CTD-3184A7	-0.456	-0.109	0.014	-0.728	-0.895	1.24E-06
GRB2	0.31	0.063	-0.124	0.247	0.371	1.25E-06
TSPAN1	0.153	0.231	0.817	0.798	1.037	1.26E-06
RP11-665C1	0.397	0.106	0.297	0.575	0.808	1.26E-06
MFN1	0.12	0.157	0.155	0.326	0.312	1.26E-06
PAICS	0.352	0.054	-0.193	0.04	-0.145	1.26E-06
RP11-134K1	0.26	-0.353	-0.293	-0.041	0.205	1.27E-06
GPR137C	0.394	0.319	0.07	-0.273	-0.523	1.27E-06
BMS1P4	-0.748	0.006	0.241	-0.82	-0.654	1.27E-06
NPFF	-0.538	-0.196	0.306	-0.756	-0.766	1.27E-06
PPID	0.093	0.096	0.173	0.406	0.303	1.30E-06
RP1-159M24	0.455	0.055	0.152	0.541	-0.101	1.30E-06
ACCS	-0.77	-0.1	0.07	-0.633	-0.146	1.30E-06
TMEM39A	-0.064	-0.042	-0.023	-0.377	-0.328	1.31E-06
NBPF20	-0.448	0.021	0.152	-0.551	-0.347	1.31E-06
ARMCX5	0.067	0.037	-0.007	0.074	-0.411	1.31E-06
RP11-446E9	-0.025	0.065	-0.033	-0.042	-0.332	1.32E-06
RP11-390P2	-0.575	-0.264	-0.356	-0.861	-0.898	1.32E-06

PTOV1	0.115	0.083	0.05	0.121	0.428	1.33E-06
PDIA3P1	0.365	0.096	0.019	0.468	0.335	1.33E-06
FAM86HP	-0.526	0.381	0.791	0.009	0.175	1.33E-06
HNRNPA0	0.327	-0.084	-0.239	0.132	0.092	1.34E-06
TMEM126A	-0.273	-0.147	-0.113	-0.466	-0.754	1.35E-06
C11orf96	1.024	-0.122	0.038	0.381	0.954	1.36E-06
SF3A3	0.385	0.189	0.126	0.468	0.354	1.36E-06
PKN3	-0.235	-0.179	-0.094	-0.194	-0.482	1.36E-06
DLEU1	-0.315	-0.323	-0.3	-0.351	-0.831	1.37E-06
KIFC3	0.194	-0.05	0.041	0.23	0.351	1.38E-06
SDHA	0.253	0.198	0.196	0.68	0.298	1.39E-06
DMXL2	-0.196	0.014	0.117	-0.101	-0.347	1.39E-06
RP11-216L1:	-0.662	-0.037	-0.135	-0.588	-1.024	1.39E-06
SDE2	0.382	0.096	-0.142	-0.004	-0.097	1.40E-06
MFSD4B	-0.544	0.053	0.162	-0.067	-0.141	1.40E-06
LIAS	-0.276	-0.108	0.063	-0.216	-0.531	1.40E-06
SNORA10	-0.376	0.042	-0.283	-0.974	-1.286	1.40E-06
RP11-351N4	0.385	-0.125	0.064	0.555	0.383	1.41E-06
BCLAF1P2	0.466	0.098	-0.078	0.191	-0.191	1.41E-06
RP11-55L3.2	0.354	-0.041	0.018	0.396	0.609	1.45E-06
C14orf156	-0.315	-0.307	-0.228	-0.593	-0.671	1.45E-06
RP11-5C23.:	-0.519	-0.057	-0.122	-0.84	-0.809	1.45E-06
CARF	-0.294	0.125	0.599	-0.007	0.025	1.47E-06
AC006273.5	-0.602	-0.23	0.505	-0.314	-0.408	1.47E-06
SYBU	0.005	-0.237	-0.429	-0.471	-0.807	1.47E-06
VWF	0.31	0.056	-0.067	0.24	0.558	1.48E-06
RP11-153A2	-0.424	-0.295	0.438	0.018	0.035	1.48E-06
ARFGEF3	0.502	0.051	0.085	0.167	0.027	1.48E-06
STPG1	0.068	0.067	0.422	0.62	0.438	1.49E-06
RP11-902B1	-0.39	0.091	0.425	-0.122	-0.166	1.49E-06
LRP6	-0.008	-0.14	-0.268	-0.259	-0.358	1.49E-06
RP11-392O1	-0.776	-0.395	-0.048	-0.491	0.581	1.50E-06
RP3-523C21	-0.642	-0.486	-0.45	-0.678	-0.806	1.51E-06
PRKAR1A	0.408	0.167	0.085	0.462	0.484	1.53E-06
SEL1L3	0.255	0.105	0.089	0.375	0.311	1.53E-06
RP11-568J2:	-0.191	-0.069	1.069	0.176	0.86	1.54E-06
HTR1D	0.774	0.482	0.78	1.043	0.603	1.54E-06
ARSB	0.295	0.194	-0.001	0.268	0.323	1.54E-06

KLF5	0.944	0.456	0.351	0.407	-0.102	1.54E-06
CEP85L	0.287	0.451	0.353	-0.123	0.244	1.55E-06
TTC39C	0.477	0.608	0.612	0.409	0.905	1.56E-06
RP5-905H16	-0.287	0.234	0.096	-1.052	-0.492	1.56E-06
CYB5R4	-0.067	0.1	-0.127	-0.229	-0.533	1.56E-06
HNRNPD	0.347	0.13	0.022	0.128	-0.126	1.57E-06
MED18	-0.378	-0.252	-0.107	0.084	-0.263	1.57E-06
PPP2R3C	0.021	0.055	-0.043	-0.097	-0.389	1.58E-06
MYBBP1A	0.013	-0.116	-0.117	-0.122	-0.463	1.58E-06
RP11-46D6.1	-0.067	-0.332	-0.069	0.165	0.678	1.59E-06
NOCT	0.306	0.251	-0.058	0.028	0.446	1.60E-06
ABHD4	0.398	0.515	0.352	0.447	0.423	1.60E-06
AC005822.1	0.283	-0.081	-0.071	-0.439	-0.249	1.61E-06
AC073150.4	0.398	0.241	0.199	0.531	0.518	1.62E-06
CWC27	0.314	0.059	0.138	0.393	0.379	1.62E-06
MTERF4	-0.09	0.048	0.057	-0.159	-0.327	1.62E-06
C18orf54	-0.252	-0.12	-0.204	-0.071	-0.426	1.62E-06
MIR5587	-0.479	-0.189	-0.031	-1.134	-1.207	1.63E-06
SNORD46	-0.331	-0.281	-0.197	-1.113	-1.256	1.63E-06
NCAPD2	-0.127	-0.221	-0.099	0.35	0.193	1.64E-06
SRRM1	0.328	0.156	-0.006	0.05	0.009	1.64E-06
SLC25A37	-0.283	-0.288	0.205	-0.52	-0.29	1.64E-06
TUT1	-0.483	-0.25	-0.092	-0.01	-0.359	1.64E-06
SLC25A16	0.045	0.018	-0.064	-0.213	-0.405	1.65E-06
QTRT2	-0.079	-0.066	-0.142	-0.113	-0.418	1.65E-06
IFI35	0.005	-0.148	-0.076	0.356	0.446	1.66E-06
RP11-45F23	0.548	-0.068	-0.285	0.456	0.465	1.68E-06
TTC7A	0.186	-0.06	0.22	0.193	0.363	1.68E-06
UBE2HP	0.187	0.217	0.405	0.537	1.131	1.69E-06
USO1	0.297	0.11	0.115	0.414	0.252	1.69E-06
H2AFJ	-0.104	-0.18	-0.139	0.13	-0.422	1.69E-06
ZNF224	-0.144	0.21	0.197	-0.39	-0.181	1.71E-06
ZNF718	-0.071	-0.116	-0.478	-0.559	-0.44	1.71E-06
MXI1	0.35	0.288	0.366	0.263	0.891	1.72E-06
SPAG16	0.067	-0.148	0.15	0.292	0.683	1.72E-06
ORMDL1	-0.336	0.127	0.05	-0.47	-0.425	1.72E-06
MIR570	0.117	0.597	0.404	-0.529	-0.48	1.72E-06
C4orf33	-0.276	-0.184	-0.27	-0.451	-0.815	1.72E-06

ZNF524	-0.254	0.037	0.094	0.213	0.365	1.73E-06
PRC1	-0.242	-0.493	-0.586	-0.188	-0.327	1.73E-06
RP11-418J1	-0.059	0.392	0.555	0.352	0.447	1.75E-06
GNB1	0.374	0.08	-0.071	0.24	0.413	1.75E-06
PMM2	0.436	0.167	0.009	0.228	0.172	1.76E-06
TRBC2	-0.369	-0.124	0.192	0.336	0.178	1.78E-06
ANKRD39	-0.066	-0.145	-0.234	-0.187	-0.661	1.78E-06
MBD5	0.378	0.46	0.531	0.434	0.55	1.80E-06
ABCB7	0.121	0.075	0.11	0.334	0.37	1.80E-06
ACTR3	0.393	0.115	-0.026	0.319	0.332	1.80E-06
EIF4H	0.425	0.289	0.227	0.528	0.472	1.81E-06
ZMAT1	-0.76	-0.034	0.398	-0.173	0.133	1.82E-06
PHC1B	-0.154	0.059	0.085	-0.379	-0.294	1.82E-06
RP11-402L1	0.397	-0.119	-0.201	0.323	0.357	1.84E-06
ZSCAN20	-0.482	0.054	0.186	-0.289	-0.186	1.85E-06
LGMN	0.319	0.145	-0.001	0.442	0.375	1.86E-06
ITGB1BP1	-0.241	-0.144	-0.033	-0.303	-0.475	1.88E-06
RP11-304L1	-0.124	-0.077	-0.122	-1.252	-0.828	1.88E-06
FAF1	0.337	0.055	0.072	0.421	0.138	1.89E-06
KMT2A	-0.152	0.092	0.06	-0.347	0.08	1.93E-06
BAG2	-0.236	-0.264	-0.294	-0.217	-0.378	1.94E-06
MCEE	-0.189	-0.059	-0.038	-0.401	-0.812	1.95E-06
CCDC138	-0.348	-0.52	-0.435	-0.509	-0.894	1.95E-06
RP11-399O1	0.944	1.072	1.061	0.452	1.385	1.99E-06
TARS	0.319	0.06	0.011	0.468	0.356	1.99E-06
AFG3L2	0.326	0.076	-0.118	0.122	-0.014	1.99E-06
GDAP1	0.504	0.412	0.148	0.145	-0.807	1.99E-06
RBM23	0.25	0.281	0.236	0.449	0.069	2.02E-06
PTPDC1	-0.167	-0.165	-0.191	-0.586	-0.527	2.02E-06
DDR1	0.489	0.287	0.059	0.014	-0.002	2.03E-06
CHST14	0.147	0.249	0.169	0.54	0.408	2.05E-06
ATP6V1B2	0.388	0.259	0.08	0.43	0.433	2.06E-06
SETD1B	0.052	-0.051	-0.178	-0.323	-0.245	2.06E-06
POLR3B	0.334	0.03	0.017	0.335	-0.269	2.06E-06
PLCD1	0.358	0.383	0.363	0.446	0.504	2.07E-06
SNORA57	-0.295	0.05	0.221	-0.944	-0.993	2.09E-06
ARHGAP42	0.039	0.319	-0.085	0.19	0.578	2.11E-06
IL1RAP	0.137	0.255	-0.079	0.106	0.498	2.11E-06

OXCT1	0.159	0.082	0.043	0.374	0.382	2.11E-06
RP11-18I14.1	-0.971	-0.314	-0.03	-0.723	-0.932	2.12E-06
TCF12	0.005	0.015	-0.081	0.185	0.352	2.14E-06
SIN3A	0.451	0.232	-0.025	0.068	0.144	2.14E-06
CTD-2368P2	-0.434	0.225	0.798	-0.077	0.24	2.15E-06
RP1-128M12	0.293	-0.033	-0.01	0.38	0.245	2.16E-06
B3GNT2	0.156	-0.013	-0.326	-0.205	-0.012	2.16E-06
SETD5	-0.037	-0.14	-0.093	-0.407	-0.22	2.16E-06
RP11-1094M	-0.331	-0.578	-0.556	-0.58	-1.158	2.17E-06
RASSF1	-0.178	-0.212	-0.164	-0.184	-0.437	2.18E-06
PLEKHF2	0.363	0.058	-0.04	-0.054	0.083	2.19E-06
RP11-1K11.1	0.238	-0.04	-0.238	0.002	-0.33	2.19E-06
C6orf136	-0.221	-0.006	-0.131	-0.22	-0.592	2.19E-06
DNAJC13	0.182	0.117	0.2	0.368	0.413	2.21E-06
TFRC	0.141	0.041	0.011	0.326	0.386	2.21E-06
MGARP	0.232	0.944	0.672	1.084	1.216	2.23E-06
ALDH4A1	-0.151	0.003	0.086	0.154	0.442	2.23E-06
PSME2	-0.088	0.189	0.183	0.022	0.374	2.23E-06
PCK2	-0.039	0.076	0.244	0.521	0.062	2.23E-06
TAOK1	0.116	0.22	0.024	0.091	0.427	2.26E-06
LDHA	0.333	0.074	0.002	0.426	0.389	2.27E-06
CLEC16A	-0.14	0.044	0.162	0.272	0.325	2.30E-06
RP11-164A7	0.113	0.257	0.099	0.136	0.423	2.31E-06
MOK	-0.292	0.027	-0.018	-0.542	-0.443	2.31E-06
NOXA1	-0.399	0.059	0.912	0.574	0.726	2.32E-06
TSEN34	0.106	0.031	-0.055	0.256	0.429	2.33E-06
CROCC	-0.372	-0.009	0.238	-0.256	-0.179	2.33E-06
AC004057.1	-0.189	-0.141	-0.12	-0.274	-0.567	2.33E-06
KLHL25	0.375	0.02	-0.282	-0.083	0.357	2.34E-06
ACP2	0.062	-0.113	-0.311	0.033	-0.461	2.34E-06
RRAGB	-0.478	-0.237	-0.065	-0.565	-0.382	2.35E-06
LRP1	-0.029	0.446	0.702	0.613	0.88	2.36E-06
NCOA3	0.319	0.303	0.26	0.131	0.324	2.36E-06
SARS	0.304	0.163	0.175	0.542	0.258	2.37E-06
XXbac-BPG5	-0.111	0.185	0.002	-0.289	-0.489	2.38E-06
RP11-50B3.4	-0.502	-0.064	-0.027	-1.143	-0.854	2.38E-06
RUFY3	-0.186	0.024	0.111	-0.498	-0.263	2.39E-06
CWF19L2	0.254	0.261	-0.033	0.346	-0.104	2.40E-06

HMGN3	-0.42	-0.335	-0.239	-0.585	-0.656	2.41E-06
GPD1L	0.169	0.007	-0.031	0.399	0.451	2.42E-06
OSMR	0.12	0.229	0.224	0.473	0.068	2.42E-06
OGFR	-0.392	0.012	-0.092	-0.169	-0.073	2.42E-06
MAD2L2	-0.104	-0.023	-0.104	0.129	-0.414	2.42E-06
LETMD1	-0.444	0.023	0.173	-0.188	-0.07	2.43E-06
AP006621.5	-0.606	-0.027	0.257	-0.466	-0.051	2.44E-06
PAK2	0.31	0.172	0.039	0.292	0.512	2.46E-06
DNAJB9	0.113	0.006	0.224	-0.171	-0.387	2.47E-06
OCIAD1	0.11	0.073	0.098	0.252	0.345	2.48E-06
ARL8A	0.341	0.168	-0.028	0.122	0.295	2.48E-06
GABPB2	0.417	0.091	0.213	0.442	-0.002	2.48E-06
AP3M2	-0.246	-0.342	-0.252	-0.593	-0.295	2.49E-06
ZDHHC8	-0.32	-0.152	-0.01	-0.341	-0.01	2.50E-06
EXD3	-0.47	-0.163	0.104	-0.155	0.188	2.52E-06
AC010878.3	0.158	-0.156	-0.151	0.016	-0.463	2.52E-06
SLC9A3R1	0.203	0.179	0.083	0.453	0.556	2.53E-06
PSMD12P	0.375	-0.066	-0.123	0.247	-0.404	2.53E-06
TIMM9	-0.54	-0.269	-0.094	-0.356	-0.523	2.53E-06
ACHE	0.23	0.719	0.408	0.29	1.071	2.55E-06
PIK3C3	0.265	0.238	0.087	0.302	0.428	2.55E-06
RPP40	0.103	-0.154	-0.239	0.014	-0.41	2.56E-06
RNF220	0.326	-0.052	-0.059	0.166	0.114	2.58E-06
PYGO2	-0.252	-0.07	-0.049	-0.246	-0.439	2.58E-06
ZFAS1	-0.315	-0.156	-0.047	-0.712	-0.635	2.58E-06
GABRE	-0.292	0.103	0.22	-0.771	0.023	2.59E-06
FAM3C	0.334	0.149	-0.012	0.179	0.194	2.63E-06
CTD-2622I13	-0.748	-0.153	-0.168	-1.249	-1.042	2.63E-06
SMIM3	0.065	-0.071	-0.137	-0.143	0.751	2.64E-06
SNRNP25	-0.23	-0.169	-0.163	-0.114	-0.391	2.64E-06
MSX2	0.947	0.455	0.065	0.358	0.437	2.67E-06
RP11-550F7	0.464	-0.028	-0.089	0.355	0.413	2.67E-06
AKAP9	-0.333	0.098	0.139	-0.293	0.108	2.69E-06
RSL1D1	0.242	0.066	0.024	0.343	0.374	2.70E-06
STIM1	0.016	0.031	0.087	0.319	0.418	2.72E-06
DHX32	0.081	0.269	0.073	0.327	0.318	2.73E-06
MRPS12	-0.12	-0.23	-0.265	-0.122	-0.394	2.73E-06
C3orf14	-0.267	0.019	0.058	-0.292	-0.659	2.73E-06

ZNF141	-0.191	-0.05	-0.18	-0.83	-0.276	2.74E-06
LYAR	0.142	0.018	-0.118	0.133	-0.34	2.74E-06
ACTN4	0.275	0.092	0.018	0.416	0.655	2.75E-06
DLD	0.26	0.204	0.098	0.488	0.284	2.75E-06
GNAI3	0.343	0.072	0.026	0.361	0.183	2.75E-06
PRPF38A	0.04	-0.025	-0.056	0.004	-0.351	2.75E-06
SNORD4A	-0.374	-0.019	-0.042	-1.045	-1.071	2.76E-06
GYPC	-0.309	-0.161	-0.035	0.044	0.968	2.77E-06
SNORA44	0.068	0.099	0.15	-0.911	-0.769	2.77E-06
GATAD2B	0.342	0.113	0.147	0.164	0.402	2.79E-06
ENOX2	0.132	0.006	0.183	0.441	0.218	2.79E-06
HSPB11	-0.374	0.008	0.005	-0.493	-0.645	2.79E-06
TBL2	-0.125	-0.254	-0.268	-0.178	-0.368	2.80E-06
RP11-453E1	-0.153	0.135	0.327	0.238	-0.101	2.81E-06
LSM8	-0.209	-0.099	-0.099	-0.549	-0.55	2.81E-06
JADE3	0.404	0.175	0.079	0.225	0.414	2.82E-06
ZNF432	-0.076	0.229	0.21	-0.539	-0.169	2.85E-06
AC093726.6	-0.489	-0.348	0.018	0.03	0.158	2.88E-06
TGFBI	0.451	0.111	-0.171	0.247	0.048	2.89E-06
SNORD50B	-0.042	0.154	0.029	-0.776	-0.869	2.90E-06
RP13-1032I1	-0.203	-0.427	-0.498	-0.814	-1.096	2.92E-06
THNSL1	-0.083	0.014	0.454	0.419	0.507	2.93E-06
RAB7A	0.421	0.161	-0.007	0.378	0.417	2.93E-06
PTCH1	-0.44	-0.52	-0.453	-0.364	-0.055	2.93E-06
NEAT1	-0.522	0.314	0.095	-0.802	-0.096	2.93E-06
CANX	0.35	0.106	-0.05	0.249	-0.127	2.93E-06
TBX1	-0.235	-0.428	-0.127	-0.399	-0.429	2.93E-06
PLEKHM3	0.556	0.401	0.215	0.25	0.274	2.94E-06
EHD2	0.326	0.089	0.08	0.563	0.679	2.95E-06
FOXM1	-0.208	-0.315	-0.334	-0.037	-0.388	2.97E-06
HCLS1	0.413	0.12	0.015	0.406	0.56	2.98E-06
RP11-257O5	-0.369	0.026	0.158	-0.72	-0.423	2.98E-06
LRRCC1	0.031	-0.043	-0.161	-0.057	-0.655	2.99E-06
RP1-239B22	0.513	0.203	0.212	-0.1	-0.248	3.00E-06
FUT4	0.039	0.108	0.097	0.114	0.512	3.01E-06
AC010129.1	0.36	-0.026	-0.17	0.178	-0.015	3.02E-06
DDX43	0.313	0.019	0.414	0.22	0.993	3.04E-06
IER3IP1	-0.037	-0.043	-0.141	-0.357	-0.474	3.04E-06

RP11-20I23.:	-0.397	-0.204	-0.053	-1.072	-0.768	3.04E-06
CTD-2002H8	0.003	0.589	0.497	-0.693	-0.507	3.06E-06
APTX	-0.063	0.036	-0.006	-0.033	-0.325	3.07E-06
ATP6V0D1	0.332	0.097	-0.013	0.45	0.433	3.08E-06
SRD5A1	0.033	-0.03	0.044	-0.407	0.082	3.10E-06
AC009948.5	-0.665	-0.305	-0.044	-0.221	-0.237	3.11E-06
OSTC	-0.253	-0.173	-0.18	-0.588	-0.685	3.11E-06
KIFAP3	0.302	0.135	0.071	0.52	0.573	3.13E-06
RP11-553A1	0.495	0.86	0.945	1.158	0.996	3.18E-06
POLR3E	-0.342	-0.05	-0.013	-0.09	-0.258	3.18E-06
BACE1	0.208	0.065	0.029	0.133	0.364	3.21E-06
RNF26	-0.16	-0.415	-0.467	-0.012	-0.162	3.21E-06
SLC2A13	0.012	0.171	-0.243	-0.452	-0.471	3.23E-06
LIPE	-0.515	-0.46	-0.603	-0.452	-0.825	3.24E-06
MAPK8IP1	0.246	0.24	0.189	0.495	1.03	3.27E-06
COQ7	0.163	-0.036	0.033	-0.005	-0.334	3.27E-06
PTGER1	-0.342	-0.301	-0.412	-0.252	0.729	3.28E-06
AC008746.1	-0.338	0.31	0.429	-0.474	-0.574	3.28E-06
PMVK	0.109	-0.002	0.011	0.371	-0.052	3.29E-06
HDAC10	-0.531	-0.188	-0.062	-0.705	-0.43	3.29E-06
RP11-325K4	-0.563	-0.056	-0.038	-1.153	-0.825	3.29E-06
CHPF	0.274	0.126	-0.14	-0.046	-1.057	3.31E-06
BRWD1	0.121	0.323	0.282	-0.079	0.186	3.33E-06
ZNF503	0.565	0.06	-0.017	0.127	0.54	3.34E-06
RANBP9	0.413	0.125	-0.107	0.075	0.001	3.34E-06
RP11-384M2	-0.952	0.04	-0.124	-0.747	-0.941	3.34E-06
SEMA6C	-0.232	-0.166	-0.237	-0.504	-0.407	3.35E-06
FAM175A	0.031	0.206	0.367	0.36	0.179	3.36E-06
RP11-380M2	-0.026	-0.286	0.326	-0.817	-1.102	3.36E-06
PPP1R2	0.357	0.25	0.07	0.186	0.12	3.37E-06
RP11-469O1	0.305	0.067	0.128	0.575	0.656	3.38E-06
ISG15	0.089	-0.046	0.038	0.302	0.469	3.38E-06
PSMC4	0.387	0.199	0.018	0.357	-0.063	3.38E-06
EXOC6B	0.474	0.184	0.226	0.546	0.326	3.41E-06
SLC35F6	-0.044	-0.115	-0.236	-0.016	-0.364	3.41E-06
GEMIN7	-0.355	-0.216	-0.108	-0.344	-0.604	3.41E-06
RP4-667H12	0.208	0.107	0.08	0.505	0.676	3.42E-06
OTUD1	0.207	0.299	-0.136	0.132	0.797	3.43E-06

MVB12B	0.38	0.185	0.036	0.376	0.552	3.47E-06
RP13-147D1	0.21	-0.415	-0.058	0.256	0.256	3.48E-06
TBRG4	-0.182	-0.069	-0.015	0.015	-0.331	3.50E-06
THRA	0.059	-0.013	0.107	0.45	0.404	3.51E-06
PCCA	0.187	0.005	0.133	0.229	0.484	3.54E-06
RP3-508I15.	-0.022	-0.022	0.477	-0.867	-0.819	3.54E-06
RP5-1086K1	-0.439	-0.132	-0.078	-1.066	-0.925	3.54E-06
IFITM1	0.1	-0.065	-0.128	0.281	0.743	3.56E-06
THTPA	-0.465	0.145	0.356	-0.111	0.067	3.57E-06
RBMS2P1	0.065	0.025	0.25	0.448	0.33	3.60E-06
RP11-295P9	-0.462	0.195	-0.139	-0.739	-0.507	3.62E-06
TSEN2	-0.588	-0.259	0.116	-0.087	-0.242	3.63E-06
RPA3	-0.329	-0.052	0.05	-0.456	-0.692	3.63E-06
EGFL8	-0.569	-0.019	0.06	-0.731	-0.905	3.63E-06
DNAJC24	-0.218	0.047	0.088	-0.302	-0.554	3.64E-06
AC079922.3	-0.583	0.159	0.585	-0.059	-0.202	3.67E-06
RAB6A	0.287	0.131	0.037	0.268	0.417	3.69E-06
RP11-672A2	-0.675	0.179	0.805	0.191	0.292	3.70E-06
SNTB2	0.03	-0.196	-0.327	-0.277	-0.053	3.71E-06
NDUFS1	0.256	0.032	0.036	0.407	0.276	3.72E-06
CAMSAP2	0.384	0.137	0.025	0.084	0.125	3.72E-06
SEC22B	0.328	0.154	0.024	0.091	0.118	3.73E-06
MAP2K1P1	0.415	0.011	-0.104	0.493	0.44	3.75E-06
FES	-0.455	-0.125	-0.098	-0.176	-0.128	3.75E-06
GPR4	0.086	-0.235	0.001	0.33	-0.151	3.75E-06
CNTN3	0.203	-0.051	-0.377	-0.408	-0.673	3.78E-06
GALNT2	0.257	0.068	-0.051	0.366	0.436	3.83E-06
CECR1	0.288	0.089	-0.032	0.297	0.384	3.83E-06
ME1	0.256	0.364	0.201	0.481	0.284	3.87E-06
UBR3	0.206	0.426	0.116	0.152	-0.069	3.89E-06
RP11-75A9.2	0.292	-0.098	-0.005	0.448	0.339	3.90E-06
AMER1	0.097	0.295	0.135	-0.503	0.002	3.90E-06
HACL1	0.084	0.036	0.051	0.479	0.34	3.93E-06
FNDC3A	0.354	0.048	-0.082	0.085	0.144	4.00E-06
MSS51	-0.196	-0.2	0.417	-0.668	-0.676	4.00E-06
RP11-484D5	0.193	-0.245	-0.396	-0.084	0.182	4.03E-06
C1QTNF12	-0.093	-0.205	0.414	-0.335	-1.044	4.04E-06
CD74	-0.221	0.341	0.209	0.463	1.145	4.05E-06

TSG101	0.227	0.074	0.135	0.395	0.253	4.07E-06
PCM1	0.083	0.161	0.125	0.389	0.444	4.13E-06
C9orf47	-0.715	0.277	0.203	-0.721	-0.204	4.13E-06
ASB7	0.446	0.326	0.071	0.241	0.303	4.16E-06
CEP135	0.023	-0.159	0.086	-0.044	-0.374	4.17E-06
ANO2	-0.063	-0.422	-0.376	-0.386	0.344	4.18E-06
GLTSCR1L	0.308	-0.046	0.268	0.389	0.11	4.18E-06
MNT	0.246	0.351	0.453	0.276	0.537	4.22E-06
AC011753.3	-0.515	-0.374	-0.211	-0.465	-0.705	4.23E-06
MRC2	0.209	-0.024	-0.099	0.194	0.42	4.24E-06
ABHD11	-0.146	-0.128	-0.128	-0.127	-0.531	4.25E-06
DIP2C	0.159	-0.11	-0.263	-0.315	0.382	4.27E-06
ANO7	0.013	-0.001	-0.292	-0.305	0.711	4.30E-06
HSBP1L1	-0.645	-0.326	-0.162	-0.609	0.263	4.31E-06
RP11-169K1	-0.302	0.112	-0.401	-0.39	-0.795	4.35E-06
CTD-2621I17	-0.596	0.292	0.035	-0.781	-0.714	4.46E-06
PANK1	0.562	0.189	-0.178	-0.263	0.511	4.51E-06
EGFR	0.521	0.419	0.323	0.241	0.365	4.52E-06
HOXB5	-0.621	-0.401	-0.129	-0.726	-0.034	4.52E-06
TCAF2	-0.043	0.182	-0.031	-0.055	-0.43	4.52E-06
CTD-2547G2	-0.664	-0.075	0.022	-0.937	-0.846	4.52E-06
PSMC1	0.257	0.219	0.061	0.092	-0.336	4.57E-06
RANGRF	-0.148	-0.17	-0.255	-0.229	-0.464	4.59E-06
H2AFZ	-0.291	-0.192	-0.109	-0.221	-0.552	4.60E-06
SNORD58C	-0.518	0.042	-0.059	-0.998	-0.906	4.60E-06
RP11-152N1	-0.552	-0.133	-0.205	-0.53	-1.173	4.62E-06
PPP2R5D	0.024	-0.1	-0.116	0.292	0.323	4.64E-06
ZNF470	-0.367	0.291	0.36	0.114	0.361	4.65E-06
SNORD45C	-0.159	0.196	0.244	-0.693	-0.92	4.65E-06
TOMM5	-0.367	-0.196	-0.145	-0.558	-0.734	4.66E-06
TPRKB	-0.434	-0.141	-0.113	-0.49	-0.794	4.66E-06
PAFAH1B2	0.235	-0.021	0.019	0.305	0.35	4.77E-06
HEXDC	-0.331	0.239	0.356	-0.167	0.01	4.77E-06
MIR1914	-0.836	-0.378	-0.529	-1.165	-0.935	4.79E-06
FUBP3	0.395	0.054	-0.076	0.129	0.119	4.81E-06
SNORA48	-0.178	0.089	0.044	-0.849	-0.788	4.82E-06
ANXA2P1	0.124	-0.199	-0.141	0.44	0.397	4.85E-06
YME1L1	0.343	0.195	0.063	0.35	0.301	4.85E-06

ALKBH8	0.345	0.04	0.039	0.257	-0.232	4.85E-06
ATP8B3	-0.938	-0.566	-0.295	-0.579	-0.684	4.85E-06
CTD-2195M1	0.34	-0.184	-0.214	0.243	0.314	4.89E-06
RP11-169K1	-0.243	-0.317	-0.216	-0.495	-0.437	4.97E-06
TMEM107	-0.387	-0.112	0.192	0.001	-0.27	4.99E-06
TXNIP	-0.362	-0.073	-0.579	-0.336	-0.058	5.03E-06
RP4-583P15	-0.246	0.038	0.203	-0.746	-0.427	5.04E-06
RP11-320N7	-0.289	-0.335	-0.096	0.178	-0.108	5.07E-06
DNAJC18	0.019	0.184	0.598	-0.053	0.279	5.10E-06
RRAGC	0.203	0.208	0.055	0.382	0.425	5.11E-06
SNORA4	-0.329	0.214	-0.082	-0.739	-0.999	5.11E-06
B3GALNT1	-0.152	-0.375	-0.407	-0.282	-0.289	5.13E-06
LIX1L	0.097	-0.006	-0.007	0.337	0.237	5.16E-06
RP4-758J18.	-0.341	-0.003	0.011	-0.06	-0.357	5.16E-06
RP11-1246C	-0.202	-0.272	0.473	0.022	0.762	5.20E-06
DNAJC21	0.318	0.348	0.229	0.3	0.349	5.23E-06
NPC1	0.261	0.158	0	0.064	0.43	5.24E-06
PPIL3	-0.18	-0.198	-0.016	-0.15	-0.671	5.24E-06
MTMR11	-0.314	0.097	0.326	-0.165	0.09	5.26E-06
PGDP1	0.374	0.047	0.004	0.472	0.652	5.27E-06
GLI3	-0.146	-0.149	0.073	0.171	0.345	5.28E-06
RP11-81G24	0.134	0.161	-0.257	-0.283	-0.493	5.32E-06
DGAT1	-0.372	-0.161	-0.124	-0.327	-0.07	5.33E-06
CCDC36	-0.794	-0.557	-0.23	-0.391	-0.396	5.37E-06
RP11-806L2.	-0.128	0.524	0.734	0.565	0.896	5.43E-06
RNF166	-0.203	-0.268	-0.121	-0.303	-0.338	5.43E-06
RP11-83A24	-0.929	-0.187	0.249	-0.045	0.067	5.44E-06
DEDD2	-0.099	0.306	0.11	0.042	0.323	5.50E-06
KLF10	-0.346	0.178	0.148	0.182	0.357	5.51E-06
HAX1	-0.115	-0.145	-0.084	-0.101	-0.398	5.51E-06
ARL16	-0.255	-0.137	-0.108	-0.475	-0.432	5.51E-06
APOBEC3F	-0.416	-0.03	0.474	0.444	0.597	5.57E-06
TOP1MT	0.189	0.129	0.175	0.387	0.576	5.59E-06
VAC14	0.176	0.021	0.003	0.393	0.333	5.60E-06
DHRS11	-0.076	-0.112	-0.326	-0.466	-0.591	5.60E-06
ABCA2	-0.026	0.1	0.201	0.002	0.343	5.61E-06
CSPP1	-0.384	-0.222	-0.238	-0.504	-0.589	5.63E-06
DCAF6	0.355	0.131	-0.015	0.263	0.425	5.66E-06

RP1-273G13	-0.06	-0.314	-0.345	-0.519	-1.012	5.66E-06
ZMYND11	0.338	0.069	-0.077	0.092	0.27	5.71E-06
RP13-554M1	-0.335	0.123	0.095	-0.835	-0.267	5.72E-06
ROCK1	0.162	0.181	0.129	0.307	0.445	5.73E-06
YWHAH	0.266	-0.019	-0.001	0.387	0.302	5.75E-06
TRAPPC9	0.391	0.021	-0.063	0.371	0.504	5.77E-06
TBX3	0.527	0.109	0.25	0.082	0.413	5.79E-06
PDLIM3	0.439	0.169	0.16	0.394	0.5	5.81E-06
PEPD	0.274	0.052	-0.038	0.317	0.462	5.81E-06
PSMF1	0.278	0.08	0.024	0.441	0.387	5.82E-06
RP11-51F16	-0.083	-0.189	-0.001	0.14	0.669	5.84E-06
SMPD4	0.029	-0.047	-0.08	0.009	-0.342	5.84E-06
AGFG2	-0.472	-0.323	-0.075	0.056	0.13	5.85E-06
DDX50	0.124	0.216	0.142	0.381	0.102	5.85E-06
RPS19BP1	-0.035	-0.202	-0.265	-0.173	-0.359	5.87E-06
AC142391.3	-0.024	0.403	0.975	-0.068	0.104	5.88E-06
MDH2	0.336	0.125	0.024	0.478	0.399	5.95E-06
RIMKLB	-0.11	-0.107	0.068	-0.538	-0.377	5.99E-06
SNRPN	0.063	0.075	0.091	0.249	0.394	6.03E-06
RPF2	-0.107	-0.074	-0.187	-0.107	-0.455	6.04E-06
ZNF441	0.015	0.175	0.1	-0.419	-0.365	6.07E-06
LONRF3	0.734	-0.021	0.139	-0.031	-0.364	6.09E-06
MRPL27	-0.03	-0.077	0.017	-0.016	-0.367	6.10E-06
GPR180	-0.5	-0.333	-0.18	-0.467	-0.312	6.11E-06
IRGQ	0.146	0.33	0.223	0.061	0.346	6.12E-06
DCTN6	-0.057	-0.089	-0.108	-0.312	-0.434	6.14E-06
RNF152	0.329	0.166	0.343	0.485	0.37	6.18E-06
SNORA21B	-0.413	0.126	-0.069	-0.772	-1.2	6.19E-06
RPL36A	-0.027	0.032	0.035	-0.397	-0.555	6.20E-06
WDR47	0.263	0.206	0.079	0.12	0.364	6.24E-06
PRR7	-0.41	-0.441	-0.074	-0.13	0.36	6.25E-06
PUDP	0.093	0.022	0.091	0.371	0.355	6.27E-06
KB-1732A1.1	0.647	0.037	0.414	0.799	0.687	6.32E-06
MAMLD1	0.023	-0.367	-0.048	0.107	0.434	6.32E-06
DHX15	0.326	0.1	-0.053	0.159	-0.06	6.32E-06
INE1	-0.325	-0.032	-0.053	-0.994	-0.683	6.32E-06
MOAP1	0.232	0.141	0.061	0.282	0.405	6.35E-06
HES7	-1.101	-0.168	-0.253	-0.546	0.105	6.35E-06

CAPN15	-0.214	-0.117	-0.164	-0.442	-0.342	6.36E-06
RAB24	-0.418	-0.085	0.018	-0.26	-0.329	6.37E-06
RNASE1	0.009	-0.215	-0.328	0.206	0.147	6.38E-06
RNASEH2C	-0.254	-0.093	-0.148	-0.401	-0.529	6.39E-06
SYNCRIP	0.431	0.056	-0.057	0.256	0.156	6.41E-06
SNORA24	-0.268	-0.187	-0.205	-0.724	-1.318	6.44E-06
TMEM234	-0.496	-0.196	-0.007	-0.128	0.022	6.48E-06
EEF1DP1	0.214	-0.008	0.075	0.308	0.404	6.49E-06
IVNS1ABP	-0.348	-0.216	-0.045	-0.329	-0.027	6.55E-06
NEK8	-0.029	0.236	0.313	-0.146	-0.331	6.59E-06
OXSM	-0.624	-0.151	-0.113	-0.422	-0.59	6.59E-06
RP11-1033A	0.354	-0.016	0.015	0.458	0.472	6.69E-06
LMNB1	0.159	-0.249	-0.389	0.061	-0.465	6.69E-06
HOXB-AS1	-0.667	-0.136	0.147	-0.896	-0.607	6.73E-06
ATAD3A	0.112	-0.02	-0.194	-0.192	-0.381	6.74E-06
SNORD27	0.289	0.207	0.117	-0.585	-0.87	6.75E-06
SNIP1	0.439	0.225	0.142	0.392	0.37	6.76E-06
FSD1L	0.245	0.032	0.177	-0.582	-0.353	6.76E-06
HNRNPA1L2	0.268	0.323	0.288	0.512	0.214	6.77E-06
HELQ	-0.192	0.106	0.027	-0.039	-0.481	6.79E-06
TEFM	-0.387	-0.185	-0.085	-0.259	-0.629	6.79E-06
CCSAP	-0.184	-0.377	-0.568	-0.242	-0.164	6.81E-06
LRRC37A2	-0.097	0.098	0.179	-0.567	-0.193	6.81E-06
UQCRC2	0.242	0.151	0.121	0.333	0.301	6.82E-06
RP3-344J20.	0.123	-0.151	-0.18	0.379	0.58	6.85E-06
HSF4	-0.403	0.014	0.306	-0.654	-0.423	6.93E-06
LYPLA2	0.202	0.137	0.183	0.325	0.479	7.00E-06
FBXO7	0.251	0.075	-0.033	0.327	0.327	7.01E-06
EPM2A	0.309	-0.079	-0.327	-0.251	-0.061	7.01E-06
ING3	-0.113	-0.085	-0.219	-0.303	-0.443	7.02E-06
NPIPP1	-0.338	-0.123	-0.347	-0.914	-0.521	7.02E-06
YWHAZP2	0.343	0.042	-0.153	0.262	0.184	7.03E-06
SNORD100	-0.33	0.275	0.058	-0.874	-0.894	7.05E-06
MAEA	0.154	0.12	0.103	0.374	0.38	7.07E-06
F3	0.01	0.523	-0.124	-0.524	-0.528	7.08E-06
PSMD5	0.225	0.049	0	0.386	0.294	7.09E-06
GDPD1	-0.95	0.164	-0.018	-0.328	-0.029	7.16E-06
MYO1C	0.485	-0.047	-0.182	0.077	0.023	7.29E-06

SCN1B	-0.217	-0.367	-0.08	-0.194	0.175	7.31E-06
BORCS8	-0.28	-0.097	-0.004	-0.051	-0.375	7.48E-06
KCNK1	0.122	0.153	0.198	0.191	0.859	7.50E-06
LINC00472	-0.552	-0.234	-0.301	-0.888	-0.902	7.55E-06
BDH2P1	-0.149	-0.111	-0.056	0.051	0.475	7.59E-06
ALG1	0.019	-0.026	-0.148	0.08	-0.328	7.60E-06
EPHX1	0.224	0.181	0.013	0.398	-0.22	7.65E-06
YEATS4	-0.159	-0.152	-0.129	-0.337	-0.723	7.68E-06
C9orf64	0.259	0.043	0.095	0.536	0.45	7.72E-06
FNBP1	0.08	0.124	0.138	0.455	0.454	7.73E-06
RTFDC1	0.277	0.091	-0.02	0.36	0.175	7.74E-06
RP11-54D18	0.401	-0.078	-0.025	0.389	0.345	7.79E-06
G3BP2	0.371	0.125	-0.057	0.308	0.391	7.83E-06
ACOX1	0.383	0.216	0.09	0.215	0.241	7.85E-06
ACP7	0.224	0.07	0.132	0.613	0.623	7.95E-06
RP11-385E5	0.153	-0.183	-0.278	-0.025	-0.489	7.96E-06
SH2D4A	0.62	-0.219	0.028	0.414	0.928	8.02E-06
PCDHGB4	0.285	0.217	-0.012	0.275	0.816	8.02E-06
PTPRCAP	-0.782	0.156	0.065	-0.747	-0.689	8.02E-06
RP5-1148A2	0.764	0.98	1.033	1.212	1.334	8.04E-06
DCLRE1C	-0.526	-0.092	-0.01	-0.181	-0.469	8.04E-06
VEGFA	-0.115	0.025	0.169	-0.499	-0.143	8.09E-06
PDLIM5	0.427	0.097	-0.234	-0.079	-0.098	8.12E-06
NRIP3	0.256	0.317	0.22	0.275	0.498	8.15E-06
HNRNPK	0.445	0.135	-0.016	0.36	0.381	8.17E-06
C1orf131	-0.131	-0.135	-0.18	-0.16	-0.515	8.17E-06
GFPT1	0.516	0.146	0.073	0.325	0.348	8.19E-06
RHPN2	0.632	0.341	0.069	0.052	0.521	8.22E-06
RP1-267D11	-0.186	-0.194	-0.182	-0.43	-0.899	8.22E-06
STARD3NL	-0.142	-0.146	-0.223	-0.437	-0.45	8.23E-06
DVL2	-0.086	-0.039	-0.21	-0.234	-0.385	8.36E-06
ADAM10	0.35	0.18	-0.071	0.246	0.204	8.48E-06
AC097523.3	0.436	-0.284	-0.769	-0.782	-0.323	8.52E-06
AP3S2	0.107	0.044	0.09	0.246	0.466	8.55E-06
SMG9	-0.077	0.063	0.032	-0.046	-0.326	8.55E-06
LRRC8D	-0.266	-0.421	-0.361	-0.3	-0.426	8.60E-06
RP11-465B2	-0.394	0.176	0.559	-0.06	0.577	8.62E-06
GLE1	0.049	0.119	0.153	0.346	-0.106	8.62E-06

SNORD93	-0.875	-0.11	-0.249	-0.877	-1.309	8.69E-06
ERP44	0.193	0.119	0.039	0.362	-0.035	8.72E-06
PIP5K1A	0.383	0.201	-0.007	0.239	-0.022	8.73E-06
CCL20	0.252	0.765	0.002	-0.28	1.062	8.75E-06
RP11-390K5	-0.637	-0.018	0.042	-0.8	-0.82	8.75E-06
PLEKHA5	-0.08	-0.004	0.037	0.196	0.523	8.76E-06
ACE	0.476	0.006	-0.256	-0.065	-0.349	8.79E-06
RNFT1	-0.174	0.069	0.275	0.034	-0.336	8.87E-06
TTC30A	-0.086	0.212	0.068	0.645	0.59	8.94E-06
ZCCHC10	-0.394	-0.186	-0.143	-0.509	-0.616	8.97E-06
RP11-196N1	-0.126	0.125	0.128	-0.64	-0.199	8.98E-06
RP11-177H2	0.361	-0.25	-0.311	0.167	-0.097	9.03E-06
GPR75	-0.081	0.214	0.264	-0.684	-0.279	9.03E-06
RP11-215D1	0.193	-0.123	0.039	-0.083	-0.489	9.03E-06
MACF1	-0.144	-0.208	-0.001	-0.328	0.31	9.11E-06
EXOSC3	-0.111	0.032	-0.161	-0.005	-0.423	9.11E-06
SEC22C	-0.23	-0.209	-0.219	-0.235	-0.333	9.24E-06
GDI2	0.386	0.096	-0.056	0.249	0.206	9.35E-06
PPP1R9B	-0.385	-0.22	-0.112	-0.216	-0.074	9.37E-06
HOTTIP	-0.427	0.15	0.36	0.413	0.623	9.41E-06
ME2	0.218	0.12	0.05	0.386	0.258	9.42E-06
PNISR	-0.336	0.059	0.151	-0.463	-0.217	9.46E-06
SPTY2D1	0.518	0.304	0.039	0.207	0.164	9.49E-06
RP11-352M1	0.075	0.164	0.153	-0.1	-0.366	9.55E-06
TALDO1	0.218	0.097	0.003	0.393	0.038	9.58E-06
CXorf38	-0.063	-0.03	0.039	0.061	-0.33	9.61E-06
DDB1	0.463	0.259	0.231	0.648	0.635	9.62E-06
RP11-903H1	0.075	-0.25	-0.213	-0.003	-0.521	9.62E-06
EXOSC6	-0.102	-0.357	-0.355	-0.246	-0.16	9.67E-06
AC000123.3	-0.32	0.053	-0.127	-1.092	-0.844	9.69E-06
RF00278	-0.365	0.258	0.345	-0.532	-0.799	9.71E-06
MAP3K8	0.555	-0.056	-0.116	-0.531	-0.192	9.76E-06
POLR1B	-0.329	0.016	-0.064	0.153	-0.329	9.76E-06
RP11-31F15	-0.217	-0.143	0.22	-0.399	-0.16	9.79E-06
ELAVL1	0.232	-0.007	-0.056	0.293	0.456	9.86E-06
SNORA33	-0.087	0.381	0.102	-0.57	-0.693	9.90E-06
TLE6	-0.565	-0.423	0.176	-0.315	0.458	1.00E-05
MAP1LC3A	-0.255	0.054	0.066	0.033	0.343	1.00E-05

DHX33	-0.037	0.037	-0.086	-0.124	-0.328	1.01E-05
MTL1	-0.066	-0.324	-0.253	-0.351	-0.873	1.01E-05
RP11-255A1	0.183	0.022	0.01	-0.589	-0.897	1.01E-05
SLC25A23	0.05	-0.076	0.009	0.082	0.39	1.02E-05
RP5-865M20	0.378	-0.004	-0.074	0.354	-0.019	1.02E-05
ZNRF3	0.35	0.145	0.001	-0.072	-0.14	1.02E-05
SLC25A11	0.033	-0.152	-0.231	0.105	-0.393	1.02E-05
C12orf76	-0.363	0.159	0.286	-0.038	0.263	1.04E-05
VBP1	-0.116	-0.112	-0.15	-0.345	-0.521	1.04E-05
ACLY	0.438	0.037	-0.137	0.198	0.437	1.05E-05
MARCH8	0.363	0.398	0.254	0.395	0.402	1.05E-05
NLN	0.463	-0.037	-0.138	0.241	0.248	1.05E-05
USP38	0.366	0.133	-0.067	0.134	0.07	1.05E-05
RP11-555H2	0.291	0.046	-0.033	0.43	0.585	1.06E-05
DDX6	0.34	0.206	0.072	0.329	0.436	1.06E-05
CTC-329H14	0.155	-0.028	0.014	0.28	0.361	1.06E-05
ANKRD36B	-0.794	-0.251	-0.062	-0.681	-0.786	1.06E-05
RP11-296P7	0.378	0.1	0.028	0.479	-0.024	1.07E-05
SPRY2	-0.33	-0.022	0.053	-0.002	-0.127	1.07E-05
MOV10L1	-0.177	0.032	0.128	-0.442	-0.399	1.07E-05
RP5-1043F6	0.379	-0.131	-0.061	0.414	0.512	1.08E-05
MILR1	-0.531	-0.513	-0.068	-0.2	0.076	1.09E-05
FAM177A1	0.326	0.125	0.075	0.162	0.199	1.10E-05
ADCK4	-0.324	0.065	0.242	0.121	-0.187	1.10E-05
S100A3	0.353	0.281	0.159	0.233	1.241	1.11E-05
GSPT1	0.362	0.014	-0.131	0.241	0.318	1.11E-05
ACTR5	-0.203	0.048	0.041	-0.124	-0.363	1.11E-05
SLC25A33	-0.248	-0.204	-0.448	-0.668	-0.428	1.11E-05
C9orf9	-0.03	0.018	0.239	0.601	0.666	1.12E-05
USP32P1	-0.482	0.07	0.173	-0.772	-0.522	1.12E-05
RP11-574K1	-0.311	-0.08	0.143	-0.487	-0.683	1.12E-05
SRPRA	0.323	0.096	-0.025	0.204	-0.23	1.13E-05
RP11-417L1!	-0.342	0.061	0.219	-0.594	-0.762	1.13E-05
HEATR5A	0.16	0.11	0.075	0.31	0.425	1.15E-05
DAAM1	0.414	0.304	-0.018	0.114	0.363	1.15E-05
OGFOD1	0.257	0.147	0.163	0.413	0.298	1.15E-05
GLA	0.047	0.235	0.115	0.324	0.201	1.15E-05
METRN	-0.166	-0.315	-0.315	-0.525	0.133	1.15E-05

MRPL47	-0.118	0.022	-0.088	-0.237	-0.378	1.15E-05
KCNMB1	0.161	-0.098	-0.022	-0.347	-0.664	1.15E-05
SNHG11	-0.627	-0.289	-0.181	-0.802	-0.669	1.15E-05
SEL1L	0.343	0.132	-0.062	0.182	0.008	1.16E-05
GPHN	0.531	0.206	0.301	0.672	0.261	1.17E-05
TRNT1	-0.416	-0.153	-0.003	-0.25	-0.186	1.17E-05
ZNF772	-0.311	0.093	-0.003	0.06	0.403	1.18E-05
CDC5L	0.451	0.251	0.016	0.43	0.293	1.18E-05
C22orf39	-0.429	-0.331	-0.159	-0.301	-0.282	1.19E-05
SHPRH	-0.205	0.04	-0.125	-0.172	-0.472	1.19E-05
RP11-393M1	0.203	-0.12	-0.051	0.06	-0.323	1.20E-05
ADGRG6	-0.051	-0.167	-0.386	-0.266	-0.346	1.20E-05
ISCA2	-0.445	-0.231	0.006	-0.086	-0.483	1.20E-05
PHYH	0.176	0.392	0.514	0.578	0.556	1.21E-05
SEC24D	0.422	-0.02	0.035	0.318	0.361	1.21E-05
RP11-144L1.	0.265	0.123	0.236	0.429	0.352	1.21E-05
GPR176	0.102	0.271	0.207	0.332	0.216	1.21E-05
RP11-196G1	-0.58	0.087	0.514	-0.75	-0.481	1.21E-05
PPAN	-0.286	-0.037	-0.059	-0.408	-0.608	1.21E-05
RP11-723P1	-0.213	-0.293	-0.298	-0.61	-0.977	1.21E-05
AC006129.4	-0.119	0.223	0.258	-0.19	1.018	1.22E-05
DDX3X	0.456	0.19	-0.022	0.164	0.083	1.22E-05
AP3M1	0.18	0.101	0.052	0.348	0.387	1.23E-05
RBM12	0.347	0.034	-0.216	0.019	-0.014	1.23E-05
UGDH	0.474	0.096	-0.004	0.314	-0.154	1.24E-05
TOP1	0.03	0.075	0.018	0.298	0.496	1.25E-05
RBM48	0.094	0.416	0.36	-0.131	-0.136	1.25E-05
NFE2L3P1	0.091	-0.003	0.309	0.543	1.055	1.26E-05
EXOC1	0.126	0.161	0.174	0.39	0.386	1.26E-05
RP4-599G15	-0.647	0.016	0.758	0.555	0.316	1.26E-05
ZNF621	0.001	0.186	0.346	0.014	-0.039	1.27E-05
SHANK3	-0.236	-0.119	0.122	-0.483	-0.297	1.27E-05
RP4-665J23.	0.082	-0.051	-0.188	-0.187	-0.874	1.27E-05
C7orf13	-0.605	-0.071	0.205	0.263	0.166	1.28E-05
AP4E1	0.324	0.318	0.261	0.366	0.089	1.28E-05
PA2G4	0.427	0.237	0.097	0.488	0.015	1.28E-05
STK3	0.055	0.353	0.031	-0.028	-0.239	1.28E-05
MAP2K2	0.13	0.079	0.045	0.248	0.404	1.29E-05

BPHL	-0.062	0.038	-0.033	0.032	0.371	1.29E-05
FAM155A	0.227	0.006	-0.362	-0.481	-0.001	1.29E-05
ASL	-0.023	0.026	-0.09	-0.081	-0.389	1.29E-05
RP11-140L2	-0.182	0.295	0.366	-0.629	-0.437	1.29E-05
SFT2D1	-0.215	-0.106	-0.013	-0.251	-0.5	1.29E-05
XPR1	0.172	0.275	0.099	0.337	0.405	1.30E-05
OAT	0.444	0.123	-0.109	0.292	0.389	1.30E-05
NDUFC1	-0.219	-0.156	-0.063	-0.312	-0.547	1.30E-05
GANAB	0.294	0.107	0.022	0.413	0.478	1.31E-05
KIF3B	0.191	0.193	0.149	0.082	0.323	1.31E-05
ZNF25	-0.099	-0.253	-0.234	-0.507	-0.439	1.31E-05
RP11-487I9.	0.334	-0.276	-0.256	-0.162	-0.945	1.31E-05
RTF1	0.255	0.084	-0.083	0.261	0.365	1.32E-05
C1D	-0.293	-0.117	-0.065	-0.655	-0.812	1.32E-05
THAP8	-0.269	0.086	0.163	0.621	-0.121	1.33E-05
COPA	0.425	0.089	0.062	0.358	0.526	1.38E-05
EEFSEC	0.314	0.223	0.363	0.673	0.246	1.38E-05
ZFC3H1	-0.441	-0.066	0.185	-0.173	-0.004	1.38E-05
RAB21	0.457	0.261	-0.056	0.106	0.046	1.39E-05
PRKAR2A	0.341	0.018	-0.107	0.096	0.014	1.40E-05
RNA5-8SN3	-0.257	0.337	-0.244	-1.117	-0.708	1.40E-05
RP1-283E3.8	-0.176	0.234	0.175	-0.629	-0.327	1.41E-05
SNORD83A	-0.64	0.087	-0.144	-0.926	-0.797	1.41E-05
IL17RC	-0.111	0.014	0.142	0.19	0.332	1.42E-05
ADAMTS13	-0.282	0.085	0.71	0.08	0.211	1.42E-05
MED26	0.42	0.079	-0.272	-0.081	-0.081	1.42E-05
TET2	-0.006	0.043	-0.201	-0.257	-0.331	1.42E-05
CAP1	0.455	0.118	-0.042	0.378	0.403	1.43E-05
CTD-2339L1	-0.099	0.498	0.61	-0.35	-0.297	1.43E-05
HSP90B2P	0.334	-0.038	-0.063	0.288	-0.082	1.44E-05
FAM104B	-0.141	-0.186	-0.232	-0.525	-0.577	1.44E-05
CCDC90B	-0.028	0.211	0.406	0.268	0.335	1.46E-05
WASHC2A	0.304	0.423	0.451	0.39	0.451	1.47E-05
BMS1	0.411	0.076	0.06	0.369	0.116	1.47E-05
LIFR	0.119	-0.074	-0.232	-0.18	-0.327	1.47E-05
DHX30	0.079	0.038	-0.117	-0.008	-0.337	1.47E-05
CLUH	-0.007	-0.068	-0.17	-0.219	-0.398	1.47E-05
RP11-395L1	-0.067	0.245	0.337	0.425	0.506	1.48E-05

GMFB	-0.442	-0.106	0.205	0.16	0.025	1.48E-05
CD99L1	-0.222	0.009	0.082	-0.349	0.119	1.49E-05
EIF4G3	0.258	0.104	0.098	0.402	0.327	1.50E-05
RP11-705C1	0.147	-0.144	-0.149	0.299	0.481	1.51E-05
USP9X	0.298	0.112	0.029	0.312	0.435	1.51E-05
C8orf82	-0.204	0.062	0.04	0.145	0.362	1.51E-05
MROH1	-0.324	0.025	0.152	-0.149	0.026	1.51E-05
ZCCHC8	-0.052	0.039	0.072	-0.077	-0.404	1.51E-05
FBXO11	0.194	0.178	0.1	0.176	0.343	1.52E-05
EMC2	0.338	0.115	0.028	0.285	-0.112	1.53E-05
LRRC2	-0.256	0.033	0.331	0.691	0.459	1.54E-05
SLC4A2	0.009	-0.075	-0.171	-0.122	-0.402	1.54E-05
GALM	-0.052	-0.135	-0.066	0.373	0.652	1.55E-05
SMU1	0.33	0.184	0.06	0.383	0.304	1.56E-05
PSMD4	0.356	0.142	0.005	0.432	0.158	1.57E-05
RP11-1277A	-0.129	0.191	0.582	0.704	0.838	1.60E-05
MORC4	0.321	0.392	0.182	0.385	-0.082	1.60E-05
AXIN2	0.415	-0.09	-0.131	-0.218	-0.335	1.60E-05
BAHCC1	0.038	-0.394	-0.092	0.035	0.265	1.61E-05
RECQL	0.352	0.153	0.078	0.445	0.416	1.62E-05
EIF1AY	0.064	0.01	-0.011	0.007	-0.367	1.62E-05
PGK1	0.358	0.068	-0.051	0.365	0.254	1.63E-05
DYNLL1	-0.07	-0.228	-0.249	-0.649	-0.288	1.63E-05
RP4-647C14	0.334	0.022	0.187	0.46	0.526	1.64E-05
RP11-721G1	0.398	0.05	-0.018	0.43	0.209	1.65E-05
FASTKD5	0.502	0.223	-0.03	0.309	0.131	1.65E-05
ZNF823	-0.262	0.137	-0.146	-0.342	-0.599	1.65E-05
RP11-422P2	-0.407	-0.391	-0.184	-0.767	-0.678	1.65E-05
TAB1	-0.14	-0.001	0.062	0.378	-0.03	1.66E-05
RP11-3D4.2	0.122	0.222	0.313	-0.891	-0.475	1.66E-05
CTD-2587H2	-0.347	0.032	-0.052	-0.849	-0.446	1.67E-05
BAIAP2	0.553	0.308	0.02	0.059	0.188	1.68E-05
CLHC1	-0.333	0.273	0.467	-0.188	-0.252	1.68E-05
CTB-58E17.1	-0.491	0.33	-0.291	-0.264	-0.919	1.68E-05
GFM1	0.27	0.087	0.019	0.384	0.309	1.69E-05
ARHGAP31	0.456	0.077	0.039	0.283	0.345	1.70E-05
CPNE5	0.256	-0.214	-0.009	-0.118	0.453	1.71E-05
SETD7	0.362	0.101	0.014	0.171	0.273	1.71E-05

GTF3A	-0.355	-0.165	-0.016	-0.146	0.17	1.71E-05
RP3-449O17	-0.216	-0.194	0.132	-0.653	0.589	1.72E-05
PLEKHA4	-0.177	0.001	0.4	0.189	0.203	1.73E-05
ACTG1	0.445	0.017	-0.197	0.085	0.034	1.73E-05
BTBD9	0.401	0.036	0.091	0.476	0.367	1.74E-05
LRRC59	0.425	0.045	-0.101	0.195	0.06	1.74E-05
ZNF584	0.368	0.145	-0.197	-0.132	-0.201	1.74E-05
DIAPH3	0.057	0.194	0.053	0.511	0.417	1.75E-05
KRBOX4	-0.281	-0.05	0.02	-0.474	-0.491	1.75E-05
SP1	0.332	0.069	0.171	0.207	-0.073	1.76E-05
SLC9A5	-0.272	-0.057	0.251	-0.521	-0.276	1.76E-05
VPS11	0.183	0.131	0.147	0.39	0.204	1.77E-05
BBS7	0.145	0.327	0.316	0.258	0.063	1.77E-05
ABCD3	0.35	0.131	0.002	0.256	-0.082	1.77E-05
AC012146.7	-0.632	-0.391	-0.354	-0.796	-0.931	1.77E-05
RP11-873E2	0.097	0.072	0.015	-0.024	1.043	1.78E-05
PPP1R7	0.297	0	-0.087	0.33	0.327	1.78E-05
FBXO4	0.09	-0.101	-0.259	-0.231	-0.546	1.78E-05
RP11-325E1	-0.674	-0.48	-0.362	-0.367	-0.88	1.78E-05
HMGB2P1	-0.295	-0.726	-0.771	-0.297	-0.925	1.78E-05
PYGB	0.345	0.006	-0.065	0.186	0.42	1.79E-05
TCP11L1	0.131	0.343	0.323	0.396	0.166	1.79E-05
PRKAR2B	0.198	0.072	0.13	0.463	0.075	1.79E-05
LIN9	-0.037	-0.186	-0.409	-0.359	-0.448	1.79E-05
RP11-722A1	0.033	0.029	0.344	0.438	0.621	1.80E-05
PAXBP1	-0.127	0.087	0.014	-0.474	-0.3	1.80E-05
RPL26L1	-0.104	-0.108	-0.037	-0.299	-0.509	1.81E-05
RP11-219A1	-0.408	-0.14	0.126	-0.949	-0.547	1.81E-05
RP5-1180D1	0.348	0.118	0.354	0.265	0.732	1.82E-05
SPPL2B	-0.422	-0.004	0.047	-0.413	-0.313	1.82E-05
BLOC1S2	0.427	0.583	0.604	0.267	0.445	1.83E-05
PRKCI	0.197	0.202	0.19	0.395	0.402	1.83E-05
SORD	0.094	-0.07	-0.12	0.266	0.379	1.83E-05
SPRTN	0.336	0.061	0.045	0.294	0.012	1.83E-05
YWHAG	0.294	0.027	-0.046	0.264	0.391	1.84E-05
HNRNPR	0.404	0.031	-0.206	0.174	0.031	1.84E-05
RP11-328D5	-0.355	0.162	0.352	-0.366	-0.071	1.84E-05
SPAG1	0.436	0.229	-0.073	0.101	-0.073	1.84E-05

TCEA1	0.329	0.196	0.083	0.311	0.35	1.85E-05
C12orf57	-0.21	-0.195	-0.146	-0.233	-0.509	1.85E-05
CKLF	-0.482	-0.335	-0.165	-0.555	-0.663	1.85E-05
IL18	-0.09	0.102	-0.466	-0.288	-1.287	1.85E-05
GTF3C3	0.102	0.132	0.099	0.336	0.139	1.86E-05
SEC24A	0.266	0.241	0.131	0.295	0.479	1.87E-05
RP11-744O1	0.299	0.016	0.02	0.281	0.419	1.87E-05
RNF6	0.366	0.194	0.059	0.291	0.282	1.87E-05
MMACHC	-0.234	0.032	0.287	0.517	0.33	1.88E-05
KARS	0.342	0.092	-0.028	0.367	0.292	1.88E-05
SNORD49A	-0.327	-0.125	0.154	-0.768	-0.913	1.88E-05
C7orf49	-0.292	-0.066	-0.122	-0.143	-0.409	1.89E-05
SH3BGRL3	0.2	-0.027	-0.002	0.267	0.335	1.90E-05
RP11-345P4	0.277	0.243	0.226	-0.223	-0.526	1.90E-05
MIR3189	1.057	1.308	1.355	0.669	1.112	1.92E-05
CES4A	-0.267	-0.004	0.435	-0.215	0.319	1.92E-05
OSGEP	-0.108	-0.038	-0.008	-0.408	-0.346	1.92E-05
RP11-166B2	-0.516	0.076	-0.344	-1.114	-0.578	1.92E-05
KCNAB2	0.248	-0.24	0.072	0.49	0.599	1.94E-05
RPL3P2	0.101	-0.135	0.144	0.228	0.402	1.94E-05
AKR1C1	-0.085	-0.225	-0.162	-0.097	-0.696	1.94E-05
PAQR3	-0.027	0.05	-0.151	-0.389	-0.297	1.95E-05
RP11-390P2	-0.81	-0.137	-0.037	-0.925	-0.341	1.95E-05
ZNF45	-0.104	0.063	-0.146	-0.226	-0.383	1.95E-05
NBPF12	-0.207	-0.037	0.11	-0.503	-0.275	1.98E-05
LOC134466	-0.459	0.067	0.334	-0.487	-0.301	1.98E-05
PUS7L	-0.185	-0.029	0.055	-0.057	-0.375	1.98E-05
MRPS31	-0.012	-0.001	0.079	0.084	-0.395	1.99E-05
C9orf78	0.319	0.098	0.007	0.347	0.17	2.02E-05
INAFM2	0.359	0.131	0.101	0.333	0.211	2.03E-05
ROCK2	0.135	0.037	0.055	0.283	0.361	2.05E-05
C8orf59	-0.367	-0.161	-0.099	-0.686	-0.659	2.05E-05
DAZAP2	0.317	0.122	0.033	0.365	0.167	2.07E-05
DYNC1LI2	-0.206	0.073	0.128	-0.356	-0.005	2.07E-05
AGAP9	-0.307	-0.315	-0.066	-0.751	-0.976	2.09E-05
PLBD2	0.224	0.111	0.13	0.417	0.544	2.12E-05
AURKAPS1	-0.804	-0.961	-1.012	-0.738	-0.399	2.12E-05
C4A	-0.407	0.427	0.361	-0.5	-0.612	2.12E-05

KIF4A	-0.259	-0.495	-0.482	-0.048	-0.109	2.13E-05
CLK4	0.066	0.18	0.038	-0.556	-0.218	2.13E-05
HES6	-0.508	-0.49	-0.764	-0.877	-0.259	2.13E-05
PYGL	0.295	0.034	0.004	0.391	0.468	2.14E-05
TBCD	0.065	-0.004	0.069	0.063	0.33	2.16E-05
TMX4	0.152	0.137	0.074	0.268	0.325	2.17E-05
NMRK1	-0.179	0.038	0.383	0.138	0.32	2.17E-05
KIAA1143	0.28	0.177	0.126	0.239	0.327	2.18E-05
XXbac-BPG1	-0.801	0.333	0.196	-0.26	-0.123	2.20E-05
ZNF431	-0.076	-0.098	-0.12	-0.728	-0.276	2.20E-05
CCDC144A	-0.493	-0.033	0.175	-0.857	-0.593	2.20E-05
CDCA7L	0.25	0.028	0.036	0.352	0.346	2.21E-05
PLAG1	0.023	0.08	-0.137	-0.304	-0.532	2.21E-05
RP11-14C22	0.071	0.133	0.336	0.123	0.957	2.23E-05
TRAF6	0.403	0.33	0.241	0.119	0.482	2.23E-05
HBS1L	0.315	0.148	0.031	0.344	0.197	2.24E-05
RSRP1	-0.384	-0.006	0.049	-0.824	-0.494	2.24E-05
CNN2	0.319	0.001	-0.139	0.068	0.382	2.25E-05
UFSP2	0.141	0.115	0.2	0.431	0.08	2.25E-05
SOCS7	0.116	-0.007	-0.101	-0.427	-0.208	2.25E-05
RP1-320J15.	0.279	0.003	0.133	0.465	0.578	2.26E-05
GNL2	0.336	0.148	0.058	0.32	-0.147	2.27E-05
RP11-236M5	0.245	-0.116	0.045	0.383	0.577	2.30E-05
ARL15	0.384	-0.021	-0.027	0.131	0.079	2.30E-05
KLHL3	-0.369	-0.601	-0.168	-0.172	0.122	2.31E-05
KLF16	-0.246	-0.421	-0.282	-0.17	0.15	2.32E-05
UNC50	-0.316	-0.136	-0.059	-0.312	-0.517	2.32E-05
ERV3-1	-0.034	0.097	0.172	-0.414	-0.052	2.33E-05
TGM1	0.126	0.299	0.532	-0.454	-0.164	2.33E-05
PAK1IP1	-0.014	-0.033	-0.069	-0.06	-0.36	2.34E-05
CCT2	0.34	0.014	-0.074	0.277	0.167	2.35E-05
RNASEH2A	-0.026	-0.021	0.022	0.365	-0.186	2.35E-05
RP11-83N9.5	0.594	1.175	1.258	1.029	0.527	2.37E-05
RP11-429G1	-0.291	0.037	-0.176	-0.173	-0.627	2.38E-05
RTCB	0.309	0.13	0.043	0.489	0.42	2.39E-05
C1orf54	-0.413	-0.215	-0.23	-0.631	0.245	2.39E-05
SNHG10	-0.692	-0.32	-0.295	-0.908	-0.629	2.39E-05
ZNF516	-0.291	-0.029	-0.013	-0.111	0.327	2.42E-05

GOLGA6L5	-0.168	0.068	0.478	-0.563	0.236	2.42E-05
UBE2MP1	0.114	-0.089	-0.122	0.064	-0.362	2.42E-05
MIR25	-0.077	-0.278	-0.422	-1.215	-0.608	2.42E-05
FAM72A	-0.771	-0.216	-0.415	-0.594	-0.776	2.42E-05
AC010877.1	-0.203	-0.203	-0.112	-0.372	-0.614	2.43E-05
PARVA	0.409	0.164	0.04	0.341	0.373	2.45E-05
RP11-365P1	-0.309	0.078	0.094	-0.902	-0.38	2.45E-05
RP11-521C2	-0.383	-0.171	0.592	0.07	0.727	2.46E-05
RPTOR	0.371	0.144	0.256	0.568	0.256	2.46E-05
POPDC3	0.015	-0.18	-0.497	-0.874	-1.155	2.46E-05
LMNA	0.432	0.174	0.125	0.567	0.588	2.47E-05
SMARCA5	0.437	0.12	-0.075	0.263	0.241	2.47E-05
LRP11	0.328	0.1	-0.043	0.268	-0.084	2.49E-05
MRPL1	-0.016	-0.188	-0.146	-0.222	-0.448	2.49E-05
C12orf29	-0.256	-0.145	-0.192	-0.441	-0.606	2.50E-05
C14orf132	0.271	-0.15	-0.442	-0.656	-0.807	2.50E-05
P2RY11	0.594	0.233	0.341	-0.16	-0.322	2.51E-05
POLR1E	0.089	-0.239	-0.424	-0.125	-0.117	2.52E-05
RP11-350G2	0.055	-0.181	-0.177	-0.125	-0.536	2.52E-05
RP1-257I20.	0.042	-0.097	-0.022	0.135	0.34	2.53E-05
STAU1	0.345	0.105	0.022	0.38	0.502	2.56E-05
HSPA4	0.273	0.054	0.015	0.463	0.329	2.56E-05
IMMP1L	-0.543	-0.389	-0.156	-0.622	-0.883	2.56E-05
PHF3	0.229	0.11	0.007	0.147	0.387	2.57E-05
AGGF1	-0.14	0.141	0.132	0.224	0.333	2.57E-05
AP002954.3	0.21	-0.113	0.221	-0.867	-0.385	2.57E-05
PDCD6IP	0.255	0.204	0.133	0.339	0.397	2.59E-05
RP11-702F3	0.206	-0.638	-0.285	0.029	0.06	2.59E-05
CNDP2	0.372	0.12	0.032	0.328	0.512	2.60E-05
CSE1L	0.287	0.146	0.068	0.487	0.307	2.60E-05
TMEM223	-0.246	-0.634	-0.364	-0.263	-0.508	2.60E-05
CEP126	-0.066	-0.14	-0.067	-0.234	-0.72	2.60E-05
PRR3	-0.151	-0.305	-0.138	-0.321	-0.504	2.61E-05
YIPF4	0.028	0.106	0.096	0.097	0.371	2.62E-05
DNPH1	-0.003	-0.12	-0.175	-0.012	0.354	2.63E-05
DUSP7	0.29	-0.117	0.033	0.343	0.066	2.63E-05
ACBD3	0.403	0.259	0.208	0.393	0.502	2.65E-05
DCTN1	0.244	0.074	0.039	0.361	0.461	2.65E-05

TLK2P1	0.424	0.035	0.034	-0.003	0.155	2.65E-05
ELOVL4	0.327	0.13	-0.181	-0.002	-0.435	2.65E-05
BLOC1S3	0.329	0.161	-0.235	-0.095	-0.064	2.68E-05
FBXW4P1	0.23	-0.031	-0.311	-0.526	-0.648	2.68E-05
CTB-50L17.8	-0.024	-0.215	-0.094	-1.058	-0.71	2.68E-05
B3GNT8	-0.031	0.143	0.111	0.407	0.468	2.70E-05
EEF2KMT	-0.147	-0.162	-0.156	-0.109	-0.503	2.70E-05
UBXN6	0.099	0.07	0.032	0.283	0.336	2.72E-05
TUBB3	0.412	0.155	-0.011	0.234	0.517	2.74E-05
RP11-983P1	-0.497	0.041	0.119	-0.284	-0.822	2.74E-05
PLIN2	0.295	-0.065	-0.085	0.422	0.138	2.78E-05
SNX8	-0.136	-0.09	-0.104	-0.558	-0.416	2.80E-05
RP11-189B4	-0.165	-0.036	0.079	-0.461	-0.706	2.80E-05
ADSS	0.203	0.099	0.109	0.451	0.246	2.85E-05
RP11-603K1	-0.228	-0.371	-0.298	-0.645	-0.534	2.86E-05
KB-1440D3.1	-1.083	-0.687	-0.357	-0.848	-1.179	2.86E-05
FAM131B	0.305	0.091	0.171	0.589	0.814	2.88E-05
GGT1	-0.088	0.095	0.237	0.392	1.155	2.89E-05
LRCH3	0.024	0.224	0.134	0.097	0.336	2.89E-05
EIF2AK2	0.252	0.114	0.142	0.279	0.335	2.90E-05
GPAT2	-0.028	0.03	-0.163	-0.089	-0.339	2.90E-05
RFFL	0.393	0.006	0.109	0.119	0.283	2.91E-05
SPG20	0.269	0.21	0.156	0.447	0.385	2.92E-05
RP11-303E1	0.213	0.101	-0.028	0.227	-0.348	2.92E-05
CLUAP1	-0.094	-0.137	-0.079	0.313	0.388	2.94E-05
GOSR2	0.323	0.088	0	0.01	0.009	2.94E-05
RP11-266F1	0.011	-0.167	-0.187	-0.404	-0.443	2.95E-05
AC079780.3	0.149	0.653	0.435	0.738	1.244	2.96E-05
GOLGA6L9	-0.712	-0.107	-0.498	-1.192	-0.675	2.97E-05
TOM1	0.436	0.024	0.039	0.403	0.218	2.99E-05
SNUPN	-0.048	-0.007	0.174	0.356	0.17	2.99E-05
CTB-67I13.1	0.365	-0.039	0.216	0.662	0.416	3.02E-05
RP11-452G1	-0.217	-0.028	0.219	-0.518	-1.143	3.03E-05
RP11-158H5	-0.291	0.048	-0.162	-0.634	-0.779	3.04E-05
GNRH1	-0.19	0.075	0.041	-0.766	-0.766	3.06E-05
ECHDC2	-0.349	0.004	0.165	-0.328	0.384	3.07E-05
TDRD7	0.034	0.041	0.217	0.403	0.436	3.08E-05
AP001258.4	-0.337	0.065	0.542	0.17	0.085	3.09E-05

RP11-21L19.	-0.138	0.186	-0.274	-0.949	-0.763	3.09E-05
PAX8-AS1	-0.462	-0.003	0.104	-0.334	-0.03	3.13E-05
RP11-365F1	-0.536	-0.405	-0.249	-0.975	-0.47	3.13E-05
ISG20L2	-0.013	-0.094	-0.219	-0.114	-0.427	3.14E-05
NOVA2	0.346	-0.178	0.018	0.24	0.231	3.20E-05
RBX1	-0.329	-0.173	-0.148	-0.473	-0.629	3.20E-05
IGLV5-52	-0.355	0.046	0.218	-0.675	-0.252	3.22E-05
CTA-217C2.1	-0.674	-0.275	-0.115	-0.447	-0.576	3.23E-05
SLFNL1-AS1	-0.368	-0.079	0.133	-0.937	-0.405	3.24E-05
CSNK2A3	0.371	0.169	0.093	0.39	0.467	3.28E-05
ASNA1	0.21	0.046	0.056	0.335	0.291	3.28E-05
RP13-685P2	-0.856	-0.709	-0.488	-0.864	-0.315	3.28E-05
GPAT3	-0.065	-0.133	-0.08	-0.118	-0.758	3.28E-05
PRDM10	0.104	0.304	0.414	0.133	0.183	3.31E-05
EFCAB4B	0.213	-0.099	0.04	0.099	0.331	3.32E-05
SELO	-0.378	-0.043	-0.052	-0.352	-0.253	3.35E-05
RAMP2	0.247	-0.078	-0.304	-0.06	-0.443	3.37E-05
MT-TT	-0.301	-0.063	0.131	-0.822	-1.026	3.37E-05
TNFSF13	-0.31	0.278	0.277	0.441	0.55	3.38E-05
HMHA1	-0.284	-0.329	0.142	0.141	-0.298	3.40E-05
CTD-2207P1	-0.321	-0.165	0.053	0.069	0.876	3.43E-05
SCARB1	0.242	0.061	-0.074	0.259	0.377	3.46E-05
ANAPC7	0.046	0.074	0.135	0.335	0.109	3.46E-05
DUS2	0.098	0.064	0.198	0.377	-0.174	3.46E-05
BLMH	0.376	0.096	-0.058	0.168	-0.04	3.48E-05
RP1-122P22	-0.322	0.239	0.606	0.818	0.875	3.50E-05
PAIP1P1	0.028	0.268	0.404	-0.399	-0.911	3.50E-05
AP1B1	0.211	0.113	0.061	0.435	0.538	3.51E-05
ZNF284	-0.316	0.287	0.095	-0.456	-0.346	3.52E-05
CTD-2506P8	-0.133	-0.411	-0.279	-0.729	-0.366	3.53E-05
OBSL1	-0.243	0.283	0.352	0.329	0.062	3.57E-05
C12orf65	-0.426	-0.058	-0.088	0.008	-0.34	3.57E-05
WASL	0.221	0.102	-0.067	0.137	0.366	3.58E-05
MTERFD3	-0.45	-0.184	-0.079	-0.561	-0.608	3.58E-05
IFT140	-0.163	-0.109	0.265	0.315	0.472	3.59E-05
NARS	0.339	0.219	0.179	0.557	0.342	3.59E-05
AC009404.2	-0.396	-0.28	0.022	-0.927	-0.718	3.59E-05
RP11-480D4	-0.353	0.026	-0.078	-0.509	-0.406	3.62E-05

RP11-598F1	0.433	0.019	-0.093	0.346	0.347	3.63E-05
TNPO1P1	-0.028	-0.261	-0.168	-0.092	-0.608	3.65E-05
APEH	0.126	0.046	0.01	0.264	0.376	3.67E-05
ATAD1	0.013	0.233	0.2	0.124	0.35	3.73E-05
PLD6	-0.817	-0.319	-0.174	-0.471	-0.725	3.74E-05
FAM102A	0.231	0.155	-0.001	-0.323	0.307	3.75E-05
CSTF3	0.037	0.318	0.229	0.362	0.091	3.75E-05
AC068302.3	-0.587	-0.222	-0.382	-1.248	-1.016	3.75E-05
LGALS3BP	0.184	-0.146	-0.184	0.135	0.551	3.76E-05
COPB2	0.489	0.217	0.1	0.541	0.51	3.76E-05
ZBED8	-0.742	-0.107	-0.111	-0.257	-0.543	3.78E-05
UBAP1L	-0.127	-0.023	-0.004	-1.089	-0.759	3.79E-05
RP11-10E18	-0.501	-0.525	-0.11	-0.559	-0.933	3.80E-05
ARHGAP39	-0.471	-0.17	0.046	-0.111	0.245	3.81E-05
GOLIM4	0.443	0.249	0.063	0.232	0.229	3.81E-05
MRPL13	-0.177	-0.072	-0.024	-0.254	-0.586	3.81E-05
MSN	0.477	0.139	-0.064	0.279	0.482	3.82E-05
RP11-159F2	-0.335	0.014	0.093	0.096	0.205	3.82E-05
CTD-2651C2	0.065	-0.112	0.207	0.457	0.633	3.84E-05
PIGA	-0.138	0.16	-0.002	-0.186	-0.375	3.85E-05
ATG2A	-0.002	0.343	0.287	0.226	0.195	3.86E-05
C12orf73	-0.307	-0.119	0.014	-0.376	-0.469	3.86E-05
MOSPD3	-0.017	0.015	0.067	0.162	0.459	3.88E-05
RP11-421L2	-0.694	-0.089	0.341	-0.172	0.008	3.90E-05
SNRNP35	0.379	-0.004	-0.002	0.291	-0.029	3.90E-05
DHX8	0.363	0.167	-0.001	0.401	0.337	3.91E-05
ICAM3	-0.431	-0.52	-0.284	-0.15	-0.21	4.00E-05
ARCN1	0.438	0.061	-0.037	0.365	0.274	4.03E-05
RP11-218F1	-0.578	-0.075	0.111	-1.04	-0.888	4.03E-05
RP11-727A2	-0.726	-0.091	0.279	-0.722	-0.612	4.05E-05
RP1-80B9.2	-0.603	-0.045	0.138	0.293	0.6	4.06E-05
HEG1	0.283	-0.005	0.015	0.317	0.349	4.06E-05
EPOP	0.214	0.021	-0.438	-0.311	0.101	4.09E-05
AC007191.4	-0.538	-0.071	-0.048	-0.863	-0.701	4.09E-05
RF01210	-0.892	-0.321	-0.04	-1.16	-0.896	4.09E-05
CAPNS1	0.344	0.1	0.083	0.481	0.398	4.10E-05
CGRRF1	-0.047	-0.093	-0.099	-0.614	-0.567	4.10E-05
MBNL3	0.537	0.413	0.36	0.442	-0.025	4.18E-05

PRPF6	0.293	0.131	0.133	0.537	0.422	4.21E-05
ANKRD10	-0.295	-0.049	0.053	-0.556	0.046	4.21E-05
RP11-66N11	0.454	-0.149	0.219	0.607	1.132	4.22E-05
SCAPER	0.031	0.144	0.333	0.491	0.327	4.23E-05
HNMT	-0.233	-0.205	-0.012	0.095	0.848	4.24E-05
INPP1	0.044	0.072	0.226	0.346	0.246	4.26E-05
IARS	0.29	0.157	0.14	0.454	0.283	4.27E-05
ATP2A2	0.336	0.118	-0.041	0.112	-0.057	4.27E-05
GPAM	-0.082	-0.016	-0.052	-0.078	-0.403	4.33E-05
TIA1	-0.323	-0.013	0.036	-0.493	0.002	4.34E-05
RP11-298I3.4	-0.74	-0.111	0.326	0.416	0.526	4.35E-05
LACTB	0.32	0.185	0.025	0.373	0.302	4.36E-05
RP5-1086K1	0.547	-0.012	0.363	0.448	0.495	4.38E-05
SPATC1L	0.075	-0.002	0.01	0.319	0.396	4.38E-05
BAIAP2-AS1	-0.163	-0.102	0.07	0.23	0.382	4.38E-05
GOLGA8H	-0.467	-0.116	0.188	-0.755	-0.722	4.38E-05
FZD2	-0.346	-0.397	-0.666	0.033	0.411	4.41E-05
ARF3	0.262	0.114	0.006	0.302	0.413	4.46E-05
SWI5	0.236	0.027	0.129	0.328	0.04	4.46E-05
ZNF100	0.101	-0.092	-0.528	-0.42	-0.269	4.50E-05
SNORA1	-0.157	0.054	-0.026	-0.817	-0.858	4.52E-05
LEO1	0.334	0.144	0.061	0.497	0.437	4.53E-05
ZNF182	-0.355	0.201	0.26	-0.001	-0.05	4.53E-05
NUBP1	0.34	0.065	-0.138	0.169	-0.063	4.53E-05
RHOQ	0.372	0.241	0.098	0.201	0.36	4.54E-05
RP11-461N2	-0.434	-0.488	-0.303	-0.326	-0.661	4.59E-05
RP11-230F1	-0.922	-0.426	-0.146	-1.193	-0.9	4.60E-05
PCDHGA11	0.165	-0.065	-0.187	0.232	0.365	4.61E-05
RAD23B	0.356	0.062	-0.061	0.21	0.114	4.64E-05
GBE1	0.526	0.315	0.215	0.544	0.507	4.68E-05
PHC1	-0.225	-0.014	0.132	-0.456	-0.363	4.74E-05
RP1-205F14	-0.101	0.495	0.514	0.609	1.266	4.75E-05
RP11-1148L	-0.039	-0.24	-0.135	-0.403	0.305	4.78E-05
ZNF316	-0.25	0.07	-0.039	-0.446	0.04	4.79E-05
OIP5	-0.511	-0.642	-0.299	-0.11	-0.59	4.84E-05
RP11-359K1	-0.324	-0.034	0.514	-0.345	-0.435	4.85E-05
RF01892	-0.543	0.092	0.104	-0.824	-0.562	4.87E-05
TTLL1	0.112	-0.067	0.004	0.447	0.396	4.90E-05

DTNA	-0.049	0.2	0.408	0.246	0.789	4.92E-05
IWS1	0.111	0.103	0.155	0.363	0.104	4.93E-05
CCDC58	-0.289	-0.13	-0.085	-0.514	-0.646	4.94E-05
STX5	0.401	0.333	0.241	0.416	0.403	4.97E-05
ZNF787	0.238	-0.15	-0.135	0.131	0.337	4.97E-05
ZNF860	-0.527	0.302	0.41	0.28	0.209	4.98E-05
AC006132.1	-1.141	-0.719	-0.262	-0.352	0.01	4.98E-05
RP11-308D1	0.433	-0.207	-0.275	-0.056	-0.061	4.99E-05
RP5-902P8.1	-0.716	0.117	0.666	0.126	0.521	5.02E-05
RP11-778J1(	0.394	0.13	0.031	0.505	0.42	5.02E-05
PSMA4	-0.081	-0.016	-0.006	-0.085	-0.415	5.03E-05
RP11-392E2	-0.411	-0.073	0.352	-0.66	-0.808	5.03E-05
PA2G4P4	0.379	0.116	0.04	0.483	-0.065	5.05E-05
AADAT	-0.093	0.09	-0.087	-0.739	-0.185	5.06E-05
RP11-152C1	-0.294	-0.344	-0.228	-0.211	-0.681	5.11E-05
HNRNPH2	0.501	0.211	0.036	0.525	0.387	5.12E-05
RP11-387H1	-0.963	-0.127	0.456	-0.135	0.266	5.13E-05
BOLA1	-0.45	-0.081	0.153	0.025	-0.417	5.13E-05
LY96	-0.405	-0.18	0.077	-0.18	0.365	5.18E-05
PAAF1	0.06	0.011	0.058	0.464	0.226	5.20E-05
RP11-566K1	0.435	-0.004	-0.432	-0.362	-0.234	5.21E-05
C19orf57	0.032	0.489	0.212	-0.131	-0.84	5.21E-05
NAA38	-0.126	-0.217	-0.139	-0.19	-0.4	5.26E-05
MCM3APAS	-0.866	-0.101	0.27	-0.282	-0.345	5.27E-05
MRPL50	-0.213	-0.105	-0.187	-0.336	-0.515	5.27E-05
NSDHL	0.412	-0.123	-0.158	0.102	0.164	5.30E-05
FBXL12	0.411	0.145	-0.02	-0.058	0.097	5.30E-05
PFDN4	-0.328	-0.041	-0.123	-0.702	-0.573	5.31E-05
RP11-98L4.1	0.338	-0.199	-0.122	0.055	-0.182	5.32E-05
COX7A2P2	-0.473	-0.308	-0.111	-0.664	-0.74	5.36E-05
KIAA1841	0.111	0.069	0.129	-0.099	0.324	5.37E-05
TMEM141	-0.333	-0.128	0.014	-0.217	-0.566	5.40E-05
ACOT7	0.379	0.041	-0.101	0.325	0.391	5.41E-05
CCT7	0.375	0.109	-0.039	0.363	0.277	5.42E-05
SSSCA1	0.008	-0.028	0.003	0.058	-0.358	5.46E-05
RP11-37B2.1	-0.296	0.438	0.267	-0.68	0.007	5.48E-05
NOSIP	0.21	0.089	0.004	0.347	0.306	5.49E-05
RP11-723J4.	-0.514	-0.085	0.02	-0.799	-0.872	5.50E-05

ZHX2	0.123	0.081	-0.092	0.067	0.577	5.53E-05
CCT6P1	-0.349	0.05	0.082	-0.694	-0.394	5.54E-05
NPRL2	-0.286	-0.052	-0.028	-0.238	-0.366	5.57E-05
RP5-1037N2	-0.484	-0.205	-0.373	-0.972	-0.934	5.59E-05
WBP11	0.357	0.154	-0.089	0.135	-0.134	5.60E-05
PIGT	0.172	0.045	-0.063	0.28	0.363	5.63E-05
FAM172A	0.389	0.102	0.033	0.318	0.164	5.63E-05
FAM212A	-0.471	-0.272	-0.351	-0.286	0.097	5.66E-05
RP11-297L1	0.352	-0.064	-0.043	0.285	0.013	5.66E-05
RAB13	-0.528	-0.108	0.077	-0.552	-0.201	5.67E-05
C12orf45	-0.082	0.113	0.229	-0.388	-0.266	5.67E-05
ZNF639	-0.101	-0.237	-0.27	-0.261	-0.344	5.67E-05
SAP30	-0.249	-0.185	-0.364	0.144	0.086	5.68E-05
CTD-2561B2	0.352	0.558	0.575	0.82	1.189	5.69E-05
ANXA7	0.249	0.176	0.099	0.348	0.015	5.69E-05
AKAP13	-0.059	-0.215	-0.229	-0.504	-0.11	5.69E-05
TMEM88	-0.557	-0.635	-0.585	-0.505	-0.627	5.70E-05
GEMIN4	0.128	-0.097	-0.25	0.002	-0.44	5.72E-05
CTD-2037K2	-0.355	0.007	-0.04	-0.35	-0.679	5.75E-05
HSD17B4	0.409	0.231	0.228	0.619	0.491	5.81E-05
RP11-255A1	-0.346	0.097	0.001	-0.634	-0.99	5.82E-05
CCDC126	-0.256	0.087	-0.09	-0.424	-0.397	5.84E-05
TMEM18	-0.252	-0.173	-0.193	-0.434	-0.476	5.87E-05
CNOT11	0.157	0.188	0.181	0.371	0.309	5.90E-05
PAPSS1	0.378	0.034	-0.16	0.178	0.113	5.90E-05
DIMT1	-0.037	-0.142	-0.149	-0.297	-0.45	5.91E-05
C17orf53	-0.05	-0.054	-0.16	-0.305	-0.626	5.92E-05
WDR5B	-0.453	0.006	-0.049	-0.155	-0.404	5.96E-05
RP11-302F1	-0.592	-0.194	-0.27	-1.047	-0.754	5.96E-05
ZNHIT2	-0.478	-0.088	-0.16	0.15	0.302	5.98E-05
SERBP1	0.413	0.119	-0.016	0.384	0.212	5.98E-05
KRT8P12	-0.521	-0.247	-0.102	-0.313	-0.913	5.98E-05
SORBS2	0.1	-0.259	-0.419	-0.115	-0.194	6.00E-05
MBTD1	-0.336	0.113	-0.051	-0.285	0.058	6.01E-05
SATB1	-0.002	-0.11	-0.296	-0.766	-0.446	6.01E-05
SMARCB1	0.36	0.023	-0.172	0.252	0.179	6.05E-05
CDK12	0.372	0.16	0.006	0.208	0.164	6.05E-05
RP11-141J1	-0.309	-0.431	-0.151	-0.615	-0.829	6.05E-05

ZNF605	-0.145	0.036	0.16	0.024	0.359	6.06E-05
IFT88	0.148	0.023	0.374	0.366	0.368	6.07E-05
ACTR2	0.439	0.17	0.029	0.394	0.363	6.08E-05
CAAP1	0.167	0.14	0.174	0.445	0.422	6.10E-05
PIGC	-0.186	-0.219	-0.254	-0.244	-0.592	6.10E-05
RP3-324O17	-0.136	-0.08	0.115	-0.807	-0.927	6.10E-05
IMPDH2	0.273	0.145	0.129	0.37	0.212	6.12E-05
NKX3-1	0.943	0.61	0.354	0.398	0.908	6.14E-05
OGFOD2	-0.217	0.283	0.181	-0.172	-0.429	6.17E-05
DDX17	-0.467	-0.131	-0.002	-0.307	-0.257	6.20E-05
RP11-151N1	-0.599	-0.121	0.062	-0.383	-0.424	6.24E-05
RP1-178F15	-0.375	0.292	0.375	-0.119	0.041	6.27E-05
AC009133.1:	-0.098	0.274	0.523	0.07	0.871	6.28E-05
ADGRE5	-0.392	-0.324	-0.179	-0.268	-0.135	6.32E-05
C1orf56	-0.037	0.001	-0.143	0.567	0.488	6.35E-05
HSP90B1	0.365	0.187	0.038	0.398	0.06	6.37E-05
ERH	-0.098	-0.18	-0.108	-0.277	-0.406	6.37E-05
TIMM8B	-0.31	-0.238	-0.101	-0.418	-0.609	6.37E-05
KIAA1429	0.269	0.271	0.198	0.423	0.371	6.39E-05
MRPS18C	-0.21	-0.057	0.044	-0.254	-0.588	6.39E-05
LYRM2	-0.128	-0.114	-0.116	-0.158	-0.393	6.50E-05
ENDOV	-0.074	0.063	0.335	0.386	0.37	6.54E-05
ATG4C	-0.173	-0.155	-0.06	0.173	-0.384	6.56E-05
CCDC61	-0.261	-0.114	-0.012	-0.164	-0.53	6.56E-05
RP11-267N1	-0.678	-0.093	0.188	-0.089	-0.553	6.56E-05
AC011816.3	-0.063	-0.109	-0.282	-0.165	-1.131	6.62E-05
LA16c-349E1	-0.626	0.128	0.231	-0.671	-0.278	6.66E-05
L3MBTL1	-0.835	0.043	0.079	-0.457	-0.201	6.67E-05
B4GALT4	0.068	0.138	0.038	-0.17	-0.334	6.69E-05
CROCCP3	-0.598	-0.22	0.276	-0.644	-0.525	6.70E-05
NR1H3	-0.371	-0.18	0.13	0.037	-0.731	6.71E-05
DHX58	-0.246	0.283	0.357	-0.049	0.386	6.72E-05
NIFK-AS1	-0.931	-0.443	-0.218	-0.236	-0.35	6.72E-05
COX10-AS1	-0.623	-0.334	-0.012	-0.575	-0.231	6.74E-05
KCTD6	0.034	-0.025	-0.112	-0.431	-0.643	6.74E-05
RP11-576D8	-0.141	0.044	0.142	-0.448	-0.654	6.78E-05
BXDC1P	-0.056	-0.306	-0.34	-0.287	-0.687	6.78E-05
PILRA	-0.547	0.099	0.33	-0.511	-0.553	6.81E-05

MRPL19	0.251	0.073	-0.053	0.335	0.123	6.82E-05
CEBPD	-0.047	-0.125	-0.635	-0.682	-0.16	6.82E-05
ZNF589	-0.17	-0.018	-0.025	-0.228	-0.429	6.83E-05
RP11-325K4	-0.41	0.175	0.103	-0.788	-0.825	6.85E-05
RP11-407G2	0.079	0.256	0.35	-0.642	-0.505	6.94E-05
RP11-807H7	0.188	-0.04	-0.565	-0.642	-1.011	6.97E-05
AC004049.2	-0.198	-0.154	-0.187	-0.301	-1.04	6.97E-05
SNORD73A	-0.213	0.28	0.206	-0.532	-0.65	6.98E-05
RP11-332H1	0.071	0.107	0.368	-0.305	-0.719	7.00E-05
NTPCR	0.043	0.079	0.302	0.309	0.485	7.01E-05
RGS7	0.406	-0.235	0.108	0.261	0.116	7.01E-05
RP11-33B1.1	-0.482	0.08	0.243	-0.505	-0.226	7.01E-05
SLC2A4RG	-0.209	-0.242	-0.285	-0.345	-0.293	7.04E-05
RP11-624L4.	-0.364	-0.082	-0.346	-0.505	-0.705	7.08E-05
APBA3	-0.017	0.382	0.215	0.039	0.146	7.09E-05
GPS2	-0.339	0.229	-0.061	-0.152	0.946	7.10E-05
LRPPRC	0.389	0.256	0.1	0.433	0.499	7.10E-05
ZNF407	0.334	0.334	0.382	0.482	0.348	7.11E-05
SNORA51	-0.25	-0.391	-0.139	-0.895	-1.103	7.11E-05
SERTAD2	0.489	0.267	0.289	0.344	0.24	7.13E-05
ZBTB7A	0.178	0.088	0.033	0.076	0.386	7.14E-05
SSB	0.362	0.242	0.073	0.432	0.24	7.26E-05
RP13-507I23	-0.672	-0.025	0.399	-0.463	0.013	7.33E-05
ZW10	0.37	0.185	0.061	0.385	-0.064	7.36E-05
ZNF559	-0.378	0.062	0.049	-0.241	-0.101	7.37E-05
AATK	-0.079	0.012	0.5	0.509	0.116	7.38E-05
UBXN8	-0.177	-0.204	-0.056	-0.19	-0.541	7.39E-05
CCM2	0.091	0.002	0.038	0.196	0.35	7.41E-05
RP11-153M7	0.247	0.102	0.311	0.554	0.838	7.45E-05
ARID5A	0.337	0.091	0.026	0.128	0.489	7.45E-05
SSTR1	-0.077	0.213	0.177	0.516	0.42	7.45E-05
CTD-2547E1	-0.468	-0.127	-0.132	-1.101	-0.673	7.45E-05
SNAPIN	-0.37	-0.252	-0.048	-0.151	-0.402	7.48E-05
SLC19A1	-0.046	-0.156	-0.019	0.334	-0.131	7.49E-05
PHF6	0.078	0.143	0.095	0.294	0.388	7.50E-05
CCT3	0.329	0.11	0.056	0.439	0.298	7.51E-05
ARFIP1	0.322	0.202	0.083	0.418	0.282	7.51E-05
ZKSCAN2	0.45	0.352	0.178	0.391	0.176	7.51E-05

AC084219.4	-0.697	-0.09	-0.011	0.024	0.342	7.56E-05
SNORD58A	-0.009	0.146	0.238	-0.72	-0.805	7.60E-05
PPP2R1A	0.398	0.061	-0.053	0.321	0.464	7.61E-05
RP5-1009N1	0.15	-0.163	-0.17	0.016	-0.649	7.62E-05
CYB5R3	0.279	0.097	0.002	0.425	0.393	7.63E-05
RCAN1	-0.119	0.176	0.079	0.115	0.421	7.64E-05
MROH6	-0.107	0.26	0.094	-0.725	-0.507	7.64E-05
KCTD11	0.421	0.442	0.268	0.137	0.366	7.65E-05
RP11-392E2	-0.168	-0.312	-0.252	-0.957	-1.204	7.66E-05
ZNF354B	-0.298	0.186	-0.076	-0.652	-0.416	7.67E-05
MIR4292	-0.098	0.141	-0.085	-0.953	-0.766	7.69E-05
CUZD1	-0.656	0.029	0.347	-0.484	-0.182	7.71E-05
CEP57L1	-0.451	-0.116	-0.099	-0.343	-0.391	7.71E-05
RP11-706O1	-0.21	-0.157	-0.292	-0.696	0.042	7.72E-05
CHST12	0.064	-0.182	-0.37	-0.064	0.015	7.79E-05
PGBD2	0.082	0.37	0.456	-0.144	-0.042	7.79E-05
FAM27B	-0.61	0.104	0.089	0.033	-0.751	7.83E-05
RPL7L1	0.329	0.122	0.046	0.288	0.121	7.84E-05
TCEAL7	0.168	0.077	0.121	0.162	0.948	7.87E-05
CHP1	0.299	0.106	0.049	0.317	0.41	7.89E-05
CCT6P3	-0.525	-0.022	0.047	-0.805	-0.437	7.90E-05
GHR	-0.084	-0.292	-0.337	-0.348	-0.642	7.90E-05
CTD-2033A1	0.334	-0.023	-0.037	0.33	0.343	7.92E-05
SNORD60	0.204	0.301	0.161	-0.401	-0.887	7.93E-05
CD99L2	0.235	0.068	0.016	0.332	0.379	7.97E-05
AF196972.4	0.49	-0.094	-0.102	0.169	0.26	8.00E-05
SUPT16H	0.393	0.131	-0.05	0.281	-0.071	8.00E-05
C19orf70	-0.305	-0.274	-0.17	-0.311	-0.429	8.20E-05
C3orf62	0.065	0.292	0.538	-0.077	0.152	8.22E-05
SDHAP3	-0.476	0.034	-0.064	-0.516	-0.457	8.27E-05
ZNF581	-0.035	0.056	0.064	0.184	0.389	8.28E-05
LINC00467	-0.103	-0.045	0.186	0.236	-0.599	8.28E-05
HCN3	-0.135	-0.274	0.035	-0.749	-0.713	8.28E-05
MIR155HG	-0.578	-0.19	-0.189	-0.837	-0.724	8.32E-05
RP11-686D2	-0.514	0.24	0.26	-0.251	0.126	8.34E-05
MSNP1	0.445	-0.054	-0.13	0.176	0.359	8.36E-05
MKRN2	0.336	0.078	-0.035	0.113	0.187	8.40E-05
NBR2	-0.467	0.255	0.273	-0.083	-0.039	8.40E-05

FECH	0.228	0.193	0.155	0.329	0.27	8.45E-05
MUT	0.268	0.167	0.089	0.371	0.165	8.52E-05
PDDC1	-0.303	-0.103	0.012	-0.339	-0.286	8.54E-05
RP3-508I15.[illegible]	-0.035	0.39	0.243	-0.717	-0.661	8.54E-05
RP11-32B5.[illegible]	0.392	0.149	0.091	0.328	0.47	8.55E-05
SEZ6L2	0.163	0.024	0.041	0.474	0.658	8.57E-05
DDAH1	0.214	0.121	0.199	0.454	0.284	8.57E-05
RP11-192H2	-0.877	-0.345	-0.157	-0.912	-0.886	8.58E-05
ATP5B	0.35	0.08	-0.023	0.362	0.233	8.63E-05
PRMT7	-0.177	-0.147	-0.171	-0.231	-0.399	8.63E-05
GNB1L	-0.075	-0.364	0.071	0.502	0.123	8.67E-05
RP11-332L1[illegible]	0.253	-0.401	-0.353	0.19	-0.171	8.68E-05
C20orf96	-0.411	-0.222	-0.064	-0.408	0.126	8.70E-05
IPP	-0.048	0.233	0.261	0.497	0.146	8.71E-05
RP11-550E2	-1.124	-0.051	0.148	-0.577	-0.526	8.73E-05
TIMP1	0.229	0.002	-0.048	0.341	0.108	8.76E-05
HOXA-AS2	-0.375	0.117	0.482	0.076	0.419	8.78E-05
FOXK2	0.123	-0.006	-0.1	0.109	0.41	8.78E-05
USP8	0.285	0.179	0.11	0.312	0.373	8.78E-05
CEND1	-0.034	-0.168	-0.208	0.084	-0.639	8.79E-05
RPL32P3	-0.454	-0.011	0.164	-0.576	-0.296	8.80E-05
RPP38	-0.01	0.057	0.024	0.084	-0.426	8.80E-05
RP11-161I2.[illegible]	-0.092	-0.091	0.226	0.359	0.929	8.92E-05
PELO	0.424	0.125	0.127	0.359	0.102	8.93E-05
ST7L	-0.197	-0.124	0.067	-0.257	-0.362	8.93E-05
RARS	0.368	0.245	0.144	0.468	0.303	8.99E-05
RP11-5A19.[illegible]	-0.73	-0.049	0.091	-0.535	-0.661	9.00E-05
SNORD38B	-0.436	-0.22	-0.001	-0.868	-0.965	9.00E-05
DENND4A	0.011	0.126	0.169	0.325	0.291	9.01E-05
LL22NC03-8[illegible]	-0.225	0.046	0.118	-0.383	-0.297	9.03E-05
CHCHD4	-0.136	-0.111	-0.104	0.071	-0.444	9.05E-05
CDK2AP2	0.159	-0.081	-0.08	0.044	-0.386	9.11E-05
PCBP1	0.355	-0.017	-0.016	0.441	0.137	9.21E-05
RALY	0.328	0.119	-0.018	0.222	0.308	9.22E-05
AC000110.1	-0.542	-0.399	-0.288	-0.473	-0.743	9.22E-05
BTBD19	0.17	0.025	0.32	-0.584	0.021	9.25E-05
RP11-934B9	-0.105	-0.239	0.193	-0.697	-0.429	9.28E-05
FAM120C	-0.444	-0.305	0.03	-0.242	-0.236	9.29E-05

KIAA0391	-0.185	0.101	0.351	-0.362	0.027	9.33E-05
RP11-51L5.2	-0.238	-0.345	-0.259	-0.392	-0.472	9.33E-05
RP11-338K1	-0.299	0.237	0.489	-0.661	-0.591	9.36E-05
RP11-223F2	0.33	0.045	0.129	0.5	0.141	9.38E-05
MIR590	0.1	0.413	0.433	-0.644	-0.415	9.38E-05
YIPF3	0.184	0.048	-0.045	0.254	0.348	9.39E-05
ANKRD9	-0.125	-0.077	-0.089	-0.244	0.322	9.39E-05
PSMD3	0.367	0.234	0.113	0.378	-0.065	9.44E-05
AGL	0.115	0.306	0.198	0.401	0.103	9.51E-05
KRT10	-0.211	-0.237	-0.19	-0.606	-0.583	9.64E-05
SLC9A3	-0.655	-0.415	-0.671	-0.738	-0.745	9.78E-05
BLVRA	0.237	0.084	0.039	0.362	0.345	9.81E-05
RP11-265B8	0.515	0.283	0.212	0.397	0.991	9.86E-05
BRMS1L	0.493	0.382	0.305	0.388	0.372	9.86E-05
NDFIP2	-0.146	-0.115	-0.111	-0.23	-0.635	9.87E-05
ADAM32	0.017	0.323	0.128	-0.441	-0.838	9.92E-05
MYOF	0.344	0.089	0.075	0.424	0.447	9.95E-05
TUBB2A	0.408	0.286	0.08	0.448	-0.088	9.96E-05
KIF1C	-0.409	-0.173	-0.018	-0.597	-0.343	9.96E-05
KDM5A	0.181	0.288	0.16	0.312	0.323	0.0001
KAT2A	-0.41	-0.23	-0.035	-0.376	-0.313	0.0001
CENPB	-0.091	-0.264	-0.205	-0.404	-0.295	0.000101
WDR53	0.076	0.083	-0.079	-0.165	-0.399	0.000101
MIR6835	-1.05	0.05	-0.064	-0.553	-0.858	0.000101
RP5-827C21	-0.276	-0.15	0.14	-0.664	-0.997	0.000101
RP11-877E1	-0.269	0.107	0.47	0.006	0.458	0.000102
SGMS2	0.389	0.084	0.049	0.301	0.195	0.000102
AC098614.2	0.353	-0.004	-0.078	0.107	0.146	0.000102
ICT1	-0.161	-0.157	-0.053	-0.116	-0.555	0.000102
LL0XNC01-7	-0.668	-0.035	-0.024	-0.784	-0.839	0.000102
RP11-792D2	-0.063	-0.317	-0.312	-0.354	0.513	0.000104
APC	0.145	0.093	0.093	0.172	0.336	0.000104
PBDC1	0.39	0.109	0.001	0.247	0.212	0.000104
EIF1B	-0.166	0.042	-0.005	-0.143	-0.394	0.000104
LAP3	0.336	0.136	0.016	0.433	0.392	0.000105
CPSF2	0.276	0.119	0.083	0.427	0.213	0.000106
COL4A1	0.437	0.124	-0.181	0.19	-0.051	0.000106
TM2D2	-0.146	-0.114	-0.188	-0.16	-0.33	0.000106

CTD-2165H1	0.199	0.202	0.416	0.372	0.742	0.000107
TULP3	0.249	0.163	0.049	0.13	0.359	0.000107
HLCS	0.242	0.07	0.081	0.33	-0.022	0.000107
KIF4B	-0.135	-0.686	-0.498	0.057	-0.239	0.000108
FAM72D	-0.616	-0.545	-0.575	0.235	-0.434	0.000108
PIN4	0.04	0.104	0.227	0.151	0.461	0.000109
AC012354.8	0.052	-0.317	-0.173	-0.093	-0.446	0.000109
RP11-342K6	0.343	0.081	0.002	0.386	0.536	0.00011
CCDC80	0.045	0.111	0.08	0.419	0.365	0.000111
UQCRC1	0.27	0.083	0.023	0.376	0.326	0.000111
RP11-783K1	-0.379	0.118	0.189	-0.349	-0.446	0.000111
RDH14	-0.081	0.073	0.118	0.094	0.387	0.000112
UBQLN1	0.408	0.179	-0.079	0.206	0.119	0.000112
ITGAE	-0.466	-0.183	-0.025	-0.588	-0.667	0.000112
TCTN2	-0.073	-0.073	-0.068	0.27	0.415	0.000113
PTTG2	-0.42	-0.756	-0.521	-0.499	-0.028	0.000113
NUDT16	-0.573	-0.347	-0.138	-0.572	-0.367	0.000113
RP11-757F1	0.819	0.693	1.136	0.305	0.978	0.000114
SND1	0.473	0.155	0.036	0.43	0.494	0.000114
RP11-642N1	0.261	0.119	0.178	0.451	0.122	0.000115
ADCY4	-0.102	0	0.163	-0.451	0.019	0.000115
TMEM170A	-0.148	0.088	0.012	-0.341	-0.18	0.000116
OTUD6B	-0.203	-0.089	-0.178	-0.122	-0.356	0.000117
RPS28	-0.32	-0.156	-0.244	-0.84	-0.542	0.000117
RAD50	0.039	0.119	0.198	0.581	0.404	0.000118
SSH3	-0.185	0.031	-0.067	-0.293	-0.435	0.000118
MRPS36	-0.062	-0.009	0.044	-0.136	-0.507	0.000118
C17orf89	-0.094	0.081	0.242	0.159	0.532	0.000119
SPA17	-0.376	-0.064	-0.142	0.208	0.239	0.000119
EIF3B	0.366	0.112	-0.007	0.301	0.335	0.00012
MALT1	0.263	0.336	0.169	0.069	-0.015	0.00012
CDC42EP1	0.355	-0.162	-0.203	0.102	0.114	0.000121
SNORA11F	-0.282	-0.099	-0.335	-0.827	-0.952	0.000121
RP11-556O5	-0.766	0.123	0.313	-0.493	-0.183	0.000123
TRIM69	-0.781	-0.158	0.043	0.009	-0.048	0.000124
RP4-774G10	0.42	0.493	0.289	0.114	-0.395	0.000124
RP13-507I23	0.371	0.047	-0.042	0.427	0.135	0.000125
MORN4	-0.117	0.077	0.346	0.601	0.471	0.000126

ATP6V1H	0.366	0.186	0.051	0.498	0.336	0.000126
ALG13	-0.222	-0.027	0.044	-0.469	-0.312	0.000126
AC001226.1	0.479	-0.03	-0.101	0.293	0.267	0.000127
TMEM60	-0.502	-0.377	-0.332	-0.294	-0.145	0.000127
ZNF75D	-0.52	-0.213	-0.097	-0.268	-0.31	0.000127
COX18	-0.191	-0.014	0.066	0.013	-0.342	0.000127
RP11-15J10.	-0.51	-0.488	0.322	-0.897	-0.84	0.000127
DCLK2	0.059	0.093	0.023	-0.444	-0.386	0.000128
SNORA81	-0.007	-0.044	0.07	-0.765	-0.879	0.000128
TAF3	0.018	0.326	0.151	0.054	0.065	0.000129
CTC-429L19	-0.696	-0.139	0.003	-0.986	-0.855	0.000129
PDE7A	-0.281	0.019	-0.21	-0.506	-0.369	0.00013
ZNF114	0.144	-0.209	-0.365	-0.45	-0.996	0.00013
FLT3LG	0.099	0.708	0.885	0.704	0.986	0.000131
FAM234A	0.16	0.029	-0.024	0.351	0.333	0.000131
CTD-2245F1	-0.365	0.111	0.134	0.231	0.376	0.000132
ERMAP	-0.107	0.078	0.325	0.057	0.238	0.000132
ERI2	-0.404	-0.057	0.094	-0.067	-0.286	0.000132
PLPP2	0.011	-0.156	-0.37	-0.305	0.649	0.000133
ZNF585B	-0.154	0.493	0.47	0.436	0.594	0.000133
XRCC6	0.379	0.171	0.029	0.48	0.18	0.000133
NSF	0.477	0.221	0.245	0.592	0.414	0.000136
WBP4	0.369	0.251	0.074	0.349	0.161	0.000137
RP3-339A18	-0.15	-0.334	-0.601	-0.431	-1.317	0.000137
CSNK2A1	0.276	0.085	-0.014	0.208	0.374	0.000138
GLUD1	0.431	0.132	-0.05	0.252	0.349	0.000138
LZIC	0.113	0.197	0.227	0.362	0.142	0.000138
STAB1	-0.261	-0.185	-0.008	-0.343	-0.031	0.000138
TUBB4B	0.247	-0.08	-0.174	0.353	-0.192	0.000138
BFSP1	-0.041	-0.313	0.002	-0.002	0.614	0.000139
CTD-2545G1	-0.745	-0.532	-0.178	-1.026	-0.835	0.000139
SNORA66	-0.658	-0.014	-0.103	-1.016	-0.952	0.000139
SVIP	-0.329	-0.221	-0.275	-0.613	-0.528	0.00014
RP11-1094M	0.347	0.091	0.08	0.558	0.3	0.000141
PCDHGB2	0.292	-0.133	-0.088	0.039	0.67	0.000142
AK6	-0.312	-0.188	-0.017	-0.307	-0.521	0.000142
RP11-326A1	0.178	-0.255	-0.24	-0.124	-0.373	0.000143
ZNF622	0.111	0.099	0.132	0.483	0.367	0.000144

DPCD	0.077	-0.028	-0.057	0.233	0.356	0.000144
SRP54	0.443	0.158	-0.002	0.396	0.23	0.000144
RP11-4K3__	0.216	-0.268	-0.415	-0.12	-0.173	0.000144
WBP1	-0.268	0.29	0.108	-0.348	-0.195	0.000144
NPDC1	-0.353	-0.148	-0.061	-0.416	-0.276	0.000144
KCNIP2	-0.588	0.004	0.215	-0.881	-0.578	0.000144
RP11-23F23	0.195	-0.101	-0.489	-0.51	-0.493	0.000145
PREP	0.332	-0.045	-0.227	0.1	0.132	0.000147
AC005251.3	-0.117	-0.193	-0.123	-0.423	-0.495	0.000147
SEC24B	0.231	0.125	0.01	0.168	0.373	0.000148
SP140L	0.092	0.093	0.266	0.185	0.325	0.000148
RP11-557H1	-0.055	-0.231	-0.753	-0.899	-0.975	0.000148
GK	-0.432	-0.012	-0.008	-0.161	-0.695	0.00015
RRM1	0.365	0.231	0.098	0.497	-0.119	0.000151
ZBTB44	0.17	0.263	0.272	0.151	0.347	0.000152
RP11-432J2	-0.493	0.206	0.597	0.241	-0.026	0.000152
POTEF	0.415	-0.218	-0.084	0.156	-0.223	0.000152
RPL37	-0.34	-0.188	-0.096	-0.623	-0.515	0.000152
RP11-567M1	-0.427	-0.126	-0.21	-0.697	-0.675	0.000152
DNAJC4	-0.123	0.061	0.032	-0.041	0.342	0.000153
MNS1	-0.066	-0.088	-0.176	0.131	-0.941	0.000154
PJA1	0.139	0.093	0.057	0.446	0.428	0.000155
LDOC1L	0.055	0.137	-0.036	0.217	0.327	0.000155
RPL38	-0.219	-0.216	-0.098	-0.454	-0.447	0.000155
RP4-614O4.	0.128	0.482	1.068	0.416	0.673	0.000156
SNW1	0.262	0.129	0.028	0.355	0.295	0.000156
JDP2	0.574	0.08	-0.087	0.066	0.278	0.000156
HMBS	-0.112	-0.281	-0.261	-0.169	-0.39	0.000156
LSM1	-0.156	-0.172	-0.123	-0.232	-0.541	0.000156
FMNL1	-0.025	0.447	0.495	0.036	0.895	0.000157
C11orf68	-0.033	0.066	0.137	0.367	0.224	0.000157
RP5-857K21	-0.231	-0.208	0.45	-0.932	-0.394	0.000157
ATP5I	-0.3	-0.195	-0.075	-0.5	-0.518	0.000157
FLII	0.189	0.086	0.119	0.334	0.364	0.000158
RTN4IP1	-0.023	-0.157	0.016	0.204	-0.501	0.000158
RP11-259N1	-0.647	-0.346	-0.443	-0.159	-0.676	0.000159
RP11-96H19	-0.075	-0.064	-0.054	-0.343	-0.934	0.000159
MYOM3	0.357	0.254	0.427	0.529	0.403	0.00016

SMPD1	0.208	0.072	-0.077	0.321	0.33	0.00016
RP11-17A4.1	-0.427	-0.337	-0.199	-0.674	-0.571	0.00016
MRPL44	0.276	0.206	0.037	0.388	0.323	0.000161
TRAIP	-0.265	-0.21	-0.237	-0.33	-0.536	0.000161
PSMD1	0.488	0.269	0.191	0.61	0.218	0.000162
SOBP	0.079	-0.197	-0.383	-0.496	0.116	0.000162
PIPSL	0.372	-0.013	-0.035	0.364	0.088	0.000162
CTC-281F24	-0.421	0.21	0.197	-0.483	-0.479	0.000162
CTC-548K16	-0.747	-0.021	0.194	-0.934	-0.603	0.000162
COMMD7	0.072	-0.016	0.027	0.171	0.358	0.000163
RF00092	-0.481	-0.226	-0.107	-0.852	-0.949	0.000163
GLDCP1	-0.098	-0.073	0.183	0.479	0.827	0.000164
SLC35A1	-0.414	-0.162	-0.085	-0.254	-0.291	0.000164
NPIPA3	-0.179	0.235	0.349	-0.674	-0.697	0.000164
HIPK2	0.254	0.121	0.255	0.306	0.339	0.000165
SNORA63	-0.48	-0.075	-0.138	-0.562	-1.059	0.000165
AEBP2	0.33	0.274	0.056	0.203	0.116	0.000166
JTB	-0.281	-0.233	-0.19	-0.468	-0.479	0.000166
NFYB	-0.147	-0.091	-0.338	-0.363	-0.283	0.000167
SCOC	-0.21	-0.144	-0.085	-0.368	-0.58	0.000167
SPACA6	-0.304	-0.276	-0.15	-0.878	-0.443	0.000168
CAPZB	0.303	0.069	-0.048	0.286	0.349	0.000169
GAS5	-0.32	0.04	0.117	-0.681	-0.383	0.00017
C2orf76	-0.318	-0.064	-0.197	-0.542	-0.676	0.000171
RP5-1103G7	-0.371	0.106	0.455	0.108	0.543	0.000172
RP11-124N1	0.099	-0.317	-0.092	-0.036	0.434	0.000172
DOCK7	0.326	0.016	-0.152	0.166	0.356	0.000172
ERCC6L2	-0.167	-0.155	0.073	0.015	0.349	0.000172
SNORD35A	-0.18	-0.226	-0.148	-0.856	-1.038	0.000172
TOM1L1	0.124	0.462	0.365	0.652	0.928	0.000173
TTC4	-0.042	-0.029	-0.01	0.315	-0.384	0.000173
THOC3	0.038	0.277	-0.106	0.447	0.477	0.000174
IK	0.253	0.181	0.134	0.459	0.368	0.000174
PPP2CB	0.369	0.156	0.06	0.33	0.25	0.000174
NFATC3	0.366	0.302	0.228	0.34	0.067	0.000174
AC007040.7	-0.688	0.017	0.457	0.542	-0.098	0.000174
RP11-571F1	0.227	-0.151	-0.386	0.004	-0.266	0.000174
THOP1	-0.065	-0.075	-0.284	-0.19	-0.407	0.000174

TIGD2	-0.008	0.212	-0.035	0.128	-0.57	0.000174
ZNF32	-0.446	-0.159	0.168	-0.01	0.265	0.000175
SIL1	0.243	-0.007	-0.085	0.377	0.144	0.000175
ARHGEF10	-0.281	0.018	0.348	-0.524	-0.17	0.000175
BCL2L12	-0.138	-0.098	-0.177	-0.172	-0.43	0.000175
DAPK1	-0.21	-0.141	0.019	-0.104	0.375	0.000176
METTL12	-0.203	0.185	-0.369	-0.359	-0.354	0.000176
NUDC	0.241	0.014	-0.06	0.314	0.349	0.000177
AC079776.2	-0.538	0.13	0.282	-0.135	0.147	0.000178
RP11-504P2	-0.661	-0.035	0.077	-0.72	-0.021	0.000178
RP11-93L9.1	-0.631	-0.159	0.196	-0.467	-0.204	0.000178
RP11-138H1	-0.139	0.564	0.376	-0.24	-0.735	0.000178
HLA-V	-0.011	-0.255	-0.041	0.22	0.701	0.000179
PGP	-0.292	-0.261	-0.259	-0.52	-0.304	0.000179
VCL	0.341	0.147	-0.094	0.078	0.364	0.000181
LBHD1	-0.57	-0.127	0.349	-0.23	-0.262	0.000181
RP11-513I15	-0.159	-0.287	-0.215	-0.416	-0.533	0.000181
HCFC1R1	0.02	-0.22	-0.23	-0.034	-0.467	0.000182
ZNF77	-0.236	-0.131	-0.247	-0.511	-0.689	0.000182
ZNF699	0.197	0.191	-0.083	-0.397	-0.475	0.000183
RP11-468E2	-0.925	0.092	-0.366	-0.951	-0.43	0.000186
EHHADH	0.208	0.055	0.21	0.717	0.386	0.000187
RP11-714G1	0.349	-0.223	-0.381	0.086	0.105	0.000187
PTMAP5	0.334	-0.063	-0.093	0.05	-0.095	0.000187
RP1-232L22_	-0.199	0.102	0.071	0.306	-0.372	0.000187
SNORA27	-0.406	0	-0.255	-1.038	-0.947	0.000187
AP000358.3	0.032	-0.164	-0.06	-0.001	-0.847	0.000188
CDKAL1	0.215	0.107	0.261	0.325	-0.005	0.000189
ARHGEF10L	0.109	0.048	0.002	-0.399	0.504	0.00019
COX20P1	-0.326	-0.217	-0.113	-0.332	-0.661	0.00019
DDX46	0.278	0.225	0.157	0.36	0.095	0.000191
CTD-2366F1	-0.561	-0.056	0.241	0.156	-0.223	0.000191
LSM5	-0.517	-0.263	-0.156	-0.564	-0.594	0.000191
UQCC2	-0.369	-0.217	-0.159	-0.427	-0.62	0.000192
FAM27E3	-0.385	0.06	0.12	-0.065	-0.768	0.000193
RF01977	-0.956	-0.299	0.198	-0.79	-0.34	0.000196
RPL37P23	-0.424	-0.312	-0.17	-0.639	-0.552	0.000196
POLR3GL	0.109	0.092	0.132	0.375	0.326	0.000197

CCDC57	-0.245	-0.052	0.27	-0.367	-0.235	0.000197
ACTR1A	0.297	0.103	0.031	0.348	0.406	0.000198
RP11-77H9.2	-0.65	0.096	0.015	-0.748	-0.115	0.000198
RP11-392E2	-0.312	-0.105	0.085	-0.716	-0.751	0.000198
CTC-429P9.5	-0.384	-0.44	0.166	-0.514	0.433	0.000199
RP11-473M2	-0.539	0.102	0.514	-0.299	0.39	0.000199
CALM3	0.152	-0.035	-0.009	0.323	0.206	0.000199
RP11-379B1	-0.955	-0.116	0.341	-0.197	0.105	0.000199
CABLES2	0.164	-0.056	-0.064	0.055	-0.379	0.0002
CTNNAP1	0.355	-0.089	0.057	0.385	0.545	0.000201
CLTC	0.453	0.18	0.09	0.538	0.493	0.000201
RP11-927P2	-0.218	0	-0.121	-0.981	-0.159	0.000201
DDX31	-0.369	-0.115	-0.116	-0.153	-0.262	0.000201
AC126118.1	0.262	0.194	-0.413	-0.466	-0.57	0.000201
ABCB9	0.038	0.255	0.335	0.284	0.35	0.000203
TCEAL3	0.213	0.13	0.116	0.416	0.275	0.000203
C12orf72	-0.432	0.172	0.302	0.326	-0.103	0.000203
CSRP2BP	-0.164	0.006	0.041	0.01	-0.488	0.000203
CTD-2006C1	0.437	0.283	-0.122	-0.312	-0.724	0.000203
SHCBP1	0.054	-0.238	-0.37	0.101	-0.146	0.000204
RASD1	0.268	0.351	-0.026	-0.028	0.511	0.000206
FAM98A	0.378	0.179	0.133	0.465	0.415	0.000207
CACFD1	-0.115	-0.236	0.227	0.336	0.077	0.000207
NCL	0.506	0.163	-0.012	0.333	0.035	0.000207
RPP21	0.003	0.091	0.045	-0.117	-0.63	0.000207
PDP2	-0.341	0.053	-0.264	-0.151	-0.343	0.000208
SYCE1L	-0.596	-0.175	0.037	-0.527	0.312	0.00021
RP11-541J2.	0.449	-0.19	-0.344	0.042	0.073	0.00021
PAF1	0.182	0.072	0.091	0.461	0.037	0.00021
LACTB2	-0.311	-0.047	0.082	-0.185	-0.562	0.00021
UBOX5	0.307	-0.014	0.054	0.396	0.35	0.000211
TMEM14A	-0.425	-0.298	-0.036	-0.068	0.268	0.000212
RP11-427L1	-0.682	0.246	-0.189	-0.978	-0.178	0.000212
SNORD57	-0.286	-0.288	-0.2	-0.963	-1.155	0.000212
CTA-407F11	0.001	-0.376	-0.288	-0.061	0.159	0.000213
OSER1	-0.148	0.124	-0.039	-0.104	-0.335	0.000213
WDR89	-0.423	-0.151	-0.192	-0.181	-0.397	0.000213
CTDP1	0.28	0.301	0.129	0.337	0.454	0.000214

XRCC5	0.442	0.197	0.066	0.543	0.333	0.000214
FGFR1OP	-0.401	-0.148	0.083	-0.178	-0.341	0.000214
TMEM218	-0.326	-0.289	-0.154	-0.383	-0.674	0.000214
ABCC2	-0.059	0.027	-0.028	-0.303	-0.819	0.000214
CLCN2	0.019	0.139	-0.031	-0.29	-0.466	0.000218
CCT8	0.34	0.177	0.022	0.329	0.135	0.00022
QRICH1	0.322	0.138	0.009	0.122	-0.023	0.000222
TRIM26	0.414	0.222	0.118	0.128	0.149	0.000224
RP11-421P1	0.548	-0.427	-0.146	-0.674	-0.618	0.000224
ARFGAP3	0.456	0.147	0.109	0.247	0.211	0.000225
VDAC1	0.391	0.128	-0.058	0.207	0.137	0.000225
LSMEM1	0.167	0.043	0.272	-0.658	-0.586	0.000227
LMO2	0.327	-0.201	-0.239	-0.018	0.007	0.000229
RP11-264M2	0.094	-0.288	-0.387	-0.494	-0.513	0.000229
STAM	0.382	0.255	-0.025	0.232	0.233	0.00023
NAT6	0.017	0.398	0.205	0.02	-0.115	0.000232
RP11-521B2	-0.077	-0.082	-0.095	-0.341	-0.637	0.000232
RP11-62H7.3	-0.691	-0.856	-0.48	-0.912	-0.884	0.000233
OGDH	0.456	0.183	0.117	0.561	0.243	0.000235
OSBPL1A	0.018	-0.001	-0.174	-0.012	0.34	0.000236
PEX11G	-0.268	0.072	0.282	0.346	0.87	0.000237
ZBTB37	-0.313	0.191	0.366	-0.148	-0.002	0.000238
SPRYD3	0.392	0.076	-0.043	0.176	-0.109	0.000241
AC019188.1	-0.326	-0.335	-0.219	-0.518	0.022	0.000243
ZNF180	0.026	-0.05	-0.132	-0.317	-0.408	0.000243
MAPK1IP1L	0.447	0.234	0.048	0.2	0.084	0.000244
GTF2F2	0.31	0.147	0.146	0.411	0.344	0.000247
PPME1	0.163	0.153	0.125	0.558	0.213	0.000247
CTB-161M19	0.4	0.125	0.048	0.322	0.14	0.000247
TGDS	-0.426	-0.144	-0.135	-0.452	-0.51	0.000247
RP5-831D17	0.128	-0.211	0.009	0.405	0.506	0.000249
RP11-512F24	0.3	0.164	0.063	0.466	0.094	0.00025
SNORD3A	0	0.583	0.545	-0.493	-0.076	0.000253
RP3-347M6.2	0.017	-0.413	-0.429	-0.215	-0.347	0.000253
IP6K1	0.182	0.125	-0.064	0.241	0.373	0.000254
GNAS	0.36	0.094	-0.078	0.221	0.036	0.000254
SMC3	0.409	0.058	-0.034	0.229	-0.049	0.000254
AC046143.5	-0.479	-0.328	-0.134	-0.54	-1.05	0.000254

RP11-611D2	-0.808	0.032	0.459	-0.29	-0.158	0.000256
RP11-736K2	-0.474	-0.507	-0.14	0.174	-0.242	0.000257
UBL3	0.408	0.227	0.007	0.281	0.34	0.000258
CCDC186	0.333	0.222	0.203	0.219	0.204	0.000258
CEP41	0.171	-0.097	0.029	0.329	0.123	0.000258
RP11-324H6	-0.353	0.097	0.039	-0.753	-0.809	0.000258
GAPDHP38	0.381	0.048	0.066	0.46	0.566	0.00026
OSBPL8	0.251	0.196	0.082	0.262	0.398	0.00026
ZNF236	-0.096	0.026	0.114	-0.163	-0.365	0.000261
ZNF446	-0.336	0.224	0.135	0.098	0.082	0.000262
MIR21	0.359	0.315	0.41	-0.746	-0.092	0.000262
ZNF593	-0.174	-0.136	-0.02	-0.019	-0.372	0.000262
RP11-1H8.2	0.317	0.048	-0.033	0.434	-0.051	0.000267
RP5-894A10	0.324	0.106	0.185	-0.616	0.139	0.000269
IRF7	0.187	0.382	0.217	-0.095	-0.021	0.000269
PGAP3	0.108	0.138	-0.008	-0.123	-0.397	0.000269
GLB1L	-0.04	-0.08	-0.061	0.175	0.573	0.00027
CTC-358I24.	-0.503	-0.002	0.158	-0.517	-0.344	0.00027
UQCR10	-0.192	-0.253	-0.094	-0.245	-0.354	0.00027
SLC2A3	-0.023	-0.136	-0.163	0.023	-0.342	0.000271
CKS1A	-0.532	-0.657	-0.364	-0.637	-0.826	0.000271
NUDT7	-0.232	-0.527	0.171	0.461	0.125	0.000272
COX20	-0.152	-0.139	-0.049	-0.206	-0.476	0.000272
COX16	-0.206	-0.156	-0.081	-0.477	-0.57	0.000273
XXbac-BPG3	-0.099	0.148	0.213	-0.921	-0.63	0.000273
HLA-K	-0.065	0.526	0.374	-0.32	0.217	0.000274
ZNF629	0.344	0.146	0.026	-0.014	0.012	0.000274
LDHAP7	0.337	-0.069	0.033	0.506	0.434	0.000275
ADO	0.151	-0.014	-0.039	0.152	0.323	0.000275
RP11-890B1	0.059	0.038	0.087	-0.025	0.587	0.000276
ESX1	-0.069	-0.494	-0.676	-0.252	-0.187	0.000276
RALGPS1	-0.079	0.373	0.764	0.237	0.522	0.000277
TMSB4XP4	-0.31	-0.437	-0.429	-1.06	-0.345	0.000279
FAM173A	-0.239	0.051	0.146	-0.264	0.327	0.00028
PMS2L3	-0.154	0.322	0.353	-0.305	-0.143	0.00028
ZNF791	0.358	0.328	0.209	0.127	0.117	0.000281
RBM43	-0.078	0.064	-0.005	0.432	0.357	0.000284
IPO5	0.387	0.112	0.014	0.288	0.309	0.000284

ZNF408	0.024	0.149	0.015	-0.065	-0.324	0.000284
PPM1J	-0.423	0.199	0.378	0.148	0.684	0.000285
TAPBPL	0.162	0.17	0.226	0.193	0.493	0.000285
RP1-146H21	-0.031	0.05	0.091	0.696	0.256	0.000285
BET1	-0.43	-0.18	0.036	-0.347	-0.268	0.000285
ARHGEF25	-0.153	0.329	0.083	-0.139	-0.403	0.000285
TM9SF4	0.18	0.1	0.042	0.277	0.341	0.000286
ZBTB49	-0.083	0.206	0.002	-0.405	-0.322	0.000286
TAF11	-0.182	-0.161	-0.087	-0.127	-0.363	0.000286
DYNC1H1	0.166	0.154	0.157	0.214	0.539	0.000288
ZNF44	-0.097	0.005	-0.161	-0.452	-0.441	0.000289
MT-TA	0.111	0.1	0.456	-0.776	-0.498	0.000289
RP11-278A1	0.056	-0.093	0.076	0.231	0.458	0.000292
SDHD	-0.011	0.001	0.006	-0.117	-0.336	0.000292
TRIM13	0.368	0.174	0.185	0.007	0.321	0.000295
SERPINE2	0.353	0.194	0.076	0.252	-0.301	0.000296
GTF2A2	-0.311	-0.124	-0.098	-0.476	-0.489	0.000296
SNORD65	-0.15	-0.06	0.133	-0.671	-0.838	0.000296
AC090018.3	-0.589	-0.042	0.054	-0.858	-0.905	0.000296
CRIPT	-0.255	-0.006	0.07	-0.198	-0.354	0.000298
RP11-196I18	-0.122	-0.197	0.065	-0.13	-0.698	0.000298
ABHD14B	0.169	0.12	0.127	0.361	0.361	0.000304
DET1	0.07	0.183	0.406	0.53	0.197	0.000304
FAM124A	-0.564	-0.333	-0.415	-0.317	-0.127	0.000304
IRF2BP1	0.012	0.001	-0.087	0.134	0.494	0.000305
RP11-105D1	-0.06	-0.315	-0.436	-0.073	-0.395	0.000305
ZNF83	-0.347	0.082	0.249	-0.381	-0.034	0.000307
CTD-2120M2	0.454	-0.027	-0.151	-0.201	-0.027	0.000308
ATAT1	-0.248	0.018	0.192	-0.41	-0.045	0.000309
FAM162A	-0.235	-0.19	-0.071	-0.269	-0.584	0.000311
RP11-849H4	-0.56	-0.091	-0.005	-0.527	-0.606	0.000311
RP11-841C1	0.146	-0.011	0.113	0.073	-0.343	0.000312
USP45	-0.359	-0.061	0.036	-0.435	-0.308	0.000313
NAGA	0.162	0.034	0.052	0.366	0.271	0.000314
RP11-631N1	-0.445	0.062	0.392	-0.483	-0.267	0.000316
RP11-69J16.	-0.395	-0.358	-0.215	-0.693	-0.724	0.000316
AC010894.4	-0.622	-0.603	-0.541	-0.651	-0.948	0.000316
L34079.4	-0.274	0.606	0.684	-0.188	0.335	0.000317

ZC3H8	-0.344	-0.096	0.019	-0.22	-0.309	0.000319
SPICE1	-0.07	0.026	-0.059	-0.203	-0.426	0.00032
TRIM23	0.364	0.327	0.197	0.181	0.014	0.000321
TTC21A	0.05	0.071	0.343	-0.523	-0.299	0.000322
VCP	0.541	0.174	-0.03	0.376	0.074	0.000325
RP11-21N3.1	-0.183	-0.244	-0.313	-0.18	-0.433	0.000325
SNORA26	-0.71	-0.151	-0.059	-0.83	-0.995	0.000326
GOLGA8O	-0.272	-0.001	0.659	-0.641	-0.027	0.000327
CTD-2013N1	-0.404	0.013	0.236	-0.636	-0.606	0.000327
NIPAL1	-0.005	0.265	0.415	0.45	0.38	0.000328
RP11-36B6.2	-0.604	-0.055	0.135	-0.55	-0.115	0.000329
ARSE	0.346	0.074	0.035	0.28	0.884	0.00033
ATIC	0.359	-0.001	-0.031	0.384	0.373	0.00033
RPL37AP1	-0.429	-0.38	-0.172	-0.583	-0.503	0.00033
PSMA6	-0.233	-0.073	-0.075	-0.393	-0.597	0.00033
RNF180	-0.231	-0.067	-0.124	-0.052	0.625	0.000331
IL6	-0.319	0.111	0.093	-0.217	-0.519	0.000331
RP1-71L16.2	-0.277	-0.273	-0.109	-0.659	-0.524	0.000331
RP11-515C1	-0.35	-0.384	-0.425	-0.47	-0.805	0.000331
KLHL35	-0.774	-0.107	0.194	0.243	0.174	0.000334
RP11-1012A	0.253	-0.04	0.003	-0.152	-0.738	0.000334
CD79B	-0.152	0.196	0.407	0.63	0.301	0.000338
ZDHHC17	-0.039	0.102	0.043	-0.353	-0.235	0.000339
TUBB	0.347	0.089	-0.007	0.496	-0.009	0.00034
PRDX3	-0.138	-0.109	-0.114	-0.238	-0.405	0.00034
OXNAD1	-0.369	-0.228	-0.101	-0.1	-0.467	0.00034
LYRM9	-0.683	0.094	0.043	-0.02	0.499	0.000341
PRRT2	-0.865	-0.444	-0.148	-0.83	-0.292	0.000342
SMIM7	-0.11	-0.079	-0.041	-0.07	-0.356	0.000344
CTD-2537I9.	-0.615	-0.501	-0.196	-1.2	-0.845	0.000344
RP11-548C2	0.217	-0.076	0.084	0.647	0.453	0.000345
HOXA7	0.297	-0.011	0.008	0.464	0.223	0.000347
DTWD1	-0.353	0.09	0.279	-0.132	-0.024	0.000347
RNA5SP383	-0.066	0.301	0.336	-0.658	-0.696	0.000347
UBXN11	-0.502	-0.033	0.188	-0.255	-0.39	0.000349
RP11-1398P	-0.106	-0.194	0.293	0.692	0.849	0.000351
MAGED2	0.268	0.111	0.017	0.403	0.278	0.000352
NFS1	0.135	-0.068	-0.056	0.372	0.017	0.000356

ATF7IP2	-0.71	-0.17	-0.109	-0.436	-0.136	0.000357
GLUD2	0.43	0.063	-0.095	0.177	0.175	0.000358
ST20	-0.54	-0.015	0.396	-0.145	0.156	0.000358
PA2G4P2	0.44	0.116	0.114	0.493	-0.074	0.000361
TRAK2	-0.486	-0.091	-0.04	-0.75	-0.543	0.000361
ATF1	-0.093	-0.037	-0.084	-0.032	-0.369	0.000363
AC009948.7	0.3	-0.151	-0.087	0.298	0.325	0.000366
ZNF880	0.084	-0.087	0.108	0.179	0.474	0.000368
RP11-488L1	-0.405	0.055	0.182	-0.647	-0.255	0.000368
WDYHV1	0.028	-0.065	-0.093	-0.456	-0.546	0.000368
RPAP3	0.397	0.163	0.055	0.392	0.194	0.000369
CAPRIN1	0.347	0.098	-0.035	0.308	0.347	0.00037
PRPF19	0.334	0.022	-0.099	0.334	0.112	0.00037
ZNF426	0.244	0.19	0.057	0.156	0.365	0.000372
LPP-AS2	-0.215	0.051	0.247	0.543	0.342	0.000375
ZNF229	0.231	0.44	0.185	0.059	0.205	0.000375
TMC7	0.555	0.112	0.217	0.244	0.438	0.000376
LIPT1	-0.282	0.031	0.12	-0.039	-0.548	0.000376
PIGBOS1	-0.153	-0.331	-0.112	-0.314	-0.505	0.00038
WDR83	-0.018	-0.04	-0.02	0.041	-0.435	0.000381
ZNF329	0.443	0.411	0.377	0.261	0.317	0.000383
SEMA4D	-0.455	-0.044	0.035	-0.108	0.102	0.000385
KIAA1586	-0.122	-0.012	-0.069	-0.201	-0.492	0.000386
TADA1	-0.072	0	-0.251	-0.256	-0.424	0.000388
ZNF101	-0.088	0.01	-0.282	-0.315	-0.375	0.000392
TBC1D31	-0.217	-0.137	-0.092	-0.117	-0.568	0.000392
NDUFB9	-0.251	-0.086	-0.063	-0.482	-0.614	0.000396
ENPP1	0.454	0.194	0.176	0.347	0.23	0.000409
ARMCX6	-0.193	-0.148	-0.172	-0.205	-0.453	0.000409
RP11-64D22	-0.328	0.205	0.318	0.029	0.525	0.00041
ZNF10	-0.073	0.124	0.045	-0.613	-0.345	0.00041
CTB-47B8.1	-0.274	-0.41	-0.374	-0.442	-0.512	0.00041
CTD-2022H1	0.102	-0.213	-0.116	0.167	0.422	0.000412
BORCS7	0.101	0.346	0.339	0.224	0.375	0.000418
LEPR	0.052	-0.092	-0.065	-0.066	-0.428	0.000418
RP11-344H1	-0.276	-0.271	-0.235	-0.681	-0.624	0.000419
FAM96A	-0.33	-0.103	-0.069	-0.37	-0.622	0.00042
TMEM67	-0.079	-0.071	0.102	-0.008	0.403	0.000421

ZNF440	-0.114	-0.007	-0.244	-0.433	-0.357	0.000421
C11orf73	-0.302	-0.117	-0.059	-0.357	-0.406	0.000421
AC138517.4	0.533	-0.045	0.004	0.354	0.369	0.000423
CBS	-0.666	-0.19	-0.467	0.239	0.035	0.000423
AF015726.1	-0.289	-0.3	-0.467	-0.779	-0.887	0.000423
FAM221A	-0.669	-0.028	0.064	-0.513	-0.23	0.000424
CCDC78	-0.152	0.062	0.051	-0.801	-0.757	0.000428
RP11-115C2	-0.727	-0.097	-0.224	-0.392	-0.625	0.000429
RP13-383K5	0.078	-0.275	-0.176	-0.033	-0.385	0.00043
POLR2J4	-0.117	-0.037	-0.114	-0.311	-0.55	0.00043
RP11-367E1	0.294	-0.04	-0.081	-0.133	-0.471	0.000431
RP11-66B24	-0.278	-0.14	-0.192	-0.027	0.631	0.000434
LRSAM1	0.018	0.084	0.23	0.338	0.23	0.000434
TARSL2	0.33	0.066	-0.135	-0.21	0.26	0.000435
MIR6845	-0.551	-0.057	0.261	-0.858	-0.683	0.000436
HGF	-0.051	0.329	0.039	-0.116	-0.733	0.000439
ZCCHC4	-0.025	0.141	0.067	-0.108	-0.326	0.000442
RP11-829H1	-0.313	-0.287	-0.358	-0.713	-0.537	0.000442
THRAP3	0.372	0.097	0.102	0.317	0.094	0.000443
RP11-66N24	-0.377	0.163	0.497	-0.184	0.248	0.000444
FAM133B	0.041	0.172	0.05	-0.471	-0.123	0.000444
RP11-701H2	-0.246	0.228	0.11	-0.717	-0.314	0.000447
PLA2G6	-0.205	0.097	0.413	-0.098	-0.353	0.000447
HDLBP	0.437	0.158	0.06	0.318	0.39	0.000451
SLC12A9	-0.103	-0.078	-0.057	-0.181	-0.348	0.000451
IMMT	0.371	0.149	0.086	0.456	0.261	0.000452
RP11-61N20	0.124	-0.146	-0.407	-0.339	-0.517	0.000455
AC005954.3	-0.094	0.279	-0.19	-0.951	-0.44	0.000457
ALPK1	-0.491	-0.122	0.108	0.051	0.301	0.00046
MSH3	0.454	0.241	0.177	0.445	0.309	0.000461
DNALI1	-0.074	0.106	0.153	0.088	0.858	0.000467
RP11-73E17	0.733	0.546	0.05	0.26	0.635	0.000468
PPIH	-0.104	-0.125	-0.179	0.065	-0.322	0.000468
TUBG1P	0.127	-0.149	-0.1	0.376	-0.258	0.000471
ADD1	0.29	0.039	-0.044	0.339	0.377	0.000472
SNHG8	-0.189	-0.01	0.078	-0.331	-0.414	0.000472
ECI1	0.051	0.007	0.026	0.201	0.35	0.000474
SNORA5C	-0.894	-0.115	-0.09	-0.706	-0.744	0.000474

HOTAIRM1	-0.629	-0.342	0.107	-0.587	-0.272	0.000478
TRAPPC2L	-0.211	-0.171	-0.137	-0.32	-0.388	0.000478
AC005682.5	0.059	0.04	0.238	-0.481	-0.449	0.000478
RP11-73M7.[illegible]	-0.347	0.169	0.599	-0.293	-0.295	0.000479
GARS	0.373	0.161	0.032	0.401	0.293	0.000482
HDAC4	0.266	0.067	0.333	0.257	0.018	0.000482
ZNF292	-0.038	0.069	0.037	-0.377	-0.021	0.000485
RP11-61L23.[illegible]	0.354	0.195	-0.083	0.231	-0.373	0.000485
MPPE1	-0.146	-0.058	-0.093	-0.389	-0.337	0.000488
RP11-708J1[illegible]	-0.061	0.098	0.331	-0.652	-0.513	0.000488
IFT80	-0.423	-0.026	0.148	-0.019	-0.023	0.000492
ELP3	0.327	0.165	0.139	0.502	0.291	0.000493
RASGRP2	-0.855	-0.432	-0.2	-0.628	-1.05	0.000493
CAND1	0.332	0.093	-0.037	0.237	0.249	0.000494
MRPS9	0.268	0.158	0.058	0.375	0.084	0.000494
ATP5G1	-0.244	-0.235	-0.158	-0.34	-0.411	0.000494
HERC2P9	-0.616	-0.117	-0.029	-0.807	-0.839	0.000494
JPX	-0.337	-0.184	-0.055	-0.417	-0.341	0.000495
KCND1	-0.369	0.217	0.366	-0.337	-0.258	0.000496
DSTNP2	0.209	-0.088	-0.034	-0.021	-0.372	0.000497
PRKCD	0.17	0.368	0.216	0.247	0.157	0.000498
MAML2	-0.022	-0.315	-0.409	-0.185	-0.051	0.000499
EID3	0.018	0.198	-0.011	-0.73	-0.706	0.000499
GCNT2	0.279	0.155	0.307	0.536	0.076	0.000504
RP13-228J1[illegible]	0.234	-0.171	-0.163	-0.134	0.372	0.000505
WBP2	0.18	0.088	0.137	0.374	0.405	0.000506
GRWD1	0.142	-0.061	-0.14	0.033	-0.327	0.000507
FOXO3B	0.665	0.242	0.21	0.254	0.562	0.000508
EIF5AL1	0.339	0.058	0.028	0.301	0.094	0.000511
NHLRC3	-0.369	-0.092	0.116	-0.19	-0.278	0.000511
PPIEL	-0.384	-0.127	0.141	-0.749	-0.505	0.000515
SLC35A3	-0.087	-0.073	-0.076	-0.195	-0.371	0.000519
PDK2	0.201	-0.014	0.074	0.304	0.405	0.00052
WDR73	-0.332	-0.166	0.044	-0.269	-0.297	0.000521
MED17	0.497	0.205	0.18	0.222	0.604	0.000525
PLGRKT	-0.101	-0.198	-0.277	-0.503	-0.465	0.000526
RAPGEF6	-0.214	0.118	-0.181	-0.345	-0.332	0.000527
CTC-534A2.[illegible]	-0.148	-0.036	-0.232	-0.559	-0.737	0.000529

NID1	0.261	0.135	-0.013	0.298	0.445	0.00053
SEC61A1	0.34	0.142	-0.003	0.207	0.121	0.000531
MST1P2	-0.655	0.027	0.147	-0.383	-0.707	0.000531
CRNKL1	0.279	0.246	0.086	0.396	0.11	0.000535
RP11-252C2	-0.209	-0.563	-0.372	0.075	-0.127	0.000536
VAMP2	-0.054	-0.015	-0.096	-0.347	-0.206	0.000537
RP11-727A2	-0.357	0.045	0.059	-0.805	-0.895	0.000537
GCNA	-0.27	0.116	0.411	-0.555	-0.005	0.00054
RPL26P30	-0.061	-0.107	-0.033	-0.286	-0.592	0.00054
FXYD5	0.243	0.042	0.023	0.363	0.214	0.000541
GNGT2	-0.341	-0.068	0.17	0.319	0.435	0.000542
RP1-68D18.2	-0.008	0.363	0.66	-0.653	0.105	0.000548
DAW1	-0.202	-0.329	0.18	0.444	-0.062	0.000548
ZNF714	-0.005	-0.201	-0.361	-0.596	-0.168	0.000548
ZBTB8OS	-0.108	-0.04	0.032	-0.24	-0.423	0.000549
ZNF215	-0.38	-0.081	-0.056	-0.162	-0.498	0.00055
HSP90AB3P	0.519	0.053	-0.029	0.417	0.274	0.000551
CBX7	-0.199	-0.082	0.397	0.14	0.107	0.000551
SCML2	0.269	0.482	0.469	0.173	-0.187	0.000552
HNRNPM	0.41	0.183	0.096	0.447	0.444	0.000553
TUBBP1	0.323	0.034	0.012	0.454	-0.084	0.000555
AVPI1	0.485	0.046	-0.217	-0.049	-0.299	0.000557
PROS1	0.359	0.296	-0.116	0.407	0.59	0.000558
C11orf83	-0.301	-0.366	-0.169	-0.249	-0.456	0.000558
RP11-478C1	-0.565	0.153	0.137	-0.666	-0.602	0.000559
SAR1B	0.009	-0.007	0.035	-0.098	-0.344	0.000561
GPI	0.369	0.1	0.052	0.497	0.482	0.000562
RP11-390F4	0.431	-0.145	-0.183	0.171	-0.088	0.000562
ISCA1	0.06	-0.108	-0.195	-0.27	-0.332	0.000568
TAP2	0.091	0.099	0.08	0.098	0.347	0.000569
KAT6B	0.301	0.238	0.33	0.059	0.072	0.000571
FRG1CP	-0.164	-0.019	-0.057	-0.455	-0.389	0.000574
C12orf48	-0.504	-0.234	-0.23	-0.383	-0.442	0.000574
FAM24B	-0.641	-0.074	0.02	-0.223	0.172	0.000577
NUP107	0.172	0.173	0.162	0.404	0.052	0.000577
SLC17A5	0.229	0.129	0.118	0.256	0.359	0.000579
ZNF282	0.053	0.096	0.068	0.119	0.353	0.000579
B4GALT6	0.139	0.265	0.132	0.174	0.661	0.000581

RP3-357D13	0.604	0.227	0.288	0.756	0.614	0.000587
HSPA9	0.451	0.234	0.034	0.386	0.133	0.000587
RP11-454H1	0.21	0.694	0.588	0.341	0.205	0.000592
RHOBTB2	0.005	-0.229	-0.157	0.088	-0.329	0.000593
MATN3	0.185	0.141	-0.066	0.214	0.58	0.000595
MICU1	0.113	0.105	0.013	0.348	0.307	0.000595
SHMT2	0.085	0.035	0.075	0.375	0.077	0.000595
EBLN2	-0.104	-0.019	0.285	-0.728	-0.455	0.000595
RP11-558F2	-0.878	-0.679	-0.413	-0.758	-1.101	0.000595
PLEKHG3	-0.383	-0.323	-0.349	-0.566	-0.179	0.000599
KMT2C	-0.288	0.015	-0.006	-0.338	0.017	0.0006
ERG	-0.009	-0.113	0.024	-0.419	-0.282	0.000603
CCDC24	-0.539	-0.033	0.167	-0.161	-0.557	0.000611
RP11-769O8	-0.827	-0.423	0.029	-0.831	-0.543	0.000612
NUP133	0.34	0.206	0.073	0.409	0.243	0.000614
CTD-2531D1	0.21	-0.158	-0.345	-0.117	0.117	0.000616
NBPF26	-0.516	-0.246	0.205	-0.543	-0.349	0.000616
CTC-338M1	-0.636	-0.26	-0.01	-0.459	-0.599	0.000616
RREB1	-0.331	-0.169	-0.155	-0.312	-0.13	0.000622
CTA-211A9.	-0.549	0.103	0.2	-0.318	-0.27	0.000624
C1orf213	-0.556	0.021	0.339	-0.608	-0.243	0.000631
RP11-255C1	0.176	-0.19	0.033	0.215	0.483	0.000634
CEP70	-0.508	-0.511	-0.291	-0.378	-0.166	0.000634
PUM1	0.178	0.097	0.012	0.173	0.37	0.000639
H6PD	0.139	0.167	0.164	0.418	0.186	0.000642
CTC-479C5.	-0.536	-0.151	-0.087	-0.813	-0.544	0.000645
NONO	0.302	0.1	-0.003	0.309	0.326	0.000647
TCF20	0.283	0.25	0.083	0.064	0.37	0.00065
SRP68	0.358	0.187	0.116	0.411	0.04	0.000652
RP11-685N1	-0.387	0.266	-0.112	-0.901	-0.667	0.000652
PFKL	0.097	0.079	0.049	0.196	0.358	0.000653
TEN1-CDK3	0.022	0.223	0.143	-0.674	-0.444	0.000654
ZNF577	-0.523	-0.087	0.138	-0.458	-0.392	0.000655
PSMG4	-0.393	-0.179	-0.12	-0.422	-0.351	0.000656
TADA2A	-0.288	-0.247	-0.121	-0.127	-0.326	0.000657
HIGD1A	-0.173	-0.084	-0.126	-0.325	-0.483	0.000659
ZNF799	-0.801	-0.337	-0.284	-0.573	-0.682	0.000661
AC092661.2	-0.266	-0.291	-0.551	-0.508	-0.576	0.000664

KIZ	0.095	0.436	0.217	0.225	-0.161	0.000666
RP11-819M1	0.159	-0.217	-0.212	0.146	-0.412	0.000667
UGGT1	0.176	0.098	0.035	0.281	0.339	0.000668
NKAP	0.156	0.223	0.137	0.408	0.289	0.000668
CNOT4	0.309	0.314	0.188	0.324	0.416	0.000669
AC078899.1	0.385	-0.027	-0.047	0.319	0.223	0.00067
RP11-732A1	-0.394	-0.025	-0.384	-0.807	-0.985	0.00067
RP11-85F14	-0.896	-0.238	-0.414	-0.644	-0.519	0.000674
INVS	-0.331	-0.044	0.061	-0.097	-0.06	0.000676
RP11-644F5	-0.338	0.067	0.214	-0.491	-0.464	0.000677
MRPS23	-0.177	-0.135	-0.044	-0.077	-0.453	0.00068
DPM3	-0.383	-0.289	-0.158	-0.275	-0.511	0.000682
RP11-764E9	-0.469	-0.097	0.172	0.177	0.398	0.000683
CTD-2196E1	-0.468	0.166	0.192	-0.61	-0.441	0.000689
VDR	0.201	0.14	-0.193	0.022	-0.487	0.00069
ZNF689	-0.05	-0.131	-0.082	-0.054	-0.428	0.000692
BEX5	-0.242	0.028	-0.034	-0.202	-0.672	0.000693
RP11-10O17	-0.455	-0.116	-0.21	-0.105	-0.792	0.000693
AQP7P1	-0.507	-0.031	0.181	-0.562	-0.855	0.000693
DPY19L4	-0.269	-0.16	-0.136	-0.282	-0.34	0.000694
CDK5RAP3	-0.209	0.101	0.064	-0.177	-0.405	0.000694
DNAJB5	-0.116	-0.123	-0.343	-0.225	0.091	0.000696
SLC37A2	0.014	0.094	0.368	0.298	0.822	0.000698
MIPEP	0.207	0.149	-0.123	0.348	0.235	0.000698
AC025171.1	-0.754	-0.292	0.061	-0.37	-0.225	0.000702
AC023356.3	0.163	0.086	-0.033	0.46	0.405	0.00071
ADAL	-0.233	0	0.282	0.324	0.251	0.000716
RGS14	-0.557	-0.399	-0.207	-0.332	-0.531	0.000716
TBC1D7	-0.126	-0.214	-0.309	-0.146	-0.409	0.000718
AC009302.3	0.514	0.046	0.054	0.4	0.049	0.000723
RP11-476C8	0.394	-0.092	-0.053	0.329	0.318	0.000726
LRRC70	-0.329	-0.394	-0.44	-0.673	-0.57	0.000728
HLA-DPB1	0.306	0.085	0.037	0.172	-0.454	0.000729
RP11-132A1	-0.369	-0.487	-0.397	-0.4	-0.995	0.00073
RP11-30E17	-0.671	0.055	0.344	-0.045	-0.463	0.000741
COBLL1	0.324	0.333	0.022	0.203	0.037	0.000746
CCDC18	-0.388	-0.24	-0.134	-0.145	-0.211	0.000747
AC005306.3	-0.067	0.23	-0.035	-0.704	-0.509	0.000747

ADRB1	-0.094	0.053	0.203	0.213	-0.34	0.000749
CTD-2267D1	-0.247	-0.023	0.228	0.477	0.212	0.000755
NPEPL1	-0.266	0.142	0.185	-0.034	0.43	0.000756
KLHDC1	-0.492	0.265	0.449	-0.139	0.112	0.000757
RB1	0.367	0.216	-0.025	0.249	0.258	0.000762
RP4-775C13	0.375	-0.042	-0.107	0.269	0.13	0.000763
RP11-288H1	-0.277	-0.192	0.013	-0.701	-0.816	0.000769
CIAPIN1P	0.315	-0.003	-0.152	0.362	0.094	0.000772
RP11-658F2	-0.05	0.021	0.512	-0.089	0.028	0.000773
DCXR	-0.093	-0.101	-0.228	-0.14	-0.406	0.000775
RP5-855D21	-0.057	0.075	0.261	-0.752	-0.385	0.000778
CFL1	0.34	0.057	-0.052	0.298	0.232	0.00078
FBXL14	0.008	-0.329	-0.374	0.029	0.108	0.000781
SPOP	0.049	0.131	0.164	0.326	0.167	0.000782
HSD17B8	-0.226	0.035	-0.111	-0.374	-0.747	0.000785
RNF20	0.148	0.055	0.163	0.461	0.313	0.000789
EFTUD2	0.404	0.167	0.016	0.414	0.33	0.00079
DUS4L	-0.421	-0.132	0.215	0.049	-0.029	0.000792
ZNHIT6	0.146	0.291	0.222	0.345	0.161	0.000794
XRCC1	0.015	-0.042	-0.037	0.389	-0.054	0.000796
PCED1B	0.073	0.155	-0.003	0.583	0.83	0.000797
STXBP1	0.186	0.008	0.087	0.278	0.338	0.000798
SLFN13	0.141	-0.216	-0.091	-0.269	-0.493	0.0008
IDH2	0.3	0.132	-0.007	0.294	0.368	0.000808
ADORA2B	0.16	-0.111	0.068	0.389	0.523	0.00081
RP11-77H9.5	-0.655	-0.118	-0.134	-1.105	-0.583	0.000811
RP11-359B1	-0.182	-0.056	0	0.097	0.855	0.000816
ZNF846	-0.043	0.479	0.521	0.281	0.614	0.000816
KLHL15	0.183	-0.115	-0.292	-0.205	-0.372	0.000821
MRPL57	-0.271	-0.337	-0.153	-0.191	-0.359	0.000825
LCORL	-0.138	-0.208	-0.443	-0.376	-0.199	0.000832
RP6-159A1.2	0.138	-0.172	-0.398	-0.027	-0.806	0.000834
RAB11FIP3	0.175	0.094	0.071	0.115	0.337	0.000835
AC012318.3	-0.035	0.095	-0.029	-0.46	-0.58	0.000839
RP11-473I1.9	0.261	0.142	0.101	0.131	0.325	0.000841
SNORA69	-0.138	-0.136	-0.179	-1.017	-0.754	0.000845
NUDT8	0.126	0.533	0.473	0.571	0.623	0.000846
HSPE1	-0.297	-0.249	-0.115	-0.529	-0.46	0.000846

AATF	0.4	0.173	0.12	0.518	0.105	0.000851
RP4-541C22	0.341	0.51	0.742	-0.022	-0.093	0.000851
PLAA	0.399	0.197	0.038	0.293	0.044	0.000852
AP000689.8	-0.721	-0.088	-0.344	-0.739	-0.857	0.000856
NDUFV2	-0.114	-0.107	-0.122	-0.287	-0.41	0.00086
ANG	0.268	-0.093	-0.353	-0.113	-0.633	0.00086
EPRS	0.399	0.184	0.07	0.56	0.312	0.000862
TTC28-AS1	-0.432	-0.17	0.036	-0.435	-0.346	0.000864
CORO6	-0.327	0.239	0.139	-0.616	0.087	0.000867
TM7SF2	-0.178	-0.068	-0.135	-0.273	0.332	0.000869
PDIK1L	-0.474	-0.116	-0.272	-0.428	-0.489	0.000871
RP11-213G2	-0.484	0.182	0.001	-0.416	-0.304	0.000872
OSBPL11	0.333	0.188	-0.061	0.201	-0.053	0.000873
AC010733.8	-0.193	0.137	0.192	-0.737	-0.692	0.000877
RP11-1033H	0.208	-0.128	0.194	0.541	0.834	0.000883
CNP	0.295	0.094	-0.054	0.334	0.32	0.000884
SNORD38A	-0.503	-0.199	0.132	-0.824	-0.895	0.000885
METTL1	0.116	0.065	0.032	-0.007	-0.354	0.000889
RP11-198P1	0.194	-0.109	-0.066	0.3	0.336	0.00089
GDF11	-0.294	0.021	0.114	-0.338	-0.057	0.000891
CCDC107	-0.214	-0.182	-0.096	-0.03	-0.384	0.000891
RP11-611O2	0.744	0.702	1.175	0.139	0.522	0.000896
ZBTB42	0.194	0.097	-0.002	0.035	0.507	0.000905
PTENP1	0.223	0.188	0.17	0.352	0.437	0.000907
AQR	0.298	0.231	0.119	0.407	0.321	0.000913
MTRNR2L12	-0.434	-0.47	0.116	-0.945	-0.676	0.000916
UHRF1BP1	0.343	0.04	0.015	0.123	0.057	0.000917
GNA14	0.755	-0.188	0.083	0.437	0.266	0.000921
AC008982.2	-0.39	-0.168	0.067	-0.522	-1.004	0.000921
PES1	0.339	0.098	-0.013	0.318	0.03	0.000923
RP11-616L1:	0.15	0.241	-0.618	-0.511	-0.268	0.000933
CTD-2647L4	-0.419	-0.072	-0.119	-0.828	-0.562	0.000933
SNHG6	-0.233	-0.085	-0.008	-0.427	-0.489	0.000936
FGFR1OP2	-0.183	-0.072	-0.131	-0.274	-0.389	0.000938
ZNF675	0.052	-0.179	-0.404	-0.488	-0.003	0.000939
SNORD56	-0.059	0.071	0.054	-0.784	-0.851	0.000942
RP13-582O9	-0.649	0.086	0.138	-0.427	-0.679	0.000949
WDR1	0.394	0.111	-0.042	0.35	0.232	0.00095

KTN1-AS1	-0.15	-0.601	-0.349	-0.39	-0.856	0.00095
ZCCHC18	-0.385	-0.303	-0.199	-0.184	0.418	0.000951
HOXB8	-0.566	-0.49	-0.259	-0.142	-0.362	0.000951
AC073869.1	0.236	-0.06	-0.33	0.124	0.4	0.000954
EXOSC4	-0.275	-0.077	0.025	0.194	-0.328	0.000956
STRBP	0.091	0.179	0.178	0.388	0.403	0.000962
ZNF74	-0.356	0.016	-0.069	-0.125	0.149	0.000963
RP11-163M2	0.397	-0.246	-0.076	0.038	-0.027	0.00097
RP11-10L12.	-0.222	-0.234	-0.288	-0.665	-0.99	0.000973
CCT5	0.3	0.054	-0.039	0.353	0.191	0.000979
HTATSF1	0.397	0.211	0.134	0.422	0.11	0.000981
OSBPL2	0.063	0.235	0.23	0.116	0.343	0.000982
KCTD18	-0.409	-0.06	0.181	0.046	-0.138	0.00099
SSRP1	0.344	0.098	-0.024	0.314	0.005	0.000991
CENPV	-0.064	-0.233	-0.257	-0.603	-0.188	0.000992
RP11-613F7	-0.553	-0.487	-0.237	-0.702	-0.573	0.000992
RP11-706O1	-0.049	0.101	0.265	-0.512	0.056	0.000993
STAMBP	0.082	-0.02	0.08	0.335	0.114	0.000998
PKP2	0.001	0.098	0.417	0.591	0.376	0.000999
CTD-2192J1	-0.124	-0.334	-0.295	-0.2	-0.072	0.001
CTBP1-AS1	-0.426	-0.135	-0.215	-0.261	-0.43	0.001
CTA-29F11.	-0.298	-0.452	-0.422	-0.557	-0.895	0.001
PAK1	0.165	0.163	-0.087	0.138	0.488	0.00101
PRKCSH	0.173	0.015	-0.027	0.31	0.34	0.00101
HADH	0.125	0.124	0.11	0.401	0.126	0.00101
TSEN15	-0.208	-0.115	-0.079	-0.077	-0.372	0.00101
CTRL	-0.446	0.01	0.106	-0.737	-0.597	0.00101
RP11-195O1	-0.266	-0.083	-0.028	-0.494	-0.652	0.00101
SNORA41	0.158	0.016	0.225	-0.74	-0.685	0.00101
RP1-69D17.	-0.629	-0.179	-0.725	-1.055	-0.821	0.00101
HSP90AB1	0.495	0.16	-0.002	0.458	0.341	0.00102
AC093734.1	-0.372	0.01	0.516	0.188	0.114	0.00102
LRP5	0.073	0.001	0.087	0.41	0.037	0.00103
RP11-488C1	-0.335	0.163	0.175	-0.481	-0.079	0.00103
SURF2	-0.061	-0.188	-0.12	-0.04	-0.457	0.00103
RNU6-301P	-0.293	0.409	0.293	-0.278	0.663	0.00104
TDRD3	0.015	-0.013	0.021	0.103	-0.355	0.00104
SNORD54	0.098	0.223	0.101	-0.646	-0.637	0.00104

LARP4P	-0.069	-0.331	-0.281	-0.275	-0.749	0.00104
RP5-1092A3	-0.191	0.124	0.754	-0.082	0.079	0.00105
RP11-305E1	-0.098	-0.554	-0.059	0.095	0.069	0.00105
RHBDL1	-0.32	0.038	0.411	-0.27	-0.5	0.00105
FOXRED2	0.162	0.113	0.145	0.426	0.29	0.00106
SEC23B	0.392	0.182	0.106	0.408	-0.092	0.00106
MTHFD1	0.337	0.075	-0.063	0.312	-0.098	0.00106
COA5	-0.562	-0.095	0.042	-0.317	-0.283	0.00106
CTD-2619J1	-0.415	0.256	0.12	-0.613	-0.638	0.00106
RP3-488M23	-0.166	-0.012	0.748	0.472	0.838	0.00107
FAM71F2	0.472	0.271	0.717	-0.229	0.702	0.00107
SLC35C1	0.252	0.038	0.118	0.273	0.438	0.00107
AGA	0.17	0.213	0.13	0.375	0.365	0.00107
BBS4	0.123	0.269	0.314	0.498	0.181	0.00107
NBPF14	-0.365	-0.205	0.143	-0.685	-0.007	0.00108
PMS2L1	-0.217	-0.284	-0.21	-0.369	-0.401	0.00108
RP1-90L14.1	-0.686	-0.117	0.165	-0.842	-0.581	0.00108
SF3A1	0.423	0.139	0.123	0.468	0.395	0.00109
AC006116.2	0.435	0.022	-0.063	-0.224	-0.577	0.00109
SEPW1	-0.318	-0.171	-0.042	-0.429	0.09	0.0011
AC004074.3	-0.196	-0.252	-0.251	-0.4	-0.486	0.0011
FRS3	-0.151	-0.084	-0.138	-0.421	-0.552	0.0011
RP11-347C1	-0.268	-0.105	0.001	-0.773	-0.7	0.0011
CHD6	0.094	0.099	0.1	0.005	0.341	0.00111
RP11-81A1.2	0.35	0.444	0.366	0.667	0.655	0.00112
PLEKHN1	-0.246	-0.118	0.439	0.041	0.398	0.00112
RP11-281P2	-0.601	-0.466	-0.403	-0.925	0.068	0.00112
AC064850.4	0.221	-0.216	-0.09	0.364	0.137	0.00113
TMEM177	-0.463	-0.201	-0.294	-0.295	-0.274	0.00113
HAUS1	-0.222	-0.021	-0.075	-0.295	-0.419	0.00113
RP11-343H5	-0.083	-0.356	-0.105	-0.544	-0.432	0.00113
COPG2	0.415	0.221	0.056	0.478	0.098	0.00115
RP11-758P1	-0.299	0.417	0.422	-0.41	-0.573	0.00115
VPS72	0.133	0.076	0.073	0.431	0.287	0.00116
PYY2	-0.223	0.75	0.735	-0.087	0.177	0.00116
ORC4	-0.387	-0.071	-0.029	-0.008	0.008	0.00116
AC005255.3	-0.148	-0.076	-0.057	-0.325	-0.483	0.00116
AC068492.3	0.428	-0.006	-0.131	0.29	0.316	0.00117

RP11-452L6.	-0.764	0.09	0.241	-0.022	-0.493	0.00117
PPIL4	0.215	0.19	0.147	0.41	0.218	0.00118
LRRC75A	-0.642	-0.473	-0.202	-0.264	0.024	0.00118
RP11-365P1	0.466	-0.101	-0.105	0.266	-0.006	0.00118
ANKRD44	-0.258	0.191	0.184	-0.077	0.468	0.00119
ST6GALNAC	-0.309	0.1	-0.078	-0.001	0.329	0.00119
AC156455.1	-0.199	0.01	-0.006	-0.362	-0.797	0.00119
SNORA71B	-0.421	-0.205	-0.114	-0.859	-0.961	0.00119
RP11-365K2	-0.39	-0.57	-0.401	-0.552	-0.087	0.0012
RP11-255N2	0.329	0.012	-0.052	-0.127	-0.426	0.0012
ZNF169	-0.287	-0.17	0.045	-0.584	-0.462	0.0012
IGF2	0.231	-0.019	-0.231	-0.115	-0.633	0.0012
AC122136.1	-0.237	-0.176	-0.114	-0.36	-0.155	0.00121
CEP192	-0.34	-0.164	-0.092	-0.066	-0.19	0.00121
MIR4691	-0.455	0	-0.189	-0.979	-0.77	0.00121
ENO1	0.366	0.079	-0.023	0.426	0.303	0.00122
ZNF658	-0.573	0.15	0.075	-0.219	-0.162	0.00122
FAM95B1	-0.098	0.279	0.648	-0.456	-0.294	0.00122
AC010746.4	0.474	-0.029	-0.097	0.205	0.013	0.00123
CEBPB	0.126	0.014	0.124	0.182	0.533	0.00124
RP11-282O1	-0.159	0.006	0.305	-0.048	0.391	0.00124
ARMCX1	0.178	0.07	0.187	0.36	0.116	0.00124
RP11-118D2	0.155	-0.137	-0.342	0.009	-0.336	0.00125
DDX21	0.441	0.158	-0.071	0.211	0.074	0.00126
ZNF784	0.388	0.079	-0.214	-0.067	-0.092	0.00126
SNRPE	-0.205	-0.068	0.013	-0.369	-0.399	0.00126
ECD	0.367	0.11	-0.029	0.323	0.113	0.00127
ACTB	0.451	0.073	-0.133	0.26	0.092	0.00127
XRCC4	0.174	0.099	0.162	0.354	-0.108	0.00127
TCEAL1	-0.303	-0.114	-0.119	-0.048	-0.322	0.00127
RPL9	-0.214	-0.081	-0.069	-0.472	-0.461	0.00127
AADAC	-0.337	0.275	0.277	-0.497	-0.057	0.00128
TMEM14C	-0.183	0.026	0.131	0.034	0.356	0.00129
TMEM45A	0.181	0.042	-0.082	-0.123	-0.428	0.00129
RP11-349A2	-0.253	-0.141	0.107	-0.437	-0.263	0.0013
C16orf91	-0.023	-0.026	-0.198	0.009	-0.369	0.0013
RP1-278E11	-0.204	-0.307	-0.104	-0.326	-0.438	0.0013
RP13-503K1	0.335	-0.205	-0.24	-0.082	0.009	0.00131

YJEFN3	-0.224	-0.091	0.523	-0.591	-0.02	0.00131
HOXA13	-0.04	-0.358	-0.416	0.057	-0.426	0.00131
SNORD6	-0.227	-0.165	-0.026	-0.863	-0.89	0.00131
RP5-1102E8	0.284	0.057	-0.001	0.395	0.102	0.00132
RP11-90H3.1	0.407	-0.014	-0.029	0.347	0.084	0.00132
RP11-686D2	0.085	-0.025	-0.042	-0.507	-0.699	0.00132
RP11-104N1	-0.556	0.295	0.434	0.181	0.656	0.00134
SEC23IP	0.239	0.133	0.108	0.37	0.361	0.00134
TMEM161A	-0.028	-0.014	-0.081	-0.011	-0.364	0.00134
SCX	0.176	-0.432	-0.367	-0.85	0.181	0.00135
AIFM1	0.339	0.087	-0.092	0.367	0.172	0.00135
FAM200B	-0.526	-0.178	0.037	-0.366	-0.058	0.00135
CTD-2033D2	0.301	0.001	0.097	0.59	0.538	0.00136
ZNF619	-0.352	0.139	0.012	-0.05	-0.239	0.00136
PSMA5	-0.137	-0.046	-0.03	-0.256	-0.387	0.00136
RP11-812E1	-0.75	-0.401	-0.005	0.155	-0.872	0.00136
FBXO21	0.375	0.198	-0.005	0.334	0.369	0.00138
TMEM129	-0.357	-0.056	-0.153	-0.11	-0.017	0.00138
ZNF35	0.362	0.183	0.084	-0.016	0.066	0.0014
SLC35A5	-0.224	-0.109	-0.02	-0.141	-0.418	0.0014
C9orf3	-0.128	-0.181	-0.149	-0.474	-0.414	0.00141
MMAA	-0.291	0.093	0.059	0.335	0.091	0.00142
RPS7	-0.203	-0.048	-0.034	-0.401	-0.42	0.00142
SMIM8	-0.603	-0.094	-0.285	-0.528	-0.506	0.00142
RP11-1217F	-0.037	-0.13	-0.044	-0.378	-0.679	0.00143
CTD-3018O1	0.073	0.031	0.445	0.417	1.026	0.00144
RF00569	-0.516	0.048	0.094	-0.714	-0.586	0.00144
ADCK2	0.041	0.137	0.222	0.323	0.04	0.00146
GHRLOS2	-0.161	0.394	0.505	-0.568	-0.155	0.00146
POLRMT	-0.372	-0.13	-0.057	-0.175	-0.289	0.00146
RP5-857K21	-0.425	-0.146	0.274	-0.824	-0.699	0.00146
MIR4784	-0.566	0.133	0.07	-0.653	-0.825	0.00146
GABBR2	0.387	0.032	-0.013	0.353	0.478	0.00147
SETD6	-0.301	-0.361	-0.002	-0.617	-0.345	0.00147
RP11-893F2	-0.407	-0.251	0.769	-0.067	-0.06	0.00148
RP11-214K3	-0.253	0.184	0.6	-0.537	-0.4	0.00148
ALKBH6	-0.354	0.135	0.225	-0.414	-0.505	0.00148
CTC-260E6.2	-0.216	-0.299	-0.1	-0.406	-0.359	0.00149

RNH1	0.226	0.027	-0.036	0.334	0.364	0.0015
GXYLT1	-0.027	0.14	0.123	-0.374	-0.136	0.0015
CCDC86	0.131	-0.079	-0.104	0.256	0.329	0.00151
TTC26	0.196	0.09	0.225	0.503	0.22	0.00151
RP11-3D23.1	0.469	0.048	0.148	0.336	0.156	0.00151
ZNF341	-0.19	-0.484	0.144	-0.034	-0.182	0.00151
CCDC134	-0.231	-0.074	-0.121	0.072	-0.508	0.00151
COX7A2	-0.418	-0.191	-0.059	-0.593	-0.657	0.00151
LINC00476	-0.712	-0.286	-0.083	-0.393	-0.122	0.00152
SNORD17	-0.113	-0.005	-0.27	-0.671	-0.699	0.00152
SDPR	-0.382	0.149	-0.108	0.102	0.128	0.00153
DMC1	0.095	0.05	-0.119	-0.179	-0.695	0.00153
ATP5A1P3	0.274	-0.035	-0.006	0.371	0.243	0.00154
MRPL36	-0.176	-0.304	-0.203	-0.231	-0.359	0.00154
RAB28	-0.088	-0.048	0.07	-0.036	-0.382	0.00155
CLEC12B	-0.318	-0.217	-0.165	-0.374	-0.761	0.00155
RAB43	-0.275	-0.043	-0.167	-0.091	0.568	0.00156
ATP1A1	0.297	0.146	0.008	0.373	0.299	0.00156
CREB3L4	-0.295	-0.051	0.222	0.326	0.041	0.00156
STXBP3	0.222	0.322	0.184	0.227	0.168	0.00157
DFFB	-0.376	-0.024	0.055	-0.183	-0.15	0.00157
ZNF785	-0.406	-0.125	-0.062	-0.382	-0.197	0.00157
RP11-21A7A	-0.575	0.188	0.211	-0.725	-0.54	0.00158
AP1G1	0.299	0.234	0.107	0.336	0.337	0.00159
SNORA53	-0.378	0.096	-0.124	-0.915	-0.778	0.00159
MIR137HG	-0.576	-0.211	-0.089	-0.268	-0.482	0.0016
RP11-715J2	-0.178	-0.33	-0.077	-0.339	-0.379	0.00161
LRP3	-0.043	-0.13	-0.351	-0.313	-0.022	0.00162
PSMA3	-0.149	-0.043	-0.019	-0.162	-0.439	0.00162
HSD3B7	-0.175	-0.173	0.316	0.029	0.343	0.00163
GRAMD4	-0.008	0.321	0.237	0.169	0.393	0.00164
SRSF5	0.061	0.07	0.126	-0.389	-0.047	0.00165
FAM98B	0.42	0.206	0.088	0.363	0.098	0.00166
LRRC75B	-0.44	0.029	-0.039	-0.492	0.062	0.00166
MEG8	-0.218	0.207	0.265	-0.49	-0.277	0.00167
PRELID3B	-0.293	-0.168	-0.108	-0.262	-0.364	0.00167
EIF5B	0.344	0.261	0.168	0.408	0.35	0.00168
USP14	0.371	0.206	0.103	0.406	0.315	0.00168

DCPS	0.348	0.072	-0.164	0.202	-0.088	0.00168
RP11-244H3	-0.076	-0.03	0.271	-0.918	-0.244	0.00168
RP11-641A6	-0.01	-0.025	0.056	0.41	0.43	0.00169
FAM201A	-0.219	0.024	0.27	-0.139	-0.644	0.0017
FAM229A	0.204	0.03	0.35	-0.32	0.863	0.00171
NDUFA6	-0.141	-0.146	-0.164	-0.14	-0.336	0.00171
RP11-680G2	0.256	-0.011	-0.077	0.439	0.598	0.00174
BGN	0.361	0.048	-0.124	0.322	0.284	0.00175
CS	0.34	0.168	0.021	0.281	0.163	0.00175
SF3B4	0.345	0.088	-0.048	0.276	-0.008	0.00175
APIP	-0.236	-0.152	-0.012	-0.204	-0.485	0.00175
CNR1	0.297	-0.116	-0.155	0.148	-0.477	0.00177
RP3-402G11	-0.485	0.395	-0.282	-0.453	-0.511	0.00177
ZNF143	0.008	0.168	0.14	0.28	0.378	0.00178
SNAI1	0.589	0.052	0.166	0.33	0.156	0.00182
ISOC1	0.422	0.251	0.064	0.241	0.129	0.00183
RP11-108A1	0.01	-0.27	-0.379	-0.021	-0.489	0.00183
DDX39B	-0.109	-0.106	0.002	-0.426	-0.557	0.00183
KCNIP4	-0.292	-0.465	-0.513	-0.187	-1.041	0.00183
CTB-52I2.4	0.399	0.085	0.085	0.302	0.149	0.00184
TTLL7	-0.007	0.219	0.32	0.008	-0.434	0.00184
MIR222	-0.557	0.357	0.368	-0.555	-0.028	0.00185
EGLN2	-0.149	-0.061	-0.21	-0.424	-0.256	0.00185
RP4-563E14	-0.7	0.156	-0.426	-0.913	-0.577	0.00186
C19orf76	-0.622	-0.322	-0.375	-0.824	-0.936	0.00186
POMK	-0.245	-0.338	-0.361	-0.389	-0.65	0.00188
RP1-228P16	-0.522	0.095	-0.08	-0.765	-0.701	0.00188
KBTBD4	0.017	0.084	-0.029	0.368	0.09	0.0019
RP11-574K1	-0.51	0.12	0.378	-0.515	-0.369	0.00191
CRNDE	-0.443	0.052	-0.132	-0.635	-0.385	0.00191
CTA-276O3.4	-0.501	-0.482	-0.323	-0.901	-0.851	0.00192
RFC1	0.342	0.131	-0.035	0.175	0.134	0.00193
SEPT15	-0.217	-0.029	-0.07	-0.452	-0.469	0.00193
CTD-2349P2	0.215	-0.089	0.093	-0.795	-0.5	0.00194
GSTM2	-0.069	0.184	0.333	-0.117	0.693	0.00195
BISPR	-0.321	-0.242	0.217	0.016	0.358	0.00195
TPT1-AS1	-0.297	-0.128	0.065	-0.552	-0.025	0.00195
ZNF124	0.278	-0.138	-0.379	-0.268	0.061	0.00196

CLASP1	-0.019	0.027	0.075	0.167	0.323	0.00198
TFG	0.331	0.079	-0.007	0.274	0.27	0.00198
RP11-386I14	-0.183	-0.45	-0.226	-0.421	-0.243	0.00198
SNORD12B	-0.031	0.038	0.08	-0.851	-0.615	0.00198
USP32	0.189	0.098	0.217	0.34	0.207	0.00199
CTD-2331H7	0.164	-0.362	-0.427	-0.296	-0.473	0.00199
CTB-50L17.1	0.077	0	-0.006	0.261	0.35	0.002
RP11-266K4	-0.199	0.7	0.77	0.099	0.121	0.002
DDX19A	0.342	0.165	0.045	0.433	0.11	0.002
U47924.31	-0.081	-0.47	-0.692	-0.342	-1.033	0.002
CTD-2078B5	0.343	-0.312	0.161	0.321	0.916	0.00201
GPKOW	0.351	0.231	0.127	0.525	0.163	0.00201
C19orf68	-0.406	-0.077	0.136	-0.557	-0.22	0.00202
NCRNA0010	-0.772	-0.213	-0.145	-0.998	-0.63	0.00202
RP11-567P1	0.252	0.143	0.227	0.298	0.417	0.00203
TPI1	0.251	0.072	-0.021	0.345	0.081	0.00203
RAB20	0.587	-0.233	-0.087	0.155	-0.124	0.00203
AC104651.1	-0.565	-0.377	-0.321	-0.717	-1.096	0.00203
MICU3	-0.353	-0.078	-0.157	-0.498	-0.426	0.00204
MFSD7	-0.423	-0.017	-0.038	-0.662	-0.506	0.00204
TBC1D3H	-0.726	0.023	0.098	-0.826	-0.595	0.00204
RP11-425I13	-0.376	-0.445	-0.393	-0.796	-1.152	0.00204
RP1-228H13	0.094	0.378	-0.219	0.183	0.666	0.00206
RNGTT	0.211	0.3	0.175	0.421	0.236	0.00206
SLIT2	0.154	0.154	0.032	0.323	-0.128	0.00206
XYLB	0.018	-0.228	-0.202	-0.391	-0.465	0.00207
RP11-566K1	-0.316	-0.682	-0.313	-1.005	-0.974	0.00207
CNIH1	-0.071	-0.104	-0.104	-0.37	-0.35	0.00208
RP11-65F13	-0.174	-0.162	0.024	-0.138	-0.388	0.00208
C5orf28	-0.304	-0.242	-0.093	-0.283	-0.52	0.00209
SNORD68	-0.126	0.136	0.131	-0.713	-0.734	0.00209
CTD-2309M1	0.397	-0.14	-0.213	0.13	-0.093	0.0021
PDCD10	-0.262	-0.083	-0.025	-0.461	-0.394	0.0021
RP11-95I19.3	-0.539	-0.286	-0.06	-0.191	-0.504	0.0021
IL20RB	-0.145	0.121	-0.057	-0.679	-0.773	0.0021
RP11-204J18	-0.008	0.044	0.146	-0.272	-0.909	0.0021
EIF4G1	0.385	0.054	-0.017	0.368	0.183	0.00211
RP11-1136G	0.394	-0.021	0.125	0.346	0.061	0.00211

RP11-35B20	-0.37	-0.327	-0.127	-0.442	-0.63	0.00212
RP11-274B2	-0.104	0.001	0.055	-0.827	-0.422	0.00213
RP11-404O1	0.36	0.172	-0.064	0.051	0.243	0.00214
ARL4D	0.172	-0.226	-0.361	0.027	-0.287	0.00214
RP11-244J1(	-0.489	-0.397	-0.276	-0.775	-0.446	0.00214
SNRPEP4	-0.14	-0.189	-0.049	-0.35	-0.477	0.00214
RP11-307L3.	-0.399	-0.231	-0.132	-0.662	-0.434	0.00216
RP11-535K1	0.536	-0.052	-0.162	0.091	0.055	0.00217
RP1-257A7.4	-0.704	-0.524	0.112	-0.51	-0.787	0.00217
POTEE	0.416	-0.145	-0.189	0.257	0.043	0.00218
AC004980.7	-0.611	-0.088	0.032	-0.454	0.274	0.00219
NCLP1	0.4	-0.102	-0.276	0.116	-0.315	0.00219
MRPL32	-0.286	-0.268	-0.23	-0.382	-0.501	0.00219
FAM195A	-0.389	-0.195	-0.148	-0.169	0.179	0.0022
TXN	-0.252	-0.074	0.004	-0.337	-0.483	0.0022
AP3B1	0.422	0.217	0.09	0.51	0.414	0.00221
CRADD	-0.067	-0.049	0.068	0.393	-0.018	0.00221
AC010095.6	-0.015	0.387	0.088	-0.093	-0.469	0.00221
THG1L	0.23	0.182	0.107	0.348	0.404	0.00222
REL	0.127	0.044	-0.144	-0.378	0.007	0.00222
POLR2I	-0.238	-0.212	-0.013	-0.14	-0.375	0.00223
THAP2	-0.122	0.125	0.306	0.154	0.421	0.00224
TTC30B	-0.204	0.204	0.244	0.641	0.351	0.00224
AC073958.2	-0.212	-0.4	-0.491	-0.829	-0.56	0.00224
ZNF625	-0.198	0.052	-0.265	-0.795	-0.744	0.00224
RP11-572H4	0.09	-0.034	0.037	0.342	0.491	0.00225
ZNF606	-0.241	0.384	0.311	0.107	0.249	0.00225
MRPL46	-0.267	-0.222	-0.084	-0.086	-0.425	0.00226
ZFHX4-AS1	-0.282	-0.082	-0.196	-0.368	-0.521	0.00226
PEX12	-0.033	0.259	0.125	0.351	-0.043	0.00227
USP11	0.448	0.241	0.065	0.281	0.064	0.00228
AMDHD2	-0.243	-0.107	-0.073	-0.574	-0.344	0.00228
ZDHHC21	-0.113	-0.109	-0.086	-0.398	-0.162	0.00229
TMLHE	0.112	0.045	0.002	0.29	0.371	0.00231
ABAT	0.323	0.058	0.231	0.616	0.53	0.00232
RAB43P1	-0.004	-0.112	-0.093	0.16	0.472	0.00232
FAIM	0.333	0.071	0.016	0.415	0.28	0.00232
RP11-554J4.	-0.291	0.125	0.037	-0.55	-0.894	0.00232

RP11-343C2	0.018	0.064	0.054	0.319	0.435	0.00233
RP11-629O1	-0.72	0.128	0.08	-0.776	-0.413	0.00233
RP11-395I14	-0.365	-0.027	0.074	-0.815	-0.716	0.00233
RP11-793H1	-0.635	-0.02	0.128	0.036	-0.823	0.00233
TNFAIP8	0.299	0.527	0.26	0.154	0.393	0.00234
RP6-145B8.3	0.313	-0.091	-0.055	0.372	0.181	0.00234
EFCAB7	-0.445	-0.246	-0.139	-0.054	-0.539	0.00234
SNORD88A	-0.394	-0.335	-0.66	-1.089	-0.883	0.00234
AP000361.2	-0.336	-0.413	-0.172	-0.51	-0.602	0.00235
TRPM4	-0.203	-0.015	-0.012	-0.329	-0.159	0.00236
NEMP2	0.021	0.218	-0.081	-0.192	-0.402	0.00236
RP11-525A1	-0.309	-0.142	0.219	0.07	0.564	0.00237
TTC27	0.307	0.197	0.094	0.404	0.194	0.00238
RP11-169K1	-0.514	-0.572	-0.136	-0.391	-0.601	0.00238
ZNF764	-0.142	0.22	-0.137	-0.358	-0.295	0.00239
MT-TE	-0.423	-0.26	0.199	-0.876	-0.612	0.00239
RP11-280G1	0.411	-0.042	0.12	0.402	0.132	0.0024
RRP1	-0.049	-0.051	-0.193	-0.187	-0.408	0.0024
RP11-617F2	0.418	-0.036	0.291	0.195	0.797	0.00241
TMEM143	-0.324	0.011	0.172	0.112	-0.382	0.00241
RN7SL81P	-0.452	0.059	0.248	-0.834	-0.483	0.00241
CARS	0.192	0.124	0.176	0.376	0.231	0.00242
LINC00493	-0.35	-0.281	-0.131	-0.436	-0.6	0.00242
RF00411	-0.115	0.226	0.487	-0.576	-0.39	0.00244
AC009120.6	-0.148	-0.074	0.291	-0.603	-0.021	0.00245
RP11-419C5	-0.279	-0.078	-0.211	-0.815	-0.769	0.00245
ZNF195	0.002	0.357	0.297	-0.015	0.23	0.00246
HARS	0.329	0.125	0.016	0.273	0.204	0.00246
RP11-449L1	0.416	-0.055	-0.069	0.268	0.086	0.00249
SLC22A5	-0.197	-0.066	0.317	0.221	0.463	0.00251
NUDT6	-0.528	-0.434	0.29	0.03	-0.018	0.00254
RP5-894A10	-0.065	0.145	0.074	-0.601	0.116	0.00255
SLC4A1AP	0.182	0.145	0.072	0.331	-0.056	0.00255
NDUFAB1	-0.156	-0.099	-0.044	-0.282	-0.422	0.00255
RP4-814E15	-0.288	-0.649	0.012	-0.247	-0.754	0.00255
ATP6V1A	0.383	0.291	0.161	0.531	0.2	0.00256
RP11-398F1	-0.82	-0.069	0.213	-0.253	0.086	0.00256
SMUG1	-0.349	-0.13	-0.102	-0.11	-0.314	0.00256

MKS1	-0.265	-0.045	0.022	0.016	-0.345	0.00256
AC017116.1	-0.037	0.602	0.419	-0.039	0.51	0.00257
ARMC7	0.439	0.107	0.063	0.247	-0.02	0.00257
CHKA	0.128	-0.086	-0.159	-0.123	0.357	0.00258
PPP1R3C	0.455	0.165	0.153	0.075	0.231	0.00258
PBX2P1	-0.368	-0.233	-0.044	-0.257	-0.295	0.00258
RPL17	-0.263	-0.012	-0.002	-0.404	-0.375	0.00258
ATRN	0.184	0.069	-0.006	0.245	0.332	0.00259
NME3	-0.328	-0.161	-0.154	-0.421	0.034	0.00259
TMEM86A	0.305	0.471	-0.165	-0.016	-0.003	0.00259
FUT10	-0.382	-0.178	-0.167	-0.22	-0.363	0.00259
RP11-511H9	-0.236	-0.391	-0.179	-0.673	-0.607	0.00259
MID1IP1	-0.013	-0.031	-0.064	-0.067	-0.392	0.00262
RP11-1149O	-0.182	0.298	0.501	0.179	0.463	0.00263
PSCA	-0.305	0.239	0.57	0.438	0.357	0.00263
COQ3	0.007	-0.14	0.081	-0.012	-0.484	0.00263
ZNF669	-0.123	-0.026	0.082	0.142	0.448	0.00264
WDR7	0.287	0.259	0.281	0.504	0.204	0.00264
HSPA1B	-0.612	-0.073	-0.326	-0.127	-0.251	0.00264
TYW5	-0.424	-0.042	0.035	-0.439	-0.259	0.00264
TRAPPC13	-0.175	0.042	0.044	-0.111	-0.456	0.00264
DLST	0.353	0.152	0.099	0.256	0.234	0.00265
CMAHP	-0.87	-0.461	-0.48	-0.75	-0.322	0.00265
GGACT	0.16	0.003	0.233	0.481	0.502	0.00266
GPATCH3	0.368	0.161	0.095	0.163	-0.018	0.00266
FOXL1	0.226	0.07	0.266	0.374	-0.108	0.00266
RP11-838N2	-0.286	-0.093	-0.512	-0.552	0.509	0.00267
BOLA2B	-0.263	-0.199	-0.232	0.218	0.42	0.00267
CLIP3	0.129	0.315	0.329	0.551	0.362	0.00269
RP11-176H8	-0.231	-0.308	-0.252	-0.444	-0.325	0.00269
MIR4321	-0.132	-0.335	-0.277	-0.654	-0.662	0.00269
LRRC37B	0.003	0.062	0.101	-0.482	-0.275	0.0027
AC009945.3	-0.258	-0.371	-0.187	0.06	-0.207	0.00271
RP11-33B1.3	-0.783	-0.144	-0.381	-0.941	-0.914	0.00271
LAMTOR5	-0.115	-0.119	-0.057	-0.276	-0.35	0.00272
CAPS	-0.256	0.168	0.406	-0.236	0.222	0.00273
IDE	0.279	0.269	0.146	0.391	0.092	0.00274
CIDECP	0.09	0.1	0.011	0.001	-0.353	0.00274

CHCHD5	-0.111	-0.182	-0.094	-0.293	-0.443	0.00274
RP11-458J1.	-0.208	-0.124	-0.29	-0.172	-0.892	0.00274
LRCH4	-0.369	0.012	-0.116	-0.447	-0.215	0.00276
SLC2A1	-0.096	-0.135	-0.328	-0.091	-0.35	0.00276
MALSU1	-0.38	-0.237	-0.142	-0.321	-0.36	0.00276
C5orf63	-0.044	-0.081	0.674	0.047	0.623	0.00278
SLC10A3	0.159	0.073	0.099	0.338	0.238	0.00278
RP11-18H7.1	-0.522	-0.328	0.179	-0.649	-0.037	0.00278
PIGL	-0.365	-0.032	-0.086	-0.546	-0.352	0.00278
CLEC2D	-0.357	0.278	0.361	-0.267	-0.015	0.00279
PARD6G	0.072	0.649	0.534	0.527	0.822	0.0028
CA13	0.548	0.614	0.469	0.288	0.382	0.0028
ACOT1	-0.098	-0.099	-0.047	0.249	0.64	0.00282
HSD17B7P2	-0.51	-0.246	-0.211	-0.931	-0.565	0.00284
RP11-478C6	-0.381	-0.298	-0.14	-0.567	-0.492	0.00285
RP4-575N6.2	-0.436	-0.368	-0.275	-0.96	-0.718	0.00285
ZNF576	-0.186	-0.135	-0.152	-0.071	-0.418	0.00288
RP11-304L19	-0.417	-0.092	-0.199	-0.741	-0.812	0.00289
TWNK	-0.192	-0.054	-0.196	-0.214	-0.372	0.0029
TMEM258	-0.228	-0.225	-0.078	-0.429	-0.421	0.0029
RP11-326I11	-0.454	0.067	0.094	0.416	-0.21	0.00291
RPL37A	-0.356	-0.162	-0.051	-0.478	-0.449	0.00291
PEMT	0.077	-0.107	-0.18	-0.129	-0.362	0.00293
GRAPL	0.481	-0.205	-0.292	-0.455	0.329	0.00296
ZNF280D	-0.224	-0.088	0.065	-0.378	-0.288	0.00296
PLCD4	0.136	-0.056	0.122	0.08	0.647	0.00297
HECTD1	0.177	0.092	0.048	0.235	0.331	0.00298
TIPRL	-0.161	-0.104	-0.05	-0.113	-0.39	0.00298
TMEM170B	-0.038	0.076	0.419	0.158	-0.07	0.00299
CTB-113I20.	-0.519	0.077	-0.112	-0.453	-0.984	0.00299
C8orf76	-0.188	-0.043	-0.145	-0.401	-0.544	0.00301
RP4-647C14	0.071	0.019	-0.101	-0.847	-0.595	0.00301
RP1-206D15	-0.188	-0.295	-0.043	-0.864	-0.786	0.00301
RP11-1136G	-0.227	-0.237	-0.118	-0.306	-0.525	0.00302
SNORD36C	-0.208	-0.156	0.076	-0.796	-0.807	0.00303
RP11-638I2.2	-0.356	0.24	0.233	-0.741	-0.48	0.00305
RP11-40F6.2	0.381	0.057	0.203	0.579	0.604	0.00306
RP11-409C1	-0.262	-0.187	-0.092	-0.324	-0.483	0.00306

SNAPC5	-0.296	-0.164	0.113	-0.062	-0.41	0.00307
NPIPB5	-0.173	0.092	0.052	-0.697	-0.545	0.00308
AC079135.1	-0.714	-0.297	-0.331	-0.311	0.156	0.00309
TMOD4	-0.233	-0.026	0.404	-0.577	-0.225	0.00309
KCNMB3	-0.918	-0.356	-0.204	-0.754	-0.569	0.0031
YAF2	-0.203	-0.103	-0.194	-0.472	0.086	0.00316
GNB2	0.224	0.072	0.042	0.365	0.29	0.00317
ZNF701	-0.421	-0.096	-0.154	-0.435	-0.124	0.00318
ASAH2B	0.469	0.11	0.491	0.465	0.443	0.00319
RP3-390M24	-0.291	-0.215	-0.372	-0.715	-0.705	0.00325
AC074289.1	0.365	0.244	0.742	0.329	1.097	0.00328
COL4A2	0.375	0.116	-0.139	0.268	0.204	0.00328
RP11-290D2	-0.529	-0.868	-0.637	-0.902	-1.083	0.00329
TANGO6	0.032	-0.073	0.096	0.381	0.083	0.00331
RPS6KA1	0.325	0.181	0.084	0.339	-0.005	0.00332
COMMD10	-0.214	-0.176	-0.182	-0.522	-0.232	0.00332
RP11-356O2	0.378	0.085	-0.017	0.353	0.167	0.00334
RP11-478K7	-0.406	-0.442	-0.299	-0.535	-0.559	0.00334
RAET1L	0.256	-0.138	-0.322	-0.184	-0.881	0.00334
AC073150.6	-0.039	-0.123	-0.404	0.372	0.347	0.00336
RP11-497H1	-0.873	-0.405	-0.141	-0.871	-0.843	0.00336
FABP5	0.024	0.014	-0.179	-0.257	0.382	0.00338
RP11-123D1	0.447	0.052	0.029	0.343	0.365	0.00338
ZNF595	-0.193	-0.106	-0.376	-0.54	-0.474	0.00338
SUB1P1	-0.088	-0.109	-0.087	-0.412	-0.478	0.00339
KLHL26	0.411	0.119	-0.035	0.086	-0.163	0.00341
SWAP70	0.345	0.075	0.018	0.31	0.21	0.00342
FOXN3	0.354	0.116	0.114	0.323	0.244	0.00343
COPG1	0.458	0.105	-0.034	0.258	0.157	0.00345
ALKBH3	0.123	0.06	0.099	0.324	0.059	0.00346
CENPH	0.009	0.127	0.189	0.473	0.081	0.00348
ARHGEF18	-0.04	0.148	0.07	0.059	0.454	0.00349
MAGEF1	0.021	0.073	0.04	0.433	0.056	0.0035
RP11-474D1	-0.622	-0.29	0.411	-0.482	0.131	0.00353
RP4-773N10	-0.215	-0.077	0.107	-0.356	0.098	0.00353
IRAK1BP1	-0.688	-0.271	0.06	-0.142	-0.025	0.00353
NUDT18	-0.488	0.013	0.035	0.114	-0.091	0.00353
PIGM	-0.104	0.004	-0.008	0.021	-0.33	0.00353

CTD-2090I13	0.416	-0.167	-0.018	0.348	0.04	0.00355
CTC-359D24	-0.268	0.055	-0.445	-0.726	-0.67	0.00355
SNORA71A	-0.738	-0.134	-0.138	-0.669	-0.959	0.00356
MAPK13	0.233	-0.052	-0.201	-0.41	0.2	0.00357
INO80B	-0.109	0.419	0.532	0.121	-0.117	0.00357
SNORD37	-0.487	0.093	0.169	-0.626	-0.741	0.00357
PARN	0.225	-0.028	0.014	0.372	0.069	0.00358
C6orf163	-0.215	0.269	0.429	-0.487	-0.398	0.0036
RP11-392A2	-0.422	-0.172	0.228	0.32	-0.291	0.00361
MIR3153	-1.018	-0.573	-0.371	-0.989	-0.85	0.00361
ZXDA	0.166	0.048	-0.018	-0.075	0.446	0.00362
RN7SL834P	-0.231	0.268	0.686	-0.363	-0.136	0.00363
RP11-758H9	-0.687	-0.268	-0.321	-0.496	-1.043	0.00365
GNG11	-0.289	-0.308	-0.263	-0.462	-0.086	0.00366
TSTD1	-0.461	-0.345	-0.209	-0.672	-0.369	0.00367
RP13-34C21	0.076	-0.324	-0.552	-0.023	0.329	0.00368
PTCD1	-0.27	-0.14	-0.298	-0.43	-0.573	0.00369
PPP1R3F	-0.275	0.186	0.406	-0.446	-0.061	0.00372
RPAIN	-0.276	-0.074	-0.008	-0.4	-0.262	0.00374
THAP6	-0.138	0.049	-0.012	-0.189	-0.503	0.00375
STMN3	0.142	0.064	0.238	0.38	-0.338	0.00376
RP11-861E2	-0.528	-0.214	0.258	-0.564	-0.448	0.0038
FBXL15	-0.539	-0.054	-0.169	-0.367	-0.115	0.00382
SNORD63B	-0.311	-0.261	-0.297	-1.042	-0.742	0.00382
FANCL	-0.222	-0.007	-0.128	-0.277	-0.338	0.00383
MDP1	-0.238	-0.268	-0.198	-0.475	-0.971	0.00385
MST1L	-0.758	-0.107	0.13	-0.53	-0.522	0.00386
SGF29	-0.077	-0.139	-0.166	-0.155	-0.516	0.00387
RP11-748L13	0.22	-0.071	0.292	0.269	0.942	0.00389
CTC-471J1.1	-0.09	-0.084	-0.019	-0.602	-0.832	0.00389
UBA1	0.295	0.11	0.039	0.423	0.461	0.0039
HIST1H2BC	0.45	0.679	0.811	0.99	0.377	0.0039
CMB9-22P13	-0.284	0.026	0.618	0.355	0.625	0.00392
EPB41L4A-A	0.026	0.126	0.071	-0.397	0.005	0.00392
RP11-365H2	-0.05	-0.249	-0.088	-0.209	-0.433	0.00393
RP11-327J17	-0.935	-0.504	-0.206	-0.461	-0.501	0.00393
EIF4E3	0.442	0.649	0.606	0.645	0.475	0.00394
GALNT14	-0.098	-0.11	-0.34	-0.035	-0.83	0.00394

RP11-356C4	-0.571	-0.403	-0.424	-1.047	-0.905	0.00394
AARS	0.244	0.091	0.061	0.481	0.228	0.00395
SNORA13	0.002	0.117	-0.104	-0.57	-0.884	0.00395
SLC25A42	-0.496	-0.115	0.005	-0.019	0.056	0.00397
FAM204A	-0.181	-0.139	-0.205	-0.335	-0.324	0.00397
H2AFZP	-0.082	-0.204	0.014	-0.083	-0.553	0.00397
SAC3D1	0.254	0.282	0.194	0.443	0.204	0.00398
MIR6753	-0.099	0.157	0.027	-0.683	-0.76	0.00398
AC004987.7	-0.306	0.034	0.453	0.008	0.384	0.004
RPL37P2	-0.388	-0.364	-0.129	-0.661	-0.643	0.00401
AC010132.1	0.193	-0.094	-0.303	0.021	-0.729	0.00401
RP4-706A16	-0.247	-0.111	0.007	-0.504	-0.357	0.00404
AC093416.3	0.057	-0.453	-0.276	-0.368	-0.852	0.00407
B9D1	-0.254	-0.071	-0.011	-0.06	-0.431	0.00408
HOXD8	-0.088	-0.044	0.059	0.04	-0.35	0.0041
SNORA3A	-0.602	0.124	0.006	-0.838	-0.356	0.0041
CTD-3222D1	-0.526	-0.131	0.054	-0.702	-0.527	0.00411
RP11-576C2	-0.915	-0.16	-0.106	-0.811	-0.653	0.00411
RP11-87H9.4	-0.55	0.111	0.288	-0.363	-0.269	0.00414
RPA1	0.364	0.137	-0.025	0.392	0.13	0.00416
ZNRF2	0.427	0.219	-0.069	-0.187	0.349	0.00418
NDUFS4	-0.175	-0.127	-0.1	-0.262	-0.428	0.00418
RP11-392E2	0.205	-0.176	0.16	-0.78	-0.53	0.00418
RP11-10B2.3	-0.579	-0.427	0.182	-0.756	-0.705	0.00419
ZNF614	-0.224	-0.156	-0.282	-0.358	-0.445	0.0042
C17orf62	-0.328	-0.156	-0.029	-0.139	-0.146	0.00421
CSRP2	0.066	0.421	0.137	0.558	-0.301	0.00422
AF001548.5	-0.206	-0.261	-0.04	0.48	0.679	0.00424
NDUFAF6	-0.02	-0.038	-0.181	-0.36	-0.409	0.00425
CTD-2066L2	0.069	-0.079	0.347	0.143	0.97	0.00428
XXbac-BPG2	-0.357	0.541	0.472	-0.008	0.025	0.0043
AC004014.4	0.188	-0.362	-0.289	-0.088	-0.053	0.00432
MEST	0.049	0.004	-0.169	-0.182	-0.543	0.00433
SNORA31	-0.464	0.08	-0.035	-0.699	-0.848	0.00433
CACNG7	0.151	0.02	0.129	0.253	-0.366	0.00434
EEF1A2	0.424	0.278	0.303	0.751	0.594	0.00435
CHIC2	0.217	0.238	0.276	0.16	0.377	0.00435
RP11-403P1	-0.375	-0.345	-0.174	-0.482	-0.396	0.00435

RP11-73M18	-0.33	-0.041	0.303	-0.509	-0.584	0.00437
RP11-178H8	-0.313	-0.337	-0.032	-0.378	-1.039	0.00438
RP11-818F2	-0.545	-0.293	0.206	-0.442	-0.224	0.0044
RP11-626A1	-0.694	-0.652	-0.458	-0.368	-1.043	0.0044
AC006483.5	0.353	-0.133	0.117	0.291	0.178	0.00442
PEF1	0.235	0.121	0.075	0.369	0.074	0.00442
PRKACA	0.357	0.036	-0.047	0.26	0.206	0.00443
PKD1P1	-0.182	0.011	0.023	-0.585	-0.151	0.00443
RP11-271B5	-0.225	-0.207	-0.063	-0.505	-0.48	0.00443
AC005546.2	-0.545	-0.409	-0.101	-0.998	-0.815	0.00444
RP11-646E1	0.457	-0.083	-0.091	0.139	0.17	0.00446
IGIP	-0.381	0.022	0.145	-0.362	-0.19	0.00447
SCARNA16	-0.594	0.119	0.307	-0.554	-0.499	0.00447
ALDOA	0.282	0.034	0.02	0.422	0.322	0.00448
PCOLCE	-0.4	-0.054	0.418	0.064	-0.258	0.00448
RP11-122G1	0.072	-0.317	-0.045	0.152	-0.88	0.00449
RARRES3	0.197	-0.125	0.083	-0.074	0.777	0.00453
MT-TY	-0.104	-0.335	0.377	-0.496	-0.723	0.00453
PPARGC1B	-0.375	-0.286	-0.007	-0.277	0.009	0.00455
TATDN3	-0.278	-0.095	-0.012	-0.159	-0.402	0.00455
DDT	-0.278	-0.067	0.001	-0.186	-0.473	0.00457
HLA-J	0.317	0.154	0.304	0.565	0.826	0.00463
NUDT13	-0.54	-0.203	0.179	-0.107	-0.218	0.00464
ASPHD1	-0.427	0.006	0.067	-0.253	-0.403	0.00465
PCGF7P	0.361	-0.053	-0.11	-0.055	-0.313	0.00467
IL11RA	-0.227	-0.135	-0.071	-0.253	-0.453	0.00467
BNIP3P	0.15	0.097	0.092	0.36	0.255	0.00468
RP5-906C1.	0.11	-0.21	-0.028	-0.427	-0.936	0.00469
RP3-339A18	-0.728	-0.197	-0.144	-0.923	-0.795	0.00471
ATP1A1OS	-0.907	-0.11	-0.271	0.168	-0.101	0.00473
TDRD9	-0.249	-0.215	0.228	-0.191	0.365	0.00474
RP11-110I1.	-0.554	0.006	0.081	0.362	-0.379	0.00475
UBQLN2	0.269	0.088	-0.087	0.267	0.357	0.00476
HSPBP1	0.274	0.065	-0.035	0.332	0.07	0.00476
RP11-332M2	0.348	0.013	-0.001	0.367	-0.021	0.00477
URB2	-0.154	-0.022	-0.228	-0.239	-0.349	0.00477
CTD-2382E5	-0.432	-0.185	-0.162	-0.973	-0.608	0.00477
PHF11	-0.029	0.21	0.394	0.192	0.326	0.00478

ADAT3	-0.173	0.331	0.032	-0.303	0.398	0.00479
EIF3E	-0.174	0.065	0.15	-0.086	0.327	0.00479
GAREM2	0.406	0.137	-0.057	0.067	0.401	0.0048
TKT	0.362	0.172	0.03	0.395	0.323	0.00481
ING2	0.55	0.307	0.19	0.436	0.161	0.00483
TPRN	-0.199	-0.02	-0.26	-0.306	-0.378	0.00484
RP11-401M1	0.232	0.154	0.13	0.407	0.403	0.00493
HNRNPU	0.335	0.164	0.077	0.384	0.34	0.00494
TRADD	-0.003	0.101	0.178	0.111	0.333	0.00495
RP11-452F1	-0.246	-0.001	-0.239	-0.51	-0.767	0.00496
ZG16B	-0.129	0.399	0.402	0.359	0.934	0.00497
FOXD2-AS1	-0.351	-0.289	-0.272	-0.684	-0.737	0.00499
RP11-1113N	0.363	0.038	-0.046	0.372	0.277	0.005
CARNMT1	0.192	0.119	0.236	0.361	0.155	0.005
RP11-135F9	0.743	0.428	0.189	0.333	0.076	0.00502
AKR1E2	-0.541	-0.264	-0.26	-0.493	-0.623	0.00503
ZNF91	0.023	0.197	0.052	0	0.436	0.00506
CTD-2373N4	-0.418	-0.04	-0.18	-0.901	-0.532	0.00508
LHPP	-0.077	-0.144	-0.151	0.071	0.438	0.00509
UTP3	0.429	0.242	0.179	0.491	0.187	0.00509
RAB38	0.192	0.353	0.531	1.017	0.479	0.0051
RP11-113K2	-0.497	-0.014	0.194	-0.077	-0.229	0.0051
RP11-389O2	-0.372	-0.353	-0.25	-0.439	-0.18	0.00511
RP11-1334A	-1.015	-0.509	-0.239	-0.263	-0.862	0.00512
GAB3	-0.11	-0.344	-0.033	-0.308	0.22	0.00514
RP11-351I24	-0.237	-0.137	-0.073	-0.577	-0.352	0.00514
IZUMO4	-0.285	0.265	0.647	-0.096	0.115	0.00516
RP3-427A4.2	-0.194	-0.151	-0.077	-0.186	-0.501	0.00516
RP11-392P7	-0.148	-0.078	-0.063	0.213	0.786	0.00519
RP11-234P3	0.128	0.097	-0.082	0.304	0.471	0.00521
ADIRF-AS1	-0.167	0.266	0.032	-0.557	-0.259	0.00523
GLUDP3	0.128	0.214	0.382	-0.237	-0.256	0.00524
TMEM188	-0.081	-0.048	-0.022	-0.33	-0.217	0.00534
ATP5S	-0.063	-0.081	-0.011	-0.163	-0.399	0.00534
GATSL3	-0.93	-0.343	-0.539	-0.427	-0.266	0.00535
RP11-397E7	-0.399	-0.398	-0.197	-0.362	-0.431	0.00539
SLC16A9	-0.083	-0.215	-0.316	-0.439	-0.132	0.00541
RP4-730K3.3	0.098	0.202	0.01	-0.523	-0.497	0.00542

CTD-2515O1	-0.384	-0.21	-0.247	-0.381	-0.621	0.00542
XXbac-BPG1	0.28	0.233	0.139	-0.316	-0.702	0.00543
ZBED3	0.402	0.143	0.07	0.312	0.441	0.00545
RP11-506M1	-0.28	-0.069	0.097	-0.622	-0.872	0.00545
SNRPEP2	-0.182	-0.112	0.005	-0.368	-0.421	0.00546
RP11-500M8	0.351	-0.009	-0.362	0.044	0.038	0.0055
ADIRF	-0.085	-0.207	-0.071	-0.201	-0.363	0.00551
PACSIN3	0.255	0.44	0.304	0.344	0.394	0.00553
RP11-230F1	-0.357	-0.262	-0.051	-0.695	-0.418	0.00556
STIP1	0.236	-0.023	-0.074	0.357	0.174	0.00557
PWWP2B	0.175	-0.005	-0.032	-0.059	0.383	0.00559
KATNBL1	-0.448	-0.036	-0.062	-0.42	-0.329	0.0056
RPS11P5	-0.316	-0.347	-0.165	-0.495	-0.305	0.00564
RP11-744D1	-0.233	-0.558	-0.645	-0.648	-0.694	0.00568
SF3B3	0.228	0.076	0.068	0.417	0.246	0.0057
RP11-251M1	-0.588	-0.555	-0.118	-0.236	-0.629	0.00571
RP11-69I8.3	-0.307	-0.128	-0.62	-0.744	-0.443	0.00573
CKS1B	-0.489	-0.263	-0.111	-0.264	-0.444	0.00575
GOLGA8B	-0.448	0.027	0.378	-0.464	-0.345	0.00577
TTC9C	-0.35	-0.267	-0.179	-0.166	-0.158	0.00583
LRRC49	0.192	-0.006	0.127	0.441	0.471	0.00584
FKBP1B	-0.106	-0.226	-0.223	-0.193	-0.345	0.00585
FUNDC2	-0.084	-0.121	-0.095	-0.159	-0.394	0.00585
GTF2H5	-0.215	-0.145	-0.056	-0.304	-0.493	0.00586
RPP25	-0.155	-0.38	-0.374	-0.236	-0.032	0.00587
GPN3	-0.056	-0.035	-0.009	-0.051	-0.346	0.00587
ARPC5	-0.156	-0.108	-0.149	-0.376	-0.38	0.00588
RP11-159J3.	0.337	-0.051	0.023	0.521	0.502	0.00589
MT-TW	-0.193	-0.154	0.3	-0.719	-0.443	0.00591
APOO	0.048	0.035	-0.073	-0.094	-0.361	0.00595
SNORA72	-0.257	0.257	0.13	-0.63	-0.61	0.00596
RP11-797A1	-0.375	0.01	0.095	-0.853	-0.525	0.00599
RUNDC3B	0.202	0.27	-0.099	-0.163	-0.357	0.006
HSP90AB2P	0.392	-0.186	-0.177	0.31	0.196	0.00603
NFIC	0.11	-0.085	0.039	0.251	0.324	0.00606
RP11-631M6	0.157	0.12	0.243	0.346	0.497	0.00608
HTATSF1P2	0.002	-0.009	0.322	0.514	-0.347	0.00609
CTD-3035D6	-0.46	-0.388	-0.272	-0.609	-0.47	0.00609

CMC1	-0.321	-0.217	-0.093	-0.349	-0.512	0.00612
CTD-2003C8	0.111	0.068	0.136	0.295	0.653	0.00615
POTEKP	0.296	-0.159	-0.372	-0.04	-0.321	0.00617
ZNF816	-0.563	-0.073	0.017	-0.126	-0.1	0.0062
RP11-305O4	0.005	-0.112	-0.25	-0.388	-0.947	0.0062
MIR7111	-0.661	-0.605	-0.608	-1.047	-1.018	0.00625
RP11-680A1	-0.211	-0.269	-0.147	-0.075	0.387	0.00626
RP11-37J13.	0.354	0.134	-0.161	0.211	0.377	0.00626
RP11-807H7	-0.449	-0.318	-0.336	-0.595	-0.987	0.00626
TYW1B	-0.355	-0.147	-0.378	-0.391	-0.761	0.00627
RP11-603J24	-0.5	-0.151	0.06	-0.573	-0.234	0.00628
TMSB15A	-0.4	-0.179	-0.164	-0.725	-0.613	0.00628
SFXN4	-0.104	-0.14	-0.086	-0.118	-0.434	0.00629
KCTD21	0.332	0.207	0.314	0.393	0.354	0.00636
TMEM240	-0.073	-0.207	-0.182	-0.325	0.471	0.00637
SMARCA4	0.247	0.04	0.029	0.37	0.286	0.00637
AC017080.1	-0.381	-0.254	-0.007	-0.564	-0.923	0.00637
RP1-182O16	-0.276	-0.347	-0.132	-0.65	-0.516	0.00644
EDA	0.59	0.196	0.335	0.478	0	0.00645
RP11-557J1(	0.47	0.018	0.086	0.504	0.454	0.00646
RP11-503E2	0.206	0.224	0.567	-0.544	-0.157	0.00646
SELT	-0.343	-0.172	-0.113	-0.54	-0.36	0.00649
NDST2	-0.391	0.193	-0.057	0.021	0.311	0.00656
AP006621.6	-0.635	0.126	0.196	-0.352	-0.184	0.00657
RP11-143K1	-0.273	-0.086	0.397	-0.748	-0.286	0.00657
RN7SL145P	-0.562	-0.007	0.142	-0.585	-0.716	0.00663
GPR157	0.326	-0.011	0.09	-0.011	-0.075	0.00666
FBXO48	-0.194	-0.009	-0.201	-0.523	-0.538	0.00673
CTD-2649C1	-0.459	-0.018	0.256	-0.567	-0.249	0.00676
RP11-1055B	-0.962	-0.378	-0.307	-0.968	-0.492	0.00676
ALKBH2	-0.412	-0.379	-0.327	-0.367	-0.661	0.00677
RP11-392O1	0.016	-0.179	-0.044	0.477	0.316	0.00679
VPS29	-0.277	-0.073	0.036	-0.23	-0.375	0.00679
RP5-1142A6	-0.225	-0.255	-0.825	-0.883	-0.62	0.0068
AC005932.1	-0.616	-0.453	-0.043	-0.328	0.076	0.00683
C11orf98	-0.083	0.057	0.04	-0.121	-0.488	0.00684
LRRC69	-0.713	0.135	0.207	-0.42	-0.303	0.00685
RAD51B	0.047	-0.194	-0.024	0.457	0.275	0.00687

NEURL2	-0.274	0.455	0.397	-0.228	0.268	0.00688
MPZ	-0.585	-0.139	-0.339	-0.483	0.264	0.00688
CAD	-0.132	-0.116	-0.074	-0.164	-0.357	0.00688
MTL5	-0.53	-0.157	0.082	-0.181	-0.318	0.00692
AC010733.5	-0.527	0.233	0.209	-0.636	-0.412	0.00692
U40455.1	-0.21	-0.515	-0.226	-0.118	-0.392	0.00694
AC141586.5	-0.212	-0.268	-0.268	-0.606	-0.459	0.00695
RP11-449M6	0.104	-0.057	-0.336	0.112	0.217	0.00696
CTD-2528L1	-0.109	-0.303	-0.218	-0.5	-0.819	0.00698
TRAPPC6A	0.019	-0.007	-0.102	0.121	0.489	0.00699
RP13-178D1	0.367	0.092	0.131	0.439	0.183	0.00703
RP11-497D6	-0.494	-0.432	-0.177	-0.52	-0.477	0.00704
CAPNS2	-0.172	-0.354	-0.361	-0.532	-1.082	0.00704
AC091729.9	-0.586	-0.202	-0.072	-0.405	-0.534	0.00711
CMB9-55F22	-0.496	-0.049	0.115	-0.431	-0.273	0.00714
TFIP11	0.263	0.238	0.117	0.443	0.226	0.00718
MIR5010	-0.248	0.421	0.551	-0.301	-0.296	0.00721
SNORA64	-0.243	-0.164	-0.049	-0.837	-0.813	0.00722
RP11-3D4.3	-0.062	0.266	0.261	-0.558	-0.249	0.00723
RP11-736L2.	0.449	0.218	0.142	0.591	0.38	0.00725
MED7	-0.419	-0.191	-0.171	-0.15	-0.427	0.00727
C14orf93	0.287	0.186	0.413	0.599	0.425	0.00732
KIF27	-0.585	0.178	0.054	-0.323	-0.1	0.00738
VAMP4	-0.365	-0.134	-0.089	-0.371	-0.128	0.00739
PMF1-BGLA	-0.385	0.194	-0.363	-0.126	0.59	0.00743
CIDEB	0.136	-0.199	-0.426	-0.454	-0.778	0.00745
KBTBD3	-0.651	-0.211	0.022	0.008	-0.178	0.00747
RP11-386G1	-0.696	-1.125	-0.556	-0.592	-0.882	0.00747
XKR6	-0.067	-0.173	0.034	-0.141	0.506	0.00753
RP11-454L9.	-0.117	0.54	0.273	-0.487	-0.328	0.00753
RN7SKP70	-0.578	0.212	0.239	-0.445	-0.548	0.00753
RP11-79P5.1	-0.155	-0.324	-0.203	-0.294	-0.589	0.00753
CEP19	0.013	0.031	0.067	0.316	0.69	0.00758
PIK3R4	0.235	0.361	0.113	0.29	0.287	0.00758
CFL1P4	0.451	0.06	-0.132	0.356	0.196	0.00758
CHCHD1	-0.264	-0.225	-0.08	-0.357	-0.441	0.00764
RP11-4C20.4	-0.518	-0.154	-0.143	-0.355	0.481	0.00766
AF129075.5	-0.272	-0.067	-0.107	-0.82	-0.793	0.00766

AC005332.1	0.014	-0.168	0.115	0.085	0.476	0.00769
MIR93	0.198	-0.121	-0.106	-0.679	-0.079	0.00769
RWDD3	-0.308	0.068	0.129	-0.364	-0.368	0.00769
RGPD5	0.719	-0.096	-0.257	0.082	0.17	0.00771
IMMTP	0.569	0.259	0.129	0.451	0.429	0.00774
HSPB6	0.28	0.213	-0.189	0.264	0.729	0.00776
RP11-142A2	0.182	0.09	0.028	0.339	0.285	0.00776
SF3B2	0.366	0.093	0.026	0.368	0.181	0.00776
CTD-2186M1	-0.017	-0.305	-0.713	-0.533	-0.772	0.00777
AC012262.1	-0.164	-0.358	-0.159	-0.08	0.485	0.00781
AC090952.4	-0.223	0.125	0.101	0.072	0.424	0.00781
ITM2C	0.26	0.221	0.211	0.476	0.498	0.00782
GGT7	0.061	0.142	0.412	-0.015	0.43	0.00788
CENPW	-0.331	-0.049	0.002	-0.078	-0.417	0.00791
ITGA4	0.225	0.024	-0.155	0.286	0.44	0.00792
RP11-535A1	-0.564	-0.201	-0.016	-0.317	0.323	0.00792
AC092662.3	0.194	-0.231	-0.163	0.383	0.13	0.00795
RP11-667M1	0.387	0.049	-0.033	0.209	0.102	0.00797
NET1	-0.34	-0.171	-0.134	-0.534	-0.298	0.00797
CCDC77	-0.193	-0.064	-0.378	-0.039	-0.082	0.00799
CGNL1	-0.326	-0.42	0.011	-0.286	-0.061	0.008
SLC2A10	0.321	0.208	0.227	0.474	0.493	0.00801
DHX9	0.46	0.135	-0.01	0.348	0.329	0.00801
RP11-44M6.1	-0.473	-0.201	-0.17	-0.678	-0.454	0.00805
RP11-402K9	0.189	-0.234	-0.433	-0.114	-0.588	0.00811
RP11-382A1	-0.295	-0.577	0.102	0.159	0.287	0.00812
VMAC	-0.293	0.144	0.412	-0.192	0.188	0.00812
RP11-667K1	0.31	0.106	0.128	0.148	-0.585	0.00813
MATR3	0.378	-0.062	-0.147	0.228	0.058	0.00814
RP11-34P13	-0.176	0.055	0.054	-0.609	-0.485	0.00815
RP3-508I15.	-0.185	0.073	-0.036	-0.571	-0.879	0.00818
USMG5	-0.405	-0.159	-0.028	-0.655	-0.512	0.00821
RP11-1112J2	0.583	0.063	-0.08	0.371	0.241	0.00822
PCBP2-OT1	-0.6	-0.142	-0.246	-0.986	-0.689	0.00822
AL132709.1	-0.237	0.096	-0.071	-0.788	-0.71	0.00822
NCRNA0018	-0.714	-0.244	0.071	-0.477	-0.246	0.00824
CTA-204B4.2	-0.499	-0.094	0.04	-0.701	-0.473	0.00824
YDJC	-0.149	-0.29	-0.338	-0.289	-0.048	0.00826

SNRPG	-0.211	0.108	0.085	-0.434	-0.575	0.00828
RP11-108L7.	-0.569	-0.497	-0.382	-0.507	-1.131	0.00831
RP11-447D1	-0.49	-0.183	-0.097	-0.129	0.348	0.0084
TMEM126B	-0.339	-0.19	-0.057	-0.54	-0.429	0.0084
AAMP	0.186	0.08	0.02	0.332	0.158	0.00843
FAM101A	0.476	0.496	0.093	0.438	0.33	0.00848
ECSIT	0.169	0.06	0.114	0.396	0.189	0.00851
SNORD26	-0.222	0.011	-0.093	-0.733	-0.84	0.00855
RP11-365D2	-0.052	-0.328	-0.144	-0.313	-0.393	0.00857
RP5-1017O1	0.324	-0.106	-0.181	0.105	0.011	0.00858
LYPLA2P2	0.286	0.003	0.195	0.325	0.53	0.00859
CTB-31O20.:	-0.304	-0.009	-0.307	-0.702	-0.498	0.00861
HNRPCP	-0.269	-0.015	0.102	-0.325	-0.645	0.00863
RP1-167A14	-0.406	-0.44	0.325	-0.469	-0.714	0.00864
STAG3L3	-0.655	-0.318	0.268	-0.153	-0.476	0.00868
RF00015	-0.253	0.018	0.055	-0.679	-0.704	0.00874
GTF2F1	0.324	0.162	0.052	0.417	0.217	0.00878
FAH	0.197	0.124	0.107	0.352	0.261	0.00884
RF00012	-0.573	-0.151	-0.092	-0.687	-0.928	0.00885
ZNF549	-0.187	0.115	0.042	-0.432	-0.105	0.00898
TMEM86B	-0.251	0.044	0.346	-0.42	-0.406	0.00898
COMMD1	-0.29	-0.11	-0.034	-0.27	-0.362	0.00902
CSNK1A1L	0.368	-0.07	-0.023	0.133	0.166	0.00903
ZNF132	-0.455	0.113	-0.132	-0.264	0.061	0.00905
LYRM5	-0.281	0.077	0.103	-0.055	-0.365	0.00906
SNORD10	-0.466	-0.256	-0.266	-0.91	-0.572	0.00907
IER5L	0.309	0.183	0.23	0.327	0.506	0.00908
CTD-2538G9	0.074	0.595	0.148	-0.558	-0.051	0.00908
ZNF708	-0.133	0.053	0.232	-0.426	0.175	0.00914
MSMP	0.138	-0.177	-0.45	-0.153	-0.006	0.00914
RP11-199A1	0.239	-0.094	-0.132	0.425	0.013	0.00915
CST1	0.473	0.138	-0.218	0.13	-0.034	0.00917
COA6	-0.289	-0.213	-0.038	-0.238	-0.502	0.0092
RP11-329L6.	-0.419	0.053	-0.121	-0.485	-0.751	0.00921
RP11-613C6	-0.244	-0.235	-0.516	-0.309	-0.315	0.00927
RPS18P12	-0.397	-0.354	-0.134	-0.535	-0.413	0.0093
UQCR11	-0.315	-0.218	-0.089	-0.352	-0.269	0.00937
MIR553	-0.32	0.59	0.199	0.024	-0.374	0.00937

LURAP1	-0.152	0.046	0.26	0.132	-0.432	0.00937
RP1-193M11	0.263	-0.01	-0.073	0.378	0.198	0.00939
IGLV1-51	-0.329	0.495	0.352	-0.451	-0.147	0.0094
C11orf54	0.076	0.157	0.26	0.416	0.232	0.00941
RP11-463H1	-0.229	-0.222	-0.147	-0.376	-0.451	0.00942
TMEM102	-0.245	-0.046	0.012	0.394	0.105	0.00945
RP11-629O4	0.134	-0.052	-0.085	-0.222	-0.879	0.00945
RP4-734P14	-0.196	-0.043	-0.248	-0.975	-0.436	0.0095
RP11-314A2	-0.681	-0.669	-0.235	-0.841	-0.678	0.0095
TXNDC17	-0.356	-0.275	-0.099	-0.544	-0.324	0.00959
RP11-697N1	0.072	-0.248	-0.205	-0.268	-0.37	0.0096
RP11-183E9	-0.097	-0.701	-0.39	-0.363	-0.94	0.00963
SPATA2	0.347	0.188	0.013	0.206	0.234	0.00965
CTD-3065B2	-0.313	-0.495	-0.327	-0.521	-0.504	0.00967
UBE2W	-0.285	-0.001	-0.024	-0.377	-0.022	0.00969
MRPL42	-0.262	-0.121	-0.173	-0.372	-0.367	0.00971
SNORD19B	-0.496	-0.043	0.083	-0.674	-0.696	0.00971
RP11-319J2	0.06	-0.118	-0.144	-0.196	0.564	0.00974
VDAC1LP	0.363	0.032	-0.119	0.182	0.053	0.00974
RP11-305M3	0.004	0.092	0.338	0.261	0.024	0.00976
RP11-65L3.2	-0.856	0.018	-0.079	-0.475	-0.109	0.00977
ZNF879	-0.475	0.031	-0.074	-0.323	-0.411	0.00977
ZNF782	-0.228	0.134	-0.001	-0.261	-0.363	0.00978
CORO1A	0.092	-0.196	-0.363	0.002	-0.435	0.00986
GSPT2	0.151	-0.032	-0.092	0.331	0.273	0.00992
RP3-323K23	-0.271	-0.453	-0.425	-0.328	-1.082	0.00994
AJ271735.3	-0.18	-0.497	-0.469	-0.536	-0.58	0.00999
RP11-335F8	0.372	-0.031	-0.132	0.324	0.075	0.01
AC055811.5	0.24	-0.35	-0.395	-0.06	0.007	0.01
CCNO	0.041	0.347	-0.085	-0.242	-0.626	0.01
MT-TI	-0.151	-0.15	0.254	-0.57	-0.741	0.01
RP11-848P1	0.294	0.315	0.397	0.175	1.08	0.0101
WDR92	-0.047	0.067	0.209	0.349	0.413	0.0101
KHK	-0.298	-0.354	-0.558	-0.071	0.187	0.0101
ZNF404	-0.534	0.15	0.395	-0.057	-0.211	0.0101
RP11-234A1	-0.28	-0.207	-0.007	-0.427	-0.377	0.0101
RP11-53O19	-0.24	-0.057	-0.031	-0.786	-0.389	0.0101
AC108463.2	0.361	-0.158	0.009	0.189	0.879	0.0102

PA2G4P1	0.576	0.15	0.112	0.422	-0.019	0.0102
RP5-1059L7.	-0.198	-0.409	-0.072	0.036	0.52	0.0103
ALDOAP1	0.197	-0.33	-0.426	-0.036	0.238	0.0103
UBE2Q2P1	-0.155	0.033	0.381	-0.435	0.16	0.0103
ELAC1	-0.247	0.042	-0.069	-0.134	-0.418	0.0103
RP11-1020A	-0.551	0.267	0.272	-0.408	-0.455	0.0103
AL353997.7	0.082	-0.111	-0.109	0.01	-0.387	0.0104
RPL35	-0.196	-0.112	-0.088	-0.334	-0.399	0.0104
WASH2P	-0.335	-0.166	0.008	-0.681	-0.492	0.0104
RP11-57H14	-0.066	-0.092	0.191	-0.03	0.418	0.0105
RHEBL1	-0.43	-0.19	0.225	-0.038	-0.179	0.0105
TBX19	-0.246	0.236	0.175	-0.449	-0.444	0.0105
DPH6	-0.057	-0.116	-0.013	0.045	-0.458	0.0105
SF3A3P1	0.364	0.148	0.12	0.465	0.331	0.0106
HOMEZ	-0.16	0.157	0.024	0.125	0.329	0.0106
RP11-835E1	-0.042	0.149	0.324	0.087	-0.067	0.0106
RP3-461F17	-0.547	-0.164	0.343	-0.539	-0.463	0.0106
RP11-618G2	-0.447	-0.286	-0.096	-0.22	-0.698	0.0106
CTB-54O9.9	-0.153	-0.297	-0.526	-0.943	-0.806	0.0106
CTB-129O4.	-0.476	-0.425	-0.425	-0.927	-0.975	0.0106
RP11-422J8.	-0.235	0.008	0.008	0.495	0.387	0.0107
EBPL	-0.224	-0.249	-0.278	-0.467	-0.141	0.0107
XXbac-B562I	-0.185	-0.066	0.053	0.171	-0.402	0.0107
RP11-39C10	0.131	-0.304	-0.283	-0.122	-0.402	0.0107
ARF4-AS1	-0.618	0.191	0.061	-0.401	-0.668	0.0107
RP11-434N1	0.259	0.093	0.144	0.175	0.485	0.0108
IGSF8	-0.038	0.029	-0.193	-0.017	-0.384	0.0108
CNN2P9	0.398	0.006	-0.051	0.19	0.427	0.0109
NFKBIL1	0.031	0.218	0.182	0.276	0.398	0.0109
ASB13	0.017	-0.2	-0.446	-0.146	0.002	0.0109
AAR2	0.329	0.127	-0.078	0.186	-0.01	0.0109
RP1-265C24	-0.405	-0.114	-0.046	-0.446	-0.624	0.0109
MRPL34	-0.226	-0.256	-0.09	-0.167	-0.324	0.011
RPS26	-0.198	-0.062	-0.085	-0.381	-0.432	0.011
NDUFB3	-0.312	-0.155	-0.048	-0.427	-0.492	0.011
RN7SL431P	0.181	-0.112	0.094	-0.655	-0.537	0.011
CNN2P4	0.56	0.002	-0.02	0.136	0.447	0.0111
RP4-652L8.2	0.492	0.115	0.049	0.028	-0.048	0.0111

RP11-352E6	-0.177	-0.331	0.02	-0.756	-0.818	0.0111
TMEM156	0.173	-0.098	-0.223	-0.013	0.411	0.0112
GOLGA8N	-0.191	0.211	0.482	-0.072	0.126	0.0112
LRRC75A-AS	-0.317	-0.19	-0.076	-0.484	-0.256	0.0112
RPS7P1	-0.223	-0.086	-0.065	-0.452	-0.394	0.0112
CENPS	-0.587	-0.427	0.005	-0.163	-0.47	0.0112
RP11-97C16	-0.369	0.057	0.149	-0.493	-0.502	0.0112
RPS10	-0.245	-0.184	-0.072	-0.405	-0.53	0.0112
AC090421.1	-0.443	-0.237	-0.28	-0.499	-0.622	0.0113
RP11-148B6	-0.298	-0.284	-0.33	-0.48	-0.668	0.0113
ZNF680	-0.22	0.076	-0.265	-0.512	-0.004	0.0114
ZFAND1	-0.333	-0.19	-0.068	-0.474	-0.366	0.0115
RNASEH1P1	0.243	-0.293	0.008	0.103	-0.474	0.0115
RP11-848P1	-0.379	-0.089	-0.601	-0.675	-0.95	0.0115
NBPF10	-0.178	0.128	-0.157	-0.46	0.144	0.0116
RP11-1A15.2	0.345	-0.143	-0.106	0.188	-0.164	0.0116
CD46P	-0.427	-0.818	-0.493	-0.322	-0.174	0.0116
MIR5581	-0.903	-0.41	-0.217	-0.912	-0.576	0.0116
NSMCE4A	-0.311	-0.132	-0.076	-0.275	-0.337	0.0117
AL121652.2	-0.097	-0.43	-0.323	-0.251	-0.612	0.0117
RP11-321E8	0.337	-0.003	-0.007	0.319	0.169	0.0118
RP11-324I22	-0.576	-0.243	0.089	-0.104	0.105	0.0118
MED11	-0.243	-0.181	-0.026	-0.144	-0.439	0.0118
RP11-3J10.5	-0.042	-0.261	-0.845	-0.176	-0.614	0.0118
RP11-420J1	0.158	-0.023	0.296	0.451	0.557	0.0119
PARP1	0.402	0.106	-0.061	0.306	0.051	0.0119
REP15	-0.394	0.078	0.285	0.163	0.346	0.012
ZSCAN26	-0.382	-0.045	0.05	-0.114	-0.082	0.012
SMDT1	-0.165	-0.185	-0.013	-0.246	-0.37	0.012
UFSP1	-0.927	-0.373	-0.474	-0.615	-0.524	0.012
CTD-2371O3	-0.621	-0.383	-0.139	-0.742	-0.61	0.012
AC002407.1	-0.366	-0.561	-0.325	-0.476	-0.774	0.012
SKIV2L2	0.346	0.183	0.079	0.386	0.238	0.0121
RP1-222H5.1	-0.24	-0.353	-0.157	0.047	-0.223	0.0121
RP11-823P9	-0.038	0.087	-0.024	-0.484	-0.372	0.0121
EFNB3	-0.034	0.321	0.059	0.175	0.563	0.0122
ITGB4	0.343	-0.15	0.028	0.439	0.274	0.0122
SNORD15B	-0.503	0.017	-0.221	-0.677	-0.867	0.0123

SERTAD4	0.404	0.036	0.186	0.299	0.591	0.0124
HLA-H	0.351	0.295	0.113	0.34	0.621	0.0125
MIR186	-0.058	0.171	0.263	-0.653	-0.221	0.0125
CTC-459F4.3	-0.416	-0.064	-0.045	-0.279	-0.309	0.0125
RP11-185N2	-0.152	0.16	0.278	-0.138	-0.412	0.0125
RP11-408A1	-0.061	0.123	0.117	-0.581	-0.561	0.0125
BLOC1S1	-0.266	-0.19	-0.101	-0.397	-0.374	0.0126
CHRNA10	-0.22	-0.118	-0.072	-0.834	-0.574	0.0126
C21orf33	0.087	-0.197	-0.074	0.515	0.464	0.0127
HS6ST1	0.301	0.202	0.091	0.34	-0.054	0.0127
C17orf58	-0.482	-0.281	0.018	-0.199	-0.237	0.0127
RP4-739H11	-0.293	-0.086	0.304	-0.598	-0.357	0.0127
ZNF107	0.013	0.022	-0.18	-0.451	-0.063	0.0128
CATSPER2	-0.478	-0.016	0.21	-0.495	-0.587	0.0128
RP11-48B3.2	-0.042	-0.131	-0.378	-0.264	0.582	0.0129
HIN1L	-0.013	0.346	0.109	0.061	0.339	0.0129
ZNF212	0.382	0.064	0.102	0.142	0.033	0.0129
ENO3	-0.203	0.073	0.465	-0.184	-0.073	0.0129
RP11-467I20	-0.333	-0.543	-0.169	-0.453	-0.604	0.0129
RP11-500G2	-0.52	-0.237	0.013	0.06	0.49	0.013
RP11-328C8	0.172	0.016	0.06	0.323	0.469	0.013
KRTCAP3	-0.037	-0.13	0.128	-0.833	-0.179	0.013
GS1-324M7.[illegible]	-0.174	-0.078	-0.09	-0.374	-0.697	0.013
RP11-341G2	-0.579	-0.491	-0.288	-0.914	-0.946	0.013
BAG6	0.184	0.17	0.161	0.397	0.258	0.0131
SUB1	-0.071	-0.054	-0.108	-0.382	-0.294	0.0131
LINC00632	-0.094	-0.008	-0.206	-0.418	-0.858	0.0132
MYL4	-0.003	0.115	0.55	0.027	0.825	0.0133
ZBTB12	-0.597	-0.649	-0.079	-0.156	-0.171	0.0133
RP11-425M5	-0.427	-0.158	-0.071	-0.872	-0.481	0.0133
AC073310.4	-0.153	-0.658	-0.465	-0.013	0.011	0.0134
RP11-261A1	0.417	-0.007	0.095	0.348	0.361	0.0135
CTNNBL1	0.286	0.065	0.033	0.382	0.225	0.0135
OLMALINC	-0.475	-0.036	-0.006	-0.414	-0.205	0.0135
RP11-169D4	-0.067	-0.128	0.123	0.407	0.393	0.0136
GPER1	0.386	-0.181	-0.137	-0.125	0.454	0.0137
AC007272.2	-0.012	0.287	-0.086	0.455	0.309	0.0138
RP13-77O11	-0.275	-0.343	-0.168	-0.313	-0.324	0.0138

RP1-12G14.3	-0.447	-0.447	-0.318	-0.53	-0.515	0.0138
RP11-182B2	-0.365	-0.582	-0.618	-0.6	-0.64	0.0139
KYNU	0.173	0.26	0.391	0.533	0.114	0.014
RP11-384L8.	0.388	-0.171	-0.16	-0.327	-0.547	0.0141
CNIH4	-0.418	-0.289	-0.11	-0.355	-0.223	0.0142
ZNF534	-0.158	-0.124	-0.229	-0.223	-0.68	0.0142
INAFM1	0.214	0.066	-0.123	-0.403	-0.08	0.0143
NSL1	-0.266	-0.122	0.019	-0.073	-0.336	0.0143
MRPL54	-0.201	-0.228	-0.142	-0.286	-0.441	0.0143
RP11-255N2	0.338	0.155	0.109	0.51	0.027	0.0144
RP11-166O4	-0.356	-0.215	-0.089	-0.396	-0.301	0.0144
RF00275	-0.421	0.103	0.285	-0.306	-0.569	0.0144
TLCD2	0.06	0.092	0.48	0.224	0.488	0.0145
RP11-318C2	-0.412	-0.455	-0.405	-0.867	-0.733	0.0145
AC023468.2	0.418	0.108	0.211	0.459	0.565	0.0146
ZNF140	0.134	0.338	0.194	-0.147	0.24	0.0146
RP11-297D2	-0.912	-0.252	-0.172	-0.456	-0.184	0.0146
DISP1	-0.149	-0.039	-0.046	-0.09	-0.512	0.0146
LINC-PINT	-0.576	0.037	0.186	-0.502	-0.187	0.0147
AK4P1	0.289	0.04	-0.113	0.183	0.442	0.0148
AC093724.1	0.422	0.293	0.371	0.232	0.408	0.0148
SLC25A45	-0.37	0.083	0.484	0.051	0.213	0.0148
KANSL1L	0.202	0.423	0.239	-0.162	0.168	0.0148
CTD-2537I9.	-0.335	0.18	0.59	-0.382	0.121	0.0148
SSR4P1	-0.134	0.273	0.465	0.242	-0.111	0.0148
MBOAT7	-0.013	-0.153	-0.341	-0.152	-0.161	0.0148
RP11-146F1	-0.243	-0.103	-0.25	-0.727	-0.86	0.0148
RP11-48B24	-0.268	-0.146	0.097	-0.343	-0.918	0.0148
VWFP1	0.382	0.111	-0.214	0.186	0.424	0.0149
REC8	-0.014	-0.183	0.196	-0.461	0.131	0.0149
PCBD2	-0.342	-0.211	0.027	-0.32	-0.255	0.0149
TMEM251	-0.085	-0.163	-0.024	-0.014	-0.431	0.0149
ZNF230	0.15	0.022	-0.097	-0.262	-0.461	0.0149
MIR4442	0.031	-0.044	-0.044	0.451	0.404	0.015
HAGHL	-0.321	-0.032	-0.146	-0.599	0.214	0.015
RP11-345K2	-0.399	-0.432	-0.164	-0.86	-0.347	0.015
RP11-1E11.1	-0.367	-0.495	-0.467	-0.023	0.382	0.0151
TAF15	0.343	0.115	0.142	0.251	0.211	0.0151

MT1P3	0.099	-0.455	0.25	0.226	-0.204	0.0151
RP11-390K5	-0.37	-0.127	0.14	-0.409	-0.403	0.0151
GPR89A	-0.097	-0.159	-0.271	-0.318	-0.507	0.0151
G6PD	0.282	0.123	0.083	0.508	0.247	0.0152
TFF3	-0.224	-0.531	-0.547	-0.376	0.183	0.0152
RP11-90E9.1	0.055	-0.27	0.231	-0.617	-0.226	0.0152
FGD1	-0.157	-0.226	-0.369	-0.247	-0.26	0.0152
NDUFB2	-0.273	-0.209	-0.149	-0.327	-0.345	0.0153
LINC00294	0.226	0.241	0.33	0.219	0.399	0.0154
TRMT61B	-0.351	-0.115	-0.036	-0.185	-0.188	0.0155
RP1-281H8.3	0.02	-0.058	-0.174	-0.429	-0.607	0.0155
AC004049.3	0.247	0.026	0.002	0.431	0.263	0.0156
FAM92A1	-0.15	-0.152	-0.161	-0.452	-0.172	0.0156
AC009303.2	0.51	0.203	0.026	0.165	-0.409	0.0156
FIRRE	-0.417	-0.098	-0.083	-0.661	-0.471	0.0156
RP11-783K1	-0.448	-0.127	0.058	-0.095	0.226	0.0157
MIR4512	-0.409	-0.458	-0.067	-0.337	-0.756	0.0157
RP4-682C21	0.378	0.078	0.025	0.292	0.244	0.0158
RPS16	-0.215	-0.157	-0.066	-0.34	-0.311	0.0158
RP3-469D22	-0.106	-0.143	-0.139	-0.157	-0.444	0.0159
RP11-151N1	-0.422	0.024	0.197	-0.729	-0.354	0.016
MAGED1	0.409	0.157	-0.019	0.329	0.201	0.0161
ZNF771	-0.426	-0.237	-0.184	-0.309	0.166	0.0161
RP11-597G2	0.04	-0.176	-0.33	-0.002	-0.895	0.0161
SNX6P1	-0.172	-0.342	-0.143	-0.114	0.008	0.0162
RP11-528A1	0.1	-0.437	-0.328	0.081	-0.497	0.0162
RP11-260M2	-0.372	-0.189	0.356	-0.544	-0.508	0.0162
CALCOCO2	0.203	0.143	0.126	0.342	-0.002	0.0163
CTD-3131K8	-0.288	-0.082	0.165	-0.598	0.15	0.0164
TMSB4X	-0.283	-0.214	-0.066	-0.388	0.085	0.0164
RP13-128O4	-0.272	-0.093	-0.482	-0.553	-0.884	0.0164
CTA-293F17	-0.474	0.119	0.337	0.261	0.523	0.0165
AF131215.9	-0.21	0.002	0.386	-0.16	0.271	0.0165
RP11-14K2.1	-0.291	-0.233	-0.125	-0.419	-0.327	0.0165
SNORA2A	-0.189	-0.441	-0.01	-0.63	-0.852	0.0165
SNORD11	-0.127	-0.279	-0.375	-0.69	-0.865	0.0165
SEC61A2	-0.203	-0.054	-0.11	-0.517	-0.35	0.0166
ROMO1	-0.437	-0.296	-0.109	-0.427	-0.385	0.0166

SLC39A11	0.149	-0.123	-0.351	0.146	0.111	0.0167
LBH	0.853	0.35	0.043	0.225	0.525	0.0168
RP11-118N2	0.346	-0.063	0.034	0.235	0.239	0.017
CTA-217C2.2	-0.512	-0.076	0.224	-0.663	-0.543	0.017
TBC1D8B	0.413	0.313	0.133	0.323	0.171	0.0172
SF3A3P2	0.318	-0.002	0.09	0.421	0.12	0.0172
MIR3682	0.361	0.03	0.187	-0.68	-0.092	0.0172
TIGD5	-0.26	-0.297	-0.226	-0.367	-0.049	0.0174
RP11-443G1	-0.329	-0.306	-0.128	-0.402	-0.457	0.0174
RP11-201K1	-0.318	-0.147	0.036	-0.425	-0.592	0.0174
CYP2U1	0.338	0.19	0.09	0.179	0.204	0.0175
TSPYL3	0.105	-0.174	-0.242	-0.176	0.386	0.0176
AP2A1	0.255	0.132	0.044	0.364	0.198	0.0176
HIST1H4H	0.05	0.715	0.708	0.625	0.632	0.0177
RP11-333E1	0.304	-0.038	0.007	0.287	0.329	0.0177
ARID1A	0.335	0.093	0.018	0.087	0.087	0.0177
ZDHHC11	-0.573	0.011	0.217	-0.274	-0.53	0.0177
RP11-385M4	0.601	-0.131	-0.151	0.252	0.583	0.0178
APOLD1	-0.643	-0.147	-0.197	-0.166	-0.004	0.0178
RP11-460N2	-0.712	-0.186	-0.305	-0.856	-0.7	0.0178
TBC1D3F	-0.576	-0.45	0.015	-0.644	0.023	0.0179
RP1-30G7.2	-0.627	-0.255	0.269	-0.32	-0.208	0.0179
RP4-669L17.	-0.172	-0.056	0.272	-0.718	-0.259	0.0179
RP11-535M1	0.284	-0.146	0.058	0.484	0.751	0.0182
KCNJ2-AS1	0.122	0.532	0.713	0.552	0.281	0.0182
SNORD62B	-0.205	0.023	0.189	-0.798	-0.265	0.0182
RP3-475B7.3	0.497	0.065	0.08	0.3	0.308	0.0183
RP11-81N13	-0.238	-0.143	-0.014	0.129	0.467	0.0184
ZNF594	-0.112	0.363	0.487	0.178	0.339	0.0184
MTX1	0.397	0.39	0.049	0.405	0.109	0.0184
RP3-497J21.	-0.233	-0.419	-0.331	-0.099	-0.129	0.0184
AC093724.2	0.13	-0.018	-0.351	-0.082	-0.327	0.0184
CTD-2286N8	0.452	0.231	-0.107	-0.269	-0.363	0.0184
SNRPGP2	-0.3	-0.096	-0.092	-0.544	-0.688	0.0184
TGFB3	-0.401	0.054	0.126	-0.403	0.194	0.0185
AC011742.3	-0.421	-0.732	-0.418	-0.388	-1.022	0.0185
RP3-499B10	-0.288	0.282	0.202	-0.425	-0.604	0.0186
RP11-175B9	-0.194	-0.321	-0.157	-0.473	-0.344	0.0187

CRP	-0.527	-0.526	-0.481	-0.341	-1.002	0.0187
SNRNP200	0.259	0.13	0.138	0.398	0.34	0.0188
RP11-390B4	0.065	0.071	-0.243	-0.02	-0.701	0.0188
ZNF570	0.305	0.458	0.184	0.061	0.416	0.0189
PDCD2L	-0.215	-0.255	-0.248	-0.01	-0.362	0.0189
ATP5E	-0.403	-0.176	-0.07	-0.595	-0.48	0.0189
CYB5A	-0.169	0.053	0.121	-0.083	-0.497	0.0189
AC006273.4	-0.236	0.041	0.175	0.013	-0.61	0.0189
LINC00997	0.151	-0.116	-0.011	-0.036	0.597	0.019
RP11-668N2	0.119	-0.155	0.054	-0.012	0.412	0.019
ZNF671	-0.102	0.331	0.151	-0.119	-0.065	0.019
NCRNA0011	-0.185	0.282	0.241	-0.614	-0.155	0.019
CTD-2301A4	0.058	-0.34	-0.337	-0.143	-0.207	0.019
RP11-1023L	-0.258	-0.092	0.26	-0.414	-0.47	0.019
RPS27	-0.091	-0.161	0.047	-0.343	-0.451	0.0191
ZNF34	-0.084	0.174	-0.181	-0.307	-0.456	0.0191
USMG5P1	-0.439	-0.258	-0.076	-0.696	-0.561	0.0191
AC018641.6	0.179	-0.123	0.05	0.373	0.207	0.0192
IFI27L1	-0.388	-0.112	-0.085	-0.3	-0.314	0.0192
RP3-467L1.6	-0.36	-0.085	-0.132	-0.804	-0.572	0.0192
AC017104.3	-0.206	-0.221	-0.179	-0.234	-0.598	0.0192
SH3GL1P1	0.257	-0.003	0.248	-0.376	-0.034	0.0193
RP11-81P15	0.185	-0.316	-0.351	-0.142	-0.338	0.0193
RP11-752G1	0.148	-0.338	-0.38	-0.284	0.024	0.0194
SNORD53B	-0.054	0.216	0.229	-0.476	-0.375	0.0194
RP11-587D2	-0.323	-0.218	-0.049	-0.618	-0.395	0.0194
TCEB1P2	-0.19	0.119	-0.039	-0.16	-0.522	0.0195
PTGR2	-0.212	-0.025	0.339	0.091	0.31	0.0196
CTB-30L5.1	-0.338	0.437	0.538	0.457	-0.046	0.0196
RP11-760D2	-0.094	-0.434	-0.332	-0.267	-0.161	0.0196
CTD-2184D3	-0.308	-0.125	0.105	-0.485	-0.464	0.0196
YAE1D1	-0.38	-0.233	-0.149	-0.365	-0.038	0.0197
TIGD7	-0.257	0.067	0.102	-0.001	-0.342	0.0197
RP5-857K21	-0.438	-0.22	-0.201	-1.014	-0.453	0.0197
ATP5EP2	-0.448	-0.274	-0.141	-0.594	-0.527	0.0197
RP11-388M2	0.127	-0.111	0.076	0.07	0.617	0.0198
CTD-2265D6	0.223	-0.166	0.013	0.401	0.099	0.0198
OSBP	0.327	0.201	0.135	0.353	0.269	0.0199

AC106862.1	0.547	0.542	0.018	-0.147	-0.051	0.0199
CMB9-94B1.	0.03	-0.136	0.169	-0.766	-0.402	0.02
DDX18P1	0.329	0.322	0.189	0.609	-0.04	0.0201
RP11-1017G	-0.628	0.126	-0.133	-0.294	-0.581	0.0202
SLX1A-SULT	-0.658	-0.304	0.05	-0.553	-0.608	0.0203
AC007277.2	0.312	0.119	-0.039	0.54	0.186	0.0205
SNRPD2	-0.238	-0.186	-0.083	-0.362	-0.386	0.0205
RP11-767N6	-0.479	-0.008	-0.001	-0.828	-0.448	0.0205
NDUFA4	-0.358	-0.114	-0.043	-0.564	-0.498	0.0205
C19orf79	-0.277	-0.186	-0.125	-0.473	-0.479	0.0206
MIR1254-1	-0.312	0.074	0.198	-0.491	-0.598	0.0209
RPS11	-0.262	-0.146	-0.035	-0.395	-0.215	0.021
UBL5	-0.278	-0.216	-0.121	-0.429	-0.356	0.021
CCDC121	-0.703	-0.169	-0.081	-0.223	-0.481	0.021
CTR9	0.259	0.235	0.163	0.433	0.284	0.0211
RP4-604A21	-0.258	-0.163	-0.17	-0.621	-0.472	0.0211
AF011889.4	-0.536	0.016	0.158	-0.584	0.093	0.0212
MFSD14C	0.108	0.186	0.234	-0.349	-0.024	0.0213
SEMA4F	0.016	0.219	0.347	0.305	0.358	0.0214
RP11-388G3	0.466	0.179	-0.087	-0.163	0.226	0.0214
NGEF	0.43	0.135	0.059	0.155	-0.332	0.0214
RP4-798C17	0.53	0.262	0.233	0.128	-0.466	0.0214
AC011330.5	-0.35	-0.454	-0.127	-0.346	-0.592	0.0214
RP1-167A19	0.308	-0.099	0.227	0.487	0.098	0.0215
CH17-260O1	0.022	-0.421	-0.067	-0.646	-0.29	0.0215
TOMM6	-0.007	-0.134	-0.044	-0.203	-0.331	0.0216
CBWD3	0.098	0.063	0.735	-0.229	-0.182	0.0217
RPL31	-0.226	-0.165	-0.031	-0.422	-0.268	0.0217
AC002066.1	-0.031	-0.094	0.4	0.284	0.825	0.0218
GART	0.256	0.078	0.076	0.334	0.224	0.0218
RP11-864N7	-0.418	-0.275	-0.106	-0.602	-0.499	0.0218
UNC45A	0.151	0.089	0.026	0.347	0.2	0.0219
ZBTB26	-0.296	-0.127	-0.069	-0.278	-0.421	0.0219
RP13-516M1	-0.464	-0.012	0.171	-0.059	-0.438	0.0219
RP11-12I24.:	0.363	0.063	-0.22	0.342	0.238	0.0221
RP4-614C10	-0.383	-0.132	0.063	-0.056	-0.055	0.0221
RP11-46C24	-0.102	0.419	0.365	0.148	-0.115	0.0221
RP13-122B2	-0.187	-0.095	0.361	-0.197	-0.319	0.0221

SNORD23	0.051	0.101	-0.258	-0.699	-0.586	0.0221
ACTG1P9	0.178	-0.183	-0.545	-0.108	-0.198	0.0223
RP13-942N8	-0.728	-0.145	0.108	-0.308	-0.447	0.0223
RP11-734K2	-0.34	-0.524	-0.19	-0.603	-0.483	0.0223
AC064836.3	-0.274	-0.859	-0.597	-0.019	-0.572	0.0223
ITPRIPL2	0.084	0.029	0.159	0.03	0.324	0.0224
RP11-315O1	0.497	-0.404	0.205	0.128	-0.013	0.0224
RP11-102M1	0.14	-0.06	-0.032	0.331	0.089	0.0226
MIR6516	-0.016	0.295	0.284	-0.536	-0.409	0.0226
RAET1G	0.19	-0.045	-0.258	-0.097	-0.726	0.0226
HSD11B1L	-0.267	-0.017	0.004	-0.446	0.217	0.0227
AC019097.7	0.344	-0.08	-0.214	0.059	-0.09	0.0227
ZNF285	-0.581	-0.062	0.012	-0.107	-0.217	0.0228
AC027763.2	-0.127	0.037	0.117	-0.305	-0.403	0.0228
RPS10P3	-0.279	-0.339	-0.252	-0.581	-0.346	0.0229
RP11-481J2.	0.33	0.058	0.445	0.173	-0.518	0.0229
FCRLB	0.152	-0.313	-0.353	-0.105	-0.722	0.0229
PKD1P5	-0.383	-0.143	-0.158	-0.49	-0.302	0.023
GKAP1	-0.343	0.326	0.387	-0.104	0.3	0.0232
ARL17A	-0.398	-0.254	0.211	-0.351	-0.332	0.0232
SDHAF4	-0.325	0.009	0.25	-0.058	-0.16	0.0233
DUXAP9	0.153	0.078	-0.026	-0.107	-0.769	0.0234
RNA5-8SN4	0.023	0.491	-0.063	-0.414	-0.449	0.0235
OCLM	-0.418	-0.167	-0.362	-0.883	-0.69	0.0235
SCARNA21	-0.647	-0.304	-0.36	-0.806	-0.884	0.0236
RP11-182J1.	-0.243	-0.062	0.421	-0.301	0.255	0.0237
PRORSD1P	-0.384	0.031	-0.12	-0.473	-0.758	0.0237
GATSL2	0.205	0.189	0.19	0.355	0.581	0.0238
TEAD3	0.582	0.422	0.337	-0.084	0.015	0.0238
RP1-90J20.7	-0.78	-0.292	-0.027	-0.286	-0.199	0.0239
SPATA25	-0.311	0.452	0.353	-0.245	-0.247	0.0239
AC112211.2	0.121	-0.367	-0.515	0.018	0.061	0.024
RP11-621H8	0.054	-0.402	-0.164	-0.138	-0.419	0.024
RP11-123J1	-0.212	-0.196	0.053	-0.278	-0.439	0.0241
C17orf67	-0.555	-0.113	0.068	-0.241	-0.089	0.0242
AC131097.3	-0.143	0.093	0.22	-0.671	-0.479	0.0242
UBE4B	0.216	0.17	0.158	0.365	0.226	0.0243
RP11-214K3	0.254	-0.214	-0.016	-0.644	-0.289	0.0243

WBSCR27	-0.387	0.114	0.378	0.273	0.499	0.0244
RP11-511P7	-0.713	-0.451	-0.127	-0.623	-0.058	0.0244
RP11-109G1	-0.417	-0.164	-0.211	-0.793	-0.687	0.0244
SLC2A11	-0.368	0.014	0.144	-0.311	-0.025	0.0245
SLX1B-SULT	-0.138	0.229	0.054	-0.735	-0.376	0.0245
EEF1B2	-0.185	-0.161	-0.083	-0.36	-0.402	0.0245
RNA5SP82	-0.543	-0.037	-0.382	-0.914	-0.514	0.0245
AC092610.12	-0.453	-0.446	-0.252	-0.422	-0.528	0.0246
RPL23AP53	0.052	0.434	0.196	-0.055	0.357	0.0247
RP5-995J12.	-0.647	-0.554	-0.264	0.135	-0.337	0.0249
RP11-381O6	-0.013	0.428	-0.061	-0.535	-0.429	0.0249
PPIL1P1	-0.059	-0.55	-0.2	-0.1	-0.647	0.0249
RP11-69E11	-0.08	-0.265	-0.268	0.432	0.271	0.0251
SWSAP1	-0.013	-0.01	-0.024	0.254	-0.39	0.0253
LITAF	0.616	0.473	0.359	0.375	0.312	0.0254
ADAMTS10	-0.35	0.096	0.092	-0.502	0.197	0.0255
ZNF737	0.619	0.816	0.678	0.719	0.849	0.0256
S100A4	-0.335	-0.355	0.044	-0.039	0.527	0.0258
RP11-638I2.	-0.349	-0.234	-0.078	-0.559	-0.497	0.0258
FHOD3	-0.079	-0.125	-0.483	-0.293	0.18	0.0259
CTD-2089O2	0.493	-0.138	0.007	0.264	-0.195	0.0259
MAFG-AS1	-0.557	-0.115	-0.054	0.036	-0.46	0.0259
RP4-545C24	-0.211	0.021	-0.04	-0.32	-0.513	0.0259
PYROXD2	-0.102	-0.039	0.427	-0.105	0.434	0.026
MRPL33	-0.336	-0.005	0.138	-0.314	-0.171	0.026
MORN1	-0.534	-0.111	0.216	-0.432	-0.175	0.026
AC005795.2	0.298	0.055	-0.196	-0.069	-0.329	0.026
RN7SL608P	-0.222	-0.139	0.212	-0.743	-0.378	0.026
KIAA1161	0.156	0.079	-0.05	0.162	0.323	0.0263
MFAP1	0.286	0.265	0.047	0.372	0.204	0.0264
RPL31P49	-0.311	-0.309	-0.078	-0.507	-0.331	0.0264
RP11-128A6	0.394	-0.002	-0.068	0.106	0.095	0.0265
RP11-320L1:	-0.156	-0.171	-0.037	-0.337	-0.271	0.0266
RP5-1056L3.	-0.319	-0.364	-0.182	-0.5	-0.349	0.0266
TASP1	-0.124	-0.002	0.052	0.111	-0.33	0.0269
RPL37AP8	-0.541	-0.461	-0.156	-0.534	-0.55	0.027
MIR7161	0.085	0.166	0.171	-0.061	-0.705	0.027
RP11-446N1	-0.274	-0.057	-0.124	-0.654	-0.846	0.0271

FDPSP4	0.465	-0.246	-0.149	0.254	0.179	0.0272
SNORA28	0.155	0.337	0.175	-0.369	-0.396	0.0272
RPS3AP5	-0.277	-0.037	0.105	-0.463	-0.381	0.0274
RP11-51B10	-0.69	-0.661	-0.599	-0.901	-0.94	0.0274
RP11-12M9.4	0.363	-0.073	-0.145	0.24	-0.02	0.0275
RP11-804L24	0.093	-0.53	-0.217	-0.272	-0.461	0.0275
DHFRL1	0.082	0.212	0.198	0.4	0.398	0.0276
RP11-45P22	0.377	0.183	-0.114	0.407	0.368	0.0276
CYP27B1	0.074	0.192	0.136	-0.526	-0.131	0.0277
RP11-459A1	-0.105	-0.352	-0.174	-0.281	-0.337	0.0277
BCL2L7P1	0.209	0.205	0.304	0.398	0.27	0.0281
RP11-363H1	0.104	-0.522	-0.093	0.263	-0.385	0.0281
NDUFA11	-0.254	-0.153	-0.183	-0.318	-0.327	0.0282
AMMECR1	0.113	0.289	0.195	0.359	0.226	0.0283
RP11-373A9	-0.231	-0.241	-0.129	-0.468	-0.422	0.0283
SNORA20	-0.082	-0.088	-0.257	-0.524	-0.885	0.0285
RP11-199F1	-0.136	-0.207	-0.267	-0.871	-0.487	0.0288
RP11-36C20	-0.268	-0.265	-0.047	-0.431	-0.368	0.0289
RP1-131H7.2	0.192	-0.312	-0.524	-0.072	0.015	0.029
ZSCAN9	-0.231	-0.201	0.024	-0.401	-0.048	0.029
RP11-85F14	-0.058	-0.147	-0.338	-0.501	-0.837	0.029
TMEM8B	-0.097	0.421	0.375	0.234	0.093	0.0291
RP11-171I2.5	-0.305	0.504	0.193	-0.367	0.012	0.0291
MIRLET7D	-0.49	-0.002	-0.234	-0.85	-0.542	0.0292
MRPL10	0.066	0.143	0.099	0.326	0.183	0.0293
TRMT11	-0.081	0.046	-0.066	-0.296	-0.347	0.0293
SNORD84	-0.218	0.057	-0.117	-0.728	-0.456	0.0293
MIR210HG	-0.212	0.16	-0.052	-0.464	-0.535	0.0293
RABL2A	-0.628	0.011	0.18	-0.178	0.096	0.0295
RP3-467N11	0.547	0.239	0.168	0.37	0	0.0295
ACTG1P1	0.261	-0.208	-0.276	-0.206	-0.377	0.0295
RP11-692C2	0.169	-0.194	-0.072	0.376	0.403	0.0297
RP11-299L1	-0.01	-0.358	-0.167	-0.912	-0.33	0.0297
RP11-108M9	-0.244	-0.085	0.128	-0.118	-0.586	0.0297
LAGE3	-0.268	-0.17	-0.179	-0.317	-0.374	0.0298
RP11-421P1	-0.694	-0.436	-0.096	-0.513	-0.53	0.0298
RP11-242D8	-0.574	-0.007	0.311	-0.456	-0.365	0.0299
RP11-597D1	-0.047	0.191	0.428	-0.058	0.512	0.0302

PRPF8	0.317	0.165	0.079	0.422	0.288	0.0302
RPS12	-0.35	-0.148	0.007	-0.52	-0.43	0.0303
RP13-204A1	-0.446	-0.242	-0.077	-0.298	-0.282	0.0308
RP11-777B9	0.199	-0.512	-0.613	-0.503	-0.438	0.0308
CTD-2066L2	-0.162	-0.195	-0.122	-0.142	0.609	0.0309
HNRNPUL2-	0.395	0.069	0.06	0.384	0.541	0.031
RP11-304F1	0.087	-0.331	-0.338	-0.032	-0.348	0.031
RP11-434I12	-0.151	-0.374	-0.193	-0.028	0.259	0.0311
RP11-274H2	-0.466	0.09	0.233	-0.191	0.449	0.0312
FKBP9L	0.22	0.009	-0.182	0.555	0.275	0.0313
C14orf2	-0.237	-0.094	-0.036	-0.372	-0.365	0.0314
PRAF2	0.04	0.066	0.033	0.195	0.348	0.0315
ANKEF1	0.026	0.188	0.304	0.504	0.346	0.0316
MIR637	-0.266	0.218	0.182	-0.598	-0.146	0.0316
RP11-168J1	-0.056	-0.354	-0.197	-0.089	0.16	0.0318
RP11-12G12	-0.387	-0.047	-0.025	-0.001	-0.365	0.0318
RNU4-82P	-0.483	-0.001	-0.032	-0.528	-0.6	0.0318
BHLHA15	0.302	-0.145	-0.05	0.037	-0.721	0.0318
AC104113.2	-0.348	-0.498	-0.363	-0.499	-0.794	0.0318
AF038458.4	-0.259	-0.052	-0.148	-0.117	-0.808	0.0321
KB-68A7.1	0.163	0.187	0.814	0.43	-0.13	0.0322
C10orf37	-0.377	-0.264	-0.276	-0.181	-0.67	0.0322
RP4-701O16	-0.27	-0.375	-0.157	-0.258	-0.328	0.0323
U2AF1L4	-0.252	0.111	0.03	-0.578	-0.391	0.0323
GOLGA8T	-0.104	0.129	0.353	-0.459	-0.254	0.0324
AOC2	-0.195	0.066	0.355	-0.364	-0.184	0.0325
RNU1-103P	0.38	-0.149	0.019	-0.595	-0.356	0.0325
RP11-813I2	0.106	0.416	-0.116	0.194	0.741	0.0326
PDF	-0.36	-0.152	-0.096	-0.109	0.275	0.0326
SNORD18C	-0.28	0.124	-0.12	-0.583	-0.73	0.0326
RP11-412D9	-0.751	-0.165	-0.29	-0.497	-0.672	0.0327
SNORA68	-0.242	0.067	-0.2	-0.559	-0.765	0.0327
RP11-442A1	-0.269	-0.216	-0.331	0.047	0.472	0.0328
ZNF695	-0.49	-0.312	-0.781	-0.509	-0.593	0.0328
RP11-467J1	0.281	-0.001	-0.15	0.346	0.144	0.033
SFR1	-0.213	-0.118	-0.024	-0.249	-0.435	0.033
RP11-334C1	-0.207	-0.017	0.083	-0.789	-0.309	0.0331
RP11-403F2	0.017	-0.202	-0.261	-0.003	-0.605	0.0331

RP11-498C9	-0.05	0.31	0.023	-0.397	-0.24	0.0333
MIR6775	0.028	0.246	0.667	-0.062	-0.122	0.0334
RP11-175M2	0.049	-0.09	0.007	0.028	-0.381	0.0334
AC067950.1	-0.431	-0.022	0.019	-0.42	-0.464	0.0334
RP11-817O1	-0.193	0.508	0.174	0.285	-0.028	0.0335
GNG10	-0.145	-0.155	-0.116	-0.479	-0.371	0.0335
CENPC2	0.241	-0.012	-0.072	-0.155	-0.354	0.0337
ZNF197	0.243	0.365	0.266	0.215	0.139	0.0338
SC22CB-1E7	-0.293	-0.267	-0.062	-0.378	-0.393	0.0339
RP11-236B1	0.144	-0.24	-0.047	0.329	-0.331	0.0341
RF01159	-0.141	-0.291	0.212	-0.498	-0.513	0.0342
RP11-242D8	-0.889	-0.366	-0.13	-0.491	-0.658	0.0342
RP11-263K1	-0.324	-0.465	-0.119	-0.247	-0.098	0.0343
RP11-332E4	-0.268	-0.761	-0.833	-0.638	-0.295	0.0343
RP5-1142A6	-0.384	-0.082	-0.018	-0.296	-0.383	0.0345
BEND7	0.211	0.161	0.258	0.187	0.461	0.0346
AC009517.2	-0.187	-0.459	-0.414	-0.326	-0.059	0.0347
RP11-358M1	-0.319	0.051	0.288	-0.595	-0.138	0.0348
RP11-459O1	0.282	-0.318	-0.592	-0.336	-0.379	0.0349
RP13-258O1	-0.417	-0.295	-0.121	-0.597	-0.342	0.035
AC023480.1	-0.024	-0.069	-0.206	-0.154	-0.445	0.035
RP11-736I24	-0.899	-0.474	-0.286	-0.677	-0.643	0.035
YBEY	0.117	0.102	0.182	0.408	0.313	0.0351
RP11-490M8	-0.369	-0.239	-0.104	-0.174	-0.212	0.0351
EIF3C	0.438	0.171	0.024	0.503	0.274	0.0352
RP5-1041C1	-0.006	-0.474	-0.125	0.145	0.137	0.0352
GOLGA8Q	0.041	0.427	0.461	-0.4	0.296	0.0353
AC009362.2	-0.137	-0.017	0.001	-0.451	-0.483	0.0353
DNAJC30	0.037	0.057	-0.027	0.34	0.266	0.0354
RP11-651L5.	0.008	0.226	0.45	-0.276	-0.403	0.0354
AC018648.5	0.476	-0.315	-0.206	-0.111	0	0.0355
RP11-553K2	-0.485	-0.515	0.162	-0.225	-0.68	0.0355
RP11-43F13	-0.154	0.044	-0.02	-0.272	-0.458	0.0357
CTD-2589H1	-0.727	0.154	-0.079	-0.336	-0.498	0.0357
RPS25	-0.339	-0.104	0.015	-0.385	-0.267	0.0358
CXCL16	0.034	0.029	-0.058	0.219	0.594	0.0361
USP27X	0.451	0.275	0.033	0.147	-0.087	0.0362
RNF148	0.273	0.192	-0.128	-0.313	-0.604	0.0362

RP11-1212A	-0.457	-0.378	-0.217	-0.774	-0.856	0.0362
RP11-93I21.	-0.021	-0.478	-0.484	0.038	-0.182	0.0363
ATF5	0.222	0.297	0.178	0.22	0.327	0.0364
RP9P	-0.072	-0.349	-0.234	-0.513	-0.249	0.0364
AC093616.3	-0.684	-0.494	-0.592	-0.981	-0.732	0.0364
RP11-95P2.1	-0.219	0.436	0.488	0.277	-0.247	0.0366
PQLC2L	-0.029	-0.272	-0.355	-0.206	-0.657	0.0366
MAD1L1	0.184	0.052	0.121	0.469	0.198	0.0367
NAGS	-0.383	0.025	0.181	0.085	-0.025	0.0367
AC006028.1	-0.465	-0.237	-0.21	-0.725	-0.621	0.0369
AC133644.3	0.381	-0.139	0.038	0.377	0.407	0.037
SNHG5	-0.25	-0.03	0.046	-0.474	-0.387	0.037
RNU6-26P	-0.208	-0.214	0.351	-0.566	0.218	0.0371
RNU6-1053F	-0.492	0.064	-0.198	-0.744	-0.605	0.0372
RP3-519P24	0.002	-0.586	-0.189	-0.534	-0.296	0.0376
ALG9	-0.02	-0.04	0.061	0.237	-0.428	0.0376
RP11-367G1	-0.496	-0.45	-0.441	-0.752	-0.702	0.0376
PRR29	-0.191	0.054	-0.041	-0.002	-0.812	0.0377
AP001340.2	-0.326	-0.477	-0.513	-0.492	0.275	0.0379
PHLPP1	0.446	0.139	0.059	0.246	0.102	0.038
SARS2	0.071	0.018	0	-0.129	-0.395	0.038
RP13-444K1	-0.147	-0.04	0.071	-0.108	-0.537	0.038
EEF1AKMT4	-0.008	-0.41	-0.378	-0.18	-0.538	0.038
RP11-798M1	-0.772	-0.033	-0.208	-0.16	-0.652	0.038
RP11-324J3.	-0.117	0.219	0.054	-0.085	0.773	0.0382
CYP4V2	-0.092	-0.112	0.154	-0.011	0.494	0.0382
RP11-22P6.3	-0.258	-0.462	-0.462	-0.298	-0.778	0.0382
MIR421	-0.541	-0.123	-0.134	-0.357	-0.64	0.0383
RP11-374F3	-0.052	-0.28	-0.218	-0.115	0.358	0.0384
AP000580.1	-0.317	0.111	0.221	-0.413	0.034	0.0384
AC000403.4	-0.049	-0.333	-0.395	0.1	-0.327	0.0385
POLA1	0.333	0.296	0.059	0.314	-0.039	0.0387
FN1	0.354	0.144	-0.092	0.218	-0.183	0.0387
RP1-178F15	-0.162	0.251	0.537	-0.104	-0.025	0.0388
RP11-498J9.	0.401	-0.268	-0.021	0.206	0.085	0.0389
SNORD24	-0.103	0.216	0.245	-0.577	-0.407	0.0389
DGCR11	-0.076	-0.227	-0.238	-0.26	-0.435	0.0389
RP11-42D20	-0.38	-0.206	-0.011	-0.527	-0.475	0.039

AC115617.2	0.217	-0.33	-0.106	0.169	-0.098	0.0391
RPL39	-0.238	-0.08	0.052	-0.406	-0.367	0.0392
RP11-496I2.5	0.194	-0.213	-0.287	0.266	0.593	0.0394
DGCR6	-0.04	0.081	0.147	-0.298	-0.586	0.0397
RP11-597A1	0.241	-0.088	-0.162	0.159	0.551	0.0398
ZNF425	0.059	0.149	0.56	0.428	0.424	0.0398
MIR503	-0.901	-0.258	-0.26	-0.711	-0.407	0.0398
ZNF137P	-0.177	-0.042	0.356	-0.345	-0.323	0.0399
RP11-74E22	-0.257	-0.507	-0.494	-0.735	-0.898	0.0401
SMIM4	-0.406	-0.296	-0.104	-0.459	-0.33	0.0402
ZNF519	0.065	-0.195	-0.193	-0.195	-0.565	0.0402
RP11-6N17.4	-0.057	-0.1	0.353	-0.069	-0.258	0.0403
CTC-338M12	-0.293	-0.415	-0.334	-0.404	-0.536	0.0403
SPINT2	-0.039	0.317	0.228	0.617	0.423	0.0404
ERO1B	0.197	0.347	0.198	-0.063	0.035	0.0404
AC023485.1	-0.127	-0.081	-0.161	-0.271	-0.489	0.0404
FAM60CP	0.079	-0.265	0.004	-0.057	0.328	0.0406
RP11-861E2	-0.606	0.096	0.315	-0.247	-0.307	0.0407
RP11-563H8	-0.36	-0.29	-0.157	-0.442	-0.339	0.041
RIMS1	0.094	-0.109	-0.135	0.014	-0.389	0.041
SLC45A4	0.122	0.196	0.288	0.352	0.037	0.0411
RP1-69E11.2	-0.419	-0.087	0.289	-0.531	-0.198	0.0411
NHLRC1	-0.421	0.091	0.174	0.076	0.246	0.0413
LL0XNC01-2	-0.335	-0.005	0.336	-0.296	-0.483	0.0414
RP11-185E8	-0.46	-0.554	-0.097	-0.364	-0.625	0.0415
AC090516.1	0.414	0.082	-0.264	-0.016	0.001	0.0417
RP11-791G1	-0.288	-0.409	-0.211	-0.45	-0.366	0.0417
AC073115.4	0.055	-0.18	-0.115	0.21	-0.422	0.0419
CTD-2302E2	-0.199	-0.365	-0.066	-0.456	-0.247	0.0421
RP11-390E2	-0.346	-0.1	-0.418	-0.276	-0.568	0.0423
RPS27A	-0.305	-0.061	0.102	-0.449	-0.43	0.0424
FER1L4	-0.369	0.006	0.058	-0.545	0.062	0.0425
RP11-16E18	-0.483	-0.077	-0.349	-0.247	-0.902	0.0425
RP11-53O19	-0.544	0.014	0.15	-0.124	0.024	0.0426
RP11-177C1	0.106	-0.245	0.216	0.207	0.611	0.0427
RP11-92K2.2	-0.342	-0.155	0.048	-0.501	-0.454	0.0428
PROCA1	-0.615	-0.087	0.022	-0.453	-0.556	0.0428
SNORD55	-0.664	-0.389	-0.296	-0.927	-0.312	0.0429

AC040160.13	-0.238	-0.095	0.196	0.351	0.091	0.043
RP11-68I3.4	-0.085	0.148	0.181	-0.524	-0.577	0.043
PHLDB3	-0.091	0.33	0.273	0.056	0.036	0.0433
RP11-592N2	-0.257	-0.185	-0.125	-0.62	-0.223	0.0433
MIR2116	-0.1	-0.275	-0.406	-0.681	-0.58	0.0433
AC007349.6	0.046	-0.378	-0.14	-0.192	-0.509	0.0434
GABARAPL3	0.43	0.019	-0.048	0.222	0.026	0.0435
RP11-7F18.2	-0.45	-0.098	-0.091	-0.82	-0.479	0.0437
FBXO36	0.079	-0.249	-0.114	-0.002	-0.457	0.0439
THOC7	-0.214	-0.123	-0.089	-0.431	-0.257	0.0442
RP11-355B1	-0.358	0.183	0.289	0.45	0.33	0.0443
ATF4P3	0.368	0.06	0.041	0.187	-0.056	0.0445
MIR4258	-0.23	0.146	0.29	-0.521	-0.439	0.0445
RP11-463C1	0.258	-0.024	0.241	0.122	0.811	0.0446
PSORS1C1	-0.094	-0.022	0.084	0.276	0.431	0.0446
TVP23CP2	0.019	-0.247	-0.052	-0.397	-0.329	0.0448
SNORA79B	-0.686	-0.351	-0.232	-0.8	-0.656	0.0448
RP11-832N8	-0.259	-0.229	-0.106	-0.317	-0.387	0.0449
RP11-433B3	-0.053	-0.19	0.29	-0.13	-0.323	0.045
IL3RA	0.159	0.061	-0.042	0.423	0.28	0.0451
RP11-33B1.2	-0.492	-0.004	0.162	-0.091	-0.303	0.0451
RP1-180E22	-0.462	-0.127	-0.009	-0.182	0.07	0.0452
ARIH2P1	0.457	0.044	0.099	0.34	0.15	0.0455
RP5-1108M1	0.153	-0.438	-0.398	-0.162	-0.135	0.0455
CTD-2224J9	-0.236	-0.104	-0.244	-0.451	-0.819	0.0455
RP11-673E1	-0.082	-0.215	-0.152	-0.376	-0.825	0.0456
CTD-2314I6.	-0.285	0.239	0.439	-0.011	0.076	0.0458
PIP4K2C	0.216	0.144	0.06	0.351	0.212	0.046
RNF7	-0.161	-0.274	-0.278	-0.344	-0.113	0.0461
ARHGAP1	0.332	0.131	0.092	0.309	0.275	0.0462
PRELID2	-0.065	-0.225	-0.4	-0.252	-0.44	0.0462
RP11-270C1	-0.421	-0.22	-0.04	-0.554	-0.519	0.0462
IQGAP2	0.388	0.352	0.187	0.416	-0.178	0.0463
RP11-577H5	-0.192	-0.075	0.282	0.155	0.646	0.0464
GTF2IRD2	-0.411	-0.308	-0.046	-0.366	0.363	0.0464
RP1-8N8.10	0.351	-0.029	-0.063	0.169	0.118	0.0465
ZFAND4	-0.343	-0.125	-0.209	-0.074	-0.535	0.0465
RBM42	0.201	0.132	0.01	0.371	0.2	0.0467

NDUFB6	-0.243	-0.173	0.018	-0.366	-0.366	0.0467
TMEM14B	-0.346	-0.073	0.059	-0.259	-0.184	0.0468
AC011290.5	-0.065	-0.078	-0.082	-0.279	-0.611	0.0472
RP11-267M2	0.446	0.251	0.187	0.368	0.607	0.0473
AP000254.8	0.202	-0.274	-0.445	-0.211	0.43	0.0473
RN7SL368P	-0.529	-0.299	0.41	-0.302	-0.14	0.0473
TMPPE	-0.222	-0.108	-0.049	-0.227	-0.448	0.0473
TCEA1P	-0.39	-0.135	0.012	-0.514	-0.734	0.0474
CSNK1A1P1	0.356	0.012	-0.058	0	0.194	0.0475
RP11-520M5	-0.457	-0.109	-0.186	0.009	-0.173	0.0476
IFIT5	0.467	-0.018	0.01	0.069	0.209	0.0481
ANKHD1	0.111	0.182	0.293	0.385	0.179	0.0481
TSTD3	-0.269	-0.308	-0.503	-0.477	-0.479	0.0481
ING1	-0.273	-0.104	-0.186	-0.183	-0.347	0.0483
AC083871.2	0.465	0.121	0.082	-0.047	-0.417	0.0483
RP11-347H1	0.263	-0.367	-0.151	-0.247	-0.128	0.0484
AC113189.5	-0.546	-0.494	-0.188	-0.36	-0.192	0.0484
RP11-54K16	-0.491	-0.589	-0.221	-0.42	-0.848	0.0484
RP4-796I17.5	-0.303	-0.242	-0.204	-0.523	-0.416	0.0485
RPS3P6	-0.182	-0.136	-0.019	-0.397	-0.007	0.0487
RP11-758P1	-0.111	0.697	0.494	0.162	-0.037	0.0487
RP5-1112D6	-0.664	-0.31	-0.117	-0.778	-0.581	0.0487
CTA-963H5.5	-0.4	0.142	0.388	-0.155	0.087	0.049
TTLL11-IT1	0.327	-0.075	0.01	0.629	0.384	0.0492
SEPW1P	-0.224	-0.351	-0.145	-0.409	0.104	0.0492
MIR7-1	-0.268	0.131	0.037	-0.645	-0.303	0.0493
ATP5J	-0.186	-0.114	-0.098	-0.282	-0.364	0.0493
ZNF775	0.056	0.084	-0.038	0.052	0.41	0.0494
MIR635	-0.462	0.005	-0.434	-0.762	-0.059	0.0495
RP11-64C12	-0.243	-0.243	-0.301	-0.562	-0.57	0.0496
RNU1-106P	-0.078	0.445	0.514	-0.265	0.359	0.0498
PRX	0.069	0.342	-0.007	-0.049	-0.103	0.0499
RP11-167P1	-0.022	0.106	0.082	-0.555	-0.592	0.0499

24 h

Gene symbc	l2fc_2hr	l2fc_4hr	l2fc_6hr	l2fc_8hr	l2fc_24hr	LRT_fdr
ARHGEF3	0.606	0.484	0.312	0.434	0.831	2.05E-12
CDCA2	-1.145	-1.069	-0.889	-0.214	-0.512	1.99E-35
SAMSN1	0.683	0.067	-0.346	-0.106	-0.463	4.35E-29
TBC1D2	0.626	0.592	0.875	1.188	0.72	7.9E-25
KIF2C	-0.606	-0.824	-0.687	-0.305	-0.517	1.27E-22
HJURP	-1.468	-0.936	-1.087	-1.073	-1.598	1.79E-79
RASSF2	-0.695	-0.323	0.102	0.015	0.8	4.22E-67
PPOX	-0.956	-0.102	0.084	-0.209	-0.217	1.74E-16
EXOC3L2	1.195	-0.159	-0.253	0.059	-1.092	8.58E-25
DUX4L50	0.717	1.317	1.246	0.812	1.544	0.00000118
THAP3	-0.605	-0.194	-0.321	-0.343	-0.201	7.28E-08
PAPLN	-0.637	0.344	0.739	0.154	0.54	2.45E-22
FAM221A	-0.669	-0.028	0.064	-0.513	-0.23	0.000424
INPP4B	-0.789	0.465	0.148	0.616	1.703	6.47E-59
SESN1	1.545	1.359	1.31	1.108	1.605	2.08E-155
VASH1	-0.764	-0.66	-0.252	-0.849	-0.334	5.85E-11
ABL2	0.595	0.101	0.307	0.317	0.485	9.75E-14
CLP1	0.588	0.327	0.211	0.512	0.296	4.54E-08
CUZD1	-0.656	0.029	0.347	-0.484	-0.182	0.0000771
PIM3	-0.669	-0.045	-0.521	-0.716	-0.328	1.16E-28
SYNGAP1	-0.965	-0.131	0.19	-0.736	-0.913	2.74E-32
ARHGAP32	-0.722	-0.114	-0.052	-0.355	0.028	1.94E-11
FDXR	1.392	1.6	1.637	1.488	1.646	1.39E-95
SLC7A2	0.976	1.278	0.64	0.136	0.018	4.29E-51
LYST	-0.789	0.064	0.201	-0.07	0.22	3.56E-22
ZSCAN16	-0.743	0.015	0.232	0.246	0.776	4.35E-13
FAM64A	-0.647	-0.906	-0.943	-0.436	-0.39	1.02E-17
SESN2	1.87	1.147	1.305	0.831	1.33	8.93E-130
CCDC150	-0.6	-0.055	-0.106	-0.927	-1.418	1.73E-18
MIR5047	-0.656	0.261	0.236	-0.811	-0.561	1.11E-11
ZNF862	-0.613	0.208	0.504	-0.202	-0.001	5.17E-17
PABPC1L	-0.734	-0.317	0.063	-0.99	-0.447	2.35E-11
LTB4R2	-0.681	0.097	0.223	-0.792	-0.671	3.33E-15
CHKB	-0.578	0.184	0.315	-0.544	-0.328	2.63E-13
HSPA1A	-0.925	-0.313	-0.185	0.122	0.077	1.51E-10
MIR3064	-0.722	0.074	0.151	-1.01	-0.726	5.75E-13
POU2F2	-1.159	0.959	1.321	0.955	2.419	1.39E-175

MPZ	-0.585	-0.139	-0.339	-0.483	0.264	0.00688
GTSE1	-0.835	-0.892	-0.61	-0.281	-0.583	2.61E-37
ZNF79	0.916	0.904	0.852	0.288	0.593	2.32E-22
DTL	0.992	0.022	-0.66	-0.57	-0.726	3.61E-83
SNPH	-0.699	-0.745	-0.138	0.193	0.449	1.41E-45
ACTA2	0.589	1.186	1.511	1.996	2.44	2.99E-74
NBEAL1	-0.592	-0.017	0.168	-0.127	-0.169	2.15E-08
LRRC4	0.88	-0.734	-1.215	-1.267	-1.871	9.66E-24
PROSER2	0.615	-0.007	-0.059	0.218	0.422	1.91E-08
SAPCD2	-0.751	-1.356	-0.97	-0.404	-0.287	8.93E-37
GAS6-AS1	0.679	1.774	2.215	1.635	1.896	1.18E-34
ZNF485	-0.889	-0.105	0.22	-0.247	-0.586	9.98E-08
CCDC144B	-0.773	-0.16	0.095	-0.932	-0.749	9.62E-08
SLC9A3	-0.655	-0.415	-0.671	-0.738	-0.745	0.0000978
SP6	-0.823	0.075	0.58	0.633	1.357	8.67E-96
CCDC68	0.76	-0.109	-0.758	-0.811	-2.356	1.36E-93
HSPA1B	-0.612	-0.073	-0.326	-0.127	-0.251	0.00264
CDKN3	-0.814	-0.906	-0.796	-0.854	-1.017	7.18E-13
CPEB4	0.604	0.322	0.076	0.096	0.226	1.95E-10
LRRC27	-0.846	-0.139	-0.043	-0.644	0.039	2.14E-10
ZNF331	-0.578	0.01	0.255	-0.421	-0.418	3.58E-07
ARHGAP11A	-0.789	-0.752	-0.652	-0.558	-1.044	1.48E-39
MZF1	-0.592	0.268	0.598	-0.096	-0.205	2.08E-22
FGFR3	0.696	0.003	-0.765	-1.193	-2.527	3.43E-62
ZNF365	0.714	-0.391	-0.132	0.241	0.65	5.74E-26
CDKN1A	2.376	2.121	1.81	1.717	2.078	3.72E-141
CDKN1B	-0.677	-0.178	-0.237	-0.16	-0.719	2.24E-33
TNFSF9	2.12	2.236	1.847	1.801	1.995	1.96E-57
CDKN2B	0.673	0.852	0.679	0.885	2.224	1.18E-94
MIR4784	-0.566	0.133	0.07	-0.653	-0.825	0.00146
CDYL2	0.722	-0.021	0.098	0.235	0.786	1.91E-43
LINC00476	-0.712	-0.286	-0.083	-0.393	-0.122	0.00152
CCSER2	0.851	0.469	0.056	0.13	0.071	2.18E-19
ARHGAP33	-0.794	-0.391	-0.362	-1.205	-1.926	1.2E-36
DBF4	-0.809	-0.754	-0.654	-0.706	-1.007	5.67E-24
CARD8	-0.6	-0.225	0.28	-0.075	0.093	1.33E-21
RUSC2	0.683	0.617	0.821	0.684	1.034	1.12E-74
PODXL	0.579	-0.047	-0.282	-0.009	1.023	2.67E-57

IQGAP3	-0.915	-0.467	0.089	-0.318	-0.474	6.11E-17
VWCE	1.286	2.246	2.343	2.015	2.606	1.35E-199
FGF2	1.11	0.858	0.306	0.435	-0.171	4.74E-64
TNFAIP8L1	-0.668	0.079	0.005	0.121	1.21	1.4E-69
ASPM	-1.222	-1.564	-1.409	-1.062	-0.734	8.06E-73
HOXB3	-0.833	-0.325	-0.035	-0.682	-0.102	3.78E-11
HOXB2	-0.737	-0.278	-0.454	-0.535	-0.392	3.06E-13
HOXB5	-0.621	-0.401	-0.129	-0.726	-0.034	0.00000452
HOXB4	-0.992	-0.264	-0.176	-0.244	-0.091	8.33E-26
TSPAN33	0.715	0.291	-0.106	-0.034	-0.928	1.31E-07
FOXC2	0.786	-0.23	-0.175	0.249	-0.417	1.26E-31
MSH5	-0.719	-0.29	0.258	-0.781	-1.145	3.31E-13
MCM10	0.614	0.088	-0.49	-0.451	-1.244	1.09E-72
PRSS53	-0.573	-0.181	0.012	-0.909	-0.764	5.13E-13
PRICKLE2	0.844	-0.157	-0.34	-0.122	0.015	4.42E-12
FOXC1	0.611	-0.145	-0.348	0.05	0.069	2.02E-11
SNORD35B	-0.694	-0.323	-0.164	-1.209	-1.237	1.15E-08
FOXF1	0.599	0.296	0.475	0.859	1.731	5.87E-24
CENPA	-1.959	-1.654	-1.115	-0.623	-0.843	6.4E-54
IL21R	1.534	0.253	-0.048	0.011	-1.148	8.67E-19
CENPE	-0.984	-0.571	-0.628	-0.143	0.129	2.59E-52
CENPF	-0.779	-1.039	-0.728	-0.337	-0.306	9.15E-37
GNB4	0.652	-0.017	-0.184	0.085	-0.025	3.53E-17
HOXD4	-0.828	-0.11	-0.046	-0.702	-0.668	1.12E-07
FAM72D	-0.616	-0.545	-0.575	0.235	-0.434	0.000108
PLEKHA2	0.714	0.001	-0.004	0.315	0.289	5.54E-21
SNAI1	0.589	0.052	0.166	0.33	0.156	0.00182
ANKS3	-0.597	-0.056	0.036	-0.511	-0.425	4.23E-07
ZEB1-AS1	-0.771	-0.282	0.03	-0.357	-0.407	3.28E-07
STK36	-0.665	-0.143	0.082	-0.424	-0.291	1.39E-09
TRIM45	-0.688	-0.145	-0.048	-0.861	-1.053	4.37E-11
NR4A1	1.999	1.172	0.879	0.446	0.388	5.41E-43
HMMR	-0.802	-1.148	-1.149	-0.857	0.041	5.31E-78
GIMAP2	-0.802	-0.168	0.101	0.16	0.218	5.92E-19
TRIM32	0.631	0.408	0.337	0.478	0.767	2.33E-16
PPM1K	-0.874	-0.334	-0.356	-0.524	-0.487	1.21E-07
HMOX1	0.681	0.397	0.486	0.897	1.125	1.78E-24
NSUN5P2	-1.028	-0.175	0.045	-0.649	-0.512	3.16E-12

HMGCS1	0.873	-0.2	-0.699	-0.745	0.01	4.16E-105
MIR7111	-0.661	-0.605	-0.608	-1.047	-1.018	0.00625
HES7	-1.101	-0.168	-0.253	-0.546	0.105	0.00000635
MTHFR	-0.621	-0.098	0.086	-0.04	-0.175	5.49E-19
FUK	-0.684	-0.124	0.015	-0.331	-0.323	1.99E-08
EDA2R	0.797	1.272	1.287	0.679	1.219	3.25E-50
FAM83G	0.683	0.227	0.777	0.802	0.627	9.29E-20
GRASP	0.984	-0.013	-0.085	-0.238	-0.314	1.08E-60
SHB	0.683	-0.082	0.048	0.45	0.779	7.97E-23
GLIS2	0.915	0.204	0.141	0.096	0.69	2.78E-28
KLF13	1.157	-0.084	0.337	0.692	0.809	3.85E-50
SELE	1.765	1.732	0.2	-0.316	2.629	1.52E-38
FBXL19-AS1	-0.633	0.008	0.294	-0.458	-0.519	1.33E-08
PLK3	1.089	0.695	0.764	0.265	0.64	1.21E-32
SOX18	0.578	-0.377	-0.394	-0.286	0.904	4.75E-35
NUF2	-0.68	-0.773	-0.557	-0.27	-0.916	1.3E-34
MIR1914	-0.836	-0.378	-0.529	-1.165	-0.935	0.00000479
VGLL3	0.641	0.136	0.227	0.537	0.306	1.83E-15
WSB1	-0.572	0.096	0.391	-0.47	-0.295	8.04E-12
RNF215	-0.636	-0.295	-0.083	-0.536	-0.52	7.55E-13
RNF207	-0.575	0.576	1.127	0.147	1.303	5.31E-20
MSX2	0.947	0.455	0.065	0.358	0.437	0.00000267
TES	0.755	0.181	0.073	0.412	0.028	7.7E-31
C14orf79	-0.835	-0.096	-0.019	-0.679	-0.255	7.56E-11
ZNF449	-0.731	-0.057	-0.034	-0.66	-0.416	5.53E-08
RHOBTB1	0.599	0.108	0.226	0.455	1.512	1.28E-107
WTIP	0.586	-0.004	-0.264	-0.518	-0.281	4.4E-44
XPC	0.726	0.986	0.91	0.898	1.323	6.37E-69
AGER	-0.639	0.045	0.124	-0.924	-1.043	2.1E-17
PRKCE	0.645	-0.121	-0.038	0.431	0.564	6.12E-26
JAG1	0.705	0.194	-0.098	-0.256	0.865	6.68E-43
MST1	-0.834	-0.018	0.237	-0.764	-0.713	2.52E-11
SPHK1	0.656	0.354	0.268	0.052	1.037	1.04E-48
HIVEP2	1.96	1.702	0.902	0.484	2.073	6.42E-54
HK2	-0.655	-0.375	-0.519	-0.586	-1.128	2.71E-18
SPSB1	1.258	0.072	0.674	1.086	2.131	4.07E-73
SKI	0.779	0.02	0.032	0.209	0.326	1.67E-22
EME2	-0.695	0.041	0.129	-0.708	-0.665	1.37E-09

COX10-AS1	-0.623	-0.334	-0.012	-0.575	-0.231	0.0000674
SKIL	0.737	0.197	0.457	0.421	0.337	1.45E-27
AIM1	-0.665	-0.09	0.326	0.559	1.254	1.57E-80
AHR	0.896	0.441	-0.216	-0.138	-0.401	1.23E-35
RAB30	0.857	0.125	-0.009	0.216	0.069	4.45E-37
BTBD11	0.683	0.005	-0.23	0.024	0.819	1.15E-10
CKS2	-1.135	-0.916	-0.563	-0.96	-0.798	0.00000118
WASF3	-0.626	-0.376	-0.343	-0.424	-0.715	2.01E-34
ALDH1A3	0.698	0.03	-0.396	-0.152	0.111	9.95E-13
PHLPP2	0.676	0.131	-0.148	-0.558	-0.074	1.29E-50
FBXL6	-0.597	0.193	0.041	-0.784	-0.606	9.91E-15
NRG1	-1.283	0.805	0.16	-0.112	-0.891	2.73E-256
ETS1	0.681	0.683	0.691	0.738	1.723	2.37E-140
ALDH3A1	1.637	2.573	2.971	3.609	3.866	1.16E-138
NR1D2	1.366	0.218	-0.069	0.577	1.054	3.95E-65
HHEX	0.627	0.135	-0.096	-0.117	-0.137	4.46E-20
CLEC2B	-0.837	-0.204	-0.3	-0.802	-1.006	7.25E-08
SPSB2	-1.018	-0.281	-0.451	-0.694	-0.95	1.21E-13
CTC1	-0.598	0.252	0.449	0.145	0.214	3.73E-39
MAML3	0.767	-0.493	0.556	0.888	1.245	1.97E-59
DBF4B	-0.861	-0.517	-0.437	-0.804	-0.94	1.84E-18
AMIGO2	0.762	0.633	0.335	0.067	1.026	5.3E-24
PSRC1	-2.334	-2.493	-1.695	-1.101	-1.16	4.18E-102
EVI2A	-0.727	-1.177	-1.096	-0.968	0.366	1.73E-12
PTPN22	0.706	-0.201	0.213	0.722	2.914	1.28E-107
FBXO43	-1.212	-0.722	-0.287	-0.309	-1.363	6.42E-15
RRM2B	0.606	1.02	1.008	1.006	1.278	3.18E-85
RND1	1.821	1.587	-0.099	-0.025	2.075	1.67E-38
ACCS	-0.77	-0.1	0.07	-0.633	-0.146	0.0000013
PLK2	1.436	1.23	1.115	0.827	1.691	4.22E-120
PAN2	-0.713	-0.145	0.318	-0.334	-0.711	8.86E-27
APLN	-1.27	-0.018	0.865	1.041	2.279	1.18E-61
HECA	0.868	0.201	0.192	0.366	0.667	1.6E-33
TRIM46	-1.143	-0.261	0.056	-0.51	-0.239	3.38E-29
BMS1P4	-0.748	0.006	0.241	-0.82	-0.654	0.00000127
ST3GAL1	0.592	-0.068	0.047	0.091	-0.183	6.67E-24
CENPS	-0.587	-0.427	0.005	-0.163	-0.47	0.0112
EZH1	-0.589	0.059	0.333	-0.41	-0.48	2.09E-18

ALPL	0.79	0.689	0.236	0.292	-1.034	2.81E-13
MIR137HG	-0.576	-0.211	-0.089	-0.268	-0.482	0.0016
ALS2CL	-0.578	0.049	0.79	0.267	-0.055	2.34E-22
ZBTB48	-0.799	-0.01	0.002	-0.569	-0.687	1.97E-23
CDCA3	-1.161	-1.101	-0.764	-0.838	-1.505	1.07E-37
MXD3	-1.364	-1.093	-0.508	-0.476	-1.281	1.28E-20
PGF	0.632	-0.047	0.252	0.376	1.5	1.35E-104
SH3BP4	0.741	0.099	0.038	0.287	0.273	5.25E-13
FAM72B	-0.919	-0.531	-0.441	-0.726	-0.984	7.95E-09
CLDN7	-0.571	-0.174	0.194	-0.083	0.823	2.94E-20
POLG2	-0.706	-0.004	0.225	-0.38	-0.59	1.73E-13
SLC25A29	-0.816	-0.315	-0.18	-1.007	-0.816	8.1E-28
PLCXD2	-0.577	0.46	0.762	0.359	1.074	3.52E-26
UNC5B	1.224	-0.184	-0.838	-0.761	0.022	8.12E-31
LRRC45	-0.735	-0.266	-0.057	-0.351	-0.366	6.65E-07
HIF3A	-0.636	-0.41	0.117	-0.272	-1.101	1.44E-40
TROAP	-1.432	-1.086	-0.705	-1.037	-1.062	3.44E-24
CBLN3	-1.016	0.169	0.677	-0.219	0.94	3.91E-29
ACP6	-0.882	-0.04	0.07	-0.574	-0.419	1.41E-10
ZNF799	-0.801	-0.337	-0.284	-0.573	-0.682	0.000661
SNHG11	-0.627	-0.289	-0.181	-0.802	-0.669	0.0000115
GNE	0.605	0.323	0.133	0.219	0.277	1.27E-11
MAP3K6	0.603	0.056	-0.2	-0.605	-1.164	3.01E-100
PIF1	-2.384	-2.239	-1.326	-1.384	-1.354	1.73E-51
FAM46A	1.02	0.669	0.152	0.079	0.076	1.5E-59
SNORA3A	-0.602	0.124	0.006	-0.838	-0.356	0.0041
MST1P2	-0.655	0.027	0.147	-0.383	-0.707	0.000531
TCEANC2	-0.832	-0.164	0.036	-0.309	-0.429	1.35E-16
SLC16A13	-0.727	0.024	0.037	0.05	-0.161	1.11E-09
KLHL17	-0.884	-0.37	-0.226	-1.043	-0.758	3.54E-14
ENOSF1	-0.715	-0.322	-0.15	-0.769	-0.91	2.14E-17
STAG3L5P-F	-0.802	0.001	0.159	-0.909	-0.928	1.86E-21
PLEK2	0.594	-0.15	-0.334	-0.098	0.448	3.07E-31
CENPI	-0.693	-0.341	-0.177	-0.247	-0.461	1.83E-17
RASSF8	0.627	0.169	0.059	0.384	0.847	2.46E-44
ENKUR	0.823	0.423	0.308	0.133	-0.752	3.74E-11
NR5A2	-0.879	-0.122	-0.765	-0.587	-0.643	4.51E-21
STAG3L5P	-0.797	-0.073	0.209	-0.877	-0.646	9.41E-14

LYRM9	-0.683	0.094	0.043	-0.02	0.499	0.000341
CXCL8	-0.937	0.012	-0.628	-1.346	-0.567	6.18E-13
WDR27	-0.818	-0.158	0.38	-0.424	-0.051	2.88E-12
ZNF692	-1.151	-0.08	0.164	-0.886	-0.686	1.83E-23
PHLDA3	1.657	1.824	1.633	1.737	2.151	1.07E-97
MDM2	2.096	1.75	1.922	1.397	1.909	2.57E-175
NIFK-AS1	-0.931	-0.443	-0.218	-0.236	-0.35	0.0000672
ADAMTS6	-0.637	0.02	-0.235	-0.807	-0.75	1.62E-16
MEF2A	0.861	0.234	0.312	0.221	0.503	1.67E-72
CXCL12	0.749	0.03	-0.782	-1	-3.244	2.27E-66
TRPM3	-0.785	-0.124	-0.496	-1.301	-1.536	4.33E-28
C15orf52	-0.728	-0.046	0.719	0.24	0.401	3.2E-27
KLF3	0.742	0.289	0.264	0.288	0.647	1.4E-24
PAPD5	0.692	0.443	0.473	0.58	0.809	1.42E-20
SLX1A-SULT	-0.658	-0.304	0.05	-0.553	-0.608	0.0203
TNFSF18	-0.978	0.252	0.425	0.086	1.224	2.15E-35
MEF2C	0.719	-0.131	-0.135	0.178	-0.092	3.24E-26
IER5	1.026	0.629	0.739	0.848	1.374	8.61E-56
ATF7IP2	-0.71	-0.17	-0.109	-0.436	-0.136	0.000357
TMEM198B	-0.593	0.328	0.549	-0.189	0.203	4.1E-15
TNFAIP8L3	0.953	-0.172	-0.419	-0.276	0.101	2.06E-46
F2RL3	0.74	0.467	1.161	1.294	2.249	6.57E-39
BIRC3	0.633	2.139	1.267	0.358	2.021	5.89E-66
FAM72A	-0.771	-0.216	-0.415	-0.594	-0.776	0.0000242
VSIG2	-0.962	-0.328	0.25	-0.894	-0.406	2.74E-10
CDC42EP4	0.698	0.032	0.225	0.61	0.427	2.51E-12
ANKRD36	-0.755	-0.193	0.016	-0.977	-0.911	2.73E-11
KBTBD3	-0.651	-0.211	0.022	0.008	-0.178	0.00747
MIR503	-0.901	-0.258	-0.26	-0.711	-0.407	0.0398
UBC	0.648	0.228	0.229	0.629	0.658	8.16E-08
FAM131A	-0.668	-0.29	-0.072	-0.07	0.155	1.06E-20
MCM6	0.583	0.067	-0.575	-0.527	-0.765	6.87E-34
GTPBP2	-0.588	-0.06	0.055	-0.395	-0.277	2.43E-14
PDE4D	0.821	0.971	0.666	0.53	0.906	2.43E-32
ABCC5	-0.745	-0.233	-0.05	-0.641	-0.272	4.28E-12
RAB20	0.587	-0.233	-0.087	0.155	-0.124	0.00203
SHROOM1	-0.781	-0.172	-0.02	-0.525	-0.165	1.5E-15
TYRO3P	0.589	-0.063	-0.291	-0.075	-0.182	4.45E-07

CLDN14	1.198	1.063	1.359	1.657	2.87	1.25E-47
FAS	0.871	1.51	1.348	0.91	1.263	4.6E-35
TYSND1	-0.826	-0.595	-0.428	-0.456	-0.364	9.26E-09
DEPDC1	-1.194	-1.64	-1.389	-1.112	-0.643	3.17E-54
G2E3	-0.935	-0.835	-0.799	-0.53	-0.297	2.22E-39
RABL2A	-0.628	0.011	0.18	-0.178	0.096	0.0295
BRICD5	-0.631	-0.283	0.005	-1.078	-0.629	1.1E-10
GJC1	0.656	0.231	-0.03	-0.214	-0.065	4.34E-46
KLF6	-0.668	0.278	0.005	-0.258	0.26	4.57E-65
SCNN1D	-0.707	0.359	0.899	0.102	0.667	1.13E-11
TNFRSF4	-0.87	0.619	1.209	0.905	3.413	1.77E-111
ANKRD23	-0.748	-0.038	-0.035	-1.098	-1.027	1.29E-09
FOXO3B	0.665	0.242	0.21	0.254	0.562	0.000508
WDR76	0.624	0.02	-0.594	-0.789	-1.588	3.11E-140
PLK1	-1.146	-2.204	-1.641	-0.69	-0.644	6.28E-77
AURKB	-0.801	-0.663	-0.378	-0.245	-1.125	5.85E-45
FOXO1	0.586	0.567	0.406	0.49	0.888	4.4E-28
TUFT1	0.776	0.258	0.014	0.276	0.917	7.63E-16
ID2	1.777	-0.006	0.227	0.29	-1.252	2.08E-49
ATOH8	1.326	-0.402	-0.24	0.105	-0.534	6.25E-50
ANKRD33B	0.768	0.583	0.178	0.097	0.178	3.61E-27
DCP1B	0.72	0.792	0.897	0.851	0.928	8.31E-18
CIT	-0.663	-0.603	-0.414	-0.393	-0.273	6.9E-20
ID1	0.955	-0.361	0.003	0.206	-1.865	5.48E-39
GSTT2B	-0.803	-0.03	0.144	-0.564	-0.96	2.14E-16
RCAN3	0.713	0.039	0.217	0.517	0.421	1.04E-08
PMCH	-0.609	-0.178	-0.388	-0.971	-1.55	6.16E-12
ARHGEF39	-1.641	-1.278	-0.793	-0.828	-1.656	1.92E-27
PMAIP1	0.575	0.715	0.526	0.14	1.243	4.79E-36
SPTBN5	-0.609	-0.293	0.148	-0.775	0.548	2.88E-20
MICE	-0.78	-0.112	-0.169	-1.001	-1.211	1.02E-07
HES2	0.677	0.433	0.892	1.227	1.993	1.74E-187
TTK	-0.795	-0.904	-0.851	-0.191	-0.319	4.28E-47
AASS	-0.633	-0.179	-0.198	-0.48	-0.649	4.97E-11
GOLGA6L9	-0.712	-0.107	-0.498	-1.192	-0.675	0.0000297
RHOB	0.867	0.173	-0.235	-0.044	0.259	9.35E-37
IFIT3	1.107	0.061	0.436	1.181	1.64	1.91E-21
ZNF696	-0.691	0.2	0.014	-0.152	-0.11	1.86E-10

SPRED3	-0.784	-0.077	0.037	-0.323	-0.262	2.02E-30
HCG25	#N/A	#N/A	#N/A	#N/A	#N/A	#N/A
ZNF546	-1.089	-0.034	-0.034	-0.264	-0.587	4.48E-08
RASSF9	1.096	0.352	0.629	0.863	1.016	2.04E-16
RCCD1	-0.697	-0.533	-0.106	-0.324	0.045	2E-14
ASGR1	-0.573	0.015	0.407	0.122	0.526	2.67E-08
ZNF514	-0.785	0.051	0.132	-0.498	-0.391	5.71E-08
KIF20A	-0.925	-1.674	-1.54	-0.572	-0.283	8.6E-50
PLAU	0.655	0.743	1.531	1.733	4.057	3.97E-296
KITLG	0.994	1.049	0.781	1.048	2.583	9.34E-145
LRRC75A	-0.642	-0.473	-0.202	-0.264	0.024	0.00118
TTC14	-0.662	0.019	0.08	-0.593	-0.265	0.00000031
LNX2	0.637	-0.116	-0.136	0.064	0.007	8.19E-26
CCDC142	-0.688	-0.104	0.128	-0.435	-0.614	1.2E-12
ARVCF	-0.848	0.088	0.475	0.123	1.517	7.92E-87
CPT1C	-0.625	-0.281	-0.041	-0.307	-0.22	4.94E-09
EXO1	0.828	0.299	-0.235	-0.317	-1.226	9.09E-82
PPP1R16A	-0.656	-0.085	-0.046	-0.339	-0.28	1.06E-10
HERC2P9	-0.616	-0.117	-0.029	-0.807	-0.839	0.000494
ZNF839	-0.732	-0.079	0.149	-0.254	-0.488	7.24E-16
HERC2P3	-1.208	-0.461	-0.229	-1.234	-1.028	4.97E-08
MN1	1.756	0.574	0.789	0.872	1.431	4.62E-43
PIK3C2B	-0.728	-0.577	-0.018	0.008	0.27	3.18E-28
TICRR	-0.664	-0.547	-0.584	-0.873	-0.968	2.12E-16
ZMAT3	0.888	1.07	0.796	0.848	1.082	3.46E-43
ATF3	3.62	2.856	2.38	1.628	2.592	4.81E-256
DEPDC1B	-0.96	-1.083	-0.913	-0.667	-1.498	1.26E-51
SAMD1	0.579	-0.223	-0.827	-1.082	-0.565	3.28E-62
CCNE2	0.858	0.194	-0.632	-1.459	-1.955	1.16E-254
DLL4	-1.421	-0.079	-0.042	0.022	0.448	2.57E-54
HTR1D	0.774	0.482	0.78	1.043	0.603	0.00000154
MRM1	-0.888	-0.045	-0.319	-0.663	-0.725	2.51E-14
MIR6835	-1.05	0.05	-0.064	-0.553	-0.858	0.000101
TP53INP1	1.753	1.398	1.291	1.006	1.758	6.4E-244
RAPGEF4	0.615	-0.109	-0.261	-0.182	0.287	7.87E-12
C10orf10	-1.602	0.34	-0.073	-0.113	1.967	6.11E-62
LBHD1	-0.57	-0.127	0.349	-0.23	-0.262	0.000181
UBE2C	-0.752	-1.399	-1.25	-0.934	-0.683	4.18E-71

TRIB1	-0.843	0.745	0.889	0.694	2.001	1.14E-156
SGO1	-0.674	-0.504	-0.4	-0.065	-0.708	4.09E-15
CBARP	-0.757	-0.109	-0.152	-0.823	-0.108	2.06E-12
RRAD	0.893	1.526	1.087	0.278	2.465	2.56E-26
MTMR4	-0.756	-0.178	-0.197	-0.364	-0.709	8.97E-41
HYLS1	-0.927	-0.851	-0.667	-0.322	-0.246	8.55E-22
MKI67	-0.9	-0.761	-0.542	-0.411	-1.14	3.01E-37
CSF3	-1.789	-0.501	0.25	-0.337	0.392	1.54E-26
CADPS2	0.616	0.087	-0.111	0.115	-0.292	5.15E-13
TBX18	-0.688	-0.212	-0.663	-0.743	-1.089	2.41E-46
SERPINB8	0.685	0.115	-0.125	0.103	0.137	5.33E-27
KNSTRN	-0.835	-0.878	-0.691	-0.312	-0.398	1.44E-40
ANKRD35	-0.682	-0.812	-0.667	-0.605	-1.376	5.73E-09
FAM193B	-0.69	0.048	-0.025	-0.904	-0.801	5.19E-23
TNK2	-0.725	-0.497	-0.092	-0.532	-0.172	1.64E-14
DHRS13	-0.661	-0.393	-0.189	-0.089	-1.123	4.33E-15
REST	1.058	0.249	0.364	0.661	0.69	7.91E-25
DDB2	0.652	1.191	1.211	1.142	1.125	8.19E-88
PARGP1	-0.616	-0.117	-0.093	-0.99	-0.593	1.1E-09
TGIF1	-0.874	0.148	0.228	0.229	1.106	1.55E-84
MIIP	-0.759	-0.358	-0.252	-0.491	-0.561	1.17E-10
GADD45A	1.233	1.076	1.153	0.88	1.778	9.5E-161
GATS	-0.604	0.217	0.267	0.143	0.835	4.59E-11
GPC2	-0.782	-0.165	-0.299	-0.896	-0.1	2.25E-07
TGFBR1	0.838	0.422	0.084	0.017	0.349	8.54E-25
SS18L2	-0.655	-0.314	-0.382	-0.725	-0.918	3.98E-11
DDX18P5	#N/A	#N/A	#N/A	#N/A	#N/A	#N/A
PPP1R35	-0.972	-0.467	-0.193	-0.313	-0.067	8.18E-15
GLI4	-0.715	0.025	0.164	-0.484	-0.705	6.62E-11
KDM7A	1.15	0.19	0.109	-0.299	0.648	5.18E-26
SMAP1	0.591	0.053	-0.211	0.001	-0.033	1.13E-12
BCL6B	-0.728	0.208	0.147	-0.443	0.602	5.94E-39
NOG	1.557	-0.278	0.083	0.402	2.14	4.34E-35
KDM3A	0.799	0.198	-0.1	-0.131	0.264	5.62E-26
KIT	-0.925	0.31	1.18	1.24	2.72	1.08E-42
SNHG10	-0.692	-0.32	-0.295	-0.908	-0.629	0.0000239
HES4	-0.561	-0.079	-0.273	-0.776	0.687	2.34E-09
DDIT3	-0.854	-0.038	-0.089	-0.597	-0.385	1.06E-10

NEDD4L	0.666	-0.074	-0.007	0.296	1.058	1.04E-57
OXSM	-0.624	-0.151	-0.113	-0.422	-0.59	0.00000659
CCDC121	-0.703	-0.169	-0.081	-0.223	-0.481	0.021
DGKQ	-0.637	-0.073	-0.174	-0.822	-0.431	4.27E-13
MFHAS1	0.868	0.084	0.148	0.225	0.65	1.38E-24
PRPF40B	-0.584	-0.169	-0.165	-0.518	-1.36	2.47E-23
NEIL1	-0.867	-0.204	0.045	-0.703	-0.593	2.3E-10
TMEM42	-0.712	-0.208	0.091	-0.281	-0.123	0.00000117
HSBP1L1	-0.645	-0.326	-0.162	-0.609	0.263	0.00000431
C3orf18	-0.628	0.279	0.465	0.055	0.108	1.25E-07
CCDC71L	-0.66	-0.101	-0.39	-0.358	-0.684	1.7E-26
SYNM	0.713	0.756	0.141	-0.364	0.069	4.79E-42
TIMP3	-0.582	0.552	0.69	0.237	0.785	6.87E-08
MIR17HG	-0.748	-0.332	-0.407	-1.57	-1.362	5.89E-18
CLEC4GP1	-1.853	-0.906	-0.02	-0.564	-0.337	3.34E-13
RPS6KA5	0.836	-0.031	-0.089	-0.295	-1.319	1.24E-46
FAM111B	0.663	0.258	-0.43	-0.596	-1.919	1.64E-87
HERC2P2	-1.062	-0.202	0.08	-0.937	-0.873	1.88E-20
FAM27B	-0.61	0.104	0.089	0.033	-0.751	0.0000783
MIR10B	#N/A	#N/A	#N/A	#N/A	#N/A	#N/A
UHRF1	0.826	-0.163	-0.763	-0.764	-0.698	5.58E-63
GJA5	0.594	-0.655	-1.596	-1.354	-3.065	3.31E-50
RGS2	-0.841	-0.599	-0.481	-0.422	0.672	7.09E-18
ZBTB18	-0.622	-0.151	-0.087	-0.245	-0.422	7E-21
ZNF780B	-0.647	-0.08	0.059	-0.352	-0.171	4.93E-07
MIR3189	1.057	1.308	1.355	0.669	1.112	0.0000192
TLL1	0.608	0.014	-0.402	-0.464	-0.784	1.32E-16
C3orf52	1.307	0.868	0.766	0.377	0.613	8.31E-31
EGFL8	-0.569	-0.019	0.06	-0.731	-0.905	0.00000363
CD274	-1.229	0.046	-0.009	-0.447	-0.201	1.37E-53
PMEPA1	0.753	-0.171	-0.357	0.388	1.39	1.98E-53
MIR3153	-1.018	-0.573	-0.371	-0.989	-0.85	0.00361
RFX2	1.24	0.035	0.165	0.46	-0.401	4.36E-29
DLC1	0.711	0.301	0.355	0.493	0.678	8.84E-22
THSD1	1.018	0.853	1.087	1.096	1.193	4.27E-74
RARB	0.705	0.361	-0.02	0.754	1.708	1.33E-30
NDRG1	0.732	0.179	0.009	0.338	0.445	1.23E-12
STEAP2	-0.994	-0.513	-0.775	-1.012	-1.371	1.94E-57

TNS2	-0.638	-0.314	-0.003	-0.323	-0.673	5.54E-31
BAMBI	0.659	-0.199	-0.411	-0.047	-0.51	2.44E-15
FAM109A	0.655	0.103	0.094	0.127	0.419	1.06E-14
SGK494	-0.872	-0.027	0.134	-0.696	-0.627	1.55E-09
ANKRD34A	-0.8	0.15	0.27	0.262	-0.05	4.34E-07
CYP26A1	-2.08	-0.601	-0.947	-1.879	-2.427	1.59E-31
ANKRD36B	-0.794	-0.251	-0.062	-0.681	-0.786	0.0000106
KCNJ2	1.253	0.974	1.622	1.421	1.33	6.59E-24
CEACAM1	0.568	1.604	1.948	2.11	3.632	2.54E-115
GSDMB	-0.652	0.249	0.594	-0.41	-0.07	5.54E-09
RAD52	-0.9	0.006	0.162	-0.537	-0.59	9.19E-22
KIF18B	-1.02	-0.798	-0.54	-0.776	-1.499	2.22E-55
GNB3	-0.621	-0.345	-0.145	-1.261	-1.067	1.15E-16
RAD51	0.573	0.08	-0.372	-0.194	-0.586	1.13E-11
NSUN5P1	-1.025	-0.406	-0.098	-0.95	-0.668	1.45E-10
QRICH2	-0.675	-0.02	0.412	-0.116	0.707	2.86E-12
GNAI1	0.597	0.015	-0.165	0.258	0.431	2.23E-11
FAM212B	1.646	1.532	1.434	1.13	1.716	1.47E-16
MYCBPAP	-0.587	0.441	1.538	0.932	2.55	2.47E-59
MIR5581	-0.903	-0.41	-0.217	-0.912	-0.576	0.0116
ZNF436	0.98	0.601	0.324	0.27	0.66	5.48E-25
PA2G4P1	0.576	0.15	0.112	0.422	-0.019	0.0102
KLF4	1.329	0.253	0.052	0.057	0.149	5.88E-24
C8orf4	1.822	0.212	-0.262	-0.414	0.8	8.18E-35
CEP44	-0.668	-0.179	-0.174	-0.484	-0.654	1.14E-11
BANP	-1.222	-0.23	-0.154	-0.333	-0.376	4.24E-27
NYAP1	-0.934	0.014	0.344	-0.114	0.172	2.94E-07
JAK1	0.636	0.179	-0.086	0.138	0.24	5.91E-08
BBS10	-0.922	-0.319	-0.217	-0.265	-0.592	7.04E-34
HYI	-0.679	-0.185	0.191	-0.014	0.557	2.72E-19
SIRT1	0.708	0.084	-0.089	0.055	0.058	1.07E-24
TOP2A	-0.618	-0.903	-0.728	-0.06	-0.241	1.02E-18
JUN	-1.228	0.261	0.284	0.323	1.119	9.67E-100
BCL3	0.676	0.497	0.152	-0.028	0.486	2.14E-13
JUNB	1.358	0.862	0.048	-0.017	1.115	7.01E-57
RASA2	-0.667	-0.023	0.131	-0.384	-0.28	3.64E-11
LRRC32	0.606	-0.123	-0.049	0.281	-0.065	9.77E-16
ITPKB	-0.629	0.174	0.603	0.705	1.95	1.22E-181

FAM24B	-0.641	-0.074	0.02	-0.223	0.172	0.000577
ACAD10	-0.601	-0.043	0.343	-0.069	0.036	2.06E-12
NPIPB4	-0.56	-0.404	-0.051	-1.197	-0.896	3.62E-15
ITGB8	-0.633	0.507	0.404	0.26	0.449	6.64E-19
DNASE1L2	-0.604	-0.155	0.141	-0.717	-1.187	5.73E-08
MIR221	-0.908	0.284	0.158	-0.964	-0.447	2.55E-08
DNASE1	-0.697	-0.088	0.306	-0.472	-0.277	1.54E-16
RYBP	0.728	0.094	-0.131	0.064	0.329	1.01E-26
PGPEP1	-0.753	0.03	0.427	0.037	0.308	2E-18
TCF7L2	1.162	0.07	0.158	0.365	0.462	5.76E-35
BEND3	0.923	0.056	-0.311	-0.192	0.644	1.39E-22
IRAK1BP1	-0.688	-0.271	0.06	-0.142	-0.025	0.00353
BUB1B	-0.606	-0.783	-0.771	-0.304	-0.575	1E-20
BUB1	-0.659	-1.189	-1.047	-0.361	-0.228	4.73E-54
GALT	-0.598	-0.092	0.053	-0.311	-0.144	0.00000102
BTG1	0.84	0.379	0.293	0.48	1.304	3.31E-136
ORC1	0.767	0.053	-0.699	-0.621	-1.408	2.39E-107
UFSP1	-0.927	-0.373	-0.474	-0.615	-0.524	0.012
PILRB	-0.778	-0.16	0.08	-0.926	-0.743	3.09E-13
SGO2	-0.893	-1.135	-1.112	-0.569	-0.783	3.04E-55
KLF5	0.944	0.456	0.351	0.407	-0.102	0.00000154
CENPL	-0.587	-0.583	-0.497	-0.597	-1.02	7.32E-20
DNMT3B	-0.979	-0.722	-0.62	-0.747	-1.208	3.3E-16
KIF18A	-0.895	-1.408	-1.215	-0.465	-0.355	4.41E-54
HOTAIRM1	-0.629	-0.342	0.107	-0.587	-0.272	0.000478
BMP4	-0.653	-0.086	-0.581	-0.489	-2.128	2.91E-102
SERPINB2	0.593	0.117	-0.355	-0.679	-1.505	1.73E-31
VASN	0.864	-0.072	-0.467	-0.708	-0.894	1.23E-14
RASSF7	-0.71	-0.053	-0.09	-0.411	-0.359	4.2E-11
ACBD4	-0.932	-0.094	0.048	-0.31	-0.241	3.26E-09
BMP2	0.568	0.699	0.78	0.88	3.549	8.73e-316
BCOR	0.966	-0.26	0.188	0.352	0.517	1.71E-18
IRF6	0.73	-0.035	-0.809	-0.98	-2.681	4.66E-109
SLC23A3	-0.598	0.174	0.788	-0.402	-0.184	1.36E-10
ANO8	-0.804	-0.345	0.161	-0.53	-0.339	3.74E-08
LIPG	0.625	-0.232	-0.804	-0.685	-2.105	2.87E-45
HEY1	1.199	-0.237	-0.721	-0.516	-0.154	2.78E-23
FRMD3	0.769	0.125	-0.075	0.078	0.479	4.42E-12

CMAHP	-0.87	-0.461	-0.48	-0.75	-0.322	0.00265
ACAD11	-0.654	0.042	0.41	-0.489	-0.013	0.00000122
DYRK3	0.735	0.179	0.465	0.803	1.196	1.01E-40
ARID5B	0.585	0.355	-0.02	0.096	0.552	1E-10
SNORD93	-0.875	-0.11	-0.249	-0.877	-1.309	0.00000869
SNORA71C	-1.216	-0.359	-0.38	-1.127	-1.457	3.41E-08
IRF1	0.798	1.008	0.237	0.132	1.986	2.1E-122
P2RY11	0.594	0.233	0.341	-0.16	-0.322	0.0000251
MYADM	0.594	0.224	-0.045	0.087	-0.304	1.2E-11
FOSL1	0.669	0.275	0.329	0.362	0.188	1.47E-11
KLF15	-1.112	-0.349	-0.996	-1.356	-2.67	3.99E-38
RECQL5	-0.614	0.057	0.271	-0.205	-0.229	1.65E-15
TSEN2	-0.588	-0.259	0.116	-0.087	-0.242	0.00000363
GATSL3	-0.93	-0.343	-0.539	-0.427	-0.266	0.00535
C7orf13	-0.605	-0.071	0.205	0.263	0.166	0.0000128
EMC9	-0.738	-0.422	-0.379	-0.47	-0.503	7.85E-08
ABCA7	-1.134	-0.403	0.316	-0.201	-0.054	3.17E-15
TBX20	0.907	0.068	-0.022	0.315	-0.147	1.81E-11
SNORA66	-0.658	-0.014	-0.103	-1.016	-0.952	0.000139
PLD6	-0.817	-0.319	-0.174	-0.471	-0.725	0.0000374
CABLES1	1.364	-0.244	0.391	1.104	1.559	1.5E-93
HIST1H2AC	0.585	0.819	1.414	1.663	1.625	2.37E-33
SNORA71A	-0.738	-0.134	-0.138	-0.669	-0.959	0.00356
ZBTB2	0.7	-0.392	-0.57	-0.386	0.169	9.88E-38
SNORA26	-0.71	-0.151	-0.059	-0.83	-0.995	0.000326
INSIG1	0.824	-0.269	-0.567	-0.514	0.749	5.49E-133
TLE6	-0.565	-0.423	0.176	-0.315	0.458	0.00001
FZD1	0.633	-0.265	-0.73	-0.378	-0.701	1.14E-33
HEY2	1.175	-0.446	-0.329	0.153	-1.524	1.69E-54
GGT5	-0.713	-0.279	0.032	0.086	-0.592	9.76E-11
FZD8	0.611	-0.308	-0.472	-0.175	1.151	6.97E-73
SHROOM3	0.664	0.15	0.655	0.891	0.976	4.94E-22
C19orf44	-0.727	0.107	0.135	-0.045	-0.125	4.67E-07
LCA5	0.668	0.629	0.374	0.436	0.527	2.64E-07
KLHL24	1.142	0.752	0.601	0.849	1.932	1.15E-67
SLC40A1	0.789	1.33	0.341	-0.448	-1.44	4.88E-119
ZMYM1	-0.672	-0.225	-0.169	-0.174	-0.308	2.81E-14
MIR126	-1.12	-0.38	-0.499	-1.52	-1.718	5.51E-26

RN7SL1	#N/A	#N/A	#N/A	#N/A	#N/A	#N/A
PCBP2-OT1	-0.6	-0.142	-0.246	-0.986	-0.689	0.00822
FNIP2	0.645	0.057	-0.035	0.08	-0.069	3.01E-24
LONRF3	0.734	-0.021	0.139	-0.031	-0.364	0.00000609
INCENP	-0.743	-0.777	-0.564	-0.203	-0.812	1.25E-31
TEAD3	0.582	0.422	0.337	-0.084	0.015	0.0238
SNORA5C	-0.894	-0.115	-0.09	-0.706	-0.744	0.000474
RNF19B	0.929	0.654	0.618	0.698	1.33	1.1E-59
GCH1	0.896	0.433	-0.13	-0.283	-0.335	4.54E-27
SNORD55	-0.664	-0.389	-0.296	-0.927	-0.312	0.0429
NUAK2	0.625	1.12	0.146	-0.099	2.459	1.06E-48
HIST1H3E	0.719	0.915	1.46	1.163	0.967	3.98E-08
C7orf61	-0.723	-0.687	-0.087	0.145	0.961	1.56E-09
GIN1	-0.609	-0.073	-0.021	-0.159	-0.573	4.49E-07
TFAP2A	0.985	0.717	-0.376	-0.773	-2.085	3.66E-46
TRIM59	-0.771	-0.695	-0.419	-0.285	-0.429	2.01E-15
BCAS3	0.673	0.17	0.14	0.626	0.877	2.62E-09
ZNF367	1.046	0.002	-0.52	-0.583	-0.774	5.37E-163
FSTL3	0.62	0.269	0.345	0.394	0.45	6.46E-22
GATA2	0.703	-0.471	-0.245	-0.038	-0.682	2.21E-45
PCDH1	0.7	-0.096	-0.185	0.183	0.621	4.76E-33
TBC1D3F	-0.576	-0.45	0.015	-0.644	0.023	0.0179
GAS2L3	-1.475	-1.35	-0.892	-0.564	0.117	3.39E-65
SNX22	-0.607	-0.641	-1.339	-1.926	-3.518	8.87E-74
GATA6	0.883	-0.232	-0.178	0.214	-0.203	1.33E-35
GBAP1	-0.819	0.095	0.43	-0.429	-0.398	5.39E-16
OSGIN2	0.675	0.046	-0.477	-0.44	-0.946	3.8E-66
RGPD5	0.719	-0.096	-0.257	0.082	0.17	0.00771
BORA	-1.358	-1.147	-0.977	-0.544	-0.653	1.55E-34
CHST3	1.008	-0.221	-0.188	-0.088	-0.158	5.81E-32
IL11	1.145	0.329	-0.282	-0.618	-0.61	3.16E-26
KIF15	-0.67	-0.46	-0.327	-0.156	-0.99	3.81E-32
SCARNA21	-0.647	-0.304	-0.36	-0.806	-0.884	0.0236
GBX2	-0.658	-0.588	-0.528	-0.241	-1.071	4.48E-26
ADAMTS9	-0.612	-0.227	0.215	0.286	-0.131	3.22E-25
TGFA	0.673	0.254	-0.504	-0.12	0.318	1.49E-07
STX3	0.803	0.147	0.071	0.138	0.383	2.63E-51
SMURF1	0.622	0.239	0.117	0.062	0.664	1.4E-41

L3MBTL1	-0.835	0.043	0.079	-0.457	-0.201	0.0000667
ZFPM1	1.234	0.696	0.768	0.419	0.972	5.69E-37
NUDT16	-0.573	-0.347	-0.138	-0.572	-0.367	0.000113
KIF23	-0.755	-0.908	-0.539	-0.208	-0.649	9.95E-43
FJX1	-0.721	-0.461	-0.252	-0.062	0.507	8.39E-45
EDA	0.59	0.196	0.335	0.478	0	0.00645
NEIL3	-0.687	-0.615	-0.403	-0.273	-1.181	5.1E-26
C9orf47	-0.715	0.277	0.203	-0.721	-0.204	0.00000413
KCNIP2	-0.588	0.004	0.215	-0.881	-0.578	0.000144
RHPN1	-1.097	-0.37	0.388	-0.055	0.773	6.46E-21
MAD2L1	-0.577	-0.549	-0.569	-0.729	-0.826	1.27E-08
TSKU	1.372	0.639	0.733	0.778	1.301	1.07E-58
ZNFX1	0.795	0.448	0.508	0.479	0.982	7.21E-41
CROCCP3	-0.598	-0.22	0.276	-0.644	-0.525	0.000067
GYPE	0.887	0.007	-0.036	-0.15	-0.333	5.19E-14
DGKE	-0.587	-0.208	-0.1	-0.744	-0.633	4.6E-08
AURKA	-1.134	-1.789	-1.506	-0.661	-0.442	4.51E-94
AURKAPS1	-0.804	-0.961	-1.012	-0.738	-0.399	0.0000212
SMAD6	1.094	-0.3	-0.888	-0.984	-2.077	2.94E-30
HOXB-AS1	-0.667	-0.136	0.147	-0.896	-0.607	0.00000673
SMAD7	1.419	-0.189	0.102	0.287	-0.718	1.29E-19
ZMIZ1	0.595	-0.398	0.169	0.603	0.598	1.03E-33
SMAD9	1.256	-0.21	-0.574	-0.431	-1.351	2.54E-54
ZC3HAV1L	-0.637	-0.225	-0.127	-0.413	-0.217	5.85E-07
PTGIR	-0.541	0.431	0.656	0.523	1.817	2.59E-25
NICN1	-0.667	-0.197	0.208	-0.217	0.138	1.2E-08
ENGASE	-0.643	-0.018	-0.153	-0.686	-0.445	4.07E-13
APOLD1	-0.643	-0.147	-0.197	-0.166	-0.004	0.0178
MSH6	0.714	-0.05	-0.582	-0.441	-0.294	8.13E-33
PTGS2	2.054	0.236	-0.45	-0.748	-1.219	6.74E-114
MFSD3	-0.698	-0.14	-0.184	-0.296	0.606	1.17E-18
CCDC14	-0.585	-0.033	0.077	-0.714	-0.758	2.26E-13
CCDC191	-0.741	0.1	0.481	-0.015	0.301	3.99E-07
MIR155HG	-0.578	-0.19	-0.189	-0.837	-0.724	0.0000832
SYCE1L	-0.596	-0.175	0.037	-0.527	0.312	0.00021
AEN	0.911	0.583	0.709	0.44	0.787	2.13E-57
POLQ	-0.622	-0.219	-0.261	-0.638	-0.935	1.51E-26
MTHFSD	-0.586	-0.001	0.162	-0.199	-0.274	7.94E-16

TMEM79	-0.699	0.064	-0.106	-0.696	-0.832	2.53E-21
ARHGAP21	1.061	0.392	0.242	0.292	0.537	6.95E-35
TCTEX1D1	-0.619	0.195	0.362	-0.246	0.133	4.65E-14
PPP1R12B	-0.731	0.02	0.198	-0.598	-0.696	3.89E-18
ZNF654	0.821	0.518	0.358	0.004	0.347	5.07E-25
GDF15	1.882	2.126	1.776	1.622	1.959	1.34E-121
E2F2	0.741	-0.278	-0.795	-1.422	-1.942	1.42E-158
LITAF	0.616	0.473	0.359	0.375	0.312	0.0254
CDK10	-0.595	-0.101	0.02	-0.735	-0.752	4.58E-17
CTC-338M12	-0.636	-0.26	-0.01	-0.459	-0.599	0.000616
ADAMTS1	0.866	0.112	-0.507	-0.557	-1.86	3.56E-143
ZNF248	-0.819	0.033	-0.058	-0.622	-0.493	3.31E-16
MIR421	-0.541	-0.123	-0.134	-0.357	-0.64	0.0383
ZNF397	-0.661	-0.065	0.028	-0.414	-0.647	5.2E-10
TAF4B	0.857	1.061	0.939	0.585	0.693	3.1E-11
PGM5P2	-0.87	-0.234	0.582	-0.263	0.093	1.4E-08
DUSP6	-1.143	-0.118	-0.237	-0.331	-0.209	1.42E-55
APPL2	0.601	0.002	-0.001	0.018	0.202	4.6E-12
LBH	0.853	0.35	0.043	0.225	0.525	0.0168
FAM83D	-1.6	-1.591	-1.24	-0.48	-0.391	1.53E-90
CCNB1	-0.946	-1.877	-1.593	-0.76	-0.194	2.59E-123
FBXO32	1.149	0.772	1.066	1.284	2.27	4.21E-44
E2F7	0.809	0.559	0.455	0.028	0.696	1.42E-43
MIR647	-0.852	-0.166	-0.242	-1.225	-0.962	1.69E-07
TIGAR	0.865	0.987	0.819	0.767	0.984	7.17E-64
ZBTB8A	1.181	0.172	0.122	0.19	0.376	2.34E-24
BTG2	2.559	1.562	1.677	1.436	2.108	8.18E-258
CYP2R1	-0.841	-0.162	0.176	-0.227	-0.486	3.8E-13
CBFB	0.6	-0.106	-0.314	-0.18	-0.159	4.49E-31
HBEGF	0.67	0.57	0.385	0.402	0.754	7.6E-32
CDT1	0.617	0.004	-0.305	-0.336	-0.293	4.08E-24
SAV1	1.106	0.424	0.011	0.147	0.294	3.06E-51
MIR34AHG	0.885	0.856	1.141	-0.114	0.358	8.93E-17
CBS	-0.666	-0.19	-0.467	0.239	0.035	0.000423
CUL9	-0.643	-0.087	0.156	-0.407	-0.477	1.79E-21
PRRT2	-0.865	-0.444	-0.148	-0.83	-0.292	0.000342
KLF11	0.812	0.14	0.16	0.503	0.632	3.48E-14
DNAAF2	-0.588	-0.481	-0.241	-0.104	0.055	2.93E-08

TSC22D4	-0.676	-0.149	-0.024	-0.145	0.043	1.98E-18
SERTAD1	1.176	0.234	0.247	0.268	-0.032	9.42E-76
FARS2	0.612	-0.062	-0.102	0.516	0.298	3.48E-09
ANKH	0.691	0.1	-0.371	-0.27	-0.232	1.45E-29
PIK3R3	0.795	-0.05	-0.054	0.308	0.147	1.22E-27
ENC1	1.274	-0.042	0.222	0.274	1.128	3.08E-63
GPSM2	-1.081	-1.468	-0.869	-0.259	0.083	6.44E-55
CDPF1	-0.588	-0.124	-0.188	-0.401	-0.687	7.4E-11
FBF1	-0.765	0.097	0.236	-0.37	-0.544	5.51E-19
MAPK8IP3	-0.647	-0.067	-0.058	-1.02	-0.61	1.54E-15
RUNX1T1	0.886	-0.443	-0.598	-0.424	-0.423	3.61E-47
DPYSL3	0.571	0.037	-0.382	-0.339	-0.649	5.3E-23
LIF	1.374	1.709	1.827	0.972	2.039	4.91E-15
SOX13	0.6	-0.428	-0.058	-0.124	-0.452	2.26E-42
ANKLE1	-0.702	-0.278	-0.317	-0.815	-0.716	8.11E-12
GEMIN2	-0.604	-0.26	-0.174	-0.561	-0.966	7.17E-14
FRMD4B	0.69	-0.081	-0.046	0.159	0.578	1.11E-33
MTRNR2L8	-0.839	-0.512	-0.276	-1.538	-0.947	1.54E-07
NR1D1	-0.828	0.081	0.294	0.379	0.148	3.36E-09
NABP1	-0.734	0.409	0.403	-0.574	-0.652	3.23E-26
SMIM8	-0.603	-0.094	-0.285	-0.528	-0.506	0.00142
PPM1D	1.464	0.785	0.634	0.457	0.875	4E-114
NEK2	-0.927	-1.25	-1.247	-0.428	-0.03	1.42E-45
ZC3H12C	0.981	0.48	0.379	0.326	0.853	1.33E-44
CCDC36	-0.794	-0.557	-0.23	-0.391	-0.396	0.00000537
LDLR	0.724	-0.516	-1.141	-1.126	0.122	1.74E-118
SPIN3	-0.677	0.138	0.145	-0.367	0.1	3.96E-07
STOM	0.761	0.881	0.723	0.797	0.711	5.73E-63
GPR37	0.83	0.095	0.014	0.265	0.153	6.97E-18
PRDM15	-0.77	0.311	0.241	-0.239	-0.347	1.35E-23
GDPD1	-0.95	0.164	-0.018	-0.328	-0.029	0.00000716
ZNF251	-0.775	-0.271	0.062	-0.567	-0.068	1.22E-08
KLHL35	-0.774	-0.107	0.194	0.243	0.174	0.000334
MIR3191	0.909	1.228	1.391	0.48	0.84	0.00000104
GNA14	0.755	-0.188	0.083	0.437	0.266	0.000921
NEDD9	0.619	0.197	-0.015	-0.06	0.242	5.62E-11
PLEKHJ1	-0.634	-0.342	-0.42	-0.747	-0.69	5.77E-23
ST3GAL4-AS	-0.839	-0.363	0.355	0.032	0.478	1.46E-15

LURAP1L	-0.905	-0.049	0.119	0.358	1.22	2.2E-27
MYEF2	-0.643	-0.191	0.266	0.167	0.7	7.17E-34
ATP8B3	-0.938	-0.566	-0.295	-0.579	-0.684	0.00000485
TACC2	0.689	-0.202	0.336	0.762	1.203	2.14E-74
SOX4	0.683	0.271	0.876	1.285	2.219	1.62E-93
C11orf96	1.024	-0.122	0.038	0.381	0.954	0.00000136
C3orf67	0.687	1.4	1.49	1.296	1.099	2.67E-18
LFNG	1.201	-0.621	-0.234	-0.285	0.611	5.23E-65
MIRLET7BH(	-0.611	-0.278	-0.395	-0.962	-1.009	2.48E-10
RIN1	-0.77	-0.603	-0.155	-0.288	-0.623	3.17E-10
KCNMB3	-0.918	-0.356	-0.204	-0.754	-0.569	0.0031
PDE7B	-0.799	-0.346	-0.117	0.059	0.247	2.77E-29
METRNL	0.813	0.055	0.05	0.435	0.329	1.77E-16
PTP4A1	0.659	0.472	0.286	0.307	0.285	1.8E-25
TRIM69	-0.781	-0.158	0.043	0.009	-0.048	0.000124
BBC3	2.09	1.701	1.827	1.627	2.389	1.64E-89
ELF4	0.74	-0.002	0.014	0.238	0.497	1.37E-26
NFE2L2	0.64	0.239	0.035	0.214	0.072	3.57E-14
NFATC1	0.786	0.188	0.089	0.23	0.165	7.52E-13
EML1	0.588	-0.204	-0.421	0.043	0.186	5.13E-32
NFATC2	0.762	0.812	1.642	1.81	2.853	3.94E-75
LUZP1	0.598	0.146	0.199	0.442	0.827	1.95E-25
SAMD10	-0.851	-0.114	0.058	0.076	0.544	1.99E-13
HEXIM1	0.642	0.144	-0.148	0.122	0.094	1.05E-07
MAP3K12	-0.865	-0.317	-0.059	-0.528	-0.128	3.74E-18
IFFO1	-0.881	-0.398	-0.155	-0.634	-0.274	5.84E-10
CDCA8	-0.947	-1.042	-0.95	-0.391	-0.772	3.67E-44
C21orf58	-1.153	-0.349	-0.187	-0.577	-0.891	5.61E-24
SENP7	-0.757	-0.248	-0.032	-0.485	-0.541	1.74E-11
IQCJ-SCHIP	1.065	0.489	0.649	0.771	1.589	2.87E-40
ANKRD13D	-0.725	-0.226	-0.14	-0.729	-0.47	5.81E-13
NFKBIA	1.116	0.659	0.216	0.081	0.853	1.35E-64
SLC30A1	0.992	0.397	0.383	0.328	0.642	1.05E-42
DHRS1	-0.728	0.015	0.118	-0.12	0.529	2.79E-17
CCNF	-1.291	-0.895	-0.695	-0.403	-0.903	5.32E-65
CCNE1	0.606	-0.068	-0.611	-1.211	-1.268	2.21E-90
ELF1	0.581	0.093	0.017	0.316	0.246	3.15E-13
KBTBD2	0.596	0.042	-0.022	0.035	-0.114	8.96E-37

C1orf74	0.597	0.135	-0.223	-0.195	-0.74	1.55E-24
ANKRD12	0.639	0.267	0.017	-0.134	-0.047	3.9E-28
CXCL2	0.752	0.74	-0.022	-1.092	0.756	3.55E-36
CXCL3	0.966	0.711	-0.194	-0.468	0.981	1.31E-18
SNORD83A	-0.64	0.087	-0.144	-0.926	-0.797	0.0000141
MTCL1	0.633	-0.193	-0.262	-0.597	-0.804	1.1E-47
NKX3-1	0.943	0.61	0.354	0.398	0.908	0.0000614
EGR1	-0.777	-0.185	-1.158	-1.788	-1.733	4.54E-23
MEGF6	-0.614	0.001	0.473	0.203	1.079	4.19E-39
TUBGCP6	-0.634	0.079	0.198	-0.648	-0.763	2.28E-21
CHTF18	-0.621	-0.03	-0.195	-0.953	-1.406	1.45E-30
METTL23	-0.628	-0.223	0.079	0.043	-0.106	3.23E-10
ZBED8	-0.742	-0.107	-0.111	-0.257	-0.543	0.0000378
CDC25A	0.825	-0.142	-0.686	-0.819	-0.874	1.52E-134
SNORD14D	-0.778	-0.495	-0.579	-1.267	-1.467	1.43E-07
ARC	0.638	0.405	0.592	0.415	0.665	7.61E-07
SNORD14E	-0.599	-0.513	-0.381	-1.672	-1.059	5.42E-09
PINLYP	-0.855	0.241	0.416	-0.328	-0.214	0.0000011
GDF7	-0.637	0.563	0.428	-0.006	0.22	1.34E-12
TBC1D3H	-0.726	0.023	0.098	-0.826	-0.595	0.00204
SCARF1	-0.641	-0.245	0.109	-0.008	0.412	6.86E-34
PTPRCAP	-0.782	0.156	0.065	-0.747	-0.689	0.00000802
SNORD14C	-0.575	-0.396	-0.354	-1.193	-1.521	6.66E-09
EFNA1	-0.686	0.125	0.259	0.204	1.464	2.12E-34
ANKS1A	-0.8	-0.465	-0.047	-0.021	-0.145	3.64E-15
KRT7	0.655	0.023	-0.243	0.074	0.971	5.88E-23
CDC6	0.76	0.119	-0.487	-0.553	-1.115	1.05E-111
ANKRD36C	-0.767	-0.144	0.154	-0.965	-1.632	2.82E-13
CDC20	-0.911	-1.817	-1.549	-0.721	-0.673	2.01E-69
C12orf60	-0.784	-0.03	-0.172	-0.627	-1.061	6.13E-09
CDK1	-0.58	-0.475	-0.509	-0.371	-0.709	4.6E-16
ESPL1	-0.963	-0.673	-0.507	-0.599	-1.448	6.14E-68
RHPN2	0.632	0.341	0.069	0.052	0.521	0.00000822
ZMAT1	-0.76	-0.034	0.398	-0.173	0.133	0.00000182
NEURL4	-0.654	-0.14	-0.114	-0.713	-0.742	1.08E-20
FAM111A	-0.642	0.036	0.19	-0.322	-0.796	2.5E-25
ZNF512	-0.588	-0.128	0.077	-0.034	0.15	1.68E-22
ZNF366	-1.302	-0.708	-0.363	0.03	0.161	8.18E-14

SEC31B	-0.798	-0.096	0.196	-0.731	-0.069	2.14E-10
PLPP3	0.867	-0.009	-0.445	0.031	-0.144	2.41E-57
DHX34	-0.691	-0.195	-0.085	-0.461	-0.648	1.45E-32
NR3C1	1.286	0.026	-0.094	0.355	0.108	4.86E-42
SH2D4A	0.62	-0.219	0.028	0.414	0.928	0.00000802
PDCD1LG2	-0.207	0.191	0.421	0.708	0.816	3.19E-56
HES1	0.026	0.061	0.706	0.57	0.977	1.17E-09
MB21D2	0.074	0.117	0.406	1.078	1.866	1.47E-93
STK32B	0.282	0.447	0.995	1.338	2.053	8.89E-182
HSD17B4	0.409	0.231	0.228	0.619	0.491	0.0000581
HSPA4L	0.554	1.116	1.236	1.591	1.511	7.21E-41
HRH1	0.512	0.166	0.451	0.597	0.973	3.78E-41
PTK2B	0.15	0.131	0.417	0.597	0.322	9.49E-07
AK5	0.208	-0.055	0.115	0.612	0.206	0.0000012
ABAT	0.323	0.058	0.231	0.616	0.53	0.00232
ABCA3	-0.18	-0.116	0.324	0.644	1.043	2.91E-32
ACSS1	-0.199	0.474	1.176	0.905	1.943	7.24E-44
CES2	0.419	0.687	0.776	0.872	0.87	2.85E-45
DRAM1	0.496	0.827	0.768	0.823	1.238	1.02E-105
BBS9	0.492	0.137	0.088	0.754	0.609	1.49E-11
TBC1D5	0.488	0.061	0.224	0.778	1.184	2.55E-54
PLEKHF1	-0.493	0.261	0.319	0.636	0.867	6.98E-11
ASCC3	0.318	0.859	0.795	1.138	1.211	9.55E-31
PARP9	0.05	0.219	0.478	0.856	1.242	6.8E-45
HDAC9	-0.055	0.766	0.563	0.599	0.986	2.11E-23
HSP90AA1	0.401	0.197	0.122	0.605	0.585	3.19E-07
CABYR	0.359	0.873	0.859	0.954	1.769	1.39E-19
ACO1	0.27	0.132	0.104	0.585	0.791	2E-10
NTN4	0.172	0.378	0.647	1.131	1.541	3.68E-72
ULBP1	0.503	0.92	0.93	1.013	0.342	0.00000103
FAM167A	0.49	0.272	0.868	1.615	0.866	2.91E-21
TNFRSF10C	0.313	1.014	1.167	1.334	1.729	4.73E-70
SLC9A1	0.528	0.908	0.867	0.988	1.394	3.18E-53
EHD1	0.487	0.232	0.477	0.945	1.364	9.51E-36
PDGFD	0.017	-0.018	0.198	0.587	1.206	1.34E-17
POLH	0.533	0.628	0.621	0.578	0.553	7.7E-21
FGF5	-0.048	0.47	0.41	0.658	0.636	1.97E-27
TNFRSF14	-0.287	0.599	1.079	0.845	1.149	2.24E-57

ELMO1	0.226	0.224	0.332	0.87	1.701	1.72E-84
GDF6	0.053	0.672	0.721	0.611	1.424	2.3E-17
ACKR4	-0.026	0.82	0.805	1.012	1.344	1.72E-09
SMARCA1	0.312	0.262	0.146	0.565	1.012	8.05E-13
IL4I1	-0.032	0.639	0.856	1.265	2.175	2.55E-54
PALD1	-0.374	-0.136	1.057	1.139	2.584	4.18E-74
NLGN1	0.291	0.011	0.241	0.604	0.923	9.82E-16
SLCO3A1	0.27	0.303	0.463	0.613	0.893	5.85E-07
ZBTB38	0.33	0.259	0.41	0.743	0.834	8.61E-24
MOB3A	-0.025	0.134	0.373	0.631	0.627	6.5E-13
PLEKHA6	-0.018	0.344	1	0.988	1.227	5.01E-41
MYO1B	0.224	0.134	0.365	0.68	1.833	1.53E-193
FHL2	0.51	0.622	0.945	1.254	1.669	4.26E-109
ADD3	0.071	0.175	0.409	0.655	1.583	1.72E-60
DGAT2	-0.424	-0.484	0.36	0.764	1.905	2.42E-51
AFAP1L1	0.078	-0.027	0.406	0.609	1.373	8.4E-99
ADPRH	0.406	0.283	0.384	0.66	0.975	3.52E-09
PARP4	0.233	-0.003	0.207	0.588	0.924	1.07E-30
NBAS	0.208	0.101	0.246	0.664	1.396	2.48E-78
FIG4	0.044	0.226	0.391	0.755	0.998	2.6E-44
HMGCL	0.185	0.239	0.375	0.744	0.659	2.49E-20
ADRB2	0.056	0.603	0.74	0.669	0.233	1.84E-25
MAP2K3	0.474	0.289	0.361	0.606	1.049	5.27E-30
MAP2K5	0.066	0.164	0.327	0.62	0.566	4.02E-11
MR1	0.075	0.36	0.422	0.722	0.663	4E-15
IRF2BPL	0.328	0.361	0.465	0.692	0.742	6.64E-11
EPRS	0.399	0.184	0.07	0.56	0.312	0.000862
AK9	-0.137	0.011	0.435	0.58	0.988	1.95E-13
TUBB6	0.453	0.23	0.198	0.677	0.465	1.66E-08
TTC30B	-0.204	0.204	0.244	0.641	0.351	0.00224
APOBEC3C	0.493	0.593	0.921	1.546	2.745	4.02E-91
SDSL	0.151	0.009	0.11	0.599	0.247	0.00000117
CCDC69	0.074	0.18	0.398	0.674	1.399	1.36E-55
PRKAR1B	0.272	0.019	0.549	1.018	1.218	1.75E-31
SLC22A23	0.364	0.661	1.375	1.269	0.883	3.85E-21
CYTL1	0.082	0.555	0.97	1.053	2.515	5.66E-198
FAXDC2	-0.137	0.136	0.526	0.903	2.254	5.91E-26
HIC1	0.286	0.31	1.019	0.828	3.179	1.56E-157

MGARP	0.232	0.944	0.672	1.084	1.216	0.00000223
STARD10	-0.102	0.268	0.645	0.615	1.224	4.75E-35
NOXA1	-0.399	0.059	0.912	0.574	0.726	0.00000232
TNFSF15	-0.336	0.56	1.691	2.056	5.46	4.85E-267
CPNE3	0.27	0.114	0.149	0.585	0.83	1.7E-23
IFIH1	-0.001	1.033	0.909	1.054	2.07	2.14E-38
DOPEY2	0.074	0.317	0.474	0.822	1.071	4.12E-21
PHGDH	0.062	-0.015	0.119	0.706	0.739	2.47E-10
UBALD2	-0.054	-0.001	0.268	0.596	1.333	1.21E-65
CARD6	0.435	0.353	0.334	0.656	0.762	1.1E-18
ALDH3A2	0.546	0.73	0.832	1.145	0.739	2.47E-63
TRAF3IP2	-0.336	-0.164	0.344	0.873	1.162	9.36E-48
FAM129A	0.26	0.57	0.669	1.187	1.469	1.6E-24
NLRC3	0.547	-0.32	0.53	0.836	1.718	1.27E-48
CMBL	0.433	0.866	1.417	1.776	2.558	8.48E-166
RINL	-0.078	0.159	0.741	1.032	0.969	1.78E-23
TSC22D1	0.426	0.323	0.412	0.657	0.92	1.42E-52
RASSF4	-0.337	0.192	0.641	0.636	1.271	2.55E-27
TOM1L1	0.124	0.462	0.365	0.652	0.928	0.000173
CLDN1	0.386	1.205	1.394	1.628	2.315	5.83E-36
PCED1B	0.073	0.155	-0.003	0.583	0.83	0.000797
FERMT1	0.443	0.122	0.044	0.629	0.875	3.61E-11
FILIP1L	0.351	0.216	0.332	0.792	1.486	3.17E-85
SLC7A7	-0.255	0.158	0.641	1.058	1.959	1.41E-197
PHACTR1	0.161	-0.515	0.498	0.771	1.209	9.56E-76
MBP	-0.024	0.244	1.004	1.693	2.13	9.1E-32
CPA4	-0.055	0.71	0.918	0.955	0.631	1.66E-16
DDX60	-0.097	0.009	0.407	0.641	1.054	2.15E-27
ANXA4	0.317	0.313	0.435	0.697	1.369	2.92E-96
SIRPB2	-0.019	-0.338	0.235	0.647	3.373	7.12E-161
IL1A	0.258	0.519	1.256	1.02	3.087	3.61E-134
SH2D2A	0.23	0.444	0.93	1.309	2.47	3.98E-54
GATB	0.257	0.228	0.242	0.602	0.503	1.46E-08
GALNT15	-0.241	0.044	1.002	1.283	1.395	3.76E-28
PSTPIP2	0.364	1.105	1.009	1.036	1.341	1.86E-70
CCDC85C	-0.226	-0.548	0.141	0.641	1.661	1.56E-146
GPRC5A	0.319	0.09	0.436	0.758	0.347	2.26E-13
AOX1	0.055	-0.018	0.191	0.903	2.374	1.89E-182

ANK3	0.327	-0.149	0.201	0.63	0.888	1.21E-22
CREBL2	0.128	0.193	0.363	0.63	0.681	9.16E-35
GALNT6	0.195	0.077	0.296	0.705	2.073	1.76E-138
CREM	-0.092	0.319	0.62	0.592	0.69	2.83E-21
GTF2IRD2B	0.524	0.199	0.591	0.686	1.386	1.92E-10
CCRL2	-0.507	-0.069	0.603	0.868	0.551	7.77E-39
TMCO4	0.037	-0.124	0.327	0.589	0.773	3.38E-09
SECTM1	0.012	0.229	0.512	0.961	1.437	3.36E-12
RAD50	0.039	0.119	0.198	0.581	0.404	0.000118
UCP2	-0.179	0.376	0.55	1.131	0.682	1.26E-15
PCDH12	-0.206	0.335	1.142	1.527	3.511	5.5E-290
COL17A1	0.015	0.148	0.705	0.634	2.376	4.37E-57
PDGFA	0.523	0.332	0.554	0.584	0.337	9.45E-16
SDHA	0.253	0.198	0.196	0.68	0.298	0.00000139
TSPAN1	0.153	0.231	0.817	0.798	1.037	0.00000126
APOD	0.28	0.737	0.982	1.319	1.961	4.04E-39
FAM107A	0.104	0.144	0.225	0.68	0.418	4.14E-09
PDGFRL	0.056	0.087	0.302	0.665	1.186	7.9E-33
SDC1	0.418	1.19	1.271	1.696	2.248	1.38E-52
C1RL	-0.094	0.079	0.273	0.58	0.744	8.97E-24
CXCL5	0.026	0.853	0.856	0.919	2.548	2.32E-43
FUCA1	0.148	0.56	0.735	0.95	1.594	2.81E-48
APLP1	0.264	0.528	0.481	0.905	0.726	1.04E-10
DDX58	-0.224	0.658	0.692	0.925	1.947	6.32E-181
MCC	0.235	0.077	0.231	0.639	1.098	2.5E-56
CROT	-0.057	0.643	0.603	0.592	0.671	4.64E-19
PCSK1	-0.04	-0.133	-0.149	0.657	1.032	6.32E-12
TMEM173	0.237	0.369	0.548	0.608	0.411	1.25E-27
STAP2	-0.138	0.39	0.919	0.727	2.356	8.58E-53
STPG1	0.068	0.067	0.422	0.62	0.438	0.00000149
TYMS	0.32	0.44	0.386	0.601	0.305	2.02E-16
RSPH3	0.246	0.463	0.485	0.803	1.382	1.29E-24
PTP4A3	0.267	-0.165	0.338	0.687	-0.175	1.35E-08
PDE1C	-0.379	0.029	0.517	0.73	0.825	2.15E-72
SLC15A3	-0.048	0.262	0.319	0.746	1.613	2.8E-22
P4HA2	0.282	0.214	0.156	0.614	0.78	8.29E-16
AQP3	0.172	0.175	0.349	0.653	-0.981	4.66E-11
CCDC125	0.052	0.014	0.541	0.705	0.886	1.95E-08

PTCHD4	-0.282	1.055	0.919	1.001	1.729	3.89E-39
ARRB1	0.059	0.096	0.555	0.71	1.405	1.06E-49
TNFSF4	-0.075	0.605	0.874	0.867	1.852	1.49E-117
TENM3	0.152	-0.008	0.201	0.677	1.38	2.92E-48
NSFP1	#N/A	#N/A	#N/A	#N/A	#N/A	#N/A
FLT3LG	0.099	0.708	0.885	0.704	0.986	0.000131
PARP14	0.006	0.602	0.871	0.889	1.905	3.07E-131
MBNL2	0.516	0.113	0.342	0.786	1.131	2.01E-50
EBI3	-0.021	0.987	0.849	1.106	1.859	2.13E-22
ZC3HAV1	0.452	0.48	0.473	0.807	0.82	6.47E-09
REPS2	0.396	0.143	0.256	0.706	0.91	1.38E-16
OPTN	0.123	0.482	0.535	0.642	1.289	5.56E-80
PKP2	0.001	0.098	0.417	0.591	0.376	0.000999
OSBP2	0.258	0.093	0.285	0.644	1.411	1.04E-42
CTSS	0.063	0.339	0.41	0.72	2.041	3.62E-185
PKM	0.362	0.117	0.089	0.569	0.833	1.42E-10
SP110	0.293	0.245	0.334	0.652	0.885	4.51E-12
PLAT	0.286	0.133	0.307	0.919	3.139	7.03E-245
FRMD4A	0.3	0.092	0.514	0.618	0.932	1.28E-78
DBNDD1	-0.077	0.051	0.362	0.818	1.428	4.68E-105
APLF	-0.173	0.825	1.102	0.951	1.11	5.33E-21
PLD1	-0.245	-0.034	0.621	0.941	1.649	1.11E-95
ARL4C	0.44	0.465	0.664	1.157	3.398	5.32E-146
CHAC1	-0.212	-0.102	0.53	0.6	0.44	1.1E-08
STX6	0.333	0.405	0.471	0.641	1.15	6.92E-44
NDST1	-0.012	-0.02	0.228	0.587	1.632	1.64E-187
B4GAT1	-0.079	0.088	0.093	0.592	0.734	4.76E-24
MMP14	0.146	0.245	0.376	0.702	1.439	2.93E-103
CSGALNAC	-0.022	0.069	0.567	1.121	2.116	2.29E-93
PIK3CD	-0.223	0.604	0.742	0.654	1.672	6.77E-118
FAM63A	-0.308	0.132	0.447	0.6	0.581	6.28E-23
ADM2	0.479	0.562	0.861	0.986	1.413	1.28E-07
DTX3L	-0.033	0.646	0.8	0.997	1.612	7.43E-90
PDLIM1	0.293	0.379	0.476	1.022	1.408	6.18E-45
AJUBA	0.012	0.181	0.547	0.603	0.517	7.01E-28
ALDH7A1	0.341	0.283	0.313	0.736	1.669	8.74E-87
PCYOX1	0.174	0.128	0.16	0.597	1.112	1.23E-38
NMI	0.168	0.07	0.156	0.578	0.672	9.62E-19

ATP2B4	0.128	-0.038	0.296	0.595	1.38	4.84E-93
ICAM2	-0.259	-0.171	0.195	0.628	0.803	9.2E-45
PHYH	0.176	0.392	0.514	0.578	0.556	0.0000121
RAB38	0.192	0.353	0.531	1.017	0.479	0.0051
MEDAG	0.283	-0.837	-0.137	0.662	1.424	2.52E-92
TMEM217	0.485	1.866	1.801	1.525	3.171	1.1E-122
HNRNPA1P7	0.222	0.219	0.265	0.624	0.475	2.4E-18
FRMD6	0.095	0.573	0.785	0.866	1.963	6.69E-145
DDIT4L	0.369	0.586	0.699	0.788	2.917	7.34E-62
ICAM1	0.116	1.137	0.956	0.963	2.611	1.57E-89
NR3C2	-0.363	-0.083	0.141	0.615	0.985	4.48E-31
DDB1	0.463	0.259	0.231	0.648	0.635	0.00000962
F8A1	0.025	0.115	0.311	0.78	0.829	5.14E-10
TGM2	0.148	-0.123	0.058	0.701	1.255	7.54E-33
EEFSEC	0.314	0.223	0.363	0.673	0.246	0.0000138
MERTK	-0.278	0.599	1.139	1.158	1.778	3.81E-87
EFL1	0.305	0.169	0.437	1.012	1.085	7.73E-27
WSCD1	-0.243	0.107	0.607	0.952	1.849	2.43E-88
REV3L	0.489	0.807	0.705	0.693	0.997	8.99E-41
OGFRL1	0.278	0.267	0.522	0.645	1.988	5.09E-209
AXL	0.1	0.095	0.328	0.672	0.779	1.05E-30
RELB	0.213	1.85	1.101	0.575	1.811	1.85E-98
C14orf93	0.287	0.186	0.413	0.599	0.425	0.00732
ICOSLG	0.307	1.197	1.432	1.036	1.96	1.79E-36
ZER1	0.205	0.198	0.382	0.709	0.777	4.34E-13
UBE2L6	0.151	0.088	0.325	0.744	2.148	1.45E-70
KISS1	-0.318	0.002	0.247	1.108	1.859	6.29E-21
IL32	0.221	0.52	0.757	0.823	2.378	3.45E-209
APOL3	0.073	0.762	1.47	1.259	2.578	4.83E-142
SASH1	0.273	0.153	0.71	0.967	1.299	1.1E-81
DYSF	0.24	0.03	0.414	0.887	2.234	1.04E-154
DARS	0.321	0.137	0.227	0.661	0.647	4.38E-16
DAB2	0.268	0.172	0.338	0.612	1.317	1.38E-102
RHBDF2	-0.181	0.245	0.795	0.85	1.64	5.35E-185
DGKA	0.067	0.597	0.871	0.66	0.567	1E-79
SYNE1	0.117	0.355	0.565	0.783	1.117	1.06E-58
KCNMA1	0.192	0.096	0.56	0.77	2.841	4.13E-190
TLR1	-0.137	0.228	0.277	0.618	0.944	6.81E-13

KCNN4	0.334	0.065	0.567	0.869	1.165	1.2E-10
TLR3	0.574	0.664	0.71	1.038	1.41	9.96E-17
NSF	0.477	0.221	0.245	0.592	0.414	0.000136
ANGPTL4	0.035	0.031	0.983	1.277	1.391	4.46E-57
GAL3ST4	0.518	1.024	1.342	1.784	2.303	5.13E-53
RAP2B	0.568	0.249	0.35	0.606	1.129	7.09E-66
C21orf33	0.087	-0.197	-0.074	0.515	0.464	0.0127
OAS2	-0.03	-0.014	0.611	1.101	2.157	5.54E-91
CITED2	0.208	0.273	0.266	0.614	0.413	0.00000029
OAS3	0.171	0.472	0.562	0.817	0.948	2.52E-17
CD82	-0.048	0.18	0.808	1.349	3.282	3.6E-162
APOL6	#N/A	#N/A	#N/A	#N/A	#N/A	#N/A
TRIP4	0.336	0.235	0.262	0.585	0.379	4.45E-10
MYO5C	-0.311	0.146	0.819	1.03	2.442	2.11E-92
JUP	0.184	0.078	0.302	0.906	2.285	1.31E-159
KIAA1211L	0.242	0.367	0.736	1.026	1.729	1.92E-83
DUSP16	0.357	0.537	0.558	0.617	1.276	4.04E-33
BBS2	-0.066	0.195	0.503	0.675	0.834	4.1E-30
DDX18P1	0.329	0.322	0.189	0.609	-0.04	0.0201
PTER	0.212	0.174	0.014	0.718	0.808	4.55E-12
DPF3	-0.523	0.143	0.774	0.584	1.058	4.78E-39
TTC12	0.205	0.318	0.65	0.948	1.095	1.49E-13
SPATA18	0.565	1.447	1.726	1.843	2.242	2.58E-158
CCND1	0.182	0.036	0.269	0.607	0.925	2.17E-59
GMDS	0.515	0.202	0.094	0.575	0.86	1.15E-22
CXorf36	0.001	-0.042	0.558	0.681	3.101	1.09E-71
ADAMTS18	-0.388	-0.065	0.617	0.693	2.15	3.17E-23
OGDH	0.456	0.183	0.117	0.561	0.243	0.000235
TRIB3	-0.296	-0.026	0.328	0.67	0.834	2.43E-26
BCL2L1	0.49	0.528	0.68	1.035	1.091	2.05E-33
FAM210B	0.483	0.452	0.59	0.883	1.646	1.31E-179
SULF2	0.454	0.35	0.465	0.883	2.26	1.29E-188
DYNC1I2P1	0.41	0.155	0.107	0.617	0.681	2.07E-12
TCEA3	-0.207	0.577	0.906	1.035	1.63	5.01E-14
ANGPTL2	0.51	0.495	0.778	1.348	2.288	7.93E-111
DYNC1I2	0.354	0.162	0.094	0.577	0.653	3.37E-14
TNIP1	0.056	0.607	0.744	0.912	1.663	7.75E-106
RHOD	-0.183	0.368	0.166	0.682	1.413	1.17E-12

ITGA2	0.069	0.913	1.015	1.131	1.776	5.77E-137
SUGCT	0.311	0.352	0.736	1.19	2.108	6.3E-39
MORN4	-0.117	0.077	0.346	0.601	0.471	0.000126
ITGA3	0.182	0.099	0.272	0.64	1.027	4.69E-35
DIAPH2	0.521	0.065	0.292	0.701	0.76	5.21E-24
GALNT1	0.068	0.228	0.279	0.596	0.861	1.02E-45
EPHX4	-0.132	0.173	0.561	1.26	3.134	5.05E-52
DLG1	-0.188	0.12	0.399	0.617	1.209	2.5E-114
CLMN	0.233	0.034	0.915	0.878	2.073	8.52E-56
PSAT1	0.174	0.017	0.248	0.712	0.454	6.43E-10
SCHIP1	-0.085	0.85	1.535	1.281	2.235	1.24E-31
THAP8	-0.269	0.086	0.163	0.621	-0.121	0.0000133
CDIP1	0.458	0.81	0.896	1.123	1.118	4.14E-27
IRAK2	0.414	0.909	0.684	0.616	1.847	7.55E-106
DENND1A	0.419	0.015	0.35	0.738	0.377	2.78E-13
WDFY4	-0.084	-0.241	0.059	0.612	0.886	1.1E-39
BNIP3L	0.211	0.322	0.589	0.994	1.679	1.68E-121
SLC7A14	-0.304	0.4	0.96	1.256	1.953	4.61E-97
HIST1H2BK	0.37	0.367	0.382	0.945	0.936	4.82E-13
P2RX4	-0.07	0.236	0.44	0.657	1.701	5.87E-90
ISCU	0.212	0.546	0.689	0.611	0.833	9.62E-42
TRIM22	0.443	0.963	1.104	1.164	1.584	1.85E-227
LOXL4	0.289	0.517	0.638	0.98	1.34	7.92E-13
LL21NC02-2	0.129	0.434	0.318	0.59	1.303	1.72E-07
CCDC136	-0.197	0.756	0.909	0.713	1.046	5.29E-17
ZFAT	0.332	0.209	0.254	0.766	0.46	1.98E-07
INPP5D	-0.19	-0.44	0.236	0.686	0.79	1.22E-44
RNASEL	-0.186	0.231	0.327	0.785	0.888	6.14E-09
TNS3	-0.077	0.098	0.594	0.855	1.453	7.43E-127
MGLL	0.244	-0.027	0.191	0.697	1.597	7.88E-112
GPHN	0.531	0.206	0.301	0.672	0.261	0.0000117
HIST1H2BC	0.45	0.679	0.811	0.99	0.377	0.0039
PALM	0.367	-0.218	0.326	0.704	1.309	7.62E-12
NRROS	0.095	-0.349	0.161	0.687	0.984	3.13E-14
SAMD9	0.374	0.304	0.429	0.83	0.996	7.92E-18
MYH16	-0.003	0.839	2.054	2.735	5.406	1.82E-176
CADM4	0.185	0.613	0.567	0.73	2.118	6.67E-31
DFNA5	0.362	0.39	0.699	1.155	1.718	2.93E-123

PDGFC	0.439	0.797	0.61	1.008	1.615	9.7E-117
IL12A	0.544	1.478	1.564	0.608	1.6	1.59E-12
MFSD6	0.216	0.496	0.382	0.614	1.45	9.69E-19
GBP3	-0.246	0.092	0.457	0.628	0.973	1.19E-77
CTNNBIP1	0.119	-0.039	0.184	0.581	1.712	3.19E-220
SH3BP5	-0.003	0.143	0.476	0.766	1.887	1.98E-146
PSMD1	0.488	0.269	0.191	0.61	0.218	0.000162
CHST1	-0.124	-0.236	0.611	0.782	2.274	4.68E-151
RPH3AL	0.059	-0.224	0.317	0.821	0.691	2.41E-16
EEF1A2	0.424	0.278	0.303	0.751	0.594	0.00435
SYT9	0.179	0.002	0.231	0.669	1.559	5.08E-73
PSMD2	0.402	0.224	0.21	0.641	0.9	1.45E-16
YBX3	0.388	0.594	0.724	0.847	1.229	1.54E-107
ARHGAP22	-0.196	0.042	0.707	0.936	1.485	1.75E-125
APOL1	0.258	0.363	0.68	1.093	2.229	2.19E-97
PSMB9	0.065	0.433	0.738	1.047	1.989	1.61E-31
NBR1	0.275	0.16	0.308	0.717	0.828	3.36E-24
EHD2	0.326	0.089	0.08	0.563	0.679	0.00000295
MARCKS	0.192	0.011	0.075	0.644	1.241	1.29E-63
SLC47A1	0.107	0.453	0.59	0.755	0.406	1.9E-17
HCG11	0.259	0.318	0.468	0.79	0.626	6.39E-07
SMAD3	0.35	0.642	0.889	0.778	1.727	1.04E-112
MYLK	0.217	0.019	0.18	0.634	1.599	3.82E-112
WDR66	0.398	0.195	0.617	0.66	1.361	4E-15
LRP1	-0.029	0.446	0.702	0.613	0.88	0.00000236
SH3RF3	0.328	-0.077	0.538	0.976	1.946	2.65E-162
NIPAL3	0.127	0.252	0.483	0.81	0.777	5.68E-45
SIPA1L3	0.216	0.016	0.728	1.071	1.93	9.87E-213
MPP4	-0.096	0.034	0.699	0.924	2.993	3.37E-143
TRIM5	0.481	0.572	0.583	0.763	0.977	5.23E-18
TMEM140	0.102	-0.034	0.192	0.572	1.78	1.37E-31
LRRC71	-0.44	-0.05	1.112	0.617	0.367	1.78E-09
LMF1	0.024	0.084	0.078	0.653	0.427	2.27E-08
LTA4H	0.17	0.226	0.316	0.768	0.909	3.76E-27
HSDL2	0.343	0.335	0.439	0.689	0.766	9.34E-22
LRP10	0.15	0.202	0.222	0.674	1.054	1.08E-39
CYFIP2	0.353	1.565	1.803	2.174	2.523	3.17E-142
EPS8L2	0.103	1.353	1.842	1.662	2.574	3.22E-137

BHLHE40	0.496	0.399	0.886	1.074	2.47	1.17E-78
CDCP1	0.112	0.138	0.556	1.101	3.544	4.06E-251
LMNA	0.432	0.174	0.125	0.567	0.588	0.0000247
LRRC2	-0.256	0.033	0.331	0.691	0.459	0.0000154
F11R	0.169	0.554	0.812	1.127	1.723	2.88E-175
PARD6A	-0.523	-0.277	0.493	1.021	0.875	4.81E-13
CCND2	-0.489	0.35	0.967	0.829	2.169	5.2E-221
PLEKHG1	-0.43	0.427	0.799	0.907	1.855	7.49E-153
SPINT2	-0.039	0.317	0.228	0.617	0.423	0.0404
RAB3D	-0.242	-0.138	0.233	0.592	0.754	4.26E-37
VSIG1	0.382	0.402	0.463	0.797	0.227	1.06E-08
CIR1	0.307	0.357	0.166	0.587	0.509	5.31E-07
GAS6-AS2	0.104	0.36	0.769	0.779	2.108	6.97E-34
TP53I11	-0.038	0.22	0.543	0.704	1.946	7.64E-172
ETNK2	0.106	0.198	0.297	0.684	0.578	1.49E-12
HIST1H2BD	0.147	0.264	0.759	1.123	1.246	5.56E-07
PNMAL1	0.032	-0.302	0.194	0.708	0.551	1.16E-14
CELF2	0.097	0.138	0.533	0.933	2.34	1.15E-67
ABLIM1	0.218	0.456	0.647	0.97	2.007	1.82E-89
KHDRBS3	0.024	0.026	0.161	0.591	0.704	8.95E-19
TTC30A	-0.086	0.212	0.068	0.645	0.59	0.00000894
DPYSL2	0.338	-0.101	0.249	0.824	1.377	4.87E-45
APOBEC3B	0.041	0.346	0.545	1.108	1.076	2.08E-24
CASP1	-0.067	0.361	0.956	1.02	2.191	3.31E-50
TAP1	0.328	0.938	0.922	0.902	1.261	7.24E-59
PNMA2	-0.564	0	0.949	1.353	2.416	1E-135
LIMS1	0.447	0.079	0.132	0.582	1.293	5.11E-79
RPTOR	0.371	0.144	0.256	0.568	0.256	0.0000246
SRGAP1	0.406	0.379	0.706	0.667	1.094	2.48E-40
EPB41L3	0.409	0.219	0.236	0.735	1.416	1.85E-46
CXCR4	-0.328	0.669	1.207	1.198	3.156	5.31E-93
CASP7	-0.045	0.311	0.608	0.652	1.356	9.09E-78
NACAD	0.004	0.49	0.639	0.653	-0.12	4.09E-08
KRT80	0.517	0.456	0.85	1.027	1.128	5.63E-67
KIAA1324	0.237	0.417	0.678	0.982	0.825	3.02E-15
GOLPH3L	-0.114	0.286	0.302	0.574	0.905	1E-30
NEK1	0.054	0.218	0.449	0.713	0.45	3.99E-17
ABCG1	0.263	0.253	0.827	1.352	2.384	7.04E-79

NUDT8	0.126	0.533	0.473	0.571	0.623	0.000846
LAMP3	-0.031	0.198	0.261	0.722	1.102	1.69E-33
EPHA4	0.198	0.74	0.852	0.657	1.331	8.88E-44
INSC	0.355	0.046	0.658	1.137	2.4	2.75E-60
NFE2L3	0.255	0.303	0.337	0.754	1.557	5.82E-72
CREB3L1	0.315	0.176	0.615	0.731	2.932	1.9E-86
CRTAC1	0.187	0.114	0.493	0.649	1.941	9.62E-24
GPX3	0.041	0.074	0.243	0.648	0.833	5.07E-34
NCOR2	0.422	-0.222	0.449	0.736	1.195	6.67E-73
LAMB3	-0.039	0.174	0.222	0.601	1.339	4.72E-28
PBXIP1	0.055	0.118	0.243	0.647	1.783	2.63E-175
LAMC2	0.301	0.138	0.436	0.755	2.528	1.42E-70
PLCL2	0.525	0.696	0.534	0.673	1.406	2.84E-24
GPC1	0.398	0.736	0.852	1.225	1.991	6.94E-83
NF2	-0.097	-0.061	0.187	0.61	1.085	5.19E-79
TRAC	0.368	0.547	1.039	1.497	2.403	5.5E-25
SPINT1	#N/A	#N/A	#N/A	#N/A	#N/A	#N/A
FEZ1	0.235	0.263	0.412	0.748	1.148	2.55E-82
ORAI3	0.17	0.361	0.505	0.685	1.511	3.66E-31
CCNG2	0.178	0.22	0.281	0.637	1.587	5.24E-110
SERPINB1	0.294	0.082	0.217	0.634	0.935	4.05E-35
CCNG1	0.545	0.765	0.763	0.696	1.176	1.5E-80
TRIM21	0.096	0.51	0.669	0.986	1.25	8.98E-51
TCAIM	-0.024	0.252	0.481	0.605	0.664	6.27E-34
ZNF385A	0.36	0.612	0.81	0.941	1.253	1.77E-25
PLA2G4C	0.007	0.712	1.364	1.767	4.326	4.6e-311
NINJ1	0.313	0.499	0.799	1.086	2.05	2.68E-147
RUVBL1	0.314	0.1	0.045	0.599	0.468	3.57E-09
KRT17	0.138	0.682	1.19	1.62	2.149	1.28E-27
HHIPL1	0.219	0.146	0.492	0.708	1.269	9.2E-16
SEMA4B	0.08	0.011	0.428	0.624	1.096	1.68E-30
YPEL1	-0.52	0.145	0.273	0.618	1.859	4.58E-39
KRT18	0.144	0.055	0.283	0.841	1.452	3.5E-65
ASTN2	0.496	1.05	1.878	1.812	2.098	1.11E-35
ACP7	0.224	0.07	0.132	0.613	0.623	0.00000795
GSN	0.352	0.269	0.273	0.788	0.807	5.32E-14
LYNX1	0.408	0.854	1.077	1.456	1.792	2.62E-30
MYRIP	0.555	-0.285	0.38	1.101	1.258	1.15E-31

CYGB	-0.149	-0.006	0.896	0.947	1.658	3.81E-16
ZNF521	0.344	0.045	0.176	0.625	0.794	1.36E-15
SYNC	0.228	0.473	0.854	0.938	1.418	3.94E-50
EHHADH	0.208	0.055	0.21	0.717	0.386	0.000187
EEF2	0.383	0.255	0.243	0.61	0.923	1.97E-16
STAT3	0.427	0.339	0.334	0.604	0.836	2.69E-15
CHMP4B	0.306	0.198	0.197	0.585	1.01	5.9E-45
FAM131B	0.305	0.091	0.171	0.589	0.814	0.0000288
ZNF185	0.345	0.061	0.211	0.699	1.148	1.67E-37
CDA	0.3	0.03	0.464	0.721	1.366	4.62E-08
SQRDL	0.222	0.138	0.254	0.682	0.668	1.11E-16
UBD	#N/A	#N/A	#N/A	#N/A	#N/A	#N/A
EFNB1	0.534	0.379	0.315	0.615	1.415	6.22E-62
STC1	-0.366	-0.237	0.43	0.582	3.786	2.13E-275
MAMDC2	0.369	0.543	0.937	1.402	2.945	0
ELFN2	0.524	0.055	0.386	0.72	0.89	3.36E-09
CD79B	-0.152	0.196	0.407	0.63	0.301	0.000338
ST13P3	0.279	0.66	0.33	0.886	0.957	1.02E-07
ST5	0.056	-0.345	0.181	0.621	0.602	1.27E-11
SLC27A3	-0.576	-0.145	-0.219	-0.718	-1.36	3.04E-50
NEAT1	-0.522	0.314	0.095	-0.802	-0.096	0.00000293
ZNF789	-0.439	-0.098	-0.012	-0.815	-0.799	4.17E-09
DNAJC19	-0.507	-0.323	-0.145	-0.67	-0.755	2.25E-08
PLCXD1	-0.263	-0.128	-0.233	-1.002	-0.733	2.88E-24
ZGRF1	-0.009	0.182	-0.308	-0.76	-0.948	3.82E-42
SNORA33	-0.087	0.381	0.102	-0.57	-0.693	0.0000099
SNORD100	-0.33	0.275	0.058	-0.874	-0.894	0.00000705
FANCB	-0.122	-0.093	-0.481	-0.77	-1.134	1.42E-25
SNORD101	-0.028	0.201	0.373	-0.774	-0.916	3.61E-08
SLC22A31	-0.119	-0.109	-0.213	-0.612	-1.353	6.47E-08
RPL37P2	-0.388	-0.364	-0.129	-0.661	-0.643	0.00401
DND1	-0.193	-0.096	0.05	-1.073	-0.854	0.00000054
GDPD3	-0.439	-0.121	0.286	-0.71	-0.559	1.84E-08
SLCO2A1	0.228	-0.284	-0.858	-0.886	-2.54	9.91E-99
NOL12	-0.554	-0.181	-0.117	-0.699	-0.982	3.39E-18
LSMEM1	0.167	0.043	0.272	-0.658	-0.586	0.000227
ZNF121	-0.066	0.279	0.173	-0.697	-0.578	3.1E-22
ATP5EP2	-0.448	-0.274	-0.141	-0.594	-0.527	0.0197

MIR4691	-0.455	0	-0.189	-0.979	-0.77	0.00121
DERL3	-0.194	-0.573	-0.567	-0.977	-1.947	1.03E-15
RN7SL431P	0.181	-0.112	0.094	-0.655	-0.537	0.011
ACAT2	0.214	-0.227	-0.528	-0.654	-0.796	1.7E-57
CACNG8	-0.361	-0.238	-0.001	-0.937	-1.149	8.65E-19
SNHG15	-0.37	-0.162	-0.191	-0.944	-1.189	7.54E-25
USP32P1	-0.482	0.07	0.173	-0.772	-0.522	0.0000112
POU4F1	-0.29	-0.177	-0.275	-0.829	-0.411	1.44E-10
KMT2B	-0.324	-0.067	-0.125	-0.674	-0.597	5E-22
AGAP6	-0.469	0.048	0.025	-0.941	-0.847	2.5E-14
PKD1P6	-0.527	-0.336	-0.311	-1.226	-0.558	1.48E-10
HSF4	-0.403	0.014	0.306	-0.654	-0.423	0.00000693
NDUFAF5	-0.282	-0.165	-0.145	-0.707	-0.529	0.00000043
MPP3	-0.163	-0.112	0.132	-0.647	-0.891	1.04E-12
AP3M2	-0.246	-0.342	-0.252	-0.593	-0.295	0.00000249
MIR1304	-0.378	0.071	0.032	-1.143	-1.197	5.16E-19
UBE2SP1	-0.221	-0.65	-0.937	-0.703	-0.598	9.9E-19
DGCR8	-0.006	-0.183	-0.344	-0.892	-0.676	2.76E-97
GPR75	-0.081	0.214	0.264	-0.684	-0.279	0.00000903
GFOD1	0.346	-0.311	-0.484	-0.665	-0.604	1.94E-41
LRP5L	-0.403	-0.201	-0.071	-0.939	-0.791	7.44E-07
POLE	0.123	0.021	-0.303	-0.845	-1.109	2.46E-110
FGF12	-0.173	-0.248	-0.94	-0.944	-1.232	2.18E-15
NPIPB11	-0.322	-0.104	0.046	-0.962	-0.669	2.61E-07
PTPDC1	-0.167	-0.165	-0.191	-0.586	-0.527	0.00000202
SIRT7	-0.512	-0.045	-0.141	-0.621	-0.596	3.48E-14
CEBPD	-0.047	-0.125	-0.635	-0.682	-0.16	0.0000682
ZNF354B	-0.298	0.186	-0.076	-0.652	-0.416	0.0000767
MEG3	-0.45	0.013	0.208	-0.875	-0.415	2.94E-12
SMARCD3	0.012	-0.269	-0.374	-0.807	-0.987	4.54E-13
NDOR1	-0.418	-0.011	-0.132	-0.721	-0.805	9.77E-29
TSPYL2	-0.155	0.019	0.002	-0.632	-0.747	1.56E-25
ZNF10	-0.073	0.124	0.045	-0.613	-0.345	0.00041
ADGRL1	0.349	-0.028	-0.413	-0.627	-0.937	1.24E-17
SPACA6	-0.304	-0.276	-0.15	-0.878	-0.443	0.000168
OCLM	-0.418	-0.167	-0.362	-0.883	-0.69	0.0235
NT5DC3	-0.012	-0.197	0.086	-0.672	-0.784	5.13E-20
SNAPC4	-0.501	-0.007	-0.101	-0.663	-0.109	1.57E-12

TMCO6	-0.504	-0.111	-0.082	-0.608	-1.05	1.88E-40
SFI1	-0.385	-0.2	-0.035	-0.62	-0.755	1.18E-17
SMO	0.004	0.067	-0.605	-0.842	-0.569	0.00000113
SNORD104	-0.276	0.199	0.293	-0.897	-0.954	1.89E-09
SRSF7	0.137	-0.192	-0.332	-0.614	-0.664	6.18E-50
GHRLOS	-0.561	0.05	0.125	-1.33	-0.929	2.22E-10
HMGCR	0.423	-0.344	-0.706	-0.597	-0.022	1.03E-82
ERCC6	-0.397	-0.15	-0.389	-0.599	-1.726	7.18E-92
ADM	-0.582	-0.092	-0.895	-1.161	-1.681	6.89E-41
SGSM2	-0.472	-0.082	0.005	-0.662	-0.149	2.65E-09
MTCP1	-0.242	0.333	0.149	-0.691	-0.466	1.03E-07
LTB4R	-0.522	0.214	0.367	-0.65	-0.727	5.79E-18
PRKD1	-0.135	-0.204	-0.372	-0.749	-0.338	2.04E-10
AFG3L1P	-0.258	0.064	0.065	-0.875	-1.008	4.79E-25
ZFAS1	-0.315	-0.156	-0.047	-0.712	-0.635	0.00000258
PKD1P1	-0.182	0.011	0.023	-0.585	-0.151	0.00443
NR2C2AP	0.14	-0.088	-0.495	-0.731	-0.974	2.29E-35
DZIP1L	-0.239	-0.02	-0.127	-0.85	-1.026	1.23E-18
XRCC2	0.249	-0.028	-0.37	-0.787	-0.903	1.45E-23
XRCC3	-0.344	-0.056	-0.22	-0.628	-1.034	1.29E-28
UBE2S	-0.442	-0.657	-0.909	-0.886	-0.253	1.3E-26
INTS6L	-0.148	0.177	0.102	-0.741	-0.796	8.98E-11
SCARF2	0.024	-0.077	-0.251	-0.73	-0.164	1.46E-07
TTLL3	-0.522	-0.133	0.202	-0.714	-0.347	2.63E-09
PAQR4	0.249	-0.134	-0.571	-0.691	-1.473	1.27E-88
SRSF2	-0.001	-0.057	-0.263	-0.633	-0.529	1.01E-29
TBC1D4	-0.03	-0.1	-0.386	-0.706	-1.055	1.02E-54
CIRBP	-0.251	0.087	-0.107	-0.598	-0.923	1.41E-31
WEE1	-0.493	-0.498	-0.554	-0.643	-0.618	1.25E-19
C16orf59	-0.268	-0.145	-0.284	-0.689	-1.097	6.31E-29
MVD	0.43	-0.169	-0.504	-0.637	-0.292	4.35E-29
GINS2	-0.074	-0.23	-0.534	-1.023	-1.288	1.54E-50
SNRPGP2	-0.3	-0.096	-0.092	-0.544	-0.688	0.0184
WDR90	-0.436	-0.002	-0.128	-1.049	-1.35	6.54E-36
PER2	0.251	-0.658	-1.325	-1.948	-1.226	1.24E-76
GOLGA8A	-0.433	0.049	0.235	-0.702	-0.34	4.54E-11
LIN7B	0.016	-0.422	-0.135	-0.868	-1.097	3.21E-08
PTBP2	-0.21	-0.007	-0.047	-0.683	-0.449	0.0000012

CBX2	0.183	0.04	-0.419	-0.623	-0.339	8.87E-17
ELMOD1	0.324	-0.695	-1.255	-1.812	-3.173	4.53E-94
SLC5A3	-0.465	0.034	-0.284	-0.63	-0.204	2.1E-09
GPR173	-0.344	-0.123	-0.054	-0.772	-0.482	9.65E-11
DLEU2	-0.552	-0.445	-0.232	-0.765	-1.357	2.4E-16
KIAA0895L	-0.296	0.002	-0.022	-1.026	-0.833	1.9E-17
CLCN6	-0.325	-0.043	-0.07	-0.685	-0.313	2.51E-10
ZC3H12A	0.091	0.235	-0.069	-0.592	0.064	2.78E-09
ATXN7L2	-0.124	-0.02	-0.277	-0.689	-0.805	2.54E-12
FANCA	-0.387	0.035	-0.073	-0.766	-1.489	2.39E-66
CENPT	-0.472	-0.071	-0.077	-0.633	-0.723	6.49E-19
MIR3916	-0.421	-0.003	0.04	-0.997	-0.506	1.01E-07
SNORA25	-0.236	-0.045	0.091	-0.827	-0.925	2.37E-07
CLK2	-0.455	0.07	0	-0.827	-0.949	1.13E-28
MIR3682	0.361	0.03	0.187	-0.68	-0.092	0.0172
RPL32P3	-0.454	-0.011	0.164	-0.576	-0.296	0.000088
EZH2	-0.138	-0.136	-0.261	-0.737	-1.018	2.71E-28
CEP152	-0.289	0.092	0.019	-0.661	-1.049	7.7E-31
MIR6845	-0.551	-0.057	0.261	-0.858	-0.683	0.000436
IMMP1L	-0.543	-0.389	-0.156	-0.622	-0.883	0.0000256
LIMCH1	0.126	-0.23	-0.917	-1.293	-1.819	7.77E-34
MIR3653	-0.371	0.35	0.292	-0.669	-0.735	4.41E-07
HES6	-0.508	-0.49	-0.764	-0.877	-0.259	0.0000213
NFKBIZ	0.182	0.354	0.25	-0.798	0.237	1.4E-10
KMT5C	-0.36	0.213	0.012	-0.601	-0.371	1.66E-07
SNORA4	-0.329	0.214	-0.082	-0.739	-0.999	0.00000511
SNORA41	0.158	0.016	0.225	-0.74	-0.685	0.00101
ITGB1BP2	-0.523	0.008	-0.014	-0.975	-1.203	0.00000097
RPL37P23	-0.424	-0.312	-0.17	-0.639	-0.552	0.000196
SNORD96A	-0.295	0.055	0.09	-0.748	-0.956	1.2E-08
SCML1	-0.149	-0.2	-0.201	-0.639	-0.754	4.7E-12
FOXD2-AS1	-0.351	-0.289	-0.272	-0.684	-0.737	0.00499
ADAT2	-0.431	-0.029	0.027	-0.636	-0.957	4.29E-17
MAT2A	-0.304	-0.214	-0.17	-0.676	-0.461	1.57E-17
ZNF625	-0.198	0.052	-0.265	-0.795	-0.744	0.00224
SCD	0.525	-0.157	-0.66	-0.657	0.098	5.8E-42
SNORA52	-0.382	-0.031	-0.275	-1.095	-1.314	7.09E-09
MSMO1	0.227	-0.503	-0.949	-1.014	-0.138	2.69E-59

COX7A2P2	-0.473	-0.308	-0.111	-0.664	-0.74	0.0000536
SATB1	-0.002	-0.11	-0.296	-0.766	-0.446	0.0000601
SNORD2	-0.153	0.041	0.066	-1.007	-1.117	5.19E-10
COX7A2	-0.418	-0.191	-0.059	-0.593	-0.657	0.00151
AMH	-0.33	0.016	-0.482	-1.649	-1.757	2.66E-20
CCDC78	-0.152	0.062	0.051	-0.801	-0.757	0.000428
SNHG1	-0.081	-0.127	-0.187	-1.043	-1.406	1.09E-32
CRY1	0.172	0.01	-0.333	-0.647	-0.284	2.78E-39
NPPA-AS1	-0.384	0.08	-0.052	-1.264	-1.082	3.67E-12
MAPK12	-0.209	-0.107	-0.207	-0.7	-0.784	1.93E-34
TIMM10	-0.437	-0.506	-0.459	-0.626	-1.018	3.44E-23
MZT1	-0.468	-0.473	-0.502	-0.799	-0.876	1.21E-10
ZNF700	-0.352	0.08	-0.162	-0.676	-0.639	1.09E-13
EIF5A2	0.158	-0.163	-0.53	-0.791	-0.592	5.52E-26
PER1	-0.223	-0.209	-0.479	-0.751	-1.218	1.32E-35
UBAP1L	-0.127	-0.023	-0.004	-1.089	-0.759	0.0000379
EP400NL	-0.384	0.07	0.08	-0.684	-0.661	1.81E-13
CASP8AP2	0.241	0.054	-0.27	-0.614	-0.495	3.06E-45
UNG	0.557	-0.172	-1.008	-0.914	-0.561	2.53E-80
HDAC10	-0.531	-0.188	-0.062	-0.705	-0.43	0.00000329
MAN2C1	-0.328	-0.023	0.062	-0.594	-0.493	1.59E-10
NPIPA3	-0.179	0.235	0.349	-0.674	-0.697	0.000164
HNRNPDL	0.047	0.034	-0.152	-0.737	-0.757	3.46E-27
PFDN4	-0.328	-0.041	-0.123	-0.702	-0.573	0.0000531
CRNDE	-0.443	0.052	-0.132	-0.635	-0.385	0.00191
TAF1C	-0.332	-0.148	-0.224	-0.937	-0.977	3.57E-23
ZNF684	-0.37	-0.243	-0.477	-0.639	-0.92	5.04E-07
MDM4	-0.451	-0.059	0.178	-0.702	-0.82	5.82E-13
CDCA7	0.051	-0.43	-1.028	-1.225	-0.901	1.77E-81
INPP5E	-0.278	-0.081	-0.11	-0.629	-0.317	1.6E-09
MAP3K14	-0.144	-0.171	-0.249	-0.702	-0.357	5.73E-09
HIST2H2BB	#N/A	#N/A	#N/A	#N/A	#N/A	#N/A
MEIS2	-0.369	0.009	-0.681	-0.646	-0.808	8.12E-17
SLX1B-SULT	-0.138	0.229	0.054	-0.735	-0.376	0.0245
USMG5	-0.405	-0.159	-0.028	-0.655	-0.512	0.00821
ORC6	0.22	-0.2	-0.572	-0.876	-1.053	2.93E-64
DNAJC27	-0.509	-0.195	-0.201	-0.718	-0.801	4.03E-08
RNU1-103P	0.38	-0.149	0.019	-0.595	-0.356	0.0325

CLEC1B	-0.456	-0.306	-0.594	-0.929	-2.082	5.73E-14
MIR93	0.198	-0.121	-0.106	-0.679	-0.079	0.00769
ZSCAN31	-0.476	-0.382	-0.675	-0.804	-0.106	1.53E-07
WASH2P	-0.335	-0.166	0.008	-0.681	-0.492	0.0104
KRTCAP3	-0.037	-0.13	0.128	-0.833	-0.179	0.013
NFKBID	-0.118	0.032	-0.163	-1.01	-1.057	5.38E-19
AMZ2P1	-0.53	-0.031	-0.06	-0.642	-0.766	5.51E-07
ATAD3B	-0.179	0.002	-0.231	-0.739	-0.666	4.24E-21
FSD1L	0.245	0.032	0.177	-0.582	-0.353	0.00000676
USMG5P1	-0.439	-0.258	-0.076	-0.696	-0.561	0.0191
GAB1	-0.063	0.358	-0.22	-0.634	0.647	2.16E-39
GABBR1	-0.403	0.114	0.259	-0.686	-0.713	3.99E-17
CROCCP2	-0.404	0.127	0.115	-0.739	-0.663	4.65E-15
ZNF117	-0.387	0.062	0.153	-0.784	-0.562	6.33E-11
SCX	0.176	-0.432	-0.367	-0.85	0.181	0.00135
IQCC	-0.412	-0.131	-0.701	-0.657	-1.428	1.45E-22
IDI1	0.31	-0.401	-0.877	-1.05	-0.374	1.68E-59
SUGP2	-0.468	-0.077	-0.041	-0.812	-0.645	2.74E-15
TUB	-0.131	-0.281	-0.649	-1.038	-1.371	2.88E-31
KCNAB3	-0.295	0.114	0.264	-0.793	-1.09	6.63E-08
FAM69B	0.003	-0.429	-0.615	-0.7	-0.418	5.13E-39
CLASRP	-0.194	-0.026	-0.071	-0.625	-0.685	1.18E-16
TMEM200B	0.31	-0.278	-0.824	-1.003	-0.752	2.36E-11
CSAD	-0.459	0.02	0.23	-0.752	-0.724	7.42E-16
LINC00472	-0.552	-0.234	-0.301	-0.888	-0.902	0.00000755
RTKN2	-0.557	-0.36	-0.295	-0.661	-0.889	1.52E-11
MIR6753	-0.099	0.157	0.027	-0.683	-0.76	0.00398
RPL37	-0.34	-0.188	-0.096	-0.623	-0.515	0.000152
MIS12	-0.206	-0.071	-0.116	-0.6	-0.931	2.61E-27
PLA2G4A	0.372	0.097	-0.555	-0.901	-3.052	1.07E-123
RPL21	-0.109	-0.072	0.059	-0.637	-0.775	1.24E-10
PAQR6	-0.16	0.424	0.107	-0.619	-0.87	5.78E-07
MFSD2A	-0.369	-0.355	-0.825	-0.959	-1.121	1.72E-21
CTRL	-0.446	0.01	0.106	-0.737	-0.597	0.00101
MSS51	-0.196	-0.2	0.417	-0.668	-0.676	0.000004
ART4	0.528	0.534	-0.303	-0.792	-1.85	1.69E-105
CRYGS	-0.339	-0.087	0.189	-1.063	-0.894	1.25E-08
LRRC17	0.302	-0.288	-0.725	-0.767	1.759	2.61E-71

MIR22HG	-0.155	0.097	-0.061	-0.969	-0.886	1.43E-21
TAF1D	-0.12	0.094	0.061	-0.573	-0.537	2.16E-08
ATAD5	0.411	0.168	-0.272	-0.683	-1.088	1.03E-75
PKDCC	-0.28	-0.217	-0.582	-1.144	-1.083	2.52E-10
SETD6	-0.301	-0.361	-0.002	-0.617	-0.345	0.00147
TMEM136	-0.447	-0.03	0.01	-0.638	-0.349	1.5E-08
CRY2	0.095	0.069	-0.529	-1.183	-0.82	6.5E-51
FASTKD3	-0.264	-0.14	-0.185	-0.624	-0.743	2.89E-07
KLRG1	-0.165	-0.796	-0.764	-1.175	-1.477	1.21E-07
DOK3	-0.435	-0.039	-0.273	-1.384	-0.82	1.58E-15
PKD1	-0.357	-0.347	-0.111	-0.81	-0.247	5.85E-08
KCTD13	-0.494	-0.056	0.067	-0.851	-0.346	7.22E-12
TMSB15A	-0.4	-0.179	-0.164	-0.725	-0.613	0.00628
RELT	0.084	-0.032	-0.365	-0.883	-0.491	4.88E-21
TEN1-CDK3	0.022	0.223	0.143	-0.674	-0.444	0.000654
GULP1	-0.262	-0.265	-0.671	-0.942	-1.418	2.62E-67
RPS28	-0.32	-0.156	-0.244	-0.84	-0.542	0.000117
ZMYND19	0.002	-0.053	-0.509	-0.783	-0.594	1.04E-38
CORO6	-0.327	0.239	0.139	-0.616	0.087	0.000867
STARD4	0.203	-0.103	-0.297	-0.69	-0.564	1.39E-20
ATP2B1	0.254	0.017	-0.591	-0.666	-0.738	2.69E-45
UNC119	-0.445	-0.145	-0.091	-0.631	-0.147	2.53E-09
RNA5SP383	-0.066	0.301	0.336	-0.658	-0.696	0.000347
C1orf54	-0.413	-0.215	-0.23	-0.631	0.245	0.0000239
GAS5	-0.32	0.04	0.117	-0.681	-0.383	0.00017
RAPGEFL1	-0.241	0.081	-0.019	-0.907	-0.362	3.59E-08
AGAP9	-0.307	-0.315	-0.066	-0.751	-0.976	0.0000209
FIRRE	-0.417	-0.098	-0.083	-0.661	-0.471	0.0156
BOLA3	-0.293	-0.198	-0.263	-0.586	-0.904	5.35E-12
SNORD11	-0.127	-0.279	-0.375	-0.69	-0.865	0.0165
FAM118A	-0.408	-0.034	0.11	-0.713	-0.544	2.55E-15
FAM76B	0.081	0.122	-0.102	-0.627	-0.512	2.76E-13
DDX11	-0.215	-0.142	-0.22	-0.887	-1.496	3.23E-85
ROGDI	-0.385	-0.12	-0.199	-0.661	-0.171	6.41E-08
SNORA32	-0.348	0.197	0.062	-0.796	-1.006	8.61E-10
GABPB1-AS	-0.427	0.008	0.432	-0.687	-0.255	2.44E-09
C1D	-0.293	-0.117	-0.065	-0.655	-0.812	0.0000132
C8orf59	-0.367	-0.161	-0.099	-0.686	-0.659	0.0000205

RBM14	0.334	0.189	-0.38	-0.621	-0.225	4.86E-15
SNORD6	-0.227	-0.165	-0.026	-0.863	-0.89	0.00131
AADAT	-0.093	0.09	-0.087	-0.739	-0.185	0.0000506
SNORA16A	-0.23	0.083	0.119	-0.66	-1.172	6.59E-10
SNORD5	-0.556	0.014	-0.005	-1.042	-1.029	7.14E-11
EGFL7	-0.533	-0.363	-0.366	-0.746	-1.174	9.46E-40
ATP5E	-0.403	-0.176	-0.07	-0.595	-0.48	0.0189
PRPF39	-0.176	0.112	-0.023	-0.767	-0.401	1.08E-09
DACH1	-0.26	-0.148	-1.007	-1.05	0.805	2.02E-28
CCDC189	-0.452	-0.147	-0.37	-1.062	-0.902	2.82E-07
RASGRP3	0.081	-0.12	-0.218	-0.657	0.012	8.73E-10
SNORD23	0.051	0.101	-0.258	-0.699	-0.586	0.0221
SNORD50B	-0.042	0.154	0.029	-0.776	-0.869	0.0000029
SNORD62B	-0.205	0.023	0.189	-0.798	-0.265	0.0182
CMC2	-0.315	-0.307	-0.234	-0.578	-1.172	1.54E-14
SNORD17	-0.113	-0.005	-0.27	-0.671	-0.699	0.00152
MIRLET7D	-0.49	-0.002	-0.234	-0.85	-0.542	0.0292
SNORD45C	-0.159	0.196	0.244	-0.693	-0.92	0.00000465
C9orf72	0.128	-0.138	-0.219	-0.744	-0.893	5.18E-13
DPF1	-0.029	0.066	-0.332	-0.648	-1.749	3.39E-47
TMSB4XP4	-0.31	-0.437	-0.429	-1.06	-0.345	0.000279
ATP8A1	-0.246	-0.037	-0.451	-0.635	-1.667	8.62E-31
ZNF467	-0.55	-0.833	-0.506	-0.71	-1.783	2.69E-27
MIR2116	-0.1	-0.275	-0.406	-0.681	-0.58	0.0433
MSANTD2	-0.206	-0.094	-0.036	-0.673	-0.541	4.57E-09
CCDC84	-0.251	0.046	0.129	-0.582	-0.545	4.08E-07
MIR4321	-0.132	-0.335	-0.277	-0.654	-0.662	0.00269
SNORD22	-0.057	0.226	-0.06	-0.954	-1.317	8.22E-14
GNRH1	-0.19	0.075	0.041	-0.766	-0.766	0.0000306
PRR22	-0.154	0.385	-0.087	-0.782	-1.034	3.14E-10
SNORD26	-0.222	0.011	-0.093	-0.733	-0.84	0.00855
SNORD27	0.289	0.207	0.117	-0.585	-0.87	0.00000675
SNORD28	0.02	-0.141	-0.067	-0.976	-1.261	4.19E-12
AGAP4	-0.523	-0.074	0.038	-0.932	-0.495	3.27E-12
MIR3176	-0.57	-0.121	-0.356	-1.576	-1.001	9.14E-09
SNORD30	-0.335	-0.188	-0.34	-1.272	-1.659	4.25E-15
UBE2T	-0.454	-0.529	-0.534	-0.942	-1.523	1.91E-27
CCT6P3	-0.525	-0.022	0.047	-0.805	-0.437	0.000079

HMGN3	-0.42	-0.335	-0.239	-0.585	-0.656	0.00000241
ZNF714	-0.005	-0.201	-0.361	-0.596	-0.168	0.000548
AKAP17A	-0.261	0.157	0.073	-0.614	-0.509	3.73E-15
MEG9	-0.26	0.142	0.243	-0.871	-0.445	2.85E-09
GIGYF1	-0.31	0.133	0.093	-0.721	-0.598	1.25E-17
NPIPB5	-0.173	0.092	0.052	-0.697	-0.545	0.00308
NBPF14	-0.365	-0.205	0.143	-0.685	-0.007	0.00108
SCDP1	0.322	-0.482	-0.778	-0.63	-0.092	2E-14
CYP1A1	0.244	-0.383	-0.528	-0.712	-1.625	2.74E-17
SNHG12	-0.081	0.113	0.097	-0.971	-1.125	1.59E-21
COMMD8	-0.356	-0.221	-0.186	-0.577	-0.98	2.31E-09
GPR135	-0.442	-0.11	-0.031	-1.044	-0.976	2.95E-08
BCL2L2	-0.384	0.066	-0.039	-0.73	-0.561	3.02E-12
JAG2	0.454	-0.321	-0.637	-0.732	-1.002	9.08E-49
MIR5587	-0.479	-0.189	-0.031	-1.134	-1.207	0.00000163
MROH6	-0.107	0.26	0.094	-0.725	-0.507	0.0000764
CHRNA10	-0.22	-0.118	-0.072	-0.834	-0.574	0.0126
DMPK	-0.125	0.16	0.263	-0.603	-0.067	1.14E-07
USP37	0.492	0.152	-0.257	-0.59	-0.725	1.65E-54
CCT6P1	-0.349	0.05	0.082	-0.694	-0.394	0.0000554
CACHD1	0.158	-0.094	-0.647	-0.589	-0.74	1.46E-21
SNORA73A	-0.256	0.097	0.088	-0.904	-1.395	4.33E-18
ELMO3	-0.041	-0.156	-0.098	-1.035	-1.227	2.77E-23
RSRP1	-0.384	-0.006	0.049	-0.824	-0.494	0.0000224
LONRF1	0.125	0.305	-0.115	-0.622	-0.308	1.6E-09
SNORD32A	#N/A	#N/A	#N/A	#N/A	#N/A	#N/A
ITGAE	-0.466	-0.183	-0.025	-0.588	-0.667	0.000112
MCTP1	-0.035	0.126	-0.424	-0.728	-1.369	3.25E-61
GMNN	0.042	-0.272	-0.614	-0.711	-1.376	1.4E-82
CCNL2	-0.454	0.205	0.195	-0.716	-0.548	7.2E-09
SNORD35A	-0.18	-0.226	-0.148	-0.856	-1.038	0.000172
SNORD14A	0.001	0.277	0.156	-0.629	-1.149	6.07E-08
MIR21	0.359	0.315	0.41	-0.746	-0.092	0.000262
PDXP	#N/A	#N/A	#N/A	#N/A	#N/A	#N/A
SNORD84	-0.218	0.057	-0.117	-0.728	-0.456	0.0293
GOLT1B	-0.102	-0.015	-0.055	-0.576	-0.615	7.13E-07
PAM16	-0.455	-0.275	-0.11	-0.8	-0.82	4.62E-08
SNORD88A	-0.394	-0.335	-0.66	-1.089	-0.883	0.00234

SNORD88B	-0.117	-0.295	-0.241	-1.158	-1.353	2.5E-09
SNHG3	-0.156	-0.126	0.024	-0.88	-1.03	1.38E-16
SNORD86	-0.457	-0.421	-0.266	-1.518	-1.325	1.58E-19
SNORA81	-0.007	-0.044	0.07	-0.765	-0.879	0.000128
SNORA27	-0.406	0	-0.255	-1.038	-0.947	0.000187
DLG4	-0.57	-0.16	-0.145	-0.619	-0.474	6.13E-16
GABRE	-0.292	0.103	0.22	-0.771	0.023	0.00000259
ABHD3	-0.236	0.041	-0.021	-0.85	-1.361	8.39E-29
SEC61G	-0.286	-0.167	-0.038	-0.601	-0.998	4.59E-07
DLX1	0.252	-0.597	-0.752	-0.78	-0.656	7.58E-09
SNORA61	-0.226	0.053	0.027	-1.161	-1.147	4.74E-18
SNORA53	-0.378	0.096	-0.124	-0.915	-0.778	0.00159
SNORA48	-0.178	0.089	0.044	-0.849	-0.788	0.00000482
MIR25	-0.077	-0.278	-0.422	-1.215	-0.608	0.0000242
SNORD110	-0.224	0.065	-0.149	-1.257	-1.151	6.21E-12
SNORD10	-0.466	-0.256	-0.266	-0.91	-0.572	0.00907
SNORD99	0.074	0.033	0.095	-0.874	-0.972	2.92E-07
RECQL4	-0.203	0.018	-0.203	-0.836	-1.196	4.05E-39
RAD54L	0.082	0.037	-0.253	-0.813	-1.474	2.88E-94
SNORA51	-0.25	-0.391	-0.139	-0.895	-1.103	0.0000711
SNORA44	0.068	0.099	0.15	-0.911	-0.769	0.00000277
ADCK5	-0.565	-0.032	-0.138	-0.739	-0.705	2.36E-10
PPP1R3E	-0.562	-0.153	-0.119	-0.757	-0.935	7.17E-12
SDHAP1	-0.199	0.135	0.044	-0.92	-0.793	2.05E-13
SNORA70	-0.203	-0.078	0.07	-0.811	-0.903	2.61E-07
IL20RB	-0.145	0.121	-0.057	-0.679	-0.773	0.0021
SNORA40	-0.133	0.124	0.125	-0.623	-0.845	2.73E-11
CENPV	-0.064	-0.233	-0.257	-0.603	-0.188	0.000992
SNORA69	-0.138	-0.136	-0.179	-1.017	-0.754	0.000845
SVIP	-0.329	-0.221	-0.275	-0.613	-0.528	0.00014
SNORA71B	-0.421	-0.205	-0.114	-0.859	-0.961	0.00119
HERC2P4	-0.538	-0.067	-0.175	-1.102	-1.029	9.25E-13
SNORD4A	-0.374	-0.019	-0.042	-1.045	-1.071	0.00000276
SNORA62	-0.205	0.336	0.242	-0.725	-0.896	2.42E-07
SNORA24	-0.268	-0.187	-0.205	-0.724	-1.318	0.00000644
TSTD1	-0.461	-0.345	-0.209	-0.672	-0.369	0.00367
SNORA31	-0.464	0.08	-0.035	-0.699	-0.848	0.00433
SNORD102	-0.343	-0.049	0.034	-0.817	-1.212	6.47E-08

SNORA73B	0.059	0.181	0.172	-0.578	-1.016	3.24E-08
SNORD65	-0.15	-0.06	0.133	-0.671	-0.838	0.000296
UBE2SP2	-0.313	-0.687	-0.955	-0.84	-0.797	2.88E-10
DHCR24	0.205	-0.324	-0.75	-0.869	-0.496	8.12E-46
FTX	-0.364	0.177	0.152	-0.597	-0.557	9.06E-22
SNORA18	-0.119	0.037	0.095	-0.8	-0.867	4.77E-11
PPIEL	-0.384	-0.127	0.141	-0.749	-0.505	0.000515
SNORA2A	-0.189	-0.441	-0.01	-0.63	-0.852	0.0165
MFSD7	-0.423	-0.017	-0.038	-0.662	-0.506	0.00204
EID3	0.018	0.198	-0.011	-0.73	-0.706	0.000499
SNORA1	-0.157	0.054	-0.026	-0.817	-0.858	0.0000452
PARP6	-0.494	-0.117	-0.013	-0.655	-0.332	9.14E-08
SPRY1	-0.167	0.079	-0.368	-0.897	-1.087	2.32E-43
TMEM50B	-0.087	-0.233	-0.189	-0.698	-0.655	7.76E-12
C14orf132	0.271	-0.15	-0.442	-0.656	-0.807	0.000025
SNORD36C	-0.208	-0.156	0.076	-0.796	-0.807	0.00303
TEF	0.197	0.085	-0.402	-0.952	-0.021	1.19E-13
CCNL1	-0.307	0.12	0.011	-0.757	-0.584	1.27E-11
SNORD36A	-0.476	-0.004	-0.132	-1.297	-1.03	7.27E-13
HCN3	-0.135	-0.274	0.035	-0.749	-0.713	0.0000828
MMD	0.238	0.12	-0.42	-0.767	-0.83	1.75E-31
SNORD45B	-0.027	0.356	0.371	-0.78	-0.816	4.4E-11
SNORD45A	-0.321	0.147	0.075	-0.914	-0.939	2.48E-07
SCARNA12	-0.407	-0.035	-0.429	-1.162	-1.249	2.75E-08
GOLGA8H	-0.467	-0.116	0.188	-0.755	-0.722	0.0000438
FREM3	0.033	-0.273	-0.822	-1.49	-3.147	5.52E-63
TIMM23B	-0.003	0.091	-0.043	-0.635	-0.671	3.23E-16
SNORD43	-0.314	-0.005	0.024	-0.996	-1.287	2.04E-08
SNORD49A	-0.327	-0.125	0.154	-0.768	-0.913	0.0000188
NPTN-IT1	-0.414	-0.107	-0.109	-1.238	-0.837	8.75E-11
SNHG4	-0.54	-0.256	-0.035	-0.888	-0.649	5.14E-08
PDIA2	-0.097	-0.173	-0.522	-1.21	-0.776	0.00000045
NR2F1	-0.389	-0.33	-0.666	-0.717	-1.919	7.98E-67
PCDH7	0.269	-0.415	-1.112	-1.081	-2.279	4.72E-45
SNORD58C	-0.518	0.042	-0.059	-0.998	-0.906	0.0000046
SNORD56	-0.059	0.071	0.054	-0.784	-0.851	0.000942
SNORD57	-0.286	-0.288	-0.2	-0.963	-1.155	0.000212
PCDH9	-0.139	-0.191	-0.977	-0.716	-0.725	8.72E-21

ZSWIM5	0.38	-0.215	-0.832	-0.925	-1.594	1.03E-48
SNORD54	0.098	0.223	0.101	-0.646	-0.637	0.00104
SNORA57	-0.295	0.05	0.221	-0.944	-0.993	0.00000209
SNORD58A	-0.009	0.146	0.238	-0.72	-0.805	0.000076
SNORA64	-0.243	-0.164	-0.049	-0.837	-0.813	0.00722
C7orf43	-0.375	0.177	-0.13	-0.725	-0.47	3.08E-16
ZNF169	-0.287	-0.17	0.045	-0.584	-0.462	0.0012
MRI1	-0.479	-0.065	-0.065	-0.655	-0.644	5.04E-18
OSTC	-0.253	-0.173	-0.18	-0.588	-0.685	0.00000311
MIR589	#N/A	#N/A	#N/A	#N/A	#N/A	#N/A
MIR590	0.1	0.413	0.433	-0.644	-0.415	0.0000938
CAPZA2	-0.23	-0.255	-0.383	-0.755	-1.019	1.2E-18
LY6G5B	0.189	0.158	0.239	-0.905	-0.493	2.27E-12
ULK3	-0.26	0.031	-0.067	-0.614	-0.726	8.02E-25
FAM117A	0.223	-0.319	-0.633	-0.608	-1.603	2.92E-45
ABRACL	-0.083	-0.076	-0.174	-0.66	-0.624	1.58E-07
SNORD12B	-0.031	0.038	0.08	-0.851	-0.615	0.00198
LENG8	-0.521	-0.015	0.11	-0.827	-0.505	6.26E-12
DGKD	-0.156	0.281	-0.06	-0.624	-0.365	2.13E-35
CTHRC1	0.171	-0.192	-0.497	-0.653	0.568	2.34E-35
VARS2	-0.454	-0.161	0.005	-0.653	-0.979	1.54E-31
SMYD4	-0.398	-0.136	-0.283	-0.595	-0.681	7.47E-17
SS18L1	0.205	0.198	0.062	-0.633	-0.63	1.41E-15
RPS15AP24	#N/A	#N/A	#N/A	#N/A	#N/A	#N/A
PRADC1	-0.246	-0.393	-0.494	-0.651	-1.355	2.26E-48
FAM219B	-0.499	0.003	0.036	-0.608	-0.289	2.24E-08
NOTCH2NL	#N/A	#N/A	#N/A	#N/A	#N/A	#N/A
SLC25A33	-0.248	-0.204	-0.448	-0.668	-0.428	0.0000111
SYDE1	-0.395	-0.135	-0.352	-0.663	-0.493	7.09E-19
SNORD68	-0.126	0.136	0.131	-0.713	-0.734	0.00209
EEF1E1	-0.286	-0.207	-0.338	-0.915	-1.538	3.28E-27
CCDC130	-0.434	0.158	-0.004	-0.771	-0.857	1.57E-37
SNORA13	0.002	0.117	-0.104	-0.57	-0.884	0.00395
MBOAT1	-0.241	0.075	0.094	-0.682	-1.08	4.65E-11
C9orf142	-0.25	-0.138	-0.189	-0.597	-1.128	4.58E-40
SNORA8	-0.416	0.064	0.11	-0.945	-0.927	6.09E-15
BTBD19	0.17	0.025	0.32	-0.584	0.021	0.0000925
KIF1C	-0.409	-0.173	-0.018	-0.597	-0.343	0.0000996

AQP7P1	-0.507	-0.031	0.181	-0.562	-0.855	0.000693
TMEM100	0.238	-0.713	-1.758	-2.047	-3.216	2.04E-47
ZGLP1	-0.345	0.259	0.291	-0.908	-0.905	1.9E-12
E2F1	0.526	0.049	-0.852	-1.497	-1.559	7.89E-190
LTK	0.004	-0.015	-0.238	-0.722	-2.437	2.5E-38
CACNB1	-0.268	-0.142	-0.09	-0.792	-0.645	1.79E-07
SNORD15B	-0.503	0.017	-0.221	-0.677	-0.867	0.0123
CDC14A	-0.01	0.315	-0.54	-0.833	-0.401	5.58E-40
PTHLH	0.532	-0.31	-0.598	-0.591	-0.368	4.75E-15
CCDC17	-0.363	0.088	0.049	-0.874	-1.079	8.3E-09
CEP85	-0.428	-0.336	-0.426	-0.768	-1.183	7.05E-63
HAGHL	-0.321	-0.032	-0.146	-0.599	0.214	0.015
INE1	-0.325	-0.032	-0.053	-0.994	-0.683	0.00000632
NAB2	0.463	-0.014	-0.442	-0.619	-0.786	4.73E-29
LMO7	-0.061	-0.054	-0.616	-0.605	-1.076	2.17E-45
ZNF841	-0.212	0.067	0.114	-0.688	-0.464	7.38E-10
NDUFA4	-0.358	-0.114	-0.043	-0.564	-0.498	0.0205
SLC43A1	-0.576	-0.296	-0.423	-0.716	-0.481	1.18E-10
CGRRF1	-0.047	-0.093	-0.099	-0.614	-0.567	0.000041
ZNF431	-0.076	-0.098	-0.12	-0.728	-0.276	0.000022
DCUN1D2	-0.136	-0.183	-0.16	-0.677	-0.514	7.72E-08
MALAT1	-0.375	0.173	0.389	-0.571	-0.353	6.94E-07
MBNL1-AS1	-0.425	-0.515	-0.476	-0.848	-1.533	2.83E-21
PLEKHG2	-0.358	-0.192	-0.227	-0.68	-0.422	5.83E-12
RN7SL81P	-0.452	0.059	0.248	-0.834	-0.483	0.00241
SNORA6	-0.316	-0.173	-0.083	-1.079	-1.361	1.86E-12
SNORA10	-0.376	0.042	-0.283	-0.974	-1.286	0.0000014
MTRNR2L12	-0.434	-0.47	0.116	-0.945	-0.676	0.000916
LRRC70	-0.329	-0.394	-0.44	-0.673	-0.57	0.000728
KIFC2	-0.536	0.148	0.291	-0.625	-0.333	6.47E-09
IGLV5-52	-0.355	0.046	0.218	-0.675	-0.252	0.0000322
TSPAN13	0.345	-0.102	-0.665	-0.899	-1.207	1.2E-73
EPHA5	-0.11	-0.142	-0.61	-0.831	-1.448	2.91E-27
PASK	-0.279	-0.099	-0.396	-1.129	-1.688	2.62E-89
DYNLL1	-0.07	-0.228	-0.249	-0.649	-0.288	0.0000163
SYTL4	-0.043	0.001	-0.359	-0.617	-0.834	2.32E-43
RPL37AP1	-0.429	-0.38	-0.172	-0.583	-0.503	0.00033
MIR100HG	-0.354	-0.346	-0.211	-0.778	-0.627	5.13E-09

GPR3	-0.58	-0.411	-0.73	-1.15	-1.06	5.22E-14
LCAT	0.291	0.25	0.336	-0.768	-0.274	2.47E-14
STRIP2	-0.268	-0.003	-0.165	-0.843	-0.445	1.42E-11
CHSY3	0.528	-0.019	-0.811	-0.99	-0.906	4.91E-35
TONSL	-0.021	-0.133	-0.444	-0.929	-1.104	1.27E-36
CAPRIN2	0.161	-0.25	0.04	-0.661	-0.707	3.57E-14
CPNE7	-0.311	-0.073	0.045	-0.716	0.045	0.00000112
CCNT2	-0.119	0.1	0.052	-0.643	-0.443	6.25E-12
RAD9A	-0.424	-0.21	-0.281	-1.161	-1.309	1.78E-36
HSD17B7P2	-0.51	-0.246	-0.211	-0.931	-0.565	0.00284
SLC30A3	0.16	-0.095	-0.493	-0.663	-1.691	1.08E-12
SLC17A9	-0.357	-0.315	-0.298	-0.995	-1.034	5.07E-27
NAPB	-0.317	0.098	0.064	-0.622	-0.628	4.26E-08
ZBTB16	0.387	0.264	-0.145	-0.764	-1.942	7.68E-80
CXCL1	0.05	0.265	-0.182	-0.633	-0.194	7.34E-23
ZNF141	-0.191	-0.05	-0.18	-0.83	-0.276	0.00000274
OTUD3	0.22	0.039	0.024	-0.66	-0.758	5.61E-40
SNORD83B	-0.3	0.194	0.01	-1.038	-1.164	9.24E-14
BLOC1S5	-0.022	-0.038	-0.407	-0.659	-1.557	7.65E-93
ZNF335	-0.115	0.1	-0.081	-0.629	-0.822	6.54E-32
LAG3	-0.311	-0.354	-0.307	-1.081	-1.342	4.96E-10
TRAK2	-0.486	-0.091	-0.04	-0.75	-0.543	0.000361
PLPP4	0.402	-0.043	-0.388	-0.603	-0.441	7.74E-27
CMTM8	0.307	-0.335	-1.062	-1.088	-1.162	9.19E-38
SLC26A6	-0.488	-0.271	-0.261	-0.999	-1.061	6.17E-19
SNORD14B	-0.194	0.181	0.147	-0.72	-1.163	4.04E-08
NKTR	-0.255	-0.047	0.02	-0.796	-0.439	1.77E-11
TEX30	-0.374	-0.062	-0.173	-1.019	-1.398	1.05E-19
PI4KAP1	-0.255	0.482	0.229	-0.75	-0.686	5.36E-25
ADIPOR2	0.128	-0.141	-0.478	-0.638	-1.048	4.4E-43
NPIPP1	-0.338	-0.123	-0.347	-0.914	-0.521	0.00000702
TDRD10	0.247	0.056	-0.101	-0.609	-1.201	3.95E-22
NEBL	0.379	-0.052	-0.416	-0.616	-1.344	3.13E-59
SNORD46	-0.331	-0.281	-0.197	-1.113	-1.256	0.00000163
SNORD38B	-0.436	-0.22	-0.001	-0.868	-0.965	0.00009
SNORD38A	-0.503	-0.199	0.132	-0.824	-0.895	0.000885
EBLN2	-0.104	-0.019	0.285	-0.728	-0.455	0.000595
VCPKMT	-0.128	0.004	-0.076	-0.588	-0.823	7.95E-09

GRB14	-0.087	-0.325	-0.433	-0.839	-1.284	4.37E-39
EFNB2	0.45	-0.129	-0.769	-0.593	-1.098	3.23E-89
CCDC144A	-0.493	-0.033	0.175	-0.857	-0.593	0.000022
RPL24P2	#N/A	#N/A	#N/A	#N/A	#N/A	#N/A
UACA	-0.187	-0.132	-0.311	-0.873	-0.307	3.39E-11
ACAP3	-0.398	-0.13	-0.117	-0.933	-0.429	8.33E-09
MIR4292	-0.098	0.141	-0.085	-0.953	-0.766	0.0000769
TMEM37	0.087	-0.65	-0.977	-1.057	-2.891	3.2E-55
RPS10P3	-0.279	-0.339	-0.252	-0.581	-0.346	0.0229
CREBZF	-0.221	0.069	0.055	-0.772	-0.643	3.7E-12
NPFF	-0.538	-0.196	0.306	-0.756	-0.766	0.00000127
ZNF333	-0.296	0.071	-0.085	-0.719	-0.819	9.69E-14
ARGLU1	-0.36	0.005	-0.029	-0.896	-0.502	3.14E-10
ADGRL2	-0.226	-0.134	-0.696	-0.797	-1.406	3.34E-68
KRT10	-0.211	-0.237	-0.19	-0.606	-0.583	0.0000964
CD58	-0.008	0.057	-0.154	-0.893	-0.828	7.01E-12
ABR	0.239	-0.07	0.072	0.479	0.795	1.04E-24
ACHE	0.23	0.719	0.408	0.29	1.071	0.00000255
ACTN1	0.4	0.061	0.043	0.49	0.996	2.88E-19
ADA	0.308	0.087	0.096	0.224	1	1.58E-41
AP2B1	0.299	0.102	0.094	0.456	0.864	4.54E-18
ANPEP	0.351	0.052	-0.012	0.408	1.049	6.31E-29
ANXA3	-0.092	-0.152	0.126	0.308	1.646	4E-98
APBB2	0.378	-0.142	0.247	0.43	0.925	4.73E-65
SHROOM2	0.163	0.034	0.114	0.478	1.172	2.6E-31
ARG2	-0.304	-0.034	0.276	0.522	0.878	9.01E-21
ARHGAP5	0.4	0.206	0.092	0.208	0.921	7.1E-41
ARSD	0.347	0.109	0.155	0.541	1.156	6.2E-49
ARSE	0.346	0.074	0.035	0.28	0.884	0.00033
BACH1	0.23	0.279	0.275	0.53	0.998	2.66E-36
BCL2A1	-0.106	-0.257	0.089	0.339	1.801	3.35E-17
PRDM1	0.43	0.761	0.415	0.06	1.151	4.89E-23
BMX	-0.058	-0.167	0.032	0.407	0.937	7.95E-44
BOK	0.194	0.013	0.223	0.45	0.971	1.54E-50
ZFP36L2	-0.183	0.393	0.014	-0.018	0.7	2.91E-24
BST1	-0.425	-0.122	0.082	0.53	0.952	1.68E-46
CALD1	0.345	0.024	0.017	0.274	0.953	4.91E-32
CAPG	0.289	0.191	0.12	0.546	1.146	9.16E-31

CAPN2	0.179	0.065	0.218	0.549	1.275	1.04E-80
CASP3	0.098	0.271	0.344	0.542	0.909	7.52E-53
CAV1	-0.221	0.049	0.249	0.554	1.076	3.98E-54
CCIN	0.231	0.071	-0.051	0.56	1.266	2.55E-22
CD34	-0.042	0.042	0.015	0.261	0.73	3.28E-62
SCARB2	0.215	0.098	0.003	0.342	0.891	1.35E-52
ENTPD1	0.333	-0.055	-0.052	-0.169	0.951	5.21E-07
CD44	0.327	0.121	-0.009	0.348	0.817	1.06E-30
CD74	-0.221	0.341	0.209	0.463	1.145	0.00000405
LRBA	0.387	0.13	0.084	0.549	0.833	1E-19
CDH2	0.466	0.151	-0.159	0.274	1.23	5.36E-52
CDH5	0.296	0.123	0.162	0.555	1.134	3.49E-58
CDH11	0.058	0.173	0.157	0.536	2.215	1.8E-250
CDH13	0.127	0.06	-0.007	0.434	1.13	6.37E-62
CHRNA1	#N/A	#N/A	#N/A	#N/A	#N/A	#N/A
TPP1	0.203	0.17	0.194	0.461	1.02	2.47E-43
COL4A5	0.176	0.167	0.305	0.37	0.909	2.32E-42
COL4A6	0.231	0.088	0.158	0.355	0.871	1.99E-34
COL5A2	0.165	0.155	0.097	0.521	0.914	8.48E-21
COL12A1	0.208	0.318	0.459	0.326	1.487	2.72E-30
ATF6B	-0.153	0.008	0.061	0.315	0.697	1.23E-50
CSF1	0.517	1.239	0.384	-0.155	1.945	2.62E-134
CSRP1	0.261	-0.012	0.031	0.362	0.884	2.16E-35
CTSB	0.246	0.054	0.004	0.405	0.971	5.72E-55
CTSK	0.175	0.331	0.277	-0.022	1.581	3.76E-59
CTSO	0.271	0.342	0.219	0.537	1.147	4.85E-26
CXADR	-0.415	-0.526	-0.075	-0.033	1.303	7.64E-72
CYLD	0.231	0.638	0.385	0.312	0.682	2.5E-27
DAPK3	0.345	0.303	0.263	0.483	1.007	5.21E-42
DDX1	0.389	0.118	0.099	0.532	0.694	1.68E-15
DUSP4	0.297	0.264	0.425	0.453	1.051	7.56E-44
DUSP5	-0.042	0.678	0.517	0.374	1.027	4.03E-53
DUSP8	0.16	0.711	0.757	0.09	1.221	5.89E-18
ECE1	0.27	0.083	0.039	0.477	0.903	1.98E-21
S1PR3	0.04	0.495	0.506	0.543	0.9	2.91E-36
DMTN	-0.035	-0.03	0.033	0.342	1.464	1.7E-45
EPHB2	0.226	-0.199	-0.071	0.444	1.161	7.2E-59
ETS2	0.338	0.42	0.334	0.359	1.136	9.03E-57

ETV1	0.118	-0.315	0.278	0.394	0.881	3.42E-11
EXTL3	0.059	-0.169	-0.064	0.319	0.911	1.49E-48
F2RL1	-0.211	0.569	0.316	0.324	1.43	3.39E-71
FAP	0.212	-0.016	0.023	0.274	1.235	2.53E-73
FAT1	0.318	0.12	0.191	0.27	1.14	7.61E-18
FBP1	-0.234	-0.209	-0.454	0.155	1.062	3.97E-14
FKBP1A	0.268	0.074	-0.003	0.245	0.799	2.99E-60
FLNB	0.041	-0.069	0.132	0.253	0.924	7.81E-36
FLNC	0.377	0.293	0.364	0.49	0.893	5.26E-15
FLT4	-0.071	-0.217	-0.075	0.065	0.988	3.45E-17
FYN	0.224	0.026	0.256	0.505	0.992	1.87E-64
GAMT	-0.091	0.056	0.269	0.552	1.049	5.63E-34
GAS6	0.212	0.162	0.22	0.401	1.647	6.9E-216
GBP1	0.239	0.367	0.266	0.454	0.9	3.39E-20
GBP2	0.546	0.259	0.091	0.46	1.89	6.4E-22
GCHFR	-0.169	-0.015	0.109	-0.09	2.012	4.21E-49
GGT1	-0.088	0.095	0.237	0.392	1.155	0.0000289
GLRB	0.088	0.193	0.287	0.259	1.06	1.6E-24
GM2A	0.218	0.099	0.129	0.554	0.69	2.73E-23
GNA12	0.263	-0.031	-0.043	0.34	0.808	2.5E-30
GNAI2	0.214	0.039	0.042	0.44	0.886	3.1E-25
GRK5	0.219	-0.353	-0.168	0.153	1.055	4.37E-91
GPS2	-0.339	0.229	-0.061	-0.152	0.946	0.000071
GTF2IP1	0.322	0.072	0.278	0.331	0.928	2.44E-11
GYPC	-0.309	-0.161	-0.035	0.044	0.968	0.00000277
SERPIND1	0.176	-0.089	-0.155	0.411	2.693	2.23E-67
HLX	0.211	0.548	-0.217	0.054	1.107	1.02E-23
HMGA1	0.129	0.007	0.081	0.47	0.947	9.79E-28
FOXA1	0.291	-0.29	-0.461	-0.436	1.001	9.02E-16
HNMT	-0.233	-0.205	-0.012	0.095	0.848	0.0000424
DNAJA1	0.235	0.245	0.221	0.52	0.728	7.96E-13
TNC	0.512	0.138	-0.034	-0.016	1.301	5.12E-20
IGBP1	0.178	0.152	0.254	0.518	0.955	2.28E-69
IL15	-0.049	0.746	0.866	0.542	1.459	3.03E-11
ITGA6	0.201	0.076	0.084	0.503	1.102	3.11E-51
IRF2	0.086	0.607	0.518	0.548	0.936	1.59E-14
ISG20	0.137	-0.042	0.156	0.458	1.412	1.94E-15
JUND	0.272	0.199	0.034	0.208	0.964	1.77E-21

KCNJ12	0.348	-0.416	-0.114	0.32	0.906	5.21E-14
KIF3C	0.196	-0.203	-0.152	0.222	0.885	1.87E-35
LAMA3	-0.028	0.448	0.646	0.53	0.999	1.11E-15
LAMA4	0.159	0.001	0.113	0.39	1.147	4.02E-77
LAMC1	0.317	0.22	0.154	0.476	1.129	4.14E-33
LGALS9	0.146	0.208	0.164	0.546	2.621	1.03E-93
FADS3	-0.208	0.268	0.546	0.285	0.861	4.08E-29
VWA5A	-0.256	-0.241	0.024	0.26	0.948	2.11E-23
LOX	0.386	0.044	-0.175	-0.033	1.452	6.15E-120
LOXL1	0.088	0.022	0.045	0.229	0.935	1.28E-19
LOXL2	0.296	0.087	-0.011	0.466	0.927	3.04E-17
LPP	0.448	0.051	0.021	0.268	0.687	3.05E-19
LRPAP1	0.163	0.241	0.122	0.489	0.736	2.32E-19
LTB	-0.335	-0.08	0.314	-0.081	4.73	1.65E-221
LTF	-0.264	-0.692	0.203	0.506	1.675	2.35E-54
SMAD1	-0.095	0.06	0.252	0.553	1.985	1.34E-134
MCAM	0.024	0.118	0.204	0.549	1.409	1.45E-88
MEF2D	0.442	0.193	0.289	0.39	1.023	1.89E-29
MET	0.285	-0.06	-0.208	0.175	0.799	6.78E-36
MFAP2	0.123	0.059	-0.018	0.312	1.671	1.56E-122
MLLT3	-0.137	-0.415	-0.104	0.306	0.942	1.45E-27
MMP1	0.188	0.008	-0.019	0.382	1.212	2.12E-56
MMP10	0.42	0.171	-0.061	0.226	0.935	6.15E-46
MMP11	-0.026	-0.142	-0.091	-0.092	1.352	3.78E-24
MMP15	0.168	-0.217	-0.107	-0.059	1.242	2.65E-32
MMP19	-0.467	0.251	0.474	0.276	0.904	1.46E-38
CD200	-0.108	-0.143	-0.163	0.183	1.263	1.09E-15
MRC1	0.188	-0.048	0.146	0.507	0.987	1.34E-09
MSRA	0.101	0.116	0.108	0.489	0.959	1.38E-23
MXI1	0.35	0.288	0.366	0.263	0.891	0.00000172
NFKBIE	0.458	0.669	0.246	0.033	0.738	2.05E-23
NPAS2	-0.016	-0.149	0.269	0.488	1.254	1.62E-55
NRGN	0.013	-0.011	-0.019	0.192	1.132	1.03E-43
NT5E	0.158	0.006	0.086	0.525	1.245	4.84E-83
ROR1	0.448	0.121	0.182	0.339	0.89	1.9E-19
GPR143	0.514	0.263	0.439	0.551	1.469	1.23E-09
OAS1	-0.157	-0.386	0.231	0.482	0.979	6.66E-10
PAK3	-0.191	0.106	0.543	0.393	1.094	2.77E-09

PAM	0.315	0.204	0.1	0.458	0.867	3.01E-28
PDE2A	0.297	0.034	0.369	0.489	1.312	2.56E-38
PDK3	0.241	0.112	-0.089	0.361	0.937	1.01E-08
PECAM1	0.173	-0.033	-0.028	0.422	1.117	1.02E-76
ATP8B1	0.028	0.087	0.388	0.571	1.539	1.54E-80
PFKFB3	0.578	0.342	0.268	0.367	1.338	9.18E-102
PHEX	0.036	-0.209	0.041	0.335	1.335	1.59E-33
PLOD2	0.051	0.145	0.131	0.387	1.01	2.39E-67
PLS3	0.535	0.219	0.078	0.494	0.857	1.07E-14
PLTP	0.366	0.239	0.083	0.454	0.999	5.88E-19
PLXNB1	0.346	0.593	0.606	0.185	1.155	1.01E-37
PPP2R3A	0.228	0.221	0.241	0.293	1.361	1.2E-49
PSMB8	0.078	0.171	0.13	0.436	0.928	3.61E-30
QSOX1	0.141	0.004	0.017	0.372	1.101	1.04E-51
PTPRE	-0.112	0.004	0.378	0.315	0.876	5.61E-46
PTPRF	-0.01	-0.035	0.161	0.444	1.002	1.97E-35
PTPRM	0.035	-0.061	0.003	-0.068	1.037	1.34E-51
PTPRS	-0.116	0.256	0.124	0.078	0.894	8.31E-13
RAC2	0.224	-0.23	-0.044	0.452	0.962	2.19E-34
RALA	0.354	-0.169	-0.301	-0.111	1.055	7.14E-150
RASGRF2	0.033	-0.176	-0.357	0.04	0.742	2.94E-40
SORT1	0.141	0.02	0.063	0.39	1.3	1.51E-34
S100A3	0.353	0.281	0.159	0.233	1.241	0.0000111
CCL2	-0.31	1.245	0.886	0.285	2.573	8.58E-49
CCL7	-0.073	1.164	1.108	0.46	1.672	3.47E-11
CCL20	0.252	0.765	0.002	-0.28	1.062	0.00000875
CXCL6	-0.159	0.539	0.144	-0.236	0.882	6.34E-30
CX3CL1	-0.005	1.347	1.105	0.319	4.73	1.02E-291
SELPLG	-0.263	-0.388	0.409	0.007	1.041	3.53E-09
SFRP1	0.265	0.104	0.026	0.42	1.077	2.4E-38
ST6GAL1	-0.1	-0.004	0.228	0.395	1.104	3.02E-49
FSCN1	0.299	-0.065	-0.138	0.295	0.947	8.12E-22
SOD2	0.192	0.439	0.226	0.268	1.227	1.45E-51
SOX12	-0.165	-0.431	0.108	0.41	1.062	3.84E-43
SPOCK1	0.332	0.159	-0.02	0.379	0.801	3.76E-21
SPTBN2	0.289	-0.066	-0.067	-0.212	0.928	8.8E-40
SRC	0.19	0.317	0.218	0.434	0.894	2.26E-32
SVIL	0.142	0.166	0.452	0.523	1.116	5.2E-124

TAPBP	0.108	0.284	0.223	0.401	0.868	1E-33
TIMP2	0.188	0.02	0.065	0.453	1.358	9.59E-60
TLE4	0.549	0.668	0.468	0.558	0.988	4.12E-31
GPR137B	0.278	0.366	0.236	0.348	0.805	2.18E-08
CLDN5	-0.447	-0.406	-0.143	0.22	1.07	9.64E-64
TNFAIP1	0.195	0.381	0.304	0.468	0.963	4.43E-45
TRAF1	-0.272	1.614	1.3	0.337	2.844	1.1E-202
TRAF3	0.167	0.429	0.202	0.116	0.686	2.38E-28
TRAF5	-0.223	-0.132	0.339	-0.058	1.319	2.5E-38
UBA7	-0.206	0.146	0.505	0.205	1.343	8.07E-45
UBE2H	0.233	0.218	0.311	0.568	1.503	4.96E-113
UGCG	-0.467	0.386	0.518	0.288	1.175	1.06E-72
UTRN	0.183	0.039	0.185	0.392	1.231	1.64E-67
VIM	0.342	0.131	0.061	0.442	1.098	2.8E-31
VLDLR	0.549	0.269	-0.184	0.268	1.224	5.93E-21
CLIP2	-0.315	0.078	0.381	0.293	0.801	7.21E-38
WNT9A	-0.125	0.45	0.945	0.235	1.874	3.96E-28
XG	0.054	-0.17	0.114	0.189	1.068	0.00000012
ZFAND5	0.532	0.318	0.173	0.139	0.893	3.16E-123
DNALI1	-0.074	0.106	0.153	0.088	0.858	0.000467
LAPTM5	0.259	0.103	0.092	0.5	0.972	4.68E-39
PXDN	0.305	0.186	0.124	0.512	1.358	1.51E-47
NRIP1	0.547	0.468	0.507	0.491	1.679	7.18E-70
CDC42BPA	0.243	-0.022	0.246	0.512	0.99	2.33E-45
SEMA7A	0.305	0.378	-0.005	-0.397	1.962	2.22E-49
PRSS12	0.139	0.155	0.266	0.554	1.093	3.38E-32
CNTNAP1	-0.324	0.168	0.606	0.501	1.532	2.8E-72
NIPSNAP1	0.267	0.02	-0.09	0.307	1.008	2.07E-39
EIF3F	0.069	0.195	0.225	0.348	0.965	6.42E-54
ABCC3	-0.056	-0.073	0.346	0.19	0.827	9.03E-27
ADAM19	0.123	-0.165	0.126	0.406	1.891	2.17E-212
RNMT	0.143	0.279	0.262	0.242	0.708	3.09E-29
TNFSF10	-0.457	-0.028	0.174	0.223	2.081	5.95E-23
TNFRSF6B	-0.182	0.124	0.358	0.313	1.088	3.93E-11
TNFRSF10B	0.268	0.646	0.424	0.098	0.79	3.93E-47
FGF16	-0.12	-0.09	0.293	0.444	2.89	7.73E-58
IQGAP1	0.357	0.176	0.111	0.397	0.698	1.09E-10
ASAP2	0.431	0.143	0.329	0.539	0.715	3.35E-37

ST3GAL5	0.317	-0.282	0.102	0.475	1.286	2.41E-48
SQSTM1	0.163	0.662	0.472	0.484	1.064	1.96E-45
EIF2B2	0.388	-0.03	-0.023	0.386	0.934	1.21E-49
CCNA1	-0.14	-0.322	-0.009	0.526	0.932	1.35E-34
RAB29	-0.185	-0.005	0.241	0.413	0.758	3.47E-42
KALRN	-0.187	0.035	0.494	0.52	1.866	1.71E-57
PSTPIP1	-0.114	-0.412	0.221	0.185	0.906	1.19E-10
SLC16A4	0.093	0.026	-0.025	0.427	0.931	1.73E-19
SYNGR3	0.157	0.229	-0.135	0.032	1.051	1.41E-09
DYRK1B	-0.03	0.048	0.204	0.398	1.005	3.1E-41
MAGI1	0.087	0.139	-0.181	-0.399	0.93	6.98E-45
DLGAP1	0.156	-0.211	-0.335	0.308	0.798	1.92E-16
DHRS3	0.279	-0.049	-0.07	0.182	1.121	6.7E-22
NREP	-0.151	-0.184	0.131	0.269	1.014	3.3E-44
TGFBRAP1	-0.059	0.174	0.395	0.518	0.9	2.04E-34
NMT2	0.158	-0.019	0.005	0.325	0.959	1.13E-97
LPXN	0.37	0.031	-0.064	0.242	2.073	3.17E-214
TJP2	0.534	0.038	0.151	0.542	1.003	5E-11
CDYL	0.084	-0.001	0.094	0.426	1.031	1.22E-44
CHST2	0.223	-0.286	-0.089	0.359	1.297	2.33E-141
MAP4K4	0.172	0.051	0.164	0.331	1.109	3.39E-163
HOMER3	-0.178	-0.149	-0.096	0.053	0.898	7.1E-85
MAPK8IP1	0.246	0.24	0.189	0.495	1.03	0.00000327
ADAMTS4	-0.305	-0.036	0.202	0.095	0.764	1.78E-34
CREB5	0.057	0.277	0.297	0.52	1.584	1.15E-24
SNCAIP	-0.496	-0.474	-0.06	-0.354	1.364	1.19E-28
SH3PXD2A	-0.4	-0.417	0.424	0.183	0.842	6.41E-26
GCC2	0.332	0.519	0.474	0.474	0.754	5.25E-26
DOCK4	0.513	-0.032	0.456	0.526	1.115	6.51E-116
LZTS3	-0.263	0.557	0.785	0.535	1.064	2.37E-26
MTSS1	0.021	-0.143	0.488	0.484	1.801	9.09E-78
SCRN1	0.161	-0.02	0.113	0.518	0.956	1.55E-39
RABGAP1L	0.556	0.104	0.139	0.551	0.713	2.12E-12
TANK	-0.228	0.166	0.318	0.239	0.799	1.34E-26
BCL2L11	0.161	0.661	0.383	0.283	2.781	7.08E-83
SH2D3C	-0.561	-0.118	0.344	0.472	0.804	5.79E-50
PTPRU	0.314	0.07	-0.001	0.205	1.254	1.33E-49
ATP9A	0.335	0.148	0.156	0.234	0.768	2.03E-20

TOB1	0.566	0.519	0.303	0.448	0.939	1.59E-20
WASF2	0.07	0.2	0.223	0.355	0.827	4.93E-33
TLR6	0.014	0.028	0.067	0.272	1.045	2.09E-07
SEMA3A	0.084	0.453	0.33	0.53	1.548	3.4E-34
BTN3A3	-0.099	-0.045	0.426	0.438	1.147	2.18E-36
BASP1	0.244	-0.103	-0.1	0.275	0.95	2.51E-69
CAP2	0.303	-0.035	0.087	0.474	0.804	9.79E-14
VAT1	0.22	0.016	0.034	0.524	1.095	3.2E-30
SEMA6B	-0.319	-0.226	0.096	0.541	1.058	6.4E-42
DPYSL4	0.108	0.364	0.18	0.16	0.859	6.26E-07
CDC42EP3	-0.473	0.29	0.381	0.389	0.739	1.18E-52
TRIM16	-0.157	-0.238	0.344	0.552	1.244	6.98E-73
PDPN	0.125	-0.125	-0.175	-0.062	1.16	2.31E-41
KHDRBS1	0.337	0.134	-0.003	0.511	0.727	3.01E-11
GRAP	0.492	-0.197	-0.082	0.196	0.716	4.08E-30
KDM5B	0.148	0.171	0.151	0.348	0.955	6.13E-40
SDCCAG8	-0.142	0.017	0.224	0.303	0.885	1.32E-29
PNRC1	0.345	0.705	0.454	0.546	1.443	2.44E-49
ACOT2	0.096	0.209	0.245	0.47	1.11	6.55E-16
BTN3A1	-0.133	-0.027	0.468	0.413	1.023	4.37E-32
CAPN11	-0.022	-0.194	0.59	0.249	1.021	3.79E-12
ADAMTS7	0.108	0.434	0.565	0.478	1.518	6.72E-105
AKAP2	-0.247	0.151	0.31	0.254	1.103	2.14E-32
RNF24	0.285	0.209	0.247	0.222	1.207	2.56E-86
NID2	0.549	0.329	0.038	0.325	1.658	5.6E-47
RASA3	0.365	-0.243	0.124	0.542	0.752	6.54E-43
PHLDA1	-0.402	0.16	0.256	0.121	0.948	6.32E-57
RNF44	-0.034	0.128	0.348	0.355	0.947	4.5E-45
DLGAP4	0.203	0.176	0.345	0.562	0.959	3.55E-24
SEC31A	0.493	0.214	0.18	0.559	0.816	1.06E-10
MLXIP	0.286	0.132	0.214	0.174	0.688	1.95E-25
TRAK1	0.071	0.338	0.482	0.542	1.106	3.75E-108
CD93	0.329	-0.055	-0.064	0.358	1.162	4.34E-66
TMCC1	0.009	0.041	0.299	0.471	0.895	2.82E-25
KDM4B	-0.12	0.375	0.419	0.55	0.922	1.86E-39
SAMD4A	0.501	0.792	0.856	0.254	0.952	2.4E-14
PEG10	0.205	-0.143	-0.183	0.132	1.196	9.5E-42
ARHGAP26	0.212	-0.104	0.254	0.315	1.202	2.27E-47

TAB2	0.378	0.408	0.285	0.466	0.739	1.98E-23
TBC1D9	0.115	0.053	0.165	0.509	1.24	1.26E-72
LPIN1	-0.093	0.117	0.277	0.393	1.217	2.51E-113
RGL1	0.349	0.493	0.521	0.458	1.199	1.35E-25
FBXL7	-0.4	-0.191	-0.139	-0.043	0.831	2.84E-47
KIAA0922	-0.094	-0.103	0.008	0.157	0.842	1.43E-22
ZC3H7B	0.04	0.144	0.199	0.335	0.898	1.66E-45
IQCE	0.288	-0.149	0.017	0.157	0.695	2.17E-24
SMG6	-0.002	0.26	0.254	0.466	1.089	2.53E-47
GPR161	0.155	0.01	-0.046	0.046	0.77	2.78E-34
SEC14L2	0.289	0.403	0.448	0.545	0.791	3.28E-17
RBFOX2	0.175	-0.116	0.083	0.366	0.921	6.2E-121
SSBP3	0.385	-0.165	-0.046	0.356	0.937	2.37E-51
PLXNB2	0.536	0.99	0.963	0.561	0.882	1.51E-56
FLRT2	-0.037	0.087	0.064	0.028	1.017	8.75E-40
PANX1	0.459	0.627	0.443	0.5	1.079	2.82E-30
KIAA1549L	0.081	-0.4	-0.101	0.29	0.778	8.1E-37
PGLS	0.083	0.072	0.086	0.369	0.813	7.76E-26
QPCT	0.263	0.104	0.035	0.325	1.094	6.51E-72
BACE2	0.221	0.038	0.011	0.355	1.299	2.87E-104
ABI3BP	0.219	0.102	0.09	0.363	0.841	2.46E-13
TMEM158	-0.113	-0.334	-0.387	-0.091	2.609	6.93E-177
SAMHD1	0.184	0.117	0.142	0.518	0.858	1.03E-16
ADGRA2	0.06	-0.12	-0.154	0.031	0.941	2.63E-54
C2CD2	-0.364	0.098	0.473	0.18	0.891	1.21E-34
SNED1	0.2	0.06	0.196	0.311	1.189	8.59E-50
LRIG1	-0.347	0.164	0.292	0.147	1.086	1.61E-34
TMEM98	0.053	0.022	0.007	0.215	1.001	8.61E-131
OSBPL3	-0.219	0.512	0.773	0.575	1.157	1.61E-75
WIPI2	0.21	0.209	0.198	0.428	0.726	1.19E-16
PITPNC1	0.142	-0.044	-0.068	0.158	1.078	2.63E-20
RGS17	-0.448	-0.141	0.339	-0.051	1.074	1.38E-18
MYEOV	-0.52	0.083	0.268	0.144	0.874	1.17E-18
HBP1	0.315	0.236	0.424	0.571	0.863	5.41E-38
SDCBP2	0.136	-0.047	0.167	0.403	1.239	2.67E-23
DKK3	0.291	0.028	-0.058	0.337	1.337	2.73E-84
GPR162	-0.423	-0.128	0.369	0.256	0.837	1.16E-16
TNFRSF21	0.384	0.236	0.274	0.495	1.893	3.4E-160

RGCC	0.21	-0.079	0.149	-0.071	1.078	2.83E-35
PYCARD	0	-0.24	-0.109	0.192	1.122	5.42E-29
DSE	0.179	0.237	0.129	0.102	1.276	4.04E-39
GLTSCR2	0.068	0.175	0.208	0.409	1.08	9.39E-89
EHD4	0.338	-0.156	-0.082	0.356	0.687	1.2E-20
NOX4	0.175	0.117	0.201	0.227	0.757	1.76E-28
PODXL2	-0.088	0.031	-0.353	-0.132	1.029	1.49E-08
ASAP1	0.307	0.128	0.45	0.472	0.939	4.83E-71
NTM	0.169	0.084	0.03	0.206	1.797	7.4E-97
SHANK1	-0.36	-0.064	0.234	0.037	0.855	7.87E-26
HDDC2	0.055	0.008	0.356	0.126	1.181	8.68E-12
PLEKHO1	0.524	-0.004	-0.1	0.027	1.137	2.58E-85
ZNF219	-0.224	0.374	0.716	0.486	1.365	6.42E-19
REEP2	-0.022	0.25	0.261	0.415	1.14	2.54E-07
AIG1	-0.026	0.087	0.443	0.52	1.054	8.25E-31
TRPV2	-0.047	-0.265	-0.033	0.386	0.879	7.07E-13
SNX9	0.343	0.156	0.171	0.357	0.932	1.03E-48
RAB6B	-0.088	-0.355	0.158	0.193	0.926	7.32E-11
DHRS7	0.14	0.013	0.068	0.243	1.238	1.45E-66
ACSL5	0.102	0.007	0.153	0.39	0.939	4.38E-34
GPRC5B	-0.108	0.48	0.368	0.514	1.36	3.85E-47
GALNT7	0.35	0.216	0.117	0.401	0.914	1.28E-33
IL17D	-0.038	0.052	0.069	0.136	0.879	3.78E-15
ZFYVE1	0.525	0.536	0.394	0.428	0.958	1.04E-29
SLC37A1	0.13	0.371	0.567	0.438	1.126	8.7E-13
GSAP	-0.403	-0.2	0.679	0.402	1.388	1.91E-19
SIAE	0.164	0.015	0.072	0.327	0.915	6.74E-43
RIN2	-0.001	0.388	0.499	0.438	0.976	2.85E-67
RHOF	0.189	-0.193	0.182	0.558	1.74	4.37E-117
DDIT4	-0.479	-0.155	0.372	0.501	0.767	6.93E-51
EGLN1	0.209	0.192	0.133	0.507	1.049	1.48E-60
TMEM132A	-0.039	0.063	0.042	0.189	1.147	2.17E-108
ZSCAN2	0.125	0.284	0.172	0.568	1.064	3.25E-17
ENOX1	0.275	-0.216	-0.453	-0.331	0.93	6.74E-32
RALGPS2	-0.023	-0.234	-0.248	-0.034	0.905	1.59E-82
KIAA1551	-0.032	0.243	0.049	-0.106	1.406	8.19E-147
LRRC20	-0.051	-0.356	-0.056	0.423	1.003	2.99E-22
FGGY	0.364	-0.03	-0.063	0.491	0.783	2.84E-08

LARP6	0.434	0.112	0.085	0.151	0.729	1.62E-13
TMEM63B	-0.215	0.111	0.349	0.295	0.96	5.35E-72
DDX43	0.313	0.019	0.414	0.22	0.993	0.00000304
RBM38	0.335	0.36	0.354	0.428	0.928	3.28E-23
DOCK10	0.169	-0.059	0.209	0.214	1.118	2.09E-108
TBC1D22B	0.212	0.547	0.44	0.484	0.904	2.17E-31
BCAS4	-0.061	0.066	0.091	0.421	1.11	2.73E-08
ARHGEF40	0.123	0.508	0.498	0.103	0.779	1.19E-12
NECAP2	0.11	0.135	0.171	0.415	0.77	1.66E-28
FAR2	0.325	-0.064	-0.103	0.334	1.364	2.59E-53
PCDHGB7	0.2	0.28	0.024	0.534	1.588	2.78E-24
PCDHGA4	0.427	0.376	0.062	0.478	1.436	2.72E-16
GSDMC	-0.129	-0.234	0.263	0.317	0.885	3.62E-15
KIAA1217	-0.157	0.106	0.211	0.209	1.757	1.89E-33
TCEAL7	0.168	0.077	0.121	0.162	0.948	0.0000787
LXN	-0.184	-0.173	-0.068	-0.055	1.103	6.32E-41
CEACAM19	-0.353	0.227	0.85	0.09	0.919	3.17E-20
RTN4	0.32	0.052	-0.03	0.307	1.081	8.23E-71
ERGIC1	0.188	-0.108	0.077	0.416	1.245	5.24E-122
TMCC3	-0.115	0.009	0.286	0.41	1.335	5.22E-57
GRAMD1B	0.098	0.139	0.393	0.138	1.067	4.27E-13
NYNRIN	0.037	-0.103	0.266	0.513	1.143	2.22E-72
ALPK3	0.008	-0.302	-0.061	0.022	1.504	2.65E-172
MARCH4	0.118	-0.093	0.291	0.831	0.963	4.41E-38
WDR48	0.19	0.491	0.451	0.542	0.89	1.76E-35
KIAA1462	-0.055	-0.089	0.177	0.31	0.998	3.99E-72
KIAA1522	0.018	0.172	0.387	0.291	1.097	3.03E-32
KIAA1549	-0.001	0.223	0.191	0.23	0.92	1.02E-40
ZSWIM6	0.335	0.157	0.778	0.549	1.343	2.76E-39
ZNF71	0.175	0.33	0.051	0.288	0.965	4.47E-28
EXOC4	0.49	0.016	-0.018	0.511	1.027	5.68E-25
CCDC81	-0.054	0.013	0.588	0.373	1.415	7.39E-11
PERP	0.252	0.177	0.2	0.565	1.213	4.18E-71
MCCC2	0.316	0.121	0.215	0.427	0.911	1.38E-17
FN3K	-0.253	-0.288	-0.078	0.352	0.929	3.16E-11
ERAP2	-0.089	0.415	0.889	0.322	1.076	1.2E-20
SMURF2	0.464	-0.067	0.166	0.121	0.963	2.18E-49
PARP12	0.03	-0.067	0.245	0.405	1.034	1.64E-42

FBXL17	0.288	0.022	0.203	0.442	0.916	1.09E-18
FAM129B	0.17	-0.088	0.123	0.515	1.03	3.87E-58
PINK1	-0.115	-0.013	0.153	0.294	0.742	1.49E-50
MARCKSL1	0.14	-0.124	-0.278	0.189	1.162	2.34E-88
ZSWIM4	0.084	0.963	0.62	0.437	1.582	4.84E-92
WNK4	0.031	0.064	0.551	0.494	1.331	1.14E-58
ABHD8	-0.323	0.208	0.182	0.567	0.929	8.03E-15
CCNJL	-0.431	-0.053	0.181	0.552	1.086	2.72E-60
ARHGAP10	0.114	0.323	0.191	0.478	0.988	2.48E-31
PPP1R3B	-0.003	0.089	0.214	0.439	0.87	2.87E-27
SAP30L	0.08	0.138	0.095	0.235	0.914	5.53E-56
VPS37B	0.558	0.264	0.336	0.553	0.893	4.6E-28
DHRS12	-0.029	0.084	0.054	0.031	0.941	9.79E-07
RNF122	0.44	0.493	0.208	0.481	1.307	1.32E-25
RIN3	0.294	0.224	0.214	0.464	1.107	1.35E-30
PRR5L	-0.335	-0.382	-0.205	-0.385	0.888	9.06E-19
KIAA0319L	-0.026	0.091	0.159	0.383	0.746	6.02E-48
ERMP1	0.132	0.009	0.06	0.386	1	1.07E-38
PCNX2	0.025	0.269	0.479	0.251	0.92	3.84E-26
UBTD1	-0.194	-0.384	-0.177	0.103	1.084	7.2E-65
FBXL18	-0.001	0.273	0.364	0.519	0.7	1.13E-09
ZNF703	0.299	-0.096	0.079	0.434	1.358	9.07E-74
ARMC9	-0.09	-0.25	0.006	0.326	1.212	6.46E-39
ACSF2	-0.51	-0.137	0.035	0.235	1.153	2.97E-28
CD276	0.242	0.019	-0.028	0.322	1.025	3.11E-42
AKNA	-0.232	0.041	0.203	0.23	0.797	1.77E-36
SLC44A4	0.061	0.22	0.313	0.438	1.168	1.88E-16
FAM49A	0.129	0.218	0.322	0.559	1.357	4.6E-87
C1orf21	0.29	0.073	-0.188	0.149	0.917	5.15E-21
TRIM8	0.338	0.084	0.236	0.55	0.968	7.67E-40
TMEM163	0.161	-0.244	0.024	0.198	1.082	8.95E-18
MAP1LC3B	0.264	0.194	0.224	0.437	1.018	2.04E-100
DOCK8	0.472	0.51	0.45	0.558	1.419	1.82E-11
RAB33B	0.246	0.295	0.571	0.517	1.06	7.21E-57
SOX7	0.015	0.482	0.322	0.495	1.643	4.76E-118
YPEL3	-0.11	0.317	0.373	0.135	0.899	5.03E-17
EVA1A	-0.47	-0.329	0.11	0.462	0.956	8.16E-46
NLRC5	0.072	0.043	0.322	0.14	0.833	4.88E-19

ANTXR1	0.09	-0.036	-0.121	0.217	1.25	1.14E-105
MEX3B	0.362	0.14	0	0.334	2.127	1.57E-51
SGIP1	-0.089	0.243	0.643	0.337	2.55	2.53E-80
SLC9A7	-0.089	-0.139	0.319	0.503	1.439	3.31E-56
FAM167B	-0.273	-0.424	0.472	0.337	1.785	2.97E-28
ADTRP	0.117	-0.138	0.346	0.313	1.375	8.83E-43
PARP10	-0.464	0.406	0.833	0.528	1.018	3.49E-40
FRMD5	-0.344	0.029	0.249	0.405	1.079	5.45E-44
MYLK2	-0.044	-0.404	0.19	0.522	1.666	1.67E-27
MICALL1	0.256	0.137	0.128	0.426	0.678	5.83E-12
KIAA1671	0.132	0.269	0.408	0.524	0.8	7.32E-19
KLHL6	0.408	-0.056	0.177	0.442	1.119	1.92E-20
PHLDB2	-0.267	0.03	0.289	0.339	1.172	3.48E-115
TSPAN18	0.209	-0.289	-0.172	0.31	1.56	1.64E-95
BMF	0.498	0.602	0.679	0.186	2.142	8.36E-51
STARD13	-0.085	0.055	0.342	0.281	0.789	2.19E-65
SPOCD1	-0.213	-0.14	0.222	0.047	0.814	4.08E-33
CCDC149	0.259	0.062	0.181	0.384	0.985	9.08E-14
TRIM47	0.415	0.508	0.322	0.324	0.901	1.2E-17
TPGS1	0.058	0.261	0.142	0.46	1.2	7.79E-15
MEX3A	0.034	-0.039	-0.123	0.082	0.948	5.9E-29
ADAMTSL1	-0.185	-0.262	-0.365	-0.339	0.733	6E-42
PEX11G	-0.268	0.072	0.282	0.346	0.87	0.000237
HTRA3	0.383	0.151	-0.038	0.121	1.054	4.28E-12
SYTL3	0.572	-0.174	0.328	0.409	1.059	1.54E-09
CMTM7	0.198	-0.246	0.108	0.319	0.893	8.3E-60
AHNAK2	-0.195	-0.216	0.52	0.31	0.673	4.99E-23
GLCCI1	0.052	0.39	0.235	0.232	0.891	1.45E-10
PIK3IP1	0.104	-0.252	0.055	0.247	2.488	1.08E-80
EFHC1	-0.527	-0.087	0.485	-0.037	0.835	1E-21
FMNL2	-0.129	-0.029	0.554	0.346	1.16	3.48E-52
TMEM200A	0.357	0.131	-0.28	0.032	1.126	4.02E-51
PWWP2A	0.093	0.116	0.109	0.327	0.728	3.96E-23
C1QTNF1	0.223	-0.201	-0.212	0.24	1.377	1.28E-45
ZNF618	0.114	-0.319	0.018	0.319	0.883	3.79E-58
RAB42	0.058	-0.354	-0.41	-0.208	1.026	1.27E-10
GPR146	0.047	-0.35	0.008	0.171	1.13	1.52E-11
LYPD1	0.107	-0.335	-0.249	0.111	0.701	6.61E-28

TWIST2	0.002	-0.308	-0.21	0.097	0.991	1.32E-19
PARD3B	-0.142	-0.177	0.229	0.306	0.952	3.02E-49
ANTXR2	0.274	-0.188	-0.218	0.104	0.944	1.31E-92
DCUN1D3	0.22	0.412	0.309	0.23	0.874	1.66E-34
IQCK	0.007	0.268	0.495	0.387	1.287	1.3E-21
ZG16B	-0.129	0.399	0.402	0.359	0.934	0.00497
MSI2	0.337	0.046	0.298	0.512	0.996	2.55E-87
TTC39C	0.477	0.608	0.612	0.409	0.905	0.00000156
TRAPPC5	-0.36	-0.055	-0.062	-0.215	0.962	9.78E-11
IFFO2	0.486	-0.062	0.265	0.562	0.904	5.98E-41
FBLN7	0.331	0.297	0.139	0.554	1.103	1.44E-07
OCIAD2	0.1	-0.18	-0.274	-0.271	1.085	3.87E-113
CD109	0.278	0.044	-0.087	0.176	0.703	7.11E-16
UBXN2B	-0.338	0.02	0.233	0.379	0.771	1.95E-21
SLITRK4	-0.183	0.006	0.029	0.066	0.749	2.78E-23
ASB9	0.052	-0.173	-0.111	0.176	1.475	3.99E-39
ZNF358	-0.106	0.024	0.003	0.221	0.892	6.4E-34
SIRPA	0.312	0.017	-0.076	0.243	1.271	9.26E-88
CFAP54	-0.074	0.144	0.443	0.275	0.997	7.16E-17
C2CD4A	#N/A	#N/A	#N/A	#N/A	#N/A	#N/A
CCBE1	0.256	-0.075	0.149	0.532	1.33	2.11E-82
FAM117B	#N/A	#N/A	#N/A	#N/A	#N/A	#N/A
GLIPR2	0.279	0.13	0.022	0.077	0.705	1.43E-21
NEK10	-0.215	0.309	1.004	0.306	1.473	6.14E-15
SH3D19	-0.157	-0.09	-0.018	0.161	0.699	1.56E-83
CCDC112	0.056	0.045	0.179	0.422	0.976	8.16E-23
SH3RF2	-0.073	-0.595	-0.431	0.17	1.509	4.63E-43
SLFN5	0.438	0.627	0.46	0.558	1.117	5.13E-42
CITED4	-0.441	-0.019	0.082	0.381	2.006	3.26E-76
APCDD1L	0.363	-0.027	-0.248	0.193	0.916	5.81E-32
HMGA1P2	0.231	0.098	0.171	0.529	1.018	1.98E-19
TMEM154	0.3	-0.229	-0.202	0.074	1.685	8.72E-60
NRK	0.122	-0.07	0.074	0.364	0.696	6.78E-16
SAMD9L	-0.235	0.187	0.544	0.467	1.076	1.06E-67
OTUD1	0.207	0.299	-0.136	0.132	0.797	0.00000343
OAF	0.188	-0.079	-0.109	0.231	0.931	2.1E-50
ADGRF5	0.018	0.04	-0.008	0.298	1.765	6.44E-47
KIAA1324L	0.099	0.182	0.198	0.509	0.953	4.38E-48

FNDC5	0.135	0.167	0.266	0.241	3.437	1.85E-64
IPMK	0.504	0.704	0.522	0.506	1.434	9.59E-86
TCP11L2	-0.072	0.405	0.45	0.194	0.929	0.00000036
NATD1	-0.019	0.172	-0.083	0.088	0.805	1.87E-17
KANK3	0.032	0.28	0.628	0.315	1.169	1.06E-28
BCL9L	0.22	-0.204	0.229	0.47	0.817	4.64E-31
P4HA3	0.192	0.068	0.028	0.248	0.876	6.07E-17
CD163L1	0.077	-0.104	0.134	0.241	0.96	1.89E-42
KSR2	-0.12	-0.429	0.466	0.455	1.32	3.17E-101
SLC46A3	0.188	0.162	0.094	0.454	1.08	2.18E-07
SH3PXD2B	0.019	-0.425	-0.166	0.039	0.677	5.18E-29
RGMB	-0.363	-0.128	0.069	0.185	1.313	1.29E-147
ANO9	#N/A	#N/A	#N/A	#N/A	#N/A	#N/A
ACER2	0.526	1.143	0.89	0.476	1.048	8.41E-30
GALNT18	0.115	0.051	0.182	0.331	0.962	1.43E-10
FAM89A	0.016	-0.059	0.168	0.034	1.396	9.62E-34
MAST4	0.197	0.482	0.53	0.432	1.169	1.97E-70
AGRN	0.041	0.265	0.372	0.447	1.275	6.28E-60
RAB15	-0.012	0.105	0.099	-0.139	0.81	1.27E-69
GPR153	-0.303	-0.061	0.046	-0.111	0.732	2.88E-15
GVINP1	-0.311	-0.054	0	-0.137	1.114	4.71E-18
TMEM233	0.187	-0.365	0.084	0.355	1.226	8.38E-38
YPEL2	-0.29	0.484	0.069	0.117	0.939	6.97E-53
MEX3D	0.257	0.07	-0.317	0.028	0.957	7.16E-46
SH2D5	-0.533	0.085	0.616	0.551	0.815	5.14E-16
CTXN1	-0.253	-0.156	-0.074	0.138	1.696	3.39E-48
C16orf74	-0.152	-0.299	0.043	0.199	0.887	6.6E-43
DENND6B	-0.253	0.025	0.249	-0.263	1.624	2.37E-33
FKBP1C	0.238	-0.082	-0.103	0.159	0.681	1.24E-33
NFE2L3P1	0.091	-0.003	0.309	0.543	1.055	0.0000126
FAM229A	0.204	0.03	0.35	-0.32	0.863	0.00171
RN7SL689P	#N/A	#N/A	#N/A	#N/A	#N/A	#N/A
ABL1	0.452	-0.128	0.075	0.424	0.59	1.79E-18
ACTN4	0.275	0.092	0.018	0.416	0.655	0.00000275
AK1	0.18	0.128	0.269	0.569	0.859	4.14E-22
AK4	0.444	0.118	0.108	0.33	0.672	1.11E-11
ALDH2	0.196	0.12	0.003	0.41	0.772	1.57E-21
BIN1	0.177	-0.158	-0.006	0.347	0.774	3.52E-40

SLC25A4	0.145	0.243	0.124	0.502	0.644	8.7E-13
ANXA2	0.328	0.096	0.037	0.534	0.649	5.91E-11
ANXA2P2	0.348	0.056	0.038	0.558	0.672	4.92E-12
ANXA2P3	0.323	-0.157	-0.26	0.38	0.588	6.56E-08
ANXA6	0.397	0.048	-0.02	0.455	0.773	7.47E-11
APAF1	0.385	0.292	0.25	0.253	0.704	2.9E-41
APBA2	0.265	-0.06	0.038	0.234	0.806	3.43E-25
APBB1	0.126	0.106	0.214	0.454	0.717	9.67E-17
APLP2	0.354	0.165	0.053	0.478	0.846	1.01E-19
APP	0.343	0.124	-0.006	0.451	0.804	4.68E-15
RHOC	0.063	0.032	0.057	0.293	0.647	3.63E-34
ARHGDIG	0.152	-0.19	-0.167	-0.524	-1.031	5.97E-08
ATP6AP1	0.138	0.03	-0.051	0.342	0.61	3.57E-16
ATRX	0.033	0.123	0.182	0.111	0.632	3.55E-33
BARD1	0.126	-0.195	-0.416	-0.46	-1.042	3.95E-52
BCKDHB	-0.138	0.004	0.124	0.377	0.599	3.91E-17
BCL7A	0.526	0.033	0.099	0.259	0.632	5.33E-11
BCS1L	-0.31	-0.075	-0.02	-0.453	-0.997	5.46E-37
BDNF	-0.059	0.212	-0.424	-0.182	-1.452	1.24E-51
BFSP1	-0.041	-0.313	0.002	-0.002	0.614	0.000139
BSG	0.194	0.036	-0.056	0.388	0.622	9.9E-13
BST2	-0.116	-0.166	-0.036	0.178	0.692	2.11E-52
BTD	0.183	0.126	0.1	0.389	0.637	5.74E-13
DAGLA	-0.335	0.214	0.361	0.304	0.589	1.8E-17
CAPN1	0.285	0.104	0.101	0.519	0.709	7.59E-09
CAST	0.347	0.224	0.2	0.517	0.71	5.39E-16
CASP6	0.081	-0.071	-0.07	0.248	0.687	7.84E-21
CAT	0.133	-0.078	-0.146	0.382	0.651	6.98E-20
RUNX1	-0.042	-0.137	0.082	0.174	0.686	1.87E-42
CD47	-0.264	-0.037	0.17	0.201	0.642	4.48E-21
CDH4	-0.01	-0.122	-0.165	0.168	0.718	2.22E-11
CDK6	0.442	0.3	0.078	-0.058	0.605	5.86E-38
CDKN2A	-0.111	-0.098	-0.141	-0.044	0.675	1.1E-09
CDKN2C	-0.494	-0.742	-0.661	-0.237	-1.402	2.99E-46
CDKN2D	-0.131	-0.501	-0.056	0.151	-1.294	5.42E-22
CHN1	0.14	-0.101	-0.039	0.003	0.815	2.92E-20
CLCN4	0.168	-0.231	-0.382	-0.091	-1.42	8.06E-92
CLCN5	-0.017	0.046	-0.253	-0.396	-1.017	1.18E-32

CLU	0.282	0.048	-0.088	0.378	0.711	3.36E-14
COL8A1	0.283	0.009	-0.186	0.216	0.673	1.81E-14
CPD	0.256	0.218	0.06	0.39	0.63	3.03E-09
CRIP1	-0.062	-0.175	-0.216	-0.32	-1.095	1.8E-15
CRP	-0.527	-0.526	-0.481	-0.341	-1.002	0.0187
HAPLN1	-0.023	-0.107	-0.429	-0.198	-1.23	2.52E-28
CRYAB	0.306	-0.092	-0.288	-0.169	-2.12	1.96E-52
CST3	0.103	-0.058	-0.056	0.255	0.728	5.7E-40
CTGF	0.08	0.015	-0.302	-0.351	-0.983	6.56E-58
CTNNA1	0.482	0.208	0.078	0.499	0.679	4.83E-07
CTSD	0.224	0.175	0.059	0.539	0.634	7.33E-07
CTSZ	0.222	0.065	0.014	0.463	0.755	2.95E-20
DAP	0.149	-0.011	-0.002	0.307	0.852	1.73E-67
DBN1	0.031	-0.173	-0.069	0.178	0.76	3.11E-50
DMD	0.066	0.016	0.188	0.235	-1.128	7.01E-50
DNA2	-0.079	0.118	-0.191	-0.358	-1.222	6.22E-46
DOCK1	0.35	0.115	0.116	0.444	0.83	4.56E-33
DPP4	0.4	-0.011	-0.063	0.295	0.749	1.03E-08
DTNA	-0.049	0.2	0.408	0.246	0.789	0.0000492
ECM1	0.043	0.149	0.161	0.2	0.651	4.83E-08
EIF4B	0.253	0.185	0.248	0.516	0.74	4.29E-44
EPB41L1	0.192	-0.15	-0.028	0.437	0.776	1.4E-18
EPOR	-0.313	-0.217	0.102	0.318	0.596	1.11E-15
NR2F6	-0.239	-0.415	-0.109	0.18	0.585	1.95E-17
ETV4	-0.376	-0.225	0.041	0.236	0.642	1.81E-38
FANCE	-0.069	-0.247	-0.371	-0.569	-1.044	2.09E-27
ACSL1	0.408	0.172	0.115	0.253	0.628	2.27E-13
ACSL3	0.189	-0.192	-0.257	0.047	0.664	6.02E-40
ACSL4	0.462	0.169	0.143	0.532	0.775	2.9E-16
FBN2	0.207	0.087	0.02	0.272	0.759	2.84E-11
FEN1	0.472	0.167	-0.181	-0.136	-0.956	1.46E-55
FGFR1	0.059	-0.094	-0.128	0.015	0.787	2.61E-61
FHL1	0.226	-0.004	-0.049	0.292	0.798	1.09E-29
FHL3	0.098	0.038	0.171	0.268	0.725	2.49E-23
FLOT2	-0.101	-0.114	0.106	0.519	0.818	1.18E-28
FLT1	0.478	-0.075	-0.503	-0.318	-1.467	1.78E-55
FUCA2	0.147	0.15	0.127	0.413	0.785	4.57E-38
ACKR1	0.06	0.113	0.025	-0.488	-1.457	7.79E-42

GAA	0.113	0.13	0.07	0.302	0.633	1.62E-11
GALNS	0.177	0.071	0.069	0.411	0.634	6.37E-10
GATA3	-0.016	-0.298	-0.483	-0.128	-1.134	1.35E-17
GCNT1	-0.037	-0.472	-0.528	-0.191	-0.959	2.35E-33
GLB1	0.25	0.097	-0.02	0.466	0.705	4.66E-19
GLDC	-0.097	-0.051	0.207	0.535	0.73	2.35E-09
GLDCP1	-0.098	-0.073	0.183	0.479	0.827	0.000164
GLS	-0.044	-0.021	0.104	0.202	0.655	1.73E-51
GNS	0.232	0.077	-0.033	0.352	0.653	2.74E-14
GRN	0.249	0.083	-0.027	0.442	0.807	5.36E-14
GRINA	0.067	0.029	-0.011	0.261	0.763	2.29E-23
GSK3B	0.301	0.177	0.169	0.422	0.762	2.05E-16
GSTM2	-0.069	0.184	0.333	-0.117	0.693	0.00195
GTF2I	-0.001	-0.179	-0.03	0.144	0.605	2.43E-16
HADHA	0.325	0.201	0.148	0.542	0.753	9.32E-11
HADHB	0.268	0.17	0.132	0.483	0.678	2.3E-19
HDAC1	0.241	-0.041	-0.027	0.089	0.612	1.94E-39
HELLS	0.074	0.357	-0.262	-0.368	-0.982	2.54E-59
HIF1A	0.219	0.084	0.114	0.442	0.776	7.15E-16
HLA-A	0.24	0.136	0.034	0.398	0.799	1.65E-23
HLA-B	0.251	0.328	0.166	0.524	0.662	4.9E-13
HLA-H	0.351	0.295	0.113	0.34	0.621	0.0125
HLA-J	0.317	0.154	0.304	0.565	0.826	0.00463
HMOX2	0.302	-0.145	-0.1	0.361	0.658	2.05E-22
HNRNPC	0.443	0.146	-0.013	0.325	0.662	5.42E-21
HOXB9	-0.045	0.004	-0.139	-0.048	0.837	9.59E-10
HSD17B2	-0.284	-0.296	-0.018	0.389	0.725	2.14E-44
HTR1B	-0.507	-0.147	-0.553	-0.526	-2.017	1.45E-61
ID3	-0.221	-0.391	-0.401	-0.179	-1.209	6.46E-21
IDS	0.256	0.085	0.061	0.084	0.842	3.71E-62
IFNAR1	-0.003	0.183	0.172	0.368	0.589	4.06E-32
IFNGR2	-0.015	0.106	0.05	0.087	0.667	3.56E-26
IGF2R	0.159	0.163	0.135	0.482	0.664	4.43E-10
IGFBP1	-0.049	-0.109	-0.561	-0.532	-4.25	4.58E-182
IGFBP3	-0.076	-0.008	-0.263	-0.119	-3.927	1.71E-150
IGFBP6	0.181	0.072	0.037	0.221	0.759	1.59E-10
IGFBP7	0.219	0.076	0.001	0.32	0.735	3.06E-28
IL4R	0.389	0.24	0.391	0.371	0.641	1.41E-29

IL18	-0.09	0.102	-0.466	-0.288	-1.287	0.0000185
ITGB5	0.263	-0.011	-0.07	0.368	0.627	8.76E-16
ITPR3	-0.115	-0.192	0.305	-0.011	0.712	1E-33
JARID2	0.141	-0.257	0.198	0.427	0.84	6.49E-35
KCNK1	0.122	0.153	0.198	0.191	0.859	0.0000075
KIF22	-0.431	-0.428	-0.438	-0.329	-1	9.45E-38
KRT15	#N/A	#N/A	#N/A	#N/A	#N/A	#N/A
KRT19	0.397	-0.285	-0.313	0.225	0.607	1.85E-11
LAMP1	0.291	0.128	-0.003	0.448	0.708	1.94E-16
LASP1	0.195	-0.033	0.027	0.454	0.651	2.13E-12
LIMK1	0.152	-0.134	-0.032	0.294	0.669	9.73E-20
LTBP2	0.343	0.006	-0.209	0.084	0.688	4.28E-13
BCAM	-0.016	0.213	0.262	0.34	0.817	5.88E-17
MAN1A1	0.151	0.115	0.005	0.101	0.611	1.54E-11
MAN2B1	0.095	0.232	0.139	0.273	0.627	1.73E-12
MANBA	-0.017	-0.083	-0.001	0.316	0.793	3.47E-11
MAOA	0.196	-0.101	-0.418	-0.229	-2.023	1.37E-44
MAP1A	0.034	0.291	0.223	0.334	-1.013	9.54E-14
MAP2	0.133	0.017	-0.416	-0.429	-1.004	2.25E-14
MAP4	0.078	0.041	0.075	0.097	0.588	2.71E-38
MFGE8	0.176	0.123	0.069	0.294	0.665	2.87E-16
MGAT5	0.148	-0.187	-0.037	0.384	0.662	3.27E-31
MGP	0.029	0.004	-0.088	-0.144	-1.331	6.27E-68
MME	0.055	0.024	0.078	0.262	0.847	1.81E-31
MMP2	0.347	0.075	-0.067	0.378	0.715	2.2E-13
MMP7	0.055	-0.042	-0.594	-0.389	-1.345	5.06E-07
MPST	-0.161	-0.188	-0.049	0.196	0.804	2.05E-55
MYH9	0.492	0.125	0.061	0.316	0.72	2.95E-07
MYH10	0.139	-0.049	-0.15	0.212	0.692	1.59E-10
MYL4	-0.003	0.115	0.55	0.027	0.825	0.0133
MYO1D	0.414	0.12	-0.223	0.106	0.833	2E-14
MYO6	0.432	0.398	0.344	0.523	0.671	1.77E-07
NFIX	0.102	-0.223	-0.014	0.436	0.798	9.13E-15
NFKB2	-0.042	1.151	0.724	0.124	0.806	4.5E-74
NOTCH2	0.069	0.064	0.04	0.196	0.655	1.56E-15
NPR1	-0.123	-0.069	-0.271	-0.252	-2.008	7.77E-47
DDR2	-0.259	-0.089	0.141	0.004	0.877	1.17E-15
NUCB1	0.251	0.109	0.041	0.482	0.59	8.73E-09

CLDN11	0.059	-0.04	0.268	0.48	0.834	2.34E-18
PEBP1	0.162	-0.005	-0.007	0.328	0.781	4.3E-40
PDCL	0.395	0.074	0.16	0.451	0.661	1.71E-16
PCBD1	-0.11	-0.115	0.079	0.277	0.772	3.24E-43
PCDHGC3	0.197	0.018	-0.004	0.312	0.729	5.55E-13
PCSK5	0.201	0.172	-0.045	0.175	0.584	6.75E-10
PFKP	0.351	-0.024	-0.142	0.247	0.59	1.59E-10
PGD	0.397	0.19	0.077	0.526	0.757	8.28E-11
PITPNA	0.334	0.004	-0.106	0.196	0.673	1.21E-26
PLAUR	0.124	-0.07	0.018	0.291	0.634	4.98E-17
PLOD1	0.217	0.029	-0.084	0.381	0.673	1.22E-11
PML	-0.006	0.198	0.288	0.391	0.86	2.12E-29
PRRX1	-0.313	-0.171	0.072	0.233	0.8	1.64E-07
PPARA	-0.36	0.142	0.311	0.31	0.844	2.44E-23
PPP3CA	0.244	-0.113	-0.152	0.092	0.781	2.81E-18
PRIM1	0.136	0.113	-0.158	-0.173	-1.21	4.63E-37
PRKAB1	-0.011	0.218	0.477	0.387	0.828	2.81E-23
PKIA	0.038	0	0.165	0.241	0.626	4.07E-17
PRKCA	0.484	0.01	0.109	0.498	0.695	1.52E-20
PRKX	0.036	0.421	0.445	0.414	0.862	1.14E-51
PRNP	0.231	-0.056	-0.202	0.094	0.722	3.95E-52
PROS1	0.359	0.296	-0.116	0.407	0.59	0.000558
PRRG1	0.132	0.229	-0.032	0.036	0.86	6.58E-51
HTRA1	0.344	0.055	-0.093	0.315	0.654	1.59E-18
PSAP	0.269	0.083	-0.014	0.406	0.705	1.23E-17
PSEN2	0.139	0.013	0.098	0.394	0.613	0.00000108
PSMB10	0.199	-0.004	0.106	0.265	0.675	8.24E-12
PSME1	0.123	0.09	0.18	0.236	0.59	3.02E-21
PTEN	0.157	0.241	0.099	0.303	0.6	6.8E-20
PTGER1	-0.342	-0.301	-0.412	-0.252	0.729	0.00000328
PTGFRN	0.403	0.121	0.13	0.417	0.837	4.11E-15
PTK2	0.209	-0.065	0.171	0.521	0.688	4.53E-45
PTPN14	0.185	0.03	0.122	0.289	0.681	7.69E-23
PTPRK	0.286	0.062	0.014	0.064	0.591	1.92E-11
PTS	-0.127	-0.038	-0.061	-0.532	-1.092	8.79E-27
RAB5B	0.122	0.039	0.143	0.254	0.613	1.41E-21
RALGDS	-0.182	0.225	0.406	0.015	0.78	8.1E-12
RARRES3	0.197	-0.125	0.083	-0.074	0.777	0.00453

RBBP8	0.346	0.337	0.023	-0.097	-1.094	8.99E-74
RCN1	0.222	0.256	0.216	0.533	0.858	1.07E-44
RFX3	-0.473	0.116	0.334	0.285	0.745	3.18E-15
RGS3	-0.308	-0.04	-0.065	-0.027	0.762	6.86E-40
RNPEP	0.214	0.075	0.058	0.382	0.681	1.43E-12
RPGR	0.196	-0.009	-0.227	-0.473	-1.627	5.56E-117
RPS6KA3	0.206	-0.079	-0.017	0.225	0.699	4.58E-46
RRBP1	0.349	0.069	0.052	0.307	0.694	2.55E-16
SDC4	0.537	0.487	0.064	-0.038	0.641	4.32E-32
SGCG	0.169	0.13	-0.553	-0.355	-2.599	7.57E-39
SH3BGRL	0.153	0.003	0.067	0.206	0.641	1.28E-32
SLC3A2	0.141	0.018	-0.143	-0.194	-1.137	1.28E-65
SLC8A1	0.049	-0.317	0.046	0.146	0.698	7.75E-10
SLC12A2	-0.039	0.159	-0.077	-0.244	0.727	2.04E-49
SMARCC1	0.411	0.126	-0.05	0.282	0.66	1.93E-11
SPR	0.067	0.016	0.125	0.415	0.634	5.96E-13
ST13	0.291	0.151	0.106	0.49	0.711	4.9E-29
STAT1	0.085	0.001	0.087	0.517	0.725	5.44E-17
SULT1E1	-0.53	-0.295	-0.105	-0.29	-1.54	1.54E-07
STK10	0.193	0.112	0.178	0.367	0.616	1.31E-08
ADAM17	0.212	0.197	0.179	0.317	0.63	2.3E-33
TBL1X	0.155	-0.189	-0.058	0.27	0.709	1.14E-21
TCF19	0.259	0.103	-0.02	-0.157	-1.266	1.09E-122
TCN2	0.204	0.018	0.066	0.525	0.687	4.58E-15
PRDX2	0.211	0.078	0.035	0.439	0.752	1.82E-25
TFDP2	0.182	0.011	-0.198	0.043	0.655	8.41E-24
TGFBR2	-0.134	-0.081	0.064	0.292	0.815	2.14E-46
TJP1	0.473	0.12	0.249	0.472	0.719	3.23E-21
TSPAN8	-0.146	0.128	-0.101	0.065	-1.36	5.89E-14
TNFRSF1B	0.105	-0.373	-0.357	-0.217	-1.109	1.11E-42
TNS1	0.397	-0.193	-0.014	0.192	0.772	1.28E-42
TOP2B	0.297	0.14	0.116	0.541	0.821	2.33E-15
TP53	0.15	0.235	0.221	0.448	0.695	5.22E-13
TSPYL1	0.245	0.111	0.021	0.48	0.607	2.55E-07
PHLDA2	0.227	-0.024	-0.254	-0.149	0.584	8.94E-16
TST	-0.053	-0.034	0.078	0.401	0.679	3.53E-16
TSTA3	-0.105	-0.01	0.066	0.021	0.779	7.93E-63
UCK2	0.323	-0.089	0	0.284	0.725	5.14E-38

VEGFB	-0.02	-0.037	0.165	0.19	0.677	1.34E-27
WIPF1	0.15	0.163	0.18	0.41	0.643	9.38E-13
YY1	0.337	0.024	-0.047	0.219	0.585	4.39E-16
ZNF134	0.384	0.259	0.218	0.335	0.597	2.2E-09
TRIM25	0.067	0.016	0.044	0.321	0.584	1.74E-21
ZNF217	0.331	0.217	0.233	0.329	0.67	1.53E-16
LRP8	-0.039	-0.134	-0.124	-0.539	-1.258	3.14E-106
TUBA1A	0.18	-0.151	-0.214	0.164	0.686	4.25E-30
TFPI2	0.063	0.032	-0.106	0.105	0.846	2.38E-83
FXR1	0.157	0.185	0.147	0.422	0.631	9.84E-20
TMEM187	-0.089	-0.065	0.143	0.57	0.686	3.39E-13
SNN	0.348	0.008	-0.373	-0.346	0.774	6.18E-43
CDC7	-0.231	-0.2	-0.22	-0.412	-1.261	4.07E-44
CDC45	0.187	0.107	-0.297	-0.526	-1.255	1.34E-72
HYAL3	0.087	0.118	0.349	0.47	0.803	1.43E-10
NCK2	0.162	-0.046	-0.203	0.029	0.575	8.22E-11
TPST2	0.003	-0.076	-0.066	-0.069	0.733	4.49E-37
PPFIBP1	0.028	0.369	0.403	0.406	0.594	4.54E-36
KCNAB2	0.248	-0.24	0.072	0.49	0.599	0.0000194
IFITM1	0.1	-0.065	-0.128	0.281	0.743	0.00000356
PIR	0.167	0.016	0.008	0.283	0.742	1.84E-40
RGS20	-0.047	0.301	0.625	0.407	0.63	3.6E-09
PLPP2	0.011	-0.156	-0.37	-0.305	0.649	0.000133
RNASET2	0.057	-0.125	-0.06	0.235	0.703	1.7E-12
PCDHGB4	0.285	0.217	-0.012	0.275	0.816	0.00000802
AKR1C3	-0.194	-0.106	-0.129	-0.071	-1.132	1.64E-103
NUMB	0.092	0.039	0.042	0.221	0.586	1.64E-24
TNKS	-0.106	0.21	0.149	0.263	0.624	1.42E-18
IRS2	-0.258	0.288	-0.266	-0.453	-1.526	8.55E-43
CTSF	-0.063	-0.001	-0.077	0.067	0.745	3.14E-26
ADAM15	-0.047	-0.053	-0.012	0.225	0.656	1.23E-27
CREG1	0.159	0.229	0.128	0.394	0.824	2.06E-21
NRP2	0.092	-0.027	-0.086	0.206	0.774	5.3E-26
SOCS2	0.067	0.49	0.17	-0.027	0.791	3.55E-07
ALDH1A2	0.207	0.017	0.153	0.51	0.614	3.44E-10
IER3	0.046	0.276	0.236	0.233	0.7	4.56E-39
ARHGEF7	-0.352	0.084	0.18	0.124	0.596	1.5E-50
SKAP2	0.191	0.105	0.081	0.531	0.678	1.19E-17

CLIC3	0.484	-0.038	-0.39	-0.193	-1.018	3.14E-18
PAPSS2	0.402	0.155	-0.132	0.146	-1.133	9.78E-11
LDB2	0.135	-0.506	-0.703	-0.576	-1.139	1.74E-34
PKMYT1	0.144	0.15	-0.198	-0.356	-1.116	9.69E-42
IL1RL1	0.252	0.076	-0.4	-0.477	-2.633	2.6E-112
ZBED1	0.435	0.196	-0.213	0.094	0.582	9.96E-19
SLC16A7	0.363	-0.124	-0.235	-0.465	-1.635	9.63E-67
CYTH3	0.412	0.101	0.073	0.304	0.586	1.82E-16
ADGRG1	0.314	0.063	0.086	0.449	0.788	4.44E-24
B4GALT6	0.139	0.265	0.132	0.174	0.661	0.000581
ZFYVE9	0.123	-0.178	0.025	0.358	0.59	4.45E-17
FAM189A2	-0.361	0.024	0.37	-0.175	0.742	1.03E-17
MED17	0.497	0.205	0.18	0.222	0.604	0.000525
ITM2B	0.235	0.138	0.019	0.271	0.674	1.98E-33
RASAL2	0.045	-0.096	-0.154	-0.121	0.627	1.44E-32
IL27RA	0.196	0.063	0.032	0.472	0.769	6.41E-21
SLC4A8	-0.382	-0.417	0.111	0.182	0.797	1.75E-29
NPEPPS	0.213	0.117	0.157	0.377	0.614	1.09E-17
VPS4B	0.373	0.279	0.213	0.464	0.62	4.56E-12
NCOR1	0.057	-0.011	0.068	0.323	0.605	2.33E-19
MICAL2	0.463	-0.059	0.103	0.525	0.731	1.5E-18
PPM1F	-0.125	-0.307	0.148	0.351	0.705	2.38E-51
SDC3	0.199	0.244	0.21	0.536	0.636	7.66E-11
RAPGEF2	0.338	0.169	0.185	0.34	0.613	1.67E-19
TRAM2	-0.154	-0.212	0.151	0.288	0.758	6.86E-63
TOX	-0.502	-0.015	-0.426	-0.202	-1.606	8.79E-42
SUSD6	0.434	0.442	0.399	0.086	0.738	2.4E-32
RB1CC1	0.257	0.314	0.243	0.405	0.594	8.59E-12
GINS1	0.054	0.009	-0.194	-0.277	-0.966	2.95E-37
CEP170	-0.076	0.033	0.074	0.252	0.606	3.31E-25
KLHL21	0.527	0.294	0.271	0.285	0.6	7.46E-21
RNF10	0.166	0.068	0.08	0.344	0.607	1.4E-20
MVP	0.297	0.051	0.061	0.498	0.78	9.43E-09
HDAC5	0.358	0.404	0.348	0.472	0.716	5.07E-13
DNAJB6	0.175	0.045	0.052	0.324	0.645	2.06E-16
TSPAN5	0.109	-0.253	-0.127	0.181	0.675	4.29E-38
SLC25A13	0.281	-0.035	0.021	0.395	0.625	4.99E-18
DHRS9	0.097	-0.059	-0.31	-0.249	-1.136	3.21E-36

MPZL2	-0.235	-0.164	-0.157	0.085	0.611	6.49E-37
PAK4	0.098	-0.258	-0.202	0.278	0.582	2.6E-15
HMG20A	0.057	0.119	0.15	0.493	0.836	6.05E-33
CPQ	0.111	0.039	-0.08	0.29	0.601	1.4E-13
COG5	0.266	0.064	0.138	0.397	0.619	1.25E-23
FST	-0.01	0.992	0.195	-0.086	-2.006	5.53E-201
UNC13B	0.042	0.175	0.472	0.576	0.636	5.75E-57
FBLN5	0.389	0.053	-0.248	0.067	-1.244	3.16E-26
SORBS1	0.504	0.174	0.178	0.15	0.626	3.96E-07
IFITM2	0.178	-0.025	0.045	0.471	0.592	1.14E-12
PAIP1	0.12	0.007	-0.175	0.093	0.597	5.52E-22
RAD51AP1	-0.125	0.033	-0.18	-0.298	-1.318	4.75E-72
CAMKK2	-0.046	0.088	0.243	0.296	0.683	6.94E-49
RRAGA	0.266	-0.039	-0.008	0.561	0.659	2.23E-16
GNB5	0.161	0.035	0.106	0.281	0.603	4.05E-10
NES	-0.221	-0.074	0.098	-0.086	0.725	1.15E-42
FGL2	0.517	0.063	-0.45	-0.081	-1.989	3.99E-80
NPFFR2	-0.08	-0.19	-0.338	0.422	0.809	2.33E-14
UGT2A1	-0.147	-0.036	-0.392	-0.284	-1.689	4.5E-22
TOMM34	0.115	-0.063	-0.026	0.308	0.66	2.91E-16
OS9	0.202	0.123	0.061	0.403	0.594	4.71E-10
CCNI	0.309	0.23	0.204	0.456	0.586	5.47E-17
MID2	-0.282	0.047	0.088	0.294	0.605	3.97E-17
SLC35D2	0.032	-0.228	0.156	0.231	0.693	2.11E-13
DNAJB4	-0.258	-0.025	-0.469	-0.31	-1.078	3.75E-24
PRSS23	0.248	0.005	-0.131	0.153	0.844	9.17E-70
BTN3A2	-0.127	0.033	0.173	0.345	0.815	9.12E-32
WDR45	-0.187	0.04	0.324	0.206	0.664	1.07E-18
RABL2B	-0.28	0.184	0.55	0.291	0.671	5.75E-24
FSTL1	0.273	0.073	-0.06	0.281	0.662	1.15E-29
SLC2A6	-0.015	0.361	0.146	0.041	0.609	4.45E-25
TP53TG1	-0.054	0.133	0.547	0.383	0.655	1.49E-07
KLF12	0.18	-0.051	0.345	0.294	0.624	5.7E-18
MGAT4B	0.14	0.123	0.019	0.127	0.711	1.24E-40
TWF2	0.268	0.04	0.007	0.455	0.844	1.03E-20
MRAS	0.29	-0.103	-0.245	-0.061	0.761	9.07E-10
CLSTN1	0.204	0.095	-0.009	0.42	0.749	4.1E-15
ZBTB1	0.354	0.093	0.28	0.428	0.657	8.15E-20

KIFAP3	0.302	0.135	0.071	0.52	0.573	0.00000313
RAB3GAP1	0.283	0.329	0.216	0.477	0.619	9.33E-10
PTGR1	0.156	0.257	0.189	0.563	0.629	3.18E-25
TTLL5	0.127	-0.076	0.113	0.48	0.724	2.12E-20
CDK19	-0.188	0.251	0.308	0.414	0.783	9.24E-22
CEP68	-0.122	-0.008	0.063	0.133	0.631	1.17E-21
CYFIP1	0.376	0.206	0.165	0.531	0.747	1.37E-09
PSME4	0.188	0.354	0.276	0.501	0.584	1.87E-17
TRIM2	0.206	0.011	-0.013	0.154	0.726	4.21E-13
MAN2B2	0.093	0.078	-0.049	0.29	0.625	2.88E-11
KHNYN	0.265	0.305	0.531	0.395	0.655	3.42E-31
HAUS5	-0.329	0.051	-0.032	-0.552	-1.133	2.99E-37
PSD3	0.309	-0.015	-0.115	0.209	0.866	6.3E-25
COTL1	0.19	0.072	0.033	0.349	0.795	1.79E-35
NCS1	0.429	0.086	-0.089	0.129	0.685	1.72E-20
NOMO1	#N/A	#N/A	#N/A	#N/A	#N/A	#N/A
RPL13A	-0.067	0.008	0.128	0.134	0.657	1.04E-28
CORO1C	0.142	-0.088	0.044	0.331	0.819	1.18E-102
CD2AP	0.231	0.208	0.085	0.382	0.627	8.05E-14
MKRN1	0.307	0.14	0.098	0.465	0.665	4.9E-12
FAM89B	0.201	-0.127	-0.163	-0.141	0.683	2.69E-54
LDOC1	0.076	-0.005	0.049	0.446	0.699	2.63E-13
PLD3	0.167	-0.008	-0.056	0.404	0.738	6.98E-20
SLC7A11	0.084	0.386	-0.171	-0.287	-1.792	7.72E-161
MAFF	-0.234	0.491	0.46	0.233	0.669	2.25E-29
APOL2	-0.065	0.348	0.702	0.382	0.711	2.56E-38
BCL2L13	0.25	0.105	0.209	0.486	0.668	1.84E-34
FBXL2	-0.315	-0.503	-0.084	-0.13	0.597	1.35E-28
DNAH1	-0.332	0.089	0.006	-0.192	0.787	1.14E-11
SIPA1L1	0.101	0.101	0.261	0.228	0.629	2.11E-16
RAB11FIP5	-0.055	-0.026	0.179	0.37	0.615	3.72E-22
RAI14	0.276	-0.099	0.249	0.499	0.603	1.79E-32
FAM127B	0.186	0.125	0.176	0.498	0.644	5.99E-11
TCTN3	0.133	0.061	0.094	0.433	0.695	9.83E-17
PHF19	-0.433	-0.584	-0.518	-0.445	-1.105	2.28E-59
RIBC2	-0.222	0.055	0.234	-0.308	-2.195	2.36E-32
FBXW8	0.262	0.026	0.195	0.233	0.601	7.11E-09
FBXO22	0.427	0.686	0.822	0.456	0.648	8.34E-22

FBXO5	-0.232	-0.27	-0.377	-0.295	-1.259	4.45E-73
SEZ6L2	0.163	0.024	0.041	0.474	0.658	0.0000857
GREM1	0.331	0.127	-0.251	-0.248	0.729	6.53E-22
STEAP1	-0.406	-0.443	-0.49	-0.449	-1.557	2.32E-79
ANKRD1	0.002	-0.047	-0.117	-0.043	-1.014	6.69E-70
STAU2	0.026	0.104	0.228	0.517	0.726	7.31E-22
FOXP1	-0.017	-0.137	-0.075	0.295	0.633	5.87E-27
SLCO4A1	-0.133	-0.32	-0.147	-0.371	-1.34	8.55E-27
AAMDC	0.156	0.022	0.138	0.36	0.648	8.3E-15
IFT81	-0.267	0.003	0.219	0.344	0.719	3.55E-12
TMOD3	0.287	0.126	0.052	0.415	0.658	9.97E-15
CARD10	-0.096	0.065	0.304	0.391	0.617	1.29E-17
PARVB	0.282	-0.006	0.022	0.379	0.714	1.02E-22
ZDHHC1	-0.127	-0.12	0.063	-0.062	0.679	6.75E-09
RBM15B	0.172	-0.023	-0.037	0.32	0.64	1.1E-16
OSGIN1	0.146	0.337	-0.261	-0.281	-1.217	1E-27
CDR2L	0.312	0.097	0.022	0.164	0.806	2.33E-15
CHST11	0.331	-0.166	-0.251	-0.037	0.639	5.87E-23
ANO7	0.013	-0.001	-0.292	-0.305	0.711	0.0000043
AK3	0.062	0.168	0.086	0.177	0.802	7.72E-64
UBXN1	0.182	0.185	0.2	0.41	0.593	1.03E-18
ZDHHC9	0.174	0.094	0.083	0.335	0.693	3.57E-19
ING4	-0.258	-0.011	0.178	0.102	0.748	1.08E-24
CERCAM	0.035	-0.031	0.077	0.263	0.665	1.42E-24
GOLM1	0.16	0.021	-0.088	0.196	0.602	2.16E-16
MEX3C	0.391	0.095	0.006	0.444	0.841	4.51E-27
SNX7	0.042	0.091	0.135	0.416	0.595	9.29E-22
EIF3L	0.234	0.169	0.228	0.429	0.684	3.68E-29
PRKAG2	0.139	0.243	0.474	0.494	0.841	4.2E-29
UBE2J1	0.399	0.023	-0.008	0.155	0.599	2.01E-45
LIMA1	0.256	0.122	0.004	0.397	0.589	1.12E-11
CXXC5	0.396	-0.328	-0.023	0.069	0.6	1.16E-11
CUTA	0.01	-0.158	-0.09	0.175	0.605	9.25E-26
YPEL5	0.054	0.204	0.212	0.076	0.795	2.83E-25
SARAF	-0.007	0.018	0.086	0.262	0.837	2.82E-79
NLK	-0.222	0.043	0.156	0.101	0.648	3.19E-48
CAB39	0.334	0.393	0.157	0.445	0.782	9.79E-21
RTEL1	-0.336	0.08	0.051	-0.445	-1.129	2.29E-39

ERAP1	0.315	0.158	0.082	0.467	0.798	8.5E-20
TM7SF3	-0.006	-0.011	0.153	0.403	0.658	1.62E-45
SIX4	0.142	0.023	-0.257	-0.095	0.582	2.85E-09
PHAX	0.224	0.133	0.078	0.445	0.601	2.64E-09
CSNK1G1	0.013	0.306	0.23	0.186	0.66	7.85E-36
A4GALT	0.211	-0.009	0.025	0.26	0.674	5.11E-13
ERRFI1	0.307	0.139	0.177	0.051	0.675	1.09E-24
C11orf71	-0.232	0.051	-0.006	-0.292	-1.258	6.56E-20
USP53	-0.302	0.047	0.05	0.052	0.765	2.9E-63
CCHCR1	-0.155	-0.087	-0.079	-0.265	-1.019	1.08E-32
EXOC6	-0.512	-0.412	-0.816	-0.525	-0.99	2.29E-62
ROBO4	-0.093	-0.147	0.1	0.415	0.791	1.86E-48
LZTFL1	0.181	0.047	0.077	0.408	0.818	8.69E-18
TRIM44	0.194	0.029	0.155	0.214	0.594	2.36E-32
BNC2	-0.035	0.028	0.118	0.315	0.6	3.89E-24
VPS13C	-0.295	0.133	0.378	0.186	0.599	2.02E-28
RETSAT	0.161	0.335	0.43	0.515	0.687	3.34E-50
FOCAD	0.328	0.01	0.073	0.379	0.693	5.7E-16
UBE2R2	0.278	-0.002	-0.034	0.296	0.685	1.26E-18
DUSP23	-0.259	-0.363	-0.35	-0.341	-1.218	2.03E-42
TMEM160	-0.209	0.043	-0.061	0.062	0.775	0.0000001
GEMIN8	-0.056	-0.177	0.163	0.359	0.603	2.26E-11
CLN6	0.077	-0.068	-0.258	-0.213	-0.984	2.66E-29
TMEM51	0.095	-0.083	-0.091	-0.134	0.812	2.65E-17
TMEM38B	-0.144	-0.009	-0.121	-0.563	-1.235	2.08E-22
NADSYN1	-0.062	0.413	0.536	0.239	0.666	4.1E-33
MAP1S	0.223	-0.094	0.208	0.49	0.639	1.54E-10
ZNF532	0.355	0.048	0.085	0.23	0.61	1.66E-32
P3H2	0.458	0.411	0.299	0.551	0.822	3.28E-09
EXD2	-0.006	0.034	0.261	0.474	0.62	8.77E-20
UBA6	0.104	0.226	0.272	0.375	0.591	1.16E-22
TMEM144	-0.097	0.67	0.904	0.123	0.714	2E-12
C19orf66	-0.307	0.282	0.505	0.265	0.743	1E-28
PI4K2A	0.219	0.093	-0.048	0.282	0.601	5.55E-09
GALNT10	0.258	0.021	0.002	0.29	0.801	2.44E-41
AMBRA1	0.207	0.236	0.13	0.312	0.646	7.31E-13
CHD7	0.379	-0.046	-0.219	-0.578	0.718	4.67E-28
BTBD2	0.054	-0.098	-0.082	0.27	0.585	4.56E-12

VPS35	0.27	0.017	0.025	0.459	0.623	4.99E-19
TMEM30A	0.224	0.28	0.16	0.418	0.708	7.88E-42
FGD6	0.339	0.307	0.163	0.261	0.659	1.79E-14
EAF2	0.133	-0.109	-0.067	-0.521	-1.294	7.27E-09
PCDHGB5	0.348	0.07	-0.081	0.085	0.704	2.18E-09
PCDHGB2	0.292	-0.133	-0.088	0.039	0.67	0.000142
SERTAD4	0.404	0.036	0.186	0.299	0.591	0.0124
BEX4	0.308	0.249	0.121	0.512	0.662	6.29E-11
CTPS2	-0.064	-0.181	0.072	0.406	0.662	1.42E-17
CHST7	-0.218	-0.179	-0.124	0.106	0.796	4.33E-14
FMN2	0.212	-0.032	-0.663	-0.491	-1.45	3.9E-12
FSTL5	-0.254	-0.067	0.317	0.226	0.673	6.62E-14
AGPAT3	0.129	0.009	0.125	0.363	0.59	4.5E-17
MAP3K7CL	-0.336	-0.194	-0.063	-0.303	0.581	1.18E-08
ARNTL2	0.066	0.138	0.236	0.305	0.669	1.92E-38
C16orf62	0.391	0.131	0.061	0.43	0.595	3.94E-10
CHMP1B	0.488	0.282	0.164	0.441	0.86	4.03E-49
RNPEPL1	0.165	-0.062	-0.005	0.171	0.77	5.68E-44
PELI1	0.1	0.025	0.174	0.28	0.762	3.32E-27
ASPHD2	-0.288	-0.138	0.019	0.266	0.748	1.12E-21
RALGAPA2	-0.257	-0.141	0.05	0.03	0.59	9.07E-17
RCN3	0.23	0.107	0.012	0.442	0.616	1.6E-11
SPC25	-0.399	-0.313	-0.249	0.081	-1.426	9.51E-53
ESYT2	0.313	0.094	0.008	0.156	0.587	5.87E-26
HECW2	0.315	0.232	0.336	0.219	0.651	2.47E-20
NCEH1	0.024	0.2	0.214	0.513	0.628	4.2E-22
SIPA1L2	0.565	0.643	0.239	0.407	0.737	4.8E-18
DOCK6	0.138	-0.026	0.256	0.11	0.821	6.2E-47
PCDH10	0.083	-0.004	-0.427	-0.253	-1.174	1.76E-38
CFAP97	0.059	0.156	0.358	0.484	0.653	1.68E-20
ZBTB4	0.138	0.043	0.195	0.409	0.749	3.76E-16
EPG5	-0.058	0.177	0.32	0.41	0.613	1.67E-25
MARK4	-0.003	0.08	0.013	0.096	0.586	4.68E-17
CXCL16	0.034	0.029	-0.058	0.219	0.594	0.0361
MESDC1	0.118	-0.146	-0.14	0.055	0.584	2.03E-24
CACNG6	0.325	-0.254	-0.424	-0.317	-1.123	1.42E-17
PLEKHA1	0.097	0.335	0.237	0.118	0.613	4.51E-22
SLC25A19	-0.341	-0.297	-0.267	-0.446	-1.105	1.15E-26

ZFAND3	0.396	0.223	0.096	0.419	0.605	1.79E-07
ROBO3	-0.437	0.126	0.23	-0.26	-1.134	1.7E-33
CREB3L2	0.006	-0.104	0.061	0.201	0.759	1.01E-27
FNDC3B	0.392	0.051	0.177	0.422	0.824	8.38E-23
CERK	0.232	-0.28	0.123	0.406	0.647	8.28E-60
RMND5A	0.336	0.128	0.073	0.296	0.627	1.26E-20
USP46	0.384	0.354	0.231	0.193	0.644	2.82E-15
RASAL3	-0.108	-0.053	0.186	0.066	0.609	1.93E-16
TMEM237	-0.198	-0.13	0.016	0.033	0.653	7.95E-21
KRI1	-0.507	-0.052	-0.031	-0.279	-0.976	1.36E-35
AACS	0.371	0.115	0.056	0.208	0.676	3.28E-16
GID4	0.165	0.346	0.227	0.302	0.612	3.74E-12
TMEM106C	-0.469	-0.082	0.111	-0.176	-1.107	4.01E-30
DSCC1	0.001	-0.059	-0.084	-0.163	-1.186	1.43E-14
BHLHE41	-0.398	0.12	-0.318	-0.284	-1.249	1.63E-07
SPAG16	0.067	-0.148	0.15	0.292	0.683	0.00000172
CHPF	0.274	0.126	-0.14	-0.046	-1.057	0.00000331
FAT4	0.15	-0.067	-0.507	-0.446	-1.112	8.32E-34
DYNC2H1	#N/A	#N/A	#N/A	#N/A	#N/A	#N/A
C11orf80	-0.311	0.007	0.407	0.167	0.607	4.42E-11
E2F8	-0.132	-0.264	-0.268	-0.319	-1.677	1.26E-72
THSD4	0.439	0.093	-0.292	-0.051	0.595	2.97E-19
FAM110D	-0.552	-0.216	-0.031	0.237	0.618	3.62E-07
SCD5	-0.035	-0.274	-0.179	0.121	0.747	3.56E-48
DSN1	-0.115	-0.02	-0.225	-0.403	-1.165	8.38E-87
SLC8B1	-0.105	0.039	0.156	0.136	0.589	3.25E-25
MYCT1	0.449	0.291	0.276	0.558	0.704	3.96E-35
CCDC92	0.361	0.228	0.286	0.355	0.72	5.79E-19
WDR26	0.019	0.148	0.181	0.211	0.648	4.41E-55
PIGZ	-0.52	-0.047	-0.15	-0.266	0.733	6.68E-15
PREX2	-0.167	-0.036	-0.225	-0.483	-1.229	6.12E-65
KCNIP4	-0.292	-0.465	-0.513	-0.187	-1.041	0.00183
TTYH3	0.236	0.032	-0.014	0.249	0.707	4.58E-19
COL18A1	0.313	0.08	-0.065	0.287	0.656	1.98E-10
CMIP	0.406	-0.159	0.087	0.517	0.775	5.06E-25
SH3BP5L	-0.052	0.144	0.112	0.268	0.589	2.02E-16
ITFG1	0.194	0.13	-0.007	0.4	0.698	6.95E-40
GDPD5	-0.397	-0.264	0.11	-0.189	0.648	2.31E-26

FBXO38	0.112	0.212	0.329	0.458	0.657	5.42E-35
TSPAN14	0.154	-0.282	-0.013	0.22	0.671	1.12E-54
TCF7L1	-0.026	-0.128	0.234	0.387	0.811	2.36E-28
PLVAP	0.296	-0.07	-0.275	0.245	-1.466	1.26E-23
AIF1L	-0.207	-0.154	0.174	0.365	0.839	3.42E-41
MARVELD1	0.066	0.075	0.085	0.258	0.617	4.55E-28
ARMC10	-0.125	-0.118	-0.001	-0.038	0.609	4.64E-21
TMTC1	-0.116	0.005	-0.344	-0.348	-2.268	4.39E-94
B3GNT5	-0.137	0.12	0.03	0.063	0.643	7.08E-18
MND1	-0.329	0.067	-0.163	-0.385	-1.267	1.86E-12
FAM161A	-0.351	-0.141	-0.111	-0.481	-1.311	7.96E-18
CAPNS2	-0.172	-0.354	-0.361	-0.532	-1.082	0.00704
FAM213A	-0.053	-0.092	0.099	0.314	0.648	1.13E-23
CYSTM1	0.044	0.044	0.124	0.218	0.638	2.34E-16
CARD11	0.217	-0.074	-0.078	0.261	0.76	1.41E-23
ZNF528	-0.235	0.081	0.304	0.126	0.59	2.46E-10
MCM8	0.003	0.135	-0.051	-0.321	-1.125	4.97E-75
PARD6G	0.072	0.649	0.534	0.527	0.822	0.0028
COL25A1	0.506	0.183	-0.004	0.004	-1.935	2.32E-69
GTPBP3	-0.446	-0.154	-0.02	-0.418	-0.969	6.61E-31
PLEKHA8	-0.25	0.207	0.29	0.207	0.625	2.47E-32
PLCD4	0.136	-0.056	0.122	0.08	0.647	0.00297
HPDL	0.196	-0.441	-0.705	-0.232	-1.13	2.96E-08
TMEM25	-0.387	-0.101	0.122	0.326	0.827	1.66E-42
RSPO3	-0.481	0.44	0.023	-0.186	-2.299	1.03E-79
TMTC4	-0.123	-0.059	0.131	0.142	0.743	1.12E-18
ZNRF1	0.234	0.301	0.16	0.267	0.78	7.48E-23
CEP19	0.013	0.031	0.067	0.316	0.69	0.00758
SMIM3	0.065	-0.071	-0.137	-0.143	0.751	0.00000264
COL27A1	-0.424	0.049	0.426	-0.377	0.581	3.92E-17
PAQR8	-0.242	-0.139	-0.002	0.182	0.656	8.06E-30
HELZ2	0.048	0.617	0.524	0.125	0.675	4.15E-27
TNKS1BP1	0.019	0.104	0.21	0.248	0.773	1.11E-38
NAV2	-0.125	-0.26	0.164	0.136	0.675	2.04E-33
SEC11C	-0.147	-0.138	-0.085	-0.175	-1.15	1.81E-18
ZNF697	0.143	0.386	0.323	0.313	0.601	2.79E-11
FMNL3	0.104	0.113	0.347	0.042	0.842	7.79E-36
CCDC74B	0.067	-0.165	-0.509	-0.547	-1.476	5.85E-14

SNX29	0.445	0.127	0.251	0.563	0.598	8.76E-08
ZNF585B	-0.154	0.493	0.47	0.436	0.594	0.000133
TIFA	0.173	0.366	-0.242	-0.447	0.645	3.31E-26
FAM114A1	0.41	0.172	0.179	0.394	0.625	1.17E-11
CCDC102A	-0.346	0.102	0.144	0.082	0.858	9.29E-18
ZNF561	0.087	0.645	0.754	0.331	0.82	5.47E-25
PLCD3	0.554	0.273	0.265	0.416	0.685	1.22E-16
KCTD12	0.321	-0.1	-0.376	-0.168	-1.187	3.39E-31
PCMTD1	0.148	0.306	0.294	0.134	0.747	1.3E-21
FCHO2	0.538	0.711	0.647	0.472	0.657	2.35E-27
HSPA12B	-0.33	-0.309	0.119	-0.044	0.791	1.01E-29
SLC18B1	-0.186	-0.341	-0.164	0.089	0.604	5.76E-16
SPPL3	0.075	0.133	0.199	0.426	0.657	7.33E-12
CMTM3	0.147	-0.113	0.06	0.179	0.642	1.41E-33
MFSD12	-0.023	0.001	-0.008	-0.079	0.666	1.03E-33
GALM	-0.052	-0.135	-0.066	0.373	0.652	0.0000155
DCBLD2	0.088	-0.138	-0.405	-0.191	0.771	1.06E-82
GLYCTK	-0.407	0.113	-0.003	-0.335	-1.037	2.52E-28
JMY	0.101	0.265	0.345	0.148	0.615	1.33E-10
TMEM171	0.093	-0.09	-0.029	0.071	-1.208	3.15E-28
PM20D2	0.058	0.308	0.353	0.46	0.799	8.29E-24
SPIN4	0.068	-0.393	-0.456	-0.249	-1.399	4.33E-40
NEK7	-0.206	-0.11	-0.366	-0.493	-1.339	3.55E-97
MIB2	-0.286	0.114	0.416	0.039	0.605	1.55E-13
EIF3LP2	0.314	0.047	0.154	0.514	0.707	2.37E-10
LDLRAD3	0.325	-0.094	0.029	0.327	0.63	3.12E-20
ST13P5	0.106	-0.043	-0.018	0.329	0.637	2.85E-10
HMGA1P3	0.13	-0.176	0.066	0.426	0.665	1.39E-08
MDP1	-0.238	-0.268	-0.198	-0.475	-0.971	0.00385
TICAM1	0.518	0.253	-0.031	0.172	0.59	3.39E-07
ZNF827	0.229	-0.055	0.358	0.385	0.653	8.53E-25
SHISA3	-0.536	-0.115	-0.549	-0.51	-2.817	1.36E-90
CREBRF	-0.125	0.484	0.206	-0.044	0.601	1.28E-10
CNKSR3	0.523	0.712	-0.058	-0.116	0.63	8.75E-21
GIMAP8	-0.477	0.157	-0.083	0.01	-0.945	2.04E-27
TMEM65	0.243	-0.016	0.047	0.318	0.725	7.74E-40
USP54	-0.155	-0.026	-0.145	0.078	0.796	5.46E-33
SPRED1	0.574	-0.081	-0.025	0.163	0.714	2.9E-36

ZNF846	-0.043	0.479	0.521	0.281	0.614	0.000816
ZNF579	0.103	0.262	0.211	0.056	0.72	0.00000038
FAM171B	0.204	0.266	0.159	0.13	0.618	1.28E-10
SCAMP5	-0.146	-0.137	0.154	0.5	0.709	5.38E-13
APOBEC3F	-0.416	-0.03	0.474	0.444	0.597	0.00000557
SPRED2	0.377	0.241	0.313	0.439	0.688	2.57E-08
CEP112	0.279	0.053	-0.003	0.171	0.623	6.53E-09
SLC25A43	0.209	0.12	0.186	0.344	0.587	2.26E-08
TRIML2	0.269	0.609	0.733	0.391	0.627	1.02E-08
DHRSX	-0.078	0.054	0.037	0.203	0.664	2.67E-17
ZCCHC24	0.179	-0.127	0.169	0.27	0.687	1.05E-10
SLC37A2	0.014	0.094	0.368	0.298	0.822	0.000698
ZNF438	-0.103	-0.086	0.376	0.567	0.713	3.8E-33
C10orf25	-0.38	-0.248	0.083	0.495	0.736	2.99E-09
REEP3	0.015	0.015	0.019	0.029	0.695	3E-33
JMJD1C	-0.291	0.081	0.2	0.133	0.752	7.63E-36
FAM171A1	0.21	0.015	-0.043	0.348	0.835	7.83E-33
SDK1	0.09	-0.1	0.049	0.064	-1.101	1.05E-29
PRR15	#N/A	#N/A	#N/A	#N/A	#N/A	#N/A
ANKRD18A	-0.079	-0.08	-0.012	-0.499	-1.258	5.48E-24
ZDHHC23	0.5	0.217	0.037	-0.354	-1.159	1.7E-33
RNF144B	-0.482	-0.213	-0.411	-0.303	-1.147	2.37E-31
SNX33	-0.166	0.062	0.065	0.321	0.783	2.54E-40
PGM2L1	0.328	0.367	0.166	0.398	0.822	3.45E-20
OTOGL	0.078	0.005	-0.538	-0.531	-3.275	7.91E-155
EXOC3L1	0.318	-0.166	-0.396	-0.485	-1.692	1.96E-39
NPW	#N/A	#N/A	#N/A	#N/A	#N/A	#N/A
FAM171A2	0.196	0.083	-0.363	-0.12	0.805	8.67E-17
KCTD1	0.049	0.106	0.066	0.373	0.678	3.63E-09
WDR62	-0.331	-0.274	-0.222	-0.579	-1.37	6.85E-50
RNF149	-0.066	-0.011	0.205	0.258	0.756	2.22E-37
SUMF1	0.153	0.109	0.093	0.435	0.743	5.13E-20
PRRT3	0.22	-0.348	0.119	0.377	0.674	9.2E-08
RNF180	-0.231	-0.067	-0.124	-0.052	0.625	0.000331
PPM1J	-0.423	0.199	0.378	0.148	0.684	0.000285
RAB43	-0.275	-0.043	-0.167	-0.091	0.568	0.00156
ZNF260	0.392	0.283	0.128	0.153	0.626	1.24E-12
SLC35E4	-0.05	0.029	0.026	0.079	0.651	3.17E-13

PGDP1	0.374	0.047	0.004	0.472	0.652	0.00000527
HNRNPCL1	0.446	-0.049	-0.118	0.232	0.594	1.05E-07
TMEM255B	-0.004	-0.041	0.079	0.114	0.618	3.15E-45
HLA-V	-0.011	-0.255	-0.041	0.22	0.701	0.000179
ZNF710	-0.079	-0.216	0.121	0.292	0.607	1.04E-20
SAMD5	0.01	0.247	0.251	0.408	0.626	5.69E-09
FRRS1	0.126	-0.047	-0.255	-0.551	-1.267	4.48E-14
C4orf48	-0.305	-0.184	-0.163	-0.207	0.702	3.9E-12
LINC00997	0.151	-0.116	-0.011	-0.036	0.597	0.019
MXRA7	0.083	0.029	0.057	0.202	0.656	8.58E-42
EIF2AK4	0.151	0.071	0.215	0.505	0.793	7.31E-32
ANKRD18B	0.212	0.012	-0.235	-0.313	-1.149	1.17E-21
NRARP	0.472	-0.237	-0.024	0.282	0.665	3.9E-20
FAM127C	0.361	0.039	0.03	0.446	0.688	1.12E-14
ACOT1	-0.098	-0.099	-0.047	0.249	0.64	0.00282
SMIM15	0.057	-0.054	0.056	0.391	0.686	1.1E-31
MAP1LC3B2	0.108	0.045	0.077	0.287	0.69	6.96E-19
HRCT1	0.128	0.052	-0.188	0.013	-1.53	7.8E-41
PEBP1P2	0.204	-0.3	-0.154	0.327	0.643	4.44E-15
LYPLA2P1	0.284	0.066	0.211	0.499	0.652	2.05E-12
C16orf52	-0.061	-0.06	-0.137	-0.535	-0.962	2.39E-29
RELL1	-0.087	0.183	0.182	0.173	0.658	9.07E-21
SLC25A21-A	-0.192	0.135	-0.179	-0.209	-1.429	8.42E-11
JMJD7	-0.434	-0.045	0.063	-0.511	-1.173	4E-17
MIR1204	#N/A	#N/A	#N/A	#N/A	#N/A	#N/A
HOTTIP	-0.427	0.15	0.36	0.413	0.623	0.00000941
PMF1-BGLA	-0.385	0.194	-0.363	-0.126	0.59	0.00743
MYZAP	0.256	-0.415	-0.021	0.114	0.748	1.17E-11
RNU6-301P	-0.293	0.409	0.293	-0.278	0.663	0.00104
ACVR1B	-0.096	0.101	0.028	-0.172	-0.664	1.69E-29
ACYP1	-0.224	-0.199	-0.009	-0.147	-0.812	3.06E-16
ADRA1B	0.417	-0.525	-0.711	-0.283	-0.769	1.79E-17
ALCAM	0.113	0.106	-0.106	0.162	-0.617	3.75E-19
ALDH1A1	0.125	0.056	-0.023	0.54	-0.718	5.56E-21
ALDOC	0.193	0.066	-0.218	0.048	-0.852	8.15E-12
ANG	0.268	-0.093	-0.353	-0.113	-0.633	0.00086
APBA1	0.012	-0.326	-0.487	-0.33	-0.799	4.25E-10
ATP6V0B	-0.148	-0.111	-0.158	-0.389	-0.704	4.97E-18

HCN2	0.31	0.016	-0.136	-0.144	-0.82	8.29E-08
BMP6	0.148	0.073	-0.165	0.09	-0.717	5.49E-19
BRCA1	0.028	0.14	0.021	0.027	-0.579	3.85E-16
BRCA2	0.082	0.052	-0.186	-0.309	-0.823	9.25E-36
CA12	0.256	-0.021	-0.177	-0.065	-0.889	1.46E-09
CBLB	-0.315	-0.097	-0.519	-0.451	-0.691	1.62E-14
CCNA2	-0.504	-0.78	-0.736	-0.267	-0.755	1.76E-48
ABCC2	-0.059	0.027	-0.028	-0.303	-0.819	0.000214
DCK	-0.013	-0.172	-0.312	-0.507	-0.704	1.07E-18
AKR1C1	-0.085	-0.225	-0.162	-0.097	-0.696	0.0000194
TIMM8A	-0.163	-0.042	0.022	-0.305	-0.769	3.38E-15
DHODH	-0.054	-0.033	-0.137	-0.235	-0.894	1.04E-17
TRDMT1	-0.061	-0.232	-0.104	-0.331	-0.815	1.08E-07
EMP1	0.387	-0.205	-0.33	-0.078	-0.645	2.75E-36
EPS8	0.287	0.339	-0.004	0.023	-0.722	6.32E-21
FANCG	-0.08	-0.08	-0.213	-0.408	-0.943	1.42E-30
GALC	0.011	0.059	-0.137	0.182	-0.629	2.23E-18
GHR	-0.084	-0.292	-0.337	-0.348	-0.642	0.000079
GK	-0.432	-0.012	-0.008	-0.161	-0.695	0.00015
GCLM	0.324	0.135	-0.109	0.046	-0.675	4.29E-23
GOLGA1	-0.329	-0.086	0.098	-0.219	-0.586	1.69E-15
GSTA4	-0.04	-0.132	-0.219	-0.204	-0.839	1.23E-16
H1F0	-0.194	-0.798	-0.386	0.026	-0.898	5.35E-45
H2AFX	-0.526	-0.741	-0.723	-0.366	-0.877	1.23E-26
HAS3	-0.287	0.111	-0.12	-0.508	-0.916	2.66E-10
HGF	-0.051	0.329	0.039	-0.116	-0.733	0.000439
HMGB2	-0.331	-0.69	-0.632	-0.279	-0.707	5.46E-29
HMGN2	-0.201	-0.097	-0.072	-0.251	-0.81	1.8E-13
HOXD1	0.183	0.438	-0.053	-0.094	-0.635	2.2E-21
IGF2	0.231	-0.019	-0.231	-0.115	-0.633	0.0012
IGHMBP2	-0.308	0.14	-0.089	-0.423	-0.677	3.77E-33
INHBA	0.145	0.28	0.016	0.068	-0.599	2.55E-30
IRF3	-0.271	-0.021	0.031	-0.194	-0.597	5.89E-14
KCNMB1	0.161	-0.098	-0.022	-0.347	-0.664	0.0000115
KIF11	-0.166	-0.343	-0.482	0.06	-0.575	3.03E-11
KIFC1	-0.573	-0.574	-0.404	-0.169	-0.901	4.33E-26
LIPE	-0.515	-0.46	-0.603	-0.452	-0.825	0.00000324
MC1R	-0.144	0.193	0.287	-0.495	-0.697	1.16E-08

MCM2	0.487	0.072	-0.443	-0.373	-0.851	1.01E-10
MCM4	0.538	0.034	-0.495	-0.496	-0.635	2.57E-26
MCM5	0.449	0.034	-0.409	-0.269	-0.797	4.04E-17
MEIS1	-0.57	-0.072	-0.369	-0.39	-0.796	2.82E-18
MGST1	0.042	-0.01	0.009	-0.068	-0.97	1.07E-15
MMP16	0.051	-0.239	-0.466	-0.527	-0.617	3.28E-23
MOCS2	-0.196	0.021	0.119	-0.173	-0.619	1.99E-08
MT1X	0.107	-0.03	-0.177	-0.171	-0.807	7E-15
MUTYH	-0.48	-0.075	-0.042	-0.444	-0.869	1.35E-15
NDUFB9	-0.251	-0.086	-0.063	-0.482	-0.614	0.000396
NDUFC2	0.005	-0.181	-0.276	-0.279	-0.76	2.16E-18
NDUFV2P1	-0.188	-0.227	-0.215	-0.272	-0.615	2.53E-08
NQO2	-0.151	-0.091	-0.051	-0.207	-0.73	5.01E-14
NOS3	-0.045	-0.427	-0.23	-0.04	-0.627	4.17E-15
NRCAM	0.273	0.074	-0.265	-0.043	-1.036	2.63E-26
CNTN3	0.203	-0.051	-0.377	-0.408	-0.673	0.00000378
PCNT	-0.278	0.079	0.056	-0.504	-0.784	1.01E-32
PDE3A	-0.427	0.181	0.074	-0.047	-0.83	5.19E-45
PFAS	-0.079	-0.039	-0.133	-0.165	-0.594	6.52E-14
ABCB1	0.051	0.453	0.074	0.27	-0.597	8.77E-07
PHKA1	0.027	-0.103	-0.156	-0.145	-0.752	1.34E-16
PHKG2	-0.212	-0.038	-0.243	-0.57	-0.686	6.6E-20
PIGC	-0.186	-0.219	-0.254	-0.244	-0.592	0.000061
PIGF	-0.225	-0.107	-0.075	-0.349	-0.649	5.88E-07
PIGH	-0.203	-0.008	0.084	-0.18	-0.733	5.62E-11
PLCL1	-0.328	-0.002	-0.897	-0.49	-0.63	2.66E-22
PMM1	-0.376	-0.147	-0.009	-0.521	-0.673	1.15E-14
EXOSC9	0.132	0.066	-0.195	-0.371	-0.824	1.92E-56
PNN	-0.177	-0.09	-0.012	-0.532	-0.584	2.7E-12
POLE2	0.016	-0.076	-0.215	-0.403	-1.025	3.05E-28
POLR2G	-0.122	-0.104	-0.034	-0.2	-0.602	8.57E-17
PSMA6	-0.233	-0.073	-0.075	-0.393	-0.597	0.00033
RAD1	0.078	-0.003	-0.23	-0.342	-0.658	2.76E-32
PEX2	-0.211	-0.311	-0.237	-0.392	-0.837	6.62E-09
RABGGTB	0.06	0.084	0.085	-0.33	-0.691	6.02E-16
RFC2	0.286	0.034	-0.355	-0.179	-0.7	7.37E-28
RFC3	0.258	0.165	-0.461	-0.432	-0.841	1.6E-61
RFC4	0.195	-0.11	-0.26	-0.356	-0.847	9.9E-50

RFC5	0.083	0.035	-0.212	-0.299	-0.96	1.56E-57
SNORA63	-0.48	-0.075	-0.138	-0.562	-1.059	0.000165
RPA3	-0.329	-0.052	0.05	-0.456	-0.692	0.00000363
RPS29	#N/A	#N/A	#N/A	#N/A	#N/A	#N/A
RRM2	0.481	0.348	0.207	0.341	-0.84	4.75E-46
SDC2	0.275	-0.05	-0.423	-0.335	-0.891	1.76E-24
TRA2B	-0.035	-0.148	-0.276	-0.351	-0.8	8.71E-46
SGK1	0.285	0.012	-0.16	-0.251	-0.668	1.31E-17
STIL	-0.09	-0.393	-0.449	-0.292	-0.71	1.04E-23
SKP2	-0.319	-0.282	-0.331	-0.302	-0.812	1.06E-18
SLC1A1	0.069	-0.149	-0.377	-0.226	-0.603	1.24E-08
SLC1A5	0.271	-0.11	-0.375	-0.173	-0.635	2.4E-14
SNCA	0.028	-0.162	-0.281	-0.138	-0.969	2.92E-47
SNRPA1	-0.065	0.094	0.037	-0.086	-0.719	7.97E-29
SNRPG	-0.211	0.108	0.085	-0.434	-0.575	0.00828
SPAG4	-0.089	-0.011	0.01	-0.373	-0.698	7.51E-08
STAC	#N/A	#N/A	#N/A	#N/A	#N/A	#N/A
HSPA13	0.17	-0.027	-0.292	-0.264	-0.638	1.01E-30
STX1A	-0.077	-0.353	-0.145	-0.539	-0.85	1.78E-15
SUV39H1	-0.119	-0.24	-0.398	-0.133	-0.825	1.59E-22
TAGLN	0.268	0.042	0.149	-0.09	-1.029	1.11E-17
TCF7	0.332	0.071	-0.457	-0.45	-0.69	2.21E-07
TFAP4	-0.337	-0.35	-0.229	0.081	-0.891	1.27E-09
TGFBR3	-0.024	-0.12	-0.261	-0.265	-0.864	4.09E-25
THBS1	0.212	0.033	-0.255	-0.194	-0.64	1.83E-18
TLR4	0.157	-0.181	-0.468	-0.378	-0.612	1.72E-23
TMPO	-0.03	-0.304	-0.429	-0.068	-0.865	1.73E-35
TOP3A	-0.058	-0.051	-0.215	-0.303	-0.626	7.74E-19
TXNRD1	0.378	0.149	-0.216	-0.095	-0.664	1.17E-17
UPP1	-0.237	-0.014	0.033	-0.152	-0.599	3.18E-18
USP1	0.462	0.05	-0.223	-0.058	-0.837	3.33E-83
ZFP36	-0.229	0.228	-0.301	-0.394	-0.81	2.89E-17
ZNF18	-0.39	-0.011	-0.178	-0.499	-0.872	3.8E-12
ZBTB25	-0.53	0.081	0.176	-0.406	-0.6	9.42E-12
ALMS1	-0.047	0.091	-0.067	-0.251	-0.734	1.39E-13
MALL	0.395	-0.206	-0.424	-0.171	-0.635	6.06E-21
HSD17B8	-0.226	0.035	-0.111	-0.374	-0.747	0.000785
YEATS4	-0.159	-0.152	-0.129	-0.337	-0.723	0.00000768

SLC7A5	-0.155	-0.339	0.023	0.008	-0.643	8.47E-12
CHAF1B	0.553	0.225	-0.276	-0.371	-0.819	3.39E-42
HIST2H2AC	#N/A	#N/A	#N/A	#N/A	#N/A	#N/A
HIRIP3	0.058	-0.002	-0.032	0.248	-0.794	1.02E-16
RGS5	0.083	-0.09	-0.165	-0.097	-0.863	4.71E-08
FCN3	0.308	-0.031	-0.264	0.109	-0.763	1.06E-08
PLPP1	0.27	0.177	-0.091	-0.045	-0.621	2.07E-15
STC2	-0.192	-0.235	0.001	0.203	-0.725	9.67E-38
JRK	-0.557	-0.195	-0.074	-0.543	-0.763	1.96E-20
FPGT	-0.515	-0.092	-0.066	-0.193	-0.684	2.61E-10
TNFRSF11A	0.151	-0.005	-0.228	-0.326	-1.091	1.33E-30
IL18R1	0.281	0.206	-0.164	-0.582	-0.603	1.99E-20
SLC5A6	-0.172	-0.183	-0.271	-0.485	-0.862	1.11E-51
TIMELESS	0.006	0.037	-0.07	-0.116	-0.819	3.89E-54
SNORD73A	-0.213	0.28	0.206	-0.532	-0.65	0.0000698
RRP9	0.164	-0.155	-0.361	-0.081	-0.614	1.02E-09
EBAG9	-0.031	-0.004	0.037	-0.048	-0.752	1.78E-17
STK17B	-0.041	-0.438	-0.676	-0.506	-1.057	4.67E-39
SLC9A3R2	0.371	-0.15	-0.443	-0.413	-0.862	2.29E-57
STOML1	-0.242	-0.179	-0.15	-0.057	-0.967	1.59E-25
ATP6V1G1	-0.168	-0.088	-0.161	-0.542	-0.711	2.01E-08
MTRF1	-0.564	0.044	0.048	-0.347	-0.654	2.04E-08
TTLL4	-0.485	-0.233	-0.159	-0.432	-0.653	1.44E-28
KNTC1	-0.269	0.153	0.057	-0.28	-0.871	2.32E-66
CCP110	0.032	0.054	-0.055	-0.117	-0.611	3.48E-22
KIAA0513	0.147	0.155	-0.052	-0.008	-0.773	2.59E-09
DNAJC6	0.476	-0.075	-0.248	-0.349	-0.731	3.93E-17
NUAK1	0.228	0.112	-0.33	-0.491	-0.658	4.3E-33
TELO2	-0.088	-0.051	-0.24	-0.548	-0.674	4.98E-17
DENND4B	-0.418	-0.193	-0.047	-0.48	-0.618	2.63E-21
DCLRE1A	-0.301	-0.235	-0.195	-0.113	-0.774	4.49E-18
RBX1	-0.329	-0.173	-0.148	-0.473	-0.629	0.000032
HDAC6	-0.185	0.072	0.207	-0.041	-0.596	1.46E-21
CHAF1A	0.453	0.004	-0.464	-0.548	-0.718	2.76E-45
ABCB6	-0.359	0.263	0.276	0.205	-0.934	4.29E-23
NR1H3	-0.371	-0.18	0.13	0.037	-0.731	0.0000671
COX17	-0.202	-0.093	-0.012	-0.541	-0.789	1.47E-08
IL18BP	-0.086	0.037	-0.003	-0.415	-0.64	4.19E-18

TSPAN2	-0.137	0.109	0.02	-0.105	-1.071	5.14E-14
FRY	-0.451	-0.28	-0.328	-0.228	-0.603	2.83E-14
FAM13A	-0.566	-0.006	-0.055	-0.297	-0.695	2.98E-14
LPCAT3	0.079	0.123	-0.012	0.191	-0.612	9.13E-19
RNF41	-0.048	-0.01	-0.306	-0.261	-0.926	9.63E-51
PRMT3	-0.119	-0.148	-0.039	0.109	-0.591	2.38E-11
MPHOSPH9	-0.362	-0.231	-0.094	-0.211	-0.724	2.11E-21
FLOT1	0.105	-0.185	-0.282	-0.086	-0.617	2.05E-23
DDX39A	-0.067	-0.172	-0.265	-0.243	-0.716	1.61E-35
SLC35B1	0.155	0.031	-0.126	-0.132	-0.9	1.02E-82
DLEU1	-0.315	-0.323	-0.3	-0.351	-0.831	0.00000137
APBB3	-0.206	0.278	0.426	-0.408	-0.622	9.86E-12
TCIRG1	-0.264	0.146	0.046	-0.486	-0.606	8.28E-18
ANAPC10	-0.143	-0.03	-0.012	-0.279	-0.759	5.15E-07
EMG1	0.143	-0.001	0.044	0.019	-0.686	6.74E-29
TACC3	-0.378	-0.644	-0.525	-0.318	-0.699	3.66E-21
NOP56	-0.272	-0.183	-0.225	-0.555	-0.906	1.2E-54
SIVA1	-0.312	-0.194	-0.24	-0.517	-0.775	4.02E-11
SPAG5	-0.351	-0.506	-0.489	-0.255	-0.655	1.26E-17
TAF6L	-0.116	-0.073	-0.229	-0.252	-0.63	2.47E-09
POSTN	0.212	0.188	-0.047	0.307	-1.011	3.5E-15
POLD3	0.195	0.124	-0.097	-0.326	-1.068	1.56E-83
PLK4	-0.164	-0.31	-0.24	-0.328	-0.873	1.25E-22
RAI2	0.502	0.022	-0.307	-0.325	-0.744	2.75E-21
ILVBL	-0.116	-0.121	-0.295	-0.141	-0.721	3.88E-21
STON1	-0.354	-0.006	-0.155	-0.096	-0.772	4.89E-09
CYB561D2	0.001	-0.151	-0.108	0.006	-0.694	7.15E-19
ZWINT	-0.02	0.035	-0.062	-0.099	-1.051	3.36E-93
KAT7	0.126	-0.079	-0.334	-0.224	-0.8	1.91E-21
DMC1	0.095	0.05	-0.119	-0.179	-0.695	0.00153
FICD	-0.047	0.094	-0.277	-0.431	-0.819	3.91E-20
LSM6	-0.213	-0.21	-0.101	-0.381	-0.818	6.06E-07
PNKP	-0.363	-0.081	0.003	-0.523	-0.66	2.54E-15
OIP5	-0.511	-0.642	-0.299	-0.11	-0.59	0.0000484
EXOSC8	-0.199	-0.132	-0.218	-0.353	-0.908	6.42E-26
BTBD3	0.236	0.021	-0.344	-0.352	-0.804	4.13E-35
DKK1	0.409	0.302	-0.019	0.051	-0.889	2.38E-55
PAXIP1	0.17	0.054	-0.113	-0.413	-0.636	1.62E-33

CAMTA2	-0.373	-0.044	0.104	-0.249	-0.601	3.45E-30
SULF1	-0.15	0.033	-0.016	0.324	-1.044	1.76E-23
SYNE2	-0.225	0.147	-0.187	-0.296	-0.966	1.28E-19
DNAJC9	0.279	0.073	-0.148	-0.178	-0.727	1.61E-74
NCAPH	0.006	-0.315	-0.394	0.085	-0.597	2.99E-09
EXOSC2	-0.071	-0.137	-0.223	-0.231	-0.606	5.93E-13
ITGB3BP	-0.452	0.035	0.159	-0.427	-0.822	1.78E-07
SLC44A1	0.311	0.005	-0.259	-0.036	-0.656	6.74E-45
ABCA6	-0.239	0.101	0.069	-0.253	-0.664	2.91E-19
PDSS1	0.15	0.009	-0.242	-0.351	-0.773	2.14E-16
SSBP2	-0.205	-0.44	-0.573	-0.537	-1.062	3.2E-11
LSM5	-0.517	-0.263	-0.156	-0.564	-0.594	0.000191
SDF2L1	-0.041	-0.304	-0.4	-0.368	-0.989	2.68E-26
IL17RA	0.134	-0.186	-0.255	-0.257	-0.581	7.82E-18
CLDN15	-0.507	-0.035	0.408	-0.566	-0.745	9.81E-22
RHBDD3	-0.165	0.076	-0.184	-0.344	-0.775	8.24E-19
ASF1A	-0.036	-0.228	-0.439	-0.298	-0.615	6.74E-14
MPC2	-0.154	-0.067	-0.163	-0.37	-0.598	9.16E-10
ZNF473	-0.566	-0.103	-0.437	-0.574	-0.713	1.66E-14
ANAPC15	-0.012	-0.025	-0.131	-0.212	-0.59	4.85E-20
ACOT11	0.536	-0.113	-0.307	-0.139	-0.657	9.22E-20
FBXW4P1	0.23	-0.031	-0.311	-0.526	-0.648	0.0000268
FAM162A	-0.235	-0.19	-0.071	-0.269	-0.584	0.000311
TIMM8B	-0.31	-0.238	-0.101	-0.418	-0.609	0.0000637
SNORD60	0.204	0.301	0.161	-0.401	-0.887	0.0000793
TRMT2A	-0.351	0.101	-0.003	-0.326	-0.692	1.17E-26
MTBP	-0.16	-0.12	-0.266	-0.266	-0.661	2.21E-08
CIDEB	0.136	-0.199	-0.426	-0.454	-0.778	0.00745
SULT1B1	-0.058	-0.026	-0.08	0.071	-0.979	2.41E-87
TMEM97	0.172	-0.113	-0.48	-0.52	-0.649	7.88E-46
TRIB2	0.129	-0.089	-0.243	-0.078	-0.675	1.65E-11
MRPS28	-0.018	-0.175	-0.249	-0.297	-0.725	6.63E-13
MAGEH1	0.153	-0.101	-0.293	-0.095	-0.649	2.89E-21
ASTE1	0.22	0.077	-0.227	-0.133	-0.62	3.87E-10
MRPL13	-0.177	-0.072	-0.024	-0.254	-0.586	0.0000381
ATAD2	0.436	0.068	-0.403	-0.426	-0.712	1.19E-35
NDUFAF4	-0.117	-0.218	-0.284	-0.377	-0.655	1.44E-07
TIMM21	-0.04	-0.011	-0.046	-0.106	-0.616	9.89E-18

TMEM208	-0.073	-0.181	-0.228	-0.29	-0.619	7.15E-10
N6AMT1	-0.468	-0.105	0.015	-0.386	-0.719	1.79E-14
NCAPH2	0.168	0.123	-0.031	-0.157	-0.885	4.97E-52
ALG5	-0.134	-0.179	-0.172	-0.353	-0.802	8.3E-11
PSMC3IP	0.288	0.108	-0.188	-0.582	-0.825	1.88E-40
GMPPB	0.096	-0.002	0.017	0	-0.608	7.96E-24
ALG6	-0.122	0.046	0.045	0.081	-0.598	1.12E-18
DONSON	0.179	0.171	-0.054	-0.11	-0.676	1.37E-41
SLC2A8	0.047	-0.188	-0.383	-0.482	-0.772	2.87E-25
PILRA	-0.547	0.099	0.33	-0.511	-0.553	0.0000681
CXXC1	-0.194	-0.112	-0.155	-0.317	-0.651	8.08E-22
NT5C	-0.016	0.071	-0.303	-0.353	-0.785	1.06E-11
ZNRD1	-0.445	-0.111	-0.089	-0.28	-0.67	1.49E-08
MTERF3	-0.285	-0.121	-0.292	-0.306	-0.596	2.82E-13
TPRKB	-0.434	-0.141	-0.113	-0.49	-0.794	0.00000466
MED31	-0.029	0.11	-0.013	-0.531	-0.929	4.77E-09
RRP15	-0.209	-0.047	-0.097	-0.172	-0.835	9.43E-33
MRPS18C	-0.21	-0.057	0.044	-0.254	-0.588	0.0000639
DERA	0.075	0.06	-0.158	-0.26	-0.743	5.4E-33
TUBE1	-0.564	-0.229	-0.008	-0.348	-0.703	2.09E-08
HSPA14	0.01	0.001	-0.203	-0.464	-0.803	3.12E-10
CKLF	-0.482	-0.335	-0.165	-0.555	-0.663	0.0000185
NUSAP1	-0.384	-0.357	-0.372	-0.147	-0.588	8.57E-18
ANKRD39	-0.066	-0.145	-0.234	-0.187	-0.661	0.00000178
COX16	-0.206	-0.156	-0.081	-0.477	-0.57	0.000273
ANKMY1	-0.205	-0.223	-0.097	-0.472	-0.875	3.72E-14
CEND1	-0.034	-0.168	-0.208	0.084	-0.639	0.0000879
THEM6	0.225	-0.298	-0.502	-0.261	-0.647	6.86E-17
POP5	-0.252	-0.128	-0.201	-0.235	-0.946	2.25E-21
POMP	-0.096	-0.092	-0.098	-0.31	-0.673	2.74E-07
NOP16	0.02	-0.142	-0.306	-0.161	-0.614	7.28E-20
NCKIPSD	-0.304	-0.059	-0.069	-0.16	-0.581	0.00000014
JKAMP	-0.207	-0.222	-0.236	-0.453	-0.66	1.02E-07
PHF7	-0.457	0.066	0.235	0.168	-0.69	2.67E-10
MTFP1	-0.17	-0.162	-0.111	-0.08	-0.61	0.00000106
SRRT	0.108	-0.069	-0.164	-0.385	-0.6	1.76E-37
PPIL1	-0.032	-0.247	-0.167	-0.09	-0.653	4.14E-13
MPC1	-0.304	-0.165	-0.158	-0.494	-0.837	3.12E-10

HSPB11	-0.374	0.008	0.005	-0.493	-0.645	0.00000279
POLR3K	-0.22	-0.305	-0.479	-0.49	-0.704	1.34E-17
PPIL3	-0.18	-0.198	-0.016	-0.15	-0.671	0.00000524
MIS18A	-0.095	-0.176	-0.32	-0.115	-0.606	5.04E-11
CYCS	0.025	0.001	-0.01	-0.273	-0.694	2.03E-14
GDAP1	0.504	0.412	0.148	0.145	-0.807	0.00000199
SLC38A2	0.201	-0.037	-0.263	-0.435	-0.795	3.5E-24
ASNSD1	-0.129	-0.034	-0.12	-0.336	-0.596	4.88E-07
NDFIP2	-0.146	-0.115	-0.111	-0.23	-0.635	0.0000987
AHI1	-0.165	0.061	0.054	-0.364	-0.746	8.4E-17
ZCCHC10	-0.394	-0.186	-0.143	-0.509	-0.616	0.00000897
ERCC6L	0.051	-0.252	-0.292	0.11	-0.621	2.37E-10
ANKRD49	-0.292	0.099	0.043	-0.465	-0.678	2.13E-10
MTMR10	-0.234	0.049	-0.201	-0.403	-1.054	6.34E-55
SEMA4C	-0.572	-0.055	-0.489	-0.584	-0.835	8.82E-23
C1orf27	-0.073	0.076	-0.076	-0.426	-0.604	1.53E-09
TIPIN	0.266	0.014	-0.34	-0.488	-1.044	3.4E-44
C1orf159	-0.336	0.04	-0.085	-0.479	-0.634	3.63E-11
MAGOHB	-0.211	-0.079	-0.119	-0.453	-0.888	2.04E-11
FIGN	-0.479	-0.424	-0.372	-0.35	-0.737	6.07E-12
MSTO1	-0.129	-0.175	-0.087	-0.186	-0.674	1.31E-09
CENPQ	0.016	0.067	-0.305	-0.465	-0.786	2.27E-11
LINS1	-0.068	0.207	-0.074	-0.51	-0.752	1.76E-20
FANCI	0.084	0.21	0.135	0.229	-0.602	3.31E-35
LRRC1	0.306	0.111	-0.385	-0.487	-0.906	7.91E-30
RNF121	0.02	-0.098	-0.298	-0.361	-1.091	1.98E-79
MNS1	-0.066	-0.088	-0.176	0.131	-0.941	0.000154
ZNF823	-0.262	0.137	-0.146	-0.342	-0.599	0.0000165
FOXRED1	-0.045	-0.005	-0.061	-0.211	-0.681	6.01E-19
SYBU	0.005	-0.237	-0.429	-0.471	-0.807	0.00000147
LIMS2	0.11	-0.183	-0.152	-0.162	-0.837	3.91E-34
TRMU	-0.226	-0.015	-0.135	-0.405	-0.602	1.78E-13
CEP72	-0.249	0.062	-0.169	-0.323	-0.922	2.02E-10
SPATA7	-0.476	0.183	0.009	-0.224	-0.713	2.5E-23
CENPJ	-0.146	-0.027	-0.126	-0.572	-0.755	2.46E-19
PBK	-0.401	-0.532	-0.512	-0.236	-0.891	1.31E-27
NXT2	0.067	0.112	-0.063	-0.152	-0.658	3.56E-07
GPCPD1	0.009	0.007	-0.054	-0.342	-0.742	7.6E-17

PPAN	-0.286	-0.037	-0.059	-0.408	-0.608	0.0000121
PANX2	0.001	0.098	-0.053	-0.067	-0.758	7.67E-07
SEMA3G	0.457	-0.114	-0.217	-0.304	-0.581	5.67E-09
DUS3L	-0.006	-0.048	-0.119	-0.368	-0.628	8.39E-13
SMCO4	0.149	-0.181	-0.386	-0.478	-0.589	1.85E-09
SDR39U1	-0.398	0.062	0.063	-0.324	-0.577	6E-15
RGL3	-0.328	0.071	0.471	-0.45	-0.587	1.21E-10
PHTF2	-0.121	-0.064	-0.322	-0.5	-0.727	9.22E-35
DOLPP1	-0.091	-0.016	-0.252	-0.224	-0.876	1.83E-27
C3orf14	-0.267	0.019	0.058	-0.292	-0.659	0.00000273
STAMBPL1	0.302	-0.116	0.469	0.547	-0.599	8.1E-08
CEP126	-0.066	-0.14	-0.067	-0.234	-0.72	0.000026
PREX1	0.345	-0.273	-0.469	-0.25	-0.686	8.78E-19
STIM2	0.002	-0.225	-0.248	-0.259	-0.663	1.29E-16
UVSSA	-0.367	0.023	0.245	-0.445	-0.702	8.27E-19
DDX55	-0.234	-0.059	-0.078	-0.477	-0.653	4.39E-14
LSM2	-0.281	-0.242	-0.086	-0.337	-0.836	1.04E-08
SRPRB	0.149	-0.157	-0.249	-0.24	-0.797	4.26E-61
PCTP	0.112	-0.21	-0.528	-0.497	-1.004	8.65E-22
ZNF77	-0.236	-0.131	-0.247	-0.511	-0.689	0.000182
NIF3L1	-0.238	-0.397	-0.112	0.075	-0.718	4.79E-15
SCOC	-0.21	-0.144	-0.085	-0.368	-0.58	0.000167
NECAB3	-0.138	0.008	-0.001	-0.542	-0.65	5.17E-17
FKBPL	-0.344	0.002	-0.162	0.167	-0.627	5.48E-07
CLSPN	0.572	0.267	-0.054	-0.213	-0.579	9.35E-38
FIGNL1	-0.127	0.023	-0.113	-0.363	-0.64	2.59E-35
CENPK	-0.386	0.001	0.024	-0.225	-0.669	2.82E-14
NCAPG	-0.272	-0.472	-0.527	-0.167	-0.776	1.15E-38
TOR3A	-0.093	-0.085	-0.188	-0.175	-0.722	4.7E-19
IKZF4	0.069	-0.085	0.015	-0.397	-0.668	1.68E-08
ATG3	-0.097	-0.136	-0.207	-0.241	-0.68	3.43E-26
METTL17	-0.259	-0.082	-0.104	-0.479	-0.743	7.36E-13
GINS3	0.522	-0.1	-0.488	-0.332	-0.939	6.16E-57
NOL6	0.15	-0.011	-0.103	-0.175	-0.678	5.95E-23
C17orf53	-0.05	-0.054	-0.16	-0.305	-0.626	0.0000592
AUNIP	0.238	-0.288	-0.612	-0.425	-0.808	5.77E-14
CENPM	-0.018	-0.072	-0.166	0.027	-0.686	6.08E-09
ELOVL6	0.104	-0.287	-0.361	-0.247	-0.87	1.25E-17

CHCHD7	-0.148	0.052	-0.132	-0.426	-0.674	1.08E-10
DHRS11	-0.076	-0.112	-0.326	-0.466	-0.591	0.0000056
CENPO	0.039	-0.297	-0.427	-0.331	-0.595	7.86E-23
CRELD2	0.17	-0.085	-0.263	-0.032	-0.691	8.17E-18
THOC6	-0.135	-0.277	0.014	-0.042	-0.725	6.44E-18
HAUS3	-0.16	0.016	-0.162	-0.376	-0.936	1.63E-27
PAGR1	-0.25	-0.353	-0.239	-0.047	-0.973	9.43E-11
NAA16	-0.054	-0.055	-0.134	-0.579	-0.586	2.75E-19
GALNT14	-0.098	-0.11	-0.34	-0.035	-0.83	0.00394
C22orf46	-0.547	0.011	0.469	-0.207	-0.804	1.25E-16
CENPU	0.127	0.13	-0.019	-0.35	-0.709	1.09E-17
YRDC	0.205	-0.155	-0.342	-0.39	-0.595	2.91E-22
SUV39H2	-0.209	-0.139	-0.279	-0.425	-0.779	5.77E-16
SH3D21	-0.219	0.207	0.22	-0.54	-0.663	1.38E-13
TEFM	-0.387	-0.185	-0.085	-0.259	-0.629	0.00000679
GEMIN7	-0.355	-0.216	-0.108	-0.344	-0.604	0.00000341
GEMIN6	-0.49	-0.282	-0.136	-0.209	-0.778	2.09E-11
CSPP1	-0.384	-0.222	-0.238	-0.504	-0.589	0.00000563
RPP21	0.003	0.091	0.045	-0.117	-0.63	0.000207
L2HGDH	0.007	0.002	0.033	-0.144	-0.738	1.25E-11
JADE1	-0.478	-0.296	-0.237	-0.202	-0.882	8.06E-33
RMI1	-0.062	-0.008	-0.265	-0.431	-0.957	3.4E-43
SEMA6D	-0.516	0.779	0.337	-0.125	-0.603	1.16E-42
ULBP2	0.365	0.223	-0.049	0.062	-0.767	1.27E-18
SLC35G2	-0.098	0.064	-0.083	-0.207	-0.685	1.73E-12
THAP7	-0.254	-0.155	-0.316	-0.176	-0.876	1.5E-16
ANP32E	0.151	-0.093	-0.239	0.08	-0.757	6.02E-16
SLC25A28	-0.004	0.046	-0.081	-0.503	-0.9	1.98E-22
AKR1E2	-0.541	-0.264	-0.26	-0.493	-0.623	0.00503
BRIP1	0.281	0.33	-0.149	-0.477	-0.777	1.94E-58
SLC10A7	0.013	0.036	-0.121	-0.231	-0.834	1.11E-27
ATRIP	-0.064	0.133	-0.112	-0.277	-0.86	1.34E-32
UTP15	-0.061	-0.053	-0.324	-0.279	-0.625	6.58E-14
FAM96A	-0.33	-0.103	-0.069	-0.37	-0.622	0.00042
POMK	-0.245	-0.338	-0.361	-0.389	-0.65	0.00188
TMEM126A	-0.273	-0.147	-0.113	-0.466	-0.754	0.00000135
ZCCHC9	-0.343	-0.069	-0.011	-0.345	-0.68	1.66E-10
SLF1	0.047	-0.224	-0.513	-0.48	-0.81	5.69E-18

EIF1AD	0.235	0.105	-0.05	-0.162	-0.586	3.02E-39
TMEM175	-0.306	0.013	0.023	-0.248	-0.803	5.07E-21
UQCC2	-0.369	-0.217	-0.159	-0.427	-0.62	0.000192
CMSS1	-0.098	-0.01	0.011	-0.041	-0.8	4.13E-17
DOT1L	0.019	0.025	-0.152	-0.531	-0.83	3.25E-44
SLX4	-0.573	-0.117	-0.083	-0.311	-1.023	7.02E-45
KBTBD8	0.191	0.143	0.102	-0.153	-0.686	2E-12
MCEE	-0.189	-0.059	-0.038	-0.401	-0.812	0.00000195
MPV17L2	0.118	-0.125	-0.188	-0.069	-0.632	3.41E-09
DDX11L2	-0.522	0.417	0.214	-0.132	-0.583	5.53E-13
LINC00467	-0.103	-0.045	0.186	0.236	-0.599	0.0000828
GPAT3	-0.065	-0.133	-0.08	-0.118	-0.758	0.0000328
PPP1R15B	0.187	0.135	-0.133	-0.237	-0.6	3.84E-37
ALG10	-0.425	-0.09	-0.146	-0.169	-0.805	3.02E-12
TMEM128	-0.109	-0.043	-0.077	0.002	-0.646	2.03E-12
LRRCC1	0.031	-0.043	-0.161	-0.057	-0.655	0.00000299
DIXDC1	0.385	-0.359	-0.584	-0.272	-0.682	1.8E-22
CEP295	-0.465	-0.108	-0.162	-0.394	-0.592	1.22E-07
KRT8P12	-0.521	-0.247	-0.102	-0.313	-0.913	0.0000598
KLHL13	0.426	0.014	-0.341	-0.148	-0.573	0.00000005
DCAF15	-0.206	-0.142	-0.282	-0.543	-0.766	7.94E-19
MED30	-0.165	-0.134	-0.346	-0.401	-0.791	1.01E-09
LYRM7	-0.423	-0.36	-0.217	-0.331	-0.752	2.02E-17
ZFAND2A	0.003	0.03	-0.046	-0.439	-0.617	1.16E-10
FAM104B	-0.141	-0.186	-0.232	-0.525	-0.577	0.0000144
CEP95	-0.139	0.248	0.223	-0.438	-0.628	1.8E-22
TRIM41	-0.307	-0.033	0.084	-0.384	-0.6	1.11E-16
C12orf29	-0.256	-0.145	-0.192	-0.441	-0.606	0.000025
PLXNA4	0.059	-0.201	-0.551	-0.071	-1.044	4.28E-25
LIN52	-0.1	-0.239	-0.467	-0.45	-0.744	4.66E-18
FDXACB1	-0.169	-0.157	0.163	0.296	-0.835	1.18E-08
TMEM88	-0.557	-0.635	-0.585	-0.505	-0.627	0.000057
LMBRD2	-0.106	0.152	-0.095	-0.209	-0.928	5.09E-38
PRR29	-0.191	0.054	-0.041	-0.002	-0.812	0.0377
MARS2	-0.527	-0.266	-0.505	-0.439	-0.844	9.03E-09
HAUS8	-0.05	-0.329	-0.359	-0.266	-0.743	8.17E-13
L3HYPDH	-0.451	-0.052	0.101	-0.457	-0.66	1.36E-07
MTFR2	-0.273	-0.285	-0.43	-0.16	-0.759	2.19E-10

CDCA5	0.181	0.002	-0.125	-0.185	-0.731	3.89E-33
TMEM106A	-0.39	0.078	0.16	0.066	-0.598	2.71E-16
TOE1	-0.008	-0.199	-0.255	-0.41	-0.847	1.27E-17
SLC25A25	-0.21	-0.102	-0.267	-0.372	-0.726	1.57E-14
STK11IP	0.115	0.088	-0.003	-0.177	-0.753	6.68E-23
TMEM123	0.311	0.1	-0.176	0.055	-0.844	7.48E-36
TLCD1	#N/A	#N/A	#N/A	#N/A	#N/A	#N/A
CNTROB	-0.433	-0.147	-0.029	-0.306	-0.734	6.33E-28
SFXN2	-0.502	-0.089	0.05	-0.238	-0.639	4.74E-14
ZNF641	-0.002	0.055	-0.145	-0.474	-1.016	4.27E-26
ALKBH2	-0.412	-0.379	-0.327	-0.367	-0.661	0.00677
LRR1	-0.173	-0.185	-0.23	-0.244	-0.798	6.18E-10
FOPNL	-0.159	-0.251	-0.311	-0.426	-0.656	1.22E-10
SPATA33	-0.23	-0.104	-0.125	-0.535	-0.774	1.28E-17
C17orf49	0.175	0.07	0.041	0.083	-0.833	2.29E-07
SHE	0.419	-0.261	-0.332	-0.193	-0.765	1.5E-37
FCRLB	0.152	-0.313	-0.353	-0.105	-0.722	0.0229
ARHGEF19	0.099	0.039	-0.094	-0.41	-0.596	6.95E-10
NUP35	-0.182	-0.414	-0.583	-0.324	-0.868	1.4E-21
C2orf76	-0.318	-0.064	-0.197	-0.542	-0.676	0.000171
TTC32	0.199	0.163	-0.024	-0.316	-0.872	9.04E-08
ZFAND2B	-0.079	-0.09	-0.207	-0.382	-0.781	1E-21
CCDC58	-0.289	-0.13	-0.085	-0.514	-0.646	0.0000494
C4orf33	-0.276	-0.184	-0.27	-0.451	-0.815	0.00000172
POC5	-0.429	-0.394	-0.345	-0.298	-0.962	2.04E-12
ZNF280B	-0.483	-0.057	-0.377	-0.479	-0.907	6.17E-07
HECTD2	-0.054	0.118	0.003	-0.539	-0.747	7.55E-21
MIPOL1	0.131	-0.148	-0.133	-0.338	-0.658	1.97E-22
EME1	-0.529	-0.176	-0.137	-0.573	-0.822	1.76E-11
ZNF534	-0.158	-0.124	-0.229	-0.223	-0.68	0.0142
SPC24	-0.48	-0.35	-0.103	0.079	-0.668	2.95E-14
DPY19L3	-0.002	0.054	-0.037	-0.287	-0.711	3.56E-28
PQLC2L	-0.029	-0.272	-0.355	-0.206	-0.657	0.0366
NFXL1	-0.105	0.161	0.078	-0.302	-0.704	5.86E-29
MBLAC2	-0.448	-0.26	-0.339	-0.216	-0.647	1.67E-07
MARVELD2	0.373	0.367	-0.13	-0.207	-0.595	2.02E-09
RAET1L	0.256	-0.138	-0.322	-0.184	-0.881	0.00334
CCDC167	-0.133	-0.144	-0.027	-0.227	-0.616	4.95E-09

KCTD7	-0.237	-0.011	0.039	-0.402	-0.619	1.48E-13
FAM201A	-0.219	0.024	0.27	-0.139	-0.644	0.0017
SLC35G1	0.093	0.115	-0.411	-0.495	-0.895	1.21E-10
CLEC14A	0.124	-0.277	-0.589	-0.172	-0.754	2.03E-27
PGBD4	-0.385	0.034	-0.106	-0.492	-0.8	4.68E-10
ZNF114	0.144	-0.209	-0.365	-0.45	-0.996	0.00013
SASS6	-0.237	0.059	-0.267	-0.194	-0.614	1.08E-09
CCDC138	-0.348	-0.52	-0.435	-0.509	-0.894	0.00000195
THAP5	-0.097	0.082	-0.145	-0.368	-0.65	2.66E-19
GIMAP7	-0.414	-0.168	-0.436	-0.073	-0.777	9.28E-16
BHLHA15	0.302	-0.145	-0.05	0.037	-0.721	0.0318
ZNF596	-0.01	0.483	0.535	-0.15	-0.769	1.07E-14
ALG14	0.077	-0.102	-0.117	-0.201	-0.743	5.49E-12
TMEM201	-0.209	-0.285	-0.467	-0.577	-1.031	4.2E-29
KCTD6	0.034	-0.025	-0.112	-0.431	-0.643	0.0000674
C4orf46	0.072	-0.367	-0.539	-0.408	-0.811	8.49E-46
TMEM218	-0.326	-0.289	-0.154	-0.383	-0.674	0.000214
TPCN2	-0.293	0.279	0.003	-0.445	-0.711	1.71E-26
SKA1	-0.001	-0.218	-0.249	0.039	-0.785	1.89E-29
SKA3	-0.468	-0.413	-0.156	-0.045	-0.724	1.63E-21
C6orf136	-0.221	-0.006	-0.131	-0.22	-0.592	0.00000219
MTURN	0.466	0.346	0.131	-0.469	-0.864	2.14E-24
SEMA3D	0.041	0.042	-0.263	-0.199	-1.06	4.22E-31
GPATCH11	0.06	0.065	-0.183	-0.131	-0.583	4.85E-17
CERS6	0.242	0.071	-0.231	-0.165	-0.71	2.09E-17
BRWD3	-0.349	0.15	0.16	-0.352	-0.602	2.83E-20
ST6GALNAC	0.006	-0.116	-0.278	-0.322	-0.662	1.68E-21
MAGI3	-0.327	-0.149	-0.455	-0.453	-0.671	1.22E-10
NPHP4	-0.039	-0.211	-0.137	-0.419	-0.69	1.46E-15
FUOM	-0.079	-0.163	-0.213	-0.299	-0.755	7.61E-15
C14orf80	-0.018	-0.05	-0.066	-0.187	-0.915	2.48E-24
TSEN54	-0.142	-0.069	-0.299	-0.563	-1.035	2.21E-36
C3orf70	0.455	0.033	-0.209	-0.344	-0.638	4.92E-10
LINC00632	-0.094	-0.008	-0.206	-0.418	-0.858	0.0132
C1orf174	-0.293	-0.313	-0.273	-0.547	-0.65	5.46E-14
BEX5	-0.242	0.028	-0.034	-0.202	-0.672	0.000693
KIF24	0.02	0.06	-0.195	-0.092	-0.726	5.36E-10
GNG5P2	0.018	-0.238	-0.199	-0.341	-0.584	2.81E-09

CTU2	0.061	-0.182	-0.271	-0.086	-0.598	4.35E-12
ZNF530	0.135	-0.303	-0.193	-0.353	-0.781	8.02E-07
GEN1	-0.275	-0.186	-0.19	-0.535	-0.67	2.26E-15
RAET1G	0.19	-0.045	-0.258	-0.097	-0.726	0.0226
WASH3P	-0.47	0.153	0.244	-0.572	-0.734	4.83E-22
C5orf34	-0.528	-0.125	-0.346	-0.166	-0.776	8.56E-08
RNF148	0.273	0.192	-0.128	-0.313	-0.604	0.0362
CLEC12B	-0.318	-0.217	-0.165	-0.374	-0.761	0.00155
ZNF324B	-0.031	0.238	-0.385	-0.44	-0.737	1.5E-13
LYSMD1	-0.098	0.235	0.041	-0.389	-0.716	7.55E-10
LINC00493	-0.35	-0.281	-0.131	-0.436	-0.6	0.00242
SLC25A35	-0.525	-0.096	-0.111	-0.368	-0.687	1.88E-09
TOMM5	-0.367	-0.196	-0.145	-0.558	-0.734	0.00000466
CENPP	-0.236	0.154	0.16	0.244	-0.647	2.98E-20
WHAMMP2	-0.485	-0.093	-0.218	-0.451	-0.715	1.3E-08
TYW1B	-0.355	-0.147	-0.378	-0.391	-0.761	0.00627
CISD2	-0.064	-0.136	-0.161	-0.396	-0.815	1.06E-16
CHAC2	-0.483	-0.186	-0.255	-0.527	-0.89	4.33E-16
DUXAP9	0.153	0.078	-0.026	-0.107	-0.769	0.0234
LARP4P	-0.069	-0.331	-0.281	-0.275	-0.749	0.00104
RPL26P30	-0.061	-0.107	-0.033	-0.286	-0.592	0.00054
WASH7P	-0.35	0.078	0.041	-0.529	-0.704	1.23E-12
SNORA20	-0.082	-0.088	-0.257	-0.524	-0.885	0.0285
HMGN2P5	-0.05	-0.177	-0.06	-0.052	-0.799	4.01E-23
D2HGDH	-0.572	-0.086	-0.007	-0.487	-0.643	6.9E-13
HMGB2P1	-0.295	-0.726	-0.771	-0.297	-0.925	0.0000178
SNHG9	-0.25	-0.213	-0.051	-0.426	-0.975	1.73E-07
KTN1-AS1	-0.15	-0.601	-0.349	-0.39	-0.856	0.00095
CCDC152	0.149	-0.009	-0.047	0.336	-0.693	5.04E-07
C11orf91	0.233	0.719	0.73	-0.298	-0.85	8.36E-19
FAM27E3	-0.385	0.06	0.12	-0.065	-0.768	0.000193
ZNF587B	-0.19	0.065	-0.068	-0.202	-0.601	8.24E-07
TNPO1P1	-0.028	-0.261	-0.168	-0.092	-0.608	0.0000365
COX20P1	-0.326	-0.217	-0.113	-0.332	-0.661	0.00019
MICA	0.167	-0.057	-0.214	-0.356	-0.921	9.8E-91
ERVK3-1	0.079	-0.033	-0.301	-0.504	-0.626	1.15E-08
MIR4512	-0.409	-0.458	-0.067	-0.337	-0.756	0.0157
MIR7161	0.085	0.166	0.171	-0.061	-0.705	0.027

KPNA2	-0.265	-0.661	-0.721	0.018	0.187	3.78E-20
CBLN2	-0.007	0.669	0.518	0.543	-0.42	1.13E-31
DLGAP5	-0.492	-0.965	-1.025	-0.297	-0.153	4.46E-34
FAM50B	-0.451	-0.764	-0.116	0.513	0.356	3.24E-27
ZNF737	0.619	0.816	0.678	0.719	0.849	0.0256
KLHL3	-0.369	-0.601	-0.168	-0.172	0.122	0.0000231
RACGAP1	-0.562	-0.862	-0.751	-0.19	-0.543	2.3E-24
ANLN	-0.33	-0.606	-0.621	-0.194	-0.346	1.99E-17
PRICKLE1	0.122	-0.641	-0.672	-0.414	-0.521	1.96E-08
CD83	0.441	1.738	0.952	-0.086	0.831	4.14E-15
TNFAIP3	0.097	0.656	0.204	-0.293	0.533	2.32E-24
JAK3	-0.161	0.71	0.891	0.303	0.518	1.87E-11
ANKRA2	0.396	0.76	0.692	0.174	0.326	4.43E-12
NDC80	-0.505	-0.85	-0.606	0.003	-0.522	1.19E-32
KIF4B	-0.135	-0.686	-0.498	0.057	-0.239	0.000108
SERINC5	0.209	0.612	0.349	0.03	0.503	1.27E-12
TPX2	-0.31	-0.7	-0.582	-0.013	-0.1	2.83E-13
EIF4E3	0.442	0.649	0.606	0.645	0.475	0.00394
CA13	0.548	0.614	0.469	0.288	0.382	0.0028
PRICKLE4	-0.37	0.663	0.892	-0.084	0.177	2.75E-10
LACC1	0.105	0.789	0.507	-0.018	0.469	1.34E-11
HIVEP1	0.564	0.825	0.501	0.224	0.283	2.1E-23
EVI2B	0.048	-0.951	-0.827	-0.152	-0.247	2.09E-12
VIP	-0.049	0.786	1.26	0.29	0.451	6.69E-07
HIST1H4H	0.05	0.715	0.708	0.625	0.632	0.0177
KIF14	-0.347	-0.883	-0.643	-0.322	-0.382	6.59E-25
PCNA	0.474	0.599	0.35	-0.004	-0.305	9.01E-57
LYL1	0.336	-0.681	-0.293	0.155	-0.114	4.58E-33
MAMDC4	-0.266	0.773	1.037	-0.068	-0.283	3.18E-24
CDKL5	-0.287	0.713	0.432	0.401	0.41	5.8E-10
MIR570	0.117	0.597	0.404	-0.529	-0.48	0.00000172
ZBTB21	0.403	0.72	0.465	0.356	0.362	4.64E-19
IL7R	-0.084	0.726	0.134	-0.198	0.027	6.91E-39
PTTG2	-0.42	-0.756	-0.521	-0.499	-0.028	0.000113
PYY2	-0.223	0.75	0.735	-0.087	0.177	0.00116
BIRC5	-0.528	-0.629	-0.731	-0.558	-0.465	4.25E-29
SEMA3B	0.325	0.699	1.031	0.161	-0.174	2.16E-07
ANKRD26P1	-0.221	1.201	1.843	1.259	2.191	1.13E-28

CKAP2	-0.454	-0.631	-0.476	-0.006	-0.02	1.77E-21
USP49	0.254	0.656	0.532	-0.321	-0.154	4.45E-19
ZBTB12	-0.597	-0.649	-0.079	-0.156	-0.171	0.0133
ARL6IP1	-0.483	-0.86	-1.032	-0.546	0.143	8.12E-113
ATG16L2	-0.548	0.874	1.135	0.066	0.179	1.74E-28
CCNB2	-0.564	-1.02	-1.08	-0.587	-0.315	5.17E-53
CDC25C	-0.447	-0.739	-0.488	0.079	-0.071	2.07E-11
CDC25B	-0.455	-0.642	-0.403	-0.207	-0.433	3.25E-19
TMEM223	-0.246	-0.634	-0.364	-0.263	-0.508	0.000026
SLC45A3	-0.011	-0.621	-0.067	0.323	0.395	5.31E-26
PRR11	-0.274	-0.665	-0.62	-0.139	0.361	2.02E-36
TRIAP1	0.342	0.581	0.613	0.401	0.415	3.11E-10
FDFT1	0.44	-0.235	-0.618	-0.497	0.134	4.43E-40
NLRP1	0.066	0.58	0.713	0.264	0.313	3.7E-27
FAM71F2	0.472	0.271	0.717	-0.229	0.702	0.00107
ZNF337	-0.029	0.53	0.69	-0.109	0.107	7.56E-18
BLOC1S2	0.427	0.583	0.604	0.267	0.445	0.0000183
MVK	0.05	-0.357	-0.591	-0.396	-0.03	2.25E-07
GOLGA8O	-0.272	-0.001	0.659	-0.641	-0.027	0.000327
FAM26E	-0.002	-0.311	-0.65	-0.425	-0.327	6.08E-19
XAF1	-0.38	-0.134	0.588	0.037	0.485	8.39E-09
PRC1	-0.242	-0.493	-0.586	-0.188	-0.327	0.00000173
FZD2	-0.346	-0.397	-0.666	0.033	0.411	0.0000441
KCNJ2-AS1	0.122	0.532	0.713	0.552	0.281	0.0182
MIR6775	0.028	0.246	0.667	-0.062	-0.122	0.0334
ADAMTS13	-0.282	0.085	0.71	0.08	0.211	0.0000142
CBWD3	0.098	0.063	0.735	-0.229	-0.182	0.0217
FAM86HP	-0.526	0.381	0.791	0.009	0.175	0.00000133
DNAJC18	0.019	0.184	0.598	-0.053	0.279	0.0000051
SYNE3	0.489	0.092	0.642	0.31	-0.158	5E-24
CYP51A1	0.347	-0.243	-0.67	-0.547	-0.189	1.55E-43
ESX1	-0.069	-0.494	-0.676	-0.252	-0.187	0.000276
C5orf63	-0.044	-0.081	0.674	0.047	0.623	0.00278
CARF	-0.294	0.125	0.599	-0.007	0.025	0.00000147
IZUMO4	-0.285	0.265	0.647	-0.096	0.115	0.00516
SWT1	0.05	0.484	0.748	0.519	0.569	0.00000102
GCKR	-0.444	0.474	0.699	0.109	-0.244	1.39E-26
KLHL4	0.297	-0.14	-0.597	-0.565	0.098	1.26E-22

LYN	0.498	-0.175	-0.656	-0.328	-0.164	7.6E-32
LPAR1	-0.629	0.13	0.996	0.823	0.248	4.69E-13
OSBPL6	-0.284	-0.481	-0.597	-0.472	-0.171	5.25E-07
RN7SL834P	-0.231	0.268	0.686	-0.363	-0.136	0.00363
ZNF695	-0.49	-0.312	-0.781	-0.509	-0.593	0.0328
KIF20B	-0.452	-0.584	-0.621	-0.243	-0.209	4.81E-15
FAM95B1	-0.098	0.279	0.648	-0.456	-0.294	0.00122
RALGPS1	-0.079	0.373	0.764	0.237	0.522	0.000277
SQLE	0.314	-0.437	-0.735	-0.559	0.162	2.29E-59
PVT1	-0.344	0.433	0.658	0.083	0.413	9.71E-14

Name	NCBI Entrez	UniProt
Rho Guanine Nucleotide Exch	Rho-like GTF	Acts as guanine nucleotide exchange fa
Cell Division Cycle Associatec	This gene en	Regulator of chromosome structure duri
SAM Domain, SH3 Domain A	SAMSN1 is a	Negative regulator of B-cell activation. D
TBC1 Domain Family Member 2		Acts as GTPase-activating protein for R/
Kinesin Family Member 2C	This gene en	In complex with KIF18B, constitutes the
Holliday Junction Recognition Protein		Centromeric protein that plays a central
Ras Association Domain Fam	This gene en	Potential tumor suppressor. Acts as a K
Protoporphyrinogen Oxidase	This gene en	Catalyzes the 6-electron oxidation of prc
Exocyst Complex Component	The protein encoded by this gene is upregulated by va	
Double Homeobox 4 Like 50 (Pseudogene)		
THAP Domain Containing 3		Component of a THAP1/THAP3-HCFC1
Papilin, Proteoglycan Like Sulfated Glycoprotein		
Family With Sequence Similarity 221 Member A		
Inositol Polyphosphate-4-Pho:	INPP4B encc	Catalyzes the hydrolysis of the 4-positior
Sestrin 1	This gene en	Functions as an intracellular leucine sen
Vasohibin 1		Tyrosine carboxypeptidase that removes
ABL Proto-Oncogene 2, Non-	This gene en	Non-receptor tyrosine-protein kinase tha
Cleavage Factor Polyribonucl	This gene en	Polynucleotide kinase that can phosphoi
CUB And Zona Pellucida Like Domains 1		CUZD1 antiserum inhibits cell attachmer
Pim-3 Proto-Oncogene, Serin	The protein e	Proto-oncogene with serine/threonine ki
Synaptic Ras GTPase Activat	This gene en	Major constituent of the PSD essential fo
Rho GTPase Activating Protei	RICS is a nei	GTPase-activating protein (GAP) promo
Ferredoxin Reductase	This gene en	Serves as the first electron transfer prote
Solute Carrier Family 7 Memb	The protein e	Functions as permease involved in the ti
Lysosomal Trafficking Regula	This gene en	Adapter protein that regulates and/or fis:
Zinc Finger And SCAN Domain Containing		May be involved in transcriptional regula
PICALM Interacting Mitotic Regulator		During mitosis, may play a role in the co
Sestrin 2	This gene en	Functions as an intracellular leucine sen
Coiled-Coil Domain Containing 150		
MicroRNA 5047	microRNAs (miRNAs) are short (20-24 nt) non-coding	
Zinc Finger Protein 862		May be involved in transcriptional regula
Poly(A) Binding Protein Cytop	This gene belongs to the polyadenylate-binding protei	
Leukotriene B4 Receptor 2		Low-affinity receptor for leukotrienes inc
Choline Kinase Beta	Choline kinas	Has a key role in phospholipid metabolis
Heat Shock Protein Family A	This intronles	(Microbial infection) In case of rotavirus
MicroRNA 3064	microRNAs (miRNAs) are short (20-24 nt) non-coding	
POU Class 2 Homeobox 2	The protein e	Transcription factor that specifically bind

Myelin Protein Zero	This gene is :	Is an adhesion molecule necessary for r
G2 And S-Phase Expressed 1	The protein e	May be involved in p53-induced cell cycl
Zinc Finger Protein 79		May be involved in transcriptional regula
Denticleless E3 Ubiquitin Protein Ligase Ho		Substrate-specific adapter of a DCX (DD
Syntaphilin	Syntaxin-1, s	Inhibits SNARE complex formation by ab
Actin Alpha 2, Smooth Muscle	This gene en	Actins are highly conserved proteins tha
Neurobeachin Like 1		
Leucine Rich Repeat Containi	This gene is :	Synaptic adhesion protein. Regulates th
Proline And Serine Rich 2		
Suppressor APC Domain Containing 2		Plays a role in planar mitotic spindle orie
GAS6 Antisense RNA 1		
Zinc Finger Protein 485		May be involved in transcriptional regula
Coiled-Coil Domain Containing 144B (Pseudogene)		
Solute Carrier Family 9 Memb	The protein e	Involved in pH regulation to eliminate ac
Sp6 Transcription Factor	SP6 belongs	Promotes cell proliferation.
Coiled-Coil Domain Containing 68		Centriolar protein required for centriole s
Heat Shock Protein Family A	This intronles	(Microbial infection) In case of rotavirus
Cyclin Dependent Kinase Inhi	The protein e	May play a role in cell cycle regulation. D
Cytoplasmic Polyadenylation Element Bindi		Sequence-specific RNA-binding protein
Leucine Rich Repeat Containing 27		
Zinc Finger Protein 331	This gene en	May be involved in transcriptional regula
Rho GTPase Activating Prote	This gene encodes a member of the Rho GTPase act	
Myeloid Zinc Finger 1		Binds to target promoter DNA and functi
Fibroblast Growth Factor Rec	This gene en	Tyrosine-protein kinase that acts as cell-
Zinc Finger Protein 365	This gene en	Involved in the regulation of neurogenes
Cyclin Dependent Kinase Inhi	This gene en	May be involved in p53/TP53 mediated i
Cyclin Dependent Kinase Inhi	This gene en	Important regulator of cell cycle progres:
TNF Superfamily Member 9	The protein e	Cytokine that binds to TNFRSF9. Induce
Cyclin Dependent Kinase Inhi	This gene lie:	Interacts strongly with CDK4 and CDK6.
MicroRNA 4784	microRNAs (miRNAs) are short (20-24 nt) non-coding	
Chromodomain Y Like 2		
Long Intergenic Non-Protein Coding RNA 476		
Coiled-Coil Serine Rich Protein 2		Microtubule-binding protein which might
Rho GTPase Activating Prote	This gene en	May be involved in several stages of intr
DBF4 Zinc Finger		Regulatory subunit for CDC7 which activ
Caspase Recruitment Domair	The protein e	Inhibits NF-kappa-B activation. May part
RUN And SH3 Domain Conta	This gene encodes a RUN and SH3 domain containin	
Podocalyxin Like	This gene en	Involved in the regulation of both adhesi

IQ Motif Containing GTPase Activating Protein 3		
Von Willebrand Factor C And EGF Domain		May be a regulatory element in the beta-
Fibroblast Growth Factor 2	The protein e	Acts as a ligand for FGFR1, FGFR2, FG
TNF Alpha Induced Protein 8 Like 1		Acts as a negative regulator of mTOR a
Assembly Factor For Spindle	This gene is	Involved in mitotic spindle regulation anc
Homeobox B3	This gene is	Sequence-specific transcription factor w
Homeobox B2	This gene is	Sequence-specific transcription factor w
Homeobox B5	This gene is	Sequence-specific transcription factor w
Homeobox B4	This gene is	Sequence-specific transcription factor w
Tetraspanin 33		Plays an important role in normal erythro
Forkhead Box C2	This gene be	Transcriptional activator. Might be involv
MutS Homolog 5	This gene en	Involved in DNA mismatch repair and m
Minichromosome Maintenanc	The protein e	Acts as a replication initiation factor that
Serine Protease 53		In vitro can degrade the fibrinogen alpha
Prickle Planar Cell Polarity Pr	This gene encodes a homolog of Drosophila prickle. T	
Forkhead Box C1	This gene be	DNA-binding transcriptional factor that p
Small Nucleolar RNA, C/D Box 35B		
Forkhead Box F1	This gene be	Probable transcription activator for a nur
Centromere Protein A	Centromeres	Histone H3-like nucleosomal protein tha
Interleukin 21 Receptor	The protein e	This is a receptor for interleukin-21.
Centromere Protein E	Centrosome-	Microtubule plus-end-directed kinetocho
Centromere Protein F	This gene en	Required for kinetochore function and ch
G Protein Subunit Beta 4	Heterotrimeri	Guanine nucleotide-binding proteins (G
Homeobox D4	This gene be	Sequence-specific transcription factor w
Family With Sequence Similarity 72 Member D		
Pleckstrin Homology Domain Containing A		Binds specifically to phosphatidylinositol
Snail Family Transcriptional R	The Drosoph	Involved in induction of the epithelial to r
Ankyrin Repeat And Sterile Alpha Motif Dor		May be involved in vasopressin signaling
ZEB1 Antisense RNA 1	This locus produces long non-coding RNA that is tran	
Serine/Threonine Kinase 36	This gene en	Serine/threonine protein kinase which pl
Tripartite Motif Containing 45	This gene en	May act as a transcriptional repressor in
Nuclear Receptor Subfamily 4	This gene en	Orphan nuclear receptor. May act conco
Hyaluronan Mediated Motility	The protein e	Receptor for hyaluronic acid (HA) (By sir
GTPase, IMAP Family Membe	This gene en	The heterodimer formed by GIMAP2 anc
Tripartite Motif Containing 32	The protein e	Has an E3 ubiquitin ligase activity. Ubiqu
Protein Phosphatase, Mg2+/M	This gene en	Regulates the mitochondrial permeability
Heme Oxygenase 1	Heme oxyge	Heme oxygenase cleaves the heme ring
NSUN5 Pseudogene 2	This locus re	May have S-adenosyl-L-methionine-dep

3-Hydroxy-3-Methylglutaryl-CoA Synthase		Catalyzes the condensation of acetyl-Co
MicroRNA 7111	microRNAs (miRNAs) are short (20-24 nt) non-coding	
Hes Family BHLH Transcriptic	This gene en	Transcriptional repressor. Represses tra
Methylenetetrahydrofolate Re	The protein e	Catalyzes the conversion of 5,10-methyl
Fucose Kinase	The protein e	Takes part in the salvage pathway for re
Ectodysplasin A2 Receptor	The protein e	Receptor for EDA isoform A2, but not fo
Family With Sequence Similarity 83 Membe		May regulate the bone morphogenetic p
Trafficking Regulator And Sca	This gene en	Plays a role in intracellular trafficking an
SH2 Domain Containing Adaptor Protein B		Adapter protein which regulates several
GLIS Family Zinc Finger 2	This gene is	Can act either as a transcriptional repres
Kruppel Like Factor 13	KLF13 belon	Represses transcription by binding to the
Selectin E	The protein e	Cell-surface glycoprotein having a role in
FBXL19 Antisense RNA 1		
Polo Like Kinase 3	The protein e	Serine/threonine-protein kinase involved
SRY-Box Transcription Factor	This gene en	Transcriptional activator that binds to the
NUF2 Component Of NDC80	This gene en	Acts as a component of the essential kir
MicroRNA 1914	microRNAs (miRNAs) are short (20-24 nt) non-coding	
Vestigial Like Family Member 3		May act as a specific coactivator for the
WD Repeat And SOCS Box C	This gene en	Probable substrate-recognition compone
Ring Finger Protein 215		
Ring Finger Protein 207		Plays a role in cardiac repolarization pos
Msh Homeobox 2	This gene en	Acts as a transcriptional regulator in bon
Testin LIM Domain Protein	Cancer-asso	Scaffold protein that may play a role in c
Clathrin Binding Box Of Aftiphilin Containing 1		
Zinc Finger Protein 449	This gene en	May be involved in transcriptional regula
Rho Related BTB Domain Co	The protein encoded by this gene belongs to the Rho	
WT1 Interacting Protein		Adapter or scaffold protein which particip
XPC Complex Subunit, DNA I	The protein e	In absence of DNA repair, the XPC com
Advanced Glycosylation End-I	The advance	Mediates interactions of advanced glyco
Protein Kinase C Epsilon	Protein kinas	Calcium-independent, phospholipid- and
Jagged Canonical Notch Ligar	The jagged 1	Ligand for multiple Notch receptors and
Macrophage Stimulating 1	The protein encoded by this gene contains four kringl	
Sphingosine Kinase 1	The protein e	Catalyzes the phosphorylation of sphing
HIVEP Zinc Finger 2	This gene en	This protein specifically binds to the DN/
Hexokinase 2	Hexokinases	Catalyzes the phosphorylation of hexose
SplA/Ryanodine Receptor Domain And SO		Substrate recognition component of a S(
SKI Proto-Oncogene	This gene en	May play a role in terminal differentiatior
Essential Meiotic Structure-Sp	EME2 forms	Interacts with MUS81 to form a DNA stru

COX10 Antisense RNA 1		
SKI Like Proto-Oncogene	The protein e	May have regulatory role in cell division (
Crystallin Beta-Gamma Domain Containing		May function as suppressor of malignant
Aryl Hydrocarbon Receptor	The protein e	Ligand-activated transcriptional activator
RAB30, Member RAS Oncogene Family		The small GTPases Rab are key regulat
BTB Domain Containing 11		
CDC28 Protein Kinase Regula	CKS2 proteir	Binds to the catalytic subunit of the cycli
WASP Family Member 3	This gene en	Downstream effector molecules involvec
Aldehyde Dehydrogenase 1 F	This gene en	NAD-dependent aldehyde dehydrogenas
PH Domain And Leucine Rich Repeat Prote		Protein phosphatase involved in regulati
F-Box And Leucine Rich Repe	This gene en	Substrate-recognition component of the
Neuregulin 1	The protein e	Direct ligand for ERBB3 and ERBB4 tyrc
ETS Proto-Oncogene 1, Trans	This gene en	Transcription factor. Directly controls the
Aldehyde Dehydrogenase 3 F	Aldehyde deł	ALDHs play a major role in the detoxifica
Nuclear Receptor Subfamily 1	This gene en	Transcriptional repressor which coordina
Hematopoietically Expressed	This gene en	Recognizes the DNA sequence 5'-ATTA
C-Type Lectin Domain Family	This gene encodes a member of the C-type lectin/C-ty	
SplA/Ryanodine Receptor Do	This gene en	Substrate recognition component of a S(
CST Telomere Replication Co	This gene en	Component of the CST complex propose
Mastermind Like Transcriptional Coactivato		Acts as a transcriptional coactivator for N
DBF4 Zinc Finger B	This gene en	Regulatory subunit for CDC7 which activ
Adhesion Molecule With Ig Like Domain 2		Required for depolarization-dependent s
Proline And Serine Rich Coile	This gene en	Required for normal progression througł
Ecotropic Viral Integration Site 2A		May complex with itself or/and other pro
Protein Tyrosine Phosphatase	This gene en	Acts as negative regulator of T-cell recep
F-Box Protein 43	Members of t	Required to establish and maintain the a
Ribonucleotide Reductase Re	This gene en	Plays a pivotal role in cell survival by rep
Rho Family GTPase 1	This gene en	Lacks intrinsic GTPase activity. Has a lo
1-Aminocyclopropane-1-Carboxylate Synth		Does not catalyze the synthesis of 1-am
Polo Like Kinase 2	The protein e	Tumor suppressor serine/threonine-prote
Poly(A) Specific Ribonuclease	This gene en	Catalytic subunit of the poly(A)-nuclease
Apelin	This gene en	(Microbial infection) Endogenous ligand
Hdc Homolog, Cell Cycle Reg	This gene en	May play an important role in some hum
Tripartite Motif Containing 46		Microtubule-associated protein that is inv
BMS1 Pseudogene 4		
ST3 Beta-Galactoside Alpha-2	The protein e	Responsible for the synthesis of the seq
Centromere Protein S	This gene wa	DNA-binding component of the Fanconi
Enhancer Of Zeste 1 Polycom	EZH1 is a co	Polycomb group (PcG) protein. Catalytic

Alkaline Phosphatase, Biomin	This gene en	This isozyme plays a key role in skeletal
MIR137 Host Gene		
ALS2 C-Terminal Like		Acts as a guanine nucleotide exchange
Zinc Finger And BTB Domain Containing 4		Telomere-binding protein that acts as a
Cell Division Cycle Associated 3		F-box-like protein which is required for e
MAX Dimerization Protein 3	This gene en	Transcriptional repressor. Binds with MA
Placental Growth Factor	This gene en	Growth factor active in angiogenesis and
SH3 Domain Binding Protein	This gene en	May function in transferrin receptor inter
Family With Sequence Similarity 72 Member B		
Claudin 7	This gene en	Plays a major role in tight junction-speci
DNA Polymerase Gamma 2,	This gene en	Mitochondrial polymerase processivity s
Solute Carrier Family 25 Mem	This gene en	Transports arginine, lysine, homoarginin
Phosphatidylinositol Specific Phospholipase C X Domain Containing 2		
Unc-5 Netrin Receptor B	This gene en	Receptor for netrin required for axon gui
Leucine Rich Repeat Containing 45		Component of the proteinaceous fiber-li
Hypoxia Inducible Factor 3 Su	The protein e	[Isoform 2]: Attenuates the ability of tran
Trophinin Associated Protein		Could be involved with bystin and trophi
Cerebellin 3 Precursor	Members of t	May be involved in synaptic functions in
Acid Phosphatase 6, Lysopho	This gene en	Hydrolyzes lysophosphatidic acid (LPA)
Zinc Finger Protein 799		May be involved in transcriptional regula
Small Nucleolar RNA Host Ge	This gene is a member of the non-protein-coding mult	
Glucosamine (UDP-N-Acetyl)-	The protein e	Regulates and initiates biosynthesis of N
Mitogen-Activated Protein Kin	This gene en	Component of a protein kinase signal tra
PIF1 5'-To-3' DNA Helicase	This gene en	DNA-dependent ATPase and 5'-3' DNA
Terminal Nucleotidyltransferase 5A		Probable nucleotidyltransferase that ma
Small Nucleolar RNA, H/ACA Box 3A		
Macrophage Stimulating 1 Pseudogene 2		
Transcription Elongation Factor A N-Terminal And Central Domain Containing 2		
Solute Carrier Family 16 Member 13		Proton-linked monocarboxylate transpor
Kelch Like Family Member 17	The protein e	Substrate-recognition component of som
Enolase Superfamily Member	This gene ca	Plays a role in the catabolism of L-fucos
STAG3L5P-PVRIG2P-PILRB	This locus represents naturally occurring readthrough	
Pleckstrin 2	The protein e	May help orchestrate cytoskeletal arrang
Centromere Protein I	This gene en	Component of the CENPA-CAD (nucleo
Ras Association Domain Fam	This gene encodes a member of the Ras-assocation	
Enkurin, TRPC Channel Intera	This gene en	Adapter that functions to localize a calci
Nuclear Receptor Subfamily 5	The protein e	Nuclear receptor that acts as a key meta
Stromal Antigen 3-Like 5 Pseudogene		

LYR Motif Containing 9		
C-X-C Motif Chemokine Ligan	The protein e	IL-8 is a chemotactic factor that attracts
WD Repeat Domain 27	This gene encodes a protein with multiple WD repeat	
Zinc Finger Protein 692		May be involved in transcriptional regula
Pleckstrin Homology Like Domain Family A		p53/TP53-regulated repressor of Akt/AK
MDM2 Proto-Oncogene	This gene en	E3 ubiquitin-protein ligase that mediates
NIFK Antisense RNA 1		
ADAM Metallopeptidase With	This gene encodes a member of the ADAMTS (a disi	
Myocyte Enhancer Factor 2A	The protein e	Transcriptional activator which binds spe
C-X-C Motif Chemokine Ligan	This antimicr	Chemoattractant active on T-lymphocyte
Transient Receptor Potential	The product	Calcium channel mediating constitutive
Coiled-Coil Domain Containing 9B		
Kruppel Like Factor 3		Binds to the CACCC box of erythroid cel
Terminal Nucleotidyltransferase 4B		Terminal nucleotidyltransferase that cat
SLX1A-SULT1A3 Readthrou	This locus represents naturally occurring read-through	
TNF Superfamily Member 18	The protein e	Cytokine that binds to TNFRSF18/AITR/
Myocyte Enhancer Factor 2C	This locus en	Transcription activator which binds spec
Immediate Early Response 5	This gene en	Plays a role as a transcription factor (Pu
Activating Transcription Factor 7 Interacting		Recruiter that couples transcriptional fac
Transmembrane Protein 198B (Pseudogene)		
TNF Alpha Induced Protein 8 Like 3		Acts as a lipid transfer protein. Preferen
F2R Like Thrombin Or Trypsi	This gene en	Receptor for activated thrombin or trypsi
Baculoviral IAP Repeat Conta	This gene en	Multi-functional protein which regulates
Family With Sequence Similarity 72 Membe		May play a role in the regulation of cellul
V-Set And Immunoglobulin Domain Containing 2		
CDC42 Effector Protein 4	The product	Probably involved in the organization of
Ankyrin Repeat Domain 36		
Kelch Repeat And BTB Domain Containing 3		
MicroRNA 503	microRNAs (miRNAs) are short (20-24 nt) non-coding	
Ubiquitin C	This gene re	[Ubiquitin]: Exists either covalently attac
Family With Sequence Similarity 131 Member A		
Minichromosome Maintenanc	The protein e	Acts as component of the MCM2-7 com
GTP Binding Protein 2	GTP-binding proteins, or G proteins, constitute a supe	
Phosphodiesterase 4D	This gene en	Hydrolyzes the second messenger cAM
ATP Binding Cassette Subfan	The protein e	Acts as a multispecific organic anion pu
RAB20, Member RAS Oncogene Family		Plays a role in apical endocytosis/recycli
Shroom Family Member 1	SHROOM fa	May be involved in the assembly of micr
TYRO3P Protein Tyrosine Kinase Pseudogene		

Claudin 14	Tight junctior	Plays a major role in tight junction-speci
Fas Cell Surface Death Rece	The protein e	Receptor for TNFSF6/FASLG. The adap
Trypsin Domain Containing 1	This gene en	Peroxisomal protease that mediates bot
DEP Domain Containing 1		May be involved in transcriptional regula
G2/M-Phase Specific E3 Ubiquitin Protein L		E3 ubiquitin-protein ligase which accept
RAB, Member Of RAS Oncog	This gene is	Plays an essential role in male fertility, s
BRICHOS Domain Containing 5		
Gap Junction Protein Gamma	This gene is	One gap junction consists of a cluster of
Kruppel Like Factor 6	This gene en	Transcriptional activator (By similarity). E
Sodium Channel Epithelial 1 Subunit Delta		Sodium permeable non-voltage-sensitiv
TNF Receptor Superfamily M	The protein e	(Microbial infection) Acts as a receptor f
Ankyrin Repeat Domain 23	This gene is	May be involved in the energy metabolis
Forkhead Box O3B		Transcription factor.
WD Repeat Domain 76		Specifically binds 5-hydroxymethylcytosi
Polo Like Kinase 1	The Ser/Thr	Serine/threonine-protein kinase that per
Aurora Kinase B	This gene en	Serine/threonine-protein kinase compon
Forkhead Box O1	This gene be	Transcription factor that is the main targ
Tuftelin 1	Tuftelin is an	Involved in the mineralization and struct
Inhibitor Of DNA Binding 2	The protein e	Transcriptional regulator (lacking a basic
Atonal BHLH Transcription Factor 8		Transcription factor that binds a palindr
Ankyrin Repeat Domain 33B		
Decapping MRNA 1B	This gene en	May play a role in the degradation of mF
Citron Rho-Interacting Serine/	This gene en	Plays a role in cytokinesis. Required for
Inhibitor Of DNA Binding 1, H	The protein e	Transcriptional regulator (lacking a basic
Glutathione S-Transferase Th	The protein e	Conjugation of reduced glutathione to a
RCAN Family Member 3		Inhibits calcineurin-dependent transcript
Pro-Melanin Concentrating H	This gene en	MCH may act as a neurotransmitter or n
Rho Guanine Nucleotide Exchange Factor		Promotes cell proliferation.
Phorbol-12-Myristate-13-Acetate-Induced F		Promotes activation of caspases and ap
Spectrin Beta, Non-Erythrocytic 5		
MHC Class I Polypeptide-Related Sequence E (Pseudogene)		
Hes Family BHLH Transcription Factor 2		Transcriptional repressor of genes that r
TTK Protein Kinase	This gene en	Phosphorylates proteins on serine, threc
Aminoadipate-Semialdehyde	This gene en	Bifunctional enzyme that catalyzes the fi
Golgin A6 Family Like 9		
Ras Homolog Family Member B		Mediates apoptosis in neoplastically trar
Interferon Induced Protein With Tetratricop		IFN-induced antiviral protein which acts
Zinc Finger Protein 696		May be involved in transcriptional regula

Sprouty Related EVH1 Domai	This gene en	Tyrosine kinase substrate that inhibits gı
HLA Complex Group 25		
Zinc Finger Protein 546		May be involved in transcriptional regula
Ras Association Domain Fam	The protein e	May play a role in regulating vesicuar tra
RCC1 Domain Containing 1		Plays a role in transcriptional repression
Asialoglycoprotein Receptor 1	This gene en	Mediates the endocytosis of plasma glyc
Zinc Finger Protein 514		May be involved in transcriptional regula
Kinesin Family Member 20A		Mitotic kinesin required for chromosome
Plasminogen Activator, Urokir	This gene en	Specifically cleaves the zymogen plasmi
KIT Ligand	This gene en	Ligand for the receptor-type protein-tyro:
Leucine Rich Repeat Containing 75A		
Tetratricopeptide Repeat Domain 14		
Ligand Of Numb-Protein X 2		
Coiled-Coil Domain Containing 142		
ARVCF Delta Catenin Family	Armadillo Re	Involved in protein-protein interactions a
Carnitine Palmitoyltransfераse	This gene en	May play a role in lipid metabolic proces
Exonuclease 1	This gene en	5'->3' double-stranded DNA exonucleas
Protein Phosphatase 1 Regul	Myosin light	Inhibits protein phosphatase 1 activity to
Hect Domain And RLD 2 Pseudogene 9		
Zinc Finger Protein 839		
Hect Domain And RLD 2 Pseudogene 3		
MN1 Proto-Oncogene, Transc	Meningioma	Transcriptional activator which specifical
Phosphatidylinositol-4-Phospl	The protein e	Phosphorylates PtdIns and PtdIns4P wit
TOPBP1 Interacting Checkpo	Treslin is inv	Regulator of DNA replication and S/M ar
Zinc Finger Matrin-Type 3	This gene en	Acts as a bona fide target gene of p53/T
Activating Transcription Facto	This gene en	This protein binds the cAMP response e
DEP Domain Containing 1B		
Sterile Alpha Motif Domain Containing 1		May play a role in atherogenesis by imm
Cyclin E2	The protein e	Essential for the control of the cell cycle
Delta Like Canonical Notch Li	This gene is	Involved in the Notch signaling pathway
5-Hydroxytryptamine Receptor 1D		G-protein coupled receptor for 5-hydrox
Mitochondrial RRNA Methyltransferase 1		S-adenosyl-L-methionine-dependent 2'-
MicroRNA 6835	microRNAs (miRNAs) are short (20-24 nt) non-coding	
Tumor Protein P53 Inducible Nuclear Prote		Antiproliferative and proapoptotic proteir
Rap Guanine Nucleotide Exchange Factor		Guanine nucleotide exchange factor (GE
DEPP1 Autophagy Regulator	The expressi	Acts as a critical modulator of FOXO3-in
LBH Domain Containing 1	This gene shares three exons in common with anothe	
Ubiquitin Conjugating Enzyme	The modifica	Accepts ubiquitin from the E1 complex a

Tribbles Pseudokinase 1		Adapter protein involved in protein degra
Shugoshin 1	The protein e	Plays a central role in chromosome cohe
CACN Subunit Beta Associated Regulatory		Negatively regulates voltage-gated calciu
RRAD, Ras Related Glycolysis Inhibitor An		May play an important role in cardiac an
Myotubularin Related Protein 4		Dephosphorylates proteins phosphorylat
HYLS1 Centriolar And Cilioge	This gene en	Plays a role in ciliogenesis.
Marker Of Proliferation Ki-67	This gene en	Required to maintain individual mitotic cl
Colony Stimulating Factor 3	This gene en	Granulocyte/macrophage colony-stimula
Calcium Dependent Secretior	This gene en	Calcium-binding protein involved in exoc
T-Box Transcription Factor 18	This genes c	Acts as transcriptional repressor involve
Serpin Family B Member 8	The protein e	Has an important role in epithelial desm
Kinetochore Localized Astrin (SPAG5) Bind		Essential component of the mitotic spinc
Ankyrin Repeat Domain 35		
Family With Sequence Similarity 193 Member B		
Tyrosine Kinase Non Recepto	This gene en	Non-receptor tyrosine-protein and serine
Dehydrogenase/Reductase 13		Putative oxidoreductase.
RE1 Silencing Transcription F	This gene wa	[Isoform 3]: Binds to the 3' region of the
Damage Specific DNA Binding	This gene en	[Isoform D1]: Inhibits UV-damaged DNA
Poly(ADP-Ribose) Glycohydrolase Pseudogene 1		
TGFB Induced Factor Homeo	The protein e	Binds to a retinoid X receptor (RXR) resp
Migration And Invasion Inhibit	This gene en	Inhibits glioma cells invasion and down-r
Growth Arrest And DNA Dama	This gene is	In T-cells, functions as a regulator of p38
CASTOR Family Member 3		
Glypican 2		Cell surface proteoglycan that bears hep
Transforming Growth Factor E	The protein e	Transmembrane serine/threonine kinase
SS18 Like 2	Synovial sarcomas occur most frequently in the extren	
DEAD-Box Helicase 18 Pseudogene 5		
Protein Phosphatase 1 Regulatory Subunit		Inhibits PPP1CA phosphatase activity.
GLI Family Zinc Finger 4		
Lysine Demethylase 7A		Histone demethylase required for brain c
Small ArfGAP 1	The protein e	GTPase activating protein that acts on A
BCL6B Transcription Repressor		Acts as a sequence-specific transcriptio
Noggin	The secreted	Inhibitor of bone morphogenetic proteins
Lysine Demethylase 3A	This gene en	Histone demethylase that specifically de
KIT Proto-Oncogene, Recepto	This gene en	Tyrosine-protein kinase that acts as cell-
Small Nucleolar RNA Host Ge	This gene is small nucleolar RNA host gene 10 and re	
Hes Family BHLH Transcription Factor 4		Transcriptional repressor. Binds DNA or
DNA Damage Inducible Trans	This gene en	[Isoform AltDDIT3]: Product of the upstre

NEDD4 Like E3 Ubiquitin Prot	This gene en	E3 ubiquitin-protein ligase which accept
3-Oxoacyl-ACP Synthase, Mit	This gene en	May play a role in the biosynthesis of lip
Coiled-Coil Domain Containing 121		
Diacylglycerol Kinase Theta	The protein e	Phosphorylates diacylglycerol (DAG) to
Malignant Fibrous Histiocytom	Identified in a	Probable GTP-binding protein (PubMed:
Pre-MRNA Processing Factor	This gene en	May be involved in pre-mRNA splicing.
Nei Like DNA Glycosylase 1	This gene is	Involved in base excision repair of DNA
Transmembrane Protein 42		
Heat Shock Factor Binding Protein 1 Like 1		
Chromosome 3 Open Reading Frame 18		
Coiled-Coil Domain Containing 71 Like		
Synemin	The protein e	Type-VI intermediate filament (IF) which
TIMP Metallopeptidase Inhibit	This gene be	Complexes with metalloproteinases (suc
MiR-17-92a-1 Cluster Host Ge	This gene is the host gene for the MIR17-92 cluster, a	
C-Type Lectin Domain Family 4 Member G Pseudogene 1		
Ribosomal Protein S6 Kinase A5		Serine/threonine-protein kinase that is re
Family With Sequence Simila	This gene encodes a protein with a trypsin-like cystei	
Hect Domain And RLD 2 Pseudogene 2		
Family With Sequence Similarity 27 Member B		
MicroRNA 10b	microRNAs (miRNAs) are short (20-24 nt) non-coding	
Ubiquitin Like With PHD And	This gene en	Multidomain protein that acts as a key e
Gap Junction Protein Alpha 5	This gene is	One gap junction consists of a cluster of
Regulator Of G Protein Signal	Regulator of	Regulates G protein-coupled receptor si
Zinc Finger And BTB Domain	This gene en	Transcriptional repressor that plays a rol
Zinc Finger Protein 780B		May be involved in transcriptional regula
MicroRNA 3189	microRNAs (miRNAs) are short (20-24 nt) non-coding	
Tolloid Like 1	This gene en	Protease which processes procollagen C
Chromosome 3 Open Reading Frame 52		
EGF Like Domain Multiple 8		
CD274 Molecule	This gene en	Plays a critical role in induction and mai
Prostate Transmembrane Pro	This gene en	Functions as a negative regulator of TG
MicroRNA 3153	microRNAs (miRNAs) are short (20-24 nt) non-coding	
Regulatory Factor X2	This gene is	Transcription factor that acts as a key re
DLC1 Rho GTPase Activating	This gene en	Functions as a GTPase-activating protei
Thrombospondin Type 1 Dom	The protein encoded by this gene contains a type 1 th	
Retinoic Acid Receptor Beta	This gene en	Receptor for retinoic acid. Retinoic acid
N-Myc Downstream Regulate	This gene is	Stress-responsive protein involved in ho
STEAP2 Metalloreductase	This gene is	Metalloreductase that has the ability to r

Tensin 2	The protein e	Tyrosine-protein phosphatase which reg
BMP And Activin Membrane E	This gene en	Negatively regulates TGF-beta signaling
PH Domain Containing Endoc	This gene en	Plays a role in endocytic trafficking. Req
Ribosomal Protein S6 Kinase Related		
Ankyrin Repeat Domain 34A		
Cytochrome P450 Family 26 S	This gene en	A cytochrome P450 monooxygenase inv
Ankyrin Repeat Domain 36B		
Potassium Inwardly Rectifying	Potassium ch	Probably participates in establishing acti
CEA Cell Adhesion Molecule	This gene en	[Isoform 1]: Cell adhesion protein that m
Gasdermin B	This gene en	The N-terminal moiety promotes pyropto
RAD52 Homolog, DNA Repai	The protein e	Involved in double-stranded break repair
Kinesin Family Member 18B		In complex with KIF2C, constitutes the m
G Protein Subunit Beta 3	Heterotrimeri	Guanine nucleotide-binding proteins (G
RAD51 Recombinase	The protein e	Plays an important role in homologous s
NSUN5 Pseudogene 1	This locus represents a transcribed pseudogene of a	
Glutamine Rich 2		Has an essential role in the formation of
G Protein Subunit Alpha I1	Guanine nuc	Guanine nucleotide-binding proteins (G
Inka Box Actin Regulator 2		Inhibitor of the serine/threonine-protein k
MYCBP Associated Protein		May play a role in spermatogenesis. Ma
MicroRNA 5581	microRNAs (miRNAs) are short (20-24 nt) non-coding	
Zinc Finger Protein 436		May be a transcriptional repressor.
Proliferation-Associated 2G4 Pseudogene 1		
Kruppel Like Factor 4	This gene en	Transcription factor; can act both as acti
Transcriptional And Immune F	This gene en	Seems to be involved in the regulation o
Centrosomal Protein 44		
BTG3 Associated Nuclear Prc	This gene en	Controls V(D)J recombination during T-c
Neuronal Tyrosine Phosphorylated Phosph		Activates PI3K and concomitantly recruit
Janus Kinase 1	This gene en	Tyrosine kinase of the non-receptor type
Bardet-Biedl Syndrome 10	This gene is	Probable molecular chaperone that assis
Hydroxypyruvate Isomerase (I	This gene en	Catalyzes the reversible isomerization b
Sirtuin 1	This gene en	(Microbial infection) In case of HIV-1 infe
DNA Topoisomerase II Alpha	This gene en	Control of topological states of DNA by t
Jun Proto-Oncogene, AP-1 Tr	This gene is	Transcription factor that recognizes and
BCL3 Transcription Coactivat	This gene is	Contributes to the regulation of transcrip
JunB Proto-Oncogene, AP-1 Transcription		Transcription factor involved in regulatin
RAS P21 Protein Activator 2	The protein e	Inhibitory regulator of the Ras-cyclic AM
Leucine Rich Repeat Containi	This gene en	Key regulator of transforming growth fac
Inositol-Trisphosphate 3-Kina	The protein encoded by this protein regulates inositol	

Family With Sequence Similarity 24 Member B		
Acyl-CoA Dehydrogenase Far	This gene en	Acyl-CoA dehydrogenase only active wit
Nuclear Pore Complex Interacting Protein Family Member B4		
Integrin Subunit Beta 8	This gene is	Integrin alpha-V:beta-8 (ITGAV:ITGB8) i
Deoxyribonuclease 1 Like 2		Divalent cation-dependent acid DNA enc
MicroRNA 221	microRNAs (miRNAs) are short (20-24 nt) non-coding	
Deoxyribonuclease 1	This gene en	Serum endocuclease secreted into body
RING1 And YY1 Binding Protein		Component of a Polycomb group (PcG)
Pyroglutamyl-Peptidase I	The gene en	Removes 5-oxoproline from various pen
Transcription Factor 7 Like 2	This gene en	Participates in the Wnt signaling pathwa
BEN Domain Containing 3		Transcriptional repressor which associat
Interleukin 1 Receptor Associated Kinase 1		Component of the IRAK1-dependent TN
BUB1 Mitotic Checkpoint Seri	This gene en	Essential component of the mitotic chec
BUB1 Mitotic Checkpoint Seri	This gene en	Serine/threonine-protein kinase that per
Galactose-1-Phosphate Uridy	Galactose-1-	Plays an important role in galactose met
BTG Anti-Proliferation Factor	This gene is	Anti-proliferative protein.
Origin Recognition Complex S	The origin re	Component of the origin recognition com
UFM1 Specific Peptidase 1 (I	This gene encodes a protein that is similar to other Uf	
Paired Immunoglobin Like Ty	The paired in	Paired receptors consist of highly relate
Shugoshin 2		Cooperates with PPP2CA to protect cen
Kruppel Like Factor 5	This gene en	Transcription factor that binds to GC bo
Centromere Protein L	CENPL is a s	Component of the CENPA-CAD (nucleo
DNA Methyltransferase 3 Beta	CpG methyla	Required for genome-wide de novo metl
Kinesin Family Member 18A	KIF18A is a r	Microtubule-depolymerizing kinesin whic
HOXA Transcript Antisense R	This non-coding locus is located in the HOX gene clus	
Bone Morphogenetic Protein 4	This gene en	Induces cartilage and bone formation. A
Serpin Family B Member 2		Inhibits urokinase-type plasminogen acti
Vasorin		May act as an inhibitor of TGF-beta sign
Ras Association Domain Family Member 7		Negatively regulates stress-induced JNK
Acyl-CoA Binding Domain Co	This gene en	Binds medium- and long-chain acyl-CoA
Bone Morphogenetic Protein 2	This gene en	Induces cartilage and bone formation (P
BCL6 Corepressor	The protein e	Transcriptional corepressor. May specifi
Interferon Regulatory Factor 6	This gene en	Probable DNA-binding transcriptional ac
Solute Carrier Family 23 Member 3		
Anoctamin 8		Does not exhibit calcium-activated chlori
Lipase G, Endothelial Type	The protein e	Exerts both phospholipase and triglyceri
Hes Related Family BHLH Tra	This gene en	Transcriptional repressor which binds pr
FERM Domain Containing 3	The protein e	Putative tumor suppressor gene that ma

Cytidine Monophospho-N-Ace	Sialic acids a	Sialic acids are components of carbohyc
Acyl-CoA Dehydrogenase Far	This gene en	Acyl-CoA dehydrogenase, that exhibits r
Dual Specificity Tyrosine Pho:	This gene pr	Dual-specificity protein kinase that prom
AT-Rich Interaction Domain 5	This gene en	Transcription coactivator that binds to th
Small Nucleolar RNA, C/D Box 93		
Small Nucleolar RNA, H/ACA Box 71C		
Interferon Regulatory Factor 1	The protein e	Transcriptional regulator which displays
Purinergic Receptor P2Y11	The product	Receptor for ATP and ADP coupled to G
Myeloid Associated Differentiation Marker		
FOS Like 1, AP-1 Transcriptic	The Fos gene family consists of 4 members: FOS, FC	
Kruppel Like Factor 15		Transcriptional regulator that binds to th
RecQ Like Helicase 5	The protein e	Isoform beta is a DNA helicase that play
TRNA Splicing Endonuclease	This gene en	Constitutes one of the two catalytic subu
Cytosolic Arginine Sensor For MTORC1 Su		Functions as an intracellular arginine se
Long Intergenic Non-Protein Coding RNA 1006		
ER Membrane Protein Complex Subunit 9		
ATP Binding Cassette Subfan	The protein e	Catalyzes the translocation of specific pl
T-Box Transcription Factor 20	This gene en	Acts as a transcriptional activator and re
Small Nucleolar RNA, H/ACA	This gene encodes a non-coding RNA that functions i	
Phospholipase D Family Member 6		Endonuclease that plays a critical role in
Cdk5 And Abl Enzyme Substr	This gene en	Cyclin-dependent kinase binding protein
H2A Clustered Histone 6	Histones are	Core component of nucleosome. Nuclec
Small Nucleolar RNA, H/ACA Box 71A		
Zinc Finger And BTB Domain Containing 2		May be involved in transcriptional regula
Small Nucleolar RNA, H/ACA Box 26		
Insulin Induced Gene 1	This gene en	Mediates feedback control of cholestero
TLE Family Member 6, Subco	This gene en	As a member of the subcortical materna
Frizzled Class Receptor 1	Members of	Receptor for Wnt proteins (PubMed:105
Hes Related Family BHLH Tra	This gene en	Downstream effector of Notch signaling
Gamma-Glutamyltransferase	This gene is	Cleaves the gamma-glutamyl peptide bo
Frizzled Class Receptor 8	This intronles	Receptor for Wnt proteins. Component c
Shroom Family Member 3	This gene en	Controls cell shape changes in the neur
Chromosome 19 Open Reading Frame 44		
Lebercilin LCA5	This gene en	Involved in intraflagellar protein (IFT) tra
Kelch Like Family Member 24	The protein e	Necessary to maintain the balance betw
Solute Carrier Family 40 Mem	The protein e	May be involved in iron export from duo
Zinc Finger MYM-Type Containing 1		
MicroRNA 126	microRNAs (miRNAs) are short (20-24 nt) non-coding	

RNA Component Of Signal Re	The signal recognition particle (SRP) is a cytoplasmic	
PCBP2 Overlapping Transcript 1		
Folliculin Interacting Protein 2	This gene en	Binding partner of the GTPase-activating
LON Peptidase N-Terminal Do	The protein encoded by this gene contains a RING fin	
Inner Centromere Protein	In mammalia	Component of the chromosomal passen
TEA Domain Transcription Fa	This gene pro	Transcription factor which plays a key ro
Small Nucleolar RNA, H/ACA	Small nucleolar RNAs (snoRNAs) are small noncoding	
Ring Finger Protein 19B	This gene en	E3 ubiquitin-protein ligase which accepts
GTP Cyclohydrolase 1	This gene en	Positively regulates nitric oxide synthesis
Small Nucleolar RNA, C/D Box 55		
NUAK Family Kinase 2		Stress-activated kinase involved in tolera
H3 Clustered Histone 6	Histones are	Core component of nucleosome. Nucleo
Chromosome 7 Open Reading Frame 61		
Gypsy Retrotransposon Integrase 1		
Transcription Factor AP-2 Alp	The protein e	Sequence-specific DNA-binding protein
Tripartite Motif Containing 59		May serve as a multifunctional regulator
BCAS3 Microtubule Associated Cell Migrati		Plays a role in angiogenesis. Participate
Zinc Finger Protein 367		Transcriptional activator. Isoform 1 may
Follistatin Like 3	Follistatin-like	Isoform 1 or the secreted form is a bindi
GATA Binding Protein 2	This gene en	Transcriptional activator which regulates
Protocadherin 1	This gene be	May be involved in cell-cell interaction pr
TBC1 Domain Family Member 3F		Acts as a GTPase activating protein for
Growth Arrest Specific 2 Like 3		Cytoskeletal linker protein. May promote
Sorting Nexin 22	The protein e	May be involved in several stages of intr
GATA Binding Protein 6	This gene is	Transcriptional activator (PubMed:19666
Glucosylceramidase Beta Pseudogene 1		
Oxidative Stress Induced Growth Inhibitor F		May be involved in meiosis or the matura
RANBP2 Like And GRIP Dom	RAN is a small GTP-binding protein of the RAS super	
BORA Aurora Kinase A Activa	BORA is an a	Required for the activation of AURKA at
Carbohydrate Sulfotransferas	This gene en	Sulfotransferase that utilizes 3'-phospho
Interleukin 11	The protein e	Cytokine that stimulates the proliferation
Kinesin Family Member 15		Plus-end directed kinesin-like motor enz
Small Cajal Body-Specific RNA 21		
Gastrulation Brain Homeobox 2		May act as a transcription factor for cell
ADAM Metallopeptidase With	This gene en	Cleaves the large aggregating proteogly
Transforming Growth Factor A	This gene en	TGF alpha is a mitogenic polypeptide tha
Syntaxin 3	The gene is a	Potentially involved in docking of synapt
SMAD Specific E3 Ubiquitin P	This gene en	E3 ubiquitin-protein ligase that acts as a

L3MBTL Histone Methyl-Lysir	This gene re|	Polycomb group (PcG) protein that spec
Zinc Finger Protein, FOG Family Member 1		Transcription regulator that plays an ess
Nudix Hydrolase 16		RNA-binding and decapping enzyme tha
Kinesin Family Member 23	The protein e	Component of the centralspindlin compl
Four-Jointed Box Kinase 1	The protein e	Acts as an inhibitor of dendrite extensior
Ectodysplasin A	The protein e	[Isoform 1]: Binds only to the receptor El
Nei Like DNA Glycosylase 3	NEIL3 belong	DNA glycosylase which prefers single-st
Chromosome 9 Open Reading Frame 47		
Potassium Voltage-Gated Cha	This gene en	Regulatory subunit of Kv4/D (Shal)-type
Rhophilin Rho GTPase Binding Protein 1		Has no enzymatic activity. May serve as
Mitotic Arrest Deficient 2 Like	MAD2L1 is a	Component of the spindle-assembly che
Tsukushi, Small Leucine Rich Proteoglycan		
Zinc Finger NFX1-Type Containing 1		
CROCC Pseudogene 3		
Glycophorin E (MNS Blood Gr	The protein e	This protein is a minor sialoglycoprotein
Diacylglycerol Kinase Epsilon	Diacylglycerc	Membrane-bound diacylglycerol kinase t
Aurora Kinase A	The protein e	Mitotic serine/threonine kinase that cont
Aurora Kinase A Pseudogene 1		
SMAD Family Member 6	The protein e	Acts as a mediator of TGF-beta and BM
HOXB Cluster Antisense RNA 1		
SMAD Family Member 7	The protein e	Antagonist of signaling by TGF-beta (tra
Zinc Finger MIZ-Type Contain	This gene en	Acts as transcriptional coactivator. Incre
SMAD Family Member 9	The protein e	Transcriptional modulator activated by B
Zinc Finger CCCH-Type Containing, Antiviral 1 Like		
Prostaglandin I2 Receptor	The protein e	Receptor for prostacyclin (prostaglandin
Nicolin 1	This protein encoded by this gene localizes to the nuc	
Endo-Beta-N-Acetylglucosam	This gene en	Endoglycosidase that releases N-glycan
Apolipoprotein L Domain Con	APOLD1 is a	May be involved in angiogenesis. May p
MutS Homolog 6	This gene en	Component of the post-replicative DNA
Prostaglandin-Endoperoxide S	Prostaglandi	Dual cyclooxygenase and peroxidase in
Major Facilitator Superfamily Domain Containing 3		
Coiled-Coil Domain Containing 14		Negatively regulates centriole duplicatio
Coiled-Coil Domain Containing 191		
MIR155 Host Gene	This gene represents a microRNA host gene. The lon	
Synaptonemal Complex Central Element P		May be involved in meiosis.
Apoptosis Enhancing Nuclease		Exonuclease with activity against single-
DNA Polymerase Theta		DNA polymerase that promotes microho
Methenyltetrahydrofolate Synthetase Domain Containing		

Transmembrane Protein 79		Contributes to the epidermal integrity an
Rho GTPase Activating Protei	ARHGAP21 f	Functions as a GTPase-activating protei
Tctex1 Domain Containing 1		
Protein Phosphatase 1 Regul	Myosin phos	Regulates myosin phosphatase activity.
Zinc Finger Protein 654		May be involved in transcriptional regula
Growth Differentiation Factor	This gene en	Regulates food intake, energy expenditu
E2F Transcription Factor 2	The protein e	Transcription activator that binds DNA c
Lipopolysaccharide Induced T	Lipopolysacc	Plays a role in endosomal protein traffick
Cyclin Dependent Kinase 10	The protein e	Cyclin-dependent kinase that phosphory
Uncharacterized LOC101928649		
ADAM Metallopeptidase With	This gene en	Cleaves aggrecan, a cartilage proteogly
Zinc Finger Protein 248		May be involved in transcriptional regula
MicroRNA 421	microRNAs (miRNAs) are short (20-24 nt) non-coding	
Zinc Finger Protein 397	This gene en	Isoform 3 acts as a DNA-dependent tran
TATA-Box Binding Protein As	TATA binding	Cell type-specific subunit of the general
Phosphoglucomutase 5 Pseudogene 2		
Dual Specificity Phosphatase	The protein e	Inactivates MAP kinases. Has a specific
Adaptor Protein, Phosphotyro	The protein e	Multifunctional adapter protein that binds
LBH Regulator Of WNT Signaling Pathway		Transcriptional activator which may act i
Family With Sequence Similarity 83 Membe		Probable proto-oncogene that regulates
Cyclin B1	The protein e	Essential for the control of the cell cycle
F-Box Protein 32	This gene en	Substrate recognition component of a S
E2F Transcription Factor 7	E2F transcrip	Atypical E2F transcription factor that par
MicroRNA 647	microRNAs (miRNAs) are short (20-24 nt) non-coding	
TP53 Induced Glycolysis Reg	This gene is	Fructose-bisphosphatase hydrolyzing fru
Zinc Finger And BTB Domain Containing 8		May be involved in transcriptional regula
BTG Anti-Proliferation Factor	The protein e	Anti-proliferative protein; the function is
Cytochrome P450 Family 2 S	This gene en	A cytochrome P450 monooxygenase inv
Core-Binding Factor Subunit	The protein e	Forms the heterodimeric complex core-b
Heparin Binding EGF Like Growth Factor		Growth factor that mediates its effects vi
Chromatin Licensing And DN	The protein e	Required for both DNA replication and m
Salvador Family WW Domain	WW domain-	Regulator of STK3/MST2 and STK4/MS
MIR34A Host Gene		
Cystathionine Beta-Synthase	The protein e	Hydro-lyase catalyzing the first step of th
Cullin 9		Core component of a Cul9-RING ubiquit
Proline Rich Transmembrane	This gene en	As a component of the outer core of AM
Kruppel Like Factor 11	The protein e	Transcription factor (PubMed:9748269,
Dynein Axonemal Assembly F	This gene en	Required for cytoplasmic pre-assembly

TSC22 Domain Family Memb	TSC22D4 is	Transcriptional repressor.
SERTA Domain Containing 1		Acts at E2F-responsive promoters as co
Phenylalanyl-TRNA Synthetas	This gene en	Is responsible for the charging of tRNA(
ANKH Inorganic Pyrophospha	This gene en	Regulates intra- and extracellular levels
Phosphoinositide-3-Kinase Re	Phosphatidyl	Binds to activated (phosphorylated) prot
Ectodermal-Neural Cortex 1	This gene en	Actin-binding protein involved in the regu
G Protein Signaling Modulato	The protein e	Plays an important role in mitotic spindle
Cysteine Rich DPF Motif Domain Containing 1		
Fas Binding Factor 1		Keratin-binding protein required for epith
Mitogen-Activated Protein Kin	The protein e	The JNK-interacting protein (JIP) group
RUNX1 Partner Transcriptiona	This gene en	Transcriptional corepressor which facilita
Dihydropyrimidinase Like 3		Necessary for signaling by class 3 sema
LIF Interleukin 6 Family Cytok	The protein e	LIF has the capacity to induce terminal
SRY-Box Transcription Facto	This gene en	Transcription factor that binds to DNA at
Ankyrin Repeat And LEM Domain Containi		Endonuclease that probably plays a role
Gem Nuclear Organelle Asso	This gene en	The SMN complex plays a catalyst role i
FERM Domain Containing 4B	This gene en	Member of GRP1 signaling complexes t
MT-RNR2 Like 8		Plays a role as a neuroprotective and ar
Nuclear Receptor Subfamily 1	This gene en	Transcriptional repressor which coordina
Nucleic Acid Binding Protein 1	Single-strand	Component of the SOSS complex, a mu
Small Integral Membrane Protein 8		
Protein Phosphatase, Mg2+/M	The protein e	Involved in the negative regulation of p5
NIMA Related Kinase 2	This gene en	[Isoform 1]: Phosphorylates and activate
Zinc Finger CCCH-Type Containing 12C		May function as RNase and regulate the
Interactor Of HORMAD1 1		Required for DNA double-strand breaks
Low Density Lipoprotein Rece	The low dens	(Microbial infection) Acts as a receptor f
Spindlin Family Member 3		Exhibits H3K4me3-binding activity.
Stomatin	This gene en	Regulates ion channel activity and trans
G Protein-Coupled Receptor	This gene is	Receptor for the neuroprotective and gli
PR/SET Domain 15		Sequence-specific DNA-binding transcri
Glycerophosphodiester Phos	This gene en	Hydrolyzes lysoglycerophospholipids to
Zinc Finger Protein 251		May be involved in transcriptional regula
Kelch Like Family Member 35		
MicroRNA 3191	microRNAs (miRNAs) are short (20-24 nt) non-coding	
G Protein Subunit Alpha 14	This gene en	Guanine nucleotide-binding proteins (G
Neural Precursor Cell Expres	The protein e	Docking protein which plays a central co
Pleckstrin Homology Domain Containing J1		
G-Quadruplex Forming Sequence Containing LncRNA		

Leucine Rich Adaptor Protein 1 Like		
Myelin Expression Factor 2		Transcriptional repressor of the myelin b
ATPase Phospholipid Transp	The protein e	P4-ATPase flippase which catalyzes the
Transforming Acidic Coiled-C	Transforming	Plays a role in the microtubule-depende
SRY-Box Transcription Facto	This intronles	Transcriptional activator that binds with
Chromosome 11 Open Reading Frame 96		
CFAP20 Domain Containing		
LFNG O-Fucosylpeptide 3-Be	This gene is	Glycosyltransferase that initiates the elo
MIRLET7B Host Gene		
Ras And Rab Interactor 1		Ras effector protein, which may serve a
Potassium Calcium-Activated	MaxiK chann	Regulatory subunit of the calcium activa
Phosphodiesterase 7B	The 3',5'-cycl	Hydrolyzes the second messenger cAM
Meteorin Like, Glial Cell Differentiation Reg		Hormone induced following exercise or
Protein Tyrosine Phosphatas	This gene en	Protein tyrosine phosphatase which stim
Tripartite Motif Containing 69	This gene en	May have E3 ubiquitin-protein ligase act
BCL2 Binding Component 3	This gene en	[Isoform 3]: Does not affect cell growth.|
E74 Like ETS Transcription F	The protein e	Transcriptional activator that binds to D
Nuclear Factor, Erythroid 2 Li	This gene en	Transcription factor that plays a key role
Nuclear Factor Of Activated T	The product	Plays a role in the inducible expression
EMAP Like 1	Human echir	Modulates the assembly and organizatic
Nuclear Factor Of Activated T	This gene is	Plays a role in the inducible expression
Leucine Zipper Protein 1	This gene encodes a protein that contains a leucine z	
Sterile Alpha Motif Domain Containing 10		
HEXIM P-TEFb Complex Sub	Expression o	Transcriptional regulator which functions
Mitogen-Activated Protein Kin	This gene en	Part of a non-canonical MAPK signaling
Intermediate Filament Family	This gene is a member of the intermediate filament fa	
Cell Division Cycle Associate	This gene en	Component of the chromosomal passen
Chromosome 21 Open Reading Frame 58		
SUMO Specific Peptidase 7	The reversibl	Protease that deconjugates SUMO2 and
IQCJ-SCHIP1 Readthrough	This locus re	May play a role in action potential condu
Ankyrin Repeat Domain 13D	The protein e	Ubiquitin-binding protein that specifically
NFKB Inhibitor Alpha	This gene en	Inhibits the activity of dimeric NF-kappa-
Solute Carrier Family 30 Member 1		May be involved in zinc transport out of
Dehydrogenase/Reductase 1	This gene encodes a member of the short-chain dehy	
Cyclin F	This gene en	Substrate recognition component of a S
Cyclin E1	The protein e	Essential for the control of the cell cycle
E74 Like ETS Transcription F	This gene en	Transcription factor that activates the LY
Kelch Repeat And BTB Domain Containing 2		

Chromosome 1 Open Reading Frame 74		
Ankyrin Repeat Domain 12	This gene en	May recruit HDACs to the p160 coactiva
C-X-C Motif Chemokine Ligan	This antimicr	Produced by activated monocytes and n
C-X-C Motif Chemokine Ligan	This antimicr	Ligand for CXCR2 (By similarity). Has ch
Small Nucleolar RNA, C/D Bo	Intronic regions of ribosomal protein genes can harbo	
Microtubule Crosslinking Factor 1		Microtubule-associated factor involved in
NK3 Homeobox 1	This gene en	Transcription factor, which binds prefere
Early Growth Response 1	The protein e	Transcriptional regulator (PubMed:2012
Multiple EGF Like Domains 6		
Tubulin Gamma Complex Ass	The protein e	Gamma-tubulin complex is necessary fo
Chromosome Transmission F	This gene en	Chromosome cohesion factor involved in
Methyltransferase Like 23	The protein e	Probable methyltransferase.
Zinc Finger BED-Type Containing 8		
Cell Division Cycle 25A	CDC25A is a	Tyrosine protein phosphatase which fun
Small Nucleolar RNA, C/D Box 14D		
Activity Regulated Cytoskeleton Associated		Master regulator of synaptic plasticity tha
Small Nucleolar RNA, C/D Box 14E		
Phospholipase A2 Inhibitor And LY6/PLAUR Domain Containing		
Growth Differentiation Factor	This gene en	May play an active role in the motor area
TBC1 Domain Family Member 3H		Acts as a GTPase activating protein for
Scavenger Receptor Class F	The protein e	Mediates the binding and degradation of
Protein Tyrosine Phosphatase	The protein encoded by this gene was identified as a	
Small Nucleolar RNA, C/D Box 14C		
Ephrin A1	This gene en	Cell surface GPI-bound ligand for Eph re
Ankyrin Repeat And Sterile Alpha Motif Dor		Regulator of different signaling pathways
Keratin 7	The protein e	Blocks interferon-dependent interphase
Cell Division Cycle 6	The protein e	Involved in the initiation of DNA replicati
Ankyrin Repeat Domain 36C		
Cell Division Cycle 20	CDC20 appe	Required for full ubiquitin ligase activity o
Chromosome 12 Open Reading Frame 60		
Cyclin Dependent Kinase 1	The protein e	(Microbial infection) Acts as a receptor fo
Extra Spindle Pole Bodies Lik	Stable cohes	Caspase-like protease, which plays a ce
Rhophilin Rho GTPase Bindir	This gene en	Binds specifically to GTP-Rho. May func
Zinc Finger Matrin-Type 1	This gene encodes a protein containing Cys2-His2 (C	
Neuralized E3 Ubiquitin Prote	The protein e	Promotes CCP110 ubiquitination and pro
Family With Sequence Similar	The protein e	Chromatin-associated protein required fo
Zinc Finger Protein 512	This gene en	May be involved in transcriptional regula
Zinc Finger Protein 366		Has transcriptional repression activity. A

SEC31 Homolog B, COPII Co	This gene en	As a component of the coat protein com
Phospholipid Phosphatase 3	The protein e	Independently of this phosphatase activi
DExH-Box Helicase 34	DEAD box pr	Probable ATP-binding RNA helicase.
Nuclear Receptor Subfamily 3	This gene en	[Isoform 10]: Has transcriptional activatio
SH2 Domain Containing 4A		Inhibits estrogen-induced cell proliferatio
Programmed Cell Death 1 Ligand 2		Involved in the costimulatory signal, ess
Hes Family BHLH Transcriptio	This protein b	Transcriptional repressor of genes that r
Mab-21 Domain Containing 2		
Serine/Threonine Kinase 32B	This gene encodes a serine-threonine protein kinase.	
Hydroxysteroid 17-Beta Dehy	The protein e	Bifunctional enzyme acting on the perox
Heat Shock Protein Family A	The protein e	Possesses chaperone activity in vitro wh
Histamine Receptor H1	Histamine is	In peripheral tissues, the H1 subclass of
Protein Tyrosine Kinase 2 Bet	This gene en	Non-receptor protein-tyrosine kinase tha
Adenylate Kinase 5	This gene en	Nucleoside monophosphate (NMP) kina
4-Aminobutyrate Aminotransfe	4-aminobutyr	Catalyzes the conversion of gamma-ami
ATP Binding Cassette Subfan	The membra	Plays an important role in the formation
Acyl-CoA Synthetase Short C	This gene en	Catalyzes the synthesis of acetyl-CoA fr
Carboxylesterase 2	This gene en	Involved in the detoxification of xenobiot
DNA Damage Regulated Auto	This gene is	Lysosomal modulator of autophagy that
Bardet-Biedl Syndrome 9	This gene is	The BBSome complex is thought to func
TBC1 Domain Family Member 5		May act as a GTPase-activating protein
Pleckstrin Homology And FYVE Domain Co		May induce apoptosis through the lysos
Activating Signal Cointegrator	This gene en	3'-5' DNA helicase involved in repair of a
Poly(ADP-Ribose) Polymerase Family Mem		ADP-ribosyltransferase which, in associa
Histone Deacetylase 9	Histones play	Isoform 3 lacks active site residues and
Heat Shock Protein 90 Alpha	The protein e	Molecular chaperone that promotes the
Calcium Binding Tyrosine Pho	To reach fert	May function as a regulator of both motil
Aconitase 1	The protein e	Catalyzes the isomerization of citrate to
Netrin 4	This gene en	May play an important role in neural, kid
UL16 Binding Protein 1	The protein e	Binds and activates the KLRK1/NKG2D
Family With Sequence Similarity 167 Member A		
TNF Receptor Superfamily Me	The protein e	Receptor for the cytotoxic ligand TRAIL.
Solute Carrier Family 9 Memb	This gene en	Involved in pH regulation to eliminate ac
EH Domain Containing 1	This gene be	ATP- and membrane-binding protein tha
Platelet Derived Growth Facto	The protein e	Growth factor that plays an essential role
DNA Polymerase Eta	This gene en	DNA polymerase specifically involved in
Fibroblast Growth Factor 5	The protein e	Plays an important role in the regulation
TNF Receptor Superfamily Me	This gene en	(Microbial infection) Acts as a receptor fo

Engulfment And Cell Motility 1	This gene en	Involved in cytoskeletal rearrangements
Growth Differentiation Factor	This gene en	Growth factor that controls proliferation a
Atypical Chemokine Receptor	The protein e	Atypical chemokine receptor that control
SWI/SNF Related, Matrix Ass	This gene en	Energy-transducing component of NURF
Interleukin 4 Induced 1	This gene en	Lysosomal L-amino-acid oxidase with hi
Phosphatase Domain Containing Paladin 1		
Neuroligin 1	This gene en	Cell surface protein involved in cell-cell-i
Solute Carrier Organic Anion Transporter F		Mediates the Na(+)-independent transpo
Zinc Finger And BTB Domain	The protein e	Transcriptional regulator with bimodal D
MOB Kinase Activator 3A		May regulate the activity of kinases.
Pleckstrin Homology Domain Containing A6		
Myosin IB		Motor protein that may participate in pro
Four And A Half LIM Domains	This gene en	May function as a molecular transmitter
Adducin 3	Adducins are	Membrane-cytoskeleton-associated prot
Diacylglycerol O-Acyltransfera	This gene en	Essential acyltransferase that catalyzes
Actin Filament Associated Protein 1 Like 1		May be involved in podosome and invad
ADP-Ribosylarginine Hydrolas	The enzyme	Specifically acts as a arginine mono-AD
Poly(ADP-Ribose) Polymeras	This gene en	Mono-ADP-ribosyltransferase that media
NBAS Subunit Of NRZ Tether	This gene en	Involved in Golgi-to-endoplasmic reticulu
FIG4 Phosphoinositide 5-Pho	The protein e	The PI(3,5)P2 regulatory complex regula
3-Hydroxy-3-Methylglutaryl-Co	The protein e	Mitochondrial 3-hydroxymethyl-3-methyl
Adrenoceptor Beta 2	This gene en	Beta-adrenergic receptors mediate the c
Mitogen-Activated Protein Kin	The protein e	Dual specificity kinase. Is activated by c
Mitogen-Activated Protein Kin	The protein e	Acts as a scaffold for the formation of a
Major Histocompatibility Com	MAIT (mucos	Antigen-presenting molecule specialized
Interferon Regulatory Factor 2	This gene en	Probable E3 ubiquitin protein ligase invo
Glutamyl-Prolyl-TRNA Synthe	Aminoacyl-tR	Multifunctional protein which is primarily
Adenylate Kinase 9	The protein e	Involved in maintaining the homeostasis
Tubulin Beta 6 Class V		Tubulin is the major constituent of micro
Tetratricopeptide Repeat Domain 30B		Required for polyglutamylation of axone
Apolipoprotein B MRNA Editir	This gene is	DNA deaminase (cytidine deaminase) w
Serine Dehydratase Like		Has low serine dehydratase and threoni
Coiled-Coil Domain Containing 69		May act as a scaffold to regulate the rec
Protein Kinase CAMP-Depen	The protein e	Regulatory subunit of the cAMP-depend
Solute Carrier Family 22 Mem	SLC22A23 belongs to a large family of transmembran	
Cytokine Like 1	C17 is a cytokine-like protein specifically expressed in	
Fatty Acid Hydroxylase Domain Containing		Promotes megakaryocyte differentiation
HIC ZBTB Transcriptional Re	This gene fu	Transcriptional repressor. Recognizes a

Mitochondria Localized Glutamic Acid Rich		Plays a role in the trafficking of mitochor
StAR Related Lipid Transfer Domain Conta		May play metabolic roles in sperm matui
NADPH Oxidase Activator 1	This gene en	Functions as an activator of NOX1, a su
TNF Superfamily Member 15	The protein e	Receptor for TNFRSF25 and TNFRSF6I
Copine 3	Calcium-dep	Calcium-dependent phospholipid-bindin
Interferon Induced With Helic	DEAD box pr	Innate immune receptor which acts as a
DOP1 Leucine Zipper Like Protein B		May be involved in protein traffic betwee
Phosphoglycerate Dehydroge	This gene en	Catalyzes the reversible oxidation of 3-p
UBA Like Domain Containing 2		
Caspase Recruitment Domair	This gene en	May be involved in apoptosis.
Aldehyde Dehydrogenase 3 F	Aldehyde del	Catalyzes the oxidation of medium and l
TRAF3 Interacting Protein 2	This gene en	Could be involved in the activation of bo
Niban Apoptosis Regulator 1	This gene en	Regulates phosphorylation of a number
NLR Family CARD Domain C	This gene en	Negative regulator of the innate immune
Carboxymethylenebutenolida	CMBL (EC 3.	Cysteine hydrolase. Can convert the pro
Ras And Rab Interactor Like		Guanine nucleotide exchange factor (GE
TSC22 Domain Family Memb	This gene en	Transcriptional repressor. Acts on the C
Ras Association Domain Fam	The function	Potential tumor suppressor. May act as
Target Of Myb1 Like 1 Membrane Traffickir		Probable adapter protein involved in sig
Claudin 1	Tight junctior	(Microbial infection) Acts as a receptor f
PC-Esterase Domain Contain	This gene encodes a protein that belongs to the GDS	
Fermitin Family Member 1	This gene en	Involved in cell adhesion. Contributes to
Filamin A Interacting Protein 1 Like		Acts as a regulator of the antiangiogenic
Solute Carrier Family 7 Memb	The protein e	Involved in the sodium-independent upta
Phosphatase And Actin Regu	The protein e	Binds actin monomers (G actin) and pla
Myelin Basic Protein	The protein e	The classic group of MBP isoforms (isof
Carboxypeptidase A4	This gene is	Metalloprotease that could be involved i
DExD/H-Box Helicase 60	DEAD box pr	Positively regulates DDX58/RIG-I- and I
Annexin A4	Annexin IV (A	Calcium/phospholipid-binding protein wh
Signal Regulatory Protein Beta 2		
Interleukin 1 Alpha	The protein e	Produced by activated macrophages, IL
SH2 Domain Containing 2A	This gene en	Could be a T-cell-specific adapter protei
Glutamyl-TRNA Amidotransferase Subunit		Allows the formation of correctly charge
Polypeptide N-Acetylgalactosaminyltransfe		Catalyzes the initial reaction in O-linked
Proline-Serine-Threonine Phosphatase Inte		Binds to F-actin. May be involved in regu
Coiled-Coil Domain Containing 85C		May play a role in cell-cell adhesion and
G Protein-Coupled Receptor (	This gene en	Orphan receptor. Could be involved in m
Aldehyde Oxidase 1	Aldehyde oxi	Oxidase with broad substrate specificity,

Ankyrin 3	Ankyrins are	[Isoform 5]: May be part of a Golgi-speci
CAMP Responsive Element E	cAMP respor	Probable regulator of CREB1 transcriptio
Polypeptide N-Acetylgalactosa	This gene en	Catalyzes the initial reaction in O-linked
CAMP Responsive Element M	This gene en	[Isoform 6]: May play a role in the regula
GTF2I Repeat Domain Contai	This gene encodes a glycosylated phosphoprotein wit	
C-C Motif Chemokine Recept	This gene en	Receptor for CCL19 and chemerin/RARI
Transmembrane And Coiled-Coil Domains 4		
Secreted And Transmembran	This gene en	May be involved in thymocyte signaling.
RAD50 Double Strand Break	The protein e	Component of the MRN complex, which
Uncoupling Protein 2	Mitochondria	UCP are mitochondrial transporter prote
Protocadherin 12	This gene be	Cellular adhesion molecule that may pla
Collagen Type XVII Alpha 1 C	This gene en	May play a role in the integrity of hemide
Platelet Derived Growth Facto	This gene en	Growth factor that plays an essential role
Succinate Dehydrogenase Co	This gene en	Flavoprotein (FP) subunit of succinate d
Tetraspanin 1	The protein encoded by this gene is a member of the	
Apolipoprotein D	This gene en	APOD occurs in the macromolecular cor
Family With Sequence Similarity 107 Memb		Stress-inducible actin-binding protein tha
Platelet Derived Growth Facto	This gene encodes a protein with significant sequence	
Syndecan 1	The protein e	Cell surface proteoglycan that bears bot
Complement C1r Subcomponent Like		Mediates the proteolytic cleavage of HP/
C-X-C Motif Chemokine Ligan	This gene en	Involved in neutrophil activation. In vitro,
Alpha-L-Fucosidase 1	The protein e	Alpha-L-fucosidase is responsible for hy
Amyloid Beta Precursor Like F	This gene en	May play a role in postsynaptic function.
DExD/H-Box Helicase 58	DEAD box pr	Innate immune receptor that senses cyto
MCC Regulator Of WNT Sign	This gene is	Candidate for the putative colorectal tum
Carnitine O-Octanoyltransfera	This gene en	Beta-oxidation of fatty acids. The highes
Proprotein Convertase Subtilis	This gene en	Involved in the processing of hormone a
Stimulator Of Interferon Resp	This gene en	(Microbial infection) Antiviral activity is a
Signal Transducing Adaptor F	This gene en	Substrate of protein kinase PTK6. May p
Sperm Tail PG-Rich Repeat Containing 1		May positively contribute to the inductior
Thymidylate Synthetase	Thymidylate s	Contributes to the de novo mitochondria
Radial Spoke Head 3	The protein e	Functions as a protein kinase A-anchori
Protein Tyrosine Phosphatase	This gene en	Protein tyrosine phosphatase which stim
Phosphodiesterase 1C	This gene en	Calmodulin-dependent cyclic nucleotide
Solute Carrier Family 15 Member 3		Proton oligopeptide cotransporter. Trans
Prolyl 4-Hydroxylase Subunit	This gene en	Catalyzes the post-translational formatio
Aquaporin 3 (Gill Blood Grour	This gene en	Water channel required to promote glyce
Coiled-Coil Domain Containing 125		May be involved in the regulation of cell

Patched Domain Containing 4		Could act as a repressor of canonical he
Arrestin Beta 1	Members of a	Functions in regulating agonist-mediatec
TNF Superfamily Member 4	This gene en	Cytokine that binds to TNFRSF4. Co-stir
Teneurin Transmembrane Prc	This gene en	Involved in neural development by regul
N-Ethylmaleimide-Sensitive Factor Pseudogene 1		
Fms Related Receptor Tyrosi	Dendritic cell	Stimulates the proliferation of early hem
Poly(ADP-Ribose) Polymeras	This gene en	ADP-ribosyltransferase that mediates m
Muscleblind Like Splicing Reg	This gene is	Mediates pre-mRNA alternative splicing
Epstein-Barr Virus Induced 3	This gene wa	Associates with IL27 to form the IL-27 in
Zinc Finger CCCH-Type Cont	This gene en	Antiviral protein which inhibits the replica
RALBP1 Associated Eps Dom	The product	Involved in growth factor signaling throu
Optineurin	This gene en	(Microbial infection) May constitute a cel
Plakophilin 2	This gene en	May play a role in junctional plaques.
Oxysterol Binding Protein 2	The protein e	Binds 7-ketocholesterol.
Cathepsin S	The prepropr	Thiol protease. Key protease responsibl
Pyruvate Kinase M1/2	This gene en	Glycolytic enzyme that catalyzes the tra
SP110 Nuclear Body Protein	The nuclear I	Transcription factor. May be a nuclear h
Plasminogen Activator, Tissue	This gene en	Converts the abundant, but inactive, zyn
FERM Domain Containing 4A	This gene en	Scaffolding protein that regulates epithe
Dysbindin Domain Containing 1		
Aprataxin And PNKP Like Fac	C2ORF13 is	Nuclease involved in single-strand and c
Phospholipase D1	This gene en	Implicated as a critical step in numerous
ADP Ribosylation Factor Like	ADP-ribosyla	Small GTP-binding protein which cycles
ChaC Glutathione Specific Ga	This gene en	Catalyzes the cleavage of glutathione in
Syntaxin 6		Involved in intracellular vesicle trafficking
N-Deacetylase And N-Sulfotra	This gene en	Essential bifunctional enzyme that cataly
Beta-1,4-Glucuronyltransferas	This gene en	Beta-1,4-glucuronyltransferase involved
Matrix Metallopeptidase 14	Proteins of th	Endopeptidase that degrades various cc
Chondroitin Sulfate N-Acetylg	This gene en	Transfers 1,4-N-acetylgalactosamine (G
Phosphatidylinositol-4,5-Bisph	Phosphoinos	Phosphoinositide-3-kinase (PI3K) that p
MINDY Lysine 48 Deubiquitinase 1		Hydrolase that can specifically remove 'L
Adrenomedullin 2	This gene en	[Adrenomedullin-2]: May play a role as p
Deltex E3 Ubiquitin Ligase 3L	DTX3L functi	E3 ubiquitin-protein ligase which, in ass
PDZ And LIM Domain 1	This gene en	Cytoskeletal protein that may act as an a
Ajuba LIM Protein		Adapter or scaffold protein which partici
Aldehyde Dehydrogenase 7 F	The protein e	Multifunctional enzyme mediating impor
Prenylcysteine Oxidase 1	Prenylcysteir	Involved in the degradation of prenylate
N-Myc And STAT Interactor	NMYC intera	May be involved in augmenting coactiva

ATPase Plasma Membrane C	The protein e	Calcium/calmodulin-regulated and magr
Intercellular Adhesion Molecu	The protein e	ICAM proteins are ligands for the leukoc
Phytanoyl-CoA 2-Hydroxylase	This gene is	Catalyzes the 2-hydroxylation of not only
RAB38, Member RAS Oncogene Family		May be involved in melanosomal transpo
Mesenteric Estrogen Dependent Adipogene		Involved in processes that promote adip
Transmembrane Protein 217		
Heterogeneous Nuclear Ribonucleoprotein A1 Pseudogene 7		
FERM Domain Containing 6		
DNA Damage Inducible Transcript 4 Like		Inhibits cell growth by regulating the TOI
Intercellular Adhesion Molecu	This gene en	(Microbial infection) Acts as a receptor fo
Nuclear Receptor Subfamily 3	This gene en	Receptor for both mineralocorticoids (MC
Damage Specific DNA Binding	The protein e	Protein, which is both involved in DNA re
Coagulation Factor VIII Assoc	This gene is	RAB5A effector molecule that is involved
Transglutaminase 2	Transglutami	Catalyzes the cross-linking of proteins, s
Eukaryotic Elongation Factor, Selenocystei		Translation factor necessary for the inco
MER Proto-Oncogene, Tyrosi	This gene is	Receptor tyrosine kinase that transduces
Elongation Factor Like GTPase 1		Involved in the biogenesis of the 60S rib
WSC Domain Containing 1		
REV3 Like, DNA Directed Pol	The protein e	Catalytic subunit of the DNA polymerase
Opioid Growth Factor Receptor Like 1		
AXL Receptor Tyrosine Kinas	The protein e	(Microbial infection) Acts as a receptor fo
RELB Proto-Oncogene, NF-KB Subunit		NF-kappa-B is a pleiotropic transcription
Chromosome 14 Open Reading Frame 93		
Inducible T Cell Costimulator Ligand		Ligand for the T-cell-specific cell surface
Zyg-11 Related Cell Cycle Re	This gene en	Serves as substrate adapter subunit in tl
Ubiquitin Conjugating Enzyme	The modifica	Catalyzes the covalent attachment of ub
KiSS-1 Metastasis Suppresso	This gene is	Metastasis suppressor protein in maligna
Interleukin 32	This gene en	Cytokine that may play a role in innate a
Apolipoprotein L3	This gene is	May affect the movement of lipids in the
SAM And SH3 Domain Conta	This gene en	Is a positive regulator of NF-kappa-B sig
Dysferlin	The protein e	Key calcium ion sensor involved in the C
Aspartyl-TRNA Synthetase 1	This gene en	Catalyzes the specific attachment of an
DAB Adaptor Protein 2	This gene en	Adapter protein that functions as clathrir
Rhomboid 5 Homolog 2		Regulates ADAM17 protease, a shedda
Diacylglycerol Kinase Alpha	The protein e	Diacylglycerol kinase that converts diacy
Spectrin Repeat Containing N	This gene en	Multi-isomeric modular protein which for
Potassium Calcium-Activated	MaxiK chann	Potassium channel activated by both me
Toll Like Receptor 1	The protein e	Participates in the innate immune respo

Potassium Calcium-Activated	The protein e	Forms a voltage-independent potassium
Toll Like Receptor 3	The protein e	Key component of innate and adaptive i
N-Ethylmaleimide Sensitive Factor, Vesicle		Required for vesicle-mediated transport.
Angiopoietin Like 4	This gene en	[ANGPTL4 N-terminal chain]: Mediates i
Galactose-3-O-Sulfotransfera	This gene en	Catalyzes the transfer of sulfate to beta-
RAP2B, Member Of RAS Onc	This intronles	Small GTP-binding protein which cycles
Glutamine Amidotransferase I	This gene encodes a potential mitochondrial protein th	
2'-5'-Oligoadenylate Syntheta	This gene en	Interferon-induced, dsRNA-activated ant
Cbp/P300 Interacting Transac	The protein e	Transcriptional coactivator of the p300/C
2'-5'-Oligoadenylate Syntheta	This gene en	Interferon-induced, dsRNA-activated ant
CD82 Molecule	This metasta	Associates with CD4 or CD8 and deliver
Apolipoprotein L6	This gene is a	May affect the movement of lipids in the
Thyroid Hormone Receptor In	This gene en	Transcription coactivator which associat
Myosin VC		May be involved in transferrin trafficking.
Junction Plakoglobin	This gene en	Common junctional plaque protein. The
CRACD Like		
Dual Specificity Phosphatase	This gene en	Dual specificity protein phosphatase inv
Bardet-Biedl Syndrome 2	This gene is a	The BBSome complex is thought to func
DEAD-Box Helicase 18 Pseudogene 1		
Phosphotriesterase Related		
Double PHD Fingers 3	This gene en	Belongs to the neuron-specific chromati
Tetratricopeptide Repeat Domain 12		
Spermatogenesis Associated	This gene en	Key regulator of mitochondrial quality th
Cyclin D1	The protein e	Regulatory component of the cyclin D1-C
GDP-Mannose 4,6-Dehydrata	GDP-manno	Catalyzes the conversion of GDP-D-mar
Divergent Protein Kinase Domain 2B		
ADAM Metallopeptidase With	This gene encodes a member of the ADAMTS (a disir	
Oxoglutarate Dehydrogenase	This gene en	2-oxoglutarate dehydrogenase (E1) com
Tribbles Pseudokinase 3	The protein e	Inactive protein kinase which acts as a r
BCL2 Like 1	The protein e	Isoform Bcl-X(L) also regulates presyna
Family With Sequence Similarity 210 Memb		Plays a role in erythroid differentiation (F
Sulfatase 2	Heparan sulf	Exhibits arylsulfatase activity and highly
Dynein Cytoplasmic 1 Intermediate Chain 2 Pseudogene 1		
Transcription Elongation Factor A3		Necessary for efficient RNA polymerase
Angiopoietin Like 2	Angiopoietins	Induces sprouting in endothelial cells thr
Dynein Cytoplasmic 1 Interme	This gene en	Acts as one of several non-catalytic acce
TNFAIP3 Interacting Protein 1	This gene en	Inhibits NF-kappa-B activation and TNF-
Ras Homolog Family Member	Ras homolog	Involved in endosome dynamics. May co

Integrin Subunit Alpha 2	This gene en	(Microbial infection) Integrin ITGA2:ITGE
Succinyl-CoA:Glutarate-CoA	This gene en	Catalyzes the succinyl-CoA-dependent c
MORN Repeat Containing 4		Plays a role in promoting axonal degene
Integrin Subunit Alpha 3	The gene en	Integrin alpha-3/beta-1 is a receptor for f
Diaphanous Related Formin 2	The product	Could be involved in oogenesis. Involved
Polypeptide N-Acetylgalactosa	This gene en	Catalyzes the initial reaction in O-linked
Epoxide Hydrolase 4		
Discs Large MAGUK Scaffold	This gene en	Essential multidomain scaffolding protein
Calmin		
Phosphoserine Aminotransfer	This gene en	Catalyzes the reversible conversion of 3
Schwannomin Interacting Protein 1		
THAP Domain Containing 8		
Cell Death Inducing P53 Target 1		Acts as an important p53/TP53-apoptoti
Interleukin 1 Receptor Associa	IRAK2 encod	Binds to the IL-1 type I receptor following
DENN Domain Containing 1A	Clathrin (see	Guanine nucleotide exchange factor (GE
WDFY Family Member 4		Plays a critical role in the regulation of cl
BCL2 Interacting Protein 3 Lik	This gene en	Induces apoptosis. Interacts with viral ar
Solute Carrier Family 7 Memb	This gene is	May be involved in arginine transport.
H2B Clustered Histone 12	Histones are	Core component of nucleosome. Nucleo
Purinergic Receptor P2X 4	The product	Receptor for ATP that acts as a ligand-g
Iron-Sulfur Cluster Assembly	This gene en	[Isoform 2]: Functions as a cytoplasmic s
Tripartite Motif Containing 22	The protein e	Interferon-induced antiviral protein involv
Lysyl Oxidase Like 4	This gene en	May modulate the formation of a collage
Uncharacterized LL21NC02-21A1.1		
Coiled-Coil Domain Containing 136		May play a role in acrosome formation ir
Zinc Finger And AT-Hook Dor	This gene en	May be involved in transcriptional regula
Inositol Polyphosphate-5-Pho	This gene is	Phosphatidylinositol (PtdIns) phosphatas
Ribonuclease L	This gene en	Endoribonuclease that functions in the ir
Tensin 3		May play a role in actin remodeling. Invo
Monoglyceride Lipase	This gene en	Converts monoacylglycerides to free fatt
Gephyrin	This gene en	Microtubule-associated protein involved
H2B Clustered Histone 4	Histones are	Core component of nucleosome. Nucleo
Paralemmin	This gene en	Involved in plasma membrane dynamics
Negative Regulator Of Reactive Oxygen Sp		Key regulator of transforming growth fac
Sterile Alpha Motif Domain Co	This gene en	May play a role in the inflammatory resp
Myosin Heavy Chain 16 Pseu	The MYH16	Has most probably lost the function in m
Cell Adhesion Molecule 4		Involved in the cell-cell adhesion. Has ca
Gasdermin E	Hearing impa	[Gasdermin-E, N-terminal]: Switches CA

Platelet Derived Growth Facto	The protein e	Growth factor that plays an essential role
Interleukin 12A	This gene en	Cytokine that can act as a growth factor
Major Facilitator Superfamily Domain Containing 6		
Guanylate Binding Protein 3	This gene en	Exhibits antiviral activity against influenz
Catenin Beta Interacting Prote	The protein e	Prevents the interaction between CTNN
SH3 Domain Binding Protein 5		Functions as guanine nucleotide exchan
Proteasome 26S Subunit, No	The 26S prot	Component of the 26S proteasome, a m
Carbohydrate Sulfotransferas	This locus er	Sulfotransferase that utilizes 3'-phospho
Rabphilin 3A Like (Without C2	The protein e	Rab GTPase effector involved in the late
Eukaryotic Translation Elonga	This gene en	This protein promotes the GTP-depende
Synaptotagmin 9		May be involved in Ca(2+)-dependent ex
Proteasome 26S Subunit, No	The 26S prot	Binds to the intracellular domain of tumo
Y-Box Binding Protein 3		Binds to the GM-CSF promoter. Seems
Rho GTPase Activating Prote	This gene en	Rho GTPase-activating protein involved
Apolipoprotein L1	This gene en	May play a role in lipid exchange and tra
Proteasome 20S Subunit Beta	The proteaso	The proteasome is a multicatalytic prote
NBR1 Autophagy Cargo Rece	The protein e	Acts probably as a receptor for selective
EH Domain Containing 2	This gene en	ATP- and membrane-binding protein tha
Myristoylated Alanine Rich Pr	The protein e	MARCKS is the most prominent cellular
Solute Carrier Family 47 Mem	This gene is	Solute transporter for tetraethylammoniu
HLA Complex Group 11		
SMAD Family Member 3	The SMAD fa	Receptor-regulated SMAD (R-SMAD) th
Myosin Light Chain Kinase	This gene, a	Calcium/calmodulin-dependent myosin l
Cilia And Flagella Associated	This protein e	Involved in spermatozoa motility (PubMe
LDL Receptor Related Protein	This gene en	(Microbial infection) Functions as a rece
SH3 Domain Containing Ring Finger 3		Has E3 ubiquitin-protein ligase activity.
NIPA Like Domain Containing 3		
Signal Induced Proliferation A	This gene be	Plays a critical role in epithelial cell morp
Membrane Palmitoylated Prot	This gene en	May play a role in retinal photoreceptors
Tripartite Motif Containing 5	The protein e	Capsid-specific restriction factor that pre
Transmembrane Protein 140		
Leucine Rich Repeat Containing 71		
Lipase Maturation Factor 1		Involved in the maturation of specific pro
Leukotriene A4 Hydrolase	The protein e	Epoxide hydrolase that catalyzes the fina
Hydroxysteroid Dehydrogenase Like 2		Has apparently no steroid dehydrogenas
LDL Receptor Related Protein	This gene en	Probable receptor, which is involved in tl
Cytoplasmic FMR1 Interacting Protein 2		Involved in T-cell adhesion and p53/TP5
EPS8 Like 2	This gene en	Stimulates guanine exchange activity of

Basic Helix-Loop-Helix Family	This gene en	Transcriptional repressor involved in the
CUB Domain Containing Prot	This gene en	May be involved in cell adhesion and ce
Lamin A/C	The nuclear l	Lamins are components of the nuclear la
Leucine Rich Repeat Containi	This gene encodes a member of the leucine-rich repe	
F11 Receptor	Tight junctior	(Microbial infection) Acts as a receptor fo
Par-6 Family Cell Polarity Reg	This gene is	Adapter protein involved in asymmetrica
Cyclin D2	The protein e	Regulatory component of the cyclin D2-C
Pleckstrin Homology And RhoGEF Domain Containing G1		
Serine Peptidase Inhibitor, Ku	This gene en	Inhibitor of HGF activator. Also inhibits p
RAB3D, Member RAS Oncogene Family		Protein transport. Probably involved in re
V-Set And Immunoglobulin Do	This gene encodes a member of the junctional adhesi	
Corepressor Interacting With RBPJ, CIR1		May modulate splice site selection durin
GAS6 Divergent Transcript		
Tumor Protein P53 Inducible Protein 11		
Ethanolamine Kinase 2	The protein e	Highly specific for ethanolamine phosph
H2B Clustered Histone 5	Histones are	Core component of nucleosome. Nucleo
PNMA Family Member 8A		
CUGBP Elav-Like Family Mem	Members of t	RNA-binding protein implicated in the re
Actin Binding LIM Protein 1	This gene en	May act as scaffold protein (By similarity
KH RNA Binding Domain Containing, Signa		(Microbial infection) Involved in post-tran
Tetratricopeptide Repeat Domain 30A		Required for polyglutamylation of axonem
Dihydropyrimidinase Like 2	This gene en	Plays a role in neuronal development an
Apolipoprotein B MRNA Editir	This gene is	DNA deaminase (cytidine deaminase) w
Caspase 1	This gene en	Thiol protease that cleaves IL-1 beta bet
Transporter 1, ATP Binding C	The membra	Involved in the transport of antigens from
PNMA Family Member 2		
LIM Zinc Finger Domain Conta	The protein e	Adapter protein in a cytoplasmic comple
Regulatory Associated Protein	This gene en	Involved in the control of the mammalian
SLIT-ROBO Rho GTPase Act	The protein e	GTPase-activating protein for RhoA and
Erythrocyte Membrane Protein Band 4.1 Lil		Tumor suppressor that inhibits cell prolif
C-X-C Motif Chemokine Rece	This gene en	(Microbial infection) Acts as a corecepto
Caspase 7	This gene en	Involved in the activation cascade of cas
NAC Alpha Domain Containing		May prevent inappropriate targeting of n
Keratin 80	Keratins are intermediate filament proteins responsibl	
Endosome-Lysosome Associa	Expression o	May protect cells from cell death by indu
Golgi Phosphoprotein 3 Like	The Golgi co	Phosphatidylinositol-4-phosphate-bindin
NIMA Related Kinase 1	The protein e	Phosphorylates serines and threonines,
ATP Binding Cassette Subfan	The protein e	Catalyzes the efflux of phospholipids suc

Nudix Hydrolase 8		Probably mediates the hydrolysis of som
Lysosomal Associated Membr	Dendritic cell	May play a role in dendritic cell function
EPH Receptor A4	This gene be	Receptor tyrosine kinase which binds m
INSC Spindle Orientation Ada	In Drosophila	May function as an adapter linking the P
Nuclear Factor, Erythroid 2 Lil	This gene en	Activates erythroid-specific, globin gene
CAMP Responsive Element B	The protein e	(Microbial infection) May play a role in lir
Cartilage Acidic Protein 1	This gene encodes a glycosylated extracellular matrix	
Glutathione Peroxidase 3	The protein e	Protects cells and enzymes from oxidati
Nuclear Receptor Corepresso	This gene en	Transcriptional corepressor (PubMed:20
Laminin Subunit Beta 3	The product	Binding to cells via a high affinity recepto
PBX Homeobox Interacting P	The protein e	Regulator of pre-B-cell leukemia transcri
Laminin Subunit Gamma 2	Laminins, a f	Binding to cells via a high affinity recepto
Phospholipase C Like 2		May play an role in the regulation of Ins(
Glypican 1	Cell surface	Cell surface proteoglycan that bears hep
Neurofibromin 2	This gene en	Probable regulator of the Hippo/SWH (S
T Cell Receptor Alpha Consta	T cell recepto	Constant region of T cell receptor (TR) a
Serine Peptidase Inhibitor, Ku	The protein e	Inhibitor of HGF activator. Also acts as a
Fasciculation And Elongation	This gene is	May be involved in axonal outgrowth as
ORAI Calcium Release-Activated Calcium I		Ca(2+) release-activated Ca(2+)-like (CF
Cyclin G2	The eukaryot	May play a role in growth regulation and
Serpin Family B Member 1	The protein e	Neutrophil serine protease inhibitor that
Cyclin G1	The eukaryot	May play a role in growth regulation. Is a
Tripartite Motif Containing 21	This gene en	E3 ubiquitin-protein ligase whose activity
T Cell Activation Inhibitor, Mitochondrial		May regulate T-cell apoptosis.
Zinc Finger Protein 385A	Zinc finger pr	RNA-binding protein that affects the loca
Phospholipase A2 Group IVC	This gene en	Has a preference for arachidonic acid at
Ninjurin 1	The ninjurin	Homophilic cell adhesion molecule that
RuvB Like AAA ATPase 1	This gene en	Possesses single-stranded DNA-stimula
Keratin 17	This gene en	Type I keratin involved in the formation a
HHIP Like 1	This gene encodes a protein that belongs to the glucc	
Semaphorin 4B		Inhibits axonal extension by providing lo
Yippee Like 1	This gene is	May play a role in epithelioid conversion
Keratin 18	KRT18 encod	Involved in the uptake of thrombin-antith
Astrotactin 2	This gene en	Mediates recycling of the neuronal cell a
Acid Phosphatase 7, Tartrate	Purple acid phosphatases (PAPs), including PAPL, ar	
Gelsolin	The protein e	Calcium-regulated, actin-modulating pro
Ly6/Neurotoxin 1	This gene en	Acts in different tissues through interacti
Myosin VIIA And Rab Interacting Protein		Rab effector protein involved in melanos

Cytoglobin	This gene en	May have a protective function during co
Zinc Finger Protein 521		Transcription factor that can both act as
Syncoilin, Intermediate Filame	This gene en	Atypical type III intermediate filament (IF
Enoyl-CoA Hydratase And 3-H	The protein e	Peroxisomal trifunctional enzyme posse
Eukaryotic Translation Elonga	This gene en	Catalyzes the GTP-dependent ribosoma
Signal Transducer And Activa	The protein e	Signal transducer and transcription activ
Charged Multivesicular Body I	This gene en	(Microbial infection) The ESCRT machin
Family With Sequence Similarity 131 Member B		
Zinc Finger Protein 185 With I	Zinc-finger pr	May be involved in the regulation of cellu
Cytidine Deaminase	This gene en	This enzyme scavenges exogenous and
Sulfide Quinone Oxidoreducta	The protein e	Catalyzes the oxidation of hydrogen sulf
Ubiquitin D	This gene en	Ubiquitin-like protein modifier which can
Ephrin B1	The protein e	Cell surface transmembrane ligand for E
Stanniocalcin 1	This gene en	Stimulates renal phosphate reabsorption
MAM Domain Containing 2		
Extracellular Leucine Rich Repeat And Fibr		Inhibits phosphatase activity of protein p
CD79b Molecule	The B lymph	Required in cooperation with CD79A for
ST13, Hsp70 Interacting Protein Pseudogene 3		
DENN Domain Containing 2B	This gene wa	[Isoform 1]: May be involved in cytoskele
Solute Carrier Family 27 Mem	This gene be	Has acyl-CoA ligase activity for long-cha
Nuclear Paraspeckle Assemb	This gene produces a long non-coding RNA (lncRNA)	
Zinc Finger Protein 789		May be involved in transcriptional regula
DnaJ Heat Shock Protein Fan	The protein e	Probable component of the PAM comple
Phosphatidylinositol Specific I	This gene is the most terminal protein-coding gene in	
Zinc Finger GRF-Type Contai	The encoded protein contains GRF zinc finger (zf-GR	
Small Nucleolar RNA, H/ACA Box 33		
Small Nucleolar RNA, C/D Box 100		
FA Complementation Group E	This gene en	DNA repair protein required for FANCD2
Small Nucleolar RNA, C/D Box 101		
Solute Carrier Family 22 Member 31		Organic anion transporter that mediates
Ribosomal Protein L37 Pseudogene 2		
DND MicroRNA-Mediated Rep	This gene en	RNA-binding factor that positively regula
Glycerophosphodiester Phosphodiesterase		Hydrolyzes lysoglycerophospholipids to
Solute Carrier Organic Anion	This gene en	May mediate the release of newly synthe
Nucleolar Protein 12		May bind to 28S rRNA.
Leucine Rich Single-Pass Membrane Protein 1		
Zinc Finger Protein 121		May be involved in transcriptional regula
ATP Synthase F1 Subunit Epsilon Pseudog		Mitochondrial membrane ATP synthase

MicroRNA 4691	microRNAs (miRNAs) are short (20-24 nt) non-coding	
Derlin 3	The protein e	Functional component of endoplasmic re
RNA, 7SL, Cytoplasmic 431, Pseudogene		
Acetyl-CoA Acetyltransferase	The product (	Involved in the biosynthetic pathway of c
Calcium Voltage-Gated Chan	The protein e	Regulates the activity of L-type calcium (
Small Nucleolar RNA Host Ge	This gene represents a snoRNA host gene that produ	
Ubiquitin Specific Peptidase 32 Pseudogene 1		
POU Class 4 Homeobox 1	This gene en	[Isoform 2]: Able to act as transcription f
Lysine Methyltransferase 2B	This gene en	Histone methyltransferase that methylat
ArfGAP With GTPase Domain, Ankyrin Rep		Putative GTPase-activating protein.
Polycystin 1, Transient Receptor Potential Channel Interacting Pseudogene 6		
Heat Shock Transcription Fac	Heat-shock t	DNA-binding protein that specifically bin
NADH:Ubiquinone Oxidoredu	The NADH-u	Arginine hydroxylase involved in the ass
Membrane Palmitoylated Prot	This gene product is a member of a family of membra	
Adaptor Related Protein Com	This gene en	Part of the AP-3 complex, an adaptor-re
MicroRNA 1304	microRNAs (miRNAs) are short (20-24 nt) non-coding	
Ubiquitin Conjugating Enzyme E2 S Pseudogene 1		
DGCR8 Microprocessor Comp	This gene en	Component of the microprocessor comp
G Protein-Coupled Receptor 7	GPR75 is a r	G protein-coupled receptor that is activa
Glucose-Fructose Oxidoreductase Domain Containing 1		
LDL Receptor Related Protein 5 Like		
DNA Polymerase Epsilon, Ca	This gene en	Catalytic component of the DNA polyme
Fibroblast Growth Factor 12	The protein e	Involved in nervous system developmen
Nuclear Pore Complex Interacting Protein Family Member B11		
Protein Tyrosine Phosphatase	The protein e	May play roles in cilia formation and/or n
Sirtuin 7	This gene en	NAD-dependent protein-lysine deacylas
CCAAT Enhancer Binding Pro	The protein e	Transcription activator that recognizes tv
Zinc Finger Protein 354B		May be involved in transcriptional regula
Maternally Expressed 3	This gene is a maternally expressed imprinted gene. I	
SWI/SNF Related, Matrix Ass	The protein e	Involved in transcriptional activation and
NADPH Dependent Diflavin O	This gene en	Component of the cytosolic iron-sulfur (F
TSPY Like 2	This gene en	Part of the CASK/TBR1/TSPYL2 transcr
Zinc Finger Protein 10	The protein e	May be involved in transcriptional regula
Adhesion G Protein-Coupled	This gene en	Calcium-independent receptor of high af
Sperm Acrosome Associated 6		Sperm protein potentially involved sperm
5'-Nucleotidase Domain Containing 3		
Small Nuclear RNA Activating	This gene en	Part of the SNAPc complex required for

Transmembrane And Coiled-Coil Domains 6		
SFI1 Centrin Binding Protein		Plays a role in the dynamic structure of c
Smoothened, Frizzled Class F	The protein e	G protein-coupled receptor that probably
Small Nucleolar RNA, C/D Box 104		
Serine And Arginine Rich Spli	The protein e	Required for pre-mRNA splicing. Can als
Ghrelin Opposite Strand/Antis	This gene is an antisense gene of the ghrelin/obestati	
3-Hydroxy-3-Methylglutaryl-Co	HMG-CoA re	Transmembrane glycoprotein that is the
ERCC Excision Repair 6, Chro	This gene en	Essential factor involved in transcription-
Adrenomedullin	The protein e	AM and PAMP are potent hypotensive a
Small G Protein Signaling Mo	The protein e	Possesses GTPase activator activity tow
Mature T Cell Proliferation 1	This gene wa	Enhances the phosphorylation and activ
Leukotriene B4 Receptor		Receptor for extracellular ATP > UTP ar
Protein Kinase D1	The protein e	Serine/threonine-protein kinase that con
AFG3 Like Matrix AAA Peptidase Subunit 1, Pseudogene		
ZNFX1 Antisense RNA 1	This gene represents a snoRNA host gene that produ	
Polycystin 1, Transient Receptor Potential Channel Interacting Pseudogene 1		
Nuclear Receptor 2C2 Associated Protein		May act as a repressor of NR2C2-media
DAZ Interacting Zinc Finger Protein 1 Like		Involved in primary cilium formation (Pub
X-Ray Repair Cross Complem	This gene en	Involved in the homologous recombinatio
X-Ray Repair Cross Complem	This gene en	Involved in the homologous recombinatio
Ubiquitin Conjugating Enzyme	This gene en	Accepts ubiquitin from the E1 complex a
Integrator Complex Subunit 6 Like		
Scavenger Receptor Class F	The protein e	Probable adhesion protein, which media
Tubulin Tyrosine Ligase Like 3		Monoglycylase which modifies alpha- an
Progestin And AdipoQ Receptor Family Member 4		
Serine And Arginine Rich Spli	The protein e	Necessary for the splicing of pre-mRNA.
TBC1 Domain Family Membe	This gene is a	May act as a GTPase-activating protein
Cold Inducible RNA Binding Protein		Cold-inducible mRNA binding protein tha
WEE1 G2 Checkpoint Kinase	This gene en	Acts as a negative regulator of entry into
Tubulin Epsilon And Delta Complex 2		Acts as a positive regulator of ciliary hed
Mevalonate Diphosphate Dec	The enzyme	Catalyzes the ATP dependent decarbox
GINS Complex Subunit 2	The yeast he	The GINS complex plays an essential ro
Small Nuclear Ribonucleoprotein Polypeptide G Pseudogene 2		
WD Repeat Domain 90		Required for efficient primary cilium form
Period Circadian Regulator 2	This gene is a	Transcriptional repressor which forms a
Golgin A8 Family Member A	The Golgi ap	May be involved in maintaining Golgi stru
Lin-7 Homolog B, Crumbs Cell Polarity Com		Plays a role in establishing and maintain
Polypyrimidine Tract Binding I	The protein e	RNA-binding protein which binds to intro

Chromobox 2	This gene en	Component of a Polycomb group (PcG)
ELMO Domain Containing 1		Acts as a GTPase-activating protein (GA
Solute Carrier Family 5 Member 3		Prevents intracellular accumulation of hi
G Protein-Coupled Receptor 1	This gene en	Is a receptor for the SMIM20 derived pe
Deleted In Lymphocytic Leuke	This locus represents a microRNA host gene and also	
KIAA0895 Like		
Chloride Voltage-Gated Chan	This gene en	Chloride transport protein, initially identif
Zinc Finger CCCH-Type Cont	ZC3H12A is a	(Microbial infection) Binds to Japanese e
Ataxin 7 Like 2		
FA Complementation Group A	The Fanconi	DNA repair protein that may operate in a
Centromere Protein T	The centrom	Component of the CENPA-NAC (nucleo
MicroRNA 3916	microRNAs (miRNAs) are short (20-24 nt) non-coding	
Small Nucleolar RNA, H/ACA Box 25		
CDC Like Kinase 2	This gene en	Dual specificity kinase acting on both se
MicroRNA 3682	microRNAs (miRNAs) are short (20-24 nt) non-coding	
Ribosomal Protein L32 Pseudogene 3		
Enhancer Of Zeste 2 Polycom	This gene en	Polycomb group (PcG) protein. Catalytic
Centrosomal Protein 152	This gene en	Necessary for centrosome duplication; tl
MicroRNA 6845	microRNAs (miRNAs) are short (20-24 nt) non-coding	
Inner Mitochondrial Membran	The mitochon	Catalyzes the removal of transit peptides
LIM And Calponin Homology Domains 1		Actin stress fibers-associated protein tha
Hes Family BHLH Transcriptio	This gene en	Does not bind DNA itself but suppresses
Hes Family BHLH Transcriptio	This gene en	Does not bind DNA itself but suppresses
NFKB Inhibitor Zeta	This gene is a	Involved in regulation of NF-kappa-B tra
Lysine Methyltransferase 5C	SUV420H2 a	Histone methyltransferase that specifica
Small Nucleolar RNA, H/ACA Box 4		
Small Nucleolar RNA, H/ACA Box 41		
Integrin Subunit Beta 1 Bindin	This gene en	May play a role during maturation and/or
Ribosomal Protein L37 Pseudogene 23		
Small Nucleolar RNA, C/D Box 96A		
Scm Polycomb Group Protein Like 1		Putative Polycomb group (PcG) protein.
FOXD2 Adjacent Opposite Strand RNA 1		
Adenosine Deaminase TRNA Specific 2		Probably participates in deamination of a
Methionine Adenosyltransfera	The protein e	Catalyzes the formation of S-adenosylm
Zinc Finger Protein 625		May be involved in transcriptional regula
Stearoyl-CoA Desaturase	This gene en	Stearyl-CoA desaturase that utilizes O(2
Small Nucleolar RNA, H/ACA Box 52		

Methylsterol Monooxygenase	Sterol-C4-me	Catalyzes the three-step monooxygenat
Cytochrome C Oxidase Subunit 7A2 Pseudogene 2		
SATB Homeobox 1	This gene en	Crucial silencing factor contributing to th
Small Nucleolar RNA, C/D Box 2		
Cytochrome C Oxidase Subur	Cytochrome	Component of the cytochrome c oxidase
Anti-Mullerian Hormone	This gene en	This glycoprotein, produced by the Serto
Coiled-Coil Domain Containin	The product	Component of the deuterosome, a struc
Small Nucleolar RNA Host Ge	This locus represents a small nucleolar RNA host gen	
Cryptochrome Circadian Regu	This gene en	Transcriptional repressor which forms a
NPPA Antisense RNA 1		
Mitogen-Activated Protein Kin	Activation of	Serine/threonine kinase which acts as a
Translocase Of Inner Mitocho	The mitochon	Mitochondrial intermembrane chaperone
Mitotic Spindle Organizing Protein 1		Required for gamma-tubulin complex re
Zinc Finger Protein 700		May be involved in transcriptional regula
Eukaryotic Translation Initiation Factor 5A2		mRNA-binding protein involved in transla
Period Circadian Regulator 1	This gene is	Transcriptional repressor which forms a
Ubiquitin Associated Protein 1 Like		
EP400 Pseudogene 1		
Caspase 8 Associated Protein	This protein i	Participates in TNF-alpha-induced block
Uracil DNA Glycosylase	This gene en	Excises uracil residues from the DNA wh
Histone Deacetylase 10	The protein e	Polyamine deacetylase (PDAC), which a
Mannosidase Alpha Class 2C Member 1		Cleaves alpha 1,2-, alpha 1,3-, and alph
Nuclear Pore Complex Interacting Protein Family Member A3		
Heterogeneous Nuclear Ribor	This gene be	Acts as a transcriptional regulator. Prom
Prefoldin Subunit 4	This gene en	Binds specifically to cytosolic chaperonin
Colorectal Neoplasia Different	This gene is transcribed into multiple transcript varian	
TATA-Box Binding Protein As	Initiation of tr	Component of the transcription factor SL
Zinc Finger Protein 684		May be involved in transcriptional regula
MDM4 Regulator Of P53	This gene en	Inhibits p53/TP53- and TP73/p73-media
Cell Division Cycle Associated	This gene wa	Participates in MYC-mediated cell transf
Inositol Polyphosphate-5-Pho	The protein e	Phosphatidylinositol (PtdIns) phosphatas
Mitogen-Activated Protein Kin	This gene en	Lymphotoxin beta-activated kinase whic
H2B Histone Pseudogene 2	Histones are basic nuclear proteins responsible for nu	
Meis Homeobox 2	This gene en	Involved in transcriptional regulation. Bin
SLX1B-SULT1A4 Readthroug	This locus represents naturally occurring read-through	
ATP Synthase Membrane Subunit DAPIT		Mitochondrial membrane ATP synthase
Origin Recognition Complex S	The origin re	Component of the origin recognition com
DnaJ Heat Shock Protein Family (Hsp40) M		GTPase which can activate the MEK/ER

RNA, U1 Small Nuclear 103, Pseudogene		
C-Type Lectin Domain Family	Natural killer	(Microbial infection) Acts as a receptor f
MicroRNA 93	microRNAs (miRNAs) are short (20-24 nt) non-coding	
Zinc Finger And SCAN Domai	This gene en	May function as a transcription factor. M
WASP Family Homolog 2, Pseudogene		Acts as a nucleation-promoting factor at
Keratinocyte Associated Protein 3		
NFKB Inhibitor Delta		Regulates the expression of IL-2, IL-6, a
Archaelysin Family Metallopeptidase 2 Pseudogene 1		
ATPase Family AAA Domain	The protein e	May play a role in a mitochondrial netwo
Fibronectin Type III And SPRY Domain Containing 1 Like		
ATP Synthase Membrane Subunit DAPIT Pseudogene 1		
GRB2 Associated Binding Pro	The protein e	Adapter protein that plays a role in intrac
Gamma-Aminobutyric Acid Ty	This gene en	Component of a heterodimeric G-protein
CROCC Pseudogene 2		
Zinc Finger Protein 117	This gene en	May be involved in transcriptional regula
Scleraxis BHLH Transcription Factor		Plays an early essential role in mesoder
IQ Motif Containing C		
Isopentenyl-Diphosphate Delt	IDI1 encodes	Catalyzes the 1,3-allylic rearrangement
SURP And G-Patch Domain C	This gene en	May play a role in mRNA splicing.
TUB Bipartite Transcription Fa	This gene en	Functions in signal transduction from he
Potassium Voltage-Gated Cha	This gene en	Accessory potassium channel protein wh
Divergent Protein Kinase Dom	This gene encodes a member of the FAM69 family of	
CLK4 Associating Serine/Arginine Rich Pro		Probably functions as an alternative splic
Transmembrane Protein 200B		
Cysteine Sulfinic Acid Decarb	This gene en	Catalyzes the decarboxylation of L-aspa
Long Intergenic Non-Protein Coding RNA 472		
Rhotekin 2		May play an important role in lymphopoi
MicroRNA 6753	microRNAs (miRNAs) are short (20-24 nt) non-coding	
Ribosomal Protein L37	Ribosomes, t	Binds to the 23S rRNA.
MIS12 Kinetochore Complex Component		Part of the MIS12 complex which is requ
Phospholipase A2 Group IVA	This gene en	Has primarily calcium-dependent phospl
Ribosomal Protein L21	Ribosomes, t	Component of the large ribosomal subun
Progestin And AdipoQ Receptor Family Me		Plasma membrane progesterone (P4) re
Major Facilitator Superfamily I	The protein e	Sodium-dependent lysophosphatidylcho
Chymotrypsin Like	This gene encodes a serine-type endopeptidase with	
MSS51 Mitochondrial Translational Activator		
ADP-Ribosyltransferase 4 (Do	This gene encodes a protein that contains a mono-AD	
Crystallin Gamma S	Crystallins ar	Crystallins are the dominant structural co

Leucine Rich Repeat Containing 17		Involved in bone homeostasis. Acts as a
MIR22 Host Gene		
TATA-Box Binding Protein As	TAF1D is a n	Component of the transcription factor SL
ATPase Family AAA Domain Containing 5		Involved in DNA damage response. Invo
Protein Kinase Domain Containing, Cytopla		Secreted tyrosine-protein kinase that me
SET Domain Containing 6, Pr	This gene en	Protein-lysine N-methyltransferase. Mon
TLC Domain Containing 5		
Cryptochrome Circadian Regu	This gene en	Transcriptional repressor which forms a
FAST Kinase Domains 3	This gene en	Required for normal mitochondrial respi
Killer Cell Lectin Like Recepto	Natural killer	Plays an inhibitory role on natural killer (
Docking Protein 3		DOK proteins are enzymatically inert ad
Polycystin 1, Transient Recep	This gene en	Component of a heteromeric calcium-pe
Potassium Channel Tetramerization Domai		Substrate-specific adapter of a BCR (BT
Thymosin Beta 15a		Plays an important role in the organizati
RELT TNF Receptor	The protein e	May play a role in apoptosis (PubMed:2
TEN1-CDK3 Readthrough (NI	This locus represents naturally occurring read-through	
GULP PTB Domain Containin	The protein e	May function as an adapter protein. Req
Ribosomal Protein S28	Ribosomes, the organelles that catalyze protein synth	
Zinc Finger MYND-Type Cont	ZMYND19 is	May be involved as a regulatory molecul
Coronin 6		
StAR Related Lipid Transfer D	Cholesterol h	Involved in the intracellular transport of c
ATPase Plasma Membrane C	The protein e	Catalyzes the hydrolysis of ATP couplec
Unc-119 Lipid Binding Chape	This gene is	Involved in synaptic functions in photore
RNA, 5S Ribosomal Pseudogene 383		
Chromosome 1 Open Reading Frame 54		
Growth Arrest Specific 5	This gene produces a spliced long non-coding RNA a	
Rap Guanine Nucleotide Exchange Factor		Probable guanine nucleotide exchange f
ArfGAP With GTPase Domain, Ankyrin Rep		Putative GTPase-activating protein.
Firre Intergenic Repeating RN	This gene produces a long RNA containing a repeatin	
BolA Family Member 3	This gene en	Acts as a mitochondrial iron-sulfur (Fe-S
Small Nucleolar RNA, C/D Box 11		
Family With Sequence Similarity 118 Member A		
Family With Sequence Similarity 76 Member B		
DEAD/H-Box Helicase 11	DEAD box pr	(Microbial infection) Required for bovine
Rogdi Atypical Leucine Zipper	This gene encodes a protein of unknown function. Lo	
Small Nucleolar RNA, H/ACA Box 32		
GABPB1 Antisense RNA 1		
C1D Nuclear Receptor Corep	The protein e	Plays a role in the recruitment of the RN

Ribosomal Biogenesis Factor		Trans-acting factor in ribosome biogenes
RNA Binding Motif Protein 14	This gene en	Isoform 1 may function as a nuclear rece
Small Nucleolar RNA, C/D Box 6		
Aminoadipate Aminotransfera	This gene en	Transaminase with broad substrate spec
Small Nucleolar RNA, H/ACA Box 16A		
Small Nucleolar RNA, C/D Box 5		
EGF Like Domain Multiple 7	This gene en	Regulates vascular tubulogenesis in vivo
ATP Synthase F1 Subunit Ep	This gene en	Mitochondrial membrane ATP synthase
Pre-MRNA Processing Factor 39		Involved in pre-mRNA splicing.
Dachshund Family Transcripti	This gene en	Transcription factor that is involved in re
Coiled-Coil Domain Containing 189		
RAS Guanyl Releasing Protei	The protein e	Guanine nucleotide exchange factor (GE
Small Nucleolar RNA, C/D Box 23		
Small Nucleolar RNA, C/D Bo	SNORD50B is a C/D box-type small nucleolar RNA (s	
Small Nucleolar RNA, C/D Box 62B		
C-X9-C Motif Containing 2		May be involved in cytochrome c oxidase
Small Nucleolar RNA, C/D Box 17		
MicroRNA Let-7d	microRNAs (miRNAs) are short (20-24 nt) non-coding	
Small Nucleolar RNA, C/D Box 45C		
C9orf72-SMCR8 Complex Su	The protein e	[Isoform 1]: Regulates stress granule as
Double PHD Fingers 1		May have an important role in developin
TMSB4X Pseudogene 4		
ATPase Phospholipid Transpo	The P-type a	Catalytic component of a P4-ATPase flip
Zinc Finger Protein 467	The protein e	Transcription factor that promotes adipo
MicroRNA 2116	microRNAs (miRNAs) are short (20-24 nt) non-coding	
Myb/SANT DNA Binding Domain Containing 2		
Centrosomal AT-AC Splicing	This gene encodes a protein thought to contain a coile	
MicroRNA 4321	microRNAs (miRNAs) are short (20-24 nt) non-coding	
Small Nucleolar RNA, C/D Box 22		
Gonadotropin Releasing Horn	This gene en	Stimulates the secretion of gonadotropin
Proline Rich 22		
Small Nucleolar RNA, C/D Box 26		
Small Nucleolar RNA, C/D Box 27		
Small Nucleolar RNA, C/D Box 28		
ArfGAP With GTPase Domain, Ankyrin Re		Putative GTPase-activating protein.
MicroRNA 3176	microRNAs (miRNAs) are short (20-24 nt) non-coding	
Small Nucleolar RNA, C/D Box 30		
Ubiquitin Conjugating Enzyme	The protein e	Accepts ubiquitin from the E1 complex a

Chaperonin Containing TCP1 Subunit 6 Pseudogene 3		
High Mobility Group Nucleoso	The protein e	Binds to nucleosomes, regulating chrom
Zinc Finger Protein 714		May be involved in transcriptional regula
A-Kinase Anchoring Protein 1	This locus er	Splice factor regulating alternative splice
Maternally Expressed 9		
GRB10 Interacting GYF Prote	This gene en	May act cooperatively with GRB10 to reg
Nuclear Pore Complex Interacting Protein Family Member B5		
NBPF Member 14	This gene is a member of the neuroblastoma breakpo	
Stearoyl-CoA Desaturase Pseudogene 1		
Cytochrome P450 Family 1 Su	This gene, C'	A cytochrome P450 monooxygenase inv
Small Nucleolar RNA Host Ge	This gene produces a long RNA that is overexpressec	
COMM Domain Containing 8	The protein e	May modulate activity of cullin-RING E3
G Protein-Coupled Receptor 135		Orphan receptor. Has spontaneous activ
BCL2 Like 2	This gene en	Promotes cell survival. Blocks dexameth
Jagged Canonical Notch Liga	The Notch si	Putative Notch ligand involved in the me
MicroRNA 5587	microRNAs (miRNAs) are short (20-24 nt) non-coding	
Maestro Heat Like Repeat Family Member 6		
Cholinergic Receptor Nicotinic Alpha 10 Su		Ionotropic receptor with a probable role i
DM1 Protein Kinase	The protein e	Non-receptor serine/threonine protein ki
Ubiquitin Specific Peptidase 37		Deubiquitinase that antagonizes the ana
Chaperonin Containing TCP1 Subunit 6 Pseudogene 1		
Cache Domain Containing 1		May regulate voltage-dependent calcium
Small Nucleolar RNA, H/ACA Box 73A		
Engulfment And Cell Motility 3	The protein e	Involved in cytoskeletal rearrangements
Arginine And Serine Rich Protein 1		
LON Peptidase N-Terminal Domain And Ring Finger 1		
Small Nucleolar RNA, C/D Box 32A		
Integrin Subunit Alpha E	Integrins are	Integrin alpha-E/beta-7 is a receptor for
Multiple C2 And Transmembrane Domain C		Calcium sensor which is essential for the
Geminin DNA Replication Inhi	This gene en	Inhibits DNA replication by preventing th
Cyclin L2	The protein e	Involved in pre-mRNA splicing. May indu
Small Nucleolar RNA, C/D Box 35A		
Small Nucleolar RNA, C/D Box 14A		
MicroRNA 21	microRNAs (miRNAs) are short (20-24 nt) non-coding	
Pyridoxal Phosphatase	Pyridoxal 5-p	Protein serine phosphatase that dephos
Small Nucleolar RNA, C/D Box 84		
Golgi Transport 1B		May be involved in fusion of ER-derived
Presequence Translocase As	This gene en	Regulates ATP-dependent protein transl

Small Nucleolar RNA, C/D Box 88A		
Small Nucleolar RNA, C/D Box 88B		
Small Nucleolar RNA Host Gene 3		
Small Nucleolar RNA, C/D Box 86		
Small Nucleolar RNA, H/ACA	Small nucleolar RNAs (snoRNAs) are small noncodin	
Small Nucleolar RNA, H/ACA Box 27		
Discs Large MAGUK Scaffold	This gene en	Postsynaptic scaffolding protein that pla
Gamma-Aminobutyric Acid Ty	The product (	GABA, the major inhibitory neurotransm
Abhydrolase Domain Containi	This gene en	Phospholipase that may play a role in pl
SEC61 Translocon Subunit G	The Sec61 c	Component of SEC61 channel-forming t
Distal-Less Homeobox 1	This gene en	Plays a role as a transcriptional activato
Small Nucleolar RNA, H/ACA Box 61		
Small Nucleolar RNA, H/ACA Box 53		
Small Nucleolar RNA, H/ACA Box 48		
MicroRNA 25	microRNAs (miRNAs) are short (20-24 nt) non-coding	
Small Nucleolar RNA, C/D Box 110		
Small Nucleolar RNA, C/D Box 10		
Small Nucleolar RNA, C/D Box 99		
RecQ Like Helicase 4	The protein e	DNA-dependent ATPase. May modulate
RAD54 Like	The protein e	Involved in DNA repair and mitotic recon
Small Nucleolar RNA, H/ACA Box 51		
Small Nucleolar RNA, H/ACA Box 44		
AarF Domain Containing Kinase 5		The function of this protein is not yet cle
Protein Phosphatase 1 Regulatory Subunit		Acts as a glycogen-targeting subunit for
Succinate Dehydrogenase Complex Flavoprotein Subunit A Pseudogene 1		
Small Nucleolar RNA, H/ACA Box 70		
Interleukin 20 Receptor Subu	IL20RB and I	The IL20RA/IL20RB dimer is a receptor
Small Nucleolar RNA, H/ACA Box 40		
Centromere Protein V		Required for distribution of pericentrome
Small Nucleolar RNA, H/ACA	This gene encodes a member of the family of box AC	
Small VCP Interacting Protein	Endoplasmic reticulum-associated degradation (ERAI	
Small Nucleolar RNA, H/ACA Box 71B		
Hect Domain And RLD 2 Pseudogene 4		
Small Nucleolar RNA, C/D Box 4A		
Small Nucleolar RNA, H/ACA Box 62		
Small Nucleolar RNA, H/ACA Box 24		
Thiosulfate Sulfurtransferase Like Domain (		Thiosulfate:glutathione sulfurtransferase
Small Nucleolar RNA, H/ACA Box 31		

Small Nucleolar RNA, C/D Box 102		
Small Nucleolar RNA, H/ACA Box 73B		
Small Nucleolar RNA, C/D Box 65		
Ubiquitin Conjugating Enzyme E2 S Pseudogene 2		
24-Dehydrocholesterol Reduc	This gene en	Catalyzes the reduction of the delta-24 c
FTX Transcript, XIST Regulat	This gene is located upstream of XIST, within the X-in	
Small Nucleolar RNA, H/ACA Box 18		
Peptidylprolyl Isomerase E Lik	This transcribed pseudogene is related to PPIE (Gene	
Small Nucleolar RNA, H/ACA Box 2A		
Solute Carrier Family 49 Member 3		
EP300 Interacting Inhibitor Of Differentiatio		Acts as a repressor of nuclear receptor-c
Small Nucleolar RNA, H/ACA Box 1		
Poly(ADP-Ribose) Polymerase Family Mem		Mono-ADP-ribosyltransferase that media
Sprouty RTK Signaling Antagonist 1		Inhibits fibroblast growth factor (FGF)-in
Transmembrane Protein 50B		
Chromosome 14 Open Reading Frame 132		
Small Nucleolar RNA, C/D Box 36C		
TEF Transcription Factor, PAI	This gene en	Transcription factor that binds to and tra
Cyclin L1		Involved in pre-mRNA splicing. Function
Small Nucleolar RNA, C/D Box 36A		
Hyperpolarization Activated C	This gene en	Hyperpolarization-activated potassium c
Monocyte To Macrophage Dif	This protein i	Involved in the dynamics of lysosomal m
Small Nucleolar RNA, C/D Box 45B		
Small Nucleolar RNA, C/D Box 45A		
Small Cajal Body-Specific RN	This gene produces a small nuclear RNA that localize	
Golgin A8 Family Member H		
FRAS1 Related Extracellular I	This gene en	Extracellular matrix protein which may pl
Translocase Of Inner Mitochondrial Membr		May participate in the translocation of tra
Small Nucleolar RNA, C/D Bo	Intronic regions of ribosomal protein genes can harbo	
Small Nucleolar RNA, C/D Box 49A		
NPTN Intronic Transcript 1		
Small Nucleolar RNA Host Gene 4		
Protein Disulfide Isomerase F	This gene en	Acts as an intracellular estrogen-binding
Nuclear Receptor Subfamily 2	The protein e	Coup (chicken ovalbumin upstream pror
Protocadherin 7	This gene belongs to the protocadherin gene family, a	
Small Nucleolar RNA, C/D Box 58C		
Small Nucleolar RNA, C/D Box 56		
Small Nucleolar RNA, C/D Box 57		

Protocadherin 9	This gene en	Potential calcium-dependent cell-adhesi
Zinc Finger SWIM-Type Containing 5		
Small Nucleolar RNA, C/D Box 54		
Small Nucleolar RNA, H/ACA Box 57		
Small Nucleolar RNA, C/D Box 58A		
Small Nucleolar RNA, H/ACA Box 64		
Microtubule Associated Protein 11		Microtubule associated protein wich see
Zinc Finger Protein 169		May be involved in transcriptional regula
Methylthioribose-1-Phosphate	This enzyme	Catalyzes the interconversion of methylt
Oligosaccharyltransferase Complex Non-Ca		Subunit of the oligosaccharyl transferase
MicroRNA 589	microRNAs (miRNAs) are short (20-24 nt) non-coding	
MicroRNA 590	microRNAs (miRNAs) are short (20-24 nt) non-coding	
Capping Actin Protein Of Mus	The protein e	F-actin-capping proteins bind in a Ca(2+
Lymphocyte Antigen 6 Family	LY6G5B belongs to a cluster of leukocyte antigen-6 (L	
Unc-51 Like Kinase 3		Serine/threonine protein kinase that acts
Family With Sequence Similarity 117 Member A		
ABRA C-Terminal Like		
Small Nucleolar RNA, C/D Box 12B		
Leukocyte Receptor Cluster Member 8		
Diacylglycerol Kinase Delta	This gene en	Isoform 2 may be involved in cell growth
Collagen Triple Helix Repeat (	This locus er	May act as a negative regulator of collag
Valyl-TRNA Synthetase 2, Mit	This gene encodes a mitochondrial aminoacyl-tRNA s	
SET And MYND Domain Containing 4		
SS18L1 Subunit Of BAF Chrc	This gene en	Transcriptional activator which is require
Ribosomal Protein S15a Pseudogene 24		
Protease Associated Domain Containing 1		Plays a role in the modulation of physica
Family With Sequence Similarity 219 Member B		
Notch 2 N-Terminal Like A		Human-specific protein that promotes ne
Solute Carrier Family 25 Mem	SLC25A33 b	Mitochondrial transporter that imports/ex
Synapse Defective Rho GTPa	The protein e	GTPase activator for the Rho-type GTPa
Small Nucleolar RNA, C/D Box 68		
Eukaryotic Translation Elonga	This gene en	Positive modulator of ATM response to I
Coiled-Coil Domain Containing 130		
Small Nucleolar RNA, H/ACA Box 13		
Membrane Bound O-Acyltrans	This gene be	Acyltransferase which mediates the conv
PAXX Non-Homologous End	The protein e	Involved in non-homologous end joining
Small Nucleolar RNA, H/ACA Box 8		
BTB Domain Containing 19		

Kinesin Family Member 1C	The protein e	Motor required for the retrograde transp
Aquaporin 7 Pseudogene 1		
Transmembrane Protein 100		Plays a role during embryonic arterial en
Zinc Finger GATA Like Protein 1		Transcriptional regulator that plays a key
E2F Transcription Factor 1	The protein e	Transcription activator that binds DNA c
Leukocyte Receptor Tyrosine	The protein e	Receptor with a tyrosine-protein kinase a
Calcium Voltage-Gated Chan	The protein e	Regulatory subunit of L-type calcium cha
Small Nucleolar RNA, C/D Box 15B		
Cell Division Cycle 14A	The protein e	Dual-specificity phosphatase. Required f
Parathyroid Hormone Like Ho	The protein e	Neuroendocrine peptide which is a critica
Coiled-Coil Domain Containing 17		
Centrosomal Protein 85	This gene en	Acts as a negative regulator of NEK2 to
Hydroxyacylglutathione Hydrolase Like		Hydrolase acting on ester bonds.
Inactivation Escape 1	X chromosome inactivation provides dosage compens	
NGFI-A Binding Protein 2	This gene en	Acts as a transcriptional repressor for zi
LIM Domain 7	This gene encodes a protein containing a calponin ho	
Zinc Finger Protein 841		May be involved in transcriptional regula
NDUFA4 Mitochondrial Comp	The protein e	Component of the cytochrome c oxidase
Solute Carrier Family 43 Mem	SLC43A1 be	Sodium-independent, high affinity transp
Cell Growth Regulator With Ring Finger Do		Able to inhibit growth in several cell lines
Zinc Finger Protein 431	This gene en	Sequence-specific DNA binding transcri
Defective In Cullin Neddylation 1 Domain C		Potently stimulates the neddylation of cu
Metastasis Associated Lung A	This gene produces a precursor transcript from which	
MBNL1 Antisense RNA 1		
Pleckstrin Homology And Rho	The protein e	May be a transforming oncogene with ex
RNA, 7SL, Cytoplasmic 81, Pseudogene		
Small Nucleolar RNA, H/ACA Box 6		
Small Nucleolar RNA, H/ACA Box 10		
MT-RNR2 Like 12		Plays a role as a neuroprotective and ar
Leucine Rich Repeat Containing 70		Renders cells highly sensitive to the acti
Kinesin Family Member C2		May play a role in microtubule-depender
Immunoglobulin Lambda Variable 5-52		V region of the variable domain of immu
Tetraspanin 13	The protein encoded by this gene is a member of the	
EPH Receptor A5	This gene be	Receptor tyrosine kinase which binds pr
PAS Domain Containing Serin	This gene en	Serine/threonine-protein kinase involved
Dynein Light Chain LC8-Type	Cytoplasmic	Acts as one of several non-catalytic acce
Synaptotagmin Like 4	This gene en	Modulates exocytosis of dense-core gra
Ribosomal Protein L37a Pseudogene 1		

Mir-100-Let-7a-2-Mir-125b-1	This gene produces long non-coding RNAs that act a	
G Protein-Coupled Receptor	This gene is	Orphan receptor with constitutive G(s) si
Lecithin-Cholesterol Acyltrans	This gene en	Central enzyme in the extracellular meta
Striatin Interacting Protein 2		Plays a role in the regulation of cell mor
Chondroitin Sulfate Synthase	CSS3 is a gl	Has both beta-1,3-glucuronic acid and b
Tonsoku Like, DNA Repair Pr	The protein	Component of the MMS22L-TONSL con
Caprin Family Member 2	The protein	Promotes phosphorylation of the Wnt co
Copine 7	This gene en	Calcium-dependent phospholipid-bindin
Cyclin T2	The protein	(Microbial infection) Promotes transcripti
RAD9 Checkpoint Clamp Con	This gene pr	Component of the 9-1-1 cell-cycle check
Hydroxysteroid 17-Beta Dehydrogenase 7 Pseudogene 2		
Solute Carrier Family 30 Member 3		Involved in accumulation of zinc in syna
Solute Carrier Family 17 Mem	This gene en	Involved in vesicular storage and exocyt
NSF Attachment Protein Beta	This gene en	Required for vesicular transport betwee
Zinc Finger And BTB Domain	This gene is	Acts as a transcriptional repressor (Pub
C-X-C Motif Chemokine Ligan	This antimicr	Has chemotactic activity for neutrophils.
Zinc Finger Protein 141	The protein	May be involved in transcriptional regula
OTU Deubiquitinase 3		Deubiquitinating enzyme that hydrolyze
Small Nucleolar RNA, C/D Bo	Intronic regions of ribosomal protein genes can harbo	
Biogenesis Of Lysosomal Org	This gene en	Component of the BLOC-1 complex, a c
Zinc Finger Protein 335	The protein	Component or associated component of
Lymphocyte Activating 3	Lymphocyte-	[Secreted lymphocyte activation gene 3
Trafficking Kinesin Protein 2		May regulate endosome-to-lysosome tr
Phospholipid Phosphatase 4		Magnesium-independent phospholipid p
CKLF Like MARVEL Transme	This gene belongs to the chemokine-like factor gene	
Solute Carrier Family 26 Mem	This gene be	[Isoform 4]: Apical membrane chloride-b
Small Nucleolar RNA, C/D Box 14B		
Natural Killer Cell Triggering	This gene en	PPIase that catalyzes the cis-trans isom
Testis Expressed 30		
Phosphatidylinositol 4-Kinase Alpha Pseudogene 1		
Adiponectin Receptor 2	The adipone	Receptor for ADIPOQ, an essential horn
Nuclear Pore Complex Interacting Protein Pseudogene 1		
Tudor Domain Containing 10		
Nebulette	This gene en	Binds to actin and plays an important ro
Small Nucleolar RNA, C/D Box 46		
Small Nucleolar RNA, C/D Box 38B		
Small Nucleolar RNA, C/D Box 38A		
Endogenous Bornavirus Like Nucleoprotein		May act as an RNA-binding protein. The

Valosin Containing Protein Lysine Methyltra		Protein-lysine N-methyltransferase that s
Growth Factor Receptor Boun	The product (	Adapter protein which modulates couplir
Ephrin B2	This gene en	(Microbial infection) Acts as a receptor fo
Coiled-Coil Domain Containing 144A		
RPL24 Pseudogene 2		
Uveal Autoantigen With Coile	This gene en	Modulates isoactin dynamics to regulate
ArfGAP With Coiled-Coil, Ankyrin Repeat A		GTPase-activating protein for the ADP ri
MicroRNA 4292	microRNAs (miRNAs) are short (20-24 nt) non-coding	
Transmembrane Protein 37		Thought to stabilize the calcium channel
Ribosomal Protein S10 Pseudogene 3		
CREB/ATF BZIP Transcription Factor		Strongly activates transcription when bo
Neuropeptide FF-Amide Pepti	This gene en	Morphine modulating peptides. Have wic
Zinc Finger Protein 333		May be involved in transcriptional regula
Arginine And Glutamate Rich 1		Required for the estrogen-dependent ex
Adhesion G Protein-Coupled	This gene en	Calcium-independent receptor of low aff
Keratin 10	This gene en	(Microbial infection) Acts as a mediator c
CD58 Molecule	This gene en	Ligand of the T-lymphocyte CD2 glycopr
ABR Activator Of RhoGEF An	This gene en	Protein with a unique structure having tw
Acetylcholinesterase (Cartwri	Acetylcholine	Terminates signal transduction at the ne
Actinin Alpha 1	Alpha actinin	F-actin cross-linking protein which is tho
Adenosine Deaminase	This gene en	Catalyzes the hydrolytic deamination of a
Adaptor Related Protein Com	The protein e	Component of the adaptor protein comp
Alanyl Aminopeptidase, Meml	Aminopeptida	(Microbial infection) Acts as a receptor fo
Annexin A3	This gene en	Inhibitor of phospholipase A2, also poss
Amyloid Beta Precursor Prote	The protein e	May modulate the internalization of amy
Shroom Family Member 2	This gene re	May be involved in endothelial cell morp
Arginase 2	Arginase cat	May play a role in the regulation of extra
Rho GTPase Activating Prote	Rho GTPase	GTPase-activating protein for Rho family
Arylsulfatase D	The protein encoded by this gene is a member of the	
Arylsulfatase L	Arylsulfatase	May be essential for the correct compos
BTB Domain And CNC Homo	This gene en	Transcriptional regulator that acts as rep
BCL2 Related Protein A1	This gene en	Retards apoptosis induced by IL-3 depri
PR/SET Domain 1	This gene en	Transcription factor that mediates a tran
BMX Non-Receptor Tyrosine	This gene en	Non-receptor tyrosine kinase that plays c
BCL2 Family Apoptosis Regul	The protein e	[Isoform 1]: Apoptosis regulator that func
ZFP36 Ring Finger Protein Lil	This gene is a	Zinc-finger RNA-binding protein that des
Bone Marrow Stromal Cell An	Bone marrow	Synthesizes the second messengers cyc
Caldesmon 1	This gene en	Actin- and myosin-binding protein implica

Capping Actin Protein, Gelsol	This gene en	Calcium-sensitive protein which reversib
Calpain 2	The calpains	Calcium-regulated non-lysosomal thiol-p
Caspase 3	The protein e	Involved in the activation cascade of cas
Caveolin 1	The scaffoldi	May act as a scaffolding protein within c
Calicin	The protein e	Possible morphogenetic cytoskeletal ele
CD34 Molecule	The protein e	Possible adhesion molecule with a role i
Scavenger Receptor Class B	The protein e	(Microbial infection) Acts as a receptor f
Ectonucleoside Triphosphate	The protein e	In the nervous system, could hydrolyze
CD44 Molecule (Indian Blood	The protein e	Cell-surface receptor that plays a role in
CD74 Molecule	The protein e	[Class-II-associated invariant chain pept
LPS Responsive Beige-Like A	The protein e	May be involved in coupling signal trans
Cadherin 2	This gene en	Calcium-dependent cell adhesion protei
Cadherin 5	This gene en	Cadherins are calcium-dependent cell a
Cadherin 11	This gene en	Cadherins are calcium-dependent cell a
Cadherin 13	This gene en	Cadherins are calcium-dependent cell a
Cholinergic Receptor Nicotinic	The muscle a	After binding acetylcholine, the AChR re
Tripeptidyl Peptidase 1	This gene en	Lysosomal serine protease with tripeptid
Collagen Type IV Alpha 5 Cha	This gene en	Type IV collagen is the major structural
Collagen Type IV Alpha 6 Cha	This gene en	Type IV collagen is the major structural
Collagen Type V Alpha 2 Cha	This gene en	Type V collagen is a member of group I
Collagen Type XII Alpha 1 Ch	This gene en	Type XII collagen interacts with type I co
Activating Transcription Facto	The protein e	Transcriptional factor that acts in the unf
Colony Stimulating Factor 1	The protein e	Cytokine that plays an essential role in t
Cysteine And Glycine Rich Pr	This gene en	Could play a role in neuronal developme
Cathepsin B	This gene en	Thiol protease which is believed to partic
Cathepsin K	The protein e	Thiol protease involved in osteoclastic b
Cathepsin O	The protein e	Proteolytic enzyme possibly involved in
CXADR Ig-Like Cell Adhesion	The protein e	(Microbial infection) Acts as a receptor f
CYLD Lysine 63 Deubiquitina	This gene is	Deubiquitinase that specifically cleaves
Death Associated Protein Kin	Death-associ	Serine/threonine kinase which is involve
DEAD-Box Helicase 1	DEAD box pr	(Microbial infection) Required for Corona
Dual Specificity Phosphatase	The protein e	Regulates mitogenic signal transduction
Dual Specificity Phosphatase	The protein	Dual specificity protein phosphatase; act
Dual Specificity Phosphatase	The protein e	Has phosphatase activity with synthetic
Endothelin Converting Enzym	The protein e	Converts big endothelin-1 to endothelin-
Sphingosine-1-Phosphate Re	This gene en	Receptor for the lysosphingolipid sphing
Dematin Actin Binding Protein	The protein e	Membrane-cytoskeleton-associated prot
EPH Receptor B2	This gene en	Receptor tyrosine kinase which binds pr

ETS Proto-Oncogene 2, Trans	This gene en	Transcription factor activating transcripti
ETS Variant Transcription Fac	This gene en	Transcriptional activator that binds to DN
Exostosin Like Glycosyltransf	This gene en	Glycosyltransferase which regulates the
F2R Like Trypsin Receptor 1	This gene en	Receptor for trypsin and trypsin-like enz
Fibroblast Activation Protein A	The protein e	Cell surface glycoprotein serine proteas
FAT Atypical Cadherin 1	This gene is	Plays an essential role for cellular polari
Fructose-Bisphosphatase 1	Fructose-1,6-	Catalyzes the hydrolysis of fructose 1,6-
FKBP Prolyl Isomerase 1A	The protein e	Keeps in an inactive conformation TGFB
Filamin B	This gene en	Connects cell membrane constituents to
Filamin C	This gene en	Muscle-specific filamin, which plays a ce
Fms Related Receptor Tyrosi	This gene en	Tyrosine-protein kinase that acts as a ce
FYN Proto-Oncogene, Src Fa	This gene is	Non-receptor tyrosine-protein kinase tha
Guanidinoacetate N-Methyltra	The protein e	Converts guanidinoacetate to creatine, u
Growth Arrest Specific 6	This gene en	(Microbial infection) Can bridge virus en
Guanylate Binding Protein 1	Guanylate bi	Hydrolyzes GTP to GMP in 2 consecutiv
Guanylate Binding Protein 2	This gene be	Hydrolyzes GTP to GMP in 2 consecutiv
GTP Cyclohydrolase I Feedba	GTP cyclohy	Mediates tetrahydrobiopterin inhibition o
Gamma-Glutamyltransferase	The enzyme	Cleaves the gamma-glutamyl bond of ex
Glycine Receptor Beta	This gene en	Glycine receptors are ligand-gated chlor
GM2 Ganglioside Activator	This gene en	The large binding pocket can accommoc
G Protein Subunit Alpha 12		Guanine nucleotide-binding proteins (G
G Protein Subunit Alpha I2	The protein e	[Isoform sGi2]: Regulates the cell surfac
G Protein-Coupled Receptor I	This gene en	Serine/threonine kinase that phosphoryl
G Protein Pathway Suppress	This gene en	(Microbial infection) Required for efficien
General Transcription Factor IIi Pseudogene 1		
Glycophorin C (Gerbich Blood	Glycophorin (	This protein is a minor sialoglycoprotein
Serpin Family D Member 1	This gene be	Peptides at the N-terminal of HC-II have
H2.0 Like Homeobox		Transcription factor required for TBX21/
High Mobility Group AT-Hook	This gene en	HMG-I/Y bind preferentially to the minor
Forkhead Box A1	This gene en	Transcription factor that is involved in en
Histamine N-Methyltransferas	In mammals,	Inactivates histamine by N-methylation.
DnaJ Heat Shock Protein Fan	This gene en	Co-chaperone for HSPA8/Hsc70 (PubM
Tenascin C	This gene en	Extracellular matrix protein implicated in
Immunoglobulin Binding Prote	The prolifera	Associated to surface IgM-receptor; may
Interleukin 15	The protein e	Cytokine that stimulates the proliferation
Integrin Subunit Alpha 6	The gene en	Integrin alpha-6/beta-1 (ITGA6:ITGB1) is
Interferon Regulatory Factor 2	IRF2 encode	Specifically binds to the upstream regula
Interferon Stimulated Exonuclease Gene 2(		Interferon-induced antiviral exoribonucle

JunD Proto-Oncogene, AP-1	The protein e	Transcription factor binding AP-1 sites.
Potassium Inwardly Rectifying	This gene en	Inward rectifying potassium channel that
Kinesin Family Member 3C		Microtubule-based anterograde transloc
Laminin Subunit Alpha 3	The protein e	Binding to cells via a high affinity recepto
Laminin Subunit Alpha 4	Laminins, a f	Binding to cells via a high affinity recepto
Laminin Subunit Gamma 1	Laminins, a f	Binding to cells via a high affinity recepto
Galectin 9	The galectins	[Isoform 2]: Acts as an eosinophil chemo
Fatty Acid Desaturase 3	The protein e	Acts as a methyl-end fatty acyl coenzym
Von Willebrand Factor A Domain Containin		May play a role in tumorigenesis as a tu
Lysyl Oxidase	This gene en	Responsible for the post-translational ox
Lysyl Oxidase Like 1	This gene en	Active on elastin and collagen substrates
Lysyl Oxidase Like 2	This gene en	Mediates the post-translational oxidative
LIM Domain Containing Prefe	This gene en	May play a structural role at sites of cell
LDL Receptor Related Protein	This gene en	Molecular chaperone for LDL receptor-re
Lymphotoxin Beta	Lymphotoxin	Cytokine that binds to LTBR/TNFRSF3.
Lactotransferrin	This gene is	[Isoform DeltaLf]: transcription factor wit
SMAD Family Member 1	The protein e	Transcriptional modulator activated by B
Melanoma Cell Adhesion Molecule		Plays a role in cell adhesion, and in coh
Myocyte Enhancer Factor 2D	This gene is	Transcriptional activator which binds spe
MET Proto-Oncogene, Recep	This gene en	(Microbial infection) Acts as a receptor f
Microfibril Associated Protein	Microfibrillar-	Component of the elastin-associated mi
MLLT3 Super Elongation Complex Subunit		Chromatin reader component of the sup
Matrix Metallopeptidase 1	This gene en	Cleaves collagens of types I, II, and III a
Matrix Metallopeptidase 10	This gene en	Can degrade fibronectin, gelatins of type
Matrix Metallopeptidase 11	Proteins of th	May play an important role in the progre
Matrix Metallopeptidase 15	This gene en	Endopeptidase that degrades various co
Matrix Metallopeptidase 19	This gene en	Endopeptidase that degrades various co
CD200 Molecule	This gene en	Costimulates T-cell proliferation. May re
Mannose Receptor C-Type 1	The recogniti	(Microbial infection) Acts as a receptor f
Methionine Sulfoxide Reducta	This gene en	Has an important function as a repair en
MAX Interactor 1, Dimerizatio	Expression o	Transcriptional repressor. MXI1 binds wi
NFKB Inhibitor Epsilon	The protein e	Inhibits NF-kappa-B by complexing with
Neuronal PAS Domain Protei	The protein e	Transcriptional activator which forms a c
Neurogranin	Neurogranin	Acts as a 'third messenger' substrate of
5'-Nucleotidase Ecto	The protein e	Hydrolyzes extracellular nucleotides into
Receptor Tyrosine Kinase Lik	This gene en	Has very low kinase activity in vitro and i
G Protein-Coupled Receptor	This gene en	Receptor for tyrosine, L-DOPA and dopa
2'-5'-Oligoadenylate Syntheta	This gene is	Interferon-induced, dsRNA-activated an

P21 (RAC1) Activated Kinase	The protein e	Serine/threonine protein kinase that play
Peptidylglycine Alpha-Amidati	This gene en	Bifunctional enzyme that catalyzes the p
Phosphodiesterase 2A		[Isoform PDE2A2]: Regulates mitochonc
Pyruvate Dehydrogenase Kin	The pyruvate	Inhibits pyruvate dehydrogenase activity
Platelet And Endothelial Cell	The protein e	[Isoform Delta15]: Does not protect agai
ATPase Phospholipid Transp	This gene en	Catalytic component of a P4-ATPase flip
6-Phosphofructo-2-Kinase/Fru	The protein e	Synthesis and degradation of fructose 2
Phosphate Regulating Endop	The protein e	Peptidase that cleaves SIBLING (small i
Procollagen-Lysine,2-Oxoglut	The protein e	Forms hydroxylysine residues in -Xaa-Ly
Plastin 3	Plastins are	Actin-bundling protein found in intestinal
Phospholipid Transfer Protein	The protein e	Mediates the transfer of phospholipids a
Plexin B1		Receptor for SEMA4D (PubMed:198435
Protein Phosphatase 2 Regul	This gene en	The B regulatory subunit might modulate
Proteasome 20S Subunit Beta	The proteaso	The proteasome is a multicatalytic prote
Quiescin Sulfhydryl Oxidase 1	This gene en	Catalyzes the oxidation of sulfhydryl gro
Protein Tyrosine Phosphatase	The protein e	Isoform 1 and isoform 2 act as a negativ
Protein Tyrosine Phosphatase	The protein e	Possible cell adhesion receptor. It posse
Protein Tyrosine Phosphatase	The protein e	Involved in cell-cell adhesion through ho
Protein Tyrosine Phosphatase	The protein e	Cell surface receptor that binds to glyco
Rac Family Small GTPase 2	This gene en	Plasma membrane-associated small GT
RAS Like Proto-Oncogene A	The product	Multifunctional GTPase involved in a var
Ras Protein Specific Guanine	RAS GTPase	Functions as a calcium-regulated nucleo
Sortilin 1	This gene en	Functions as a sorting receptor in the Go
S100 Calcium Binding Protein	The protein e	Binds both calcium and zinc. May be inv
C-C Motif Chemokine Ligand	This gene is	Acts as a ligand for C-C chemokine rece
C-C Motif Chemokine Ligand	This gene en	Chemotactic factor that attracts monocy
C-C Motif Chemokine Ligand	This antimicr	Acts as a ligand for C-C chemokine rece
C-X-C Motif Chemokine Ligan	The protein e	Chemotactic for neutrophil granulocytes
C-X3-C Motif Chemokine Liga	This gene be	Acts as a ligand for both CX3CR1 and ir
Selectin P Ligand	This gene en	(Microbial infection) Acts as a receptor f
Secreted Frizzled Related Pro	This gene en	Soluble frizzled-related proteins (sFRPS
ST6 Beta-Galactoside Alpha-2	This gene en	Transfers sialic acid from CMP-sialic aci
Fascin Actin-Bundling Protein	This gene en	Actin-binding protein that contains 2 maj
Superoxide Dismutase 2	This gene is	Destroys superoxide anion radicals whic
SRY-Box Transcription Factor	Members of t	Transcription factor that binds to DNA at
SPARC (Osteonectin), Cwcv	This gene en	May play a role in cell-cell and cell-matri
Spectrin Beta, Non-Erythrocyt	Spectrins are	Probably plays an important role in neur
SRC Proto-Oncogene, Non-R	This gene is	Non-receptor protein tyrosine kinase wh

Supervillin	This gene en	[Isoform 1]: Forms a high-affinity link bet
TAP Binding Protein	This gene en	Involved in the association of MHC class
TIMP Metallopeptidase Inhibit	This gene is	Complexes with metalloproteinases (suc
TLE Family Member 4, Transcriptional Core		Transcriptional corepressor that binds to
G Protein-Coupled Receptor 137B		Acts also as a negative regulator of oste
Claudin 5	This gene en	Plays a major role in tight junction-speci
TNF Alpha Induced Protein 1	This gene wa	Substrate-specific adapter of a BCR (BT
TNF Receptor Associated Fa	The protein e	Adapter molecule that regulates the acti
TNF Receptor Associated Fa	The protein e	Regulates pathways leading to the activa
TNF Receptor Associated Fa	The scaffold	Adapter protein and signal transducer th
Ubiquitin Like Modifier Activat	The modifica	Activates ubiquitin by first adenylating wi
Ubiquitin Conjugating Enzyme	The modifica	Accepts ubiquitin from the E1 complex a
UDP-Glucose Ceramide Gluc	This gene en	Catalyzes at the cytosolic surface of the
Utrophin	This gene sh	May play a role in anchoring the cytoske
Vimentin	This gene en	Involved with LARP6 in the stabilization
Very Low Density Lipoprotein	The low dens	Binds VLDL and transports it into cells b
CAP-Gly Domain Containing I	The protein e	Seems to link microtubules to dendritic l
Wnt Family Member 9A	The WNT ge	Ligand for members of the frizzled famil
Xg Glycoprotein (Xg Blood Gr	This gene encodes the XG blood group antigen, and i	
Zinc Finger AN1-Type Containing 5		Involved in protein degradation via the u
Dynein Axonemal Light Interm	This gene is	May play a dynamic role in flagellar moti
Lysosomal Protein Transmem	This gene en	May have a special functional role durin
Peroxidasin	This gene en	Displays low peroxidase activity and is li
Nuclear Receptor Interacting I	Nuclear rece	Modulates transcriptional activation by s
CDC42 Binding Protein Kinas	The protein e	Serine/threonine-protein kinase which is
Semaphorin 7A (John Milton I	This gene en	Plays an important role in integrin-media
Serine Protease 12	This gene en	Plays a role in neuronal plasticity and th
Contactin Associated Protein	The gene prc	Required, with CNTNAP2, for radial and
Nipsnap Homolog 1	This gene encodes a member of the NipSnap family c	
Eukaryotic Translation Initiation Factor 3 Su		Component of the eukaryotic translation
ATP Binding Cassette Subfan	The protein e	May act as an inducible transporter in th
ADAM Metallopeptidase Dom	This gene en	Participates in the proteolytic processing
RNA Guanine-7 Methyltransferase		Catalytic subunit of the mRNA-capping r
TNF Superfamily Member 10	The protein e	Cytokine that binds to TNFRSF10A/TRA
TNF Receptor Superfamily Me	This gene be	Decoy receptor that can neutralize the c
TNF Receptor Superfamily Me	The protein e	Receptor for the cytotoxic ligand TNFSF
Fibroblast Growth Factor 16	This gene en	Plays an important role in the regulation
IQ Motif Containing GTPase /	This gene en	Plays a crucial role in regulating the dyn

ArfGAP With SH3 Domain, Ar	This gene en	Activates the small GTPases ARF1, ARI
ST3 Beta-Galactoside Alpha-2	Ganglioside (	Catalyzes the formation of ganglioside G
Sequestosome 1	This gene en	Autophagy receptor required for selectiv
Eukaryotic Translation Initiatic	This gene en	Catalyzes the exchange of eukaryotic in
Cyclin A1	The protein e	May be involved in the control of the cell
RAB29, Member RAS Oncogene Family		The small GTPases Rab are key regulat
Kalirin RhoGEF Kinase	Huntington's	Promotes the exchange of GDP by GTP
Proline-Serine-Threonine Pho	This gene en	Involved in regulation of the actin cytosk
Solute Carrier Family 16 Member 4		Proton-linked monocarboxylate transpor
Synaptogyrin 3	This gene en	May play a role in regulated exocytosis.
Dual Specificity Tyrosine Pho	This gene en	Dual-specificity kinase which possesses
Membrane Associated Guany	The protein e	May play a role as scaffolding protein at
DLG Associated Protein 1		Part of the postsynaptic scaffold in neur
Dehydrogenase/Reductase 3	Short-chain c	Catalyzes the reduction of all-trans-retin
Neuronal Regeneration Related Protein		May have roles in neural function. Ectop
Transforming Growth Factor E	This gene en	Plays a role in the TGF-beta/activin sign
N-Myristoyltransferase 2	This gene en	Adds a myristoyl group to the N-terminal
Leupaxin	The product (	Transcriptional coactivator for androgen
Tight Junction Protein 2	This gene en	Plays a role in tight junctions and adhere
Chromodomain Y Like	Chromodoma	[Isoform 1]: Not able to recognize and bi
Carbohydrate Sulfotransferas	This locus er	Sulfotransferase that utilizes 3'-phospho
Mitogen-Activated Protein Kin	The protein e	Serine/threonine kinase that may play a
Homer Scaffold Protein 3	This gene en	Postsynaptic density scaffolding protein.
Mitogen-Activated Protein Kin	This gene en	The JNK-interacting protein (JIP) group
ADAM Metallopeptidase With	This gene en	Cleaves aggrecan, a cartilage proteoglyc
CAMP Responsive Element E	The product (	Binds to the cAMP response element an
Synuclein Alpha Interacting P	This gene en	Isoform 2 inhibits the ubiquitin ligase act
SH3 And PX Domains 2A		Adapter protein involved in invadopodia
GRIP And Coiled-Coil Domair	The protein e	Golgin which probably tethers transport
Dedicator Of Cytokinesis 4	This gene is	[Isoform 2]: Has a higher guanine nucle
Leucine Zipper Tumor Suppressor Family M		May be involved in promoting the matura
MTSS I-BAR Domain Containing 1		May be related to cancer progression or
Secernin 1	This gene lik	Regulates exocytosis in mast cells. Incre
RAB GTPase Activating Protein 1 Like		GTP-hydrolysis activating protein (GAP)
TRAF Family Member Associa	The TRAF (t	Adapter protein involved in I-kappa-B-kir
BCL2 Like 11	The protein e	Induces apoptosis and anoikis. Isoform I
SH2 Domain Containing 3C	This gene en	[Isoform 1]: Important regulator of chem
Protein Tyrosine Phosphatase	The protein e	Tyrosine-protein phosphatase which dep

ATPase Phospholipid Transporting 9A (Putative)		
Transducer Of ERBB2, 1	This gene en	Anti-proliferative protein; the function is r
WASP Family Member 2	This gene en	Downstream effector molecule involved
Toll Like Receptor 6	The protein e	Participates in the innate immune respor
Semaphorin 3A	This gene is a	Involved in the development of the olfac
Butyrophilin Subfamily 3 Mem	The butyroph	Plays a role in T-cell responses in the ac
Brain Abundant Membrane At	This gene encodes a membrane bound protein with s	
Cyclase Associated Actin Cyt	This gene wa	May have a regulatory bifunctional role.
Vesicle Amine Transport 1	Synaptic vesi	Possesses ATPase activity (By similarity
Semaphorin 6B	This gene en	May play a role in both peripheral and ce
Dihydropyrimidinase Like 4		Necessary for signaling by class 3 sema
CDC42 Effector Protein 3	This gene en	Probably involved in the organization of
Tripartite Motif Containing 16	The protein e	E3 ubiquitin ligase that plays an essentia
Podoplanin	This gene en	Mediates effects on cell migration and a
KH RNA Binding Domain Con	This gene en	Isoform 3, which is expressed in growth-
GRB2 Related Adaptor Protei	This gene en	Couples signals from receptor and cytop
Lysine Demethylase 5B	This gene en	Histone demethylase that demethylates
SHH Signaling And Ciliogenes	This gene en	Plays a role in the establishment of cell p
Proline Rich Nuclear Receptor Coactivator		Nuclear receptor coactivator. May play a
Acyl-CoA Thioesterase 2	This gene en	Acyl-CoA thioesterases are a group of e
Butyrophilin Subfamily 3 Mem	The butyroph	Plays a role in T-cell activation and in th
Calpain 11	Calpains con	Calcium-regulated non-lysosomal thiol-p
ADAM Metallopeptidase With	The protein e	Metalloprotease that may play a role in t
A-Kinase Anchoring Protein 2		Binds to regulatory subunit (RII) of prote
Ring Finger Protein 24	This gene en	May play a role in TRPCs intracellular tr
Nidogen 2	This gene en	Cell adhesion glycoprotein which is wide
RAS P21 Protein Activator 3	This gene en	Inhibitory regulator of the Ras-cyclic AM
Pleckstrin Homology Like Dor	This gene en	Seems to be involved in regulation of ap
Ring Finger Protein 44	The protein encoded by this gene contains a RING fin	
DLG Associated Protein 4	The product o	May play a role in the molecular organiz
SEC31 Homolog A, COPII Co	The protein e	Component of the coat protein complex
MLX Interacting Protein	This gene en	Binds DNA as a heterodimer with MLX a
Trafficking Kinesin Protein 1		Involved in the regulation of endosome-t
CD93 Molecule	The protein e	Receptor (or element of a larger recepto
Transmembrane And Coiled-Coil Domain F		Endoplasmic reticulum membrane prote
Lysine Demethylase 4B		Histone demethylase that specifically de
Sterile Alpha Motif Domain Co	Sterile alpha	Acts as a translational repressor of SRE
Paternally Expressed 10	This is a pate	Prevents apoptosis in hepatocellular car

Rho GTPase Activating Protei	Interaction of	GTPase-activating protein for RHOA and
TGF-Beta Activated Kinase 1	The protein e	Adapter linking MAP3K7/TAK1 and TRA
TBC1 Domain Family Member 9		May act as a GTPase-activating protein
Lipin 1	This gene en	Plays important roles in controlling the m
Ral Guanine Nucleotide Dissociation Stimu		Probable guanine nucleotide exchange f
F-Box And Leucine Rich Repe	This gene en	Substrate recognition component of a S
Transmembrane 131 Like		[Isoform 1]: Membrane-associated form
Zinc Finger CCCH-Type Cont	This gene en	May be a specific regulator of miRNA bi
IQ Motif Containing E		Component of the EvC complex that pos
SMG6 Nonsense Mediated M	This gene en	Component of the telomerase ribonucle
G Protein-Coupled Receptor	The protein e	Key negative regulator of Shh signaling,
SEC14 Like Lipid Binding 2	This gene en	Carrier protein. Binds to some hydropho
RNA Binding Fox-1 Homolog	This gene is	RNA-binding protein that regulates alter
Single Stranded DNA Binding Protein 3		May be involved in transcription regulati
Plexin B2	Members of t	Cell surface receptor for SEMA4C, SEM
Fibronectin Leucine Rich Trar	This gene en	Functions in cell-cell adhesion, cell migr
Pannexin 1	The protein e	Structural component of the gap junctior
KIAA1549 Like		
6-Phosphogluconolactonase		Hydrolysis of 6-phosphogluconolactone
Glutaminyl-Peptide Cyclotrans	This gene en	Responsible for the biosynthesis of pyro
Beta-Secretase 2	This gene en	Responsible for the proteolytic processir
ABI Family Member 3 Binding Protein		
Transmembrane Protein 158	Constitutive a	Receptor for brain injury-derived neurotr
SAM And HD Domain Contair	This gene ma	Protein that acts both as a host restrictic
Adhesion G Protein-Coupled Receptor A2		Endothelial receptor which functions tog
C2 Calcium Dependent Domain Containing 2		
Sushi, Nidogen And EGF Like Domains 1		
Leucine Rich Repeats And Immunoglobulin		Acts as a feedback negative regulator of
Transmembrane Protein 98	This gene en	Functions as a negative regulator of MY
Oxysterol Binding Protein Like	This gene en	Phosphoinositide-binding protein which a
WD Repeat Domain, Phosph	WD40 repea	[Isoform 4]: Recruits the ATG12-ATG5-A
Phosphatidylinositol Transfer	This gene en	Phosphatidylinositol transfer proteins me
Regulator Of G Protein Signal	This gene en	Regulates G protein-coupled receptor si
Myeloma Overexpressed		
HMG-Box Transcription Factor 1		Transcriptional repressor that binds to th
Syndecan Binding Protein 2	The protein e	Binds phosphatidylinositol 4,5-bisphosph
Dickkopf WNT Signaling Path	This gene en	Antagonizes canonical Wnt signaling by
G Protein-Coupled Receptor	This gene wa	Orphan receptor.

TNF Receptor Superfamily Me	This gene en	Promotes apoptosis, possibly via a pathw
Regulator Of Cell Cycle	This gene is	Modulates the activity of cell cycle-speci
PYD And CARD Domain Con	This gene en	Functions as key mediator in apoptosis a
Dermatan Sulfate Epimerase	The protein e	Converts D-glucuronic acid to L-iduronic
NOP53 Ribosome Biogenesis Factor		Nucleolar protein which is involved in the
EH Domain Containing 4		ATP- and membrane-binding protein tha
NADPH Oxidase 4	This gene en	[Isoform 4]: Involved in redox signaling ir
Podocalyxin Like 2	This gene is	Acts as a ligand for vascular selectins. M
ArfGAP With SH3 Domain, Ar	This gene en	Possesses phosphatidylinositol 4,5-bisp
Neurotrimin	This gene en	Neural cell adhesion molecule.
SH3 And Multiple Ankyrin Re	This gene en	Seems to be an adapter protein in the p
HD Domain Containing 2		Catalyzes the dephosphorylation of the
Pleckstrin Homology Domain Containing O		Plays a role in the regulation of the actin
Zinc Finger Protein 219	This gene is	Transcriptional regulator (PubMed:1462
Receptor Accessory Protein 2	This gene en	Required for endoplasmic reticulum (ER
Androgen Induced 1		Hydrolyzes bioactive fatty-acid esters of
Transient Receptor Potential	This gene en	Calcium-permeable, non-selective cation
Sorting Nexin 9	This gene en	Involved in endocytosis and intracellular
RAB6B, Member RAS Oncogene Family		Seems to have a role in retrograde mem
Dehydrogenase/Reductase 7	This gene encodes a member of the short-chain dehy	
Acyl-CoA Synthetase Long Ch	The protein e	Catalyzes the conversion of long-chain f
G Protein-Coupled Receptor	This gene en	Unknown. This retinoic acid-inducible G-
Polypeptide N-Acetylgalactosa	This gene en	Glycopeptide transferase involved in O-l
Interleukin 17D	The protein e	Induces expression of IL6, CXCL8/IL8, a
Zinc Finger FYVE-Type Conta	The FYVE do	Plays a role in the formation of lipid drop
Solute Carrier Family 37 Mem	The protein e	Inorganic phosphate and glucose-6-pho
Gamma-Secretase Activating	Accumulation	Regulator of gamma-secretase activity,
Sialic Acid Acetylesterase	This gene en	Catalyzes the removal of O-acetyl ester
Ras And Rab Interactor 2	The RAB5 pr	Ras effector protein. May function as an
Ras Homolog Family Member F, Filopodia		Plasma membrane-associated small GT
DNA Damage Inducible Transcript 4		Regulates cell growth, proliferation and
Egl-9 Family Hypoxia Inducibl	The protein e	Cellular oxygen sensor that catalyzes, u
Transmembrane Protein 132A	This gene en	May play a role in embryonic and postna
Zinc Finger And SCAN Domai	The protein e	May be involved in transcriptional regula
Ecto-NOX Disulfide-Thiol Exc	The protein e	Probably acts as a terminal oxidase of p
Ral GEF With PH Domain And SH3 Binding		Guanine nucleotide exchange factor for
Retroelement Silencing Factor 1		Plays a role in the regulation of imprinte
Leucine Rich Repeat Containing 20		

FGGY Carbohydrate Kinase D	This gene encodes a protein that phosphorylates carb	
La Ribonucleoprotein 6, Translational Regu		Regulates the coordinated translation of
Transmembrane Protein 63B		Acts as an osmosensitive calcium-perme
DEAD-Box Helicase 43	The protein encoded by this gene is an ATP-depende	
RNA Binding Motif Protein 38		(Microbial infection) Essential factor for t
Dedicator Of Cytokinesis 10	This gene en	Guanine nucleotide-exchange factor (GE
TBC1 Domain Family Member 22B		May act as a GTPase-activating protein
Breast Carcinoma Amplified Sequence 4		
Rho Guanine Nucleotide Exch	This gene en	May act as a guanine nucleotide exchan
NECAP Endocytosis Associat	This gene lik	Involved in endocytosis.
Fatty Acyl-CoA Reductase 2	This gene be	Catalyzes the reduction of saturated but
Protocadherin Gamma Subfa	This gene is a	Potential calcium-dependent cell-adhesi
Protocadherin Gamma Subfa	This gene is a	Potential calcium-dependent cell-adhesi
Gasdermin C		The N-terminal moiety promotes pyropto
KIAA1217		Required for normal development of inte
Transcription Elongation Factor A Like 7		Plays a role in the negative regulation of
Latexin	This gene en	Hardly reversible, non-competitive, and p
CEA Cell Adhesion Molecule 19		
Reticulon 4	This gene be	[Isoform A]: Developmental neurite grow
Endoplasmic Reticulum-Golgi	This gene en	Possible role in transport between endop
Transmembrane And Coiled-Coil Domain Family 3		
GRAM Domain Containing 1B		Cholesterol transporter that mediates no
NYN Domain And Retroviral Integrase Containing		
Alpha Kinase 3		Involved in cardiomyocyte differentiation
Membrane Associated Ring-C	MARCH4 is a	E3 ubiquitin-protein ligase that may med
WD Repeat Domain 48	The protein e	Regulator of deubiquitinating complexes
Junctional Cadherin 5 Associa	This gene encodes an endothelial cell-to-cell junction	
KIAA1522		
KIAA1549	The protein e	May play a role in photoreceptor function
Zinc Finger SWIM-Type Conta	The protein e	involved in nervous system developmen
Zinc Finger Protein 71		May be involved in transcriptional regula
Exocyst Complex Component	The protein e	Component of the exocyst complex invo
Coiled-Coil Domain Containing 81		
P53 Apoptosis Effector Related To PMP22		Component of intercellular desmosome
Methylcrotonoyl-CoA Carboxy	This gene en	Carboxyltransferase subunit of the 3-me
Fructosamine 3 Kinase	A high conce	Fructosamine-3-kinase involved in prote
Endoplasmic Reticulum Amin	This gene en	Aminopeptidase that plays a central role
SMAD Specific E3 Ubiquitin Protein Ligase		E3 ubiquitin-protein ligase which accepts

Poly(ADP-Ribose) Polymerase Family Mem		Mono-ADP-ribosyltransferase that media
F-Box And Leucine Rich Repe	Members of t	Substrate-recognition component of the
Niban Apoptosis Regulator 2		May play a role in apoptosis suppressior
PTEN Induced Kinase 1	This gene en	Protects against mitochondrial dysfuncti
MARCKS Like 1	This gene en	Controls cell movement by regulating ac
Zinc Finger SWIM-Type Containing 4		
WNK Lysine Deficient Protein	This gene en	Serine/threonine kinase which plays an i
Abhydrolase Domain Containi	This gene is upstream of, and in a head-to-head orier	
Cyclin J Like		
Rho GTPase Activating Protein 10		GTPase activator for the small GTPases
Protein Phosphatase 1 Regula	This gene en	Acts as a glycogen-targeting subunit for
SAP30 Like		[Isoform 1]: Functions as transcription re
VPS37B Subunit Of ESCRT-I		Component of the ESCRT-I complex, a
Dehydrogenase/Reductase 12	This gene en	Putative oxidoreductase.
Ring Finger Protein 122	The encoded	May induce necrosis and apoptosis. May
Ras And Rab Interactor 3	Summary: Th	Ras effector protein that functions as a g
Proline Rich 5 Like		Associates with the mTORC2 complex tl
KIAA0319 Like	This gene is a	(Microbial infection) Acts as a receptor fo
Endoplasmic Reticulum Metallopeptidase 1		Within the ovary, required for the organiz
Pecanex 2	This gene co	May play a role in tumorigenesis of color
Ubiquitin Domain Containing 1	The degrada	May be involved in the regulation of cellu
F-Box And Leucine Rich Repe	The protein e	Substrate-recognition component of the
Zinc Finger Protein 703		Transcriptional corepressor which does
Armadillo Repeat Containing 9		Acts as a positive regulator of hedgehog
Acyl-CoA Synthetase Family Member 2		Acyl-CoA synthases catalyze the initial r
CD276 Molecule	The protein e	May participate in the regulation of T-cel
AT-Hook Transcription Factor		Centrosomal protein that plays a key rol
Solute Carrier Family 44 Mem	The protein e	[Isoform 3]: Has also thiamine pyrophos
CYFIP Related Rac1 Interactor A		May negatively regulate RAC1 signaling
Chromosome 1 Open Reading Frame 21		
Tripartite Motif Containing 8	This gene en	E3 ubiquitin-protein ligase which plays d
Transmembrane Protein 163		May bind zinc and other divalent cations
Microtubule Associated Protei	The product	Ubiquitin-like modifier involved in format
Dedicator Of Cytokinesis 8	This gene en	Guanine nucleotide exchange factor (GE
RAB33B, Member RAS Onco	This gene en	Protein transport. Acts, in coordination w
SRY-Box Transcription Factor	This gene en	Binds to and activates the CDH5 promot
Yippee Like 3		Involved in proliferation and apoptosis in
Eva-1 Homolog A, Regulator Of Programm		Acts as a regulator of programmed cell d

NLR Family CARD Domain C	This gene en	Probable regulator of the NF-kappa-B ar
ANTXR Cell Adhesion Molecu	This gene en	Plays a role in cell attachment and migra
Mex-3 RNA Binding Family M	This gene en	RNA-binding protein. May be involved in
SH3GL Interacting Endocytic	SGIP1 functi	May function in clathrin-mediated endoc
Solute Carrier Family 9 Memb	This gene en	Mediates electroneutral exchange of pro
Family With Sequence Similarity 167 Member B		
Androgen Dependent TFPI Regulating Prot		Hydrolyzes bioactive fatty-acid esters of
Poly(ADP-Ribose) Polymeras	Poly(ADP-rib	ADP-ribosyltransferase that mediates m
FERM Domain Containing 5		May be involved in regulation of cell mig
Myosin Light Chain Kinase 2	This gene en	Implicated in the level of global muscle c
MICAL Like 1		Probable lipid-binding protein with highe
KIAA1671		
Kelch Like Family Member 6	This gene en	Involved in B-lymphocyte antigen recept
Pleckstrin Homology Like Domain Family B		Seems to be involved in the assembly of
Tetraspanin 18		
Bcl2 Modifying Factor	The protein e	May play a role in apoptosis. Isoform 1 s
StAR Related Lipid Transfer D	This gene en	GTPase-activating protein for RhoA, and
SPOC Domain Containing 1	This gene encodes a protein that belongs to the TFIIS	
Coiled-Coil Domain Containing 149		
Tripartite Motif Containing 47		E3 ubiquitin-protein ligase that mediates
Tubulin Polyglutamylase Complex Subunit		May act in the targeting of the tubulin po
Mex-3 RNA Binding Family Member A		RNA binding protein, may be involved in
ADAMTS Like 1	This gene encodes a secreted protein and member of	
Peroxisomal Biogenesis Facto	The protein e	Promotes membrane protrusion and elo
HtrA Serine Peptidase 3		Serine protease that cleaves beta-casei
Synaptotagmin Like 3	The protein e	May act as Rab effector protein and play
CKLF Like MARVEL Transme	This gene belongs to the chemokine-like factor gene s	
AHNAK Nucleoprotein 2	This gene encodes a large nucleoprotein. The encode	
Glucocorticoid Induced 1	This gene encodes a protein of unknown function. Ex	
Phosphoinositide-3-Kinase Interacting Prot		Negative regulator of hepatic phosphatio
EF-Hand Domain Containing	This gene en	Microtubule-associated protein which re
Formin Like 2	This gene en	Plays a role in the regulation of cell mor
Transmembrane Protein 200A		
PWWP Domain Containing 2A		H2A.Z-specific chromatin binding protei
C1q And TNF Related 1		
Zinc Finger Protein 618		May be involved in transcriptional regula
RAB42, Member RAS Oncogene Family		
G Protein-Coupled Receptor 146		Orphan receptor.

LY6/PLAUR Domain Containing 1		Believed to act as a modulator of nicotin
Twist Family BHLH Transcript	The protein e	Binds to the E-box consensus sequence
Par-3 Family Cell Polarity Regulator Beta		Putative adapter protein involved in asyr
ANTXR Cell Adhesion Molecu	This gene en	Necessary for cellular interactions with la
Defective In Cullin Neddylation 1 Domain C		Antagonizes DCUN1D1-mediated CUL1
IQ Motif Containing K	This gene belongs to the IQ motif-containing family of	
Zymogen Granule Protein 16B		
Musashi RNA Binding Protein	This gene en	RNA binding protein that regulates the e
Tetratricopeptide Repeat Domain 39C		
Trafficking Protein Particle Complex 5		May play a role in vesicular transport fro
Intermediate Filament Family Orphan 2		
Fibulin 7		An adhesion molecule that interacts with
OCIA Domain Containing 2		
CD109 Molecule	This gene en	Modulates negatively TGFB1 signaling in
UBX Domain Protein 2B		Adapter protein required for Golgi and er
SLIT And NTRK Like Family M	This gene en	It is involved in synaptogenesis and pror
Ankyrin Repeat And SOCS Bo	This gene en	Substrate-recognition component of a S
Zinc Finger Protein 358		May be involved in transcriptional regula
Signal Regulatory Protein Alph	The protein e	Immunoglobulin-like cell surface recepto
Cilia And Flagella Associated Protein 54		Required for assembly and function of c
C2 Calcium Dependent Domain Containing		May be involved in inflammatory process
Collagen And Calcium Binding	This gene is	Required for lymphangioblast budding a
Family With Sequence Similarity 117 Member B		
GLI Pathogenesis Related 2		
NIMA Related Kinase 10		Plays a role in the cellular response to U
SH3 Domain Containing 19	This gene en	May play a role in regulating A disintegri
Coiled-Coil Domain Containing 112		
SH3 Domain Containing Ring Finger 2		Has E3 ubiquitin-protein ligase activity (F
Schlafen Family Member 5		May have a role in hematopoietic cell dif
Cbp/P300 Interacting Transac	The protein e	Acts as transcriptional coactivator for TF
APC Down-Regulated 1 Like		
High Mobility Group AT-Hook 1 Pseudogene 2		
Transmembrane Protein 154		
Nik Related Kinase	The mouse c	May phosphorylate cofilin-1 and induce a
Sterile Alpha Motif Domain Co	This gene en	May be involved in endosome fusion. Me
OTU Deubiquitinase 1	Deubiquitinat	Deubiquitinating enzyme that specifically
Out At First Homolog		
Adhesion G Protein-Coupled Receptor F5		Receptor that plays a critical role in lung

Endosome-Lysosome Associated Apoptosi		Functions as a regulator of the BMP sigr
Fibronectin Type III Domain C	This gene en	[Irisin]: Contrary to mouse, may not be ir
Inositol Polyphosphate Multiki	This gene en	Inositol phosphate kinase with a broad s
T-Complex 11 Like 2		
N-Acetyltransferase Domain Containing 1		
KN Motif And Ankyrin Repeat Domains 3		May be involved in the control of cytoske
BCL9 Like		Transcriptional regulator that acts as an
Prolyl 4-Hydroxylase Subunit ,	This gene en	Catalyzes the post-translational formatic
CD163 Molecule Like 1	This gene encodes a member of the scavenger recep	
Kinase Suppressor Of Ras 2		Location-regulated scaffold connecting M
Solute Carrier Family 46 Mem	The protein encoded by this gene is a member of a tra	
SH3 And PX Domains 2B	This gene en	Adapter protein involved in invadopodia
Repulsive Guidance Molecule	RGMB is a gl	Member of the repulsive guidance molec
Anoctamin 9	The protein e	Has calcium-dependent phospholipid sc
Alkaline Ceramidase 2	The sphingol	Golgi ceramidase that catalyzes the hyd
Polypeptide N-Acetylgalactosaminyltransfe		Catalyzes the initial reaction in O-linked
Family With Sequence Similarity 89 Member A		
Microtubule Associated Serine	This gene encodes a member of the microtubule-assc	
Agrin	This gene en	[Agrin C-terminal 22 kDa fragment]: this
RAB15, Member RAS Oncogene Family		May act in concert with RAB3A in regula
G Protein-Coupled Receptor '	This gene en	Orphan receptor.
GTPase, Very Large Interferon Inducible Pseudogene 1		
Transmembrane Protein 233		
Yippee Like 2		
Mex-3 RNA Binding Family Member D		RNA binding protein, may be involved in
SH2 Domain Containing 5		May be involved in synaptic plasticity reg
Cortexin 1		May mediate extracellular or intracellular
Chromosome 16 Open Reading Frame 74		
DENN Domain Containing 6B		Guanine nucleotide exchange factor (GE
FKBP Prolyl Isomerase 1C		
Nuclear Factor, Erythroid 2 Like 3 Pseudogene 1		
Family With Sequence Similarity 229 Member A		
RNA, 7SL, Cytoplasmic 689, Pseudogene		
ABL Proto-Oncogene 1, Non-	This gene is	Non-receptor tyrosine-protein kinase tha
Actinin Alpha 4	Alpha actinir	F-actin cross-linking protein which is tho
Adenylate Kinase 1	This gene en	Catalyzes the reversible transfer of the t
Adenylate Kinase 4	This gene en	Involved in maintaining the homeostasis
Aldehyde Dehydrogenase 2 F	This protein belongs to the aldehyde dehydrogenase	

Bridging Integrator 1	This gene en	Is a key player in the control of plasma r
Solute Carrier Family 25 Mem	This gene is	Involved in mitochondrial ADP/ATP trans
Annexin A2	This gene en	Calcium-regulated membrane-binding pr
Annexin A2 Pseudogene 2		Calcium-regulated membrane-binding pr
Annexin A2 Pseudogene 3		
Annexin A6	Annexin VI b	May associate with CD21. May regulate
Apoptotic Peptidase Activating	This gene en	Oligomeric Apaf-1 mediates the cytochro
Amyloid Beta Precursor Prote	The protein e	Putative function in synaptic vesicle exo
Amyloid Beta Precursor Prote	The protein e	Transcription coregulator that can have
Amyloid Beta Precursor Like I	This gene en	May play a role in the regulation of hem
Amyloid Beta Precursor Prote	This gene en	Amyloid-beta peptides are lipophilic met
Ras Homolog Family Member	This gene en	Regulates a signal transduction pathway
Rho GDP Dissociation Inhibit	The GDP-dis	Inhibits GDP/GTP exchange reaction of
ATPase H+ Transporting Acc	This gene en	Accessory subunit of the proton-transpo
ATRX Chromatin Remodeler	The protein e	Involved in transcriptional regulation and
BRCA1 Associated RING Dor	This gene en	E3 ubiquitin-protein ligase. The BRCA1-
Branched Chain Keto Acid De	This gene en	The branched-chain alpha-keto dehydro
BAF Chromatin Remodeling C	This gene is directly involved, with Myc and IgH, in a t	
BCS1 Homolog, Ubiquinol-Cy	This gene en	Chaperone necessary for the assembly
Brain Derived Neurotrophic Fa	This gene en	[BDNF precursor form]: Important signal
Beaded Filament Structural P	This gene en	Required for the correct formation of len
Basigin (Ok Blood Group)	The protein e	(Microbial infection) Erythrocyte recepto
Bone Marrow Stromal Cell An	Bone marrow	IFN-induced antiviral host restriction fac
Biotinidase	The protein e	Catalytic release of biotin from biocytin,
Diacylglycerol Lipase Alpha	This gene en	Catalyzes the hydrolysis of diacylglycero
Calpain 1	The calpains	Calcium-regulated non-lysosomal thiol-p
Calpastatin	The protein e	Specific inhibition of calpain (calcium-de
Caspase 6	This gene en	Involved in the activation cascade of cas
Catalase	This gene en	Occurs in almost all aerobically respiring
RUNX Family Transcription Fa	Core binding	Forms the heterodimeric complex core-b
CD47 Molecule	This gene en	Has a role in both cell adhesion by actin
Cadherin 4	This gene is	Cadherins are calcium-dependent cell a
Cyclin Dependent Kinase 6	The protein e	Serine/threonine-protein kinase involved
Cyclin Dependent Kinase Inhi	This gene ge	Acts as a negative regulator of the prolif
Cyclin Dependent Kinase Inhi	The protein e	Interacts strongly with CDK6, weakly wit
Cyclin Dependent Kinase Inhi	The protein e	Interacts strongly with CDK4 and CDK6
Chimerin 1	This gene en	GTPase-activating protein for p21-rac ar
Chloride Voltage-Gated Chan	The CLCN fa	Proton-coupled chloride transporter. Fur

Chloride Voltage-Gated Chan	This gene en	Proton-coupled chloride transporter. Fur
Clusterin	The protein e	[Isoform 1]: Functions as extracellular ch
Collagen Type VIII Alpha 1 Ch	This gene en	Macromolecular component of the suber
Carboxypeptidase D	The metallocarboxypeptidase family of enzymes is div	
Cysteine Rich Protein 1	Cysteine-rich	Seems to have a role in zinc absorption
C-Reactive Protein	The protein e	Displays several functions associated wi
Hyaluronan And Proteoglycan Link Protein		Stabilizes the aggregates of proteoglyca
Crystallin Alpha B	Mammalian l	May contribute to the transparency and r
Cystatin C	The cystatin s	As an inhibitor of cysteine proteinases, t
Cellular Communication Netw	The protein e	Major connective tissue mitoattractant se
Catenin Alpha 1	This gene en	Associates with the cytoplasmic domain
Cathepsin D	This gene en	Acid protease active in intracellular prote
Cathepsin Z	The protein e	Exhibits carboxy-monopeptidase as well
Death Associated Protein	This gene en	Negative regulator of autophagy. Involve
Drebrin 1	The protein e	Actin cytoskeleton-organizing protein tha
Dystrophin	This gene sp	Anchors the extracellular matrix to the cy
DNA Replication Helicase/Nu	This gene en	Key enzyme involved in DNA replication
Dedicator Of Cytokinesis 1	This gene en	Involved in cytoskeletal rearrangements
Dipeptidyl Peptidase 4	The DPP4 ge	Cell surface glycoprotein receptor involv
Dystrobrevin Alpha	The protein e	May be involved in the formation and sta
Extracellular Matrix Protein 1	This gene en	Involved in endochondral bone formation
Eukaryotic Translation Initiation Factor 4B		Required for the binding of mRNA to ribo
Erythrocyte Membrane Protei	Erythrocyte n	May function to confer stability and plast
Erythropoietin Receptor	This gene en	Isoform EPOR-T acts as a dominant-neg
Nuclear Receptor Subfamily 2 Group F Mer		Transcription factor predominantly involv
ETS Variant Transcription Factor 4		Transcriptional activator that binds to the
FA Complementation Group E	The Fanconi	As part of the Fanconi anemia (FA) com
Acyl-CoA Synthetase Long Ch	The protein e	Catalyzes the conversion of long-chain f
Acyl-CoA Synthetase Long Ch	The protein e	Acyl-CoA synthetases (ACSL) activates
Acyl-CoA Synthetase Long Ch	The protein e	Catalyzes the conversion of long-chain f
Fibrillin 2	The protein e	[Fibrillin-2]: Fibrillins are structural comp
Flap Structure-Specific Endor	The protein e	Structure-specific nuclease with 5'-flap e
Fibroblast Growth Factor Rec	The protein e	Tyrosine-protein kinase that acts as cell-
Four And A Half LIM Domains	This gene en	May have an involvement in muscle dev
Four And A Half LIM Domains	The protein e	Recruited by SOX15 to FOXK1 promote
Flotillin 2	Caveolae are	May act as a scaffolding protein within ca
Fms Related Receptor Tyrosi	This gene en	Tyrosine-protein kinase that acts as a ce
Alpha-L-Fucosidase 2	This gene en	Alpha-L-fucosidase is responsible for hy

Atypical Chemokine Receptor	The protein e	Atypical chemokine receptor that control
Glucosidase Alpha, Acid	This gene en	Essential for the degradation of glycoge
Galactosamine (N-Acetyl)-6-S	This gene encodes N-acetylgalactosamine-6-sulfatas	
GATA Binding Protein 3	This gene en	Transcriptional activator which binds to t
Glucosaminyl (N-Acetyl) Tran	This gene is	Glycosyltransferase that catalyzes the tr
Galactosidase Beta 1	This gene en	[Isoform 1]: Cleaves beta-linked termina
Glycine Decarboxylase	Degradation	The glycine cleavage system catalyzes t
Glycine Decarboxylase Pseudogene 1		
Glutaminase	This gene en	[Isoform 2]: Lacks catalytic activity.||Cat
Glucosamine (N-Acetyl)-6-Sul	The product of this gene is a lysosomal enzyme foun	
Granulin Precursor	Granulins ar	[Granulin-3]: Inhibits epithelial cell prolif
Glutamate Ionotropic Receptor	NMDA Typ	Potential apoptotic regulator.
Glycogen Synthase Kinase 3	The protein e	Constitutively active protein kinase that
Glutathione S-Transferase M	Cytosolic an	Conjugation of reduced glutathione to a
General Transcription Factor	This gene en	Interacts with the basal transcription ma
Hydroxyacyl-CoA Dehydroger	This gene en	Mitochondrial trifunctional enzyme catal
Hydroxyacyl-CoA Dehydroger	This gene en	Mitochondrial trifunctional enzyme catal
Histone Deacetylase 1	Histone acet	Responsible for the deacetylation of lysi
Helicase, Lymphoid Specific	This gene en	Plays an essential role in normal develo
Hypoxia Inducible Factor 1 S	This gene en	Functions as a master transcriptional re
Major Histocompatibility Com	HLA-A belon	Allele A*01:01: Presents a restricted pe
Major Histocompatibility Com	HLA-B belon	Allele B*07:02: Displays peptides sharin
Major Histocompatibility Com	This major hi	Involved in the presentation of foreign a
Major Histocompatibility Com	This major histocompatibility complex gene represent	
Heme Oxygenase 2	Heme oxyge	Heme oxygenase cleaves the heme ring
Heterogeneous Nuclear Ribo	This gene be	Binds pre-mRNA and nucleates the ass
Homeobox B9	This gene is	Sequence-specific transcription factor w
Hydroxysteroid 17-Beta Dehydrogenase 2		Capable of catalyzing the interconversio
5-Hydroxytryptamine Recepto	The protein e	G-protein coupled receptor for 5-hydrox
Inhibitor Of DNA Binding 3, H	The protein e	Transcriptional regulator (lacking a basi
Iduronate 2-Sulfatase	This gene en	Lysosomal enzyme involved in the degra
Interferon Alpha And Beta Re	The protein e	Component of the receptor for type I int
Interferon Gamma Receptor 2	This gene (IF	Associates with IFNGR1 to form a recep
Insulin Like Growth Factor 2 F	This gene en	Transport of phosphorylated lysosomal
Insulin Like Growth Factor Bir	This gene is	IGF-binding proteins prolong the half-life
Insulin Like Growth Factor Bir	This gene is	IGF-binding proteins prolong the half-life
Insulin Like Growth Factor Binding Protein (		IGF-binding proteins prolong the half-life
Insulin Like Growth Factor Bir	This gene en	Binds IGF-I and IGF-II with a relatively l

Interleukin 4 Receptor	This gene en	Receptor for both interleukin 4 and interl
Interleukin 18	The protein e	A proinflammatory cytokine primarily inv
Integrin Subunit Beta 5	This gene en	(Microbial infection) Integrin ITGAV:ITGI
Inositol 1,4,5-Trisphosphate F	This gene en	Receptor for inositol 1,4,5-trisphosphate
Jumonji And AT-Rich Interacti	This gene en	Regulator of histone methyltransferase c
Potassium Two Pore Domain	This gene en	Ion channel that contributes to passive t
Kinesin Family Member 22	The protein e	Kinesin family member that is involved ir
Keratin 15	The protein encoded by this gene is a member of the	
Keratin 19	The protein e	Involved in the organization of myofibers
Lysosomal Associated Membr	The protein e	Acts as a receptor for Lassa virus protei
LIM And SH3 Protein 1	This gene en	Plays an important role in the regulation
LIM Domain Kinase 1	There are ap	[Isoform 3]: Has a dominant negative eff
Latent Transforming Growth F	The protein e	May play an integral structural role in ela
Basal Cell Adhesion Molecule	This gene en	Laminin alpha-5 receptor. May mediate i
Mannosidase Alpha Class 1A	This gene en	Involved in the maturation of Asn-linked
Mannosidase Alpha Class 2B	This gene en	Necessary for the catabolism of N-linkec
Mannosidase Beta	This gene en	Exoglycosidase that cleaves the single b
Monoamine Oxidase A	This gene is	Catalyzes the oxidative deamination of b
Microtubule Associated Protei	This gene en	Structural protein involved in the filamen
Microtubule Associated Protei	This gene en	The exact function of MAP2 is unknown
Microtubule Associated Protei	The protein e	Non-neuronal microtubule-associated pr
Milk Fat Globule EGF And Fa	This gene en	Medin is the main constituent of aortic m
Alpha-1,6-Mannosylglycoprote	The protein e	[Secreted alpha-1,6-mannosylglycoprote
Matrix Gla Protein	This gene en	Associates with the organic matrix of bo
Membrane Metalloendopeptid	The protein e	Thermolysin-like specificity, but is almos
Matrix Metallopeptidase 2	This gene is	[Isoform 2]: Mediates the proteolysis of (
Matrix Metallopeptidase 7	This gene en	Degrades casein, gelatins of types I, III,
Mercaptopyruvate Sulfurtrans	This protein e	Transfer of a sulfur ion to cyanide or to c
Myosin Heavy Chain 9	This gene en	Cellular myosin that appears to play a ro
Myosin Heavy Chain 10	This gene en	Cellular myosin that appears to play a ro
Myosin Light Chain 4	Myosin is a h	Regulatory light chain of myosin. Does n
Myosin ID		Unconventional myosin that functions as
Myosin VI	This gene en	Myosins are actin-based motor molecule
Nuclear Factor I X	The protein e	Recognizes and binds the palindromic s
Nuclear Factor Kappa B Subu	This gene en	NF-kappa-B is a pleiotropic transcription
Notch Receptor 2	This gene en	Functions as a receptor for membrane-b
Natriuretic Peptide Receptor 1	Guanylyl cycl	Receptor for the atrial natriuretic peptide
Discoidin Domain Receptor T	This gene en	Tyrosine kinase involved in the regulatio

Nucleobindin 1	This gene en	Major calcium-binding protein of the Gol
Claudin 11	This gene en	Plays a major role in tight junction-speci
Phosphatidylethanolamine Bi	This gene en	Binds ATP, opioids and phosphatidyleth
Phosducin Like	Phosducin-lik	[Isoform 1]: Functions as a co-chaperon
Pterin-4 Alpha-Carbinolamine	This gene en	Involved in tetrahydrobiopterin biosynthe
Protocadherin Gamma Subfa	This gene is	Potential calcium-dependent cell-adhesi
Proprotein Convertase Subtili	This gene en	Serine endoprotease that processes var
Phosphofructokinase, Platele	This gene en	Catalyzes the phosphorylation of D-fruct
Phosphogluconate Dehydrog	6-phosphogl	Catalyzes the oxidative decarboxylation
Phosphatidylinositol Transfer	This gene en	Catalyzes the transfer of PtdIns and pho
Plasminogen Activator, Uroki	This gene en	Acts as a receptor for urokinase plasmin
Procollagen-Lysine,2-Oxoglut	Lysyl hydrox	Part of a complex composed of PLOD1,
PML Nuclear Body Scaffold	The protein e	Exhibits antiviral activity against both D
Paired Related Homeobox 1	The DNA-ass	Acts as a transcriptional regulator of mu
Peroxisome Proliferator Activ	Peroxisome	Ligand-activated transcription factor. Ke
Protein Phosphatase 3 Catalytic Subunit Al		Calcium-dependent, calmodulin-stimulat
DNA Primase Subunit 1	The replicati	Catalytic subunit of the DNA primase co
Protein Kinase AMP-Activate	The protein e	Non-catalytic subunit of AMP-activated
CAMP-Dependent Protein Ki	The protein e	Extremely potent competitive inhibitor of
Protein Kinase C Alpha	Protein kinas	Calcium-activated, phospholipid- and di
Protein Kinase X-Linked	This gene en	Serine/threonine protein kinase regulate
Prion Protein	The protein e	Its primary physiological function is uncl
Protein S	This gene en	Anticoagulant plasma protein; it is a cof
Proline Rich And Gla Domain	This gene encodes a vitamin K-dependent, gamma-c	
HtrA Serine Peptidase 1	This gene en	Serine protease with a variety of targets
Prosaposin	This gene en	[Prosaposin]: Behaves as a myelinotrop
Presenilin 2	Alzheimer's	Probable catalytic subunit of the gamma
Proteasome 20S Subunit Bet	The proteas	The proteasome is a multicatalytic prote
Proteasome Activator Subuni	The 26S prot	Implicated in immunoproteasome assem
Phosphatase And Tensin Ho	This gene w	[Isoform alpha]: Functional kinase, like i
Prostaglandin E Receptor 1	The protein e	Receptor for prostaglandin E2 (PGE2).
Prostaglandin F2 Receptor Inhibitor		Inhibits the binding of prostaglandin F2-
Protein Tyrosine Kinase 2	This gene en	[Isoform 6]: Isoform 6 (FRNK) does not
Protein Tyrosine Phosphatas	The protein e	Protein tyrosine phosphatase which ma
Protein Tyrosine Phosphatas	The protein e	Regulation of processes involving cell c
6-Pyruvoyltetrahydropterin Sy	The enzyme	Involved in the biosynthesis of tetrahydr
RAB5B, Member RAS Oncogene Family		Protein transport. Probably involved in v
Ral Guanine Nucleotide Diss	Guanine nuc	Stimulates the dissociation of GDP from

Phospholipase A And Acyltrar	Retinoids exe	Exhibits both phospholipase A1/2 and ac
RB Binding Protein 8, Endonu	The protein e	Endonuclease that cooperates with the I
Reticulocalbin 1	Reticulocalbi	May regulate calcium-dependent activitie
Regulatory Factor X3	This gene is a	Transcription factor required for ciliogene
Regulator Of G Protein Signal	This gene en	Down-regulates signaling from heterotrir
Arginyl Aminopeptidase		Exopeptidase which selectively removes
Retinitis Pigmentosa GTPase	This gene en	Could be a guanine-nucleotide releasing
Ribosomal Protein S6 Kinase	This gene en	Serine/threonine-protein kinase that acts
Ribosome Binding Protein 1	This gene en	Acts as a ribosome receptor and mediat
Syndecan 4	The protein e	Cell surface proteoglycan that bears hep
Sarcoglycan Gamma	This gene en	Component of the sarcoglycan complex,
SH3 Domain Binding Glutamate Rich Protein Like		
Solute Carrier Family 3 Memb	This gene is a	Component of several heterodimeric am
Solute Carrier Family 8 Memb	In cardiac my	Mediates the exchange of one Ca(2+) io
Solute Carrier Family 12 Mem	The protein e	Electrically silent transporter system. Me
SWI/SNF Related, Matrix Ass	The protein e	Involved in transcriptional activation and
Sepiapterin Reductase	This gene en	Catalyzes the final one or two reductions
ST13 Hsp70 Interacting Prote	The protein e	One HIP oligomer binds the ATPase dor
Signal Transducer And Activa	The protein e	Signal transducer and transcription activ
Sulfotransferase Family 1E M	Sulfotransfer	Sulfotransferase that utilizes 3'-phospho
Serine/Threonine Kinase 10	This gene en	Serine/threonine-protein kinase involved
ADAM Metallopeptidase Dom	This gene en	Cleaves the membrane-bound precursor
Transducin Beta Like 1 X-Link	The protein e	F-box-like protein involved in the recruitr
Transcription Factor 19	This gene en	Potential trans-activating factor that coul
Transcobalamin 2	This gene en	Primary vitamin B12-binding and transpo
Peroxiredoxin 2	This gene en	Thiol-specific peroxidase that catalyzes
Transcription Factor Dp-2	The gene is a	Can stimulate E2F-dependent transcript
Transforming Growth Factor E	The protein e	Transmembrane serine/threonine kinase
Tight Junction Protein 1	This gene en	TJP1, TJP2, and TJP3 are closely relate
Tetraspanin 8	The protein encoded by this gene is a member of the	
TNF Receptor Superfamily Me	The protein e	Receptor with high affinity for TNFSF2/T
Tensin 1	The protein e	Involved in fibrillar adhesion formation. M
DNA Topoisomerase II Beta	This gene en	Control of topological states of DNA by t
Tumor Protein P53	This gene en	Acts as a tumor suppressor in many tum
TSPY Like 1	The protein encoded by this gene is found in the nucle	
Pleckstrin Homology Like Dor	This gene is	Plays a role in regulating placenta growt
Thiosulfate Sulfurtransferase	This is one o	Formation of iron-sulfur complexes, cyar
GDP-L-Fucose Synthase	Tissue specif	Catalyzes the two-step NADP-depender

Uridine-Cytidine Kinase 2	This gene en	Phosphorylates uridine and cytidine to u
Vascular Endothelial Growth F	This gene en	Growth factor for endothelial cells. VEGI
WAS/WASL Interacting Prote	This gene en	Plays a role in the reorganization of the a
YY1 Transcription Factor	YY1 is a ubiq	Multifunctional transcription factor that e
Zinc Finger Protein 134		May be involved in transcriptional regula
Tripartite Motif Containing 25	The protein e	Functions as a ubiquitin E3 ligase and a
Zinc Finger Protein 217		Binds to the promoters of target genes a
LDL Receptor Related Proteir	This gene en	Cell surface receptor for Reelin (RELN) a
Tubulin Alpha 1a	Microtubules	Tubulin is the major constituent of micro
Tissue Factor Pathway Inhibit	This gene en	May play a role in the regulation of plasr
FMR1 Autosomal Homolog 1	The protein e	RNA-binding protein required for embryc
Transmembrane Protein 187	This gene consists of two exons and encodes a multi-	
Stannin		Plays a role in the toxic effects of organc
Cell Division Cycle 7	This gene en	Seems to phosphorylate critical substrat
Cell Division Cycle 45	The protein e	Required for initiation of chromosomal D
Hyaluronidase 3	This gene en	Facilitates sperm penetration into the lay
NCK Adaptor Protein 2	This gene en	Adapter protein which associates with ty
Tyrosylprotein Sulfotransferas	The protein e	Catalyzes the O-sulfation of tyrosine res
PPFIA Binding Protein 1	The protein e	May regulate the disassembly of focal ac
Potassium Voltage-Gated Cha	Voltage-gate	Cytoplasmic potassium channel subunit
Interferon Induced Transmembrane Protein		IFN-induced antiviral protein which inhib
Pirin	This gene en	Transcriptional coregulator of NF-kappa-
Regulator Of G Protein Signal	The protein e	Inhibits signal transduction by increasing
Phospholipid Phosphatase 2	The protein e	Magnesium-independent phospholipid p
Ribonuclease T2	This ribonucl	Ribonuclease that plays an essential rol
Protocadherin Gamma Subfai	This gene is a	Potential calcium-dependent cell-adhesi
Aldo-Keto Reductase Family 1	This gene en	Catalyzes the conversion of aldehydes a
NUMB Endocytic Adaptor Pro	The protein e	Plays a role in the process of neurogene
Tankyrase		Poly-ADP-ribosyltransferase involved in
Insulin Receptor Substrate 2	This gene en	May mediate the control of various cellul
Cathepsin F	Cathepsins a	Thiol protease which is believed to partic
ADAM Metallopeptidase Dom	The protein e	Active metalloproteinase with gelatinolyt
Cellular Repressor Of E1A Sti	The adenovir	May contribute to the transcriptional con
Neuropilin 2	This gene en	(Microbial infection) Acts as a receptor fo
Suppressor Of Cytokine Signa	This gene en	SOCS family proteins form part of a clas
Aldehyde Dehydrogenase 1 F	This protein b	Converts retinaldehyde to retinoic acid (I
Immediate Early Response 3	This gene fur	May play a role in the ERK signaling pat
Rho Guanine Nucleotide Exch	This gene en	Acts as a RAC1 guanine nucleotide excl

Src Kinase Associated Phosp	The protein e	May be involved in B-cell and macropha
Chloride Intracellular Channel	Chloride cha	Can insert into membranes and form chl
3'-Phosphoadenosine 5'-Phos	Sulfation is a	Bifunctional enzyme with both ATP sulfu
LIM Domain Binding 2	The protein e	Binds to the LIM domain of a wide variet
Protein Kinase, Membrane As	This gene en	Acts as a negative regulator of entry intc
Interleukin 1 Receptor Like 1	The protein e	[Isoform B]: Inhibits IL-33 signaling.||Rec
Zinc Finger BED-Type Contai	This gene is	Binds to 5'-TGTCG[CT]GA[CT]A-3' DNA
Solute Carrier Family 16 Mem	This gene is	Proton-coupled monocarboxylate transp
Cytohesin 3	This gene en	Promotes guanine-nucleotide exchange
Adhesion G Protein-Coupled	This gene en	[ADGRG1 N-terminal fragment]: Plays a
Beta-1,4-Galactosyltransferas	This gene is	Catalyzes the synthesis of lactosylceram
Zinc Finger FYVE-Type Conta	This gene en	Early endosomal protein that functions t
Family With Sequence Similarity 189 Member A2		
Mediator Complex Subunit 17	The activatio	Component of the Mediator complex, a
Integral Membrane Protein 2E	Amyloid prec	Bri23 peptide prevents aggregation of A
RAS Protein Activator Like 2	This gene en	Inhibitory regulator of the Ras-cyclic AM
Interleukin 27 Receptor Subu	In mice, CD4	Receptor for IL27. Requires IL6ST/gp13
Solute Carrier Family 4 Memb	The protein e	Mediates electroneutral sodium- and ca
Aminopeptidase Puromycin S	This gene en	Aminopeptidase with broad substrate sp
Vacuolar Protein Sorting 4 Ho	The protein e	Involved in late steps of the endosomal
Nuclear Receptor Corepresso	This gene en	Mediates transcriptional repression by c
Microtubule Associated Mono	The protein e	Nuclear monooxygenase that promotes
Protein Phosphatase, Mg2+/M	The protein e	Dephosphorylates and concomitantly de
Syndecan 3	The protein e	Cell surface proteoglycan that may bear
Rap Guanine Nucleotide Exch	Members of t	Functions as a guanine nucleotide exch
Translocation Associated Mer	TRAM2 is a	Necessary for collagen type I synthesis.
Thymocyte Selection Associa	The protein e	Transcriptional regulator with a major rol
Sushi Domain Containing 6		May play a role in growth-suppressive a
RB1 Inducible Coiled-Coil 1	The protein e	Involved in autophagy (PubMed:217758
GINS Complex Subunit 1	The yeast he	Required for correct functioning of the G
Centrosomal Protein 170	The product	Plays a role in microtubule organization
Kelch Like Family Member 21		Substrate-specific adapter of a BCR (BT
Ring Finger Protein 10	The protein e	Transcriptional factor involved in the reg
Major Vault Protein	This gene en	Required for normal vault structure. Vau
Histone Deacetylase 5	Histones play	Responsible for the deacetylation of lysi
DnaJ Heat Shock Protein Fan	This gene en	Plays an indispensable role in the organ
Tetraspanin 5	The protein e	Regulates ADAM10 maturation and traff
Solute Carrier Family 25 Mem	This gene is	Mitochondrial and calcium-binding carrie

Dehydrogenase/Reductase 9	This gene en	3-alpha-hydroxysteroid dehydrogenase t
Myelin Protein Zero Like 2	Thymus deve	Mediates homophilic cell-cell adhesion.
P21 (RAC1) Activated Kinase	PAK proteins	Serine/threonine protein kinase that play
High Mobility Group 20A		Plays a role in neuronal differentiation as
Carboxypeptidase Q	This gene en	Carboxypeptidase that may play an impo
Component Of Oligomeric Go	The protein e	Required for normal Golgi function.
Follistatin	Follistatin is	Binds directly to activin and functions as
Unc-13 Homolog B	This gene is	Plays a role in vesicle maturation during
Fibulin 5	The protein e	Essential for elastic fiber formation, is in
Sorbin And SH3 Domain Cont	This gene en	Plays a role in tyrosine phosphorylation
Interferon Induced Transmembrane Protein		IFN-induced antiviral protein which inhib
Poly(A) Binding Protein Intera	The protein e	Acts as a coactivator in the regulation of
RAD51 Associated Protein 1		May participate in a common DNA dama
Calcium/Calmodulin Depende	The product	Calcium/calmodulin-dependent protein k
Ras Related GTP Binding A		(Microbial infection) May alternatively ac
G Protein Subunit Beta 5	Heterotrimeri	Enhances GTPase-activating protein (G
Nestin	This gene en	Required for brain and eye development
Fibrinogen Like 2	The protein e	May play a role in physiologic lymphocyt
Neuropeptide FF Receptor 2	This gene en	Receptor for NPAF (A-18-F-amide) and
UDP Glucuronosyltransferase	The protein e	UDP-glucuronosyltransferases catalyze
Translocase Of Outer Mitocho	The protein e	Plays a role in the import of cytosolically
OS9 Endoplasmic Reticulum	This gene en	Lectin which functions in endoplasmic re
Cyclin I	The protein encoded by this gene belongs to the highl	
Midline 2	The protein e	May play a role in microtubule stabilizati
Solute Carrier Family 35 Mem	Nucleotide su	Antiporter transporting nucleotide sugars
DnaJ Heat Shock Protein Fan	The protein e	Probable chaperone. Stimulates ATP hy
Serine Protease 23	This gene encodes a conserved member of the trypsi	
Butyrophilin Subfamily 3 Mem	This gene en	Plays a role in T-cell responses in the ac
WD Repeat Domain 45	This gene en	Component of the autophagy machinery
RAB, Member Of RAS Oncog	The RABL2B	Small GTPase required for ciliation. Acti
Follistatin Like 1	This gene en	May modulate the action of some growth
Solute Carrier Family 2 Memb	Hexose trans	Probable sugar transporter that acts as a
TP53 Target 1		
Kruppel Like Factor 12	Activator prot	Confers strong transcriptional repressior
Alpha-1,3-Mannosyl-Glycopro	This gene en	Glycosyltransferase that participates in t
Twinfilin Actin Binding Protein	The protein e	Actin-binding protein involved in motile a
Muscle RAS Oncogene Homo	This gene en	Serves as an important signal transduce
Calsyntenin 1	This gene is	Induces KLC1 association with vesicles

Zinc Finger And BTB Domain Containing 1		Acts as a transcriptional repressor (PubM
Kinesin Associated Protein 3	The small G	Involved in tethering the chromosomes t
RAB3 GTPase Activating Prot	This gene en	Probable catalytic subunit of a GTPase a
Prostaglandin Reductase 1	This gene en	Functions as 15-oxo-prostaglandin 13-re
Tubulin Tyrosine Ligase Like !	This gene en	Polyglutamylase which preferentially mo
Cyclin Dependent Kinase 19	This gene encodes a protein that is one of the compo	
Centrosomal Protein 68		Involved in maintenance of centrosome
Cytoplasmic FMR1 Interacting	This gene en	Component of the CYFIP1-EIF4E-FMR1
Proteasome Activator Subunit 4		Associated component of the proteasom
Tripartite Motif Containing 2	The protein e	UBE2D1-dependent E3 ubiquitin-protein
Mannosidase Alpha Class 2B Member 2		
KH And NYN Domain Contain	This gene encodes a protein containing a ribonucleas	
HAUS Augmin Like Complex :	HAUS5 is 1 c	Contributes to mitotic spindle assembly,
Pleckstrin And Sec7 Domain Containing 3		Guanine nucleotide exchange factor for
Coactosin Like F-Actin Bindin	This gene en	Binds to F-actin in a calcium-independen
Neuronal Calcium Sensor 1	This gene is a	Neuronal calcium sensor, regulator of G
NODAL Modulator 1	This gene en	May antagonize Nodal signaling.
Ribosomal Protein L13a	Ribosomes, t	Associated with ribosomes but is not rec
Coronin 1C	This gene en	[Isoform 3]: Involved in myogenic differe
CD2 Associated Protein	This gene en	Seems to act as an adapter protein betw
Makorin Ring Finger Protein 1	This gene en	E3 ubiquitin ligase catalyzing the covale
Family With Sequence Similarity 89 Membe		Negatively regulates TGF-beta-induced
LDOC1 Regulator Of NFKB S	The protein e	May have an important role in the develo
Phospholipase D Family Mem	This gene en	5'->3' DNA exonuclease which digests s
Solute Carrier Family 7 Memb	This gene en	Sodium-independent, high-affinity excha
MAF BZIP Transcription Facto	The protein e	Since they lack a putative transactivation
Apolipoprotein L2	This gene is a	May affect the movement of lipids in the
BCL2 Like 13	This gene en	May promote the activation of caspase-3
F-Box And Leucine Rich Repe	This gene en	Calcium-activated substrate recognition
Dynein Axonemal Heavy Chai	This gene en	Force generating protein of cilia required
Signal Induced Proliferation Associated 1 L		Stimulates the GTPase activity of RAP2
RAB11 Family Interacting Protein 5		Rab effector involved in protein traffickin
Retinoic Acid Induced 14		Plays a role in actin regulation at the ect
Retrotransposon Gag Like 8A		
Tectonic Family Member 3	This gene en	Part of the tectonic-like complex which is
PHD Finger Protein 19		Polycomb group (PcG) that specifically b
RIB43A Domain With Coiled-Coils 2		
F-Box And WD Repeat Doma	This gene en	Substrate-recognition component of a C

F-Box Protein 22	This gene en	Substrate-recognition component of the
F-Box Protein 5	This gene en	Regulator of APC activity during mitotic a
Seizure Related 6 Homolog Li	This gene en	May contribute to specialized endoplasm
Gremlin 1, DAN Family BMP A	This gene en	Cytokine that may play an important role
STEAP Family Member 1	This gene is	Metalloreductase that has the ability to r
Ankyrin Repeat Domain 1	The protein e	May play an important role in endothelia
Staufen Double-Stranded RNA	Staufen hom	RNA-binding protein required for the mic
Forkhead Box P1	This gene be	[Isoform 8]: Involved in transcriptional re
Solute Carrier Organic Anion Transporter F		Mediates the Na(+)-independent transpo
Adipogenesis Associated Mth938 Domain C		May play a role in preadipocyte different
Intraflagellar Transport 81	The protein e	Component of the intraflagellar transpor
Tropomodulin 3		Blocks the elongation and depolymeriza
Caspase Recruitment Domain	The caspase	Activates NF-kappa-B via BCL10 and IK
Parvin Beta	This gene en	Adapter protein that plays a role in integ
Zinc Finger DHHC-Type Containing 1		Could also be involved in DNA virus-trigg
RNA Binding Motif Protein 15B	Members of t	RNA-binding protein that acts as a key r
Oxidative Stress Induced Gro	This gene en	Regulates the differentiation and prolifer
Cerebellar Degeneration Related Protein 2 Like		
Carbohydrate Sulfotransferas	The protein e	Catalyzes the transfer of sulfate to positi
Anoctamin 7	This prostate	Has calcium-dependent phospholipid sc
Adenylate Kinase 3	The protein e	Involved in maintaining the homeostasis
UBX Domain Protein 1		Ubiquitin-binding protein that plays a role
Zinc Finger DHHC-Type Palm	This gene en	The ZDHHC9-GOLGA7 complex is a pa
Inhibitor Of Growth Family Me	This gene en	Component of HBO1 complexes, which
Cerebral Endothelial Cell Adhesion Molecu		Probable cell adhesion protein involved
Golgi Membrane Protein 1	The Golgi co	Unknown. Cellular response protein to v
Mex-3 RNA Binding Family M	This gene en	E3 ubiquitin ligase responsible for the po
Sorting Nexin 7	This gene en	May be involved in several stages of intr
Eukaryotic Translation Initiation Factor 3 Su		(Microbial infection) In case of FCV infec
Protein Kinase AMP-Activated	AMP-activate	AMP/ATP-binding subunit of AMP-activa
Ubiquitin Conjugating Enzyme	The modifica	Catalyzes the covalent attachment of ub
LIM Domain And Actin Binding	This gene en	Actin-binding protein involved in actin cy
CXXC Finger Protein 5	The protein e	May indirectly participate in activation of
CutA Divalent Cation Tolerance Homolog		May form part of a complex of membran
Yippee Like 5		Component of the CTLH E3 ubiquitin-pro
Store-Operated Calcium Entry Associated R		Negative regulator of store-operated Ca
Nemo Like Kinase		Serine/threonine-protein kinase that regu
Calcium Binding Protein 39		Component of a complex that binds and

Regulator Of Telomere Elonga	This gene en	ATP-dependent DNA helicase implicated
Endoplasmic Reticulum Amino	The protein e	Aminopeptidase that plays a central role
Transmembrane 7 Superfamily Member 3		Involved in the inhibition of cytokine-indu
SIX Homeobox 4	This gene en	Transcriptional regulator which can act a
Phosphorylated Adaptor For RNA Export		A phosphoprotein adapter involved in the
Casein Kinase 1 Gamma 1	This gene en	Serine/threonine-protein kinase. Casein
Alpha 1,4-Galactosyltransfera	The protein e	(Microbial infection) Globotriaosylcerami
ERBB Receptor Feedback Inh	ERRFI1 is a	Negative regulator of EGFR signaling in
Chromosome 11 Open Reading Frame 71		
Ubiquitin Specific Peptidase 53		Tight junction-associated protein that is i
Coiled-Coil Alpha-Helical Rod	This gene en	May be a regulator of keratinocyte prolife
Exocyst Complex Component	The protein e	Component of the exocyst complex invo
Roundabout Guidance Receptor 4		Receptor for Slit proteins, at least for SL
Leucine Zipper Transcription I	This gene en	Regulates ciliary localization of the BBS
Tripartite Motif Containing 44	This gene en	May play a role in the process of differen
Basonuclin 2	This gene en	Probable transcription factor specific for
Vacuolar Protein Sorting 13 H	This gene en	Necessary for proper mitochondrial func
Retinol Saturase		Catalyzes the saturation of all-trans-retin
Focadhesin		Potential tumor suppressor in gliomas.
Ubiquitin Conjugating Enzyme	Protein kinas	Accepts ubiquitin from the E1 complex a
Dual Specificity Phosphatase 23		Protein phosphatase that mediates deph
Transmembrane Protein 160		
Gem Nuclear Organelle Asso	The protein e	The SMN complex plays a catalyst role i
CLN6 Transmembrane ER Pr	This gene is one of eight which have been associated	
Transmembrane Protein 51		
Transmembrane Protein 38B	This gene en	Monovalent cation channel required for r
NAD Synthetase 1	Nicotinamide	Catalyzes the ATP-dependent amidation
Microtubule Associated Protein 1S		Microtubule-associated protein that med
Zinc Finger Protein 532		May be involved in transcriptional regula
Prolyl 3-Hydroxylase 2	This gene en	Prolyl 3-hydroxylase that catalyzes the p
Exonuclease 3'-5' Domain Containing 2		Exonuclease that has both 3'-5' exoribon
Ubiquitin Like Modifier Activat	Modification	Activates ubiquitin by first adenylating its
Transmembrane Protein 144		
Shiftless Antiviral Inhibitor Of Ribosomal Fr		Inhibits programmed -1 ribosomal frame
Phosphatidylinositol 4-Kinase	Phosphatidyl	Membrane-bound phosphatidylinositol-4
Polypeptide N-Acetylgalactosa	This gene en	Catalyzes the initial reaction in O-linked
Autophagy And Beclin 1 Regulator 1		Regulates autophagy and development
Chromodomain Helicase DNA	This gene en	Probable transcription regulator. Maybe

BTB Domain Containing 2	The C-terminus of the protein encoded by this gene b	
VPS35 Retromer Complex Co	This gene be	(Microbial infection) The heterotrimeric r
Transmembrane Protein 30A		Accessory component of a P4-ATPase f
FYVE, RhoGEF And PH Domain Containin		May activate CDC42, a member of the F
ELL Associated Factor 2		Acts as a transcriptional transactivator o
Protocadherin Gamma Subfa	This gene is	Potential calcium-dependent cell-adhesi
Protocadherin Gamma Subfa	This gene is	Potential calcium-dependent cell-adhesi
SERTA Domain Containing 4		
Brain Expressed X-Linked 4	This gene is	May play a role in microtubule deacetyla
CTP Synthase 2	The protein e	Catalyzes the ATP-dependent aminatior
Carbohydrate Sulfotransferas	This gene is	Sulfotransferase that utilizes 3'-phospho
Formin 2	This gene is	Actin-binding protein that is involved in a
Follistatin Like 5		
1-Acylglycerol-3-Phosphate O	The protein e	Converts 1-acyl-sn-glycerol-3-phosphate
MAP3K7 C-Terminal Like		
Aryl Hydrocarbon Receptor N	This gene en	Transcriptional activator which forms a c
VPS35 Endosomal Protein Sorting Factor L		(Microbial infection) The heterotrimeric r
Charged Multivesicular Body I	CHMP1B bel	Probable peripherally associated compo
Arginyl Aminopeptidase Like 1		Broad specificity aminopeptidase which
Pellino E3 Ubiquitin Protein Ligase 1		E3 ubiquitin ligase catalyzing the covaler
Aspartate Beta-Hydroxylase Domain Conta		May function as 2-oxoglutarate-depende
Ral GTPase Activating Protein Catalytic Su		Catalytic subunit of the heterodimeric Ra
Reticulocalbin 3		Probable molecular chaperone assisting
SPC25 Component Of NDC8	This gene en	Acts as a component of the essential kir
Extended Synaptotagmin 2		Tethers the endoplasmic reticulum to the
HECT, C2 And WW Domain (	This gene en	E3 ubiquitin-protein ligase that mediates
Neutral Cholesterol Ester Hydrolase 1		Hydrolyzes 2-acetyl monoalkylglycerol e
Signal Induced Proliferation A	This gene encodes a member of the signal-induced p	
Dedicator Of Cytokinesis 6	This gene en	Acts as guanine nucleotide exchange fa
Protocadherin 10	This gene be	Potential calcium-dependent cell-adhesi
Cilia And Flagella Associated Protein 97		
Zinc Finger And BTB Domain Containing 4		Transcriptional repressor with bimodal D
Ectopic P-Granules Autophag	This gene en	Involved in autophagy. May play a role ir
Microtubule Affinity Regulating	This gene en	Serine/threonine-protein kinase (PubMe
C-X-C Motif Chemokine Ligand 16		Acts as a scavenger receptor on macrop
Talin Rod Domain Containing	This gene en	Actin-binding protein which may have ar
Calcium Voltage-Gated Chan	Voltage-depe	Regulates the activity of L-type calcium (
Pleckstrin Homology Domain	This gene en	Binds specifically to phosphatidylinositol

Solute Carrier Family 25 Mem	This gene en	Mitochondrial transporter mediating upta
Zinc Finger AN1-Type Containing 3		
Roundabout Guidance Recep	This gene is	Thought to be involved during neural de
CAMP Responsive Element B	This gene en	Transcription factor involved in unfolded
Fibronectin Type III Domain Containing 3B		May be a positive regulator of adipogene
Ceramide Kinase	CERK conve	Catalyzes specifically the phosphorylatic
Required For Meiotic Nuclear Division 5 Ho		Core component of the CTLH E3 ubiquit
Ubiquitin Specific Peptidase 4	Modification	Deubiquitinating enzyme that plays a rol
RAS Protein Activator Like 3	This gene be	Functions as a Ras GTPase-activating p
Transmembrane Protein 237	The protein e	Component of the transition zone in prim
KRI1 Homolog	This gene overlaps with the gene for cysteine endope	
Acetoacetyl-CoA Synthetase		Activates acetoacetate to acetoacetyl-C
GID Complex Subunit 4 Hom	The multiprot	Substrate-recognition subunit of the CTL
Transmembrane Protein 106C		
DNA Replication And Sister C	CHTF18 (MI	Loads PCNA onto primed templates reg
Basic Helix-Loop-Helix Family	This gene en	Transcriptional repressor involved in the
Sperm Associated Antigen 16	Cilia and flag	Necessary for sperm flagellar function. F
Chondroitin Polymerizing Factor		Has both beta-1,3-glucuronic acid and b
FAT Atypical Cadherin 4	The protein e	Cadherins are calcium-dependent cell a
Dynein Cytoplasmic 2 Heavy	This gene en	May function as a motor for intraflagellar
Chromosome 11 Open Reading Frame 80		[Isoform 3]: Component of a topoisomer
E2F Transcription Factor 8	This gene en	Atypical E2F transcription factor that par
Thrombospondin Type 1 Domain Containin		Promotes FBN1 matrix assembly. Atten
Family With Sequence Similarity 110 Member D		
Stearoyl-CoA Desaturase 5	Stearoyl-CoA	Stearyl-CoA desaturase that utilizes O(2
DSN1 Component Of MIS12	This gene en	Part of the MIS12 complex which is requ
Solute Carrier Family 8 Memb	SLC24A6 be	Mitochondrial sodium/calcium antiporter
MYC Target 1		May regulate certain MYC target genes,
Coiled-Coil Domain Containing 92		
WD Repeat Domain 26	This gene en	G-beta-like protein involved in cell signal
Phosphatidylinositol Glycan A	The glycosyl	Mannosyltransferase involved in glycosy
Phosphatidylinositol-3,4,5-Tris	The protein e	Functions as a RAC1 guanine nucleotid
Potassium Voltage-Gated Ch	This gene en	Regulatory subunit of Kv4/D (Shal)-type
Tweety Family Member 3	This gene en	Probable large-conductance Ca(2+)-acti
Collagen Type XVIII Alpha 1	This gene en	[Non-collagenous domain 1]: May regula
C-Maf Inducing Protein	This gene en	Plays a role in T-cell signaling pathway.
SH3 Binding Domain Protein 5 Like		Functions as guanine nucleotide exchan
Integrin Alpha FG-GAP Repeat Containing		Modulator of T-cell function. Has a prote

Glycerophosphodiester Phosp	Glycerophos	Glycerophosphodiester phosphodiester
F-Box Protein 38	This gene en	Substrate recognition component of a S
Tetraspanin 14		Regulates maturation and trafficking of t
Transcription Factor 7 Like 1	This gene en	Participates in the Wnt signaling pathwa
Plasmalemma Vesicle Associated Protein		Endothelial cell-specific membrane prot
Allograft Inflammatory Factor 1 Like		Actin-binding protein that promotes acti
MARVEL Domain Containing 1		Microtubule-associated protein that exhi
Armadillo Repeat Containing	This gene en	May play a role in cell survival and cell g
Transmembrane O-Mannosyltransferase T		Transfers mannosyl residues to the hyd
UDP-GlcNAc:BetaGal Beta-1,	This gene en	Beta-1,3-N-acetylglucosaminyltransfera
Meiotic Nuclear Divisions 1	The product	Required for proper homologous chrom
FAM161 Centrosomal Protein	This gene be	Involved in ciliogenesis.
Calpain Small Subunit 2		Calcium-regulated non-lysosomal thiol-p
Peroxiredoxin Like 2A		Involved in redox regulation of the cell (
Cysteine Rich Transmembrane Module Containing 1		
Caspase Recruitment Domain	The protein	Involved in the costimulatory signal esse
Zinc Finger Protein 528		May be involved in transcriptional regula
Minichromosome Maintenanc	The protein	Component of the MCM8-MCM9 compl
Par-6 Family Cell Polarity Regulator Gamm		Adapter protein involved in asymmetrica
Collagen Type XXV Alpha 1 C	This gene en	Inhibits fibrillization of amyloid-beta pept
GTP Binding Protein 3, Mitoc	This locus en	GTPase involved in the 5-carboxymethy
Pleckstrin Homology Domain Containing A		Cargo transport protein that is required f
Phospholipase C Delta 4	This gene en	[Isoform 2]: Acts as a non-receptor guan
4-Hydroxyphenylpyruvate Dioxygenase Lik		May have dioxygenase activity.
Transmembrane Protein 25		In neurons, modulates the degradation
R-Spondin 3	This gene be	Activator of the canonical Wnt signaling
Transmembrane O-Mannosyl	This gene en	Transfers mannosyl residues to the hyd
Zinc And Ring Finger 1	This gene en	E3 ubiquitin-protein ligase that mediates
Centrosomal Protein 19	The protein	Required for ciliation (PubMed:286255
Small Integral Membrane Protein 3		
Collagen Type XXVII Alpha 1	This gene en	Plays a role during the calcification of ca
Progestin And AdipoQ Receptor Family Me		Plasma membrane progesterone (P4) r
Helicase With Zinc Finger 2	The protein	Helicase that acts as a transcriptional c
Tankyrase 1 Binding Protein 1		
Neuron Navigator 2	This gene en	Possesses 3' to 5' helicase activity and
SEC11 Homolog C, Signal Peptidase Com		Component of the microsomal signal pe
Zinc Finger Protein 697		May be involved in transcriptional regula
Formin Like 3	The protein	Plays a role in the regulation of cell mor

Coiled-Coil Domain Containing 74B		
Sorting Nexin 29		
Zinc Finger Protein 585B		May be involved in transcriptional regula
TRAF Interacting Protein With	This gene en	Adapter molecule that plays a key role in
Family With Sequence Similar	The protein e	May play a role in neuronal cell develop
Coiled-Coil Domain Containing 102A		
Zinc Finger Protein 561		May be involved in transcriptional regula
Phospholipase C Delta 3	This gene en	Hydrolyzes the phosphatidylinositol 4,5-
Potassium Channel Tetramerization Domai		Auxiliary subunit of GABA-B receptors th
Protein-L-Isoaspartate (D-Aspartate) O-Methyltransferase Domain Containing 1		
FCH And Mu Domain Containing Endocytic		Functions in an early step of clathrin-me
Heat Shock Protein Family A	The protein encoded by this gene contains an atypica	
Solute Carrier Family 18 Mem	This gene encodes a protein, which has high sequenc	
Signal Peptide Peptidase Like 3		Intramembrane-cleaving aspartic protea
CKLF Like MARVEL Transme	This gene belongs to the chemokine-like factor gene	
Major Facilitator Superfamily Domain Containing 12		
Galactose Mutarotase	This gene en	Mutarotase that catalyzes the interconve
Discoidin, CUB And LCCL Domain Containing 2		
Glycerate Kinase	This locus encodes a member of the glycerate kinase	
Junction Mediating And Regulatory Protein		Acts both as a nuclear p53/TP53-cofact
Transmembrane Protein 171		
Peptidase M20 Domain Containing 2		
Spindlin Family Member 4		Exhibits H3K4me3-binding activity.
NIMA Related Kinase 7	NIMA-related	Protein kinase which plays an important
Mindbomb E3 Ubiquitin Protei	The protein e	E3 ubiquitin-protein ligase that mediates
Eukaryotic Translation Initiation Factor 3 Subunit L Pseudogene 2		
Low Density Lipoprotein Receptor Class A		May influence APP processing, resulting
ST13, Hsp70 Interacting Protein Pseudogene 5		
High Mobility Group AT-Hook 1 Pseudogene 3		
Magnesium Dependent Phosphatase 1		Magnesium-dependent phosphatase wh
Toll Like Receptor Adaptor M	This gene en	Involved in innate immunity against inva
Zinc Finger Protein 827		May be involved in transcriptional regula
Shisa Family Member 3	This gene en	Plays an essential role in the maturation
CREB3 Regulatory Factor		Acts as a negative regulator of the endo
CNKSR Family Member 3		Involved in transepithelial sodium transp
GTPase, IMAP Family Memb	This gene en	Exerts an anti-apoptotic effect in the imn
Transmembrane Protein 65		May play an important role in cardiac de
Ubiquitin Specific Peptidase 54		Has no peptidase activity.

Sprouty Related EVH1 Domai	The protein e	Tyrosine kinase substrate that inhibits gı
Zinc Finger Protein 846		May be involved in transcriptional regula
Zinc Finger Protein 579		May be involved in transcriptional regula
Family With Sequence Similarity 171 Member B		
Secretory Carrier Membrane Protein 5		Required for the calcium-dependent exo
Apolipoprotein B MRNA Editir	This gene is	DNA deaminase (cytidine deaminase) w
Sprouty Related EVH1 Domai	SPRED2 is a	Negatively regulates Ras signaling pathv
Centrosomal Protein 112	This gene encodes a coiled-coil domain containing pr	
Solute Carrier Family 25 Mem	This gene encodes a member of the mitochondrial ca	
Tripartite Motif Family Like 2	This gene encodes a member of the tri-partite motif (T	
Dehydrogenase/Reductase X-Linked		Involved in the positive regulation of star
Zinc Finger CCHC-Type Containing 24		
Solute Carrier Family 37 Member 2		Inorganic phosphate and glucose-6-pho
Zinc Finger Protein 438		Isoform 1 acts as a transcriptional repres
ZNF22 Antisense RNA 1		
Receptor Accessory Protein 3		Microtubule-binding protein required to e
Jumonji Domain Containing 1	The protein e	Probable histone demethylase that spec
Family With Sequence Similarity 171 Memb		Involved in the regulation of the cytoskel
Sidekick Cell Adhesion Molec	The protein e	Adhesion molecule that promotes lamina
Proline Rich 15		May have a role in proliferation and/or di
Ankyrin Repeat Domain 18A		
Zinc Finger DHHC-Type Palmitoyltransfera		Palmitoyltransferase that mediates palm
Ring Finger Protein 144B		E3 ubiquitin-protein ligase which accepts
Sorting Nexin 33	The protein e	Plays a role in the reorganization of the
Phosphoglucomutase 2 Like 1		Glucose 1,6-bisphosphate synthase usir
Otogelin Like	The protein encoded by this gene belongs to the otog	
Exocyst Complex Component 3 Like 1		As part of the exocyst, may play a role ir
Neuropeptide W	The product	Plays a regulatory role in the organizatio
Family With Sequence Similarity 171 Member A2		
Potassium Channel Tetrameri	This gene en	May repress the transcriptional activity o
WD Repeat Domain 62	This gene is	Required for cerebral cortical developme
Ring Finger Protein 149		E3 ubiquitin-protein ligase. Ubiquitinates
Sulfatase Modifying Factor 1	This gene en	Oxidase that catalyzes the conversion o
Proline Rich Transmembrane Protein 3		
Ring Finger Protein 180		E3 ubiquitin-protein ligase which promot
Protein Phosphatase, Mg2+/M	This gene encodes the serine/threonine protein phosp	
RAB43, Member RAS Oncogene Family		The small GTPases Rab are key regulat
Zinc Finger Protein 260		Transcription factor that acts as a cardia

Solute Carrier Family 35 Member E4		Putative transporter.
Phosphogluconate Dehydrogenase Pseudogene 1		
Heterogeneous Nuclear Ribonucleoprotein		May play a role in nucleosome assembly
Transmembrane Protein 255B		
Major Histocompatibility Complex, Class I, V (Pseudogene)		
Zinc Finger Protein 710		May be involved in transcriptional regula
Sterile Alpha Motif Domain Containing 5		
Ferric Chelate Reductase 1	Members of t	Ferric-chelate reductases reduce Fe(3+)
Chromosome 4 Open Reading Frame 48		
Long Intergenic Non-Protein Coding RNA 997		
Matrix Remodeling Associated 7		
Eukaryotic Translation Initiatio	This gene en	(Microbial infection) Plays a role in modu
Ankyrin Repeat Domain 18B		
NOTCH Regulated Ankyrin Repeat Protein		Downstream effector of Notch signaling.
Retrotransposon Gag Like 8B		
Acyl-CoA Thioesterase 1		Acyl-CoA thioesterases are a group of e
Small Integral Membrane Protein 15		
Microtubule Associated Protein 1 Light Cha		Ubiquitin-like modifier involved in format
Histidine Rich Carboxyl Terminus 1		
Phosphatidylethanolamine Binding Protein 1 Pseudogene 2		
LYPLA2 Pseudogene 1		
Modulator Of Smoothened		Acts as a negative regulator of hedgeho
RELT Like 1		Induces activation of MAPK14/p38 casca
SLC25A21 Antisense RNA 1		
Jumonji Domain Containing 7	This gene en	Bifunctional enzyme that acts both as an
MicroRNA 1204	microRNAs (miRNAs) are short (20-24 nt) non-coding	
HOXA Distal Transcript Antise	This gene produces a long RNA in antisense to the H	
PMF1-BGLAP Readthrough	This locus represents naturally occurring read-through	
Myocardial Zonula Adherens	This gene en	Plays a role in cellular signaling via Rho-
RNA, U6 Small Nuclear 301, Pseudogene		
Activin A Receptor Type 1B	This gene en	Transmembrane serine/threonine kinase
Acylphosphatase 1	This gene is	Its physiological role is not yet clear.
Adrenoceptor Alpha 1B	Alpha-1-adre	This alpha-adrenergic receptor mediates
Activated Leukocyte Cell Adh	This gene en	[Isoform 3]: Inhibits activities of membra
Aldehyde Dehydrogenase 1 F	The protein e	Can convert/oxidize retinaldehyde to reti
Aldolase, Fructose-Bisphosph	This gene encodes a member of the class I fructose-b	
Angiogenin	The protein e	Binds to actin on the surface of endothe
Amyloid Beta Precursor Prote	The protein e	Putative function in synaptic vesicle exo

ATPase H+ Transporting V0 S	This gene en	Proton-conducting pore forming subunit
Hyperpolarization Activated C	The protein e	Hyperpolarization-activated ion channel
Bone Morphogenetic Protein (	This gene en	Induces cartilage and bone formation.
BRCA1 DNA Repair Associat	This gene en	E3 ubiquitin-protein ligase that specifical
BRCA2 DNA Repair Associat	Inherited mu	Involved in double-strand break repair a
Carbonic Anhydrase 12	Carbonic anh	Reversible hydration of carbon dioxide.
Cbl Proto-Oncogene B	This gene en	E3 ubiquitin-protein ligase which accept
Cyclin A2	The protein e	Cyclin which controls both the G1/S and
ATP Binding Cassette Subfan	The protein e	Mediates hepatobiliary excretion of num
Deoxycytidine Kinase	Deoxycytidir	Phosphorylates the deoxyribonucleoside
Aldo-Keto Reductase Family	This gene en	Converts progesterone to its inactive for
Translocase Of Inner Mitocho	This transloc	Mitochondrial intermembrane chaperone
Dihydroorotate Dehydrogenas	The protein e	Catalyzes the conversion of dihydroorot
TRNA Aspartic Acid Methyltra	This gene en	Specifically methylates cytosine 38 in th
Epithelial Membrane Protein 1		
Epidermal Growth Factor Rec	This gene en	Signaling adapter that controls various c
FA Complementation Group (	The Fanconi	DNA repair protein that may operate in a
Galactosylceramidase	This gene en	Hydrolyzes the galactose ester bonds of
Growth Hormone Receptor	This gene en	Isoform 2 up-regulates the production of
Glycerol Kinase	The protein e	Key enzyme in the regulation of glycerol
Glutamate-Cysteine Ligase M	Glutamate-cysteine ligase, also known as gamma-glu	
Golgin A1	The Golgi ap	Involved in vesicular trafficking at the Go
Glutathione S-Transferase Al	Cytosolic and	Conjugation of reduced glutathione to a
H1.0 Linker Histone	Histones are	Histones H1 are necessary for the cond
H2A.X Variant Histone	Histones are	Variant histone H2A which replaces con
Hyaluronan Synthase 3	The protein e	Catalyzes the addition of GlcNAc or Glc
Hepatocyte Growth Factor	This gene en	Potent mitogen for mature parenchymal
High Mobility Group Box 2	This gene en	Multifunctional protein with various roles
High Mobility Group Nucleoso	The protein e	Binds to the inner side of the nucleosom
Homeobox D1	This gene is	Sequence-specific transcription factor w
Insulin Like Growth Factor 2	This gene en	Preptin undergoes glucose-mediated co
Immunoglobulin Mu DNA Binc	This gene en	5' to 3' helicase that unwinds RNA and D
Inhibin Subunit Beta A	This gene en	Inhibins and activins inhibit and activate,
Interferon Regulatory Factor 3	This gene en	Key transcriptional regulator of type I int
Potassium Calcium-Activated	MaxiK chann	Regulatory subunit of the calcium activa
Kinesin Family Member 11	This gene en	Motor protein required for establishing a
Kinesin Family Member C1		Minus end-directed microtubule-depend
Lipase E, Hormone Sensitive	The protein e	In adipose tissue and heart, it primarily h

Melanocortin 1 Receptor	This intronles	Receptor for MSH (alpha, beta and gam
Minichromosome Maintenanc	The protein e	Acts as component of the MCM2-7 com
Minichromosome Maintenanc	The protein e	Acts as component of the MCM2-7 com
Minichromosome Maintenanc	The protein e	Acts as component of the MCM2-7 com
Meis Homeobox 1	Homeobox g	Acts as a transcriptional regulator of PA
Microsomal Glutathione S-Tra	The MAPEG	Conjugation of reduced glutathione to a
Matrix Metallopeptidase 16	Proteins of th	Endopeptidase that degrades various co
Molybdenum Cofactor Synthe	Eukaryotic m	Acts as a sulfur carrier required for moly
Metallothionein 1X		Metallothioneins have a high content of
MutY DNA Glycosylase	This gene en	Involved in oxidative DNA damage repai
NADH:Ubiquinone Oxidoredu	The protein e	Accessory subunit of the mitochondrial r
NADH:Ubiquinone Oxidoreductase Subunit		Accessory subunit of the mitochondrial r
NADH:Ubiquinone Oxidoreductase Core Subunit V2 Pseudogene 1		
N-Ribosyldihydronicotinamide	This gene en	The enzyme apparently serves as a quir
Nitric Oxide Synthase 3	Nitric oxide is	[Isoform eNOS13C]: Lacks eNOS activit
Neuronal Cell Adhesion Mole	Cell adhesior	Cell adhesion protein that is required for
Contactin 3		Contactins mediate cell surface interacti
Pericentrin	The protein e	Integral component of the filamentous m
Phosphodiesterase 3A	This gene en	Cyclic nucleotide phosphodiesterase wit
Phosphoribosylformylglycinan	Purines are r	Phosphoribosylformylglycinamidine synt
ATP Binding Cassette Subfan	The membra	Translocates drugs and phospholipids a
Phosphorylase Kinase Regula	Phosphorylas	Phosphorylase b kinase catalyzes the pl
Phosphorylase Kinase Cataly	Phosphorylas	Catalytic subunit of the phosphorylase b
Phosphatidylinositol Glycan A	This gene en	Involved in GPI anchor biosynthesis (Pu
Phosphatidylinositol Glycan A	This gene en	Involved in GPI-anchor biosynthesis thro
Phosphatidylinositol Glycan A	This gene en	Part of the complex catalyzing the transf
Phospholipase C Like 1 (Inactive)		Involved in an inositol phospholipid-base
Phosphomannomutase 1	Phosphomar	Involved in the synthesis of the GDP-ma
Exosome Component 9	This gene en	Non-catalytic component of the RNA exo
Pinin, Desmosome Associated Protein		Transcriptional activator binding to the E
DNA Polymerase Epsilon 2, A	DNA polymer	Accessory component of the DNA polym
RNA Polymerase II Subunit G	This gene en	DNA-dependent RNA polymerase cataly
Proteasome 20S Subunit Alph	The proteaso	Component of the 20S core proteasome
RAD1 Checkpoint DNA Exonu	This gene en	Component of the 9-1-1 cell-cycle check
Peroxisomal Biogenesis Facto	This gene en	Somewhat implicated in the biogenesis
Rab Geranylgeranyltransferas	This gene en	Catalyzes the transfer of a geranylgeran
Replication Factor C Subunit	This gene en	The elongation of primed DNA template
Replication Factor C Subunit	The elongatio	The elongation of primed DNA template

Replication Factor C Subunit	The elongatio	The elongation of primed DNA templates
Replication Factor C Subunit	This gene en	The elongation of primed DNA templates
Small Nucleolar RNA, H/ACA Box 63		
Replication Protein A3		As part of the heterotrimeric replication
Ribosomal Protein S29	Ribosomes, the organelles that catalyze protein synth	
Ribonucleotide Reductase Re	This gene en	Provides the precursors necessary for D
Syndecan 2	The protein e	Cell surface proteoglycan that bears hep
Transformer 2 Beta Homolog	This gene en	Sequence-specific RNA-binding protein
Serum/Glucocorticoid Regula	This gene en	Serine/threonine-protein kinase which is
STIL Centriolar Assembly Pro	This gene en	Immediate-early gene. Plays an importa
S-Phase Kinase Associated F	This gene en	Substrate recognition component of a S
Solute Carrier Family 1 Memb	This gene en	Sodium-dependent, high-affinity amino a
Solute Carrier Family 1 Memb	The SLC1A5	(Microbial infection) Acts as a cell surfac
Synuclein Alpha	Alpha-synucl	Neuronal protein that plays several roles
Small Nuclear Ribonucleoprotein Polypepti		Involved in pre-mRNA splicing as compo
Small Nuclear Ribonucleopro	The protein e	Plays role in pre-mRNA splicing as core
Sperm Associated Antigen 4	The mammal	Involved in spermatogenesis. Required f
SH3 And Cysteine Rich Domain		Promotes expression of the ion channel
Heat Shock Protein Family A	The protein e	Has peptide-independent ATPase activit
Syntaxin 1A	This gene en	Plays an essential role in hormone and r
Suppressor Of Variegation 3-	This gene en	Histone methyltransferase that specifica
Transgelin	This gene en	Actin cross-linking/gelling protein (By sin
Transcription Factor 7	This gene en	Transcriptional activator involved in T-ce
Transcription Factor AP-4	Transcription	Transcription factor that activates both v
Transforming Growth Factor E	This locus er	Binds to TGF-beta. Could be involved in
Thrombospondin 1	The protein e	Adhesive glycoprotein that mediates cell
Toll Like Receptor 4	The protein e	Cooperates with LY96 and CD14 to mec
Thymopoietin	Through alte	May be involved in the structural organiz
DNA Topoisomerase III Alpha	This gene en	Releases the supercoiling and torsional
Thioredoxin Reductase 1	The protein e	Isoform 1 may possess glutaredoxin acti
Uridine Phosphorylase 1	This gene en	Catalyzes the reversible phosphorylytic
Ubiquitin Specific Peptidase 1	This gene en	Negative regulator of DNA damage repa
ZFP36 Ring Finger Protein		(Microbial infection) Negatively regulates
Zinc Finger Protein 18		May be involved in transcriptional regula
Zinc Finger And BTB Domain Containing 2		May be involved in transcriptional regula
ALMS1 Centrosome And Bas	This gene en	Involved in PCM1-dependent intracellula
Mal, T Cell Differentiation Pro	This gene encodes an element of the machinery for ra	
Hydroxysteroid 17-Beta Dehy	In mice, the	NAD-dependent 17-beta-hydroxysteroid

YEATS Domain Containing 4	The protein e	Chromatin reader component of the NuA
Solute Carrier Family 7 Member 5		The heterodimer with SLC3A2 functions
Chromatin Assembly Factor 1	Chromatin as	Complex that is thought to mediate chro
H2A Clustered Histone 20	Histones are	Core component of nucleosome. Nucleo
HIRA Interacting Protein 3	The HIRA pro	May play a role in chromatin function an
Regulator Of G Protein Signal	This gene en	Inhibits signal transduction by increasing
Ficolin 3	Ficolins are a	May function in innate immunity through
Phospholipid Phosphatase 1	The protein e	Magnesium-independent phospholipid p
Stanniocalcin 2	This gene en	Has an anti-hypocalcemic action on calc
Jrk Helix-Turn-Helix Protein	This gene en	May bind DNA.
Fucose-1-Phosphate Guanyly	L-fucose is a	Catalyzes the formation of GDP-L-fucos
TNF Receptor Superfamily Me	The protein e	Receptor for TNFSF11/RANKL/TRANCE
Interleukin 18 Receptor 1	The protein e	Within the IL18 receptor complex, respo
Solute Carrier Family 5 Member 6		Transports pantothenate, biotin and lipo
Timeless Circadian Regulator	The protein e	Plays an important role in the control of
Small Nucleolar RNA, C/D Box 73A		
Ribosomal RNA Processing 9	This gene en	Component of a nucleolar small nuclear
Estrogen Receptor Binding Si	This gene wa	May participate in suppression of cell pro
Serine/Threonine Kinase 17b		Phosphorylates myosin light chains (By s
SLC9A3 Regulator 2	This gene en	Scaffold protein that connects plasma m
Stomatin Like 1		May play a role in cholesterol transfer to
ATPase H+ Transporting V1 S	This gene en	Catalytic subunit of the peripheral V1 co
Mitochondrial Translation Rele	The protein e	Mitochondrial peptide chain release facto
Tubulin Tyrosine Ligase Like 4		Glutamylase which preferentially modifie
Kinetochore Associated 1	This gene en	Essential component of the mitotic chec
Centriolar Coiled-Coil Protein 110		Necessary for centrosome duplication at
KIAA0513		
DnaJ Heat Shock Protein Fan	DNAJC6 belo	Recruits HSPA8/HSC70 to clathrin-coate
NUAK Family Kinase 1		Serine/threonine-protein kinase involved
Telomere Maintenance 2	This gene en	Regulator of the DNA damage response
DENN Domain Containing 4B		Guanine nucleotide exchange factor (GE
DNA Cross-Link Repair 1A	This gene en	May be required for DNA interstrand cro
Ring-Box 1	This locus en	E3 ubiquitin ligase component of multiple
Histone Deacetylase 6	Histones play	Responsible for the deacetylation of lysi
Chromatin Assembly Factor 1	Chromatin as	Core component of the CAF-1 complex,
ATP Binding Cassette Subfan	This gene en	Binds heme and porphyrins and functior
Nuclear Receptor Subfamily 1	The protein e	Nuclear receptor that exhibits a ligand-de
Cytochrome C Oxidase Coppe	Cytochrome c	Copper metallochaperone essential for t

Interleukin 18 Binding Protein	The protein e	Isoform A binds to IL-18 and inhibits its a
Tetraspanin 2	The protein e	May play a role in signalling in oligodenc
FRY Microtubule Binding Protein		Plays a crucial role in the structural integ
Family With Sequence Similarity 13 Member A		
Lysophosphatidylcholine Acyltransferase 3		Acyltransferase which mediates the conv
Ring Finger Protein 41	This gene en	Acts as E3 ubiquitin-protein ligase and re
Protein Arginine Methyltransfe	This gene be	Methylates (mono and asymmetric dime
M-Phase Phosphoprotein 9		
Flotillin 1	This gene en	May act as a scaffolding protein within ca
DExD-Box Helicase 39A	This gene en	[Isoform 1]: Involved in pre-mRNA splicin
Solute Carrier Family 35 Mem	This gene en	Probable sugar transporter.
Deleted In Lymphocytic Leukemia 1		May act as a tumor suppressor.
Amyloid Beta Precursor Prote	The protein e	May modulate the internalization of amy
T Cell Immune Regulator 1, A	This gene en	Part of the proton channel of V-ATPases
Anaphase Promoting Comple	ANAPC10 is	Component of the anaphase promoting
EMG1 N1-Specific Pseudouri	This gene en	S-adenosyl-L-methionine-dependent pse
Transforming Acidic Coiled-Co	This gene en	Plays a role in the microtubule-depende
NOP56 Ribonucleoprotein	Nop56p is a y	Involved in the early to middle stages of
SIVA1 Apoptosis Inducing Fa	This gene en	Induces CD27-mediated apoptosis. Inhib
Sperm Associated Antigen 5	This gene en	Essential component of the mitotic spinc
TATA-Box Binding Protein As	Initiation of tr	Functions as a component of the PCAF
Periostin	This gene en	Induces cell attachment and spreading a
DNA Polymerase Delta 3, Acc	This gene en	Accessory component of both the DNA p
Polo Like Kinase 4	This gene en	Serine/threonine-protein kinase that play
Retinoic Acid Induced 2	Retinoic acid	plays a critical role in development, cellu
IlvB Acetolactate Synthase Lik	The protein e	Endoplasmic reticulum 2-OH acyl-CoA ly
Stonin 1	Endocytosis c	May be involved in the endocytic machin
Cytochrome B561 Family Member D2		Two-heme-containing cytochrome that c
ZW10 Interacting Kinetochore	This gene en	Part of the MIS12 complex, which is requ
Lysine Acetyltransferase 7	The protein e	Catalytic subunit of histone acetyltransfe
DNA Meiotic Recombinase 1	This gene en	May participate in meiotic recombination
FIC Domain Protein Adenylyltransferase		Protein that can both mediate the additio
LSM6 Homolog, U6 Small Nu	Sm-like prote	Plays role in pre-mRNA splicing as comp
Polynucleotide Kinase 3'-Phos	This locus re	Plays a key role in the repair of DNA dar
Opa Interacting Protein 5	The protein e	Required for recruitment of CENPA to ce
Exosome Component 8	This gene en	Non-catalytic component of the RNA exo
BTB Domain Containing 3		Acts as a key regulator of dendritic field
Dickkopf WNT Signaling Path	This gene en	Antagonizes canonical Wnt signaling by

PAX Interacting Protein 1	This gene is	Involved in DNA damage response and
Calmodulin Binding Transcrip	The protein e	Transcription activator. May act as tumo
Sulfatase 1	This gene en	Exhibits arylsulfatase activity and highly
Spectrin Repeat Containing N	The protein e	Multi-isomeric modular protein which for
DnaJ Heat Shock Protein Family (Hsp40) M		May play a role as co-chaperone of the I
Non-SMC Condensin I Compl	This gene en	Regulatory subunit of the condensin con
Exosome Component 2		Non-catalytic component of the RNA exo
Integrin Subunit Beta 3 Bindin	This gene en	Transcription coregulator that can have I
Solute Carrier Family 44 Member 1		Choline transporter. May be involved in r
ATP Binding Cassette Subfan	The membra	Probable transporter which may play a r
Decaprenyl Diphosphate Synt	The protein e	Supplies decaprenyl diphosphate, the pr
Single Stranded DNA Binding	This gene encodes a subunit of a protein complex tha	
LSM5 Homolog, U6 Small Nu	Sm-like prote	Plays role in pre-mRNA splicing as comp
Stromal Cell Derived Factor 2 Like 1		
Interleukin 17 Receptor A	Interleukin 17	Receptor for IL17A (PubMed:17911633,
Claudin 15	This gene en	Claudins function as major constituents
Rhomboid Domain Containing 3		
Anti-Silencing Function 1A His	This gene en	Histone chaperone that facilitates histon
Mitochondrial Pyruvate Carrier 2		Mediates the uptake of pyruvate into mit
Zinc Finger Protein 473	This gene en	Involved in histone 3'-end pre-mRNA pro
Anaphase Promoting Complex Subunit 15		Component of the anaphase promoting
Acyl-CoA Thioesterase 11	This gene en	Has an acyl-CoA thioesterase activity wi
F-Box And WD Repeat Domain Containing 4 Pseudogene 1		
Family With Sequence Similarity 162 Memb		Proposed to be involved in regulation of
Translocase Of Inner Mitocho	This gene en	Probable mitochondrial intermembrane c
Small Nucleolar RNA, C/D Box 60		
TRNA Methyltransferase 2 Ho	The protein e	May be involved in nucleic acid metaboli
MDM2 Binding Protein	This gene en	Inhibits cell migration in vitro and suppre
Cell Death Inducing DFFA Like Effector B		Activates apoptosis.
Sulfotransferase Family 1B M	Sulfotransfer	Sulfotransferase that utilizes 3'-phospho
Transmembrane Protein 97	TMEM97 is a	Intracellular orphan receptor that binds r
Tribbles Pseudokinase 2	This gene en	Interacts with MAPK kinases and regula
Mitochondrial Ribosomal Prot	Mammalian mitochondrial ribosomal proteins are enco	
MAGE Family Member H1	This gene belongs to the non-CT (non cancer/testis) s	
Asteroid Homolog 1		Possible role in EGF receptor signaling.
Mitochondrial Ribosomal Prot	Mammalian mitochondrial ribosomal proteins are enco	
ATPase Family AAA Domain	A large family	May be a transcriptional coactivator of th
NADH:Ubiquinone Oxidoredu	NADH:ubiqui	Involved in the assembly of mitochondria

Translocase Of Inner Mitochondrial Membr		Participates in the translocation of transi
Transmembrane Protein 208	This gene en	May function as a negative regulator of
N-6 Adenine-Specific DNA Me	This gene en	Methyltransferase that can methylate bo
Non-SMC Condensin II Comp	This gene en	Regulatory subunit of the condensin-2 c
ALG5 Dolichyl-Phosphate Be	This gene encodes a member of the glycosyltransfera	
PSMC3 Interacting Protein	This gene en	Plays an important role in meiotic recom
GDP-Mannose Pyrophosphor	This gene is	Catalyzes the formation of GDP-mannos
ALG6 Alpha-1,3-Glucosyltran	This gene en	Adds the first glucose residue to the lipi
DNA Replication Fork Stabiliz	This gene lie	Replisome component that maintains ge
Solute Carrier Family 2 Memb	This gene be	Insulin-regulated facilitative hexose tran
Paired Immunoglobin Like Ty	Cell signalin	(Microbial infection) Acts as an entry co-
CXXC Finger Protein 1	This gene en	Transcriptional activator that exhibits a
5', 3'-Nucleotidase, Cytosolic	This gene en	Dephosphorylates the 5' and 2'(3')-phos
RNA Polymerase I Subunit H	This gene en	DNA-dependent RNA polymerase cataly
Mitochondrial Transcription Termination Fa		Binds promoter DNA and regulates initia
TP53RK Binding Protein		Component of the EKC/KEOPS comple
Mediator Complex Subunit 31		Component of the Mediator complex, a
Ribosomal RNA Processing 1	This gene encodes a protein that co-purifies with hum	
Mitochondrial Ribosomal Prot	Mammalian mitochondrial ribosomal proteins are enc	
Deoxyribose-Phosphate Aldolase		Catalyzes a reversible aldol reaction bet
Tubulin Epsilon 1	This gene encodes a member of the tubulin superfam	
Heat Shock Protein Family A (Hsp70) Mem		Component of the ribosome-associated
Chemokine Like Factor	The product	[Isoform 1]: has chemotactic response in
Nucleolar And Spindle Associ	NUSAP1 is a	Microtubule-associated protein with the
Ankyrin Repeat Domain 39		
Cytochrome C Oxidase Assembly Factor C		Required for the assembly of the mitoch
Ankyrin Repeat And MYND Domain Containing 1		
Cell Cycle Exit And Neuronal	The protein e	Involved in neuronal differentiation.
Thioesterase Superfamily Member 6		
POP5 Homolog, Ribonuclease P/MRP Sub		Component of ribonuclease P, a protein
Proteasome Maturation Prote	The protein e	Molecular chaperone essential for the as
NOP16 Nucleolar Protein	This gene encodes a protein that is localized to the nu	
NCK Interacting Protein With	The protein e	Has an important role in stress fiber for
JNK1/MAPK8 Associated Membrane Prote		May be a regulator of the duration of MA
PHD Finger Protein 7	Spermatoger	May play a role in spermatogenesis.
Mitochondrial Fission Process	MTP18 is a n	Involved in the mitochondrial division pro
Serrate, RNA Effector Molecule		Acts as a mediator between the cap-bin
Peptidylprolyl Isomerase Like	This gene is	Involved in pre-mRNA splicing as compo

Mitochondrial Pyruvate Carrie	The protein e	Mediates the uptake of pyruvate into mit
Heat Shock Protein Family B (Small) Memb		Component of the IFT complex B require
RNA Polymerase III Subunit K	This gene en	DNA-dependent RNA polymerase cataly
Peptidylprolyl Isomerase Like	This gene en	PPIases accelerate the folding of protein
MIS18 Kinetochore Protein A		Required for recruitment of CENPA to ce
Cytochrome C, Somatic	This gene en	Electron carrier protein. The oxidized for
Ganglioside Induced Different	This gene en	Regulates the mitochondrial network by
Solute Carrier Family 38 Member 2		Functions as a sodium-dependent amino
Asparagine Synthetase Domain Containing 1		
Nedd4 Family Interacting Protein 2		Activates HECT domain-containing E3 u
Abelson Helper Integration Sit	This gene is a	Involved in vesicle trafficking and require
Zinc Finger CCHC-Type Containing 10		
ERCC Excision Repair 6 Like,	This gene en	DNA helicase that acts as an essential c
Ankyrin Repeat Domain 49		Induces HBG1 expression (PubMed:161
Myotubularin Related Protein 10		
Semaphorin 4C		Cell surface receptor for PLXNB2 that pl
Odr-4 GPCR Localization Factor Homolog		May play a role in the trafficking of a sub
TIMELESS Interacting Protein	The protein e	Plays an important role in the control of
Chromosome 1 Open Reading Frame 159		
Mago Homolog B, Exon Junction Complex		Required for pre-mRNA splicing as comp
Fidgetin, Microtubule Severing Factor		ATP-dependent microtubule severing pr
Misato Mitochondrial Distribution And Morp		Involved in the regulation of mitochondri
Centromere Protein Q	CENPQ is a s	Component of the CENPA-CAD (nucleo
Lines Homolog 1	The Drosophila segment polarity gene lin encodes a p	
FA Complementation Group I	The Fanconi	Plays an essential role in the repair of D
Leucine Rich Repeat Containing 1		
Ring Finger Protein 121	The protein encoded by this gene contains a RING fin	
Meiosis Specific Nuclear Stru	This gene en	May play a role in the control of meiotic c
Zinc Finger Protein 823		May be involved in transcriptional regula
FAD Dependent Oxidoreducta	This gene en	Required for the assembly of the mitoch
Syntabulin	Syntabulin/G	Part of a kinesin motor-adapter complex
LIM Zinc Finger Domain Conta	This gene en	Adapter protein in a cytoplasmic comple
TRNA 5-Methylaminomethyl-2	This nuclear	Catalyzes the 2-thiolation of uridine at th
Centrosomal Protein 72	The product o	Involved in the recruitment of key centro
Spermatogenesis Associated	This gene, or	Involved in the maintenance of both rod
Centromere Protein J	This gene en	Plays an important role in cell division ar
PDZ Binding Kinase	This gene en	Phosphorylates MAP kinase p38. Seems
Nuclear Transport Factor 2 Lik	The protein e	Regulator of protein export for NES-cont

Glycerophosphocholine Phosphodiesterase		May be involved in the negative regulatio
Peter Pan Homolog	The protein e	May have a role in cell growth.
Pannexin 2	The protein e	Structural component of the gap junction
Semaphorin 3G	The transcrip	Has chemorepulsive activities for sympa
Dihydrouridine Synthase 3 Like		Catalyzes the synthesis of dihydrouridine
Single-Pass Membrane Protein With Coiled-Coil Domains 4		
Short Chain Dehydrogenase/I	This gene en	Putative NADP-dependent oxidoreducta
Ral Guanine Nucleotide Dissociation Stimu		Guanine nucleotide exchange factor (GE
Putative Homeodomain Transcription Facto		May play a role in transcription regulatio
Dolichyldiphosphatase 1	A similar gen	Required for efficient N-glycosylation. Ne
Chromosome 3 Open Reading Frame 14		
STAM Binding Protein Like 1		Zinc metalloprotease that specifically cle
Centrosomal Protein 126		Participates in cytokinesis (PubMed:197
Phosphatidylinositol-3,4,5-Tris	The protein e	Functions as a RAC guanine nucleotide
Stromal Interaction Molecule 2	This gene is a	Plays a role in mediating store-operated
UV Stimulated Scaffold Protei	The protein e	Factor involved in transcription-coupled
DEAD-Box Helicase 55	This gene en	Probable ATP-binding RNA helicase.
LSM2 Homolog, U6 Small Nu	This gene en	Plays role in pre-mRNA splicing as comp
SRP Receptor Subunit Beta	The protein e	Component of the SRP (signal recognitio
Phosphatidylcholine Transfer Protein		Catalyzes the transfer of phosphatidylch
Zinc Finger Protein 77		May be involved in transcriptional regula
NGG1 Interacting Factor 3 Like 1		May function as a transcriptional corepre
Short Coiled-Coil Protein	This gene en	Positive regulator of amino acid starvatio
N-Terminal EF-Hand Calcium	The protein e	Inhibits the interaction of APBA2 with an
FKBP Prolyl Isomerase Like	The protein e	May be involved in response to X-ray. R
Claspin	The product o	Required for checkpoint mediated cell cy
Fidgetin Like 1	This gene en	Involved in DNA double-strand break (D
Centromere Protein K	CENPK is a s	Component of the CENPA-CAD (nucleo
Non-SMC Condensin I Compl	This gene en	Regulatory subunit of the condensin con
Torsin Family 3 Member A		
IKAROS Family Zinc Finger 4	Members of t	DNA-binding protein that binds to the 5'C
Autophagy Related 3	This gene en	E2 conjugating enzyme required for the
Methyltransferase Like 17		May be a component of the mitochondria
GINS Complex Subunit 3	This gene en	The GINS complex plays an essential ro
Nucleolar Protein 6	The nucleolus is a dense subnuclear membraneless c	
Homologous Recombination Factor With O		DNA-binding protein involved in homolog
Aurora Kinase A And Ninein Interacting Pro		DNA-binding protein that accumulates a
Centromere Protein M	The protein e	Component of the CENPA-NAC (nucleo

ELOVL Fatty Acid Elongase 6	Fatty acid elc	Catalyzes the first and rate-limiting react
Coiled-Coil-Helix-Coiled-Coil-Helix Domain Containing 7		
Dehydrogenase/Reductase 11		Catalyzes the conversion of the 17-keto
Centromere Protein O	This gene en	Component of the CENPA-CAD (nucleo
Cysteine Rich With EGF Like Domains 2		Protein disulfide isomerase (By similarity
THO Complex 6	This gene en	Acts as component of the THO subcomp
HAUS Augmin Like Complex :	This gene en	Contributes to mitotic spindle assembly,
PAXIP1 Associated Glutamat	C16ORF53 (	Its association with the histone methyltra
N-Alpha-Acetyltransferase 16, NatA Auxilia		Auxillary subunit of the N-terminal acetyl
Polypeptide N-Acetylgalactos	This gene en	Catalyzes the initial reaction in O-linked
Chromosome 22 Open Reading Frame 46		
Centromere Protein U	The centrom	Component of the CENPA-NAC (nucleo
YrdC N6-Threonylcarbamoyltransferase Dc		May regulate the activity of some transp
Suppressor Of Variegation 3-9 Homolog 2		Histone methyltransferase that specifica
SH3 Domain Containing 21		
Transcription Elongation Factor, Mitochond		Transcription elongation factor which inc
Gem Nuclear Organelle Asso	The protein e	The SMN complex plays a catalyst role i
Gem Nuclear Organelle Asso	GEMIN6 is p	The SMN complex plays a catalyst role i
Centrosome And Spindle Pole	This gene en	May play a role in cell-cycle-dependent r
Ribonuclease P/MRP Subunit	RPP21 is a p	Component of ribonuclease P, a ribonuc
L-2-Hydroxyglutarate Dehydrc	This gene encodes L-2-hydroxyglutarate dehydrogena	
Jade Family PHD Finger 1		Scaffold subunit of some HBO1 comple
RecQ Mediated Genome Insta	RMI1 is a co	Essential component of the RMI comple
Semaphorin 6D	Semaphorins	Shows growth cone collapsing activity o
UL16 Binding Protein 2	This gene en	Binds and activates the KLRK1/NKG2D
Solute Carrier Family 35 Member G2		May play a role in cell proliferation.
THAP Domain Containing 7		Chromatin-associated, histone tail-bindi
Acidic Nuclear Phosphoprotein 32 Family M		Histone chaperone that specifically med
Solute Carrier Family 25 Member 28		Mitochondrial iron transporter that media
Aldo-Keto Reductase Family '	The protein e	Catalyzes the NADPH-dependent reduc
BRCA1 Interacting Protein C-'	The protein e	DNA-dependent ATPase and 5' to 3' DN
Solute Carrier Family 10 Member 7		Involved in teeth and skeletal developme
ATR Interacting Protein	This gene en	Required for checkpoint signaling after D
UTP15 Small Subunit Processome Compo		Ribosome biogenesis factor. Involved in
Cytosolic Iron-Sulfur Assembly Component		Component of the cytosolic iron-sulfur p
Protein O-Mannose Kinase	This gene en	Protein O-mannose kinase that specifica
Transmembrane Protein 126A	The protein encoded by this gene is a mitochondrial m	
Zinc Finger CCHC-Type Containing 9		May down-regulate transcription mediate

SMC5-SMC6 Complex Localization Factor		Plays a role in the DNA damage respons
Eukaryotic Translation Initiation Factor 1A		Plays a role into cellular response to oxi
Transmembrane Protein 175		Organelle-specific potassium channel s
Ubiquinol-Cytochrome C Red	This gene en	Required for the assembly of the ubiqui
Cms1 Ribosomal Small Subunit Homolog		
DOT1 Like Histone Lysine Me	The protein	Histone methyltransferase. Methylates 'L
SLX4 Structure-Specific Endo	This gene en	Regulatory subunit that interacts with an
Kelch Repeat And BTB Domain Containing		Substrate-specific adapter of a BCR (BT
Methylmalonyl-CoA Epimeras	The product	Methylmalonyl-CoA epimerase involved
MPV17 Mitochondrial Inner Membrane Pro		Required for the assembly and stability
DEAD/H-Box Helicase 11 Like 2 (Pseudogene)		
Long Intergenic Non-Protein Coding RNA 467		
Glycerol-3-Phosphate Acyltra	This gene en	Converts glycerol-3-phosphate to 1-acyl
Protein Phosphatase 1 Regul	This gene en	Maintains low levels of EIF2S1 phospho
ALG10 Alpha-1,2-Glucosyltra	This gene en	Adds the third glucose residue to the lipi
Transmembrane Protein 128		
Leucine Rich Repeat And Coi	This gene en	Required for the organization of the mitc
DIX Domain Containing 1	The protein	Positive effector of the Wnt signaling pa
Centrosomal Protein 295		Centriole-enriched microtubule-binding
Keratin 8 Pseudogene 12		
Kelch Like Family Member 13	This gene en	Substrate-specific adapter of a BCR (BT
DDB1 And CUL4 Associated Factor 15		Binding of aryl sulfonamide anticancer d
Mediator Complex Subunit 30	The multipro	Component of the Mediator complex, a
LYR Motif Containing 7	Inner mitoch	Assembly factor required for Rieske Fe-
Zinc Finger AN1-Type Containing 2A		
Family With Sequence Similarity 104 Member B		
Centrosomal Protein 95		
Tripartite Motif Containing 41	This gene en	Functions as an E3 ligase that catalyzes
Chromosome 12 Open Reading Frame 29		
Plexin A4		Coreceptor for SEMA3A. Necessary for
Lin-52 DREAM MuvB Core Complex Component		
Ferredoxin-Fold Anticodon Bi	This gene encodes a protein which contains a ferredo	
Transmembrane Protein 88		Inhibits the Wnt/beta-catenin signaling p
LMBR1 Domain Containing 2		Recruited to ligand-activated beta-2 adre
Proline Rich 29		
Methionyl-TRNA Synthetase	This gene produces a mitochondrial methionyl-tRNA s	
HAUS Augmin Like Complex	HAUS8 is 1	Contributes to mitotic spindle assembly,
Trans-L-3-Hydroxyproline De	The protein	Catalyzes the dehydration of trans-3-hyd

Mitochondrial Fission Regulator 2		May play a role in mitochondrial aerobic
Cell Division Cycle Associated 5		Regulator of sister chromatid cohesion ir
Transmembrane Protein 106A		Activates macrophages and polarizes th
Target Of EGR1, Exonuclease		Inhibits cell growth rate and cell cycle. In
Solute Carrier Family 25 Mem	The protein e	Calcium-dependent mitochondrial solute
Serine/Threonine Kinase 11 Interacting Pro		May regulate STK11/LKB1 function by c
Transmembrane Protein 123	This gene en	Implicated in oncotic cell death, characte
TLC Domain Containing 1		Regulates the composition and fluidity o
Centrobin, Centriole Duplicati	This gene en	Required for centriole duplication. Inhibit
Sideroflexin 2		Mitochondrial amino-acid transporter tha
Zinc Finger Protein 641		Transcriptional activator. Activates trans
AlkB Homolog 2, Alpha-Ketog	The Escheric	Dioxygenase that repairs alkylated DNA
Leucine Rich Repeat Protein	The protein e	May negatively regulate the 4-1BB-medi
Centrosomal Protein 20		Involved in the biogenesis of cilia (PubM
Spermatogenesis Associated 33		
Chromosome 17 Open Reading Frame 49		Component of chromatin complexes suc
Src Homology 2 Domain Containing E		
Fc Receptor Like B	FCRL2 belongs to the Fc receptor family. Fc receptors	
Rho Guanine Nucleotide Exch	Guanine nuc	Acts as guanine nucleotide exchange fa
Nucleoporin 35	This gene en	Functions as a component of the nuclea
Chromosome 2 Open Reading Frame 76		
Tetratricopeptide Repeat Domain 32		
Zinc Finger AN1-Type Contair	This gene en	Plays a role in protein homeostasis by re
Coiled-Coil Domain Containing 58		
Chromosome 4 Open Reading Frame 33		
POC5 Centriolar Protein		Essential for the assembly of the distal h
Zinc Finger Protein 280B	The protein e	May function as a transcription factor.
HECT Domain E3 Ubiquitin Protein Ligase		Probable E3 ubiquitin-protein ligase whic
Mirror-Image Polydactyly 1	This gene encodes a coiled-coil domain-containing pr	
Essential Meiotic Structure-Sp	This gene en	Interacts with MUS81 to form a DNA stru
Zinc Finger Protein 534		May be involved in transcriptional regula
SPC24 Component Of NDC80 Kinetochore		Acts as a component of the essential kir
Dpy-19 Like C-Mannosyltransferase 3		Probable C-mannosyltransferase that m
Solute Carrier Family 66 Member 1 Like		
Nuclear Transcription Factor, X-Box Binding Like 1		
Metallo-Beta-Lactamase Domain Containing 2		
MARVEL Domain Containing	The protein e	Plays a role in the formation of tricellular
Retinoic Acid Early Transcript	RAET1L belc	Binds and activates the KLRK1/NKG2D

Coiled-Coil Domain Containing 167		
Potassium Channel Tetrameri	This gene en	May be involved in the control of excitab
Family With Sequence Similarity 201 Member A		
Solute Carrier Family 35 Mem	This gene en	May play a role in intracellular calcium s
C-Type Lectin Domain Contai	This gene encodes a member of the C-type lectin/C-ty	
PiggyBac Transposable Elem	The piggyBac family of proteins, found in diverse anim	
Zinc Finger Protein 114		May be involved in transcriptional regula
SAS-6 Centriolar Assembly P	The protein e	Central scaffolding component of the ce
Coiled-Coil Domain Containing 138		
THAP Domain Containing 5		Has sequence-specific DNA-binding acti
GTPase, IMAP Family Membe	This gene en	The dimer has GTPase activity; the activ
Basic Helix-Loop-Helix Family Member A15		Plays a role in controlling the transcriptio
Zinc Finger Protein 596		May be involved in transcriptional regula
ALG14 UDP-N-Acetylglucosa	This gene is	May be involved in protein N-glycosylatio
Transmembrane Protein 201		[Isoform 2]: May define a distinct membr
Potassium Channel Tetramerization Domai		Probable substrate-specific adapter of a
Chromosome 4 Open Readin	This gene encodes a small, conserved protein of unkn	
Transmembrane Protein 218		May be involved in ciliary biogenesis or f
Two Pore Segment Channel 2	This gene en	Nicotinic acid adenine dinucleotide phos
Spindle And Kinetochore Associated Comp		Component of the SKA1 complex, a mic
Spindle And Kinetochore Ass	This gene en	Component of the SKA1 complex, a mic
Chromosome 6 Open Reading Frame 136		
Maturin, Neural Progenitor Differentiation R		Promotes megakaryocyte differentiation
Semaphorin 3D	This gene en	Induces the collapse and paralysis of ne
G-Patch Domain Containing 11		
Ceramide Synthase 6		Ceramide synthase that catalyzes forma
Bromodomain And WD Repea	The protein e	Plays a role in the regulation of cell mor
ST6 N-Acetylgalactosaminide	ST6GALNAC	Involved in the biosynthesis of gangliosi
Membrane Associated Guanylate Kinase, W		Acts as a scaffolding protein at cell-cell j
Nephrocystin 4	This gene en	Involved in the organization of apical jun
Fucose Mutarotase		Involved in the interconversion between
Tubulin Epsilon And Delta Complex 1		Acts as a positive regulator of ciliary hed
TRNA Splicing Endonuclease	This gene en	Non-catalytic subunit of the tRNA-splicin
Chromosome 3 Open Reading Frame 70		May play a role in neuronal and neurobe
Long Intergenic Non-Protein Coding RNA 632		
Chromosome 1 Open Reading Frame 174		
Brain Expressed X-Linked 5		
Kinesin Family Member 24	This gene en	Microtubule-dependent motor protein tha

G Protein Subunit Gamma 5 Pseudogene 2		
Cytosolic Thiouridylase Subur	This gene en	Plays a central role in 2-thiolation of mcr
Zinc Finger Protein 530		May be involved in transcriptional regula
GEN1 Holliday Junction 5' Fla	This gene en	Endonuclease which resolves Holliday ju
Retinoic Acid Early Transcript	This gene en	[Isoform 1]: Binds and activates the KLR
WASP Family Homolog 3, Pseudogene		Acts as a nucleation-promoting factor at
Chromosome 5 Open Reading Frame 34		
Ring Finger Protein 148		
C-Type Lectin Domain Family 12 Member E		Cell surface receptor that protects target
Zinc Finger Protein 324B		May be involved in transcriptional regula
LysM Domain Containing 1		
Small Integral Membrane Protein 26		
Solute Carrier Family 25 Mem	SLC25A35 belongs to the SLC25 family of mitochond	
Translocase Of Outer Mitochondrial Membrane 5		
Centromere Protein P	CENPP is a s	Component of the CENPA-CAD (nucleo
WAS Protein Homolog Associated With Actin, Golgi Membranes And Microtubules F		
TRNA-YW Synthesizing Prote	Wybutosine i	Probable component of the wybutosine I
CDGSH Iron Sulfur Domain 2	The protein e	Regulator of autophagy that contributes
ChaC Glutathione Specific Ga	The protein e	Catalyzes the cleavage of glutathione int
Double Homeobox A Pseudog	Homeobox genes encode DNA-binding proteins, man	
Small Nucleolar RNA, C/D Bo	Small nucleolar RNAs (snoRNAs) of the C/D class, su	
Ribosomal Protein L26 Pseudogene 30		
WASP Family Homolog 7, Pseudogene		
Small Nucleolar RNA, H/ACA Box 20		
High Mobility Group Nucleosomal Binding Domain 2 Pseudogene 5		
D-2-Hydroxyglutarate Dehydro	This gene en	Catalyzes the oxidation of D-2-hydroxyg
High Mobility Group Box 2 Pseudogene 1		
Small Nucleolar RNA Host Gene 9		
KTN1 Antisense RNA 1		
Coiled-Coil Domain Containing 152		
Chromosome 11 Open Reading Frame 91		
Family With Sequence Similarity 27 Member E3		
Zinc Finger Protein 587B		May be involved in transcriptional regula
Transportin 1 Pseudogene 1		
COX20 Pseudogene 1		
MHC Class I Polypeptide-Rela	This gene en	Seems to have no role in antigen preser
Endogenous Retrovirus Group K3 Member 1		
MicroRNA 4512	microRNAs (miRNAs) are short (20-24 nt) non-coding	

MicroRNA 7161	microRNAs (miRNAs) are short (20-24 nt) non-coding	
Karyopherin Subunit Alpha 2	The import o	Functions in nuclear protein import as ar
Cerebellin 2 Precursor		Acts as a synaptic organizer in specific s
DLG Associated Protein 5		Potential cell cycle regulator that may pl
Family With Sequence Similai	This gene contains an intronless ORF that arose from	
Zinc Finger Protein 737		May be involved in transcriptional regula
Kelch Like Family Member 3	This gene is	Substrate-specific adapter of a BCR (BT
Rac GTPase Activating Protei	This gene en	Component of the centralspindlin compl
Anillin Actin Binding Protein	This gene en	Required for cytokinesis (PubMed:1604(
Prickle Planar Cell Polarity Pr	This gene en	Involved in the planar cell polarity pathw
CD83 Molecule	The protein e	May play a significant role in antigen pre
TNF Alpha Induced Protein 3	This gene wa	Ubiquitin-editing enzyme that contains b
Janus Kinase 3	The protein e	Non-receptor tyrosine kinase involved in
Ankyrin Repeat Family A Member 2		May regulate the interaction between th
NDC80 Kinetochore Complex	This gene en	Acts as a component of the essential kir
Kinesin Family Member 4B	This gene is	Iron-sulfur (Fe-S) cluster binding motor p
Serine Incorporator 5		Restriction factor required to restrict infe
TPX2 Microtubule Nucleation Factor		Spindle assembly factor required for nor
Eukaryotic Translation Initiatic	EIF4E3 belor	Recognizes and binds the 7-methylguan
Carbonic Anhydrase 13	Carbonic anh	Reversible hydration of carbon dioxide.
Prickle Planar Cell Polarity Pr	C6ORF49 is a member of the LIM domain protein fam	
Laccase Domain Containing 1	This gene en	Purine nucleoside enzyme that catalyze
HIVEP Zinc Finger 1	This gene en	This protein specifically binds to the DN
Ecotropic Viral Integration Site 2B		Required for granulocyte differentiation a
Vasoactive Intestinal Peptide	The protein e	PHM and PHV also cause vasodilation.
H4 Clustered Histone 8	Histones are	Core component of nucleosome. Nucleo
Kinesin Family Member 14	This gene en	Microtubule motor protein that binds to r
Proliferating Cell Nuclear Anti	The protein e	Auxiliary protein of DNA polymerase del
LYL1 Basic Helix-Loop-Helix I	This gene represents a basic helix-loop-helix transcrip	
MAM Domain Containing 4		Probably involved in the sorting and sele
Cyclin Dependent Kinase Like	This gene is	Mediates phosphorylation of MECP2 (Pu
MicroRNA 570	microRNAs (miRNAs) are short (20-24 nt) non-coding	
Zinc Finger And BTB Domain Containing 2		Acts as a transcription repressor.
Interleukin 7 Receptor	The protein e	Receptor for interleukin-7. Also acts as a
Pituitary Tumor-Transforming 2		
Peptide YY 2 (Pseudogene)		
Baculoviral IAP Repeat Conta	This gene is	Multitasking protein that has dual roles i
Semaphorin 3B	The protein e	Inhibits axonal extension by providing lo

Ankyrin Repeat Domain 26 Pseudogene 1		
Cytoskeleton Associated Prot	This gene en	Possesses microtubule stabilizing prope
Ubiquitin Specific Peptidase 49		Specifically deubiquitinates histone H2B
Zinc Finger And BTB Domain Containing 1		May be involved in transcriptional regula
ADP Ribosylation Factor Like	This gene be	Positively regulates SLC1A1/EAAC1-me
Autophagy Related 16 Like 2		May play a role in autophagy.
Cyclin B2	Cyclin B2 is a	Essential for the control of the cell cycle
Cell Division Cycle 25C	This gene en	Functions as a dosage-dependent induc
Cell Division Cycle 25B	CDC25B is a	Tyrosine protein phosphatase which fun
Transmembrane Protein 223		
Solute Carrier Family 45 Member 3		
Proline Rich 11		Plays a critical role in cell cycle progress
TP53 Regulated Inhibitor Of Apoptosis 1		Involved in the modulation of the mitoch
Farnesyl-Diphosphate Farnes	This gene en	Catalyzes the condensation of 2 farnesy
NLR Family Pyrin Domain Co	This gene en	As the sensor component of the NLRP1
Family With Sequence Similarity 71 Member F2		
Zinc Finger Protein 337	This gene en	May be involved in transcriptional regula
Biogenesis Of Lysosomal Org	This gene en	Component of the BLOC-1 complex, a c
Mevalonate Kinase	This gene en	Catalyzes the phosphorylation of mevalo
Golgin A8 Family Member O		
Calcium Homeostasis Modulator Family Me		Pore-forming subunit of a voltage-gated
XIAP Associated Factor 1	This gene en	Seems to function as a negative regulat
Protein Regulator Of Cytokine	This gene en	Key regulator of cytokinesis that cross-li
Frizzled Class Receptor 2	This intronles	Receptor for Wnt proteins. Most of frizzl
KCNJ2 Antisense RNA 1		
MicroRNA 6775	microRNAs (miRNAs) are short (20-24 nt) non-coding	
ADAM Metallopeptidase With	This gene en	Cleaves the vWF multimers in plasma in
COBW Domain Containing 3		
Family With Sequence Similarity 86 Member H, Pseudogene		
DnaJ Heat Shock Protein Family (Hsp40) Member C18		
Spectrin Repeat Containing Nuclear Envelo		As a component of the LINC (LInker of N
Cytochrome P450 Family 51 S	This gene en	A cytochrome P450 monooxygenase inv
ESX Homeobox 1	This gene en	May coordinately regulate cell cycle prog
Chromosome 5 Open Reading Frame 63		
Calcium Responsive Transcription Factor		Acts as a transcriptional activator that m
IZUMO Family Member 4		
SWT1 RNA Endoribonuclease Homolog		
Glucokinase Regulator	This gene en	Regulates glucokinase (GCK) by forming

Kelch Like Family Member 4	This gene encodes a member of the kelch family of pr	
LYN Proto-Oncogene, Src Fa	This gene en	Non-receptor tyrosine-protein kinase tha
Lysophosphatidic Acid Recep	The integral r	Receptor for lysophosphatidic acid (LPA
Oxysterol Binding Protein Like	This gene en	Regulates cellular transport and efflux o
RNA, 7SL, Cytoplasmic 834, Pseudogene		
Zinc Finger Protein 695		May be involved in transcriptional regula
Kinesin Family Member 20B		Plus-end-directed motor enzyme that is
Family With Sequence Similarity 95 Member B1		
Ral GEF With PH Domain And SH3 Binding		Guanine nucleotide exchange factor (GE
Squalene Epoxidase	Squalene epo	Catalyzes the stereospecific oxidation of

ctor (GEF) for RhoA and RhoB GTPases.
ng mitosis required for condensin-depleted chromosomes to retain their compact architecture t
own-regulates cell proliferation (in vitro). Promotes RAC1-dependent membrane ruffle formatic
AB7A. Signal effector acting as a linker between RAC1 and RAB7A, leading to RAB7A inactiva
major microtubule plus-end depolymerizing activity in mitotic cells (PubMed:21820309). Regula
role in the incorporation and maintenance of histone H3-like variant CENPA at centromeres. A
RAS-specific effector protein. May promote apoptosis and cell cycle arrest. Stabilizes STK3/MS
otoporphyrinogen-IX to form protoporphyrin-IX.
ascular endothelial growth factor A and interacts with exocyst complex component 4. The enco

-OGT complex that is required for the regulation of the transcriptional activity of RRM1.

n phosphate of phosphatidylinositol 3,4-bisphosphate, inositol 1,3,4-trisphosphate and inositol
isor that negatively regulates the TORC1 signaling pathway through the GATOR complex. In al
s the C-terminal tyrosine residue of alpha-tubulin, thereby regulating microtubule dynamics and
it plays an ABL1-overlapping role in key processes linked to cell growth and survival such as cy
rylate the 5'-hydroxyl groups of double-stranded RNA (dsRNA), single-stranded RNA (ssRNA),
nt and proliferation of ovarian cancer cells so may be involved in these processes. May also pla
nase activity that can prevent apoptosis, promote cell survival and protein translation. May con
or postsynaptic signaling. Inhibitory regulator of the Ras-cAMP pathway. Member of the NMDA
ting GTP hydrolysis on RHOA, CDC42 and RAC1 small GTPases. May be involved in the diffe
ein in all the mitochondrial P450 systems including cholesterol side chain cleavage in all steroi
ransport of the cationic amino acids (arginine, lysine and ornithine); the affinity for its substrate
sion of intracellular vesicles such as lysosomes (PubMed:11984006, PubMed:25216107). Migh
ition.
ntrol of metaphase-to-anaphase transition.
isor that negatively regulates the TORC1 signaling pathway through the GATOR complex. In al

RNAs that are involved in post-transcriptional regulation of gene expression in multicellular or
ition.
in type-1 family of proteins. Members of this family bind to the polyA tails of mRNAs to regulate
luding leukotriene B4. Mediates chemotaxis of granulocytes and macrophages. The response i
sm, and catalyzes the first step of phosphatidylethanolamine and phosphatidylcholine biosynthe
A infection, serves as a post-attachment receptor for the virus to facilitate entry into the cell.||M
RNAs that are involved in post-transcriptional regulation of gene expression in multicellular or
ls to the octamer motif (5'-ATTTGCAT-3'). Regulates transcription in a number of tissues in ad

ıormal myelination in the peripheral nervous system. It mediates adhesion between adjacent m
le arrest in G2/M phase by interfering with microtubule rearrangements that are required to ent
ıtion.
)B1-CUL4-X-box) E3 ubiquitin-protein ligase complex required for cell cycle control, DNA dama
ɔsorbing free syntaxin-1.
t are involved in various types of cell motility and are ubiquitously expressed in all eukaryotic c

e formation of exitatory synapses through the recruitment of pre-and-postsynaptic proteins. Or

entation in retinal progenitor cells (RPCs) and promotes the production of symmetric terminal di

ıtion.

ids generated by active metabolism or to counter adverse environmental conditions. Major pro

subdistal appendage assembly and microtubule anchoring in interphase cells (PubMed:284220
A infection, serves as a post-attachment receptor for the virus to facilitate entry into the cell.||M
)ual specificity phosphatase active toward substrates containing either phosphotyrosine or pho
that binds to the cytoplasmic polyadenylation element (CPE), an uridine-rich sequence elemen

ıtion. May play a role in spermatogenesis.
ivating protein family. In response to DNA damage, the encoded protein interacts with the p53
ons as transcription regulator. Regulates transcription from the PADI1 and CDH2 promoter. Ma
-surface receptor for fibroblast growth factors and plays an essential role in the regulation of ce
is. Negatively regulates neurite outgrowth (PubMed:17389905). Involved in the morphogenesis
nhibition of cellular proliferation in response to DNA damage. Binds to and inhibits cyclin-depe
sion. Inhibits the kinase activity of CDK2 bound to cyclin A, but has little inhibitory activity on CI
es the proliferation of activated peripheral blood T-cells. May have a role in activation-induced c
Potent inhibitor. Potential effector of TGF-beta induced cell cycle arrest.
RNAs that are involved in post-transcriptional regulation of gene expression in multicellular or

play a role in microtubule bundling.
acellular trafficking. Could play an important role in the regulation of glucose transport by insuli
vates its kinase activity thereby playing a central role in DNA replication and cell proliferation. R
icipate in a regulatory mechanism that coordinates cellular responses controlled by NF-kappa-
g protein that interacts with Rab1b and Rab1-binding protein GM130. Alternatively spliced tran
on and cell morphology and cancer progression. Functions as an anti-adhesive molecule that r

-catenin signaling pathway and a target for chemoprevention of hapatocellular carcinoma.
FR3 and FGFR4 (PubMed:8663044). Also acts as an integrin ligand which is required for FGF
ctivity.
d coordination of mitotic processes. The function in regulating microtubule dynamics at spindle
hich is part of a developmental regulatory system that provides cells with specific positional ide
hich is part of a developmental regulatory system that provides cells with specific positional ide
hich is part of a developmental regulatory system that provides cells with specific positional ide
hich is part of a developmental regulatory system that provides cells with specific positional ide
poiesis (By similarity). It has a role in the differentiation of erythroid progenitors (By similarity).
ed in the formation of special mesenchymal tissues.
eiotic recombination processes. Facilitates crossovers between homologs during meiosis (By s
brings together the MCM2-7 helicase and the DNA polymerase alpha/primase complex in orde
chain of as well as pro-urokinase-type plasminogen activator.
The exact function of this gene is not known, however, studies in mice suggest that it may be in
lays a role in a broad range of cellular and developmental processes such as eye, bones, card

nber of lung-specific genes.
t is specifically found in centromeric nucleosomes (PubMed:7962047, PubMed:9024683, PubM

re motor which plays an important role in chromosome congression, microtubule-kinetochore c
romosome segregation in mitosis. Required for kinetochore localization of dynein, LIS1, NDE1
proteins) are involved as a modulator or transducer in various transmembrane signaling systen
hich is part of a developmental regulatory system that provides cells with specific positional ide

3,4-diphosphate (PtdIns3,4P2), but not to other phosphoinositides. May recruit other proteins
mesenchymal transition (EMT), formation and maintenance of embryonic mesoderm, growth ar
g in the kidney.
scribed from a shared bi-directional promoter with zinc finger E-box binding homeobox 1 (ZEB
ays an important role in the sonic hedgehog (Shh) pathway by regulating the activity of GLI tra
mitogen-activated protein kinase signaling pathway.
mitantly with NURR1 in regulating the expression of delayed-early genes during liver regenera
milarity). Involved in cell motility (By similarity). When hyaluronan binds to HMMR, the phospho
d GIMAP7 has GTPase activity. In contrast, GIMAP2 has no GTPase activity by itself.
uitinates DTNBP1 (dysbindin) and promotes its degradation. May ubiquitinate BBS2. May play
y transition pore and is essential for cellular survival and development.
at the alpha methene bridge to form biliverdin. Biliverdin is subsequently converted to bilirubin
endent methyl-transferase activity.

ɔA with acetoacetyl-CoA to form HMG-CoA, which is converted by HMG-CoA reductase (HMG(
RNAs that are involved in post-transcriptional regulation of gene expression in multicellular org
ınscription from both N box- and E box-containing promoters. May with HES1, cooperatively reg
enetetrahydrofolate to 5-methyltetrahydrofolate, a co-substrate for homocysteine remethylation
utilization of fucose from the degradation of oligosaccharides.
r EDA isoform A1. Mediates the activation of the NF-kappa-B and JNK pathways. Activation se
roteins (BMP) pathway.
d contributes to the macromolecular organization of group 1 metabotropic glutamate receptors
signal transduction cascades by linking activated receptors to downstream signaling componen
ssor or as a transcriptional activator, depending on the cell context. Acts as a repressor of the H
e BTE site, a GC-rich DNA element, in competition with the activator SP1. It also represses tran
ı immunoadhesion. Mediates in the adhesion of blood neutrophils in cytokine-activated endoth

l in cell cycle regulation, response to stress and Golgi disassembly. Polo-like kinases act by bir
e consensus sequence 5'-AACAAAG-3' in the promoter of target genes and plays an essential
ıetochore-associated NDC80 complex, which is required for chromosome segregation and spir
RNAs that are involved in post-transcriptional regulation of gene expression in multicellular org
mammalian TEFs.
ent of a SCF-like ECS (Elongin-Cullin-SOCS-box protein) E3 ubiquitin ligase complex which me

ssibly by stabilizing membrane expression of the potassium channel KCNH2/HERG, or by assis
ıe development. Represses the ALPL promoter activity and antagonizes the stimulatory effect o
cell adhesion, cell spreading and in the reorganization of the actin cytoskeleton. Plays a role in t

ıtion.
family of the small GTPase superfamily. It contains a GTPase domain, a proline-rich region, a
pates in the assembly of numerous protein complexes and is involved in several cellular proces
plex also acts as a transcription coactivator: XPC interacts with the DNA-binding transcription f
sylation end products (AGE). These are nonenzymatically glycosylated proteins which accumu
d diacylglycerol (DAG)-dependent serine/threonine-protein kinase that plays essential roles in th
involved in the mediation of Notch signaling (PubMed:18660822, PubMed:20437614). May be
e domains and a serine protease domain, similar to that found in hepatic growth factor. Despite
osine to form sphingosine 1-phosphate (SPP), a lipid mediator with both intra- and extracellula
A sequence 5'-GGGACTTTCC-3' which is found in the enhancer elements of numerous viral pr
e, such as D-glucose and D-fructose, to hexose 6-phosphate (D-glucose 6-phosphate and D-fru
CF-like ECS (Elongin BC-CUL2/5-SOCS-box protein) E3 ubiquitin-protein ligase complex which
ı of skeletal muscle cells but not in the determination of cells to the myogenic lineage. Function
ucture-specific endonuclease which cleaves substrates such as 3'-flap structures.

or differentiation in response to extracellular signals.
t melanoma. It may exert its effects through interactions with the cytoskeleton.
·. Binds to the XRE promoter region of genes it activates (PubMed:10395741, PubMed:286028
tors of intracellular membrane trafficking, from the formation of transport vesicles to their fusion

n dependent kinases and is essential for their biological function.
d in the transmission of signals from tyrosine kinase receptors and small GTPases to the actin
se that catalyzes the formation of retinoic acid (PubMed:27759097). Has high activity with all-tr
on of Akt and PKC signaling. Mediates dephosphorylation in the C-terminal domain hydrophob
SCF (SKP1-CUL1-F-box protein)-type E3 ubiquitin ligase complex.
osine kinase receptors. Concomitantly recruits ERBB1 and ERBB2 coreceptors, resulting in liga
e expression of cytokine and chemokine genes in a wide variety of different cellular contexts. M
ation of alcohol-derived acetaldehyde (Probable). They are involved in the metabolism of cortic
ates circadian rhythm and metabolic pathways in a heme-dependent manner. Integral compone
A-3' (By similarity). Transcriptional repressor (By similarity). Activator of WNT-mediated transc
ype lectin-like domain (CTL/CTLD) superfamily. Members of this family share a common protei
CF-like ECS (Elongin BC-CUL2/5-SOCS-box protein) E3 ubiquitin-protein ligase complex which
ed to act as a specialized replication factor promoting DNA replication under conditions of repli
NOTCH proteins. Has been shown to amplify NOTCH-induced transcription of HES1.
vates its kinase activity thereby playing a central role in DNA replication and cell proliferation. R
survival of cultured cerebellar granule neurons. May mediate homophilic as well as heterophilic
n mitosis. Required for normal congress of chromosomes at the metaphase plate, and for norm
teins within the membrane, to function as part of a cell-surface receptor.
ptor (TCR) signaling by direct dephosphorylation of the Src family kinases LCK and FYN, ITAM
arrest of oocytes at the second meiotic metaphase until fertilization. Probably acts by inhibiting
pairing damaged DNA in a p53/TP53-dependent manner. Supplies deoxyribonucleotides for DN
w affinity for GDP, and constitutively binds GTP. Controls rearrangements of the actin cytoskel
inocyclopropane-1-carboxylate but is capable of catalyzing the deamination of L-vinylglycine.
ein kinase involved in synaptic plasticity, centriole duplication and G1/S phase transition. Polo-
e (PAN) deadenylation complex, one of two cytoplasmic mRNA deadenylases involved in gener
for the apelin receptor (APLNR), an alternative coreceptor with CD4 for HIV-1 infection (PubMe
an cancers. May be part of the regulatory mechanism in the development of epithelial tube net
volved in the formation of parallel microtubule bundles linked by cross-bridges in the proximal a

uence NeuAc-alpha-2,3-Gal-beta-1,3-GalNAc- found on sugar chains O-linked to Thr or Ser ar
anemia (FA) core complex. Required for the normal activation of the FA pathway, leading to m
subunit of the PRC2/EED-EZH1 complex, which methylates 'Lys-27' of histone H3, leading to

mineralization by regulating levels of diphosphate (PPi).

factor (GEF) for Rab5 GTPase. Regulates the ALS2-mediated endosome dynamics.
regulator of telomere length (PubMed:28500257, PubMed:28082411). Directly binds the telom
ntry into mitosis. Acts by participating in E3 ligase complexes that mediate the ubiquitination ar
X to form a sequence-specific DNA-binding protein complex which recognizes the core sequer
d endothelial cell growth, stimulating their proliferation and migration. It binds to the receptor FL
nalization at the plasma membrane through a cargo-specific control of clathrin-mediated endoc

fic obliteration of the intercellular space.
ubunit. It regulates the polymerase and exonuclease activities promoting processive DNA synth
e, methylarginine and, to a much lesser extent, ornithine and histidine (PubMed:24652292). Ca

dance. Mediates axon repulsion of neuronal growth cones in the developing nervous system u
ke linker between two centrioles, required for centrosome cohesion.
scription factor HIF1A to bind to hypoxia-responsive elements (HRE) located within the enhanc
nin in a cell adhesion molecule complex that mediates an initial attachment of the blastocyst to
the CNS.
containing a medium length fatty acid chain to the corresponding monoacylglycerol. Has highe
tion.
tiple snoRNA host gene family. Two snoRNAs are derived from the introns of this host gene. Al
N-acetylneuraminic acid (NeuAc), a precursor of sialic acids. Plays an essential role in early dev
ansduction cascade. Activates the JNK, but not ERK or p38 kinase pathways.
helicase required for the maintenance of both mitochondrial and nuclear genome stability. Effic
y act as a non-canonical poly(A) RNA polymerase.

ter. May catalyze the transport of monocarboxylates across the plasma membrane.
ne cullin-RING-based BCR (BTB-CUL3-RBX1) E3 ubiquitin-protein ligase complex. The BCR(K
e, a sugar that is part of the carbohydrates that are attached to cellular glycoproteins. Catalyze
transcription among the neighboring LOC101735302 (stromal antigen 3 pseudogene), LOC10
gement. Contribute to lamellipodia formation.
some distal) complex, a complex recruited to centromeres which is involved in assembly of kin
domain family (RASSF) of tumor suppressor proteins. This gene is essential for maintaining ad
um-sensitive signal transduction machinery in sperm to a calcium-permeable ion channel.
abolic sensor by regulating the expression of genes involved in bile acid synthesis, cholesterol l

neutrophils, basophils, and T-cells, but not monocytes. It is also involved in neutrophil activatio
s. Proteins with these repeats may form scaffolds for protein-protein interaction and play key ro
tion.
T1 signaling. Represses AKT1 by preventing AKT1-binding to membrane lipids, thereby inhibit
ubiquitination of p53/TP53, leading to its degradation by the proteasome. Inhibits p53/TP53- a

ntegrin and metalloproteinase with thrombospondin motifs) protein family. Members of the fami
ecifically to the MEF2 element, 5'-YTA[AT](4)TAR-3', found in numerous muscle-specific genes
es and monocytes but not neutrophils. Activates the C-X-C chemokine receptor CXCR4 to indu
calcium ion entry. Its activity is increased by reduction in extracellular osmolarity, by store deple

ll-expressed genes. May play a role in hematopoiesis (By similarity).
alyzes preferentially the transfert of ATP and GTP on RNA 3' poly(A) tail creating a heterogene
n transcription between the neighboring SLX1A (SLX1 structure-specific endonuclease subunit
GITR. Regulates T-cell responses. Can function as costimulator and lower the threshold for T-
ifically to the MEF2 element present in the regulatory regions of many muscle-specific genes. (
bMed:22132193, PubMed:25355627). Mediates positive transcriptional regulation of several ch
tors to general transcription apparatus and thereby modulates transcription regulation and chro

tially captures and shuttles two lipid second messengers, i.e., phosphatidylinositol 4,5- bisphos
in coupled to G proteins that stimulate phosphoinositide hydrolysis. May play a role in platelets
not only caspases and apoptosis, but also modulates inflammatory signaling and immunity, mit
lar reactive oxygen species metabolism. May participate in cell growth regulation.

the actin cytoskeleton. May act downstream of CDC42 to induce actin filament assembly leadi

RNAs that are involved in post-transcriptional regulation of gene expression in multicellular org
hed to another protein, or free (unanchored). When covalently bound, it is conjugated to target

plex (MCM complex) which is the putative replicative helicase essential for 'once per cell cycle'
erfamily capable of binding GTP or GDP. G proteins are activated by binding GTP and are inac
P, which is a key regulator of many important physiological processes.
mp which can transport nucleotide analogs. Heme transporter required for the translocation of
ing. Plays a role in the maturation and acidification of phagosomes that engulf pathogens, such
otubule arrays during cell elongation.

fic obliteration of the intercellular space, through calcium-independent cell-adhesion activity.
›ter molecule FADD recruits caspase-8 to the activated receptor. The resulting death-inducing
h the removal of the leader peptide from proteins containing a PTS2 target sequence and proc
ıtion as a transcriptional corepressor. The DEPDC1A-ZNF224 complex may play a critical role i
s ubiquitin from an E2 ubiquitin-conjugating enzyme in the form of a thioester and then directly
perm intra-flagellar transport, and tail assembly. Binds, in a GTP-regulated manner, to a specif

closely packed pairs of transmembrane channels, the connexons, through which materials of
Binds a GC box motif. Could play a role in B-cell growth and development.
e ion channel inhibited by the diuretic amiloride. Mediates the electrodiffusion of the luminal so
or human herpesvirus 6B/HHV-6B.||Receptor for TNFSF4/OX40L/GP34. Is a costimulatory mol
ım. Could be a molecular link between myofibrillar stretch-induced signaling pathways and mus

ine (5hmC), suggesting that it acts as a specific reader of 5hmC.
forms several important functions throughout M phase of the cell cycle, including the regulation
ent of the chromosomal passenger complex (CPC), a complex that acts as a key regulator of r
et of insulin signaling and regulates metabolic homeostasis in response to oxidative stress (Pu
ural organization of enamel.
DNA binding domain) which negatively regulates the basic helix-loop-helix (bHLH) transcriptic
mic (canonical) core consensus DNA sequence 5'-CANNTG- 3' known as an E-box element, p

RNAs, both in normal mRNA turnover and in nonsense-mediated mRNA decay. May remove th
KIF14 localization to the central spindle and midbody. Putative RHO/RAC effector that binds to
DNA binding domain) which negatively regulates the basic helix-loop-helix (bHLH) transcriptic
wide number of exogenous and endogenous hydrophobic electrophiles. Has a sulfatase activit
ional responses by binding to the catalytic domain of calcineurin A. Could play a role during ce
euromodulator in a broad array of neuronal functions directed toward the regulation of goal-dir

optosis. Promotes mitochondrial membrane changes and efflux of apoptogenic proteins from t

equire a bHLH protein for their transcription.
nine, and tyrosine. Probably associated with cell proliferation. Essential for chromosome align
irst two steps in lysine degradation. The N-terminal and the C-terminal contain lysine-ketogluta

nsformed cells after DNA damage. Not essential for development but affects cell adhesion and
as an inhibitor of cellular as well as viral processes, cell migration, proliferation, signaling, and
ition.

rowth-factor-mediated activation of MAP kinase (By similarity). Inhibits fibroblast growth factor (

ition.
afficking in cells.
of satellite repeats, possibly by regulating H3K36 methylation levels in centromeric regions tog
coproteins to which the terminal sialic acid residue on their complex carbohydrate moieties has
ition.
passenger complex (CPC)-mediated cytokinesis. Following phosphorylation by PLK1, involvec
inogen to form the active enzyme plasmin.
sine kinase KIT. Plays an essential role in the regulation of cell survival and proliferation, hema

t adherens junctions.
s.
e which may also possess a cryptic 3'->5' double-stranded DNA exonuclease activity. Function
ward phosphorylase, myosin light chain and myosin substrates.

lly regulates expression of TBX22 in the posterior region of the developing palate. Required du
h a preference for PtdIns. Does not phosphorylate PtdIns(4,5)P2. May be involved in EGF and
nd G2/M checkpoints. Regulates the triggering of DNA replication initiation via its interaction wi
P53. May play a role in the TP53-dependent growth regulatory pathway. May contribute to TP!
lement (CRE) (consensus: 5'-GTGACGT[AC][AG]-3'), a sequence present in many viral and ce

nobilizing LDL in the atherial wall.
at the late G1 and early S phase.
as Notch ligand (PubMed:11134954). Activates NOTCH1 and NOTCH4. Involved in angiogene
ytryptamine (serotonin). Also functions as a receptor for ergot alkaloid derivatives, various anxi
O-ribose methyltransferase that catalyzes the formation of 2'-O-methylguanosine at position 11
RNAs that are involved in post-transcriptional regulation of gene expression in multicellular or
n involved in cell stress response which acts as a dual regulator of transcription and autophagy
EF) for RAP1A, RAP1B and RAP2A small GTPases that is activated by binding cAMP. Seems
nduced autophagy via increased cellular ROS.
r gene, chromosome 11 open reading frame 98 (GeneID:102288414), but the encoded protein
nd catalyzes its covalent attachment to other proteins. In vitro catalyzes 'Lys-11'- and 'Lys-48'-

adation by interacting with COP1 ubiquitin ligase (PubMed:27041596). The COP1-binding moti
esion during mitosis by preventing premature dissociation of cohesin complex from centromere
um channels by preventing the interaction between their alpha and beta subunits. Thereby, ne
tiarrhythmia via the strong suppression of voltage-gated L-type Ca(2+) currents. Regulates vol
ted on Ser, Thr, and Tyr residues and low molecular weight phosphatase substrate para-nitrop

hromosomes dispersed in the cytoplasm following nuclear envelope disassembly (PubMed:27
ting factors are cytokines that act in hematopoiesis by controlling the production, differentiatio
ytosis of vesicles filled with neurotransmitters and neuropeptides. Probably acts upstream of f
d in developmental processes of a variety of tissues and organs, including the heart and coron
osome-mediated cell-cell adhesion.
lle required for faithful chromosome segregation and progression into anaphase (PubMed:196

e/threonine-protein kinase that is implicated in cell spreading and migration, cell survival, cell gr

neuron-restrictive silencer element (NRSE), with lower affinity than full-length REST isoform 1
repair.||[Isoform D2]: Inhibits UV-damaged DNA repair.||Protein, which is both involved in DN

ponsive element from the cellular retinol-binding protein II promoter (CRBPII-RXRE). Inhibits th
regulates adhesion- and motility-associated genes such as NFKB2 and ICAM1. Exhibits oppos
3 MAPKs by inhibiting p88 phosphorylation and activity (By similarity). Might affect PCNA intera

paran sulfate. May fulfill a function related to the motile behaviors of developing neurons (By si
e forming with the TGF-beta type II serine/threonine kinase receptor, TGFBR2, the non-promis
nities around large joints. More than 90% of cases have a recurrent and specific chromosomal

development. Specifically demethylates dimethylated 'Lys-9' and 'Lys-27' (H3K9me2 and H3K2
RF6. Plays a role in clathrin-dependent endocytosis. May play a role in erythropoiesis (By simi
nal repressor in association with BCL6. May function in a narrow stage or be related to some e
s (BMP) signaling which is required for growth and patterning of the neural tube and somite. Es
methylates 'Lys-9' of histone H3, thereby playing a central role in histone code. Preferentially d
-surface receptor for the cytokine KITLG/SCF and plays an essential role in the regulation of ce
epresents a non-protein coding RNA. Alternatively spliced transcript variants have been found f
N-box motifs: 5'-CACNAG-3' (By similarity).
eam open reading frame (uORF) of DDIT3/CHOP that is specifically produced in absence of st

s ubiquitin from an E2 ubiquitin-conjugating enzyme in the form of a thioester and then directly
oic acid as well as longer chain fatty acids required for optimal mitochondrial function.

generate phosphatidic acid (PA). May regulate the activity of protein kinase C by controlling the
:24286120). Functions in innate immunity and more specifically the inflammatory response as a

damaged by oxidation or by mutagenic agents. Acts as DNA glycosylase that recognizes and r

plays an important cytoskeletal role within the muscle cell cytoskeleton. It forms heteropolyme
ch as collagenases) and irreversibly inactivates them by binding to their catalytic zinc cofactor. I
a group of at least six microRNAs (miRNAs) that may be involved in cell survival, proliferation, c

equired for the mitogen or stress-induced phosphorylation of the transcription factors CREB1 ar
ne/serine peptidase domain in the C-terminus. Mutations in this gene are associated with an au

RNAs that are involved in post-transcriptional regulation of gene expression in multicellular org
pigenetic regulator by bridging DNA methylation and chromatin modification. Specifically recog
closely packed pairs of transmembrane channels, the connexons, through which materials of
gnaling cascades. Inhibits signal transduction by increasing the GTPase activity of G protein al
le in various developmental processes such as myogenesis and brain development. Plays a ke
tion.
RNAs that are involved in post-transcriptional regulation of gene expression in multicellular org
C-propeptides, such as chordin, pro-biglycan and pro-lysyl oxidase. Required for the embryonic

ntenance of immune tolerance to self (PubMed:11015443, PubMed:28813417, PubMed:28813
F-beta signaling and thereby probably plays a role in cell proliferation, differentiation, apoptosis
RNAs that are involved in post-transcriptional regulation of gene expression in multicellular org
gulator of spermatogenesis. Acts by regulating expression of genes required for the haploid ph
in for the small GTPases RHOA, RHOB, RHOC and CDC42, terminating their downstream sigr
rombospondin domain, which is found in a number of proteins involved in the complement patl
receptors bind as heterodimers to their target response elements in response to their ligands, a
rmone responses, cell growth, and differentiation. Acts as a tumor suppressor in many cell type
educe both Fe^{3+} to Fe^{2+} and Cu^{2+} to Cu^{1+}. Uses NAD(+) as acceptor (By similarity).

ulates cell motility proliferation and muscle-response to insulin (PubMed:15817639, PubMed:2
|.
uired for receptor recycling from endosomes, both to the trans-Golgi network and the plasma n

volved in the metabolism of all-trans retinoic acid (atRA), a signaling molecule that binds to retir

ion potential waveform and excitability of neuronal and muscle tissues. Inward rectifier potassiu
ediates homophilic cell adhesion in a calcium-independent manner (By similarity). Plays a role
osis. May be acting by homooligomerizing within the membrane and forming pores (PubMed:27
r. Plays a central role in genetic recombination and DNA repair by promoting the annealing of c
najor microtubule plus-end depolymerizing activity in mitotic cells. Its major role may be to trans
proteins) are involved as a modulator or transducer in various transmembrane signaling systen
strand exchange, a key step in DNA repair through homologous recombination (HR) (PubMed:2
nearby locus on chromosome 7, which encodes a putative methyltransferase. There is also a t
sperm flagella and flagellar structure maintainance. It acts as a suppressor of ubiquitination ar
proteins) function as transducers downstream of G protein-coupled receptors (GPCRs) in num
kinase PAK4. Acts by binding PAK4 in a substrate-like manner, inhibiting the protein kinase act
y be involved in synaptic processes (By similarity).
RNAs that are involved in post-transcriptional regulation of gene expression in multicellular org

vator and as repressor. Binds the 5'-CACCC-3' core sequence. Binds to the promoter region o
f cell growth an differentiation, may play different and opposite roles depending on the tissue c

cell development by repressing T-cell receptor (TCR) beta enhancer function. Binds to scaffold/
ts the WAVE1 complex to the close vicinity of PI3K and regulates neuronal morphogenesis.
e, involved in the IFN-alpha/beta/gamma signal pathway (PubMed:7615558). Kinase partner for
sts the folding of proteins upon ATP hydrolysis (PubMed:20080638). Plays a role in the assem
etween hydroxypyruvate and 2-hydroxy-3-oxopropanoate (also termed tartronate semialdehyde
ection, interacts with and deacetylates the viral Tat protein. The viral Tat protein inhibits SIRT1
ransient breakage and subsequent rejoining of DNA strands. Topoisomerase II makes double-
binds to the enhancer heptamer motif 5'-TGA[CG]TCA-3' (PubMed:10995748, PubMed:22083
tional activation of NF-kappa-B target genes. In the cytoplasm, inhibits the nuclear translocatio
g gene activity following the primary growth factor response. Binds to the DNA sequence 5'-TG
P pathway. Binds inositol tetrakisphosphate (IP4).
tor beta (TGFB1, TGFB2 and TGFB3) that controls TGF-beta activation by maintaining it in a l
phosphate metabolism by phosphorylation of second messenger inositol 1,4,5-trisphosphate t

:h R- and S-2-methyl-C15-CoA.

is a receptor for fibronectin (PubMed:1918072). It recognizes the sequence R-G-D in its ligands
donuclease involved in the breakdown of the nucleus during corneocyte formation of epidermal
RNAs that are involved in post-transcriptional regulation of gene expression in multicellular orc
/ fluids by a wide variety of exocrine and endocrine organs (PubMed:2251263, PubMed:112412
multiprotein PRC1-like complex, a complex class required to maintain the transcriptionally repr
ultimate amino acid residues except L-proline.
y and modulates MYC expression by binding to its promoter in a sequence-specific manner. Ac
tes with the NoRC (nucleolar remodeling complex) complex and plays a key role in repressing r
IFRSF1A signaling pathway that leads to NF-kappa-B activation and is required for cell survival
kpoint. Required for normal mitosis progression. The mitotic checkpoint delays anaphase until
forms 2 crucial functions during mitosis: it is essential for spindle-assembly checkpoint signaling
:abolism.

nplex (ORC) that binds origins of replication. DNA-binding is ATP-dependent. The DNA sequen
m1-specific proteases. Studies in mouse determined that Ufsp1 releases Ufm1 (ubiquitin-fold r
d activating and inhibitory receptors and are widely involved in the regulation of the immune sys
tromeric cohesin from separase-mediated cleavage in oocytes specifically during meiosis I. Ha
< promoter elements. Activates the transcription of these genes.
some distal) complex, a complex recruited to centromeres which is involved in assembly of kin
hylation and is essential for the establishment of DNA methylation patterns during developmen
:h plays a role in chromosome congression by reducing the amplitude of preanaphase oscillatio
ster. Transcription of this locus is induced by retinoic acid, and transcripts likely function in regu
lso acts in mesoderm induction, tooth development, limb formation and fracture repair. Acts in
ivator. The monocyte derived PAI-2 is distinct from the endothelial cell-derived PAI-1.
aling.
< activation and apoptosis by promoting MAP2K7 phosphorylation and inhibiting its ability to ac
esters and may function as an intracellular carrier of acyl-CoA esters.
ubMed:3201241). Stimulates the differentiation of myoblasts into osteoblasts via the EIF2AK3-
cally inhibit gene expression when recruited to promoter regions by sequence-specific DNA-bir
:tivator. Key determinant of the keratinocyte proliferation-differentiation switch involved in appro

ide channel (CaCC) activity.
de lipase activities (PubMed:12032167, PubMed:10318835, PubMed:10192396). More active a
eferentially to the canonical E box sequence 5'-CACGTG-3' (PubMed:11095750). Downstream
y be implicated in the origin and progression of lung cancer.

drate chains of glycoconjugates and are involved in cell-cell recognition and cell-pathogen intera
naximal activity towards saturated C22-CoA (PubMed:21237683). Probably participates in beta
otes disassembly of several types of membraneless organelles during mitosis, such as stress g
e 5'-AATA[CT]-3' core sequence and plays a key role in adipogenesis and liver development. A

a remarkable functional diversity in the regulation of cellular responses. These include the regu
G-proteins that activate both phosphatidylinositol-calcium and adenylyl cyclase second messeng

OSB, FOSL1, and FOSL2. These genes encode leucine zipper proteins that can dimerize with p
e GA element of the CLCNKA promoter. Binds to the KCNIP2 promoter and regulates KCNIP2
's an important role in DNA replication, transcription and repair. Inhibits elongation of stalled tra
unit of the tRNA-splicing endonuclease complex, a complex responsible for identification and cl
nsor within the amino acid-sensing branch of the TORC1 signaling pathway. As a homodimer o

hospholipids from the cytoplasmic to the extracellular/lumenal leaflet of membrane coupled to t
pressor required for cardiac development and may have key roles in the maintenance of functi
n the biogenesis of other small nuclear RNAs. This RNA is found in the nucleolus, where it may
PIWI-interacting RNA (piRNA) biogenesis during spermatogenesis. piRNAs provide essential
. Enhances cyclin-dependent kinase tyrosine phosphorylation by nonreceptor tyrosine kinases
osomes wrap and compact DNA into chromatin, limiting DNA accessibility to the cellular machin

tion.

l synthesis by controlling SCAP and HMGCR. Functions by blocking the processing of sterol re
l complex (SCMC), plays an essential role for zygotes to progress beyond the first embryonic c
57084). Activated by WNT3A, WNT3, WNT1 and to a lesser extent WNT2, but apparently not
which may be required for cardiovascular development. Transcriptional repressor which binds
ond of glutathione conjugates, but maybe not glutathione itself. Converts leukotriene C4 (LTC4)
of the Wnt-Fzd-LRP5-LRP6 complex that triggers beta-catenin signaling through inducing aggre
oepithelium during neural tube closure. Induces apical constriction in epithelial cells by promoti

nsport in photoreceptor cilia.
een intermediate filament stability and degradation, a process that is essential for skin integrity
denal epithelial cell and also in transfer of iron between maternal and fetal circulation. Mediates

RNAs that are involved in post-transcriptional regulation of gene expression in multicellular org

ribonucleoprotein complex that mediates cotranslational insertion of secretory proteins into the

g protein FLCN: involved in the cellular response to amino acid availability by regulating the mT
nger domain, a motif present in a variety of functionally distinct proteins and known to be involv
nger complex (CPC), a complex that acts as a key regulator of mitosis. The CPC complex has e
ole in the Hippo signaling pathway, a pathway involved in organ size control and tumor suppres
g RNAs involved in RNA processing. Box H/ACA snoRNAs, such as SNORA5C, direct the con
s ubiquitin from E2 ubiquitin-conjugating enzymes UBE2L3 and UBE2L6 in the form of a thioes
s in umbilical vein endothelial cells (HUVECs). May be involved in dopamine synthesis. May m

ance to glucose starvation. Induces cell-cell detachment by increasing F-actin conversion to G-
osomes wrap and compact DNA into chromatin, limiting DNA accessibility to the cellular machir

that interacts with inducible viral and cellular enhancer elements to regulate transcription of sel
for innate immune signaling pathways.
s in the regulation of cell polarity and directional endothelial cell migration by mediating both th
be involved in transcriptional activation of erythroid genes.
ng and antagonizing protein for members of the TGF-beta family, such us activin, BMP2 and M
endothelin-1 gene expression in endothelial cells. Binds to the consensus sequence 5'-AGAT
rocesses and in cell adhesion.
RAB5. Does not act on RAB4 or RAB11 (By similarity).
and stabilize the formation of the actin and microtubule network.
acellular trafficking (By similarity). Interacts with membranes containing phosphatidylinositol 3-
5519, PubMed:27756709, PubMed:22750565, PubMed:22824924). Regulates SEMA3C and P

ation of germ cells.
family that is associated with the nuclear membrane and is thought to control a variety of cellul
the onset of mitosis.
-5'-adenylyl sulfate (PAPS) as sulfonate donor to catalyze the transfer of sulfate to position 6 o
of hematopoietic stem cells and megakaryocyte progenitor cells and induces megakaryocyte
yme involved in mitotic spindle assembly.

pluripotency and differentiation in the embryo.
cans, aggrecan (at the '1838-Glu-|-Ala-1839' site) and versican (at the '1428-Glu-|-Ala-1429' si
at is able to bind to the EGF receptor/EGFR and to act synergistically with TGF beta to promot
ic vesicles at presynaptic active zones.
negative regulator of BMP signaling pathway. Mediates ubiquitination and degradation of SMA

ifically recognizes and binds mono- and dimethyllysine residues on target proteins, therey actir
ential role in erythroid and megakaryocytic cell differentiation. Essential cofactor that acts via tl
t catalyzes the cleavage of the cap structure of snoRNAs and mRNAs in a metal-dependent m
ex that serves as a microtubule-dependent and Rho-mediated signaling required for the myosi
and branching.
DAR, while isoform 3 binds exclusively to the receptor EDA2R.||[Isoform 3]: Binds only to the re
randed DNA (ssDNA), or partially ssDNA structures such as bubble and fork structures, to dou

voltage-gated rapidly inactivating A-type potassium channels. Modulates channel density, inac
a target for Rho, and interact with some cytoskeletal component upon Rho binding or relay a F
ckpoint that prevents the onset of anaphase until all chromosomes are properly aligned at the

in human erythrocyte membranes.
that converts diacylglycerol/DAG into phosphatidic acid/phosphatidate/PA and regulates the re
ributes to the regulation of cell cycle progression (PubMed:26246606). Associates with the cen

P antiflammatory activity. Suppresses IL1R-TLR signaling through its direct interaction with PE

nsforming growth factor) type 1 receptor superfamily members; has been shown to inhibit TGF
ases ligand-dependent transcriptional activity of AR and promotes AR sumoylation. The stimul
MP (bone morphogenetic proteins) type 1 receptor kinase. SMAD9 is a receptor-regulated SM

I2 or PGI2). The activity of this receptor is mediated by G(s) proteins which activate adenylate
leus and is expressed in numerous tissues including brain, testis, liver, and kidney. This refsec
s from glycoproteins by cleaving the beta-1,4-glycosidic bond in the N,N'-diacetylchitobiose co
lay a role in activity-dependent changes of brain vasculature. May affect blood-brain permeabil
mismatch repair system (MMR). Heterodimerizes with MSH2 to form MutS alpha, which binds t
the biosynthesis pathway of prostanoids, a class of C20 oxylipins mainly derived from arachid

n. Negatively regulates CEP63 and CDK2 centrosomal localization.

g RNA transcribed from this gene is expressed at high levels in lymphoma and may function a

and double-stranded DNA and RNA. Mediates p53-induced apoptosis. When induced by p53
mology-mediated end-joining (MMEJ), an alternative non-homologous end-joining (NHEJ) ma

d skin barrier function. Plays a role in the lamellar granule (LG) secretory system and in the str
in (GAP) for RHOA and CDC42. Downstream partner of ARF1 which may control Golgi appara

Augments Ca(2+) sensitivity of the contractile apparatus.
ition.
ire and body weight in response to metabolic and toxin-induced stresses (PubMed:28953886, I
ooperatively with DP proteins through the E2 recognition site, 5'-TTTC[CG]CGC-3' found in the
king and in targeting proteins for lysosomal degradation (PubMed:23166352). Plays a role in ta
lates the transcription factor ETS2 (in vitro) and positively controls its proteasomal degradation

can, at the '1938-Glu-|-Leu-1939' site (within the chondroitin sulfate attachment domain), and n
ition.
RNAs that are involved in post-transcriptional regulation of gene expression in multicellular or
iscriptional repressor.
transcription factor TFIID that may function as a gene-selective coactivator in certain cells. TFI

ity for the ERK family (PubMed:9858808). Plays an important role in alleviating chronic postope
s to various membrane receptors, nuclear factors and signaling proteins to regulate many proc
n mitogen-activated protein kinase signaling pathway.
cell proliferation, growth, migration and epithelial to mesenchymal transition. Through the degr
at the G2/M (mitosis) transition.
CF (SKP1-CUL1-F-box protein) E3 ubiquitin-protein ligase complex which mediates the ubiquit
ticipates in various processes such as angiogenesis, polyploidization of specialized cells and D
RNAs that are involved in post-transcriptional regulation of gene expression in multicellular or
ictose-2,6-bisphosphate as well as fructose-1,6-bisphosphate (PubMed:19015259). Acts as a
ition.
mediated by association with deadenylase subunits of the CCR4-NOT complex. Activates mRN
volved in activation of vitamin D precursors. Catalyzes hydroxylation at C-25 of both forms of vit
binding factor (CBF) with RUNX family proteins (RUNX1, RUNX2, and RUNX3). RUNX membe
ia EGFR, ERBB2 and ERBB4. Required for normal cardiac valve formation and normal heart fu
nitosis (PubMed:11125146, PubMed:22581055, PubMed:21856198, PubMed:14993212, PubM
T1 in the Hippo signaling pathway which plays a pivotal role in organ size control and tumor su

he transsulfuration pathway, where the hydroxyl group of L-serine is displaced by L-homocystei
tin-protein ligase complex, a complex that mediates ubiquitination and subsequent degradation
PAR complex, may be involved in synaptic transmission in the central nervous system. In hipp
PubMed:10207080). Activates the epsilon- and gamma-globin gene promoters and, to a much
of axonemal dyneins, thereby playing a central role in motility in cilia and flagella. Involved in pr

regulator to integrate signals provided by PHD- and/or bromodomain-containing transcription fa
Phe) with phenylalanine in mitochondrial translation. To a lesser extent, also catalyzes direct at
of inorganic pyrophosphate (PPi), probably functioning as PPi transporter.
ein-tyrosine kinases through its SH2 domain and regulates their kinase activity. During insulin s
ulation of neuronal process formation and in differentiation of neural crest cells. Down-regulates
pole organization via its interaction with NUMA1 (PubMed:11781568, PubMed:15632202, Pul

helial cell polarization. Involved in apical junction complex (AJC) assembly via its interaction with
of scaffold proteins selectively mediates JNK signaling by aggregating specific components of
ates transcriptional repression via its association with DNA-binding transcription factors and rec
phorins and subsequent remodeling of the cytoskeleton. Plays a role in axon guidance, neuron
differentiation in leukemic cells. Its activities include the induction of hematopoietic differentiatio
the consensus sequence 5'-AACAAT-3' (PubMed:10871192). Binds to the proximal promoter
in the DNA damage response and DNA repair.
n the assembly of small nuclear ribonucleoproteins (snRNPs), the building blocks of the splice
hat are acutely recruited to plasma membrane ruffles in response to insulin receptor signaling.
tiapoptotic factor.
ates circadian rhythm and metabolic pathways in a heme-dependent manner. Integral compone
ltiprotein complex that functions downstream of the MRN complex to promote DNA repair and

3 expression (PubMed:23242139). Required for the relief of p53-dependent checkpoint mediat
s NEK11 in G1/S-arrested cells.||[Isoform 2]: Not present in the nucleolus and, in contrast to is
levels of target RNA species.
(DSBs) formation in unsynapsed regions during meiotic recombination. Probably acts by formi
or hepatitis C virus in hepatocytes, but not through a direct interaction with viral proteins.||(Micr

membrane ion transport. Regulates ASIC2 and ASIC3 channel activity.
protective factor prosaposin. Ligand binding induces endocytosis, followed by an ERK phosph
ptional regulator. Plays a role as a molecular node in a transcriptional network regulating embr
produce lysophosphatidic acid (LPA) and the corresponding amines. Shows a preference for 1
tion.

RNAs that are involved in post-transcriptional regulation of gene expression in multicellular org
proteins) are involved as modulators or transducers in various transmembrane signaling syster
ordinating role for tyrosine-kinase-based signaling related to cell adhesion. May function in trar

›asic protein gene (MBP). Binds to the proximal MB1 element 5'-TTGTCC-3' of the MBP promo
hydrolysis of ATP coupled to the transport of aminophospholipids from the outer to the inner le
nt coupling of the nucleus and the centrosome. Involved in the processes that regulate centros
nigh affinity to the T-cell enhancer motif 5'-AACAAAG-3' motif (PubMed:30661772). Required f

ngation of O-linked fucose residues attached to EGF-like repeats in the extracellular domain of

s an inhibitory modulator of neuronal plasticity in aversive memory formation. Can affect Ras si
ted potassium KCNMA1 (maxiK) channel. Modulates the calcium sensitivity and gating kinetics
P, which is a key regulator of many important physiological processes. May be involved in the c
cold exposure that promotes energy expenditure. Induced either in the skeletal muscle after ex
nulates progression from G1 into S phase during mitosis. May play a role in the development ar
ivity. May play a role in apoptosis.
|Essential mediator of p53/TP53-dependent and p53/TP53-independent apoptosis (PubMed:1
NA sequences containing the consensus 5'-WGGA-3'. Transactivates promoters of the hemato
in the response to oxidative stress: binds to antioxidant response (ARE) elements present in tl
of cytokine genes in T-cells, especially in the induction of the IL-2 or IL-4 gene transcription. Als
on of the microtubule cytoskeleton, and probably plays a role in regulating the orientation of the
of cytokine genes in T-cells, especially in the induction of the IL-2, IL-3, IL-4, TNF-alpha or GM-
ipper motif. The exact function of the encoded protein is not known. In mice this gene affects n

s as a general RNA polymerase II transcription inhibitor (PubMed:14580347, PubMed:1571366
pathway (PubMed:28111074). Activated by APOE, enhances the AP-1-mediated transcription
mily. Intermediate filaments are proteins which are primordial components of the cytoskeleton
ger complex (CPC), a complex that acts as a key regulator of mitosis. The CPC complex has e

d SUMO3 from targeted proteins, but not SUMO1. Catalyzes the deconjugation of poly-SUMO2
iction in myelinated cells through the organization of molecular complexes at nodes of Ranvier
recognizes and binds 'Lys-63'-linked ubiquitin. Does not bind 'Lys-48'-linked ubiquitin. Positive
B/REL complexes by trapping REL dimers in the cytoplasm through masking of their nuclear lo
the cell.
drogenases/reductases (SDR) family. The encoded enzyme contains a conserved catalytic do
CF (SKP1-CUL1-F-box protein) E3 ubiquitin-protein ligase complex which mediates the ubiquit
at the G1/S (start) transition.
'N and BLK promoters. Appears to be required for the T-cell-receptor-mediated trans activatior

tors/nuclear receptor complex to inhibit ligand-dependent transactivation.
ieutrophils and expressed at sites of inflammation. Hematoregulatory chemokine, which, in vitrc
iemotactic activity for neutrophils. May play a role in inflammation and exert its effects on endo
r noncoding small nucleolar RNAs (snoRNAs), like SNORD83A, which are generated during pr
i the late phase of epithelial polarization and microtubule dynamics regulation. Plays a role in t
ntially the consensus sequence 5'-TAAGT[AG]-3' and can behave as a transcriptional represso
1949). Recognizes and binds to the DNA sequence 5'-GCG(T/G)GGGCG-3'(EGR-site) in the p

r microtubule nucleation at the centrosome.
i sister chromatid cohesion and fidelity of chromosome transmission. Component of one of the

ctions as a dosage-dependent inducer of mitotic progression. Directly dephosphorylates CDK1

at self-assembles into virion-like capsids that encapsulate RNAs and mediate intercellular RNA

a of the primate neocortex.
RAB5. Does not act on RAB4 or RAB11 (By similarity).
f acetylated low density lipoprotein (Ac-LDL). Mediates heterophilic interactions, suggesting a fu
transmembrane phosphoprotein specifically associated with tyrosine phosphatase PTPRC/CD

eceptors, a family of receptor tyrosine kinases which are crucial for migration, repulsion and ad
s. Regulates EPHA8 receptor tyrosine kinase signaling to control cell migration and neurite retr
and stimulates DNA synthesis in cells. Involved in the translational regulation of the human pa
on. Also participates in checkpoint controls that ensure DNA replication is completed before mi

of the anaphase promoting complex/cyclosome (APC/C) and may confer substrate specificity u

or hepatitis C virus (HCV) in hepatocytes and facilitates its cell entry.||Plays a key role in the co
entral role in the chromosome segregation by cleaving the SCC1/RAD21 subunit of the cohesin
ction in a Rho pathway to limit stress fiber formation and/or increase the turnover of F-actin stru
2H2)-type zinc fingers, which are similar to those found in the nuclear matrix protein matrin 3. A
oteasome-dependent degradation. By counteracting accumulation of CP110, maintains normal
or PCNA loading on replication sites. Promotes S-phase entry and DNA synthesis (PubMed:24
tion.
cts as corepressor of ESR1; the function seems to involve CTBP1 and histone deacetylases.

plex II (COPII), may function in vesicle budding and cargo export from the endoplasmic reticulu
ity may also function in the Wnt signaling pathway and the stabilization of beta-catenin/CTNNB

on activity.||[Isoform Alpha]: Has transcriptional activation and repression activity (PubMed:158
on by competing with PLCG for binding to ESR1, blocking the effect of estrogen on PLCG and
ential for T-cell proliferation and IFNG production in a PDCD1-independent manner. Interaction
equire a bHLH protein for their transcription. May act as a negative regulator of myogenesis by

Serine-threonine kinases transfer phosphate molecules to the oxygen atoms of serine and thr
isomal beta-oxidation pathway for fatty acids. Catalyzes the formation of 3-ketoacyl-CoA intern
here it inhibits aggregation of citrate synthase.
histamine receptors mediates the contraction of smooth muscles, increase in capillary permea
it regulates reorganization of the actin cytoskeleton, cell polarization, cell migration, adhesion,
se that catalyzes the reversible transfer of the terminal phosphate group between nucleoside t
inobutyrate and L-beta-aminoisobutyrate to succinate semialdehyde and methylmalonate semi
of pulmonary surfactant, probably by transporting lipids such as cholesterol.
om short-chain fatty acids (PubMed:16788062). Acetate is the preferred substrate (PubMed:16
ics and in the activation of ester and amide prodrugs (PubMed:9169443). Shows high catalytic
plays a central role in p53/TP53-mediated apoptosis. Not involved in p73/TP73-mediated auto
ction as a coat complex required for sorting of specific membrane proteins to the primary cilia.
(GAP) for Rab family protein(s). May act as a GAP for RAB7A. Can displace RAB7A and retro
omal-mitochondrial pathway. Translocates to the lysosome initiating the permeabilization of lys
alkylated DNA. Promotes DNA unwinding to generate single-stranded substrate needed for AL
ation with E3 ligase DTX3L, plays a role in DNA damage repair and in immune responses inclu
therefore is catalytically inactive. Represses MEF2-dependent transcription by recruiting HDAC
maturation, structural maintenance and proper regulation of specific target proteins involved fo
lity- and head-associated functions such as capacitation and the acrosome reaction. Isoform 1
isocitrate via cis-aconitate.||Iron sensor. Binds a 4Fe-4S cluster and functions as aconitase wh
ney and vascular development. Promotes neurite elongation from olfactory bulb explants.
receptor, mediating natural killer cell cytotoxicity.

Lacks a cytoplasmic death domain and hence is not capable of inducing apoptosis. May prote
ids generated by active metabolism or to counter adverse environmental conditions. Major pro
it controls membrane reorganization/tubulation upon ATP hydrolysis. In vitro causes vesiculatio
e in the regulation of embryonic development, cell proliferation, cell migration, survival and che
the DNA repair by translesion synthesis (TLS) (PubMed:10385124, PubMed:11743006, PubM
of cell proliferation and cell differentiation. Required for normal regulation of the hair growth cy
or Herpes simplex virus 1/HHV-1.||(Microbial infection) Acts as a receptor for Herpes simplex vi

required for phagocytosis of apoptotic cells and cell motility. Acts in association with DOCK1 a
and cellular differentiation in the retina and bone formation. Plays a key role in regulating apopt
ls chemokine levels and localization via high-affinity chemokine binding that is uncoupled from
F (nucleosome-remodeling factor) and CERF (CECR2-containing-remodeling factor) complexes
ghest specific activity with phenylalanine. May play a role in lysosomal antigen processing and

nteractions via its interactions with neurexin family members. Plays a role in synapse function a
ort of organic anions such as estrone-3-sulfate (PubMed:10873595). Mediates transport of pros
NA-binding specificity. Binds with a higher affinity to methylated CpG dinucleotides in the conse

cess critical to neuronal development and function such as cell migration, neurite outgrowth an
linking various signaling pathways to transcriptional regulation. Negatively regulates the transcr
tein that promotes the assembly of the spectrin-actin network. Plays a role in actin filament cap
the terminal and only committed step in triacylglycerol synthesis by using diacylglycerol and fat
losome formation.
P-ribosylhydrolase by mediating the removal of mono-ADP-ribose attached to arginine residues
ates mono-ADP-ribosylation of target proteins.
um (ER) retrograde transport; the function is proposed to depend on its association in the NRZ
ates both the synthesis and turnover of phosphatidylinositol 3,5-bisphosphate (PtdIns(3,5)P2). I
glutaryl-CoA lyase that catalyzes a cation-dependent cleavage of (S)-3-hydroxy-3-methylglutar
catecholamine-induced activation of adenylate cyclase through the action of G proteins. The be
ytokines and environmental stress in vivo. Catalyzes the concomitant phosphorylation of a thre
ternary MAP3K2/MAP3K3-MAP3K5-MAPK7 signaling complex. Activation of this pathway appe
l in displaying microbial pyrimidine-based metabolites to alpha-beta T cell receptors (TCR) on i
olved in the proteasome-mediated ubiquitin-dependent degradation of target proteins (PubMed:
part of the aminoacyl-tRNA synthetase multienzyme complex, also know as multisynthetase co
of cellular nucleotides by catalyzing the interconversion of nucleoside phosphates. Has both n
tubules. It binds two moles of GTP, one at an exchangeable site on the beta chain and one at a
mal tubulin. Plays a role in anterograde intraflagellar transport (IFT), the process by which cilia
hich acts as an inhibitor of retrovirus replication and retrotransposon mobility via deaminase-de
ne dehydratase activity.
ruitment and assembly of spindle midzone components. Required for the localization of AURK
ent protein kinases involved in cAMP signaling in cells.
ne proteins that function as uniporters, symporters, and antiporters to transport organic ions acr
bone marrow and cord blood mononuclear cells that bear the CD34 (MIM 142230) surface ma
by enhancing ERK phosphorylation and up-regulating RUNX1 expression.
nd binds to the consensus sequence '5-[CG]NG[CG]GGGCA[CA]CC-3'. May act as a tumor su

ndria along microtubules. Regulates the kinesin-mediated axonal transport of mitochondria to n
ration or fertilization (By similarity). Phospholipid transfer protein that preferentially selects lipid
peroxide-producing NADPH oxidase. Functions in the production of reactive oxygen species (F
B. Mediates activation of NF-kappa-B. Inhibits vascular endothelial growth and angiogenesis (ir
g protein that plays a role in ERBB2-mediated tumor cell migration in response to growth factor
cytoplasmic sensor of viral nucleic acids and plays a major role in sensing viral infection and ir
n late Golgi and early endosomes.
hospho-D-glycerate to 3-phosphonooxypyruvate, the first step of the phosphorylated L-serine b

ong chain aliphatic aldehydes to fatty acids. Active on a variety of saturated and unsaturated a
th NF-kappa-B via a NF-kappa-B inhibitor kinase (IKK)-dependent mechanism and stress-activ
of proteins involved in translation regulation including EIF2A, EIF4EBP1 and RPS6KB1. May b
response (PubMed:15705585, PubMed:22863753, PubMed:25277106). Attenuates signaling
drug olmesartan medoxomil into its pharmacologically active metabolite olmerstatan, an angio
EF) for RAB5A and RAB22A that activates RAB5A and RAB22A by exchanging bound GDP for
-type natriuretic peptide (CNP) promoter.
a KRAS effector protein. May promote apoptosis and cell cycle arrest.
naling pathways. Interacts with the SH2 and SH3 domains of various signaling proteins when it
or hepatitis C virus (HCV) in hepatocytes (PubMed:17325668, PubMed:20375010). Associates
L/SGNH-like acyl-esterase family. Members of this family are hydrolases thought to function in
integrin activation. When coexpressed with talin, potentiates activation of ITGA2B. Required fo
activity on endothelial cells. When overexpressed in endothelial cells, leads to inhibition of cel
ake of dibasic amino acids and sodium-dependent uptake of some neutral amino acids. Requir
ys a role in multiple processes including the regulation of actin cytoskeleton dynamics, actin str
orm 4-isoform 14) are with PLP the most abundant protein components of the myelin membrar
n the histone hyperacetylation pathway (PubMed:10383164). Releases a C-terminal amino aci
FIH1/MDA5-dependent type I interferon and interferon inducible gene expression in response t
hich promotes membrane fusion and is involved in exocytosis.

-1 stimulates thymocyte proliferation by inducing IL-2 release, B-cell maturation and proliferatio
n involved in the control of T-cell activation. May play a role in the CD4-p56-LCK-dependent sig
d Gln-tRNA(Gln) through the transamidation of misacylated Glu-tRNA(Gln) in the mitochondria.
oligosaccharide biosynthesis, the transfer of an N-acetyl-D-galactosamine residue to a serine o
ulation of the actin cytoskeleton (By similarity).
epithelium development through its interaction with proteins of the beta-catenin family (Probab
nodulating differentiation and maintaining homeostasis of epithelial cells. This retinoic acid-indu
, oxidizing aromatic azaheterocycles, such as N1-methylnicotinamide, N-methylphthalazinium a

ific membrane cytoskeleton in association with beta-spectrin.||In skeletal muscle, required for c
onal activity which is involved in adipose cells differentiation. May also play a regulatory role in
oligosaccharide biosynthesis, the transfer of an N-acetyl-D-galactosamine residue to a serine c
ition of the circadian clock: acts as a transcriptional repressor of the core circadian component
h a leucine zipper motif, two helix-loop-helix motifs (I repeats) that are similar to domains founc
RES2. Does not appear to be a signaling receptor, but may have a role in modulating chemoki

plays a central role in double-strand break (DSB) repair, DNA recombination, maintenance of t
ins that create proton leaks across the inner mitochondrial membrane, thus uncoupling oxidativ
y an important role in cell-cell interactions at interendothelial junctions (By similarity). Acts as a
esmosome and the attachment of basal keratinocytes to the underlying basement membrane.||
e in the regulation of embryonic development, cell proliferation, cell migration, survival and che
ehydrogenase (SDH) that is involved in complex II of the mitochondrial electron transport chair
transmembrane 4 superfamily, also known as the tetraspanin family. Most of these members a
mplex with lecithin-cholesterol acyltransferase. It is probably involved in the transport and bindi
at plays a role in synaptic and cognitive functions by modulating actin filamentous (F-actin) dyn
e similarity to the ligand binding domain of platelet-derived growth factor receptor beta. Mutatio
h heparan sulfate and chondroitin sulfate and that links the cytoskeleton to the interstitial matri
/haptoglobin in the endoplasmic reticulum.
ENA-78(8-78) and ENA-78(9-78) show a threefold higher chemotactic activity for neutrophil gr
drolyzing the alpha-1,6-linked fucose joined to the reducing-end N-acetylglucosamine of the ca
The C-terminal gamma-secretase processed fragment, ALID1, activates transcription activatic
oplasmic viral nucleic acids and activates a downstream signaling cascade leading to the produ
nor suppressor gene located at 5q21. Suppresses cell proliferation and the Wnt/b-catenin path
t activity concerns the C6 to C10 chain length substrate. Converts the end product of pristanic
nd other protein precursors at sites comprised of pairs of basic amino acid residues. Substrate
ntagonized by oncoproteins, such as papillomavirus (HPV) protein E7 and adenovirus early E1
play a regulatory role in the acute-phase response in systemic inflammation and may modulate
of apoptosis triggered by O(6)-methylguanine.
I thymidylate biosynthesis pathway.
ng protein that scaffolds the cAMP-dependent protein kinase holoenzyme. May serve as a poir
nulates progression from G1 into S phase during mitosis. Enhances cell proliferation, cell motilit
phosphodiesterase with a dual-specificity for the second messengers cAMP and cGMP, which
sports free histidine and certain di- and tripeptides (By similarity).
on of 4-hydroxyproline in -Xaa-Pro-Gly- sequences in collagens and other proteins.
erol permeability and water transport across cell membranes (PubMed:12239222, PubMed:30
migration.

edgehog signaling by antagonizing the effects of SMO, as suggested by down-regulation of hed
d G-protein coupled receptor (GPCR) signaling by mediating both receptor desensitization and
mulates T-cell proliferation and cytokine production.
ating the establishment of proper connectivity within the nervous system. Acts in both pre- and

atopoietic cells by activating FLT3. Synergizes well with a number of other colony stimulating fa
ono-ADP-ribosylation of glutamate residues on target proteins (PubMed:16061477, PubMed:2
regulation. Acts either as activator or repressor of splicing on specific pre-mRNA targets. Inhib
nterleukin, a heterodimeric cytokine which functions in innate immunity. IL-27 has pro- and anti-
ation of viruses by recruiting the cellular RNA degradation machineries to degrade the viral mR
gh its influence on the Ral signaling pathway.
lular target for adenovirus E3 14.7 and Bluetongue virus protein NS3 to inhibit innate immune r

e for the removal of the invariant chain from MHC class II molecules. The bond-specificity of th
nsfer of a phosphoryl group from phosphoenolpyruvate (PEP) to ADP, generating ATP. Stimula
ormone receptor coactivator. Enhances transcription of genes with retinoic acid response elem
nogen plasminogen to plasmin by hydrolyzing a single Arg-Val bond in plasminogen. By contro
lial cell polarity by connecting ARF6 activation with the PAR3 complex (By similarity). Plays a re

double-strand DNA break repair (PubMed:17353262, PubMed:17396150). Recruited to sites of
cellular pathways, including signal transduction, membrane trafficking, and the regulation of m
between an inactive GDP-bound and an active GTP-bound form, and the rate of cycling is reg
to 5-oxo-L-proline and a Cys-Gly dipeptide. Acts specifically on glutathione, but not on other ga
g.
yzes both the N-deacetylation and the N-sulfation of glucosamine (GlcNAc) of the glycosamino
in O-mannosylation of alpha-dystroglycan (DAG1). Transfers a glucuronic acid (GlcA) residue
omponents of the extracellular matrix such as collagen. Activates progelatinase A. Essential for
alNAc) from UDP-GalNAc to the non-reducing end of glucuronic acid (GlcUA). Required for ad
hosphorylates PtdIns(4,5)P2 (Phosphatidylinositol 4,5-bisphosphate) to generate phosphatidyli
Lys-48'-linked conjugated ubiquitin from proteins. Has exodeubiquitinase activity and has a pre
physiological regulators of gastrointestinal, cardiovascular bioactivities mediated by the CALCR
ociation with ADP-ribosyltransferase PARP9, plays a role in DNA damage repair and in interfer
adapter that brings other proteins (like kinases) to the cytoskeleton (PubMed:10861853). Involv
pates in the assembly of numerous protein complexes and is involved in several cellular proces
tant protective effects. Metabolizes betaine aldehyde to betaine, an important cellular osmolyte
d proteins. Cleaves the thioether bond of prenyl-L-cysteines, such as farnesylcysteine and gera
tor protein recruitment to a group of sequence-specific transcription factors. Augments cytokine

nesium-dependent enzyme that catalyzes the hydrolysis of ATP coupled with the transport of ca
;yte adhesion protein LFA-1 (integrin alpha-L/beta-2). ICAM2 may play a role in lymphocyte rec
/ racemic phytanoyl-CoA and the isomers of 3-methylhexadecanoyl-CoA, but also a variety of c
ɔrt and docking. Involved in the proper sorting of TYRP1. Involved in peripheral melanosomal c
ocyte differentiation, lipid accumulation, and glucose uptake in mature adipocytes.

R signaling pathway upstream of the TSC1-TSC2 complex and downstream of AKT1.
ɔr Coxsackievirus A21 capsid proteins.||(Microbial infection) Acts as a receptor for major recep
C) such as aldosterone and glucocorticoids (GC) such as corticosterone or cortisol. Binds to mi
epair and protein ubiquitination, as part of the UV-DDB complex and DCX (DDB1-CUL4-X-box)
d in vesicular trafficking of early endosomes (PubMed:16476778). Mediates the recruitment of
such as WDR54, and the conjugation of polyamines to proteins.
rporation of selenocysteine into proteins. It probably replaces EF-Tu for the insertion of seleno
s signals from the extracellular matrix into the cytoplasm by binding to several ligands including
osomal subunit and translational activation of ribosomes. Together with SBDS, triggers the GT

e zeta complex, an error-prone polymerase specialized in translesion DNA synthesis (TLS). Lac

ɔr Ebolavirus, possibly through GAS6 binding to phosphatidyl-serine at the surface of virion env
factor which is present in almost all cell types and is involved in many biological processed su

e receptor ICOS. Acts as a costimulatory signal for T-cell proliferation and cytokine secretion; in
he E3 ubiquitin ligase complex ZYG11B-CUL2-Elongin BC (PubMed:17304241, PubMed:3127
iquitin or ISG15 to other proteins. Functions in the E6/E6-AP-induced ubiquitination of p53/TP5
ant melanomas and in some breast cancers. May regulate events downstream of cell-matrix ac
nd adaptive immune responses. It induces various cytokines such as TNFA/TNF-alpha and IL8
cytoplasm or allow the binding of lipids to organelles.
naling downstream of TLR4 activation. It acts as a scaffold molecule to assemble a molecular
Ca(2+)-triggered synaptic vesicle-plasma membrane fusion. Plays a role in the sarcolemma rep
amino acid to its cognate tRNA in a 2 step reaction: the amino acid (AA) is first activated by AT
n-associated sorting protein (CLASP) required for clathrin-mediated endocytosis of selected car
se of the epidermal growth factor (EGF) receptor ligands and TNF, thereby plays a role in sleep
/lglycerol/DAG into phosphatidic acid/phosphatidate/PA and regulates the respective levels of t
ms a linking network between organelles and the actin cytoskeleton to maintain the subcellular
embrane depolarization or increase in cytosolic Ca(2+) that mediates export of K(+) (PubMed:2
nse to microbial agents. Specifically recognizes diacylated and triacylated lipopeptides. Cooper

ı channel that is activated by intracellular calcium (PubMed:26148990). Activation is followed b
mmunity. TLRs (Toll-like receptors) control host immune response against pathogens through ı
Catalyzes the fusion of transport vesicles within the Golgi cisternae. Is also required for transp
inactivation of the lipoprotein lipase LPL, and thereby plays an important role in the regulation c
-1,3-linked galactose residues in O-linked glycoproteins. Good substrates include asialofetuin, (
between a GDP-bound inactive and a GTP-bound active form. Involved in EGFR and CHRM3
ıat is a member of the DJ-1/PfpI gene family. This protein is overexpressed in fetal Down synd
tiviral enzyme which plays a critical role in cellular innate antiviral response (PubMed:10464285
CBP-mediated transcription complex. Acts as a bridge, linking TFAP2 transcription factors and t
tiviral enzyme which plays a critical role in cellular innate antiviral response. In addition, it may a
's costimulatory signals for the TCR/CD3 pathway.
cytoplasm or allow the binding of lipids to organelles.
es with nuclear receptors, transcriptional coactivators including EP300, CREBBP and NCOA1,
. Likely to power actin-based membrane trafficking in many physiologically crucial tissues.
membrane-associated plaques are architectural elements in an important strategic position to

olved in the inactivation of MAP kinases. Dephosphorylates MAPK10 bound to ARRB2.
ction as a coat complex required for sorting of specific membrane proteins to the primary cilia. T

n remodeling complex (nBAF complex). During neural development a switch from a stem/proge

at mediates the repairing or degradation of unhealthy mitochondria in response to mitochondria
CDK4 (DC) complex that phosphorylates and inhibits members of the retinoblastoma (RB) prot
ınose to GDP-4-dehydro-6-deoxy-D-mannose.

ntegrin and metalloproteinase with thrombospondin motifs) protein family. ADAMTS family mer
ıponent of the 2-oxoglutarate dehydrogenase complex, which mediates the decarboxylation of
egulator of the integrated stress response (ISR), a process for adaptation to various stress (Pu
otic plasticity, including neurotransmitter release and recovery, number of axonal mitochondria
PubMed:26968549). Involved in cell proliferation and tumor cell growth suppression (PubMed:2
specific endoglucosamine-6-sulfatase activity. It can remove sulfate from the C-6 position of gl

II transcription elongation past template-encoded arresting sites. The arresting sites in DNA ha
rough an autocrine and paracrine action.
essory components of the cytoplasmic dynein 1 complex that are thought to be involved in linki
-induced NF-kappa-B-dependent gene expression by regulating A20/TNFAIP3-mediated deubi
oordinate membrane transport with the function of the cytoskeleton. Involved in the internalizati

31 acts as a receptor for Human echoviruses 1 and 8.||(Microbial infection) Integrin ITGA2:ITGI
conversion of glutarate to glutaryl-CoA. Can use different dicarboxylic acids as CoA acceptors,
eration following neuronal injury by toxic insult or trauma.
fibronectin, laminin, collagen, epiligrin, thrombospondin and CSPG4. Integrin alpha-3/beta-1 pr
d in the regulation of endosome dynamics. Implicated in a novel signal transduction pathway, ir
oligosaccharide biosynthesis, the transfer of an N-acetyl-D-galactosamine residue to a serine c

n required for normal development (By similarity). Recruits channels, receptors and signaling m

-phosphohydroxypyruvate to phosphoserine and of 3-hydroxy-2-oxo-4-phosphonooxybutanoat

c effector. Regulates TNF-alpha-mediated apoptosis in a p53/TP53-dependent manner.
g IL-1 engagement, triggering intracellular signaling cascades leading to transcriptional up-regu
EF) regulating clathrin-mediated endocytosis through RAB35 activation. Promotes the exchang
DC1-mediated cross-presentation of viral and tumor antigens in dendritic cells. Mechanistically
nd cellular anti-apoptosis proteins. Can overcome the suppressors BCL-2 and BCL-XL, althoug

osomes wrap and compact DNA into chromatin, limiting DNA accessibility to the cellular machir
ated ion channel. This receptor is insensitive to the antagonists PPADS and suramin.
scaffold protein for the de novo synthesis of iron-sulfur clusters in the cytoplasm.||Scaffold prot
ved in cell innate immunity. The antiviral activity could in part be mediated by TRIM22-depende
nous extracellular matrix.

n spermatogenesis and in fertilization.
tion. Overexpression causes down-regulation of a number of genes involved in the immune res
se that specifically hydrolyzes the 5-phosphate of phosphatidylinositol-3,4,5-trisphosphate (Ptd
nterferon (IFN) antiviral response. In INF treated and virus infected cells, RNASEL probably me
lved in the dissociation of the integrin-tensin-actin complex. EGF activates TNS4 and down-re
ty acids and glycerol (PubMed:19029917, PubMed:20079333, PubMed:21049984, PubMed:22
in membrane protein-cytoskeleton interactions. It is thought to anchor the inhibitory glycine rec
osomes wrap and compact DNA into chromatin, limiting DNA accessibility to the cellular machir
; and cell process formation. Isoform 1 and isoform 2 are necessary for axonal and dendritic filo
tor beta-1 (TGFB1) specifically required for microglia function in the nervous system (By simila
onse to tissue injury and the control of extra-osseous calcification, acting as a downstream tarç
asticatory muscles contraction suspected for its homologs in dog (AC F1PT61) and apes.
alcium- and magnesium-independent cell-cell adhesion activity. May have tumor-suppressor ac
SP3-mediated apoptosis induced by TNF or danger signals, such as chemotherapy drugs, to p

e in the regulation of embryonic development, cell proliferation, cell migration, survival and che
for activated T and NK cells, enhance the lytic activity of NK/lymphokine-activated killer cells, a

a virus.
B1 and TCF family members, and acts as negative regulator of the Wnt signaling pathway.
ge factor (GEF) with specificity for RAB11A and RAB25 (PubMed:26506309, PubMed:302179
ultiprotein complex involved in the ATP-dependent degradation of ubiquitinated proteins. This
-5'-adenylyl sulfate (PAPS) as sulfonate donor to catalyze the transfer of sulfate to position 6 o
e steps of regulated exocytosis, both in endocrine and exocrine cells (By similarity). Acts as a p
ent binding of aminoacyl-tRNA to the A-site of ribosomes during protein biosynthesis.
xocytosis of secretory vesicles through Ca(2+) and phospholipid binding to the C2 domain or m
or necrosis factor type 1 receptor. The binding domain of TRAP1 and TRAP2 resides outside th
to act as a repressor. Binds also to full-length mRNA and to short RNA sequences containing t
in the signal transduction pathway that regulates endothelial cell capillary tube formation durin
nsport throughout the body. May participate in reverse cholesterol transport from peripheral ce
inase complex which is characterized by its ability to cleave peptides with Arg, Phe, Tyr, Leu, a
autophagosomal degradation of ubiquitinated targets.
t controls membrane reorganization/tubulation upon ATP hydrolysis (By similarity). Plays a role
substrate for protein kinase C. This protein binds calmodulin, actin, and synapsin. MARCKS is
um (TEA), 1-methyl-4-phenylpyridinium (MPP), cimetidine, N-methylnicotinamide (NMN), metfo

at is an intracellular signal transducer and transcriptional modulator activated by TGF-beta (tra
ight chain kinase implicated in smooth muscle contraction via phosphorylation of myosin light c
ed:30122540, PubMed:30122541). May also regulate cilium motility through its role in the asse
ptor for Pseudomonas aeruginosa exotoxin A.||Endocytic receptor involved in endocytosis and

hogenesis, polarity, adhesion and cytoskeletal organization in the lens (PubMed:26231217).
development.
events infection from non-host-adapted retroviruses. Blocks viral replication early in the life cycl

teins in the endoplasmic reticulum. Required for maturation and transport of active lipoprotein
al step in the biosynthesis of the proinflammatory mediator leukotriene B4. Has also aminopep
se activity.
he internalization of lipophilic molecules and/or signal transduction. May be involved in the upta
3-dependent induction of apoptosis. Does not bind RNA. As component of the WAVE1 comple
SOS1. May play a role in membrane ruffling and remodeling of the actin cytoskeleton. In the c

regulation of the circadian rhythm by negatively regulating the activity of the clock genes and c
ll matrix association. May play a role in the regulation of anchorage versus migration or prolifer
amina, a fibrous layer on the nucleoplasmic side of the inner nuclear membrane, which is thoug
at-containing family of proteins, which function in diverse biological pathways. This family mem
or Human Rotavirus strain Wa.||(Microbial infection) Acts as a receptor for Mammalian reovirus
l cell division and cell polarization processes. Probably involved in the formation of epithelial tig
CDK4 (DC) complex that phosphorylates and inhibits members of the retinoblastoma (RB) prot

plasmin, plasma and tissue kallikrein, and factor XIa.
egulated exocytosis (By similarity).
ion molecule (JAM) family. The encoded protein contains multiple glycosylation sites at the N-t
g alternative splicing of pre-mRNAs (By similarity). Regulates transcription and acts as corepre

orylation. Does not have choline kinase activity (By similarity).
osomes wrap and compact DNA into chromatin, limiting DNA accessibility to the cellular machir

gulation of several post-transcriptional events. Involved in pre-mRNA alternative splicing, mRN
). May play a role in the development of the retina. Has been suggested to play a role in axon
nscriptional regulation of HIV-1 gene expression.||RNA-binding protein that plays a role in the re
mal tubulin. Plays a role in anterograde intraflagellar transport (IFT), the process by which cilia
d polarity, as well as in axon growth and guidance, neuronal growth cone collapse and cell mig
hich acts as an inhibitor of retrovirus replication and retrotransposon mobility via deaminase-de
tween an Asp and an Ala, releasing the mature cytokine which is involved in a variety of inflamı
n the cytoplasm to the endoplasmic reticulum for association with MHC class I molecules. Also

x linking beta-integrins to the actin cytoskeleton, bridges the complex to cell surface receptor ty
target of rapamycin complex 1 (mTORC1) activity which regulates cell growth and survival, ar
Cdc42 small GTPases. Together with CDC42 seems to be involved in the pathway mediating
eration and promotes apoptosis. Modulates the activity of protein arginine N-methyltransferase
r (CD4 being the primary receptor) for human immunodeficiency virus-1/HIV-1 X4 isolates and
spases responsible for apoptosis execution. Cleaves and activates sterol regulatory element bi
on-secretory polypeptides to the endoplasmic reticulum (ER). May bind to nascent polypeptide
e for the structural integrity of epithelial cells and are subdivided into epithelial keratins and hai
cing cytosolic vacuolization and upregulating the autophagy pathway (PubMed:21072319). Ma
g protein that may antagonize the action of GOLPH3 which is required for the process of vesic
but also appears to possess tyrosine kinase activity (PubMed:20230784). Involved in DNA dar
ch as sphingomyelin, cholesterol and its oxygenated derivatives like 7beta-hydroxycholesterol a

ne nucleoside diphosphate derivatives.
and in adaptive immunity.
embrane-bound ephrin family ligands residing on adjacent cells, leading to contact-dependent
'ar3 complex to the GPSM1/GPSM2 complex (PubMed:16458856). Involved in spindle orientat
expression.
niting virus spread by inhibiting proliferation of virus-infected cells. Upon infection with diverse
protein that is found in the interterritorial matrix of articular deep zone cartilage. This protein is
ve damage, by catalyzing the reduction of hydrogen peroxide, lipid peroxides and organic hydr
)812024). Mediates the transcriptional repression activity of some nuclear receptors by promoti
or, laminin is thought to mediate the attachment, migration and organization of cells into tissues
iption factors (BPXs) function. Inhibits the binding of PBX1-HOX complex to DNA and blocks th
or, laminin is thought to mediate the attachment, migration and organization of cells into tissues
1,4,5)P3 around the endoplasmic reticulum.
)aran sulfate. Binds, via the heparan sulfate side chains, alpha-4 (V) collagen and participates
;av/Wts/Hpo) signaling pathway, a signaling pathway that plays a pivotal role in tumor suppress
lpha chain (PubMed:24600447). Alpha-beta T cell receptors are antigen specific receptors whi
an inhibitor of matriptase (ST14).
component of the network of molecules that regulate cellular morphology and axon guidance n
RAC-like) channel subunit which mediates Ca(2+) influx and increase in Ca(2+)-selective curre
in negative regulation of cell cycle progression.
plays an essential role in the regulation of the innate immune response, inflammation and cellu
associated with G2/M phase arrest in response to DNA damage. May be an intermediate by wh
/ is dependent on E2 enzymes, UBE2D1, UBE2D2, UBE2E1 and UBE2E2. Forms a ubiquitin li

alization and the translation of a subset of mRNA. May play a role in adipogenesis through bind
the sn-2 position of phosphatidylcholine as compared with palmitic acid.
promotes axonal growth. May play a role in nerve regeneration and in the formation and functic
ited ATPase and ATP-dependent DNA helicase (3' to 5') activity; hexamerization is thought to b
and maintenance of various skin appendages, specifically in determining shape and orientation
)se/sorbosone dehydrogenase family. The encoded protein also contains a domain that binds f
cal signals to specify territories inaccessible for growing axons.
of fibroblasts.
rombin complexes by hepatic cells (By similarity). When phosphorylated, plays a role in filame
dhesion molecule ASTN1 to the anterior pole of the cell membrane in migrating neurons. Pron
e a family of binuclear metallohydrolases that have been identified in plants, animals, and fung
tein that binds to the plus (or barbed) ends of actin monomers or filaments, preventing monom
on to nicotinic acetylcholine receptors (nAChRs) (PubMed:21252236). The proposed role as m
some transport. Serves as link between melanosome-bound RAB27A and the motor proteins M

onditions of oxidative stress. May be involved in intracellular oxygen storage or transfer.
an activator or a repressor depending on the context. Involved in BMP signaling and in the reg
) protein that may play a supportive role in the efficient coupling of mechanical stress between
ssing 2-enoyl-CoA hydratase, 3-hydroxyacyl-CoA dehydrogenase, and delta 3, delta 2-enoyl-C
l translocation step during translation elongation. During this step, the ribosome changes from
ator that mediates cellular responses to interleukins, KITLG/SCF, LEP and other growth factor
ery also functions in topologically equivalent membrane fission events, such as the budding of

lar proliferation and/or differentiation.
endogenous cytidine and 2'-deoxycytidine for UMP synthesis.
ide with the help of a quinone, such as ubiquinone-10, giving rise to thiosulfate and ultimately t
be covalently attached to target protein and subsequently leads to their degradation by the 26
ph receptors, a family of receptor tyrosine kinases which are crucial for migration, repulsion ar
, and could therefore prevent hypercalcemia.

hosphatase 1 (PP1) complexes.
initiation of the signal transduction cascade activated by the B-cell antigen receptor complex (

tal organization and tumorogenicity. Seems to be involved in a signaling transduction pathway
in and very-long-chain fatty acids (PubMed:23936004). Does not exhibit fatty acid transport ac
transcribed from the multiple endocrine neoplasia locus. This lncRNA is retained in the nucleu
tion.
x, a complex required for the translocation of transit peptide-containing proteins from the inner
the pseudoautosomal (PAR) region on chromosomes X and Y. Alternate splicing results in mu
F) and transmembrane domains. GRF zinc fingers are found in a number of DNA-binding prote

ubiquitination.

the uptake of ions.

tes gene expression by prohibiting miRNA-mediated gene suppression. Relieves miRNA repre
produce lysophosphatidic acid (LPA) and the corresponding amines. Shows a preference for 1
sized prostaglandins from cells, the transepithelial transport of prostaglandins, and the clearar

tion.
(F(1)F(0) ATP synthase or Complex V) produces ATP from ADP in the presence of a proton g

RNAs that are involved in post-transcriptional regulation of gene expression in multicellular org
eticulum-associated degradation (ERAD) for misfolded lumenal glycoproteins, but not that of m

cholesterol.
channels that contain CACNA1C as pore-forming subunit (By similarity). Regulates the trafficki
ces a short-lived long non-coding RNA. This non-coding RNA is upregulated in tumor cells and

actor, cannot regulate the expression of the same subset of genes than isoform 1. Does not ha
es 'Lys-4' of histone H3 (PubMed:17707229). H3 'Lys-4' methylation represents a specific tag f

ds heat shock promoter elements (HSE). Isoform HSF4A represses transcription while the isof
embly of mitochondrial NADH:ubiquinone oxidoreductase complex (complex I, MT-ND1) at ear
ne-associated proteins termed MAGUKs (membrane-associated guanylate kinase homologs).
lated complex which is not clathrin-associated. The complex is associated with the Golgi region
RNAs that are involved in post-transcriptional regulation of gene expression in multicellular org

lex that acts as a RNA- and heme-binding protein that is involved in the initial step of microRN
ted by the chemokine CCL5/RANTES. Probably coupled to heterotrimeric Gq proteins, it stimu

rase epsilon complex (PubMed:10801849). Participates in chromosomal DNA replication (By s
t and function. Involved in the positive regulation of voltage-gated sodium channel activity. Pro

naintenance.
e that can act both as a deacetylase or deacylase (desuccinylase, depropionylase and degluta
wo different DNA motifs: the CCAAT homology common to many promoters and the enhanced
tion.
Multiple alternatively spliced transcript variants have been transcribed from this gene and all of
repression of select genes by chromatin remodeling (alteration of DNA-nucleosome topology).
Fe-S) protein assembly (CIA) machinery. Required for the maturation of extramitochondrial Fe-
iptional complex which modulates gene expression in response to neuronal synaptic activity, p
tion.
ffinity for alpha-latrotoxin, an excitatory neurotoxin present in black widow spider venom which
n-egg fusion.

the transcription of both RNA polymerase II and III small-nuclear RNA genes. Binds to the prox

:entrosome-associated contractile fibers via its interaction with CETN2.
/ associates with the patched protein (PTCH) to transduce the hedgehog's proteins signal. Binc

so modulate alternative splicing in vitro. Represses the splicing of MAPT/Tau exon 10. May fun
n prepropeptide gene. Alternatively spliced transcript variants have been identified and they ma
rate-limiting enzyme in cholesterol biosynthesis as well as in the biosynthesis of nonsterol isop
-coupled nucleotide excision repair which allows RNA polymerase II-blocking lesions to be rapi
ınd vasodilatator agents. Numerous actions have been reported most related to the physiologic
vards RAB32, RAB33B and RAB38 (PubMed:26620560, PubMed:21808068). Regulates the tra
ation of AKT1 and AKT2.
nd ADP. The activity of this receptor is mediated by G proteins which activate a phosphatidylinc
verts transient diacylglycerol (DAG) signals into prolonged physiological effects downstream of

ces a non-coding RNA. Increased expression or amplification of this locus is associated with ca

ited transactivation by suppressing the binding between NR2C2/TR4 and the TR4-response ele
ɔMed:19852954, PubMed:28530676). Probably acts as a transition zone protein required for lo
on repair (HRR) pathway of double-stranded DNA, thought to repair chromosomal fragmentatio
on repair (HRR) pathway of double-stranded DNA, thought to repair chromosomal fragmentatio
ınd catalyzes its covalent attachment to other proteins (PubMed:22496338). Catalyzes 'Lys-11'

ıtes homophilic and heterophilic interactions. In contrast to SCARF1, it poorly mediates the binc
ıd beta-tubulin, generating side chains of glycine on the gamma-carboxyl groups of specific glu

. It is required for formation of the earliest ATP-dependent splicing complex and interacts with s
for RAB2A, RAB8A, RAB10 and RAB14. Isoform 2 promotes insulin-induced glucose transport
ıt plays a protective role in the genotoxic stress response by stabilizing transcripts of genes inv
› mitosis (G2 to M transition) by protecting the nucleus from cytoplasmically activated cyclin B1-
lgehog signaling. Required for centriole stability.
ylation of (R)-5-diphosphomevalonate to form isopentenyl diphosphate (IPP). Functions in the r
ɔle in the initiation of DNA replication, and progression of DNA replication forks. GINS complex

ıation.
core component of the circadian clock. The circadian clock, an internal time-keeping system, r
ucture.
ıing the asymmetric distribution of channels and receptors at the plasma membrane of polarize
ɔnic polypyrimidine tracts and mediates negative regulation of exons splicing. May antagonize ir

multiprotein PRC1-like complex, a complex class required to maintain the transcriptionally repr
\P) toward guanine nucleotide exchange factors like ARL2, ARL3, ARF1 and ARF6, but not for
gh concentrations of myo-inositol (an osmolyte) that result in impairment of cellular function.
ptides Phoenixin-14 and Phoenixin-20 (By similarity). It mediates the Phoenixin-14 and Phoeni:
produces long alternatively spliced non-coding RNAs. This genome region was observed to b

'ied as voltage-gated chloride channel. The presence of the conserved gating glutamate residu
encephalitis virus (JEV) and Dengue virus (DEN) RNAs.||(Microbial infection) Exhibits antiviral a

a postreplication repair or a cell cycle checkpoint function. May be involved in interstrand DNA c
some-associated) complex, a complex that plays a central role in assembly of kinetochore prot
RNAs that are involved in post-transcriptional regulation of gene expression in multicellular or

rine/threonine and tyrosine-containing substrates. Phosphorylates serine- and arginine-rich (SF
RNAs that are involved in post-transcriptional regulation of gene expression in multicellular or

: subunit of the PRC2/EED-EZH2 complex, which methylates 'Lys-9' (H3K9me) and 'Lys-27' (H
he function seems also to involve CEP63, CDK5RAP2 and WDR62 through a stepwise asseml
RNAs that are involved in post-transcriptional regulation of gene expression in multicellular or
s required for the targeting of proteins from the mitochondrial matrix, across the inner membrar
at activates non-muscle myosin IIa. Activates the non-muscle myosin IIa complex by promoting

s both HES1-mediated N box-dependent transcriptional repression and binding of HES1 to E b
s both HES1-mediated N box-dependent transcriptional repression and binding of HES1 to E b
nscription factor complexes. Inhibits NF-kappa-B activity without affecting its nuclear translocat
lly methylates monomethylated 'Lys-20' (H4K20me1) and dimethylated 'Lys-20' (H4K20me2) o

r organization of muscles cells.

PcG proteins act by forming multiprotein complexes, which are required to maintain the transc

adenosine-34 to inosine in many tRNAs.
ethionine from methionine and ATP. The reaction comprises two steps that are both catalyzed
ition.
?) and electrons from reduced cytochrome b5 to introduce the first double bond into saturated fa

ion required for the demethylation of 4,4-dimethyl and 4alpha-methylsterols, which can be subs

e initiation of X inactivation mediated by Xist RNA that occurs during embryogenesis and in lym

e, the last enzyme in the mitochondrial electron transport chain which drives oxidative phosphor
oli cells of the testis, causes regression of the Muellerian duct. It is also able to inhibit the growt
ture that promotes de novo centriole amplification in multiciliated cells that can generate more
ne that produces multiple alternatively spliced long non-coding RNAs. This gene is upregulated
core component of the circadian clock. The circadian clock, an internal time-keeping system, r

n essential component of the MAP kinase signal transduction pathway. MAPK12 is one of the f
e that participates in the import and insertion of multi-pass transmembrane proteins into the mit
cruitment to the centrosome.
tion.
ation elongation. Has an important function at the level of mRNA turnover, probably acting dow
core component of the circadian clock. The circadian clock, an internal time-keeping system, r

ade of glucocorticoid receptor (GR) transactivation at the nuclear receptor coactivator level, up
hich can arise as a result of misincorporation of dUMP residues by DNA polymerase or due to d
acts preferentially on N(8)-acetylspermidine, and also on acetylcadaverine and acetylputrescine
a 1,6-linked mannose residues from glycoproteins. Involved in the degradation of free oligosac

otes transcription repression. Promotes transcription activation in differentiated myotubes (By s
n (c-CPN) and transfers target proteins to it. Binds to nascent polypeptide chain and promotes
ts, some of which may function as non-coding RNAs. One of the transcript variants encodes a
L1/TIF-IB complex, which is involved in the assembly of the PIC (preinitiation complex) during F
tion.
ted cell cycle arrest and apoptosis by binding its transcriptional activation domain. Inhibits degr
formation and apoptosis; induces anchorage-independent growth and clonogenicity in lymphob
se that specifically hydrolyzes the 5-phosphate of phosphatidylinositol-3,4,5-trisphosphate (Ptd
h seems to be exclusively involved in the activation of NF-kappa-B and its transcriptional activit
ucleosome structure of the chromosomal fiber in eukaryotes. Two molecules of each of the four
nds to HOX or PBX proteins to form dimers, or to a DNA-bound dimer of PBX and HOX protein
n transcription between the neighboring SLX1B (SLX1 structure-specific endonuclease subunit
(F(1)F(0) ATP synthase or Complex V) produces ATP from ADP in the presence of a proton g
nplex (ORC) that binds origins of replication. DNA-binding is ATP-dependent. The specific DNA
RK pathway and induce cell transformation when overexpressed. May act as a nuclear scaffold

or the platelet-aggregating snake venom protein rhodocytin. Rhodocytin binding leads to tyrosi
RNAs that are involved in post-transcriptional regulation of gene expression in multicellular or
ay be involved in the development of multiple embryonic organs.
the surface of endosomes, where it recruits and activates the Arp2/3 complex to induce actin

ınd other cytokines through regulation on NF-kappa-B activity. Functions in the regulation of inf

ırk organization typical for stem cells, characterized by reduced mitochondrial metabolism, low

cellular signaling cascades triggered by activated receptor-type kinases. Plays a role in FGFR1
ı coupled receptor for GABA, formed by GABBR1 and GABBR2 (PubMed:9872316, PubMed:9

ıtion.
m formation, as well as a later role in formation of somite-derived chondrogenic lineages.

of the homoallylic substrate isopentenyl (IPP) to its highly electrophilic allylic isomer, dimethylal

terotrimeric G protein-coupled receptors. Binds to membranes containing phosphatidylinositol
hich modulates the activity of the pore-forming alpha subunit. Alters the functional properties of
cysteine-rich type II transmembrane proteins. These proteins localize to the endoplasmic retic
cing regulator. May regulate the mRNA splicing of genes such as CLK1. May act by regulating

rtate, 3-sulfino-L-alanine (cysteine sulfinic acid), and L-cysteate to beta-alanine, hypotaurine a

esis.
RNAs that are involved in post-transcriptional regulation of gene expression in multicellular or

iired for normal chromosome alignment and segregation and for kinetochore formation during
holipase and lysophospholipase activities, with a major role in membrane lipid remodeling and
ıit.
eceptor coupled to G proteins (PubMed:23763432, PubMed:23161870). Seems to act through
line (LPC) symporter, which plays an essential role for blood-brain barrier formation and functic
chymotrypsin- and elastase-2-like activities. The gene encoding this zymogen is expressed sp

P-ribosylation (ART) motif. It is a member of the ADP-ribosyltransferase gene family but enzy
omponents of the vertebrate eye lens.

ı negative regulator of RANKL-induced osteoclast precursor differentiation from bone marrow p

_1/TIF-IB complex, which is involved in the assembly of the PIC (preinitiation complex) during F
ılved in a RAD9A-related damage checkpoint, a pathway that is important in determining wheth
ədiates phosphorylation of extracellular proteins and endogenous proteins in the secretory path
ıomethylates 'Lys-310' of the RELA subunit of NF-kappa-B complex, leading to down-regulate I

core component of the circadian clock. The circadian clock, an internal time-keeping system, r
ration (PubMed:20869947). Increases steady-state levels and half-lives of a subset of mature r
NK) cells and T-cell functions upon binding to their non-MHC ligands. May mediate missing sel
aptor or scaffolding proteins. They provide a docking platform for the assembly of multimolecul
ermeable ion channel formed by PKD1 and PKD2 that is activated by interaction between PKD1
B-CUL3-RBX1) E3 ubiquitin-protein ligase complex required for synaptic transmission (PubMe
on of the cytoskeleton. Binds to and sequesters actin monomers (G actin) and therefore inhibits
8688764, PubMed:19969290). Induces activation of MAPK14/p38 and MAPK8/JNK MAPK cas
ı transcription between the neighboring TEN1 telomerase capping complex subunit homolog (S
luired for efficient phagocytosis of apoptotic cells. Modulates cellular glycosphingolipid and cho
esis, consist of a small 40S subunit and a large 60S subunit. Together these subunits are com
le in GPR24/MCH-R1 signaling.

cholesterol. Binds cholesterol or other sterols.
l with the transport of calcium from the cytoplasm to the extracellular space thereby maintaining
ceptor cells, the signal transduction in immune cells as a Src family kinase activator, endosome

nd is a member of the 5' terminal oligo-pyrimidine class of genes. It is a small nucleolar RNA h
factor (GEF).

g sequence. This RNA interacts with heterogeneous nuclear ribonucleoprotein U and coats ch
) cluster assembly factor that facilitates (Fe-S) cluster insertion into a subset of mitochondrial p

papillomavirus type 1 regulatory protein E2 loading onto mitotic chromosomes during DNA rep
ss-of-function mutation in this gene cause Kohlschutter-Tonz syndrome. Alternate splicing resu

A exosome complex to pre-rRNA to mediate the 3'-5' end processing of the 5.8S rRNA; this fu

sis required for efficient 40S and 60S subunit production.
eptor coactivator, enhancing transcription through other coactivators such as NCOA6 and CITE

cificity. Has transaminase activity towards aminoadipate, kynurenine, methionine and glutamate

). Inhibits platelet-derived growth factor (PDGF)-BB-induced smooth muscle cell migration and
(F(1)F(0) ATP synthase or Complex V) produces ATP from ADP in the presence of a proton gr

gulation of organogenesis. Seems to be a regulator of SIX1, SIX6 and probably SIX5. Corepre

EF) for Ras and Rap1.

snoRNA) that is predicted to function as a guide RNA in 2-prime O-methylation of 28S ribosoma

e biogenesis.

RNAs that are involved in post-transcriptional regulation of gene expression in multicellular org

sembly in response to cellular stress.||[Isoform 2]: Does not play a role in regulation of stress g
g neurons by participating in regulation of cell survival, possibly as a neurospecific transcription

pase complex which catalyzes the hydrolysis of ATP coupled to the transport of aminophosph
cyte differentiation and suppresses osteoblast differentiation in the bone marrow. Enhances th
RNAs that are involved in post-transcriptional regulation of gene expression in multicellular org

ed coil motif. No function has been determined for the encoded protein. A pseudogene of this g
RNAs that are involved in post-transcriptional regulation of gene expression in multicellular org

ns; it stimulates the secretion of both luteinizing and follicle-stimulating hormones.

RNAs that are involved in post-transcriptional regulation of gene expression in multicellular org

and catalyzes its covalent attachment to other proteins. Catalyzes monoubiquitination. Involved

ıatin structure and consequently, chromatin-dependent processes such as transcription, DNA r
ıtion.
site selection for certain mRNA precursors. Mediates regulation of pre-mRNA splicing in a PK

gulate tyrosine kinase receptor signaling. May increase IGF1 receptor phosphorylation under IG

oint family (NBPF) which consists of dozens of recently duplicated genes primarily located in se

volved in the metabolism of various endogenous substrates, including fatty acids, steroid hormo
d in tumor cells. This RNA may promote tumorigenesis by acting as a sponge for microRNAs. [
ubiquitin ligase (CRL) complexes (PubMed:21778237). May down-regulate activation of NF-ka
vity for beta-arrestin recruitment (PubMed:28827538). Shows a reciprocal regulatory interactior
ıasone-induced apoptosis. Mediates survival of postmitotic Sertoli cells by suppressing death-p
diation of Notch signaling. Involved in limb development (By similarity).
RNAs that are involved in post-transcriptional regulation of gene expression in multicellular org

in the modulation of auditory stimuli. Agonist binding may induce an extensive change in confo
nase which is necessary for the maintenance of skeletal muscle structure and function. May pl
phase-promoting complex (APC/C) during G1/S transition by mediating deubiquitination of cyc

channels.

required for phagocytosis of apoptotic cells and cell motility. Acts in association with DOCK1 a

E-cadherin. It mediates adhesion of intra-epithelial T-lymphocytes to epithelial cell monolayers
stabilization of normal baseline neurotransmitter release and for the induction and long-term
e incorporation of MCM complex into pre-replication complex (pre-RC) (PubMed:9635433, Pub
uce cell death, possibly by acting on the transcription and RNA processing of apoptosis-related

RNAs that are involved in post-transcriptional regulation of gene expression in multicellular org
phorylates 'Ser-3' in cofilin and probably also dephosphorylates phospho-serine residues in DS

transport vesicles with the Golgi complex.
location into the mitochondrial matrix. Inhibits DNAJC19 stimulation of HSPA9/Mortalin ATPase

g RNAs involved in RNA processing. Box H/ACA snoRNAs, such as SNORA81, direct the conv

ys a critical role in synaptogenesis and synaptic plasticity by providing a platform for the postsy
itter in the vertebrate brain, mediates neuronal inhibition by binding to the GABA/benzodiazepi
ıospholipids remodeling. May selectively cleave myristate (C14)-containing phosphatidylcholine
ranslocon complex that mediates transport of signal peptide-containing precursor polypeptides
r or repressor (PubMed:14671321). Inhibits several cytokine signaling pathways, such as TGFE

RNAs that are involved in post-transcriptional regulation of gene expression in multicellular org

chromosome segregation.
nbination. Functions in the recombinational DNA repair (RAD52) pathway. Dissociates RAD51

ar. It is not known if it has protein kinase activity and what type of substrate it would phosphory
PP1. PP1 is involved in glycogen metabolism and contributes to the activation of glycogen syn

for IL19, IL20 and IL24. The IL22RA1/IL20RB dimer is a receptor for IL20 and IL24.

eric heterochromatin in interphase nuclei and for centromere formation and organization, chrom
A small nucleolar RNAs, characterized by a consensus ACA motif positioned 3 nucleotides fro
D) is the pathway by which misfolded proteins in the endoplasmic reticulum are targeted to the

(TST) required to produce S-sulfanylglutathione (GSS(-)), a central intermediate in hydrogen s

double bond of sterol intermediates during cholesterol biosynthesis (PubMed:11519011, PubM
nactivation center (XIC). It produces a spliced long non-coding RNA that is thought to positively

e ID: 10450). Expression of this pseudogene may be downregulated in non-small cell lung canc

dependent transcription possibly by interfering with CREBBP-dependent coactivation. May func

ates mono-ADP-ribosylation of target proteins.
duced retinal lens fiber differentiation, probably by inhibiting FGF-mediated phosphorylation of

nsactivates the TSHB promoter. Binds to a minimal DNA-binding sequence 5'-[TC][AG][AG]TT
s in association with cyclin-dependent kinases (CDKs) (PubMed:18216018). Inhibited by the C

hannel. May also facilitate the permeation of sodium ions.
nembranes associated with microglial activation following brain lesion.

s specifically to Cajal bodies, which are conserved subnuclear organelles that are present in th

lay a role in cell adhesion.
ansit peptide-containing proteins across the mitochondrial inner membrane. the PAM complex (
r noncoding small nucleolar RNAs (snoRNAs), like SNORD43, which are generated during pre

protein. May be involved in modulating cellular levels and biological functions of estrogens in t
noter) transcription factor binds to the ovalbumin promoter and, in conjunction with another pro
a subfamily of the cadherin superfamily. The gene encodes a protein with an extracellular doma

on protein.

ms to play a role in spindle dynamics and cell division (PubMed:30715179, PubMed:30447097
ition.
hioribose-1-phosphate (MTR-1-P) into methylthioribulose-1-phosphate (MTRu-1-P). Independe
e (OST) complex that catalyzes the initial transfer of a defined glycan (Glc(3)Man(9)GlcNAc(2)
RNAs that are involved in post-transcriptional regulation of gene expression in multicellular or
RNAs that are involved in post-transcriptional regulation of gene expression in multicellular or
)-independent manner to the fast growing ends of actin filaments (barbed end) thereby blockin
Y6) genes located in the major histocompatibility complex (MHC) class III region on chromoso
s as a regulator of Sonic hedgehog (SHH) signaling and autophagy. Acts as a negative regulat

and tumorigenesis. Involved in clathrin-dependent endocytosis.||May function as signaling mo
gen matrix deposition.
synthetase, which catalyzes the attachment of valine to tRNA(Val) for mitochondrial translation.

d for calcium-dependent dendritic growth and branching in cortical neurons. Recruits CREB-bi

l activity and adiposity.

eural progenitor proliferation and evolutionary expansion of the brain neocortex by regulating th
ports pyrimidine nucleotides into and from mitochondria (PubMed:25320081). Transports pref
ases. As a GCM1 downstream effector, it is involved in placental development and positively re

DNA damage.

version of lysophosphatidylserine (1-acyl-2-hydroxy-sn-glycero-3-phospho-L-serine or LPS) int
(NHEJ), a major pathway to repair double-strand breaks in DNA. May act as a scaffold require

ɔrt of Golgi vesicles to the endoplasmic reticulum. Has a microtubule plus end-directed motility.

ndothelium differentiation and vascular morphogenesis through the ACVRL1 receptor-depender
/ role in germ cell development. Determines the oogenic fate by activating key genes for the oc
ooperatively with DP proteins through the E2 recognition site, 5'-TTTC[CG]CGC-3' found in the
activity. The exact function of this protein is not known. Studies with chimeric proteins (replacin
annels (PubMed:1309651, PubMed:8107964, PubMed:15615847). Regulates the activity of L-t

for centrosome separation and productive cytokinesis during cell division. Dephosphorylates SI
al regulator of cellular and organ growth, development, migration, differentiation and survival ar

maintain the centrosome integrity in interphase. Suppresses centrosome disjunction by inhibiti

sation for the expression level of X-linked genes from the single X in males and the two in fema
nc finger transcription factors EGR1 and EGR2. Isoform 2 lacks repression ability (By similarity
mology (CH) domain, a PDZ domain, and a LIM domain, and may be involved in protein-protei
ıtion.
ɛ, the last enzyme in the mitochondrial electron transport chain which drives oxidative phosphor
ɔort of large neutral amino acids. Has narrower substrate selectivity compared to SLC7A5 and
ɜ.
ptional repressor. Represses target gene transcription by recruiting HDAC1 and HDAC2 histon
ıllin components of SCF-type E3 ubiquitin ligase complexes from the NEDD8-conjugating E2 e
a long non-coding RNA is derived by RNase P cleavage of a tRNA-like small ncRNA (known a

xchange activity for CDC42 (By similarity). May be a guanine-nucleotide exchange factor (GEF

ntiapoptotic factor.
vation by cytokines and lipopolysaccharide (LPS).
nt retrograde axonal transport. May function as the motor for the transport of multivesicular bod
noglobulin light chains that participates in the antigen recognition (PubMed:24600447). Immun
transmembrane 4 superfamily, also known as the tetraspanin family. Most of these members a
omiscuously GPI-anchored ephrin-A family ligands residing on adjacent cells, leading to contac
l in energy homeostasis and protein translation. Phosphorylates EEF1A1, GYS1, PDX1 and RI
essory components of the cytoplasmic dynein 1 complex that are thought to be involved in linki
nules and secretion of hormones in the pancreas and the pituitary. Interacts with vesicles conta

s regulators of cell proliferation. Alternative promoter usage and splicing results in multiple trans
ignaling activity that activate cyclic AMP. Has a potential role in modulating a number of brain fu
abolism of plasma lipoproteins. Synthesized mainly in the liver and secreted into plasma where
phology and cytoskeletal organization. Required in the control of cell shape.
eta-1,4-N-acetylgalactosamine transferase activity. Transfers glucuronic acid (GlcUA) from UD
nplex, a complex that stimulates the recombination-dependent repair of stalled or collapsed rep
oreceptor LRP6, leading to increased activity of the canonical Wnt signaling pathway (PubMed:
g protein that may play a role in calcium-mediated intracellular processes.
ional activation of early and late herpes simplex virus 1/HHV-1 promoters.||Regulatory subunit
kpoint response complex that plays a major role in DNA repair. The 9-1-1 complex is recruited t

ptic vesicles.
osis of ATP. May accumulate ATP and other nucleotides in secretory vesicles such as adrenal
n the endoplasmic reticulum and the Golgi apparatus.
Med:24359566). May play a role in myeloid maturation and in the development and/or mainten
May play a role in inflammation and exerts its effects on endothelial cells in an autocrine fashio
ation as a repressor. Plays a role in limb development.
s 'Lys-6'- and 'Lys-11'-linked polyubiquitin. Also hydrolyzes heterotypic (mixed and branched) an
r noncoding small nucleolar RNAs (snoRNAs), like SNORD83B, which are generated during pr
complex that is required for normal biogenesis of lysosome-related organelles (LRO), such as p
f some histone methyltransferase complexes may regulate transcription through recruitment of
protein]: May function as a ligand for MHC class II (MHC-II) on antigen-presenting cells (APC),
afficking of membrane cargo, including EGFR.
hosphatase with broad substrate specificity (PubMed:17590538). Preferentially catalyzes the c
superfamily, a novel family that is similar to the chemokine and the transmembrane 4 superfam
icarbonate exchanger. Its association with carbonic anhydrase CA2 forms a bicarbonate transp

erization of proline imidic peptide bonds in oligopeptides and may therefore assist protein foldi

mone secreted by adipocytes that regulates glucose and lipid metabolism (PubMed:12802337,

le in the assembly of the Z-disk. May functionally link sarcomeric actin to the desmin intermedia

C-terminal region is highly homologous to the bornavirus nucleocapsid N protein that binds vir

specifically trimethylates 'Lys-315' of VCP/p97; this modification may decrease VCP ATPase a
ng of cell surface receptor kinases with specific signaling pathways. Binds to, and suppresses s
or Hendra virus and Nipah virus.||Cell surface transmembrane ligand for Eph receptors, a famil

the morphological alterations required for cell growth and motility. Interaction with ARF6 may r
ibosylation factor family.
RNAs that are involved in post-transcriptional regulation of gene expression in multicellular org
l in an inactivated (closed) state. Modulates calcium current when coexpressed with CACNA1G

und to HCFC1. Suppresses the expression of HSV proteins in cells infected with the virus in a
de-ranging physiologic effects, including the modulation of morphine-induced analgesia, elevati
ition.
pression of ESR1 target genes. Can act in cooperation with MED1.
inity for alpha-latrotoxin, an excitatory neurotoxin present in black widow spider venom which t
of S.aureus adherence to desquamated nasal epithelial cells via clfB, and hence may play a ro
rotein. This interaction is important in mediating thymocyte interactions with thymic epithelial ce
wo opposing regulatory activities toward small GTP-binding proteins. The C-terminus is a GTPa
euromuscular junction by rapid hydrolysis of the acetylcholine released into the synaptic cleft. R
ught to anchor actin to a variety of intracellular structures. This is a bundling protein.
adenosine and 2-deoxyadenosine (PubMed:8452534, PubMed:16670267). Plays an important
lex 2 (AP-2). Adaptor protein complexes function in protein transport via transport vesicles in d
or human coronavirus 229E/HCoV-229E. In case of human coronavirus 229E (HCoV-229E) inf
esses anti-coagulant properties. Also cleaves the cyclic bond of inositol 1,2-cyclic phosphate to
loid-beta precursor protein.
hology changes during cell spreading. In the retinal pigment epithelium, may regulate the bioge
-urea cycle arginine metabolism and also in down-regulation of nitric oxide synthesis. Extrahep
y members (PubMed:8537347).
sulfatase family. Sulfatases are essential for the correct composition of bone and cartilage ma
ition of cartilage and bone matrix during development. Has no activity toward steroid sulfates.
pressor or activator, depending on the context. Binds to NF-E2 DNA binding sites. Plays import
vation. May function in the response of hemopoietic cells to external signals and in maintaining
scriptional program in various innate and adaptive immune tissue-resident lymphocyte T cell ty
central but diverse modulatory roles in various signaling processes involved in the regulation of
ctions through different apoptotic signaling pathways (PubMed:27076518, PubMed:15102863,
stabilizes several cytoplasmic AU-rich element (ARE)-containing mRNA transcripts by promotin
clic ADP-ribose and nicotinate-adenine dinucleotide phosphate, the former a second messeng
ated in the regulation of actomyosin interactions in smooth muscle and nonmuscle cells (could

ıly blocks the barbed ends of actin filaments but does not sever preformed actin filaments. May
ɔrotease which catalyzes limited proteolysis of substrates involved in cytoskeletal remodeling aı
ıspases responsible for apoptosis execution. At the onset of apoptosis it proteolytically cleaves ı
aveolar membranes (PubMed:11751885). Forms a stable heterooligomeric complex with CAV2
ːment in spermiogenic differentiation.
n early hematopoiesis by mediating the attachment of stem cells to the bone marrow extracellu
ɔr enterovirus 71.||Acts as a lysosomal receptor for glucosylceramidase (GBA) targeting.
ATP and other nucleotides to regulate purinergic neurotransmission. Could also be implicated i
cell-cell interactions, cell adhesion and migration, helping them to sense and respond to chang
ide]: Binds to the peptide-binding site of MHC class II alpha/beta heterodimers forming an alph
duction and vesicle trafficking to enable polarized secretion and/or membrane deposition of imı
n; preferentially mediates homotypic cell-cell adhesion by dimerization with a CDH2 chain from
dhesion proteins (By similarity). They preferentially interact with themselves in a homophilic ma
dhesion proteins. They preferentially interact with themselves in a homophilic manner in conne
dhesion proteins. They preferentially interact with themselves in a homophilic manner in conne
sponds by an extensive change in conformation that affects all subunits and leads to opening ‹
lyl-peptidase I activity (PubMed:11054422, PubMed:19038966, PubMed:19038967). May act a
component of glomerular basement membranes (GBM), forming a 'chicken-wire' meshwork tog
component of glomerular basement membranes (GBM), forming a 'chicken-wire' meshwork tog
collagen (fibrillar forming collagen). It is a minor connective tissue component of nearly ubiquit‹
ɔllagen-containing fibrils, the COL1 domain could be associated with the surface of the fibrils, a
folded protein response (UPR) pathway by activating UPR target genes induced during ER stre
he regulation of survival, proliferation and differentiation of hematopoietic precursor cells, espe
ːnt.
ɔipate in intracellular degradation and turnover of proteins (PubMed:12220505). Cleaves matrix
one resorption and may participate partially in the disorder of bone remodeling. Displays poten
normal cellular protein degradation and turnover.
ɔr adenovirus type C.||(Microbial infection) Acts as a receptor for Coxsackievirus B1 to B6.||Coı
'Lys-63'- and linear 'Met-1'-linked polyubiquitin chains and is involved in NF-kappa-B activation
d in the regulation of apoptosis, autophagy, transcription, translation and actin cytoskeleton reɑ
avirus IBV replication.||(Microbial infection) Required for HIV-1 Rev function as well as for HIV-ʼ
by dephosphorylating both Thr and Tyr residues on MAP kinases ERK1 and ERK2.
tive with phosphotyrosine, phosphoserine and phosphothreonine residues. The highest relative
phosphatase substrates and negatively regulates mitogen-activated protein kinase activity, pre
ʼ1.
osine 1-phosphate (S1P). S1P is a bioactive lysophospholipid that elicits diverse physiological
ːein with F-actin-binding activity that induces F-actin bundles formation and stabilization. Its F-a
omiscuously transmembrane ephrin-B family ligands residing on adjacent cells, leading to cont

on. Binds specifically the DNA GGAA/T core motif (Ets-binding site or EBS) in gene promoters
NA sequences containing the consensus pentanucleotide 5'-CGGA[AT]-3'.
biosynthesis of heparan sulfate (HS). Important for both skeletal development and hematopoie
ymes coupled to G proteins (PubMed:28445455). Its function is mediated through the activatio
e that participates in extracellular matrix degradation and involved in many cellular processes i
zation, directed cell migration and modulating cell-cell contact.
bisphosphate to fructose 6-phosphate in the presence of divalent cations, acting as a rate-limit
3R1, the TGF-beta type I serine/threonine kinase receptor, preventing TGF-beta receptor activa
the actin cytoskeleton. May promote orthogonal branching of actin filaments and links actin fil
entral role in muscle cells, probably by functioning as a large actin-cross-linking protein. May be
ell-surface receptor for VEGFC and VEGFD, and plays an essential role in adult lymphangioge
it plays a role in many biological processes including regulation of cell growth and survival, cell
using S-adenosylmethionine as the methyl donor (PubMed:26003046, PubMed:24415674, Pub
velope phosphatidylserine to the TAM receptor tyrosine kinase Axl to mediate viral entry by apo
ve cleavage reactions. Exhibits antiviral activity against influenza virus. Promotes oxidative killin
ve cleavage reactions, but the major reaction product is GDP (PubMed:8706832). Exhibits antiv
f GTP cyclohydrolase 1. This inhibition is reversed by L-phenylalanine.
xtracellular glutathione (gamma-Glu-Cys-Gly), glutathione conjugates, and other gamma-glutan
ide channels. GLRB does not form ligand-gated ion channels by itself, but is part of heteromeri
date several single chain phospholipids and fatty acids, GM2A also exhibits some calcium-inde
proteins) are involved as modulators or transducers in various transmembrane signaling syster
e density of dopamine receptors DRD2 by sequestrating them as an intracellular pool.||Guanin
ates preferentially the activated forms of a variety of G-protein-coupled receptors (GPCRs). Su
it replication of hepatitis C virus (HCV) by promoting the interaction between VAPA and HCV vi

in human erythrocyte membranes. The blood group Gerbich antigens and receptors for Plasm
chemotactic activity for both monocytes and neutrophils.||Thrombin inhibitor activated by the g
T-bet-dependent maturation of Th1 cells as well as maintenance of Th1-specific gene expressi
groove of A+T rich regions in double-stranded DNA. It is suggested that these proteins could f
nbryonic development, establishment of tissue-specific gene expression and regulation of gene
Plays an important role in degrading histamine and in regulating the airway response to histam
ed:10816573). Stimulates ATP hydrolysis, but not the folding of unfolded proteins mediated by
guidance of migrating neurons as well as axons during development, synaptic plasticity as wel
/ be involved in signal transduction. Involved in regulation of the catalytic activity of the phospha
of T-lymphocytes (PubMed:8178155). Stimulation by IL15 requires interaction of IL15 with cor
s a receptor for laminin on platelets (By similarity). Integrin alpha-6/beta-1 (ITGA6:ITGB1) is pre
atory region of type I IFN and IFN-inducible MHC class I genes (the interferon consensus seque
ase that acts on single-stranded RNA and also has minor activity towards single-stranded DNA

t is activated by phosphatidylinositol 4,5-bisphosphate and that probably participates in controll
ator for membranous organelles.
or, laminin is thought to mediate the attachment, migration and organization of cells into tissues
or, laminin is thought to mediate the attachment, migration and organization of cells into tissues
or, laminin is thought to mediate the attachment, migration and organization of cells into tissues
oattractant (PubMed:9642261). It also inhibits angiogenesis (PubMed:24333696). Suppresses
ne A (CoA) desaturase that introduces a cis double bond between the preexisting double bond
mor suppressor. Altered expression of this protein and disruption of the molecular pathway it is
xidative deamination of peptidyl lysine residues in precursors to fibrous collagen and elastin (Pu
s.
deamination of lysine residues on target proteins leading to the formation of deaminated lysin
adhesion in maintaining cell shape and motility. In addition to these structural functions, it may
elated proteins that may regulate their ligand binding activity along the secretory pathway.
May play a specific role in immune response regulation. Provides the membrane anchor for th
h antiproliferative properties and ability to induce cell cycle arrest. Binds to the DeltaLf respons
MP (bone morphogenetic proteins) type 1 receptor kinase. SMAD1 is a receptor-regulated SM
esion of the endothelial monolayer at intercellular junctions in vascular tissue. Its expression m
ecifically to the MEF2 element, 5'-YTA[AT](4)TAR-3', found in numerous muscle-specific, growt
or Listeria monocytogenes internalin InlB, mediating entry of the pathogen into cells.||Receptor
crofibrils.
er elongation complex (SEC), a complex required to increase the catalytic rate of RNA polyme
t one site in the helical domain. Also cleaves collagens of types VII and X (PubMed:2557822, I
e I, III, IV, and V; weakly collagens III, IV, and V. Activates procollagenase.
ssion of epithelial malignancies.
omponents of the extracellular matrix. May activate progelatinase A.
omponents of the extracellular matrix, such as aggrecan and cartilage oligomeric matrix protein
gulate myeloid cell activity in a variety of tissues.
or Dengue virus envelope protein E.||(Microbial infection) Acts as phagocytic receptor for bacte
nzyme for proteins that have been inactivated by oxidation. Catalyzes the reversible oxidation-re
ith MAX to form a sequence-specific DNA-binding protein complex which recognizes the core s
and trapping it in the cytoplasm. Inhibits DNA-binding of NF-kappa-B p50-p65 and p50-c-Rel c
core component of the circadian clock. The circadian clock, an internal time-keeping system, re
protein kinase C-mediated molecular cascades during synaptic development and remodeling. I
membrane permeable nucleosides. Exhibits AMP-, NAD-, and NMN-nucleosidase activities.
is unlikely to function as a tyrosine kinase in vivo (PubMed:25029443). Receptor for ligand WN
amine. After binding to L-DOPA, stimulates Ca(2+) influx into the cytoplasm, increases secretio
tiviral enzyme which plays a critical role in cellular innate antiviral response. In addition, it may

ys a role in a variety of different signaling pathways including cytoskeleton regulation, cell migra
post-translational modification of inactive peptidylglycine precursors to the corresponding bioact
drial cAMP levels and respiration (By similarity). Involved in the regulation of mitochondria morp
by phosphorylation of the E1 subunit PDHA1, and thereby regulates glucose metabolism and
nst apoptosis.||Cell adhesion molecule which is required for leukocyte transendothelial migratic
pase complex which catalyzes the hydrolysis of ATP coupled to the transport of aminophosph
,6-bisphosphate.
ntegrin-binding ligand, N-linked glycoprotein)-derived ASARM peptides, thus regulating their bi
ys-Gly- sequences in collagens. These hydroxylysines serve as sites of attachment for carbohy
microvilli, hair cell stereocilia, and fibroblast filopodia. May play a role in the regulation of bone
nd free cholesterol from triglyceride-rich lipoproteins (low density lipoproteins or LDL and very l
518, PubMed:20877282, PubMed:21912513). Plays a role in GABAergic synapse development
e substrate selectivity and catalytic activity, and also might direct the localization of the catalytic
inase complex which is characterized by its ability to cleave peptides with Arg, Phe, Tyr, Leu, a
ups in peptide and protein thiols to disulfides with the reduction of oxygen to hydrogen peroxide
ve regulator of FceRI-mediated signal transduction leading to cytokine production and degranul
esses an intrinsic protein tyrosine phosphatase activity (PTPase) and dephosphorylates EPHA2
mophilic interactions. May play a key role in signal transduction and growth control.
saminoglycans, including chondroitin sulfate proteoglycans and heparan sulfate proteoglycan (
TPase which cycles between an active GTP-bound and inactive GDP-bound state. In active stat
riety of cellular processes including gene expression, cell migration, cell proliferation, oncogenic
otide exchange factor activating both Ras and RAC1 through the exchange of bound GDP for G
olgi compartment and as a clearance receptor on the cell surface. Required for protein transpo
volved in calcium-dependent cuticle cell differentiation, hair shaft and hair cuticular barrier forma
eptor CCR2 (PubMed:9837883, PubMed:10587439, PubMed:10529171). Signals through bindi
tes and eosinophils, but not neutrophils. Augments monocyte anti-tumor activity. Also induces
eptor CCR6. Signals through binding and activation of CCR6 and induces a strong chemotactic
. Signals through binding and activation of its receptors (CXCR1 and CXCR2). In addition to its
ntegrins. Binds to CX3CR1 (PubMed:23125415, PubMed:9931005, PubMed:21829356). Binds
or enterovirus 71.||A SLe(x)-type proteoglycan, which through high affinity, calcium-dependent
) function as modulators of Wnt signaling through direct interaction with Wnts. They have a rol
d to galactose-containing acceptor substrates.
jor actin binding sites (PubMed:21685497, PubMed:23184945). Organizes filamentous actin in
ch are normally produced within the cells and which are toxic to biological systems.
: the consensus sequence 5'-ACCAAAG-3' (By similarity). Acts as a transcriptional activator (By
x interactions. May contribute to various neuronal mechanisms in the central nervous system.
onal membrane skeleton.
ich is activated following engagement of many different classes of cellular receptors including i

:ween the actin cytoskeleton and the membrane. Is among the first costameric proteins to asse
; I with transporter associated with antigen processing (TAP) and in the assembly of MHC class
:h as collagenases) and irreversibly inactivates them by binding to their catalytic zinc cofactor. I
) a number of transcription factors. Inhibits the transcriptional activation mediated by PAX5, and
:oclast activity (By similarity). Involved in interleukin-4-induced M2 macrophage polarization (By
fic obliteration of the intercellular space.
'B-CUL3-RBX1) E3 ubiquitin-protein ligase complex involved in regulation of cytoskeleton struc
vation of NF-kappa-B and JNK. Plays a role in the regulation of cell survival and apoptosis. The
ation of NF-kappa-B and MAP kinases, and plays a central role in the regulation of B-cell surviv
at links members of the tumor necrosis factor receptor family to different signaling pathways by
ith ATP its C-terminal glycine residue and thereafter linking this residue to the side chain of a c
ind catalyzes its covalent attachment to other proteins (PubMed:8132613, PubMed:17588522,
Golgi, the initial step of the glucosylceramide-based glycosphingolipid/GSL synthetic pathway,
leton to the plasma membrane.
of type I collagen mRNAs for CO1A1 and CO1A2.||Vimentins are class-III intermediate filamen
y endocytosis. In order to be internalized, the receptor-ligand complexes must first cluster into
amellar body (DLB), a membranous organelle predominantly present in bulbous dendritic appe
y of seven transmembrane receptors. Functions in the canonical Wnt/beta-catenin signaling pa
s located at the pseudoautosomal boundary on the short (p) arm of chromosome X. The three
biquitin-proteasome system. May act by anchoring ubiquitinated proteins to the proteasome. P
lity.
g embryogenesis and in adult hematopoietic cells.
kely to participate in H(2)O(2) metabolism and peroxidative reactions in the cardiovascular syst
teroid receptors such as NR3C1, NR3C2 and ESR1. Also modulates transcriptional repression
an important downstream effector of CDC42 and plays a role in the regulation of cytoskeleton
ited signaling and functions both in regulating cell migration and immune responses. Promotes
e proteolytic action may subserve structural reorganizations associated with learning and mem
longitudinal organization of myelinated axons. Plays a role in the formation of functional distin
of proteins that may be involved in vesicular transport. A similar protein in mice inhibits the calci
initiation factor 3 (eIF-3) complex, which is required for several steps in the initiation of protein
e biliary and intestinal excretion of organic anions. Acts as an alternative route for the export of
g of beta-type neuregulin isoforms which are involved in neurogenesis and synaptogenesis, sug
methyltransferase RNMT:RAMAC complex that methylates the N7 position of the added guano
ILR1, TNFRSF10B/TRAILR2, TNFRSF10C/TRAILR3, TNFRSF10D/TRAILR4 and possibly als
ytotoxic ligands TNFS14/LIGHT, TNFSF15 and TNFSF6/FASL. Protects against apoptosis.
10/TRAIL (PubMed:10549288). The adapter molecule FADD recruits caspase-8 to the activate
of embryonic development, cell proliferation and cell differentiation, and is required for normal
amics and assembly of the actin cytoskeleton. Binds to activated CDC42 but does not stimulat

F5 and ARF6. Regulates the formation of post-Golgi vesicles and modulates constitutive secre
GM3 (alpha-N-acetylneuraminyl-2,3-beta-D-galactosyl-1, 4-beta-D-glucosylceramide).
e macroautophagy (aggrephagy). Functions as a bridge between polyubiquitinated cargo and a
itiation factor 2-bound GDP for GTP.
l cycle at the G1/S (start) and G2/M (mitosis) transitions. May primarily function in the control of
tors in vesicle trafficking (PubMed:24788816). Essential for maintaining the integrity of the endo
. Activates specific Rho GTPase family members, thereby inducing various signaling mechanis
eleton. May regulate WAS actin-bundling activity. Bridges the interaction between ABL1 and P
ter. Catalyzes the rapid transport across the plasma membrane of many monocarboxylates su
May indirectly regulate the activity of the plasma membrane dopamine transporter SLC6A3 an
both serine/threonine and tyrosine kinase activities. Enhances the transcriptional activity of TC
cell-cell junctions. May regulate acid-induced ASIC3 currents by modulating its expression at t
onal cells.
al to all-trans-retinol in the presence of NADPH.
ic expression augments motility of gliomas. Promotes also axonal regeneration (By similarity).
aling pathway. It associates with inactive heteromeric TGF-beta and activin receptor complexes
l glycine residue of certain cellular and viral proteins.
receptor (AR) and serum response factor (SRF). Contributes to the regulation of cell adhesion
ens junctions.
nd histone H3K9me3, histone H3K27me2 and histone H3K27me3, due to the presence of a N
-5'-adenylyl sulfate (PAPS) as sulfonate donor to catalyze the transfer of sulfate to position 6 o
role in the response to environmental stress and cytokines such as TNF-alpha. Appears to act
. Binds and cross-links cytoplasmic regions of GRM1, GRM5, ITPR1, DNM3, RYR1, RYR2, SH
of scaffold proteins selectively mediates JNK signaling by aggregating specific components of
can, and may be involved in its turnover. May play an important role in the destruction of aggre
d activates transcription.
tivity of SIAH1 and inhibits proteasomal degradation of target proteins. Isoform 2 inhibits autoub
and podosome formation, extracellular matrix degradation and invasiveness of some cancer ce
vesicles to the trans-Golgi network (TGN) and regulates vesicular transport between the endos
otide exchange factor activity compared to other isoforms.||Functions as a guanine nucleotide e
ation of dendritic spines, probably via regulating SIPA1L1 levels at the postsynaptic density of s
tumor metastasis in a variety of organ sites, most likely through an interaction with the actin cy
eases both the extent of secretion and the sensitivity of mast cells to stimulation with calcium (B
for small GTPase RAB22A, converting active RAB22A-GTP to the inactive form RAB22A-GDP
nase (IKK) regulation which constitutively binds TBK1 and IKBKE playing a role in antiviral inna
BimL is more potent than isoform BimEL. Isoform Bim-alpha1, isoform Bim-alpha2 and isoform
okine-induced, integrin-mediated T lymphocyte adhesion and migration, acting upstream of RA
phosphorylates CTNNB1. Regulates CTNNB1 function both in cell adhesion and signaling. May

mediated by association with deadenylase subunits of the CCR4-NOT complex (PubMed:8632
in the transmission of signals from tyrosine kinase receptors and small GTPases to the actin cy
nse to Gram-positive bacteria and fungi. Specifically recognizes diacylated and, to a lesser exte
tory system and in neuronal control of puberty. Induces the collapse and paralysis of neuronal
daptive immune response.
everal transient phosphorylation sites and PEST motifs. Conservation of proteins with PEST se

/). Plays a part in calcium-regulated keratinocyte activation in epidermal repair mechanisms. Ha
entral nervous system development.
phorins and subsequent remodeling of the cytoskeleton. Plays a role in axon guidance, neuror
the actin cytoskeleton. May act downstream of CDC42 to induce actin filament assembly leadi
al role in the organization of autophagic response and ubiquitination upon lysosomal and phag
dhesion through its different partners. During development plays a role in blood and lymphatic
-arrested cells only, inhibits S phase.||Recruited and tyrosine phosphorylated by several recept
plasmic tyrosine kinases to the Ras signaling pathway. Plays a role in the inner ear and in heari
'Lys-4' of histone H3, thereby playing a central role in histone code (PubMed:24952722, PubM
polarity and epithelial lumen formation (By similarity). Plays also an essential role in ciliogenesi
a role in signal transduction.
nzymes that catalyze the hydrolysis of acyl-CoAs to the free fatty acid and coenzyme A (CoAS
e adaptive immune response. Regulates the proliferation of activated T-cells. Regulates the rel
protease which catalyzes limited proteolysis of substrates involved in cytoskeletal remodeling ar
the degradation of COMP.
in kinase A. May be involved in establishing polarity in signaling systems or in integrating PKA-
afficking.
ely distributed in basement membranes. Binds to collagens I and IV, to perlecan and to laminin
P pathway. Binds inositol tetrakisphosphate (IP4) with high affinity. Might be a specific IP4 rece
poptosis. May be involved in detachment-mediated programmed cell death. May mediate apopt
nger, a motif present in a variety of functionally distinct proteins and known to be involved in pro
ation of synapses and neuronal cell signaling. Could be an adapter protein linking ion channel
II (COPII) which promotes the formation of transport vesicles from the endoplasmic reticulum (
and activates transcription. Binds to the canonical E box sequence 5'-CACGTG-3'. Plays a role
to-lysosome trafficking, including endocytic trafficking of EGF-EGFR complexes and GABA-A r
r complex) for C1q, mannose-binding lectin (MBL2) and pulmonary surfactant protein A (SPA).
in that promotes endoplasmic reticulum-associated endosome fission (PubMed:30220460). Lo
emethylates 'Lys-9' of histone H3, thereby playing a role in histone code. Does not demethylate
-containing messengers.
rcinoma (HCC) cells through interaction with SIAH1, a mediator of apoptosis. May also have a r

d CDC42.
ꓥF6. Promotes MAP3K7 activation in the IL1 signaling pathway. The binding of 'Lys-63'-linked p
for Rab family protein(s).
netabolism of fatty acids at different levels. Acts as a magnesium-dependent phosphatidate pho
factor.
CF (SKP1-CUL1-F-box protein) E3 ubiquitin-protein ligase complex (PubMed:25778398). Durir
that antagonizes canonical Wnt signaling by triggering lysosome-dependent degradation of Wı
ogenesis. Binds to microRNAs MIR7-1, MIR16-2 and MIR29A hairpins recognizing the 'ATA(A/
sitively regulates ciliary Hedgehog (Hh) signaling (By similarity). Required for proper limb morph
oprotein (RNP) complex that is essential for the replication of chromosome termini (PubMed:19
which promotes the processing of GLI3 into GLI3R during neural tube development. Recruited
bic molecules and promotes their transfer between the different cellular sites. Binds with high a
native splicing events by binding to 5'-UGCAUGU-3' elements. Prevents binding of U2AF2 to th
on of the alpha 2(I) collagen gene where it binds to the single-stranded polypyrimidine sequenc
A4D and SEMA4G that plays an important role in cell-cell signaling (By similarity). Plays a role
ation and axon guidance. Mediates cell-cell adhesion via its interactions with ADGRL3 and prol
ns and the hemichannels involved in the ATP release and nucleotide permeation (PubMed:169

to 6-phosphogluconate.
glutamyl peptides. Has a bias against acidic and tryptophan residues adjacent to the N-termina
ng of the amyloid precursor protein (APP). Cleaves APP, between residues 690 and 691, leadi

ophic peptide (BINP), a synthetic 13-mer peptide.
on factor involved in defense response to virus and as a regulator of DNA end resection at stall
ether with RECK to enable brain endothelial cells to selectively respond to Wnt7 signals (WNT

f signaling by receptor tyrosine kinases, through a mechanism that involves enhancement of re
RF in oligodendrocyte differentiation and myelination. Interacts with the C-terminal of MYRF inl
associates with both cell and endoplasmic reticulum (ER) membranes (PubMed:16143324). Ca
ATG16L1 complex to omegasomes and preautophagosomal structures, resulting in ATG8 famil
ediate the monomeric transport of lipids by shielding a lipid from the aqueous environment and
gnaling cascades, including signaling via muscarinic acetylcholine receptor CHRM2 and dopan

ne promoter region of target genes. Plays a role in the regulation of the cell cycle and of the Wr
nate (PIP2). May play a role in the organization of nuclear PIP2, cell division and cell survival (F
inhibiting LRP5/6 interaction with Wnt and by forming a ternary complex with the transmembra

way that involves the activation of NF-kappa-B. Can also promote apoptosis mediated by BAX
fic kinases. Enhances CDK1 activity. May contribute to the regulation of the cell cycle. May inh
and inflammation. Promotes caspase-mediated apoptosis involving predominantly caspase-8 a
acid (IdoUA) residues.
e integration of the 5S RNP into the ribosomal large subunit during ribosome biogenesis (PubM
it probably controls membrane reorganization/tubulation upon ATP hydrolysis. Plays a role in e
n vascular cells. Constitutively and NADPH-dependently generates reactive oxygen species (R
Mediates rapid rolling of leukocytes over vascular surfaces through high affinity divalent cation-c
hosphate-dependent GTPase-activating protein activity for ARF1 (ADP ribosylation factor 1) ar

ostsynaptic density (PSD) of excitatory synapses that interconnects receptors of the postsynap
nucleoside 5'-monophosphates deoxyadenosine monophosphate (dAMP), deoxycytidine mono
cytoskeleton through its interactions with actin capping protein (CP). May function to target CK
1294, PubMed:19549071). Recognizes and binds 2 copies of the core DNA sequence motif 5'-
) network formation, shaping and remodeling. May enhance the cell surface expression of odor
hydroxy-fatty acids (FAHFAs), but not other major classes of lipids (PubMed:27018888). Show
n channel with an outward rectification. Seems to be regulated, at least in part, by IGF-I, PDGF
vesicle trafficking, both during interphase and at the end of mitosis. Required for efficient prog
nbrane traffic at the level of the Golgi complex. May function in retrograde transport in neuronal
drogenases/reductases (SDR) family, which has over 46,000 members. Members in this family
fatty acids to their active form acyl-CoAs for both synthesis of cellular lipids, and degradation vi
-protein coupled receptor provide evidence for a possible interaction between retinoid and G-pr
inked oligosaccharide biosynthesis, which catalyzes the transfer of an N-acetyl-D-galactosamir
and CSF2/GM-CSF from endothelial cells.
plets (LDs) which are storage organelles at the center of lipid and energy homeostasis (PubMed
sphate antiporter. May transport cytoplasmic glucose-6-phosphate into the lumen of the endop
which specifically activates the production of amyloid-beta protein (amyloid-beta protein 40 and
groups from position 9 of the parent sialic acid, N-acetylneuraminic acid.
upstream activator and/or downstream effector for RAB5B in endocytic pathway. May function
Pase which cycles between an active GTP-bound and an inactive GDP-bound state. Causes t
survival via inhibition of the activity of the mammalian target of rapamycin complex 1 (mTORC1
nder normoxic conditions, the post-translational formation of 4-hydroxyproline in hypoxia-induc
atal development of the brain. Increased resistance to cell death induced by serum starvation ir
ation during the post-meiotic stages of spermatogenesis.
lasma electron transport from cytosolic NAD(P)H via hydroquinones to acceptors at the cell su
the small GTPase RALA. May be involved in cytoskeletal organization. May also be involved ir
d gene expression, regulates repressive epigenetic modifications associated with SETDB1. Re

ɔohydrates such as ribulose, ribitol, and L-arabinitol. Genome-wide association studies in some
type I collagen alpha-1 and alpha-2 mRNAs, CO1A1 and CO1A2. Stabilizes mRNAs through I
ɜable cation channel (By similarity). Mechanosensitive ion channel that converts mechanical st
ɜnt RNA helicase in the DEAD-box family and displays tumor-specific expression. [provided by
the splicing of the pre-mRNAs of human parvovirus B19 (B19V) and for the expression of B19V
EF) that activates CDC42 and RAC1 by exchanging bound GDP for free GTP. Essential for der
for Rab family protein(s).

ıge factor (GEF).

not unsaturated C16 or C18 fatty acyl-CoA to fatty alcohols. A lower activity can be observed
on protein. May be involved in the establishment and maintenance of specific neuronal connec
on protein. May be involved in the establishment and maintenance of specific neuronal connec
ɔsis. May be acting by homooligomerizing within the membrane and forming pores (PubMed:27
ɛrvertebral disks.
NF-kappa-B signaling at the basal level by modulating transcriptional activity of NF-kappa-B o
potent inhibitor of CPA1, CPA2 and CPA4. May play a role in inflammation.

th regulatory factor with a role as a negative regulator of axon-axon adhesion and growth, and
plasmic reticulum and Golgi.

ɔn-vesicular transport of cholesterol from the plasma membrane (PM) to the endoplasmic reticu

.
liate ubiquitination of MHC-I and CD4, and promote their subsequent endocytosis and sorting t
. Acts as a strong activator of USP1 and USP46 (PubMed:18082604, PubMed:19075014, Pub
protein. Naturally occurring mutations in this gene are associated with coronary artery disease

ı.
t, important for striatal morphology and motor regulation.
ıtion.
lved in the docking of exocytic vesicles with fusion sites on the plasma membrane.

junctions. Plays a role in stratified epithelial integrity and cell-cell adhesion by promoting desm
ɛthylcrotonyl-CoA carboxylase, an enzyme that catalyzes the conversion of 3-methylcrotonyl-Co
in deglycation by mediating phosphorylation of fructoselysine residues on glycated proteins, to
in peptide trimming, a step required for the generation of most HLA class I-binding peptides. F
s ubiquitin from an E2 ubiquitin-conjugating enzyme in the form of a thioester and then directly

ates mono-ADP-ribosylation of target proteins.
SCF(FBXL17) E3 ubiquitin ligase complex, a key component of a quality control pathway requi
ı. May promote melanoma cell invasion in vitro.
on during cellular stress by phosphorylating mitochondrial proteins. Involved in the clearance o
tin cytoskeleton homeostasis and filopodium and lamellipodium formation (PubMed:22751924

important role in the regulation of electrolyte homeostasis, cell signaling, survival and proliferati
ıtation with the gene for the mitochondrial ribosomal protein L34. The predicted protein contain

RhoA and Cdc42 by converting them to an inactive GDP-bound state. Essential for PTKB2 re
phosphatase PP1. Facilitates interaction of the PP1 with enzymes of the glycogen metabolism
epressor, probably via its interaction with histone deacetylase complexes (PubMed:16820529, I
regulator of vesicular trafficking process. Required for the sorting of endocytic ubiquitinated car

y play a role in cell viability.
guanine nucleotide exchange (GEF) for RAB5B and RAB31, by exchanging bound GDP for fre
hat regulates cellular processes including survival and organization of the cytoskeleton (PubMe
or adeno-associated virus and is involved in adeno-associated virus infection through endocyto
zation of somatic cells and oocytes into discrete follicular structures.
rectal carcinomas with high microsatellite instability (MSI-H).
ular senescence through a positive feedback loop with TP53. Is a TP53 downstream target ger
SCF (SKP1-CUL1-F-box protein)-type E3 ubiquitin ligase complex.
not bind directly to DNA and may regulate transcription through recruitment of histone deacetyl
(Hh)signaling (By similarity). Involved in ciliogenesis (By similarity). May participate in the traffi
eaction in fatty acid metabolism, by forming a thioester with CoA (PubMed:17762044). Has sor
ll-mediated immune response. May play a protective role in tumor cells by inhibiting natural-kille
e in cell delamination by regulating microtubule organization (By similarity). Required for the de
phate transporter activity.||Choline transporter that plays a role in the choline-acetylcholine syst
and RAC1-driven cytoskeletal remodeling (Probable). May regulate chemotaxis, cell migration

ifferent roles in immune pathways. Participates in the activation of interferon-gamma signaling
and recruit them to vesicular organelles.
ion of autophagosomal vacuoles (autophagosomes). Plays a role in mitophagy which contribut
EF) which specifically activates small GTPase CDC42 by exchanging bound GDP for free GTP
vith RAB6A, to regulate intra-Golgi retrograde trafficking. It is involved in autophagy, acting as a
ter, hence plays a role in the transcriptional regulation of genes expressed in the hemogenic er
myeloid precursor cells.
death, mediating both autophagy and apoptosis.

nd type I interferon signaling pathways. May also regulate the type II interferon signaling pathw
ation. Interacts with extracellular matrix proteins and with the actin cytoskeleton. Mediates adhe
post-transcriptional regulatory mechanisms.
ytosis. Has both a membrane binding/tubulating activity and the ability to recruit proteins essen
tons for Na(+) and K(+) across endomembranes. May contribute to the regulation of Golgi app

hydroxy-fatty acids (FAHFAs), but not other major classes of lipids (PubMed:27018888). Show
ono-ADP-ribosylation of glutamate and aspartate residues on target proteins (PubMed:188518
ration (PubMed:22846708, PubMed:25448675). May regulate cell-matrix interactions via its int
contraction and cardiac function. Phosphorylates a specific serine in the N-terminus of a myosi
r affinity for phosphatidic acid, a lipid enriched in recycling endosome membranes. On endoso

or signaling and germinal center formation.
f the postsynaptic apparatus. May play a role in acetyl-choline receptor (AChR) aggregation in

seems to be the main initiator.
d perhaps for Cdc42. May be involved in regulation of cytoskeletal reorganization, cell proliferat
family of transcription factors. Alternate splicing results in multiple transcript variants. [provide

the ubiquitination and proteasomal degradation of CYLD.
lyglutamylase complex. Required for the development of the spermatid flagellum (By similarity)
post-transcriptional regulatory mechanisms.
f the ADAMTS (a disintegrin and metalloproteinase with thrombospondin motif) family. This pro
ngation on the peroxisomal surface.
n/CSN2 as well as several extracellular matrix (ECM) proteoglycans such as decorin/DCN, bigl
a role in vesicle trafficking. Binds phospholipids in the presence of calcium ions (By similarity)
superfamily, a novel family that is similar to the chemokine and transmembrane 4 superfamilies
ed protein has a tripartite domain structure with a relatively short N-terminus and a long C-termi
pression of this gene is induced by glucocorticoids and may be an early marker for glucocortico
dylinositol 3-kinase (PI3K) activity.
gulates cell division and neuronal migration during cortical development. Necessary for mitotic
phology and cytoskeletal organization. Required in the cortical actin filament dynamics.

which may play an important role in the neural crest stem cell migration and differentiation du

tion.

ic acetylcholine receptors (nAChRs) activity. In vitro increases receptor desensitization and de
5'-CANNTG-3' as a heterodimer and inhibits transcriptional activation by MYOD1, MYOG, ME
nmetrical cell division and cell polarization processes. May play a role in the formation of epithe
aminin and the extracellular matrix.
neddylation by sequestering CUL1 at the cell membrane (PubMed:25349211). When overexp
proteins. The IQ motif serves as a binding site for different EF-hand proteins such as calmodu

xpression of target mRNAs at the translation level. May play a role in the proliferation and mai

m endoplasmic reticulum to Golgi.

extracellular matrix molecules in developing teeth and may play important roles in differentiati

n keratinocytes.
ndoplasmic reticulum biogenesis (PubMed:17141156). Involved in Golgi and endoplasmic retic
notes synapse differentiation (PubMed:27812321). Suppresses neurite outgrowth (By similarity
CF-like ECS (Elongin-Cullin-SOCS-box protein) E3 ubiquitin-protein ligase complex which med
tion.
r for CD47. Acts as docking protein and induces translocation of PTPN6, PTPN11 and other b
ilia and flagella.
. May regulate cell architecture and adhesion.
nd angiogenic sprouting from venous endothelium during embryogenesis.

V irradiation. Mediates G2/M cell cycle arrest, MEK autoactivation and ERK1/2-signaling pathw
n and metalloproteases (ADAMs) in the signaling of EGFR-ligand shedding. May be involved i

PubMed:24130170). Acts as an anti-apoptotic regulator of the JNK pathway by ubiquitinating a
ferentiation.
AP2/AP-2. Enhances estrogen-dependent transactivation mediated by estrogen receptors. Ma

actin polymerization through this process, during the late stages of embryogenesis. Involved in
ediates down-regulation of growth factor signaling via internalization of growth factor receptors.
hydrolyzes 'Lys-63'-linked polyubiquitin to monoubiquitin.

surfactant homeostasis. May play a role in controlling adipocyte function.

naling pathway and may be involved in epidermal differentiation.
nvolved in the beneficial effects of muscular exercise, nor in the induction of browning of humai
ubstrate specificity (PubMed:12027805, PubMed:12223481, PubMed:28882892, PubMed:304

eleton formation by regulating actin polymerization.
activator. Promotes beta-catenin transcriptional activity. Plays a role in tumorigenesis. Enhanc
on of 4-hydroxyproline in -Xaa-Pro-Gly- sequences in collagens and other proteins.
tor cysteine-rich (SRCR) superfamily. Members of this family are secreted or membrane-ancho
MEK to RAF. Has very low protein kinase activity and can phosphorylate MAP2K1 at several S
ansmembrane protein family that transports small molecules across membranes. The encoded
and podosome formation and extracellular matrix degradation. Binds matrix metalloproteinase
cule (RGM) family that contributes to the patterning of the developing nervous system (By simil
ramblase activity; scrambles phosphatidylserine, phosphatidylcholine and galactosylceramide
rolysis of ceramides into sphingoid bases like sphingosine and free fatty acids at alkaline pH (
oligosaccharide biosynthesis, the transfer of an N-acetyl-D-galactosamine residue to a serine

ociated serine/threonine protein kinases. The proteins in this family contain a domain that gives
released fragment is important for agrin signaling and to exert a maximal dendritic filopodia-in
ting aspects of synaptic vesicle membrane flow within the nerve terminal.

post-transcriptional regulatory mechanisms.
gulation through the control of Rac-GTP levels.
r signaling of cortical neurons during forebrain development.

EF) for RAB14. Also has some, lesser GEF activity towards RAB35.

t plays a role in many key processes linked to cell growth and survival such as cytoskeleton re
ught to anchor actin to a variety of intracellular structures. This is a bundling protein (Probable)
erminal phosphate group between ATP and AMP. Also displays broad nucleoside diphosphate
of cellular nucleotides by catalyzing the interconversion of nucleoside phosphates (PubMed:1
family of proteins. Aldehyde dehydrogenase is the second enzyme of the major oxidative pathv

nembrane curvature, membrane shaping and membrane remodeling. Required in muscle cells
sport. Catalyzes the exchange of cytoplasmic ADP with mitochondrial ATP across the mitochor
rotein whose affinity for calcium is greatly enhanced by anionic phospholipids. It binds two calc
rotein whose affinity for calcium is greatly enhanced by anionic phospholipids. It binds two calc

the release of Ca(2+) from intracellular stores.
ome c-dependent autocatalytic activation of pro-caspase-9 (Apaf-3), leading to the activation of
cytosis by binding to STXBP1, an essential component of the synaptic vesicle exocytotic mach
both coactivator and corepressor functions. Adapter protein that forms a transcriptionally active
ostasis. The soluble form may have inhibitory properties towards coagulation factors. May inter
al chelators with metal-reducing activity. Bind transient metals such as copper, zinc and iron. Ir
/ linking plasma membrane receptors to the assembly of focal adhesions and actin stress fibers
RhoB. Interacts specifically with the GDP- and GTP-bound forms of post-translationally proces
rting vacuolar (V)-ATPase protein pump, which is required for luminal acidification of secretory
l chromatin remodeling. Facilitates DNA replication in multiple cellular environments and is requ
BARD1 heterodimer specifically mediates the formation of 'Lys-6'-linked polyubiquitin chains ar
genase complex catalyzes the overall conversion of alpha-keto acids to acyl-CoA and CO(2). I
hree-way gene translocation in a Burkitt lymphoma cell line. As a result of the gene translocati
of mitochondrial respiratory chain complex III. Plays an important role in the maintenance of m
ing molecule that activates signaling cascades downstream of NTRK2. Activates signaling cas
s intermediate filaments as part of a complex composed of BFSP1, BFSP2 and CRYAA (PubM
r for P.falciparum RH5 which is essential for erythrocyte invasion by the merozoite stage of P.fa
tor which efficiently blocks the release of diverse mammalian enveloped viruses by directly teth
the product of biotin-dependent carboxylases degradation.
ol (DAG) to 2-arachidonoyl-glycerol (2-AG), the most abundant endocannabinoid in tissues. Re
rotease which catalyzes limited proteolysis of substrates involved in cytoskeletal remodeling ar
pendent cysteine protease). Plays a key role in postmortem tenderization of meat and have be
spases responsible for apoptosis execution. Cleaves poly(ADP-ribose) polymerase in vitro, as v
j organisms and serves to protect cells from the toxic effects of hydrogen peroxide. Promotes g
binding factor (CBF) with CBFB. RUNX members modulate the transcription of their target gene
g as an adhesion receptor for THBS1 on platelets, and in the modulation of integrins. Plays an
dhesion proteins. They preferentially interact with themselves in a homophilic manner in conne
l in the control of the cell cycle and differentiation; promotes G1/S transition. Phosphorylates pF
eration of normal cells by interacting strongly with CDK4 and CDK6. This inhibits their ability to
h CDK4. Inhibits cell growth and proliferation with a correlated dependence on endogenous ret
and inhibits them.
nd a phorbol ester receptor. Involved in the assembly of neuronal locomotor circuits as a direct
nctions as antiport system and exchanges chloride ions against protons.

ıctions as antiport system and exchanges chloride ions against protons. Important for normal a
ıaperone that prevents aggregation of non native proteins (PubMed:11123922, PubMed:19535
ndothelium. Major component of the Descemet's membrane (basement membrane) of corneal
/ided into 2 subfamilies based on sequence similarities. The pancreatic carboxypeptidase-like a
and may function as an intracellular zinc transport protein.
ith host defense: it promotes agglutination, bacterial capsular swelling, phagocytosis and comp
n monomers with hyaluronic acid in the extracellular cartilage matrix.
refractive index of the lens. Has chaperone-like activity, preventing aggregation of various prote
his protein is thought to serve an important physiological role as a local regulator of this enzym
ecreted by vascular endothelial cells. Promotes proliferation and differentiation of chondrocytes
of a variety of cadherins. The association of catenins to cadherins produces a complex which i
ein breakdown. Plays a role in APP processing following cleavage and activation by ADAM30 w
as carboxy-dipeptidase activity (PubMed:10504234). Capable of producing kinin potentiating p
ed in mediating interferon-gamma-induced cell death.
at plays a role in the formation of cell projections (PubMed:20215400). Required for actin polym
ytoskeleton via F-actin. Ligand for dystroglycan. Component of the dystrophin-associated glyco
and DNA repair in nucleus and mitochondrion. Involved in Okazaki fragments processing by cl
required for phagocytosis of apoptotic cells and cell motility. Along with DOCK1, mediates CRK
ed in the costimulatory signal essential for T-cell receptor (TCR)-mediated T-cell activation. Ac
ability of synapses as well as being involved in the clustering of nicotinic acetylcholine receptors
ı as negative regulator of bone mineralization. Stimulates the proliferation of endothelial cells a
osomes. Functions in close association with EIF4-F and EIF4-A. Binds near the 5'-terminal cap
ticity to neuronal membrane via multiple interactions, including the spectrin-actin-based cytoske
gative receptor of EPOR-mediated signaling.||Receptor for erythropoietin. Mediates erythropoie
/ed in transcriptional repression. Binds to promoter/enhancer response elements that contain th
e enhancer of the adenovirus E1A gene; the core-binding sequence is 5'[AC]GGA[AT]GT-3'.
plex functions in DNA cross-links repair. Required for the nuclear accumulation of FANCC and
fatty acids to their active form acyl-CoAs for both synthesis of cellular lipids, and degradation vi
long-chain fatty acids for both synthesis of cellular lipids, and degradation via beta-oxidation (F
fatty acids to their active form acyl-CoA for both synthesis of cellular lipids, and degradation via
onents of 10-12 nm extracellular calcium-binding microfibrils, which occur either in association
endonuclease and 5'-3' exonuclease activities involved in DNA replication and repair. During DN
-surface receptor for fibroblast growth factors and plays an essential role in the regulation of en
elopment or hypertrophy.
rs where it acts as a transcriptional coactivator of FOXK1.
aveolar membranes, functionally participating in formation of caveolae or caveolae-like vesicles
ell-surface receptor for VEGFA, VEGFB and PGF, and plays an essential role in the developme
drolyzing the alpha-1,6-linked fucose joined to the reducing-end N-acetylglucosamine of the ca

ls chemokine levels and localization via high-affinity chemokine binding that is uncoupled from
n in lysosomes (PubMed:1856189, PubMed:7717400, PubMed:14695532, PubMed:18429042
e which is a lysosomal exohydrolase required for the degradation of the glycosaminoglycans, k
the enhancer of the T-cell receptor alpha and delta genes. Binds to the consensus sequence 5
ansfer of an N-acetylglucosamine moiety onto mucin-type core 1 O-glycan to form the branche
l galactosyl residues from gangliosides, glycoproteins, and glycosaminoglycans.||[Isoform 2]: H
the degradation of glycine. The P protein (GLDC) binds the alpha-amino group of glycine throu

alyzes the first reaction in the primary pathway for the renal catabolism of glutamine. Plays a ro
d in all cells. It is involved in the catabolism of heparin, heparan sulphate, and keratan sulphate
eration and induces epithelial cells to secrete IL-8.||[Granulin-4]: Promotes proliferation of the e

acts as a negative regulator in the hormonal control of glucose homeostasis, Wnt signaling and
wide number of exogenous and endogenous hydrophobic electrophiles. Participates in the forr
chinery by coordinating the formation of a multiprotein complex at the C-FOS promoter, and lin
yzes the last three of the four reactions of the mitochondrial beta-oxidation pathway (PubMed:8
yzes the last three of the four reactions of the mitochondrial beta-oxidation pathway (PubMed:8
ne residues on the N-terminal part of the core histones (H2A, H2B, H3 and H4). Histone deace
pment and survival. Involved in regulation of the expansion or survival of lymphoid cells. Requi
gulator of the adaptive response to hypoxia. Under hypoxic conditions, activates the transcriptio
ptide repertoire including viral epitopes derived from IAV NP/nucleoprotein (CTELKLSDY), IAV
g a common signature motif, namely a Pro residue at position 2 and mainly a Leu anchor resid
ntigens to the immune system.
s a transcribed pseudogene, possibly derived from HLA-A. [provided by RefSeq, May 2010]
g at the alpha methene bridge to form biliverdin. Biliverdin is subsequently converted to bilirubin
embly of 40S hnRNP particles (PubMed:8264621). Interacts with poly-U tracts in the 3'-UTR or
hich is part of a developmental regulatory system that provides cells with specific positional ide
n of testosterone and androstenedione, as well as estradiol and estrone. Also has 20-alpha-HS
ytryptamine (serotonin). Also functions as a receptor for ergot alkaloid derivatives, various anxi
c DNA binding domain) which negatively regulates the basic helix-loop-helix (bHLH) transcriptio
adation pathway of dermatan sulfate and heparan sulfate.
erferons, including interferons alpha, IFNB1 and IFNW1 (PubMed:2153461, PubMed:7665574,
ptor for the cytokine interferon gamma (IFNG) (PubMed:8124716, PubMed:7673114,PubMed:7
enzymes from the Golgi complex and the cell surface to lysosomes. Lysosomal enzymes beari
e of the IGFs and have been shown to either inhibit or stimulate the growth promoting effects of
e of the IGFs and have been shown to either inhibit or stimulate the growth promoting effects of
e of the IGFs and have been shown to either inhibit or stimulate the growth promoting effects of
ow affinity. Stimulates prostacyclin (PGI2) production. Stimulates cell adhesion.

leukin 13. Couples to the JAK1/2/3-STAT6 pathway. The IL4 response is involved in promoting
olved in polarized T-helper 1 (Th1) cell and natural killer (NK) cell immune responses (Probable
35 acts as a receptor for adenovirus type C.||Integrin alpha-V/beta-5 (ITGAV:ITGB5) is a recep
, a second messenger that mediates the release of intracellular calcium.
complexes that plays an essential role in embryonic development, including heart and liver dev
ransmembrane potassium transport and to the regulation of the resting membrane potential in
spindle formation and the movements of chromosomes during mitosis and meiosis. Binds to
keratin gene family. The keratins are intermediate filament proteins responsible for the structu
. Together with KRT8, helps to link the contractile apparatus to dystrophin at the costameres c
n.||Presents carbohydrate ligands to selectins. Also implicated in tumor cell metastasis.
of dynamic actin-based, cytoskeletal activities. Agonist-dependent changes in LASP1 phospho
ect on actin cytoskeletal changes. Required for atypical chemokine receptor ACKR2-induced p
astic-fiber architectural organization and/or assembly.
intracellular signaling.
oligosaccharides. Progressively trim alpha-1,2-linked mannose residues from Man(9)GlcNAc(2
carbohydrates released during glycoprotein turnover. Cleaves all known types of alpha-mann
eta-linked mannose residue from the non-reducing end of all N-linked glycoprotein oligosacch
iogenic and xenobiotic amines and has important functions in the metabolism of neuroactive a
tous cross-bridging between microtubules and other skeletal elements.
but MAPs may stabilize the microtubules against depolymerization. They also seem to have a
otein. Promotes microtubule assembly.
edial amyloid.||Plays an important role in the maintenance of intestinal epithelial homeostasis
in 6-beta-N-acetylglucosaminyltransferase A]: Promotes proliferation of umbilical vein endothe
ne and cartilage. Thought to act as an inhibitor of bone formation.
t confined on acting on polypeptides of up to 30 amino acids (PubMed:15283675, PubMed:81
CHUK/IKKA and initiates a primary innate immune response by inducing mitochondrial-nuclear
IV, and V, and fibronectin. Activates procollagenase.
other thiol compounds. Also has weak rhodanese activity. Detoxifies cyanide and is required fo
le in cytokinesis, cell shape, and specialized functions such as secretion and capping. During
le in cytokinesis, cell shape, and specialized functions such as secretion and capping. Involve
ot bind calcium.
actin-based motor protein with ATPase activity (By similarity). Plays a role in endosomal prote
s with ATPase activity (By similarity). Unconventional myosins serve in intracellular movement
equence 5'-TTGGCNNNNNGCCAA-3' present in viral and cellular promoters and in the origin
factor present in almost all cell types and is the endpoint of a series of signal transduction eve
ound ligands Jagged-1 (JAG1), Jagged-2 (JAG2) and Delta-1 (DLL1) to regulate cell-fate dete
NPPA/ANP and the brain natriuretic peptide NPPB/BNP which are potent vasoactive hormon
n of tissues remodeling (PubMed:30449416). It functions as cell surface receptor for fibrillar co

gi which may have a role in calcium homeostasis (By similarity). Acts as a non-receptor guanin
fic obliteration of the intercellular space, through calcium-independent cell-adhesion activity.
anolamine. Has lower affinity for phosphatidylinositol and phosphatidylcholine. Serine protease
e for CCT in the assembly of heterotrimeric G protein complexes, facilitates the assembly of bo
esis. Seems to both prevent the formation of 7-pterins and accelerate the formation of quinonoi
on protein. May be involved in the establishment and maintenance of specific neuronal connec
rious proproteins by cleavage at paired basic amino acids, recognizing the RXXX[KR]R consens
tose 6-phosphate to fructose 1,6-bisphosphate by ATP, the first committing step of glycolysis.
of 6-phosphogluconate to ribulose 5-phosphate and CO(2), with concomitant reduction of NAD
osphatidylcholine between membranes.
nogen activator. Plays a role in localizing and promoting plasmin formation. Mediates the protec
P3H3 and P3H4 that catalyzes hydroxylation of lysine residues in collagen alpha chains and is
NA and RNA viruses. The antiviral activity can involve one or several isoform(s) and can be enh
scle creatine kinase (MCK) and so has a role in the establishment of diverse mesodermal mus
y regulator of lipid metabolism. Activated by the endogenous ligand 1-palmitoyl-2-oleoyl-sn-glyc
ted protein phosphatase which plays an essential role in the transduction of intracellular Ca(2+)
mplex and component of the DNA polymerase alpha complex (also known as the alpha DNA p
protein kinase (AMPK), an energy sensor protein kinase that plays a key role in regulating cellu
cAMP-dependent protein kinase activity, this protein interacts with the catalytic subunit of the
acylglycerol (DAG)-dependent serine/threonine-protein kinase that is involved in positive and ne
d by and mediating cAMP signaling in cells. Acts through phosphorylation of downstream targe
ear. May play a role in neuronal development and synaptic plasticity. May be required for neuro
actor to activated protein C in the degradation of coagulation factors Va and VIIIa. It helps to pr
arboxyglutamic acid (Gla)-containing, single-pass transmembrane protein. This protein contains
, including extracellular matrix proteins such as fibronectin. HTRA1-generated fibronectin fragm
hic and neurotrophic factor, these effects are mediated by its G-protein-coupled receptors, GPI
-secretase complex, an endoprotease complex that catalyzes the intramembrane cleavage of i
inase complex which is characterized by its ability to cleave peptides with Arg, Phe, Tyr, Leu, a
nbly and required for efficient antigen processing. The PA28 activator complex enhances the ge
soform 1 it antagonizes the PI3K-AKT/PKB signaling pathway. Plays a role in mitochondrial ene
The activity of this receptor is mediated by G(q) proteins which activate a phosphatidylinositol-c
alpha (PGF2-alpha) to its specific FP receptor, by decreasing the receptor number rather than t
contain a kinase domain and inhibits PTK2/FAK1 phosphorylation and signaling. Its enhanced
play a role in the regulation of lymphangiogenesis, cell-cell adhesion, cell-matrix adhesion, ce
ontact and adhesion such as growth control, tumor invasion, and metastasis. Negative regulato
obiopterin, an essential cofactor of aromatic amino acid hydroxylases. Catalyzes the transforma
esicular traffic (By similarity).
the Ras-related RalA and RalB GTPases which allows GTP binding and activation of the GTP

cyltransferase activities (PubMed:19615464, PubMed:22605381, PubMed:22825852, PubMed
MRE11-RAD50-NBN (MRN) complex in DNA-end resection, the first step of double-strand brea
es in the endoplasmic reticulum lumen or post-ER compartment.
esis and islet cell differentiation during endocrine pancreas development. Essential for the diffe
neric G-proteins by increasing the GTPase activity of the alpha subunits, thereby driving them
arginine and/or lysine residues from the N-terminus of several peptide substrates including Ar
factor. Plays a role in ciliogenesis. Probably regulates cilia formation by regulating actin stress
downstream of ERK (MAPK1/ERK2 and MAPK3/ERK1) signaling and mediates mitogenic an
es interaction between the ribosome and the endoplasmic reticulum membrane.
paran sulfate. Regulates exosome biogenesis in concert with SDCBP and PDCD6IP (PubMed:
, a subcomplex of the dystrophin-glycoprotein complex which forms a link between the F-actin

ino acid transporter complexes (PubMed:11557028, PubMed:9829974, PubMed:9751058, Pul
n against three to four Na(+) ions across the cell membrane, and thereby contributes to the reg
diates sodium and chloride reabsorption. Plays a vital role in the regulation of ionic balance an
repression of select genes by chromatin remodeling (alteration of DNA-nucleosome topology).
in tetra-hydrobiopterin biosynthesis to form 5,6,7,8-tetrahydrobiopterin.
mains of at least two HSC70 molecules dependent on activation of the HSC70 ATPase by HSF
ator that mediates cellular responses to interferons (IFNs), cytokine KITLG/SCF and other cyto
-5'-adenylyl sulfate (PAPS) as sulfonate donor to catalyze the sulfate conjugation of estradiol a
in regulation of lymphocyte migration. Phosphorylates MSN, and possibly PLK1. Involved in re
r of TNF-alpha to its mature soluble form (PubMed:9034191). Responsible for the proteolytical
nent of the ubiquitin/19S proteasome complex to nuclear receptor-regulated transcription units
ld play an important role in the transcription of genes required for the later stages of cell cycle p
ort protein. Delivers cobalamin to cells.
the reduction of hydrogen peroxide and organic hydroperoxides to water and alcohols, respecti
ion. Binds DNA cooperatively with E2F family members through the E2 recognition site, 5'-TTT
forming with the TGF-beta type I serine/threonine kinase receptor, TGFBR1, the non-promisc
ed scaffolding proteins that link tight junction (TJ) transmembrane proteins such as claudins, jun
transmembrane 4 superfamily, also known as the tetraspanin family. Most of these members a
NF-alpha and approximately 5-fold lower affinity for homotrimeric TNFSF1/lymphotoxin-alpha.
May be involved in cell migration, cartilage development and in linking signal transduction pathw
ransient breakage and subsequent rejoining of DNA strands. Topoisomerase II makes double-
nor types; induces growth arrest or apoptosis depending on the physiological circumstances an
eolus and is similar to that of a family of genes on the Y-chromosome. This gene is intronless.
h. May act via its PH domain that competes with other PH domain-containing proteins, thereby
nide detoxification or modification of sulfur-containing enzymes. Other thiol compounds, beside
nt conversion of GDP-4-dehydro-6-deoxy-D-mannose to GDP-fucose, involving an epimerase a

ridine monophosphate and cytidine monophosphate. Does not phosphorylate deoxyribonucleo
=-B167 binds heparin and neuropilin-1 whereas the binding to neuropilin-1 of VEGF-B186 is re
actin cytoskeleton. Contributes with NCK1 and GRB2 in the recruitment and activation of WAS
xhibits positive and negative control on a large number of cellular and viral genes by binding to
ıtion.
s an ISG15 E3 ligase (PubMed:16352599). Involved in innate immune defense against viruses
ınd functions as repressor. Promotes cell proliferation and antagonizes cell death. Promotes ph
and apolipoprotein E (apoE)-containing ligands. LRP8 participates in transmitting the extracellu
tubules. It binds two moles of GTP, one at an exchangeable site on the beta chain and one at
nin-mediated matrix remodeling. Inhibits trypsin, plasmin, factor VIIa/tissue factor and weakly fa
ɔnic and postnatal development of muscle tissue. May regulate intracellular transport and local
-pass membrane protein. An alternatively spliced transcript variant encoding the same protein h
ɔtins (PubMed:15269288). Plays a role in endosomal maturation (PubMed:27015288).
ɛes that regulate the G1/S phase transition and/or DNA replication. Can phosphorylate MCM2 a
NA replication.
ver of cumulus cells surrounding the egg by digesting hyaluronic acid. Involved in induction of tl
rosine-phosphorylated growth factor receptors or their cellular substrates. Maintains low levels
idues within acidic motifs of polypeptides, using 3'-phosphoadenylyl sulfate (PAPS) as cosubst
dhesions. Did not bind receptor-like tyrosine phosphatases type 2A.
that modulates the characteristics of the channel-forming alpha-subunits (PubMed:7649300, F
its the entry of viruses to the host cell cytoplasm, permitting endocytosis, but preventing subse
-B which facilitates binding of NF-kappa-B proteins to target kappa-B genes in a redox-state-de
ȷ the GTPase activity of G protein alpha subunits thereby driving them into their inactive GDP-b
hosphatase that catalyzes the dephosphorylation of a variety of glycerolipid and sphingolipid p
e in innate immune response by recognizing and degrading RNAs from microbial pathogens th
on protein. May be involved in the establishment and maintenance of specific neuronal connec
ınd ketones to alcohols. Catalyzes the reduction of prostaglandin (PG) D2, PGH2 and phenant
ɛsis. Required throughout embryonic neurogenesis to maintain neural progenitor cells, also call
various processes such as Wnt signaling pathway, telomere length and vesicle trafficking (Pub
lar processes by insulin.
cipate in intracellular degradation and turnover of proteins. Has also been implicated in tumor ir
ic and collagenolytic activity. Plays a role in the wound healing process. Mediates both heteroty
trol of cell growth and differentiation. Antagonizes transcriptional activation and cellular transfo
ɔr human cytomegalovirus pentamer-dependent entry in epithelial and endothelial cells.||High a
ssical negative feedback system that regulates cytokine signal transduction. SOCS2 appears tc
PubMed:29240402). Recognizes as substrates free retinal and cellular retinol-binding protein-b
hway by inhibiting the dephosphorylation of ERK by phosphatase PP2A-PPP2R5C holoenzym
hange factor (GEF) and can induce membrane ruffling. Functions in cell migration, attachment

ge adhesion processes. In B-cells, may act by coupling the B-cell receptor (BCR) to integrin ac
loride ion channels. May participate in cellular growth control.
ırylase and APS kinase activity, which mediates two steps in the sulfate activation pathway. Th
:y of LIM domain-containing transcription factors.
› mitosis (G2 to M transition) by phosphorylation of the CDK1 kinase specifically when CDK1 is
:eptor for interleukin-33 (IL-33); signaling requires association of the coreceptor IL1RAP. Its sti
ı elements found in the promoter regions of a number of genes related to cell proliferation. Binc
orter. Catalyzes the rapid transport across the plasma membrane of many monocarboxylates s
on ARF1 and ARF6. Promotes the activation of ARF factors through replacement of GDP with
critical role in cancer progression by activating VEGFA production and angiogenesis through a
ıide (LacCer) via the transfer of galactose from UDP-galactose to glucosylceramide (GlcCer) (F
o recruit SMAD2/SMAD3 to intracellular membranes and to the TGF-beta receptor. Plays a sig

:oactivator involved in the regulated transcription of nearly all RNA polymerase II-dependent ge
PP amyloid-beta protein 42 into toxic oligomers.||Mature BRI2 (mBRI2) functions as a modulat
P pathway.
0 to mediate signal transduction in response to IL27. This signaling system acts through STAT
rbonate-dependent chloride-HCO3(-) exchange with a Na(+):HCO3(-) stoichiometry of 2:1. Play
ecificity for several peptides. Involved in proteolytic events essential for cell growth and viability
multivesicular bodies (MVB) pathway. Recognizes membrane-associated ESCRT-III assemblie
ertain nuclear receptors (PubMed:20812024). Part of a complex which promotes histone deace
depolymerization of F-actin by mediating oxidation of specific methionine residues on actin to f
activates CaM-kinase II activated upon autophosphorylation, and CaM-kinases IV and I activat
heparan sulfate (By similarity). May have a role in the organization of cell shape by affecting th
ange factor (GEF), which activates Rap and Ras family of small GTPases by exchanging boun
May couple the activity of the ER Ca(2+) pump SERCA2B with the activity of the translocon. T
le in neural stem cell commitment and corticogenesis as well as in lymphoid cell development a
ctivity and cell death (PubMed:24652652). May be involved in the production of chemokine mo
23). Regulates early events but also late events of autophagosome formation through direct int
INS complex, a complex that plays an essential role in the initiation of DNA replication, and pro
(PubMed:15616186). Required for centriole subdistal appendage assembly (PubMed:2842209
B-CUL3-RBX1) E3 ubiquitin-protein ligase complex required for efficient chromosome alignme
ulation of MAG (Myelin-associated glycoprotein) expression. Acts as a regulator of Schwann c
lts are multi-subunit structures that may act as scaffolds for proteins involved in signal transduc
ne residues on the N-terminal part of the core histones (H2A, H2B, H3 and H4). Histone deace
ization of KRT8/KRT18 filaments. Acts as an endogenous molecular chaperone for neuronal p
ficking to the cell surface. Promotes ADAM10-mediated cleavage of CD44.
er that catalyzes the calcium-dependent exchange of cytoplasmic glutamate with mitochondrial

that converts 3-alpha-tetrahydroprogesterone (allopregnanolone) to dihydroxyprogesterone and

/s a role in a variety of different signaling pathways including cytoskeleton regulation, cell migra
s chromatin-associated protein. Acts as inhibitor of HMG20B. Overcomes the repressive effect
ortant role in the hydrolysis of circulating peptides. Catalyzes the hydrolysis of dipeptides with u

an activin antagonist. Specific inhibitor of the biosynthesis and secretion of pituitary follicle stir
exocytosis as a target of the diacylglycerol second messenger pathway. Is involved in neurotra
volved in the assembly of continuous elastin (ELN) polymer and promotes the interaction of mi
of CBL by linking CBL to the insulin receptor. Required for insulin-stimulated glucose transport.
its the entry of viruses to the host cell cytoplasm, permitting endocytosis, but preventing subse
translation initiation of poly(A)-containing mRNAs. Its stimulatory activity on translation is medi
age response pathway associated with the activation of homologous recombination and double
kinase belonging to a proposed calcium-triggered signaling cascade involved in a number of ce
t as a cellular target for adenovirus E3-14.7K, an inhibitor of TNF-alpha functions, thereby affe
AP) activity of regulator of G protein signaling (RGS) proteins, hence involved in the terminatio
t. Promotes the disassembly of phosphorylated vimentin intermediate filaments (IF) during mito
e functions at mucosal sites.
NPFF (F-8-F-amide) neuropeptides, also known as morphine-modulating peptides. Can also b
phase II biotransformation reactions in which lipophilic substrates are conjugated with glucuror
synthesized preproteins into mitochondria. Binds the mature portion of precursor proteins. Inte
eticulum (ER) quality control and ER-associated degradation (ERAD). May bind terminally misfo
ly conserved cyclin family, whose members are characterized by a dramatic periodicity in prote
on.
s such as UDP-N-acetylglucosamine (UDP-GlcNAc), UDP-glucose (UDP-Glc) and GDP-manno
drolysis and the folding of unfolded proteins mediated by HSPA1A/B (in vitro) (PubMed:24318
n family of serine proteases. Mouse studies found a decrease of mRNA levels of this gene afte
daptive immune response. Inhibits the release of IFNG from activated T-cells.
that controls the major intracellular degradation process by which cytoplasmic materials are pa
vated in a guanine nucleotide exchange factor (GEF)-independent manner via its intrinsic GDP
n factors on cell proliferation and differentiation. Binds heparin (By similarity).
a regulator of glycolysis in macrophages (Probable). Does not transport glucose (PubMed:3043

n to the AP-2-alpha gene. Binds to a regulatory element (A32) in the AP-2-alpha gene promote
he transfer of N-acetylglucosamine (GlcNAc) to the core mannose residues of N-linked glycans
and morphological processes. Inhibits actin polymerization, likely by sequestering G-actin. By ca
er for a novel upstream stimuli in controlling cell proliferation. Activates the MAP kinase pathway
and functions as a cargo in axonal anterograde transport. Complex formation with APBA2 and

Med:20797634). Represses cAMP-responsive element (CRE)-mediated transcriptional activatio
o the spindle pole and in chromosome movement. Binds to the tail domain of the KIF3A/KIF3B
activating protein that has specificity for Rab3 subfamily (RAB3A, RAB3B, RAB3C and RAB3D
eductase and acts on 15-oxo-PGE1, 15-oxo-PGE2 and 15-oxo-PGE2-alpha. Has no activity tov
difies alpha-tubulin (By similarity). Involved in the side-chain initiation step of the polyglutamyla
nents of the Mediator co-activator complex. The Mediator complex is a multi-protein complex re
cohesion, probably as part of a linker structure which prevents centrosome splitting (PubMed:1
complex which binds to the mRNA cap and mediates translational repression. In the CYFIP1-
e that specifically recognizes acetylated histones and promotes ATP- and ubiquitin-independe
ligase that mediates the ubiquitination of NEFL and of phosphorylated BCL2L11. Plays a neu

e NYN domain. The function of this protein has yet to be determined. Alternative splicing result
maintenance of centrosome integrity and completion of cytokinesis as part of the HAUS augm
ARF6.
nt manner. Has no direct effect on actin depolymerization. Acts as a chaperone for ALOX5 (5L
protein-coupled receptor phosphorylation in a calcium dependent manner. Directly regulates G

quired for canonical ribosome function and has extra-ribosomal functions. Component of the GA
ntiation.||Plays a role in directed cell migration by regulating the activation and subcellular locat
veen membrane proteins and the actin cytoskeleton (PubMed:10339567). In collaboration with
nt attachment of ubiquitin moieties onto substrate proteins. These substrates include FILIP1, p
signaling; in cooperation with SKI prevents the translocation of SMAD2 from the nucleus to the
opment and/or progression of some cancers.
ingle-stranded DNA (ssDNA) (PubMed:30312375). Regulates inflammatory cytokine responses
nge of anionic amino acids with high specificity for anionic form of cystine and glutamate.
domain, the small Mafs behave as transcriptional repressors when they dimerize among them
cytoplasm or allow the binding of lipids to organelles.
3 and apoptosis.
component of the SCF (SKP1-cullin-F-box protein) E3 ubiquitin-protein ligase complex, SCF(F
d for sperm flagellum motility. Produces force towards the minus ends of microtubules. Dynein
A. Promotes reorganization of the actin cytoskeleton and recruits DLG4 to F-actin. Contributes
g from apical recycling endosomes to the apical plasma membrane. Involved in insulin granule
oplasmic specialization, a type of cell junction specific to testis. Important for establishment of

s required for tissue-specific ciliogenesis and may regulate ciliary membrane composition (By s
binds histone H3 trimethylated at 'Lys-36' (H3K36me3) and recruits the PRC2 complex. Probab

ul7-RING ubiquitin-protein ligase complex, which mediates the ubiquitination and subsequent p

SCF (SKP1-CUL1-F-box protein)-type E3 ubiquitin ligase complex. Promotes the proteasome-
and meiotic cell cycle (PubMed:17485488, PubMed:17234884, PubMed:17875940, PubMed:2
nic reticulum functions in neurons.
during carcinogenesis and metanephric kidney organogenesis, as a BMP antagonist required
educe both Fe(3+) to Fe(2+) and Cu(2+) to Cu(1+). Uses NAD(+) as acceptor.
l cell activation. May act as a nuclear transcription factor that negatively regulates the expressi
crotubule-dependent transport of neuronal RNA from the cell body to the dendrite. As protein sy
gulation in embryonic stem cells (ESCs). Stimulates expression of transcription factors that are
ort of organic anions such as the thyroid hormones T3 (triiodo-L-thyronine), T4 (thyroxine) and
iation and adipogenesis.
t (IFT) complex B: together with IFT74, forms a tubulin-binding module that specifically mediate
tion of the actin filaments at the pointed end. The Tmod/TM complex contributes to the formati
LK.
rin signaling via ILK and in activation of the GTPases CDC42 and RAC1 by guanine exchange
gered and CGAS-mediated innate immune response through the regulation of MITA activation
egulator of N6-methyladenosine (m6A) methylation of RNAs, thereby regulating different proce
ation through the regulation of cell death.

ion 4 of the N-acetylgalactosamine (GalNAc) residue of chondroitin. Chondroitin sulfate constit
ramblase activity; scrambles phosphatidylserine, phosphatidylcholine and galactosylceramide
of cellular nucleotides by catalyzing the interconversion of nucleoside phosphates. Has GTP:A
in the modulation of innate immune response. Blocks both the RIG-I-like receptors (RLR) and
lmitoyltransferase specific for HRAS and NRAS.
specifically mediate acetylation of histone H3 at 'Lys-14' (H3K14ac), and have reduced activity
in leukocyte transmigration across the blood-brain barrier. Does not express any beta-galactos
iral infection.
ost-transcriptional regulation of common HLA-A allotypes. Binds to the 3' UTR of HLA-A2 mRN
acellular trafficking.
ction, plays a role in the ribosomal termination-reinitiation event leading to the translation of VP
ated protein kinase (AMPK), an energy sensor protein kinase that plays a key role in regulating
iquitin to other proteins. Functions in the selective degradation of misfolded membrane protein
toskeleton regulation and dynamics. Increases the number and size of actin stress fibers and i
the NF-kappa-B and MAPK pathways. Acts as a mediator of BMP4-mediated modulation of ca
e proteins attached to acetylcholinesterase (AChE).
otein ligase complex that selectively accepts ubiquitin from UBE2H and mediates ubiquitinatior
(2+) entry (SOCE) involved in protecting cells from Ca(2+) overfilling. In response to cytosolic C
ulates a number of transcription factors with key roles in cell fate determination. Positive effect
activates STK11/LKB1. In the complex, required to stabilize the interaction between CAB39/M

d in telomere-length regulation, DNA repair and the maintenance of genomic stability. Acts as a
in peptide trimming, a step required for the generation of most HLA class I-binding peptides. F
uced death of pancreatic beta cells. Involved in the promotion of insulin secretion from pancrea
as both a transcriptional repressor and activator by binding a DNA sequence on these target ge
e XPO1-mediated U snRNA export from the nucleus. Bridge components required for U snRN/
kinases are operationally defined by their preferential utilization of acidic proteins such as case
de is one of the cellular ligands for bacterial verotoxins.||Catalyzes the transfer of galactose fro
skin morphogenesis. Acts as a negative regulator for several EGFR family members, including

involved in the survival of auditory hair cells and hearing. Maybe by modulating the barrier prop
eration or differentiation.
lved in the docking of exocytic vesicles with fusion sites on the plasma membrane. Together w
IT2, and seems to be involved in angiogenesis and vascular patterning. May mediate the inhib
ome complex. Together with the BBSome complex, controls SMO ciliary trafficking and contribu
ntiation and maturation of neuronal cells (By similarity). May regulate the activity of TRIM17. Is
skin keratinocytes. May play a role in the differentiation of spermatozoa and oocytes (PubMed
tion and maintenance of mitochondrial transmembrane potential. Involved in the regulation of F
nol to all-trans-13,14-dihydroretinol. Does not exhibit any activity toward all-trans-retinoic acid, r

nd catalyzes its covalent attachment to other proteins. In vitro catalyzes monoubiquitination an
osphorylation of proteins phosphorylated on Tyr and Ser/Thr residues. In vitro, it can dephosp

n the assembly of small nuclear ribonucleoproteins (snRNPs), the building blocks of the splice
with neuronal ceroid lipofuscinoses (NCL). Also referred to as Batten disease, NCL comprises

maintenance of rapid intracellular calcium release. May act as a potassium counter-ion channe
of deamido-NAD to form NAD (PubMed:12547821). Uses L-glutamine as a nitrogen source (F
liates aggregation of mitochondria resulting in cell death and genomic destruction (MAGD). Pla
tion.
ost-translational formation of 3-hydroxyproline on collagens (PubMed:18487197). Contributes
uclease and exodeoxyribonuclease activities, depending on the divalent metal cation used as
C-terminal glycine residue with ATP, and thereafter linking this residue to the side chain of a c

shifting (-1PRF) of a variety of mRNAs from viruses, such as HIV1, and cellular genes, such as
kinase (PI4-kinase) that catalyzes the phosphorylation of phosphatidylinositol (PI) to phospha
oligosaccharide biosynthesis, the transfer of an N-acetyl-D-galactosamine residue to a serine c
of the nervous system. Involved in autophagy in controlling protein turnover during neuronal de
involved in the in 45S precursor rRNA production.

inds topoisomerase I. The N-terminus contains a proline-rich region and a BTB/POZ domain (b
etromer cargo-selective complex (CSC) mediates the exit of human papillomavirus from the ea
'lippase complex which catalyzes the hydrolysis of ATP coupled to the transport of aminophosp
Ras-like family of Rho- and Rac proteins, by exchanging bound GDP for free GTP. May play a r
f TCEA1 elongation activity (By similarity). Acts as a transcriptional transactivator of ELL and E
on protein. May be involved in the establishment and maintenance of specific neuronal connec
on protein. May be involved in the establishment and maintenance of specific neuronal connec

ition by negatively regulating the SIRT2 deacetylase activity toward alpha-tubulin and thereby p
n of UTP to CTP with either L-glutamine or ammonia as the source of nitrogen. Constitutes the
-5'-adenylyl sulfate (PAPS) as sulfonate donor to catalyze the transfer of sulfate to position 6 o
ctin cytoskeleton assembly and reorganization (PubMed:22330775, PubMed:21730168). Acts

e (lysophosphatidic acid or LPA) into 1,2-diacyl-sn-glycerol-3-phosphate (phosphatidic acid or F

core component of the circadian clock. The circadian clock, an internal time-keeping system, re
etriever complex, in collaboration with the CCC complex, mediates the exit of human papilloma
nent of the endosomal sorting required for transport complex III (ESCRT-III) which is involved i
preferentially hydrolyzes an N-terminal methionine, citrulline or glutamine.
nt attachment of ubiquitin moieties onto substrate proteins. Involved in the TLR and IL-1 signal
ent dioxygenase.
alGAP2 complex which acts as a GTPase activator for the Ras-like small GTPases RALA and I
protein biosynthesis and transport in the endoplasmic reticulum (PubMed:16433634, PubMed
netochore-associated NDC80 complex, which is required for chromosome segregation and spir
e cell membrane and promotes the formation of appositions between the endoplasmic reticulur
ubiquitination of TP73. Acts to stabilize TP73 and enhance activation of transcription by TP73
ther, the penultimate precursor of the pathway for de novo synthesis of platelet-activating facto
roliferation-associated 1 like family. Members of this family contain a GTPase activating domai
ctor (GEF) for CDC42 and RAC1 small GTPases. Through its activation of CDC42 and RAC1,
on protein.

DNA-binding specificity. Represses transcription in a methyl-CpG-dependent manner. Binds witl
n a late step of autophagy, such as clearance of autophagosomal cargo. Plays a key role in inn
d:15009667, PubMed:14594945, PubMed:23666762, PubMed:23184942). Phosphorylates the
phages, which specifically binds to OxLDL (oxidized low density lipoprotein), suggesting that it r
n oncogenic function and regulates cell proliferation, migration and invasion in cancer cells.
channels that contain CACNA1C as pore-forming subunit.
3,4-diphosphate (PtdIns3,4P2), but not to other phosphoinositides. May recruit other proteins

ıke of thiamine pyrophosphate (ThPP) into mitochondria.

velopment in axonal navigation at the ventral midline of the neural tube (By similarity). In spinal
protein response (UPR). In the absence of endoplasmic reticulum (ER) stress, inserted into El
esis.
on of ceramide to form ceramide 1-phosphate (PubMed:11956206, PubMed:16269826, PubMe
tin-protein ligase complex that selectively accepts ubiquitin from UBE2H and mediates ubiquitir
e in behavior, possibly by regulating GABA action. May act by mediating the deubiquitination o
protein. Plays an important role in the expansion and functions of natural killer T (NKT) cells in t
nary cilia. Required for ciliogenesis.
ptidase AUT-like 4 in a head-to-tail orientation. [provided by RefSeq, Jul 2008]
oA. May be involved in utilizing ketone body for the fatty acid-synthesis during adipose tissue d
LH E3 ubiquitin-protein ligase complex that selectively accepts ubiquitin from UBE2H and medi

ulating velocity, spacing and restart activity of replication forks. May couple DNA replication to s
regulation of the circadian rhythm by negatively regulating the activity of the clock genes and c
Plays a role in motile ciliogenesis. May help to recruit STK36 to the cilium or apical surface of th
eta-1,4-N-acetylgalactosamine transferase activity. Transfers glucuronic acid (GlcUA) from UD
dhesion proteins. FAT4 plays a role in the maintenance of planar cell polarity as well as in inhib
retrograde transport. Functions in cilia biogenesis. May play a role in transport between endop
ase 6 complex specifically required for meiotic recombination. Together with SPO11, mediates
ticipates in various processes such as angiogenesis and polyploidization of specialized cells. M
uates TGFB signaling, possibly by accelerating the sequestration of large latent complexes of T

2) and electrons from reduced cytochrome b5 to introduce the first double bond into saturated fa
iired for normal chromosome alignment and segregation and kinetochore formation during mito
that mediates sodium-dependent calcium efflux from mitochondrion, by mediating the exchang
MYC seems to be a direct upstream transcriptional activator. Does not seem to significantly af

l transduction (PubMed:15378603, PubMed:19446606, PubMed:22065575, PubMed:2362592
ylphosphatidylinositol-anchor biosynthesis. Transfers a fourth mannose to some trimannosyl-GI
e exchange factor (GEF), activating Rac proteins by exchanging bound GDP for free GTP. Its a
voltage-gated rapidly inactivating A-type potassium channels. Modulates KCND2 channel dens
vated chloride channel. May play a role in Ca(2+) signal transduction.
ate extracellular matrix-dependent motility and morphogenesis of endothelial and non-endotheli
Isoform 2 may play a role in T-helper 2 (Th2) signaling pathway and seems to represent the fir
nge factor (GEF) for RAB11A.
ective effect in graft versus host disease model (By similarity).

ase that promotes neurite formation and drives spinal motor neuron differentiation (By similarity
CF (SKP1-CUL1-F-box protein) E3 ubiquitin-protein ligase complex which mediates the ubiquit
he transmembrane metalloprotease ADAM10 (PubMed:26668317, PubMed:23035126, PubMe
y. Binds to DNA and acts as a repressor in the absence of CTNNB1, and as an activator in its
ein involved in the formation of the diaphragms that bridge endothelial fenestrae. It is also requ
ı bundling. May neither bind calcium nor depend on calcium for function.
bits cell cycle-dependent localization and can inhibit cell proliferation and migration.
ırowth. May suppress the transcriptional activity of p53/TP53.
oxyl group of serine or threonine residues. The 4 members of the TMTC family are O-mannos
se that plays a key role in the synthesis of lacto- or neolacto-series carbohydrate chains on glyc
osome pairing and efficient cross-over and intragenic recombination during meiosis (By similari

ɔrotease which catalyzes limited proteolysis of substrates involved in cytoskeletal remodeling aı
PubMed:26438880, PubMed:19951071). Acts as an antioxidant (PubMed:19951071, PubMed:

ential for T-cell receptor (TCR)-mediated T-cell activation. Its binding to DPP4 induces T-cell prı
ıtion.
ex, a complex involved in the repair of double-stranded DNA breaks (DBSs) and DNA interstrar
l cell division and cell polarization processes. May play a role in the formation of epithelial tight
ide during the elongation phase. Has also been shown to assemble amyloid fibrils into proteas
laminomethyl modification (mnm(5)s(2)U34) of the wobble uridine base in mitochondrial tRNAs
or apical transport from the Golgi complex. Transports AQP2 from the trans-Golgi network (TG
ıine nucleotide exchange factor which binds to and activates guanine nucleotide-binding proteiı

of NMDA receptor GRIN2B subunit. Plays a role in the regulation of neuronal excitability.
pathway by acting as a ligand for LGR4-6 receptors, which acts as a key regulator of angiogen
oxyl group of serine or threonine residues. The 4 members of the TMTC family are O-mannos
the ubiquitination of AKT1 and GLUL, thereby playing a role in neuron cells differentiation. Pla
5, PubMed:28428259, PubMed:28659385). Recruits the RABL2B GTPase to the ciliary base to

ırtilage and the transition of cartilage to bone.
eceptor coupled to G proteins (PubMed:23763432). Seems to act through a G(i) mediated path
activator for a number of nuclear receptors including PPARA, PPARG, THRA, THRB and RXF

exonuclease activity. Involved in neuronal development, specifically in the development of diffe
ptidase complex which removes signal peptides from nascent proteins as they are translocatec
ıtion.
phology and cytoskeletal organization. Required in the control of cell shape and migration. Req

ıtion.
ı the activation of proinflammatory NF-kappa-B signaling following detection of bacterial pathoç
ment.

ıtion.
bisphosphate (PIP2) to generate 2 second messenger molecules diacylglycerol (DAG) and inos
hat determine the pharmacology and kinetics of the receptor response. Increases agonist poter

diated endocytosis. Has both a membrane binding/bending activity and the ability to recruit pro
l heat shock protein 70 (Hsp70) ATPase domain and is therefore a distant member of the mam
ce similarity to rat, xenopus and zebrafish proteins. The protein function is unknown. [provided l
se (I-CLiP) that cleaves type II membrane protein substrates in or close to their luminal transm
superfamily, a novel family that is similar to the chemokine and the transmembrane 4 superfam

ersion of beta-D-galactose and alpha-D-galactose during galactose metabolism (PubMed:1275

type-2 family. The encoded enzyme catalyzes the phosphorylation of (R)-glycerate and may b
or and a cytoplasmic regulator of actin dynamics depending on conditions. In nucleus, acts as a

role in mitotic cell cycle progression. Required for microtubule nucleation activity of the centros
ubiquitination of Delta receptors, which act as ligands of Notch proteins. Positively regulates tl

in a decrease in sAPP-alpha production and increased amyloidogenic P3 peptide production.

ıich may act as a tyrosine phosphatase.
ding pathogens. Adapter used by TLR3, TLR4 (through TICAM2) and TLR5 to mediate NF-kap
ıtion.
of presomitic mesoderm cells by individual attenuation of both FGF and WNT signaling.
plasmic reticulum stress response or unfolded protein response (UPR). Represses the transcri
ort. Regulates aldosterone-induced and epithelial sodium channel (ENaC)-mediated sodium tr
nune system and is involved in responses to infections.
velopment and function. May regulate cardiac conduction and the function of the gap junction ı

rowth-factor-mediated activation of MAP kinase (By similarity). Negatively regulates hematopoi
ition.
ition.

ocytosis of signal sequence-containing cytokines such as CCL5. Probably acts in cooperation w
'hich acts as an inhibitor of retrovirus replication and retrotransposon mobility via deaminase-de
ways and downstream activation of MAP kinases (PubMed:15683364). Inhibits fibroblast growt
otein that belongs to the cell division control protein 42 effector protein family. In neurons, it loc
rrier family of proteins.[provided by RefSeq, Dec 2009]
TRIM) family of proteins. This protein may be regulated by the tumor suppressor p53 and may r
rvation-induced autophagy (PubMed:25076851).

sphate antiporter. May transport cytoplasmic glucose-6-phosphate into the lumen of the endop
ssor.

ensure proper cell division and nuclear envelope reassembly by sequestering the endoplasmic
cifically demethylates 'Lys-9' of histone H3, thereby playing a central role in histone code. Deme
letal dynamics, plays a role in actin stress fiber formation.
a-specific synaptic connections in the retina. Expressed in specific subsets of interneurons and
ifferentiation.

itoylation of KCNMA1, regulating localization of KCNMA1 to the plasma membrane. May be in
s ubiquitin from E2 ubiquitin-conjugating enzymes UBE2L3 and UBE2L6 in the form of a thioes
cytoskeleton, endocytosis and cellular vesicle trafficking via its interactions with membranes, W
ng 1,3-bisphosphoglycerate as a phosphate donor and a series of 1-phosphate sugars as acce
elin family. This gene is expressed in the inner ear of vertebrates with the highest level of expre
n regulated exocytosis of insulin granules.
on of neuroendocrine signals accessing the anterior pituitary gland. Stimulates water drinking a

of AP-2 family members, including TFAP2A, TFAP2B and TFAP2C to various extent.
ent. Plays a role in neuronal proliferation and migration (PubMed:20890278, PubMed:2072983
s BRAF, inducing its proteasomal degradation.
f cysteine to 3-oxoalanine on target proteins, using molecular oxygen and an unidentified reduc

es polyubiquitination and degradation by the proteasome pathway of ZIC2.
phatase. The mouse homolog of this gene apparently belongs to the protein phosphatase 2C fa
tors of intracellular membrane trafficking, from the formation of transport vesicles to their fusion
c regulator and an effector of alpha1-adrenergic signaling. Binds to PE response elements (PE

/ by neutralizing basic proteins such as A and B core hnRNPs.

ıtion.

) to Fe(2+) before its transport from the endosome to the cytoplasm.

ılating the adaptive immune response to yellow fever virus infection; promotes dendritic cells tc

Involved in the regulation of liver cancer cells self-renewal (PubMed:25985737). Involved in ar

nzymes that catalyze the hydrolysis of acyl-CoAs into free fatty acids and coenzyme A (CoASH

ion of autophagosomal vacuoles (autophagosomes). Plays a role in mitophagy which contribut

g signaling probably by promoting internalization and subsequent degradation of smoothened p
ade, when overexpressed (PubMed:28688764). Induces apoptosis, when overexpressed (Publ

ı endopeptidase and 2-oxoglutarate-dependent monoxygenase (PubMed:28847961, PubMed:2
RNAs that are involved in post-transcriptional regulation of gene expression in multicellular org
OXA gene cluster. This transcript may regulate expression of HOXA genes in cis. This gene is
ı transcription between the neighboring PMF1 (polyamine-modulated factor 1) and BGLAP (bo
-related GTP-binding proteins and subsequent activation of transcription factor SRF (By similar

e activin type-1 receptor forming an activin receptor complex with activin receptor type-2 (ACVF

s its action by association with G proteins that activate a phosphatidylinositol-calcium second m
ne-bound isoforms by competing for the same interaction partners. Inhibits cell attachment via
inoic acid. Binds free retinal and cellular retinol-binding protein-bound retinal (By similarity). Ma
ɔiphosphate aldolase gene family. Expressed specifically in the hippocampus and Purkinje cells
lial cells; once bound, angiogenin is endocytosed and translocated to the nucleus. Stimulates r
cytosis by binding to Munc18-1, an essential component of the synaptic vesicle exocytotic mac

of the membrane integral V0 complex of vacuolar ATPase. V-ATPase is responsible for acidify
exhibiting weak selectivity for potassium over sodium ions. Contributes to the native pacemake

lly mediates the formation of 'Lys-6'-linked polyubiquitin chains and plays a central role in DNA
nd/or homologous recombination. Binds RAD51 and potentiates recombinational DNA repair b

s ubiquitin from specific E2 ubiquitin-conjugating enzymes, and transfers it to substrates, gener
the G2/M transition phases of the cell cycle. Functions through the formation of specific serine
erous organic anions and conjugated organic anions such as methotrexate, 17beta-estradiol 1
es deoxycytidine, deoxyguanosine and deoxyadenosine (PubMed:1996353, PubMed:1280844
m, 20-alpha-dihydroxyprogesterone (20-alpha-OHP). In the liver and intestine, may have a role
that participates in the import and insertion of some multi-pass transmembrane proteins into t
ate to orotate with quinone as electron acceptor.
e anticodon loop of tRNA(Asp).

ellular protrusions by regulating actin cytoskeleton dynamics and architecture. Depending on it
postreplication repair or a cell cycle checkpoint function. May be implicated in interstrand DN
galactosylceramide, galactosylsphingosine, lactosylceramide, and monogalactosyldiglyceride.
GHBP and acts as a negative inhibitor of GH signaling.||Receptor for pituitary gland growth ho
uptake and metabolism.
tamylcysteine synthetase, is the first rate limiting enzyme of glutathione synthesis. The enzyme
lgi apparatus level. Involved in endosome-to-Golgi trafficking.
wide number of exogenous and endogenous hydrophobic electrophiles. This isozyme has a hi
ensation of nucleosome chains into higher-order structures. The histones H1.0 are found in cel
ventional H2A in a subset of nucleosomes. Nucleosomes wrap and compact DNA into chroma
UA monosaccharides to the nascent hyaluronan polymer. Therefore, it is essential to hyalurona
hepatocyte cells, seems to be a hepatotrophic factor, and acts as a growth factor for a broad s
in different cellular compartments. May act in a redox sensitive manner. In the nucleus is an a
al DNA thus altering the interaction between the DNA and the histone octamer. May be involve
hich is part of a developmental regulatory system that provides cells with specific positional ide
-secretion with insulin, and acts as physiological amplifier of glucose-mediated insulin secretio
NA duplices in an ATP-dependent reaction. Acts as a transcription regulator. Required for the
, respectively, the secretion of follitropin by the pituitary gland. Inhibins/activins are involved in r
erferon (IFN)-dependent immune responses which plays a critical role in the innate immune re
ted potassium KCNMA1 (maxiK) channel. Modulates the calcium sensitivity and gating kinetics
bipolar spindle during mitosis (PubMed:19001501). Required in non-mitotic cells for transport
ent motor required for bipolar spindle formation (PubMed:15843429). May contribute to movem
ydrolyzes stored triglycerides to free fatty acids, while in steroidogenic tissues, it principally co

ma) and ACTH. The activity of this receptor is mediated by G proteins which activate adenylate
plex (MCM complex) which is the putative replicative helicase essential for 'once per cell cycle'
plex (MCM complex) which is the putative replicative helicase essential for 'once per cell cycle'
plex (MCM complex) which is the putative replicative helicase essential for 'once per cell cycle'
X6. Acts as a transcriptional activator of PF4 in complex with PBX1 or PBX2. Required for hem
wide number of exogenous and endogenous hydrophobic electrophiles. Has a wide substrate s
mponents of the extracellular matrix, such as collagen type III and fibronectin. Activates proge
bdopterin biosynthesis. Component of the molybdopterin synthase complex that catalyzes the
cysteine residues that bind various heavy metals; these proteins are transcriptionally regulated
r. Initiates repair of A*oxoG to C*G by removing the inappropriately paired adenine base from t
nembrane respiratory chain NADH dehydrogenase (Complex I), that is believed to be not invol
nembrane respiratory chain NADH dehydrogenase (Complex I), that is believed not to be invol

none reductase in connection with conjugation reactions of hydroquinones involved in detoxifica
y, dominant-negative form that may down-regulate eNOS activity by forming heterodimers with
normal responses to cell-cell contacts in brain and in the peripheral nervous system. Plays a r
ons during nervous system development. Has some neurite outgrowth-promoting activity (By s
atrix of the centrosome involved in the initial establishment of organized microtubule arrays in
h a dual-specificity for the second messengers cAMP and cGMP, which are key regulators of r
hase involved in the purines biosynthetic pathway. Catalyzes the ATP-dependent conversion o
cross the membrane (PubMed:8898203, PubMed:2897240, PubMed:9038218). Catalyzes the
hosphorylation of serine in certain substrates, including troponin I. The alpha chain may bind ca
kinase (PHK), which mediates the neural and hormonal regulation of glycogen breakdown (gly
bMed:8806613, PubMed:27694521). Part of the complex catalyzing the transfer of N-acetylglu
ugh the transfer of ethanolamine phosphate to the third mannose of GPI.
er of N-acetylglucosamine from UDP-N-acetylglucosamine to phosphatidylinositol, the first step
ed intracellular signaling cascade. Shows no PLC activity to phosphatidylinositol 4,5-bisphospha
nnose and dolichol-phosphate-mannose required for a number of critical mannosyl transfer rea
osome complex which has 3'->5' exoribonuclease activity and participates in a multitude of cellu
-box 1 core sequence of the E-cadherin promoter gene; the core-binding sequence is 5'CAGG
nerase epsilon complex (PubMed:10801849). Participates in DNA repair and in chromosomal D
yzes the transcription of DNA into RNA using the four ribonucleoside triphosphates as substrate
complex involved in the proteolytic degradation of most intracellular proteins. This complex pla
point response complex that plays a major role in DNA repair (PubMed:10846170, PubMed:1C
of peroxisomes.
yl moiety from geranylgeranyl diphosphate to both cysteines of Rab proteins with the C-termina
s by DNA polymerase delta and epsilon requires the action of the accessory proteins proliferati
s by DNA polymerase delta and epsilon requires the action of the accessory proteins proliferati

s by DNA polymerase delta and epsilon requires the action of the accessory proteins proliferati
s by DNA polymerase delta and epsilon requires the action of the accessory proteins proliferati

protein A complex (RPA/RP-A), binds and stabilizes single-stranded DNA intermediates that fo
esis, consist of a small 40S subunit and a large 60S subunit. Together these subunits are com
NA synthesis. Catalyzes the biosynthesis of deoxyribonucleotides from the corresponding ribo
paran sulfate. Regulates dendritic arbor morphogenesis (By similarity).
which participates in the control of pre-mRNA splicing. Can either activate or suppress exon in
involved in the regulation of a wide variety of ion channels, membrane transporters, cellular er
nt role in embryonic development as well as in cellular growth and proliferation; its long-term si
CF (SKP1-CUL1-F-box protein) E3 ubiquitin-protein ligase complex which mediates the ubiquit
acid transporter that mediates the uptake of L-glutamate and also L-aspartate and D-aspartate
e receptor for Baboon M7 endogenous virus.||(Microbial infection) Acts as a cell surface recep
in synaptic activity such as regulation of synaptic vesicle trafficking and subsequent neurotran
onent of the spliceosome (PubMed:11991638, PubMed:28502770, PubMed:28781166, PubMe
component of the SMN-Sm complex that mediates spliceosomal snRNP assembly and as com
for sperm head formation but not required to establish and maintain general polarity of the sper
CACNA1H at the cell membrane, and thereby contributes to the regulation of channel activity.
ty.
neurotransmitter calcium-dependent exocytosis and endocytosis (PubMed:26635000). Part of t
lly trimethylates 'Lys-9' of histone H3 using monomethylated H3 'Lys-9' as substrate. Also weal
nilarity). Involved in calcium interactions and contractile properties of the cell that may contribut
ell lymphocyte differentiation. Necessary for the survival of CD4(+) CD8(+) immature thymocyte
iral and cellular genes by binding to the symmetrical DNA sequence 5'-CAGCTG-3'.
capturing and retaining TGF-beta for presentation to the signaling receptors.
l-to-cell and cell-to-matrix interactions. Binds heparin. May play a role in dentinogenesis and/or
diate the innate immune response to bacterial lipopolysaccharide (LPS) (PubMed:27022195). A
zation of the nucleus and in the post-mitotic nuclear assembly. Plays an important role, togethe
tension of DNA introduced during the DNA replication and transcription by transiently cleaving
ivity as well as thioredoxin reductase activity and induces actin and tubulin polymerization, lead
cleavage of uridine and deoxyuridine to uracil and ribose- or deoxyribose-1-phosphate (PubMe
ir which specifically deubiquitinates monoubiquitinated FANCD2 (PubMed:15694335). Also inv
HTLV-1 TAX-dependent transactivation of viral long terminal repeat (LTR) promoter.||Zinc-fin
tion.
tion.
r transport. Required, directly or indirectly, for the localization of NCAPD2 to the proximal ends
aft-mediated trafficking in endothelial cells. The encoded protein, a member of the MAL proteol
dehydrogenase with highest activity towards estradiol. Has very low activity towards testostero

\4 histone acetyltransferase (HAT) complex, a complex involved in transcriptional activation of
as sodium-independent, high-affinity transporter that mediates uptake of large neutral amino a
matin assembly in DNA replication and DNA repair. Assembles histone octamers onto replicati
›somes wrap and compact DNA into chromatin, limiting DNA accessibility to the cellular machir
d histone metabolism via its interaction with HIRA and histones.
j the GTPase activity of G protein alpha subunits thereby driving them into their inactive GDP-b
activation of the lectin complement pathway. Calcium-dependent and GlcNAc-binding lectin. H
hosphatase of the plasma membrane that catalyzes the dephosphorylation of a variety of glyce
:ium and phosphate homeostasis.

e from GTP and L-fucose-1-phosphate. Functions as a salvage pathway to reutilize L-fucose a
E/OPGL; essential for RANKL-mediated osteoclastogenesis. Involved in the regulation of intera
nsible for the binding of the proinflammatory cytokine IL18, but not IL1A nor IL1B (PubMed:86[illegible]
ate in the presence of sodium.
DNA replication, maintenance of replication fork stability, maintenance of genome stability thro

ribonucleoprotein particle (snoRNP) thought to participate in the processing and modification c
oliferation and induces apoptotic cell death through activation of interleukin-1-beta converting e
similarity). Acts as a positive regulator of apoptosis.
ıembrane proteins with members of the ezrin/moesin/radixin family and thereby helps to link the
late endosomes (PubMed:19696025). May play a role in modulating membrane acid-sensing i
mplex of vacuolar ATPase (V-ATPase). V-ATPase is responsible for acidifying a variety of intra
or that directs the termination of translation in response to the peptide chain non-cognate termi
es beta-tubulin and non-tubulin proteins, such as NAP1L1, NAP1L4 and CGAS. Involved in the
kpoint, which prevents cells from prematurely exiting mitosis. Required for the assembly of the
t different stages of procentriole formation. Acts as a key negative regulator of ciliogenesis in co

ed vesicles and promotes uncoating of clathrin-coated vesicles. Plays a role in clathrin-mediate
l in various processes such as cell adhesion, regulation of cell ploidy and senescence, cell prol
(DDR). Part of the TTT complex that is required to stabilize protein levels of the phosphatidylir
EF) which may activate RAB10. Promotes the exchange of GDP to GTP, converting inactive GI
ss-link repair. Also required for checkpoint mediated cell cycle arrest in early prophase in respo
e cullin-RING-based E3 ubiquitin-protein ligase (CRLs) complexes which mediate the ubiquitina
ne residues on the N-terminal part of the core histones (H2A, H2B, H3 and H4) (PubMed:1022
a complex that is thought to mediate chromatin assembly in DNA replication and DNA repair. A
ıs in their ATP-dependent uptake into the mitochondria. Plays a crucial role in heme synthesis.
ependent transcriptional activation activity (PubMed:19481530, PubMed:25661920). Interactio
the assembly of the mitochondrial respiratory chain complex IV (CIV), also known as cytochrom

activity. Functions as an inhibitor of the early TH1 cytokine response.
lrocytes in the early stages of their terminal differentiation into myelin-forming glia and may also
grity of mitotic centrosomes and in the maintenance of spindle bipolarity by promoting PLK1 act

version of lysophosphatidylcholine (1-acyl-sn-glycero-3-phosphocholine or LPC) into phosphati
egulates the degradation of target proteins. Polyubiquitinates MYD88. Negatively regulates MY
thylation) the guanidino nitrogens of arginyl residues in some proteins.

aveolar membranes, functionally participating in formation of caveolae or caveolae-like vesicles
ng. Required for the export of mRNA out of the nucleus.

loid-beta precursor protein.
s (By similarity). Seems to be directly involved in T-cell activation.
complex/cyclosome (APC/C), a cell cycle-regulated E3 ubiquitin ligase that controls progressio
eudouridine N(1)-methyltransferase that methylates pseudouridine at position 1248 (Psi1248) i
nt coupling of the nucleus and the centrosome. Involved in the processes that regulate centros
60S ribosomal subunit biogenesis. Core component of box C/D small nucleolar ribonucleoprot
bits BCL2L1 isoform Bcl-x(L) anti-apoptotic activity. Inhibits activation of NF-kappa-B and prom
dle required for normal chromosome segregation and progression into anaphase (PubMed:117
complex. The PCAF complex is capable of efficiently acetylating histones in a nucleosomal co
and plays a role in cell adhesion (PubMed:12235007). Enhances incorporation of BMP1 in the
polymerase delta complex and the DNA polymerase zeta complex (PubMed:22801543, PubMe
ys a central role in centriole duplication. Able to trigger procentriole formation on the surface of
ular growth, and differentiation. The specific function of this retinoic acid-induced gene has not
yase involved in the cleavage (C1 removal) reaction in the fatty acid alpha-oxydation in a thiam
nery.
catalyzes ascorbate-dependent trans-membrane ferric-chelate reduction.
uired for kinetochore formation and spindle checkpoint activity. Required to target ZW10 to the
erase HBO1 complexes, which specifically mediate acetylation of histone H3 at 'Lys-14' (H3K14
, specifically in homologous strand assimilation, which is required for the resolution of meiotic
on of adenosine 5'-monophosphate (AMP) to specific residues of target proteins (AMPylation),
ponent of the U4/U6-U5 tri-snRNP complex that is involved in spliceosome assembly, and as c
mage, functioning as part of both the non-homologous end-joining (NHEJ) and base excision re
entromeres and normal chromosome segregation during mitosis.
osome complex which has 3'->5' exoribonuclease activity and participates in a multitude of cell
orientation during development of sensory cortex. Also directs dendrites toward active axon te
inhibiting LRP5/6 interaction with Wnt and by forming a ternary complex with the transmembra

in transcriptional regulation through histone methyltransferase (HMT) complexes. Plays a role i
r suppressor.
specific endoglucosamine-6-sulfatase activity. It can remove sulfate from the C-6 position of gl
ms a linking network between organelles and the actin cytoskeleton to maintain the subcellular
Hsp70 family proteins HSPA1A, HSPA1B and HSPA8.
nplex, a complex required for conversion of interphase chromatin into mitotic-like condense chr
osome complex which has 3'->5' exoribonuclease activity and participates in a multitude of cellu
both coactivator and corepressor functions. Isoform 1, but not other isoforms, is involved in the
membrane synthesis and myelin production.
ole in macrophage lipid homeostasis.
recursor for the side chain of the isoprenoid quinones ubiquinone-10.
it interacts with single-stranded DNA and is involved in the DNA damage response and mainter
oonent of the U4/U6-U5 tri-snRNP complex that is involved in spliceosome assembly, and as c

PubMed:9367539). Receptor for IL17F (PubMed:19838198, PubMed:17911633). Binds to IL1
of the tight junction complexes that regulate the permeability of epithelia. While some claudin f

e deposition and histone exchange and removal during nucleosome assembly and disassembl
ochondria.
ocessing by associating with U7 snRNP and interacting with SLBP/pre-mRNA complex. Increas
complex/cyclosome (APC/C), a cell cycle-regulated E3 ubiquitin ligase that controls progressio
th a preference for the long chain fatty acyl-CoA thioesters hexadecanoyl-CoA/palmitoyl-CoA a

apoptosis; the exact mechanism may differ between cell types/tissues. May be involved in hyp
chaperone that participates in the import and insertion of some multi-pass transmembrane prot

ism and/or modifications.
esses the invasive behavior of tumor cells (By similarity). May play a role in MDM2-dependent p

-5'-adenylyl sulfate (PAPS) as sulfonate donor to catalyze the sulfate conjugation of many horr
numerous drugs and which is highly expressed in various proliferating cancer cells (PubMed:28
tes activation of MAP kinases. Does not display kinase activity (By similarity).
oded by nuclear genes and help in protein synthesis within the mitochondrion. Mitochondrial rib
subgroup of the melanoma-associated antigen (MAGE) superfamily. The encoded protein is like

oded by nuclear genes and help in protein synthesis within the mitochondrion. Mitochondrial rib
ne nuclear receptor ESR1 required to induce the expression of a subset of estradiol target gene
al NADH:ubiquinone oxidoreductase complex (complex I) (PubMed:18179882, PubMed:28853

t peptide-containing proteins across the mitochondrial inner membrane. Also required for asse
endoplasmic reticulum-stress induced autophagy.
th proteins and DNA, and to a lower extent, arsenic (PubMed:18539146, PubMed:21193388, F
omplex, a complex that seems to provide chromosomes with an additional level of organizatior
se 2 family. The encoded protein participates in glucosylation of the oligomannose core in N-li
bination. Stimulates DMC1-mediated strand exchange required for pairing homologous chrom
se, an essential precursor of glycan moieties of glycoproteins and glycolipids.
l-linked oligosaccharide precursor for N-linked glycosylation. Transfers glucose from dolichyl p
nome stability by protecting stalled or damaged replication forks. After the induction of replicat
sporter that mediates the transport of glucose and fructose. Also able to mediate the transport
receptor for herpes simplex virus 1.||Paired receptors consist of highly related activating and in
nique DNA binding specificity for CpG unmethylated motifs with a preference for CpGG.
phates of deoxyribonucleotides, with a preference for dUMP and dTMP, intermediate activity tc
yzes the transcription of DNA into RNA using the four ribonucleoside triphosphates as substrate
tion of transcription (PubMed:17662942). Required for normal mitochondrial transcription and
x that is required for the formation of a threonylcarbamoyl group on adenosine at position 37 (t(
coactivator involved in the regulated transcription of nearly all RNA polymerase II-dependent ge
an nucleoli. A similar protein in budding yeast is a component of pre-60S ribosomal particles, a
oded by nuclear genes and help in protein synthesis within the mitochondrion. Mitochondrial rib
ween acetaldehyde and D-glyceraldehyde 3-phosphate to generate 2-deoxy-D-ribose 5-phosp
ily. This protein localizes to the centriolar sub-distal appendages that are associated with the o
complex (RAC), a complex involved in folding or maintaining nascent polypeptides in a folding-
monocytes, neutrophils and lymphocytes (PubMed:11415443). Binds CCR4 (PubMed:16137
capacity to bundle and stabilize microtubules (By similarity). May associate with chromosomes

ondrial respiratory chain complex IV (CIV), also known as cytochrome c oxidase (PubMed:293

complex that generates mature tRNA molecules by cleaving their 5'-ends (PubMed:11413139
ssembly of standard proteasomes and immunoproteasomes. Degraded after completion of pro
ucleolus. Expression of this gene is induced by estrogens and Myc protein and is a marker of p
nation induced by active diaphanous protein homolog 1 (DRF1). Induces microspike formation,
PK8 activity in response to various stress stimuli. Facilitates degradation of misfolded endopla

obably by regulating membrane fission. Loss-of-function induces the release of cytochrome c, v
ding complex (CBC) and the primary microRNAs (miRNAs) processing machinery during cell p
onent of the spliceosome (PubMed:11991638, PubMed:28502770, PubMed:28076346). PPIase

:ochondria.
ed for sonic hedgehog/SHH signaling. May mediate transport of SHH components: required for
/zes the transcription of DNA into RNA using the four ribonucleoside triphosphates as substrate
ıs. It catalyzes the cis-trans isomerization of proline imidic peptide bonds in oligopeptides. May
entromeres and normal chromosome segregation during mitosis.
m of the cytochrome c heme group can accept an electron from the heme group of the cytochr
promoting mitochondrial fission.
o acid transporter. Mediates the saturable, pH-sensitive and electrogenic cotransport of neutral

ıbiquitin-protein ligases, including ITCH, NEDD4, NEDD4L, SMURF2, WWP1 and WWP2, and
ed for ciliogenesis, formation of primary non-motile cilium, and recruitment of RAB8A to the bas

component of the spindle assembly checkpoint. Contributes to the mitotic checkpoint by recruiti
131492, PubMed:11162141). May have a role in spermatogenesis where it promotes autophag

lays an important role in cell-cell signaling. PLXNB2 binding promotes downstream activation o
oset of G-protein coupled receptors.
DNA replication and the maintenance of replication fork stability. Important for cell survival afte

ponent of the spliceosome (PubMed:28502770, PubMed:29301961, PubMed:30705154). Plays
otein. Severs microtubules along their length and depolymerizes their ends, primarily the minus
al distribution and morphology (PubMed:17349998, PubMed:28554942, PubMed:28544275). F
some distal) complex, a complex recruited to centromeres which is involved in assembly of kin
protein, lines, which plays important roles in development of the epidermis and hindgut. This ge
NA double-strand breaks by homologous recombination and in the repair of interstrand DNA cr

ıger, a motif present in a variety of functionally distinct proteins and known to be involved in pro
division and germ cell differentiation through regulation of pairing and recombination during me
ıtion.
ondrial membrane respiratory chain NADH dehydrogenase (Complex I) (PubMed:20858599, P
that is critical for the anterograde axonal transport of active zone components and contributes
x linking beta-integrins to the actin cytoskeleton, bridges the complex to cell surface receptor ty
ıe wobble position (U34) of mitochondrial tRNA(Lys), tRNA(Glu) and tRNA(Gln). Required for th
somal proteins to the centrosome. Provides centrosomal microtubule-nucleation activity on the
and cone photoreceptor cells (By similarity). It is required for recruitment and proper localizatio
ıd centrosome function by participating in centriole duplication (PubMed:17681131, PubMed:2
s to be active only in mitosis. May also play a role in the activation of lymphoid cells. When pho
taining proteins. Also plays a role in mRNA nuclear export.

on of skeletal muscle differentiation, independently of its glycerophosphocholine phosphodieste

ns and the hemichannels.
thetic axons. Ligand of NRP2 (By similarity).
e, a modified base found in the D-loop of most tRNAs.

se.
EF) for Ral-A. Potential effector of GTPase HRas and Ras-related protein M-Ras. Negatively re
n.
ecessary for maintaining optimal levels of dolichol-linked oligosaccharides. Hydrolyzes dolichyl

eaves 'Lys-63'-linked polyubiquitin chains. Does not cleave 'Lys-48'-linked polyubiquitin chains.
99413). Necessary for microtubules and mitotic spindle organization (PubMed:24867236). Invo
exchange factor (GEF), which activates the Rac proteins by exchanging bound GDP for free G
Ca(2+) entry (SOCE), a Ca(2+) influx following depletion of intracellular Ca(2+) stores. Functio
nucleotide excision repair (TC-NER) in response to UV damage. TC-NER allows RNA polymer

ponent of the U4/U6-U5 tri-snRNP complex that is involved in spliceosome assembly, and as c
on particle) receptor. Ensures, in conjunction with the signal recognition particle, the correct tar
oline between membranes. Binds a single lipid molecule.
tion.
essor through its interaction with COPS2, negatively regulating the expression of genes involve
on-induced autophagy.
nyloid-beta precursor protein (APP), and hence allows formation of amyloid-beta. May enhance
egulates p21 protein stability by binding to Hsp90 and p21.
ycle arrest in response to inhibition of DNA replication or to DNA damage induced by both ioniz
BS) repair via homologous recombination (HR). Recruited at DSB sites independently of BRCA
some distal) complex, a complex recruited to centromeres which is involved in assembly of kin
nplex, a complex required for conversion of interphase chromatin into mitotic-like condense chr

GGAATRCC-3' Ikaros-binding sequence. Transcriptional repressor. Interacts with SPI1 and M
cytoplasm to vacuole transport (Cvt), autophagy, and mitochondrial homeostasis. Responsible
al small ribosomal subunit.
le in the initiation of DNA replication, and progression of DNA replication forks. GINS complex
organelle that assembles around clusters of rRNA genes and functions in ribosome biogenesis.
gous recombination that acts by recruiting the MCM8-MCM9 helicase complex to sites of DNA
t DNA double-strand breaks (DSBs) following DNA damage and promotes DNA resection and
some-associated) complex, a complex that plays a central role in assembly of kinetochore prot

tion of the four reactions that constitute the long-chain fatty acids elongation cycle. This endopl

group of estrone, 4- and 5-androstenes and 5-alpha-androstanes into their 17-beta-hydroxyl m
some distal) complex, a complex recruited to centromeres which is involved in assembly of kin
/). Might play a role in the unfolded protein response (By similarity). May regulate transport of a
plex of the TREX complex which is thought to couple mRNA transcription, processing and nucl
maintenance of centrosome integrity and completion of cytokinesis as part of the HAUS augm
ansferase MLL2/MLL3 complex is suggesting a role in epigenetic transcriptional activation. How
ltransferase A (NatA) complex which displays alpha (N-terminal) acetyltransferase activity.
oligosaccharide biosynthesis, the transfer of an N-acetyl-D-galactosamine residue to a serine c

some-associated) complex, a complex that plays a central role in assembly of kinetochore prot
orters.
lly trimethylates 'Lys-9' of histone H3 using monomethylated H3 'Lys-9' as substrate. H3 'Lys-9

creases mitochondrial RNA polymerase processivity. Regulates transcription of the mitochondri
n the assembly of small nuclear ribonucleoproteins (snRNPs), the building blocks of the splice
n the assembly of small nuclear ribonucleoproteins (snRNPs), the building blocks of the splice
nicrotubule organization.
cleoprotein complex that generates mature tRNA molecules by cleaving their 5'-ends.
ase, a FAD-dependent enzyme that oxidizes L-2-hydroxyglutarate to alpha-ketoglutarate in a va
xes, which have a histone H4 acetyltransferase activity (PubMed:16387653, PubMed:1918776
x, a complex that plays an important role in the processing of homologous recombination interr
n dorsal root ganglion (DRG) neurons in vitro. May be a stop signal for the DRG neurons in the
receptor, mediating natural killer cell cytotoxicity.

ng protein that represses transcription via recruitment of HDAC3 and nuclear hormone recepto
iates the genome-wide removal of histone H2A.Z/H2AZ1 from the nucleosome: removes H2A.
ates iron uptake. Probably required for heme synthesis of hemoproteins and Fe-S cluster asser
tion of 1,5-anhydro-D-fructose (AF) to 1,5-anhydro-D-glucitol (By similarity). Has low NADPH-d
IA helicase required for the maintenance of chromosomal stability. Acts late in the Fanconi ane
ent. Has an essential role in the biosynthesis and trafficking of glycosaminoglycans and glycop
DNA damage. Required for ATR expression, possibly by stabilizing the protein.
nucleolar processing of pre-18S ribosomal RNA. Required for optimal pre-ribosomal RNA tran
rotein assembly (CIA) complex, a multiprotein complex that mediates the incorporation of iron-
ally mediates phosphorylation at the 6-position of an O-mannose of the trisaccharide (N-acetylg
nembrane protein of unknown function. Defects in this gene are a cause of optic atrophy type 7
ed by NF-kappa-B and the serum response element.

se (DDR) pathway by regulating postreplication repair of UV-damaged DNA and genomic stabi
dative stress. Decreases cell proliferation.
pecifically responsible for potassium conductance in endosomes and lysosomes. Forms a pota
nol-cytochrome c reductase complex (mitochondrial respiratory chain complex III or cytochrome

Lys-79' of histone H3. Nucleosomes are preferred as substrate compared to free histones (Pub
d increases the activity of different structure-specific endonucleases. Has several distinct roles
TB-CUL3-RBX1) E3 ubiquitin ligase complex that acts as a regulator of neural crest specificatio
in propionyl-CoA metabolism.
of the mitochondrial ribosome (PubMed:24948607). Is a positive regulator of mitochondrial prot

-sn-glycerol-3-phosphate (lysophosphatidic acid or LPA) by incorporating an acyl moiety at the
rylation in unstressed cells by promoting its dephosphorylation by PP1.
d-linked oligosaccharide precursor for N-linked glycosylation. Transfers glucose from dolichyl p

otic spindle. Maintains the structural integrity of centrosomes during mitosis.
thway; activates WNT3A signaling via DVL2. Regulates JNK activation by AXIN1 and DVL2.
protein involved in centriole biogenesis (PubMed:20844083, PubMed:25131205, PubMed:2718

TB-CUL3-RBX1) E3 ubiquitin-protein ligase complex required for mitotic progression and cytoki
rugs, such as indisulam (E7070) or E7820, change the substrate specificity of the DCX(DCAF
coactivator involved in the regulated transcription of nearly all RNA polymerase II-dependent ge
S protein UQCRFS1 incorporation into the cytochrome b-c1 (CIII) complex. Functions as a cha

the ubiquitin-mediated degradation of protein kinase C.

signaling by class 3 semaphorins and subsequent remodeling of the cytoskeleton. Plays a role

oxin-fold anticodon-binding domain which is contained in a subset of phenylalanyl tRNA synthet
pathway. Crucial for heart development and acts downstream of GATA factors in the pre-cardia
energic receptor/ADRB2, it negatively regulates the adrenergic receptor signaling pathway (Pul

synthetase protein that is encoded by the nuclear genome and imported to the mitochondrion.
maintenance of centrosome integrity and completion of cytokinesis as part of the HAUS augm
droxy-L-proline to Delta(1)-pyrroline-2-carboxylate (Pyr2C). May be required to degrade trans-3

respiration essentially in the testis. Can also promote mitochondrial fission (By similarity).
n mitosis stabilizing cohesin complex association with chromatin. May antagonize the action of
em into M1-like macrophages through the activation of the MAPK and NF-kappaB signaling pa
nduces CDKN1A expression as well as TGF-beta expression. Mediates the inhibitory growth eff
carrier. Mitochondrial solute carriers shuttle metabolites, nucleotides, and cofactors through th
controlling its subcellular localization.
erized by cell swelling, organelle swelling, vacuolization and increased membrane permeability.
f the plasma membrane (PubMed:30509349). Inhibits the incorporation of membrane-fluidizing
tion of centriole duplication leading to defects in cytokinesis.
t mediates transport of serine into mitochondria.
criptional activities of SRE and AP-1.
and RNA containing 1-methyladenine and 3-methylcytosine by oxidative demethylation. Can a
ated signaling cascades which result in the activation of NK-kappaB and JNK1. Probable subs
led:20551181). Required for the recruitment of PLK1 to centrosomes and S phase progression

ch as the MLL1/MLL and NURF complexes.

s are involved in phagocytosis, antibody-dependent cell cytotoxicity, immediate hypersensitivity
ctor (GEF) for RhoA GTPase.
r pore complex (NPC). NPC components, collectively referred to as nucleoporins (NUPs), can

egulating both the translocation and the ubiquitin-mediated proteasomal degradation of nascen

half of centrioles, required for centriole elongation.

ch accepts ubiquitin from an E2 ubiquitin-conjugating enzyme in the form of a thioester and the
otein. The encoded protein may function as a tumor suppressor. A translocation that results in
ucture-specific endonuclease with substrate preference for branched DNA structures with a 5'-
tion.
etochore-associated NDC80 complex, which is required for chromosome segregation and spir
ediates C-mannosylation of tryptophan residues on target proteins.

tight junctions and of epithelial barriers (By similarity). Required for normal hearing via its role
receptor, mediating natural killer cell cytotoxicity.

ility of cortical neurons.

ensing and homeostasis. May act as a negative regulator of plasma membrane calcium-transp
ype lectin-like domain (CTL/CTLD) superfamily. Members of this family share a common protei
nals, are transposases related to the transposase of the canonical piggyBac transposon from tl
ition.
ntrioles ensuring their 9-fold symmetry. Required for centrosome biogenesis and duplication: re

ivity and can function as transcriptional repressor (in vitro) (PubMed:21110952). May be a regu
ve site contains residues from both subunits.
onal activity of MYOD1, ensuring that expanding myoblast populations remain undifferentiated.
ition.
on. May play a role in the second step of the dolichol-linked oligosaccharide pathway. May ancl
rane domain in the vicinity of the mitotic spindle (PubMed:19494128). Involved in the organizati
BCR (BTB-CUL3-RBX1) E3 ubiquitin-protein ligase complex mediating the ubiquitination and
nown function that is expressed in a variety of tissues. There are pseudogenes for this gene on
function.
phate (NAADP) receptor that may function as one of the major voltage-gated Ca(2+) channels
rotubule-binding subcomplex of the outer kinetochore that is essential for proper chromosome
rotubule-binding subcomplex of the outer kinetochore that is essential for proper chromosome

by enhancing ERK and JNK signaling as well as up-regulating RUNX1 and FLI1 expression (F
euronal growth cones. Could potentially act as repulsive cues toward specific neuronal populatio

ition of ceramide from sphinganine and acyl-CoA substrates, with high selectivity toward palmit
phology and cytoskeletal organization. Required in the control of cell shape.
de GD1A from GM1B. Transfers CMP-NeuAc with an alpha-2,6-linkage to GalNAc residue on N
unctions, thereby regulating various cellular and signaling processes. Cooperates with PTEN to
ctions; the function is proposed to implicate a NPHP1-4-8 module (PubMed:19755384, PubMe
alpha- and beta-L-fucoses. L-Fucose (6-deoxy-L-galactose) exists as alpha-L-fucose (29.5%)
lgehog signaling. Required for centriole stability (By similarity). May play a role in counteracting
g endonuclease complex, a complex responsible for identification and cleavage of the splice s
ehavioral development.

at acts as a negative regulator of ciliogenesis by mediating recruitment of CCP110 to mother ce

n(5)S(2)U at tRNA wobble positions of tRNA(Lys), tRNA(Glu) and tRNA(Gln). May act by formi
ition.
unctions (HJs) by the introduction of symmetrically related cuts across the junction point, to pro
K1/NKG2D receptor, mediating natural killer cell cytotoxicity.||[Isoform 2]: Stimulates natural ki
the surface of endosomes, where it recruits and activates the Arp2/3 complex to induce actin p

t cells against natural killer cell-mediated lysis. Modulates signaling cascades and mediates tyr
ition.

rial carrier proteins (Haitina et al., 2006 [PubMed 16949250]).[supplied by OMIM, Mar 2008]

some distal) complex, a complex recruited to centromeres which is involved in assembly of kin
Pseudogene 2
biosynthesis pathway. Wybutosine is a hyper modified guanosine with a tricyclic base found at
to antagonize BECN1-mediated cellular autophagy at the endoplasmic reticulum. Participates
to 5-oxo-L-proline and a Cys-Gly dipeptide. Acts specifically on glutathione, but not on other ga
y of which are thought to be involved in early embryonic development. Homeobox genes encod
ich as SNORD87, are involved in 2-prime-methylation of preribosomal RNA precursors (Gogol

lutarate to alpha-ketoglutarate.

ition.

ntation. Acts as a stress-induced self-antigen that is recognized by gamma delta T-cells. Ligand

RNAs that are involved in post-transcriptional regulation of gene expression in multicellular org

RNAs that are involved in post-transcriptional regulation of gene expression in multicellular org
n adapter protein for nuclear receptor KPNB1. Binds specifically and directly to substrates cont
subsets of neurons in the brain (By similarity). Essential for long-term maintenance but not esta
ay a role in carcinogenesis of cancer cells. Mitotic phosphoprotein regulated by the ubiquitin-pr
ancestral retroposition. The encoded protein is related to a plant protein that plays a role in th
ation.
TB-CUL3-RBX1) E3 ubiquitin ligase complex that acts as a regulator of ion transport in the dista
ex that serves as a microtubule-dependent and Rho-mediated signaling required for the myosin
0610). Essential for the structural integrity of the cleavage furrow and for completion of cleavag
ay that controls convergent extension during gastrulation and neural tube closure. Convergent
sentation or the cellular interactions that follow lymphocyte activation.
oth ubiquitin ligase and deubiquitinase activities. Involved in immune and inflammatory respons
various processes such as cell growth, development, or differentiation. Mediates essential sig
e 3M complex and the histone deacetylases HDAC4 and HDAC5 (PubMed:25752541). May als
netochore-associated NDC80 complex, which is required for chromosome segregation and spin
protein that has a role in chromosome segregation during mitosis (By similarity). Translocates F
ctivity of lentiviruses, such as HIV-1: acts by inhibiting an early step of viral infection. Impairs th
rmal assembly of mitotic spindles. Required for normal assembly of microtubules during apopto
nosine-containing mRNA cap during an early step in the initiation of protein synthesis. May act a

nily (Teufel et al., 2005 [PubMed 15702247]).[supplied by OMIM, Mar 2008]
s the phosphorolysis of adenosine, guanosine and inosine nucleosides, yielding D-ribose 1-pho
A sequence 5'-GGGACTTTCC-3' which is found in the enhancer elements of numerous viral pr
and functionality of hematopoietic progenitor cells through the control of cell cycle progression a
PHM-27 is a potent agonist of the calcitonin receptor CALCR, with similar efficacy as calcitonin
osomes wrap and compact DNA into chromatin, limiting DNA accessibility to the cellular machin
microtubules with high affinity through each tubulin heterodimer and has an ATPase activity (By
ta and is involved in the control of eukaryotic DNA replication by increasing the polymerase's p
otion factor. The encoded protein may play roles in blood vessel maturation and hematopoeisis
ective transport of receptors and ligands across polarized epithelia.
ubMed:15917271, PubMed:16935860). May regulate ciliogenesis (PubMed:29420175).
RNAs that are involved in post-transcriptional regulation of gene expression in multicellular org

a receptor for thymic stromal lymphopoietin (TSLP).

n promoting cell proliferation and preventing apoptosis (PubMed:9859993, PubMed:21364656,
cal signals to specify territories inaccessible for growing axons.

ɪrties. Involved in regulating aneuploidy, cell cycling, and cell death in a p53/TP53-dependent m
at 'Lys-120' (H2BK120Ub). H2BK120Ub is a specific tag for epigenetic transcriptional activatic
ıtion.
ediated glutamate transport by increasing its affinity for glutamate in a PKC activity-dependent r

at the G2/M (mitosis) transition.
:er in mitotic control. Tyrosine protein phosphatase required for progression of the cell cycle. W
ctions as a dosage-dependent inducer of mitotic progression. Required for G2/M phases of the

ɪion.
ondrial apoptotic pathway by ensuring the accumulation of cardiolipin (CL) in mitochondrial me
'l pyrophosphate (FPP) moieties to form squalene. Proceeds in two distinct steps. In the first ha
inflammasome, plays a crucial role in innate immunity and inflammation. In response to pathog

ıtion.
:omplex that is required for normal biogenesis of lysosome-related organelles (LRO), such as p
ɔnate to mevalonate 5-phosphate, a key step in isoprenoid and cholesterol biosynthesis (PubM

ion channel.
ɔr of members of the IAP (inhibitor of apoptosis protein) family. Inhibits anti-caspase activity of
nks antiparrallel microtubules at an average distance of 35 nM. Essential for controlling the spa
ed receptors are coupled to the beta-catenin canonical signaling pathway, which leads to the a

RNAs that are involved in post-transcriptional regulation of gene expression in multicellular org
ıto smaller forms thereby controlling vWF-mediated platelet thrombus formation.

Nucleoskeleton and Cytoskeleton) complex involved in the connection between the nuclear lam
'olved in sterol biosynthesis. Catalyzes 14-alpha demethylation of lanosterol and 24,25-dihydro
gression and transcription during spermatogenesis. Inhibits degradation of polyubiquitinated cy

ediates the calcium- and neuron-selective induction of BDNF exon III transcription. Binds to the

g an inactive complex with this enzyme (PubMed:23621087, PubMed:23733961). Acts by prom

roteins, which are characterized by kelch repeat motifs and a POZ/BTB protein-binding domain
t transmits signals from cell surface receptors and plays an important role in the regulation of i
.) (PubMed:9070858, PubMed:19306925, PubMed:25025571, PubMed:26091040). Plays a rol
f cholesterol (PubMed:26941018). Plays a role in phosphatidylinositol-4-phophate (PI4P) turno

tion.
required for completion of cytokinesis (PubMed:11470801, PubMed:12740395). Required for p

EF) for the small GTPase RALA. May be involved in cytoskeletal organization (By similarity). Gu
f squalene to (S)-2,3-epoxysqualene, and is considered to be a rate-limiting enzyme in steroid I

hrough anaphase. Acts by mediating the recruitment of phopsphatase PP1-gamma subunit (Pl
on and reorganization of the actin cytoskeleton. Regulates cell spreading and cell polarization. :
ation and subsequent inhibition of cadherin degradation and reduced cell-cell adhesion.
ates the turnover of microtubules at the kinetochore and functions in chromosome segregation
cts as a specific chaperone for CENPA and is required for the incorporation of newly synthesiz
ST2 by protecting it from proteasomal degradation.

ded protein may be part of an exocyst complex that plays a role in cell membrane dynamics. M

3,4-trisphosphate (PubMed:24070612, PubMed:24591580). Plays a role in the late stages of m
bsence of leucine, binds the GATOR subcomplex GATOR2 and prevents TORC1 signaling. Bi
l function (PubMed:29146869, PubMed:31270470, PubMed:31235910, PubMed:31171830, Pu
ytoskeleton remodeling in response to extracellular stimuli, cell motility and adhesion and recep
double-stranded DNA (dsDNA) and double-stranded DNA:RNA hybrids. dsRNA is phosphoryl
ay a role in the uterus during late pregnancy and/or in trypsin activation in pancreatic acinar cel
tribute to tumorigenesis through: the delivery of survival signaling through phosphorylation of B
R signaling complex in excitatory synapses, it may play a role in NMDAR-dependent control of
rentiation of neuronal cells during the formation of neurite extensions. Involved in NMDA recep
dogenic tissues, steroid 11-beta hydroxylation in the adrenal cortex, 25-OH-vitamin D3-24 hydr
s differs between isoforms created by alternative splicing. Isoform 1 functions as permease tha
nt regulate trafficking of effectors involved in exocytosis (PubMed:25425525). In cytotoxic T-cel

bsence of leucine, binds the GATOR subcomplex GATOR2 and prevents TORC1 signaling (Pu

ganisms by affecting both the stability and translation of mRNAs. miRNAs are transcribed by R

mRNA stability and translation. The mouse ortholog of this gene is required for female fertility.
is mediated via G-proteins that activate a phosphatidylinositol-calcium second messenger syst
esis.
lolecular chaperone implicated in a wide variety of cellular processes, including protection of th
ganisms by affecting both the stability and translation of mRNAs. miRNAs are transcribed by R
dition to activating immunoglobulin gene expression. Modulates transcription transactivation by

ıyelin wraps and ultimately drives myelin compaction.
er mitosis. Overexpression delays G2/M phase progression.

ıge response and translesion DNA synthesis. The DCX(DTL) complex, also named CRL4(CDT

ells.

ganize the lamina/pathway-specific differentiation of dendrites. Plays an important role for audi

ıvisions (By similarity). Negatively regulates the mitotic apical cortex localization of GPSM2 (Pu

ton extruding system driven by the inward sodium ion chemical gradient (PubMed:26358773). I

)92). Together with CCDC120, cooperate with subdistal appendage components ODF2, NIN ar
lolecular chaperone implicated in a wide variety of cellular processes, including protection of th
ısphoserine residues. Dephosphorylates CDK2 at 'Thr-160' in a cyclin-dependent manner.
ıt (consensus sequence 5'-UUUUUAU-3') within the mRNA 3'-UTR (PubMed:24990967). RNA

tumor suppressor protein and stimulates its tetramerization, which results in cell-cycle arrest aı
ay be one regulator of transcriptional events during hemopoietic development.
ıll proliferation, differentiation and apoptosis. Plays an essential role in the regulation of chondr
s of basket cells in the somatosensory cortex during embryogenesis. Involved in the positive re
ndent kinase activity, preventing phosphorylation of critical cyclin-dependent kinase substrates
DK2 bound to SPDYA (PubMed:28666995). Involved in G1 arrest. Potent inhibitor of cyclin E- a
cell death (AICD). May play a role in cognate interactions between T-cells and B-cells/macropha

ganisms by affecting both the stability and translation of mRNAs. miRNAs are transcribed by R

in. May act as a downstream effector of RHOQ/TC10 in the regulation of insulin-stimulated gluc
equired for progression of S phase. The complex CDC7-DBF4A selectively phosphorylates MC
B transcription factor. May be a component of the inflammasome, a protein complex which also
script variants have been observed for this gene. [provided by RefSeq, Jun 2012]
naintains an open filtration pathway between neighboring foot processes in the podocyte by ch

2 signaling (PubMed:28302677). Binds to integrin ITGAV:ITGB3 (PubMed:28302677). Plays a

poles including spindle orientation, astral microtubule density and poleward microtubule flux se
ntities on the anterior-posterior axis.
ntities on the anterior-posterior axis.
ntities on the anterior-posterior axis.
ntities on the anterior-posterior axis.
Regulates maturation and trafficking of the transmembrane metalloprotease ADAM10 (PubMe

imilarity).
r to initiate DNA replication. Additionally, plays a role in preventing DNA damage during replica

volved in seizure prevention. Mutations in this gene are associated with progressive myoclonic
iovascular, kidney and skin development (PubMed:11782474, PubMed:15299087, PubMed:15

Med:11756469, PubMed:14667408, PubMed:15702419, PubMed:15475964, PubMed:152826C

onjugation and spindle assembly checkpoint activation. Drives chromosome congression (align
l and NDEL1. Regulates recycling of the plasma membrane by acting as a link between recycli
ns. The beta and gamma chains are required for the GTPase activity, for replacement of GDP
ntities on the anterior-posterior axis.

to the plasma membrane (By similarity).
rest, survival and cell migration. Binds to 3 E-boxes of the E-cadherin/CDH1 gene promoter ar

1). This transcript binds lysine methyltransferase 2A and promotes histone modifications that a
nscription factors (PubMed:10806483). Controls the activity of the transcriptional regulators GL

tion. Binds the NGFI-B response element (NBRE) 5'-AAAAGGTCA-3' (By similarity). May inhib
rylation of a number of proteins, including PTK2/FAK1 occurs. May also be involved in cellular

a significant role in mediating the biological activity of the HIV-1 Tat protein in vivo. Binds speci

by biliverdin reductase. Under physiological conditions, the activity of heme oxygenase is high

CR) into mevalonate, a precursor for cholesterol synthesis.
ganisms by affecting both the stability and translation of mRNAs. miRNAs are transcribed by R
gulate somite formation in the presomitic mesoderm (PSM). May function as a segmentation cl
1 to methionine.

eems to be mediated by binding to TRAF3 and TRAF6.

(mGluRs) at synapses.
nts. May play a role in angiogenesis by regulating FGFR1, VEGFR2 and PDGFR signaling. Ma
Hedgehog signaling pathway (By similarity). Represses the Hedgehog-dependent expression o
nscription by interacting with the corepressor Sin3A and HDAC1. Activates RANTES expressio
elium through interaction with SELPLG/PSGL1. May have a role in capillary morphogenesis.

nding and phosphorylating proteins are that already phosphorylated on a specific motif recogniz
role in embryonic cardiovascular development and lymphangiogenesis. Activates transcription
ndle checkpoint activity (PubMed:12438418, PubMed:14654001, PubMed:15062103, PubMed:
ganisms by affecting both the stability and translation of mRNAs. miRNAs are transcribed by R

ediates the ubiquitination and subsequent proteasomal degradation of target proteins. Recogni

sting its synthesis, folding or export from the endoplasmic reticulum, in a heat shock protein-de
of DLX5 on ALPL expression during osteoblast differentiation. Probable morphogenetic role. M
the regulation of cell proliferation. May act as a tumor suppressor. Inhibits tumor cell growth.

tandem of 2 BTB (broad complex, tramtrack, and bric-a-brac) domains, and a conserved C-ter
sses such as cell fate determination, cytoskeletal organization, repression of gene transcription
actor E2F1 at a subset of promoters to recruit KAT2A and histone acetyltransferase complexes
ulate in vascular tissue in aging and at an accelerated rate in diabetes. Acts as a mediator of bo
he regulation of multiple cellular processes linked to cytoskeletal proteins, such as cell adhesio
involved in cell-fate decisions during hematopoiesis (PubMed:9462510). Seems to be involved
e the presence of the serine protease domain, the encoded protein may not have any proteolyti
r functions. Also acts on D-erythro-sphingosine and to a lesser extent sphinganine, but not oth
romoters such as those of SV40, CMV, or HIV1. In addition, related sequences are found in the
uctose 6-phosphate, respectively) (PubMed:23185017, PubMed:26985301, PubMed:2929888
n mediates the ubiquitination and subsequent proteasomal degradation of target proteins (Publ
ns as a repressor of TGF-beta signaling.

320, PubMed:7961644, PubMed:23275542, PubMed:30373764). Activates the expression of m
n with membranes. Rabs cycle between an inactive GDP-bound form and an active GTP-bound

cytoskeleton. Plays a role in the regulation of cell morphology and cytoskeletal organization. Re
rans retinal, and has much lower in vitro activity with acetaldehyde (PubMed:27759097). Requi
sic motif of members of the AGC Ser/Thr protein kinase family; specifically acts on 'Ser-473' of

and-stimulated tyrosine phosphorylation and activation of the ERBB receptors. The multiple iso
ay control the differentiation, survival and proliferation of lymphoid cells. May also regulate ang
osteroids, biogenic amines, neurotransmitters, and lipid peroxidation (Probable). Oxidizes med
ent of the complex transcription machinery that governs circadian rhythmicity and forms a critic
ription in conjunction with CTNNB1 (PubMed:20028982). Establishes anterior identity at two le
n fold and have diverse functions, such as cell adhesion, cell-cell signalling, glycoprotein turno
n mediates the ubiquitination and subsequent proteasomal degradation of target proteins (Pub
cation stress or natural replication barriers such as the telomere duplex. The CST complex bin

Required for progression of S and M phases. The complex CDC7-DBF4B selectively phosphory
cell-cell interaction with AMIGO1 or AMIGO3. May contribute to signal transduction through its
nal rate of chromosomal segregation during anaphase. Plays a role in the regulation of mitotic s

ls of the TCRz/CD3 complex, as well as ZAP70, VAV, VCP and other key signaling molecules
the anaphase-promoting complex/cyclosome (APC/C) ubiquitin ligase. Probably recognizes an
NA repair in cells arrested at G1 or G2. Contains an iron-tyrosyl free radical center required for
leton. Induces the Rac-dependent neuritic process formation in part by disruption of the cortica

like kinases act by binding and phosphorylating proteins are that already phosphorylated on a
ral and miRNA-mediated mRNA turnover. PAN specifically shortens poly(A) tails of RNA and th
ed:11090199). Inhibits HIV-1 entry in cells coexpressing CD4 and APLNR (PubMed:11090199)
works such as the circulatory system and lungs.
axon. Required for the uniform orientation and maintenance of the parallel microtubule fascicle

nd also as a terminal sequence on certain gangliosides. SIAT4A and SIAT4B sialylate the sam
monoubiquitination of the FANCI-FANCD2 complex in response to DNA damage, cellular resist
transcriptional repression of the affected target gene. Able to mono-, di- and trimethylate 'Lys-

eric double-stranded 5'-TTAGGG-3' repeat (PubMed:28500257, PubMed:28082411). Preferen
nd degradation of WEE1 kinase at G2/M phase (By similarity).
nce 5'-CAC[GA]TG-3'. Antagonizes MYC transcriptional activity by competing for MAX and sup
T1/VEGFR-1. Isoform PlGF-2 binds NRP1/neuropilin-1 and NRP2/neuropilin-2 in a heparin-de
cytosis. Alternatively, may act as a negative regulator of the amino acid-induced TOR signaling

nesis. Binds to ss-DNA.
an restore ornithine transport in cells lacking the primary mitochondrial ornithine transporter SL

pon ligand binding. Axon repulsion in growth cones may be caused by its association with DCC

cer/promoter of hypoxia-inducible target genes and hence inhibits HRE-driven transcriptional ac
uterine epithelial cells at the time of the embryo implantation.

st activity with lysophosphatidic acid containing myristate (C14:0), monounsaturated oleate (C

lthough many alternative splice variants have been observed, the gene is thought to have no p
velopment (By similarity). Required for normal sialylation in hematopoietic cells. Sialylation is in

ciently unwinds G-quadruplex (G4) DNA structures and forked RNA-DNA hybrids. Resolves G4

LHL17) mediates the ubiquitination and subsequenct degradation of GLUR6. May play a role i
s the dehydration of L-fuconate to 2-keto-3-deoxy-L-fuconate by the abstraction of the 2-proto
1752334 (poliovirus receptor related immunoglobulin domain containing pseudogene) and PIL

etochore proteins, mitotic progression and chromosome segregation. May be involved in incor
herens junction function in epithelial cells and has a role in epithelial cell migration. It is a lung

homeostasis and triglyceride synthesis. Together with the oxysterol receptors NR1H3/LXR-alp

ɪn. It is released from several cell types in response to an inflammatory stimulus. IL-8(6-77) has
oles in cell signalling. Alternative splicing results in multiple transcript variants, but the full-length

ting AKT1 translocation to the cellular membrane and activation. Contributes to p53/TP53-depe
and p73/TP73-mediated cell cycle arrest and apoptosis by binding its transcriptional activation

ly share several distinct protein modules, including a propeptide region, a metalloproteinase do
. Also involved in the activation of numerous growth factor- and stress-induced genes. Mediate
ce a rapid and transient rise in the level of intracellular calcium ions and chemotaxis. SDF-1-be
etion and muscarinic receptor activation.

ous 3' poly(A) tail leading to mRNAs stabilization by protecting mRNAs from active deadenylati
homolog A) and SULT1A3 (sulfotransferase family, cytosolic, 1A, phenol-preferring, member 3
cell activation and T-cell proliferation. Important for interactions between activated T-lymphocy
Controls cardiac morphogenesis and myogenesis, and is also involved in vascular developmen
naperone genes during the heat shock response in a HSF1-dependent manner (PubMed:2535
omatin formation. Can both act as an activator or a repressor depending on the context. Media

phate and phosphatidylinositol 3,4,5-trisphosphate and increases their levels in the plasma me
activation.
ogenic kinase signaling and cell proliferation, as well as cell invasion and metastasis. Acts as a

ng to cell shape changes. Induces pseudopodia formation, when overexpressed in fibroblasts.

ganisms by affecting both the stability and translation of mRNAs. miRNAs are transcribed by R
proteins via an isopeptide bond either as a monomer (monoubiquitin), a polymer linked via diff

DNA replication initiation and elongation in eukaryotic cells. The active ATPase sites in the MC
tivated by hydrolyzing GTP to GDP. This general mechanism enables G proteins to perform a

cytosolic heme to the secretory pathway (PubMed:24836561).
as S.aureus and M.tuberculosis. Plays a role in the fusion of phagosomes with lysosomes.

signaling complex (DISC) performs caspase-8 proteolytic activation which initiates the subsequ
esses several PTS1-containing proteins. Catalyzes the processing of PTS1-proteins involved i
in bladder carcinogenesis by repressing the transcription of the A20 gene, leading to transport
transfers the ubiquitin to targeted substrates. Essential in early embryonic development to prev
ic set of effector proteins including key proteins involved in cilia development and function and

low MW diffuse from one cell to a neighboring cell.

dium (and water, which follows osmotically) through the apical membrane of epithelial cells. Co
lecule implicated in long-term T-cell immunity.
scle gene expression.

of centrosome maturation and spindle assembly, the removal of cohesins from chromosome a
nitosis. The CPC complex has essential functions at the centromere in ensuring correct chrom
bMed:10358076, PubMed:12228231, PubMed:15220471, PubMed:15890677, PubMed:18356

n factors by forming heterodimers and inhibiting their DNA binding and transcriptional activity.
possibly as a heterodimer with other bHLH proteins (PubMed:24236640). Regulates endothelia

e 7-methyl guanine cap structure from mRNA molecules, yielding a 5'-phosphorylated mRNA f
the GTP-bound forms of RHO and RAC1. It probably binds p21 with a tighter specificity in viv
n factors by forming heterodimers and inhibiting their DNA binding and transcriptional activity.
y.
ntral nervous system development (By similarity).
ected behavior, such as food intake, and general arousal. May also have a role in spermatocyt

he mitochondria. Contributes to p53/TP53-dependent apoptosis after radiation exposure. Prom

ment by enhancing AURKB activity (via direct CDCA8 phosphorylation) at the centromere, and
rate reductase and saccharopine dehydrogenase activity, respectively.

growth factor signaling in transformed cells. Plays a negative role in tumorigenesis as deletion
viral replication. Enhances MAVS-mediated host antiviral responses by serving as an adapter b

(FGF)-induced retinal lens fiber differentiation, probably by inhibiting FGF-mediated phosphoryl

gether with KDM8 (PubMed:24981860). Possibly together with KDM8, is involved in proper mitc
been removed. The receptor recognizes terminal galactose and N-acetylgalactosamine units.

d in recruitment of PLK1 to the central spindle. Interacts with guanosine triphosphate (GTP)-bo

topoiesis, stem cell maintenance, gametogenesis, mast cell development, migration and functi

s in DNA mismatch repair (MMR) to excise mismatch-containing DNA tracts directed by strand

ring later stages of palate development for growth and medial fusion of the palatal shelves. Pro
PDGF signaling cascades.
th TOPBP1 by participating in CDK2-mediated loading of CDC45L onto replication origins. Req
53-mediated apoptosis by regulation of TP53 expression and translocation to the nucleus and r
ellular promoters. Represses transcription from promoters with ATF sites. It may repress transc

esis; negatively regulates endothelial cell proliferation and migration and angiogenic sprouting (
olytic and antidepressant drugs and other psychoactive substances. Ligand binding causes a c
45 (Gm1145) in the 16S mitochondrial large subunit ribosomal RNA (mtLSU rRNA), a universa
ganisms by affecting both the stability and translation of mRNAs. miRNAs are transcribed by R
. Acts as a positive regulator of autophagy. In response to cellular stress or activation of autoph
not to activate RAB3A. Involved in cAMP-dependent, PKA-independent exocytosis through inte

uses a reading frame that is different from that of the chromosome 11 open reading frame 98
linked polyubiquitination. Acts as an essential factor of the anaphase promoting complex/cyclos

f is masked by autoinhibitory interactions with the protein kinase domain (PubMed:26455797).
s after prophase, when most of cohesin complex dissociates from chromosomes arms. May ac
gatively regulates calcium channels activity at the plasma membrane and indirectly inhibits calc
tage-dependent L-type calcium channel subunit alpha-1C trafficking to the cell membrane (By s
henylphosphate. Phosphorylates phosphatidylinositol 3,4,5-trisphosphate (PIP3).

362226). Associates with the surface of the mitotic chromosome, the perichromosomal layer, a
n, and function of 2 related white cell populations of the blood, the granulocytes and the monoc
usion in the biogenesis or maintenance of mature secretory vesicles. Regulates neurotrophin re
ary vessels, the ureter and the vertebral column. Required for embryonic development of the si

67759). Promotes the metaphase-to-anaphase transition and is required for chromosome align

owth and proliferation. Transduces extracellular signals to cytosolic and nuclear effectors. Pho

(By similarity). Exhibits weaker repressor activity compared to isoform 1 (PubMed:11779185). I
repair and protein ubiquitination, as part of the UV-DDB complex and DCX (DDB1-CUL4-X-bo

e 9-cis-retinoic acid-dependent RXR alpha transcription activation of the retinoic acid responsi
ing effects to IGFBP2 on cell invasion.
action with some CDK (cell division protein kinase) complexes; stimulates DNA excision repair i

milarity).
cuous receptor for the TGF-beta cytokines TGFB1, TGFB2 and TGFB3. Transduces the TGFB
translocation, t(X;18)(p11.2;q11.2), in which the 5-prime end of the SS18 gene (MIM 600192)

7me2, respectively) of histone H3 and monomethylated histone H4 'Lys-20' residue (H4K20Me
larity).
vents in the early B-cell development.
sential for cartilage morphogenesis and joint formation. Inhibits chondrocyte differentiation thro
lemethylates mono- and dimethylated H3 'Lys-9' residue, with a preference for dimethylated re
ell survival and proliferation, hematopoiesis, stem cell maintenance, gametogenesis, mast cell c
or this gene. [provided by RefSeq, Jan 2013]

ress, thereby preventing translation of downstream stress effector DDIT3/CHOP.||Multifunction

transfers the ubiquitin to targeted substrates. Inhibits TGF-beta signaling by triggering SMAD2

e balance between these two signaling lipids. Activated in the nucleus in response to alpha-thrc
a regulator of the Toll-like receptor TLR2 and TLR4 signaling pathways (PubMed:26599367, Pu

emoves damaged bases. Has a preference for oxidized pyrimidines, such as thymine glycol, fc

ric IFs with desmin and/or vimentin, and via its interaction with cytoskeletal proteins alpha-dyst
May form part of a tissue-specific acute response to remodeling stimuli. Known to act on MMP-
differentiation, and angiogenesis. Amplification of this gene has been found in several lymphom

nd ATF1 and for the regulation of the transcription factors RELA, STAT3 and ETV1/ER81, and
itosomal dominant form of hereditary fibrosing poikiloderma (HFP). Affected individuals display

ganisms by affecting both the stability and translation of mRNAs. miRNAs are transcribed by R
nizes and binds hemimethylated DNA at replication forks via its YDG domain and recruits DNM
low MW diffuse from one cell to a neighboring cell.
pha subunits, thereby driving them into their inactive GDP-bound form (PubMed:11063746, Pu
ey role in myogenesis by directly repressing the expression of ID2 and ID3, 2 inhibitors of skele

ganisms by affecting both the stability and translation of mRNAs. miRNAs are transcribed by R
development. Predominant protease, which in the development, influences dorsal-ventral patt

410). As a ligand for the inhibitory receptor PDCD1/PD-1, modulates the activation threshold o
, motility, extracellular matrix production and immunosuppression. In the canonical TGF-beta p
ganisms by affecting both the stability and translation of mRNAs. miRNAs are transcribed by R
hase during spermiogenesis, such as genes required for cilium assembly and function (By simil
naling. This induces morphological changes and detachment through cytoskeletal reorganizatic
hway, as well as in extracellular matrix proteins. Alternatively spliced transcript variants encodir
all-trans or 9-cis retinoic acid, and regulate gene expression in various biological processes. Th
es. Necessary but not sufficient for p53/TP53-mediated caspase activation and apoptosis. Has

3401856). In muscles and under catabolic conditions, dephosphorylates IRS1 leading to its de

nembrane.

noic acid receptors and regulates gene transcription. Mechanistically, uses molecular oxygen ir

um channels are characterized by a greater tendency to allow potassium to flow into the cell rat
as coinhibitory receptor in immune response, insulin action and functions also as an activator
7281216). The physiological relevance of this observation is unknown (Probable).
complementary single-stranded DNA and by stimulation of the RAD51 recombinase.
sport KIF2C and/or MAPRE1 along microtubules.
ns. The beta and gamma chains are required for the GTPase activity, for replacement of GDP
28575658). Binds to single and double-stranded DNA and exhibits DNA-dependent ATPase ac
hird closely related pseudogene locus in this region. Alternative splicing results in multiple trans
nd degradation of proteins involved in flagellar development and motility.
erous signaling cascades. The alpha chain contains the guanine nucleotide binding site and al
tivity.

ganisms by affecting both the stability and translation of mRNAs. miRNAs are transcribed by R

f its own gene and can activate its own transcription. Regulates the expression of key transcrip
r cell type. May enhance the WNT-CTNNB1 pathway by relieving antagonistic activity of CBY1

/matrix attachment region beta (S/MARbeta), an ATC-rich DNA sequence located upstream of

r the interleukin (IL)-2 receptor (PubMed:11909529) as well as interleukin (IL)-10 receptor (Pub
bly of BBSome, a complex involved in ciliogenesis regulating transports vesicles to the cilia (Pu
e).
deacetylation activity toward RELA/NF-kappa-B p65, thereby potentiates its transcriptional acti
strand breaks. Essential during mitosis and meiosis for proper segregation of daughter chromo
952). Promotes activity of NR5A1 when phosphorylated by HIPK3 leading to increased steroid
n of the NF-kappa-B p50 subunit. In the nucleus, acts as transcriptional activator that promotes
A[CG]TCA-3'.

atent state during storage in extracellular space (PubMed:19750484, PubMed:19651619, PubM
Ins(1,3,4,5)P4. The activity of this encoded protein is responsible for regulating the levels of a

s (PubMed:1918072). Integrin alpha-V:beta-6 (ITGAV:ITGB6) mediates R-G-D-dependent rele
l keratinocytes. May play an immune role by eliminating harmful DNA released into the extrace
janisms by affecting both the stability and translation of mRNAs. miRNAs are transcribed by R
278, PubMed:2277032). Expressed by non-hematopoietic tissues and preferentially cleaves pr
essive state of many genes, including Hox genes, throughout development. PcG PRC1-like co

cts as repressor in the absence of CTNNB1, and as activator in its presence. Activates transcri
rDNA transcription. The sumoylated form modulates the stability of the NoRC complex compon
l. Acts by enhancing RELA transcriptional activity (By similarity).
all chromosomes are properly attached to the mitotic spindle. One of its checkpoint functions is
g and for correct chromosome alignment. Has a key role in the assembly of checkpoint protein

nces that define origins of replication have not been identified yet. ORC is required to assemble
modifier 1) from its bound conjugated complexes which also makes it into an active form. Beca
stem. PILRB is thought to act as a cellular signaling activating receptor that associates with ITA
s a crucial role in protecting REC8 at centromeres from cleavage by separase. During meiosis

etochore proteins, mitotic progression and chromosome segregation. May be involved in incor
t. DNA methylation is coordinated with methylation of histones. May preferentially methylates n
ons and slowing poleward movement during anaphase, thus suppressing chromosome movem
ulation of myelopoiesis through transcriptional activation of several genes in the HOXA cluster,
concert with PTHLH/PTHRP to stimulate ductal outgrowth during embryonic mammary develo

tivate JNK. Following prolonged stress, anti-apoptotic effect stops because of degradation of R

-EIF2A- ATF4 pathway. BMP2 activation of EIF2AK3 stimulates phosphorylation of EIF2A whic
nding proteins such as BCL6 and MLLT3. This repression may be mediated at least in part by h
opriate epidermal development (By similarity). Plays a role in regulating mammary epithelial cell

as a phospholipase than a triglyceride lipase (PubMed:12032167). Hydrolyzes triglycerides, bo
effector of Notch signaling required for cardiovascular development. Specifically required for t

actions. That protein has no CMP-N-acetylneuraminate monooxygenase activity and is not abl
a-oxydation and energy production but could also play a role in the metabolism of specific fatty
granules, nuclear speckles and pericentriolar material (PubMed:29973724). Dual-specificity tyr
Acts by forming a complex with phosphorylated PHF2, which mediates demethylation at Lys-33

ulation of IFN and IFN-inducible genes, host response to viral and bacterial infections, regulatic
ger systems. Not activated by UTP or UDP.

proteins of the JUN family, thereby forming the transcription factor complex AP-1. As such, the
circadian expression in the heart (By similarity). Is a repressor of CCN2 expression, involved i
nscripts at DNA damage sites by binding to the RNA polymerase II subunit POLR2A and block
eavage of the splice sites in pre-tRNA. It cleaves pre-tRNA at the 5'- and 3'-splice sites to relea
or a heterodimer with CASTOR2, binds and inhibits the GATOR subcomplex GATOR2 and the

the hydrolysis of ATP (PubMed:24097981). Transports preferentially phosphatidylserine over p
onal and structural phenotypes in adult heart.
y be involved in the pseudouridylation of 18S ribosomal RNA. This RNA is found associated wi
protection against the activity of mobile genetic elements (By similarity). piRNA-mediated trans
, such as that of CDK5 by activated ABL1, which leads to increased CDK5 activity and is critica
neries which require DNA as a template. Histones thereby play a central role in transcription reg

gulatory element-binding proteins (SREBPs). Capable of retaining the SCAP-SREBF2 comple
cell divisions.
by WNT4, WNT5A, WNT5B, WNT6, WNT7A or WNT7B (PubMed:10557084). Contradictory re
preferentially to the canonical E box sequence 5'-CACGTG-3'. Represses transcription by the
) to leukotriene D4 (LTD4).
egation of receptor-ligand complexes into ribosome-sized signalosomes. The beta-catenin can
ng the apical accumulation of F-actin and myosin II, and probably by bundling stress fibers (By

(PubMed:27889062). As part of the BCR(KLHL24) E3 ubiquitin ligase complex, mediates ubic
iron efflux in the presence of a ferroxidase (hephaestin and/or ceruloplasmin).

ganisms by affecting both the stability and translation of mRNAs. miRNAs are transcribed by R

; lumen of the endoplasmic reticulum. The SRP consists of 6 polypeptides (e.g., SRP19; MIM 1

'ORC1 signaling cascade controlling the MiT/TFE factors TFEB and TFE3 (PubMed:18663353
ed in protein-protein and protein-DNA interactions. Multiple alternatively spliced transcript varia
essential functions at the centromere in ensuring correct chromosome alignment and segregati
sion by restricting proliferation and promoting apoptosis. The core of this pathway is composed
version of uridine to pseudouridine at specific residues of ribosomal RNAs or small nuclear RN
ter and then directly transfers the ubiquitin to targeted substrates, such as UCKL1 (PubMed:16
odify pain sensitivity and persistence. Isoform GCH-1 is the functional enzyme, the potential fu

actin. Expression is induced by CD95 or TNF-alpha, via NF-kappa-B. Protects cells from CD95
neries which require DNA as a template. Histones thereby play a central role in transcription reg

ected genes. AP-2 factors bind to the consensus sequence 5'-GCCNNNGGC-3' and activate g

e activation and recruitment of CDC42 and the reorganization of the actin cytoskeleton at the c

ISTN. Inhibits activin A-, activin B-, BMP2- and MSDT-induced cellular signaling; more effective
AG-3'.

phosphate (PtdIns(3P)).
'LXNA2 (PubMed:19666519). Involved in gene regulation specifically in the gastric epithelium (

ar functions through its interactions with other proteins. This gene shares a high degree of seq

f the N-acetylgalactosamine (GalNAc) residue of chondroitin. Chondroitin sulfate constitutes th
maturation resulting in increased platelet production (PubMed:2145578). Also promotes the pro

te). Has a protease-independent function in promoting the transport from the endoplasmic retic
e anchorage-independent cell proliferation in soft agar.

AD1 and SMAD5, 2 receptor-regulated SMADs specific for the BMP pathway. Promotes ubiquit

ng as a 'reader' of a network of post-translational modifications. PcG proteins maintain the trans
ne formation of a heterodimer with transcription factors of the GATA family GATA1, GATA2 an
nanner. Part of the U8 snoRNP complex that is required for the accumulation of mature 5.8S ar
n contractile ring formation during the cell cycle cytokinesis. Essential for cytokinesis in Rho-me

eceptor EDA2R.||Cytokine which is involved in epithelial-mesenchymal signaling during morpho
ble-stranded DNA (dsDNA). In vitro, displays strong glycosylase activity towards the hydantoin

tivation kinetics and rate of recovery from inactivation in a calcium-dependent and isoform-spe
Rho signal to other molecules.
metaphase plate. Required for the execution of the mitotic checkpoint which monitors the proc

spective levels of these two bioactive lipids (PubMed:15544348, PubMed:19744926, PubMed:
trosome and the spindle microtubules during mitosis and plays a critical role in various mitotic

L1, preventing NF-kappa-B activation, nuclear transport and NF-kappa-B-mediated expression

-beta (Transforming growth factor) and activin signaling by associating with their receptors thus
ation of AR activity is dependent upon sumoylation (PubMed:14609956, PubMed:26522984). A
AD (R-SMAD).

cyclase.
contains genomic sequence in its 3' UTR which is not supported by experimental evidence. C
e. Involved in the processing of free oligosaccharides in the cytosol.
ity.
to DNA mismatches thereby initiating DNA repair. When bound, MutS alpha bends the DNA he
onate, with a particular role in the inflammatory response (PubMed:7947975, PubMed:759259

s an oncogene. [provided by RefSeq, Dec 2017]

following DNA damage, digests double-stranded DNA to form single-stranded DNA and amplif
chinery triggered in response to double-strand breaks in DNA (PubMed:25642963, PubMed:25

atum corneum (SC) epithelial cell formation (By similarity).
tus structure and function. Also required for CTNNA1 recruitment to adherens junctions.

PubMed:28846097, PubMed:28846098, PubMed:28846099, PubMed:23468844, PubMed:290
promoter region of a number of genes whose products are involved in cell cycle regulation or
rgeting endocytosed EGFR and ERGG3 for lysosomal degradation, and thereby helps downre
(in cells) (PubMed:24218572). Involved in the regulation of actin cytoskeleton organization thr

nay be involved in its turnover (By similarity). Has angiogenic inhibitor activity. Active metallopr

ganisms by affecting both the stability and translation of mRNAs. miRNAs are transcribed by R

ID is a multimeric protein complex that plays a central role in mediating promoter responses to

erative pain. Necessary for the normal dephosphorylation of the long-lasting phosphorylated fo
esses, such as cell proliferation, immune response, endosomal trafficking and cell metabolism

adation of FBXW7, may act indirectly on the expression and downstream signaling of MTOR,

ination and subsequent proteasomal degradation of target proteins. Probably recognizes and b
NA damage response. Mainly acts as a transcription repressor that binds DNA independently
ganisms by affecting both the stability and translation of mRNAs. miRNAs are transcribed by R
negative regulator of glycolysis by lowering intracellular levels of fructose-2,6-bisphosphate in a

IA deadenylation in a CNOT6 and CNOT7-dependent manner. In vitro can inhibit deadenylase
tamin D, vitamin D(2) and D(3) (calciol) (PubMed:12867411, PubMed:15465040, PubMed:185
rs modulate the transcription of their target genes through recognizing the core consensus binc
unction. Promotes smooth muscle cell proliferation. May be involved in macrophage-mediated
led:26842564). DNA replication licensing factor, required for pre-replication complex assembly
ppression by restricting proliferation and promoting apoptosis. The core of this pathway is com

ine in a beta-replacement reaction to form L-cystathionine, the precursor of L-cysteine. This ca
of BIRC5 and is required to maintain microtubule dynamics and genome integrity. Acts downs
ocampal neurons, in presynaptic terminals, plays an important role in the final steps of neurotra
lower degree, the beta-globin gene and represses promoters containing SP1-like binding inhib
re-assembly of dynein arm complexes in the cytoplasm before intraflagellar transport loads the

actors. Stimulates E2F1/TFDP1 transcriptional activity. Renders the activity of cyclin D1/CDK4
tachment of m-Tyr (an oxidized version of Phe) to tRNA(Phe), thereby opening the way for del

stimulation, it also binds to IRS-1.
s transcription factor NF2L2/NRF2 by decreasing the rate of protein synthesis and not via a ubi
bMed:21816348). Required for cortical dynein-dynactin complex recruitment during metaphase

h PARD3. Required for ciliogenesis.
the MAPK cascade to form a functional JNK signaling module (PubMed:12189133). May functi
cruitment of other corepressors and histone-modifying enzymes (PubMed:12559562, PubMed:
nal growth cone collapse and cell migration (By similarity).
on in normal and myeloid leukemia cells, the induction of neuronal cell differentiation, and the s
region of the myelin protein MPZ gene, and may thereby be involved in the differentiation of ol

osome. Thereby, plays an important role in the splicing of cellular pre-mRNAs. Most spliceosor
May function as a scaffolding protein that regulates epithelial cell polarity by connecting ARF6

ent of the complex transcription machinery that governs circadian rhythmicity and forms a critic
G2/M checkpoint. In the SOSS complex, acts as a sensor of single-stranded DNA that binds to

ted cell cycle arrest. Binds to and dephosphorylates 'Ser-15' of TP53 and 'Ser-345' of CHEK1 v
oform 1, does not phosphorylate and activate NEK11 in G1/S-arrested cells.||Protein kinase wl

ng a complex with MEI4 and REC114, which activates DSBs formation in unsynapsed regions,
obial infection) Acts as a receptor for Vesicular stomatitis virus.||(Microbial infection) In case of

horylation cascade.
yonic development and cell fate decision. Stimulates the expression of upstream key transcript
-O-alkyl-sn-glycero-3-phosphocholine (lyso-PAF), lysophosphatidylethanolamine (lyso-PE) and

ganisms by affecting both the stability and translation of mRNAs. miRNAs are transcribed by R
ns.
nsmitting growth control signals between focal adhesions at the cell periphery and the mitotic s

oter. Its binding to MB1 and function are inhibited by PURA (By similarity).
eaflet of various membranes and ensures the maintenance of asymmetric distribution of phosp
ome-mediated interkinetic nuclear migration (INM) of neural progenitors (By similarity). May pla
or IL17A-producing Vgamma2-positive gamma-delta T-cell maturation and development, via bi

Notch molecules. Modulates NOTCH1 activity by modifying O-fucose residues at specific EGI

gnaling at different levels. First, by competing with RAF1 protein for binding to activated Ras. S
of KCNMA1, thereby contributing to KCNMA1 channel diversity. Alters the functional propertie
control of cAMP-mediated neural activity and cAMP metabolism in the brain.
ercise or in adipose tissue following cold exposure and is present in the circulation. Able to stin
nd maintenance of differentiating epithelial tissues. Enhances cell proliferation, cell motility and

1463391). Functions by promoting partial unfolding of BCL2L1 and dissociation of BCL2L1 fron
poietic growth factor genes CSF2, IL3, IL8, and of the bovine lysozyme gene. Acts synergistica
he promoter region of many cytoprotective genes, such as phase 2 detoxifying enzymes, and p
so controls gene expression in embryonic cardiac cells. Could regulate not only the activation a
mitotic spindle and the orientation of the plane of cell division. Required for normal proliferatio
-CSF. Promotes invasive migration through the activation of GPC6 expression and WNT5A sig
neural tube closure. Alternative splicing results in multiple transcript variants. [provided by RefS

1, PubMed:15201869). Core component of the 7SK RNP complex: in cooperation with 7SK sn
of APP, via a MAP kinase signal transduction pathway composed of MAP2K7 and MAPK1/ER
and nuclear envelope. The proteins encoded by the members of this gene family are evolution
essential functions at the centromere in ensuring correct chromosome alignment and segregati

2 and poly-SUMO3 chains. Has very low efficiency in processing full-length SUMO proteins to t
and axon initial segments (PubMed:25950943). May also play a role in axon outgrowth and gu
ly regulates the internalization of ligand-activated EGFR by binding to the Ub moiety of ubiquiti
ocalization signals. On cellular stimulation by immune and proinflammatory responses, become

main and likely functions as an oxidoreductase. Multiple alternatively spliced variants, encodin
ination and subsequent proteasomal degradation of CP110 during G2 phase, thereby acting a

of HIV-2 gene expression. Binds specifically to two purine-rich motifs in the HIV-2 enhancer.

ɔ, suppresses hematopoietic progenitor cell proliferation. GRO-beta(5-73) shows a highly enha
ɪthelial cells in an autocrine fashion. In vitro, the processed form GRO-gamma(5-73) shows a fi
re-mRNA processing. snoRNAs form part of the small nucleolar ribonucleoprotein particles (snc
he development and maintenance of non-centrosomal microtubule bundles at the lateral memb
ɔr. Plays an important role in normal prostate development, regulating proliferation of glandular
promoter region of target genes (By similarity). Binds double-stranded target DNA, irrespective

cell nuclear antigen loader complexes, CTF18-replication factor C (CTF18-RFC), which consis

and stimulates its kinase activity. Also dephosphorylates CDK2 in complex with cyclin E, in vit

transfer in the nervous system. ARC protein is released from neurons in extracellular vesicles

unction as adhesion protein. Plays a role in the regulation of neurite-like outgrowth (By similarit
45, a key regulator of T- and B-lymphocyte activation. The interaction with PTPRC may be requ

hesion during neuronal, vascular and epithelial development. Binds promiscuously Eph recept
action (By similarity).
pillomavirus type 16 E7 mRNA (HPV16 E7).
tosis is initiated.

ipon the complex. Is regulated by MAD2L1: in metaphase the MAD2L1-CDC20-APC/C ternary

ntrol of the eukaryotic cell cycle by modulating the centrosome cycle as well as mitotic onset; p
complex at the onset of anaphase. During most of the cell cycle, it is inactivated by different m
ictures in the absence of high levels of RhoA activity.
Alternatively spliced transcript variants have been found for this gene. [provided by RefSeq, Jai
l centriolar homeostasis and preventing formation of ectopic microtubular organizing centers.
561620). May directly function at replication forks, explaining why Simian virus 40 (SV40) inter

ım.
·1, thereby regulating cell proliferation, migration and differentiation in angiogenesis or yet in tuı

66175, PubMed:19248771, PubMed:20484466, PubMed:23820903, PubMed:11435610, PubM
repressing estrogen-induced proliferation. May play a role in T-cell development and function.
ı with PDCD1 inhibits T-cell proliferation by blocking cell cycle progression and cytokine produc
inhibiting the functions of MYOD1 and ASH1. Binds DNA on N-box motifs: 5'-CACNAG-3' with

eonine. A genomic deletion affecting this gene has been associated with Ellis-van Creveld sync
nediates from straight-chain, 2-methyl-branched-chain fatty acids bile acid intermediates. With

ability due to contraction of terminal venules, and catecholamine release from adrenal medulla,
spreading and bone remodeling. Plays a role in the regulation of the humoral immune response
riphosphates and monophosphates. Active on AMP and dAMP with ATP as a donor. When GT
aldehyde, respectively. Can also convert delta-aminovalerate and beta-alanine.

5788062). Can also utilize propionate with a much lower affinity (By similarity). Provides acetyl-
efficiency for hydrolysis of cocaine, 4-methylumbelliferyl acetate, heroin and 6-monoacetylmor
phagy.
The BBSome complex is required for ciliogenesis but is dispensable for centriolar satellite funct
mer CSC subcomplex from the endosomal membrane to the cytosol; at least retromer displace
osomal membrane (LMP) and resulting in the release of CTSD and CTSL to the cytoplasm. Tri
KBH3, enabling ALKBH3 to process alkylated N3-methylcytosine (3mC) within double-stranded
ding interferon-mediated antiviral defenses (PubMed:16809771, PubMed:23230272, PubMed:
C1 and/or HDAC3. Seems to inhibit skeletal myogenesis and to be involved in heart developme
r instance in cell cycle control and signal transduction. Undergoes a functional cycle that is link
binds calcium in vitro. Isoform 2 and isoform 6 probably bind calcium. Isoform 3 and isoform 5
en cellular iron levels are high. Functions as mRNA binding protein that regulates uptake, sequ

ct cells against TRAIL mediated apoptosis by competing with TRAIL-R1 and R2 for binding to t
ton extruding system driven by the inward sodium ion chemical gradient. Plays an important rol
on of endocytic membranes (PubMed:24019528). Acts in early endocytic membrane fusion and
motaxis. Potent mitogen for cells of mesenchymal origin. Plays an important role in wound hea
led:24449906, PubMed:24553286, PubMed:16357261). Due to low processivity on both damag
cle. Functions as an inhibitor of hair elongation by promoting progression from anagen, the gro
irus 2/HHV-2.||Receptor for four distinct ligands: The TNF superfamily members TNFSF14/LIG

nd CRK. Was initially proposed to be required in complex with DOCK1 to activate Rac Rho sm
tosis during retinal development. Establishes dorsal-ventral positional information in the retina a
classic ligand-driven signal transduction cascades, resulting instead in chemokine sequestratic
s. Both complexes facilitate the perturbation of chromatin structure in an ATP-dependent mann
presentation (By similarity).

and synaptic signal transmission, and probably mediates its effects by recruiting and clustering
staglandins (PG) E1 and E2, thyroxine (T4), deltorphin II, BQ-123 and vasopressin, but not DPI
ensus sequence 5'-CGCG-3' but can also bind to E-box elements (5'-CACGTG-3'). Can also bi

d vesicular transport.
riptional repressor E4F1 and may function in cell growth. Inhibits the transcriptional activity of F
ping (PubMed:23836506). Binds to calmodulin.
tty acyl CoA as substrates. Required for synthesis and storage of intracellular triglycerides (Pub

s on proteins.

complex which is believed to play a role in SNARE assembly at the ER (PubMed:19369418).
In vitro, hydrolyzes all three D5-phosphorylated polyphosphoinositide substrates in the order Pt
yl-CoA into acetyl-CoA and acetoacetate, a key step in ketogenesis. Terminal step in leucine c
ta-2-adrenergic receptor binds epinephrine with an approximately 30-fold greater affinity than i
onine and a tyrosine residue in the MAP kinase p38. Part of a signaling cascade that begins wi
ears to play a critical role in protecting cells from stress-induced apoptosis, neuronal survival ar
nnate-type mucosal-associated invariant T (MAIT) cells (PubMed:23051753, PubMed:2679525
29374064). Through the degradation of CTNNB1, functions downstream of FOXF2 to negative
omplex, that catalyzes the attachment of the cognate amino acid to the corresponding tRNA in
ucleoside monophosphate and diphosphate kinase activities. Catalyzes the phosphorylation of
a non-exchangeable site on the alpha chain.
precursors are transported from the base of the cilium to the site of their incorporation at the ti
ependent and -independent mechanisms. After the penetration of retroviral nucleocapsids into

B and PLK1 to the spindle midzone.

ross cell membranes (Jacobsson et al., 2007 [PubMed 17714910]).[supplied by OMIM, Mar 20
arker (Liu et al., 2000 [PubMed 10857752]).[supplied by OMIM, Mar 2008]

ppressor. May be involved in development of head, face, limbs and ventral body wall. Involved

erve terminals along microtubules during hypoxia. Participates in the translocation of TRAK2/G
species containing a palmitoyl or stearoyl chain on the sn-1 and an unsaturated fatty acyl chai
ROS) which participate in a variety of biological processes including host defense, hormone bio
n vitro). Promotes activation of caspases and apoptosis.
r heregulin stimulation (PubMed:20010870).
n the activation of a cascade of antiviral responses including the induction of type I interferons a

biosynthesis pathway. Also catalyzes the reversible oxidation of 2-hydroxyglutarate to 2-oxoglu

liphatic aldehydes between 6 and 24 carbons in length (PubMed:9133646, PubMed:22633490
vated protein kinase (SAPK)/JNK.
be involved in the endoplasmic reticulum stress response (By similarity).
pathways activated by Toll-like receptors (TLRs) and the DNA sensor STING/TMEM173 in res
tensin receptor blocker, in liver and intestine. May also activate beta-lactam antibiotics faropen
r free GTP. Plays a role in endocytosis via its role in activating Rab family members (By similar

is phosphorylated. May promote FYN activation, possibly by disrupting intramolecular SH3-de
with CD81 and the CLDN1-CD81 receptor complex is essential for HCV entry into host cell (P
modification of biopolymers on the cell surface. Alternate splicing results in multiple transcript
or normal keratinocyte proliferation. Required for normal polarization of basal keratinocytes in s
l proliferation and migration and an increase in apoptosis. Inhibits melanoma growth When exp
es coexpression with SLC3A2/4F2hc to mediate the uptake of arginine, leucine and glutamine.
ress fibers formation, cell motility and survival, formation of tubules by endothelial cells, and reg
ne in the CNS. They have a role in both its formation and stabilization. The smaller isoforms mi
d, with preference for -Phe, -Leu, -Ile, -Met, -Tyr and -Val (PubMed:20385563).
to viral infection. Binds ssRNA, dsRNA and dsDNA and can promote the binding of DDX58/RIG

on, and fibroblast growth factor activity. IL-1 proteins are involved in the inflammatory response,
gnal transduction pathway. Could also play an important role in normal and pathological angiog
. The reaction takes place in the presence of glutamine and ATP through an activated gamma-
or threonine residue on the protein receptor. Although it displays a much weaker activity toward

ble). May play an important role in cortical development, especially in the maintenance of radial
ucible GPCR provide evidence for a possible interaction between retinoid and G-protein signalin
and phthalazine, as well as aldehydes, such as benzaldehyde, retinal, pyridoxal, and vanillin. Pl

ostamere localization of DMD and betaDAG1 (By similarity). Membrane-cytoskeleton linker. Ma
the cell cycle. Identification in a chromosomal region frequently deleted in various cancers sug
or threonine residue on the protein receptor. May participate in synthesis of oncofetal fibronecti
PER1 by directly binding to cAMP response elements in its promoter.||Transcriptional regulato
d in the TFII-I family of transcription factors, one CHARLIE8 transposable element-like sequenc
ne-triggered immune responses by capturing and internalizing CCL19 or by presenting RARRE

telomere integrity and meiosis. The complex possesses single-strand endonuclease activity an
ve phosphorylation from ATP synthesis. As a result, energy is dissipated in the form of heat.
regulator of cell migration, probably via increasing cell-cell adhesion (PubMed:21402705). Pro
The 120 kDa linear IgA disease antigen is an anchoring filament component involved in derma
motaxis. Potent mitogen for cells of mesenchymal origin. Required for normal lung alveolar sep
n and is responsible for transferring electrons from succinate to ubiquinone (coenzyme Q) (Pub
are cell-surface proteins that are characterized by the presence of four hydrophobic domains. T
ng of bilin. Appears to be able to transport a variety of ligands in a number of different contexts
amics. Mediates polymerization of globular actin to F-actin. Also binds to, stabilizes and bundle
ns in this gene, or deletion of a chromosomal segment containing this gene, are associated wit
x. Regulates exosome biogenesis in concert with SDCBP and PDCD6IP (PubMed:22660413).

ranulocytes.
arbohydrate moieties of glycoproteins.
on through APBB1 (Fe65) binding (By similarity). Couples to JIP signal transduction through C-t
uction of type I interferons and proinflammatory cytokines. Forms a ribonucleoprotein complex v
way in colorectal cancer cells. Inhibits DNA binding of b-catenin/TCF/LEF transcription factors.
acid beta oxidation, 4,8-dimethylnonanoyl-CoA, to its corresponding carnitine ester.
s include POMC, renin, enkephalin, dynorphin, somatostatin, insulin and AGRP.
A protein (PubMed:26405230). Such oncoproteins prevent the ability to sense cytosolic DNA (I
STAT3 activity.

nt of convergence for MAPK and PKA signaling in cilia.
ty and invasive activity, and promotes cancer metastasis. May be involved in the progression o
n are key regulators of many important physiological processes (PubMed:8557689, PubMed:29

420639). Acts as a glycerol transporter in skin and plays an important role in regulating SC (stra

dgehog target genes, including GLI1, PTCH1, and PTCH2 in PTCHD4-expressing cells.
resensitization processes. During homologous desensitization, beta-arrestins bind to the GPR

postsynaptic neurons in the hippocampus to control the assembly of a precise topographic pro

actors and interleukins.
7796300, PubMed:18851833, PubMed:25043379). In contrast to PARP1 and PARP2, it is not
its cardiac troponin-T (TNNT2) pre-mRNA exon inclusion but induces insulin receptor (IR) pre-
inflammatory properties, that can regulate T-helper cell development, suppress T-cell prolifera
NAs. Binds to a ZAP-responsive element (ZRE) present in the target viral mRNA, recruits cellu

response.||Plays an important role in the maintenance of the Golgi complex, in membrane traff

is proteinase is in part similar to the specificities of cathepsin L.
ates POU5F1-mediated transcriptional activation. Plays a general role in caspase independent
ents (RARE).
lling plasmin-mediated proteolysis, it plays an important role in tissue remodeling and degrada
edundant role with FRMD4B in epithelial polarization (By similarity). May regulate MAPT secret

DNA damage through interaction with poly(ADP-ribose), a polymeric post-translational modific
nitosis. May be involved in the regulation of perinuclear intravesicular membrane traffic (By simi
ulated by guanine nucleotide exchange factors (GEF) and GTPase-activating proteins (GAP).
amma-glutamyl peptides (PubMed:27913623). Glutathione depletion is an important factor for

glycan in heparan sulfate. Modifies the GlcNAc-GlcA disaccharide repeating sugar backbone t
onto a xylose (Xyl) acceptor to produce the glucuronyl-beta-1,4-xylose-beta disaccharide prime
pericellular collagenolysis and modeling of skeletal and extraskeletal connective tissues durin
dition of the first GalNAc to the core tetrasaccharide linker and for elongation of chondroitin ch
inositol 3,4,5-trisphosphate (PIP3). PIP3 plays a key role by recruiting PH domain-containing p
ference for long polyubiquitin chains. May play a regulatory role at the level of protein turnover.
L/RAMPs receptor complexes. Activates the cAMP-dependent pathway.||[Intermedin-short]: M
on-mediated antiviral responses (PubMed:12670957, PubMed:19818714, PubMed:26479788,
ved in assembly, disassembly and directioning of stress fibers in fibroblasts. Required for the lo
sses such as cell fate determination, cytoskeletal organization, repression of gene transcription
and methyl donor. Protects cells from oxidative stress by metabolizing a number of lipid perox
anylgeranylcysteine.
e-mediated STAT transcription. Enhances CBP/p300 coactivator protein recruitment to STAT1

alcium out of the cell (PubMed:8530416). By regulating sperm cell calcium homeostasis, may p
irculation by blocking LFA-1-dependent cell adhesion. It mediates adhesive interactions import
other mono-branched 3-methylacyl-CoA esters (with a chain length of at least seven carbon ato
listribution of TYRP1 in melanocytes; the function, which probably is implicating vesicle-traffick

tor group rhinovirus A-B capsid proteins.||(Microbial infection) Upon Kaposi's sarcoma-associat
ineralocorticoid response elements (MRE) and transactivates target genes. The effect of MC is
complexes, respectively (PubMed:15448697, PubMed:14739464, PubMed:16260596, PubMe
HTT by RAB5A onto early endosomes. The HTT-F8A1/F8A2/F8A3-RAB5A complex stimulates

cysteine directed by the UGA codon. SelB binds GTP and GDP.
LGALS3, TUB, TULP1 or GAS6. Regulates many physiological processes including cell survi
P-dependent release of EIF6 from 60S pre-ribosomes in the cytoplasm, thereby activating ribo

cks an intrinsic 3'-5' exonuclease activity and thus has no proofreading function.

velope.||(Microbial infection) Acts as a receptor for lassa virus and lymphocytic choriomeningitis
ch as inflammation, immunity, differentiation, cell growth, tumorigenesis and apoptosis. NF-kap

nduces also B-cell proliferation and differentiation into plasma cells. Could play an important rol
3098). Acts redudantly with ZYG11B to target substrates bearing N-terminal glycine degrons fo
53. Promotes ubiquitination and subsequent proteasomal degradation of FLT3.
dhesion, perhaps involving cytoskeletal reorganization. Generates a C-terminally amidated pep
3. It activates typical cytokine signal pathways of NF-kappa-B and p38 MAPK.

complex that includes TRAF6, MAP3K7, CHUK and IKBKB, thereby facilitating NF-kappa-B sig
air mechanism of both skeletal muscle and cardiomyocytes that permits rapid resealing of mem
P to form AA-AMP and then transferred to the acceptor end of the tRNA.
rgo proteins. Can bind and assemble clathrin, and binds simultaneously to phosphatidylinositol
o, cell survival, proliferation, migration and inflammation. Does not exhibit any protease activity
these two bioactive lipids (PubMed:2175712, PubMed:15544348). Thereby, acts as a central s
spatial organization. As a component of the LINC (LInker of Nucleoskeleton and Cytoskeleton
9330545). It is also activated by the concentration of cytosolic Mg(2+). Its activation dampens t
rates with TLR2 to mediate the innate immune response to bacterial lipoproteins or lipopeptides

y membrane hyperpolarization which promotes calcium influx. Required for maximal calcium in
recognition of molecular patterns specific to microorganisms. TLR3 is a nucleotide-sensing TLF
port from the endoplasmic reticulum to the Golgi stack. Seems to function as a fusion protein re
of triglyceride clearance from the blood serum and in lipid metabolism (PubMed:19270337, Pub
Gal-beta-1,3-GalNAc and Gal-beta-1,3 (GlcNAc-beta-1,6)GalNAc.
signaling pathways through stimulation of PLCE1. May play a role in cytoskeletal rearrangeme
rome brain. Alternate splicing results in multiple transcript variants. [provided by RefSeq, May 2
5, PubMed:9880569). Activated by detection of double stranded RNA (dsRNA): polymerizes hig
the p300/CBP transcriptional coactivator complex in order to stimulate TFAP2-mediated transc
also play a role in other cellular processes such as apoptosis, cell growth, differentiation and ge

and basal transcription factors like TBP and TFIIA to facilitate nuclear receptors-mediated tran

influence the arrangement and function of both the cytoskeleton and the cells within the tissue.

The BBSome complex is required for ciliogenesis but is dispensable for centriolar satellite funct

enitor to a post-mitotic chromatin remodeling mechanism occurs as neurons exit the cell cycle a

al damage. Mediator of mitochondrial protein catabolic process (also named MALM) by mediati
ein family including RB1 and regulates the cell-cycle during G(1)/S transition. Phosphorylation

nbers share several distinct protein modules, including a propeptide region, a metalloproteinas
alpha-ketoglutarate (PubMed:24495017). The 2-oxoglutarate dehydrogenase complex catalyze
bMed:15781252, PubMed:15775988). Inhibits the transcriptional activity of DDIT3/CHOP and i
as well as size and number of synaptic vesicle clusters. During synaptic stimulation, increases
8594398). Involved in the metabolic reprogramming of cancer cells in a PDK4-dependent man
ucosamine within specific subregions of intact heparin.

ave the property of trapping a certain fraction of elongating RNA polymerases that pass througl

ng dynein to cargos and to adapter proteins that regulate dynein function. Cytoplasmic dynein
quitination of IKBKG; proposed to link A20/TNFAIP3 to ubiquitinated IKBKG. Involved in regula
on and trafficking of activated tyrosine kinase receptors such as PDGFRB. Participates in the r

B1 acts as a receptor for Human rotavirus A.||Integrin alpha-2/beta-1 is a receptor for laminin, c
the preferred ones are glutarate, succinate, adipate, and 3-hydroxymethylglutarate.

ovides a docking site for FAP (seprase) at invadopodia plasma membranes in a collagen-depe
ı which isoform 3 and CSK are sequentially activated by RHOD to regulate the motility of early
or threonine residue on the protein receptor. Has a broad spectrum of substrates for peptides s

ıolecules to discrete plasma membrane domains in polarized cells. May play a role in adherens

e to phosphohydroxythreonine.

ılation and mRNA stabilization.
e of GDP to GTP, converting inactive GDP-bound RAB35 into its active GTP-bound form. Reg
, acts near the plasma membrane and interacts with endosomal membranes to promote endos
ıh high levels of BCL-XL expression will inhibit apoptosis. Inhibits apoptosis induced by BNIP3.

ıeries which require DNA as a template. Histones thereby play a central role in transcription reç

ein for the de novo synthesis of iron-sulfur (Fe-S) clusters within mitochondria, which is require
ınt ubiquitination of viral proteins. Plays a role in restricting the replication of HIV-1, encephalon

sponse. Some genes are also up-regulated (By similarity).
Ins(3,4,5)P3) to produce PtdIns(3,4)P2, thereby negatively regulating the PI3K (phosphoinositi
ediates its antiviral effects through a combination of direct cleavage of single-stranded viral RN/
gulates TNS3 which results in capping the tail of ITGB1. Seems to be involved in mammary cel
969151, PubMed:24368842). Hydrolyzes the endocannabinoid 2-arachidonoylglycerol, and the
eptor (GLYR) to subsynaptic microtubules. Catalyzes two steps in the biosynthesis of the moly
ıeries which require DNA as a template. Histones thereby play a central role in transcription reç
opodia induction, for dendritic spine maturation and synapse formation in a palmitoylation-depe
rity). Required for activation of latent TGF-beta-1 in macrophages and microglia: associates sp
get of TNF-alpha signaling. Involved in the regulation of EGR1, in coordination with RGL2. May

ctivity.
pyroptosis (PubMed:28459430, PubMed:27281216). Produced by the cleavage of GSDME by (

motaxis. Potent mitogen and chemoattractant for cells of mesenchymal origin. Required for nor
ınd stimulate the production of IFN-gamma by resting PBMC.

79). Inhibits the auto- and transphosphorylation activity of BTK. Plays a negative regulatory rol
complex plays a key role in the maintenance of protein homeostasis by removing misfolded or
of galactose (Gal) residues of keratan. Has a preference for sulfating keratan sulfate, but it also
otential RAB3B effector protein in epithelial cells.

ıay serve as Ca(2+) sensors in the process of vesicular trafficking and exocytosis.
ıe death domain of TNFR1.||Component of the 26S proteasome, a multiprotein complex involv
the consensus site 5'-UCCAUCA-3'. May have a role in translation repression (By similarity).
g angiogenesis. Acts as a GTPase activator for the RAC1 by converting it to an inactive GDP-b
ells to the liver.
ınd Glu adjacent to the leaving group at neutral or slightly basic pH. The proteasome has an AT

e in membrane trafficking between the plasma membrane and endosomes (PubMed:17233914
a filamentous (F) actin cross-linking protein.
rmin, creatinine, guanidine, procainamide, topotecan, estrone sulfate, acyclovir, ganciclovir and

nsforming growth factor) and activin type 1 receptor kinases. Binds the TRE element in the pro
chains (MLC). Also regulates actin-myosin interaction through a non-kinase activity. Phosphoryl
mbly of the axonemal radial spokes (By similarity).
in phagocytosis of apoptotic cells. Required for early embryonic development. Involved in cellu

e, after viral entry but before reverse transcription. In addition to acting as a capsid-specific res

lipase (LPL) through the secretory pathway. Each LMF1 molecule chaperones 50 or more mol
tidase activity.

ake of lipoprotein APOE in liver (By similarity).
ex, required for BDNF-NTRK2 endocytic trafficking and signaling from early endosomes (By sin
ochlea, is required for stereocilia maintenance in adult hair cells (By similarity).

clock-controlled genes (PubMed:12397359, PubMed:18411297). Acts as the negative limb of a
ation versus differentiation via its phosphorylation. May be a novel marker for leukemia diagno
ght to provide a framework for the nuclear envelope and may also interact with chromatin. Lam
nber may possibly be a nuclear protein. Similarity to the RAS suppressor protein, as well as exp
s sigma-1.||Seems to play a role in epithelial tight junction formation. Appears early in primordia
ght junctions. Association with PARD3 may prevent the interaction of PARD3 with F11R/JAM1,
ein family including RB1 and regulates the cell-cycle during G(1)/S transition. Phosphorylation

erminal region, and multiple phosphorylation sites and glutamic acid/proline (EP) repeats at the
ssor for RBPJ. Recruits RBPJ to the Sin3-histone deacetylase complex (HDAC). Required for

neries which require DNA as a template. Histones thereby play a central role in transcription re

A translation and stability. Mediates exon inclusion and/or exclusion in pre-mRNA that are subj
guidance.
egulation of alternative splicing and influences mRNA splice site selection and exon inclusion. E
precursors are transported from the base of the cilium to the site of their incorporation at the ti
gration. Necessary for signaling by class 3 semaphorins and subsequent remodeling of the cyto
ependent and -independent mechanisms. After the penetration of retroviral nucleocapsids into
matory processes. Important for defense against pathogens. Cleaves and activates sterol regu
acts as a molecular scaffold for the final stage of MHC class I folding, namely the binding of pe

yrosine kinases and growth factor receptors. Involved in the regulation of cell survival, cell proli
nd autophagy in response to nutrient and hormonal signals; functions as a scaffold for recruitin
the repulsive signaling of Robo and Slit proteins in neuronal migration. SLIT2, probably throug
s, including PRMT3 and PRMT5.
as a primary receptor for some HIV-2 isolates. Promotes Env-mediated fusion of the virus (Pu
nding proteins (SREBPs). Proteolytically cleaves poly(ADP-ribose) polymerase (PARP) at a '21
chains as they emerge from the ribosome and block their interaction with the signal recognitio
r keratins. This gene's expression profile shows that it encodes a type II epithelial keratin, altho
ay play a role in apoptosis and cell proliferation through its interaction with HSPA5 (PubMed:26
le budding at the Golgi and anterograde transport to the plasma membrane.
mage checkpoint control and for proper DNA damage repair (PubMed:20230784). In response
and this transport is coupled to hydrlysis of ATP (PubMed:17408620, PubMed:24576892). The

bidirectional signaling into neighboring cells. The signaling pathway downstream of the recepto
ion during mitosis. May regulate cell proliferation and differentiation in the developing nervous

DNA and RNA viruses, inhibits cell-cycle progression by binding to promoters and activating tra
used as a marker to distinguish chondrocytes from osteoblasts and mesenchymal stem cells i
operoxide, by glutathione.
ng chromatin condensation, thus preventing access of the basal transcription. Isoform 1 and is
s during embryonic development by interacting with other extracellular matrix components.
ne transcriptional activity of E2A-PBX1. Tethers estrogen receptor-alpha (ESR1) to microtubule
s during embryonic development by interacting with other extracellular matrix components. Lad

in Schwann cell myelination (By similarity). May act as a catalyst in increasing the rate of conve
sion by restricting proliferation and promoting apoptosis. Along with WWC1 can synergistically i
ich are essential to the immune response and are present on the cell surface of T lymphocytes

nachinery. Able to restore partial locomotion and axonal fasciculation to C.elegans unc-76 muta
nt by synergy with the Ca(2+) sensor, STIM1.

ular homeostasis (PubMed:30692621). Acts primarily to protect the cell from proteases release
ich p53 mediates its role as an inhibitor of cellular proliferation (By similarity).
igase complex in cooperation with the E2 UBE2D2 that is used not only for the ubiquitination of

ling to the 3'-UTR of CEBPA mRNA and regulation of its translation. Targets ITPR1 mRNA to c

on of other tissues. Cell adhesion requires divalent cations.
be critical for ATP hydrolysis and adjacent subunits in the ring-like structure contribute to the A
of hair (By similarity). Required for the correct growth of hair follicles, in particular for the persi
olate and reduced folic acid derivatives. [provided by RefSeq, Jul 2016]

nt reorganization. Involved in the delivery of mutated CFTR to the plasma membrane. Togethe
notes ASTN1 internalization and intracellular transport of endocytosed ASTN1 (By similarity). S
gi (Flanagan et al., 2006 [PubMed 16793224]).[supplied by OMIM, Mar 2008]
er exchange (end-blocking or capping). It can promote the assembly of monomers into filamen
nodulator of nAChR activity seems to be dependent on the nAChR subtype and stoichiometry, a
IYO5A and MYO7A. May link RAB27A-containing vesicles to actin filaments. Functions as a pr

ulation of the immature compartment of the hematopoietic system. Associates with SMADs in
the myofibril and fiber exterior. May facilitate lateral force transmission during skeletal muscle
oA isomerase activities. Catalyzes two of the four reactions of the long straight chain fatty acid
the pre-translocational (PRE) to the post-translocational (POST) state as the newly formed A-s
s (PubMed:10688651, PubMed:12359225, PubMed:12873986, PubMed:15194700, PubMed:1
enveloped viruses (HIV-1 and other lentiviruses). Via its interaction with PDCD6IP involved in

o sulfane (molecular sulfur) atoms. Requires an additional electron acceptor; can use sulfite, s
S proteasome, in a NUB1-dependent manner. Probably functions as a survival factor. Conjuga
nd adhesion during neuronal, vascular and epithelial development (PubMed:8070404, PubMed

BCR) which leads to internalization of the complex, trafficking to late endosomes and antigen p

leading to activation of MAPK1/ERK2. Plays a role in EGFR trafficking from recycling endoso
tivity (By similarity).
ıs where it forms the core structural component of the paraspeckle sub-organelles. It may act a

membrane into the mitochondrial matrix in an ATP-dependent manner. May act as a co-chap
ltiple transcript variants. [provided by RefSeq, Mar 2010]
eins. Alternative splicing results in multiple transcript variants encoding different isoforms. [prov

ession in germline cells (By similarity). Prohibits the function of several miRNAs by blocking the
-O-alkyl-sn-glycero-3-phosphocholine (lyso-PAF) and lysophosphatidylcholine (lyso-PC), and t
nce of prostaglandins from the circulation. Transports PGD2, as well as PGE1, PGE2 and PGF

radient across the membrane which is generated by electron transport complexes of the respir

ganisms by affecting both the stability and translation of mRNAs. miRNAs are transcribed by R
isfolded nonglycoproteins. May act by forming a channel that allows the retrotranslocation of m

ng and gating properties of AMPA-selective glutamate receptors (AMPARs). Promotes their tar
l may contribute to cell proliferation by acting as a sponge for microRNAs. [provided by RefSeq

ave antiapoptotic effect on neuronal cells.||Multifunctional transcription factor with different regio
or epigenetic transcriptional activation (PubMed:17707229). Plays a central role in beta-globin

orm HSF4B activates transcription.
ly stages (PubMed:18940309, PubMed:27226634). Acts by mediating hydroxylation of 'Arg-11
MAGUKs interact with the cytoskeleton and regulate cell proliferation, signaling pathways, and
n as well as more peripheral structures. It facilitates the budding of vesicles from the Golgi mem
ganisms by affecting both the stability and translation of mRNAs. miRNAs are transcribed by R

A (miRNA) biogenesis. Component of the microprocessor complex that is required to process p
lates inositol trisphosphate production and calcium mobilization upon activation. Together with

imilarity). Required during synthesis of the leading DNA strands at the replication fork, binds at
motes neuronal excitability by elevating the voltage dependence of neuronal sodium channel S

rylase), depending on the context (PubMed:22722849, PubMed:26907567, PubMed:3065331C
core homology common to many enhancers (PubMed:16397300). Important transcription facto

them are long non-coding RNAs (lncRNAs). This gene is expressed in many normal tissues, b
. Component of SWI/SNF chromatin remodeling complexes that carry out key enzymatic activit
S proteins (By similarity). Part of an electron transfer chain functioning in an early step of cytos
robably by facilitating nucleosome assembly. May inhibit cell proliferation by inducing p53-depe

triggers massive exocytosis from neurons and neuroendocrine cells. Receptor for TENM2 that

ximal sequence element (PSE), a non-TATA-box basal promoter element common to these 2 t

ding of sonic hedgehog (SHH) to its receptor patched is thought to prevent normal inhibition by

ction as export adapter involved in mRNA nuclear export such as of histone H2A. Binds mRNA
ay function as non-coding regulatory RNAs. [provided by RefSeq, Jan 2013]
renoids that are essential for normal cell function including ubiquinone and geranylgeranyl pro
dly removed from the transcribed strand of active genes (PubMed:20541997, PubMed:266207
control of fluid and electrolyte homeostasis. In the kidney, am is diuretic and natriuretic, and b
afficking of melanogenic enzymes TYR, TYRP1 and DCT/TYRP2 to melanosomes in melanoc

sitol-calcium second messenger system. May be the cardiac P2Y receptor involved in the regu
PKC, and is involved in the regulation of MAPK8/JNK1 and Ras signaling, Golgi membrane in

ancer progression and metastasis. This transcript regulates expression of genes involved in dif

ement in target genes.
calization of PKD1/PC1 and PKD2/PC2 to the ciliary membrane (PubMed:28530676).
n, translocations and deletions. Part of the Rad21 paralog protein complex BCDX2 which acts
n, translocations and deletions. Part of the RAD21 paralog protein complex CX3 which acts in
-linked polyubiquitination. Acts as an essential factor of the anaphase promoting complex/cycl

ding and degradation of acetylated low density lipoprotein (Ac-LDL) (By similarity).
tamate residues within the C-terminal tail of alpha- and beta-tubulin. Involved in the side-chain

spliceosomal components bound to both the 5'- and 3'-splice sites during spliceosome assembl
ter SLC2A4/GLUT4 translocation at the plasma membrane, thus increasing glucose uptake.
olved in cell survival. Acts as a translational activator. Seems to play an essential role in cold-i
-complexed CDK1 before the onset of mitosis by mediating phosphorylation of CDK1 on 'Tyr-1

mevalonate (MVA) pathway leading to isopentenyl diphosphate (IPP), a key precursor for the b
seems to bind preferentially to single-stranded DNA.

egulates various physiological processes through the generation of approximately 24 hour circa

d cells. Forms membrane-associated multiprotein complexes that may regulate delivery and re
n a tissue-specific manner the ability of NOVA1 to activate exon selection. In addition to its fun

ressive state of many genes, including Hox genes, throughout development (PubMed:2128253
GTPases outside the Arf family.

xin-20 augmentation of gonadotropin-releasing hormone (GNRH) signaling in the hypothalamu
e deleted or epigenetically suppressed in leukemia, and was implicated as a negative regulato

es suggests that is functions as antiporter.
activity against HIV-1 in lymphocytes by decreasing the abundance of HIV-1 viral RNA species.

cross-link repair and in the maintenance of normal chromosome stability.
teins, mitotic progression and chromosome segregation. The CENPA-NAC complex recruits the
ganisms by affecting both the stability and translation of mRNAs. miRNAs are transcribed by R

R) proteins of the spliceosomal complex. May be a constituent of a network of regulatory mecha
ganisms by affecting both the stability and translation of mRNAs. miRNAs are transcribed by R

3K27me) of histone H3, leading to transcriptional repression of the affected target gene. Able t
bled complex at the centrosome that recruits CDK2 required for centriole duplication (PubMed:
ganisms by affecting both the stability and translation of mRNAs. miRNAs are transcribed by R
ne, into the inter-membrane space. Known to process the nuclear encoded protein DIABLO.
the phosphorylation of its regulatory subunit MRLC/MYL9. Through the activation of non-musc

ox sequences. Also suppresses HES1-mediated inhibition of the heterodimer formed by ASCL
ox sequences. Also suppresses HES1-mediated inhibition of the heterodimer formed by ASCL
tion upon stimulation. Inhibits DNA-binding of RELA and NFKB1/p50, and of the NF-kappa-B p
f histone H4 to produce respectively dimethylated 'Lys-20' (H4K20me2) and trimethylated 'Lys-

riptionally repressive state of homeotic genes throughout development. May be involved in spe

by the same enzyme: formation of S-adenosylmethionine (AdoMet) and triphosphate, and sub

atty acyl-CoA substrates (PubMed:15907797, PubMed:18765284). Catalyzes the insertion of a

sequently metabolized to cholesterol (PubMed:21285510, PubMed:28673550, PubMed:23583

nphoma (By similarity). Binds to DNA at special AT-rich sequences, the consensus SATB1-bin

rylation. The respiratory chain contains 3 multisubunit complexes succinate dehydrogenase (co
h of tumors derived from tissues of Muellerian duct origin.
than 100 centrioles. Deuterosome-mediated centriole amplification occurs in terminally differen
in cancers and is thought to act as promoter of cell proliferation. This transcript negatively regu
egulates various physiological processes through the generation of approximately 24 hour circa

four p38 MAPKs which play an important role in the cascades of cellular responses evoked by
ochondrial inner membrane. May also be required for the transfer of beta-barrel precursors fro

nstream of decapping. Involved in actin dynamics and cell cycle progression, mRNA decay and
egulates various physiological processes through the generation of approximately 24 hour circa

stream and independently of NF-kappa-B. Suppresses both NCOA2- and NCOA3-induced enh
deamination of cytosine.
e (PubMed:28516954). Exhibits attenuated catalytic activity toward N(1),N(8)-diacetylspermidin
charides in the cytoplasm.

similarity). Binds to double- and single-stranded DNA sequences. Binds to the transcription sup
folding in an environment in which there are many competing pathways for nonnative proteins.
putative short protein that is localized to the nucleus (PMID:25978564). Expression of this locu
RNA polymerase I-dependent transcription. The rate of PIC formation probably is primarily depe

radation of MDM2. Can reverse MDM2-targeted degradation of TP53 while maintaining suppre
lastoid cells. Insufficient to induce tumorigenicity when overexpressed but contributes to MYC-
Ins(3,4,5)P3), phosphatidylinositol 4,5-bisphosphate (PtdIns(4,5)P2) and phosphatidylinositol 3
ty. Promotes proteolytic processing of NFKB2/P100, which leads to activation of NF-kappa-B v
core histones (H2A, H2B, H3, and H4) form an octamer, around which approximately 146 bp
s and thought to have a role in stabilization of the homeoprotein-DNA complex. Isoform 3 is rec
homolog B) and SULT1A4 (sulfotransferase family, cytosolic, 1A, phenol-preferring, member 4
radient across the membrane which is generated by electron transport complexes of the respira
sequences that define origins of replication have not been identified yet. ORC is required to a
for MAPK1, probably by association with MAPK1 nuclear export signal leading to enhanced EF

ne phosphorylation and this promotes the binding of spleen tyrosine kinase (SYK) and initiation
ganisms by affecting both the stability and translation of mRNAs. miRNAs are transcribed by R

polymerization, playing a key role in the fission of tubules that serve as transport intermediates

flammatory responses. Involved in the induction of T helper 17 cells (Th17) differentiation upon

mtDNA copies and fragmentated mitochondrial network. may act by suppressing ATAD3A func

signaling. Probably involved in signaling by the epidermal growth factor receptor (EGFR) and t
)872744, PubMed:15617512, PubMed:18165688, PubMed:22660477, PubMed:24305054). Wi

llyl diphosphate (DMAPP).

4,5-bisphosphate. Can bind DNA (in vitro). May contribute to the regulation of transcription in th
f Kv1.5.
ulum but their specific functions are unknown. [provided by RefSeq, Nov 2011]
members of the CLK kinase family (By similarity).

nd taurine, respectively. The preferred substrate is 3-sulfino-L-alanine. Does not exhibit any de

ganisms by affecting both the stability and translation of mRNAs. miRNAs are transcribed by R

nitosis (PubMed:12515822, PubMed:15502821, PubMed:16585270). Essential for proper kine
biosynthesis of lipid mediators of the inflammatory response (PubMed:7794891, PubMed:861[illegible]

a G(s) mediated pathway (PubMed:23161870). Involved in neurosteroid inhibition of apoptosis
on (By similarity). Specifically expressed in endothelium of the blood-brain barrier of micro-vess
ecifically in the pancreas and likely functions as a digestive enzyme. [provided by RefSeq, Sep

natic activity has not been demonstrated experimentally. Antigens of the Dombrock blood grou

›recursors (By similarity).

ꓤNA polymerase I-dependent transcription. The rate of PIC formation probably is primarily depe
ıer DNA damage is compatible with cell survival or whether it requires cell elimination by apopt
ıway, which is essential for patterning at organogenesis stages. Mediates phosphorylation of M
ᴎF-kappa-B transcription factor activity (PubMed:21131967). Monomethylates 'Lys-8' of H2AZ

egulates various physiological processes through the generation of approximately 24 hour circa
nitochondrial mRNAs MT-ND2, MT-ND3, MT-CYTB, MT-CO2, and MT-ATP8/6. Promotes MT-
lf recognition by binding to a highly conserved site on classical cadherins, enabling it to monito
ar signaling complexes. DOK3 is a negative regulator of JNK signaling in B-cells through intera
1 and a Wnt family member, such as WNT3A and WNT9B (PubMed:27214281). Both PKD1 ar
ɘd:19782033). The BCR(KCTD13) E3 ubiquitin ligase complex mediates the ubiquitination of R
s actin polymerization (By similarity).
cades, when overexpressed (PubMed:16530727). Involved in dental enamel formation (PubM
Ƨ. cerevisiae) and cyclin-dependent kinase 3 (CDK3) genes. The read-through transcript is a ca
lesterol transport. May play a role in the internalization and endosomal trafficking of various LR
posed of 4 RNA species and approximately 80 structurally distinct proteins. This gene encodes

ɡ intracellular calcium homeostasis. Plays a role in blood pressure regulation through regulatio
e recycling, the uptake of bacteria and endocytosis, protein trafficking in sensory neurons and a

ost gene, containing multiple C/D box snoRNA genes in its introns. Part of the secondary RNA

romosomes, where it plays an important role in the maintenance of repressive chromatin. This
›roteins. Probably acts together with NFU1 (PubMed:27532772).

›lication for the viral genome to be maintained and segregated.||DNA-dependent ATPase and A
ılts in multiple transcript variants. [provided by RefSeq, Mar 2012]

ınction may include MPHOSPH6. Can activate PRKDC not only in the presence of linear DNA b

ED1. Isoform 2, functions as a transcriptional repressor, modulating transcriptional activities of c

e. Shows activity also towards tryptophan, aspartate and hydroxykynurenine. Accepts a variety

promotes endothelial cell adhesion to the extracellular matrix and angiogenesis.
radient across the membrane which is generated by electron transport complexes of the respira

ssion of precursor cell proliferation in myoblasts by SIX1 is switched to coactivation through rec

al RNA (Tanaka et al., 2000 [PubMed 10792466]).[supplied by OMIM, Feb 2010]

ganisms by affecting both the stability and translation of mRNAs. miRNAs are transcribed by R

ranule assembly in response to cellular stress.||Component of the C9orf72-SMCR8 complex, a
n factor. Belongs to the neuron-specific chromatin remodeling complex (nBAF complex). During

olipids from the outer to the inner leaflet of various membranes and ensures the maintenance o
e osteoclast-supporting ability of stromal cells. Binds with STAT3 the consensus sequence 5'-C
ganisms by affecting both the stability and translation of mRNAs. miRNAs are transcribed by R

gene is located on chromosome 20. Alternate splicing results in multiple transcript variants. [pro
ganisms by affecting both the stability and translation of mRNAs. miRNAs are transcribed by R

ganisms by affecting both the stability and translation of mRNAs. miRNAs are transcribed by R

in mitomycin-C (MMC)-induced DNA repair. Acts as a specific E2 ubiquitin-conjugating enzyme

eplication and DNA repair. Affects both insulin and glucagon levels and modulates the express

A-dependent manner.

GF1 stimulation as well as phosphorylation of IRS1 and SHC1 (By similarity).

gmental duplications on human chromosome 1. This gene family has experienced its greatest

ones and vitamins (PubMed:11555828, PubMed:14559847, PubMed:12865317, PubMed:1580
provided by RefSeq, Dec 2017]
ppa-B (PubMed:15799966).
n with the melatonin receptor MTNR1B most likely through receptor heteromerization (PubMed:
romoting activity of BAX.

ganisms by affecting both the stability and translation of mRNAs. miRNAs are transcribed by R

rmation that affects all subunits and leads to opening of an ion-conducting channel across the
ay a role in myocyte differentiation and survival by regulating the integrity of the nuclear envelo
lin-A (CCNA1 and CCNA2), thereby promoting S phase entry. Specifically mediates deubiquitir

nd CRK. Was initially proposed to be required in complex with DOCK1 to activate Rac Rho sm

.
maintenance of presynaptic homeostatic plasticity.
Med:14993212, PubMed:20129055, PubMed:24064211). It is degraded during the mitotic pha
factors.

ganisms by affecting both the stability and translation of mRNAs. miRNAs are transcribed by R
TN. Regulates cofilin-dependent actin cytoskeleton reorganization. Required for normal progre

e activity.

ersion of uridine to pseudouridine at specific residues of ribosomal RNAs or small nuclear RN

naptic clustering of crucial synaptic proteins. Interacts with the cytoplasmic tail of NMDA recep
ne receptor and opening an integral chloride channel.
es through its predominant phospholipase 1 activity, cleaving preferentially acyl groups in sn1 p
across endoplasmic reticulum (ER).
31, activin-A/INHBA and BMP4 by interfering with the transcriptional stimulatory activity of trans

ganisms by affecting both the stability and translation of mRNAs. miRNAs are transcribed by R

from nucleoprotein filaments formed on dsDNA. Could be involved in the turnover of RAD51 p

late (Ser, Thr or Tyr).
thase leading to an increase in glycogen synthesis.

osome alignment and cytokinesis.
n the 3' end of the RNAs and a double-hairpin structure. The box ACA small nucleolar RNAs n
proteasome for degradation. Multiple specialized proteins interact with one another during ER

sulfide metabolism (PubMed:24981631). Provides the link between the first step in mammalian

ed:21671375, PubMed:25637936, PubMed:22178193). In addition to its cholesterol-synthesizi
regulate the expression of XIST, which is essential for the initiation and spread of X-inactivatio

:er (NSCLC). Differential DNA methylation of this locus may be associated with intellectual disa

:tion as a coinhibitor of other CREBBP/EP300-dependent transcription factors.||Tissue-specific

ERK1/2 (By similarity). Inhibits TGFB-induced epithelial-to-mesenchymal transition in lens epitl

A[TC][AG]-3'.
DK-specific inhibitor CDKN1A/p21 (PubMed:11980906). May play a role in the regulation of RI

ie nucleoplasm. This RNA is processed from an intron of the prohibitin 2 host gene. It includes

(By similarity).
-mRNA processing. snoRNAs form part of the small nucleolar ribonucleoprotein particles (snoF

:he pancreas. May act as a chaperone that inhibits aggregation of misfolded proteins.
tein (S300-II) stimulates initiation of transcription. Binds to both direct repeats and palindromes
ain containing 7 cadherin repeats. The gene product is an integral membrane protein that is thc

'). Modulates YAP1 activity as transcriptional regulator (PubMed:30447097).

ently from catalytic activity, promotes cell invasion in response to constitutive RhoA activation b
in eukaryotes) from the lipid carrier dolichol-pyrophosphate to an asparagine residue within an
ganisms by affecting both the stability and translation of mRNAs. miRNAs are transcribed by R
ganisms by affecting both the stability and translation of mRNAs. miRNAs are transcribed by R
g the exchange of subunits at these ends. Unlike other capping proteins (such as gelsolin and
me 6. Members of the LY6 superfamily typically contain 70 to 80 amino acids, including 8 to 1(
or of SHH signaling in the absence of SHH ligand: interacts with SUFU, thereby inactivating the

lecule.

Mutations in this gene cause combined oxidative phosphorylation deficiency-20, and are also

nding protein (CREBBP) to nuclear bodies. Component of the CREST-BRG1 complex, a multip

ie Notch signaling pathway (PubMed:29856954, PubMed:29856955, PubMed:29561261). Able
erentially uracil, thymine, and cytosine (deoxy)nucleoside di- and triphosphates by an antiport r
egulates trophoblast cells migration. It regulates cytoskeletal remodeling by controlling the activ

o phosphatidylserine (1,2-diacyl-sn-glycero-3-phospho-L-serine or PS) (LPSAT activity). Prefer
d to stabilize the Ku heterodimer, composed of XRCC5/Ku80 and XRCC6/Ku70, at double-stra

.

nt signaling pathway upon stimulation by bone morphogenetic proteins, such as GDF2/BMP9 a
ɔgenic program and meiotic prophase entry. Acts downstream of bone morphogenetic protein (
promoter region of a number of genes whose products are involved in cell cycle regulation or
g its extracellular region with that of several known growth factor receptors, such as EGFR and
ype calcium channels that contain CACNA1A as pore-forming subunit (By similarity). Regulate

IRT2 around early anaphase. May dephosphorylate the APC subunit FZR1/CDH1, thereby pro
nd of epithelial calcium ion transport. Regulates endochondral bone development and epithelia

ng NEK2 kinase activity (PubMed:26220856).

ales. This X chromosome gene is intronless and was identified because its transcription escape
).
n interactions. Several alternatively spliced transcript variants encoding different isoforms have

rylation. The respiratory chain contains 3 multisubunit complexes succinate dehydrogenase (co
SLC7A8 and mainly transports branched-chain amino acids and phenylalanine. Plays a role in

e deacetylases. Acts as a specific transcriptional repressor for PTCH1 during embryonic devel
nzyme UBC12. Neddylation of cullins play an essential role in the regulation of SCF-type comp
ıs mascRNA) from its 3' end. The resultant mature transcript lacks a canonical poly(A) tail but i

) for RAC1 and CDC42. Activated by the binding to subunits beta and gamma of the heterotrim

ly (MVB)-like organelles in dendrites (By similarity).
oglobulins, also known as antibodies, are membrane-bound or secreted glycoproteins produce
are cell-surface proteins that are characterized by the presence of four hydrophobic domains. T
ct-dependent bidirectional signaling into neighboring cells. The signaling pathway downstream
PS6. Probably plays a role under changing environmental conditions (oxygen, glucose, nutrition
ng dynein to cargos and to adapter proteins that regulate dynein function. Cytoplasmic dynein
aining negatively charged phospholipids in a Ca(2+)-independent manner (By similarity).

script variants. Some transcript variants may promote growth, while others may act to negativel
unctions, including behavioral responses to stress (By similarity), amyloid-beta peptide generat
it converts cholesterol and phosphatidylcholines (lecithins) to cholesteryl esters and lysophosp

P-GlcUA and N-acetylgalactosamine (GalNAc) from UDP-GalNAc to the non-reducing end of t
plication forks. The MMS22L-TONSL complex is required to maintain genome integrity during D
18762581). Facilitates constitutive LRP6 phosphorylation by CDK14/CCNY during G2/M stage

of the cyclin-dependent kinase pair (CDK9/cyclin T) complex, also called positive transcription
o DNA lesion upon damage by the RAD17-replication factor C (RFC) clamp loader complex. A

chromaffin granules and synaptic vesicles.

ance of other differentiated tissues. Probable substrate-recognition component of an E3 ubiqui
on. In vitro, the processed forms GRO-alpha(4-73), GRO-alpha(5-73) and GRO-alpha(6-73) sh

nd homotypic chains.
e-mRNA processing. snoRNAs form part of the small nucleolar ribonucleoprotein particles (sno
latelet dense granules and melanosomes. In concert with the AP-3 complex, the BLOC-1 com
those complexes on gene promoters (PubMed:19131338, PubMed:23178126). Enhances ligan
promoting APC activation/maturation and driving Th1 immune response.||Lymphocyte activati

conversion of diacylglycerol pyrophosphate into phosphatidate but can also act on phosphatida
ilies. This gene is one of several chemokine-like factor genes located in a cluster on chromoso
port metabolon; hence maximizes the local concentration of bicarbonate at the transporter site.

ng (PubMed:20676357). Component of a putative tumor-recognition complex involved in the fu

PubMed:25855295). Required for normal body fat and glucose homeostasis. ADIPOQ-binding

ate filaments in the heart muscle sarcomeres (PubMed:27733623). Isoform 2 might play a role

al RNA and oligomerizes. The viral protein also possesses a nuclear import and a nuclear exp

ctivity.
signals from, the activated insulin receptor (INSR). Potent inhibitor of insulin-stimulated MAPK3
y of receptor tyrosine kinases which are crucial for migration, repulsion and adhesion during ne

modulate cell shape and motility after injury. May be involved in multiple neurite formation (By s

ganisms by affecting both the stability and translation of mRNAs. miRNAs are transcribed by R
(By similarity).

HCFC1-dependent manner. Also suppresses the HCFC1-dependent transcriptional activation l
ion of arterial blood pressure, and increased somatostatin secretion from the pancreas. Neurop

riggers massive exocytosis from neurons and neuroendocrine cells. Receptor probably implicat
le in nasal colonization.||(Microbial infection) Binds S.pneumoniae PsrP, mediating adherence
lls, antigen-independent and -dependent interactions of T-lymphocytes with target cells and an
se-activating protein domain which stimulates GTP hydrolysis by RAC1, RAC2 and CDC42. Ac
ole in neuronal apoptosis.

role in purine metabolism and in adenosine homeostasis. Modulates signaling by extracellular
ifferent membrane traffic pathways. Adaptor protein complexes are vesicle coat components a
ection, serves as receptor for HCoV-229E spike glycoprotein.||(Microbial infection) Mediates as
form inositol 1-phosphate.

enesis of melanosomes and promote their association with the apical cell surface by inducing g
atic arginase functions to regulate L-arginine bioavailability to nitric oxid synthase (NOS). Argir

trix. The encoded protein is postranslationally glycosylated and localized to the lysosome. This

ant roles in coordinating transcription activation and repression by MAFK (By similarity). Togeth
endothelial survival during infection (By similarity). Can inhibit apoptosis induced by serum sta
pes such as tissue-resident memory T (Trm), natural killer (trNK) and natural killer T (NKT) cell
actin reorganization, cell migration, cell proliferation and survival, cell adhesion, and apoptosis
PubMed:20673843). Plays a roles as pro-apoptotic protein that positively regulates intrinsic ap
g their poly(A) tail removal or deadenylation, and hence provide a mechanism for attenuating p
er that elicits calcium release from intracellular stores. May be involved in pre-B-cell growth.
act as a bridge between myosin and actin filaments). Stimulates actin binding of tropomyosin

/ play an important role in macrophage function. May play a role in regulating cytoplasmic and/c
nd signal transduction. Proteolytically cleaves MYOC at 'Arg-226' (PubMed:17650508). Proteol
poly(ADP-ribose) polymerase (PARP) at a '216-Asp-|-Gly-217' bond. Cleaves and activates ste
2 that targets to lipid rafts and drives caveolae formation. Mediates the recruitment of CAVIN pr

ular matrix or directly to stromal cells. Could act as a scaffold for the attachment of lineage spec

n the prevention of platelet aggregation by hydrolyzing platelet-activating ADP to AMP. Hydroly
ges in the tissue microenvironment (PubMed:16541107, PubMed:19703720, PubMed:2272606
a-beta-CLIP complex, thereby preventing the loading of antigenic peptides to the MHC class II
mune effector molecules.
another cell. Cadherins may thus contribute to the sorting of heterogeneous cell types. Acts as
anner in connecting cells; cadherins may thus contribute to the sorting of heterogeneous cell typ
cting cells; cadherins may thus contribute to the sorting of heterogeneous cell types.
cting cells; cadherins may thus contribute to the sorting of heterogeneous cell types. May act a
of an ion-conducting channel across the plasma membrane.
s a non-specific lysosomal peptidase which generates tripeptides from the breakdown products
gether with laminins, proteoglycans and entactin/nidogen.
gether with laminins, proteoglycans and entactin/nidogen.
ous distribution. Type V collagen binds to DNA, heparan sulfate, thrombospondin, heparin, and
nd the COL2 and NC3 domains may be localized in the perifibrillar matrix.
ess. Binds DNA on the 5'-CCAC[GA]-3' half of the ER stress response element (ERSE) (5'-CCA
cially mononuclear phagocytes, such as macrophages and monocytes. Promotes the release c

extracellular phosphoglycoprotein MEPE (PubMed:12220505). Involved in the solubilization o
t endoprotease activity against fibrinogen at acid pH. May play an important role in extracellula

mponent of the epithelial apical junction complex that may function as a homophilic cell adhesi
and TNF-alpha-induced necroptosis (PubMed:18636086, PubMed:26670046, PubMed:27458
organization. Involved in the regulation of smooth muscle contraction. Regulates both type I (ca
1 and coronavirus IBV replication. Binds to the RRE sequence of HIV-1 mRNAs.||Acts as an AT

activity is toward ERK1.
sumably by catalysing their dephosphorylation. Expected to display protein phosphatase activit

effect on most types of cells and tissues. When expressed in rat HTC4 hepatoma cells, is capa
actin-bundling activity is reversibly regulated upon its phosphorylation by the cAMP-dependent p
act-dependent bidirectional signaling into neighboring cells. The signaling pathway downstrean

and stimulates transcription.

esis, through the formation of HS proteoglycans (HSPGs) (PubMed:28132690, PubMed:2814
n of several signaling pathways including phospholipase C (PLC), intracellular calcium, mitoge
ncluding tissue remodeling, fibrosis, wound healing, inflammation and tumor growth. Both plas

ting enzyme in gluconeogenesis. Plays a role in regulating glucose sensing and insulin secretio
ation in absence of ligand. Recruits SMAD7 to ACVR1B which prevents the association of SM
aments to membrane glycoproteins. Anchors various transmembrane proteins to the actin cyto
involved in reorganizing the actin cytoskeleton in response to signaling events, and may also
nesis and in the development of the vascular network and the cardiovascular system during em
adhesion, integrin-mediated signaling, cytoskeletal remodeling, cell motility, immune response
Med:26319512). Important in nervous system development (PubMed:24415674).
ptotic mimicry (PubMed:21501828). Plays a role in Dengue cell entry by apoptotic mimicry (Pu
g and delivers antimicrobial peptides to autophagolysosomes, providing broad host protection
iral activity against influenza virus. Promotes oxidative killing and delivers antimicrobial peptide

nyl compounds. The metabolism of glutathione releases free glutamate and the dipeptide cyste
ic ligand-gated chloride channels. Channel opening is triggered by extracellular glycine (PubMe
pendent phospholipase activity (By similarity). Binds gangliosides and stimulates ganglioside G
ns (PubMed:22609986, PubMed:15525651, PubMed:15240885, PubMed:17565996, PubMed:
e nucleotide-binding proteins (G proteins) are involved as modulators or transducers in various
ch receptor phosphorylation initiates beta-arrestin-mediated receptor desensitization, internaliz
irus protein NS5A.||Key regulator of inflammation, lipid metabolism and mitochondrion homeos

odium falciparum merozoites are most likely located within the extracellular domain. Glycophor
lycosaminoglycans, heparin or dermatan sulfate. In the presence of the latter, HC-II becomes
on. Involved in embryogenesis and hematopoiesis (By similarity).
unction in nucleosome phasing and in the 3'-end processing of mRNA transcripts. They are als
expression in differentiated tissues. Is thought to act as a 'pioneer' factor opening the compac
ine.
HSPA1A (in vitro) (PubMed:24318877). Plays a role in protein transport into mitochondria via
l as neuronal regeneration. Promotes neurite outgrowth from cortical neurons grown on a mon
atases PP2A, PP4 and PP6 by protecting their partially folded catalytic subunits from degradat
mponents of the IL2 receptor, including IL2RB and probably IL2RG but not IL2RA (PubMed:81
esent in oocytes and is involved in sperm-egg fusion (By similarity). Integrin alpha-6/beta-4 (IT
ence (ICS)) and represses those genes. Also acts as an activator for several genes including H
. Exhibits antiviral activity against RNA viruses including hepatitis C virus (HCV), hepatitis A vi

ing the resting membrane potential in electrically excitable cells. Probably participates in establ

s during embryonic development by interacting with other extracellular matrix components.||Lar
s during embryonic development by interacting with other extracellular matrix components.
s during embryonic development by interacting with other extracellular matrix components.
IFNG production by natural killer cells (By similarity).||Binds galactosides (PubMed:18005988).
and the terminal methyl group of the fatty acyl chain. Desaturates (11E)-octadecenoate (trans-
involved in, may contribute directly to or modify tumorigenesis.
ubMed:26838787). Regulator of Ras expression. May play a role in tumor suppression. Plays a

e (allysine) (PubMed:27735137). Acts as a transcription corepressor and specifically mediates
also be implicated in signaling events and activation of gene transcription. May be involved in

e attachment of the heterotrimeric complex to the cell surface. Isoform 2 is probably non-functi
e element found in the SKP1, BAX, DCPS, and SELENOH promoters.||Kaliocin-1 has antimicr
AD (R-SMAD). SMAD1/OAZ1/PSMB4 complex mediates the degradation of the CREBBP/EP3
ay allow melanoma cells to interact with cellular elements of the vascular system, thereby enha
h factor- and stress-induced genes. Mediates cellular functions not only in skeletal and cardiac
tyrosine kinase that transduces signals from the extracellular matrix into the cytoplasm by bind

rase II transcription by suppressing transient pausing by the polymerase at multiple sites along
PubMed:2153297, PubMed:1645757). In case of HIV infection, interacts and cleaves the secre

(comp), during development, haemostasis and pathological conditions (arthritic disease). May

ria, fungi and other pathogens.||(Microbial infection) Interacts with Hepatitis B virus envelope p
eduction of methionine sulfoxide in proteins to methionine.
sequence 5'-CAC[GA]TG-3'. MXI1 thus antagonizes MYC transcriptional activity by competing f
omplexes.
gulates various physiological processes through the generation of approximately 24 hour circa
Binds to calmodulin in the absence of calcium (By similarity).

T5A which activate downstream NFkB signaling pathway and may result in the inhibition of WN
n of the neurotrophic factor SERPINF1 and relocalizes beta arrestin at the plasma membrane;
also play a role in other cellular processes such as apoptosis, cell growth, differentiation and ge

ation, or cell cycle regulation. Plays a role in dendrite spine morphogenesis as well as synapse
tive alpha-amidated peptides, a terminal modification in biosynthesis of many neural and endoc
phology/dynamics and apoptotic cell death via local modulation of cAMP/PKA signaling in the m
aerobic respiration. Can also phosphorylate PDHA2. Decreases glucose utilization and increas
on (TEM) under most inflammatory conditions (PubMed:19342684, PubMed:17580308). Tyr-69
olipids from the outer to the inner leaflet of various membranes and ensures the maintenance

ological activity (PubMed:9593714, PubMed:15664000, PubMed:18162525, PubMed:1859763
drate units and are essential for the stability of the intermolecular collagen cross-links.
development.
low density lipoproteins or VLDL) into high-density lipoproteins (HDL) as well as the exchange
(By similarity). Mediates SEMA4A- and SEMA4D-dependent inhibitory synapse development
enzyme to a particular subcellular compartment.
and Glu adjacent to the leaving group at neutral or slightly basic pH. The proteasome has an AT
e (PubMed:17331072, PubMed:18393449, PubMed:23704371, PubMed:30367560, PubMed:2
ation, most likely by acting at the level of SYK to affect downstream events such as phosphory
2 regulating its activity.||The first PTPase domain has enzymatic activity, while the second one

PubMed:21454754). Binding to chondroitin sulfate and heparan sulfate proteoglycans has opp
te binds to a variety of effector proteins to regulate cellular responses, such as secretory proce
c transformation and membrane trafficking. Accomplishes its multiple functions by interacting w
GTP. Preferentially activates HRAS in vivo compared to RRAS based on their different types of
rt from the Golgi apparatus to the lysosomes by a pathway that is independent of the mannose
ation.
ng and activation of CCR2 and induces a strong chemotactic response and mobilization of intra
the release of gelatinase B. This protein can bind heparin. Binds to CCR1, CCR2 and CCR3.
response and mobilization of intracellular calcium ions (PubMed:11352563, PubMed:1103508
chemotactic and angiogenic properties, it has strong antibacterial activity against Gram-positiv
to integrins ITGAV:ITGB3 and ITGA4:ITGB1. Can activate integrins in both a CX3CR1-depend
interactions with E-, P- and L-selectins, mediates rapid rolling of leukocytes over vascular surfa
e in regulating cell growth and differentiation in specific cell types. SFRP1 decreases intracellul

to parallel bundles (PubMed:20393565, PubMed:21685497, PubMed:23184945). Plays a role i

y similarity). Binds cooperatively with POU3F2/BRN2 or POU3F1/OCT6 to gene promoters, wh

mmune response receptors, integrins and other adhesion receptors, receptor protein tyrosine k

ɛmble during myogenesis and it contributes to myogenic membrane structure and differentiatior
s I with peptide (peptide loading).
Known to act on MMP-1, MMP-2, MMP-3, MMP-7, MMP-8, MMP-9, MMP-10, MMP-13, MMP-1
d by CTNNB1 and TCF family members in Wnt signaling. The effects of full-length TLE family r
' similarity).||Lysosomal integral membrane protein that regulates the localization and activity of

cture. The BCR(TNFAIP1) E3 ubiquitin ligase complex mediates the ubiquitination of RHOA, lea
e heterotrimer formed by TRAF1 and TRAF2 is part of a E3 ubiquitin-protein ligase complex tha
val. Part of signaling pathways leading to the production of cytokines and interferon. Required f
y association with the receptor cytoplasmic domain and kinases. Mediates activation of NF-kap
ysteine residue in E1, yielding a ubiquitin-E1 thioester and free AMP. Catalyzes the ISGylation
PubMed:20061386). E2 ubiquitin conjugating enzyme that transfers ubiquitin to MAEA, a core
the transfer of glucose from UDP-glucose to ceramide to produce glucosylceramide/GlcCer (F

its found in various non-epithelial cells, especially mesenchymal cells. Vimentin is attached to t
clathrin-coated pits. Binding to Reelin induces tyrosine phosphorylation of Dab1 and modulatio
endages of neurons linked by dendrodendritic gap junctions. May operate in the control of brain
thway. Required for normal timing of IHH expression during embryonic bone development, nor
5' exons reside in the pseudoautosomal region and the remaining exons within the X-specific e
lays a role in ubiquitin-mediated protein degradation during muscle atrophy. Plays a role in the

tem. Plays a role in extracellular matrix formation.
by nuclear hormone receptors. Positive regulator of the circadian clock gene expression: stimu
reorganization and cell migration (PubMed:15723050, PubMed:9418861, PubMed:9092543). I
formation of focal adhesion complexes, activation of the protein kinase PTK2/FAK1 and subse
ory operations.
ct domains critical for saltatory conduction of nerve impulses in myelinated nerve fibers. Demar
ium channel TRPV6, and is also localized to the inner mitochondrial membrane where it may p
synthesis (PubMed:17581632, PubMed:25849773, PubMed:27462815). The eIF-3 complex a
f bile acids and glucuronides from cholestatic hepatocytes (By similarity).
ggesting a regulatory role in glial cell. Also cleaves alpha-2 macroglobulin. May be involved in o
sine to the 5'-cap structure of mRNAs (PubMed:9790902, PubMed:9705270, PubMed:103472
so to TNFRSF11B/OPG (PubMed:26457518, PubMed:10549288). Induces apoptosis. Its activi

ed receptor. The resulting death-inducing signaling complex (DISC) performs caspase-8 proteo
cardiomyocyte proliferation and heart development.
e its GTPase activity. It associates with calmodulin. Could serve as an assembly scaffold for th

tion. Modulates phagocytosis mediated by Fc gamma receptor and ARF6. Modulates PXN recr

autophagosomes. Interacts directly with both the cargo to become degraded and an autophagy

f the germline meiotic cell cycle and additionally in the control of mitotic cell cycle in some soma
osome-trans-Golgi network structure (By similarity). Together with LRRK2, plays a role in the re
sms that regulate neuronal shape, growth, and plasticity, through their effects on the actin cytos
TPN18 leading to ABL1 dephosphorylation. May play a role as a scaffold protein between PTP
ch as lactate, pyruvate, branched-chain oxo acids derived from leucine, valine and isoleucine, a
d thereby regulate dopamine transport back from the synaptic cleft into the presynaptic termina
CF1/HNF1A and FOXO1. Inhibits epithelial cell migration. Mediates colon carcinoma cell surviva
he cell surface (By similarity).

May also have functions in cellular differentiation (By similarity). Induces differentiation of fibrol
s, mainly through the type II receptor, and is released upon activation of signaling. May recruit

, spreading and cell migration and acts as a negative regulator in integrin-mediated cell adhesi

-terminal extension that inactivates the chromo domain (PubMed:19808672).||[Isoform 2]: Chro
of non-reducing N-acetylglucosamine (GlcNAc) residues within keratan-like structures on N-link
upstream of the JUN N-terminal pathway. Phosphorylates SMAD1 on Thr-322.
ANK1 and SHANK3. By physically linking GRM1 and GRM5 with ER-associated ITPR1 recept
the MAPK cascade to form a functional JNK signaling module. Required for JNK activation in r
can in arthritic diseases. Could also be a critical factor in the exacerbation of neurodegeneratic

biquitination and proteasomal degradation of SIAH1, and thereby increases cellular levels of SI
ells. Binds matrix metalloproteinases (ADAMs), NADPH oxidases (NOXs) and phosphoinositide
somes and the Golgi. As a RAB9A effector it is involved in recycling of the mannose 6-phospha
exchange factor (GEF) that promotes the exchange of GDP to GTP, converting inactive GDP-b
synapses.
ytoskeleton.
By similarity).
P (PubMed:16923123). Plays a role in endocytosis and intracellular protein transport. Recruitec
ite immunity. Acts as a regulator of TRAF function by maintaining them in a latent state. Blocks
Bim-alpha3 induce apoptosis, although less potent than isoform BimEL, isoform BimL and iso
AP1 (By similarity). Required for tissue-specific adhesion of T lymphocytes to peripheral tissues
y function in cell proliferation and migration and play a role in the maintenance of epithelial integ

892, PubMed:23236473). Mediates CPEB3-accelerated mRNA deadenylation by binding to CI
ytoskeleton. Promotes formation of actin filaments. Part of the WAVE complex that regulates la
ent, triacylated lipopeptides (PubMed:20037584). In response to diacylated lipopeptides, forms
growth cones. Could serve as a ligand that guides specific growth cones by a motility-inhibiting

equences among different species supports their functional significance. PEST sequences typi

as no effect on cell proliferation. Negatively regulates mitochondrial fusion in cooperation with r

ial growth cone collapse and cell migration (By similarity).
ng to cell shape changes. Induces pseudopodia formation in fibroblasts.
osomal damages. Plays a role in the stress-induced biogenesis and degradation of protein agg
vessels separation by binding CLEC1B, triggering CLEC1B activation in platelets and leading t
or systems, for example the T-cell, leptin and insulin receptors. Once phosphorylated, function
ing (PubMed:30610177).
led:27214403, PubMed:28262558). Does not demethylate histone H3 'Lys-9' or H3 'Lys-27'. De
s and subsequent Hedgehog signaling pathway that requires the presence of intact primary cili

H), providing the potential to regulate intracellular levels of acyl-CoAs, free fatty acids and CoA
lease of cytokines and IFNG by activated T-cells. Mediates the response of T-cells toward infe
nd signal transduction.

RII isoforms with downstream effectors to capture, amplify and focus diffuse, trans-cellular sig

1. Does not bind fibulins. It probably has a role in cell-extracellular matrix interactions.
eptor.
osis during neuronal development. May be involved in regulation of anti-apoptotic effects of IG
otein-protein and protein-DNA interactions. [provided by RefSeq, Jul 2008]
to the subsynaptic cytoskeleton. May induce enrichment of PSD-95/SAP90 at the plasma mem
ER) (PubMed:10788476). The coat has two main functions, the physical deformation of the en
in transcriptional activation of glycolytic target genes. Involved in glucose-responsive gene reg
eceptors (PubMed:18675823). Involved in mitochondrial motility. When O-glycosylated, abolish
. May mediate the enhancement of phagocytosis in monocytes and macrophages upon interac
calizes to contact sites between the endoplasmic reticulum and endosomes and acts by promo
histone H3 'Lys-4', H3 'Lys-27', H3 'Lys-36' nor H4 'Lys-20'. Only able to demethylate trimethyl

role in cell growth promotion and hepatoma formation. Inhibits the TGF-beta signaling by intera

›olyubiquitin chains to TAB2 promotes autophosphorylation of MAP3K7 at 'Thr-187'. Involved ir

›osphatase enzyme which catalyzes the conversion of phosphatidic acid to diacylglycerol during

ıg mitosis, it mediates the ubiquitination and subsequent proteasomal degradation of AURKA,
ıt-activated LRP6. Regulates thymocyte proliferation.
T)' motif in the apical loop.
ıogenesis (PubMed:28488682).
)179534). May have a general role in telomere regulation (PubMed:12676087, PubMed:12699(
I by TULP3 and the IFT-A complex to primary cilia and acts as a regulator of the PKA-depende
ıffinity to alpha-tocopherol. Also binds with a weaker affinity to other tocopherols and to tocotrie
ıe 3'-splice site. Regulates alternative splicing of tissue-specific exons and of differentially splic
ːes in the promoter region.
in glutamatergic synapse development and is required for SEMA4A-mediated excitatory synap
bably also other latrophilins that are expressed at the surface of adjacent cells. May play a role
08669, PubMed:20829356, PubMed:30918116). May play a role as a Ca(2+)-leak channel to r

ıl glutaminyl residue and a lack of importance of chain length after the second residue. Also ca
ıg to the generation and extracellular release of beta-cleaved soluble APP, and a correspondir

ed replication forks (PubMed:19525956, PubMed:21613998, PubMed:21720370, PubMed:236
7A or WNT7B) (PubMed:28289266, PubMed:30026314). Plays a key role in Wnt7-specific res

ːceptor ubiquitination and accelerated intracellular degradation.
hibiting MYRF self-cleavage and N-fragment nuclear translocation. The secreted form promote
ın bind to the ER membrane protein VAPA and recruit VAPA to plasma membrane sites, thus
ly proteins lipidation and starvation-induced autophagy. Isoform 4 is also required for autophag
binding the lipid in a hydrophobic cavity. Able to transfer phosphatidylinositol in vitro.
nine receptor DRD2. Inhibits signal transduction by increasing the GTPase activity of G protein

ıt pathway. Binds preferentially to the sequence 5'-TTCATTCATTCA-3'. Binding to the histone
PubMed:15961997).
ıne protein KREMEN that promotes internalization of LRP5/6. DKKs play an important role in v

and by the release of cytochrome c from the mitochondria into the cytoplasm. Plays a role in n
ibit growth of glioma cells by promoting arrest of mitotic progression at the G2/M transition. Fib
ınd also caspase-9 in a probable cell type-specific manner. Involved in activation of the mitocho

led:24120868). In ribosome biogenesis, may also play a role in rRNA transcription (PubMed:27
arly endosomal transport.
OS). Modulates the nuclear activation of ERK1/2 and the ELK1 transcription factor, and is capa
dependent interactions with E-, P- and L-selectins.
nd ARF5 and a lesser activity towards ARF6. May coordinate membrane trafficking with cell gro

tic membrane including NMDA-type and metabotropic glutamate receptors via complexes with
phosphate (dCMP), deoxyguanosine monophosphate (dGMP) and deoxythymidine monophos
K2 to the plasma membrane thereby serving as an adapter to facilitate the phosphorylation of C
GGGGG-3' (PubMed:14621294). Binds to the HMGN1 promoter and may repress HMGN1 exp
rant receptors (By similarity).
a preference for FAHFAs with branching distal from the carboxylate head group of the lipids (
and neuropeptide head activator. May transduce physical stimuli in mast cells. Activated by te
ress through mitosis and cytokinesis. Required for normal formation of the cleavage furrow at t
cells.
are enzymes that metabolize many different compounds, such as steroid hormones, prostagla
a beta-oxidation (PubMed:17681178, PubMed:24269233, PubMed:22633490). ACSL5 may ac
otein signaling pathways.
ne residue to an already glycosylated peptide. In contrast to other proteins of the family, it does

d:30970241). Regulates the morphology, size and distribution of LDs (PubMed:31293035, Pub
lasmic reticulum and translocate inorganic phosphate into the opposite direction. Independent
l amyloid-beta protein 42), without affecting the cleavage of other gamma-secretase targets su

as a guanine nucleotide exchange (GEF) of RAB5B, required for activating the RAB5 proteins
he formation of thin, actin-rich surface projections called filopodia. Functions cooperatively with
). Inhibition of mTORC1 is mediated by a pathway that involves DDIT4/REDD1, AKT1, the TS
ible factor (HIF) alpha proteins. Hydroxylates a specific proline found in each of the oxygen-dep
cultured cells. Regulates cAMP-induced GFAP gene expression via STAT3 phosphorylation (

rface. Hydroquinone oxidase activity alternates with a protein disulfide-thiol interchange/oxidore
the stimulation of transcription in a Ras-independent fashion (By similarity).
quired for the recruitment or accumulation of SETDB1 to the endogenous retroviruses (ERVs)

populations have found an association between polymorphisms in this gene and sporadic am
high-affinity binding of a stem-loop structure in their 5' UTR. This regulation requires VIM and M
imuli into a flow of ion (By similarity).
RefSeq, Jul 2008]
/ 11-kDa protein, which enhances viral replication.||RNA-binding protein that specifically bind th
ndritic spine morphogenesis in Purkinje cells and in hippocampal neurons, via a CDC42-media

with shorter fatty acyl-CoA substrates (PubMed:15220348). It may play a role in the production
tions in the brain.
tions in the brain.
281216). The physiological relevance of this observation is unknown (Probable).

n its target gene promoters. Associates with cyclin D1 promoter containing Myc E-box sequen

as a facilitator of neurite branching. Regulates neurite fasciculation, branching and extension i

lum (ER) (By similarity). Contains unique domains for binding cholesterol and the PM, thereby

o lysosomes via multivesicular bodies. E3 ubiquitin ligases accept ubiquitin from an E2 ubiquiti
Med:26388029). Enhances the USP1-mediated deubiquitination of FANCD2; USP1 being alm
, late onset alzheimer disease, and emphysema distribution. [provided by RefSeq, Mar 2017]

osome assembly. Plays a role as an effector in the TP53-dependent apoptotic pathway (By sim
A to 3-methylglutaconyl-CoA, a critical step for leucine and isovaleric acid catabolism.
generate fructoselysine-3 phosphate (PubMed:11016445, PubMed:11522682, PubMed:1197
Peptide trimming is essential to customize longer precursor peptides to fit them to the correct le
transfers the ubiquitin to targeted substrates. Interacts with SMAD1 and SMAD7 in order to trig

ired to ensure functional dimerization of BTB domain-containing proteins (dimerization quality c

f damaged mitochondria via selective autophagy (mitophagy) by mediating activation and trans
). When unphosphorylated, induces cell migration (By similarity). When phosphorylated by MAI

ion. Acts as an activator and inhibitor of sodium-coupled chloride cotransporters and potassiun
s alpha/beta hydrolase fold and secretory lipase domains. [provided by RefSeq, Jul 2008]

gulation of cytoskeletal organization via Rho family GTPases. Inhibits PAK2 proteolytic fragme
and regulates its activity. Suppresses the rate at which PP1 dephosphorylates (inactivates) gl
PubMed:18070604). Involved in the functional recruitment of the class 1 Sin3-histone deacetyla
rgos into multivesicular bodies. May be involved in cell growth and differentiation.

e GTP. Required for normal RAB31 function.
ed:17461779). Regulates the activity of the mTORC2 complex in a substrate-specific manner p
osis system.||Possible role in axon guidance through interaction with RTN4R.

ne that increases the stability of TP53 protein by promoting the ubiquitination and degradation c

lases to gene promoters. Regulates cell adhesion, migration and proliferation. May be required
icking and/or retention of GLI2 and GLI3 proteins at the ciliary tip (By similarity).
me preference toward medium-chain substrates (PubMed:17762044). Plays a role in adipocyte
er mediated cell lysis as well as a role of marker for detection of neuroblastoma cells. May be ir
lamination and retention of neural stem cells from the subventricular zone during neurogenesis
tem and is required to the efferent innervation of hair cells in the olivocochlear bundle for the m
and epithelial polarization by controlling the polarity, plasticity, duration and extent of protrusio

by promoting proteasomal degradation of the repressor SOCS1 (PubMed:12163497). Plays a

es to regulate mitochondrial quantity and quality by eliminating the mitochondria to a basal leve
(PubMed:28028151, PubMed:22461490). During immune responses, required for interstitial d
a modulator of autophagosome formation.
ndothelium and blocks further differentiation into blood precursors (By similarity). May be requir

ay. Plays a role in homeostatic control of innate immunity and in antiviral defense mechanisms
esion of cells to type 1 collagen and gelatin, reorganization of the actin cytoskeleton and promo

itial to the formation of functional clathrin-coated pits. Has a preference for membranes enriche
aratus volume and pH.

a preference for FAHFAs with branching distal from the carboxylate head group of the lipids (
33, PubMed:23332125, PubMed:23474714, PubMed:25043379). In contrast to PARP1 and P/
eraction with ITGB5 and modifying ITGB5 cytoplasmic tail interactions such as with FERMT2 a
n light chain.
me membranes, may act as a downstream effector of Rab proteins recruiting cytosolic proteins

the postsynaptic membrane (By similarity).

tion and cell motility. Acts a tumor suppressor in hepatocellular carcinoma cells.
d by RefSeq, Aug 2013]

).

tein lacks the metalloproteinase and disintegrin-like domains, which are typical of the ADAMTS

ycan/BGN and fibronectin/FN1. Inhibits signaling mediated by TGF-beta family proteins possib
.
. This gene is one of several chemokine-like factor genes located in a cluster on chromosome
inus, separated by a large body of repeats. The N-terminal PSD-95/Discs-large/ZO-1 (PDZ)-lik
oid-induced apoptosis. Single nucleotide polymorphisms in this gene are associated with a dec

spindle organization (PubMed:19734894, PubMed:28370826). Necessary for radial and tange

ring early development. Also required for proper mitosis progression.

creases affinity for ACh of alpha-4:beta-2-containing nAChRs. May play a role in the intracellula
F2A and MEF2C. Also repressses expression of proinflammatory cytokines such as TNFA and
elial tight junctions.

ressed in transformed cells, may promote mesenchymal to epithelial-like changes and inhibit c
lin. This gene was identified as a potential candidate gene for obsessive-compulsive disorder i

ntenance of stem cells in the central nervous system (By similarity).

on and maintenance of odontoblasts as well as in dentin formation.

ulum maintenance during interphase and in their reassembly at the end of mitosis (PubMed:17
y).
liates the ubiquitination and subsequent proteasomal degradation of target proteins. Recognize

inding partners from the cytosol to the plasma membrane. Supports adhesion of cerebellar neu

vay activation in response to UV irradiation.
n suppression of Ras-induced cellular transformation and Ras-mediated activation of ELK1. Pla

nd promoting the degradation of SH3RF1, a scaffold protein that is required for pro-apoptotic J

y function as an inhibitor of transactivation by HIF1A by disrupting HIF1A interaction with CRE

the TNF-alpha-induced signaling pathway (By similarity).

ı white adipose tissue.
20721, PubMed:30624931). Phosphorylates inositol 1,4,5-trisphosphate (Ins(1,4,5)P3) first to i

es the neoplastic transforming activity of CTNNB1 (By similarity).

ɔred proteins mainly found in cells associated with the immune system. The SRCR family is de
ər and Thr residues with very low efficiency (in vitro). Acts as MAP2K1/MEK1-dependent allost
l protein has been found in lysosomal membranes, where it can transport catabolites from the l
s (ADAMs), NADPH oxidases (NOXs) and phosphoinositides. Acts as an organizer protein that
arity). Acts as a bone morphogenetic protein (BMP) coreceptor that potentiates BMP signaling
(By similarity). Does not exhibit calcium-activated chloride channel (CaCC) activity (PubMed:22
PubMed:16940153, PubMed:18945876, PubMed:20207939, PubMed:20089856). Ceramides, s
ɔr threonine residue on the protein receptor.

ɜ the kinase the ability to determine its own scaffold to control the effects of their kinase activitie
ducing effect. All 'z' splice variants (z+) of this fragment also show an increase in the number of

modeling in response to extracellular stimuli, cell motility and adhesion, receptor endocytosis, a
ı. Probably involved in vesicular trafficking via its association with the CART complex. The CAR
ɜ kinase activity. Plays an important role in cellular energy homeostasis and in adenine nucleoti
9073142, PubMed:19766732, PubMed:23416111, PubMed:24767988). Efficiently phosphoryla
vay of alcohol metabolism. Two major liver isoforms of aldehyde dehydrogenase, cytosolic and

for the formation of T-tubules, tubular invaginations of the plasma membrane that function in c
ndrial inner membrane.
ium ions with high affinity. May be involved in heat-stress response. Inhibits PCSK9-enhanced
ium ions with high affinity. May be involved in heat-stress response.

f caspase-3 and apoptosis. This activation requires ATP. Isoform 6 is less effective in inducing
inery. May modulate processing of the amyloid-beta precursor protein (APP) and hence format
e complex with the gamma-secretase-derived amyloid precursor protein (APP) intracellular dom
act with cellular G-protein signaling pathways. May bind to the DNA 5'-GTCACATG-3'(CDEI bo
n vitro, can reduce Cu(2+) and Fe(3+) to Cu(+) and Fe(2+), respectively. Amyloid-beta protein 4
s. Serves as a microtubule-dependent signal that is required for the myosin contractile ring form
ssed Rhob and Rhog proteins, both of which show a growth-regulated expression in mammalia
vesicles. Guides the V-type ATPase into specialized subcellular compartments, such as neuro
uired for efficient replication of a subset of genomic loci. Binds to DNA tandem repeat sequenc
nd coordinates a diverse range of cellular pathways such as DNA damage repair, ubiquitination
t contains multiple copies of three enzymatic components: branched-chain alpha-keto acid dec
on, the N-terminal region of the gene product is disrupted, which is thought to be related to the
itochondrial tubular networks, respiratory chain assembly and formation of the LETM1 complex
cades via the heterodimeric receptor formed by NGFR and SORCS2 (PubMed:24908487, Pub
Med:28935373). Involved in altering the calcium regulation of MIP water permeability (PubMed:
alciparum isolates 3D7, Dd2, 7G8 and HB3 (PubMed:22080952, PubMed:26195724). Binding
ering nascent virions to the membranes of infected cells. Acts as a direct physical tether, holdi

quired for axonal growth during development and for retrograde synaptic signaling at mature sy
nd signal transduction.
een proposed to be involved in muscle protein degradation in living tissue.
well as lamins. Overexpression promotes programmed cell death.
growth of cells including T-cells, B-cells, myeloid leukemia cells, melanoma cells, mastocytoma
es through recognizing the core consensus binding sequence 5'-TGTGGT-3', or very rarely, 5'-
important role in memory formation and synaptic plasticity in the hippocampus (By similarity).
cting cells; cadherins may thus contribute to the sorting of heterogeneous cell types. May play
RB/RB1 and NPM1. Interacts with D-type G1 cyclins during interphase at G1 to form a pRB/RB
interact with cyclins D and to phosphorylate the retinoblastoma protein.||Capable of inducing c
tinoblastoma protein RB.

effector of EPHA4 in axon guidance.

ıcidification of the endosome lumen. May play an important role in renal tubular function.
ɔ339). Prevents stress-induced aggregation of blood plasma proteins (PubMed:11123922, Pub
endothelial cells. Also component of the endothelia of blood vessels. Necessary for migration a
and the regulatory B-type carboxypeptidase subfamilies. Carboxypeptidase D has been identifi

lement fixation through its calcium-dependent binding to phosphorylcholine. Can interact with D

eins under a wide range of stress conditions.
e activity.
s. Mediates heparin- and divalent cation-dependent cell adhesion in many cell types including fi
is linked to the actin filament network, and which seems to be of primary importance for cadher
which leads to APP degradation (PubMed:27333034). Involved in the pathogenesis of several d
peptides (By similarity).

nerization at immunological synapses (IS) and for the recruitment of the chemokine receptor CX
protein complex which accumulates at the neuromuscular junction (NMJ) and at a variety of sy
leaving long flaps that escape FEN1: flaps that are longer than 27 nucleotides are coated by re
K/CRKL regulation of epithelial and endothelial cell spreading and migration on type IV collagen
ts as a positive regulator of T-cell coactivation, by binding at least ADA, CAV1, IGF2R, and PT
s.
and promotes angiogenesis. Inhibits MMP9 proteolytic activity.
of mRNA in presence of EIF-4F and ATP. Promotes the ATPase activity and the ATP-depend
eleton, integral membrane channels and membrane-associated guanylate kinases.
etin-induced erythroblast proliferation and differentiation. Upon EPO stimulation, EPOR dimeriz
he imperfect 5'-AGGTCA-3' direct or inverted repeats with various spacings which are also reco

provides a critical bridge between the FA complex and FANCD2.
a beta-oxidation (PubMed:24269233, PubMed:22633490). Preferentially uses palmitoleate, ole
PubMed:22633490). Required for the incorporation of fatty acids into phosphatidylcholine, the m
beta-oxidation (PubMed:24269233, PubMed:22633490, PubMed:21242590). Preferentially ac
with elastin or in elastin-free bundles. Fibrillin-2-containing microfibrils regulate the early proces
NA replication, cleaves the 5'-overhanging flap structure that is generated by displacement synt
nbryonic development, cell proliferation, differentiation and migration. Required for normal mes

s. May be involved in epidermal cell adhesion and epidermal structure and function.
ent of embryonic vasculature, the regulation of angiogenesis, cell survival, cell migration, macro
arbohydrate moieties of glycoproteins.

classic ligand-driven signal transduction cascades, resulting instead in chemokine sequestratic
). Has highest activity on alpha-1,4-linked glycosidic linkages, but can also hydrolyze alpha-1,6-
eratan sulfate, and chondroitin 6-sulfate. Sequence alterations including point, missense and r
'-AGATAG-3'. Required for the T-helper 2 (Th2) differentiation process following immune and ir
ed mucin-type core 2 O-glycan. Mucin-type core 2 O-glycans play an important role in leukocyte
las no beta-galactosidase catalytic activity, but plays functional roles in the formation of extrace
gh its pyridoxal phosphate cofactor; CO(2) is released and the remaining methylamine moiety i

le in maintaining acid-base homeostasis. Regulates the levels of the neurotransmitter glutamat
. Deficiency of this enzyme results in the accumulation of undegraded substrate and the lysosc
pithelial cell line A431 in culture.||[Granulin-7]: Stabilizes CTSD through interaction with CTSD

l regulation of transcription factors and microtubules, by phosphorylating and inactivating glyco
nation of novel hepoxilin regioisomers (PubMed:21046276).
king specific signal responsive activator complexes. Promotes the formation of stable high-ord
3135828, PubMed:1550553, PubMed:29915090, PubMed:30850536). The mitochondrial beta-c
3135828, PubMed:29915090, PubMed:30850536). The mitochondrial beta-oxidation pathway is
tylation gives a tag for epigenetic repression and plays an important role in transcriptional regu
red for de novo or maintenance DNA methylation. May control silencing of the imprinted CDKN
on of over 40 genes, including erythropoietin, glucose transporters, glycolytic enzymes, vascula
PB1/polymerase basic protein 1 (VSDGGPNLY), HAdV-11 capsid L3/hexon protein (LTDLGQI
ue at the C-terminus (PubMed:7743181). Presents a long peptide (APRGPHGGAASGL) derive

by biliverdin reductase. Under physiological conditions, the activity of heme oxygenase is high
5'-UTR of mRNA and modulates the stability and the level of translation of bound mRNA mole
ntities on the anterior-posterior axis.
SD activity. Uses NADH while EDH17B3 uses NADPH.
olytic and antidepressant drugs and other psychoactive substances, such as lysergic acid dieth
on factors by forming heterodimers and inhibiting their DNA binding and transcriptional activity.

PubMed:10049744, PubMed:14532120, PubMed:15337770, PubMed:21854986). Functions i
615558). Ligand binding stimulates activation of the JAK/STAT signaling pathway (PubMed:81
ng phosphomannosyl residues bind specifically to mannose-6-phosphate receptors in the Golg
the IGFs on cell culture. They alter the interaction of IGFs with their cell surface receptors. Pro
the IGFs on cell culture. They alter the interaction of IGFs with their cell surface receptors. Als
the IGFs on cell culture. They alter the interaction of IGFs with their cell surface receptors.

| Th2 differentiation. The IL4/IL13 responses are involved in regulating IgE production and, che
e). Upon binding to IL18R1 and IL18RAP, forms a signaling ternary complex which activates N
otor for fibronectin. It recognizes the sequence R-G-D in its ligand.

elopment, neural tube fusion process and hematopoiesis. Acts by modulating histone methyltra
brain astrocytes, but also in kidney and in other tissues (PubMed:15820677, PubMed:2165322
microtubules and to DNA (By similarity). Plays a role in congression of laterally attached chrom
ral integrity of epithelial cells and are subdivided into cytokeratins and hair keratins. Most of the
of striated muscle.

orylation may also serve to regulate actin-associated ion transport activities, not only in the pari
phosphorylation of cofilin (CFL1).||Serine/threonine-protein kinase that plays an essential role in

2) to produce Man(5)GlcNAc(2).
osidic linkages.
arides.
and vasoactive amines in the central nervous system and peripheral tissues. MAOA preferentia

stiffening effect on microtubules.

and the promotion of mucosal healing. Promotes VEGF-dependent neovascularization (By sim
elial cells and angiogenesis, at least in part by promoting the release of the growth factor FGF2

68535). Biologically important in the destruction of opioid peptides such as Met- and Leu-enkep
stress signaling with activation of the pro-inflammatory NF-kappaB, NFAT and IRF transcriptio

r thiosulfate biosynthesis. Acts as an antioxidant. In combination with cysteine aminotransferas
cell spreading, plays an important role in cytoskeleton reorganization, focal contacts formation
d with LARP6 in the stabilization of type I collagen mRNAs for CO1A1 and CO1A2. During cell

ein trafficking, and especially in the transfer of cargo proteins from early to recycling endosomes
s (By similarity). Myosin 6 is a reverse-direction motor protein that moves towards the minus-en
of replication of adenovirus type 2. These proteins are individually capable of activating transcr
ents that are initiated by a vast array of stimuli related to many biological processes such as infl
rmination. Upon ligand activation through the released notch intracellular domain (NICD) it form
es playing a key role in cardiovascular homeostasis. Has guanylate cyclase activity upon bindir
ollagen and regulates cell differentiation, remodeling of the extracellular matrix, cell migration ar

e nucleotide exchange factor which binds to and activates alpha subunits of guanine nucleotid

› inhibitor which inhibits thrombin, neuropsin and chymotrypsin but not trypsin, tissue type plasr
›th Gbeta-Ggamma and RGS-Gbeta5 heterodimers.||[Isoform 2]: Acts as a negative regulator c
d-BH2. Coactivator for HNF1A-dependent transcription. Regulates the dimerization of homeod
:tions in the brain.
sus motif. Likely functions in the constitutive and regulated secretory pathways. Plays an esser

)P to NADPH.

›lysis-independent signal transduction activation effects of U-PA. It is subject to negative-feedb
; required for normal assembly and cross-linkling of collagen fibrils (By similarity). Forms hydro:
ıanced by the permanent PML-NB-associated protein DAXX or by the recruitment of p53/TP53
cle types. The protein binds to an A/T-rich element in the muscle creatine enhancer (By similar
:erol-3-phosphocholine (16:0/18:1-GPC). Activated by oleylethanolamide, a naturally occurring
)-mediated signals (PubMed:15671020, PubMed:18838687, PubMed:19154138, PubMed:2346
›olymerase-primase complex) which play an essential role in the initiation of DNA synthesis (Pu
lar energy metabolism. In response to reduction of intracellular ATP levels, AMPK activates en
enzyme after the cAMP-induced dissociation of its regulatory chains.
egative regulation of cell proliferation, apoptosis, differentiation, migration and adhesion, tumor
›ts that may include CREB, SMAD6 and PKD1 and has multiple functions in cellular differentiat
›nal myelin sheath maintenance. May promote myelin homeostasis through acting as an agoni:
event coagulation and stimulating fibrinolysis.
s a Gla domain at the N-terminus, preceded by a propeptide sequence required for post-transl
ıents further induce synovial cells to up-regulate MMP1 and MMP3 production. May also degra
R37 and GPR37L1, undergoing ligand-mediated internalization followed by ERK phosphorylati
integral membrane proteins such as Notch receptors and APP (amyloid-beta precursor protein
ınd Glu adjacent to the leaving group at neutral or slightly basic pH. The proteasome has an A
eneration of class I binding peptides by altering the cleavage pattern of the proteasome.
ergetic metabolism by promoting COX activity and ATP production, via collaboration with isofor
:alcium second messenger system. May play a role as an important modulator of renal function
the affinity constant. Functional coupling with the prostaglandin F2-alpha receptor seems to oc
expression can attenuate the nuclear accumulation of LPXN and limit its ability to enhance ser
ll migration, cell growth and also regulates TGF-beta gene expression, thereby modulating epit
r of EGFR signaling pathway. Forms complexes with beta-catenin and gamma-catenin/plakogl
ation of 7,8-dihydroneopterin triphosphate into 6-pyruvoyl tetrahydropterin.

ases. Interacts and acts as an effector molecule for R-Ras, H-Ras, K-Ras, and Rap.

:26503625). Shows phospholipase A1 (PLA1) and A2 (PLA2), catalyzing the calcium-indepenc
ak (DSB) repair through the homologous recombination (HR) pathway. HR is restricted to S anc

erentiation of nodal monocilia and left-right asymmetry specification during embryogenesis. Rec
into their inactive GDP-bound form. Down-regulates G-protein-mediated release of inositol pho
g(0)-Leu-enkephalin, Arg(0)-Met-enkephalin and Arg(-1)-Lys(0)-somatostatin-14. Can hydrolyz
s filaments and cell contractility. Plays an important role in photoreceptor integrity. May play a c
d stress-induced activation of the transcription factors CREB1, ETV1/ER81 and NR4A1/NUR7

22660413).
cytoskeleton and the extracellular matrix.

bMed:10391915, PubMed:10574970, PubMed:11311135). The precise substrate specificity de
gulation of cytoplasmic Ca(2+) levels and Ca(2+)-dependent cellular processes (PubMed:1374
d cell volume.
. Component of SWI/SNF chromatin remodeling complexes that carry out key enzymatic activit

P40. Stabilizes the ADP state of HSC70 that has a high affinity for substrate protein. Through it
kines and other growth factors. Following type I IFN (IFN-alpha and IFN-beta) binding to cell su
and estrone (PubMed:7779757, PubMed:11884392, PubMed:11006110). Is a key enzyme in es
egulation of lymphocyte migration by mediating phosphorylation of ERM proteins such as MSN
release of soluble JAM3 from endothelial cells surface (PubMed:20592283). Responsible for t
(PubMed:14980219). Plays an essential role in transcription activation mediated by nuclear re
progression.

ively. Plays a role in cell protection against oxidative stress by detoxifying peroxides and as ser
C[CG]CGC-3', found in the promoter region of a number of genes whose products are involved
uous receptor for the TGF-beta cytokines TGFB1, TGFB2 and TGFB3. Transduces the TGFB
nctional adhesion molecules, and occludin to the actin cytoskeleton (PubMed:7798316, PubMe
are cell-surface proteins that are characterized by the presence of four hydrophobic domains. T
The TRAF1/TRAF2 complex recruits the apoptotic suppressors BIRC2 and BIRC3 to TNFRSF
ways to the cytoskeleton.
strand breaks.
d cell type. Involved in cell cycle regulation as a trans-activator that acts to negatively regulate
Defects in this gene are a cause of sudden infant death with dysgenesis of the testes syndrom
preventing their binding to membrane lipids (By similarity).
s cyanide, can act as sulfur ion acceptors. Also has weak mercaptopyruvate sulfurtransferase
and a reductase reaction.

sides or purine ribonucleosides. Can use ATP or GTP as a phosphate donor. Can also phosph
gulated by proteolysis.
L. May participate in regulating the subcellular localization of WASL, resulting in the disassemb
sites overlapping the transcription start site. Binds to the consensus sequence 5'-CCGCCATN

by mediating ubiquitination of DDX58 and IFIH1 (PubMed:17392790, PubMed:30193849). Me
nosphorylation of AKT1 at 'Ser-473'.
ular Reelin signal to intracellular signaling processes, by binding to DAB1 on its cytoplasmic tail
a non-exchangeable site on the alpha chain.
actor Xa. Has no effect on thrombin.
translation of certain mRNAs (By similarity).
has been found, but its biological validity is not determined. [provided by RefSeq, May 2010]

and MCM3.

he acrosome reaction in the sperm. Involved in follicular atresia, the breakdown of immature ov
of EIF2S1 phosphorylation by promoting its dephosphorylation by PP1. Plays a role in ELK1-d
rate.

PubMed:11825900). Contributes to the regulation of nerve signaling, and prevents neuronal hyp
quent viral fusion and release of viral contents into the cytosol. Active against multiple viruses,
ependent manner. May be required for efficient terminal myeloid maturation of hematopoietic ce
bound form. Binds selectively to G(z)-alpha and G(alpha)-i2 subunits, accelerates their GTPase
hosphate esters including phosphatidate/PA, lysophosphatidate/LPA, sphingosine 1-phosphate
at are subsequently sensed by TLR8 (PubMed:31778653). Cleaves preferentially single-strand
tions in the brain.
hrenequinone (PQ) and the oxidation of 9-alpha,11-beta-PGF2 to PGD2. Functions as a bi-dire
ed radial glial cells (RGCs), by allowing their daughter cells to choose progenitor over neurona
Med:10988299, PubMed:11739745, PubMed:16076287, PubMed:19759537, PubMed:214788

nvasion and metastasis.
ypic intraepithelial cell/T-cell interactions and homotypic T-cell aggregation. Inhibits beta-1 integ
rmation by the adenovirus E1A protein. The transcriptional control activity of cell growth require
affinity receptor for semaphorins 3C, 3F, VEGF-165 and VEGF-145 isoforms of VEGF, and the
be a negative regulator in the growth hormone/IGF1 signaling pathway. Probable substrate re
bound retinal. Can metabolize octanal and decanal, but has only very low activity with benzalde
e. Acts also as an ERK downstream effector mediating survival. As a member of the NUPR1/R
and cell spreading. Promotes targeting of RAC1 to focal adhesions (By similarity). May functio

:tivation. May play a role in src signaling pathway.

e first step is the transfer of a sulfate group to ATP to yield adenosine 5'-phosphosulfate (APS)

complexed to cyclins. Mediates phosphorylation of CDK1 predominantly on 'Thr-14'. Also invo
mulation recruits MYD88, IRAK1, IRAK4, and TRAF6, followed by phosphorylation of MAPK3/E
ds to the histone H1 promoter and stimulates transcription. Was first identified as gene weakly :
such as lactate, pyruvate, branched-chain oxo acids derived from leucine, valine and isoleucine
GTP. Plays a role in the epithelial polarization (By similarity).
a signaling pathway mediated by PRKCA (PubMed:21724588).||Receptor involved in cell adhes
PubMed:3099851, PubMed:1551920, PubMed:24498430). LacCer is the starting point in the bi
nificant role in TGF-mediated signaling by regulating the subcellular location of SMAD2 and SM

enes. Mediator functions as a bridge to convey information from gene-specific regulatory protei
or of the amyloid-beta A4 precursor protein (APP) processing leading to a strong reduction in th

'3 and STAT1. Involved in the regulation of Th1-type immune responses. Also appears to be in
ys a major role in pH regulation in neurons. May be involved in cell pH regulation by transportin
y. May act as regulator of neuropeptide activity. Plays a role in the antigen-processing pathway
es and catalyzes their disassembly, possibly in combination with membrane fission. Redistribute
etylation and the formation of repressive chromatin structures which may impede the access of
form methionine-sulfoxide, resulting in actin filament disassembly and preventing repolymerizat
ted upon phosphorylation by CaM-kinase kinase. Promotes apoptosis.
he actin cytoskeleton, possibly by transferring signals from the cell surface in a sugar-depender
d GDP for free GTP in a cAMP-dependent manner. Serves as a link between cell surface rece
This coupling may increase the local Ca(2+) concentration at the site of collagen synthesis, and
and lymphoid tissue organogenesis (By similarity). Binds to GC-rich DNA sequences in the pro
lecules in umbilical vein endothelial cells (HUVECs) cultured in THP1 monocyte LPS-induced r
teraction with Atg16L1 (PubMed:23392225). Required for the formation of the autophagosome
ogression of DNA replication forks. GINS complex seems to bind preferentially to single-strande
)2).
nt and cytokinesis. The BCR(KLHL21) E3 ubiquitin ligase complex regulates localization of the
ell differentiation and myelination.
ction. Vaults may also play a role in nucleo-cytoplasmic transport. Down-regulates IFNG-media
etylation gives a tag for epigenetic repression and plays an important role in transcriptional regu
roteins including huntingtin. Suppresses aggregation and toxicity of polyglutamine-containing, a

aspartate across the mitochondrial inner membrane (PubMed:11566871, PubMed:25410934).

d 3-alpha-androstanediol to dihydroxyprogesterone (PubMed:11294878, PubMed:29541409). F

ation, growth, proliferation or cell survival. Activation by various effectors including growth factor
s of the neuronal silencer REST and induces the activation of neuronal-specific genes. Involve
ınsubstituted terminals into amino acids. May play a role in the liberation of thyroxine hormone

nulating hormone (FSH).
ansmitter release by acting in synaptic vesicle priming prior to vesicle fusion and participates in
crofibrils and ELN (PubMed:18185537). Stabilizes and organizes elastic fibers in the skin, lung
. Involved in formation of actin stress fibers and focal adhesions (By similarity).
quent viral fusion and release of viral contents into the cytosol. Active against multiple viruses,
iated via its action on PABPC1. Competes with PAIP2 for binding to PABPC1. Its association w
-strand break repair. Functionally cooperates with PALB2 in promoting of D-loop formation by I
llular processes. Isoform 1, isoform 2 and isoform 3 phosphorylate CAMK1 and CAMK4. Isofor
cting cell death.||Guanine nucleotide-binding protein that plays a crucial role in the cellular resp
n of the signaling initiated by the G protein coupled receptors (GPCRs) by accelerating the GTI
ɔsis and may play a role in the trafficking and distribution of IF proteins and other cellular factor

e activated by a variety of naturally occurring or synthetic FMRF-amide like ligands. This recep
ic acid to increase water solubility and enhance excretion. They are of major importance in the
eracts with cellular components, and possesses weak ATPase activity. May be a chaperone-lik
olded non-glycosylated proteins as well as improperly folded glycoproteins, retain them in the E
in abundance through the cell cycle. Cyclins function as regulators of CDK kinases. Different c

ose (GDP-Man) pooled in the cytosol into the lumen of the Golgi in exchange for the correspon
877).
er ovulation was induced. Alternative splicing results in multiple transcript variants. [provided by

ackaged into autophagosomes and delivered to lysosomes for degradation (PubMed:2343508
 for GTP nucleotide exchange ability (PubMed:28625565). Involved in ciliary assembly by bind

31159).

r.
s. Catalyzes the formation of the GlcNAcbeta1-4 branch on the GlcNAcbeta1-2Manalpha1-3 ar
apping the barbed ends of filaments, it also regulates motility. Seems to play an important role
y.
APP, stabilizes APP metabolism and enhances APBA2-mediated suppression of beta-APP40

on (PubMed:21706167). In addition, has a role in translesion DNA synthesis. Requires for UV-i
} heterodimer to form a heterotrimeric KIF3 complex and may regulate the membrane binding c
). Rab3 proteins are involved in regulated exocytosis of neurotransmitters and hormones. Spec
wards PGE1, PGE2 and PGE2-alpha (By similarity). Catalyzes the conversion of leukotriene B4
ition reaction rather than in the elongation step (By similarity). Required for CCSAP localization
equired for transcriptional activation by DNA binding transcription factors of genes transcribed b
8042621). Required for localization of CDK5RAP2 to the centrosome during interphase (PubM
EIF4E-FMR1 complex this subunit is an adapter between EIF4E and FMR1. Promotes the tran
nt degradation of core histones during spermatogenesis and DNA damage response. Recogni
roprotective function. May play a role in neuronal rapid ischemic tolerance.

ts in multiple transcript variants. [provided by RefSeq, Mar 2014]
in-like complex.

O), influencing both its stability and activity in leukotrienes synthesis.
GRK1 (RHOK), but not GRK2 to GRK5. Can substitute for calmodulin (By similarity). Stimulates

AIT (gamma interferon-activated inhibitor of translation) complex which mediates interferon-gar
tion of RAC1 (PubMed:25074804, PubMed:25925950). Increases the presence of activated RA
CBLC, modulates the rate of RET turnover and may act as regulatory checkpoint that limits the
53/TP53, CDKN1A and TERT. Keeps cells alive by suppressing p53/TP53 under normal condi
cytoplasm in response to TGF-beta. Acts as an adapter that mediates the specific recognition

s via the degradation of nucleic acids, by reducing the concentration of ssDNA able to stimulate

nselves (PubMed:8932385). However, they seem to serve as transcriptional activators by dime

BXL2), which mediates the ubiquitination and subsequent proteasomal degradation of target p
has ATPase activity; the force-producing power stroke is thought to occur on release of ADP. F
to the regulation of dendritic spine morphogenesis (By similarity).
exocytosis. May regulate V-ATPase intracellular transport in response to extracellular acidosis
sperm polarity and normal spermatid adhesion. May also promote integrity of Sertoli cell tight ju

similarity). May be involved in apoptosis regulation. Necessary for signal transduction through th
ly involved in the transition from an active state to a repressed state in embryonic stem cells: a

proteasomal degradation of target proteins. The Cul7-RING(FBXW8) complex mediates ubiquit

-dependent degradation of key sarcomeric proteins, such as alpha-actinin (ACTN2) and filamin
3708001, PubMed:23708605, PubMed:16921029). During mitotic cell cycle plays a role as bot

for early limb outgrowth and patterning in maintaining the FGF4-SHH feedback loop. Down-re

on of cardiac genes. Induction seems to be correlated with apoptotic cell death in hepatoma ce
ynthesis occurs within the dendrite, the localization of specific mRNAs to dendrites may be a pr
e required for pluripotency and decreases expression of differentiation-associated genes. Has c
rT3, and of estrone-3-sulfate and taurocholate.

es transport of tubulin within the cilium. Binds tubulin via its CH (calponin-homology)-like region
on of the short actin protofilament, which in turn defines the geometry of the membrane skeleto

factors, such as ARHGEF6. Is involved in the reorganization of the actin cytoskeleton and forn
by cGAMP (PubMed:25299331). The function in innate immune response is probably indepen
esses, such as alternative splicing of mRNAs and X chromosome inactivation mediated by Xist

utes the predominant proteoglycan present in cartilage and is distributed on the surfaces of ma
(By similarity). Does not exhibit calcium-activated chloride channel (CaCC) activity (PubMed:22
AMP phosphotransferase and ITP:AMP phosphotransferase activities.
d NF-kappa-B pathways. Following viral infection, UBXN1 is induced and recruited to the RLR c

toward histone H4 (PubMed:16387653). Through chromatin acetylation it may function in DNA
yltransferase activity in vitro.

A, and regulates its levels by promoting mRNA decay. RNA binding is sufficient to prevent tran

2 (PubMed:18056426).||Component of the eukaryotic translation initiation factor 3 (eIF-3) comp
cellular energy metabolism. In response to reduction of intracellular ATP levels, AMPK activate
s from the endoplasmic reticulum (ERAD).
nhibits membrane ruffling. Inhibits actin filament depolymerization. Bundles actin filaments, del
anonical Wnt signaling activity in neural stem cells (By similarity). Required for DNA damage-in

n and subsequent proteasomal degradation of the transcription factor HBP1 (PubMed:2991197
Ca(2+) elevation after endoplasmic reticulum Ca(2+) refilling, promotes a slow inactivation of S
or of the non-canonical Wnt signaling pathway, acting downstream of WNT5A, MAP3K7/TAK1
MO25 (CAB39/MO25alpha or CAB39L/MO25beta) and STK11/LKB1.

an anti-recombinase to counteract toxic recombination and limit crossover during meiosis. Regu
Peptide trimming is essential to customize longer precursor peptides to fit them to the correct le
tic beta cells (PubMed:21853325). Is a downstream transcriptional target of p53/TP53, and act
enes and is involved in processes like cell differentiation, cell migration and cell survival. Transa
A export, the cap binding complex (CBC)-bound snRNA on the one hand and the GTPase Ran
eins as substrates. It can phosphorylate a large number of proteins. Participates in Wnt signalir
om UDP-alpha-D-galactose to lactosylceramide/beta-D-galactosyl-(1->4)-beta-D-glucosyl-(1<->
g ERBB2, ERBB3 and ERBB4. Inhibits EGFR catalytic activity by interfering with its dimerizatior

perties and mechanical stability of tight junctions (By similarity). Has no peptidase activity (PubM

ith RAB11A, RAB3IP, RAB8A, PARD3, PRKCI, ANXA2, CDC42 and DNMBP promotes transcy
ition of primary endothelial cell migration by Slit proteins (By similarity). Involved in the mainten
utes to the sonic hedgehog (SHH) pathway regulation. May play a role in neurite outgrowth. Ma
a negative regulator of PAX6 expression (PubMed:26394807).
:14988505). May also play an important role in early urinary-tract development (PubMed:3105
PINK1/PRKN-mediated mitophagy in response to mitochondrial depolarization.
nor 9-cis, 11-cis or 13-cis-retinol isomers. May play a role in the metabolism of vitamin A. Indep

d 'Lys-48'-linked polyubiquitination. May be involved in degradation of katenin.
horylate p44-ERK1 (MAPK3) but not p54 SAPK-beta (MAPK10) in vitro. Able to enhance activa

osome. Thereby, plays an important role in the splicing of cellular pre-mRNAs. Most spliceosor
s a class of autosomal recessive, neurodegenerative disorders affecting children. The genes re

l that functions in synchronization with calcium release from intracellular stores.
PubMed:12547821).
ys a role in anchoring the microtubule organizing center to the centrosomes. Binds to DNA. Pla

to proline 3-hydroxylation of collagen COL4A1 and COL1A1 in tendons, the eye sclera and in t
cofactor (PubMed:29335528, PubMed:31127291). In presence of Mg(2+), only shows 3'-5' exo
cysteine residue in E1, yielding a ubiquitin-E1 thioester and free AMP. Specific for ubiquitin, do

s PEG10. Interacts with the -1PRF signal of target mRNA and translating ribosomes and cause
tidylinositol 4-phosphate (PI4P), a lipid that plays important roles in endocytosis, Golgi function
or threonine residue on the protein receptor. Has activity toward Muc5Ac and EA2 peptide subs
evelopment, and in regulating normal cell survival and proliferation (By similarity).

)road-complex, Tramtrack and bric a brac/Pox virus and Zinc finger), both of which are typically
ırly endosome and the delivery to the Golgi apparatus.||Acts as component of the retromer carç
)holipids from the outer to the inner leaflet of various membranes and ensures the maintenancı
role in regulating the actin cytoskeleton and cell shape (By similarity).
LL2 elongation activities. Potent inducer of apoptosis in prostatic and non-prostatic cell lines. I
ctions in the brain.
ctions in the brain.

)articipate in the control of cell cycle progression and genomic stability.
rate-limiting enzyme in the synthesis of cytosine nucleotides.
of non-reducing N-acetylglucosamine (GlcNAc) residues. Preferentially acts on mannose-linked
as an actin nucleation factor and promotes assembly of actin filaments together with SPIRE1 a

PA) by incorporating an acyl moiety at the sn-2 position of the glycerol backbone (PubMed:211

gulates various physiological processes through the generation of approximately 24 hour circa
avirus to the cell surface.||Acts as component of the retriever complex. The retriever complex is
in multivesicular bodies (MVBs) formation and sorting of endosomal cargo proteins into MVBs.

ing pathways via interaction with the complex containing IRAK kinases and TRAF6. Mediates 'l

RALB.
:28939891). Required for the proper biosynthesis and transport of pulmonary surfactant-assoc
ndle checkpoint activity (PubMed:14699129, PubMed:14738735). Required for kinetochore inte
n and the cell membrane. Binds glycerophospholipids in a barrel-like domain and may play a ro
(PubMed:12890487). Involved in the regulation of mitotic metaphase/anaphase transition (Pul
or. May be responsible for cholesterol ester hydrolysis in macrophages, thereby contributing to t
n, a PDZ domain and a C-terminal coiled-coil domain with a leucine zipper. A similar protein in
may regulate neurite outgrowth (By similarity).

h a higher affinity to methylated CpG dinucleotides in the consensus sequence 5'-CGCG-3' but
nate and adaptive immune response triggered by unmethylated cytidine-phosphate-guanosine (
microtubule-associated protein MAPT/TAU (PubMed:14594945, PubMed:23666762). Also ph
may be involved in pathophysiology such as atherogenesis (By similarity). Induces a strong che

to the plasma membrane.

l chord development plays a role in guiding commissural axons probably by preventing prematu
R membranes, with N-terminal DNA-binding and transcription activation domains oriented towa

ed:19168031). Acts efficiently on natural and analog ceramides (C6, C8, C16 ceramides, and C
nation and subsequent proteasomal degradation of the transcription factor HBP1. MAEA and R
f GAD1/GAD67 (By similarity). Has almost no deubiquitinating activity by itself and requires the
the liver by negatively regulating RAS activity and the down-stream ERK signaling pathway.

evelopment (By similarity).
ates ubiquitination and subsequent proteasomal degradation of the transcription factor HBP1 (

sister chromatid cohesion through regulation of the acetylation of the cohesin subunit SMC3.
clock-controlled genes (PubMed:11278948, PubMed:14672706, PubMed:15193144, PubMed:1
he cell to initiate subsequent steps of construction of the central pair apparatus of motile cilia (E
P-GlcUA and N-acetylgalactosamine (GalNAc) from UDP-GalNAc to the non-reducing end of t
bition of YAP1-mediated neuroprogenitor cell proliferation and differentiation (By similarity).
plasmic reticulum and Golgi or organization of the Golgi in cells (By similarity).
DNA cleavage that forms the double-strand breaks (DSB) that initiate meiotic recombination. T
Mainly acts as a transcription repressor that binds DNA independently of DP proteins and speci
TGFB or active TGFB by FBN1 microfibril assembly, thereby negatively regulating the expressio

atty acyl-CoA substrates. Catalyzes the insertion of a cis double bond at the delta-9 position int
osis.
ge of 3 sodium ions per 1 calcium ion (PubMed:20018762, PubMed:22829870, PubMed:23056
fect growth cell capacity. Overexpression seems to mediate many of the known phenotypic fea

7, PubMed:27098453, PubMed:26895380). Acts as a negative regulator in MAPK signaling pat
PIs during GPI precursor assembly. The presence of a fourth mannose in GPI is facultative and
activity is synergistically activated by phosphatidylinositol 3,4,5-trisphosphate and the beta gam
sity, inactivation kinetics and rate of recovery from inactivation in a calcium-dependent and isof

ial cells; the function requires homotrimerization and implicates MAPK signaling.||Endostatin: P
st proximal signaling protein that links T-cell receptor-mediated signal to the activation of c-Maf

). Mediates the cleavage of glycosylphosphatidylinositol (GPI) anchor of target proteins: remov
ination and subsequent proteasomal degradation of PDCD1/PD-1, thereby regulating T-cells-m
ed:26686862). Negatively regulates ADAM10-mediated cleavage of GP6 (By similarity). Promo
presence. Necessary for the terminal differentiation of epidermal cells, the formation of kerato
ired for the formation of stomata of caveolae and transendothelial channels. Functions in micro

yl-transferases dedicated primarily to the cadherin superfamily, each member seems to have a
colipids, notably by participating in biosynthesis of HNK-1 and Lewis X carbohydrate structures
ty). Stimulates both DMC1- and RAD51-mediated homologous strand assimilation, which is rec

nd signal transduction. This small subunit may act as a tissue-specific chaperone of the large s
26438880). Inhibits TNFSF11-induced NFKB1 and JUN activation and osteoclast differentiation

oliferation and NF-kappa-B activation in a T-cell receptor/CD3-dependent manner. Activates NI

nd cross-links (ICLs) by homologous recombination (HR) (PubMed:23401855). Required for DN
junctions. The PARD6-PARD3 complex links GTP-bound Rho small GTPases to atypical prote
e-resistant aggregates. Binds heparin.
s.
N) to sites of AQP2 phosphorylation. Mediates the non-vesicular transport of glucosylceramide
n (G-protein) alpha subunit GNAI3.||Hydrolyzes the phosphatidylinositol 4,5-bisphosphate (PIP

nesis. Upon binding to LGR4-6 (LGR4, LGR5 or LGR6), LGR4-6 associate with phosphorylated
yl-transferases dedicated primarily to the cadherin superfamily, each member seems to have a
ays a role in the establishment and maintenance of neuronal transmission and plasticity. Regula
o initiate ciliation. After specifically capturing the activated GTP-bound RABL2B, the CEP19-RA

way (PubMed:23763432). May be involved in oocyte maturation (By similarity). Also binds deh
RA.

rent sensory organs.
d into the lumen of the endoplasmic reticulum.

uired for developmental angiogenesis (By similarity). In this process, required for microtubule r

jen-associated molecular pattern metabolites (PAMPs) (PubMed:12566447, PubMed:1549222

sitol 1,4,5-trisphosphate (IP3). DAG mediates the activation of protein kinase C (PKC), while IF
ncy and markedly alter the G-protein signaling of the receptors by accelerating onset and prom

teins essential to the formation of functional clathrin-coated pits. Has a lipid-binding activity wit
nmalian Hsp70 family. This gene may be involved in susceptibility to atherosclerosis. Alternativ
by RefSeq, Jul 2008]
embrane domain boundaries (PubMed:16873890, PubMed:25354954, PubMed:25827571). Ac
nilies of signaling molecules. This gene is one of several chemokine-like factor genes located ir

3898). Beta-D-galactose is metabolized in the liver into glucose 1-phosphate, the primary meta

e involved in serine degradation and fructose metabolism. Decreased activity of the encoded e
a cofactor that increases p53/TP53 response via its interaction with p300/EP300. Increases p5

some, robust mitotic spindle formation and cytokinesis. Phosphorylates RPS6KB1.
ne Delta-mediated Notch signaling by ubiquitinating the intracellular domain of Delta, leading tc

ppa-B and interferon-regulatory factor (IRF) activation, and to induce apoptosis (PubMed:12471

ptional activity of CREB3 during the UPR. Recruits CREB3 into nuclear foci.
ansport through regulation of ENaC cell surface expression. Acts as a scaffold protein coordina

protein GJA1. May contribute to the stability and proper localization of GJA1 to cardiac intercal

esis of bone marrow (By similarity). Inhibits fibroblast growth factor (FGF)-induced retinal lens f

vith the SNARE machinery. May play a role in accumulation of expanded polyglutamine (polyQ)
ependent and -independent mechanisms. Exhibits antiviral activity against vif-deficient HIV-1. A
h factor (FGF)-induced retinal lens fiber differentiation, probably by inhibiting FGF-mediated ph
alizes to the cytoplasm of dendrites and is also enriched in the nucleus where it interacts with t

regulate p53 through the enhancement of p53 SUMOylation. Alternative splicing results in mult

lasmic reticulum and translocate inorganic phosphate into the opposite direction. Independent

reticulum away from chromosomes during mitosis. Probably acts by clearing the endoplasmic r
ethylation of Lys residue generates formaldehyde and succinate. May be involved in hormone-c

retinal ganglion cells (RGCs) and promotes synaptic connectivity via homophilic interactions.

volved in NOS1 regulation and targeting to the synaptic membrane.
ter and then directly transfers the ubiquitin to targeted substrates such as LCMT2, thereby pro
/ASL, DNM1 and DNM2. Acts both during interphase and at the end of mitotic cell divisions. Re
ptors, including glucose 1-phosphate, mannose 1-phosphate, ribose 1-phosphate and deoxyrib
ession seen at the embryonic stage and lowest in adult. Knockdown studies in zebrafish sugge

nd food intake. May play a role in the hypothalamic response to stress (By similarity). NPW23 a

1). Plays a role in mother-centriole-dependent centriole duplication; the function seems also to

cing agent (PubMed:12757706, PubMed:15657036, PubMed:15907468, PubMed:25931126, P

amily of genes. The exact function of this gene is not yet known. [provided by RefSeq, Jul 2008
with membranes. Rabs cycle between an inactive GDP-bound form and an active GTP-bound
ERE) present in the promoter of genes such as ANF/NPPA and acts as a direct transcriptional a

) initiate autophagy and antigene presentation to both CD4(+) and CD8(+) T-cells under amino

ngiogenesis acting downstream of Notch at branch points to regulate vascular density. Propose

H), regulating intracellular levels of acyl-CoAs, free fatty acids and CoASH. More active towards

es to regulate mitochondrial quantity and quality by eliminating the mitochondria to a basal leve

protein (SMO) present in the ciliary membrane. Plays a role in sonic hedgehog (SHH)-induced
Med:19969290).

29915238). Endopeptidase that cleaves histones N-terminal tails at the carboxyl side of methyl
ganisms by affecting both the stability and translation of mRNAs. miRNAs are transcribed by R
upregulated in tumors and is implicated in the promotion of cell proliferation. [provided by RefS
ne gamma-carboxyglutamate Gla protein) genes on chromosome 1. Alternative splicing results
rity). Targets TJP1 to cell junctions. In cortical neurons, may play a role in glutaminergic signal t

R2A or ACVR2B). Transduces the activin signal from the cell surface to the cytoplasm and is th

nessenger system. Its effect is mediated by G(q) and G(11) proteins. Nuclear ADRA1A-ADRA1
homotypic interactions. Promotes endothelial cell migration. Inhibits endothelial cell tube forma
y have a broader specificity and oxidize other aldehydes in vivo (PubMed:19296407, PubMed:
s of the brain, the encoded protein is a glycolytic enzyme that catalyzes the reversible aldol clea
ribosomal RNA synthesis including that containing the initiation site sequences of 45S rRNA. C
hinery. May modulate processing of the amyloid-beta precursor protein (APP) and hence forma

/ing a variety of intracellular compartments in eukaryotic cells.
ɘr currents in heart (If) and in neurons (Ih). Can also transport ammonium in the distal nephron.

repair by facilitating cellular responses to DNA damage. It is unclear whether it also mediates t
y promoting assembly of RAD51 onto single-stranded DNA (ssDNA). Acts by targeting RAD51

ally promoting their degradation by the proteasome. Negatively regulates TCR (T-cell receptor
e/threonine protein kinase holoenzyme complexes with the cyclin-dependent protein kinases CD
7-glucosiduronic acid and leukotriene C4 (PubMed:11500505). Also transports sulfated bile sal
5, PubMed:18377927, PubMed:19159229, PubMed:20614893). Has broad substrate specificity
e in the transport of bile. May have a role in monitoring the intrahepatic bile acid concentration.
the mitochondrial inner membrane. Also required for the transfer of beta-barrel precursors from

s association with other signal transducers, can regulate different processes. Together with SC
A cross-link repair and in the maintenance of normal chromosome stability. Candidate tumor su
Enzyme with very low activity responsible for the lysosomal catabolism of galactosylceramide,
rmone involved in regulating postnatal body growth. On ligand binding, couples to the JAK2/ST

e consists of two subunits, a heavy catalytic subunit and a light regulatory subunit. Gamma glut

gh catalytic efficiency with 4-hydroxyalkenals such as 4-hydroxynonenal (4-HNE).
lls that are in terminal stages of differentiation or that have low rates of cell division.
tin, limiting DNA accessibility to the cellular machineries which require DNA as a template. Hist
an synthesis a major component of most extracellular matrices that has a structural role in tissu
spectrum of tissues and cell types. Activating ligand for the receptor tyrosine kinase MET by bin
bundant chromatin-associated non-histone protein involved in transcription, chromatin remodel
ed in the process which maintains transcribable genes in a unique chromatin conformation (By
ntities on the anterior-posterior axis. Acts on the anterior body structures.
n. Exhibits osteogenic properties by increasing osteoblast mitogenic activity through phosphoac
transcriptional activation of the flounder liver-type antifreeze protein gene. Exhibits strong bind
regulating a number of diverse functions such as hypothalamic and pituitary hormone secretion
sponse against DNA and RNA viruses (PubMed:22394562, PubMed:25636800, PubMed:2730
s of KCNMA1, thereby contributing to KCNMA1 channel diversity. Increases the apparent Ca(2
of secretory proteins from the Golgi complex to the cell surface (PubMed:23857769).
nent of early endocytic vesicles (By similarity). Regulates cilium formation and structure (By sim
nverts cholesteryl esters to free cholesterol for steroid hormone production.

e cyclase.
DNA replication initiation and elongation in eukaryotic cells. The active ATPase sites in the MC
DNA replication initiation and elongation in eukaryotic cells. The active ATPase sites in the MC
DNA replication initiation and elongation in eukaryotic cells. The active ATPase sites in the MC
atopoiesis, megakaryocyte lineage development and vascular patterning. May function as a co
specificity.
latinase A. Involved in the matrix remodeling of blood vessels. Isoform short cleaves fibronectin
conversion of precursor Z into molybdopterin by mediating the incorporation of 2 sulfur atoms i
by both heavy metals and glucocorticoids. May be involved in FAM168A anti-apoptotic signalin
he DNA backbone. Possesses both adenine and 2-OH-A DNA glycosylase activities.
ved in catalysis. Complex I functions in the transfer of electrons from NADH to the respiratory c
ved in catalysis. Complex I functions in the transfer of electrons from NADH to the respiratory c

ation pathways as well as in biosynthetic processes such as the vitamin K-dependent gamma-c
isoform 1.||Produces nitric oxide (NO) which is implicated in vascular smooth muscle relaxatio
ole in neurite outgrowth in response to contactin binding. Plays a role in mediating cell-cell con
imilarity).
both mitosis and meiosis. Plays a role, together with DISC1, in the microtubule network formati
nany important physiological processes.
f formylglycinamide ribonucleotide (FGAR) and glutamine to yield formylglycinamidine ribonucl
flop of phospholipids from the cytoplasmic to the exoplasmic leaflet of the apical membrane. P
almodulin.
cogenolysis) by phosphorylating and thereby activating glycogen phosphorylase. May regulate
cosamine from UDP-N-acetylglucosamine to phosphatidylinositol, the first step of GPI biosynth

of GPI biosynthesis.
ate and phosphatidylinositol. Component in the phospho-dependent endocytosis process of G/
actions. In addition, may be responsible for the degradation of glucose-1,6-bisphosphate in iscl
ular RNA processing and degradation events. In the nucleus, the RNA exosome complex is inv
TG-3'. Capable of reversing CTBP1-mediated transcription repression. Auxiliary component of
NA replication (By similarity).
es. Component of RNA polymerase II which synthesizes mRNA precursors and many functiona
ays numerous essential roles within the cell by associating with different regulatory particles. As
884395). The 9-1-1 complex is recruited to DNA lesion upon damage by the RAD17-replicatior

al sequence -XXCC, -XCXC and -CCXX, such as RAB1A, RAB3A, RAB5A and RAB7A.
ng cell nuclear antigen (PCNA) and activator 1. This subunit binds ATP (By similarity).
ng cell nuclear antigen (PCNA) and activator 1.

ng cell nuclear antigen (PCNA) and activator 1. This subunit may be involved in the elongation
ng cell nuclear antigen (PCNA) and activator 1.

rm during DNA replication or upon DNA stress. It prevents their reannealing and in parallel, rec
posed of 4 RNA species and approximately 80 structurally distinct proteins. This gene encodes
nucleotides. Inhibits Wnt signaling.

clusion. Acts additively with RBMX to promote exon 7 inclusion of the survival motor neuron SM
nzymes, transcription factors, neuronal excitability, cell growth, proliferation, survival, migration
lencing affects cell survival and cell cycle distribution as well as decreases CDK1 activity correl
ination and subsequent proteasomal degradation of target proteins involved in cell cycle progre
(PubMed:7914198, PubMed:7521911, PubMed:8857541, PubMed:26690923, PubMed:21123
tor for Feline endogenous virus RD114.||(Microbial infection) Acts as a cell surface receptor for
ısmitter release. Participates as a monomer in synaptic vesicle exocytosis by enhancing vesicle
d:28076346, PubMed:27035939). Associated with sn-RNP U2, where it contributes to the bind
nponent of the spliceosomal U1, U2, U4 and U5 small nuclear ribonucleoproteins (snRNPs), th
rm head. Required for anchoring and organization of the manchette. Required for targeting of S
Plays a minor and redundant role in promoting the expression of calcium channel CACNA1S a

the SNARE (Soluble NSF Attachment Receptor) complex composed of SNAP25, STX1A and V
kly methylates histone H1 (in vitro). H3 'Lys-9' trimethylation represents a specific tag for epiger
te to replicative senescence.
s. Isoforms lacking the N-terminal CTNNB1 binding domain cannot fulfill this role. Binds to the

maintenance of dentin and dental pulp (By similarity). Ligand for CD36 mediating antiangiogen
Acts via MYD88, TIRAP and TRAF6, leading to NF-kappa-B activation, cytokine secretion and t
r with LMNA, in the nuclear anchorage of RB1.||May help direct the assembly of the nuclear lar
and rejoining one strand of the DNA duplex. Introduces a single-strand break via transesterifica
ling to formation of cell membrane protrusions. Isoform 4 enhances the transcriptional activity c
d:7488099). The produced molecules are then utilized as carbon and energy sources or in the
volved in PCNA-mediated translesion synthesis (TLS) by deubiquitinating monoubiquitinated PC
ger RNA-binding protein that destabilizes several cytoplasmic AU-rich element (ARE)-containin

s of centrioles. Required for proper formation and/or maintenance of primary cilia (PC), microtul
ipid family, predominantly localizes in glycolipid- and cholesterol-enriched membrane (GEM) ra
one (PubMed:17978863). The heterotetramer with CBR4 has NADH-dependent 3-ketoacyl-acyl

select genes principally by acetylation of nucleosomal histones H4 and H2A (PubMed:1296372
acids such as phenylalanine, tyrosine, L-DOPA, leucine, histidine, methionine and tryptophan (I
ng DNA in vitro. CAF-1 performs the first step of the nucleosome assembly process, bringing n
neries which require DNA as a template. Histones thereby play a central role in transcription reg

bound form. Binds to G(i)-alpha and G(o)-alpha, but not to G(s)-alpha (By similarity).
Has affinity with GalNAc, GlcNAc, D-fucose, as mono/oligosaccharide and lipopolysaccharides
erolipid and sphingolipid phosphate esters including phosphatidate/PA, lysophosphatidate/LPA

rising from the turnover of glycoproteins and glycolipids.
actions between T-cells and dendritic cells.
26725, PubMed:14528293, PubMed:25261253, PubMed:25500532). Involved in IL18-mediated

ughout normal DNA replication, DNA repair and in the regulation of the circadian clock (PubMe

of pre-ribosomal RNA (pre-rRNA).
enzyme (ICE)-like proteases.

em to the actin cytoskeleton and to regulate their surface expression. Necessary for cAMP-med
ion channels. Can specifically inhibit proton-gated current of ASIC1 isoform 1. Can increase ina
acellular compartments in eukaryotic cells. In aerobic conditions, involved in intracellular iron ho
ination stop codons AGG and AGA.
side-chain initiation step of the polyglutamylation reaction rather than in the elongation step. In
dynein-dynactin and MAD1-MAD2 complexes onto kinetochores (PubMed:11146660, PubMed
ollaboration with CEP97 by capping the mother centriole thereby preventing cilia formation (Pu

ed endocytosis in neurons (By similarity).
iferation and tumor progression. Phosphorylates ATM, CASP6, LATS1, PPP1R12A and p53/T
nositol 3-kinase-related protein kinase (PIKK) family proteins. The TTT complex is involved in t
DP-bound Rab proteins into their active GTP-bound form.
onse to mitotic spindle poisons.
ation and subsequent proteasomal degradation of target proteins, including proteins involved in
0385). Histone deacetylation gives a tag for epigenetic repression and plays an important role
Assembles histone octamers onto replicating DNA in vitro. CAF-1 performs the first step of the

n with retinoic acid receptor (RXR) shifts RXR from its role as a silent DNA-binding partner to a
ne c oxidase. Binds two copper ions and delivers them to the metallochaperone SCO1 which tr

ɔ function in stabilizing the mature sheath.
tivity at the spindle poles in early mitosis. May function as a scaffold promoting the interaction b

idylcholine (1,2-diacyl-sn-glycero-3-phosphocholine or PC) (LPCAT activity). Catalyzes also the
'D88-dependent production of proinflammatory cytokines. Can promote TRIF-dependent produ

s.

n through mitosis and the G1 phase of the cell cycle. The APC/C complex acts by mediating ub
n 18S rRNA. Involved the biosynthesis of the hypermodified N1-methyl-N3-(3-amino-3-carboxy
ome-mediated interkinetic nuclear migration (INM) of neural progenitors (By similarity). Acts as
ein (snoRNP) particles. Required for the biogenesis of box C/D snoRNAs such U3, U8 and U1
otes T-cell receptor-mediated apoptosis.
24960, PubMed:12356910, PubMed:27462074). Required for chromosome alignment, normal
ntext. The PCAF complex could be considered as the human version of the yeast SAGA comp
fibronectin matrix of connective tissues, and subsequent proteolytic activation of lysyl oxidase L
ed:17317665, PubMed:24449906). As a component of the trimeric and tetrameric DNA polymer
the parental centriole cylinder, leading to the recruitment of centriole biogenesis proteins such
yet been determined but it may play a role in development. The chromosomal location of this g
ine pyrophosphate (TPP)-dependent manner. Involved in the phytosphingosine degradation pa

kinetochore at prometaphase.
4ac), thereby regulating various processes, such as gene transcription, protein ubiquitination, ir
double-strand breaks.
and the removal of the same modification from target proteins (de-AMPylation), depending on
omponent of the precatalytic spliceosome (spliceosome B complex) (PubMed:28781166). The
epair (BER) pathways. Through its two catalytic activities, PNK ensures that DNA termini are cc

ular RNA processing and degradation events. In the nucleus, the RNA exosome complex is inv
rminals when ectopically expressed (By similarity).
ane protein KREMEN that promotes internalization of LRP5/6 (PubMed:22000856). DKKs play

in early development. In DNA damage response is required for cell survival after ionizing radiat

ucosamine within specific subregions of intact heparin. Diminishes HSPG (heparan sulfate pro
spatial organization. As a component of the LINC (LInker of Nucleoskeleton and Cytoskeleton

omosomes. The condensin complex probably introduces positive supercoils into relaxed DNA
ular RNA processing and degradation events. In the nucleus, the RNA exosome complex is inv
coactivation of nuclear receptors for retinoid X (RXRs) and thyroid hormone (TRs) in a ligand-

nance of genome stability. The encoded protein may also play a role in telomere repair. A varia
omponent of the precatalytic spliceosome (spliceosome B complex) (PubMed:28781166). The

7A with higher affinity than to IL17F (PubMed:17911633). Binds IL17A and IL17F homodimers
amily members function as impermeable barriers, others mediate the permeability to ions and

ly. Cooperates with chromatin assembly factor 1 (CAF-1) to promote replication-dependent chr

ses histone 3'-end pre-mRNA processing but has no effect on U7 snRNP levels, when overexp
n through mitosis and the G1 phase of the cell cycle. In the complex, plays a role in the release
and tetradecanoyl-CoA/myristoyl-CoA which are the main substrates in the mitochondrial beta-

oxia-induced cell death of transformed cells implicating cytochrome C release and caspase ac
eins into the mitochondrial inner membrane. Also required for the transfer of beta-barrel precu

53/TP53 homeostasis in unstressed cells. Inhibits autoubiquitination of MDM2, thereby enhan

nones, neurotransmitters, drugs and xenobiotic compounds. Sulfonation increases the water s
559337). Corresponds to the sigma-2 receptor, which is thought to play important role in regul

osomes (mitoribosomes) consist of a small 28S subunit and a large 39S subunit. They have a
ely associated with apoptosis, cell cycle arrest, growth inhibition or cell differentiation. The prot

osomes (mitoribosomes) consist of a small 28S subunit and a large 39S subunit. They have a
es, such as CCND1, MYC and E2F1. May play a role in the recruitment or occupancy of CREB
723). May be involved in cell proliferation and survival of hormone-dependent tumor cells. May

mbly of mitochondrial respiratory chain complex I and complex IV as component of the MITRA(

PubMed:30017583). Catalytic subunit of a heterodimer with TRMT112, which catalyzes N5-me
ı and rigidity and in establishing mitotic chromosome architecture (PubMed:14532007). May pr
nked glycosylation of proteins. The addition of glucose residues to the oligomannose core is ne
osomes during meiosis. The complex PSMC3IP/MND1 binds DNA, stimulates the recombinase

hosphate glucose (Dol-P-Glc) onto the lipid-linked oligosaccharide Man(9)GlcNAc(2)-PP-Dol.
ion stress, required for the stabilization of stalled replication forks, the efficient activation of the
of dehydroascorbate.
ıhibitory receptors and are widely involved in the regulation of the immune system. PILRA is tho

owards dGMP, and low activity towards dCMP and dAMP.
es. Component of RNA polymerase I which synthesizes ribosomal RNA precursors.
translation, and for normal assembly of mitochondrial respiratory complexes. Required for norn
(6)A37) in tRNAs that read codons beginning with adenine (PubMed:22912744, PubMed:2880
enes. Mediator functions as a bridge to convey information from gene-specific regulatory protei
and is required for the early maturation steps of the 60S subunit. [provided by RefSeq, Jul 2008
osomes (mitoribosomes) consist of a small 28S subunit and a large 39S subunit. They have a
hate. Participates in stress granule (SG) assembly. May allow ATP production from extracellula
lder of the two centrioles after centrosome duplication. This protein plays a central role in orga
-competent state. In the RAC complex, binds to the nascent polypeptide chain, while DNAJC2
713).||May play an important role in inflammation and regeneration of skeletal muscle. Partly in
and promote the organization of mitotic spindle microtubules around them.

55485, PubMed:29381136). Promotes the insertion of copper into the active site of cytochrome

, PubMed:30454648). Also a component of the MRP ribonuclease complex, which cleaves pre-
teasome maturation. Mediates the association of 20S preproteasome with the endoplasmic ret
oor patient survival in breast cancer. Alternative splicing results in multiple transcript variants. [
in vivo (By similarity). In vitro, stimulates N-WASP-induced ARP2/3 complex activation in the a
smic reticulum (ER) luminal proteins through the recruitment of components of the proteasome

which activates the caspase cascade and leads to apoptosis.
roliferation. Contributes to the stability and delivery of capped primary miRNA transcripts to the
es accelerate the folding of proteins. It catalyzes the cis-trans isomerization of proline imidic pe

· the export of SMO and PTCH1 receptors out of the cilium and the accumulation of GLI2 at the
əs. Component of RNA polymerase III which synthesizes small RNAs, such as 5S rRNA and tF
· be involved in pre-mRNA splicing.

ome c1 subunit of cytochrome reductase. Cytochrome c then transfers this electron to the cytc

amino acids and sodium ions with a stoichiometry of 1:1. May function in the transport of amir

consequently modulates the stability of their targets. As a result, may control many cellular prc
sal body of primary cilium. Component of the tectonic-like complex, a complex localized at the t

ng MAD2 to kinetochores and monitoring tension on centromeric chromatin (PubMed:1721825
y in response to serum starvation, via the NF-kappaB pathway (By similarity).

f RHOA and phosphorylation of ERBB2 at 'Tyr-1248'. Required for normal brain development,

r DNA damage or replication stress. May be specifically required for the ATR-CHEK1 pathway

s a redundant role with MAGOH in the exon junction complex and in the nonsense-mediated d
s-end, that may lead to the suppression of microtubule growth from and attachment to centrosc
Required for mitochondrial fusion and mitochondrial network formation (PubMed:28554942, Pu
etochore proteins, mitotic progression and chromosome segregation. May be involved in incorp
ene encodes a protein containing a lines-like domain. This gene is located on chromosome 15 a
oss-links (ICLs) by promoting FANCD2 monoubiquitination by FANCL and participating in recru

otein-protein and protein-DNA interactions. Several alternatively spliced transcript variants have
eiosis.

ubMed:25678554). Involved in mid-late stages of complex I assembly (PubMed:25678554).
to activity-dependent presynaptic assembly during neuronal development.
yrosine kinases and growth factor receptors. Plays a role in modulating cell spreading and migr
ne formation of 5-taurinomethyl-2-thiouridine (tm5s2U) of mitochondrial tRNA(Lys), tRNA(Glu),
gamma-tubulin ring complexes (gamma-TuRCs) and has critical roles in forming a focused bip
on of RPGRIP1 to the photoreceptor connecting cilium (CC), as well as photoreceptor-specific l
0531387). Inhibits microtubule nucleation from the centrosome. Involved in the regulation of slc
osphorylated, forms a complex with TP53, leading to TP53 destabilization and attenuation of G

erase activity.

egulates Elk-1-dependent gene induction downstream of HRas and MEKK1 (By similarity).

pyrophosphate at a very high rate and dolichyl monophosphate at a much lower rate. Does no

olved in primary cilium formation (PubMed:24867236).
GTP. Its activity is synergistically activated by phosphatidylinositol 3,4,5-trisphosphate and the b
ons as a highly sensitive Ca(2+) sensor in the endoplasmic reticulum which activates both store
ase II-blocking lesions to be rapidly removed from the transcribed strand of active genes. Acts

omponent of the precatalytic spliceosome (spliceosome B complex) (PubMed:28781166). The
geting of the nascent secretory proteins to the endoplasmic reticulum membrane system. Has

d in neuronal differentiation.

e the activity of HIF1A and thus promote glycolysis under normoxic conditions; the function requ

zing and UV irradiation. Adapter protein which binds to BRCA1 and the checkpoint kinase CHE
A2, RAD51 and RAD51 paralogs in a H2AX-dependent manner. May regulate osteoblast prolife
etochore proteins, mitotic progression and chromosome segregation. May be involved in incorp
romosomes. The condensin complex probably introduces positive supercoils into relaxed DNA

MITF to repress transcription of the CTSK and ACP5 promoters via recruitment of corepressors
for the E2-like covalent binding of phosphatidylethanolamine to the C-terminal Gly of ATG8-lik

seems to bind preferentially to single-stranded DNA.
. This gene encodes a nucleolar RNA-associated protein that is highly conserved between spe
damage to promote DNA repair synthesis.
homologous recombination (PubMed:29042561). Serves as a sensor of DNA damage: binds D
teins, mitotic progression and chromosome segregation. The CENPA-NAC complex recruits the

asmic reticulum-bound enzymatic process allows the addition of 2 carbons to the chain of long

ıetabolites and the conversion of the 3-keto group of 3-, 3,17- and 3,20- diketosteroids into thei
etochore proteins, mitotic progression and chromosome segregation. May be involved in incorp
ılpha4-beta2 neuronal acetylcholine receptor (PubMed:16238698).
ear export, and which specifically associates with spliced mRNA and not with unspliced pre-mF
in-like complex.
vever, in association with PAXIP1/PTIP is proposed to function at least in part independently of

or threonine residue on the protein receptor. Displays activity toward mucin-derived peptide sub

teins, mitotic progression and chromosome segregation. The CENPA-NAC complex recruits the

' trimethylation represents a specific tag for epigenetic transcriptional repression by recruiting H

al genome, including genes important for the oxidative phosphorylation machinery.
osome. Thereby, plays an important role in the splicing of cellular pre-mRNAs. Most spliceosor
osome. Thereby, plays an important role in the splicing of cellular pre-mRNAs. Most spliceosor

ariety of mammalian tissues. Mutations in this gene cause L-2-hydroxyglutaric aciduria, a rare a
6, PubMed:20129055, PubMed:24065767). Plays a key role in HBO1 complex by directing KA
nediates to limit DNA crossover formation in cells. Promotes TOP3A binding to double Holliday
ir target areas, and possibly also for other neurons. May also be involved in the maintenance a

r corepressors.
Z/H2AZ1 from its normal sites of deposition, especially from enhancer and insulator regions. N
nbly in non-erythroid cells. The iron delivered into the mitochondria, presumably as Fe(2+), is t
ependent reductase activity towards 9,10-phenanthrenequinone (in vitro) (PubMed:12604216,
emia pathway, after FANCD2 ubiquitination. Involved in the repair of DNA double-strand breaks
roteins, to produce a proper functioning extracellular matrix. Required for extracellular matrix m

scription by RNA polymerase I.
sulfur cluster into extramitochondrial Fe/S proteins (PubMed:23891004). As a CIA complex cor
galactosamine (GalNAc)-beta-1,3-N-acetylglucosamine (GlcNAc)-beta-1,4-mannose) to genera
' (OPA7). Two transcript variants encoding different isoforms have been found for this gene. [p

lity maintenance (PubMed:25931565). The SLF1-SLF2 complex acts to link RAD18 with the SI

ssium-permeable leak-like channel, which regulates lumenal pH stability and is required for aut
b-c1 complex). Plays a role in the modulation of respiratory chain activities such as oxygen co

Med:12123582). Binds to DNA (PubMed:12628190).
in protecting genome stability by resolving diverse forms of deleterious DNA structures origina
n (PubMed:26399832). The BCR(KBTBD8) complex acts by mediating monoubiquitination of N

ein synthesis (PubMed:24948607).

sn-1 position of the glycerol backbone (PubMed:17170135). Also converts LPA into 1,2-diacyl-

hosphate glucose (Dol-P-Glc) onto the lipid-linked oligosaccharide Glc(2)Man(9)GlcNAc(2)-PP

5865). Essential for the generation of the distal portion of new-born centrioles in a CENPJ- and

nesis. The BCR(KLHL9-KLHL13) E3 ubiquitin ligase complex mediates the ubiquitination of AU
15) complex, leading to promote ubiquitination and degradation of splicing factor RBM39 (PubM
enes. Mediator functions as a bridge to convey information from gene-specific regulatory protei
perone, binding to this subunit within the mitochondrial matrix and stabilizing it prior to its trans

in axon guidance in the developing nervous system. Class 3 semaphorins bind to a complex c

ases. Alternative splicing results in multiple transcript variants. [provided by RefSeq, Aug 2011
c mesoderm to specify lineage commitment of cardiomyocyte development.
Med:28388415). May also regulate other G-protein coupled receptors including type-1 angiote

This protein likely functions as a monomer and is predicted to localize to the mitochondrial mat
in-like complex.
-hydroxy-L-proline from the diet and originating from the degradation of proteins such as collag

WAPL which stimulates cohesin dissociation from chromatin. Cohesion ensures that chromos
thway. Upon activation, upregulates the expression of CD80, CD86, CD69 and MHC II on mac
fect of EGR1. Involved in the maturation of snRNAs and snRNA 3'-tail processing (PubMed:28
e mitochondrial inner membrane. May act as a ATP-Mg/Pi exchanger that mediates the trans

phospholipids containing omega-3 long-chain polyunsaturated fatty acids (LCPUFA) and ther

lso repair alkylated DNA containing 1-ethenoadenine (in vitro). Has strong preference for doub
trate recognition subunit of an ECS (Elongin BC-CUL2/5-SOCS-box protein) E3 ubiquitin-prote
(PubMed:24018379).

, and transcytosis of immunoglobulins via their ability to bind immunoglobulin (Ig) constant regi

play the role of both NPC structural components and of docking or interaction partners for tran

t proteins at the endoplasmic reticulum. It is involved in the regulation of signal-mediated trans

n directly transfers the ubiquitin to targeted substrates.
truncation of the protein encoded by this locus has been associated with mirror-image polydac
end at the branch nick. Typical substrates include 3'-flap structures, replication forks and nicke

dle checkpoint activity (PubMed:14738735). Required for kinetochore integrity and the organiz

in the separation of the endolymphatic and perilymphatic spaces of the organ of Corti in the in

orting ATPases preventing calcium efflux from the cell.
n fold and have diverse functions, such as cell adhesion, cell-cell signalling, glycoprotein turno
ne moth, Trichoplusia ni. This family also includes genes in several genomes, including human

equired both for mother-centriole-dependent centriole duplication and deuterosome-dependent

ilator of cell cycle: THAP5 overexpression in human cell lines causes cell cycle arrest at G2/M

Repression may occur through muscle-specific E-box occupancy by homodimers. May also ne

hor the catalytic subunit ALG13 to the ER.
ion of the nuclear envelope implicating EMD, SUN1 and A-type lamina (PubMed:21610090).||I
subsequent proteasomal degradation of target proteins. Promotes the ubiquitination of HDAC1
chromosomes 6, 8, 16, and X. Alternative splicing results in multiple transcript variants. [provi

(VDCC) across the lysosomal membrane. May be involved in smooth muscle contraction.
segregation (PubMed:17093495, PubMed:19289083, PubMed:23085020). Required for timely
segregation (PubMed:19289083, PubMed:19360002, PubMed:23085020). The SKA1 complex

ubMed:24681962). Represses NF-kappa-B transcriptional activity by inhibiting phosphorylatio
ons. Binds to neuropilin (By similarity).

oyl-CoA (hexadecanoyl-CoA; C16:0-CoA) as acyl donor (PubMed:17977534, PubMed:176092

NeuAc-alpha-2,3-Gal-beta-1,3-GalNAc of glycoproteins and glycolipids. ST6GalNAcIII prefers g
modulate the kinase activity of AKT1. Its interaction with PTPRB and tyrosine phosphorylated
ed:21565611). Does not seem to be strictly required for ciliogenesis (PubMed:21565611). Requ
and beta-L-fucose (70.5%), the beta-form is metabolized through the salvage pathway. GDP-L
perturbation of actin filaments, such as after treatment with the actin depolymerizing microbial
ites in pre-tRNA. It cleaves pre-tRNA at the 5' and 3' splice sites to release the intron. The proc

entriole in cycling cells, leading to restrict nucleation of cilia at centrioles. Mediates depolymeriz

ng a heterodimer with CTU1/ATPBD3 that ligates sulfur from thiocarboxylated URM1 onto the

duce nicked duplex products in which the nicks can be readily ligated. Four-way DNA intermed
ller cells to secrete IFNG.||[Isoform 3]: Down-regulates the expression of KLRK1 and stimulate:
olymerization, playing a key role in the fission of tubules that serve as transport intermediates

osine phosphorylation of target MAP kinases.

etochore proteins, mitotic progression and chromosome segregation. May be involved in incorp

the 3'-position adjacent to the anticodon of eukaryotic phenylalanine tRNA. Catalyzes the conc
in the interaction of BCL2 with BECN1 and is required for BCL2-mediated depression of endop
amma-glutamyl peptides.
de a DNA-binding domain of 60 to 63 amino acids referred to as the homeodomain. This pseuc
evskaya et al., 2002 [PubMed 12119114]).[supplied by OMIM, Aug 2008]

d for the KLRK1/NKG2D receptor. Binding to KLRK1 leads to cell lysis.

ganisms by affecting both the stability and translation of mRNAs. miRNAs are transcribed by R

ganisms by affecting both the stability and translation of mRNAs. miRNAs are transcribed by R
aining either a simple or bipartite NLS motif. Docking of the importin/substrate complex to the r
ablishment of excitatory synapses (By similarity).
oteasome pathway. Key regulator of adherens junction integrity and differentiation that may be
e circadian clock. This gene is adjacent to a differentially methylated region (DMR) and is impri

al nephron (PubMed:14528312, PubMed:22406640, PubMed:23387299, PubMed:23453970, P
n contractile ring formation during the cell cycle cytokinesis. Required for proper attachment of
ge furrow ingression. Plays a role in bleb assembly during metaphase and anaphase of mitosis
extension is a complex morphogenetic process during which cells elongate, move mediolatera

ses signaled by cytokines, such as TNF-alpha and IL-1 beta, or pathogens via Toll-like recepto
naling events in both innate and adaptive immunity and plays a crucial role in hematopoiesis du
so regulate LRP2/megalin (By similarity).
ndle checkpoint activity (PubMed:9315664, PubMed:12351790, PubMed:14654001, PubMed:1
PRC1 to the plus ends of interdigitating spindle microtubules during the metaphase to anaphase
ne penetration of the viral particle into the cytoplasm (PubMed:26416733, PubMed:26416734).
osis. Required for chromatin and/or kinetochore dependent microtubule nucleation. Mediates AU
as an inhibitor of EIF4E1 activity (By similarity).

osphate and the respective free bases, adenine, guanine and hypoxanthine (PubMed:3197834
romoters such as those of SV40, CMV, or HIV-1. In addition, related sequences are found in th
and survival of hematopoietic progenitor cells.
n.||VIP causes vasodilation, lowers arterial blood pressure, stimulates myocardial contractility, in
neries which require DNA as a template. Histones thereby play a central role in transcription reg
y similarity). Plays a role in many processes like cell division, cytokinesis and also in cell prolifer
rocessibility during elongation of the leading strand. Induces a robust stimulatory effect on the
. A translocation between this locus and the T cell receptor beta locus (GeneID 6957) on chrom

ganisms by affecting both the stability and translation of mRNAs. miRNAs are transcribed by R

PubMed:20627126, PubMed:25778398, PubMed:28218735). Component of a chromosome p

ıanner (By similarity).
ɔn and acts as a regulator of mRNA splicing. Deubiquitination is required for efficient cotranscri

ıanner. Promotes the catalytic efficiency of SLC1A1/EAAC1 probably by reducing its interactic

'hen phosphorylated, highly effective in activating G2 cells into prophase. Directly dephosphory
cell cycle progression and abscission during cytokinesis in a ECT2-dependent manner. Direct

mbranes. In vitro, the TRIAP1:PRELID1 complex mediates the transfer of phosphatidic acid (P
alf-reaction, two molecules of FPP react to form the stable presqualene diphosphate intermedia
gens and other damage-associated signals, initiates the formation of the inflammasome polym

latelet dense granules and melanosomes (PubMed:15102850, PubMed:17182842). In concert
led:9325256, PubMed:18302342, PubMed:9392419, PubMed:11278915).

BIRC4. Induces cleavage and inactivation of BIRC4 independent of caspase activation. Media
atiotemporal formation of the midzone and successful cytokinesis. Required for KIF14 localizati
ctivation of disheveled proteins, inhibition of GSK-3 kinase, nuclear accumulation of beta-cater

ganisms by affecting both the stability and translation of mRNAs. miRNAs are transcribed by R

ıina and the cytoskeleton. The nucleocytoplasmic interactions established by the LINC comple
lanosterol likely through sequential oxidative conversion of 14-alpha methyl group to hydroxym
clin A and cyclin B1 and thereby arrests the cell cycle at early M phase. ESXR1-N acts as a tra

consensus calcium-response element CaRE1 5'-CTATTTCGAG-3' sequence.

ıoting GCK recruitment to the nucleus, possibly to provide a reserve of GCK that can be quickl

ı. It is thought that kelch repeats are actin binding domains. However, the specific function of th
nnate and adaptive immune responses, hematopoiesis, responses to growth factors and cytoki
e in the reorganization of the actin cytoskeleton, cell migration, differentiation and proliferation,
ver at the neuronal membrane (By similarity). Binds via its PH domain PI4P, phosphatidylinosit

›roper midbody organization and abscission in polarized cortical stem cells. Plays a role in the r

ɹanine nucleotide exchange factor for.
biosynthesis.

PP1CC) to chromatin at anaphase and into the following interphase. At anaphase onset, its as
Stimulates HDAC1 activity. Regulates LYN activity by modulating its tyrosine phosphorylation (

during mitosis (PubMed:19060894). Plays a role in chromosome congression and is required f
ed CENPA molecules into nucleosomes at replicated centromeres. Prevents CENPA-H4 tetra

lutations in this gene may be associated with Alzheimer's disease. [provided by RefSeq, May 2

acropinocytosis by dephosphorylating phosphatidylinositol 3,4-bisphosphate in membrane ruf
nding of leucine to SESN2 disrupts its interaction with GATOR2 thereby activating the TORC1
ubMed:31235911). Critical for spindle function and accurate chromosome segregation during m
tor endocytosis. Coordinates actin remodeling through tyrosine phosphorylation of proteins co
ated more efficiently than dsDNA, and the RNA component of a DNA:RNA hybrid is phosphory
ls.
AD which induces release of the anti-apoptotic protein Bcl-X(L), the regulation of cell cycle pro
AMPAR potentiation, AMPAR membrane trafficking and synaptic plasticity. Regulates AMPAR
tor activity-dependent actin reorganization in dendritic spines. May mediate cross-talks betwee
oxylation in the kidney, and sterol C-27 hydroxylation in the liver.
t mediates the transport of the cationic amino acids (arginine, lysine and ornithine), and it has
ls and natural killer (NK) cells, has role in the regulation of size, number and exocytosis of lytic

ubMed:18692468, PubMed:25263562, PubMed:25457612, PubMed:26449471, PubMed:2661

NA polymerase II as part of capped and polyadenylated primary transcripts (pri-miRNAs) that

. In human, expression of a functional protein is regulated by alternative splicing. The protein-c
em. The rank order of affinities for the leukotrienes is LTB4 > 12-epi-LTB4 > LTB5 > LTB3.

e proteome from stress, folding and transport of newly synthesized polypeptides, activation of
NA polymerase II as part of capped and polyadenylated primary transcripts (pri-miRNAs) that
NR3C1, AR and PGR. Isoform 5 activates the U2 small nuclear RNA (snRNA) promoter.

'2) complex, mediates the polyubiquitination and subsequent degradation of CDT1, CDKN1A/p

tory synaptic responses. Involved in the suppression of glioma (By similarity).

bMed:26766442). Involved also in positive regulation of cell proliferation and tumor cell growth

Plays an important role in signal transduction.

id CEP170 for hierarchical subdistal appendage assembly (PubMed:28422092).
e proteome from stress, folding and transport of newly synthesized polypeptides, activation of

binding results in a clear conformational change analogous to the Venus fly trap mechanism (F

nd apoptosis. A chromosomal deletion that includes this gene is one cause of Prader-Willi sync

ocyte differentiation, proliferation and apoptosis, and is required for normal skeleton developme
gulation of oligodendrocyte differentiation during postnatal growth. Involved in dendritic arboriz
and blocking cell cycle progression. Functions in the nuclear localization and assembly of cycl
ınd cyclin A-CDK2 complexes. Forms a complex with cyclin type D-CDK4 complexes and is inv
ages.

NA polymerase II as part of capped and polyadenylated primary transcripts (pri-miRNAs) that c

cose transport (By similarity).
CM2 subunit at 'Ser-40' and 'Ser-53' and then is involved in regulating the initiation of DNA repli
includes PYCARD, NALP2 and CASP1 and whose function would be the activation of proinfl

arge repulsion. Acts as a pro-adhesive molecule, enhancing the adherence of cells to immobil

n important role in the regulation of cell survival, cell division, cell differentiation and cell migrat

ɜems to depend on the association with the katanin complex formed by KATNA1 and KATNB1.

ɜd:26686862, PubMed:30463011). Negatively regulates ligand-induced Notch activity probably

ation. Key effector of the RBBP6 and ZBTB38-mediated regulation of DNA-replication and com

: epilepsy type 5. [provided by RefSeq, Dec 2011]
ǝ684392, PubMed:16492674, PubMed:27907090, PubMed:14506133, PubMed:14578375, Pub

)8, PubMed:17651496, PubMed:19114591, PubMed:27499292, PubMed:20739937). Replaces

ıment of chromosomes at the spindle equator resulting in the formation of the metaphase plate
ng vesicles and the microtubule network though its association with STX4 and SNAP25. Acts a
by GTP, and for G protein-effector interaction.

ıd to the promoters of CLDN7 and KRT8 and, in association with histone demethylase KDM1A

re thought to promote expression of ZEB1. Expression of this gene is correlated with tumor pro
.I1, GLI2 and GLI3 by opposing the effect of SUFU and promoting their nuclear localization (Pu

it NF-kappa-B transactivation of IL2. Participates in energy homeostasis by sequestrating the k
transformation and metastasis formation, and in regulating extracellular-regulated kinase (ERK

fically to the activation domain of HIV-1 Tat and can also interact with the HIV-2 and EIAV Tat

ıest in the spleen, where senescent erythrocytes are sequestrated and destroyed. Exhibits cyto

NA polymerase II as part of capped and polyadenylated primary transcripts (pri-miRNAs) that c
ock, which is essential for coordinated somite segmentation (By similarity).

y also play a role in T-cell antigen receptor/TCR signaling, interleukin-2 signaling, apoptosis an
f Wnt4 (By similarity). Necessary to maintain the differentiated epithelial phenotype in renal cel
n in T-cells.

zed by the POLO box domains. Phosphorylates ATF2, BCL2L1, CDC25A, CDC25C, CHEK2, H
of PROX1 and other genes coding for lymphatic endothelial markers. Plays an essential role ir
15235793, PubMed:15239953, PubMed:15548592, PubMed:17535814). Required for kinetoch
NA polymerase II as part of capped and polyadenylated primary transcripts (pri-miRNAs) that c

zes type II iodothyronine deiodinase/DIO2. Confers constitutive instability to HIPK2 through pro

pendent manner.
ay play a role in limb-pattern formation. In osteoblasts, suppresses transcription driven by the c

rminal region. The protein plays a role in small GTPase-mediated signal transduction and the o
, cell-cell adhesion, cell differentiation, proliferation and migration. Positively regulates microRN
s (HAT) (PubMed:29973595, PubMed:31527837). KAT2A recruitment specifically promotes ac
oth acute and chronic vascular inflammation in conditions such as atherosclerosis and in particu
n, motility, migration and cell cycle, functions in neuron growth and ion channel regulation, and
d in early and late stages of mammalian cardiovascular development. Inhibits myoblast differen
ic activity. The receptor for this protein is RON tyrosine kinase, which upon activation stimulate
er lipids, such as D,L-threo-dihydrosphingosine, N,N-dimethylsphingosine, diacylglycerol, ceram
e enhancer elements of a number of cellular promoters, including those of the class I MHC, inte
)). Mediates the initial step of glycolysis by catalyzing phosphorylation of D-glucose to D-glucos
Med:15601820, PubMed:21199876). Negatively regulates nitric oxide (NO) production and limi

ıultiple phase I and II xenobiotic chemical metabolizing enzyme genes (such as the CYP1A1 ge
ɫ form that is able to recruit to membranes different set of downstream effectors directly respon

ɘquired in the control of cell shape.
red for the biosynthesis of normal levels of retinoic acid in the embryonic ocular and nasal regic
AKT1, 'Ser-660' of PRKCB isoform beta-II and 'Ser-657' of PRKCA. Akt regulates the balance

forms perform diverse functions such as inducing growth and differentiation of epithelial, glial, ı
jiogenesis through regulation of expression of genes controlling endothelial cell migration and i
lium and long chain aldehydes into non-toxic fatty acids (PubMed:1737758). Preferentially oxid
al negative limb of the circadian clock by directly repressing the expression of core clock comp
vels; acts early to enhance canonical WNT-signaling by repressing expression of TLE4, and ac
ver, and roles in inflammation and immune response. The encoded type 2 transmembrane pro
Med:15601820, PubMed:21199876). Negatively regulates nitric oxide (NO) production and limi
ds single-stranded DNA with high affinity in a sequence-independent manner, while isolated su

lates MCM2 subunit at 'Ser-40' and then is involved in regulating the initiation of DNA replicatic
intracellular domain. May be required for tumorigenesis of a subset of gastric adenocarcinoma
spindle dynamics. Increases the rate of turnover of microtubules on metaphase spindles, and c

(PubMed:16461343, PubMed:18056643). Associates with and probably dephosphorylates CBI
d binds to some phosphorylated proteins and promotes their ubiquitination and degradation (P
catalysis. Forms an active ribonucleotide reductase (RNR) complex with RRM1 which is expres
I actin filaments. Causes the formation of many neuritic processes from the cell body with disru

specific motif recognized by the POLO box domains. Phosphorylates CENPJ, NPM1, RAPGEF
ıe activity is stimulated by poly(A)-binding protein (PABP). PAN deadenylation is followed by ra
). Apelin-36 has a greater inhibitory activity on HIV infection than other synthetic apelin derivati

s, which are important for efficient cargo delivery and trafficking in axons. Thereby also require

e acceptor substrates but exhibit different Km values.
ınce to DNA cross-linking drugs, and prevention of chromosomal breakage (PubMed:2034742
27' of histone H3 to form H3K27me1, H3K27me2 and H3K27me3, respectively. Required for e

tially binds to telomeres that have a low concentration of shelterin complex and acts as a regul

presses MYC dependent cell transformation (By similarity).
ependent manner. Also promotes cell tumor growth.
by inhibiting the formation of active Rag GTPase complexes. Preferentially binds inactive Rag

C25A15 (PubMed:19287344). Does not transport carnitine nor acylcarnitines (PubMed:246522

; that may trigger signaling for repulsion (By similarity). Functions as netrin receptor that negati

ctivation. Also inhibits hypoxia-inducible ARNT-mediated gene expression.||[Isoform 3]: Attenua

18:1) or palmitate (C16:0), and lower activity with C18:0 and C6:0 lysophosphatidic acid.

rotein-coding potential. [provided by RefSeq, Jul 2008]
nplicated in cell adhesion, signal transduction, tumorigenicity and metastatic behavior of malign

structures, preventing replication pausing and double-strand breaks (DSBs) at G4 motifs. Invo

n the actin-based neuronal function (By similarity).
n to generate an enediolate intermediate that is stabilized by the magnesium ion (PubMed:246
RB (paired immunoglobin-like type 2 receptor beta) genes on chromosome 7. The readthrough

poration of newly synthesized CENPA into centromeres via its interaction with the CENPA-NAC
tumor suppressor gene candidate. A chromosomal translocation t(12;22)(p11.2;q13.3) leading

na and NR1H2/LXR-beta, acts as an essential transcriptional regulator of lipid metabolism. Pla

s a 5-10-fold higher activity on neutrophil activation, IL-8(5-77) has increased activity on neutrop
n structure of some of these variants cannot be determined. [provided by RefSeq, Nov 2015]

endent apoptosis by repressing AKT1 activity. Its direct transcription regulation by p53/TP53 ma
domain. Also acts as a ubiquitin ligase E3 toward itself and ARRB1. Permits the nuclear export

omain, a disintegrin-like domain, and a thrombospondin type 1 (TS) motif. Individual members c
es cellular functions not only in skeletal and cardiac muscle development, but also in neuronal c
eta(3-72) and SDF-1-alpha(3-67) show a reduced chemotactic activity. Binding to cell surface p

ion (PubMed:21788334, PubMed:30026317). Also functions as a catalytic subunit of a TRAMP
3) genes on the short arm of chromosome 16. A duplicate read-through locus also exists betwe
tes and endothelial cells. Mediates activation of NF-kappa-B. Triggers increased phosphorylati
t. Enhances transcriptional activation mediated by SOX18. Plays an essential role in hippocam
5627, PubMed:25816751). Mediates negative transcriptional regulation of CDC25B expression
tes MBD1-dependent transcriptional repression, probably by recruiting complexes containing S

embrane. Additionally, may also function as a lipid-presenting protein to enhance the activity of

an E3 ubiquitin-protein ligase regulating NF-kappa-B signaling and regulates both canonical an

NA polymerase II as part of capped and polyadenylated primary transcripts (pri-miRNAs) that c
ferent Lys residues of the ubiquitin (polyubiquitin chains) or a linear polymer linked via the initia

CM2-7 ring are formed through the interaction surfaces of two neighboring subunits such that a
wide range of biologic activities.[supplied by OMIM, Jan 2003]

ıent cascade of caspases (aspartate-specific cysteine proteases) mediating apoptosis. FAS-me
n the peroxisomal beta-oxidation of fatty acids.
of NF-KB protein into the nucleus, resulting in suppression of apoptosis of bladder cancer cells
vent apoptotic death.
delivers them into the growing sperm tail.

ontrols the reabsorption of sodium in kidney, colon, lung and sweat glands. Also plays a role in

arms, the inactivation of anaphase-promoting complex/cyclosome (APC/C) inhibitors, and the r
osome alignment and segregation and is required for chromatin-induced microtubule stabilizati
527, PubMed:19221179, PubMed:20543840, PubMed:21245099). Binds to the insulin respons

Implicated in regulating a variety of cellular processes, including cellular growth, senescence, c
l cell proliferation, migration and tube-like structures formation (PubMed:24463812). Modulates

ragment and 7m-GDP (By similarity).
o. Displays serine/threonine protein kinase activity. Plays an important role in the regulation of
Implicated in regulating a variety of cellular processes, including cellular growth, senescence, c

e differentiation.

notes proteasomal degradation of MCL1. Competes with BAK1 for binding to MCL1 and can dis

for the mitotic checkpoint.

causes tumor formation. Involved in intracellular protein trafficking of a number of proteins. Ta
bridging TBK1 to MAVS which leads to the activation of TBK1 and phosphorylation of IRF3 and

lation of ERK1/2 (By similarity). Inhibits TGFB-induced epithelial-to-mesenchymal transition in l

otic spindle organization and chromosome segregation (PubMed:24981860). Plays a role in reg
After ligand binding to the receptor, the resulting complex is internalized and transported to a s

und forms of RAB6A and RAB6B. May act as a motor required for the retrograde RAB6 regulat

ion, and in melanogenesis. KITLG/SCF binding can activate several signaling pathways. Promo

breaks located either 5' or 3' to the mismatch. Also exhibits endonuclease activity against 5'-ov

omotes maturation and normal function of calvarial osteoblasts, including expression of the oste

quired for the transition from pre-replication complex (pre-RC) to pre-initiation complex (pre-IC).
nucleolus.
cription by stabilizing the binding of inhibitory cofactors at the promoter. Isoform 2 activates tran

(PubMed:20616313). Essential for retinal progenitor proliferation. Required for suppressing rod
conformation change that triggers signaling via guanine nucleotide-binding proteins (G proteins)
ally conserved modification in the peptidyl transferase domain of the mtLSU rRNA.
NA polymerase II as part of capped and polyadenylated primary transcripts (pri-miRNAs) that c
hagy, relocates to autophagosomes where it interacts with autophagosome-associated proteins
eraction with RIMS2 (By similarity).

gene. [provided by RefSeq, Nov 2017]
some (APC/C), a cell cycle-regulated ubiquitin ligase that controls progression through mitosis.

Serves to alter COP1 substrate specificity by directing the activity of COP1 toward CEBPA (Pu
ct by preventing phosphorylation of the STAG2 subunit of cohesin complex at the centromere, (
cium-regulated exocytosis.
similarity). Inhibits cardiac hypertrophy through the calmodulin-dependent kinase II (CaMKII) pa

nd covers a substantial fraction of the chromosome surface (PubMed:27362226). Prevents chr
cytes-macrophages. This CSF induces granulocytes.
elease from granule cells leading to regulate cell differentiation and survival during cerebellar d
ino atrial node (SAN) head area.

ment, normal timing of sister chromatid segregation, and maintenance of spindle pole architec

sphorylates AKT1, AR, MCF2, WASL and WWOX. Implicated in trafficking and clathrin-mediat

May negatively regulate the repressor activity of isoform 1 by binding to isoform 1, thereby prev
ox) complexes, respectively (PubMed:10882109, PubMed:11278856, PubMed:11705987, Publ

ve element. Active transcriptional corepressor of SMAD2. Links the nodal signaling pathway to

in vitro and inhibits entry of cells into S phase.

31, TGFB2 and TGFB3 signal from the cell surface to the cytoplasm and is thus regulating a ple
is fused in-frame to the 3-prime end of the SSX1 (MIM 312820), SSX2 (MIM 300192), or SSX4

e1), thereby playing a central role in histone code. Specifically binds trimethylated 'Lys-4' of hist

ough its interaction with GDF5 and, probably, GDF6 (PubMed:21976273, PubMed:26643732).
sidue, while it has weak or no activity on trimethylated H3 'Lys-9'. Demethylation of Lys residue
development, migration and function, and in melanogenesis. In response to KITLG/SCF binding

al transcription factor in endoplasmic reticulum (ER) stress response (PubMed:15322075, Pub

and TGFBR1 ubiquitination and proteasome-dependent degradation. Promotes ubiquitination

ombin and nerve growth factor (By similarity). May be involved in cAMP-induced activation of N
ubMed:28471450, PubMed:28609714). Negatively regulates the part of the TLR4 signaling pat

ormamidopyrimidine (Fapy) and 5-hydroxyuracil. Has marginal activity towards 8-oxoguanine. H

robrevin, dystrophin, talin-1, utrophin and vinculin, is able to link these heteropolymeric IFs to a
-1, MMP-2, MMP-3, MMP-7, MMP-9, MMP-13, MMP-14 and MMP-15.
nas and solid tumors. Two non-protein coding transcript variants have been found for this host g

that contributes to gene activation by histone phosphorylation and functions in the regulation c
mottled pigmentation, telangiectasia, epidermal atrophy, tendon contractures, and progressive

NA polymerase II as part of capped and polyadenylated primary transcripts (pri-miRNAs) that c
MT1 methyltransferase to ensure faithful propagation of the DNA methylation patterns through D

ubMed:19478087). It is involved in the negative regulation of the angiotensin-activated signaling
tal myogenesis. Also involved in controlling cell division of progenitor cells and regulating the su

NA polymerase II as part of capped and polyadenylated primary transcripts (pri-miRNAs) that c
terning and skeletogenesis.

f T-cells and limits T-cell effector response (PubMed:11015443, PubMed:28813417, PubMed:2
pathway, ZFYVE9/SARA recruits the intracellular signal transducer and transcriptional modulato
NA polymerase II as part of capped and polyadenylated primary transcripts (pri-miRNAs) that c
arity). Recognizes and binds the X-box, a regulatory motif with DNA sequence 5'-GTNRCC(0-3
on, playing a critical role in biological processes such as cell migration and proliferation. Also fu
ng different isoforms have been observed for this gene. [provided by RefSeq, Jan 2009]
ne RXR/RAR heterodimers bind to the retinoic acid response elements (RARE) composed of ta
a role in cell trafficking, notably of the Schwann cell, and is necessary for the maintenance and

gradation and muscle atrophy (PubMed:23401856). Negatively regulates PI3K-AKT pathway a

ıserting one oxygen atom into a substrate, and reducing the second into a water molecule, with

:her than out of it. Their voltage dependence is regulated by the concentration of extracellular p
during angiogenesis (PubMed:18424730, PubMed:23696226, PubMed:25363763). Its coinhibi

by GTP, and for G protein-effector interaction.
tivity. Catalyzes the recognition of homology and strand exchange between homologous DNA p
script variants of this gene. [provided by RefSeq, Jul 2013]

ternates between an active, GTP-bound state and an inactive, GDP-bound state. Signaling by

NA polymerase II as part of capped and polyadenylated primary transcripts (pri-miRNAs) that c

tion factors during embryonic development. Plays an important role in maintaining embryonic s
(PubMed:16424001, PubMed:16730711). Enhances the proliferation of follicular dendritic cell

the TCR beta enhancer. Represses cyclin D1 transcription by recruiting HDAC1 to its promoter

›Med:12133952).
ıbMed:20080638). Involved in adipogenic differentiation (PubMed:19190184).

ivity and SIRT1 is proposed to contribute to T-cell hyperactivation during infection.||[Isoform 2]:
ısomes. May play a role in regulating the period length of ARNTL/BMAL1 transcriptional oscilla
ogenic gene expression upon cAMP signaling pathway stimulation (PubMed:17210646). Involv
s transcription of NF-kappa-B target genes. Contributes to the regulation of cell proliferation (By

Med:22278742). Associates specifically via disulfide bonds with the Latency-associated peptide
a large number of inositol polyphosphates that are important in cellular signaling. Both calcium/

ase of transforming growth factor beta-1 (TGF-beta-1) from regulatory Latency-associated pep
llular environment by damaged epidermal cells.
NA polymerase II as part of capped and polyadenylated primary transcripts (pri-miRNAs) that c
otein-free DNA (By similarity). Among other functions, seems to be involved in cell death by ap
mplex acts via chromatin remodeling and modification of histones; it mediates monoubiquitinat

ption from promoters with several copies of the Tcf motif 5'-CCTTTGATC-3' in the presence of
ent BAZ2A/TIP5 by controlling its USP21-mediated deubiquitination (PubMed:21914818, Pub

to inhibit the activity of the anaphase-promoting complex/cyclosome (APC/C) by blocking the
s at the kinetochore, being required for the subsequent localization of CENPF, BUB1B, CENPE

the pre-replication complex necessary to initiate DNA replication.
use the human UFSP1 protein is shorter on the N-terminus and lacks a conserved Cys active s
M-bearing adapter molecules on the cell surface.
, protects centromeric cohesion complexes until metaphase II/anaphase II transition, preventin

oration of newly synthesized CENPA into centromeres via its interaction with the CENPA-NAC
ucleosomal DNA within the nucleosome core region. May function as transcriptional co-repress
ents. May stabilize the CENPE-BUB1B complex at the kinetochores during early mitosis and m
in addition to several beta-2 integrins. [provided by RefSeq, Jan 2013]
oment and to inhibit hair follicle induction (By similarity).

ASSF7 protein via the ubiquitin-proteasome pathway. Required for the activation of AURKB ar

h leads to increased expression of ATF4 which plays a central role in osteoblast differentiation
istone deacetylase activities which can associate with this corepressor. Involved in the repress
l proliferation (By similarity). May regulate WDR65 transcription (By similarity).

th with short-chain fatty acyl groups (tributyrin) and long-chain fatty acyl groups (triolein) with si
he Notch-induced endocardial epithelial to mesenchymal transition, which is itself criticial for ca

e to convert CMP-N-acetylneuraminic acid (CMP-Neu5Ac) into its hydroxylated derivative CMP
acids to control fatty acids composition of cellular lipids in brain (Probable).
osine-regulated kinases (DYRKs) autophosphorylate a critical tyrosine residue in their activatio
36, leading to target the PHF2-ARID5B complex to target promoters, where PHF2 mediates der

on of many genes expressed during hematopoiesis, inflammation, immune responses and cell p

FOS proteins have been implicated as regulators of cell proliferation, differentiation, and transf
n the control of cardiac fibrosis. It is also involved in the control of cardiac hypertrophy acting th
king the TCEA1 binding site. Required for mitotic chromosome separation after cross-over ever
ase the intron. The products are an intron and two tRNA half-molecules bearing 2',3'-cyclic pho
reby mTORC1. Binding of arginine to CASTOR1 allosterically disrupts the interaction of CAST(

hosphatidylcholine (PubMed:24097981). Plays a role in lipid homeostasis and macrophage-me

th the GAR1 protein. [provided by RefSeq, Apr 2009]
sposon silencing is thus critical for maintaining genome stability, in particular in germline cells w
al for neuronal development, and that of CDK2 by WEE1, which leads to decreased CDK2 activ
gulation, DNA repair, DNA replication and chromosomal stability. DNA accessibility is regulated

x in the ER thus preventing it from escorting SREBPs to the Golgi. Initiates the sterol-mediated

esults showing activation by WNT7B have been described for mouse (By similarity). Functions
cardiac transcriptional activators GATA4 and GATA6.

onical signaling pathway leads to the activation of disheveled proteins, inhibition of GSK-3 kina
similarity). Induces apicobasal cell elongation by redistributing gamma-tubulin and directing th

quitination of KRT14 and controls its levels during keratinocytes differentiation (PubMed:27798(

NA polymerase II as part of capped and polyadenylated primary transcripts (pri-miRNAs) that c

182175) and a 7SL RNA molecule, such as RN7SL1, that is partially homologous to Alu DNA (I

, PubMed:31672913). In low-amino acid conditions, component of the lysosomal folliculin comp
nts have been suggested, but their full length natures are not clear. [provided by RefSeq, Jul 2
on and is required for chromatin-induced microtubule stabilization and spindle assembly. Acts a
l of a kinase cascade wherein MST1/MST2, in complex with its regulatory protein SAV1, phosp
As (snRNAs) (Gu et al., 2005).[supplied by OMIM, Mar 2008]
5709802, PubMed:27485036). Involved in the cytolytic activity of natural killer cells and cytotoxi
nction of the enzymatically inactive isoforms remains unknown.

5-mediated apoptosis and is required for the increased motility and invasiveness of CD95-activa
gulation, DNA repair, DNA replication and chromosomal stability. DNA accessibility is regulated

genes involved in a large spectrum of important biological functions including proper eye, face,

cell leading edge. Promotes filipodia formation (By similarity). Functions synergistically with PEL

e on activin A than on activin B. Involved in bone formation; inhibits osteoclast differentiationc.

PubMed:9315713). May regulate genes that protect epithelial cells from bacterial infection (Pul

uence identity with RANBP2, a large RAN-binding protein localized at the cytoplasmic side of t

ne predominant proteoglycan present in cartilage and is distributed on the surfaces of many cel
oliferation of hepatocytes in response to liver damage. Binding to its receptor formed by IL6ST

culum to the Golgi apparatus of a variety of secretory cargos.

ination and subsequent proteasomal degradation of TRAF family members and RHOA. Promo

scriptionally repressive state of genes: acts as a chromatin compaction factor by recognizing ar
d GATA3. Such heterodimer can both activate or repress transcriptional activity, depending on
nd 28S rRNA. Has diphosphatase activity and removes m7G and/or m227G caps from U8 snoF
ediated signaling. Required for the localization of ECT2 to the central spindle. Plus-end-directed

ogenesis of ectodermal organs. Functions as a ligand activating the DEATH-domain containing
lesions spiroiminodihydantoin (Sp) and guanidinohydantoin (Gh) in both ssDNA and dsDNA; a

cific manner. In vitro, modulates KCND2/Kv4.2 and KCND3/Kv4.3 currents. Involved in KCND2

ess of kinetochore-spindle attachment and inhibits the activity of the anaphase promoting comp

22108654, PubMed:21477596, PubMed:23949095). Thereby, acts as a central switch between
events including the establishment of mitotic spindle, centrosome duplication, centrosome sepa

of proinflammatory genes. May block the BMP-SMAD1 signaling pathway by competing with S

s preventing SMAD2 access. Functions as an adapter to recruit SMURF2 to the TGF-beta rece
Also functions as a transcriptional coactivator in the TGF-beta signaling pathway by increasing

omputer predictions indicate that this region of the 3' UTR contains hairpin-forming self-comple

elix and shields approximately 20 base pairs, and recognizes single base mismatches and dinu
9, PubMed:9261177, PubMed:16373578, PubMed:22942274, PubMed:26859324, PubMed:27

fies DNA damage signals, leading to enhancement of apoptosis.
643323). MMEJ is an error-prone repair pathway that produces deletions of sequences from th

46435). Binds to its receptor, GFRAL, and activates GFRAL-expressing neurons localized in th
in DNA replication. The DRTF1/E2F complex functions in the control of cell-cycle progression f
gulate downstream signaling cascades (PubMed:23166352). Helps recruit the ESCRT comple
ough the phosphorylation of actin dynamics regulators such as PKN2. Is a negative regulator c

otease, which may be associated with various inflammatory processes as well as development

NA polymerase II as part of capped and polyadenylated primary transcripts (pri-miRNAs) that c

various activators and repressors. TAF4B is a transcriptional coactivator of the p65/RELA NF-

rms of spinal MAPK1/3 and MAP kinase p38 induced by peripheral surgery, which drives the re
(PubMed:26583432, PubMed:15016378, PubMed:24879834). Regulates signaling pathway le

JUN and MYC (PubMed:24344117). May play also a role in cell proliferation through activation

binds to phosphorylated target proteins during skeletal muscle atrophy. Recognizes TERF1.
of DP proteins and specifically recognizes the E2 recognition site 5'-TTTC[CG]CGC-3'. Directly
NA polymerase II as part of capped and polyadenylated primary transcripts (pri-miRNAs) that c
a p53/TP53-dependent manner, resulting in the pentose phosphate pathway (PPP) activation a

activity of CNOT7 and CNOT8. Involved in cell cycle regulation. Could be involved in the grow
11070). Can metabolize vitamin D analogs/prodrugs 1alpha-hydroxyvitamin D(2) (doxercalcifer
ding sequence 5'-TGTGGT-3', or very rarely, 5'-TGCGGT-3', within their regulatory regions via
cellular proliferation. It is mitogenic for fibroblasts, but not endothelial cells. It is able to bind EG
. Cooperates with CDC6 and the origin recognition complex (ORC) during G1 phase of the cell
posed of a kinase cascade wherein STK3/MST2 and STK4/MST1, in complex with its regulato

tabolic route allows the elimination of L-methionine and the toxic metabolite L-homocysteine (F
tream of the 3M complex, which inhibits CUL9 activity, leading to prevent ubiquitination of BIR
ansmitter release, possibly by regulating Ca(2+)-sensing. In the cerebellum, may inhibit SNARE
iting cell growth (PubMed:9748269, PubMed:10207080, PubMed:16131492). Represses trans
m for the ciliary compartment.

resistant to the inhibitory effects of CDKN2A/p16INK4A.
ivery of the misacylated tRNA to the ribosome and incorporation of ROS-damaged amino acid

iquitin-mediated proteasomal degradation mechanism.
(PubMed:22327364). Plays a role in metaphase spindle orientation (PubMed:22327364). Play

ion as a regulator of vesicle transport, through interactions with the JNK-signaling components
15203199). Can repress the expression of MMP7 in a ZBTB33-dependent manner (PubMed:2

timulation of acute-phase protein synthesis in hepatocytes.
igodendroglia in the developing spinal tube (By similarity). Binds to the gene promoter of MBP

nal snRNPs contain a common set of Sm proteins SNRPB, SNRPD1, SNRPD2, SNRPD3, SN
activation with the PAR3 complex. Plays a redundant role with FRMD4A in epithelial polarizatic

al negative limb of the circadian clock by directly repressing the expression of core clock comp
single-stranded DNA, in particular to polypyrimidines. The SOSS complex associates with DN

which contributes to the functional inactivation of these proteins (PubMed:15870257, PubMed:1
hich is involved in the control of centrosome separation and bipolar spindle formation in mitotic

an essential step to ensure completion of synapsis. Not required for HORMAD1 functions in p
HIV-1 infection, may function as a receptor for extracellular Tat in neurons, mediating its intern

ional activators and repressors of the Wnt/beta-catenin and MAPK/ERK pathways, respectivel
lysophosphatidylcholine (lyso-PC). May be involved in bioactive N-acylethanolamine biosynth

NA polymerase II as part of capped and polyadenylated primary transcripts (pri-miRNAs) that

pindle in response to adhesion or growth factor signals initiating cell proliferation. May play an

holipids. Phospholipid translocation seems also to be implicated in vesicle formation and in up
ay a role in organizing centrosomal microtubules. May act as a tumor suppressor protein. May
inding to regulator loci of RORC to modulate expression (By similarity).

-like domains resulting in inhibition of NOTCH1 activation by JAG1 and enhancement of NOT

Second, by enhancing signaling from ABL1 and ABL2, which regulate cytoskeletal remodeling.
es of the current expressed by the KCNMA1 channel. Isoform 2, isoform 3 and isoform 4 partial

nulate energy expenditure associated with the browning of the white fat depots and improves g
invasive activity, and promotes cancer metastasis.

n p53/TP53. Regulates ER stress-induced neuronal apoptosis (PubMed:23340338).
ally with RUNX1 to transactivate the IL3 promoter (By similarity). Also transactivates the PRF1
promotes their expression, thereby neutralizing reactive electrophiles (PubMed:11035812, Pub
and proliferation but also the differentiation and programmed death of T-lymphocytes as well as
n of neuronal progenitor cells in the developing brain and for normal brain development. Does
naling pathway.
eq, Dec 2008]

RNA sequesters P-TEFb in a large inactive 7SK snRNP complex preventing RNA polymerase
K2 and MAPK3/ERK1 (PubMed:28111074). May be an activator of the JNK/SAPK pathway.
arily and structurally related but have limited sequence homology, with the exception of the cer
on and is required for chromatin-induced microtubule stabilization and spindle assembly. Major

heir mature forms.
idance (By similarity).
inated EGFR at the cell membrane.
s phosphorylated promoting ubiquitination and degradation, enabling the dimeric RELA to tran

g the same protein, have been identified. [provided by RefSeq, Nov 2008]
s an inhibitor of centrosome reduplication.

nced hematopoietic activity.
vefold higher chemotactic activity for neutrophilic granulocytes.
oRNPs) involved in pre-rRNA processing and modification. snoRNAs of the box C/D class, like
orane in polarized epithelial cells.
epithelium and in the formation of ducts in prostate. Acts as a tumor suppressor controlling pr
of the cytosine methylation status (PubMed:25258363, PubMed:25999311). Regulates the tran

sts of CTF18, CTF8, DCC1, RFC2, RFC3, RFC4 and RFC5. The CTF18-RFC complex binds to

ro.

that mediate the transfer of ARC mRNA into new target cells, where ARC mRNA can undergo

y).
uired for the stable expression of this protein. [provided by RefSeq, Jul 2008]

ors residing on adjacent cells, leading to contact-dependent bidirectional signaling into neighbo

complex is inactive and in anaphase the CDC20-APC/C binary complex is active in degrading

promotes G2-M transition, and regulates G1 progress and G1-S transition via association with r
nechanisms.

n 2012]

acts with FAM111A to overcome host range restriction (PubMed:23093934).

mor growth (PubMed:20123964, PubMed:21569306). Also plays a role in integrin-mediated ce

/led:15769988, PubMed:17635946, PubMed:19141540, PubMed:21664385). Mediates glucoco

:tion (By similarity).
ı high affinity and on E-box motifs: 5'-CANNTG-3' with low affinity (By similarity). May play a rol

drome, an autosomal recessive skeletal dysplasia. Alternative splicing results in multiple transc
EHHADH, catalyzes the hydration of trans-2-enoyl-CoA and the dehydrogenation of 3-hydroxy

as well as mediating neurotransmission in the central nervous system.
e, and is required for normal levels of marginal B-cells in the spleen and normal migration of sp
'P is used as phosphate donor, the enzyme phosphorylates AMP, CMP, and to a small extent c

CoA that is utilized mainly for oxidation under ketogenic conditions (By similarity). Involved in th
·phine (PubMed:9169443). Hydrolyzes aspirin, substrates with large alcohol group and small a

tion. This ciliogenic function is mediated in part by the Rab8 GDP/GTP exchange factor, which
ement seems to require its catalytic activity (PubMed:19531583, PubMed:20923837). Required
iggers the caspase-independent apoptosis by altering mitochondrial membrane permeabilizatic
l regions (PubMed:22055184). Part of the ASC-1 complex that enhances NF-kappa-B, SRF an
26479788, PubMed:27796300). Within the complex, enhances DTX3L E3 ligase activity which
nt. Protects neurons from apoptosis, both by inhibiting JUN phosphorylation by MAPK10 and b
ed to its ATPase activity which is essential for its chaperone activity. This cycle probably induc
do not bind calcium in vitro. Isoform 4 probably does not bind calcium.
ıestration and utilization of iron when cellular iron levels are low. Binds to iron-responsive elem

:he ligand.
le in signal transduction.
d membrane trafficking of recycling endosomes (PubMed:15020713, PubMed:17233914, PubM
ling. Induces macrophage recruitment, increased interstitial pressure, and blood vessel matura
ged and normal DNA, cooperates with the heterotetrameric (REV3L, REV7, POLD2 and POLD
wth phase of the hair follicle, into catagen the apoptosis-induced regression phase (By similari
HT and homotrimeric LTA/lymphotoxin-alpha and the immunoglobulin superfamily members B

all GTPases. May enhance the guanine nucleotide exchange factor (GEF) activity of DOCK1.
and controls the formation of the retinotectal map (PubMed:23307924). Required for normal foı
ɔn, degradation, or transcytosis. Also known as interceptor (internalizing receptor) or chemokine
ıer. Potentiates neurite outgrowth. May be involved in brain development by regulating En-1 an

other synaptic proteins. May promote the initial formation of synapses, but is not essential for
DPE (a derivative of enkephalin lacking an N-terminal tyrosine residue), estrone-3-sulfate, taur
nd specifically to a single methyl-CpG pair. Represses transcription in a methyl-CpG-depender

OXO1 and its apoptotic function by enhancing the interaction of FOXO1 with SIRT1 and FOX

ɔMed:27184406). Probably plays a central role in cytosolic lipid accumulation. In liver, is primar

tdIns(4,5)P2 > PtdIns(3,5)P2 > PtdIns(3,4,5)P3. Plays a role in the biogenesis of endosome ca
atabolism. Ketone bodies (beta-hydroxybutyrate, acetoacetate and acetone) are essential as a
t does norepinephrine.
ith the activation of the adrenergic receptor ADRA1B and leads to the activation of MAPK14.
ıd cardiac development and angiogenesis.
51, PubMed:12794138, PubMed:19416870, PubMed:22692454, PubMed:23846752). In compl
ely regulate the Wnt signaling pathway (PubMed:29374064). Probably plays a role in the devel
a two-step reaction: the amino acid is first activated by ATP to form a covalent intermediate wi
f AMP, dAMP, CMP and dCMP with ATP as phosphate donor and of CMP with GTP as phosph

p.
target cells of infection and the initiation of reverse transcription, it can induce the conversion o

08]

in down-regulation of SIRT1 and thereby is involved in regulation of p53/TP53-dependent apo

ЗRIF1 from the cytoplasm to the mitochondrion. Also plays a role in steroidogenesis through ma
n (18:1 or 18:2) on the sn-2 position. Able to transfer phosphatidylcholine (PC) and phosphatid
synthesis, oxygen sensing and signal transduction. May also activate CYBB/gp91phox and NC

and proinflammatory cytokines. Its ligands include mRNA lacking 2'-O-methylation at their 5' ca

tarate and the reversible oxidation of (S)-malate to oxaloacetate.

, PubMed:25047030, PubMed:18035827, PubMed:9662422, PubMed:18182499). Responsible

ponse to pathogen-associated molecular patterns, such as intracellular poly(dA:dT), but not pc
em medoxomil and lenampicillin.
ity).

pendent interactions (By similarity).
ubMed:20375010). Acts as a receptor for dengue virus (PubMed:24074594).||Claudins functior
variants encoding different isoforms. [provided by RefSeq, Jul 2013]
kin, and for normal cell shape. Required for normal adhesion of keratinocytes to fibronectin an
pressed in tumor-associated vasculature.
. Plays a role in nitric oxide synthesis in human umbilical vein endothelial cells (HUVECs) via tr
gulation of PPP1CA activity (PubMed:21798305, PubMed:21939755). Involved in the regulatior
ght have an important role in remyelination of denuded axons in multiple sclerosis. The non-cla

G-I to dsRNA. Exhibits antiviral activity against hepatitis C virus and vesicular stomatitis virus (V

, being identified as endogenous pyrogens, and are reported to stimulate the release of prostag
genesis. Could be an adapter protein that facilitates and regulates interaction of KDR with effec
phospho-Glu-tRNA(Gln).
d all substrates tested compared to GALNT2, it is able to transfer up to seven GalNAc residues

glia (By similarity).
ng pathways. Functions as a negative modulator of EGFR signaling (By similarity). May act as a
lays a key role in the metabolism of xenobiotics and drugs containing aromatic azaheterocyclic

ay participate in the maintenance/targeting of ion channels and cell adhesion molecules at the
gests that it might act as a tumor suppressor.
n. Has activity toward Muc1a, Muc2, EA2 and fibronectin peptides.
r that binds the cAMP response element (CRE), a sequence present in many viral and cellular
ce, and a BED zinc finger. This gene lies within a region that is deleted in Williams-Beuren sync
ES2 ligand to CMKLR1, a functional signaling receptors. Plays a critical role for the developmer

d double-strand-specific 3'-5' exonuclease activity, which are provided by MRE11. RAD50 may

motes homotypic calcium-dependent aggregation and adhesion and clusters at intercellular jur
l-epidermal cohesion. Is the target of linear IgA bullous dermatosis autoantibodies.
otum formation during embryogenesis, normal development of the gastrointestinal tract, normal
Med:24781757). Can act as a tumor suppressor (PubMed:20484225).
he proteins mediate signal transduction events that play a role in the regulation of cell develop
;.
es F-actin. Involved in synaptic function by regulating neurite outgrowth in an actin-dependent r
h sporadic hepatocellular carcinomas, colorectal cancers, and non-small cell lung cancers. Thi

terminal binding. May interact with cellular G-protein signaling pathways. Can regulate neurite c
with viral RNAs on which it homooligomerizes to form filaments. The homooligomerization allov
Involved in cell migration independently of RAC1, CDC42 and p21-activated kinase (PAK) acti

PubMed:26405230).||Facilitator of innate immune signaling that acts as a sensor of cytosolic D

f cardiac hypertrophy by inhibiting intracellular calcium mobilization in response to angiotensin
860631). Has a high affinity for both cAMP and cGMP (PubMed:8557689). Modulates the amp

atum corneum) and epidermal glycerol content. Involved in skin hydration, wound healing, and

K-phosphorylated receptor and sterically preclude its coupling to the cognate G-protein; the bin

ɔjection: required in both CA1 and subicular neurons for the precise targeting of proximal CA1 a

able to mediate poly-ADP-ribosylation (PubMed:25043379). Has been shown to catalyze the m
mRNA exon inclusion in muscle. Antagonizes the alternative splicing activity pattern of CELF p
tion, stimulate cytotoxic T-cell activity, induce isotype switching in B-cells, and that has diverse
lar poly(A)-specific ribonuclease PARN to remove the poly(A) tail, and the 3'-5' exoribonucleas

icking, in exocytosis, through its interaction with myosin VI and Rab8 (PubMed:27534431). Link

cell death of tumor cells. The ratio between the highly active tetrameric form and nearly inactiv

tion, in cell migration and many other physiopathological events. Plays a direct role in facilitatin
ion by activating ARF6-signaling (PubMed:27044754).

ation synthesized transiently at sites of chromosomal damage to accelerate DNA strand break
ilarity).
GTP-binding protein that does not act as an allosteric activator of the cholera toxin catalytic sub
apoptosis initiation and execution. Acts as a pro-apoptotic component of the unfolded protein re

ɔ make N-sulfated heparosan, a prerequisite substrate for later modifications in heparin biosyn
er, which is further elongated by LARGE1, during synthesis of phosphorylated O-mannosyl glyc
g development (By similarity). May be involved in actin cytoskeleton reorganization by cleaving
ains. Important role in chondroitin chain biosynthesis in cartilage formation and subsequent enc
roteins to the membrane, including AKT1 and PDPK1, activating signaling cascades involved ir

ay play a role as physiological regulators of gastrointestinal, cardiovascular bioactivities mediat
PubMed:23230272). Monoubiquitinates several histones, including histone H2A, H2B, H3 and
calization of ACTN1 and PALLD to stress fibers. Required for cell migration and in maintaining
, mitosis, cell-cell adhesion, cell differentiation, proliferation and migration. Contributes to the li
idation-derived aldehydes. Involved in lysine catabolism.

and STAT5.

ɔlay a role in sperm motility (By similarity).
:ant for antigen-specific immune response, NK-cell mediated clearance, lymphocyte recirculatic
ɔms) and straight-chain acyl-CoA esters (with a chain length longer than four carbon atoms) (P
ing, includes cooperation with ANKRD27 and VAMP7 (By similarity). Plays a role in the matura

ted herpesvirus/HHV-8 infection, is degraded by viral E3 ubiquitin ligase MIR2, presumably to p
to increase ion and water transport and thus raise extracellular fluid volume and blood pressu
d:16482215, PubMed:17079684, PubMed:16407242, PubMed:16407252, PubMed:16940174
s early endosomal interaction with actin filaments and inhibits interaction with microtubules, lea

val, migration, differentiation, and phagocytosis of apoptotic cells (efferocytosis). Ligand bindin
somes for translation competence by allowing 80S ribosome assembly and facilitating EIF6 re

s virus, possibly through GAS6 binding to phosphatidyl-serine at the surface of virion envelope.
ɔpa-B is a homo- or heterodimeric complex formed by the Rel-like domain-containing proteins I

e in mediating local tissue responses to inflammatory conditions, as well as in modulating the s
ɔr proteasomal degradation. Involved in the clearance of proteolytic fragments generated by ca

tide, metastin which functions as the endogenous ligand of the G-protein coupled receptor GP

ɡnaling activation (PubMed:23776175). Regulates TRAF6 and MAP3K7 ubiquitination (PubMe
nbranes disrupted by mechanical stress (By similarity).

4,5-bisphosphate (PtdIns(4,5)P2) and cargos containing non-phosphorylated NPXY internaliz
on its own.
witch between the signaling pathways activated by these second messengers with different cel
) complex involved in the connection between the nuclear lamina and the cytoskeleton. The nu
the excitatory events that elevate the cytosolic Ca(2+) concentration and/or depolarize the cell
s (PubMed:21078852). Forms the activation cluster TLR2:TLR1:CD14 in response to triacylate

ıflux and proliferation during the reactivation of naive T-cells (PubMed:17157250, PubMed:187[illegible]
ʞ which is activated by double-stranded RNA, a sign of viral infection. Acts via the adapter TRI[illegible]
equired for the delivery of cargo proteins to all compartments of the Golgi stack independent of
ɔMed:21398697, PubMed:27929370, PubMed:29899144). Has higher activity in LPL inactivatio

ɛnts and regulate cell spreading through activation of the effector TNIK. May regulate membran
2010]
gher oligomers of 2'-5'-oligoadenylates (2-5A) from ATP which then bind to the inactive monom
riptional activation. Positively regulates TGF-beta signaling through its association with the SM
ene regulation. Synthesizes preferentially dimers of 2'-5'-oligoadenylates (2-5A) from ATP whic

ıscription. May thereby play an important role in establishing distinct coactivator complexes unc

. The presence of plakoglobin in both the desmosomes and in the intermediate junctions sugge

tion. This ciliogenic function is mediated in part by the Rab8 GDP/GTP exchange factor, which

and become committed to their adult state. The transition from proliferating neural stem/progen

ng the degradation of damaged proteins inside mitochondria by promoting the accumulation in
of RB1 allows dissociation of the transcription factor E2F from the RB/E2F complex and the su

e domain, a disintegrin-like domain, and a thrombospondin type 1 (TS) motif. Individual membe
es the overall conversion of 2-oxoglutarate to succinyl-CoA and CO(2) (PubMed:24495017). Th
is involved in DDIT3/CHOP-dependent cell death during ER stress (PubMed:15781252, PubM
ATP availability from mitochondria through regulation of mitochondrial membrane ATP syntha
ner (PubMed:28594398).

h, resulting in locked ternary complexes. Cleavage of the nascent transcript by S-II allows the r

1 acts as a motor for the intracellular retrograde motility of vesicles and organelles along micro
ation of EGF-induced ERK1/ERK2 signaling pathway; blocks MAPK3/MAPK1 nuclear transloca
reorganization of actin cytoskeleton; the function seems to involve WHAMM and includes regul

:ollagen, collagen C-propeptides, fibronectin and E-cadherin. It recognizes the proline-hydroxyl

:ndent manner and hence may participate in the adhesion, formation of invadopodia and matrix
endosomes through interactions with the actin cytoskeleton.
:uch as EA2, Muc5AC, Muc1a, Muc1b and Muc7.

: junction assembly, signal transduction, cell proliferation, synaptogenesis and lymphocyte acti

ulates clathrin-mediated endocytosis of synaptic vesicles and mediates exit from early endosor
:omal-to-cytosol antigen trafficking. Plays also a role in B-cell survival through regulation of aut
Involved in mitochondrial quality control via its interaction with SPATA18/MIEAP: in response t

gulation, DNA repair, DNA replication and chromosomal stability. DNA accessibility is regulated

d for maturation of both mitochondrial and cytoplasmic [2Fe-2S] and [4Fe-4S] proteins (PubMe
nyocarditis virus (EMCV) and hepatitis B virus (HBV). Acts as a transcriptional repressor of HB

de 3-kinase) pathways (PubMed:8723348, PubMed:10764818, PubMed:8769125). Able also t
As, inhibition of protein synthesis through the degradation of rRNA, induction of apoptosis, and
ll migration. May be involved in cell migration and bone development (By similarity).
ereby contributes to the regulation of endocannabinoid signaling, nociperception and perceptio
bdenum cofactor. In the first step, molybdopterin is adenylated. Subsequently, molybdate is in
gulation, DNA repair, DNA replication and chromosomal stability. DNA accessibility is regulated
:ndent manner.
ecifically via disulfide bonds with the Latency-associated peptide (LAP), which is the regulatory
be involved in endosome fusion.

CASP3, perforates cell membrane and thereby induces pyroptosis. After cleavage, moves to th

rmal skeleton formation during embryonic development, especially for normal development of t

e in BTK-related cytoplasmic signaling in B-cells. May be involved in BCR-induced apoptotic ce
damaged proteins, which could impair cellular functions, and by removing proteins whose func
transfers sulfate to the unsulfated polymer. The sulfotransferase activity on sialyl LacNAc stru

ed in the ATP-dependent degradation of ubiquitinated proteins. This complex plays a key role i

bound state. Inhibits RAC1-dependent lamellipodia formation. May also play a role in transcripti

TP-dependent proteolytic activity. This subunit is involved in antigen processing to generate cla

4). Important for the internalization of GLUT4. Required for fusion of myoblasts to skeletal musc

d also the zwitterionic cephalosporin, cephalexin and cephradin. Seems to also play a role in th

moter region of many genes that are regulated by TGF-beta and, on formation of the SMAD3/S
lates PTK2B/PYK2 and myosin light-chains. Involved in the inflammatory response (e.g. apopto

ular lipid homeostasis. Involved in the plasma clearance of chylomicron remnants and activated

triction factor, also acts as a pattern recognition receptor that activates innate immune signalin

ecules of LPL.

nilarity).

ı novel autoregulatory feedback loop (DEC loop) which differs from the one formed by the PER
sis and for immature hematopoietic stem cell subsets. Belongs to the tetraspanin web involved
in A and C are present in equal amounts in the lamina of mammals. Plays an important role in
ɔression down-regulation observed in tumor cells, suggests that it may function as a tumor sup
ıl forms of cell junctions and recruits PARD3 (PubMed:11489913). The association of the PARI
thereby preventing tight junction assembly. The PARD6-PARD3 complex links GTP-bound Rh
of RB1 allows dissociation of the transcription factor E2F from the RB/E2F complex and the su

ɘ C-terminal region. The gene is expressed in normal stomach and testis, as well as in gastric,
RBPJ-mediated repression of transcription.

ɟulation, DNA repair, DNA replication and chromosomal stability. DNA accessibility is regulatec

ɪect to tissue-specific and developmentally regulated alternative splicing. Specifically activates

Binds preferentially to the 5'-[AU]UAAA-3' motif in vitro. Binds optimally to RNA containing 5'-[A
p.
ɔskeleton. May play a role in endocytosis.
target cells of infection and the initiation of reverse transcription, it can induce the conversion o
ɪlatory element binding proteins (SREBPs). Can also promote apoptosis. Upon inflammasome
ɘptide. Nascent MHC class I molecules associate with TAP via tapasin. Inhibited by the covale

feration and cell differentiation.
g mTORC1 substrates. mTORC1 is activated in response to growth factors or amino acids. Grc
h interaction with ROBO1, increases the interaction of SRGAP1 with ROBO1 and inactivates C

bMed:8849450, PubMed:8929542, PubMed:9427609, PubMed:10074122, PubMed:10756055
6-Asp-|-Gly-217' bond. Overexpression promotes programmed cell death.
n particle (SRP), which normally targets nascent secretory peptides to the ER. May also reduc
ɔugh structurally the encoded protein is more like a type II hair keratin. This protein is involved i
045166).

to injury that includes DNA damage, NEK1 phosphorylates VDAC1 to limit mitochondrial cell d
lipid efflux is ALB-dependent (PubMed:16702602). Is an active component of the macrophage

ır is referred to as forward signaling while the signaling pathway downstream of the ephrin ligar
system. May play a role in the asymmetric division of fibroblasts and participate in the process

anscription of genes encoding cell-cycle inhibitors, such as p21/CDKN1A (PubMed:21767813).
n culture. The presence of FG-GAP motifs and an RGD integrin-binding motif suggests that thi

oform 4 have different affinities for different nuclear receptors. Involved in the regulation BCL6

s and allows them to influence estrogen receptors-alpha signaling.
sin exerts cell-scattering activity toward a wide variety of cells, including epithelial, endothelial,

ersion of prion protein PRPN(C) to PRNP(Sc) via associating (via the heparan sulfate side chai
induce the phosphorylation of LATS1 and LATS2 and can probably function in the regulation o
. Recognize peptide-major histocompatibility (MH) (pMH) complexes that are displayed by anti

ants in germline transformation experiments. May participate in the transport of mitochondria a

d in the cytoplasm during stress or infection. These proteases are important in killing microbes

f USP4 and IKBKB but also for its self-ubiquitination. Component of cullin-RING-based SCF (S

lendrites in Purkinje cells, and may regulate its activity-dependent translation. With ELAVL1, bi

TPase activity (PubMed:17157868). Component of the NuA4 histone acetyltransferase comple
stence of the anagen (growth) state (By similarity). Modulates the function of TNF-alpha in the

r with KRT8, is involved in interleukin-6 (IL-6)-mediated barrier protection.
Selectively binds inositol-4,5-bisphosphate, inositol-3,4,5-trisphosphate and inositol-1,3,4,5-tetra

ts (nucleation) as well as sever filaments already formed. Plays a role in ciliogenesis.
and to involve an effect on nAChR trafficking and its cell surface expression, and on single cha
otein kinase A-anchoring protein (AKAP). May act as a scaffolding protein that links PKA to co

response to BMP2 leading to activate transcription of BMP target genes. Acts as a transcriptior
contraction. Does not form homofilaments nor heterofilaments with other IF proteins.
s peroxisomal beta-oxidation pathway. Optimal isomerase for 2,5 double bonds into 3,5 form is
site-bound peptidyl-tRNA and P-site-bound deacylated tRNA move to the P and E sites, respec
7344214, PubMed:18242580, PubMed:23084476, PubMed:31462771). Once activated, recrui
HIV-1 p6- and p9-dependent virus release.||Probable core component of the endosomal sortin

ulfide or cyanide (in vitro) (PubMed:22852582). It is believed the in vivo electron acceptor is glu
tion ability activated by UBA6. Promotes the expression of the proteasome subunit beta type-9
:7973638). Binding to Eph receptors residing on adjacent cells leads to contact-dependent bidi

resentation. Enhances phosphorylation of CD79A, possibly by recruiting kinases which phosph

nes back to the cell membrane (PubMed:29030480).||[Isoform 2]: Guanine nucleotide exchang

is a transcriptional regulator for numerous genes, including some genes involved in cancer pro

erone that stimulate the ATP-dependent activity (By similarity).

ided by RefSeq, Apr 2017]

accessibility of target mRNAs. Sequence-specific RNA-binding factor that binds specifically to
o a lesser extent for lysophosphatidylethanolamine (lyso-PE). Does not display glycerophospho
F2A.

atory chain. F-type ATPases consist of two structural domains, F(1) - containing the extramem

NA polymerase II as part of capped and polyadenylated primary transcripts (pri-miRNAs) that c
nisfolded glycoproteins into the cytosol where they are ubiquitinated and degraded by the protea

rgeting to the cell membrane and synapses and modulates their gating properties by slowing th
], Dec 2017]

ons mediating its different effects. Acts by binding (via its C-terminal domain) to sequences rela
locus transcription regulation by being recruited by NFE2 (PubMed:17707229). Plays an impor

1' of NDUFS7 (PubMed:27226634). May also have methyltransferase activity (Probable).
intracellular junctions. This protein contains a conserved sequence, called the SH3 (src homo
nbrane and may be directly involved in trafficking to lysosomes. In concert with the BLOC-1 cor
NA polymerase II as part of capped and polyadenylated primary transcripts (pri-miRNAs) that c

primary miRNA transcripts (pri-miRNAs) to release precursor miRNA (pre-miRNA) in the nucleu
CCL5/RANTES, may play a role in neuron survival through activation of a downstream signalir

/or near replication origins and moves along DNA with the replication fork (By similarity). Has 3
CN8A fast inactivation.

), PubMed:31542297). Specifically mediates deacetylation of histone H3 at 'Lys-18' (H3K18Ac)
or regulating the expression of genes involved in immune and inflammatory responses (PubMe

ut its expression is lost in multiple cancer cell lines of various tissue origins. It inhibits tumor ce
ties, changing chromatin structure by altering DNA-histone contacts within a nucleosome in an
olic Fe-S biogenesis. Transfers electrons from NADPH to the Fe/S cluster of CIAPIN1.
endent CDKN1A expression.

mediates heterophilic synaptic cell-cell contact and postsynaptic specialization. Receptor proba

ypes of genes. Recruits TBP and BRF2 to the U6 snRNA TATA box.

patched of smoothened (SMO). Required for the accumulation of KIF7, GLI2 and GLI3 in the c

\ which is thought to be transferred to the NXF1-NXT1 heterodimer for export (TAP/NXF1 path

teins.

05). Upon DNA-binding, it locally modifies DNA conformation by wrapping the DNA around itse

oth am and pamp inhibit aldosterone secretion by direct adrenal actions. In pituitary gland, bot

ytes by inactivating RAB32 and RAB38. Inhibits RAB32 and RAB38 activation both directly by p

ulation of cardiac muscle contraction through modulation of L-type calcium currents. Is a recept

tegrity and trafficking, cell survival through NF-kappa-B activation, cell migration, cell differentia

ferentiation. It may act a molecular sponge for microRNAs. Alternatively spliced transcript varia

in the BRCA1-BRCA2-dependent HR pathway. Upon DNA damage, BCDX2 acts downstream

the BRCA1-BRCA2-dependent HR pathway. Upon DNA damage, CX3 acts downstream of R

osome (APC/C), a cell cycle-regulated ubiquitin ligase that controls progression through mitosis

initiation step of the glycylation reaction by adding a single glycine chain to generate monoglyc

ly. It also is required for ATP-dependent interactions of both U1 and U2 snRNPs with pre-mRN

nduced suppression of cell proliferation. Binds specifically to the 3'-untranslated regions (3'-UT

5'. Specifically phosphorylates and inactivates cyclin B1-complexed CDK1 reaching a maximur

biosynthesis of isoprenoids and sterol synthesis.

adian rhythms in gene expression, which are translated into rhythms in metabolism and behavi

ecycling of proteins to the correct membrane domains. The tripartite complex composed of LIN

ction in pre-mRNA splicing, plays also a role in the regulation of translation. Isoform 5 has a re

0). PcG PRC1 complex acts via chromatin remodeling and modification of histones; it mediates

s and pituitary gland (By similarity). In the ovary, it mediates the effects of Phoenixin-14 and Ph
r of cell proliferation. However, an alternative transcript produced at this locus was also found t

.||Endoribonuclease involved in various biological functions such as cellular inflammatory respo

e CENPA-CAD (nucleosome distal) complex and may be involved in incorporation of newly syn
NA polymerase II as part of capped and polyadenylated primary transcripts (pri-miRNAs) that c

anisms that enable SR proteins to control RNA splicing and can cause redistribution of SR prot
NA polymerase II as part of capped and polyadenylated primary transcripts (pri-miRNAs) that c

o mono-, di- and trimethylate 'Lys-27' of histone H3 to form H3K27me1, H3K27me2 and H3K2
26297806). Acts as a molecular scaffold facilitating the interaction of PLK4 and CENPJ, 2 mole
NA polymerase II as part of capped and polyadenylated primary transcripts (pri-miRNAs) that c

cle myosin IIa, positively regulates actin stress fibers assembly and stabilizes focal adhesions.

1/MASH1 and TCF3/E47, allowing ASCL1 and TCF3 to up-regulate transcription in its presenc
1/MASH1 and TCF3/E47, allowing ASCL1 and TCF3 to up-regulate transcription in its presenc
65-p50 heterodimer and the NF-kappa-B p50-p50 homodimer. Seems also to activate NF-kapp
-20' (H4K20me3) and thus regulates transcription and maintenance of genome integrity (PubM

ermatogenesis during sexual maturation (By similarity).

sequent hydrolysis of the triphosphate.

cis double bond at the delta-9 position into fatty acyl-CoA substrates including palmitoyl-CoA a

456, PubMed:26114596). Also involved in drug metabolism, as it can metabolize eldecalcitol (E

ding sequence (CSBS), at nuclear matrix- or scaffold-associated regions. Thought to recognize

omplex II, CII), ubiquinol-cytochrome c oxidoreductase (cytochrome b-c1 complex, complex III,

tiated multiciliated cells (G1/0) and not in S phase. Essential for centriole amplification and is r
ulates tumor suppressor genes such as tumor protein p53. Expression of this locus may be a m
adian rhythms in gene expression, which are translated into rhythms in metabolism and behavi

extracellular stimuli such as proinflammatory cytokines or physical stress leading to direct activ
m the TOM complex to the sorting and assembly machinery (SAM complex) of the outer memb

d probably in a pathway involved in stress response and maintenance of cell wall integrity. Fun
adian rhythms in gene expression, which are translated into rhythms in metabolism and behavi

nancement of GR transactivation. Involved in TNF-alpha-induced activation of NF-kappa-B via a

e and very low activity, if any, toward N(1)-acetylspermidine (PubMed:28516954). Histone deac

opressor CATR sequence of the COX5B promoter (By similarity). Binds with high affinity to RNA

s is increased in proliferating tissues, including certain tumors such as colorectal adenomas ar
endent on the rate of association of SL1/TIF-IB with the rDNA promoter. SL1/TIF-IB is involved

ssion of TP53 transactivation and apoptotic functions.
mediated tumorigenesis. May play a role as transcriptional regulator.
3,5-bisphosphate (PtdIns(3,5)P2) (PubMed:10764818) (By similarity). Specific for lipid substrate
ia the non-canonical pathway. Could act in a receptor-selective manner.
of DNA is wrapped in repeating units, called nucleosomes. The linker histone, H1, interacts wit
quired for the activity of a PDX1:PBX1b:MEIS2b complex in pancreatic acinar cells involved in t
4) genes on the short arm of chromosome 16. A duplicate read-through locus also exists betwe
atory chain. F-type ATPases consist of two structural domains, F(1) - containing the extramem
ssemble the pre-replication complex necessary to initiate DNA replication. Does not bind histo
RK1/ERK2 signaling.

ı of downstream tyrosine phosphorylation events and activation of PLCG2 (PubMed:16174766,
NA polymerase II as part of capped and polyadenylated primary transcripts (pri-miRNAs) that c

during endosome sorting. Involved in endocytic trafficking of EGF. Involved in transferrin recep

recognition of antigen by T cell antigen receptor (TCR). May also regulate TCR-induced negat

:tion, interfering with ATAD3A interaction with matrix nucleoid complexes.

the insulin receptor (INSR). Involved in the MET/HGF-signaling pathway (PubMed:29408807).
thin the heterodimeric GABA receptor, only GABBR1 seems to bind agonists, while GABBR2 r

ıe nucleus. Could be involved in the hypothalamic regulation of body weight (By similarity). Cor

carboxylation activity toward glutamate.

NA polymerase II as part of capped and polyadenylated primary transcripts (pri-miRNAs) that c

tochore microtubule attachments (PubMed:23891108).
9991, PubMed:8702602, PubMed:9425121, PubMed:10358058, PubMed:14709560, PubMed:1

(PubMed:23161870). May be involved in regulating rapid P4 signaling in the nervous system (
sels and transports LPC into the brain (By similarity). Transport of LPC is essential because it c
2016]

ıp system are located on the gene product, which is glycosylphosphatidylinosotol-anchored to t

endent on the rate of association of SL1/TIF-IB with the rDNA promoter. SL1/TIF-IB is involved
osis. Modulates the RAD9A interaction with BCL2 and thereby induces DNA damages-induced
MP1, MMP13, MMP14, MMP19 and ERP29 (PubMed:25171405). Probably plays a role in plat
(H2AZK8me1) (PubMed:23324626). Required for the maintenance of embryonic stem cell self

adian rhythms in gene expression, which are translated into rhythms in metabolism and behavi
CO1 mRNA translation and increases mitochondrial complex IV assembly and activity (PubMe
r expression of E-cadherin/CDH1, N-cadherin/CDH2 and R-cadherin/CDH4 on target cells.
iction with INPP5D/SHIP1. May modulate ABL1 function (By similarity).
nd PKD2 are required for channel activity (PubMed:27214281). Involved in renal tubulogenesis
HOA, leading to its degradation by the proteasome (PubMed:19782033) Degradation of RHOA

ed:30506946).
andidate for nonsense-mediated mRNA decay (NMD), and is therefore unlikely to produce a pr
P1 ligands, such as PSAP. Increases cellular levels of GTP-bound ARF6.
s a ribosomal protein that is a component of the 40S subunit. The protein belongs to the S28E

n of intracellular calcium concentration and nitric oxide production leading to regulation of vasc
as lipid-binding chaperone with specificity for a diverse subset of myristoylated proteins. Specif

structure of the encoded transcript mimics glucocorticoid response element (GRE) which mea

transcript can also act post-transcriptionally to regulate the stability of mRNAs during the innat

ATP-dependent DNA helicase that participates in various functions in genomic stability, includir

out also in the presence of supercoiled DNA. Can induce apoptosis in a p53/TP53 dependent n

:oactivators including isoform 1, NCOA6 and CITED1 (PubMed:11443112). Regulates centriol

of oxo-acids as amino-group acceptors, with a preference for 2-oxoglutarate, 2-oxocaproic aci

atory chain. F-type ATPases consist of two structural domains, F(1) - containing the extramem

:ruitment of EYA3 to the SIX1-DACH1 complex. Transcriptional activation seems also to involv

NA polymerase II as part of capped and polyadenylated primary transcripts (pri-miRNAs) that c

ι complex that has guanine nucleotide exchange factor (GEF) activity and regulates autophagy
neural development a switch from a stem/progenitor to a post-mitotic chromatin remodeling r

of asymmetric distribution of phospholipids. Phospholipid translocation seems also to be implic
:TTCTGGGAAGA-3' of the acute phase response element (APRE). Transactivates several pro
NA polymerase II as part of capped and polyadenylated primary transcripts (pri-miRNAs) that c

ovided by RefSeq, Aug 2013]
NA polymerase II as part of capped and polyadenylated primary transcripts (pri-miRNAs) that c

NA polymerase II as part of capped and polyadenylated primary transcripts (pri-miRNAs) that c

e for the Fanconi anemia complex by associating with E3 ubiquitin-protein ligase FANCL and c

ion of pancreatic genes involved in insulin secretion. Regulates the expression of the glucose t

expansion within the human lineage and has expanded, to a lesser extent, among primates in

[illegible]5301, PubMed:15041462, PubMed:18577768, PubMed:19965576, PubMed:20972997, PubM[illegible]

:28827538).

NA polymerase II as part of capped and polyadenylated primary transcripts (pri-miRNAs) that [illegible]

plasma membrane. The channel is permeable to a range of divalent cations including calcium,
pe and the expression of muscle-specific genes. May also phosphorylate PPP1R12A and inhib[illegible]
nation of 'Lys-11'-linked polyubiquitin chains, a specific ubiquitin-linkage type mediated by the [illegible]

all GTPases. May enhance the guanine nucleotide exchange factor (GEF) activity of DOCK1 ([illegible]

ase of the cell cycle (PubMed:9635433, PubMed:14993212, PubMed:24064211). Its destructio[illegible]

NA polymerase II as part of capped and polyadenylated primary transcripts (pri-miRNAs) that [illegible]
ess through mitosis and normal cytokinesis. Does not dephosphorylate phospho-threonines in [illegible]

As (snRNAs) (Gu et al., 2005).[supplied by OMIM, Mar 2008]

tor subunits and shaker-type potassium channels. Required for synaptic plasticity associated w

position. In parallel, may have a minor phospholipase 2 activity acting on acyl groups in positior

scription factors, such as MSX2, FAST2, SMAD2 and SMAD3 during hematopoietic cell differe

NA polymerase II as part of capped and polyadenylated primary transcripts (pri-miRNAs) that c

rotein-dsDNA filaments (By similarity). May play also an essential role in telomere length maint

nay play a role in rRNA maturation. This gene is co-transcribed with the ribosomal protein L39
AD to complete this process. The protein encoded by this gene is an inhibitor of ERAD, functior

H(2)S metabolism performed by the sulfide:quinone oxidoreductase (SQOR) which catalyzes

ng activity, can protect cells from oxidative stress by reducing caspase 3 activity during apopto
n. [provided by RefSeq, May 2015]

ability and bipolar disorder in human patients. [provided by RefSeq, Sep 2016]

component of the SMC5-SMC6 complex, a complex involved in repair of DNA double-strand

helial cells (By similarity).

NA polymerase II (pol II). May be a candidate proto-oncogene in head and neck squamous cell

both an H/ACA box and a C/D box, and is thought to guide the pseudouridylation of residue U

RNPs) involved in pre-rRNA processing and modification. snoRNAs of the box C/D class, like S

of the 5'-AGGTCA-3' motif. Represses transcriptional activity of LHCG.
ought to function in cell-cell recognition and adhesion. Alternative splicing yields isoforms with u

y promoting FAK tyrosine phosphorylation and stress fiber turnover.
Asn-X-Ser/Thr consensus motif in nascent polypeptide chains, the first step in protein N-glyco[illegible]
NA polymerase II as part of capped and polyadenylated primary transcripts (pri-miRNAs) that [illegible]
NA polymerase II as part of capped and polyadenylated primary transcripts (pri-miRNAs) that [illegible]
severin), these proteins do not sever actin filaments.
[illegible] cysteines. Most LY6 proteins are attached to the cell surface by a glycosylphosphatidylinosit[illegible]
[illegible] protein kinase activity and preventing phosphorylation of GLI proteins (GLI1, GLI2 and/or GLI[illegible]

associated with early-onset mitochondrial encephalopathies. Alternative splicing of this gene re[illegible]

[illegible]rotein complex that regulates promoter activation by orchestrating a calcium-dependent releas[illegible]

[illegible] to promote neural progenitor self-renewal, possibly by down-regulating neuronal differentiatio[illegible]
nechanism (PubMed:25320081). Also transports guanine but not adenine (deoxy)nucleotides ([illegible]
ity of Rho GTPases including RHOA, CDC42 and RAC1 (PubMed:27917469).

s oleoyl-CoA as the acyl donor. Lysophospholipid acyltransferases (LPLATs) catalyze the reac[illegible]
[illegible]and break sites and promote the assembly and/or stability of the NHEJ machinery.

ınd BMP10. Involved in the regulation of nociception, acting as a modulator of the interaction b
BMP) by regulating expression of genes required for the oogenic programs, which are represse
in DNA replication. The DRTF1/E2F complex functions in the control of cell-cycle progression f
l CSFIR) demonstrate its ability to promote growth and specifically neurite outgrowth, and cell s
s the activity of L-type calcium channels that contain CACNA1C as pore-forming subunit and ir

moting APC-FZR1 dependent degradation of mitotic cyclins and subsequent exit from mitosis.
l-mesenchymal interactions during the formation of the mammary glands and teeth. Required f

es X inactivation in females. This gene does not make a protein.[provided by RefSeq, May 201(

been found for this gene, however, the full-length nature of some variants is not known. [provi

omplex II, CII), ubiquinol-cytochrome c oxidoreductase (cytochrome b-c1 complex, complex III,
the development of human prostate cancer, from prostatic intraepithelial neoplasia to invasive

opment. Required for osteoblast differentiation and sonic hedgehog/SHH signaling response. E
lexes activity.
s instead stabilized by a 3' triple helical structure. This transcript is retained in the nucleus wher

ıeric guanine nucleotide-binding protein (G protein) (PubMed:18045877). Involved in the regula

d by B lymphocytes. In the recognition phase of humoral immunity, the membrane-bound imm
he proteins mediate signal transduction events that play a role in the regulation of cell develop
of the receptor is referred to as forward signaling while the signaling pathway downstream of th
ı), rather than under standard conditions. Acts as a sensor involved in energy homeostasis: reg
1 acts as a motor for the intracellular retrograde motility of vesicles and organelles along micro

ly regulate cell division. [provided by RefSeq, May 2016]
:ion in neurons and neurite outgrowth (By similarity). Maintains also meiotic arrest in oocytes (B
ɔhatidylcholines on the surface of high and low density lipoproteins (HDLs and LDLs) (PubMed:

he elongating chondroitin polymer. Specific activity is much reduced compared to CHSY1.
)NA replication by promoting homologous recombination-mediated repair of replication fork-ass
of the cell cycle, which may potentiate cells for Wnt signaling (PubMed:27821587). May regul

elongation factor B (P-TEFB), which is proposed to facilitate the transition from abortive to proc
cts then as a sliding clamp platform on DNA for several proteins involved in long-patch base e

tin-protein ligase complex which mediates the ubiquitination and subsequent proteasomal deg
ow a 30-fold higher chemotactic activity.

ɔRNPs) involved in pre-rRNA processing and modification. snoRNAs of the box C/D class, like
plex is required to target membrane protein cargos into vesicles assembled at cell bodies for d
nd-dependent transcriptional activation by nuclear hormone receptors (PubMed:12215545, Pu
on gene 3 protein: Inhibitory receptor on antigen activated T-cells (PubMed:7805750, PubMed:

te and lysophosphatidate (PubMed:17590538). Phospholipid phosphatases are involved in bot
ɔme 3. This gene acts as a tumor suppressor, and plays a role in regulating the migration of tu
||Apical membrane anion-exchanger with wide epithelial distribution that plays a role as a com

nction of NK cells (PubMed:8421688).

activates a signaling cascade that leads to increased PPARA activity, and ultimately to increa

in the assembly of focal adhesion (PubMed:15004028).

ort signal. These 2 signals seem absent in EBLN-2 supporting an unrelated function in Human

; phosphorylation. Plays a critical role regulating PDPK1 membrane translocation in response t
euronal, vascular and epithelial development. Binds promiscuously Eph receptors residing on a

similarity).||Regulates APAF1 expression and plays an important role in the regulation of stress-

NA polymerase II as part of capped and polyadenylated primary transcripts (pri-miRNAs) that c

by CREB3 and reduces the amount of CREB3 in the cell. Able to down-regulate expression of
peptide FF potentiates and sensitizes ASIC1 and ASIC3 channels.

ted in the regulation of exocytosis.
of the bacteria to lung cell lines. Reduction of levels of KRT10 keratin decrease adherence, ov
ntigen-presenting cells and the T-lymphocyte rosetting with erythrocytes. In addition, the LFA-3/
ccelerates the intrinsic rate of GTP hydrolysis of RAC1 or CDC42, leading to down-regulation o

adenosine, and so contributes indirectly to cellular signaling events. Acts as a positive regulat
nd appear to be involved in cargo selection and vesicle formation. AP-2 is involved in clathrin-c
s well Human cytomegalovirus (HCMV) infection.||Broad specificity aminopeptidase which plays

gamma-tubulin redistribution (By similarity).
nine metabolism is a critical regulator of innate and adaptive immune responses. Seems to be i

gene is located within a cluster of similar arylsulfatase genes on chromosome X. A related pse

her with MAF, represses the transcription of genes under the control of the NFE2L2 oxidative s
arvation in the mammary epithelial cell line HC11 (By similarity).
ls and negatively regulates gene expression of proteins that promote the egress of tissue-resid
s. Participates in signal transduction stimulated by growth factor receptors, cytokine receptors,
optotic process in a BAX- and BAK1-dependent manner or in a BAX- and BAK1-independent r
protein synthesis (PubMed:25106868, PubMed:14981510). Acts as a 3'-untranslated region (U

which increases the stabilization of actin filament structure. In muscle tissues, inhibits the actor

or nuclear structures through potential interactions with actin. May bind DNA.
ytically cleaves CPEB3 following neuronal stimulation which abolishes CPEB3 translational rep
rol regulatory element binding proteins (SREBPs) between the basic helix-loop-helix leucine zi
roteins (CAVIN1/2/3/4) to the caveolae (PubMed:19262564). Interacts directly with G-protein al

cific glycans, allowing stem cells to bind to lectins expressed by stromal cells or other marrow c

yzes ATP and ADP equally well.
36). Participates thereby in a wide variety of cellular functions including the activation, recirculat
complex until its release by HLA-DM in the endosome.||Plays a critical role in MHC class II an

s a regulator of neural stem cells quiescence by mediating anchorage of neural stem cells to ep
pes (PubMed:21269602). This cadherin may play a important role in endothelial cell biology thr

is a negative regulator of neural cell growth.

s produced by lysosomal proteinases (PubMed:11054422, PubMed:19038966, PubMed:19038

l insulin. Type V collagen is a key determinant in the assembly of tissue-specific matrices (By s

ATN(9)CCAC[GA]-3') when NF-Y is bound to ERSE.
of proinflammatory chemokines, and thereby plays an important role in innate immunity and in i

f cross-linked TG/thyroglobulin in the thyroid follicle lumen (By similarity). Has also been implic
r matrix degradation. Involved in the release of thyroid hormone thyroxine (T4) by limited prote

on molecule and is essential for tight junction integrity. Also involved in transepithelial migratior
237, PubMed:26997266, PubMed:27591049, PubMed:29291351, PubMed:18313383). Plays a
aspase-dependent) apoptotic and type II (caspase-independent) autophagic cell deaths signal,
TP-dependent RNA helicase, able to unwind both RNA-RNA and RNA-DNA duplexes. Possess

ty toward phosphotyrosine, phosphoserine and phosphothreonine residues.

able of mediating S1P-induced cell proliferation and suppression of apoptosis.
protein kinase A (PKA). Binds to the erythrocyte membrane glucose transporter-1 SLC2A1/GLU
n of the receptor is referred to as forward signaling while the signaling pathway downstream of

688). Required for the function of REG3A in regulating keratinocyte proliferation and differentia
n-activated protein kinase (MAPK), I-kappaB kinase/NF-kappaB and Rho (PubMed:28445455).
na membrane and soluble forms exhibit post-proline cleaving endopeptidase activity, with a ma

n of pancreatic beta-cells. Appears to modulate glycerol gluconeogenesis in liver. Important re
D2 and SMAD3 with the activin receptor complex, thereby blocking the activin signal. May mo
skeleton. Interaction with FLNA may allow neuroblast migration from the ventricular zone into t
display structural functions at the Z lines in muscle cells. Critical for normal myogenesis and fo
nbryonic development. Promotes proliferation, survival and migration of endothelial cells, and r
and axon guidance. Inactive FYN is phosphorylated on its C-terminal tail within the catalytic d

ubMed:23084921). Plays a role in Vaccinia virus cell entry by apoptotic mimicry (PubMed:2150
against different pathogen classes.
es to autophagolysosomes, providing broad host protection against different pathogen classes

einyl-glycine, which is hydrolyzed to cysteine and glycine by dipeptidases. In the presence of hi
ed:8717357, PubMed:15302677, PubMed:16144831, PubMed:22715885, PubMed:25445488,
GM2 degradation. It stimulates only the breakdown of ganglioside GM2 and glycolipid GA2 by b
:12515866, PubMed:16787920, PubMed:16705036, PubMed:23762476, PubMed:27084452).
s transmembrane signaling systems. The G(i) proteins are involved in hormonal regulation of a
zation, and signaling events leading to their down-regulation. Phosphorylates a variety of GPC
tasis that acts by inhibiting the activity of the ubiquitin-conjugating enzyme UBE2N/Ubc13, the

rin-C plays an important role in regulating the stability of red cells.
the predominant thrombin inhibitor in place of antithrombin III (AT-III). Also inhibits chymotryps

so involved in the transcription regulation of genes containing, or in close proximity to A+T-rich
cted chromatin for other proteins through interactions with nucleosomal core histones and there

its role as co-chaperone. Functions as co-chaperone for HSPA1B and negatively regulates the
olayer of astrocytes. Ligand for integrins alpha-8/beta-1, alpha-9/beta-1, alpha-V/beta-3 and al
ive polyubiquitination until they associate with regulatory subunits.
78155). In neutrophils, stimulates phagocytosis probably by signaling through the IL15 recepto
GA6:ITGB4) is a receptor for laminin in epithelial cells and it plays a critical structural role in the
I4 and IL7. Constitutively binds to the ISRE promoter to activate IL7. Involved in cell cycle regu
rus (HAV) and yellow fever virus (YFV) in an exonuclease-dependent manner. May also play a

lishing action potential waveform and excitability of neuronal and muscle tissues. Inward rectifie

ninin-5 is thought to be involved in (1) cell adhesion via integrin alpha-3/beta-1 in focal adhesio

Has high affinity for the Forssman pentasaccharide (PubMed:18005988). Ligand for HAVCR2
vaccenoate) at carbon 13 to generate (11E,13Z)-octadecadienoate, likely participating in the bi

role in the aortic wall architecture (By similarity).

deamination of trimethylated 'Lys-4' of histone H3 (H3K4me3), a specific tag for epigenetic trai
signal transduction from cell adhesion sites to the nucleus allowing successful integration of sig

onal.
obial activity and is able to permeabilize different ions through liposomal membranes.||Lactofer
300 repressor SNIP1. May act synergistically with SMAD4 and YY1 in bone morphogenetic prot
ancing hematogeneous tumor spread. Could be an adhesion molecule active in neural crest ce
muscle development, but also in neuronal differentiation and survival. Plays diverse roles in th
ling to hepatocyte growth factor/HGF ligand. Regulates many physiological processes including

the DNA (PubMed:20159561, PubMed:20471948, PubMed:25417107, PubMed:27105114, Pu
ted viral Tat protein, leading to a decrease in neuronal Tat's mediated neurotoxicity (PubMed:1

also play a role in neovascularization or angiogenesis. Hydrolyzes collagen type IV, laminin, ni

rotein.||Mediates the endocytosis of glycoproteins by macrophages. Binds both sulfated and no

or MAX.

dian rhythms in gene expression, which are translated into rhythms in metabolism and behavio

NT3A-mediated signaling (PubMed:25029443, PubMed:27162350). In inner ear, crucial for spir
this ligand-dependent signaling occurs through a G(q)-mediated pathway in melanocytic cells.
ene regulation. Synthesizes higher oligomers of 2'-5'-oligoadenylates (2-5A) from ATP which th

formation and plasticity. Acts as downstream effector of the small GTPases CDC42 and RAC1
crine peptides (PubMed:12699694). Alpha-amidation involves two sequential reactions, both of
nitochondrion, including the monitoring of local cAMP levels at the outer mitochondrial membra
ses fat metabolism in response to prolonged fasting, and as adaptation to a high-fat diet. Plays
)0 plays a critical role in TEM and is required for efficient trafficking of PECAM1 to and from the
of asymmetric distribution of phospholipids. Phospholipid translocation seems also to be implic

2). Cleaves ASARM peptides between Ser and Glu or Asp residues (PubMed:18597632). Reg

of phospholipids between triglyceride-rich lipoproteins themselves (PubMed:7654777, PubMed
(By similarity). Plays a role in RHOA activation and subsequent changes of the actin cytoskelet

TP-dependent proteolytic activity. This subunit is involved in antigen processing to generate cla
3867277). Plays a role in disulfide bond formation in a variety of extracellular proteins (PubMe
lation of SLP76 and LAT and mobilization of Ca(2+).||Isoform 1 plays a critical role in signaling
seems to affect the substrate specificity of the first one.

osite effects on PTPRS oligomerization and regulation of neurite outgrowth. Contributes to the
sses, phagocytose of apoptotic cells and epithelial cell polarization. Augments the production c
vith distinct downstream effectors. Acts as a GTP sensor for GTP-dependent exocytosis of den
prenylation. Functions in synaptic plasticity by contributing to the induction of long term potenti
-6-phosphate receptor (M6PR). Also required for protein transport from the Golgi apparatus to

acellular calcium ions (PubMed:9837883, PubMed:10587439). Exhibits a chemotactic activity f

6, PubMed:20068036). The ligand-receptor pair CCL20-CCR6 is responsible for the chemotax
ve and Gram-negative bacteria (90-fold-higher when compared to CXCL5 and CXCL7).
dent and CX3CR1-independent manner. In the presence of CX3CR1, activates integrins by bin
aces during the initial steps in inflammation. Critical for the initial leukocyte capture.
lar beta-catenin levels (By similarity). Has antiproliferative effects on vascular cells, in vitro and

in the organization of actin filament bundles and the formation of microspikes, membrane ruffle

ich enhances transcriptional activation (By similarity). Involved in the differentiation of naive CD

kinases, G protein-coupled receptors as well as cytokine receptors. Participates in signaling pat

ı (PubMed:12711699). Appears to be involved in myosin II assembly. May modulate myosin II

4, MMP-15, MMP-16 and MMP-19.
nembers may be modulated by association with dominant-negative AES. Essential for the tran
mTORC1, a signaling complex promoting cell growth in response to growth factors, energy le

ading to its degradation by the proteasome, thereby regulating the actin cytoskeleton and cell n
at promotes ubiquitination of target proteins, such as MAP3K14. The TRAF1/TRAF2 complex r
or normal antibody isotype switching from IgM to IgG. Plays a role T-cell dependent immune re
pa-B and probably JNK. Seems to be involved in apoptosis. Plays a role in mediating activatio
of influenza A virus NS1 protein.
component of the CTLH E3 ubiquitin-protein ligase complex (PubMed:29911972). In vitro cata
PubMed:8643456, PubMed:1532799). Glucosylceramide is the core component of glycosphing

he nucleus, endoplasmic reticulum, and mitochondria, either laterally or terminally.
n of Tau phosphorylation (By similarity).
-specific organelle translocations (By similarity).
mal chondrocyte maturation and for normal bone mineralization during embryonic bone develo
end. A truncated copy of this gene is found on the Y chromosome at the pseudoautosomal bou
regulation of NF-kappa-B activation and apoptosis. Inhibits NF-kappa-B activation triggered by

ulates transcription of ARNTL/BMAL1, CLOCK and CRY1 by acting as a coactivator for RORA
Regulates actin cytoskeletal reorganization via phosphorylation of PPP1R12C and MYL9/MLC
equent phosphorylation of MAPK1 and MAPK3. Promotes production of proinflammatory cytoki

cates the paranodal region of the axo-glial junction. In association with contactin involved in th
lay a role in mitochondrial DNA maintenance. A pseudogene of this gene is located on the sho
ssociates with the 40S ribosome and facilitates the recruitment of eIF-1, eIF-1A, eIF-2:GTP:me

steoblast differentiation and/or osteoblast activity in bone (By similarity).
20, PubMed:11114884, PubMed:22099306, PubMed:27422871). Binds RNA containing 5'-tern
ty may be modulated by binding to the decoy receptors TNFRSF10C/TRAILR3, TNFRSF10D/T

lytic activation which initiates the subsequent cascade of caspases (aspartate-specific cysteine

e organization of a multimolecular complex that would interface incoming signals to the reorga

ruitment to focal contacts and cell migration.

/ modifier of the MAP1 LC3 family (PubMed:16286508, PubMed:20168092, PubMed:24128730

atic cells.
etrograde trafficking pathway for recycling proteins, such as mannose 6 phosphate receptor (M
skeleton. Induces lamellipodia independent of its GEF activity.
'N12 and WAS and allow PTPN12 to dephosphorylate WAS. Has the potential to physically co
and the ketone bodies acetoacetate, beta-hydroxybutyrate and acetate (By similarity).
al.
al in mitogen-poor environments. Inhibits the SHH and WNT1 pathways, thereby enhancing ad

blast into myofibroblast and myofibroblast ameboid migration. Increases retinoic-acid regulatio
SMAD4 to the vicinity of the receptor complex and facilitate its interaction with receptor-regulat

on events. Suppresses the integrin-induced tyrosine phosphorylation of paxillin (PXN). May pla

omatin reader protein that recognizes and binds histone H3 trimethylated at 'Lys-9', dimethylate
ed glycans and within mucin-associated glycans that can ultimately serve as SELL ligands. SE

ors, it aids the coupling of surface receptors to intracellular calcium release. Isoforms can be di
esponse to excitotoxic stress. Cytoplasmic MAPK8IP1 causes inhibition of JNK-regulated activ
on in Alzheimer disease. Cleaves aggrecan at the '392-Glu-|-Ala-393' site.

AH. Isoform 2 modulates SNCA monoubiquitination by SIAH1.
es. Acts as an organizer protein that allows NOX1- or NOX3-dependent reactive oxygen specie
te receptor from the late endosomes to the TGN. May also play a role in transport between the
ound small GTPases into their active GTP-bound form (PubMed:12628187, PubMed:1646446

d by ANK2 to phosphatidylinositol 3-phosphate (PI3P)-positive early endosomes, where it inacti
TRAF2 binding to LMP1 and inhibits LMP1-mediated NF-kappa-B activation. Negatively regul
form BimS. Isoform Bim-gamma induces apoptosis. Isoform Bim-alpha3 induces apoptosis po
(By similarity). Required for basal and CXCL2 stimulated serine-threonine phosphorylation of I
grity. May play a role in megakaryocytopoiesis.

ⲢEB3 and recruiting CNOT7 which leads to target mRNA deadenylation and decay (PubMed:2
amellipodia formation. The WAVE complex regulates actin filament reorganization via its intera
the activation cluster TLR2:TLR6:CD14:CD36, this cluster triggers signaling from the cell surf
mechanism. Binds to the complex neuropilin-1/plexin-1.

cally occur in proteins with high turnover rates. Immunological characteristics of this protein are

nitofusin proteins (MFN1-2).

resomes by regulating the p62-KEAP1-NRF2 signaling and particularly by modulating the ubiq
o platelet activation and/or aggregation (PubMed:14522983, PubMed:15231832, PubMed:176
s as an adapter protein in signal transduction cascades by binding to SH2 and SH3 domain-co

emethylates trimethylated, dimethylated and monomethylated H3 'Lys-4'. Acts as a transcriptio
a for pathway activation. Mechanistically, interacts with and mediates RABEP2 centrosomal lo

SH (PubMed:16940157). Acyl-coenzyme A thioesterase 2/ACOT2 displays higher activity tow
cted and transformed cells that are characterized by high levels of phosphorylated metabolites,

nals carried by cAMP (By similarity).

F1. May be involved in translational regulation.

brane.
doplasmic reticulum membrane into vesicles and the selection of cargo molecules (By similarit
ulation.
nes mitochondrial motility. Crucial for recruiting OGT to the mitochondrial surface of neuronal p
tion with soluble defense collagens. May play a role in intercellular adhesion.
oting recruitment of the endoplasmic reticulum to endosome tubules for fission (PubMed:30220
ated H3 'Lys-9', with a weaker activity than KDM4A, KDM4C and KDM4D. Demethylation of Ly

cting with the TGF-beta receptor ALK1. When overexpressed, induces the formation of cellula

ı heart development.

ǀ triglyceride, phosphatidylcholine and phosphatidylethanolamine biosynthesis in the reticulum ‹

causing mitotic arrest (By similarity). It also regulates mitochondrial function by mediating the u

529). Promotes in vitro the ability of TERT to elongate telomeres (PubMed:12676087, PubMed
ent basal repression machinery in Shh signaling by increasing cAMP levels, leading to promote
enols. May have a transcriptional activatory activity via its association with alpha-tocopherol. Pr
ed exons during erythropoiesis (By similarity). RNA-binding protein that seems to act as a core

ose development (By similarity). Binding to class 4 semaphorins promotes downstream activati
in the migration of cortical neurons during brain development via its interaction with UNC5D. M
regulate ER Ca(2+) homeostasis (PubMed:16908669). Plays a critical role in oogenesis (PubM

talyzes N-terminal pyroglutamate formation. In vitro, catalyzes pyroglutamate formation of N-te
ng cell-associated C-terminal fragment which is later released by gamma-secretase. It has also

02554, PubMed:23601106, PubMed:22056990, PubMed:24336198, PubMed:26294762, PubM
ponses, such as endothelial cell sprouting and migration in the forebrain and neural tube, and ‹

s differentiation of T helper 1 cells (Th1).
linking these intracellular compartments (PubMed:25447204). The ORP3-VAPA complex stimu
ic clearance of pathogenic bacteria. Isoform 4 binds the membrane surrounding Salmonella ar

alpha subunits, thereby driving them into their inactive GDP-bound form (PubMed:15096504).

H1.0 promoter is enhanced by interaction with RB1. Disrupts the interaction between DNA anc

ertebrate development, where they locally inhibit Wnt regulated processes such as antero-post

euronal apoptosis, including apoptosis in response to amyloid peptides derived from APP, and
rogenic factor contributing to the pathogenesis of renal fibrosis through fibroblast activation.
ondrial apoptotic pathway, promotes caspase-8-dependent proteolytic maturation of BID indep

7729611). Also functions as a nucleolar sensor that regulates the activation of p53/TP53 in res

able of inducing nuclear DNA damage. Displays an increased activity relative to isoform 1.||Co

owth or actin cytoskeleton remodeling by binding to both SRC and PIP2. May function as a sig

GKAP/PSD-95 and Homer, respectively, and the actin-based cytoskeleton. Plays a role in the
phate (dTMP).
CP by protein kinase 2 (CK2). Appears to target ATM to the plasma membrane. Appears to als
oression (PubMed:14621294). Regulates SNCA expression in primary cortical neurons (PubMe

PubMed:27018888).
mperatures higher than 52 degrees Celsius; is not activated by vanilloids and acidic pH.
the end of mitosis. Plays a role in endocytosis via clathrin-coated pits, but also clathrin-indepen

andins, retinoids, lipids and xenobiotics. [provided by RefSeq, Apr 2016]
tivate fatty acids from exogenous sources for the synthesis of triacylglycerol destined for intrac

not act as a peptide transferase that transfers GalNAc onto serine or threonine residue on the

Med:30970241). Mediates the formation of endoplasmic reticulum-lipid droplets (ER-LD) conta
of a lumenal glucose-6-phosphatase. May not play a role in homeostatic regulation of blood gl
ch has Notch. The gamma-secretase complex is an endoprotease complex that catalyzes the i

by exchanging bound GDP for free GTP.
CDC42 and Rac to generate additional structures, increasing the diversity of actin-based mor
C1-TSC2 complex and the GTPase RHEB. Plays an important role in responses to cellular ene
pendent degradation (ODD) domains (N-terminal, NODD, and C-terminal, CODD) of HIF1A. Al
By similarity).

eductase activity which may control physical membrane displacements associated with vesicle

and maintenance of repressive chromatin configuration, contributing to a subset of the SETDB

yotrophic lateral sclerosis, but studies of other populations have not been able to replicate this
MYH10 filaments, and the helicase DHX9.

ne 3'-UTR of CDKN1A transcripts, leading to maintain the stability of CDKN1A transcripts, there
ted pathway. Sustains B-cell lymphopoiesis in secondary lymphoid tissues and regulates FCEI

of ether lipids/plasmalogens and wax monoesters which synthesis requires fatty alcohols as su

ce and transcriptionally represses cyclin D1 expression. Regulates telomerase reverse transcri

n the developing nervous system. Involved in down-regulation of growth, stabilization of wiring

serving as a molecular bridge for the transfer of cholesterol from the PM to the ER (By similari

n-conjugating enzyme in the form of a thioester and then directly transfer the ubiquitin to target
ost inactive by itself (PubMed:18082604). Also activates deubiquitinating activity of complexes

nilarity).

5663). Fructoselysine-3 phosphate adducts are unstable and decompose under physiological c
ngth required for presentation on MHC class I molecules. Preferentially hydrolyzes the basic re
gger their ubiquitination and proteasome-dependent degradation. In addition, interaction with SI

control, DQC) (PubMed:30190310). FBXL17 specifically recognizes and binds a conserved deg

slocation of PRKN (PubMed:14607334, PubMed:15087508, PubMed:19229105, PubMed:1996
PK8, induces actin bundles formation and stabilization, thereby reducing actin plasticity, hence

n-coupled chloride cotransporters respectively. Activates SCNN1A, SCNN1B, SCNN1D, SGK1,

nt PAK-2p34 kinase activity and changes its localization from the nucleus to the perinuclear re
ycogen phosphorylase and enhances the rate at which it activates glycogen synthase and there
ase complex (HDAC) to the nucleolus (PubMed:16820529). Binds DNA, apparently without sec

reventing for instance the specific phosphorylation of PKCs and thereby controlling cell migrati

of MDM2.

for segmental gene expression during hindbrain development.

differentiation (PubMed:16380219).
nvolved in the development of acute and chronic transplant rejection and in the regulation of ly
s (By similarity). Also regulates the epithelial-to-mesenchymal transition in other epithelial cells
naintenance of physiological function of outer hair cells and the protection of hair cells from aco
ns (Probable).

positive role in the TNFalpha and IL-1beta signaling pathways. Mechanistically, induces the 'ly

el to fulfill cellular energy requirements and preventing excess ROS production. While LC3s are
endritic cell (DC) migration by locally activating CDC42 at the leading edge membrane of DC (

ed for the survival of both hematopoietic and endothelial precursors during specification (By si

.

tes cell spreading. Plays a role in the angiogenic response of cultured umbilical vein endothelia

ed in phosphatidylserine and phosphoinositides and is required for the endocytosis of the trans

PubMed:27018888). Regulates the expression and the cell-associated anticoagulant activity of
ARP2, it is not able to mediate poly-ADP-ribosylation (PubMed:18851833). Catalyzes mono-AD
nd TLN1. May regulate ROCK1 kinase activity possibly involved in regulation of actin stress fib

s to regulate membrane tubulation. May be involved in a late step of receptor-mediated endocy

S family, but contains other ADAMTS domains, including the thrombospondin type 1 motif. This

ly indirectly by degradation of these ECM proteoglycans (By similarity). May act as a tumor sup

3. This gene acts as a tumor suppressor that regulates G1/S transition in the cell cycle, and ep
e domain is thought to function in the formation of stable homodimers. The encoded protein ma
reased response to inhaled glucocorticoids in asthmatic patients. [provided by RefSeq, Feb 20

ntial cell migration during brain development, possibly acting as a regulator of cell morphology a

ar trafficking of alpha-4:beta-2 and alpha-7-containing nAChRs and may inhibit their expressior
IL1B. Involved in postnatal glycogen storage and energy metabolism (By similarity). Inhibits the

olony formation in soft agar (PubMed:25349211).
n a genome-wide association study. Alternative splicing of this gene results in multiple transcrip

'141156). The complex formed with VCP has membrane fusion activity; membrane fusion activ

es at least two forms of creatine kinase, CKB and CKMT1A.

urons, neurite outgrowth and glial cell attachment. May play a key role in intracellular signaling

ays a role in the regulation of cell morphology and cytoskeletal organization.

NK activation (PubMed:22128169). Facilitates TNF-alpha-mediated recruitment of adapter pro

BBP. May be involved in regulation of gene expression during development and differentiation

inositol 1,3,4,5-tetrakisphosphate and then to inositol 1,3,4,5,6-pentakisphosphate (Ins(1,3,4,5,

fined by a 100-110 amino acid SRCR domain, which may mediate protein-protein interaction a
eric activator of BRAF; upon binding to MAP2K1/MEK1, dimerizes with BRAF and promotes BI
ysosomes to the cytoplasm. This protein has been shown to be an effective transporter of the
t allows NOX1- or NOX3-dependent reactive oxygen species (ROS) generation and ROS locali
(By similarity). Promotes neuronal adhesion (By similarity). May inhibit neurite outgrowth.
2178883). Can inhibit the activity of ANO1 (PubMed:20056604, PubMed:22946059).
sphingosine, and its phosphorylated form sphingosine-1-phosphate are bioactive lipids that me

es. Alternative splicing results in multiple transcript variants encoding different isoforms. [provid
filopodia.||[Agrin N-terminal 110 kDa subunit]: is involved in regulation of neurite outgrowth pro

autophagy, DNA damage response and apoptosis. Coordinates actin remodeling through tyrosi
RT complex is necessary for efficient transferrin receptor recycling but not for EGFR degradatio
de metabolism.
ates AMP and dAMP using ATP as phosphate donor, but phosphorylates only AMP when using
I mitochondrial, can be distinguished by their electrophoretic mobilities, kinetic properties, and s

depolarization-contraction coupling (PubMed:24755653). Is a negative regulator of endocytosis

LDLR degradation, probably reduces PCSK9 protein levels via a translational mechanism but a

apoptosis.
tion of APP-beta.
main. Plays a central role in the response to DNA damage by translocating to the nucleus and ir
ox). Inhibits trypsin, chymotrypsin, plasmin, factor XIA and plasma and glandular kallikrein. Mod
42 is a more effective reductant than amyloid-beta protein 40. Amyloid-beta peptides bind to lip
nation during cell cycle cytokinesis. Regulates apical junction formation in bronchial epithelial c
n cells. Stimulates the release of the GDP-bound but not the GTP-bound RhoB protein. Also in
endocrine regulated secretory vesicles or the ruffled border of the osteoclast, thereby regulatin
es in both telomeres and euchromatin and in vitro binds DNA quadruplex structures. May help
n and transcriptional regulation to maintain genomic stability. Plays a central role in the control o
carboxylase (E1), lipoamide acyltransferase (E2) and lipoamide dehydrogenase (E3).
pathogenesis of a subset of high-grade B cell non-Hodgkin lymphoma. The N-terminal segmen
K.
Med:29909994). Signaling via NGFR and SORCS2 plays a role in synaptic plasticity and long-
30790544).
of P.falciparum RH5 results in BSG dimerization which triggers an increase in intracellular Ca(2
ng virions to the cell membrane and linking virions to each other. The tethered virions can be ir

ynapses.

cells and normal and transformed fibroblast cells.
TGCGGT-3', within their regulatory regions via their runt domain, while CBFB is a non-DNA-bin
Receptor for SIRPA, binding to which prevents maturation of immature dendritic cells and inhib
an important role in retinal development.
31 kinase and controls the entrance into the cell cycle. Involved in initiation and maintenance of
cell cycle arrest in G1 and G2 phases. Acts as a tumor suppressor. Binds to MDM2 and blocks

Med:12176985, PubMed:17260971, PubMed:19996109). Inhibits formation of amyloid fibrils b
and proliferation of vascular smooth muscle cells and thus, has a potential role in the maintena
ed as a regulatory B-type carboxypeptidase. CPD is a homolog of duck gp180, a hepatitis B vi

DNA and histones and may scavenge nuclear material released from damaged circulating cells

ibroblasts, myofibroblasts, endothelial and epithelial cells. Enhances fibroblast growth factor-in
rins cell-adhesion properties. Can associate with both E- and N-cadherins. Originally believed t
liseases such as breast cancer and possibly Alzheimer disease.

XCR4 to IS (PubMed:20215400). Plays a role in dendritic spine morphogenesis and organizati
ynapses in the peripheral and central nervous systems and has a structural function in stabilizi
eplication protein A complex (RPA), leading to recruit DNA2 which cleaves the flap until it is too
n (PubMed:19004829). Functions as a guanine nucleotide exchange factor (GEF), which activa
PRC. Its binding to CAV1 and CARD11 induces T-cell proliferation and NF-kappa-B activation

ent RNA unwinding activity of both EIF4-A and EIF4-F.

es triggering the JAK2/STAT5 signaling cascade. In some cell types, can also activate STAT1
ognized by other nuclear hormone receptors. Involved in modulation of hormonal responses. R

eate and linoleate (PubMed:24269233). Preferentially activates arachidonate than epoxyeicosa
najor phospholipid located on the surface of VLDL (very low density lipoproteins) (PubMed:180
tivates arachidonate and eicosapentaenoate as substrates (PubMed:21242590). Preferentially
ss of elastic fiber assembly. Regulates osteoblast maturation by controlling TGF-beta bioavaila
thesis when DNA polymerase encounters the 5'-end of a downstream Okazaki fragment. It ent
oderm patterning and correct axial organization during embryonic development, normal skelet

phage function, chemotaxis, and cancer cell invasion. May play an essential role as a negativ

ɔn, degradation, or transcytosis. Also known as interceptor (internalizing receptor) or chemokine
-linked glucans (PubMed:29061980).
ıonsense mutations, as well as those that affect splicing, result in a deficiency of this enzyme. I
nflammatory responses.
extravasation as they serve as scaffolds for the display of the selectin ligand sialyl Lewis X by
ellular elastic fibers (elastogenesis) and in the development of connective tissue. Seems to be i
s then transferred to the lipoamide cofactor of the H protein (GCSH).

te, the main excitatory neurotransmitter in the brain (PubMed:30575854, PubMed:30239721, P
ɔmal storage disorder mucopolysaccharidosis type IIID (Sanfilippo D syndrome). Mucopolysacc
leading to maintain its aspartic-type peptidase activity.||Secreted protein that acts as a key regı

gen synthase (GYS1 or GYS2), EIF2B, CTNNB1/beta-catenin, APC, AXIN1, DPYSL2/CRMP2,

er complexes of SRF and PHOX1 and interacts cooperatively with PHOX1 to promote serum-ir
ɔxidation pathway is the major energy-producing process in tissues and is performed through fo
s the major energy-producing process in tissues and is performed through four consecutive rea
ılation, cell cycle progression and developmental events. Histone deacetylases act via the form
I1C gene through DNA methylation. May play a role in formation and organization of heterochro
ır endothelial growth factor, HILPDA, and other genes whose protein products increase oxygen
NLLY) as well as tumor peptide antigens including MAGE1 (EADPTGHSY), MAGEA3 (EVDPIG
ed from the cancer-testis antigen CTAG1A/NY-ESO-1, eliciting a polyclonal CD8-positive T cell

ıest in the spleen, where senescent erythrocytes are sequestrated and destroyed. Heme oxyge
cules (PubMed:12509468, PubMed:16010978, PubMed:7567451, PubMed:8264621). Single H

ıylamide (LSD). Ligand binding causes a conformation change that triggers signaling via guanir
Implicated in regulating a variety of cellular processes, including cellular growth, senescence, c

n general as heterodimer with IFNAR2 (PubMed:7665574, PubMed:10049744, PubMed:21854
24716, PubMed:7673114, PubMed:15356148). Required for signal transduction in contrast to
i apparatus and the resulting receptor-ligand complex is transported to an acidic prelyosomal c
ɔmotes cell migration.
so exhibits IGF-independent antiproliferative and apoptotic effects mediated by its receptor TMI

:mokine and mucus production at sites of allergic inflammation. In certain cell types, can signal
F-kappa-B, triggering synthesis of inflammatory mediators (PubMed:14528293, PubMed:2550(

ansferase activity and promoting the recruitment of histone methyltransferase complexes to the
27). Forms dimeric channels through which potassium ions pass in accordance with their electr
iosomes in NDC80-depleted cells (PubMed:25743205).
: type I cytokeratins consist of acidic proteins which are arranged in pairs of heterotypic keratin

etal cell but also in certain other F-actin-rich secretory epithelial cell types (By similarity).
ı the regulation of actin filament dynamics. Acts downstream of several Rho family GTPase sig

lly oxidizes biogenic amines such as 5-hydroxytryptamine (5-HT), norepinephrine and epinephı

ilarity). Contributes to phagocytic removal of apoptotic cells in many tissues. Specific ligand for
from the extracellular matrix.||Catalyzes the addition of N-acetylglucosamine (GlcNAc) in beta

ɔhalins by cleavage of a Gly-Phe bond (PubMed:17101991). Able to cleave angiotensin-1, angi
ɔnal pathways.||PEX, the C-terminal non-catalytic fragment of MMP2, posseses anti-angiogenic

ıe (CAT), contributes to the catabolism of cysteine and is an important producer of hydrogen sı
(in the margins but not the central part of spreading cells), and lamellipodial retraction; this fun(
spreading, plays an important role in cytoskeleton reorganization, focal contacts formation (in t

s (By similarity). Required for normal planar cell polarity in ciliated tracheal cells, for normal rota
ıd of actin filaments (PubMed:10519557). Has slow rate of actin-activated ADP release due to
iption and replication.
ammation, immunity, differentiation, cell growth, tumorigenesis and apoptosis. NF-kappa-B is a
ns a transcriptional activator complex with RBPJ/RBPSUH and activates genes of the enhance
ıg of the ligand.
ıd cell proliferation. Required for normal bone development. Regulates osteoblast differentiatio

e-binding proteins (G proteins) (By similarity).

minogen activator and elastase (By similarity). Inhibits the kinase activity of RAF1 by inhibiting i
of heterotrimeric G proteins assembly by trapping the preloaded G beta subunits inside the CC
omain protein HNF1A and enhances its transcriptional activity.

ntial role in pregnancy establishment by proteolytic activation of a number of important factors s

ack regulation by U-PA which cleaves it into an inactive form.
xylysine residues in -Xaa-Lys-Gly- sequences in collagens (PubMed:8621606, PubMed:10686
within these structures. Isoform PML-4 restricts varicella zoster virus (VZV) via sequestration c
ity).
lipid that regulates satiety. Receptor for peroxisome proliferators such as hypolipidemic drugs
68591). Many of the substrates contain a PxIxIT motif and/or a LxVP motif (PubMed:17498738
ubMed:9268648, PubMed:9705292, PubMed:17893144). During the S phase of the cell cycle, t
ergy-producing pathways and inhibits energy-consuming processes: inhibits protein, carbohyd

igenesis, cardiac hypertrophy, angiogenesis, platelet function and inflammation, by directly pho
tion and epithelial morphogenesis. Regulates myeloid cell differentiation through SMAD6 phos
st for ADGRG6 receptor. May play a role in iron uptake and iron homeostasis. Soluble oligome

ational gamma-carboxylation of specific glutamic acid residues by a vitamin K-dependent gamr
de proteoglycans, such as aggrecan, decorin and fibromodulin. Through cleavage of proteogly
on signaling.||Saposin-A and saposin-C stimulate the hydrolysis of glucosylceramide by beta-gl
). Requires the other members of the gamma-secretase complex to have a protease activity. M
TP-dependent proteolytic activity. This subunit is involved in antigen processing to generate cla

m 1 in increasing protein levels of PINK1.||Tumor suppressor. Acts as a dual-specificity protei
. Implicated the smooth muscle contractile response to PGE2 in various tissues.
cur (By similarity). In myoblasts, associates with tetraspanins CD9 and CD81 to prevent myotu
um response factor (SRF)-dependent gene transcription.||Non-receptor protein-tyrosine kinase
thelial-mesenchymal transition. Mediates beta-catenin dephosphorylation at adhesion junctions
obin. Beta-catenin may be a substrate for the catalytic activity of PTPRK/PTP-kappa.

dent release of fatty acids from the sn-1 or sn-2 position of glycerophospholipids (PubMed:196
d G2 phases of the cell cycle and preferentially repairs DSBs resulting from replication fork coll

quired for the biogenesis of motile cilia by governing growth and beating efficiency of motile cel
osphates and activation of MAP kinases.
e leukotriene A4 (LTA-4) into leukotriene B4 (LTB-4) (By similarity).
critical role in spermatogenesis and in intraflagellar transport processes (By similarity). May be i
7, regulates translation through RPS6 and EIF4B phosphorylation, and mediates cellular prolife

pends on the other subunit in the heterodimer (PubMed:9829974, PubMed:9751058, PubMed:
913, PubMed:11241183, PubMed:1476165). Contributes to Ca(2+) transport during excitation-

ties, changing chromatin structure by altering DNA-histone contacts within a nucleosome in an

s own chaperone activity, it may contribute to the interaction of HSC70 with various target prote
urface receptors, signaling via protein kinases leads to activation of Jak kinases (TYK2 and JA
strogen homeostasis, the sulfation of estrogens leads to their inactivation. Also sulfates dehydr
. Acts as a negative regulator of MAP3K1/MEKK1. May also act as a cell cycle regulator by act
he proteolytic release of several other cell-surface proteins, including p75 TNF-receptor, interle
ceptors. Probably acts as integral component of corepressor complexes that mediates the recr

nsor of hydrogen peroxide-mediated signaling events. Might participate in the signaling cascad
d in cell cycle regulation or in DNA replication. The TFDP2:E2F complex functions in the contro
1, TGFB2 and TGFB3 signal from the cell surface to the cytoplasm and is thus regulating a ple
ed:9792688). The tight junction acts to limit movement of substances through the paracellular s
The proteins mediate signal transduction events that play a role in the regulation of cell develop
F1B/TNFR2. This receptor mediates most of the metabolic effects of TNF-alpha. Isoform 2 bloc

cell division by controlling a set of genes required for this process. One of the activated genes
e (SIDDT). [provided by RefSeq, Dec 2009]

(MST) activity (By similarity). Together with MRPL18, acts as a mitochondrial import factor for t

ıorylate cytidine and uridine nucleoside analogs such as 6-azauridine, 5-fluorouridine, 4-thiouri

ɔly of stress fibers in favor of filopodia formation. Plays a role in the formation of cell ruffles (By
ITT-3'; some genes have been shown to contain a longer binding motif allowing enhanced bind

ədiates 'Lys-63'-linked polyubiquitination of the DDX58 N-terminal CARD-like region and may pl

l. Reelin acts via both the VLDL receptor (VLDLR) and LRP8 to regulate DAB1 tyrosine phosph

varian follicles that are not selected to ovulate. Induces ovarian granulosa cell apoptosis, possil
lependent transcriptional activation in response to activated Ras signaling.

perexcitability (By similarity). Promotes expression of the pore-forming alpha subunits at the cel
including influenza A virus, SARS coronavirus (SARS-CoV), Marburg virus (MARV), Ebola viru
ells. Has quercetin 2,3-dioxygenase activity (in vitro).
ə activity and regulates their signaling activities. The G(z)-alpha activity is inhibited by the phosp
e/S1P and ceramide 1-phosphate/C1P (PubMed:9705349, PubMed:9607309, PubMed:164673
led RNA molecules between purine and uridine residues, which critically contributes to the sup

ectional 3-alpha-, 17-beta- and 20-alpha HSD. Can interconvert active androgens, estrogens a
l cell fate. Not required for the proliferation of neural progenitor cells before the onset of neurog
359, PubMed:22864114, PubMed:23622245, PubMed:25043379). Acts as an activator of the W

grin-mediated cell adhesion and migration of airway smooth muscle cells. Suppresses cell moti
əs interaction with IGF2R.
PLGF-2 isoform of PGF.
ecognition component of a SCF-like ECS (Elongin BC-CUL2/5-SOCS-box protein) E3 ubiquitin-
hyde, acetaldehyde and propanal. Displays complete lack of activity with citral (By similarity).
RELB/IER3 survival pathway, may provide pancreatic ductal adenocarcinoma with remarkable r
n as a positive regulator of apoptosis. Downstream of NMDA receptors and CaMKK-CaMK1 sig

), and the second step is the transfer of a phosphate group from ATP to APS yielding 3'-phospl

lved in Golgi fragmentation. May be involved in phosphorylation of CDK1 on 'Tyr-15' to a lesse
ERK1 and/or MAPK1/ERK2, MAPK14, and MAPK8. Possibly involved in helper T-cell function.
similar to Ac transposable elements, but does not code for any transposase activity.
e, and the ketone bodies acetoacetate, beta-hydroxybutyrate and acetate. Functions as high-af

sion and probably in cell-cell interactions. Mediates cell matrix adhesion in developing neurons
osynthesis of all gangliosides (membrane-bound glycosphingolipids) which play pivotal roles in
MAD3 and modulating the transcriptional activity of the SMAD3/SMAD4 complex. Possibly asso

ns to the basal RNA polymerase II transcription machinery. Mediator is recruited to promoters
ne secretion of secretase-processed amyloid-beta protein 40 and amyloid-beta protein 42.||Pla

volved in innate defense mechanisms.
g HCO3(-) from blood to cell. Enhanced expression in severe acid stress could be important fo
for MHC class I molecules. Involved in the N-terminal trimming of cytotoxic T-cell epitope prec
es the ESCRT-III components to the cytoplasm for further rounds of MVB sorting. MVBs contai
basal transcription factors. Participates in the transcriptional repressor activity produced by BC
tion (PubMed:24440334). In the absence of actin, it also functions as a NADPH oxidase produc

nt mechanism.
ptors and Rap/Ras GTPases in intracellular signaling cascades. Acts also as an effector for Ra
a high Ca(2+) concentration may be necessary for the function of molecular chaperones involv
ximity of transcription start sites and may alter chromatin structure, modifying access of transcr
medium (PubMed:20236627). Plays a role in preventing tumor onset (By similarity).
-like double-membrane structure that surrounds the Salmonella-containing vacuole (SCV) durii
ed DNA.

chromosomal passenger complex (CPC) from chromosomes to the spindle midzone in anaph

ted STAT1 signaling and subsequent activation of JAK. Down-regulates SRC activity and signa
ulation, cell cycle progression and developmental events. Histone deacetylases act via the form
aggregation-prone proteins. Isoform B but not isoform A inhibits huntingtin aggregation. Has a

May have a function in the urea cycle (PubMed:11566871).

Plays also a role in the biosynthesis of retinoic acid from retinaldehyde (PubMed:11304534, Pu

r receptors or active CDC42 and RAC1 results in a conformational change and a subsequent a
d in the recruitment of the histone methyltransferase KMT2A/MLL1 and consequent increased
from its thyroglobulin (Tg) precursor.

the activity-depending refilling of readily releasable vesicle pool (RRP) (By similarity). Essentia
and vasculature (By similarity). Promotes adhesion of endothelial cells through interaction of i

including influenza A virus, SARS coronavirus (SARS-CoV), Marburg virus (MARV), Ebola viru
with EIF4A and PABPC1 may potentiate contacts between mRNA termini. May also be involvec
RAD51. Binds to single and double stranded DNA, and is capable of aggregating DNA. Also bi
rm 3 phosphorylates CAMK1D. Isoform 4, isoform 5 and isoform 6 lacking part of the calmoduli
ponse to amino acid availability through regulation of the mTORC1 signaling cascade (PubMed
P hydrolysis on the G-alpha subunits, thereby promoting their inactivation (Probable). Increase
s to daughter cells during progenitor cell division. Required for survival, renewal and mitogen-s

tor mediates its action by association with G proteins that activate a phosphatidylinositol-calciu
conjugation and subsequent elimination of potentially toxic xenobiotics and endogenous comp
e protein that helps to keep newly synthesized precursors in an unfolded import compatible sta
ER, and possibly transfer them to the ubiquitination machinery and promote their degradation. F
yclins exhibit distinct expression and degradation patterns which contribute to the temporal coo

ding nucleosides monophosphates (UMP for UDP-sugars and GMP for GDP-sugars). May take

RefSeq, Jun 2014]

6, PubMed:28561066). Activated by the STK11/AMPK signaling pathway upon starvation, WDI
ling the intraflagellar transport (IFT) complex B from the large pool pre-docked at the base of th

m of the core structure of N-linked glycans. Essential for the production of tri- and tetra-antenn
in clathrin-mediated endocytosis and distribution of endocytic organelles. May play a role in reg

secretion, due to the retardation of intracellular APP maturation. In complex with APBA2 and C

nducible RAD18 loading, PCNA monoubiquitination, POLH recruitment to replication factories
of this complex (By similarity).
cifically converts active Rab3-GTP to the inactive form Rab3-GDP. Required for normal eye an
4 into its biologically less active metabolite, 12-oxo-leukotriene B4. This is an initial and key ste
to both spindle and cilia microtubules (PubMed:22493317). Increases the effects of NCOA2 in
by RNA polymerase II. The protein encoded by this gene is similar to cyclin-dependent kinase
Med:24554434, PubMed:25503564).
nslation repression activity of FMR1 in brain probably by mediating its association with EIF4E a
zes and binds acetylated histones via its bromodomain-like (BRDL) region and activates the pr

s PI4KB kinase activity (By similarity). Involved in long-term synaptic plasticity through its intera

mma-induced transcript-selective translation inhibition in inflammation processes. Upon interfe
AC1 at the leading edge of migrating cells (PubMed:25074804, PubMed:25925950). Required
e potency of GDNF on neuronal survival. Controls CBLC function, converting it from an inhibito
tions, but stimulates apoptosis by repressing CDKN1A under stress conditions. Acts as a nega
of LIMK1 by CDC42BPA and CDC42BPB in the lamellipodia. LRAP25-mediated CDC42BPA/

e TLR9, a nucleotide-sensing receptor in collaboration with PLD4 (By similarity). May be impor

rizing with other (usually larger) basic-zipper proteins, such as NFE2L1/NRF1, and recruiting th

roteins. Unlike many F-box proteins, FBXL2 does not seem to target phosphodegron within its
Required in spermatozoa for the formation of the inner dynein arms and biogenesis of the axon

s.
unctions at the blood-testis barrier.

he sonic hedgehog (Shh) signaling pathway.
acts by binding to H3K36me3, a mark for transcriptional activation, and recruiting H3K36me3 hi

tination and consequent degradation of GORASP1, acting as a component of the ubiquitin liga

-C (FLNC), essential for maintenance of normal contractile function.
h substrate and inhibitor of APC-FZR1 complex (PubMed:29875408, PubMed:17485488, PubM

gulates the BMP4 signaling in a dose-dependent manner (By similarity). Antagonist of BMP2; in

ells.
erequisite for neurite outgrowth and plasticity at sites distant from the cell body (By similarity).
distinct DNA-binding specifities as compared to the canonical form and preferentially binds DNA

(PubMed:23990561). Required for ciliogenesis (PubMed:27666822, PubMed:23990561). Req
on (By similarity).

nation of lamellipodia. Plays a role in cell adhesion, cell spreading, establishment or maintenan
dent of the palmitoyltransferase activity (PubMed:25299331).||Has a palmitoyltransferase activ
RNA (PubMed:16129689, PubMed:27602518). Associated component of the WMM complex, a

any cells and extracellular matrices. Can also sulfate Gal residues in desulfated dermatan sulfa
2075693). May play a role in cell-cell interactions (PubMed:17308099).

component MAVS. In turn, interferes with MAVS oligomerization, and disrupts the MAVS/TRAF

A replication (PubMed:16387653). May inhibit tumor progression by modulating the transcriptio

slation, but ubiquitin ligase activity is required for mRNA degradation.

plex, which is required for several steps in the initiation of protein synthesis (PubMed:17581632
es energy-producing pathways and inhibits energy-consuming processes: inhibits protein, carb

ays filament nucleation and reduces formation of branched filaments (PubMed:12566430). Pla
duced ATM phosphorylation, p53 activation and cell cycle arrest. Involved in myelopoiesis. Tra

2). Required for normal cell proliferation (By similarity).
TIM (STIM1 or STIM2)-dependent SOCE activity: possibly act by facilitating the deoligomerizati
and HIPK2. Activation of this pathway causes binding to and phosphorylation of the histone m

ulates meiotic recombination and crossover homeostasis by physically dissociating strand invas
ngth required for presentation on MHC class I molecules. Strongly prefers substrates 9-16 resi
s as a pro-survival homeostatic factor that attenuates the development of cellular stress. Maint
activates gene expression by binding a 5'-[CAT]A[CT][CT][CTG]GA[GAT]-3' motif present in the
in its active GTP-bound form together with the export receptor XPO1 on the other. Its phospho
ng. Regulates fast synaptic transmission mediated by glutamate (By similarity). Phosphorylates
1)-ceramide(d18:1(4E)) to produce globotriaosylceramide/globoside Gb3Cer (d18:1(4E)) (PubM
n. Inhibits autophosphorylation of EGFR, ERBB2 and ERBB4. Important for normal keratinocyt

Med:14715245).

ytosis of PODXL to the apical membrane initiation sites (AMIS), apical surface formation and lu
ance of endothelial barrier organization and function (PubMed:30455415).
ay have tumor suppressor function.

1115).

endently of retinol conversion, may regulate liver metabolism upstream of MLXIPL/ChREBP. M

ation of JNK and p38 (MAPK14).

nal snRNPs contain a common set of Sm proteins SNRPB, SNRPD1, SNRPD2, SNRPD3, SN
sponsible likely encode proteins involved in the degradation of post-translationally modified pro

ays a role in apoptosis. Involved in the formation of microtubule bundles (By similarity).

the eye lens capsule (By similarity). Has high activity with the type IV collagen COL4A1, and lo
ribonuclease activity, while it shows both exoribonuclease and exodeoxyribonuclease activities
es not activate ubiquitin-like peptides. Differs from UBE1 in its specificity for substrate E2 charg

s premature translation termination at the frameshifting site (PubMed:30682371). Regulates H
, protein sorting and membrane trafficking and is required for prolonged survival of neurons. Be
strates.

/ involved in protein-protein interactions. Subcellularly, the protein localizes to cytoplasmic bodi
jo-selective complex (CSC). The CSC is believed to be the core functional component of retror
e of asymmetric distribution of phospholipids. Phospholipid translocation seems also to be impl

nhibits prostate tumor growth in vivo.

GlcNAc. Also able to catalyze the transfer of sulfate to position 6 of the N-acetylgalactosamine
and SPIRE2 (PubMed:22330775, PubMed:21730168). Involved in intracellular vesicle transpor

73190). Acts on LPA containing saturated or unsaturated fatty acids C16:0-C20:4 at the sn-1 p

dian rhythms in gene expression, which are translated into rhythms in metabolism and behavio
a heterotrimeric complex related to retromer cargo-selective complex (CSC) and essential for
MVBs contain intraluminal vesicles (ILVs) that are generated by invagination and scission from

Lys-63'-linked polyubiquitination of IRAK1 allowing subsequent NF-kappa-B activation (PubMe

iated protein A/SP-A, pulmonary surfactant-associated protein D/SP-D and the lipid transporte
egrity and the organization of stable microtubule binding sites in the outer plate of the kinetocho
ole in cellular lipid transport. Plays a role in FGF signaling via its role in the rapid internalization
oMed:24163370).
the development of atherosclerosis. Also involved in organ detoxification by hydrolyzing exoger
rat acts as a GTPases for the small GTPase Rap. [provided by RefSeq, Sep 2015]

t can also bind to the non-methylated consensus sequence 5'-CTGCNA-3' also known as the c
(CpG) dinucleotides from pathogens, and mediated by the nucleotide-sensing receptor TLR9. I
osphorylates the microtubule-associated proteins MAP2 and MAP4 (PubMed:14594945). Invo
emotactic response. Induces calcium mobilization. Binds to CXCR6/Bonzo.

ure sensitivity to Slit proteins thus inhibiting Slit signaling through ROBO1 (By similarity). Requi
ırd the cytosolic face of the membrane. In response to ER stress, transported to the Golgi, whe

:8-dihydroceramide), to a lesser extent on C2-ceramide and C6-dihydroceramide, but not on ot
.MND5A are both required for catalytic activity of the CTLH E3 ubiquitin-protein ligase complex
: interaction with WDR48 to have a high activity (PubMed:19075014, PubMed:26388029). Not

Probable) (PubMed:29911972). Binds proteins and peptides with a Pro/N-degron consisting of

15560782, PubMed:18411297, PubMed:19786558, PubMed:25083013). Acts as the negative l
3y similarity).
he elongating chondroitin polymer. Isoform 2 may facilitate PRKN transport into the mitochond

The complex promotes relaxation of negative and positive supercoiled DNA and DNA decatena
fically recognizes the E2 recognition site 5'-TTTC[CG]CGC-3'. Directly represses transcription
on of TGFB regulatory targets, such as POSTN (By similarity).

to fatty acyl-CoA substrates including palmitoyl-CoA and stearoyl-CoA (PubMed:15610069, Pu

385, PubMed:24898248, PubMed:28219928). Plays a central role in mitochondrial calcium ho
ıtures associated with MYC, including promotion of apoptosis, alteration of morphology, enhan

thway (PubMed:15378603). Functions as a scaffolding protein to promote G beta:gamma-medi
d only scarcely detected, suggesting that it only exists in some tissues.
ıma subunits of heterotrimeric G protein. Mediates the activation of RAC1 in a PI3K-dependent
form-specific manner (PubMed:11847232, PubMed:18957440, PubMed:23576435). Modulates

Potently inhibits endothelial cell proliferation and angiogenesis (PubMed:9459295). May inhibit a
f Th2 specific factor.

ves the GPI-anchor of RECK, leading to release RECK from the plasma membrane (By similarit
nediated immunity (PubMed:30487606). Required for anti-tumor activity of T-cells by promoting
tes ADAM10-mediated cleavage of CDH5 (By similarity).
nyalin granules and the development of the barrier function of the epidermis (By similarity). Dov
ovascular permeability, endothelial fenestrae contributing to the passage of water and solutes a

distinct role in decorating the cadherin domains with O-linked mannose glycans at specific reg
. Has strong activity toward lactosylceramide (LacCer) and neolactotetraosylceramide (nLc(4)C
quired for the resolution of meiotic double-strand breaks.

ubunit, possibly by helping it fold into its correct conformation for activity.
n (PubMed:19951071). May affect bone resorption and help to maintain bone mass (PubMed:1

F-kappa-B via BCL10 and IKK. Stimulates the phosphorylation of BCL10. Also activates the TC

NA resection by the MRE11-RAD50-NBN/NBS1 (MRN) complex by recruiting the MRN complex
ein kinase C proteins (By similarity).

e (GlcCer) from the trans-Golgi network (TGN) to the plasma membrane and plays a pivotal role
2) to generate 2 second messenger molecules diacylglycerol (DAG) and inositol 1,4,5-trisphos

LRP6 and frizzled receptors that are activated by extracellular Wnt receptors, triggering the ca
distinct role in decorating the cadherin domains with O-linked mannose glycans at specific reg
ates Schwann cells differentiation by mediating ubiquitination of GLUL. Promotes neurodegene
ABL2B complex binds intraflagellar transport (IFT) complex B from the large pool pre-docked at

ydroepiandrosterone (DHEA), pregnanolone, pregnenolone and allopregnanolone (PubMed:23

eorganization and for efficient endothelial cell elongation. In quiescent endothelial cells, trigger

:6, PubMed:26068852, PubMed:28877472, PubMed:28222186, PubMed:30111836). Promotes

'3 releases Ca(2+) from intracellular stores. Essential for trophoblast and placental developme
oting desensitization (By similarity).

h a preference for membranes enriched in phosphatidylserine and phosphoinositides (Pi(4,5) b
e splicing results in multiple transcript variants encoding different isoforms. [provided by RefSe

cts like a sheddase by mediating the proteolytic release and secretion of active site-containing
a cluster on chromosome 16. Alternatively spliced transcript variants containing different 5' U

bolic fuel, by the action of four enzymes that constitute the Leloir pathway: GALM, GALK1 (gal

nzyme may be associated with the disease D-glyceric aciduria. Alternatively spliced transcript
3/TP53-dependent transcription and apoptosis, suggesting an important role in p53/TP53 stres

endocytosis of Delta receptors.

1095, PubMed:12539043, PubMed:14739303). Ligand binding to these receptors results in TR

ating the assembly of an ENaC-regulatory complex (ERC).

ated disk thereby regulating gap junction communication (By similarity). May also play a role in

fiber differentiation, probably by inhibiting FGF-mediated phosphorylation of ERK1/2 (By similar

) protein huntingtin (HTT) in case of endoplasmic reticulum stress by inhibiting the endocytosis
After the penetration of retroviral nucleocapsids into target cells of infection and the initiation of
osphorylation of ERK1/2 (By similarity). Inhibits TGFB-induced epithelial-to-mesenchymal tran
the RNA polymerase III transcriptional repressor Maf1 to regulate gamma-aminobutyric acid A

iple transcript variants. [provided by RefSeq, Dec 2014]

of a lumenal glucose-6-phosphatase. May not play a role in homeostatic regulation of blood gl

reticulum membrane from metaphase chromosomes.
dependent transcriptional activation, by participating in recruitment to androgen-receptor target

moting their degradation. Induces apoptosis via a p53/TP53-dependent but caspase-independ
equired for efficient progress through mitosis and cytokinesis. Required for normal formation of
ose 1-phosphate. 5 or 6-phosphosugars are bad substrates, with the exception of glucose 6-p
st that this gene is essential for normal inner ear function. Mutations in this gene are associate

activates GPR7 and GPR8 more efficiently than NPW30.

involve CEP152, CDK5RAP2 and CEP63 through a stepwise assembled complex at the centr

PubMed:16368756, PubMed:21224894). 3-oxoalanine modification, which is also named formyl

3]
d form that is able to recruit to membranes different set of downstream effectors directly respon
activator of NPPA. Also acts as a cofactor with GATA4, a key cardiac regulator (By similarity).

acid starvation (PubMed:24310610).||Metabolic-stress sensing protein kinase that phosphoryla

ed to integrate endothelial Notch and Wnt signaling to control stalk cell proliferation and to stab

s saturated and unsaturated long chain fatty acyl-CoAs (C12-C20).

el to fulfill cellular energy requirements and preventing excess ROS production. Whereas LC3s

spinal neural progenitor cells differentiation.

ated arginine or lysine residues, to generate 'tailless nucleosomes', which may trigger transcrip
NA polymerase II as part of capped and polyadenylated primary transcripts (pri-miRNAs) that c
Seq, Dec 2017]
s in multiple transcript variants encoding isoforms that share sequence identity with the upstrea
transduction through interaction with the NMDA receptor subunit GRIN1 (By similarity).

us regulating a many physiological and pathological processes including neuronal differentiatio

B heterooligomers regulate phenylephrine (PE)-stimulated ERK signaling in cardiac myocytes.
ation.||Cell adhesion molecule that mediates both heterotypic cell-cell contacts via its interactio
26373694, PubMed:25450233).
avage of fructose-1,6-biphosphate and fructose 1-phosphate to dihydroxyacetone phosphate a
leaves tRNA within anticodon loops to produce tRNA-derived stress-induced fragments (tiRNA
ation of APP-beta. Component of the LIN-10-LIN-2-LIN-7 complex, which associates with the m

. Produces a large instantaneous current. Modulated by intracellular chloride ions and pH; acid

the formation of other types of polyubiquitin chains. The E3 ubiquitin-protein ligase activity is re
to ssDNA over double-stranded DNA, enabling RAD51 to displace replication protein-A (RPA)

), BCR (B-cell receptor) and FCER1 (high affinity immunoglobulin epsilon receptor) signal trans
)K1 or CDK2. The cyclin subunit confers the substrate specificity of these complexes and diffe
lt such as taurolithocholate sulfate (PubMed:16332456). May function as a cellular cisplatin tra
/, and does not display selectivity based on the chirality of the substrate. It is also an essential
Has a low bile-binding ability. May play a role in myelin formation.
the TOM complex to the sorting and assembly machinery (SAM complex) of the outer membra

)S1 and ABI1, forms a trimeric complex that participates in transduction of signals from Ras to
ıppressor gene.
a major lipid in myelin, kidney and epithelial cells of small intestine and colon.
TAT5 pathway (By similarity).||The soluble form (GHBP) acts as a reservoir of growth hormone

amylcysteine synthetase deficiency has been implicated in some forms of hemolytic anemia. A

ones thereby play a central role in transcription regulation, DNA repair, DNA replication and ch
ıes architectures and regulates cell adhesion, migration and differentiation. This is one of the is
ıding to it and promoting its dimerization.
ling and V(D)J recombination and probably other processes. Binds DNA with a preference to n
similarity).

ctivation of MAPK1 and MAPK3.||The insulin-like growth factors possess growth-promoting acti
ling specificity to the enhancer element B of the flounder antifreeze protein gene intron. Binds t
ı, gonadal hormone secretion, germ cell development and maturation, erythroid differentiation,
2953). Regulates the transcription of type I IFN genes (IFN-alpha and IFN-beta) and IFN-stimu
+)/voltage sensitivity of the KCNMA1 channel. It also modifies KCNMA1 channel kinetics and a

ıilarity).

M2-7 ring are formed through the interaction surfaces of two neighboring subunits such that a
M2-7 ring are formed through the interaction surfaces of two neighboring subunits such that a
M2-7 ring are formed through the interaction surfaces of two neighboring subunits such that a
factor for HOXA7 and HOXA9 in the induction of myeloid leukemias.

and also collagen type III, but at lower rate. It has no effect on type I, II, IV and V collagen. H
nto precursor Z to generate a dithiolene group. In the complex, serves as sulfur donor by bein
g (PubMed:23251525).

hain. The immediate electron acceptor for the enzyme is believed to be ubiquinone.
hain. The immediate electron acceptor for the enzyme is believed to be ubiquinone.

arboxylation of glutamate residues in prothrombin synthesis.
n through a cGMP-mediated signal transduction pathway. NO mediates vascular endothelial g
tacts between Schwann cells and axons. Plays a role in the formation and maintenance of the

ion. Is an integral component of the pericentriolar material (PCM). May play an important role i

eotide (FGAM) and glutamate (By similarity).
articipates mainly to the flop of phosphatidylcholine, phosphatidylethanolamine, beta-D-glucos

glycogeneolysis in the testis. In vitro, phosphorylates PYGM (By similarity).
esis (ECO:0000269|PubMed:27694521).

BA A receptor (By similarity). Regulates the turnover of receptors and thus contributes to the
hemic brain.
olved in proper maturation of stable RNA species such as rRNA, snRNA and snoRNA, in the
the splicing-dependent multiprotein exon junction complex (EJC) deposited at splice junction

l non-coding RNAs. Pol II is the central component of the basal RNA polymerase II transcripti
ssociated with two 19S regulatory particles, forms the 26S proteasome and thus participates in
n factor C (RFC) clamp loader complex (PubMed:12578958). Acts then as a sliding clamp plat

of the multiprimed DNA template.

ruits and activates different proteins and complexes involved in DNA metabolism. Thereby, it p
s a ribosomal protein that is a component of the 40S subunit and a member of the S14P family

MN2. Activates the splicing of MAPT/Tau exon 10. Alters pre-mRNA splicing patterns by antago
and apoptosis. Plays an important role in cellular stress response. Contributes to regulation of
lated with reduced phosphorylation of CDK1. Plays a role as a positive regulator of the sonic he
ession, signal transduction and transcription. Specifically recognizes phosphorylated CDKN1B/
949). Can also transport L-cysteine (PubMed:21123949). Functions as a symporter that transp
type D simian retroviruses.||Sodium-dependent amino acids transporter that has a broad subs
priming, fusion and dilation of exocytotic fusion pores (PubMed:28288128, PubMed:3040482
ling of stem loop IV of U2 snRNA (PubMed:9716128, PubMed:27035939).
e building blocks of the spliceosome (PubMed:11991638, PubMed:18984161, PubMed:19325
SUN3 and probably SYNE1 through a probable SUN1:SYNE3 LINC complex to the nuclear env
at the cell membrane, and thereby contributes to increased channel activity. Slows down the ina

/AMP2 which mediates the fusion of synaptic vesicles with the presynaptic plasma membrane.
netic transcriptional repression by recruiting HP1 (CBX1, CBX3 and/or CBX5) proteins to meth

T-lymphocyte-specific enhancer element (5'-WWCAAAG-3') found in the promoter of the CD3

ic properties. Plays a role in ER stress response, via its interaction with the activating transcrip
the inflammatory response (PubMed:9237759, PubMed:10835634, PubMed:27022195). Also i
mina and thereby help maintain the structural organization of the nuclear envelope. Possible re
ation at a target site in duplex DNA. The scissile phosphodiester is attacked by the catalytic tyr
of estrogen receptors alpha and beta while isoform 5 enhances the transcriptional activity of the
rescue of pyrimidine bases for nucleotide synthesis.
CNA (PubMed:16531995). Has almost no deubiquitinating activity by itself and requires the inte
ng mRNA transcripts by promoting their poly(A) tail removal or deadenylation, and hence provid

bule-based structures that protrude from the surface of epithelial cells.
afts. It interacts with caveolin-1. [provided by RefSeq, Jul 2008]
carrier protein reductase activity, and thereby plays a role in mitochondrial fatty acid biosynthe

28, PubMed:14966270). Specifically recognizes and binds acylated histone H3, with a preferer
PubMed:9751058, PubMed:10049700, PubMed:11557028, PubMed:10391915, PubMed:1057
newly synthesized histones H3 and H4 to replicating DNA; histones H2A/H2B can bind to this c
gulation, DNA repair, DNA replication and chromosomal stability. DNA accessibility is regulated

from S.typhimurium and S.minnesota.
, diacylglycerol pyrophosphate/DGPP, sphingosine 1-phosphate/S1P and ceramide 1-phospha

d IFNG synthesis from T-helper 1 (Th1) cells (PubMed:10653850). Contributes to IL18-induced

ed:9856465, PubMed:17141802, PubMed:17296725, PubMed:23418588, PubMed:26344098).

diated phosphorylation and inhibition of SLC9A3 (PubMed:18829453). May also act as scaffold
activation speed of ASIC3. May be involved in regulation of proton sensing in dorsal root gangli
omeostasis, thus triggering the activity of Fe(2+) prolyl hydroxylase (PHD) enzymes, and leadin

nvolved in formation of short side-chains. Mediates initiation of polyglutamylation of nucleosome
d:11590237, PubMed:15824131). Its function related to the spindle assembly machinery is prop
bMed:17719545 PubMed:17681131, PubMed:23486064). Also involved in promoting ciliogene

P53. Acts as a regulator of cellular senescence and cellular ploidy by mediating phosphorylatio
he cellular resistance to DNA damage stresses, like ionizing radiation (IR), ultraviolet (UV) and

n cell cycle progression, signal transduction, transcription and transcription-coupled nucleotide
in transcriptional regulation, cell cycle progression and developmental events (PubMed:102203
nucleosome assembly process, bringing newly synthesized histones H3 and H4 to replicating

n active ligand-binding subunit in mediating retinoid responses through target genes defined by
ansports the copper ions to the Cu(A) site on the cytochrome c oxidase subunit II (MT-CO2/CC

between AURKA and PLK1, thereby enhancing AURKA-mediated PLK1 phosphorylation.

conversion of lysophosphatidylserine (1-acyl-2-hydroxy-sn-glycero-3-phospho-L-serine or LPS
uction of type I interferon and inhibits infection with vesicular stomatitis virus (By similarity). Pror

biquitination and subsequent degradation of target proteins: it mainly mediates the formation of
propyl) pseudouridine (m1acp3-Psi) conserved in eukaryotic 18S rRNA. Is not able to methylat
component of the TACC3/ch-TOG/clathrin complex proposed to contribute to stabilization of k
4 snoRNAs.

timing of sister chromatid segregation, and maintenance of spindle pole architecture (PubMed
lex (Probable). With TAF5L, acts as an epigenetic regulator essential for somatic reprogrammi
LOX (By similarity).
rase delta complexes (Pol-delta3 and Pol-delta4, respectively), plays a role in high fidelity geno
as SASS6, CENPJ/CPAP, CCP110, CEP135 and gamma-tubulin. When overexpressed, it is a
gene designates it to be a candidate for diseases such as Nance-Horan syndrome, sensorineur
athway.

nmune regulation, stem cell pluripotent and self-renewal maintenance and embryonic developr

the context (By similarity). The side chain of Glu-231 determines which of the two opposing act
heptameric LSM2-8 complex binds specifically to the 3'-terminal U-tract of U6 snRNA (PubMe
ompatible with extension and ligation by either removing 3'-phosphates from, or by phosphoryla

volved in proper maturation of stable RNA species such as rRNA, snRNA and snoRNA, in the e

an important role in vertebrate development, where they locally inhibit Wnt regulated processe

ion. In vitro shown to be involved in the homologous recombination mechanism for the repair o

teoglycans) sulfation, inhibits signaling by heparin-dependent growth factors, diminishes prolife
) complex involved in the connection between the nuclear lamina and the cytoskeleton. The nu

in the presence of type I topoisomerases and converts nicked DNA into positive knotted forms
volved in proper maturation of stable RNA species such as rRNA, snRNA and snoRNA, in the e
dependent fashion. In contrast, it does not coactivate nuclear receptors for retinoic acid, vitami

ant of this gene may be associated with survival in human glioblastoma patients. [provided by R
heptameric LSM2-8 complex binds specifically to the 3'-terminal U-tract of U6 snRNA (PubMe

s as part of a heterodimeric complex with IL17RC (PubMed:16785495). Also binds heterodimer
small molecules. Often, several claudin family members are coexpressed and interact with eac

omatin assembly and with HIRA to promote replication-independent chromatin assembly. Requ

ressed. Required for cell cycle progression from G1 to S phases.
e of the mitotic checkpoint complex (MCC) from the APC/C: not required for APC/C activity itse
oxidation pathway.

tivation (such as CASP9) and inducing mitochondrial permeability transition. May be involved i
rsors from the TOM complex to the sorting and assembly machinery (SAM complex) of the out

cing MDM2 stability. This promotes MDM2-mediated ubiquitination of p53/TP53 and its subseq

olubility of most compounds, and therefore their renal excretion, but it can also result in bioacti
ating cell survival, morphology and differentiation (PubMed:23922215, PubMed:25620095). Un

n estimated 75% protein to rRNA composition compared to prokaryotic ribosomes, where this r
ein may be involved in the atRA (all-trans retinoic acid) signaling through the STAT1-alpha (sig

n estimated 75% protein to rRNA composition compared to prokaryotic ribosomes, where this r
BP at some ESR1 target gene promoters. May be required for histone hyperacetylation. Involv
be a regulator of breast tumor cell invasion.

C (mitochondrial translation regulation assembly intermediate of cytochrome c oxidase comple

thylation of Glu residue of proteins with a Gly-Gln-Xaa-Xaa-Xaa-Arg motif (PubMed:18539146)
omote the resolution of double-strand DNA catenanes (intertwines) between sister chromatids.
ecessary to ensure substrate recognition, and therefore, effectual transfer of the oligomannose
e activity of DMC1 as well as DMC1 D-loop formation from double-strand DNA. This complex st

intra-S-phase and G/2M cell-cycle checkpoints and the maintenance of genome stability.

ought to act as a cellular signaling inhibitory receptor by recruiting cytoplasmic phosphatases lik

nal mitochondrial function (By similarity). Maintains 16S rRNA levels and functions in mitochon
5828). The complex is probably involved in the transfer of the threonylcarbamoyl moiety of thre
ns to the basal RNA polymerase II transcription machinery. Mediator is recruited to promoters b
3]
n estimated 75% protein to rRNA composition compared to prokaryotic ribosomes, where this r
ar deoxyinosine in conditions of energy deprivation.
nization of the microtubules during centriole duplication. A pseudogene of this gene is found on
stimulates its ATPase activity.
hibited by interleukin 10.

e c oxidase subunit II (MT-CO2/COX2) (PubMed:29355485, PubMed:29381136). Interacts spe

-rRNA sequences (PubMed:28115465).
ticulum.
provided by RefSeq, Dec 2015]
absence of CDC42 (By similarity). May play an important role in the maintenance of sarcomere
e and endoplasmic reticulum-associated degradation (ERAD) system (By similarity).

primary miRNA processing complex containing DGCR8 and DROSHA, thereby playing a role
eptide bonds in oligopeptides (PubMed:16595688).

ɘ ciliary tip in response to activation of the SHH pathway, suggesting it is involved in the dynam
ҠNAs. Plays a key role in sensing and limiting infection by intracellular bacteria and DNA viruse

ɔchrome oxidase complex, the final protein carrier in the mitochondrial electron-transport chain.

no acids at the blood-brain barrier and in the supply of maternal nutrients to the fetus through th

ɔcesses. Recruits ITCH, NEDD4 and SMURF2 to endosomal membranes. Negatively regulates
ransition zone of primary cilia and acting as a barrier that prevents diffusion of transmembrane

58). Acts as a tension sensor that associates with catenated DNA which is stretched under tens

axon guidance and cell migration (By similarity). Probable signaling receptor which may play a

in the replication checkpoint induced by hydroxyurea or ultraviolet light. Forms a complex with

ecay (NMD) pathway (PubMed:23917022).
ɔmes. Microtubule severing may promote rapid reorganization of cellular microtubule arrays and
bMed:28544275).
ɔoration of newly synthesized CENPA into centromeres via its interaction with the CENPA-NAC
and clustered with the gene encoding ankyrin repeat and SOCS box-containing protein 7. Alter
uitment to DNA repair sites. Required for maintenance of chromosomal stability. Specifically bi

ɘ been noted for this gene, however, not all are likely to encode viable protein products. [provid

ation.
and tRNA(Gln) at the wobble position. ATP is required to activate the C2 atom of the wobble b
ɔolar spindle, which is needed for proper tension generation between sister chromatids. Requir
ocalization of proximal CC proteins at the distal CC (By similarity). Maintenance of protein loca
ɔw processive growth of centriolar microtubules. Acts as microtubule plus-end tracking protein
2/M checkpoint during doxorubicin-induced DNA damage.

t act on phosphatidate (By similarity).

ɔeta gamma subunits of heterotrimeric G protein. May function downstream of heterotrimeric G
ɛ-operated and store-independent Ca(2+)-influx. Regulates basal cytosolic and endoplasmic re
by promoting stabilization of ERCC6 by recruiting deubiquitinating enzyme USP7 to TC-NER c

heptameric LSM2-8 complex binds specifically to the 3'-terminal U-tract of U6 snRNA (PubMe
GTPase activity. May mediate the membrane association of SRPR (By similarity).

uires its ABM domain and may implicate the stabilization of the interaction between HIF1AN an

K1 and facilitates the ATR-dependent phosphorylation of both proteins. Can also bind specifica
eration and differentiation (PubMed:23754376). May play a role in the control of male meiosis c
ɔoration of newly synthesized CENPA into centromeres via its interaction with the CENPA-NAC
in the presence of type I topoisomerases and converts nicked DNA into positive knotted forms

SIN3A and CTBP2. May be involved in the development of central and peripheral nervous sys
e proteins (GABARAP, GABARAPL1, GABARAPL2 or MAP1LC3A). The ATG12-ATG5 conjug

cies. RNase treatment of permeabilized cells indicates that the nucleolar localization is RNA de

NA with a strong preference for DNA substrates that mimic structures generated at stalled rep
e CENPA-CAD (nucleosome distal) complex and may be involved in incorporation of newly syn

- and very long-chain fatty acids (VLCFAs) per cycle. Condensing enzyme that elongates fatty

ir 3-hydroxyl metabolites. Exhibits reductive 3-beta-hydroxysteroid dehydrogenase activity towa
poration of newly synthesized CENPA into centromeres via its interaction with the CENPA-NAC

RNA. TREX is recruited to spliced mRNAs by a transcription-independent mechanism, binds to

the MLL2/MLL3 complex. Proposed to be recruited by PAXIP1 to sites of DNA damage where

bstrates such as Muc2, Muc5AC, Muc7, and Muc13 (-58). May be involved in O-glycosylation ir

e CENPA-CAD (nucleosome distal) complex and may be involved in incorporation of newly syn

HP1 (CBX1, CBX3 and/or CBX5) proteins to methylated histones. Mainly functions in heterochr

nal snRNPs contain a common set of Sm proteins SNRPB, SNRPD1, SNRPD2, SNRPD3, SNI
nal snRNPs contain a common set of Sm proteins SNRPB, SNRPD1, SNRPD2, SNRPD3, SNI

autosomal recessive neurometabolic disorder resulting in moderate to severe cognitive disabilit
T7/HBO1 specificity towards histone H4 acetylation (H4K5ac, H4K8ac and H4K12ac), regulatir
junctions (DHJ) and hence stimulates TOP3A-mediated dissolution. Required for BLM phospl
nd remodeling of neuronal connections.

ot involved in deposition of H2A.Z/H2AZ1 in the nucleosome. May stabilize the evicted H2A.Z/I
hen probably delivered to ferrochelatase to catalyze Fe(2+) incorporation into protoprophyrin IX
PubMed:15118078).
by homologous recombination in a manner that depends on its association with BRCA1.
ineralization (PubMed:30082715, PubMed:29878199). Also involved in the regulation of cellula

nponent and in collaboration with CIAO1 specifically matures ACO1 and stabilizes IREB2, coni
te phosphorylated O-mannosyl trisaccharide (N-acetylgalactosamine-beta-1,3-N-acetylglucosa
rovided by RefSeq, Sep 2011]

MC5-SMC6 complex at replication-coupled interstrand cross-links (ICL) and DNA double-stranc

tophagosome-lysosome fusion. Constitutes the major lysosomal potassium channel.
onsumption and ATP production and via its modulation of the respiratory chain activity can regu

ting from replication and recombination intermediates and from DNA damage. Component of t
NOLC1 and TCOF1: monoubiquitination promotes the formation of a NOLC1-TCOF1 complex t

-sn-glycerol-3-phosphate (phosphatidic acid or PA) by incorporating an acyl moiety at the sn-2

'-Dol.

d CEP120-mediated elongation dependent manner during the cell cycle S/G2 phase after form

JRKB and controls the dynamic behavior of AURKB on mitotic chromosomes and thereby coor
Med:28437394, PubMed:28302793, PubMed:31693891, PubMed:31452512). RBM39 degradat
ns to the basal RNA polymerase II transcription machinery. Mediator is recruited to promoters l
location and insertion into the late CIII dimeric intermediate within the mitochondrial inner mem

composed of a neuropilin and a plexin. The plexin modulates the affinity of the complex for spe

]

ensin II receptor/AGTR1 (Probable).

rix. Mutations in this gene are associated with the autosomal recessive neurodegenerative dise

gen-IV that contain it.

ome partitioning is accurate in both meiotic and mitotic cells and plays an important role in DN/
:rophages, and induces the release of pro-inflammatory cytokines such as TNF, IL1B, IL6, CCL
092684).
ɔort of Mg-ATP in exchange for phosphate, catalyzing the net uptake or efflux of adenine nucle

eby promotes membrane rigidity (PubMed:30509349). Does not appear to have any effect on L

le-stranded DNA. Has low efficiency with single-stranded substrates. Requires molecular oxyg
in ligase complex which mediates the ubiquitination and subsequent proteasomal degradation

ions (Chikaev et al., 2005 [PubMed 15676285]).[supplied by OMIM, Mar 2008]

siently associated nuclear transport factors. May play a role in the association of MAD1 with th

location of proteins into the endoplasmic reticulum. It also plays a role in the ubiquitin-mediate

:tyly, also known as Laurin-Sandrow Syndrome. Alternatively spliced transcript variants have be
d Holliday junctions. May be required in mitosis for the processing of stalled or collapsed replic

zation of stable microtubule binding sites in the outer plate of the kinetochore (PubMed:147387

ner ear, and for normal survival of hair cells in the organ of Corti (PubMed:17186462).

ver, and roles in inflammation and immune response. This family member plays a role in cell-c
, that appear to have been derived from the piggyBac transposons. This gene belongs to the s

centriole amplification in multiciliated cells. Overexpression results in excess foci-bearing cent

phase (PubMed:19502560).

egatively regulate bHLH-mediated transcription through an N-terminal repressor domain. Serve

nvolved in nuclear movement during fibroblast polarization and migration. Proposed to be invol
; the function seems to depend on KCTD11:KCTD6 oligomerization. Can function as antagonis
ded by RefSeq, Feb 2013]

anaphase onset during mitosis, when chromosomes undergo bipolar attachment on spindle m
is a direct component of the kinetochore-microtubule interface and directly associates with mi

of RELA at 'Ser-536' (PubMed:24681962). May be involved in early neuronal development (B

14, PubMed:23530041, PubMed:26887952). Can use other acyl donors, but with less efficienc

glycolipids to glycoproteins (By similarity).
proteins suggests that it may link receptor tyrosine phosphatase with its substrates at the plas
uired for building functional cilia. Involved in the organization of the subapical actin network in m
-fucose formed either by the de novo or salvage pathways is transported into the endoplasmic
metabolite Chivosazole F (PubMed:28796488).
ducts are an intron and two tRNA half-molecules bearing 2',3' cyclic phosphate and 5'-OH termi

zation of microtubules of centriolar origin, possibly to suppress aberrant cilia formation (PubMe

uridine of tRNAs at wobble position.

liates, also known as Holliday junctions, are formed during homologous recombination and DN/
s natural killer cells to secrete IFNG.
during endosome sorting (PubMed:18159949, PubMed:20175130). Involved in endocytic traffi

poration of newly synthesized CENPA into centromeres via its interaction with the CENPA-NAC

densation of N-methylguanine with 2 carbon atoms from pyruvate to form the tricyclic 4-demeth
plasmic reticulum Ca(2+) stores during autophagy. Contributes to BIK-initiated autophagy, while

dogene is a member of the DUXA homeobox gene family. [provided by RefSeq, Jul 2008]

NA polymerase II as part of capped and polyadenylated primary transcripts (pri-miRNAs) that c

NA polymerase II as part of capped and polyadenylated primary transcripts (pri-miRNAs) that c
nuclear pore complex (NPC) is mediated by KPNB1 through binding to nucleoporin FxFG repea

involved in CDH1-mediated adhesion and signaling in epithelial cells.
nted and paternally expressed in many tissues. [provided by RefSeq, Nov 2015]

PubMed:23576762, PubMed:23665031). The BCR(KLHL3) complex acts by mediating ubiquitin
the midbody to the cell membrane during cytokinesis. Plays key roles in controlling cell growth
(PubMed:23870127). May play a significant role in podocyte cell migration (PubMed:2467663
lly, and intercalate between neighboring cells, leading to convergence toward the mediolateral

rs (TLRs) through terminating NF-kappa-B activity. Essential component of a ubiquitin-editing p
uring T-cells development. In the cytoplasm, plays a pivotal role in signal transduction via its as

4699129, PubMed:15062103, PubMed:15235793, PubMed:15239953, PubMed:15548592, Pu
e transition, an essential step for the formation of an organized central spindle midzone and mi
Enhances the incorporation of serine into phosphatidylserine and sphingolipids. May play a rol
URKA localization to spindle microtubules (PubMed:18663142, PubMed:19208764). Activates

5). Also catalyzes the phosphorolysis of S-methyl-5'-thioadenosine into adenine and S-methyl-
e enhancer elements of a number of cellular promoters, including those of the class I MHC, int

ncreases glycogenolysis and relaxes the smooth muscle of trachea, stomach and gall bladder.
gulation, DNA repair, DNA replication and chromosomal stability. DNA accessibility is regulated
ration and apoptosis (PubMed:24784001, PubMed:16648480). During cytokinesis, targets to ce
3'-5' exonuclease and 3'-phosphodiesterase, but not apurinic-apyrimidinic (AP) endonuclease,
nosome 7 has been associated with acute lymphoblastic leukemia. [provided by RefSeq, Sep 2

NA polymerase II as part of capped and polyadenylated primary transcripts (pri-miRNAs) that c

passage protein complex (CPC) which is essential for chromosome alignment and segregation

ptional splicing of a large set of exons.

on with ARL6IP5, a negative regulator of SLC1A1/EAAC1-mediated glutamate transport (By sin

ylates CDK1 and activates its kinase activity.
tly dephosphorylates CDK1 and stimulates its kinase activity. The three isoforms seem to have

'A) between liposomes and probably functions as a PA transporter across the mitochondrion in
ate (PSQPP), with concomitant release of a proton and a molecule of inorganic diphosphate. In
eric complex, made of NLRP1, CASP1, and possibly PYCARD. Recruitment of proCASP1 to th

: with the AP-3 complex, the BLOC-1 complex is required to target membrane protein cargos in

tes TNF-alpha-induced apoptosis and is involved in apoptosis in trophoblast cells. May inhibit E
on to the central spindle and midbody. Required to recruit PLK1 to the spindle. Stimulates PLK
nin and activation of Wnt target genes (PubMed:25759469). A second signaling pathway involv

NA polymerase II as part of capped and polyadenylated primary transcripts (pri-miRNAs) that c

x play an important role in the transmission of mechanical forces across the nuclear envelope a
nethyl, then to carboxylaldehyde, followed by the formation of the delta 14,15 double bond in th
nscriptional repressor. Binds to the sequence 5'-TAATGTTATTA-3' which is present within the

y released in the cytoplasm after a meal (PubMed:10456334). The affinity of GCKR for GCK is

ıis protein has not been determined. Alternative splicing of this gene results in two transcript va
ines, integrin signaling, but also responses to DNA damage and genotoxic agents. Functions p
and thereby contributes to the responses to tissue damage and infectious agents. Activates do
:ol-4,5-diphosphate, phosphatidylinositol-3,4,5-triphosphate, and phosphatidic acid (By similarit

·egulation of neuronal polarization by mediating the transport of specific cargos. Participates in

sociation with chromatin targets a pool of PPP1CC to dephosphorylate substrates.
By similarity).

for the lateral to end-on conversion of the chromosome-microtubule attachment (PubMed:2389
nerization and prevents premature DNA binding by the CENPA-H4 tetramer. Directly binds Hol

2017]

fles (PubMed:24591580). The lipid phosphatase activity is critical for tumor suppressor functior
signaling pathway (PubMed:25263562, PubMed:26449471). This stress-inducible metabolic re
nitosis since microtubule detyronisation regulates mitotic spindle length and postioning (PubMed
ntrolling cytoskeleton dynamics like MYH10 (involved in movement); CTTN (involved in signalir
lated more efficiently than the DNA component. Plays a key role in both tRNA splicing and mR

gression, protein synthesis and by regulation of MYC transcriptional activity. Additionally to this
R-mediated miniature excitatory postsynaptic currents. Exhibits dual GTPase-activating specific
n Ras- and Rho-regulated signaling pathways in cell growth regulation. Isoform 2 has higher G

much higher affinity for arginine than isoform 2. Isoform 2 functions as low-affinity, high capacit
granules (PubMed:26478006). In macrophages and dendritic cells, regulates phagosome matu

2684, PubMed:26586190). Binding of leucine to SESN2 disrupts its interaction with GATOR2 th

can be either protein-coding or non-coding. The primary transcript is cleaved by the Drosha ribo

oding splice variant for this gene is abundantly expressed in human oocytes, while a noncoding

proteolysis of misfolded proteins and the formation and dissociation of protein complexes. Play
can be either protein-coding or non-coding. The primary transcript is cleaved by the Drosha ribo

ɔ21(CIP1), FBH1, KMT5A and SDE2 (PubMed:16861906, PubMed:16949367, PubMed:16964[illegible]

(PubMed:23576022, PubMed:23704824).

proteolysis of misfolded proteins and the formation and dissociation of protein complexes. Play

PubMed:24990967). Regulates activation of unfolded protein response (UPR) in the process of

drome, and an intronic variant of this gene may be associated with sleep duration in children. T

ent. Regulates both osteogenesis and postnatal bone mineralization by osteoblasts. Promotes
ation, morphogenesis of spine density dendrite, and establishment of postsynaptic dendrite de
in D-CDK4 complex and promotes its kinase activity towards RB1. At higher stoichiometric rati
volved in the assembly, stability, and modulation of CCND1-CDK4 complex activation. Acts eith

can be either protein-coding or non-coding. The primary transcript is cleaved by the Drosha rib

ication during cell cycle.
ammatory caspases.

ized ligands, increasing the rate of migration and cell-cell contacts in an integrin-dependent ma

ion (PubMed:8663044, PubMed:28302677). Functions as a potent mitogen in vitro (PubMed:1

. Enhances the microtubule lattice severing activity of KATNA1 by recruiting the katanin comple

by regulating ADAM10 activity (PubMed:26686862). Mediates docking of ADAM10 to zonula a

mon fragile sites stability; acts as a direct target of transcriptional repression by ZBTB38 (Pub

Med:15277473, PubMed:16449236, PubMed:17210863, PubMed:19793056, PubMed:19279

conventional H3 in the nucleosome core of centromeric chromatin at the inner plate of the kin

e) by mediating the lateral sliding of polar chromosomes along spindle microtubules towards the
as a potential inhibitor of pocket protein-mediated cellular processes during development by re

which it recruits to the promoters, causes a decrease in dimethylated H3K4 levels and repress

gression. Alternative splicing results in multiple transcript variants. [provided by RefSeq, Aug 2
bMed:10806483). GLI2 requires an additional function of STK36 to become transcriptionally a

inase STK11 in the nucleus, thereby attenuating cytoplasmic AMPK activation. Plays a role in
) activity. May act as a regulator of adipogenisis (By similarity).

proteins in vivo.

protective effects since excess of free heme sensitizes cells to undergo apoptosis.

can be either protein-coding or non-coding. The primary transcript is cleaved by the Drosha ribo

d neuronal cells differentiation by mediating basic-FGF and NGF-induced signaling cascades.
ls through the inhibition of SNAI1, which itself induces the epithelial-to-mesenchymal transition

HIF1A, JUN, p53/TP53, p73/TP73, PTEN, TOP2A and VRK1. Involved in cell cycle regulation: r
triggering the differentiation of lymph vessels, but is not required for the maintenance of differ
nore integrity and the organization of stable microtubule binding sites in the outer plate of the ki
can be either protein-coding or non-coding. The primary transcript is cleaved by the Drosha ribo

oteasomal degradation.

osteocalcin FGF response element (OCFRE). Binds to the homeodomain-response element of

rganization of the actin filament system. Alternate splicing results in multiple transcript variants
NA (miRNA)-mediated gene silencing. Negatively regulates Hippo signaling pathway and antag
etylation of histone variant H2A.Z.1/H2A.Z, but not H2A.Z.2/H2A.V, thereby promoting express
ular as a complication of diabetes. AGE/RAGE signaling plays an important role in regulating th
is involved in immune response, cancer cell invasion and regulation of apoptosis. Mediates ce
tiation (By similarity). Enhances fibroblast growth factor-induced angiogenesis (in vitro).
s ciliary motility of ciliated epithelial lung cells. This protein is secreted and cleaved to form an a
mide, or phosphatidylinositol (PubMed:20577214, PubMed:23602659, PubMed:29662056, Pub
erleukin-2 receptor, somatostatin receptor II, and interferon-beta genes. It may act in T-cell acti
se 6-phosphate (PubMed:29298880). Plays a key role in maintaining the integrity of the outer m
ts cellular toxicity in activated macrophages by mediating the ubiquitination and proteasomal de

ene) (PubMed:7961644). Mediates biochemical and toxic effects of halogenated aromatic hydr
sible for vesicle formation, movement, tethering and fusion (By similarity). Required for mainta

ons; retinoic acid is required for normal embryonic development of the eye and the nasal regio
between cell survival and apoptosis through a cascade that primarily alters the function of tran

neuronal, and skeletal muscle cells; inducing expression of acetylcholine receptor in synaptic v
nvasion.
izes aromatic aldehyde substrates (PubMed:1737758). Comprises about 50 percent of cornea
onents ARNTL/BMAL1 and CLOCK. Also regulates genes involved in metabolic functions, incl
ts later to inhibit NODAL-signaling by directly targeting NODAL (By similarity). May play a role
tein may function as a cell activation antigen. An alternative splice variant has been described
ts cellular toxicity in activated macrophages by mediating the ubiquitination and proteasomal d
bunits bind DNA with low affinity by themselves. Initially the CST complex has been proposed

n during cell cycle.
as.
ontributes to the generation of normal tension across sister kinetochores. Recruits KIF2A and

. Dephosphorylates LCK at its activating 'Tyr-394' residue (PubMed:21719704). Dephosphory
robable).
ssed both in resting and proliferating cells in response to DNA damage.
uption of the cortical actin filaments.

2, RASGRF1, SNCA, SIPA1L1 and SYNGAP1. Plays a key role in synaptic plasticity and mem
pid degradation of the shortened mRNA tails by the CCR4-NOT complex. Deadenylated mRN
ves (PubMed:11090199).||Endogenous ligand for the apelin receptor (APLNR) (PubMed:10525

d for proper axon specification, the establishment of neuronal polarity and proper neuronal mig

3, PubMed:20347429). In complex with CENPX (MHF heterodimer), crucial cofactor for FANC
mbryonic stem cell derivation and self-renewal, suggesting that it is involved in safeguarding e

ator of telomere length by initiating telomere trimming, a process that prevents the accumulatic

GTPase complexes and prevents their interaction with the mTORC1 complex inhibiting its relo

!92). Functions by both counter-exchange and uniport mechanisms (PubMed:24652292).

vely regulates vascular branching during angiogenesis. Mediates retraction of tip cell filopodia

ates the ability of transcription factor HIF1A to bind to hypoxia-responsive elements (HRE) loca

ıant cells.

lved in the maintenance of telomeric DNA. Inhibits telomere elongation, de novo telomere forn

97329).

ı transcript is a candidate for nonsense-mediated mRNA decay (NMD), and is unlikely to produ

complex. Required for the localization of CENPF, MAD1L1 and MAD2 (MAD2L1 or MAD2L2)

to the fusion of this gene and the FBLN1 gene is found in a complex type of synpolydactyly. M

ys an anti-inflammatory role during the hepatic acute phase response by acting as a corepress

ɔhil activation and IL-8(7-77) has a higher affinity to receptors CXCR1 and CXCR2 as compare

ay explain how p53/TP53 can negatively regulate AKT1. May act as a tumor suppressor.
of p53/TP53. Promotes proteasome-dependent ubiquitin-independent degradation of retinobla

of this family differ in the number of C-terminal TS motifs, and some have unique C-terminal dc
differentiation and survival. Plays diverse roles in the control of cell growth, survival and apopto
roteoglycans seems to inhibit formation of SDF-1-alpha(3-67) and thus to preserve activity on l

-like complex which has a poly(A) RNA polymerase activity and is involved in a post-transcripti
en the SLX1B and SULT1A4 genes located approximately 730 kb upstream on the same chro
on of STAT1 and up-regulates expression of VCAM1 and ICAM1 (PubMed:23892569). Promot
ipal-dependent learning and memory by suppressing the number of excitatory synapses and th
ı (PubMed:22132193). Plays a role in the dephosphorylation of the heat shock factor HSF1 and
ETDB1. The complex formed with MBD1 and SETDB1 represses transcription and probably co

the PI3K-AKT and MEK-ERK pathways. May act as a regulator of tumorigenesis through its ac

d non-canonical NF-kappa-B signaling by acting in opposite directions: acts as a positive regul

can be either protein-coding or non-coding. The primary transcript is cleaved by the Drosha ribo
tor Met of the ubiquitin (linear polyubiquitin chains). Polyubiquitin chains, when attached to a ta

critical structure of a conserved arginine finger motif is provided in trans relative to the ATP-bir

ediated apoptosis may have a role in the induction of peripheral tolerance, in the antigen-stimu

s.

taste perception.

egulation of mitotic exit and cytokinesis. Polo-like kinase proteins acts by binding and phospho
on and spindle assembly. Involved in the bipolar attachment of spindle microtubules to kinetoc
se element (IRE) with consensus sequence 5'-TT[G/A]TTTG-3' and the related Daf-16 family I

differentiation, apoptosis, angiogenesis, and neoplastic transformation. Inhibits skeletal muscle
s endothelial cell differentiation through NOS3 (PubMed:24463812). May be implicated in speci

cytokinesis and the development of the central nervous system. Phosphorylates MYL9/MLC2.
differentiation, apoptosis, angiogenesis, and neoplastic transformation. Inhibits skeletal muscle

splace BAK1 from its binding site on MCL1 (By similarity). Competes with BIM/BCL2L11 for bin

rgets PKN1 to endosomes and is involved in trafficking of the EGF receptor from late endosom
l phosphorylated IRF3 translocates into nucleus to promote antiviral gene transcription. Exihibi

ens epithelial cells (By similarity).

gulating alpha-tubulin deacetylation and cytoskeletal microtubule stability, thereby promoting ce
orting organelle, where receptor and ligand are disassociated. The receptor then returns to the

ted transport of Golgi membranes and associated vesicles along microtubules. Has a microtub

otes phosphorylation of PIK3R1, the regulatory subunit of phosphatidylinositol 3-kinase, and su

verhanging flap structures similar to those generated by displacement synthesis when DNA pol

eoclastogenic cytokine TNFSF11/RANKL. Necessary for normal development of the membran

Required to prevent mitotic entry after treatment with ionizing radiation.

scription presumably by sequestering inhibitory cofactors away from the promoters.

fates in late retinal progenitors as well as for proper generation of other retinal cell types (By s
) and modulates the activity of down-stream effectors, such as adenylate cyclase. Signaling inh

can be either protein-coding or non-coding. The primary transcript is cleaved by the Drosha ribo
s GABARAP, GABARAPL1/L2, MAP1LC3A/B/C and regulates autophagy. Acts as an antioxida

Acts by initiating 'Lys-11'-linked polyubiquitin chains on APC/C substrates, leading to the degr

ıbMed:27041596). Binds selectively the recognition sequence of CEBPA (PubMed:26455797).
ensuring cohesin persistence at centromere until cohesin cleavage by ESPL1/separase at ana

athway. Inhibits phosphorylation and activation of CAMK2D.

omosomes from collapsing into a single chromatin mass by forming a steric and electrostatic c

evelopment. May specifically mediate the Ca(2+)-dependent exocytosis of large dense-core ve

ture (PubMed:19667759, PubMed:22110139). The astrin (SPAG5)-kinastrin (SKAP) complex p

ted endocytosis through binding to epidermal growth factor receptor (EGFR) and clathrin. Binds

venting its binding to NRSE and leading to derepression of target genes (PubMed:11779185). H
Med:9892649, PubMed:12732143, PubMed:15882621, PubMed:16473935, PubMed:1859389

the bifurcation of the forebrain and the establishment of ventral midline structures. May partici

ethora of physiological and pathological processes including cell cycle arrest in epithelial and he
4 (MIM 300326) gene. The SS18L2 gene is homologous to SS18.[supplied by OMIM, Jul 2002]

tone H3 (H3K4me3), affecting histone demethylase specificity: in presence of H3K4me3, it has

generates formaldehyde and succinate. Involved in hormone-dependent transcriptional activa
g, KIT can activate several signaling pathways. Phosphorylates PIK3R1, PLCG1, SH2B2/APS

Med:15775988, PubMed:19672300). Plays an essential role in the response to a wide variety

and internalization of various plasma membrane channels such as ENaC, SCN2A/Nav1.2, SC

R5A1 and subsequent steroidogenic gene transcription by delivering PA as ligand for NR5A1.
hway that leads to the activation of the transcription factor AP-1. By retaining the phosphatase

Has AP (apurinic/apyrimidinic) lyase activity and introduces nicks in the DNA strand. Cleaves th

dherens-type junctions, such as to the costameres, neuromuscular junctions, and myotendino

gene, but only the longest is a polycistronic transcript containing the MIR17-92 cluster. [provide

of inflammatory genes (PubMed:11909979, PubMed:12569367, PubMed:12763138, PubMed:9
e pulmonary fibrosis. Alternative splicing results in multiple transcript variants encoding distinct

can be either protein-coding or non-coding. The primary transcript is cleaved by the Drosha ribo
DNA replication. In addition to its role in maintenance of DNA methylation, also plays a key role

g pathway (PubMed:28784619). Plays a role in the regulation of blood pressure in response to
urvival of postmitotic cortical neurons. Specifically binds the consensus DNA sequence 5'-[AC]

can be either protein-coding or non-coding. The primary transcript is cleaved by the Drosha ribo

28813410). Through a yet unknown activating receptor, may costimulate T-cell subsets that pre
ors SMAD2 and SMAD3 to the TGF-beta receptor. Phosphorylated by the receptor, SMAD2 an
can be either protein-coding or non-coding. The primary transcript is cleaved by the Drosha ribo
3N)RGYAAC-3' present on promoters (PubMed:10330134). Probably activates transcription of
nctions in vivo as an activator of the phospholipase PLCD1. Active DLC1 increases cell migrati

ndem 5'-AGGTCA-3' sites known as DR1-DR5. In the absence or presence of hormone ligand
d development of the peripheral nerve myelin sheath. Required for vesicular recycling of CDH1

ctivation (PubMed:15817639, PubMed:23401856).

ı two electrons provided by NADPH via cytochrome P450 reductase (CPR; NADPH-ferrihemop

otassium; as external potassium is raised, the voltage range of the channel opening shifts to m
tory receptor function is phosphorylation- and PTPN6 -dependent, which in turn, suppress sign

partners to form a joint molecule between a processed DNA break and the repair template. Bin

an activated GPCR promotes GDP release and GTP binding. The alpha subunit has a low GTI

can be either protein-coding or non-coding. The primary transcript is cleaved by the Drosha ribo

stem cells, and in preventing their differentiation. Required for establishing the barrier function c
s (PubMed:16730711). Plays a role in the mitogen-activated MAPK2/3 signaling pathway, posi

r, thereby diminishing H3K9ac, H3S10ph and H4K8ac levels. Promotes TP53 'Ser-15' phospho

Deacetylates 'Lys-382' of p53/TP53, however with lower activity than isoform 1. In combinatior
tion (By similarity).
ved in activated KRAS-mediated transcriptional activation of USP28 in colorectal cancer (CRC)
y similarity).

e (LAP), which is the regulatory chain of TGF-beta, and regulates integrin-dependent activation
calmodulin and protein phosphorylation mechanisms control its activity. [provided by RefSeq, J

tide (LAP), thereby playing a key role in TGF-beta-1 activation on the surface of activated regu

:an be either protein-coding or non-coding. The primary transcript is cleaved by the Drosha ribo
optosis (PubMed:11241278). Binds specifically to G-actin and blocks actin polymerization (By s
tion of histone H2A 'Lys-119', rendering chromatin heritably changed in its expressibility (PubM

CTNNB1. TLE1, TLE2, TLE3 and TLE4 repress transactivation mediated by TCF7L2/TCF4 ar
Med:26100909). Binds to unmethylated major satellite DNA and is involved in the recruitment c

binding of CDC20 to APC/C, independently of its kinase activity. The other is to monitor kineto
E and MAD2L1. Required for the kinetochore localization of PLK1. Required for centromeric en

site, it is predicted to be non-functional.[provided by RefSeq, Nov 2009]

g premature release of meiosis-specific REC8 cohesin complexes from anaphase I centromere

: complex.
sor by associating with CBX4 and independently of DNA methylation. Seems to be involved in
naintains CENPE levels at kinetochores during chromosome congression.

nd chromosomal congression during mitosis where it stimulates microtubule polymerization.

. In addition stimulates TMEM119, which upregulates the expression of ATF4 (PubMed:243624
sion of TFAP2A; impairs binding of BCL6 and KDM2B to TFAP2A promoter regions. Via repres

imilar levels of activity toward both types of substrates (PubMed:12032167). Hydrolyzes high d
ardiac valve and septum development. May be required in conjunction with HEY2 to specify art

-N-glycolylneuraminic acid (CMP-Neu5Gc), a sialic acid abundantly expressed at the surface o

n loop and phosphorylate their substrate on serine and threonine residues (PubMed:9748265,
nethylation of dimethylated 'Lys-9' of histone H3 (H3K9me2), followed by transcription activatic

proliferation and differentiation, regulation of the cell cycle and induction of growth arrest and p

formation. Several transcript variants encoding different isoforms have been found for this gene
hrough the inhibition of MEF2A and GATA4 (By similarity). Involved in podocyte differentiation (
nts and cell cycle progress. Required for efficient DNA repair, including repair of inter-strand cro
sphate and 5'-OH termini. There are no conserved sequences at the splice sites, but the intron
OR1-containing dimers with GATOR2 which can in turn activate mTORC1 and the TORC1 sign

ediated phagocytosis (PubMed:14592415, PubMed:12917409, PubMed:12925201, PubMed:14

vhen transposons are mobilized as a consequence of wide-spread genomic demethylation (By
vity and growth inhibition. Positively affects neuronal outgrowth. Plays a role as a regulator for p
l via a complex set of post-translational modifications of histones, also called histone code, and

l ubiquitin-mediated endoplasmic reticulum-associated degradation (ERAD) of HMGCR via recr

in the canonical Wnt/beta-catenin signaling pathway (PubMed:10557084). The canonical Wnt/

se, nuclear accumulation of beta-catenin and activation of Wnt target genes. A second signalir
e assembly of robust apicobasal microtubule arrays (By similarity).

626). Specifically reduces kainate receptor-mediated currents in hippocampal neurons, most pr

can be either protein-coding or non-coding. The primary transcript is cleaved by the Drosha ribo

Ullu and Weiner, 1984 [PubMed 6084597]).[supplied by OMIM, Jul 2008]

plex (LFC) on the membrane of lysosomes, which inhibits the GTPase-activating activity of FL(
008]
as a scaffold regulating CPC localization and activity. The C-terminus associates with AURKB (
horylates and activates LATS1/2 in complex with its regulatory protein MOB1, which in turn ph

ic T-cells (PubMed:10438909). Protects against staurosporin-induced cell death (PubMed:274[illegible]

ated tumor cells. Able to phosphorylate 'Ser-464' of LATS1.
l via a complex set of post-translational modifications of histones, also called histone code, and

body wall, limb and neural tube development. They also suppress a number of genes including

.P1 as a transcriptional coactivator of estrogen receptor-responsive genes. Stimulates histone

Involved in hematopoiesis; involved in differentiation of hemopoietic progenitor cells, increases

bMed:16968778). Involved in bone morphogenetic protein (BMP)-mediated cardiac-specific ge

he nuclear pore complex. It is believed that this RANBP2 gene family member arose from a du

ls and extracellular matrices. Can also sulfate Gal residues of keratan sulfate, another glycosa
and either IL11RA1 or IL11RA2 activates a signaling cascade that promotes cell proliferation ([illegible]

tes ubiquitination and subsequent proteasomal degradation of MAVS (PubMed:23087404). Pla

nd binding mono- and dimethylated histone H1b/H1-4 at 'Lys-26' (H1bK26me1 and H1bK26me
the cell and promoter context. The heterodimer formed with GATA proteins is essential to acti
RNA and leaves a 5'monophosphate on the RNA. Catalyzes also the cleavage of the cap struc
d motor enzyme that moves antiparallel microtubules in vitro.

receptors EDAR and EDA2R (PubMed:8696334, PubMed:11039935, PubMed:27144394). Ma
also recognizes FapyA, FapyG, 5-OHU, 5-OHC, 5-OHMH, Tg and 8-oxoA lesions in ssDNA. No

2 and KCND3 trafficking to the cell surface. May be required for the expression of I(To) current

plex by sequestering CDC20 until all chromosomes are aligned at the metaphase plate.

the signaling pathways activated by these second messengers with different cellular targets a
aration as well as maturation, chromosomal alignment, spindle assembly checkpoint, and cytok

SMAD4 for receptor-activated SMAD1-binding. Binds to regulatory elements in target promoter

eptor complex. Also acts by recruiting the PPP1R15A-PP1 complex to TGFBR1, which promote
the activity of the SMAD3/SMAD4 transcriptional complex (PubMed:16777850). Involved in tra

ementary sequence which is possibly excised after transcription. This gene has a pseudogene

cleotide insertion-deletion loops (IDL) in the DNA. After mismatch binding, forms a ternary com
226593, PubMed:11939906, PubMed:19540099). The cyclooxygenase activity oxygenates ara

e strand being repaired and promotes genomic rearrangements, such as telomere fusions, so

ne area postrema and nucleus tractus solitarius of the brainstem (PubMed:28953886, PubMed:
from g1 to s phase. E2F2 binds specifically to RB1 in a cell-cycle dependent manner.
x components TSG101, HGS and STAM to cytoplasmic membranes (PubMed:23166352). Pro
of ciliogenesis through phosphorylation of PKN2 and promotion of RhoA signaling (PubMed:27

of cancer cachexia. May play a critical role in follicular rupture.

can be either protein-coding or non-coding. The primary transcript is cleaved by the Drosha ribo

kappa-B subunit. Involved in the activation of a subset of antiapoptotic genes including TNFAIF

esolution of acute postoperative allodynia (By similarity). Also important for dephosphorylation o
ading to cell proliferation through interaction with RAB5A and subunits of the NuRD/MeCP1 co

of the ERK1/ERK2 signaling cascade (PubMed:25646692). May also be important for proper c

/ represses transcription of classical E2F transcription factors such as E2F1. Acts as a regulato
can be either protein-coding or non-coding. The primary transcript is cleaved by the Drosha ribo
and NADPH production (PubMed:16839880, PubMed:22887998). Contributes to the generation

th arrest and differentiation of the neuronal precursors (By similarity). Modulates transcription r
ol) and 1alpha-hydroxyvitamin D(3) (alfacalcidol) forming 25-hydroxy derivatives (PubMed:154
their runt domain, while CBFB is a non-DNA-binding regulatory subunit that allosterically enhar
GF receptor/EGFR with higher affinity than EGF itself and is a far more potent mitogen for smoo
cycle to promote the loading of the mini-chromosome maintenance (MCM) complex onto DNA
ry protein SAV1, phosphorylates and activates LATS1/2 in complex with its regulatory protein M

PubMed:23981774, PubMed:20506325, PubMed:23974653). Also involved in the production of
C5 (PubMed:24793696). Cytoplasmic anchor protein in p53/TP53-associated protein complex.
E complex formation and downregulate short-term facilitation.
cription of SMAD7 which enhances TGF-beta signaling (By similarity). Induces apoptosis (By s

into proteins.

/s also an important role in asymmetric cell divisions (PubMed:21816348). Has guanine nucleo

and motor proteins (By similarity). Promotes neuronal axon elongation in a kinesin- and JNK-d
3251453). Can repress transactivation mediated by TCF12 (PubMed:16803958). Acts as a neg

and acts as a transcriptional repressor (By similarity). Binds to and modifies the activity of TCF

RPE, SNRPF and SNRPG that assemble in a heptameric protein ring on the Sm site of the sm
ɔn.

onents ARTNL/BMAL1, CLOCK and CRY1. Also regulates genes involved in metabolic functio
IA lesions and influences diverse endpoints in the cellular DNA damage response including cel

16311512). Mediates MAPK14 dephosphorylation and inactivation (PubMed:21283629). Is alsc
cells and chromatin condensation in meiotic cells. Regulates centrosome separation (essentia

airing-independent synaptonemal complex formation, ATR recruitment to unsynapsed axes, m
ıalization in uninfected cells.||Binds LDL, the major cholesterol-carrying lipoprotein of plasma, a

y, that are essential for naive pluripotency and self-renewal maintenance of embryonic stem ce
esis. Does not display glycerophosphodiester phosphodiesterase activity, since it cannot hydro

can be either protein-coding or non-coding. The primary transcript is cleaved by the Drosha ribc

important role in integrin beta-1 or B cell antigen receptor (BCR) mediated signaling in B- and T

take of lipid signaling molecules. May be responsible for the maintenance of asymmetric distrib
represent a tumor progression marker.

CH1 activation by DLL1 via an increase in its binding to DLL1 (By similarity). Decreases the bin

Third, by activating RAB5A, possibly by functioning as a guanine nucleotide exchange factor (
lly inactivate the current of KCNBMA. Isoform 4 induces a fast and incomplete inactivation of K

lucose tolerance. Does not promote an increase in a thermogenic gene program via direct acti

promoter in natural killer (NK) cells. Plays a role in the development and function of NK and NK
Med:19489739, PubMed:29018201, PubMed:31398338). In normal conditions, ubiquitinated a
lymphoid and non-lymphoid cells (PubMed:10358178). Required for osteoclastogenesis and r
not affect neuron migration per se.

II phosphorylation and subsequent transcriptional elongation (PubMed:12832472, PubMed:14

ntral rod domain. Multiple alternatively spliced transcript variants encoding different isoforms ha
effector of the TTK kinase in the control of attachment-error-correction and chromosome align

slocate to the nucleus and activate transcription.

SNORD83A, function in 2-prime-O-ribose methylation of rRNAs (Duga et al., 2000 [PubMed 1(

ostate carcinogenesis, as shown by the ability to inhibit proliferation and invasion activities of P
nscription of numerous target genes, and thereby plays an important role in regulating the respo

› single-stranded and primed DNAs and has weak ATPase activity that is stimulated by the pre

activity-dependent translation. ARC capsids are endocytosed and are able to transfer ARC mF

ring cells. Plays an important role in angiogenesis and tumor neovascularization. The recruitme

substrates. The CDC20-APC/C complex positively regulates the formation of synaptic vesicle c

nultiple interphase cyclins. Required in higher cells for entry into S-phase and mitosis. Phospho

ll-cell adhesion in angiogenesis (PubMed:12660161, PubMed:16099422).||Magnesium-indepe

orticoid-induced apoptosis (PubMed:23303127). Promotes accurate chromosome segregation

e in a functional FA core complex response to DNA cross-link damage, being required for the s

ript variants. [provided by RefSeq, Sep 2016]
acyl-CoA, but with opposite chiral specificity (PubMed:10671535).

lenic B-cells. Required for normal macrophage polarization and migration towards sites of infla
dCMP. Also displays broad nucleoside diphosphate kinase activity.

hermogenesis under ketogenic conditions, using acetate as a vital fuel when carbohydrate avai
cyl group and endogenous lipids such as triacylglycerol (PubMed:28677105). Converts monoa

localizes to the basal body and contacts the BBSome. Rab8(GTP) enters the primary cilium ar
for retrograde transport of cargo proteins from endosomes to the trans-Golgi network (TGN); t
on (MMP) resulting in the release of PDCD8.
d AP1 transactivation (PubMed:12077347).
is further enhanced by PARP9 binding to poly(ADP-ribose) (PubMed:28525742). In associatio
by repressing JUN transcription via HDAC1 recruitment to JUN promoter.||Responsible for the c
es conformational changes in the client proteins, thereby causing their activation. Interacts dyn

ents (IRES) in target mRNA species when iron levels are low. Binding of a 4Fe-4S cluster prec

Med:20801876). Recruited to endosomal membranes upon nerve growth factor stimulation, indi
ation during angiogenesis. Can initiate events that lead to a mesangial proliferative glomerulone
3) POLZ complex for complete bypass of DNA lesions. Inserts one or 2 nucleotide(s) opposite
ty).
TLA and CD160, altogether defining a complex stimulatory and inhibitory signaling network (Pu

rmation of bones and joints in the limbs, skull, digits and axial skeleton. Plays a key role in esta
e-scavenging receptor or chemokine decoy receptor. Acts as a receptor for chemokines CCL2,
d En-2 expression. May be involved in the development of luteal cells.

this. In vitro, triggers the de novo formation of presynaptic structures. May be involved in specit
ocholate, digoxin nor DHEAS (PubMed:16971491).
it manner (PubMed:16354688). Plays an important role in regulating DNA replication and comı

)1 deacetylation. Negatively regulates the calcineurin/NFAT signaling pathway in cardiomyocyt

ily responsible for incorporating endogenously synthesized fatty acids into triglycerides (By sim

rrier vesicles (ECV) / multivesicular bodies (MVB) transport intermediates from early endosome
in alternative source of energy to glucose, as lipid precursors and as regulators of metabolism.

ex with B2M preferentially presents riboflavin-derived metabolites to semi-invariant TRAV1-2 T
opment of the central nervous system and in neuronal maintenance (Probable). Also acts as a
th AMP and is then transferred to the acceptor end of the cognate tRNA (PubMed:1756734, Pı
iate donor. Also catalyzes the production of ATP, CTP, GTP, UTP, dATP, dCTP, dGTP and T

f cytosine to uracil in the minus-sense single-strand viral DNA, leading to G-to-A hypermutatior

ptotic DNA-damage responses. The specific target gene promoter association seems to be de

aintenance of mitochondrial abundance and morphology (By similarity).
yetanolamline (PE) between membranes.
›X3.

ıp and long-dsRNA (>1 kb in length). Upon ligand binding it associates with mitochondria antivi

ɘ for conversion of the sphingosine 1-phosphate (S1P) degradation product hexadecenal to he

›ly(I:C), or in response to DNA virus infection, including that of Herpes simplex virus 1 (HSV1) (

ı as major constituents of the tight junction complexes that regulate the permeability of epitheli

d laminin, and for normal keratinocyte migration to wound sites. May mediate TGF-beta 1 signa

ansport of L-arginine. Involved in the transport of L-arginine in monocytes.
ı of cortical neuron migration and dendrite arborization (By similarity).
ıssic group of MBP isoforms (isoform 1-isoform 3/Golli-MBPs) may preferentially have a role in

SV).

glandin and collagenase from synovial cells.
:tor proteins important to endothelial cell survival and proliferation.

to the Muc5AC peptide, suggesting that it can fill vicinal Thr/Ser residues in cooperation with c

a lung tumor suppressor (PubMed:18000218).
substituents. Participates in the bioactivation of prodrugs such as famciclovir, catalyzing the ox

nodes of Ranvier and axonal initial segments. Regulates KCNA1 channel activity in function of

promoters. Isoforms are either transcriptional activators or repressors. Plays a role in spermatc
drome. Alternatively spliced variants which encode different protein isoforms have been descrit
nt of Th2 responses.

be required to bind DNA ends and hold them in close proximity. This could facilitate searches

nctions (By similarity). Unable to bind to catenins, weakly associates with the cytoskeleton (By s

development of Leydig cells and spermatogenesis. Required for normal oligodendrocyte deve

ment, activation, growth and motility. [provided by RefSeq, Jul 2008]

nanner and for the acquisition of hippocampus-dependent cognitive function, such as learning
s suggests this gene product may function as a tumor suppressor. [provided by RefSeq, Jul 2C

outgrowth through binding to components of the extracellular matrix such as heparin and collag
vs the recruitment of RNF135 an E3 ubiquitin-protein ligase that activates and amplifies the RI
vation (PubMed:18591935, PubMed:19555689, PubMed:22480440). Represses the beta-cate

NA from bacteria and viruses and promotes the production of type I interferon (IFN-alpha and

II.
litude and duration of the cAMP signal in sensory cilia in response to odorant stimulation, henc

tumorigenesis. Provides kidney medullary collecting duct with high permeability to water, there

ıding appears to require additional receptor determinants exposed only in the active receptor cc

ıxons to distal subiculum, probably by promoting homophilic cell adhesion. Required for proper

ıono-ADP-ribosylation of STAT1 at 'Glu-657' and 'Glu-705', thus decreasing STAT1 phosphory
roteins. RNA-binding protein that binds to 5'ACACCC-3' core sequence, termed zipcode, withir
effects on innate immune cells. Among its target cells are CD4 T-helper cells which can differe
e complex exosome to degrade the RNA body from the 3'-end. It also recruits the decapping cc

‹s myosin VI to the Golgi complex and plays an important role in Golgi ribbon formation (PubM

e dimeric form determines whether glucose carbons are channeled to biosynthetic processes c

g neuronal migration.

repair reactions (PubMed:17353262, PubMed:17396150, PubMed:21211721). Displays apurir

ɔunit. May be involved in transport between a perinuclear compartment and the plasma membr
esponse pathway by mediating the pro-apoptotic effects of the ATF4-ATF3-DDIT3/CHOP casca

thesis (PubMed:10758005, PubMed:12634318). Plays a role in determining the extent and pat
can (PubMed:25279699, PubMed:25279697). Phosphorylated O-mannosyl glycan is a carbohy
PTK7 (PubMed:20837484). Acts as a positive regulator of cell growth and migration via activat
dochondral ossification (PubMed:11788602, PubMed:12163485, PubMed:12446672, PubMed:
ı cell growth, survival, proliferation, motility and morphology. Mediates immune responses. Play

ed by the CALCRL/RAMPs receptor complexes. Activates the cAMP-dependent pathway.
H4 (PubMed:28525742). In response to DNA damage, mediates monoubiquitination of 'Lys-9
ı cell polarity of fibroblasts (By similarity).
nking and/or strengthening of epithelia cell-cell junctions in part by linking adhesive receptors tc

on, and other cellular interactions important for immune response and surveillance.
ubMed:9326939, PubMed:10744784, PubMed:12031666, PubMed:12923223). Does not hydro
tion of phagosomes that engulf pathogens, such as S.aureus and M.tuberculosis (PubMed:212

prevent lysis of infected cells by cytotoxic T-lymphocytes and NK cell.||ICAM proteins are ligand
re and lower potassium levels.
). Core component of the UV-DDB complex (UV-damaged DNA-binding protein complex), a co
ding to the reduction of endosome motility (PubMed:16476778).

g at the cell surface induces autophosphorylation of MERTK on its intracellular domain that pro
cycling to the nucleus, where it is required for 60S rRNA processing and nuclear export. Has lo

.||Receptor tyrosine kinase that transduces signals from the extracellular matrix into the cytopla
RELA/p65, RELB, NFKB1/p105, NFKB1/p50, REL and NFKB2/p52. The dimers bind at kappa-

secondary immune response by co-stimulating memory T-cell function (By similarity).
spase cleavage during apoptosis since N-terminal glycine degrons are strongly enriched at cas

R54. Activation of the receptor inhibits cell proliferation and cell migration, key characteristics o

d:23776175). Involved in the regulation of cell mobility (PubMed:23333244, PubMed:23776175

ation motifs, such as the LDL receptor, to recruit them to clathrin-coated pits. Can function in cl

llular targets and opposite effects in numerous biological processes (PubMed:2175712, PubMe
ucleocytoplasmic interactions established by the LINC complex play an important role in the tra
membrane. It therefore contributes to repolarization of the membrane potential. Plays a key rol
ed lipopeptides, this cluster triggers signaling from the cell surface and subsequently is targeted

96614). Plays a role in the late stages of EGF-induced macropinocytosis (PubMed:24591580).
F/TICAM1, leading to NF-kappa-B activation, IRF3 nuclear translocation, cytokine secretion an
vesicle origin. Interaction with AMPAR subunit GRIA2 leads to influence GRIA2 membrane cyc
on than the uncleaved protein (PubMed:19270337, PubMed:21398697).||Mediates inactivation

e vesiculation in red blood cells.

eric form of ribonuclease L (RNASEL) leading to its dimerization and subsequent activation (Pu
AD/p300/CBP-mediated transcriptional coactivator complex. Stimulates the peroxisome prolifer
h then bind to the inactive monomeric form of ribonuclease L (RNase L) leading to its dimeriza

der different cellular conditions. Plays a role in thyroid hormone receptor and estrogen receptor

ests that it plays a central role in the structure and function of submembranous plaques. Acts as

localizes to the basal body and contacts the BBSome. Rab8(GTP) enters the primary cilium ar

itor cells to post-mitotic neurons requires a switch in subunit composition of the npBAF and nB

the mitochondrial matrix of hydrolases that are characteristic of the lysosomal lumen. Also invo
bsequent transcription of E2F target genes which are responsible for the progression through t

ers of this family differ in the number of C-terminal TS motifs, and some have unique C-termina
e 2-oxoglutarate dehydrogenase complex is mainly active in the mitochondrion (PubMed:2921
ed:15775988). May play a role in programmed neuronal cell death but does not appear to affec
se F(1)F(0) activity and regulates endocytic vesicle retrieval in hippocampal neurons through as

esumption of elongation from the new 3'-terminus.

tubules. The intermediate chains mediate the binding of dynein to dynactin via its 150 kDa com
tion and MAPK1-dependent transcription. Increases cell surface CD4(T4) antigen expression.
ation of filopodia formation and actin filament bundling. Can modulate the effect of DAPK3 in re

lated sequence G-F-P-G-E-R in collagen. It is responsible for adhesion of platelets and other c

‹ degradation processes, promoting cell invasion. Alpha-3/beta-1 may mediate with LGALS3 th

vation. Regulates the excitability of cardiac myocytes by modulating the functional expression o

nes (PubMed:20154091, PubMed:20937701). Binds phosphatidylinositol-phosphates (PtdInsP
ophagy.
to mitochondrial damage, participates in mitochondrial protein catabolic process (also named M

l via a complex set of post-translational modifications of histones, also called histone code, and

ed:11060020). First, a [2Fe-2S] cluster is transiently assembled on the scaffold protein ISCU. In
V core promoter. May have E3 ubiquitin-protein ligase activity.

o hydrolyzes the 5-phosphate of phosphatidylinositol-4,5-bisphosphate (PtdIns(4,5)P3) and ino
induction of other antiviral genes. RNASEL mediated apoptosis is the result of a JNK-depende

n of pain (PubMed:19029917, PubMed:20079333, PubMed:21049984, PubMed:22969151, Pu
serted into adenylated molybdopterin and AMP is released.
l via a complex set of post-translational modifications of histones, also called histone code, and

/ chain of TGFB1, and regulates integrin-dependent activation of TGF-beta-1 (By similarity). TG

ne plasma membrane where it strongly binds to inner leaflet lipids, bisphosphorylated phosphat

:he craniofacial skeleton and for normal development of the palate. Required for normal skin m

:ll death.
:tions are no longer required. Therefore, the proteasome participates in numerous cellular proc
:ctures is much higher than the corresponding desialylated substrate, and only internal Gal resi

n the maintenance of protein homeostasis by removing misfolded or damaged proteins, which

ion regulation via its interaction with VEZF1, by regulating activity of the endothelin-1 (EDN1) p

ss I binding peptides. Replacement of PSMB6 by PSMB9 increases the capacity of the immun

:le myotubes. Required for normal translocation of FER1L5 to the plasma membrane (By simila

ıe uptake of oxaliplatin (a new platinum anticancer agent). Able to transport paraquat (PQ or N,

SMAD4 complex, activates transcription. Also can form a SMAD3/SMAD4/JUN/FOS complex a
osis, vascular permeability, leukocyte diapedesis), cell motility and morphology, airway hyperre

l LRPAP1 (alpha 2-macroglobulin), as well as the local metabolism of complexes between plas

g in response to the retroviral capsid lattice. Binding to the viral capsid triggers its E3 ubiquitin l

and CRY transcriptional repressors (PER/CRY loop) (PubMed:14672706). Both these loops a
in tumor progression and metastasis.
nuclear assembly, chromatin organization, nuclear membrane and telomere dynamics. Requir
pressor. The gene is located in the chromosome 3 common eliminated region 1 (C3CER1), a 1
D6-PARD3 complex may prevent the interaction of PARD3 with JAM1, thereby preventing tight
o small GTPases to atypical protein kinase C proteins (PubMed:10873802). Regulates centros
bsequent transcription of E2F target genes which are responsible for the progression through t

esophageal and ovarian cancers. Alternatively spliced transcript variants encoding different iso

l via a complex set of post-translational modifications of histones, also called histone code, and

exon 5 inclusion of TNNT2 in embryonic, but not adult, skeletal muscle. Activates TNNT2 exon

U]UAA-3' as a bipartite motif spaced by more than 15 nucleotides. Binds poly(A). RNA-binding

f cytosine to uracil in the minus-sense single-strand viral DNA, leading to G-to-A hypermutation
activation, during DNA virus infection but not RNA virus challenge, controls antiviral immunity th
nt attachment of herpes simplex virus ICP47 protein, which blocks the peptide-binding site of T

owth factor-stimulated mTORC1 activation involves a AKT1-mediated phosphorylation of TSC1
DC42.

).||Receptor for the C-X-C chemokine CXCL12/SDF-1 that transduces a signal by increasing in

e the inherent affinity of ribosomes for protein translocation sites in the ER membrane (M sites)
n cell differentiation, localizing near desmosomal plaques in earlier stages of differentiation but

eath (PubMed:20230784). May be implicated in the control of meiosis (By similarity). Involved i
e lipid export complex. Could also be involved in intracellular lipid transport processes. The role

ıd is referred to as reverse signaling. Highly promiscuous, it has the unique property among Ep
of stratification of the squamous epithelium (By similarity).

||Transcription factor involved in unfolded protein response (UPR). Binds the DNA consensus s
s protein may be involved in cell-cell or cell-matrix interactions. Copy number alterations in this

-dependent of the germinal center (GC) reactions, mainly through the control of the GC B-cells

and fibroblastic cells.

ns) with both forms of PRPN, targeting them to lipid rafts and facilitating their interaction. Requ
f the Hippo/SWH (Sav/Wts/Hpo) signaling pathway. May act as a membrane stabilizing protein
gen presenting cells (APC), a prerequisite for efficient T cell adaptive immunity against pathoge

nd other cargos along microtubules.

but when released from granules, these potent enzymes also destroy host proteins and contrik

KP1-CUL1-F-box protein) E3 ubiquitin-protein ligase complexes such as SCF(SKP2)-like comp

nds the 3'-UTR of p53/TP53 mRNAs to control their nuclear export induced by CDKN2A. Henc

x which is involved in transcriptional activation of select genes principally by acetylation of nucl
specific context of hair cycling. Regulates protein synthesis and epithelial cell growth through b

ıkisphosphate, suggesting it is recruited to membranes that contain lipids with a phosphoinositi

ınnel properties of the nAChR inserted in the plasma membrane. Modulates functional properti
mponents of the exocytosis machinery, thus facilitating exocytosis, including insulin release (By

ıal repressor via its interaction with EBF1, a transcription factor involved specification of B-cell

somerization in a range of enoyl-CoA species (Probable). Also able to isomerize both 3-cis and
:tively. Catalyzes the coordinated movement of the two tRNA molecules, the mRNA and confor
ts coactivators, such as NCOA1 or MED1, to the promoter region of the target gene (PubMed:
g required for transport complex III (ESCRT-III) which is involved in multivesicular bodies (MVE

ıtathione (PubMed:25225291,PubMed:29715001).
(PSMB9/LMP2). Regulates TNF-alpha-induced and LPS-mediated activation of the central me
rectional signaling into neighboring cells (PubMed:8070404, PubMed:7973638). Shows high a

ıorylate CD79A or by recruiting proteins which bind to CD79A and protect it from dephosphoryl

ȷe factor (GEF) which may activate RAB9A and RAB9B. Promotes the exchange of GDP to GT

gression. [provided by RefSeq, Mar 2015]

U-rich regions (URRs) in the 3' untranslated region (3'-UTR) of several mRNAs. Does not bind
ɔdiester phosphodiesterase activity, since it cannot hydrolyze either glycerophosphoinositol or

ɔraneous catalytic core, and F(0) - containing the membrane proton channel, linked together b

can be either protein-coding or non-coding. The primary transcript is cleaved by the Drosha ribo
asome. May mediate the interaction between VCP and the misfolded glycoproteins (PubMed:1

heir rates of activation, deactivation and desensitization and by mediating their resensitization. D

ated to the consensus octamer motif 5'-ATGCAAAT-3' in the regulatory regions of its target gen
rtant role in controlling bulk H3K4me during oocyte growth and preimplantation development (B

logy 3) motif, found in several other proteins that associate with the cytoskeleton and are suspe
mplex, AP-3 is required to target cargos into vesicles assembled at cell bodies for delivery into
can be either protein-coding or non-coding. The primary transcript is cleaved by the Drosha ribo

us. Within the microprocessor complex, DGCR8 function as a molecular anchor necessary for t
ng pathway involving the PI3, Akt and MAP kinases. CCL5/RANTES may also regulate insulin s

3'-5' proofreading exonuclease activity that corrects errors arising during DNA replication (By sin

) (PubMed:22722849, PubMed:30420520). In contrast to other histone deacetylases, displays s
ed:1741402, PubMed:16397300). Transcriptional activator that enhances IL6 transcription alone

ll proliferation in vitro. It also interacts with the tumor suppressor p53, and regulates p53 target
ATP-dependent manner. Stimulates nuclear receptor mediated transcription. Belongs to the ne

ably implicated in the regulation of exocytosis (By similarity).

cilia (PubMed:19592253). Interacts with DLG5 at the ciliary base to induce the accumulation of

way); enhances NXF1-NXT1 RNA-binding activity. RNA-binding is semi-sequence specific.

elf, thereby modifying the interface between stalled RNA polymerase II and DNA (PubMed:155

h peptides at physiologically relevant doses inhibit basal ACTH secretion. Both peptides appea

promoting their GTPase activity and indirectly by disrupting the RAB9A-HPS4 interaction which

tor for leukotriene B4, a potent chemoattractant involved in inflammation and immune response

ation by mediating HDAC7 nuclear export, cell proliferation via MAPK1/3 (ERK1/2) signaling, a

ants have been observed. [provided by RefSeq, Dec 2017]

of BRCA2 recruitment and upstream of RAD51 recruitment. BCDX2 binds predominantly to th

AD51 recruitment; the complex binds predominantly to the intersection of the four duplex arms

. Acts by specifically elongating 'Lys-11'-linked polyubiquitin chains initiated by the E2 enzyme

cine side chains. Not involved in elongation step of the polyglycylation reaction.

A. Interacts with other spliceosomal components, via the RS domains, to form a bridge betwee

Rs) of stress-responsive transcripts RPA2 and TXN. Acts as a translational repressor (By simil

n during G2 phase and a minimum as cells enter M phase. Phosphorylation of cyclin B1-CDK1

or. It is derived from the Latin roots 'circa' (about) and 'diem' (day) and acts as an important re

7 (LIN7A, LIN7B or LIN7C), CASK and APBA1 associates with the motor protein KIF17 to trans

duced affinity for RNA.

s monoubiquitination of histone H2A 'Lys-119', rendering chromatin heritably changed in its exp

hoenixin-20 induced granulosa cell proliferation during follicular growth (PubMed:30933929).
o promote progression through the cell cycle via angiotensin I converting enzyme 2 and cyclin

onse and immune homeostasis, glial differentiation of neuroprogenitor cells, cell death of cardic

ithesized CENPA into centromeres. Part of a nucleosome-associated complex that binds speci
can be either protein-coding or non-coding. The primary transcript is cleaved by the Drosha ribo

teins from speckles to a diffuse nucleoplasmic distribution. Acts as a suppressor of hepatic gluc
can be either protein-coding or non-coding. The primary transcript is cleaved by the Drosha ribo

7me3, respectively. Displays a preference for substrates with less methylation, loses activity wh
ecules involved in centriole formation (PubMed:21059844, PubMed:20852615). Proposed to sr
can be either protein-coding or non-coding. The primary transcript is cleaved by the Drosha ribo

It therefore negatively regulates cell spreading and cell migration.

e. Promotes cell differentiation (By similarity).
e. Promotes cell differentiation (By similarity).
pa-B-mediated transcription. In vitro, upon association with NFKB1/p50 has transcriptional activ
ed:24396869, PubMed:28114273). In vitro also methylates unmodified 'Lys-20' (H4K20me0) of

and stearoyl-CoA (PubMed:15907797, PubMed:18765284). Gives rise to a mixture of 16:1 and

ED-71 or 1alpha,25-dihydroxy-2beta-(3-hydroxypropoxy)-cholecalciferol), a second-generation v

e the sugar-phosphate structure of double-stranded DNA. Transcriptional repressor controlling r

CIII) and cytochrome c oxidase (complex IV, CIV), that cooperate to transfer electrons derived

equired for CEP152 localization to the deuterosome.
narker of tumor progression. [provided by RefSeq, Dec 2017]
or. It is derived from the Latin roots 'circa' (about) and 'diem' (day) and acts as an important reg

vation of transcription factors such as ELK1 and ATF2. Accordingly, p38 MAPKs phosphorylate
brane. Acts as a chaperone-like protein that protects the hydrophobic precursors from aggregat

ctions as a regulator of apoptosis. Mediates effects of polyamines on neuronal process extensi
or. It is derived from the Latin roots 'circa' (about) and 'diem' (day) and acts as an important reg

a TRAF2-dependent pathway. Acts as a downstream mediator for CASP8-induced activation o

cetylase activity has been observed in vitro (PubMed:11861901, PubMed:11726666, PubMed:

A molecules that contain AU-rich elements (AREs) found within the 3'-UTR of many proto-onco

nd adenocarcinomas. Transcription from this gene is negatively regulated by insulin and insulin
in stabilization of nucleolar transcription factor 1/UBTF on rDNA. Formation of SL1/TIF-IB excl

es, inactive towards water soluble inositol phosphates (PubMed:10764818). Plays an essential

h linker DNA between nucleosomes and functions in the compaction of chromatin into higher o
the transcriptional activation of the ELA1 enhancer; the complex binds to the enhancer B elem
en the SLX1A and SULT1A3 genes located approximately 730 kb downstream on the same ch
braneous catalytic core and F(0) - containing the membrane proton channel, linked together by
ne H3 and H4 trimethylation marks H3K9me3, H3K27me3 and H4K20me3.

, PubMed:18955485).||(Microbial infection) Acts as an attachment factor for Human immunode
:an be either protein-coding or non-coding. The primary transcript is cleaved by the Drosha ribo

›tor recycling. Regulates the trafficking of endosomal alpha5beta1 integrin to the plasma memb

:ive selection of thymocytes.

nediates coupling to G proteins (PubMed:18165688). Ligand binding causes a conformation ch

ntribute to stimulation of phagocytosis of apoptotic retinal pigment epithelium (RPE) cells and n

:an be either protein-coding or non-coding. The primary transcript is cleaved by the Drosha ribo

16617059, PubMed:17472963, PubMed:27642067, PubMed:18451993). Plays an important ro

PubMed:23763432). Also binds dehydroepiandrosterone (DHEA), pregnanolone, pregnenolone
:onstitutes the major mechanism by which docosahexaenoic acid (DHA), an omega-3 fatty acid

he erythrocyte membrane. Allelic variants, some of which lead to adverse transfusion reactions

in stabilization of nucleolar transcription factor 1/UBTF on rDNA. Formation of SL1/TIF-IB excl
l apoptosis.
telets: rapidly and quantitatively secreted from platelets in response to stimulation of platelet de
-renewal (By similarity).

or. It is derived from the Latin roots 'circa' (about) and 'diem' (day) and acts as an important reg
d:27789713).

(PubMed:12482949). Involved in fluid-flow mechanosensation by the primary cilium in renal ep
regulates the actin cytoskeleton and promotes synaptic transmission (By similarity).

otein product. [provided by RefSeq, Oct 2011]

family of ribosomal proteins. It is located in the cytoplasm. As is typical for genes encoding ribo

ular smooth muscle cells vasoconstriction. Positively regulates bone mineralization through abs
ically binds the myristoyl moiety of a subset of N-terminally myristoylated proteins and is requir

ns it can bind to the DNA binding domain of the glucocorticoid receptor (nuclear receptor subfa

e immune response. [provided by RefSeq, Dec 2017]

ng DNA replication, DNA repair and heterochromatin organization as well as in ribosomal RNA

nanner. May regulate the TRAX/TSN complex formation. Potentiates transcriptional repression

e biogenesis by suppressing the formation of aberrant centriolar protein complexes in the cytop

d, phenylpyruvate and alpha-oxo-gamma-methiol butyric acid. Can also use glyoxylate as amir

praneous catalytic core, and F(0) - containing the membrane proton channel, linked together by

e association of CREBBP. Seems to act as a corepressor of SIX6 in regulating proliferation by

can be either protein-coding or non-coding. The primary transcript is cleaved by the Drosha ribo

(PubMed:27193190, PubMed:27103069, PubMed:27617292, PubMed:28195531). In the com
nechanism occurs as neurons exit the cell cycle and become committed to their adult state. The

ated in vesicle formation and in uptake of lipid signaling molecules. In vitro, its ATPase activity
omoters including FOS, OSM and PPARG. Recruits a histone deacetylase complex (By similari
can be either protein-coding or non-coding. The primary transcript is cleaved by the Drosha ribo

can be either protein-coding or non-coding. The primary transcript is cleaved by the Drosha ribo

can be either protein-coding or non-coding. The primary transcript is cleaved by the Drosha ribo

catalyzing monoubiquitination of FANCD2, a key step in the DNA damage pathway (PubMed:16

:ransporter SLC2A2 by binding specifically to its promoter region and recruiting PDX1 and addi

general. Members of this gene family are characterized by tandemly repeated copies of DUF1:

ed:10681376). Mechanistically, uses molecular oxygen inserting one oxygen atom into a subst

:an be either protein-coding or non-coding. The primary transcript is cleaved by the Drosha ribo

the influx of which may activate a potassium current which hyperpolarizes the cell membrane.
oit the myosin phosphatase activity to regulate myosin phosphorylation. Also critical to the modu
APC/C complex. Also mediates deubiquitination of 'Lys-48'-linked polyubiquitin chains in vitro. F

By similarity).

n at the metaphase-anaphase transition permits replication in the succeeding cell cycle (PubMe

:an be either protein-coding or non-coding. The primary transcript is cleaved by the Drosha ribo
LIMK1. Does not dephosphorylate peptides containing phospho-tyrosine (PubMed:15580268).

vith NMDA receptor signaling. Overexpression or depletion of DLG4 changes the ratio of excita

ı sn2. In addition to (C14)-containing phosphatidylcholines, may also act on other medium-cha

ntiation (PubMed:14671321). Plays a role in terminal differentiation of interneurons, such as ar

can be either protein-coding or non-coding. The primary transcript is cleaved by the Drosha ribc

enance and telomere capping in mammalian cells.

gene. [provided by RefSeq, Oct 2009]
ning to disrupt the interaction of these protein components. This downregulation of ERAD may

the conversion of H(2)S to thiosulfate, and the sulfur dioxygenase (SDO) which uses GSS(-) as

sis induced by oxidative stress (PubMed:11007892, PubMed:22010141). Also protects against

oreaks by homologous recombination. The complex may promote sister chromatid homologous

carcinomas (HNSCC) (PubMed:12414649, PubMed:15700036).

46 in the U5 small nuclear RNA. [provided by RefSeq, Jun 2010]

NORD43, function in 2-prime-O-ribose methylation of rRNAs (Duga et al., 2000 [PubMed 1068

ınique cytoplasmic tails. [provided by RefSeq, Jul 2008]

sylation. N-glycosylation occurs cotranslationally and the complex associates with the Sec61 co
can be either protein-coding or non-coding. The primary transcript is cleaved by the Drosha ribo
can be either protein-coding or non-coding. The primary transcript is cleaved by the Drosha ribo

l (GPI) anchor that is directly involved in signal transduction (Mallya et al., 2002 [PubMed 1207
3). Positively regulates SHH signaling in the presence of SHH: dissociates from SUFU, autoph

esults in multiple transcript variants. [provided by RefSeq, Aug 2014]

se of a repressor complex and a recruitment of an activator complex. In resting neurons, transc

n genes, thereby delaying the differentiation of neuronal progenitors and leading to an overall f
PubMed:25320081). Is inhibited strongly by pyridoxal 5'-phosphate, 4,7-diphenyl-1,10-phenant

ylation step of the phospholipid remodeling pathway also known as the Lands cycle.

etween TRPA1 and TRPV1, two molecular sensors and mediators of pain signals in dorsal roc
ed by Polycomb activities in sexually uncommitted germ cells. Regulates expression of STRA8
rom G1 to S phase. E2F1 binds preferentially RB1 in a cell-cycle dependent manner. It can me
survival. Signaling appears to involve the PI3 kinase pathway. Involved in regulation of the secr
ncreases the presence of the channel complex at the cell membrane (PubMed:15615847). Rec

Required for normal hearing (PubMed:29293958).
for skeletal homeostasis. Promotes mammary mesenchyme differentiation and bud outgrowth l

)]

ded by RefSeq, Jan 2009]

CIII) and cytochrome c oxidase (complex IV, CIV), that cooperate to transfer electrons derived
prostate cancer.

Binds to the consensus site 5'-GCGCCC-3' in the promoter of PTCH1 (By similarity).

re it is thought to form molecular scaffolds for ribonucleoprotein complexes. It may act as a trar

ation of actin polymerization (PubMed:26573021).

unoglobulins serve as receptors which, upon binding of a specific antigen, trigger the clonal ex
ment, activation, growth and motility. [provided by RefSeq, Jul 2008]
e ephrin ligand is referred to as reverse signaling. Among GPI-anchored ephrin-A ligands, EFN
gulates glycogen synthase synthesis by mediating phosphorylation of GYS1, leading to GYS1 i
tubules. May play a role in changing or maintaining the spatial distribution of cytoskeletal struct

3y similarity).
10329423, PubMed:19065001, PubMed:26195816). The cholesterol ester is then transported

sociated double-strand breaks. It may act by mediating the assembly of RAD51 filaments on ss
ate the transport and translation of mRNAs, modulating for instance the expression of proteins

duction elongation by phosphorylating the CTD (carboxy-terminal domain) of the large subunit c
xcision repair (LP-BER). The 9-1-1 complex stimulates DNA polymerase beta (POLB) activity b

radation of target proteins (PubMed:14528312).

SNORD83B, function in 2-prime-O-ribose methylation of rRNAs (Duga et al., 2000 [PubMed 1(
elivery into neurites and nerve terminals. The BLOC-1 complex, in association with SNARE pro
bMed:18180299, PubMed:19131338). Plays an important role in neural progenitor cell prolifera
:8647185, PubMed:20421648). Delivers inhibitory signals upon binding to ligands, such as FGL

h the synthesis of lipids and the degradation or generation of lipid-signaling molecules like diac
nor cells. The encoded protein is thought to function as a a negative regulator of epidermal gro
ponent of the pH buffering system for maintaining acid-base homeostasis. Acts as a versatile D

sed fatty acid oxidation and glucose uptake. Has intermediate affinity for globular and full-lengtl

.

o insulin stimulation and serves as an adapter protein to recruit PDPK1 to activated insulin rece
ıdjacent cells, leading to contact-dependent bidirectional signaling into neighboring cells. The s

-induced apoptosis. Promotes apoptosis by regulating three pathways, apoptosome up-regulat

can be either protein-coding or non-coding. The primary transcript is cleaved by the Drosha ribo

some cellular genes in CREBZF-expressing cells.

erexpression increases adherence. Neither protein has to be glycosylated for the interaction to
/CD2 interaction may prime response by both the CD2+ and LFA-3+ cells.
of the active GTP-bound form (PubMed:7479768, PubMed:17116687). The central Dbl homolog

or of T-cell coactivation, by binding DPP4 (PubMed:20959412). Its interaction with DPP4 regula
dependent endocytosis in which cargo proteins are incorporated into vesicles surrounded by cla
s a role in the final digestion of peptides generated from hydrolysis of proteins by gastric and pa

nvolved in negative regulation of the survival capacity of activated CD4(+) and CD8(+) T cells (

eudogene has been identified in the pseudoautosomal region of chromosome Y. [provided by R

tress pathway (PubMed:24035498).

ent T-cell populations from non-lymphoid organs. Plays a role in the development, retention an
G-protein coupled receptors, antigen receptors and integrins. Induces tyrosine phosphorylation
manner (PubMed:27076518, PubMed:15102863). In response to endoplasmic reticulum stress
TR) ARE mRNA-binding adapter protein to communicate signaling events to the mRNA decay

nyosin ATPase by binding to F-actin. This inhibition is attenuated by calcium-calmodulin and is

pressor activity, leading to translation of CPEB3 target mRNAs (By similarity).
pper domain and the membrane attachment domain. Cleaves and activates caspase-6, -7 and
lpha subunits and can functionally regulate their activity (By similarity). Involved in the costimula

components. Presents carbohydrate ligands to selectins.

tion and homing of T-lymphocytes, hematopoiesis, inflammation and response to bacterial infe
tigen processing by stabilizing peptide-free class II alpha/beta heterodimers in a complex soon

pendymocytes in the adult subependymal zone: upon cleavage by MMP24, CDH2-mediated ar
rough control of the cohesion and organization of the intercellular junctions (By similarity). It ass

8967). Requires substrates with an unsubstituted N-terminus (PubMed:19038966).

similarity).

inflammatory processes. Plays an important role in the regulation of osteoclast proliferation and

ated in tumor invasion and metastasis (PubMed:3972105).
olysis of TG/thyroglobulin in the thyroid follicle lumen (PubMed:11082042).

of leukocytes through adhesive interactions with JAML a transmembrane protein of the plasm
n important role in the regulation of pathways leading to NF-kappa-B activation (PubMed:1291
depending on the cellular setting. Involved in regulation of starvation-induced autophagy. Regu
ses 5' single-stranded RNA overhang nuclease activity. Possesses ATPase activity on various l

JT1, and hence stabilizes and attaches the spectrin-actin network to the erythrocytic plasma m
the ephrin ligand is referred to as reverse signaling. Functions in axon guidance during develo

ıtion (PubMed:22727489).
. Can also be transactivated by cleaved F2R/PAR1. Involved in modulation of inflammatory res
ırked preference for Ala/Ser-Gly-Pro-Ser/Asn/Ala consensus sequences, on substrate such as

gulator of appetite and adiposity; increased expression of the protein in liver after nutrient exce
dulate the RYR1 calcium channel activity. PPIases accelerate the folding of proteins. It catalyz
he cortical plate. Various interactions and localizations of isoforms affect myotube morphology
r maintaining the structural integrity of the muscle fibers.
egulates angiogenic sprouting. Signaling by activated FLT4 leads to enhanced production of VI
omain. Following activation by PKA, the protein subsequently associates with PTK2/FAK1, allo

1828). Plays a role in ebolavirus and marburgvirus cell entry by apoptotic mimicry (PubMed:17

(By similarity).

gh concentrations of dipeptides and some amino acids, can also catalyze a transpeptidation re
PubMed:11929858, PubMed:23238346). Heteropentameric channels composed of GLRB and
ıeta-hexosaminidase A. It extracts single GM2 molecules from membranes and presents them
Activates effector molecule RhoA by binding and activating RhoGEFs (ARHGEF12/LARG) (Pu
denylate cyclase: they inhibit the cyclase in response to beta-adrenergic stimuli. May play a rol
Rs, including adrenergic receptors, muscarinic acetylcholine receptors (more specifically Gi-cou
reby inhibiting 'Lys-63'-linked ubiquitination (By similarity). In the nucleus, can both acts as a co

in, but in a glycosaminoglycan-independent manner.

regions.
eby replacing linker histones at target enhancer and/or promoter sites. Binds DNA with the cons

translocation of BAX from the cytosol to mitochondria in response to cellular stress, thereby p
pha-V/beta-6. In tumors, stimulates angiogenesis by elongation, migration and sprouting of enc

r, composed of the subunits IL15RA, IL2RB and IL2RG, which results in kinase SYK activation
hemidesmosome (By similarity). ITGA6:ITGB4 binds to NRG1 (via EGF domain) and this binc
lation through binding the site II (HiNF-M) promoter region of H4 and activating transcription du
dditional roles in the maturation of snRNAs and rRNAs, and in ribosome biogenesis.

ɘr potassium channels are characterized by a greater tendency to allow potassium to flow into

n and integrin alpha-6/beta-4 in hemidesmosomes, (2) signal transduction via tyrosine phosph

/TIM3 (PubMed:16286920). Binding to HAVCR2 induces T-helper type 1 lymphocyte (Th1) dea
iohydrogenation pathway of linoleic acid (LA) (18:2n-6).

nscriptional activation (PubMed:27735137). Shows no activity against histone H3 when it is trin
gnals arising from soluble factors and cell-cell adhesion sites. Also suggested to serve as a sca

ricin binds to the bacterial surface and is crucial for the bactericidal functions. Has some antivi
tein (BMP)-mediated cardiac-specific gene expression.
lls during embryonic development. Acts as surface receptor that triggers tyrosine phosphorylati
ne control of cell growth, survival and apoptosis via p38 MAPK signaling in muscle-specific and
g proliferation, scattering, morphogenesis and survival. Ligand binding at the cell surface induc

ubMed:27545619). Specifically recognizes and binds acylated histone H3, with a preference fo
16807369).

idogen, nascin-C isoform, fibronectin, and type I gelatin.

on-sulfated polysaccharide chains.

r. It is derived from the Latin roots 'circa' (about) and 'diem' (day) and acts as an important regu

ral ganglion neurons to innervate auditory hair cells (PubMed:27162350).
Its activity is mediated by G proteins which activate the phosphoinositide signaling pathway. P
hen bind to the inactive monomeric form of ribonuclease L (RNase L) leading to its dimerization

. Activation by the binding of active CDC42 and RAC1 results in a conformational change and
' which are catalyzed by separate catalytic domains of the enzyme. The first step, catalyzed by
ne and of PKA-dependent phosphorylation of DNM1L (PubMed:28463107).||Cyclic nucleotide
a role in glucose homeostasis and in maintaining normal blood glucose levels in function of nu
lateral border recycling compartment (LBRC) and is also essential for the LBRC membrane tc
ated in vesicle formation and in uptake of lipid signaling molecules. May play a role in asymme

ulates osteogenic cell differentiation and bone mineralization through the cleavage of the MEP

l:9132017, PubMed:11013307, PubMed:19321130, PubMed:21515415, PubMed:29883800). F
on (PubMed:12196628, PubMed:15210733). Plays a role in axon guidance, invasive growth ar

ss I binding peptides. Replacement of PSMB5 by PSMB8 increases the capacity of the immun
d:17331072, PubMed:30367560, PubMed:22801504, PubMed:23867277). In fibroblasts, requi
transduction pathways and phosphoprotein network topology in red blood cells. May play a rol

inhibition of neurite and axonal outgrowth by chondroitin sulfate proteoglycans, also after nerv
of reactive oxygen species (ROS) by NADPH oxidase.
se core vesicles. The RALA-exocyst complex regulates integrin-dependent membrane raft exo
iation.
the endosomes. Promotes neuronal apoptosis by mediating endocytosis of the proapoptotic p

for monocytes and basophils but not neutrophils or eosinophils (PubMed:8627182, PubMed:97

is of dendritic cells (DC), effector/memory T-cells and B-cells and plays an important role at sk

ding to the classical ligand-binding site (site 1) in integrins. In the absence of CX3CR1, binds t

in vivo, and can induce, in vivo, an angiogenic response. In vascular cell cycle, delays the G1

es, and stress fibers (PubMed:22155786). Important for the formation of a diverse set of cell pr

04-positive T-cells into peripherally induced regulatory T (pT reg) cells under inflammatory conc

thways that control a diverse spectrum of biological activities including gene transcription, imm

regulation through MLCK during cell spreading, an initial step in cell migration. May play a role

scriptional repressor activity of SIX3 during retina and lens development and for SIX3 transcrip
vels, and amino acids (PubMed:31036939). Interacts with Rag GTPases and increases the lyso

nigration. Its interaction with RHOB may regulate apoptosis. May enhance the PCNA-depender
ecruits the antiapoptotic E3 protein-ubiquitin ligases BIRC2 and BIRC3 to TNFRSF1B/TNFR2.
esponses. Plays a role in the regulation of antiviral responses. Is an essential constituent of sev
n of NF-kappa-B by EIF2AK2/PKR.

lyzes 'Lys-11'- and 'Lys-48'-linked polyubiquitination (PubMed:20061386). Capable, in vitro, to
olipids/GSLs, amphipathic molecules consisting of a ceramide lipid moiety embedded in the ou

pment. Plays a redundant role in maintaining joint integrity.
ndary. It is transcribed, but not expected to make a Y-chromosome specific gene product. Alte
overexpression of RIPK1 and TRAF6 but not of RELA. Inhibits also tumor necrosis factor (TN

and RORC. Involved in the regulation of ovarian function (By similarity). Plays a role in renal d
2 (PubMed:21457715). In concert with MYO18A and LURAP1, is involved in modulating lamella
ines by monocytes and macrophages. Plays an important role in modulating inflammation and

e signaling between axons and myelinating glial cells.
rt arm of chromosome 17. Alternatively spliced transcript variants encoding multiple isoforms h
ethionyl-tRNAi and eIF-5 to form the 43S pre-initiation complex (43S PIC). The eIF-3 complex s

ninal GpppC (PubMed:11114884).
RAILR4 and TNFRSF11B/OPG that cannot induce apoptosis.

proteases) mediating apoptosis. Promotes the activation of NF-kappa-B. Essential for ER stre

nization of the actin cytoskeleton at the plasma membrane. May promote neurite outgrowth (Pu

), PubMed:28404643, PubMed:22622177). Along with WDFY3, involved in the formation and a

6PR), between lysosomes and the Golgi apparatus in a retromer-dependent manner (PubMed:

uple CD2 and CD2AP to WAS. Acts downstream of CD2 and CD2AP to recruit WAS to the T-c

ipogenesis. In addition, promotes expression of the gluconeogenic enzyme glucose-6-phospha

ı of lipid-droplet biogenesis (By similarity). Down-regulates the expression of TGFB1 and TGFE
ed Smads, such as SMAD2.||Plays a role in vesicle-mediated protein trafficking of the endocyti

ıy a critical role as an adapter protein in the formation of the adhesion zone in osteoclasts. Neg

ɪd at 'Lys-27' and trimethylated at 'Lys-27' (H3K9me3, H3K27me2 and H3K27me3, respectively
LL ligands are present in high endothelial cells (HEVs) and play a central role in lymphocyte hc

ifferently regulated and may play an important role in maintaining the plasticity at glutamatergic
ity by retaining JNK in the cytoplasm and inhibiting JNK phosphorylation of c-Jun. May also par

ɪs (ROS) generation and ROS localization. In association with ADAM12, mediates the neurotox
ɪ recycling endosomes and the Golgi. Required for maintenance of the Golgi structure, it is invc
7). Involved in regulation of adherens junction between cells (PubMed:12628187). Plays a role

ivates RAB22A, and promotes polarized trafficking to the leading edge of the migrating cells. P
ates NF-kappaB signaling and cell survival upon DNA damage (PubMed:25861989). Plays a rc
ssibly through a caspase-mediated pathway. Isoform BimAC and isoform BimABC lack the abil
NEDD9 (By similarity). May be involved in the regulation of T-cell receptor-mediated IL2 produc

1336257).
ction with the Arp2/3 complex.
ace and subsequently is targeted to the Golgi in a lipid-raft dependent pathway (PubMed:1688(

species specific. This protein also undergoes N-terminal myristoylation. Alternative splicing re

uitination levels and thus stability of NRF2. Acts as a scaffold protein and facilitates autophagic
16532, PubMed:18215137, PubMed:17222411). Interaction with CD9, on the contrary, attenua
ntaining proteins. Role in G2-M progression in the cell cycle. Represses CBP-dependent transc

nal corepressor for FOXG1B and PAX9. Favors the proliferation of breast cancer cells by repre
calization which is critical for ciliogenesis (PubMed:27224062).

ard long chain acyl CoAs (C14-C20) (PubMed:16940157, PubMed:10944470). The enzyme is i
such as isopentenyl pyrophosphate.

y).

rocesses (PubMed:24995978). TRAK1 and RHOT form an essential protein complex that links

)460). Endosome membrane fission of early and late endosomes is essential to separate regio
s residue generates formaldehyde and succinate.

r extension, such as filipodia in association with ALK1. Involved at the immediate early stage o

endoplasmic membrane. Acts also as a nuclear transcriptional coactivator for PPARGC1A/PP/

ibiquitination and proteasomal degradation of the apoptosis inhibitor BIRC5 (PubMed:2577839

:12699629). Overexpression induces telomere uncapping, chromosomal end-to-end fusions (te
the PKA-dependent processing of GLI3 into GLI3R and repress the Shh signaling. In presence
obably recognizes and binds some squalene structure, suggesting that it may regulate choleste
gulatory factor of ER-alpha.

on of RHOA and phosphorylation of ERBB2 at 'Tyr-1248' (By similarity). Required for normal di
Mediates axon growth cone collapse and plays a repulsive role in neuron guidance via its intera
ed:30918116).

rminally truncated form of APP amyloid-beta peptides [Glu-3]-amyloid-beta. May be involved ir
been shown that it can cleave APP between residues 671 and 672. Responsible also for the p

Med:26431200, PubMed:28229507, PubMed:28834754, PubMed:29670289). Has deoxynuclec
establishment of the blood-brain barrier (By similarity). Acts as a Wnt7-specific coactivator of ca

ulates RRAS signaling which in turn attenuates integrin beta-1 (ITGB1) activation at the cell sur
nd recruits the ATG12-5-16L1 complex, initiating LC3 conjugation, autophagosomal membrane

Binds selectively to GNAZ and GNAI2 subunits, accelerates their GTPase activity and regulate

l TCF4.

terior axial patterning, limb development, somitogenesis and eye formation. In the adult, Dkks a

is required for both normal cell body death and axonal pruning. Trophic-factor deprivation trigg

endently of FADD in certain cell types and also mediates mitochondrial translocation of BAX ar

ponse to ribosome biogenesis perturbation, DNA damage and other stress conditions (PubMec

nstitutive NADPH oxidase which generates superoxide intracellularly upon formation of a comp

al transduction protein involved in the differentiation of fibroblasts into adipocytes and possibly

structural and functional organization of the dendritic spine and synaptic junction.

inhibit tumor cell growth by inhibiting AKT-mediated cell-survival. Also implicated in PI3K-regu
ed:19549071). Binds to the COL2A1 promoter and activates COL2A1 expression, as part of a c

dent, actin-dependent fluid-phase endocytosis. Plays a role in macropinocytosis. Promotes inte

ellular storage (By similarity). Utilizes a wide range of saturated fatty acids with a preference fo

protein receptor, but instead requires the prior addition of a GalNAc on a peptide before addin

cts by forming a complex with RAB18 and ZW10 (PubMed:30970241). Binds to phosphatidylin
ucose levels.
intramembrane cleavage of integral membrane proteins such as Notch receptors and APP (am

phology.
ergy levels and cellular stress, including responses to hypoxia and DNA damage. Regulates p5
so hydroxylates HIF2A. Has a preference for the CODD site for both HIF1A and HIF1B. Hydrox

budding or cell enlargement. The activities oscillate with a period length of 24 minutes and pla

1-dependent ERV silencing in embryonic stem cells.

association. Alternative splicing results in multiple transcript variants. [provided by RefSeq, Ma

ɘby acting as a mediator of the p53/TP53 family to regulate CDKN1A. CDKN1A is a cyclin-depe
R2/CD23 expression.

ubstrates (By similarity).

otase expression and telomerase activity in both ALT (alternative lengthening of telomeres)and

and restriction of plasticity in the adult CNS (PubMed:10667797, PubMed:11201742). Regulat

ty). Plays a crucial role in cholesterol homeostasis in the adrenal gland and has the unique abil

ted substrates.
containing USP12 (PubMed:19075014, PubMed:27650958, PubMed:27373336). Docks at the

onditions (PubMed:11522682, PubMed:11975663). Involved in intracellular deglycation in eryth
esidues Arg and Lys.
MAD7 activates autocatalytic degradation, which is prevented by interaction with SCYE1. Form

ɟron of non-consecutive residues present at the interface of BTB dimers of aberrant compositio

6284, PubMed:20404107, PubMed:20798600, PubMed:23620051, PubMed:23754282, PubM
restricting cell movement, including neuronal migration (By similarity). May be involved in coup

, TRPV5 and TRPV6. Regulates the activity of the thiazide-sensitive Na-Cl cotransporter, SLC

gion. Stabilizes PAK-2p34 thereby increasing stimulation of cell death (By similarity).
efore limits glycogen breakdown. Its activity is inhibited by PYGL, resulting in inhibition of the gl
quence-specificity, and bends bound double-stranded DNA (PubMed:19015240). Binds phosph

on (PubMed:22609986). Plays a role in the stimulation of ZFP36-mediated mRNA decay of sev

mphocytic activity at mucosal surfaces. Could also play a key role in providing the placenta anc
(By similarity). Acts by increasing centrosomal microtubule nucleation and recruiting nucleatior
ustic injury (By similarity) (PubMed:23651124, PubMed:28013291). Also described as a thiami

s-63' polyubiquitination of MAP3K7/TAK1 component leading to the activation of NF-kappa-B (

involved in elongation of the phagophore membrane, the GABARAP/GATE-16 subfamily is es
By similarity). Required for CD4(+) T-cell migration in response to chemokine stimulation by prc

nilarity). Competes with GATA4 for binding and activation of the FGF3 promoter (By similarity).

al cells.

ferrin receptor. May also bind tubulin. May play a role in the regulation of energy homeostasis.

the inhibitor TFPI in endothelial cells (in vitro) (PubMed:21868574).
P-ribosylation of GSK3B, leading to negatively regulate GSK3B kinase activity (PubMed:2333
er formation (PubMed:25448675).

tosis regulating for instance endocytosed-EGF receptor trafficking. Alternatively, may regulate

protein may have important functions in the extracellular matrix. Alternative splicing results in

pressor. Negatively regulates, in vitro, trophoblast invasion during placental development and

idermal growth factor receptor/protein kinase B signaling during tumor pathogenesis. Alternati
ay play a role in calcium signaling by associating with calcium channel proteins. Alternative spli
12]

and process formation during migration (PubMed:22926142). May enhance calcium influx thro

ı at the cell surface. May be involved in the control of anxiety.
ɛ premature or ectopic differentiation of preosteoblast cells during osteogenesis, possibly by ch

ɔt variants. [provided by RefSeq, Feb 2015]

ʋity requires USO1-GOLGA2 tethering and BET1L (PubMed:17141156). VCPIP1 is also require

during synaptogenesis and in synaptic function (By similarity). Involved in the negative regulatio

teins TRADD and RIPK1 to TNFRSF1A and regulates PAK4 protein stability via inhibition of its

of blood cells, endothelial cells and mammary epithelial cells.

,6)P5) (PubMed:12027805, PubMed:12223481, PubMed:28882892, PubMed:30624931). Phos

nd ligand binding. The encoded protein contains twelve SRCR domains, a transmembrane reg
RAF-mediated phosphorylation of MAP2K1/MEK1 (PubMed:29433126). Interaction with BRAF
cytotoxic drug maytansine, which is used in antibody-based targeting of cancer cells. [provided
zation. Plays a role in mitotic clonal expansion during the immediate early stage of adipocyte d

diate cellular signaling pathways regulating several biological processes including cell prolifera

ed by RefSeq, Mar 2014]
bably due to the presence of the glycosaminoglcan (GAG) side chains of heparan and chondr

ine phosphorylation of proteins controlling cytoskeleton dynamics like WASF3 (involved in bran
n (PubMed:15772161). Involved in tight junction assembly in epithelial cells probably through ir

GTP as phosphate donor (PubMed:19073142, PubMed:19766732, PubMed:23416111). Also
subcellular localizations. Most Caucasians have two major isozymes, while approximately 50%

(By similarity). Is also involved in the regulation of intracellular vesicles sorting, modulation of I

also competes with LDLR for binding with PCSK9 (PubMed:18799458, PubMed:24808179, Pu

nducing apoptosis. May act by specifically recognizing and binding histone H2AX phosphorylate
lulates the Cu/Zn nitric oxide-catalyzed autodegradation of GPC1 heparan sulfate side chains i
oproteins and apolipoproteins E and J in the CSF and to HDL particles in plasma, inhibiting me
ells.
hibits the GDP/GTP exchange of RhoB but shows less ability to inhibit the dissociation of preb
g its activity. Involved in membrane trafficking and Ca(2+)-dependent membrane fusion. May p
stabilizing G-rich regions into regular chromatin structures by remodeling G4 DNA and incorpo
of the cell cycle in response to DNA damage. Acts by mediating ubiquitin E3 ligase activity that

nt involved in the translocation includes the region that shares a strong sequence similarity with

term depression (LTD). Binding to NGFR and SORCS2 promotes neuronal apoptosis. Promote

2+) in the erythrocyte (PubMed:28409866). This essential step leads to a rearrangement of the
nternalized by endocytosis and subsequently degraded or they can remain on the cell surface.

ding regulatory subunit that allosterically enhances the sequence-specific DNA-binding capacit
its cytokine production by mature dendritic cells. Interaction with SIRPG mediates cell-cell adh

cell cycle exit during cell differentiation; prevents cell proliferation and regulates negatively cel
its nucleocytoplasmic shuttling by sequestering it in the nucleolus. This inhibits the oncogenic a

y APP, APOC2, B2M, CALCA, CSN3, SNCA and aggregation-prone LYZ variants (in vitro) (Pu
ınce of vessel wall integrity and structure, in particular in atherogenesis.||Vastatin, the C-termin
rus-binding protein. Transcript variants utilizing alternative polyadenylation signals exist for this

;.

duced DNA synthesis.
o be a stable component of E-cadherin/catenin adhesion complexes and to mediate the linkag

ɔn, including the localization of the dopamine receptor DRD1 to the dendritic spines (By similar
ıg the sarcolemma. Also implicated in signaling events and synaptic transmission.
short to bind RPA and becomes a substrate for FEN1. Also involved in 5'-end resection of DN
ıtes Rac Rho small GTPases by exchanging bound GDP for free GTP. Its GEF activity may be
in a T-cell receptor/CD3-dependent manner. Its interaction with ADA also regulates lymphocyt

and STAT3. May also activate the LYN tyrosine kinase.
epresses transcriptional activity of the lutropin-choriogonadotropic hormone receptor/LHCGR g

trienoic acids (EETs) or hydroxyeicosatrienoic acids (HETEs) (By similarity).
)03621). Has mainly an anabolic role in energy metabolism. Mediates hepatic lipogenesis. Pref
' activates 8,9-EET > 14,15-EET > 5,6-EET > 11,12-EET. Modulates glucose-stimulated insulir
bility and calibrating TGF-beta and BMP levels, respectively.
ers the flap from the 5'-end and then tracks to cleave the flap base, leaving a nick for ligation. A
ɔgenesis and normal development of the gonadotropin-releasing hormone (GnRH) neuronal sy

ə regulator of embryonic angiogenesis by inhibiting excessive proliferation of endothelial cells. (

e-scavenging receptor or chemokine decoy receptor. Has a promiscuous chemokine-binding pr

Deficiencies of this enzyme lead to Morquio A syndrome, a lysosomal storage disorder. [provide

leukocytes.
dentical to the elastin-binding protein (EBP), a major component of the non-integrin cell surface

PubMed:30970188).
charidosis type IIID is the least common of the four subtypes of Sanfilippo syndrome. [provided
ulator of lysosomal function and as a growth factor involved in inflammation, wound healing and

, JUN, NFATC1/NFATC, MAPT/TAU and MACF1. Requires primed phosphorylation of the maj

nducible transcription of a reporter gene deriven by the C-FOS serum response element (SRE)
our consecutive reactions breaking down fatty acids into acetyl-CoA (PubMed:29915090). Amo
ctions breaking down fatty acids into acetyl-CoA (PubMed:29915090). Among the enzymes inv
ation of large multiprotein complexes. Deacetylates SP proteins, SP1 and SP3, and regulates
omatin, implying a functional role in the regulation of transcription and mitosis (By similarity).
delivery or facilitate metabolic adaptation to hypoxia. Plays an essential role in embryonic vas
GHLY) and WT1 (TSEKRPFMCAY), all having in common a canonical motif with a negatively ch
I response against tumor cells (PubMed:29531227). Presents viral epitopes derived from HIV-1

enase 2 could be implicated in the production of carbon monoxide in brain where it could act as
HNRNPC tetramers bind 230-240 nucleotides. Trimers of HNRNPC tetramers bind 700 nucleot

ne nucleotide-binding proteins (G proteins) and modulates the activity of down-stream effectors
differentiation, apoptosis, angiogenesis, and neoplastic transformation. Involved in myogenesis

4986). Type I interferon binding activates the JAK-STAT signaling cascade, and triggers tyrosin
other receptor subunit responsible for ligand binding (PubMed:7673114).
compartment where the low pH mediates the dissociation of the complex. This receptor also bir

EM219/IGFBP-3R.

through activation of insulin receptor substrates, IRS1/IRS2.||Soluble IL4R (sIL4R) inhibits IL4
)532). Synergizes with IL12/interleukin-12 to induce IFNG synthesis from T-helper 1 (Th1) cells

ɛir target genes. Binds DNA and mediates the recruitment of the PRC2 complex to target genes
ochemical gradient. The channel is selective for K(+) ions at physiological potassium concentra

chains and are clustered in a region on chromosome 17q21.2. [provided by RefSeq, Jul 2008]

ınal transduction pathways (PubMed:10436159, PubMed:11832213, PubMed:12807904, PubM

rine.

the alpha-v/beta-3 and alpha-v/beta-5 receptors. Also binds to phosphatidylserine-enriched ce
1-6 linkage to the alpha-linked mannose of biantennary N-linked oligosaccharides (PubMed:10

iotensin-2 and angiotensin 1-9 (PubMed:15283675). Involved in the degradation of atrial natriu
: and anti-tumor properties and inhibits cell migration and cell adhesion to FGF2 and vitronectin

ılfide in the brain, retina and vascular endothelial cells. Hydrogen sulfide H(2)S is an important
ction is mechanically antagonized by MYH10.
:he central part but not the margins of spreading cells), and lamellipodial extension; this functio

ational polarity of cilia, and for coordinated, unidirectional ciliary movement in the trachea. Requ
weak ATP binding (By similarity). Functions in a variety of intracellular processes such as vesi

a homo- or heterodimeric complex formed by the Rel-like domain-containing proteins RELA/p65
r of split locus (PubMed:21378985, PubMed:21378989). Affects the implementation of differen

ɔn and chondrocyte maturation via a signaling pathway that involves MAP kinases and leads to

its activation and by dissociating the RAF1/MEK complex and acting as a competitive inhibitor (
T chaperonin.||Acts as a positive regulator of hedgehog signaling and regulates ciliary function

such as BMP2, CALD1 and alpha-integrins.

424, PubMed:15854030). These hydroxylysines serve as sites of attachment for carbohydrate
of virion capsids in PML-NBs thereby preventing their nuclear egress and inhibiting formation of

and fatty acids. Regulates the peroxisomal beta-oxidation pathway of fatty acids. Functions as
, PubMed:17502104, PubMed:23468591, PubMed:27974827, PubMed:22343722). In respons
the DNA polymerase alpha complex (composed of a catalytic subunit POLA1, an accessory su
rate and lipid biosynthesis, as well as cell growth and proliferation. AMPK acts via direct phosp

sphorylating targets such as RAF1, BCL2, CSPG4, TNNT2/CTNT, or activating signaling casc
phorylation. Involved in nephrogenesis by stimulating renal epithelial cell migration and tubulog
rs are toxic to cultured neuroblastoma cells and induce apoptosis (in vitro) (By similarity). Asso

na-carboxylase. The C-terminus is proline-rich containing PPXY and PXXP motifs found in a va
cans, may release soluble FGF-glycosaminoglycan complexes that promote the range and inte
lucosylceramidase (EC 3.2.1.45) and galactosylceramide by beta-galactosylceramidase (EC 3.
lay play a role in intracellular signaling and gene expression or in linking chromatin to the nucle
ss I binding peptides.

phosphatase, dephosphorylating tyrosine-, serine- and threonine-phosphorylated proteins. Als

be fusion during muscle regeneration (By similarity).
that plays an essential role in regulating cell migration, adhesion, spreading, reorganization of
. Acts as a negative regulator of the oncogenic property of YAP, a downstream target of the hi

15464, PubMed:22605381, PubMed:22825852). For most substrates, PLA1 activity is much hig
apse. Key determinant of DSB repair pathway choice, as it commits cells to HR by preventing c

ls. Also required for ciliated ependymal cell differentiation. Regulates the expression of genes i

nvolved in microtubule organization and regulation of transport in primary cilia.
eration, survival, and differentiation by modulating mTOR signaling and repressing pro-apoptoti

:10391915, PubMed:10574970, PubMed:30867591, PubMed:10903140). The heterodimer with
contraction coupling in muscle. In a first phase, voltage-gated channels mediate the rapid incre

ATP-dependent manner. May stimulate the ATPase activity of the catalytic subunit of the comp

eins (By similarity).
K1) and to tyrosine phosphorylation of STAT1 and STAT2. The phosphorylated STATs dimeriz
oepiandrosterone (DHEA), pregnenolone, (24S)-hydroxycholesterol and xenobiotic compounds
ing as a polo kinase kinase: mediates phosphorylation of PLK1 in vitro; however such data req
ukin 1 receptor type II, p55 TNF-receptor, transforming growth factor-alpha, L-selectin, growth
uitment of the 19S proteasome complex, leading to the subsequent proteasomal degradation c

es of growth factors and tumor necrosis factor-alpha by regulating the intracellular concentratio
l of cell-cycle progression from G1 to S phase. The E2F1:DP complex appears to mediate both
thora of physiological and pathological processes including cell cycle arrest in epithelial and he
space and as a boundary between the compositionally distinct apical and basolateral plasma m
ment, activation, growth and motility. This encoded protein is a cell surface glycoprotein that is
ks TNF-alpha-induced apoptosis, which suggests that it regulates TNF-alpha function by antag

is an inhibitor of cyclin-dependent kinases. Apoptosis induction seems to be mediated either by

the cytosolic 5S rRNA. Only the nascent unfolded cytoplasmic form is able to bind to the 5S rR

dine, 5-bromouridine, N(4)-acetylcytidine, N(4)-benzoylcytidine, 5-fluorocytidine, 2-thiocytidine,

similarity). Plays an important role in the intracellular motility of vaccinia virus by functioning as
ling; the initial CG dinucleotide can be methylated greatly reducing the binding affinity. The effe

lay a role in signal transduction that leads to the production of interferons in response to viral ir

norylation and microtubule function in neurons. LRP8 has higher affinity for Reelin than VLDLR

oly via apoptotic signaling pathway involving CASP8 and CASP3 activation, and poly(ADP-ribos

ll membrane, and thereby increases channel activity (By similarity). Promotes potassium chann
s (EBOV), Dengue virus (DNV), West Nile virus (WNV), human immunodeficiency virus type 1

phorylation and palmitoylation of the G-protein. Negatively regulates mu-opioid receptor-mediat
304). Has no apparent extracellular phosphatase activity and therefore most probably acts intra
ply of catabolic uridine and the generation of purine-2',3'-cyclophosphate-terminated oligoribon

nd progestins with their cognate inactive metabolites. Preferentially transforms androstenedion
genesis. Also involved postnatally in the subventricular zone (SVZ) neurogenesis by regulating
Wnt signaling pathway by mediating poly-ADP-ribosylation (PARsylation) of AXIN1 and AXIN2,

lity on or towards fibronectin possibly by driving alpha-v/beta-1 integrin (ITAGV-ITGB1) cell sur

protein ligase complex which mediates the ubiquitination and subsequent proteasomal degrad

esistance to cell stress, such as starvation or gemcitabine treatment.
gnaling cascade, promotes the formation of spines and synapses in hippocampal neurons.

hoadenylylsulfate (PAPS: activated sulfate donor used by sulfotransferase). In mammals, PAP

r degree, however tyrosine kinase activity is unclear and may be indirect. May be a downstrear

finity pyruvate transporter.

and hematopoietic stem cells. Receptor for collagen III/COL3A1 in the developing brain and in
the CNS including neuronal maturation and axonal and myelin formation (By similarity).
ciated with TGF-beta receptor internalization.

by direct interactions with regulatory proteins and serves as a scaffold for the assembly of a fu
ys a regulatory role in the processing of the amyloid-beta A4 precursor protein (APP) and acts

r cell survival by mediating the influx of HCO3(-) into the cells. Also mediates lithium-depender
ursors. Digests the poly-Q peptides found in many cellular proteins. Digests tau from normal b
n intraluminal vesicles (ILVs) that are generated by invagination and scission from the limiting
L6. Recruited by ZBTB7A to the androgen response elements/ARE on target genes, negativel
cing H(2)O(2) (By similarity). Acts as a key regulator of the SRF signaling pathway elicited by n

p1 by direct association with Rap1-GTP thereby leading to the amplification of Rap1-mediated
ed in collagen folding. Required for proper insertion of the first transmembrane helix N-termin
iption factors to DNA. During cortical development, controls the neural stem cell pool by inhibiti

g S.typhimurium infection and subsequent xenophagy (By similarity). Involved in repair of DN

ase and mediates the ubiquitination of AURKB. Ubiquitination of AURKB by BCR(KLHL21) E3

aling through MAP kinases.
ation of large multiprotein complexes. Involved in muscle maturation by repressing transcriptio
stimulatory effect on the ATPase activity of HSP70 in a dose-dependent and time-dependent m

ıbMed:12618084). Can utilize both NADH and NADPH.

utophosphorylation on several serine and/or threonine residues. Phosphorylates and inactivate
methylation of histone H3 lysine 4 (By similarity).

ıl for synaptic vesicle maturation in a subset of excitatory/glutamatergic but not inhibitory/GABA
ntegrins and the RGD motif. Vascular ligand for integrin receptors which may play a role in vas

s (EBOV), Dengue virus (DNV), West Nile virus (WNV), human immunodeficiency virus type 1
d in translationally coupled mRNA turnover. Implicated with other RNA-binding proteins in the c
nds RNA.
in-binding domain are inactive. Efficiently phosphorylates 5'-AMP-activated protein kinase (AM
:20381137, PubMed:24095279, PubMed:25936802). Forms heterodimeric Rag complexes wit
s RGS9 GTPase-activating protein (GAP) activity, hence contributes to the deactivation of G p
timulated proliferation of neural progenitor cells (By similarity).

m second messenger system.
ɔounds. Active on odorants and seems to be involved in olfaction; it could help clear lipophilic c
ıte.
Possible targets include TRPV4.
ɔrdination of each mitotic event. This cyclin shows the highest similarity with cyclin G. The trans

ɜ part in heparan sulfate synthesis by supplying UDP-Glc-NAc, the donor substrate, and thus b

R45 is involved in autophagosome assembly downstream of WIPI2, regulating the size of formi
ne cilium and thus triggers its entry into the cilia (PubMed:28625565, PubMed:28428259).

ary N-linked sugar chains. Has lower affinities for donors or acceptors than MGAT4A, suggesti
gulating the mature length of the middle and short rows of stereocilia (By similarity).

C99, a C-terminal APP fragment, abolishes C99 interaction with PSEN1 and thus APP C99 cle

and efficient translesion DNA synthesis (PubMed:24657165). Plays a key role in the transcripti

d brain development. May participate in neurodevelopmental processes such as proliferation, r
p of metabolic inactivation of leukotriene B4.
ı glucocorticoid receptor-mediated repression and induction and in androgen receptor-mediatec
3 which can also be a component of the Mediator complex. Alternative splicing results in multip

nd mRNA (By similarity). Regulates formation of membrane ruffles and lamellipodia. Plays a ro
oteasome by opening the gated channel for substrate entry. Binds to the core proteasome via

ction with PICK1 (By similarity). May also play a role in neuron differentiation through inhibition

ron-gamma activation and subsequent phosphorylation dissociates from the ribosome and asse
for normal organization of the cytoskeleton, including the actin cytoskeleton, microtubules and
r to a promoter of RET degradation (By similarity). May play a role in receptor clustering and cy
itive regulator of telomerase. Has negative and positive effects on RNA polymerase II-depende
CDC42BPB targeting to LIMK1 and the lamellipodium results in LIMK1 activation and the subs

tant in myotube formation (PubMed:22428023). Plays a role in lysosomal homeostasis (PubMe

ıem to specific DNA-binding sites. Interacts with the upstream promoter region of the oxytocin ı

substrates but rather calmodulin-binding motifs and is thereby antagonized by calmodulin. This
eme (PubMed:24360805).

stone demethylases RIOX1 or KDM2B, leading to demethylation of H3K36 and recruitment of

se pathway that regulates Golgi morphogenesis and dendrite patterning in brain (PubMed:2157

Med:17234884, PubMed:17875940, PubMed:23708001, PubMed:23708605, PubMed:1692102

nhibits BMP2-mediated differentiation of osteoblasts (in vitro) (PubMed:27036124). Acts as inh

A with the sequence 5'-CGATACAA-3' (or closely related sequences) (PubMed:21924763). Pro

quired for proper regulation of SHH signaling (PubMed:27666822).

nce of cell polarity, and cell migration.
ity and palmitoylates NCDN thereby regulating its association with endosome membranes.
a complex that mediates N6-methyladenosine (m6A) methylation of RNAs, a modification that

te. Preferentially sulfates in GlcA->GalNAc unit than in IdoA->GalNAc unit. Does not form 4, 6-

3/TRAF6 signalosome. This function probably serves as a brake to prevent excessive RLR sig

nal output of signaling pathways which regulate cell proliferation (PubMed:15251430, PubMed:

2, PubMed:25849773, PubMed:27462815). The eIF-3 complex associates with the 40S riboson
ohydrate and lipid biosynthesis, as well as cell growth and proliferation. AMPK acts via direct p

ays a role in cholesterol homeostasis. Influences plasma cholesterol levels through regulation o
nscription factor. Binds to the oxygen responsive element of COX4I2 and represses its transcri

ion of STIM to efficiently turn off ORAI when the endoplasmic reticulum lumen is filled with the
ethyltransferase SETDB1. The NLK-SETDB1 complex subsequently interacts with PPARG, lea

sion events and thereby promotes noncrossover repair by meiotic synthesis dependent strand
dues long. Rapidly degrades 13-mer to a 9-mer and then stops. Preferentially hydrolyzes the r
tains protein homeostasis and promotes cell survival through attenuation of endoplasmic reticu
Trex site and a 5'-TCA[AG][AG]TTNC-3' motif present in the MEF3 site of the muscle-specific
rylation in the nucleus is required for U snRNA export complex assembly and export, while its
CLSPN.
Med:10748143). Also able to transfer galactose to galactosylceramide/beta-D-Gal-(1<->1')-Cer
e proliferation and differentiation. Plays a role in modulating the response to steroid hormones

umenogenesis (By similarity).

May play a role in adipocyte differentiation.

RPE, SNRPF and SNRPG that assemble in a heptameric protein ring on the Sm site of the sm
oteins in lysosomes. The primary defect in NCL disorders is thought to be associated with lysos

wer activity with COL1A1 (PubMed:18487197). Catalyzes hydroxylation of the first Pro in Gly-P
s in presence of Mn(2+) (PubMed:29335528, PubMed:31127291). Acts as an exoribonuclease
ging. Does not charge cell cycle E2s, such as CDC34. Essential for embryonic development. R

IV1 GAG-POL expression by inhibiting -1PRF (PubMed:30682371). Exhibits antiviral activity a
esides, phosphorylation of phosphatidylinositol (PI) to phosphatidylinositol 4-phosphate (PI4P)

es. [provided by RefSeq, Jul 2008]
ner or respective retromer complex variants acting to prevent missorting of selected transmem
licated in vesicle formation and in uptake of lipid signaling molecules. The beta subunit may as

e (GalNAc) residue of chondroitin. Also acts on core 2 mucin-type oligosaccharide and N-acety
t along actin fibers, providing a novel link between actin cytoskeleton dynamics and intracellula

osition using C18:1, C20:4 or C18:2-CoA as the acyl donor (PubMed:21173190). Also acts on

r. It is derived from the Latin roots 'circa' (about) and 'diem' (day) and acts as an important regu
retromer-independent retrieval and recycling of numerous cargos such as integrin alpha-5/beta
n the limiting membrane of the endosome and mostly are delivered to lysosomes enabling degr

d:12496252, PubMed:17675297). Mediates 'Lys-48'-linked polyubiquitination of RIPK3 leading

r ABCA3 (By similarity). By regulating both the proper expression and the degradation through
ore (PubMed:14738735, PubMed:14699129). The NDC80 complex synergistically enhances the
of FGFR1 that has been activated by FGF1 binding; this occurs most likely via the AP-2 comp

nous organophosphorus compounds. May contribute to cancer pathogenesis by promoting tum

onsensus kaiso binding site (KBS). Can also bind specifically to a single methyl-CpG pair and
t is necessary for the translocation of CpG dinucleotides from early endosomes to late endoso
lved in regulation of the microtubule network, causing reorganization of microtubules into bund

red for hindbrain axon midline crossing (PubMed:15105459).
re it is cleaved in a site-specific manner by resident proteases S1P/MBTPS1 and S2P/MBTPS

her lipids, such as various sphingosines (PubMed:11956206, PubMed:16269826, PubMed:19
(PubMed:29911972). Catalytic activity of the complex is required for normal cell proliferation (
involved in deubiquitination of monoubiquitinated FANCD2 (PubMed:19075014).

an unmodified N-terminal Pro followed by a small residue, and has the highest affinity for the

imb of a novel autoregulatory feedback loop (DEC loop) which differs from the one formed by t

ria. In collaboration with PRKN, isoform 2 may enhance cell viability and protect cells from oxid

ation through cleavage and ligation cycles.
of classical E2F transcription factors such as E2F1: component of a feedback loop in S phase

bMed:15907797, PubMed:22745828). Gives rise to a mixture of 16:1 and 18:1 unsaturated fat

meostasis by mediating mitochondrial calcium extrusion: calcium efflux is essential for mitocho
cement of anchorage-independent growth, tumorigenic conversion, promotion of genomic insta

iated PLCB2 plasma membrane translocation and subsequent activation in leukocytes (PubMe

manner. May be an important mediator of Rac signaling, acting directly downstream of both G
KCND3/Kv4.3 currents (PubMed:23576435). Isoform 4 does not increase KCND2 expression

angiogenesis by binding to the heparan sulfate proteoglycans involved in growth factor signalin

ty). May contribute to the osmotic regulation of cellular glycerophosphocholine (By similarity).
g the degradation of PDCD1/PD-1; the PDCD1-mediated inhibitory pathway being exploited by

wn-regulates NQO1, leading to increased mitomycin c resistance.
and regulating transcellular versus paracellular flow in different organs. Plays a specific role in e

gions. Also acts as O-mannosyl-transferase on other proteins such as PDIA3.
Cer; paragloboside), resulting in the synthesis of Lc(3)Cer and neolactopentaosylceramide (nLc

19951071). Acts as a negative regulator of macrophage-mediated inflammation by inhibiting ma

ORC1 signaling pathway.

x to the repair site and by promoting the complex nuclease activity (PubMed:26215093). Proba

e in the synthesis of complex glycosphingolipids. Binding of both phosphatidylinositol 4-phosph
phate (IP3). DAG mediates the activation of protein kinase C (PKC), while IP3 releases Ca(2+)

anonical Wnt signaling pathway to increase expression of target genes. Also regulates the canc
gions. Also acts as O-mannosyl-transferase on other proteins such as PDIA3.
eration by mediating 'Lys-48'-linked polyubiquitination and subsequent degradation of AKT1 in a
t the base of the cilium and thus triggers its entry into the cilia (PubMed:28625565, PubMed:28

3161870).

s rearrangement of the actin cytoskeleton, but does not alter microtubule alignement.

s activation of an innate immune response by inducing the oligomerization and polyubiquitinatio

nt. May participate in cytokinesis by hydrolyzing PIP2 at the cleavage furrow.

biphosphate) like the plasma membrane. Its membrane-bending activity might be important for
q, Dec 2015]

ectodomains of glycan-modifiying glycosidase and glycosyltransferase enzymes such as MGAT
TRs, but encoding the same protein, have been identified. [provided by RefSeq, Jul 2008]

lactokinase), GALT (galactose-1-phosphate uridylyltransferase) and GALE (UDP-galactose-4'-e

variants have been described. [provided by RefSeq, Jan 2009]
ss response such as DNA damage. In cytoplasm, acts as a nucleation-promoting factor for both

IF recruitment through its TIR domain (PubMed:12471095, PubMed:12539043, PubMed:1473

the regulation of mitochondrial respiration and mitochondrial DNA copy number maintenance (

rity). Attenuates actin stress fiber formation via inhibition of TESK1-mediated phosphorylation c

pathway.
reverse transcription, it can induce the conversion of cytosine to uracil in the minus-sense sing
sition in lens epithelial cells (By similarity).
receptor surface expression. In addition, the protein has been identified as a component of the

ucose levels.

genes (By similarity).

ent mechanism. However, its overexpression also produces a decrease of the ubiquitin-depend
the cleavage furrow at the end of mitosis. Modulates endocytosis of cell-surface proteins, such
phosphate. Also synthesizes ribose 1,5-bisphosphate. Has only low phosphopentomutase and
d with autosomal recessive deafness. [provided by RefSeq, Dec 2012]

osome that recruits CDK2 required for centriole duplication (PubMed:26297806).

lglycine (fGly), occurs in the maturation of arylsulfatases and some alkaline phosphatases that

sible for vesicle formation, movement, tethering and fusion. The low intrinsic GTPase activity c

ates the alpha subunit of eukaryotic translation initiation factor 2 (EIF2S1/eIF-2-alpha) in respo

lilize new endothelial connections during angiogenesis (PubMed:19154719). During somitogen

are involved in elongation of the phagophore membrane, the GABARAP/GATE-16 subfamily i

ption elongation (PubMed:28847961). Preferentially recognizes and cleaves monomethylated a
can be either protein-coding or non-coding. The primary transcript is cleaved by the Drosha ribo

m gene product, but they contain distinct C-termini due to frameshifts versus the downstream g

on and neuronal survival, hair follicle development and cycling, FSH production by the pituitary

with CD6, as well as homotypic cell-cell contacts (PubMed:7760007, PubMed:15496415, Pub

nd either glyceraldehyde-3-phosphate or glyceraldehyde, respectively. [provided by RefSeq, J
s) which inhibit protein synthesis and triggers the assembly of stress granules (SGs). Angiogen
notor protein KIF17 to transport vesicles containing N-methyl-D-aspartate (NMDA) receptor sub

ic pH shifts the activation to more negative voltages (By similarity).

quired for its tumor suppressor function. The BRCA1-BARD1 heterodimer coordinates a divers
from ssDNA and stabilizing RAD51-ssDNA filaments by blocking ATP hydrolysis. Part of a PAI

duction pathways. In naive T-cells, inhibits VAV1 activation upon TCR engagement and impos
rentially interacts with and activates CDK1 and CDK2 throughout the cell cycle.
nsporter.
enzyme for the phosphorylation of numerous nucleoside analogs widely employed as antiviral a

ane. Acts as a chaperone-like protein that protects the hydrophobic precursors from aggregatic

Rac by activating the Rac-specific guanine nucleotide exchange factor (GEF) activity. Acts as a

in plasma and may be a modulator/inhibitor of GH signaling.

lternative splicing results in multiple transcript variants encoding different isoforms. [provided b

romosomal stability. DNA accessibility is regulated via a complex set of post-translational modi
sozymes catalyzing that reaction (By similarity).

on-canonical DNA structures such as single-stranded DNA. Can bent DNA and enhance DNA

vity. Major fetal growth hormone in mammals. Plays a key role in regulating fetoplacental deve
to the insulin II gene RIPE3B enhancer region. May be involved in translation (By similarity). DN
insulin secretion, nerve cell survival, embryonic axial development or bone growth, depending
lated genes (ISG) by binding to an interferon-stimulated response element (ISRE) in their prom
lters its pharmacological properties. It slows down the activation and the deactivation kinetics c

critical structure of a conserved arginine finger motif is provided in trans relative to the ATP-bir
critical structure of a conserved arginine finger motif is provided in trans relative to the ATP-bir
critical structure of a conserved arginine finger motif is provided in trans relative to the ATP-bir

owever, upon interaction with CSPG4, it may be involved in degradation and invasion of type I
thiocarboxylated (-COSH) at its C-terminus by MOCS3. After interaction with MOCS2B, the s

rowth factor (VEGF)-induced angiogenesis in coronary vessels and promotes blood clotting thr
nodes of Ranvier on myelinated axons. Nodes of Ranvier contain clustered sodium channels t

preventing premature centrosome splitting during interphase by inhibiting NEK2 kinase activit

ylceramides and sphingomyelins (PubMed:8898203). Energy-dependent efflux pump responsi

maintenance of GABA-mediated synaptic inhibition. Its aberrant expression could contribute to

elimination of RNA processing by-products and non-coding 'pervasive' transcripts, such as antis
on mRNAs. The EJC is a dynamic structure consisting of core proteins and several peripheral r

on machinery. It is composed of mobile elements that move relative to each other. RPB7 is par
the ATP-dependent degradation of ubiquitinated proteins. The 26S proteasome plays a key ro
form on DNA for several proteins involved in long-patch base excision repair (LP-BER) (PubMe

ılays an essential role both in DNA replication and the cellular response to DNA damage (PubM
of ribosomal proteins. The protein, which contains a C2-C2 zinc finger-like domain that can bi

ɔnizing the effects of splicing regulators, like RBMX. Binds to the AG-rich SE2 domain in the SM
renal Na(+) retention, renal K(+) elimination, salt appetite, gastric acid secretion, intestinal Na(
edgehog pathway, acting downstream of PTCH1 (PubMed:16024801, PubMed:9372240). Play
p27kip and is involved in regulation of G1/S transition. Degradation of CDKN1B/p27kip also rec
orts one amino acid molecule together with two or three Na(+) ions and one proton, in parallel
strate specificity, with a preference for zwitterionic amino acids. It accepts as substrates all neu
8). Mechanistically, acts by increasing local Ca(2+) release from microdomains which is essent

628, PubMed:23333303, PubMed:25555158, PubMed:26912367, PubMed:28502770, PubMec
velope and involved in accurate posterior sperm head localization of the complex. May anchor
activation rate of the calcium channel CACNA1C.

STX1A and SNAP25 are localized on the plasma membrane while VAMP2 resides in synaptic
ylated histones. Mainly functions in heterochromatin regions, thereby playing a central role in th

E gene. Represses expression of the T-cell receptor gamma gene in alpha-beta T-cell lineages

ɔtion factor 6 alpha (ATF6) which produces adaptive ER stress response factors (By similarity).
nvolved in LPS-independent inflammatory responses triggered by free fatty acids, such as palm
ceptor for attachment of lamin filaments to the inner nuclear membrane. May be involved in th
osine of the enzyme, resulting in the formation of a DNA-(5'-phosphotyrosyl)-enzyme intermedi
e beta receptor only. Isoform 5 also mediates cell death induced by a combination of interferon

eraction with WDR48 to have a high activity (PubMed:18082604, PubMed:26388029).
le a mechanism for attenuating protein synthesis (PubMed:9703499, PubMed:10330172, PubM

esis (PubMed:19571038, PubMed:25203508). Within the heterotetramer, HSD17B8 binds NAD

ıce for histone H3 diacetylated at 'Lys-18' and 'Lys-27' (H3K18ac and H3K27ac) or histone H3
4970, PubMed:11311135, PubMed:11564694, PubMed:12117417, PubMed:12225859, PubMe
hromatin precursor subsequent to DNA replication to complete the histone octamer.
I via a complex set of post-translational modifications of histones, also called histone code, and

ıte/C1P (PubMed:9305923, PubMed:9705349, PubMed:9607309, PubMed:10962286, PubMed

I cytokine production, either independently of SLC12A3, or as a complex with SLC12A3 (By sir

Required to stabilize replication forks during DNA replication by forming a complex with TIPIN:

ɟ protein in the nucleus.
ions (By similarity). May play a role in protecting FBXW7 isoform 3 from degradation (PubMed:
ıg to HIF1A hydroxylation and subsequent proteasomal degradation (PubMed:28296633).

e assembly proteins NAP1L1 and NAP1L4. Also acts as a monoglutamylase: generates monog
ɔosed to depend on its association in the mitotic RZZ complex.
ɛsis. May play a role in the assembly of the mother centriole subdistal appendages (SDA) there

ɔn of 'Ser-464' of LATS1, thereby controlling its stability. Controls cell adhesion by regulating ac
mitomycin C (MMC). Together with the TTT complex and HSP90 may participate in the proper

excision repair (PubMed:10230407, PubMed:10579999, PubMed:15983046, PubMed:166781
385). Histone deacetylases act via the formation of large multiprotein complexes (PubMed:102
DNA; histones H2A/H2B can bind to this chromatin precursor subsequent to DNA replication to

y LXRES (By similarity). LXRES are DR4-type response elements characterized by direct repea
ɔX2).

;) into phosphatidylserine (1,2-diacyl-sn-glycero-3-phospho-L-serine or PS) (LPSAT activity). H
notes also activation of TBK1 and IRF3. Involved in the ubiquitination of erythropoietin (EPO) a

' 'Lys-11'-linked polyubiquitin chains and, to a lower extent, the formation of 'Lys-48'- and 'Lys-6
te uridine at this position (PubMed:20047967). Has also an essential role in 40S ribosomal sub
inetochore fibers of the mitotic spindle by acting as inter-microtubule bridge. The TACC3/ch-T

:17664331, PubMed:27462074). In complex with SKAP, promotes stable microtubule-kinetoch
ng. Regulates target genes through H3K9ac deposition and MYC recruitment which trigger MY

ome replication, including in lagging strand synthesis, and repair. Required for optimal Pol-delta
able to induce centrosome amplification through the simultaneous generation of multiple procer
al deafness, non-specific X-linked cognitive disability, oral-facial-digital syndrome, and Fried sy

nent (PubMed:16387653, PubMed:21753189, PubMed:24065767, PubMed:26620551, PubMe

tivities (AMPylase or de-AMPylase) will take place (By similarity). Acts as a key regulator of the
d:10523320). Component of LSm protein complexes, which are involved in RNA processing an
ating 5'-hydroxyl groups on, the ribose sugar of the DNA backbone.

elimination of RNA processing by-products and non-coding 'pervasive' transcripts, such as antis

s such as antero-posterior axial patterning, limb development, somitogenesis and eye formatio

f double-strand breaks (DSBs). Its localization to DNA damage foci requires RNF8 and UBE2N

eration, and facilitates apoptosis in response to exogenous stimulation.
icleocytoplasmic interactions established by the LINC complex play an important role in the tra

in the presence of type II topoisomerases (PubMed:11136719). Early in neurogenesis, may pl
elimination of RNA processing by-products and non-coding 'pervasive' transcripts, such as antis
n D, progesterone receptor, nor glucocorticoid. Acts as a coactivator for estrogen receptor alph

RefSeq, Sep 2016]
d:10523320).

s formed by IL17A and IL17F as part of a heterodimeric complex with IL17RC (PubMed:18684
h other, and this determines the overall permeability. CLDN15 forms tight junctions that media

uired for the formation of senescence-associated heterochromatin foci (SAHF) and efficient se

lf, but promotes the turnover of CDC20 and MCC on the APC/C, thereby participating in the re

n hypoxia-induced cell death of neuronal cells probably by promoting release of AIFM1 from mi
er membrane. Acts as a chaperone-like protein that protects the hydrophobic precursors from a

uent degradation.

vation to form active metabolites. Sulfates dopamine, small phenols such as 1-naphthol and p-
nder investigation for its potential diagnostic and therapeutic uses (PubMed:23922215, PubMec

ratio is reversed. Another difference between mammalian mitoribosomes and prokaryotic ribos
nal transducer and activator of transcription 1-alpha) pathway. [provided by RefSeq, Aug 2013

ratio is reversed. Another difference between mammalian mitoribosomes and prokaryotic ribos
ed in the estrogen-induced cell proliferation and cell cycle progression of breast cancer cells.

x) complex. Probably shuttles between the presequence translocase and respiratory-chain ass

(By similarity). Methylates ETF1 on 'Gln-185'; ETF1 needs to be complexed to ERF3 in its GT
Condensin-mediated compaction likely increases tension in catenated sister chromatids, provi
core to the nascent glycoproteins. Multiple transcript variants encoding different isoforms have
tabilizes presynaptic RAD51 and DMC1 filaments formed on single strand DNA to capture doul

ke PTPN6/SHP-1 and PTPN11/SHP-2 via their SH2 domains that block signal transduction thr

drial ribosome assembly by regulating the biogenesis of the 39S ribosomal subunit (By similari
onylcarbamoyl-AMP (TC-AMP) to the N6 group of A37 (PubMed:22912744, PubMed:2880582
by direct interactions with regulatory proteins and serves as a scaffold for the assembly of a fu

ratio is reversed. Another difference between mammalian mitoribosomes and prokaryotic ribos

chromosome 5.[provided by RefSeq, Jan 2009]

cifically with newly synthesized MT-CO2/COX and its copper center-forming metallochaperone

s and/or in the assembly of myofibrils into sarcomeres. Implicated in regulation of actin polyme

in RNA-mediated gene silencing (RNAi) by miRNAs. Binds capped RNAs (m7GpppG-capped

ιic transport of SHH signaling molecules within the cilium. Not required for ciliary assembly. Its ı
s. Acts as nuclear and cytosolic DNA sensor involved in innate immune response. Can sense ı

.||Plays a role in apoptosis. Suppression of the anti-apoptotic members or activation of the pro-

ıe placenta.

ɜ KCNH2 potassium channel activity by decreasing its cell-surface expression and interfering w
proteins between the cilia and plasma membranes. Involved in neuronal differentiation. As a p

ɜion until it is resolved during anaphase (PubMed:17218258, PubMed:23973328). Functions as

role in myogenic differentiation through activation of the stress-activated MAPK cascade.

TIMELESS and this complex regulates DNA replication processes under both normal and stres

d the release of microtubules from the centrosome following nucleation. Microtubule release fro

; complex (PubMed:16622420). Plays an important role in chromosome congression and in the
native splicing results in multiple transcript variants. [provided by RefSeq, Jun 2017]
nds branched DNA: binds both single-stranded DNA (ssDNA) and double-stranded DNA (dsDN

ed by RefSeq, Sep 2008]

ɔase.
ɾed for localization of KIZ, AKAP9 and gamma-tubulin ring complexes (gamma-TuRCs) (PubMe
lization at the photoreceptor-specific distal CC is essential for normal microtubule stability and
that stabilizes centriolar microtubules and inhibits microtubule polymerization and extension fro

proteins in neutrophils.
ticulum Ca(2+) concentrations. Upon mild variations of the endoplasmic reticulum Ca(2+) conc
omplexes, preventing UV-induced degradation of ERCC6 by the proteasome. Interacts with the

d:10523320).

d APBA3.

ally to branched DNA structures and may associate with S-phase chromatin following formation
lynamic (By similarity).
complex. Acts in coordination with KNL1 to recruit the NDC80 complex to the outer kinetocho
in the presence of type II topoisomerases.

stems. Essential for the inhibitory function of regulatory T-cells (Treg). Mediates FOXP3-mediat
gate plays a role of an E3 and promotes the transfer of ATG8-like proteins from ATG3 to phosp

ependent. Further studies suggest that the protein is associated with ribosome biogenesis throu

lication forks, and anchors RBBP8/CtIP to DSB sites to promote DNA end resection and ensui
thesized CENPA into centromeres.

acids with 12, 14 and 16 carbons with higher activity toward C16:0 acyl-CoAs. Catalyzes the sy

ard 5-beta-androstanes, 5-beta-pregnanes, 4-pregnenes and bile acids. May also reduce endog
C complex. Modulates the kinetochore-bound levels of NDC80 complex.

mRNA upstream of the exon-junction complex (EJC) and is recruited in a splicing- and cap-de

e the PAGR1:PAXIP1 complex is required for cell survival in response to DNA damage indepen

n kidney.

nthesized CENPA into centromeres. Plays an important role in the correct PLK1 localization to t

omatin regions, thereby playing a central role in the establishment of constitutive heterochroma

RPE, SNRPF and SNRPG that assemble in a heptameric protein ring on the Sm site of the sm
RPE, SNRPF and SNRPG that assemble in a heptameric protein ring on the Sm site of the sm

y. [provided by RefSeq, Jul 2008]
ng DNA replication initiation, regulating DNA replication initiation (PubMed:20129055, PubMed:
horylation during mitosis. Within the BLM complex, required for BLM and TOP3A stability.

H2AZ1-H2B dimer, thus shifting the equilibrium towards dissociation and the off-chromatin stat
K to make heme (By similarity).

ar calcium homeostasis (PubMed:30082715, PubMed:31191616). Does not show transport act

necting cytosolic iron-sulfur protein maturation with cellular iron regulation (PubMed:23891004)
mine-beta-1,4-(phosphate-6-)mannose). Phosphorylated O-mannosyl trisaccharide is a carboh

d breaks (DSBs) sites on chromatin during DNA repair in response to stalled replication forks (F

ulate skeletal muscle differentiation and insulin secretion by pancreatic beta-cells. Involved in cy

he SLX1-SLX4 structure-specific endonuclease that resolves DNA secondary structures genera
that acts as a platform to connect RNA polymerase I with enzymes responsible for ribosomal p

position of the glycerol backbone (PubMed:19318427).

ation of the initiating cartwheel structure (PubMed:27185865). Required for the recruitment of c

dinates faithful mitotic progression and completion of cytokinesis.
tion results in splicing defects and death in cancer cell lines (PubMed:28437394, PubMed:2830
by direct interactions with regulatory proteins and serves as a scaffold for the assembly of a fur
ıbrane.

cific semaphorins, and its cytoplasmic domain is required for the activation of down-stream sigı

ease spastic ataxia-3 (SPAX3). [provided by RefSeq, Apr 2014]

\ repair. Required for efficient DNA double-stranded break repair.
.2 and nitric oxide (By similarity). May play a role in inhibition of proliferation and migration (Pul

otides into or from the mitochondria.

.CPUFA synthesis (PubMed:30509349).

en, alpha-ketoglutarate and iron.
of target proteins.

e NPC.

d proteasomal degradation of proteins for which signal-mediated translocation to the endoplasr

een described. [provided by RefSeq, Sep 2010]
ation forks.

35). The NDC80 complex synergistically enhances the affinity of the SKA1 complex for microtu

ell adhesion and angiogenesis. It functions in filopodia formation, cell migration and tube forma
ubfamily of piggyBac transposable element derived (PGBD) genes. The PGBD proteins appea

riolar markers. Required for the recruitment of STIL to the procentriole and for STIL-mediated c

s as a key regulator of acinar cell function, stability, and identity. Also required for normal orgar

ved in actin-dependent nuclear movement via association with transmembrane actin-associate
st of the Hedgehog pathway by affecting the nuclear transfer of transcription factor GLI1; the fu

nicrotubules leading to silencing of the spindle checkpoint (PubMed:17093495). The SKA1 com
icrotubules as oligomeric assemblies (PubMed:19289083, PubMed:19360002). The complex fa

3y similarity).

:y (By similarity). Ceramides generated by CERS6 play a role in inflammatory response (By sim

sma membrane. In polarized epithelial cells, involved in efficient trafficking of TGFA to the cell s
nulticiliated epithelial cells. Seems to recruit INT to basal bodies of motile cilia which subsequer
reticulum, where it serves as a substrate for N- and O-glycosylations by fucosyltransferases. F

ini. There are no conserved sequences at the splice sites, but the intron is invariably located at

d:21620453). Following activation by NEK2 involved in disassembly of primary cilium during G2

A repair, and their resolution is necessary for proper chromosome segregation (PubMed:1902[illegible]

cking of EGF (PubMed:20175130). Involved in transferrin receptor recycling. Regulates the tra[illegible]

[illegible] complex.

[illegible]ylwyosine, an intermediate in wybutosine biosynthesis (By similarity).
[illegible] it is not involved in BIK-dependent activation of caspases. Involved in life span control, probal[illegible]

[illegible]an be either protein-coding or non-coding. The primary transcript is cleaved by the Drosha ribo[illegible]

:an be either protein-coding or non-coding. The primary transcript is cleaved by the Drosha ribc
ats and the complex is subsequently translocated through the pore by an energy requiring, Ran

ıation of WNK4, an inhibitor of potassium channel KCNJ1, leading to WNK4 degradation (PubM
and differentiation of hematopoietic cells through mechanisms other than regulating Rac GTPa
;).
axis and extension along the anteroposterior axis. Necessary for nuclear localization of REST.

protein complex, comprising also RNF11, ITCH and TAX1BP1, that ensures the transient natur
sociation with type I receptors sharing the common subunit gamma such as IL2R, IL4R, IL7R,

ıbMed:16732327). Required for kinetochore integrity and the organization of stable microtubule
idbody and for successful cytokinesis (By similarity). May play a role in mitotic chromosomal pc
e in providing serine molecules for the formation of myelin glycosphingolipids in oligodendrocyt
AURKA by promoting its autophosphorylation at 'Thr-288' and protects this residue against dep

5-thio-alpha-D-ribose 1-phosphate (PubMed:31978345). Also has adenosine deaminase activi
terleukin-2 receptor, and interferon-beta genes. It may act in T-cell activation. Involved in activa

l via a complex set of post-translational modifications of histones, also called histone code, anc
entral spindle and midbody through its interaction with PRC1 and CIT respectively (PubMed:16
APEX2 activities. Has to be loaded onto DNA in order to be able to stimulate APEX2. Plays a l
2010]

:an be either protein-coding or non-coding. The primary transcript is cleaved by the Drosha ribc

during mitosis and cytokinesis (PubMed:16322459). Acts as an important regulator of the local

nilarity). Plays a role in the formation and stabilization of endoplasmic reticulum tubules (PubM

a different level of activity.

termembrane space to provide PA for CL synthesis in the inner membrane (PubMed:2393175

the second half-reaction, PSQPP undergoes heterolysis, isomerization, and reduction with N

e inflammasome promotes its activation and CASP1-catalyzed IL1B and IL18 maturation and

to vesicles assembled at cell bodies for delivery into neurites and nerve terminals. The BLOC-

3IRC4 indirectly by activating the mitochondrial apoptosis pathway. After translocation to mitoc

1 phosphorylation of RACGAP1 to allow recruitment of ECT2 to the central spindle. Acts as a

ing PKC and calcium fluxes has been seen for some family members, but it is not yet clear if it

can be either protein-coding or non-coding. The primary transcript is cleaved by the Drosha ribo

and in nuclear movement and positioning. Probable anchoring protein which tethers the nucleu

e sterol core and concomitant release of formic acid (PubMed:20149798, PubMed:8619637).

first intron of the KRAS gene and inhibits its expression. ESXR1-C has the ability to inhibit cyc

modulated by fructose metabolites: GCKR with bound fructose 6-phosphate has increased aff

riants encoding different isoforms. [provided by RefSeq, Jul 2008]
rimarily as negative regulator, but can also function as activator, depending on the context. Re
ownstream signaling cascades via the G(i)/G(o), G(12)/G(13), and G(q) families of heteromeric
y). Weakly binds 25-hydroxycholesterol (PubMed:17428193).

the mobilization of SHTN1 and in the accumulation of PIP3 in the growth cone of primary hipp

ı1108).
lliday junctions.

ı. Antagonizes the PI3K-AKT/PKB signaling pathway by dephosphorylating phosphoinositides a
egulator may also play a role in protection against oxidative and genotoxic stresses (By similarit
:31171830). Acts as an angiogenesis inhibitor: inhibits migration, proliferation and network forn
ıg); or TUBA1 and TUBB (microtubule subunits). Binds directly F-actin and regulates actin cyto
NA 3'-end formation. Component of the tRNA splicing endonuclease complex: phosphorylates

; role on tumorigenesis, can also negatively regulate insulin secretion by inhibiting the activatior
ity for Ras and Rap. May be involved in certain forms of brain injury, leading to long-term learni
AP activity (By similarity).

ty permease involved in the transport of the cationic amino acids (arginine, lysine and ornithine
uration by controlling the conversion of early phagosomal compartments into late phagosomes

ıereby activating the TORC1 signaling pathway (PubMed:26449471, PubMed:26586190). This

onuclease III enzyme to produce an approximately 70-nt stem-loop precursor miRNA (pre-miRN

g splice variant subject to nonsense-mediated decay is the predominant splice variant expresse

's a pivotal role in the protein quality control system, ensuring the correct folding of proteins, the
onuclease III enzyme to produce an approximately 70-nt stem-loop precursor miRNA (pre-miRN

240, PubMed:17085480, PubMed:18703516, PubMed:18794347, PubMed:18794348, PubMed

's a pivotal role in the protein quality control system, ensuring the correct folding of proteins, the

adaptation to ER stress in liver, by maintaining translation of CPE-regulated mRNAs in conditi

This gene is highly expressed in colon cancers and in a human basal-like breast cancer cell line

apoptosis in chondrocytes, but can also promote cancer cell proliferation. Required for normal
nsity in cortical pyramidal neurons (By similarity). Involved in homologous recombination (HR) r
os, inhibits the kinase activity of the cyclin D-CDK4 complex. Inhibits DNA synthesis by DNA pc
ner as an inhibitor or an activator of cyclin type D-CDK4 complexes depending on its phosphory

onuclease III enzyme to produce an approximately 70-nt stem-loop precursor miRNA (pre-miRI

nner. Induces the formation of apical actin-dependent microvilli. Involved in the formation of a

721615, PubMed:3964259, PubMed:3732516). Can induce angiogenesis (PubMed:23469107,

ex to microtubules. Can block microtubule minus-end growth and reversely this function can be

dherens by interacting with ADAM10 and, in a PDZD11-dependent manner, with the zonula ad

Med:24726359).

310, PubMed:25786029, PubMed:27804176). Acts either as a transcriptional activator or repres

etochore (PubMed:18072184). The presence of CENPA subtly modifies the nucleosome struc

e spindle equator and by aiding the establishment and maintenance of connections between ki
gulating the activity of RB proteins during cell division and proliferation. May play a regulatory o

ses transcription (PubMed:20389281, PubMed:20562920). The N-terminal SNAG domain comp

2017]
ctive, but the enzyme does not need to possess an active kinase catalytic site for this to occur

the vascular response to injury (By similarity).

ɔnuclease III enzyme to produce an approximately 70-nt stem-loop precursor miRNA (pre-miRI

May also regulate IRS1 and IRS2 signaling in insulin-producing cells.
(By similarity). Represses transcriptional activation mediated by CTNNB1 in the Wnt signaling

required for entry into S phase and cytokinesis. Phosphorylates BCL2L1, leading to regulate th
entiated lymphatic endothelial cells. Plays an important role in postnatal angiogenesis, where i
inetochore (PubMed:15548592). The NDC80 complex synergistically enhances the affinity of th
ɔnuclease III enzyme to produce an approximately 70-nt stem-loop precursor miRNA (pre-miRI

the ALPL promoter.

. [provided by RefSeq, Dec 2008]
onizes phosphorylation of YAP1. Acts as a transcriptional corepressor for SNAI1 and SNAI2/SI
sion of target genes (PubMed:31527837).||Involved in global genome nucleotide excision repair
ne production/expression of TNF-alpha, oxidative stress, and endothelial dysfunction in type 2 c
ell adhesion to the extracellular matrix via integrin-dependent signaling, by mediating angiotensi

alpha chain and a beta chain bridged by disulfide bonds. [provided by RefSeq, Jan 2010]
oMed:24929359, PubMed:11923095). In contrast to proapoptotic SPHK2, has a negative effect
vation.
nitochondrial membrane by preventing the release of apoptogenic molecules from the intermen
egradation of NOS2 (PubMed:21199876). Acts as a bridge which links NOS2 with the ECS E3

ocarbons (PubMed:7961644). Involved in cell-cycle regulation (PubMed:12213388). Likely to p
ining the structural integrity of the Golgi apparatus, possibly by mediating interactions with cyto

ı (By similarity).
scription factors that regulate pro- and antiapoptotic genes. Dephosphorylation of 'Ser-473' of /

esicles during the formation of the neuromuscular junction; stimulating lobuloalveolar budding a

l epithelial soluble proteins (By similarity). May play a role in preventing corneal damage cause
uding lipid metabolism and the inflammatory response. Acts as a receptor for heme which stim
in hematopoietic differentiation (PubMed:8096636).
but its full-length sequence has not been determined. This gene is closely linked to other CTL/
egradation of NOS2 (PubMed:21199876). Acts as a bridge which links NOS2 with the ECS E3
to protect telomeres from DNA degradation (PubMed:19854130). However, the CST complex l

ANKRD53 to the mitotic spindle and spindle poles. May participate in p53/TP53-regulated grow

lates ZAP70 at its activating 'Tyr-493' residue (PubMed:16461343). Dephosphorylates the imm

nory by regulating the Ras and Rap protein signaling: required for overactivity-dependent spine
As are then degraded by two alternative mechanisms, namely exosome-mediated 3'-5' exonucl
5157). Drives internalization of the apelin receptor (By similarity). Apelin-36 dissociates more ha

ration.

M in both binding and ATP-dependent remodeling of DNA. Stabilizes FANCM (PubMed:203474
nbryonic stem cell identity. Compared to EZH2-containing complexes, it is less abundant in em

ɔn of aberrantly long telomeres (PubMed:28082411). Also acts as a transcription regulator that

ɔcalization to lysosomes and its activation. Thereby, may indirectly regulate cell growth, prolifera

on endothelial growth cones in response to netrin (By similarity). It also acts as a dependence r

ted within the enhancer/promoter of hypoxia-inducible target genes and hence inhibits HRE-dri

nation and telomere addition to DSBs via catalytic inhibition of telomerase. Reduces the proces

ce a protein product. [provided by RefSeq, Jun 2013]

to kinetochores. Involved in the response of gonadal tissues to follicle-stimulating hormone.
lultiple alternatively spliced transcript variants encoding different isoforms have been found for

or: inhibits the hepatic acute phase response by preventing dissociation of the N-Cor corepress

ed to IL-8(1-77), respectively.

astoma RB1 protein. Inhibits DAXX-mediated apoptosis by inducing its ubiquitination and degra

omains. The encoded preproprotein is proteolytically processed to generate the mature enzyme
sis via p38 MAPK signaling in muscle-specific and/or growth factor-related transcription. In cer
local sites. Also binds to atypical chemokine receptor ACKR3, which activates the beta-arrestin

onal quality control mechanism. Polyadenylation with short oligo(A) tails is required for the deg
mosome. The read-through transcript is a candidate for nonsense-mediated mRNA decay (NM
tes leukocyte adhesion to endothelial cells (PubMed:23892569). Regulates migration of monoc
hus regulating basal and evoked synaptic transmission. Crucial for normal neuronal developme
d ribosomal protein S6 kinase (S6K) by the protein phosphatase PP2A (PubMed:25816751, Pu
ouples DNA methylation and histone H3 'Lys-9' trimethylation (H3K9me3) activity (Probable).

ctivation of phospholipid signaling.

ator of the canonical pathway and suppresses constitutive activation of non-canonical NF-kapp

onuclease III enzyme to produce an approximately 70-nt stem-loop precursor miRNA (pre-miRN
rget protein, have different functions depending on the Lys residue of the ubiquitin that is linke

nding site of the Walker A box of the adjacent subunit. The six ATPase active sites, however, a

lated suicide of mature T-cells, or both. The secreted isoforms 2 to 6 block apoptosis (in vitro).

rylating proteins are that already phosphorylated on a specific motif recognized by the POLO b
hores and is a key regulator for the onset of cytokinesis during mitosis. Required for central/mi
binding element (DBE) with consensus sequence 5'-TT[G/A]TTTAC-3' (PubMed:10358076). Ac

and cardiac myocyte differentiation. Regulates the circadian clock by repressing the transcripti
ification and differentiation of neuronal cell lineages in the brain (By similarity). May participate

and cardiac myocyte differentiation. Regulates the circadian clock by repressing the transcripti

ding to MCL1 and can displace BIM/BCL2L11 from its binding site on MCL1.

es to lysosomes. Also required for stability and nuclear trafficking of AKT1/AKT which promote
ts an antiproliferative activity via the up-regulation of cell cycle negative regulators CDKN1A/p2

ell migration and TGF-beta-induced epithelial to mesenchymal transition (EMT), potentially throu
cell membrane surface.

ule plus end-directed motility.

ubsequent activation of the kinase AKT1. KITLG/SCF and KIT also transmit signals via GRB2 a

lymerase encounters the 5'-end of a downstream Okazaki fragment. Required for somatic hype

ous bones of the skull (By similarity). May play a role in tumor suppression (Probable).

imilarity). During spinal cord neurogenesis, inhibits V2a interneuron fate (PubMed:17728344).
ibits adenylate cyclase activity. Regulates the release of 5-hydroxytryptamine in the brain, and

onuclease III enzyme to produce an approximately 70-nt stem-loop precursor miRNA (pre-miRN
nt and plays a major role in p53/TP53-driven oxidative stress response. Possesses both a p53

adation of APC/C substrates by the proteasome and promoting mitotic exit.

Regulates myeloid cell differentiation by altering the expression of CEBPA in a COP1-depende
phase. Essential for proper chromosome segregation during mitosis and this function requires

:harge barrier: the protein has a high net electrical charge and acts as a surfactant, dispersing c

:sicles (DCVs) and other dense-core vesicles (By similarity).

promotes stable microtubule-kinetochore attachments (PubMed:21402792). Required for kineto

s to both poly- and mono-ubiquitin and regulates ligand-induced degradation of EGFR, thereby

However, in another study, does not appear to be implicated in repressor activity of a NRSE mo
9). Core component of the UV-DDB complex (UV-damaged DNA-binding protein complex), a co

pate in the transmission of nuclear signals during development and in the adult, as illustrated b

ematopoietic cells, control of mesenchymal cell proliferation and differentiation, wound healing,

s no demethylase activity toward H3K9me2, while it has high activity toward H3K27me2. Demet

tion, by participating in recruitment to androgen-receptor target genes, resulting in H3 'Lys-9' de
and CBL. Activates the AKT1 signaling pathway by phosphorylation of PIK3R1, the regulatory

of cell stresses and induces cell cycle arrest and apoptosis in response to ER stress (PubMed:

N3A/Nav1.3, SCN5A/Nav1.5, SCN9A/Nav1.7, SCN10A/Nav1.8, KCNA3/Kv1.3, KCNH2, EAAT

Acts synergistically with NR5A1 on CYP17 transcriptional activity.
complex PP2A into the cytoplasm, prevents the dephosphorylation of the AP-1 subunit JUN w

e DNA backbone by beta-delta elimination to generate a single-strand break at the site of the r

us junctions within striated muscle cells.

ed by RefSeq, May 2012]

0687510, PubMed:18511904, PubMed:9873047). Phosphorylates CREB1 and ATF1 in respons
isoforms. A paralog of this gene which also has a trypsin‐like peptidase domain, FAM1

onuclease III enzyme to produce an approximately 70-nt stem-loop precursor miRNA (pre-miRN
in chromatin modification: through its tudor-like regions and PHD-type zinc fingers, specifically

signaling via G protein-coupled receptors and GNAQ. Plays a role in regulating the constrictior
ACATCTG[GT][AC]-3' which contains the E box core, and acts by recruiting chromatin remodel

onuclease III enzyme to produce an approximately 70-nt stem-loop precursor miRNA (pre-miRN

edominantly produce interleukin-10 (IL10) (PubMed:10581077).||The PDCD1-mediated inhibito
d SMAD3 then form a heteromeric complex with SMAD4 that translocates to the nucleus to reg
onuclease III enzyme to produce an approximately 70-nt stem-loop precursor miRNA (pre-miRN
the testis-specific histone gene H1-6 (By similarity).
ion velocity but reduces directionality.

, acts mainly as an activator of gene expression due to weak binding to corepressors (PubMed
and TF. May also function in lipid trafficking. Protects cells from spindle disruption damage. Fu

rotein reductase). Catalyzes the hydroxylation of carbon hydrogen bonds of atRA primarily at C

nore positive voltages. The inward rectification is mainly due to the blockage of outward current
al transduction of associated receptors by dephosphorylation of their downstream effectors. Pl

ds to single-stranded DNA in an ATP-dependent manner to form nucleoprotein filaments which

Pase activity that converts bound GTP to GDP, thereby terminating the signal. Both GDP relea

onuclease III enzyme to produce an approximately 70-nt stem-loop precursor miRNA (pre-miRN

of the skin and for postnatal maturation and maintenance of the ocular surface. Involved in the
tively regulates G1-to-S-phase transition of the cell cycle (PubMed:18959821). In endothelial c

orylation and nuclear accumulation, which causes cell cycle arrest (By similarity).

n, the two isoforms exert an additive effect. Isoform 2 regulates p53/TP53 expression and cellu

cells (PubMed:24623306). Binds to the USP28 promoter in colorectal cancer (CRC) cells (Pub

of TGF-beta (PubMed:22278742). Able to outcompete LTBP1 for binding to LAP regulatory ch
Jul 2008]

latory T-cells (Tregs) (Probable). Required during vasculogenesis (By similarity).

onuclease III enzyme to produce an approximately 70-nt stem-loop precursor miRNA (pre-miRN
similarity). Together with DNASE1L3, plays a key role in degrading neutrophil extracellular trap
ed:25519132). Component of a PRC1-like complex that mediates monoubiquitination of histon

nd CTNNB1. Expression of dominant-negative mutants results in cell-cycle arrest in G1. Neces
of the Polycomb repressive complex 2 (PRC2) to major satellites (By similarity). Stimulates the

chore activities that depend on the kinetochore motor CENPE. Required for kinetochore localiz
richment of AUKRB in prometaphase. Plays an important role in defining SGO1 localization an

es. Is thus essential for an accurate gametogenesis. May act by targeting PPP2CA to centrome

gene silencing (By similarity). In association with DNMT1 and via the recruitment of CTCFL/BC

451).
sion of TFAP2A acts as a negative regulator of osteo-dentiogenic capacity in adult stem cells;

ensity lipoproteins (HDL) more efficiently than other lipoproteins (PubMed:12032167, PubMed:
erial cell fate or identity. Promotes maintenance of neuronal precursor cells and glial versus ne

›f many cells in vertebrates (PubMed:9624188). However, it may play a role in Wnt signaling (F

PubMed:29634919). Acts as a central dissolvase of membraneless organelles during the G2-t
›n of target genes. The PHF2-ARID5B complex acts as a coactivator of HNF4A in liver. Require

rogrammed cell death following DNA damage. Stimulates both innate and acquired immune re

e. [provided by RefSeq, Jul 2014]
(By similarity). Inhibits MYOCD activity. Is a negative regulator of TP53 acetylation. Inhibits NF-
›ss-links. Stimulates DNA decatenation mediated by TOP2A. Prevents sister chromatid exchar
is invariably located at the same site in the gene, placing the splice sites an invariant distance
ıaling pathway.

4570867). Binds APOA1 and may function in apolipoprotein-mediated phospholipid efflux from

similarity). Has been proposed to act as a cardiolipin hydrolase to generate phosphatidic acid a
›53/p73-induced cell death (By similarity).
d nucleosome remodeling.

ruitment of the reductase to the ubiquitin ligase, AMFR/gp78. May play a role in growth and diff

/beta-catenin signaling pathway leads to the activation of disheveled proteins, inhibition of GSK

ıg pathway involving PKC and calcium fluxes has been seen for some family members, but it is

robably by modulating channel properties (By similarity).

›nuclease III enzyme to produce an approximately 70-nt stem-loop precursor miRNA (pre-miRN

CN, thereby inactivating mTORC1 and promoting nuclear translocation of TFEB and TFE3 (Pub

or AURKC, the N-terminus associated with BIRC5/survivin and CDCA8/borealin tethers the CP
osphorylates and inactivates YAP1 oncoprotein and WWTR1/TAZ. Acts by mediating gene exp

35036).

d nucleosome remodeling.

g MCAM/MUC18, C/EBP alpha and MYC. AP-2-alpha is the only AP-2 protein required for early

acetyltransferase activity. Binds to chromatin.

hematopoietic cell adhesion to fibronectin and seems to contribute to the adhesion of hemato

ne expression (By similarity). Binds to BMP response element (BMPRE) DNA sequences withi

plication event 3 Mb distal to RANBP2. Alternative splicing has been observed for this locus ar

minoglycan, and the Gal residues in sialyl N-acetyllactosamine (sialyl LacNAc) oligosaccharide
PubMed:12919066). Signaling leads to the activation of intracellular protein kinases and the ph

ays a role in dendrite formation by melanocytes (PubMed:23999003).

2) and histone H4 at 'Lys-20' (H4K20me1 and H4K20me2), leading to condense chromatin and
vate expression of genes such as NFE2, ITGA2B, alpha- and beta-globin, while it represses ex
ture on mRNAs. Does not hydrolyze cap analog structures like 7-methylguanosine nucleoside t

ay also play a role in cell adhesion (By similarity).
activity on 8-oxoG detected. Also shows weak DNA-(apurinic or apyrimidinic site) lyase activity

s in the heart (By similarity).

nd opposite effects in numerous biological processes (PubMed:8626589, PubMed:15544348).
kinesis (PubMed:26246606). Required for normal spindle positioning during mitosis and for the

regions.

s its dephosphorylation. Positively regulates PDPK1 kinase activity by stimulating its dissociati
nscriptional activation of a subset of NOTCH1 target genes including MYC. Involved in thymoc

on chromosome X. [provided by RefSeq, Jul 2008]

plex with the MutL alpha heterodimer, which is thought to be responsible for directing the dow
chidonate (AA, C20:4(n-6)) to the hydroperoxy endoperoxide prostaglandin G2 (PGG2), and th

ne of them leading to cellular transformation (PubMed:25642963, PubMed:25643323). POLQ

:28846097, PubMed:28846098, PubMed:28846099). It then triggers the activation of neurons l

bably plays a role in regulating protein degradation via its interaction with NEDD4 (PubMed:157
104747).

ɔnuclease III enzyme to produce an approximately 70-nt stem-loop precursor miRNA (pre-miRN

ᑭ3. May be involved in regulating folliculogenesis. Through interaction with OCBA/POU2AF1, a

ɔf MAPK1/3 in local wound tissue, which further contributes to resolution of acute pain (By simi
mplex (PubMed:15016378). Plays a role in immune response by modulating phagocytosis, infla

chromosome congression and alignment during mitosis through its interaction with KIF22.

ɔr of S-phase by recognizing and binding the E2-related site 5'-TTCCCGCC-3' and mediating re
ɔnuclease III enzyme to produce an approximately 70-nt stem-loop precursor miRNA (pre-miRN
ı of reduced glutathione to cause a decrease in intracellular reactive oxygen species (ROS) cor

regulation mediated by ESR1. Involved in mitochondrial depolarization and neurite outgrowth.
65040, PubMed:18511070). Mechanistically, uses molecular oxygen inserting one oxygen atom
ıces the sequence-specific DNA-binding capacity of RUNX. The heterodimers bind to the core
oth muscle cells than EGF. Also acts as a diphtheria toxin receptor.
to generate pre-replication complexes (pre-RC)(PubMed:14672932). Required also for mitosis
MOB1, which in turn phosphorylates and inactivates YAP1 oncoprotein and WWTR1/TAZ. Pho

hydrogen sulfide, a gasotransmitter with signaling and cytoprotective effects on neurons (By si
Regulates the subcellular localization of p53/TP53 and subsequent function (PubMed:1252679

similarity).

ɔtide dissociation inhibitor (GDI) activity towards G(i) alpha proteins, such as GNAI1 and GNAI3

ependent manner. Activates cofilin at axon tips via local activation of JNK, thereby regulating fi
gative regulator of adipogenesis (By similarity). The AML1-MTG8/ETO fusion protein frequently

7/TCF1, thereby inhibiting transcription and modulates normal gamma-delta T-cell developmer

all nuclear RNA to form the core snRNP. In the cytosol, the Sm proteins SNRPD1, SNRPD2, S

ns, including lipid and bile acid metabolism, adipogenesis, gluconeogenesis and the macropha
l-cycle checkpoint activation, recombinational repair and maintenance of genomic stability. Re

an important regulator of global heterochromatin silencing and critical in maintaining genome
l for the formation of bipolar spindles and high-fidelity chromosome separation) by phosphoryla

eiotic silencing of unsynapsed chromatin (MSUC) or meiotic surveillance.
and transports it into cells by endocytosis. In order to be internalized, the receptor-ligand compl

ells (ESCs). Specifically promotes SPRY1 and RSPO1 transcription activation through recogniti
lyze either glycerophosphoinositol or glycerophosphocholine.

onuclease III enzyme to produce an approximately 70-nt stem-loop precursor miRNA (pre-miRI

T-cells. Integrin beta-1 stimulation leads to recruitment of various proteins including CRK, NCK

ɔution of phosphatidylserine (PS) in spermatozoa membranes. Involved in acrosome reactions

ıding of JAG1 to NOTCH2 but not that of DLL1 (PubMed:11346656). Essential mediator of son

GEF) for RAB5A, by exchanging bound GDP for free GTP, and facilitating Ras-activated recep
CNMA1 channel that is detectable only at large depolarizations. In contrast, isoform 1 does no

on on adipocytes, but acts by stimulating several immune cell subtypes to enter the adipose tis

T-cells and in innate immunity. Controls the proliferation and homing of CD8+ T-cells via the k
nd degraded in the cytoplasm by the BCR(KEAP1) complex (PubMed:11035812, PubMed:156
regulates many genes important for osteoclast differentiation and function (By similarity).

580347, PubMed:15713661, PubMed:15201869). May also regulate NF-kappa-B, ESR1, NR3

ıve been found for this gene. [provided by RefSeq, Jul 2010]
ıment.

0684968]).[supplied by OMIM, Mar 2008]

'C-3 prostate cancer cells.
onse to growth factors, DNA damage, and ischemia. Plays a role in the regulation of cell surviv

esence of primed DNA, replication protein A (RPA) and by proliferating cell nuclear antigen (PC

RNA into the cytoplasm of neurons. Acts as a key regulator of synaptic plasticity: required for p

ent of VAV2, VAV3 and PI3-kinase p85 subunit by phosphorylated EPHA2 is critical for EFNA1

clustering at active zone to the presynaptic membrane in postmitotic neurons. CDC20-APC/C-ir

orylates PARVA/actopaxin, APC, AMPH, APC, BARD1, Bcl-xL/BCL2L1, BRCA2, CALD1, CAS

ndent phospholipid phosphatase of the plasma membrane that catalyzes the dephosphorylatio

during mitosis (PubMed:25847991). May act as a tumor suppressor (PubMed:25847991). May

stability and nuclear localization of FA core complex proteins, as well as for FANCD2 monoubic

mmation. Regulates cytoskeleton rearrangement and cell spreading in T-cells, and contributes

lability is insufficient (By similarity).
cylglycerides to free fatty acids and glycerol. Hydrolyzes of 2-arachidonoylglycerol and prostagl

nd promotes extension of the ciliary membrane. Firstly the BBSome associates with the ciliary r
he function seems to require its catalytic activity. Involved in regulation of autophagy (PubMed:

on with DTX3L and in presence of E1 and E2 enzymes, mediates NAD(+)-dependent mono-AD
deacetylation of lysine residues on the N-terminal part of the core histones (H2A, H2B, H3 and
amically with various co-chaperones that modulate its substrate recognition, ATPase cycle and

ludes RNA binding.

rectly regulates neurite outgrowth (By similarity). Plays a role in myoblast fusion (By similarity).
ephritis, including influx of monocytes and macrophages and production of extracellular matrix (
the lesion, the primer is further extended by the tetrameric POLZ complex. In the case of 1,2-i

ubMed:9462508, PubMed:10754304, PubMed:18193050, PubMed:23761635). Signals via the

ıblishing boundaries between skeletal elements during development. Regulation of GDF6 expr
CCL8, CCL13, CCL19, CCL21 and CCL25. Chemokine-binding does not activate G-protein-m

fication of excitatory synapses. Required to maintain wakefulness quality and normal synchron

mon fragile sites (CFS) stability in a RBBP6- and MCM10-dependent manner; represses expre

:es (PubMed:28717008).

ıilarity). Functions also as an acyl-CoA retinol acyltransferase (ARAT) (By similarity). Also able

es.

CRs on MAIT cells, guiding immune surveillance of the microbial metabolome at mucosal epith
transcriptional regulator of genes controlling female reproductive function. May play a role in g
ubMed:24100331, PubMed:23263184). The phosphorylation of EPRS1, induced by interferon-
TP from the corresponding diphosphate substrates with either ATP or GTP as phosphate dono

ns in the subsequent plus-strand viral DNA. The resultant detrimental levels of mutations in the

pend on corepressors, such as CTBP1 or CTBP2 and MTA1. The regulation of SIRT1 transcri

ral signaling protein (MAVS/IPS1) which activates the IKK-related kinases: TBK1 and IKBKE w

xadecenoic acid (PubMed:22633490).

By similarity) (PubMed:22863753). May affect TLR4 signaling by acting at the level of TRAF6 u

a. While some claudin family members play essential roles in the formation of impermeable ba

aling in tumor progression.

the early developing brain long before myelination, maybe as components of transcriptional co

other GALNT proteins. Prefers Muc1a as substrate.

xidation step from 6-deoxypenciclovir to penciclovir, which is a potent antiviral agent. Is probab

dietary Mg(2+) levels, and thereby contributes to the regulation of renal Mg(2+) reabsorption (I

ɔgenesis and is involved in spermatid maturation (PubMed:10373550).
ɔed; however, not all variants have been fully characterized. [provided by RefSeq, Jul 2008]

for short or long regions of sequence homology in the recombining DNA templates, and may a

similarity).

ɛlopment and normal myelination in the spinal cord and cerebellum. Plays an important role in v

and long-term memory (By similarity). Plays a role in the actin and microtubule cytoskeleton or
)08]

jen I.||The gamma-CTF peptide, C30, is a potent enhancer of neuronal apoptosis.
G-I-mediated antiviral signaling in an RNA length-dependent manner through ubiquitination-dep
nin pathway (canonical Wnt signaling pathway) in a CCAR2-dependent manner by sequesterin

IFN-beta) (PubMed:18724357, PubMed:18818105, PubMed:19433799, PubMed:19776740, P

ɔe contributing to the generation of action potentials. Regulates smooth muscle cell proliferatior

by permitting water to move in the direction of an osmotic gradient. Slightly permeable to urea

onformation. The beta-arrestins target many receptors for internalization by acting as endocytic

r dendrite morphogenesis and axon targeting in the vertebrate visual system, thereby playing a

lation which negatively regulates pro-inflammatory cytokine production in macrophages in resp
n the 3'UTR of ITGA3. Binds to CUG triplet repeat expansion in myotonic dystrophy muscle cell
entiate in type 1 effector cells (TH1), type 2 effector cells (TH2) and IL17 producing helper T-cel
omplex DCP1-DCP2 through RNA helicase p72 (DDX17) to remove the cap structure of the vir

ed:27534431). Plays a role in the activation of innate immune response during viral infection. M

or used for glycolytic ATP production. The transition between the 2 forms contributes to the cor

nic-apyrimidinic (AP) endonuclease and 3'-5' exonuclease activities in vitro. Also able to introdu

rane, apparently linked to the ABCA1-mediated cholesterol secretion pathway. Recruits CYTH1
ade (PubMed:19109178). Negative regulator of Notch signaling pathway involved in embryonic

tern of sulfation of heparan sulfate. Compared to other NDST enzymes, its presence is absolut
drate is a carbohydrate structure present in alpha-dystroglycan (DAG1), which is required for b
tion of MMP15. Involved in the formation of the fibrovascular tissues in association with pro-MM
17145758). Moreover, is involved in the metabolism of aggrecan (By similarity).
ys a role in B-cell development, proliferation, migration, and function. Required for B-cell recep

1' of histone H4 (H4K91ub1) (PubMed:19818714). The exact role of H4K91ub1 in DNA damag

o the actin cytoskeleton. May be involved in signal transduction from cell adhesion sites to the r

ɔxylate long and very long straight chain acyl-CoAs or 2-methyl- and 4-methyl-branched acyl-C
255211). Plays an important role in the control of melanin production and melanosome biogene

ls for the leukocyte adhesion protein LFA-1 (integrin alpha-L/beta-2). During leukocyte trans-er

mplex that recognizes UV-induced DNA damage and recruit proteins of the nucleotide excision

ovides docking sites for downstream signaling molecules. Following activation by ligand, interac
w intrinsic GTPase activity. GTPase activity is increased by contact with 60S ribosome subunit

ısm by binding growth factor GAS6 and which is thus regulating many physiological processes
B sites in the DNA of their target genes and the individual dimers have distinct preferences for

spase cleavage sites. Also important in the quality control of protein N-myristoylation in which N

f tumor metastasis. Kp-10 is a decapeptide derived from the primary translation product, isolat

5, PubMed:25315659). Regulates lipolysaccharide (LPS)-induced endothelial cell migration (Pu

athrin-mediated endocytosis independently of the AP-2 complex. Involved in endocytosis of int

ed:15544348). Also plays an important role in the biosynthesis of complex lipids (Probable). Ca
nsmission of mechanical forces across the nuclear envelope and in nuclear movement and pos
le in controlling excitability in a number of systems, such as regulation of the contraction of smo
l to the Golgi in a lipid-raft dependent pathway (PubMed:16880211). Acts via MYD88 and TRA

d the inflammatory response.
cling (By similarity).
of the lipoprotein lipase LPL, and thereby plays a role in the regulation of triglyceride clearance

ubMed:10464285, PubMed:9880569, PubMed:11682059). Activation of RNASEL leads to degr
rator-activated receptors PPARA transcriptional activity. Enhances estrogen-dependent transa
tion and subsequent activation. Activation of RNase L leads to degradation of cellular as well a

transactivation (PubMed:10454579, PubMed:25219498). Also involved in androgen receptor t

s a substrate for VE-PTP and is required by it to stimulate VE-cadherin function in endothelial c

nd promotes extension of the ciliary membrane. Firstly the BBSome associates with the ciliary r

AF complexes. As neural progenitors exit mitosis and differentiate into neurons, npBAF comple

olved in mitochondrion degradation of damaged mitochondria by promoting the formation of va
the G(1) phase. Hypophosphorylates RB1 in early G(1) phase. Cyclin D-CDK4 complexes are r

al domains. The encoded preproprotein is proteolytically processed to generate the mature prot
1711). A fraction of the 2-oxoglutarate dehydrogenase complex also localizes in the nucleus a
ct non-neuronal cells (PubMed:15781252, PubMed:15775988). Acts as a negative feedback re
ssociation with DMN1L and stimulation of its GTPase activity in synaptic vesicles. May attenua

mponent (p150-glued) DCNT1. Involved in membrane-transport, such as Golgi apparatus, late e
Involved in the anti-inflammatory response of macrophages and positively regulates TLR-induc
eorganization of actin cytoskeleton and focal adhesion dissolution.

ells to collagens, modulation of collagen and collagenase gene expression, force generation ar

e stimulation by CSPG4 of endothelial cells migration.

of Kv4 channels. Functional regulator of Kv1.5 channel. During long-term depression in hippoca

's), with some preference for PtdIns(3)P (By similarity).

MALM) leading to the degradation of damaged proteins inside mitochondria. The physical intera

d nucleosome remodeling.||Has broad antibacterial activity. May contribute to the formation of t

n a second step, the cluster is released from ISCU, transferred to a glutaredoxin GLRX5, follow

ositol 1,3,4,5-tetrakisphosphate (PubMed:9108392, PubMed:10764818, PubMed:8769125). Act
ent stress-response pathway leading to cytochrome c release from mitochondria and caspase-c

bMed:24368842). Regulates the levels of fatty acids that serve as signaling molecules and pro

d nucleosome remodeling.||Has broad antibacterial activity. May contribute to the formation of t

GF-beta-1 activation mediated by LRRC33/NRROS is highly localized: there is little spreading o

tidylinositols, such as phosphatidylinositol (4,5)-bisphosphate (PubMed:28459430). Mediates s

orphogenesis during embryonic development. Plays an important role in wound healing, where

esses, including cell cycle progression, apoptosis, or DNA damage repair.
dues are sulfated. May function in the sulfation of sialyl N-acetyllactosamine oligosaccharide ch

could impair cellular functions, and by removing proteins whose functions are no longer require

romoter (By similarity).

oproteasome to cleave model peptides after hydrophobic and basic residues.

arity). Regulates the equilibrium between cell surface-associated and cell surface-dissociated c

,N-dimethyl-4-4'-bipiridinium); a widely used herbicid. Responsible for the secretion of cationic d

t the AP-1/SMAD site to regulate TGF-beta-mediated transcription. Has an inhibitory effect on
activity and other activities relevant to asthma. Required for tonic airway smooth muscle contra

minogen activators and their endogenous inhibitors. May modulate cellular events, such as AP

ligase activity, and in concert with the heterodimeric ubiquitin conjugating enzyme complex UBI

re interlocked as it represses the expression of PER1/2 and in turn is repressed by PER1/2 and

ed for normal development of peripheral nervous system and skeletal muscle and for muscle s
.4 Mb region that is commonly deleted in diverse tumors. A related pseudogene has been ider
junction assembly (By similarity). Plays a role in regulating monocyte transmigration involved i
some organization and function. Essential for the centrosomal recruitment of key proteins that c
the G(1) phase. Hypophosphorylates RB1 in early G(1) phase. Cyclin D-CDK4 complexes are r

forms have been found for this gene. [provided by RefSeq, Dec 2009]

nucleosome remodeling.

5 inclusion by antagonizing the repressive effect of PTB. Acts as both an activator and repress

abilities are down-regulated by tyrosine kinase PTK6 (PubMed:10564820, PubMed:19561594

ns in the subsequent plus-strand viral DNA. The resultant detrimental levels of mutations in the
rough the cleavage of CGAS, rendering it inactive (PubMed:28314590). In apoptotic cells, clea
AP. Inhibited by human cytomegalovirus US6 glycoprotein, which binds to the lumenal side of

-TSC2, which leads to the activation of the RHEB GTPase that potently activates the protein k

tracellular calcium ion levels and enhancing MAPK1/MAPK3 activation (PubMed:10452968, Pu

) (By similarity).
then dispersing throughout the cytoplasm in terminally differentiating cells. The type II keratins

n cilium assembly (PubMed:21211617).
in cellular lipid homeostasis may not be limited to macrophages. Prevents cell death by transp

ɔh receptors to bind and to be physiologically activated by both GPI-anchored ephrin-A and trar

sequence 5'-GTGXGCXGC-3' (PubMed:21767813). In the absence of endoplasmic reticulum (I
gene have been observed in neurofibromatosis type 1-associated glomus tumors. Alternative

proliferation and survival. Recruited by ZBTB7A to the androgen response elements/ARE on t

iired for proper skeletal muscle differentiation by sequestering FGF2 in lipid rafts preventing its
ı. May inhibit PI3 kinase by binding to AGAP2 and impairing its stimulating activity. Suppresses
ens (PubMed:25493333). Binding of alpha-beta TR to pMH complex initiates TR-CD3 clustering

oute to mortality. Regulates the activity of the neutrophil proteases elastase, cathepsin G, prote

olexes. A TRIM21-containing SCF(SKP2)-like complex is shown to mediate ubiquitination of CD

ce, may regulate p53/TP53 expression and mediate in part the CDKN2A anti-proliferative activit

eosomal histones H4 and H2A (PubMed:14966270). This modification may both alter nucleosc
binding to the adapter protein SFN and by stimulating Akt/mTOR pathway (By similarity). Involv

ide headgroup (Ref.6).

es of nicotinic acetylcholine receptors (nAChRs) to prevent excessive excitation, and hence ne
/ similarity).

lineage; this interaction preventing EBF1 to bind DNA and activate target genes.

3-trans double bonds into the 2-trans form in a range of enoyl-CoA species (By similarity). Wit
mational changes in the ribosome.
17344214). May mediate cellular responses to activated FGFR1, FGFR2, FGFR3 and FGFR4
3s) formation and sorting of endosomal cargo proteins into MVBs. MVBs contain intraluminal ve

ediator of innate immunity NF-kappa-B by promoting TNF-alpha-mediated proteasomal degrada
ffinity for the receptor tyrosine kinase EPHB1/ELK (PubMed:8070404, PubMed:7973638). Can

ation.

P, converting inactive GDP-bound Rab proteins into their active GTP-bound form.||[Isoform 3]:

to miRNAs. May play a role during primordial germ cell (PGC) survival (By similarity). However
glycerophosphocholine.

a central stalk and a peripheral stalk. During catalysis, ATP synthesis in the catalytic domain

onuclease III enzyme to produce an approximately 70-nt stem-loop precursor miRNA (pre-miRN
6449189, PubMed:22607976). May be involved in endoplasmic reticulum stress-induced pre-e

Does not show subunit-specific AMPA receptor regulation and regulates all AMPAR subunits.

nes. Regulates the expression of specific genes involved in differentiation and survival within a
3y similarity). Required during the transcriptionally active period of oocyte growth for the establis

ected to play important roles in signal transduction. Alternatively spliced transcript variants have
neurites and nerve terminals.
onuclease III enzyme to produce an approximately 70-nt stem-loop precursor miRNA (pre-miRN

the recognition of pri-miRNA at dsRNA-ssRNA junction and directs DROSHA to cleave 11 bp a
secretion by pancreatic islet cells through activation of this receptor.

nilarity). Involved in DNA synthesis during DNA repair (PubMed:20227374, PubMed:27573199

strong preference for a specific histone mark, H3K18Ac, directly linked to control of gene expre
e and as heterodimer with CEBPB (PubMed:1741402).

gene expression. Its deletion enhances angiogenesis in vivo. Many experimental evidences de
eural progenitors-specific chromatin remodeling complex (npBAF complex) and the neuron-spe

' KIF7 and GLI2 at the ciliary tip for GLI2 activation (By similarity).

48521). It is required for transcription-coupled repair complex formation (PubMed:16916636). It
ir to act in brain and pituitary gland to facilitate the loss of plasma volume, actions which compl
is required for RAB32/38 activation (PubMed:26620560).

e.
nd plays a role in cardiac hypertrophy, VEGFA-induced angiogenesis, genotoxic-induced apopt

ie intersection of the four duplex arms of the Holliday junction and to junction of replication fork
of the Holliday junction (HJ) and to junctions of replication forks. Involved in HJ resolution and
UBE2C/UBCH10 on APC/C substrates, enhancing the degradation of APC/C substrates by th

en the 5'- and 3'-splice site binding components, U1 snRNP and U2AF. Binds to purine-rich RN

arity). Promotes assembly of stress granules (SGs), when overexpressed.
occurs exclusively on 'Tyr-15' and phosphorylation of monomeric CDK1 does not occur. Its ac

gulator of a wide array of physiological functions including metabolism, sleep, body temperature

sport vesicles containing N-methyl-D-aspartate (NMDA) receptor subunit NR2B along microtubu

ɔressibility (PubMed:21282530). Binds to histone H3 trimethylated at 'Lys-9' (H3K9me3) or at 'L

D1. [provided by RefSeq, Dec 2017]

ɔmyocytes, adipogenesis and angiogenesis. Functions as an endoribonuclease involved in mRI

fically to histone H3-containing nucleosomes at the centromere, as opposed to nucleosomes c
ɔnuclease III enzyme to produce an approximately 70-nt stem-loop precursor miRNA (pre-miRN

coneogenesis and glucose output by repressing PPARGC1A transcriptional activity on glucone
ɔnuclease III enzyme to produce an approximately 70-nt stem-loop precursor miRNA (pre-miRN

hen progressively more methyl groups are incorporated into H3K27, H3K27me0 > H3K27me1 :
ıatch PLK4 away from PLK4:CEP92 complexes in early G1 daughter centriole and to repositio
ɔnuclease III enzyme to produce an approximately 70-nt stem-loop precursor miRNA (pre-miRN

ɿation activity and, together with NFKB1/p50 and RELA, is recruited to LCN2 promoters. Promo
f histone H4 and nucleosomes (PubMed:24396869). H4 'Lys-20' trimethylation represents a sp

ł 18:1 unsaturated fatty acids (PubMed:15610069). Plays an important role in lipid biosynthesis

vitamin D analog, into 1alpha,2beta,25-trihydroxy vitamin D3; this reaction occurs via enzymatic

nuclear and viral gene expression in a phosphorylated and acetylated status-dependent manner

from NADH and succinate to molecular oxygen, creating an electrochemical gradient over the

gulator of a wide array of physiological functions including metabolism, sleep, body temperature

a broad range of proteins and it has been estimated that they may have approximately 200 to
tion and guide them through the mitochondrial intermembrane space.

ion and survival. May play an important role in brain development and function, and in skeletal
gulator of a wide array of physiological functions including metabolism, sleep, body temperature

f NF-kappa-B. Required for the activation of CASP8 in FAS-mediated apoptosis. Required for

11677242, PubMed:11739383). Has also been shown to be involved in MSH2 deacetylation (P

genes and cytokine mRNAs. Binds both to nuclear and cytoplasmic poly(A) mRNAs. Binds to p

-like growth factors, and may regulate the expression of genes involved in metabolism. [provide
udes the association of TBP with TFIID subunits. Recruits RNA polymerase I to the rRNA gene

role in the primary cilium by controlling ciliary growth and phosphoinositide 3-kinase (PI3K) sig

rder structures. This gene is a histone pseudogene found on chromosome 1. [provided by Ref
ent and cooperates with the transcription factor 1 complex (PTF1) bound to the enhancer A ele
romosome. The read-through transcript is a candidate for nonsense-mediated mRNA decay (N
a central stalk and a peripheral stalk. During catalysis, ATP synthesis in the catalytic domain o

ficiency virus type 1 (HIV-1) and facilitates its capture by platelets (PubMed:16940507).||C-type
onuclease III enzyme to produce an approximately 70-nt stem-loop precursor miRNA (pre-miRN

orane and involved in invasive cell migration. In T-cells involved in endosome-to-membrane rec

nange that triggers signaling via guanine nucleotide-binding proteins (G proteins) and modulate

nacrophages.

onuclease III enzyme to produce an approximately 70-nt stem-loop precursor miRNA (pre-miRN

le in embryo implantation and parturition through its ability to trigger prostanoid production (By

e and allopregnanolone (PubMed:23763432, PubMed:23161870).
that is essential for normal brain growth and cognitive function, enters the brain (PubMed:260

s, are known. [provided by RefSeq, Jul 2008]

udes the association of TBP with TFIID subunits.

egranulation (PubMed:25171405). May also have serine/threonine protein kinase activity. Requ

gulator of a wide array of physiological functions including metabolism, sleep, body temperature

pithelium (By similarity). Acts as a regulator of cilium length, together with PKD2 (By similarity).

osomal proteins, there are multiple processed pseudogenes of this gene dispersed through the

sorption of calcium from the intestine. Plays dual roles in osteoclast differentiation and survival
ed for their localization. Binds myristoylated GNAT1 and is required for G-protein localization a

mily 3, group C, member 1). This action blocks the glucocorticoid receptor from being activated

synthesis (PubMed:10648783, PubMed:21854770, PubMed:23797032, PubMed:26089203, P

by NR1D1 and THRB (By similarity).

plasm and thus preserving mitotic spindle integrity. Prevents the formation of the STIL-CENPJ c

no-group acceptor (in vitro).

y a central stalk and a peripheral stalk. During catalysis, ATP synthesis in the catalytic domain c

directly repressing cyclin-dependent kinase inhibitors, including the p27Kip1 promoter (By sim

onuclease III enzyme to produce an approximately 70-nt stem-loop precursor miRNA (pre-miRN

plex, C9orf72 and SMCR8 probably constitute the catalytic subunits that promote the exchang
e transition from proliferating neural stem/progenitor cells to post-mitotic neurons requires a sw

is selectively and stereospecifically stimulated by phosphatidylserine (PS). The flippase compl
ty).
onuclease III enzyme to produce an approximately 70-nt stem-loop precursor miRNA (pre-miRN

onuclease III enzyme to produce an approximately 70-nt stem-loop precursor miRNA (pre-miRN

onuclease III enzyme to produce an approximately 70-nt stem-loop precursor miRNA (pre-miRN

5916645, PubMed:17938197, PubMed:19111657, PubMed:19589784, PubMed:28437106). Als

tional transcription factors. Regulates the expression of SLC6A9, a glycine transporter which re

220 protein domains. Gene copy number variations in the human chromosomal region 1q21.1,

rate, and reducing the second into a water molecule, with two electrons provided by NADPH vi

onuclease III enzyme to produce an approximately 70-nt stem-loop precursor miRNA (pre-miRN

In the ear, this may lead to a reduction in basilar membrane motion, altering the activity of aud
ulation of cardiac contractility and to the maintenance of proper cardiac conduction activity prob
Phosphorylation at Ser-628 during G1/S phase maximizes the deubiquitinase activity, leading to

ed:9635433, PubMed:14993212, PubMed:24064211). Inhibits histone acetyltransferase activity

onuclease III enzyme to produce an approximately 70-nt stem-loop precursor miRNA (pre-miRN
Pyridoxal phosphate (PLP) phosphatase, which also catalyzes the dephosphorylation of pyrido

tory to inhibitory synapses in hippocampal neurons. May reduce the amplitude of ASIC3 acid-e

in-containing and oxidatively truncated phospholipids.

nacrine and bipolar cells in the developing retina (By similarity). Likely to play a regulatory role

onuclease III enzyme to produce an approximately 70-nt stem-loop precursor miRNA (pre-miRN

be needed to protect the cell from overactive protein degradation. Several transcript variants e

s substrate (PubMed:24981631). The thermodynamic coupling of the irreversible SDO and rev

amyloid-beta peptide-induced apoptosis (PubMed:11007892).

s recombination by recruiting the SMC1-SMC3 cohesin complex to double-strand breaks. The c

34968]).[supplied by OMIM, Mar 2008]

ɔmplex at the channel-forming translocon complex that mediates protein translocation across tl
ɔnuclease III enzyme to produce an approximately 70-nt stem-loop precursor miRNA (pre-miRI
ɔnuclease III enzyme to produce an approximately 70-nt stem-loop precursor miRNA (pre-miRI

'9290]).[supplied by OMIM, Mar 2008]
osphorylates and mediates phosphorylation of GLI2, activating it and promoting its nuclear trar

ription of the c-FOS promoter is inhibited by BRG1-dependent recruitment of a phospho-RB1-I

inal increase in neuronal production (PubMed:29856954). Acts by enhancing the Notch signalii
throline, tannic acid, and mercurials (mercury dichloride, mersalyl acid, p-hydroxymercuribenzo

ɔt ganglia (DRG) neurons. Mechanistically, it weakens their interaction, thereby releasing the in
, a central downstream effector for the meiotic program. Acts independentely of retinoic acid (F
ediate both cell proliferation and TP53/p53-dependent apoptosis. Blocks adipocyte differentiatio
etory pathway involving endoplasmic reticulum (ER) export sites (ERESs) and ER to Golgi tran
quired for functional expression L-type calcium channels that contain CACNA1D as pore-formin

by modulating mesenchymal cell responsiveness to BMPs. Upregulates BMPR1A expression i

from NADH and succinate to molecular oxygen, creating an electrochemical gradient over the

nscriptional regulator for numerous genes, including some genes involved in cancer metastasis

pansion and differentiation of B lymphocytes into immunoglobulins-secreting plasma cells. Sec

NA5 most probably constitutes the cognate/functional ligand for EPHA5. Functions as an axon
nactivation. May be involved in glucose-stimulated insulin production in pancreas and regulatio
tures.||Binds and inhibits the catalytic activity of neuronal nitric oxide synthase.||Promotes trans

back to the liver. Has a preference for plasma 16:0-18:2 or 18:O-18:2 phosphatidylcholines (Pu

DNA. Within the complex, may act as a scaffold.
involved in synaptic plasticity in neurons (By similarity). Involved in regulation of growth as eryt

of RNA polymerase II (RNAP II) (PubMed:9499409, PubMed:15563843). The activity of this co
y increasing its affinity for the 3'-OH end of the primer-template and stabilizes POLB to those s

0684968]).[supplied by OMIM, Mar 2008]
teins, is also proposed to be involved in neurite extension. Plays a role in intracellular vesicle t
ition and self-renewal through the regulation of specific genes involved brain development, incl
.1 (By similarity). FGL1 constitutes a major ligand of LAG3 and is responsible for LAG3 T-cell i

cylglycerol (PubMed:28851360).
wth factor-induced signaling. Alternative splicing results in multiple transcript variants encoding
IDS-sensitive inorganic and organic anion transporter that mediates the uptake of monovalent

h adiponectin. Required for normal revascularization after chronic ischemia caused by severing

ɘptor, thus promoting PKB/AKT1 phosphorylation and transduction of the insulin signal.
ignaling pathway downstream of the receptor is referred to as forward signaling while the signa

ion, LGALS3/galectin-3 down-regulation and NF-kappa-B inactivation. Regulates the redistribu

ɔnuclease III enzyme to produce an approximately 70-nt stem-loop precursor miRNA (pre-miRN

occur.||Plays a role in the establishment of the epidermal barrier on plantar skin.

gy (DH) domain functions as guanine nucleotide exchange factor (GEF) that modulates the GT

ates lymphocyte-epithelial cell adhesion (PubMed:11772392). Enhances dendritic cell immuno
athrin (clathrin-coated vesicles, CCVs) which are destined for fusion with the early endosome. T
ancreatic proteases. Also involved in the processing of various peptides including peptide horm

(PubMed:27745970). May suppress inflammation-related signaling in asthmatic airway epitheliu

RefSeq, Jul 2011]

d long-term establishment of adaptive and innate tissue-resident lymphocyte T cell types in no
of BCAR1 in response to integrin regulation. Activation of BMX by integrins is mediated by PT
promotes mitochondrial apoptosis through downstream BAX/BAK1 activation and positive reg
machinery (PubMed:25106868). Functions by recruiting the CCR4-NOT deadenylase complex

potentiated by tropomyosin. Interacts with actin, myosin, two molecules of tropomyosin and wi

-9. Involved in the cleavage of huntingtin. Triggers cell adhesion in sympathetic neurons throu
atory signal essential for T-cell receptor (TCR)-mediated T-cell activation. Its binding to DPP4 i

ction (PubMed:7528188). Engages, through its ectodomain, extracellular matrix components su
after their synthesis and directing transport of the complex from the endoplasmic reticulum to t

nchorage is affected, leading to modulate neural stem cell quiescence. CDH2 may be involved
sociates with alpha-catenin forming a link to the cytoskeleton (PubMed:10861224). Acts in con

d differentiation, the regulation of bone resorption, and is required for normal bone developmen

na membrane of leukocytes. The interaction between both receptors also mediates the activatic
7689, PubMed:12917691). Contributes to the regulation of cell survival, proliferation and differe
ulates myosin phosphorylation in both smooth muscle and non-muscle cells. In smooth muscle,
RNA, but not DNA polynucleotides. May play a role in RNA clearance at DNA double-strand br

embrane. Plays a role in maintaining the functional integrity of PKA-activated erythrocyte shap
pment. Involved in the guidance of commissural axons, that form a major interhemispheric con

ponses and regulation of innate and adaptive immunity, and acts as a sensor for proteolytic en
alpha-2-antiplasmin SERPINF2 and SPRY2 (PubMed:14751930, PubMed:16223769, PubMe

ss increases circulating satiety hormones and reduces appetite-stimulating neuropeptides and
es the cis-trans isomerization of proline imidic peptide bonds in oligopeptides.
and myogenesis. Isoform 6 accelerates muscle differentiation in vitro.

EGFC, and to a lesser degree VEGFA, thereby creating a positive feedback loop that enhance
wing PTK2/FAK1 phosphorylation, activation and targeting to focal adhesions. Involved in the r

005688).||Ligand for tyrosine-protein kinase receptors AXL, TYRO3 and MER whose signaling

action, transferring the gamma-glutamyl moiety to an acceptor amino acid to form a new gamm
GLRA1 are activated by lower glycine levels than homopentameric GLRA1 (PubMed:8717357
in soluble form to beta-hexosaminidase A for cleavage of N-acetyl-D-galactosamine and conve
bMed:15240885, PubMed:12515866, PubMed:16202387). GNA12-dependent Rho signaling s
e in cell division.
pled M2/M4 subtypes), dopamine receptors and opioid receptors. In addition to GPCRs, also p
repressor and coactivator of transcription, depending on the context (PubMed:24943844). Acts

sensus sequence 5'-[AC]A[AT]T[AG]TT[GT][AG][CT]T[CT]-3' (By similarity). Proposed to play a

rotecting cells against apoptosis (PubMed:14752510). Promotes apoptosis in response to cellu
dothelial cells (PubMed:19884327).

(PubMed:15123770).
ding is essential for NRG1-ERBB signaling (PubMed:20682778). ITGA6:ITGB4 binds to IGF1 a
ring cell growth. Antagonizes IRF1 transcriptional activation.

the cell rather than out of it. Their voltage dependence is regulated by the concentration of extr

orylation of pp125-FAK and p80, (3) differentiation of keratinocytes.

ath (PubMed:16286920). Also stimulates bactericidal activity in infected macrophages by causi

nethylated on 'Lys-9' (H3K9me3) or 'Lys-27' (H3K27me3) or when 'Lys-4' is monomethylated (H
affold protein upon which distinct protein complexes are assembled in the cytoplasm and in the

ral activity against papillomavirus infection. N-terminal region shows strong antifungal activity a

ion of FYN and PTK2/FAK1, and a transient increase in the intracellular calcium concentration.
/or growth factor-related transcription. Plays a critical role in the regulation of neuronal apoptos
es autophosphorylation of MET on its intracellular domain that provides docking sites for downs

r histone H3 that is crotonylated (PubMed:25417107, PubMed:27105114, PubMed:27545619,

ulator of a wide array of physiological functions including metabolism, sleep, body temperature,

lays also a role as an intracellular G protein-coupled receptor involved in melanosome biogene
and subsequent activation. Activation of RNase L leads to degradation of cellular as well as vi

a subsequent autophosphorylation on several serine and/or threonine residues. Phosphorylate
peptidyl alpha-hydroxylating monoxygenase (PHM) domain, is the copper-, ascorbate-, and O2
phosphodiesterase with a dual-specificity for the second messengers cAMP and cGMP, which
ıtrient levels and under starvation. Plays a role in the generation of reactive oxygen species.
› be targeted around migrating leukocytes (PubMed:19342684). Trans-homophilic interaction n
:tric distribution of phospholipids in the canicular membrane. May have a role in transport of bile

E-derived ASARM peptide (PubMed:18597632). Promotes dentin mineralization and renal pho

Facilitates the transfer of a spectrum of different lipid molecules, including diacylglycerol, phosp
ıd cell migration (PubMed:12198496).

oproteasome to cleave model peptides after hydrophobic and basic residues. Involved in the g
red for normal incorporation of laminin into the extracellular matrix, and thereby for normal cell-
e in osteoclast formation and function (By similarity).||Isoform 2 acts as a negative regulator of

e transection. Plays a role in stimulating neurite outgrowth in response to the heparan sulfate p

cytosis and growth signaling (PubMed:20005108). Key regulator of LPAR1 signaling and comp

recursor forms of BDNF (proBDNF) and NGFB (proNGFB). Also acts as a receptor for neurote

92674, PubMed:8195247). May be involved in the recruitment of monocytes into the arterial wa

in and mucosal surfaces under homeostatic and inflammatory conditions, as well as in patholo

o a second site (site 2) in integrins which is distinct from site 1 and enhances the binding of oth

phase and entry into the S phase (By similarity). In kidney development, inhibits tubule formatio

otrusions, such as filopodia, and for cell motility and migration (PubMed:20393565, PubMed:21

ditions (By similarity). Binds to the promoter region of the FOXP3 gene and promotes its transc

une response, cell adhesion, cell cycle progression, apoptosis, migration, and transformation. D

in invadopodial function (PubMed:19109420).||[Isoform 2]: May be involved in modulation of fc

tional auto-repression (By similarity).
osomial localization and activity of Rag GTPases and thereby regulates mTORC1 translocatior

nt DNA polymerase delta activity.

veral E3 ubiquitin-protein ligase complexes. May have E3 ubiquitin-protein ligase activity and pı

ubiquitinate histone H2A (PubMed:8132613).
ter leaflet of the membrane, linked to one of hundreds of different externally oriented oligosacc

rnatively spliced transcript variants encoding different isoforms have been found for this gene.
F), IL-1 and TLR4-induced NF-kappa-B activation in a dose-dependent manner. Overexpressic

evelopment (PubMed:28381549).
ar actomyosin retrograde flow that is crucial to cell protrusion and migration (PubMed:1885416
T-cell-mediated immune responses. Promotes axon growth in the embryonic olfactory bulb. Pr

ıave been observed for this gene. [provided by RefSeq, Feb 2011]
stimulates mRNA recruitment to the 43S PIC and scanning of the mRNA for AUG recognition. T

ess-induced apoptosis.

ıbMed:15695813). May play a possible role in cell cycle regulation by contributing to cell cycle

ıutophagic degradation of cytoplasmic ubiquitin-containing inclusions (p62 bodies, ALIS/aggres

:24788816). Recruits LRRK2 to the Golgi complex and stimulates LRRK2 kinase activity (PubM

:ell:APC contact site so as to promote the actin polymerization required for synapse induction c

ıtase (G6PC).

32 but not of TGFB3 (By similarity). May play a role in the regulation of alveolar generation.
ic membrane transport pathway. Believed to act as a component of the putative CORVET endc

jatively regulates B-cell antigen receptor (BCR) signaling.

/) (PubMed:19808672, PubMed:28402439). Part of multimeric repressive chromatin complexes
ming at sites of inflammation. Participates in biosynthesis of the SELL ligand sialyl 6-sulfo Lew

: synapses. Negatively regulates T cell activation by inhibiting the calcineurin-NFAT pathway. A
rticipate in ApoER2-specific reelin signaling. Directly, or indirectly, regulates GLUT2 gene expre

kic effect of amyloid-beta peptide.
ılved in the biogenesis of noncentrosomal, Golgi-associated microtubules through recruitment c
in cell migration (PubMed:20679435).

art of the ANK2/RABGAP1L complex which is required for the polarized recycling of fibronectir
ole as an adapter to assemble ZC3H12A, USP10 in a deubiquitination complex which plays a n
lity to induce apoptosis.
:tion through the activation of the JNK pathway in T-cells (By similarity).||[Isoform 2]: May be in

)211). Acts via MYD88 and TRAF6, leading to NF-kappa-B activation, cytokine secretion and th

sults in multiple transcript variants that encode the same protein. [provided by RefSeq, Oct 201

: degradation of protein aggregates by interacting with p62/SQSTM, ATG16L1 and LC3B/MAP1
ates platelet aggregation induced by PDPN (PubMed:18541721). Through MSN or EZR interac
criptional activation apparently by competing with other nuclear factors for binding to CBP. Also

ssing tumor suppressor genes such as BRCA1 and HOXA5 (PubMed:24952722). In contrast,

involved in enhancing the hepatic fatty acid oxidation in mitochondria (By similarity).

KIF5 to mitochondria for light chain-independent, anterograde transport of mitochondria (By si

ns destined for lysosomal degradation from carriers to be recycled to the plasma membrane (P

f adipocyte differentiation (By similarity). May bind to the 5'-GCCTGTCTTT-3' DNA sequence o

\RA to modulate lipid metabolism gene expression (By similarity). Is involved in adipocyte diffe

8, PubMed:28218735).

elomeric DNA persists at the fusion points) and did not perturb TRF2 telomeric localization (Pul
e of SHH, it is removed from primary cilia and is internalized into recycling endosomes, prevent
erol biosynthesis by increasing the transfer of squalene to a metabolic active pool in the cell.

ifferentiation and migration of neuronal cells during brain corticogenesis and for normal embryc
iction with UNC5D, and possibly also other UNC-5 family members. Plays a role in fibroblast gr

ı the N-terminal pyroglutamate formation of several amyloid-related plaque-forming peptides.
proteolytic processing of CLTRN in pancreatic beta cells (PubMed:21907142).

oside triphosphate (dNTPase) activity, which is required to restrict infection by viruses, such as
anonical Wnt signaling: required to deliver RECK-bound Wnt7 to frizzled by assembling a highe

face (PubMed:18270267, PubMed:25447204). With VAPA, may regulate ER morphology (Pub
formation, and engulfment of Salmonella.||Component of the autophagy machinery that contro

es their signaling activities. Negatively regulates mu-opioid receptor-mediated activation of the

are implicated in bone formation and bone disease, cancer and Alzheimer disease (By similarity

jers the cleavage of surface APP by beta-secretase to release sAPP-beta which is further clea

ıd activates BAX-dependent apoptosis coupled to activation of caspase-9, -2 and -3. Involved i

ł:21741933, PubMed:24120868, PubMed:27829214). DNA damage or perturbation of ribosom

lex with CYBA/p22phox. Regulates signaling cascades probably through phosphatases inhibiti

other cell types (By similarity). Plays a role in ciliogenesis.

ılated muscle differentiation, the regulation of AP-1 activity (plasma membrane bound AP-1 re
complex with SOX9 (By similarity). Plays a role in chondrocyte differentiation (By similarity).

ernalization of TNFR. Promotes degradation of EGFR after EGF signaling. Stimulates the GTP

r C16-C18 unsaturated fatty acids (By similarity). It was suggested that it may also stimulate fa

ıg additional GalNAc moieties. Some peptide transferase activity is however not excluded, con

ıositol 3-phosphate (PtdIns3P) through FYVE-type zinc finger (PubMed:11739631, PubMed:11

ıyloid-beta precursor protein). Specifically promotes the gamma-cleavage of APP CTF-alpha (a

3/TP53-mediated apoptosis in response to DNA damage via its effect on mTORC1 activity. Its
ylated HIFs are then targeted for proteasomal degradation via the von Hippel-Lindau ubiquitin

y a role in control of the ultradian cellular biological clock.

y 2013]

endent kinase inhibitor transcriptionally regulated by the p53/TP53 family to induce cell cycle a

telomerase-positive cell lines.

es the radial migration of cortical neurons via an RTN4R-LINGO1 containing receptor complex

lity to localize to the PM based on the level of membrane cholesterol (By similarity). In lipid-poo

distal end of the USP12 fingers domain and induces a cascade of structural changes leading

rocytes (PubMed:11975663). Involved in the response to oxidative stress by mediating deglyc

s a stable complex with the TGF-beta receptor-mediated phosphorylated SMAD2 and SMAD3

ɔn: aberrant BTB dimer are then ubiquitinated by the SCF(FBXL17) complex and degraded by t

ed:23933751, PubMed:24660806, PubMed:24751536, PubMed:24784582, PubMed:2489617[illegible]
ɔling the protein kinase C and calmodulin signal transduction systems (By similarity).

12A3, by phosphorylation which appears to prevent membrane trafficking of SLC12A3. Also inh

lycogen synthase and glycogen phosphorylase phosphatase activities of PP1. Dramatically incr
ıoinositol phosphates (phosphoinositol 3-phosphate, phosphoinositol 4-phosphate and phosph

veral ZFP36-associated mRNAs, such as TNF-alpha and GM-CSF, in response to stress (PubM

d fetus with a suitable immunological environment throughout pregnancy. Both isoform 1 and is
ı factors and minus-end stabilizers, thereby destabilizing microtubules at the adherens junction
ne pyrophosphate transporter in colon, may mediate the absorption of microbiota-generated th

PubMed:22084099, PubMed:23152791). Modulates also STAT3 activity through negative regu

ssential for a later stage in autophagosome maturation. Promotes primary ciliogenesis by remo
ɔmoting CDC42 activation at T cell leading edge membrane (PubMed:28028151). Is involved ir

. Represses Wnt/beta-catenin-stimulated transcription, probably by targeting CTNNB1 to prote

2125). Involved in translesion DNA synthesis in response to DNA damage via its interaction wit

slow endocytic recycling of endocytosed proteins back to the plasma membrane. May indirectly

multiple transcript variants encoding distinct proteins. [provided by RefSeq, Jul 2008]

may be involved in the development of the placenta in vivo. May also have a role in ovarian de

ve splicing results in multiple transcript variants encoding different isoforms. [provided by RefS
cing results in multiple transcript variants. [provided by RefSeq, Apr 2017]

ugh CACNA1E and stimulate programmed cell death (PubMed:15258581).

ıanging the internal signal transduction response of osteoblasts to external growth factors.

ed, but not its deubiquitinating activity (PubMed:17141156). Together with NSFL1C/p47, regula

on of receptor tyrosine kinase-coupled cellular responses induced by cell adhesion, growth fact

; ubiquitin-mediated proteasomal degradation (PubMed:24130170). Inhibits PPP1CA phosphat

sphorylates inositol 1,3,4,6-tetrakisphosphate (Ins(1,3,4,6)P4) (PubMed:12223481). Phosphory

ion and a cytoplasmic domain. Alternative splicing results in multiple transcript variants encodi
enhances KSR2-mediated phosphorylation of MAP2K1 (in vitro). Blocks MAP3K8 kinase activ
by RefSeq, Dec 2016]
ifferentiation (By similarity).

tion, apoptosis and differentiation (PubMed:20207939). Has a better catalytic efficiency toward

oitin sulfate attached to the Ser/Thr- and Gly/Ser-rich regions. Also involved in modulation of g

ich formation); ANXA1 (involved in membrane anchoring); DBN1, DBNL, CTTN, RAPH1 and E
nteraction with MICALL2. Links MICALL2 to the actin cytoskeleton and recruits it to the tight jur

displays broad nucleoside diphosphate kinase activity (PubMed:19073142, PubMed:1976673
of East Asians have the cytosolic isozyme but not the mitochondrial isozyme. A remarkably hi

3ACE1 trafficking and the control of amyloid-beta production (PubMed:27179792). In neuronal

ıbMed:22848640).

ɜd on 'Tyr-142' (H2AXY142ph) at double-strand breaks (DSBs), recruiting other pro-apoptosis t
n fibroblasts (By similarity).
ɜtal-catalyzed oxidation of lipoproteins. APP42-beta may activate mononuclear phagocytes in tl

ound GTP.
ɔlay a role in the assembly of the V-type ATPase complex. In aerobic conditions, involved in int
rating H3.3-containing nucleosomes. Catalytic component of the chromatin remodeling comple
: is required for its tumor suppressor function. Also forms a heterodimer with CSTF1/CSTF-50 t

ı those of BCL7B and BCL7C. Two transcript variants encoding different isoforms have been fo

ɜs neuronal growth cone collapse (By similarity).||Important signaling molecule that activates si

erythrocyte cytoskeleton required for the merozoite invasion (PubMed:28409866).||[Isoform 1]
In either case, their spread as cell-free virions is restricted. Its target viruses belong to diverse t

ty of RUNX. The heterodimers bind to the core site of a number of enhancers and promoters, ir
esion, enhances superantigen-dependent T-cell-mediated proliferation and costimulates T-cell

l differentiation, but is required for the proliferation of specific cell types (e.g. erythroid and hem
action of MDM2 by blocking MDM2-induced degradation of p53 and enhancing p53-dependent

bMed:12047389, PubMed:17412999, PubMed:17407782). Does not require ATP (PubMed:11
al fragment comprising the NC1 domain, inhibits aortic endothelial cell proliferation and causes
gene. [provided by RefSeq, Jul 2008]

e of cadherins to the actin cytoskeleton at adherens junctions. In contrast, cortical actin was fo

ity). Involved in memory-related synaptic plasticity in the hippocampus (By similarity).

A during double-strand break (DSB) repair: recruited by BLM and mediates the cleavage of 5'-s
enhanced by ELMO1 (PubMed:8657152).
e-epithelial cell adhesion. In association with FAP is involved in the pericellular proteolysis of th

gene, the renin/REN gene and the oxytocin-neurophysin/OXT gene. Represses the triiodothyro

erentially uses myristate, laurate, arachidonate and eicosapentaenoate as substrates. Both iso
secretion by regulating the levels of unesterified EETs (By similarity). Modulates prostaglandi

Also involved in the long patch base excision repair (LP-BER) pathway, by cleaving within the a
stem. Phosphorylates PLCG1, FRS2, GAB1 and SHB. Ligand binding leads to the activation o

Can promote endothelial cell proliferation, survival and angiogenesis in adulthood. Its function i

rofile, interacting with inflammatory chemokines of both the CXC and the CC subfamilies but nc

ed by RefSeq, Jul 2008]

e receptor expressed on fibroblasts, smooth muscle cells, chondroblasts, leukocytes, and certa

by RefSeq, Jul 2008]
d cell proliferation (PubMed:28541286, PubMed:28073925, PubMed:18378771, PubMed:2845

ority of its substrates. In skeletal muscle, contributes to insulin regulation of glycogen synthesis

. Acts as a coregulator for USF1 by binding independently two promoter elements, a pyrimidine
ong the enzymes involved in this pathway, the trifunctional enzyme exhibits specificity for long-c
volved in this pathway, the trifunctional enzyme exhibits specificity for long-chain fatty acids (Pu
their function. Component of the BRG1-RB1-HDAC1 complex, which negatively regulates the (

cularization, tumor angiogenesis and pathophysiology of ischemic disease. Heterodimerizes wi
harged Asp or Glu residue at position 3 and a Tyr anchor residue at the C-terminus (PubMed:1
l gag-pol (TPQDLNTML) and Nef (RPQVPLRPM) (PubMed:25808313). Displays self-peptides

a neurotransmitter.
tides (PubMed:8264621). May play a role in the early steps of spliceosome assembly and pre-r

s, such as adenylate cyclase. Signaling inhibits adenylate cyclase activity. Arrestin family memb
by inhibiting skeletal muscle and cardiac myocyte differentiation and promoting muscle precur

e phosphorylation of a number of proteins including JAKs, TYK2, STAT proteins and the IFNR

nds IGF2. Acts as a positive regulator of T-cell coactivation, by binding DPP4.

-mediated cell proliferation and IL5 up-regulation by T-cells.
; and natural killer (NK) cells (Probable) (PubMed:10653850).

; in embryonic stem cells. Does not have histone demethylase activity but regulates activity of \
ations and at neutral pH, but becomes permeable to Na(+) at subphysiological K(+) levels and \

led:15660133, PubMed:16230460, PubMed:18028908, PubMed:22328514, PubMed:2363367

ell surfaces in a receptor-independent manner. Zona pellucida-binding protein which may play a
)395745, PubMed:30140003). Catalyzes an important step in the biosynthesis of branched, cor

retic factor (ANF) (PubMed:2531377, PubMed:2972276). Displays UV-inducible elastase activi
ı. Ligand for integrinv/beta3 on the surface of blood vessels.||Ubiquitinous metalloproteinase th

synaptic modulator, signaling molecule, smooth muscle contractor and neuroprotectant. Its prc

n is mechanically antagonized by MYH9.

uired for normal, polarized cilia organization in brain ependymal epithelial cells (By similarity).
cular membrane trafficking and cell migration (By similarity). Required for the structural integrity

5, RELB, NFKB1/p105, NFKB1/p50, REL and NFKB2/p52. The dimers bind at kappa-B sites in
itiation, proliferation and apoptotic programs (By similarity). Involved in bone remodeling and hc

the activation of the transcription factor RUNX2. Regulates remodeling of the extracellular mat

of MEK phosphorylation.||HCNP may be involved in the function of the presynaptic cholinergic
.

units and are essential for the stability of the intermolecular collagen cross-links (Probable).
f infectious virus particles. The sumoylated isoform PML-4 restricts rabies virus by inhibiting vir

transcription activator for the ACOX1 and P450 genes. Transactivation activity requires heterc
e to increased Ca(2+) levels, dephosphorylates and activates phosphatase SSH1 which result
bunit POLA2 and two primase subunits, the catalytic subunit PRIM1 and the regulatory subuni
horylation of metabolic enzymes, and by longer-term effects via phosphorylation of transcriptio

ade involving MAPK1/3 (ERK1/2) and RAP1GAP. Involved in cell proliferation and cell growth
enesis. Also involved in angiogenesis through stimulation of endothelial cell proliferation, migra
ciation with GPC1 (via its heparan sulfate chains) targets PRNP to lipid rafts. Also provides Cu

ariety of signaling and cytoskeletal proteins. This gene is highly expressed in the spinal cord. S
ensity of FGF signals in the extracellular space. Regulates the availability of insulin-like growth
2.1.46). Saposin-C apparently acts by combining with the enzyme and acidic lipid to form an a
ar membrane. May function in the cytoplasmic partitioning of proteins. The holoprotein functio

so acts as a lipid phosphatase, removing the phosphate in the D3 position of the inositol ring fr

the actin cytoskeleton, formation and disassembly of focal adhesions and cell protrusions, cell
ppo pathway, in a cell density-dependent manner. May function as a tumor suppressor.

gher than PLA2 activity (PubMed:19615464). Shows O-acyltransferase activity, catalyzing the t
classical non-homologous end-joining (NHEJ). Functions downstream of the MRN complex and

nvolved in ciliary assembly (DYNC2LI1, FOXJ1 and BBS4) and genes involved in ciliary motility

c function of BAD and DAPK1 (PubMed:9770464, PubMed:16223362, PubMed:17360704, Pu

n SLC3A2 functions as sodium-independent, high-affinity transporter that mediates uptake of la
ease of cytoplasmic Ca(2+) levels due to release of Ca(2+) stores from the endoplasmic reticul

plex (PubMed:10078207, PubMed:29374058). Belongs to the neural progenitors-specific chrom

ze and associate with ISGF3G/IRF-9 to form a complex termed ISGF3 transcription factor, that
s like ethinylestradiol, equalenin, diethyl stilbesterol and 1-naphthol at significantly lower efficie
quire additional evidences in vivo.
hormone receptor, MUC1 and the amyloid precursor protein (PubMed:12441351). Acts as an a
of transcription repressor complexes, thereby allowing cofactor exchange (PubMed:21240272).

ns of H(2)O(2).
n cell proliferation and apoptosis. Blocks adipocyte differentiation by repressing CEBPA binding
ematopoietic cells, control of mesenchymal cell proliferation and differentiation, wound healing,
embrane domains of epithelial and endothelial cells. Necessary for lumenogenesis, and particu
known to complex with integrins. This gene is expressed in different carcinomas. The use of a
gonizing its biological activity.

y stimulation of BAX and FAS antigen expression, or by repression of Bcl-2 expression. Its pro-

NA.

5-methylcytidine, and N(4)-anisoylcytidine.

ś an adapter for recruiting WASL to vaccinia virus.
ect on transcription regulation is depending upon the context in which it binds and diverse mech

nfection (PubMed:17392790, PubMed:23950712). Mediates 'Lys-63'-linked polyubiquitination o

. LRP8 is thus a key component of the Reelin pathway which governs neuronal layering of the

se) polymerase (PARP) cleavage. Has no hyaluronidase activity in embryonic fibroblasts in vitr

nel closure via a mechanism that does not involve physical obstruction of the channel pore (Pul
(HIV-1) and hepatitis C virus (HCV). Can inhibit: influenza virus hemagglutinin protein-mediate

ted activation of the G-proteins (By similarity).
ncellularly (PubMed:16467304). Also acts on N-oleoyl ethanolamine phosphate/N-(9Z-octadece
nucleotides (PubMed:31778653). In turn, RNase T2 degradation products promote the RNA-de

e (4-dione) to testosterone.
SVZ neuroblasts survival and ependymal wall integrity. May also mediate local repair of brain v
2 key components of the beta-catenin destruction complex: poly-ADP-ribosylated target protein

face expression via ERK1/2 inactivation. Cleaves E-cadherin in response to growth factor depr

ation of target proteins.

S is the sole source of sulfate; APS appears to be only an intermediate in the sulfate-activation

n target of Notch signaling pathway during eye development.

volved in regulation of cortical development, specifically in maintenance of the pial basement n

nctional preinitiation complex with RNA polymerase II and the general transcription factors.
as an inhibitor of the amyloid-beta peptide aggregation and fibrils deposition. Plays a role in th

nt HCO3(-) cotransport. May be regulated by osmolarity.
rain more efficiently than tau from Alzheimer disease brain.
membrane of the endosome and mostly are delivered to lysosomes enabling degradation of m
ly regulates androgen receptor signaling and androgen-induced cell proliferation (PubMed:208
erve growth factor and serum: mediates oxidation and subsequent depolymerization of nuclear

l signaling. Shows weak activity on HRAS. It is controversial whether RAPGEF2 binds cAMP a
us of TM4SF20 into the ER lumen, may act as a ceramide sensor for regulated alternative tran
ing the switch from proliferative to differentiating progenitors. Beyond progenitor cells, promote

A damage caused by ionizing radiation, which subsequently improves cell survival by decreasi

ubiquitin ligase complex may not lead to its degradation by the proteasome.

n of myocyte enhancer MEF2C. During muscle differentiation, it shuttles into the cytoplasm, all
nanner and hence acts as a co-chaperone of HSP70. Also reduces cellular toxicity and caspas

es the protein phosphatase SSH1, leading to increased inhibitory phosphorylation of the actin b

A-mediated synapses (By similarity). In collaboration with UNC13A, facilitates neuronal dense c
cular development and remodeling (PubMed:10428823). May act as an adapter that mediates

(HIV-1) and vesicular stomatitis virus (VSV). Can inhibit: influenza virus hemagglutinin protein-
ytoplasmic deadenylation/translational and decay interplay of the FOS mRNA mediated by the

PK) trimer, including that consisting of PRKAA1, PRKAB1 and PRKAG1. This phosphorylation
n RRAGC or RRAGD and cycles between an inactive GDP-bound and an active GTP-bound fo
rotein signaling initiated by D(2) dopamine receptors (PubMed:27677260). May play an importa

odorant molecules from the sensory epithelium.

script of this gene was found to be expressed constantly during cell cycle progression. [provided

e involved in growth factor signaling.

ing autophagosomes (PubMed:28561066). Probably recruited to membranes through its PtdIns

ing that, under physiological conditions, it is not the main contributor in N-glycan biosynthesis.

avage by gamma-secretase, most probably through stabilization of the direct interaction betwee

onal regulation of T lymphocyte development (By similarity).

nigration and differentiation before synapse formation, and non-synaptic vesicular release of n

d induction (PubMed:17116691).
le transcript variants encoding distinct isoforms. [provided by RefSeq, Jul 2014]

le in axon outgrowth. Binds to F-actin but not to RNA. Part of the WAVE complex that regulate
its C-terminus, which occupies the same binding sites as the proteasomal ATPases, opening t

of the activity of N-type voltage-gated calcium channel (By similarity).

embles into the GAIT complex which binds to stem loop-containing GAIT elements in the 3'-UT
the vimentin intermediate filaments (By similarity). Plays a role in endoplasmic reticulum-assoc
toskeletal polarity in the junction between T-cell and antigen-presenting cell (By similarity). Ma
nt transcription.
equent phosphorylation of CFL1 which is important for lamellipodial F-actin regulation.

d:28128235). Involved in the regulation of endosomal protein sorting (PubMed:29368044).

receptor gene (PubMed:8932385, PubMed:16549056). May be a transcriptional enhancer in th

is the case for the cyclins CCND2 and CCND3 which polyubiquitination and subsequent degr

the PRC2 complex that mediates H3K27me3 methylation, followed by de novo silencing. Recr

72988). Mediates ubiquitination and degradation of IRS1 in a mTOR-dependent manner: the C

29). During G1 phase, plays a role as substrate of APC-FZR1 complex E3 ligase (PubMed:298

ibitor of monocyte chemotaxis. Can inhibit the growth or viability of normal cells but not transfo

ımotes ESC self-renewal and pluripotency (By similarity).||Transcriptional repressor (PubMed:1

plays a role in the efficiency of mRNA splicing and RNA processing (PubMed:27602518). Plays

di-O-sulfated GalNAc when chondroitin sulfate C is used as an acceptor.

naling (PubMed:23545497). Interferes with the TNFalpha-triggered NF-kappa-B pathway by in

15528276). Can suppress brain tumor angiogenesis through transcriptional repression of REL

ne and facilitates the recruitment of eIF-1, eIF-1A, eIF-2:GTP:methionyl-tRNAi and eIF-5 to for
hosphorylation of metabolic enzymes, and by longer-term effects via phosphorylation of transc

f intestinal cholesterol absorption. May act as a scaffold protein by regulating NPC1L1 transpo
iption under hypoxia conditions (4% oxygen), as well as normoxia conditions (20% oxygen) (Pu

appropriate Ca(2+) levels, and thus preventing the overload of the cell with excessive Ca(2+) i
ading to methylation of PPARG target promoters at histone H3K9 and transcriptional silencing.

annealing (SDSA) as well as disassembly of D loop recombination intermediates. Also disasse
esidue Leu and peptides with a hydrophobic C-terminus, while it has weak activity toward pepti
lum (ER) stress and the subsequent induction of unfolded protein response (UPR) (PubMed:27
genes enhancer. Acts cooperatively with EYA proteins to transactivate their target genes throu
dephosphorylation in the cytoplasm causes export complex disassembly. It is recycled back to

(PubMed:10748143). Globoside Gb3Cer is a glycosphingolipid of the globo serie, one of the m
in the uterus. Required for normal response to progesterone in the uterus and for fertility. Med

all nuclear RNA to form the core snRNP. In the cytosol, the Sm proteins SNRPD1, SNRPD2, S
somal storage function. [provided by RefSeq, Oct 2008]

ro-Hyp sequences where Hyp is 4-hydroxyproline (PubMed:18487197). Has no activity on subs
in mitochondrion, possibly by regulating ATP production and mitochondrial translation (PubMe
equired for UBD/FAT10 conjugation. Isoform 2 may play a key role in ubiquitin system and may

gainst dengue virus (DENV) and can inhibit the replication of all DENV serotypes. May block th
is the first committed step in the generation of phosphatidylinositol 4,5-bisphosphate (PIP2), a

brane cargo proteins into the lysosomal degradation pathway. The recruitment of the CSC to th
sist in binding of the phospholipid substrate. Required for the proper folding, assembly and ER

llactosamine oligomer with a lower efficiency. Has weak or no activity toward keratan sulfate ar
ar transport (By similarity). Required for asymmetric spindle positioning, asymmetric oocyte divi

lysophosphatidylcholine, lysophosphatidylinositol and lysophosphatidylserine using C18:1 or C

ulator of a wide array of physiological functions including metabolism, sleep, body temperature,
a-1 (ITGA5:ITGB1) (PubMed:28892079). The recruitment of the retriever complex to the endos
radation of membrane proteins, such as stimulated growth factor receptors, lysosomal enzymes

to its subsequent proteasome-dependent degradation; preferentially recognizes and mediates

the endoplasmic reticulum-associated protein degradation pathway of these proteins plays a c
e affinity of the SKA1 complex for microtubules and may allow the NDC80 complex to track dep
lex. Promotes the localization of SACM1L at endoplasmic reticulum-plasma membrane contac

nor cell migration.

can bind hemimethylated DNA but with a lower affinity compared to methylated DNA (PubMed:
mes and lysosomes, where TLR9 is located (PubMed:29130391).
les (PubMed:14594945, PubMed:25123532). Required for the initiation of axoneme extension

2. The released N-terminal cytosolic domain is translocated to the nucleus to effect transcriptic

168031). Shows a greater preference for D-erythro isomer of ceramides (PubMed:16269826). I
PubMed:29911972). The CTLH E3 ubiquitin-protein ligase complex is not required for the degra

peptide Pro-Gly-Leu-Trp (PubMed:29632410). Binds peptides with an N-terminal sequence of t

the PER and CRY transcriptional repressors (PER/CRY loop). Both these loops are interlocked

lative stress.

by repressing the expression of E2F1, thereby preventing p53/TP53-dependent apoptosis. Pla

ty acids (PubMed:15610069, PubMed:15907797). Involved in neuronal cell proliferation and di

ndrial function and cell survival, notably in cardiomyocytes (By similarity). Regulates rates of gl
ability, and inhibition of hematopoietic differentiation (By similarity).

ed:22065575, PubMed:23625927). Core component of the CTLH E3 ubiquitin-protein ligase co

protein-coupled receptors and phosphoinositide 3-kinase.
at the cell membrane (PubMed:18957440). Isoform 4 retains KCND3 in the endoplasmic reticu

g (By similarity). Inhibits VEGFA-induced endothelial cell proliferation and migration. Seems to

tumors to attenuate anti-tumor immunity and facilitate tumor survival (PubMed:30487606). May

embryonic development.

(5)Cer), respectively. Probably plays a central role in regulating neolacto-series glycolipid synth

acrophage production of inflammatory cytokines, probably through suppression of the MAPK si

bly by regulating the localization of the MNR complex, indirectly regulates the recruitment of dc

ate (PIP) and ARF1 are essential for the GlcCer transfer ability. Also required for primary ciliun
from intracellular stores. Required for acrosome reaction in sperm during fertilization, probably

onical Wnt/beta-catenin-dependent pathway and non-canonical Wnt signaling by acting as an i

axons: degradation of AKT1 prevents AKT1-mediated phosphorylation of GSK3B, leading to G
428259). Involved in the early steps in cilia formation by recruiting the ciliary vesicles (CVs) to t

on of TRAF6, which leads to the activation of TAK1 and IKK through a proteasome-independen

the subsequent action of clathrin and adaptors in the formation of clathrin-coated vesicles. Inv

T5, B4GAT1 and B4GALT1 (PubMed:25354954, PubMed:25827571). Catalyzes the intramem

epimerase) (PubMed:30451973). Involved in the maintenance of the equilibrium between the b

branched and unbranched actin filaments. Activates the Arp2/3 complex to induce branched

9303). Distinct protein-interaction motifs allow recruitment of the effector proteins TBK1, TRAF

(PubMed:28295037).

of cofilin (PubMed:18216281). Inhibits TGFB-induced epithelial-to-mesenchymal transition in le

le-strand viral DNA, leading to G-to-A hypermutations in the subsequent plus-strand viral DNA.

human centrosome. Alternative splicing results in multiple transcript variants. [provided by Ref

dent stability of BAX, a pro-apoptotic protein, ultimately leading to protection of cell death; But,
n as APP and PRNP; this then modulates the secretion of APP and PRNP peptides. Promotes
phosphoglucomutase activities.

use the hydrated form of 3-oxoalanine as a catalytic nucleophile (PubMed:12757706, PubMed

of RAB43 is activated by USP6NL. Involved in retrograde transport from the endocytic pathway

ıse to low amino acid availability (PubMed:25329545). Plays a role as an activator of the integı

ıesis involved in maintenance of proper somite segmentation and proper numbers of somites a

s essential for a later stage in autophagosome maturation (By similarity).

nd dimethylated arginine residues of histones H2, H3 and H4 (PubMed:28847961). After initial
ɔnuclease III enzyme to produce an approximately 70-nt stem-loop precursor miRNA (pre-miRN

ȷene coding sequence. [provided by RefSeq, Dec 2010]

gland, wound healing, extracellular matrix production, immunosuppression and carcinogenesis

ɔMed:15048703, PubMed:16352806, PubMed:23169771, PubMed:24945728). Promotes T-cel

ul 2008]
ıin induces vascularization of normal and malignant tissues. Angiogenic activity is regulated by
ɔunit NR2B along microtubules (By similarity).

se range of cellular pathways such as DNA damage repair, ubiquitination and transcriptional reg
LB2-scaffolded HR complex containing RAD51C and which is thought to play a role in DNA rep

ses a requirement for CD28 costimulation for proliferation and IL-2 production. Also acts by pron

and chemotherapeutic agents (PubMed:12808445).

on and guide them through the mitochondrial intermembrane space. The TIMM8-TIMM13 comp

a direct regulator of actin dynamics by binding actin filaments and has both barbed-end actin fil

by RefSeq, Apr 2015]

ifications of histones, also called histone code, and nucleosome remodeling. Required for chec

flexibility by looping thus providing a mechanism to promote activities on various gene promote

lopment. IGF-II is influenced by placental lactogen. Also involved in tissue differentiation. Posit
NA-binding protein specific to 5'-phosphorylated single-stranded guanine-rich sequence related
on their subunit composition. Inhibins appear to oppose the functions of activins.
moters (PubMed:11846977, PubMed:16846591, PubMed:16979567, PubMed:20049431). Acts
of the channel. Acts as a negative regulator of smooth muscle contraction by enhancing the cal

ıding site of the Walker A box of the adjacent subunit. The six ATPase active sites, however, a
ıding site of the Walker A box of the adjacent subunit. The six ATPase active sites, however, a
ıding site of the Walker A box of the adjacent subunit. The six ATPase active sites, however, a

collagen by melanoma cells.
ılfur is then transferred to precursor Z to form molybdopterin.||Catalytic subunit of the molybdo[illegible]

ough the activation of platelets.
hat are crucial for the saltatory propagation of action potentials along myelinated axons. During

:y at the centrosome.

ble for decreased drug accumulation in multidrug-resistant cells (PubMed:2897240, PubMed:9[illegible]

the genesis and progression of lung carcinoma. Acts as an inhibitor of PPP1C.

sense RNA species and promoter-upstream transcripts (PROMPTs), and of mRNAs with proce
ıuclear and cytoplasmic associated factors that join the complex only transiently either during E

t of a subcomplex with RPB4 that binds to a pocket formed by RPB1, RPB2 and RPB6 at the b
ɔle in the maintenance of protein homeostasis by removing misfolded or damaged proteins that
ed:15871698). The 9-1-1 complex stimulates DNA polymerase beta (POLB) activity by increasi[illegible]

/led:9430682). In the cellular response to DNA damage, the RPA complex controls DNA repair
ıd to zinc, can enhance the tumor suppressor activity of Ras-related protein 1A (KREV1). It is l

/IN exon 7 RNA. Binds to pre-mRNA.
+)/H(+) exchange and nutrient transport, insulin-dependent salt sensitivity of blood pressure, sa
s an important role in the regulation of centriole duplication. Required for the onset of procentri
quires CKS1. Recognizes target proteins ORC1, CDT1, RBL2, KMT2A/MLL1, CDK9, RAG2, F(
with the counter-transport of one K(+) ion (PubMed:7521911, PubMed:8857541, PubMed:266
tral amino acids, including glutamine, asparagine, and branched-chain and aromatic amino aci
tial for the enhancement of ATP-induced exocytosis (PubMed:30404828). Acts also as a molec

l:28781166, PubMed:28076346). Component of both the pre-catalytic spliceosome B complex
SUN3 the nuclear envelope. Involved in maintenance of the nuclear envelope integrity. May as

; vesicles. The pairing of the three SNAREs from the N-terminal SNARE motifs to the C-termina
ıe establishment of constitutive heterochromatin at pericentric and telomere regions. H3 'Lys-9

; (By similarity). Required for the development of natural killer receptor-positive lymphoid tissue

ıitate, and Ni(2+). Responses triggered by Ni(2+) require non-conserved histidines and are, the
e control of initiation of DNA replication through its interaction with NAKAP95.||Thymopoietin (T
ate and the expulsion of a 3'-OH DNA strand. The free DNA strand then undergoes passage a
-beta and retinoic acid.

/led:10751406, PubMed:11279239, PubMed:12115244, PubMed:12748283, PubMed:151871(

)H; CBR4 binds NADPD (PubMed:25203508).

diacetylated at 'Lys-14' and 'Lys-27' (H3K14ac and H3K27ac) (PubMed:29437725, PubMed:30
ed:25998567, PubMed:30867591). Functions as an amino acid exchanger (PubMed:11557028

d nucleosome remodeling.

d:17379599). Also acts on N-oleoyl ethanolamine phosphate/N-(9Z-octadecenoyl)-ethanolamin

milarity).

: this complex regulates DNA replication processes under both normal and stress conditions, st

23082202).

glutamylation of CGAS, leading to impair the nucleotidyltransferase activity of CGAS.

by effecting the fusion of recycling endosomes to basal bodies during cilia formation (By simila

ctivity of the myosin protein phosphatase 1 (PP1) complex. Acts by mediating phosphorylation o
r folding of newly synthesized PIKKs. Promotes assembly, stabilizes and maintains the activity

10, PubMed:19112177, PubMed:19679664, PubMed:23455478, PubMed:27565346, PubMed:2
20385). In addition to histones, deacetylates other proteins: plays a central role in microtubule-
o complete the histone octamer. It may play a role in heterochromatin maintenance in proliferati

ats of two similar hexanuclotide half-sites spaced by four nucleotides (By similarity). Plays an im

Ias also weak lysophosphatidylethanolamine acyltransferase activity (LPEAT activity). Favors p
and interleukin-3 (IL-3) receptors. Thus, through maintaining basal levels of cytokine receptors,

63'-linked polyubiquitin chains.
unit biogenesis independent on its methyltransferase activity, facilitating the incorporation of rit
OG/clathrin complex is required for the maintenance of kinetochore fiber tension (PubMed:212

ore attachments. May contribute to the regulation of separase activity. May regulate AURKA lo
C regulatory network to orchestrate gene expression programs to control embryonic stem cell s

activity. Stabilizes the Pol-delta complex and plays a major role in Pol-delta stimulation by PC
ntrioles adjoining each parental centriole during S phase. Phosphorylates 'Ser-151' of FBXW5
yndrome. Alternate splicing results in multiple transcript variants. [provided by RefSeq, Feb 201

ed:31767635, PubMed:31827282). Some complexes also catalyze acetylation of histone H4 at

ERN1/IRE1-mediated unfolded protein response (UPR) by mediating AMPylation or de-AMPy
d may function in a chaperone-like manner, facilitating the efficient association of RNA proces

sense RNA species and promoter-upstream transcripts (PROMPTs), and of mRNAs with proce

n. In the adult, Dkks are implicated in bone formation and bone disease, cancer and Alzheimer

l. Recruits TP53BP1 to DNA damage foci and, at least in particular repair processes, effective

nsmission of mechanical forces across the nuclear envelope and in nuclear movement and pos

ay an essential role to ensure accurate mitotic chromosome condensation in neuron stem cells
sense RNA species and promoter-upstream transcripts (PROMPTs), and of mRNAs with proce
na. Acts as a transcriptional corepressor via its interaction with the NFKB1 NF-kappa-B subunit

971). Receptor for IL17C as part of a heterodimeric complex with IL17RE (PubMed:21993848)
te the paracellular transport of small monovalent cations along a concentration gradient, due to

nescence-associated cell cycle exit.

sponsiveness of the spindle assembly checkpoint. Also required for degradation of CDC20.

itochondria to cytoplasm and its translocation to the nucleus; however, the involvement of casp
aggregation and guide them through the mitochondrial intermembrane space (By similarity).

-nitrophenol and thyroid hormones, including 3,3'-diiodothyronine, triidothyronine, reverse triiod
d:25620095). May play a role as a regulator of cellular cholesterol homeostasis (PubMed:1958[illegible]

omes is that the latter contain a 5S rRNA. Among different species, the proteins comprising the
]

omes is that the latter contain a 5S rRNA. Among different species, the proteins comprising the

embly intermediates in a process that promotes incorporation of early nuclear-encoded subuni

P-bound form to be efficiently methylated (PubMed:18539146, PubMed:20606008). Also acts a
iding directionality for type II topoisomerase-mediated strand exchanges toward chromatid dec
been found for this gene. [provided by RefSeq, Nov 2008]
ble-strand DNA. This complex stimulates both synaptic and presynaptic critical steps in RAD51

ough dephosphorylation of signaling molecules. Receptor for PIANP.

ty).
8). TPRKB acts as an allosteric effector that regulates the t(6)A activity of the complex. TPRKE
nctional preinitiation complex with RNA polymerase II and the general transcription factors.

omes is that the latter contain a 5S rRNA. Among different species, the proteins comprising the

s SCO1, SCO2 and COA6 (PubMed:29381136). Probably facilitates MT-CO2/COX2 associatio

rization and cell adhesion. Plays a role in angiogenesis.

RNA); however interaction is probably mediated via its interaction with NCBP1/CBP80 compon

role in intraflagellar transport is mainly seen in tissues rich in ciliated cells such as kidney and t
non-self dsDNA that serves as template for transcription into dsRNA. The non-self RNA polyme

apoptotic members of the Bcl-2 family leads to altered mitochondrial membrane permeability re

vith channel maturation through recruitment of NEDD4L to the Golgi apparatus and multivesicul
positive modulator of classical Wnt signaling, may play a crucial role in ciliary signaling during c

ATP-dependent DNA translocase (PubMed:23973328, PubMed:28977671). Can promote Hol

ss conditions, stabilizes replication forks and influences both CHEK1 phosphorylation and the i

om the mitotic spindle poles may allow depolymerization of the microtubule end proximal to the

recruitment of CENP-O complex (which comprises CENPO, CENPP, CENPQ and CENPU), C

NA). Participates in S phase and G2 phase checkpoint activation upon DNA damage.

ed:19536135). Involved in centriole duplication. Required for CDK5RAP22, CEP152, WDR62 a
to prevent photoreceptor degeneration (By similarity).
om the distal ends of centrioles (PubMed:15047868, PubMed:27219064, PubMed:27306797). I

entration, translocates from the endoplasmic reticulum to the plasma membrane where it proba
e elongating form of RNA polymerase II (RNA pol IIo) and facilitates its ubiquitination at UV dar

ı of the pre-replication complex (pre-RC). This may indicate a role for this protein as a sensor w

re.

ed gene silencing in regulatory T-cells (Treg) via recruitment of corepressor CTBP1 (By similar
hatidylethanolamine (PE). This step is required for the membrane association of ATG8-like prc

ugh an interaction with pre-rRNA primary transcripts. Alternative splicing has been observed at

ng homologous recombination repair (PubMed:29042561). Inhibits non-homologous end joining

ynthesis of unsaturated C16 long chain fatty acids and, to a lesser extent, C18:0 and those witl

genous and exogenous alpha-dicarbonyl compounds and xenobiotic alicyclic ketones.

pendent manner to a region near the 5' end of the mRNA where it functions in mRNA export to

dently of the MLL2/MLL3 complex (PubMed:19124460). However, its function in DNA damage

the mitotic kinetochores. A scaffold protein responsible for the initial recruitment and maintenar

atin at pericentric and telomere regions. H3 'Lys-9' trimethylation is also required to direct DNA

all nuclear RNA to form the core snRNP. In the cytosol, the Sm proteins SNRPD1, SNRPD2, S
all nuclear RNA to form the core snRNP. In the cytosol, the Sm proteins SNRPD1, SNRPD2, S

24065767). May also promote acetylation of nucleosomal histone H4 by KAT5 (PubMed:15502

e (PubMed:24463511). Inhibits activity of protein phosphatase 2A (PP2A). Does not inhibit pro

ivity towards bile acids or steroid sulfates (including taurocholate, cholate, chenodeoxycholate,

). May play a role in chromosome segregation through establishment of sister chromatid cohes
ydrate structure present in alpha-dystroglycan (DAG1), which is required for binding laminin G

ʾubMed:25931565). Promotes the recruitment of SLF2 and the SMC5-SMC6 complex to DNA

ytochrome b translation and/or stability.

ated during DNA repair and recombination. Has endonuclease activity towards branched DNA
rocessing and modification, leading to remodel the translational program of differentiating cells

centriolar proteins, such as POC1B, POC5 and CEP135, into the distal portion of centrioles (Pu

)2793, PubMed:31693891). Aryl sulfonamide anticancer drugs change the substrate specificity
nctional preinitiation complex with RNA polymerase II and the general transcription factors.

naling events in the cytoplasm (By similarity).

ɔMed:30456879, PubMed:29131025).

nic reticulum has failed. May therefore function in the endoplasmic reticulum stress-induced pr[illegible]

ıbules and may allow the NDC80 complex to track depolymerizing microtubules (PubMed:2308[illegible]

ıtion. Due to its presence at higher levels in tumor endothelium than in normal tissue endotheliu
r to be novel, with no obvious relationship to other transposases, or other known protein familie

centriole amplification (PubMed:22020124).

ıelle localization in exocrine cells and for mitochondrial calcium ion transport. May function as a

d nuclear (TAN) lines which are bound to F-actin cables and couple the nucleus to retrograde a
ınction probably occurs via HDAC1 down-regulation, keeping GLI1 acetylated and inactive. Inhi

ıplex is a direct component of the kinetochore-microtubule interface and directly associates wit
acilitates the processive movement of microspheres along a microtubule in a depolymerization-

ıilarity). Acts as a regulator of metabolism and hepatic lipid accumulation (By similarity). Under

surface. Regulates the ability of LPAR2 to activate ERK and RhoA pathways. Regulates the JN
ıtly interacts with actin-modifying proteins such as DAAM1 (By similarity). In cooperation with II
ucosylated structures expressed on cell surfaces or secreted in biological fluids are believed to

the same site in the gene, placing the splice sites an invariant distance from the constant struc

2/M phase but does not disassemble fully formed ciliary axonemes. As cilium assembly and dis

)614, PubMed:26682650). Cleaves HJs by a nick and counter-nick mechanism involving dual c

fficking of endosomal alpha5beta1 integrin to the plasma membrane and involved in invasive c

bly via its function as regulator of autophagy.

onuclease III enzyme to produce an approximately 70-nt stem-loop precursor miRNA (pre-miRN

›nuclease III enzyme to produce an approximately 70-nt stem-loop precursor miRNA (pre-miRI
ı-dependent mechanism. At the nucleoplasmic side of the NPC, Ran binds to importin-beta anc

Med:23387299, PubMed:23453970, PubMed:23576762, PubMed:23665031). The BCR(KLHL3
ase activity. Also involved in the regulation of growth-related processes in adipocytes and myob

May serve as nuclear receptor.

e of inflammatory signaling pathways. In cooperation with TAX1BP1 promotes disassembly of
IL9R, IL15R and IL21R. Following ligand binding to cell surface receptors, phosphorylates spe

binding sites in the outer plate of the kinetochore (PubMed:15548592). The NDC80 complex
ositioning and bipolar spindle stabilization (By similarity).
tes (By similarity).
phosphorylation (PubMed:18663142, PubMed:19208764). TPX2 is inactivated upon binding to

ty (PubMed:31978345). Acts as a regulator of innate immunity in macrophages by modulating
ating HIV-1 gene expression. Isoform 2 and isoform 3 also bind to the IPCS (IRF1 and p53 com

d nucleosome remodeling.
431929). Regulates cell growth through regulation of cell cycle progression and cytokinesis (Pu
key role in DNA damage response (DDR) by being conveniently positioned at the replication fo

›nuclease III enzyme to produce an approximately 70-nt stem-loop precursor miRNA (pre-miRI

ization of this complex; directs CPC movement to different locations from the inner centromere

ed:24262037). Negatively regulates apoptosis, possibly by modulating the activity of caspase-9

9). Likewise, the TRIAP1:PRELID3A complex mediates the transfer of phosphatidic acid (PA) b
ADPH or NADH to form squalene. It is the first committed enzyme of the sterol biosynthesis pat
secretion in the extracellular milieu. Activation of NLRP1 inflammasome is also required for HM

1 complex, in association with SNARE proteins, is also proposed to be involved in neurite exter

hondria, promotes translocation of BAX to mitochondria and cytochrome c release from mitoch
oncogene for promoting bladder cancer cells proliferation, apoptosis inhibition and carcinoger
represents a distinct pathway or if it can be integrated in the canonical pathway, as PKC seem

onuclease III enzyme to produce an approximately 70-nt stem-loop precursor miRNA (pre-miRN

s to the cytoskeleton by binding PLEC which can associate with the intermediate filament syste
Mechanistically, uses molecular oxygen inserting one oxygen atom into a substrate, and reduci
lin turnover.

finity for GCK, while GCKR with bound fructose 1-phosphate has strongly decreased affinity for

quired for the initiation of the B-cell response, but also for its down-regulation and termination.
G proteins. Signaling inhibits adenylyl cyclase activity and decreases cellular cAMP levels (Pu

ocampal neurons in a tubulin and actin-dependent manner. In the developing telencephalon, c

and thereby modulating cell cycle progression and cell survival (PubMed:19647222, PubMed:2
y). May positively regulate the transcription by NFE2L2 of genes involved in the response to ox
nation by endothelial cells as well as angiogenesis (PubMed:15467828, PubMed:16488400, Pu
skeletal structure through its F-actin-bundling activity. Involved in the regulation of cell adhesio
the 5'-terminus of the tRNA 3'-exon during tRNA splicing; this phosphorylation event is a prere

n of MAPK1/3 (ERK1/2), through SOCS6. Involved also in the control of energy metabolism an
ing and memory deficits (By similarity).

) (PubMed:9174363). May play a role in classical or alternative activation of macrophages via i
(By similarity). In macrophages and dendritic cells, specifically involved in TLR3- and TLR4-inc

stress-inducible metabolic regulator also plays a role in protection against oxidative and genot

NA), which is further cleaved by the cytoplasmic Dicer ribonuclease to generate the mature miF

ed in somatic tissues. [provided by RefSeq, Aug 2019]

e re-folding of misfolded proteins and controlling the targeting of proteins for subsequent degra
NA), which is further cleaved by the cytoplasmic Dicer ribonuclease to generate the mature miF

l:19332548, PubMed:20129063, PubMed:23478441, PubMed:23478445, PubMed:23677613, I

e re-folding of misfolded proteins and controlling the targeting of proteins for subsequent degra

ons in which global protein synthesis is inhibited (By similarity). Required for cell cycle progress

. This gene also produces a ARHGAP11A-SCG5 readthrough transcript and ARHGAP11A-SC

development of the inner ear. Phosphorylates PLCG1, CBL and FRS2. Ligand binding leads t
repair pathway. Required for proper resolution of DNA double-strand breaks (DSBs) by HR. Is r
olymerase delta by competing with POLD3 for PCNA binding (PubMed:11595739). Plays an im
ylation state and/or stoichometry.

NA), which is further cleaved by the cytoplasmic Dicer ribonuclease to generate the mature miF

preapical plasma membrane subdomain to set up initial epithelial polarization and the apical lu

PubMed:28302677). Mediates phosphorylation of ERK1/2 and thereby promotes retinal lens f

enhanced by the katanin complex (PubMed:28436967). May have a preferential role in regula

lherens protein PLEKHA7 (PubMed:30463011).

ssor (PubMed:11782474). Binds to the consensus binding site 5'-[G/C][A/T]AAA[T/C]AA[A/C]-3

ture and the way DNA is wrapped around the nucleosome and gives rise to protruding DNA en

netochores and spindle microtubules (PubMed:7889940, PubMed:23891108, PubMed:253955
r permissive role in the normal embryonic cardiomyocyte cell cycle and in promoting continued

betes with histone H3 for the same binding site on the histone demethylase complex formed by

(PubMed:10806483). Required for postnatal development, possibly by regulating the homeosta

NA), which is further cleaved by the cytoplasmic Dicer ribonuclease to generate the mature miR

pathway. May act by recruiting the corepressors CTBP1 and HDAC3. May be involved in neur

e G2 checkpoint and progression to cytokinesis during mitosis. Plays a key role in response to
t is functionally redundant with SOX17. Interaction with MEF2C enhances transcriptional activa
he SKA1 complex for microtubules and may allow the NDC80 complex to track depolymerizing
NA), which is further cleaved by the cytoplasmic Dicer ribonuclease to generate the mature miR

LUG-dependent repression of E-cadherin transcription. Acts as a hypoxic regulator by bridging
r (GG-NER) by acting as damage sensing and DNA-binding factor component of the XPC comp
diabetes. Interaction with S100A12 on endothelium, mononuclear phagocytes, and lymphocyte
in-2-induced activation of integrin beta-1 (ITGB1) in cardiac fibroblasts. Phosphorylates MARC

t on intracellular ceramide levels, enhances cell growth and inhibits apoptosis (PubMed:161182

mbrane space and subsequent apoptosis (PubMed:18350175).
ubiquitin ligase complex components ELOC and CUL5 (PubMed:21199876).

lay an important role in the development and maturation of many tissues (PubMed:12213388).
plasmic scaffolding proteins.

\kt triggers apoptosis and decreases cell proliferation. Also controls the phosphorylation of AK

and milk production in the mammary gland and inducing differentiation of mammary tumor cell

d by ultraviolet light (By similarity).
ulates its interaction with the NCOR1/HDAC3 corepressor complex, enhancing transcriptional r

CTLD superfamily members on chromosome 12p13 in the natural killer gene complex region. [
ubiquitin ligase complex components ELOC and CUL5 (PubMed:21199876).
nas been shown to be involved in several aspects of telomere replication. The CST complex in

vth suppression.

nune system activator SKAP2 (PubMed:21719704). Positively regulates toll-like receptor (TLR)

remodeling by phosphorylating the Ras activator RASGRF1 and the Rap inhibitor SIPA1L1 lea
leolytic degradation, or deadenlyation-dependent mRNA decaping and subsequent 5'-3' exonu
ardly than (pyroglu)apelin-13 from APLNR (By similarity). Hormone involved in the regulation of

428, PubMed:20347429). In complex with CENPX and FANCM (but not other FANC proteins), r
nbryonic stem cells, has weak methyltransferase activity and plays a less critical role in forming

binds to promoter regions (PubMed:7969177, PubMed:24382891, PubMed:28500257). Regula

ation and autophagy.

receptor required for apoptosis induction when not associated with netrin ligand (PubMed:1259

ven transcriptional activation.||[Isoform 4]: Attenuates the ability of transcription factor HIF1A a

ssivity of telomerase by displacing active telomerase from DNA ends. Releases telomerase by

this gene. [provided by RefSeq, May 2011]

sor complex (PubMed:20159957). Binds to the sequence element 5'-AACGACCGACCTTGAG-

ıdation. Component of the TRIM28/KAP1-MDM2-p53/TP53 complex involved in stabilizing p53

:. Expression of this gene may be regulated by the cytokine TNF-alpha. [provided by RefSeq, I
ebellar granule neurons, phosphorylated and sumoylated MEF2A represses transcription of NU
ı pathway and acts as a scavenger receptor for SDF-1. Binds to the allosteric site (site 2) of inte

radative activity of the exosome on several of its nuclear RNA substrates. Doesn't need a cofa
ID), and is thus unlikely to produce a protein product. [provided by RefSeq, Feb 2017]
:ytes from the splenic reservoir to sites of inflammation (By similarity).
nt, distribution, and electrical activity in the neocortex. Necessary for proper development of me
ıbMed:26496226). Involved in the regulation of cell proliferation and resistance to thermal stres

ɔa-B signaling. The target proteins for its E3 ubiquitin-protein ligase activity include: RIPK1, RIP

NA), which is further cleaved by the cytoplasmic Dicer ribonuclease to generate the mature miF
d: Lys-6-linked may be involved in DNA repair; Lys-11-linked is involved in ERAD (endoplasmic

ıre likely to contribute differentially to the complex helicase activity.

ox domains. Phosphorylates BORA, BUB1B/BUBR1, CCNB1, CDC25C, CEP55, ECT2, ERCC
dzone spindle assembly and cleavage furrow formation. Key component of the cytokinesis che
ctivity suppressed by insulin (PubMed:10358076). Main regulator of redox balance and osteobl

ional activator activity of the CLOCK-ARNTL/BMAL1 heterodimer. Restricts the CLOCK and AI
in kidney development and may be involved in podocyte differentiation (By similarity). During e

ional activator activity of the CLOCK-ARNTL/BMAL1 heterodimer (By similarity).

es endothelial cell survival during vascular development. Serves as a microtubule-dependent si
1 and CDKN1B/p27. Normally, CDKN1B/p27 turnover is regulated by COPS5, which binds CD

ugh the inhibition of KDM8 (PubMed:28455245).

ınd activation of RAS, RAF1 and the MAP kinases MAPK1/ERK2 and/or MAPK3/ERK1. KITLG

ərmutation (SHM) and class switch recombination (CSR) of immunoglobulin genes. Essential fc

thereby affects neural activity. May also play a role in regulating the release of other neurotrar

NA), which is further cleaved by the cytoplasmic Dicer ribonuclease to generate the mature miF
3/TP53-independent intracellular reactive oxygen species (ROS) regulatory function and a p53/

ent manner (By similarity). Controls macrophage, eosinophil and neutrophil differentiation via th
interaction with PPP2R1A. Its phosphorylated form is necessary for chromosome congression

chromosomes and enabling independent chromosome motility (PubMed:27362226). Binds DN

ochore oscillations and dynamics of microtubule plus-ends during live cell mitosis, possibly by f

contributing to the accumulation of EGFR at the limiting membrane of early endosomes. Dowr

otif-containing reporter construct nor in inhibitory activity on the isoform 1 transcriptional repres
omplex that recognizes UV-induced DNA damage and recruit proteins of the nucleotide excisio

y the down-modulation of the RXR alpha activities.

extracellular matrix production, immunosuppression and carcinogenesis. The formation of the

thylates H3K9me2 in absence of H3K4me3. Has activity toward H4K20Me1 only when nucleos

emethylation and transcriptional activation. Involved in spermatogenesis by regulating expressi
subunit of phosphatidylinositol 3-kinase. Activated KIT also transmits signals via GRB2 and act

15322075, PubMed:15775988). Plays a dual role both as an inhibitor of CCAAT/enhancer-bin

1, KCNQ2/Kv7.2, KCNQ3/Kv7.3 or CLC5 (PubMed:26363003, PubMed:27445338). Promotes

hich is required for proper activation of the transcription factor (PubMed:28609714). Both inhibi

emoved base with both 3'- and 5'-phosphates. Has DNA glycosylase/lyase activity towards mis

se to mitogenic or stress stimuli such as UV-C irradiation, epidermal growth factor (EGF) and a
11A, is located only 16 kb from this gene on human chromosome 11q12.1. [provided by RefSe

NA), which is further cleaved by the cytoplasmic Dicer ribonuclease to generate the mature miF
recognizes and binds histone H3 trimethylated at 'Lys-9' (H3K9me3) and unmethylated at 'Arg

and relaxation of vascular smooth muscle (By similarity). Binds EIF2B5 and blocks its activity,
ling multiprotein complexes. May also play a role in the organization of chromosomes in the nu

NA), which is further cleaved by the cytoplasmic Dicer ribonuclease to generate the mature miF

ry pathway is exploited by tumors to attenuate anti-tumor immunity and escape destruction by
gulate transcription. Through interaction with SMAD2 and SMAD3, LDLRAD4 may compete wit
NA), which is further cleaved by the cytoplasmic Dicer ribonuclease to generate the mature miF

:12554770). The RXRA/RARB heterodimer can act as a repressor on the DR1 element and as
unctions in p53/TP53-dependent mitotic spindle checkpoint. Regulates microtubule dynamics a

C-4 and C-18. Has no activity toward 9-cis and 13-cis retinoic acid stereoisomers (PubMed:220

t by internal magnesium. Can be blocked by extracellular barium or cesium.
ays a role in immune response, of T cells, natural killer (NK) and neutrophils (PubMed:184247

n are essential for the homology search and strand exchange (PubMed:26681308). Part of a P

se and GTP hydrolysis are modulated by numerous regulatory proteins (PubMed:8774883, Pu

NA), which is further cleaved by the cytoplasmic Dicer ribonuclease to generate the mature miR

differentiation of epithelial cells and may also function in skeletal and kidney development. Co
ells, enhances key inflammatory mediators and inflammatory response through the modulation

lar stress response and is in turn repressed by p53/TP53 presenting a SIRT1 isoform-depende

bMed:24623306).

hain of TGF-beta (PubMed:22278742). Controls activation of TGF-beta-1 (TGFB1) on the surfa

NA), which is further cleaved by the cytoplasmic Dicer ribonuclease to generate the mature miF
s (NETs) (By similarity). NETs are mainly composed of DNA fibers and are released by neutro
e H2A 'Lys-119' on the X chromosome and is required for normal silencing of one copy of the X

sary for the maintenance of the epithelial stem-cell compartment of the small intestine.
ERCC6L translocase and ATPase activities (PubMed:28977671).

zation of CENPE. Negatively regulates PLK1 activity in interphase cells and suppresses centros
d thereby affects sister chromatid cohesion. Acts as a substrate for anaphase-promoting comp

eres, thus leading to cohesin dephosphorylation (By similarity). Essential for recruiting KIF2C to

RIS, involved in activation of BAG1 gene expression by modulating dimethylation of promoter

the function implies inhibition of methylation on histone H3 'Lys-4' (H3K4me3) and 'Lys-36' (H3

10192396).
euronal fate specification. Represses transcription by the cardiac transcriptional activators GAT

ubMed:19890979).

o-M transition, after the nuclear-envelope breakdown: acts by mediating phosphorylation of mu
ed for adipogenesis: regulates triglyceride metabolism in adipocytes by regulating expression o

sponses through the activation of specific target genes and can act as a transcriptional activat

kappa-B activation through repression of EP300-dependent RELA acetylation.
nge and homologous recombination.
from the constant structural features of the tRNA body. Isoform 1 probably carries the active s

cells (PubMed:12917409, PubMed:14570867, PubMed:14592415). May also mediate cholest

at mitochondrial surface (By similarity). Although it cannot be excluded that it can act as a phos

erentiation of tissues involved in metabolic control. May play a regulatory role during G0/G1 tra

-3 kinase, nuclear accumulation of beta-catenin and activation of Wnt target genes (PubMed:1

not yet clear if it represents a distinct pathway or if it can be integrated in the canonical pathw

NA), which is further cleaved by the cytoplasmic Dicer ribonuclease to generate the mature miR

›Med:31672913). Upon amino acid restimulation, disassembly of the LFC complex liberates th

C to the inner centromere, and the microtubule binding activity within the central SAH domain
›ression of YAP1 and WWTR1/TAZ, thereby regulating cell proliferation, migration and epitheli

/ morphogenesis of the lens vesicle. Together with the CITED2 coactivator, stimulates the PIT

poietic precursor cells to the bone marrow stroma. Isoform 2 or the nuclear form is probably inv

n cardiac activating regions (By similarity).

ıd two variants are described. Additional splicing is suggested but complete sequence for furth

›s. May play a role in the maintenance of naive T-lymphocytes in the spleen.
ıosphorylation of STAT3.

d repress transcription. Recognizes and binds p53/TP53 monomethylated at 'Lys-382', leading
:pression of KLF1. May be involved in regulation of some genes in gonads. May also be involve
triphosphate (m7GpppG). Also hydrolysis m7G- and m227G U3-capped RNAs but with less eff

y. In vivo, appears to be the primary enzyme involved in removing Sp and Gh from ssDNA in n

Also plays an important role in the biosynthesis of complex lipids (PubMed:8626589). Display
localization of NUMA1 and DCTN1 to the cell cortex during metaphase (PubMed:27335426). I

on from the 14-3-3 protein YWHAQ which acts as a negative regulator.
yte and T cell development (By similarity). Involved in the regulation of postmitotic positioning c

ıstream MMR events, including strand discrimination, excision, and resynthesis. ATP binding a
ıe peroxidase activity reduces PGG2 to the hydroxy endoperoxide PGH2, the precursor of all 2

acts as an inhibitor of homology-recombination repair (HR) pathway by limiting RAD51 accumu

ocalized within the parabrachial nucleus and central amygdala, which contitutes part of the 'em

776429). May also contribute to the regulation of gene expression in the nucleus (PubMed:102

NA), which is further cleaved by the cytoplasmic Dicer ribonuclease to generate the mature miF

cts as a coactivator of B-cell-specific transcription. Plays a role in spermiogenesis and oogenes

larity). Promotes cell differentiation by regulating MAPK1/MAPK3 activity and regulating the ex
ammatory and innate immune responses. In macrophages, enhances Fc-gamma receptor-mec

epression of G1/S-regulated genes. Plays a key role in polyploidization of cells in placenta and
NA), which is further cleaved by the cytoplasmic Dicer ribonuclease to generate the mature miF
ntent, correlating with its ability to protect cells from oxidative or metabolic stress-induced cell d

n into a substrate, and reducing the second into a water molecule, with two electrons provided
site of a number of enhancers and promoters, including murine leukemia virus, polyomavirus e

s by promoting stable kinetochore-microtubule attachments (PubMed:22581055). Potential onc
sphorylation of YAP1 by LATS1/2 inhibits its translocation into the nucleus to regulate cellular g

imilarity).
91, PubMed:17332328).

;, and thereby regulates their activity (By similarity).

lopodial dynamics and enhancing axon elongation. Its binding to kinesin heavy chains (KHC), p
found in leukemic cells is involved in leukemogenesis and contributes to hematopoietic stem/p

nt and differentiation of IL17A expressing gamma-delta T-cells (By similarity). Regulates expres

SNRPE, SNRPF and SNRPG are trapped in an inactive 6S pICln-Sm complex by the chaperon

ge inflammatory response. Acts as a receptor for heme which stimulates its interaction with the
quired for efficient homologous recombination-dependent repair of double-strand breaks (DSBs

integrity (By similarity).
ating centrosomal proteins such as CROCC, CEP250 and NINL, resulting in their displacement

exes must first cluster into clathrin-coated pits.

on and direct binding of a specific DNA sequence in their promoter regions. Involved in early e

NA), which is further cleaved by the cytoplasmic Dicer ribonuclease to generate the mature miR

and SHPTP2 to the tyrosine phosphorylated form. Required for correct adhesion and migration

and binding of spermatozoa to zona pellucida.

nite segmentation and patterning (By similarity).

tor endocytosis.
t induce detectable inactivation of KCNMA1. Two or more subunits of KCNMB3 are required to

sue and activate their prothermogenic actions. Stimulates an eosinophil-dependent increase ir

Kruppel-like factors KLF4 and KLF2 (By similarity). Controls cell senescence in a p53-dependen
01839, PubMed:29018201). In response to oxidative stress, electrophile metabolites inhibit act

C1 and CIITA-dependent transcriptional activity (PubMed:15940264, PubMed:15941832, PubM

al, proliferation and cell death. Activates expression of p53/TP53 and TGFB1, and thereby help

NA). The CTF18-RFC complex catalyzes the ATP-dependent loading of PCNA onto primed an

rotein synthesis-dependent forms of long-term potentiation (LTP) and depression (LTD) and fo

-induced RAC1 GTPase activation and vascular endothelial cell migration and assembly. Exer

nduced degradation of NEUROD2 induces presynaptic differentiation.

P8, CDC7, CDC20, CDC25A, CDC25C, CC2D1A, CENPA, CSNK2 proteins/CKII, FZR1/CDH1

n of a variety of glycerolipid and sphingolipid phosphate esters including phosphatidate/PA, lys

play a negative role in adipogenesis through the regulation of lipolytic and antilipogenic gene e

quitination in response to DNA damage.

to the regulation of T-cell responses. Promotes osteoclastic bone resorption; this requires both

andins (PubMed:21049984).

membrane and binds to RAB3IP/Rabin8, the guanosyl exchange factor (GEF) for Rab8 and the
22354992). May act as a molecular switch between endosomal and autophagosomal transpor

P-ribosylation of ubiquitin which prevents ubiquitin conjugation to substrates such as histones
H4). Histone deacetylation gives a tag for epigenetic repression and plays an important role in
l chaperone function (PubMed:11274138, PubMed:15577939, PubMed:15937123, PubMed:27

Involved in the unidirectional retrograde dendritic transport of endocytosed BACE1 and in effic
(By similarity).
ntrastrand d(GpG)-cisplatin cross-link, inserts dCTP opposite the 3' guanine (PubMed:2444990

TRAF2-TRAF3 E3 ligase pathway to promote immune cell survival and differentiation (PubMed

ession seems to be a mechanism for evolving species-specific changes in skeletal structures. S
nediated signal transduction but instead induces beta-arrestin recruitment, leading to ligand inte

y of cerebral cortex activity during wakefulness and sleep.

ssion of MCM10 which plays an important role in DNA-replication (PubMed:24726359). Acts as

to use 1-monoalkylglycerol (1-MAkG) as an acyl acceptor for the synthesis of monoalkyl-mono

nelial barriers (PubMed:26795251, PubMed:24695216, PubMed:20581831). Signature pyrimidi
ene transcription by transactivating GNRH1 promoter and repressing PENK promoter (By simila
gamma, dissociates the protein from the aminoacyl-tRNA synthetase multienzyme complex and
r. Shows substrate preference of CDP > UDP > ADP > GDP > TDP.

proviral genome, along with a deamination-independent mechanism that works prior to the pro

otion in response to nutrient deprivation seems to involve CTBP1. In cooperation with MTA1 (in

hich phosphorylate interferon regulatory factors: IRF3 and IRF7 which in turn activate transcrip

ubiquitination, decreasing the activating 'Lys-63'-linked ubiquitination and leaving unchanged th

rriers, others mediate the permeability to ions and small molecules. Often, several claudin fam

omplexes, and may also be involved in signaling pathways in T-cells and neural cells. Differenti

ly involved in the regulation of reactive oxygen species homeostasis. May be a prominent sour

PubMed:23903368).

ılso stimulate the activity of DNA ligases and/or restrict the nuclease activity of MRE11 to preve

vound healing. Signaling is modulated by the formation of heterodimers with PDGFB (By simila

ganization; negatively regulates focal adhesion (FA) assembly promoting malignant glial cell m

ɔendent and -independent mechanisms (PubMed:28469175, PubMed:31006531). Upon activa
ıg CCAR2 to the cytoplasm, thereby impairing its ability to inhibit SIRT1 which is involved in the

ubMed:23027953, PubMed:23910378, PubMed:23747010, PubMed:30842659). Innate immun

ı. Regulates the stability of growth factor receptors, including PDGFRB (Probable).

and may function as a water and urea exit mechanism in antidiuresis in collecting duct cells. It

; adapters (CLASPs, clathrin-associated sorting proteins) and recruiting the GPRCs to the adap

key role in the development of the visual pathway. Regulates the formation in ipsilateral retina

ɔonse to IFNG stimulation (PubMed:27796300). However, the role of ADP-ribosylation in the pr
ls by sequestering the target RNAs. Seems to regulate expression and localization of ITGA3 by
lls (TH17). It drives rapid clonal expansion of naive but not memory CD4 T-cells. It also strongly
al mRNA to initiate its degradation from the 5'-end. Its target viruses belong to families which in

Mechanistically, recruits TBK1 at the Golgi apparatus, promoting its trans-phosphorylation after

ıtrol of glycolysis and is important for tumor cell proliferation and survival (PubMed:17308100, I

ıce nicks at hydroxyuracil and other types of pyrimidine base damage (PubMed:17353262, Pub

I, CYTH2, CYTH3 and CYTH4 to the plasma membrane in the GDP-bound form. Regulates the
; neurogenesis: acts by inhibiting Notch cleavage by furin, maintaining Notch in an immature ina

tely required. Participates in biosynthesis of heparan sulfate that can ultimately serve as L-sele
ɔinding laminin G-like domain-containing extracellular proteins with high affinity (PubMed:2527S
MP2 (PubMed:12714657). Cleaves ADGRB1 to release vasculostatin-40 which inhibits angioge

tor (BCR) signaling. Mediates B-cell proliferation response to anti-IgM, anti-CD40 and IL4 stimu

e response is still unclear but it may function as a licensing signal for additional histone H4 pos

nucleus. Plays an important role in regulation of the kinase activity of AURKA for mitotic commi

oAs (PubMed:10744784, PubMed:12923223).
esis (PubMed:23084991). In concert with RAB32, regulates the proper trafficking of melanogen

ndothelial migration, ICAM1 engagement promotes the assembly of endothelial apical cups thro

ı repair pathway (the NER pathway) to initiate DNA repair (PubMed:15448697, PubMed:16260

cts with GRB2 or PLCG2 and induces phosphorylation of MAPK1, MAPK2, FAK/PTK2 or RAC1
s.

including cell survival, cell proliferation, migration and differentiation. Ligand binding at the cell
different kappa-B sites that they can bind with distinguishable affinity and specificity. Different

l-terminal glycine degrons are conditionally exposed after a failure of N-myristoylation (PubMec

ed in conditioned medium of first trimester trophoblast. Kp-10, but not other kisspeptins, increa

ıbMed:23776175). Is involved in the regulation of skin pigmentation through the control of mela

egrin beta-1; this function seems to redundant with the AP-2 complex and seems to require DA

n also phosphorylate 1-alkyl-2-acylglycerol in vitro as efficiently as diacylglycerol provided it co
sitioning. May be involved in nucleus-centrosome attachment and nuclear migration in neural p
ooth muscle, the tuning of hair cells in the cochlea, regulation of transmitter release, and innate
F6, leading to NF-kappa-B activation, cytokine secretion and the inflammatory response.

e from the blood serum and in lipid metabolism (PubMed:19270337, PubMed:21398697, PubM

radation of cellular as well as viral RNA, resulting in the inhibition of protein synthesis, thus terr
ctivation mediated by estrogen receptors. Acts also as a transcriptional corepressor; interferes
is viral RNA, resulting in the inhibition of protein synthesis, thus terminating viral replication. Ca

ransactivation (By similarity). Plays a pivotal role in the transactivation of NF-kappa-B, SRF and

cells. Can replace beta-catenin in E-cadherin/catenin adhesion complexes which are proposed

membrane and binds to RAB3IP/Rabin8, the guanosyl exchange factor (GEF) for Rab8 and the

exes which contain ACTL6A/BAF53A and PHF10/BAF45A, are exchanged for homologous alte

cuole-like structures (named MIV), which engulf and degrade unhealthy mitochondria by accun
major integrators of various mitogenenic and antimitogenic signals. Also substrate for SMAD3,

tein, which may regulate hemostatic balance and function as a tumor suppressor. Mutations in
nd is required for lysine succinylation of histones: associates with KAT2A on chromatin and pro
gulator of the ATF4-dependent transcription during the ISR: while TRIB3 expression is promote
te inflammation impairing NLRP1-inflammasome activation, hence CASP1 activation and IL1B

endosomes and lysosomes. Has a critical role in neurodevelopment (PubMed:31079899).
ced activation of CEBPB. Involved in the prevention of autoimmunity; this function implicates bi

ıd organization of newly synthesized extracellular matrix.

ampal neurons, it recruits ADAM10 to the plasma membrane (PubMed:23676497).

action of SPATA18/MIEAP, BNIP3 and BNIP3L/NIX at the mitochondrial outer membrane regul

he functional antimicrobial barrier of the colonic epithelium, and to the bactericidal activity of ar

ved by the formation of mitochondrial [2Fe-2S] proteins, the synthesis of [4Fe-4S] clusters and

ts as a negative regulator of B-cell antigen receptor signaling. Mediates signaling from the FC-
dependent apoptosis. Therefore, activation of RNASEL could lead to elimination of virus infecte

mote cancer cell migration, invasion and tumor growth (PubMed:20079333).

he functional antimicrobial barrier of the colonic epithelium, and to the bactericidal activity of ar

of TGF-beta-1 activated from one microglial cell to neighboring microglia, suggesting the exister

econdary necrosis downstream of the mitochondrial apoptotic pathway and CASP3 activation a

: it appears to be involved in three stages: inflammation, proliferation and remodeling. Plays an

hains attached to glycoproteins. Participates in biosynthesis of selectin ligands. Selectin ligands

ed. Therefore, the proteasome participates in numerous cellular processes, including cell cycle

:aveolae by constraining caveolae at the cell membrane (PubMed:25588833).

drugs across the brush border membranes.

wound healing probably by modulating both growth and migration of primary keratinocytes and
iction that is necessary for physiological and asthmatic airway resistance. Necessary for gastro

'P metabolism, kinase-dependent intracellular signaling, neuronal calcium signaling as well as

E2V1-UBE2N (also known as UBC13-UEV1A complex) generates 'Lys-63'-linked polyubiquitin

d CRY1/2 (PubMed:15193144). Represses the activity of the circadian transcriptional activator

atellite cell proliferation (PubMed:10080180, PubMed:22431096, PubMed:10814726, PubMed
ntified on chromosome 2. [provided by RefSeq, Sep 2011]
n integrity of epithelial barrier (By similarity). Ligand for integrin alpha-L/beta-2 involved in mem
control centrosomal microtubule organization (PubMed:20719959).
major integrators of various mitogenenic and antimitogenic signals. Also substrate for SMAD3,

sor of a pair of coregulated exons: promotes inclusion of the smooth muscle (SM) exon but exc

, PubMed:26758068). Involved in splice site selection of vascular endothelial growth factor (Pu

proviral genome, along with a deamination-independent mechanism that works prior to the pro
aves SPHK2 which is released from cells and remains enzymatically active extracellularly (Pub
the TAP complex and inhibits peptide translocation by specifically blocking ATP-binding to TAF

inase activity of mTORC1. Amino acid-signaling to mTORC1 requires its relocalization to the ly

ubMed:28978524, PubMed:18799424, PubMed:24912431). Involved in the AKT signaling casc

s are clustered in a region of chromosome 12q13. Two transcript variants encoding two differen

orting cytotoxic 7beta-hydroxycholesterol (PubMed:17408620).

ısmembrane ephrin-B ligands including EFNA1 and EFNB3. Upon activation by ephrin ligands,

ER) stress, inserted into ER membranes, with N-terminal DNA-binding and transcription activat
splicing results in multiple transcript variants. [provided by RefSeq, Aug 2011]

arget genes, negatively regulates androgen receptor signaling and androgen-induced cell proli

binding to receptors (FGFRs) and inhibiting the FGF-mediated signaling.
cell proliferation and tumorigenesis by inhibiting the CUL4A-RBX1-DDB1-VprBP/DCAF1 E3 ul
g on the cell surface and intracellular activation of LCK that phosphorylates the ITAM motifs of

inase-3, chymase, chymotrypsin, and kallikrein-3 (PubMed:11747453, PubMed:30692621). Ac

KN1B ('Thr-187' phosphorylated-form), thereby promoting its degradation by the proteasome.

ty. May also bind CCNB1 mRNA. Alternatively, may also regulate p53/TP53 activity through dir

ome-DNA interactions and promote interaction of the modified histones with other proteins whic
ed in tissue repair. May be a marker of basal cell differentiation in complex epithelia and theref

urodegeneration. Enhances desensitization by increasing both the rate and extent of desensiti

:h HSD17B4, catalyzes the hydration of trans-2-enoyl-CoA and the dehydrogenation of 3-hydro

(PubMed:12873986). Binds to the interleukin-6 (IL-6)-responsive elements identified in the pro
esicles (ILVs) that are generated by invagination and scission from the limiting membrane of the

ation of ubiquitinated-I-kappa-B-alpha. Required for TNF-alpha-induced p65 nuclear translocat
also bind EPHB2 and EPHB3 (PubMed:8070404). Binds to, and induces collapse of, commiss

May block ERK2 activation stimulated by ABL1 (Probable). May alter cell morphology and cell

', does not seem to be essential for PGC migration (By similarity).

of F(1) is coupled via a rotary mechanism of the central stalk subunits to proton translocation. I

NA), which is further cleaved by the cytoplasmic Dicer ribonuclease to generate the mature miF
emptive quality control, a mechanism that selectively attenuates the translocation of newly synth

subset of neuronal lineages. It has been shown that activation of some of these genes requires
shment and/or maintenance of bulk H3K4 trimethylation (H3K4me3), global transcriptional silen

e been identified. One transcript variant is experimentally supported, but it doesn't encode a pro

NA), which is further cleaved by the cytoplasmic Dicer ribonuclease to generate the mature miF

way form the junction to release hairpin-shaped pre-miRNAs that are subsequently cut by the c

). Along with DNA polymerase POLD1 and DNA polymerase POLK, has a role in excision repa

ession (PubMed:22722849, PubMed:30653310). H3K18Ac is mainly present around the transcr

emonstrate that this gene is a lncRNA tumor suppressor. [provided by RefSeq, Mar 2012]
ecific chromatin remodeling complex (nBAF complex). During neural development a switch from

: recruits the CSA complex (DCX(ERCC8) complex), nucleotide excision repair proteins and EF
ement their hypotensive effects in blood vessels.

tosis and flagellin-stimulated inflammatory response. Phosphorylates the epidermal growth fact

s. The BCDX2 complex was originally reported to bind single-stranded DNA, single-stranded g
thus in processing HR intermediates late in the DNA repair process; the function may be linkec
e proteasome and promoting mitotic exit (PubMed:19820702, PubMed:19822757, PubMed:27

A sequences, either 5'-AGSAGAGTA-3' (S=C or G) or 5'-GTTCGAGTA-3'. Can bind to beta-glc

tivity increases during S and G2 phases and decreases at M phase when it is hyperphosphory

e, blood pressure, endocrine, immune, cardiovascular, and renal function. Consists of two majc

ules (By similarity). This complex may have the potential to couple synaptic vesicle exocytosis t

.ys-27' (H3K27me3) (By similarity). Plays a role in the lineage differentiation of the germ layers

NA decay (PubMed:19909337). Modulates the inflammatory response by promoting the degrac

:ontaining CENPA. Component of the heterotetrameric CENP-T-W-S-X complex that binds and
NA), which is further cleaved by the cytoplasmic Dicer ribonuclease to generate the mature miF

:ogenic genes via its phosphorylation. Phosphorylates PPP2R5B thereby stimulating the assem
NA), which is further cleaved by the cytoplasmic Dicer ribonuclease to generate the mature miF

> H3K27me2 (PubMed:22323599, PubMed:30923826). Compared to EZH1-containing comple
n PLK4 at the outer boundary of a newly forming CEP152 ring structure (PubMed:24997597). /
NA), which is further cleaved by the cytoplasmic Dicer ribonuclease to generate the mature miF

otes transcription of LCN2 and DEFB4. Is recruited to IL-6 promoters and activates IL-6 but dec
ecific tag for epigenetic transcriptional repression. Mainly functions in pericentric heterochroma

:. Plays an important role in regulating the expression of genes that are involved in lipogenesis

: hydroxylation and spontaneous O-dehydroxypropylation (PubMed:26038696).

ɜr, by binding to matrix attachment regions (MARs) of DNA and inducing a local chromatin-loop

inner membrane that drives transmembrane transport and the ATP synthase. Cytochrome c o:

ɘ, blood pressure, endocrine, immune, cardiovascular, and renal function. Consists of two majc

300 substrates each. Some of the targets are downstream kinases such as MAPKAPK2, whicl

muscle stem cell differentiation (By similarity).
ɘ, blood pressure, endocrine, immune, cardiovascular, and renal function. Consists of two majc

histone gene transcription and progression through S phase.

'ubMed:26221039). The physiological relevance of protein/histone deacetylase activity is uncle

ɔoly(G) and poly(A), but not to poly(U) or poly(C) RNA homopolymers. Binds to the 5'-ACUAGC

ɘd by RefSeq, May 2015]
ɘ promoter via interaction with RRN3.

ɿnaling and stability (By similarity).

Seq, Oct 2015]
ɘment; MEIS2 is not involved in complex DNA-binding. Probably in complex with PBX1, is involv
NMD), and is thus unlikely to produce a protein product. [provided by RefSeq, Feb 2017]
ɔf F(1) is coupled via a rotary mechanism of the central stalk subunits to proton translocation (F

e lectin-like receptor that functions as a platelet receptor for the lymphatic endothelial marker, F
NA), which is further cleaved by the cytoplasmic Dicer ribonuclease to generate the mature miF

cycling of receptors including T-cell receptor (TCR), CD28 and ITGAL; proposed to be implicate

s the activity of down-stream effectors, such as adenylate cyclase (PubMed:10906333, PubMe

NA), which is further cleaved by the cytoplasmic Dicer ribonuclease to generate the mature miF

similarity). Preferentially hydrolyzes the ester bond of the fatty acyl group attached at sn-2 pos

05868). Transports LPC carrying long-chain fatty acids such LPC oleate and LPC palmitate wit

ıired for longitudinal bone growth through regulation of chondrocyte differentiation. May be indiı

ə, blood pressure, endocrine, immune, cardiovascular, and renal function. Consists of two majc

The dynamic control of cilium length is essential in the regulation of mechanotransductive sigr

genome. [provided by RefSeq, Jul 2008]

by regulating RANKL-induced calcium oscillations in preosteoclasts and mediating calcium ext
nd trafficking in sensory neurons. Probably plays a role in trafficking proteins in photoreceptor

d and thereby stops it from regulating the transcription of its target genes. This transcript is alsc

ubMed:26503245). Its double-stranded DNA helicase activity requires either a minimal 5'-singlε

complex (which can induce the formation of aberrant centriolar protein complexes) by interferin

of F(1) is coupled via a rotary mechanism of the central stalk subunits to proton translocation. I

ilarity). Inhibits TGF-beta signaling through interaction with SMAD4 and NCOR1. Binds to chro

NA), which is further cleaved by the cytoplasmic Dicer ribonuclease to generate the mature miF

e of GDP to GTP, converting inactive GDP-bound RAB8A and RAB39B into their active GTP-b
itch in subunit composition of the npBAF and nBAF complexes. As neural progenitors exit mito

ex ATP8A1:TMEM30A seems to play a role in regulation of cell migration probably involving fli

NA), which is further cleaved by the cytoplasmic Dicer ribonuclease to generate the mature miF

NA), which is further cleaved by the cytoplasmic Dicer ribonuclease to generate the mature miF

NA), which is further cleaved by the cytoplasmic Dicer ribonuclease to generate the mature miF

so mediates monoubiquitination of FANCL and FANCI (PubMed:16916645, PubMed:1793819

egulates the glycine concentration in synaptic junctions in the central nervous system, by bindir

where most DUF1220 domains are located, have been implicated in a number of developmen

a cytochrome P450 reductase (NADPH--hemoprotein reductase) (PubMed:11555828, PubMec

NA), which is further cleaved by the cytoplasmic Dicer ribonuclease to generate the mature miF

itory nerve fibers and reducing the range of dynamic hearing. This may protect against acousti
bably through the regulation of cellular calcium homeostasis. Phosphorylates PLN, a regulator
o prevent degradation of cyclin-A (CCNA1 and CCNA2) (PubMed:21596315). Plays an importa

y of KAT7/HBO1 in a CDT1-dependent manner, inhibiting histone H4 acetylation and DNA repl

NA), which is further cleaved by the cytoplasmic Dicer ribonuclease to generate the mature miF
oxine 5'-phosphate (PNP) and pyridoxamine 5'-phosphate (PMP), with order of substrate prefer

evoked currents by retaining the channel intracellularly. May regulate the intracellular trafficking

in the development of the ventral forebrain (By similarity). May play a role in craniofacial patter

NA), which is further cleaved by the cytoplasmic Dicer ribonuclease to generate the mature miF

ncoding different isoforms have been found for this gene. [provided by RefSeq, Feb 2016]

ersible TST reactions provides a model for the physiologically relevant reaction with thiosulfate

complex is required for telomere maintenance via recombination and mediates sumoylation of s

he endoplasmic reticulum (ER). All subunits are required for a maximal enzyme activity. May b[illegible]
NA), which is further cleaved by the cytoplasmic Dicer ribonuclease to generate the mature mi[illegible]
NA), which is further cleaved by the cytoplasmic Dicer ribonuclease to generate the mature mi[illegible]

nslocation. Phosphorylates in vitro GLI2, as well as GLI1 and GLI3, although less efficiently. Al[illegible]

HDAC1 repressor complex. Upon calcium influx, RB1 is dephosphorylated by calcineurin, whic[illegible]

ng pathway via two different mechanisms that probably work in parallel to reach the same effe[illegible]
ate) (PubMed:25320081). Participates in mitochondrial genome maintenance, regulation of mi[illegible]

hibition of TRPA1 by TRPV1 and increasing the single-channel open probability of the TRPA1-
RA). In males, not required for germ-cell sex determination, but required to allow the spermatog
on by binding to specific promoters repressing CEBPA binding to its target gene promoters (Pu
nsport.
ng subunit (PubMed:1309651). Regulates the activity of L-type calcium channels that contain C

n the mammary mesenchyme and this increases the sensitivity of these cells to BMPs and allo

inner membrane that drives transmembrane transport and the ATP synthase. Cytochrome c o

and cell migration, and it is involved in cell cycle regulation. Its upregulation in multiple cancer

reted immunoglobulins mediate the effector phase of humoral immunity, which results in the el

guidance molecule during development and may be involved in the development of the retinote
n of glucagon secretion by glucose in alpha cells; however such data require additional eviden
activation functions of ESR1 and plays a role in the nuclear localization of ESR1.||Regulates a

ubMed:8820107). Also produced in the brain by primary astrocytes, and esterifies free choleste

hroblasts shift from a highly proliferative state towards their terminal phase of differentiation (Pu

mplex is regulated by binding with 7SK snRNA (PubMed:11713533). Plays a role during muscl
sites where LP-BER proceeds; endonuclease FEN1 cleavage activity on substrates with double

rafficking.
uding REST (PubMed:23178126). Also controls the expression of genes involved in somatic de
nhibitory function (By similarity). Following TCR engagement, LAG3 associates with CD3-TCR

g different isoforms. [provided by RefSeq, Feb 2016]
anions like chloride, bicarbonate, formate and hydroxyl ion and divalent anions like sulfate and

g of blood vessels (By similarity).

aling pathway downstream of the ephrin ligand is referred to as reverse signaling. Binds to rece

tion of APAF1 into the nucleus after proapoptotic stress. Down-regulates the expression of LG/

NA), which is further cleaved by the cytoplasmic Dicer ribonuclease to generate the mature miF

'Pases CDC42, RHOA and RAC1. Promotes the conversion of CDC42, RHOA and RAC1 from

genicity by affecting dendritic cell costimulatory molecule expression and cytokines and chemo
The clathrin lattice serves as a mechanical scaffold but is itself unable to bind directly to membr
iones, such as angiotensin III and IV, neuropeptides, and chemokines. May also be involved th

um (PubMed:27214549). May contribute to the immune evasion of H.pylori by restricting M1 ma

n-lymphoid organs, such as the skin and gut, but also in other nonbarrier tissues like liver and
'K2/FAK1, a key mediator of integrin signaling events leading to the regulation of actin cytoskel
ulation of PERK-mediated unfolded protein response (By similarity). Activates apoptosis indepe
and probably other components of the cytoplasmic RNA decay machinery to the bound ARE-c

ith calmodulin. Also plays an essential role during cellular mitosis and receptor capping. Involve

gh RET cleavage.
nduces T-cell proliferation and NF-kappa-B activation in a T-cell receptor/CD3-dependent man

uch as hyaluronan/HA, collagen, growth factors, cytokines or proteases and serves as a platfor
the endosomal/lysosomal system where the antigen processing and binding of antigenic peptid

in neuronal recognition mechanism. In hippocampal neurons, may regulate dendritic spine den
cert with KRIT1 and MPP5 to establish and maintain correct endothelial cell polarity and vascu

it. Required for normal male and female fertility. Promotes reorganization of the actin cytoskele

on of gamma-delta T-cells, a subpopulation of T-cells residing in epithelia and involved in tissue
entiation via its effects on NF-kappa-B activation (PubMed:12917690). Negative regulator of W
, regulates myosin either directly by phosphorylating MYL12B and MYL9 or through inhibition o
eaks (DSBs), thereby facilitating the template-guided repair of transcriptionally active regions o

e and the membrane mechanical properties. Plays also a role as a modulator of actin dynamic
nection between the 2 temporal lobes of the cerebral cortex. Also involved in guidance of cont

ızymes generated during infection. Generally is promoting inflammation. Can signal synergistic
d:16480718, PubMed:16410248, PubMed:17381073, PubMed:18095711, PubMed:21288888,

thus seems to provide a feedback mechanism to limit weight gain.

s FLT4 signaling. Modulates KDR signaling by forming heterodimers. The secreted isoform 3 n
egulation of cell adhesion and motility through phosphorylation of CTNNB1 (beta-catenin) and

is implicated in cell growth and survival, cell adhesion and cell migration. GAS6/AXL signaling

ıa-glutamyl compound. Initiates extracellular glutathione (GSH) breakdown, provides cells with
). Plays an important role in the down-regulation of neuronal excitability (PubMed:11929858, F
ersion to GM3 (By similarity). Has cholesterol transfer activity (PubMed:17552909).
ubsequently regulates transcription factor AP-1 (activating protein-1) (By similarity). GNA12-de

phosphorylates various substrates: Hsc70-interacting protein/ST13, TP53/p53, HDAC5, and arr
s as a transcription coactivator in adipocytes by promoting the recruitment of PPARG to promot

a role in translating the epigenetic signatures into cell type-specific enhancer-driven transcriptio

ılar stress mediated by exposure to anisomycin or UV (PubMed:24512202).

nd this binding is essential for IGF1 signaling (PubMed:22351760). ITGA6:ITGB4 binds to IGF

racellular potassium; as external potassium is raised, the voltage range of the channel opening

ng macrophage activation and IL1B secretion which restricts intracellular bacterial growth (By s

H3K4me1) or dimethylated (H3K4me2) (PubMed:27735137). Also mediates deamination of me
nucleus.

gainst C.albicans. Contains two BBXB heparin-binding consensus sequences that appear to fo

sis (By similarity).
stream signaling molecules. Following activation by ligand, interacts with the PI3-kinase subuni

PubMed:30374167, PubMed:30385749). Crotonylation marks active promoters and enhancers

, blood pressure, endocrine, immune, cardiovascular, and renal function. Consists of two major

esis, organization and transport.
ral RNA, resulting in the inhibition of protein synthesis, thus terminating viral replication. Can m

›s MAPK4 and MAPK6 and activates the downstream target MAPKAPK5, a regulator of F-actin
2- dependent stereospecific hydroxylation (with S stereochemistry) at the alpha-carbon (C-alph
are key regulators of many important physiological processes. Plays an important role in growt

nay play a role in endothelial cell-cell adhesion via cell junctions (PubMed:27958302). Heteropl
e acids into the canaliculus, uptake of bile acids from intestinal contents into intestinal mucosa

sphate reabsorption by cleaving DMP1- and MEPE-derived ASARM peptides (PubMed:18597

hatidic acid, sphingomyelin, phosphatidylcholine, phosphatidylinositol, phosphatidylglycerol, ce

eneration of spliced peptides resulting from the ligation of two separate proteasomal cleavage
-cell adhesion and cell migration (PubMed:23704371, PubMed:30367560, PubMed:23867277).
insulin receptor (IR) signaling in skeletal muscle. Regulates insulin-induced tyrosine phosphory

roteoglycan GPC2. Required for normal brain development, especially for normal developmen

etes with GRK2 for binding to LPAR1 thus affecting the signaling properties of the receptor. R

nsin. May promote mineralization of the extracellular matrix during osteogenic differentiation by

all during the disease process of atherosclerosis (PubMed:8107690).

gy, including cancer and various autoimmune diseases (PubMed:21376174). CCL20 acts as a

ier integrin ligands to site 1 (PubMed:23125415, PubMed:24789099). The soluble form is chem

on and bud growth in metanephroi (By similarity). Inhibits WNT1/WNT4-mediated TCF-depend

1685497, PubMed:23184945). Mediates reorganization of the actin cytoskeleton and axon grov

ription, and might thereby contribute to pT reg cell differentiation in the spleen and lymph node

Due to functional redundancy between members of the SRC kinase family, identification of the

ocal adhesions. Supervillin-mediated down-regulation of focal adhesions involves binding to TR

n and activity in lysosome (PubMed:31036939). Involved in the regulation of lysosomal morpho

romote 'Lys-63'-linked ubiquitination of target proteins. Inhibits activation of NF-kappa-B in resp

charide structures (PubMed:8643456). Glycosphingolipids are essential components of membra

[provided by RefSeq, Nov 2008]
on sensitizes cells to TNF-induced apoptosis. Is a potent inhibitory factor for osteoclast differen

0). Phosphorylates: PPP1R12A, LIMK1 and LIMK2 (PubMed:11340065, PubMed:11399775). I
omotes attachment, spreading and dendrite outgrowth in melanocytes.

The eIF-3 complex is also required for disassembly and recycling of post-termination ribosomal

progression after DNA replication arrest (PubMed:20883816).

ome-like induced structures). Along with WDFY3, required to recruit ubiquitinated proteins to F

Med:29212815). Regulates neuronal process morphology in the intact central nervous system (

during T-cell activation (By similarity). Down-regulates CD2-stimulated adhesion through the co

osomal tethering complexes which is proposed to be involved in the Rab5-to-Rab7 endosome c

s, where it is required for transmission and restoration of repressive histone marks, thereby pre
wis X and in lymphocyte homing to Peyer patches. Has no activity toward O-linked sugars. Its su

cts by competing with calcineurin/PPP3CA for NFAT protein binding, hence preventing NFAT a
ession and beta-cell function. Appears to have a role in cell signaling in mature and developing

of CLASP1 and CLASP2.

receptor ITGA5 ITGB1 to the plasma membrane that enables continuous directional cell migr
negative feedback response to attenuate NF-kappaB activation through the deubiquitination of

volved in the BCAR1/CAS-mediated JNK activation pathway.||Acts as an adapter protein that n

ne inflammatory response. Recognizes mycoplasmal macrophage-activating lipopeptide-2kD (I

I2]

1LC3B. In turn, protects the cell against oxidative stress-induced cell death as a consequence
tion promotes epithelial-mesenchymal transition (EMT) leading to ERZ phosphorylation and trig
acts as a putative regulator of mRNA stability and/or translation rates and mediates mRNA nu

may act as a tumor suppressor for melanoma. Represses the CLOCK-ARNTL/BMAL1 heterod

milarity).

'ubMed:30220460).

f the MB1 domain in the myelin basic protein (MBP) promoter (By similarity).

rentiation. May also be involved in mitochondrial fission by converting phosphatidic acid to diac

bMed:12676087, PubMed:12699629). Binds to the single-stranded 5'-(GTGTGG)(4)GTGT-3' te
:ing its activity and allowing activation of the Shh signaling. Its ligand is unknown (By similarity).

ɔnic brain development (By similarity). Regulates the migration of cerebellar granule cells in the
rowth factor-mediated signaling cascades. Required for normal organization of the cardiac base

HIV-1: dNTPase activity reduces cellular dNTP levels to levels too low for retroviral reverse tra
er-order RECK-ADGRA2-Fzd-LRP5-LRP6 complex (PubMed:30026314). ADGRA2-tethering fu

ɔMed:16143324). Has a role in regulation of the actin cytoskeleton, cell polarity and cell adhesi
ɔls the major intracellular degradation process by which cytoplasmic materials are packaged int

G-proteins (By similarity).

y).

ved to release an N-terminal fragment of APP (N-APP). N-APP binds TNFRSF21; this triggers

n macrophage pyroptosis, a caspase-1-dependent inflammatory form of cell death and is the n

e biogenesis disrupt the interaction between NOP53 and RPL11 allowing RPL11 transport to tl

on. May function as an oxygen sensor regulating the KCNK3/TASK-1 potassium channel and H

gulator that translocates to the nucleus) and the promotion of apoptosis induced by tumor necr

ase activity of DNM1. Promotes DNM1 oligomerization. Promotes activation of the Arp2/3 com

tty acid oxidation (By similarity). At the villus tip of the crypt-villus axis of the small intestine ma

sidering that its appropriate peptide substrate may remain unidentified.

256955).

ılso named APP-CTF) by the gamma-secretase complex to generate amyloid-beta, while it red

role in the response to hypoxia depends on the cell type; it mediates mTORC1 inhibition in fibr
ation complex. Under hypoxic conditions, the hydroxylation reaction is attenuated allowing HIFs

rrest. Isoform 1, but not isoform 2, has the ability to induce cell cycle arrest in G1 and maintain

(By similarity). Acts as a negative regulator of central nervous system angiogenesis. Inhibits sp

r conditions localizes to the ER membrane and in response to excess cholesterol in the PM is

to the activation of the enzyme (PubMed:27650958, PubMed:27373336). Activates deubiquitin

ation of NFE2L2/NRF2, glycation impairing NFE2L2/NRF2 function (By similarity). Also able to

. In this way, SMAD2 may recruit substrates, such as SNON, for ubiquitin-mediated degradatio

:he proteaseome (PubMed:30190310). The ability of the SCF(FBXL17) complex to eliminate cc

), PubMed:25527291). Targets PRKN to dysfunctional depolarized mitochondria through the pl

ıibits the renal K(+) channel, KCNJ1, via a kinase-independent mechanism by which it induces

reases basal and insulin-stimulated glycogen synthesis upon overexpression in hepatocytes (B
oinositol 5-phosphate) via the same basic sequence motif that mediates DNA binding and nucl

Med:21964062). Required for ZFP36 localization to cytoplasmic stress granule (SG) and P-boc

ıoform 2 appear to be redundant in their ability to modulate CD4 T-cell responses. Isoform 2 is
s and mediating constriction of the apical endfoot (By similarity). In addition, may also act as a
ıiamine pyrophosphate and contribute to host thiamine (vitamin B1) homeostasis (PubMed:243

ılation of PIAS3, either by degradation of PIAS3 through the ubiquitin-proteasome pathway or e

ving OFD1 from centriolar satellites via the autophagic pathway. Through its interaction with th
ı NK cell cytotoxicity by controlling polarization of microtubule-organizing center (MTOC), and p

asomal degradation. Binds the DNA sequence 5'-AACAAT-3'.

:h PCNA (PubMed:24695737).

/ play a role in neurite outgrowth.

velopment, granulosa cell differentiation and luteinization (PubMed:21321049, PubMed:22229[illegible]

eq, Feb 2016]

ıtes the centrosomal levels of kinase AURKA/Aurora A during mitotic progression by promoting

tors or insulin. Mediates negative regulation of phagocytosis, mast cell activation and dendritic

:ase activity (PubMed:19945436, PubMed:19389623).

ylates glycero-3-phospho-1D-myo-inositol 4,5-bisphosphate to glycero-3-phospho-1D-myo-inos

ng different isoforms. [provided by RefSeq, Jul 2014]
ity and MAP3K8-mediated signaling. Acts as a negative regulator of MAP3K3-mediated activat

ls unsaturated long-chain ceramides, including C18:1-, C20:1- and C24:1-ceramides (PubMed:

rowth factor signaling (By similarity).||[Isoform 1]: heparan sulfate basal lamina glycoprotein tha

NAH (involved in signaling); or MAPT and PXN (microtubule-binding proteins). Phosphorylation
nctions (By similarity). May also function as a transcriptional coactivator, stimulating transcriptio

2, PubMed:23416111). Plays a role in controlling cellular ATP levels by regulating phosphorylat
gher frequency of acute alcohol intoxication among East Asians than among Caucasians could

circuits, endocytosis regulation may influence the internalization of PHF-tau aggregates (By si

factors such as MAPK8/JNK1. Required for histone H4 acetylation at double-strand breaks (DS

he brain and elicit inflammatory responses. Promotes both tau aggregation and TPK II-mediate

racellular iron homeostasis, thus triggering the activity of Fe(2+) prolyl hydroxylase (PHD) enzy
x ATRX:DAXX which has ATP-dependent DNA translocase activity and catalyzes the replicatio
o modulate mRNA processing and RNAP II stability by inhibiting pre-mRNA 3' cleavage.

ound for this gene. [provided by RefSeq, Jul 2008]

gnaling cascades downstream of NTRK2 (PubMed:11152678). During development, promotes

: May induce the production of pro-inflammatory cytokines such as IL6 in monocytes.||Plays ar
families, including retroviridae: human immunodeficiency virus type 1 (HIV-1), human immunoc

ncluding murine leukemia virus, polyomavirus enhancer, T-cell receptor enhancers, LCK, IL3 a
activation. May play a role in membrane transport and/or integrin dependent signal transductio

atopoietic cells). Essential for cell proliferation within the dentate gyrus of the hippocampus an
transactivation and apoptosis. Also induces G2 arrest and apoptosis in a p53-independent ma

123922). Maintains partially unfolded proteins in a state appropriate for subsequent refolding b
cell apoptosis.

und to be much more dynamic than E-cadherin/catenin complexes and CTNNA1 was shown n

ssDNA, while the 3'-ssDNA cleavage is prevented by the presence of RPA. Also involved in DN

e extracellular matrix (ECM), the migration and invasion of endothelial cells into the ECM. May

nine-dependent and -independent transcriptional activity of the thyroid hormone receptor gene

forms exhibit the same level of activity (By similarity).
E2 secretion (PubMed:21242590).

purinic/apyrimidinic (AP) site-terminated flap. Acts as a genome stabilization factor that preven
f several signaling cascades. Activation of PLCG1 leads to the production of the cellular signal

n promoting cell proliferation seems to be cell-type specific. Promotes PGF-mediated prolifera

ɔt with homeostatic chemokines. Acts as a receptor for chemokines including CCL2, CCL5, CC

ain cancer cell types. In elastin producing cells, associates with tropoelastin intracellularly and fı

3791, PubMed:12526812). Regulates protein trafficking to lysosomes and, also the activity of I

; by phosphorylating and inhibiting GYS1 activity and hence glycogen synthesis. May also med

e-rich initiator (Inr) and an upstream E-box. Required for the formation of functional ARID3A DN
chain fatty acids (PubMed:30850536). Mitochondrial trifunctional enzyme is a heterotetrameric
ubMed:30850536). Mitochondrial trifunctional enzyme is a heterotetrameric complex composed
CREST-mediated transcription in resting neurons. Upon calcium stimulation, HDAC1 is release

ith ARNT; heterodimer binds to core DNA sequence 5'-TACGTG-3' within the hypoxia respons
402688, PubMed:7504010, PubMed:17189421, PubMed:20364150, PubMed:25880248, PubM
including a peptide derived from the signal sequence of HLA-DPB1 (APRTVALTA) (PubMed:7

mRNA splicing. N6-methyladenosine (m6A) has been shown to alter the local structure in mRN

ɔers inhibit signaling via G proteins and mediate activation of alternative signaling pathways. Re
sor cells proliferation. Inhibits the binding of E2A-containing protein complexes to muscle creat

alpha- and beta-subunits themselves (PubMed:7665574, PubMed:21854986). Can form an ac

various histone methyltransferase complexes. In embryonic stem cells, it associates with the Pl
upon acidification of the extracellular medium (PubMed:21653227, PubMed:22431633). The hc

7). Activated by upstream kinases including ROCK1, PAK1 and PAK4, which phosphorylate LI

a role in gamete interaction.
mplex-type N-glycans, such as those found on EGFR, TGFR (TGF-beta receptor) and CDH2 (F

ity toward skin preelastic and elastic fibers (PubMed:20876573).
hat is involved in diverse functions such as remodeling of the vasculature, angiogenesis, tissue

oduction by the 3MST/CAT pathway is regulated by calcium ions.

y of the Golgi apparatus via the p53-dependent pro-survival pathway (PubMed:16507995). App

the DNA of their target genes and the individual dimers have distinct preferences for different
omeostasis. In collaboration with RELA/p65 enhances NFATc1 promoter activity and positively

trix by up-regulation of the collagenases MMP1, MMP2 and MMP13, and thereby facilitates cel

neurons of the central nervous system. HCNP increases the production of choline acetyltransf[illegible]

al mRNA and protein synthesis. The cytoplasmic isoform PML-14 can restrict herpes simplex v[illegible]

odimerization with RXRA and is antagonized by NR2C2. May be required for the propagation of
s in cofilin dephosphorylation (PubMed:15671020). In response to increased Ca(2+) levels foll[illegible]
t PRIM2) is recruited to DNA at the replicative forks via direct interactions with MCM10 and WD[illegible]
n regulators. Also acts as a regulator of cellular polarity by remodeling the actin cytoskeleton; p[illegible]

arrest by positive and negative regulation of the cell cycle. Can promote cell growth by phosph[illegible]
ation and vascular-like structure formation.
(2+) or ZN(2+) for the ascorbate-mediated GPC1 deaminase degradation of its heparan sulfate

everal alternatively spliced transcript variants have been found for this gene. [provided by RefS[illegible]
factors (IGFs) by cleaving IGF-binding proteins. Inhibits signaling mediated by TGF-beta family
ctivated complex, rather than by solubilizing the substrate.||Saposin-B stimulates the hydrolysis
ns as a calcium-leak channel that allows the passive movement of calcium from endoplasmic r[illegible]

om phosphatidylinositol 3,4,5-trisphosphate, phosphatidylinositol 3,4-diphosphate, phosphatidy[illegible]

cycle progression, cell proliferation and apoptosis. Required for early embryonic development

ransfer of a fatty acyl group from glycerophospholipid to the hydroxyl group of lysophospholipic
l ATM, promotes ATR activation and its recruitment to DSBs in the S/G2 phase facilitating the

y (DNAH11, DNAH9 and DNAH5) (By similarity). Together with RFX6, participates in the differe

bMed:16213824). In fibroblast, is required for EGF-stimulated phosphorylation of CREB1 and I

arge neutral amino acids such as phenylalanine, tyrosine, L-DOPA, leucine, histidine, methionir
um. SLC8A1 mediates the export of Ca(2+) from the cell during the next phase, so that cytopla

natin remodeling complex (npBAF complex) and the neuron-specific chromatin remodeling com

enters the nucleus (PubMed:28753426). ISGF3 binds to the IFN stimulated response element
ncy (PubMed:11006110, PubMed:19589875). Does not sulfonate cortisol, testosterone and do

activator of Notch pathway by mediating cleavage of Notch, generating the membrane-associa

g to its target gene promoters (PubMed:20176812).
extracellular matrix production, immunosuppression and carcinogenesis. The formation of the
ularly efficient epithelial polarization and barrier formation (By similarity). Plays a role in the regu
lternate polyadenylation sites has been found for this gene. [provided by RefSeq, Jul 2008]

-apoptotic activity is activated via its interaction with PPP1R13B/ASPP1 or TP53BP2/ASPP2 (F

ıanisms of action include direct activation or repression, indirect activation or repression via co[illegible]

f IFIH1 (PubMed:30193849). Promotes ISGylation of 14-3-3 sigma (SFN), an adapter protein i[illegible]

forebrain during embryonic brain development. Binds the endoplasmic reticulum resident rece[illegible]

o. Has no hyaluronidase activity in granulosa cells in vitro.

[illegible]Med:7649300, PubMed:11825900). Promotes KCNA4 channel closure (PubMed:7649300, P[illegible]
[illegible]d viral entry, MARV and EBOV GP1,2-mediated viral entry and SARS-CoV S protein-mediated

[illegible]noyl)-ethanolamine phosphate, a potential physiological compound (PubMed:9607309). Throu[illegible]
pendent activation of TLR8 (PubMed:31778653). Plays also a key role in degradation of mitoc[illegible]

[illegible]entricular wall damage.
ıs are recognized by RNF146, which mediates their ubiquitination and subsequent degradation

[illegible]ivation. Plays a role in glomerular cell migration. Plays a role in pathological neovascularizatio[illegible]

ı pathway. May have an important role in skeletogenesis during postnatal growth (By similarity)

nembrane integrity and in cortical lamination (By similarity). Binding to the COL3A1 ligand inhib

e induction of neurite outgrowth. Functions as a protease inhibitor by blocking access of secret

embrane proteins, such as stimulated growth factor receptors, lysosomal enzymes and lipids. I
12024). Mediates the NR1D1-dependent repression and circadian regulation of TSHB expressi
· actin, leading to increase MKL1/MRTF-A presence in the nucleus and promote SRF:MKL1/MI

ıd cGMP (PubMed:23800469, PubMed:10801446) or not (PubMed:10608844, PubMed:10548
slocation (RAT) (PubMed:27499293).
s neurite outgrowth in newborn neurons migrating to reach the cortical plate. May activate or re

ıg apoptosis (By similarity). Inhibits PTK2/FAK1 and PTK2B/PYK2 kinase activity, affecting the

owing the expression of myocyte enhancer factors. Involved in the MTA1-mediated epigenetic
e-3 activity.

›inding/depolymerizing factor cofilin. Decreased cofilin activity may lead to stabilization of actin

ore vesicles fusion as well as controls the location and efficiency of their synaptic release (By s
the interaction between FBN1 and ELN (PubMed:17255108).

-mediated viral entry, MARV and EBOV GP1,2-mediated viral entry, SARS-CoV S protein-medi
major coding-region determinant of instability (mCRD) domain.

is stimulated in response to Ca(2+) signals (By similarity). Seems to be involved in hippocampa
›rm (PubMed:20381137, PubMed:24095279, PubMed:25936802). In its active form participates
ant role in neuronal signaling, including in the parasympathetic, but not sympathetic, control of l

d by RefSeq, Jan 2017]

s3P activity (PubMed:28561066).

en APBA2 and APP. The intracellular fragment AlcICD suppresses APBB1-dependent transact

eurotransmitters.

s actin filament reorganization via its interaction with the Arp2/3 complex. Actin remodeling acti
he closed structure of the proteasome via an active gating mechanism. Component of the sper

'R of diverse inflammatory mRNAs (such as ceruplasmin) and suppresses their translation. In t
iated endosome fission: localizes to endosome membrane tubules and promotes recruitment c
y anchor the podocyte slit diaphragm to the actin cytoskeleton in renal glomerolus. Also require

ie up-regulation of the oxytocin receptor gene at parturition (PubMed:10527846).

adation are inhibited by calmodulin. Through CCND2 and CCND3 degradation induces cell-cyc

uits the PRC2 complex to CpG islands and contributes to embryonic stem cell self-renewal. Als

ul7-RING(FBXW8) complex recognizes and binds IRS1 previously phosphorylated by S6 kinas

75408). Then switches as an inhibitor of APC-FZR1 complex during S and G2 leading to cell-c

rmed cells when is overexpressed (By similarity).

8347093, PubMed:26647308). Can act with CTBP1 to synergistically repress transcription but

s a key role in m6A methylation, possibly by binding target RNAs and recruiting the WMM comp

teracting with cellular inhibitors of apoptosis proteins (cIAPs) and thereby inhibiting their recruit

A/NFKB3 target genes when complexed with RELA (PubMed:15029197). May also specifically

m the 43S pre-initiation complex (43S PIC). The eIF-3 complex stimulates mRNA recruitment t
ription regulators. Also acts as a regulator of cellular polarity by remodeling the actin cytoskelet

rtation, an essential protein for cholesterol absorption, to the plasma membrane by recruiting M
ıbMed:23303788). May repress COX4I2 transactivation induced by CHCHD2 and RBPJ (PubM

ons.
The resulting loss of PPARG target gene transcription inhibits adipogenesis and promotes oste

embles T loops and prevents telomere fragility by counteracting telomeric G4-DNA structures, w
des with charged C-terminus. May play a role in the inactivation of peptide hormones. May be
7740623).
ugh interaction and nuclear translocation of EYA protein. Acts synergistically with SIX1 to regul
the nucleus via the importin alpha/beta heterodimeric import receptor. The directionality of nuc

major types of neutral root structures of glycosphingolipids, that constitute a significant portion o
iates epithelial estrogen responses in the uterus by regulating ESR1 levels and activation. Imp

SNRPE, SNRPF and SNRPG are trapped in an inactive 6S pICln-Sm complex by the chaperon

strates that lack 4-hydroxyproline in the third position (PubMed:18487197).
ed:29335528). Also involved in the response to DNA damage (PubMed:26807646, PubMed:31
y influence spermatogenesis and male fertility.

e protein translation of DENV RNA via its association with cellular mRNA-binding proteins and
precursor of the second messenger inositol 1,4,5-trisphosphate (InsP3).

ne endosomal membrane involves RAB7A and SNX3. The CSC seems to associate with the cy
to Golgi exit of the ATP8A2:TMEM30A flippase complex. ATP8A2:TMEM30A may be involvec

nd oligosaccharides containing the Galbeta1-4GlcNAc. Catalyzes 6-O-sulfation of beta-benzyl (
sion and polar body extrusion during female germ cell meiosis (By similarity). Plays a role in re:

:20:4-CoA (PubMed:21173190). Has a preference for arachidonoyl-CoA as a donor (By similari

, blood pressure, endocrine, immune, cardiovascular, and renal function. Consists of two major
omal membrane involves CCC and WASH complexes (PubMed:28892079). In the endosomes
s and lipids. The MVB pathway appears to require the sequential function of ESCRT-O, -I,-II ar

the degradation of the 'Thr-182' phosphorylated form of RIPK3 (PubMed:29883609). Negative

rucial role in pulmonary surfactant homeostasis (By similarity). Has an anti-fibrotic activity by ne
oolymerizing microtubules (PubMed:23085020).
t sites (EPCS) (PubMed:27044890).

:16354688). Plays a role in postnatal myogenesis, may be involved in the regulation of satellite

during cilium assembly (PubMed:23400999). Regulates the centrosomal location of ODF2 and

on of specific target genes. Plays a critical role in chondrogenesis by activating the transcription

Binds phosphoinositides (PubMed:19168031).
adation of enzymes involved in gluconeogenesis, such as FBP1 (PubMed:29911972).

the type Pro-[Ala,Gly]-[Leu,Met,Gln,Ser,Tyr]-[Glu,Gly,His,Ser,Val,Trp,Tyr]. Does not bind peptic

as it represses the expression of PER1 and in turn is repressed by PER1/2 and CRY1/2. Repr

ys a key role in polyploidization of cells in placenta and liver by regulating the endocycle, proba

fferentiation through down-regulation of EGFR/AKT/MAPK and Wnt signaling pathways (PubM

ucose-dependent insulin secretion in pancreatic beta-cells during the first phase of insulin secr

mplex that selectively accepts ubiquitin from UBE2H and mediates ubiquitination and subseque

ulum and negatively regulates its expression at the cell membrane.

inhibit VEGFA-mediated signaling by blocking the interaction of VEGFA to its receptor KDR/VE

/ indirectly stimulate the activity of transcription factor KLF7, a regulator of neuronal differentiat

ıesis during embryonic development.

gnaling pathway (PubMed:26438880).

›wnstream effector RAD51 to DNA damage sites including DBSs and ICLs (PubMed:23401855

n formation, possibly by being involved in the transport of raft lipids to the apical membrane, ar
/ by acting as an important enzyme for intracellular Ca(2+) mobilization in the zona pellucida-in

nhibitor of ZNRF3, an important regulator of the Wnt signaling pathway. Acts as a ligand for friz

SK3B activation and phosphorylation of DPYSL2/CRMP2 followed by destabilization of microtu
the distal end of the mother centriole where they fuse to initiate cilium assembly. Involved in mi

ıt mechanism (PubMed:15492226, PubMed:26068852). TIFA-dependent innate immune respo

ɔlved in adaptor protein complex AP-2-dependent endocytosis of the transferrin receptor, it als

brane cleavage of the envelope glycoprotein gp130 and/or the leader peptide gp18LP of the si

ɔeta- and alpha-anomers of galactose, therefore ensuring a sufficient supply of the alpha-anom

actin filament networks. Also catalyzes actin polymerization in the absence of Arp2/3, creating

3 and RIPK1, which in turn, lead to the activation of transcription factors IRF3 and IRF7, NF-ka

ns epithelial cells (By similarity).

. The resultant detrimental levels of mutations in the proviral genome, along with a deamination

fSeq, Nov 2014]

it is not an anti-apoptotic protein per se.
membrane tubulation (in vitro). May promote the formation of macropinosomes.

l:15657036, PubMed:15907468, PubMed:25931126, PubMed:16368756). Known substrates in

to the Golgi apparatus. Involved in the transport of Shiga toxin from early and recycling endos

rated stress response (ISR) required for adaptation to amino acid starvation (By similarity). EIF

nd vertebrae. Required for proper anterior-posterior somite patterning. Proposed to function in

cleavage, continues to digest histones tails via its aminopeptidase activity (PubMed:28847961
NA), which is further cleaved by the cytoplasmic Dicer ribonuclease to generate the mature miF

. Activin is also thought to have a paracrine or autocrine role in follicular development in the ov

l activation and proliferation via its interactions with CD6 (PubMed:15048703, PubMed:163528

interaction with RNH1 in vivo.

gulation to maintain genomic stability. Regulates centrosomal microtubule nucleation. Requirec
pair by HR. May participate in S phase checkpoint activation. Binds selectively to ssDNA, and to

moting PIK3R1/p85 ubiquitination, which impairs its recruitment to the TCR and subsequent ac

plex mediates the import of proteins such as TIMM23, SLC25A12/ARALAR1 and SLC25A13/AI

lament capping and actin bundling activities depending on the context. Displays barbed-end ac

kpoint-mediated arrest of cell cycle progression in response to low doses of ionizing radiation a

ers by enhancing transcription factor binding and/or bringing distant regulatory sequences into c

ively regulates myogenic transcription factor MYOD1 function by facilitating the recruitment of t
to the immunoglobulin mu chain switch region. Preferentially binds to the 5'-GGGCT-3' motif. I

as a more potent activator of the IFN-beta (IFNB) gene than the IFN-alpha (IFNA) gene and pl
lcium sensitivity to KCNMA1. Its presence is also a requirement for internal binding of the KCN

ıre likely to contribute differentially to the complex helicase activity. Required for the entry in S p
ıre likely to contribute differentially to the complex helicase activity.
ıre likely to contribute differentially to the complex helicase activity (By similarity). Interacts with

pterin synthase complex, a complex that catalyzes the conversion of precursor Z into molybdop

ı development, nodes of Ranvier are formed by the fusion of two heminodes. Required for norr

038218).

ssing defects, thereby limiting or excluding their export to the cytoplasm. The RNA exosome m
EJC assembly or during subsequent mRNA metabolism. Participates in the regulation of alterna

base of the clamp element. The RBP4-RPB7 subcomplex seems to lock the clamp via RPB7 in
t could impair cellular functions, and by removing proteins whose functions are no longer requir
ng its affinity for the 3'-OH end of the primer-template and stabilizes POLB to those sites where

and DNA damage checkpoint activation. Through recruitment of ATRIP activates the ATR kina
ocated in the cytoplasm. Variable expression of this gene in colorectal cancers compared to ac

alt sensitivity of peripheral glucose uptake, cardiac repolarization and memory consolidation. U
ole formation and proper mitotic progression. During procentriole formation, is essential for the
OXO1, UBP43, and probably MYC, TOB1 and TAL1. Degradation of TAL1 also requires STUB
90923). Mediates Cl(-) flux that is not coupled to amino acid transport; this avoids the accumula
ds, and excludes methylated, anionic, and cationic amino acids (PubMed:8702519, PubMed:2
cular chaperone in its multimeric membrane-bound state, assisting in the folding of synaptic fus

and activated spliceosome C complexes (PubMed:11991638, PubMed:28502770, PubMed:28
sist the organization and assembly of outer dense fibers (ODFs), a specific structure of the spe

al anchors leads to the formation of the SNARE complex, which brings membranes into close p
' trimethylation is also required to direct DNA methylation at pericentric repeats. SUV39H1 is ta

inducer T-cells (By similarity). TLE1, TLE2, TLE3 and TLE4 repress transactivation mediated

erefore, species-specific (PubMed:20711192). Both M.tuberculosis HSP70 (dnaK) and HSP65
P) and Thymopentin (TP5) may play a role in T-cell development and function. TP5 is an immu
round the unbroken strand thus removing DNA supercoils. Finally, in the religation step, the DN

)1, PubMed:15634918, PubMed:17030620, PubMed:16702957, PubMed:20702587, PubMed:2

)071723, PubMed:29900004). Also able to recognize and bind crotonylated histone H3 (PubMe
3, PubMed:12117417, PubMed:12225859, PubMed:30867591). May play a role in the transport

ie phosphate, a potential physiological compound (PubMed:9607309). Through its extracellular

tabilizes replication forks and influences both CHEK1 phosphorylation and the intra-S phase ch

rity). Required for correct spindle formation and has a role in regulating cytokinesis and genom

of PPP1R12A subunit of myosin PP1: phosphorylated PPP1R12A then interacts with 14-3-3, le
of mTORC1 and mTORC2 complexes, which regulate cell growth and survival in response to r

29769719, PubMed:11961546, PubMed:22748924). CRLs complexes and ARIH1 collaborate i
dependent cell motility by mediating deacetylation of tubulin (PubMed:12024216, PubMed:203
ing cells by bringing newly synthesized cbx proteins to heterochromatic DNA replication foci.

nportant role in the regulation of cholesterol homeostasis, regulating cholesterol uptake throug

olyunsaturated fatty acyl-CoAs as acyl donors compared to saturated fatty acyl-CoAs. Seems
RNF41 is involved in the control of hematopoietic progenitor cell differentiation into myeloeryth

osomal protein S19 during the formation of pre-ribosomes (By similarity).
97582, PubMed:23532825). May be involved in the control of cell growth and differentiation. M

calization to mitotic spindle, but not to centrosomes and CCNB1 localization to both mitotic spi
state. Functions with MYC to activate target gene expression through RNA polymerase II paus

NA (PubMed:10219083, PubMed:10852724, PubMed:11595739, PubMed:16510448, PubMed
during the G1/S transition, leading to inhibit FBXW5 ability to ubiquitinate SASS6. Its central ro
0]

'Lys-5', 'Lys-8' and 'Lys-12' (H4K5ac, H4K8ac and H4K12ac, respectively), regulating DNA rep

lation of HSPA5/BiP (PubMed:25601083). In unstressed cells, acts as an adenylyltransferase
sing factors with their substrates. Component of the cytoplasmic LSM1-LSM7 complex, which i

ssing defects, thereby limiting or excluding their export to the cytoplasm. The RNA exosome m

disease (PubMed:17143291). Inhibits the pro-apoptotic function of KREMEN1 in a Wnt-indep

DNA damage response appears to require the association with TP53BP1 phosphorylated by A

sitioning. Specifically, SYNE2 and SUN2 assemble in arrays of transmembrane actin-associate

;, ultimately affecting neuron pool and cortex size (PubMed:27737959).
ssing defects, thereby limiting or excluding their export to the cytoplasm. The RNA exosome m
, possibly by interfering with the transactivation domain of NFKB1. Induces apoptosis in breast

). Activation of IL17RA leads to induction of expression of inflammatory chemokines and cytoki
selective permeability for Na(+), Li(+) and K(+) ions, but selects against Cl(-) ions. Plays an im

ases has been reported conflictingly.

othyronine and thyroxine.
3955). May function as sterol isomerase (PubMed:25566323). May alter the activity of some cy

e mitoribosome differ greatly in sequence, and sometimes in biochemical properties, which prev

e mitoribosome differ greatly in sequence, and sometimes in biochemical properties, which prev

ts into these complexes.

as a N(6)-adenine-specific DNA methyltransferase by mediating methylation of DNA on the 6th
atenation. Required for decatenation of chromatin bridges at anaphase. Early in neurogenesis,

and DMC1-promoted homologous pairing. May inhibit HIV-1 viral protein TAT activity and moc

3 is not required for tRNA modification (PubMed:22912744, PubMed:28805828).

e mitoribosome differ greatly in sequence, and sometimes in biochemical properties, which prev

on with the MITRAC assembly intermediate containing MT-CO1/COX1, thereby participating in

ent of the CBC complex. Involved in cell cycle progression at S phase. Does not directly confer

estis. Essential for male fertility, spermiogenesis and sperm flagella formation. Plays a role in tl
erase III transcripts, such as Epstein-Barr virus-encoded RNAs (EBERs) induce type I interfero

esulting in release of cytochrome c into the cytosol. Binding of cytochrome c to Apaf-1 triggers

lar body where it mediates KCNH2 degradation (PubMed:26363003). May modulate EGFR sig
erebellum embryonic development (PubMed:21623382).

lliday junction branch migration (in vitro) (PubMed:23973328).

ntra-S phase checkpoint in response to genotoxic stress.

spindle pole, leading to poleward microtubule flux and poleward motion of chromosome.

CENPE and PLK1 to the kinetochores (PubMed:25395579).

nd CEP63 centrosomal localization and promotes the centrosomal localization of CDK2 (PubM

Required for centriole elongation and for STIL-mediated centriole amplification (PubMed:2202C

ably activates the Ca(2+) release-activated Ca(2+) (CRAC) channels ORAI1, ORAI2 and ORAI
nage sites, leading to promote RNA pol IIo backtracking to allow access to the nucleotide excis

vhich monitors the integrity of DNA replication forks.

ity).
oteins. The formation of the ATG8-phosphatidylethanolamine conjugates is essential for autoph

this locus and two splice variants encoding distinct isoforms have been identified. [provided by

g (NHEJ) (PubMed:29042561). Required for the dynamic movement of AURKA at the centroso

low desaturation degree. May participate in the production of saturated and monounsaturate

the cytoplasm via the TAP/NFX1 pathway. The TREX complex is essential for the export of K

has been questioned (By similarity). During immunoglobulin class switching in activated B-cell

ice of the kinetochore PLK1 population until its degradation. Involved in transcriptional repress

methylation at pericentric repeats. SUV39H1 is targeted to histone H3 via its interaction with R

NRPE, SNRPF and SNRPG are trapped in an inactive 6S pICln-Sm complex by the chaperon
NRPE, SNRPF and SNRPG are trapped in an inactive 6S pICln-Sm complex by the chaperon

158). Promotes apoptosis (PubMed:16046545). May act as a renal tumor suppressor (PubMe

tein phosphatase 1. May play a role in cerebellar development and synaptogenesis.

estrone-3-sulfate, dehydroepiandrosterone sulfate (DHEAS) and pregnenolone sulfate).

ion. May induce apoptosis in collaboration with APAF1 (PubMed:25716227).
-like domain-containing extracellular proteins with high affinity. Only shows kinase activity whe

lesions (PubMed:25931565).

substrates, introducing single-strand cuts in duplex DNA close to junctions with ss-DNA. Has a
in favor of neural crest specification (PubMed:26399832).

ıbMed:27185865). Also required for centriole-to-centrosome conversion during mitotic progres:

of DCAF15 by acting as a molecular glue that promotes binding between DCAF15 and weak a

e-emptive quality control, a mechanism that selectively attenuates the translocation of newly sy

35020).

um, it is considered to be a candidate for tumor vascular targeting. [provided by RefSeq, Jan 20
es. [provided by RefSeq, Jul 2008]

a unique regulator of gene expression in several different embryonic and postnatal cell lineages

actin flow (By similarity). Overexpression can recruit Ran GTPase to the nuclear periphery (Pub
ibits cell growth and tumorigenicity of medulloblastoma (MDB) (PubMed:21472142). Involved in

h microtubules as oligomeric assemblies (PubMed:19289083). The complex facilitates the proc
-coupled manner (PubMed:19289083). In the complex, it mediates the microtubule-stimulated

high fat diet, palmitoyl- (C16:0-) ceramides generated by CERS6 specifically bind the mitochon

IK signaling cascade via its interaction with FZD4 and VANGL2.
NVS may downregulate the canonical Wnt pathway and promote the Wnt-PCP pathway by reg
play a critical role in cell-cell adhesion and recognition processes.

ctural features of the tRNA body. The tRNA splicing endonuclease is also involved in mRNA pr

assembly is proposed to coexist in a dynamic equilibrium may suppress nascent cilium assem

:oordinated incisions that lead to the formation of ligatable nicked duplex products. Cleavage o

:ell migration (By similarity). In T-cells involved in endosome-to-membrane recycling of receptor

NA), which is further cleaved by the cytoplasmic Dicer ribonuclease to generate the mature miF

NA), which is further cleaved by the cytoplasmic Dicer ribonuclease to generate the mature miR
d the three components separate and importin-alpha and -beta are re-exported from the nucleu

3) complex also mediates ubiquitination and degradation of CLDN8, a tight-junction protein requ
plasts. May be involved in regulating spermatogenesis and in the RACGAP1 pathway in neuron

E2-E3 ubiquitin protein ligase complexes in IL-1R and TNFR-1 pathways; affected are at least
cific tyrosine residues on the cytoplasmic tails of the receptor, creating docking sites for STATs

synergistically enhances the affinity of the SKA1 complex for microtubules and may allow the N

importin-alpha (PubMed:26165940). At the onset of mitosis, GOLGA2 interacts with importin-a

the purine nucleotide metabolism, thereby regulating the metabolic function and bioenergetic s
mon sequence) DNA sequence in the promoter region of interferon regulatory factor 1 and p53

ubMed:24854087). During cell cycle progression acts through SCF-dependent proteasomal ubi
rk to coordinate DNA replication with DNA repair and DNA damage tolerance pathways (PubM

NA), which is further cleaved by the cytoplasmic Dicer ribonuclease to generate the mature miR

e during prometaphase to midbody during cytokinesis and participates in the organization of the

) (CASP9). Inhibits cleavage of CASP9-dependent substrates and downstream markers of apo

between liposomes (in vitro) and probably functions as a PA transporter across the mitochondri
thway.
/IGB1 secretion. The active cytokines and HMGB1 stimulate inflammatory responses. Inflamma

nsion (By similarity). As part of the BORC complex may play a role in lysosomes movement an

iondria. Seems to promote the redistribution of BIRC4 from the cytoplasm to the nucleus, proba
nic progression (PubMed:17409436).
is to be required for Wnt-mediated inactivation of GSK-3 kinase. Both pathways seem to involv

NA), which is further cleaved by the cytoplasmic Dicer ribonuclease to generate the mature miF

em. Plays a role in the regulation of aortic epithelial cell morphology, and is required for flow-inc
ng the second into a water molecule, with two electrons provided by NADPH via cytochrome P

GCK and does not inhibit GCK activity (PubMed:23621087, PubMed:23733961).

Plays an important role in the regulation of B-cell differentiation, proliferation, survival and apop
bMed:26091040). Signaling triggers an increase of cytoplasmic Ca(2+) levels (PubMed:19656(

ooperates with SHTN1 to promote both the transition from the multipolar to the bipolar stage a

24070612).
kidative stress by facilitating the SQSTM1-mediated autophagic degradation of KEAP1 (PubMe
ubMed:16707096, PubMed:19204325). This inhibitory effect is selective to endothelial cells as
n and motility through phosphorylation of key regulators of these processes such as CRK, CRK
equisite for the subsequent ligation of the two exon halves and the production of a mature tRNA

d regulation of AMPK activity in modulating MYC and PPARGC1A protein levels and cell growt

ts role in arginine transport.
duced production of pro-inflammatory cytokines by regulating the endosomal TLR3- TICAM1/TI

toxic stresses. May negatively regulate protein translation in response to endoplasmic reticulum

RNA and antisense miRNA star (miRNA*) products. The mature miRNA is incorporated into a R

dation. This is achieved through cycles of ATP binding, ATP hydrolysis and ADP release, medi
RNA and antisense miRNA star (miRNA*) products. The mature miRNA is incorporated into a R

PubMed:27906959). CDT1 degradation in response to DNA damage is necessary to ensure pr

dation. This is achieved through cycles of ATP binding, ATP hydrolysis and ADP release, medi

sion, specifically for cytokinesis and chromosomal segregation (PubMed:26398195). Plays a ro

G5 protein. [provided by RefSeq, Feb 2019]

the activation of several signaling cascades. Activation of PLCG1 leads to the production of t
required for recovery of stalled replication forks, and directly contributes to genomic stability. In
portant role in controlling cell cycle progression and DNA damage-induced G2 arrest (PubMed

RNA and antisense miRNA star (miRNA*) products. The mature miRNA is incorporated into a R

men formation during renal tubulogenesis. Plays a role in cancer development and aggressive

iber differentiation (PubMed:29501879).

ting neurogenesis.

3' in promoter of target genes (PubMed:7957066, PubMed:11782474, PubMed:12533514, Pub

ds that are less well-ordered and rigid compared to nucleosomes containing histone H3 (PubM

579). The transport of pole-proximal chromosomes towards the spindle equator is favored by m
mitosis in transformed, abnormally dividing neonatal cardiomyocytes. Interaction with RB direc

KDM1A and RCOR1, and thereby inhibits demethylation of histone H3 at 'Lys-4' (in vitro) (Pub

asis of cerebral spinal fluid or ciliary function (By similarity). Essential for construction of the cer

ɾNA and antisense miRNA star (miRNA*) products. The mature miRNA is incorporated into a F

·on differentiation (By similarity).

stress: rapidly activated upon stress stimulation, such as ionizing radiation, reactive oxygen sp
ıtion. Besides, required for normal hair development.
microtubules (PubMed:23085020).
ɾNA and antisense miRNA star (miRNA*) products. The mature miRNA is incorporated into a F

an association between the prolyl hydroxylases and VHL enabling efficient degradation of HIF
ɔlex (PubMed:10734143, PubMed:19609301, PubMed:20649465, PubMed:9734359, PubMed:
s triggers cellular activation, with generation of key proinflammatory mediators. Interaction with
KS, which phosphorylates and activates PTK2/FAK, leading to the spread of cardiomyocytes. I

ɩ19). Involved in the regulation of inflammatory response and neuroinflammation. Via the produ

Regulates the circadian clock by inhibiting the basal and circadian expression of the core circa

T3. Dephosphorylates STK4 on 'Thr-387' leading to STK4 activation and apoptosis (PubMed:2(

s; stimulating Schwann cell proliferation; implication in the development of the myocardium suc

epression. Recognizes two classes of DNA response elements within the promoter of its target

provided by RefSeq, Jul 2008]

hibits telomerase and is involved in telomere length homeostasis; it is proposed to bind to newl

-induced type 1 interferon production (PubMed:23871208). Promotes host antiviral responses r

ading to their degradation by the proteasome. Conversely, phosphorylates the Rap activator RA
cleolytic degradation by XRN1. Also acts as an important regulator of the HIF1A-mediated hyp
cardiac precursor cell movements during gastrulation and heart morphogenesis (By similarity)

rapidly recruited to blocked forks and promotes gene conversion at blocked replication forks (P
H3K27me3, which is required for embryonic stem cell identity and proper differentiation.

ates expression of a small subset of genes, including MTFP1 (PubMed:28500257). Regulates [illegible]

[illegible]8906). Mediates apoptosis by activating DAPK1. In the absence of NTN1, activates DAPK1 by

nd EPAS1/HIF2A to bind to hypoxia-responsive elements (HRE) located within the enhancer/p

unwinding the short telomerase RNA/telomeric DNA hybrid that is the intermediate in the telom

-3' of the enhancer II of hepatitis B virus genes, a critical cis-element of their expression and re

/TP53. Also component of the TRIM28/KAP1-ERBB4-MDM2 complex which links growth facto

Mar 2016]
JR77 promoting synaptic differentiation. Associates with chromatin to the ZNF16 promoter.
egrins and activates integrins ITGAV:ITGB3, ITGA4:ITGB1 and ITGA5:ITGB1 in a CXCR4-inde

ctor for polyadenylation activity (in vitro) (PubMed:21788334, PubMed:21855801). Required fo

egakaryocytes and platelets and for bone marrow B-lymphopoiesis. Required for B-cell survival
ss (PubMed:22132193, PubMed:25355627, PubMed:26496226). Involved in the cell cycle chec

K2, RIPK3, RIPK4, CASP3, CASP7, CASP8, IKBKE, TRAF1, and BCL10. Acts as an importa

RNA and antisense miRNA star (miRNA*) products. The mature miRNA is incorporated into a R
: reticulum-associated degradation) and in cell-cycle regulation; Lys-29-linked is involved in lys

C6L, FBXO5/EMI1, FOXM1, KIF20A/MKLP2, CENPU, NEDD1, NINL, NPM1, NUDC, PKMYT1/
ckpoint, a process required to delay abscission to prevent both premature resolution of intercel
ast numbers and controls bone mass (By similarity). Orchestrates the endocrine function of the

RNTL/BMAL1 localization to the cytoplasm. Plays a role in both the input and output pathways
early embryonic development is involved in tissue-specific differentiation processes that are dep

gnal that is required for the myosin contractile ring formation during cell cycle cytokinesis. Requ
KN1B/p27 in the nucleus and exports it to the cytoplasm for ubiquitin-dependent degradation.

i/SCF and KIT promote activation of STAT family members STAT1, STAT3 and STAT5. KITLG

or male and female meiosis.

ismitters. May play a role in vasoconstriction.

RNA and antisense miRNA star (miRNA*) products. The mature miRNA is incorporated into a F
TP53-dependent transcription regulatory function. Positively regulates p53/TP53 and p73/TP73

ıe COP1-binding domain (By similarity). Interacts with MAPK kinases and regulates activation ‹
and for the proper attachment of spindle microtubule to the kinetochore. Necessary for kinetoc

A, with a preference for supercoiled DNA and AT-rich DNA (PubMed:10878551). Does not con

orming a link between spindle microtubule plus-ends and mitotic chromosomes to achieve faith

ıstream effector of CDC42 which mediates CDC42-dependent cell migration via phosphorylatic

sor activity (PubMed:11741002). Post-transcriptional inactivation of REST by SRRM4-depende
ɔn repair pathway (the NER pathway) to initiate DNA repair (PubMed:10882109, PubMed:1127

receptor complex composed of 2 TGFBR1 and 2 TGFBR2 molecules symmetrically bound to

ome is used as a substrate and when not histone octamer is used as substrate.

on of target genes such as PRM1 and TNP1 which are required for packaging and condensati
tivation of RAS, RAF1 and the MAP kinases MAPK1/ERK2 and/or MAPK3/ERK1. Promotes ac

ding protein (C/EBP) function and as an activator of other genes (By similarity). Acts as a domi

ubiquitination and degradation of SGK1 and TNK2. Ubiquitinates BRAT1 and this ubiquitinatio

its and activates the TLR2-dependent signaling pathway (PubMed:26599367). Positively regula

smatched uracil and thymine, in particular in U:C and T:C mismatches. Specifically binds 5-hyd

nisomycin (PubMed:11909979, PubMed:9873047). Plays an essential role in the control of RE
eq, Apr 2014]

RNA and antisense miRNA star (miRNA*) products. The mature miRNA is incorporated into a F
g-2' (H3R2me0), respectively, and recruits chromatin proteins. Enriched in pericentric heterochr

thereby inhibiting the translation of mRNA into protein (PubMed:19736320).
cleus.

RNA and antisense miRNA star (miRNA*) products. The mature miRNA is incorporated into a F

the immune system, thereby facilitating tumor survival (PubMed:28813417, PubMed:2881341(
h ZFYVE9 and SMAD4 and prevent propagation of the intracellular signal (PubMed:20129061,
RNA and antisense miRNA star (miRNA*) products. The mature miRNA is incorporated into a F

an activator on the DR5 element (PubMed:29021580). In concert with RARG, required for ske
and maintains euploidy.

20119, PubMed:9228017, PubMed:9716180). May play a role in the oxidative metabolism of x

30, PubMed:23696226). Upon TCR/CD3 complex stimulation, inhibits TCR-mediated cytotoxici

ALB2-scaffolded HR complex containing BRCA2 and RAD51C and which is thought to play a r

bMed:18434541). Signaling is mediated via effector proteins, such as adenylate cyclase. Inhibi

RNA and antisense miRNA star (miRNA*) products. The mature miRNA is incorporated into a R

ntributes to the down-regulation of p53/TP53 transcription.
n of NF-kappaB transcriptional regulatory activity (PubMed:19684084). Involved in the regulatio

ent auto-regulatory loop.||[SirtT1 75 kDa fragment]: Catalytically inactive 75SirT1 may be involv

ace of activated regulatory T-cells (Tregs) (PubMed:19750484, PubMed:19651619). Required f

RNA and antisense miRNA star (miRNA*) products. The mature miRNA is incorporated into a R
phils to bind pathogens during inflammation (By similarity). Degradation of intravascular NETs b
X chromosome in XX females. May stimulate ubiquitination of histone H2A 'Lys-119' by recruitin

some amplification. Also implicated in triggering apoptosis in polyploid cells that exit aberrantly
plex or cyclosome (APC/C) in complex with its activator CDH1 (APC/C-Cdh1). Necessary for en

the inner centromere and for correcting defective kinetochore attachments. Involved in centro

histone H3 at H3K4 and H3K9. Isoforms 4 and 5 are probably not functional due to the deletion

3K36me2).

A4 and GATA6 and by the neuronal bHLH factors ASCL1/MASH1 and NEUROD4/MATH3 (Pu

ıltiple serine and threonine residues in unstructured domains of proteins, such as SRRM1 and
ıf adipogenic genes. Overexpression leads to induction of smooth muscle marker genes, sugge

ɔr and repressor regulating target genes by binding to an interferon-stimulated response eleme

ite for 5'-splice site cleavage. The tRNA splicing endonuclease is also involved in mRNA proce

ɜrol efflux (PubMed:14570867). May regulate cellular ceramide homeostasis during keratinocyt

ıpholipase in some circumstances, it should be noted that cardiolipin hydrolase activity is either

ınsition of cell growth.

0557084). A second signaling pathway involving PKC and calcium fluxes has been seen for sc

ıay, as PKC seems to be required for Wnt-mediated inactivation of GSK-3 kinase. Both pathwa

ʀNA and antisense miRNA star (miRNA*) products. The mature miRNA is incorporated into a R

e GTPase-activating activity of FLCN, leading to activation of mTORC1 and subsequent cytopl

directs AURKB/C toward substrates near microtubules (PubMed:15316025, PubMed:12925766
al mesenchymal transition (EMT) induction. Binds to multiple functional elements of the humar

X2 P1 promoter transcription activation. Associates with chromatin to the PITX2 P1 promoter re

volved in transcriptional regulation via interaction with MLLT10.

er transcripts has not been determined. [provided by RefSeq, Jul 2008]

to repress p53/TP53-target genes. Also recognizes and binds RB1/RB monomethylated at 'Ly:
ed in cardiac development, in a non-redundant way with ZFPM2/FOG2 (By similarity).
ficiencies. Has broad substrate specificity with manganese or cobalt as cofactor and can act on

eonatal tissues. Seems to be an important facilitator of cell proliferation in certain populations,

s specificity for diacylglycerol substrates with an arachidonoyl acyl chain at the sn-2 position, w
Required for initial activation of CDK1 at centrosomes. Phosphorylates numerous target protein

f pyramidal neurons in the developing cerebral cortex (PubMed:30639322).

nd hydrolysis play a pivotal role in mismatch repair functions. The ATPase activity associated
-series prostaglandins and thromboxanes (PubMed:7947975, PubMed:7592599, PubMed:926

lation at resected ends (PubMed:25642963). POLQ-mediated MMEJ may be required to prom

ıergency circuit' that shapes feeding responses to stressful conditions (PubMed:28953886). Or

00294, PubMed:15793005). Binds DNA (in vitro) and may play a synergistic role with STAT6 ir

ᚱNA and antisense miRNA star (miRNA*) products. The mature miRNA is incorporated into a F

sis.

pression of AP1 transcription factors (PubMed:29043977).
diated phagocytosis through interaction with RAB31 leading to activation of PI3K/Akt signaling.

liver by regulating the endocycle, probably by repressing genes promoting cytokinesis and ant
ᚱNA and antisense miRNA star (miRNA*) products. The mature miRNA is incorporated into a F
leath (PubMed:16839880, PubMed:19713938, PubMed:23726973, PubMed:22887998, PubMe

by NADPH via cytochrome P450 reductase (CPR; NADPH-ferrihemoprotein reductase) (PubM
enhancer, T-cell receptor enhancers, LCK, IL3 and GM-CSF promoters. CBF complexes repres

cogene (By similarity).
genes important for cell proliferation, cell death, and cell migration. SAV1 is required for STK3/

ɔromotes kinesin-1 motility along microtubules and is essential for axon elongation and regene
ɔrogenitor cell self-renewal (PubMed:23812588).

ssion of BLK in the differentiation of IL17A expressing gamma-delta T-cells (By similarity). Prom

e CLNS1A that controls the assembly of the core snRNP. Dissociation by the SMN complex of

e NCOR1/HDAC3 corepressor complex, enhancing transcriptional repression. Recognizes two
s) and ATM-dependent signaling pathways.

: from the centrosomes. Regulates kinetochore microtubule attachment stability in mitosis via p

mbryo development (By similarity). Plays also a role in induced pluripotent stem cells (iPSCs) r

RNA and antisense miRNA star (miRNA*) products. The mature miRNA is incorporated into a R

ı of T-cells (PubMed:17174122).

› block the KCNMA1 tetramer.

ı IL4 expression and promotes alternative activation of adipose tissue macrophages, which are

nt manner. Can also promote cellular transformation through inhibition of the p16 pathway.
tivity of the BCR(KEAP1) complex, promoting nuclear accumulation of NFE2L2/NRF2, heterodi

Med:17088550). Plays a role in the regulation of DNA virus-mediated innate immune response l

ɔs prevent tumor formation. Required for normal progress through mitosis and normal proliferat

ıd gapped DNA. Interacts with and stimulates DNA polymerase POLH. During DNA repair syntl

r the formation of long-term memory. Regulates synaptic plasticity by promoting endocytosis of

ts anti-oncogenic effects in tumor cells through activation and down-regulation of EPHA2. Activ

, CDK7, CEBPB, CHAMP1, DMD/dystrophin, EEF1 proteins/EF-1, EZH2, KIF11/EG5, EGFR,

ophosphatidate/LPA, diacylglycerol pyrophosphate/DGPP, sphingosine 1-phosphate/S1P and

expression (By similarity).||[Isoform Alpha-2]: Has lower transcriptional activation activity than is

PTK2B/PYK2 and SRC. May inhibit differentiation and activity of osteoprogenitor cells. Funct

en the Rab8-GTP localizes to the cilium and promotes docking and fusion of carrier vesicles to
t and is involved in reprogramming vesicle trafficking upon autophagy induction. Involved in the

(PubMed:28525742). During DNA repair, PARP1 recruits PARP9/BAL1-DTX3L complex to DN
transcriptional regulation, cell cycle progression and developmental events. Represses MEF2-
'353360, PubMed:29127155). Engages with a range of client protein classes via its interaction

cient sorting of BACE1 to axons implicating a function in neuronal APP processing (By similarity

)6). Particularly important for the repair of UV-induced pyrimidine dimers (PubMed:10385124, F

d:19915044, PubMed:9153189, PubMed:9162022). Participates in bidirectional cell-cell contac

Seems to positively regulate differentiation of chondrogenic tissue through the growth factor rec
ernalization. Plays an important role in controlling the migration of immune and cancer cells tha

s a transcriptional activator. May be involved in the differentiation and/or survival of late postmit

acylglycerol (MAMAG) (PubMed:28420705).

ne-based microbial antigens are generated via non-enzymatic condensation of metabolite inter
arity).
d recruits it to the GAIT complex that binds to stem loop-containing GAIT elements in the 3'-UT

oviral integration, together exert efficient antiretroviral effects in infected target cells. Selectively

ndicative for an association with the NuRD complex) represses transcription from CCND1/cyclir

ɔtion of antiviral immunological genes, including interferons (IFNs); IFN-alpha and IFN-beta. Re

ıe degradative 'Lys-48'-linked ubiquitination (PubMed:22863753). Inhibits the PI3K-AKT-mTOR

ily members are coexpressed and interact with each other, and this determines the overall perı

ial splicing events combined with optional post-translational modifications give a wide spectrum

ce of superoxide generation via the one-electron reduction of molecular oxygen. Also may cata

ent nucleolytic degradation past a given point (PubMed:11741547, PubMed:9590181, PubMed:

arity).

igration in an actin-, microtubule- and MAP1A-dependent manner (PubMed:20543869). Also in

tion, associates with mitochondria antiviral signaling protein (MAVS/IPS1) that activates the IK
e deacetylation and negative regulation of beta-catenin (CTNB1) transcriptional activity (PubMe

ne response is triggered in response to non-CpG double-stranded DNA from viruses and bacte

may play an important role in gastrointestinal tract water transport and in glycerol metabolism (

oter protein 2 complex 2 (AP-2) in clathrin-coated pits (CCPs). However, the extent of beta-arre

l mapping to both the dorsal lateral geniculate nucleus (dLGN) and the superior colliculus (SC)

evention of STAT1 phosphorylation has been called into question and it has been suggested th
y transporting it from the nucleus to cytoplasm at adhesion plaques. May play a role in myotoni
y synergizes with IL-12 to trigger interferon-gamma/IFN-gamma production of naive CD4 T-cell
nclude retroviridae: human immunodeficiency virus type 1 (HIV-1), moloney and murine leukem

RLR or TLR3 stimulation (PubMed:27538435). In turn, activated TBK1 phosphorylates its dow

PubMed:18191611, PubMed:21620138). Promotes in a STAT1-dependent manner, the expres

bMed:17396150). Together with PARP3, promotes the retention of the LIG4-XRCC4 complex c

e microtubule-dependent intracellular vesicular transport from early endosome to recycling end
active form, thereby promoting neurogenesis in embryos (PubMed:22445366).

ctin ligands, thereby playing a role in inflammatory response (PubMed:10758005, PubMed:126
0699, PubMed:25279697). Required for axon guidance; via its function in O-mannosylation of a
enesis (PubMed:22330140).

ulation. Promotes cytokine production in response to TLR4 and TLR9. Required for antibody cl

st-translational modifications such as H4 'Lys-20' methylation (H4K20me) (PubMed:19818714).

itment. Also a component of the IL-1 signaling pathway modulating IL-1-induced NFKB1 activa

ıic enzymes TYR, TYRP1 and DCT/TYRP2 to melanosomes in melanocytes (By similarity).

ough ARHGEF26/SGEF and RHOG activation.

596, PubMed:16407242, PubMed:16940174). The UV-DDB complex preferentially binds to cy

. MERTK signaling plays a role in various processes such as macrophage clearance of apopto

surface induces dimerization and autophosphorylation of AXL. Following activation by ligand,
dimer combinations act as transcriptional activators or repressors, respectively. NF-kappa-B is

:31273098).

sed intracellular Ca(2+) levels in isolated first trimester trophoblasts. Kp-10 is a paracrine/endo

ınocyte migration in the epidermis (PubMed:23333244).

B2 binding to endocytosis accessory EH domain-containing proteins such as EPS15, EPS15L

ntains an arachidonoyl group (PubMed:15544348). Also involved in the production of alkyl-lyso
rogenitors implicating LINC complex association with SUN1/2 and probably association with cy
immunity. In smooth muscles, its activation by high level of Ca(2+), caused by ryanodine rece

led:27929370, PubMed:29899144). May also play a role in regulating glucose homeostasis anc

ninating viral replication (PubMed:10464285, PubMed:9880569). Can mediate the antiviral effe
with the binding of the transcription factors HIF1A or STAT2 and the p300/CBP transcriptional
n mediate the antiviral effect via the classical RNase L-dependent pathway or an alternative ar

d AP1. Acts as a mediator of transrepression between nuclear receptor and either AP1 or NF-k

to couple cadherins to the actin cytoskeleton (By similarity).

en the Rab8-GTP localizes to the cilium and promotes docking and fusion of carrier vesicles to

ernative ACTL6B/BAF53B and DPF1/BAF45B or DPF3/BAF45C subunits in neuron-specific co

nulating lysosomes. The physical interaction of SPATA18/MIEAP, BNIP3 and BNIP3L/NIX at tl
phosphorylating SMAD3 in a cell-cycle-dependent manner and repressing its transcriptional ac

this gene may be associated with microcornea, myopic chorioretinal atrophy, and telecanthus
ovides succinyl-CoA to histone succinyltransferase KAT2A (PubMed:29211711).
ed by ATF4, TRIB3 protein interacts with ATF4 and inhibits ATF4 transcription activity (By simil
release (PubMed:17418785).||Isoform Bcl-X(S) promotes apoptosis.||Potent inhibitor of cell de

nding to polyubiquitin. Involved in leukocyte integrin activation during inflammation; this functior

ates the opening of a pore in the mitochondrial double membrane in order to mediate the trans

nniotic fluid.

their target-specific insertion into the recipient apoproteins. Cluster assembly on ISCU depends

gamma-RIIB receptor (FCGR2B), playing a central role in terminating signal transduction from a
ed cells under some circumstances. In the crosstalk between autophagy and apoptosis propose

nniotic fluid.

nce of localized and selective activation of TGF-beta-1 by LRRC33/NRROS (By similarity). Indi

as well as in response to viral agents (PubMed:28045099). Exhibits bactericidal activity (PubMe

important role in angiogenesis and blood vessel development. Involved in fibrotic processes, i

s are present in high endothelial cells (HEVs) and play a central role in lymphocyte homing at s

progression, apoptosis, or DNA damage repair.

l by altering the TGF-mediated chemotaxis of monocytes. This effect on wound healing appear
intestinal motility. Implicated in the regulation of endothelial as well as vascular permeability, p

neurotransmission (PubMed:11907044, PubMed:12888553, PubMed:12713657). Acts as an a

chains, which in turn are catalysts in the autophosphorylation of the MAP3K7/TAK1 complex (i

: CLOCK-ARNTL/BMAL1|ARNTL2/BMAL2 heterodimer by competing for the binding to E-box e

:11799477, PubMed:18551513). Required for osteoblastogenesis and bone formation (PubMe

nory T-cell and neutrophil transmigration (PubMed:11812992). Involved in platelet activation (Pu

phosphorylating SMAD3 in a cell-cycle-dependent manner and repressing its transcriptional ac

clusion of the non-muscle (NM) exon in actinin pre-mRNAs. Promotes inclusion of exonS 21 an

ıbMed:15901763). In vitro regulates CD44 alternative splicing by direct binding to purine-rich ex

oviral integration, together exert efficient antiretroviral effects in infected target cells. Selectively
Med:20197547).
P1 and prevents the conformational rearrangement of TAP induced by peptide binding. Inhibite

ysosomes mediated by the Ragulator complex and the Rag GTPases. Activated mTORC1 up-re

ade (PubMed:24912431). Plays a role in regulation of cell migration, e.g. during wound healing

nt fully functional isoforms have been found for this gene.[provided by RefSeq, Oct 2010]

, modulates cell morphology and integrin-dependent cell adhesion through regulation of the Ra

:ion domains oriented toward the cytosolic face of the membrane. In response to ER stress, tra

feration (PubMed:20812024).

biquitin-protein ligase complex.
CD3G, CD3D, CD3E and CD247 enabling the recruitment of ZAP70. In turn, ZAP70 phosphor

:ts also as a potent intracellular inhibitor of GZMH by directly blocking its proteolytic activity (Pu

Monoubiquitinates IKBKB that will negatively regulates Tax-induced NF-kappa-B signaling. Ne

:ect protein-protein interaction. Interacts with p53/TP53 and promotes cell-cycle arrest over apo

:h positively regulate transcription (PubMed:14966270). This complex may be required for the a
:ore indicative of a certain type of epithelial 'stem cells'. Acts as a promoter of epithelial prolifera

zation of alpha-4:beta-2-containing nAChRs and slowing recovery from desensitization. Promo

xyacyl-CoA, but with opposite chiral specificity (PubMed:15060085). Regulates the amount of [illegible]

moters of various acute-phase protein genes (PubMed:12359225). Activated by IL31 through I[illegible]

e endosome and mostly are delivered to lysosomes enabling degradation of membrane protein[illegible]

ion in renal tubular epithelial cells (RTECs). May be involved in dendritic cell (DC) maturation, t[illegible]

sural axons/growth cones in vitro (By similarity). May play a role in constraining the orientation [illegible]

growth (Probable).

Part of the complex F(1) domain and of the central stalk which is part of the complex rotary ele[illegible]

RNA and antisense miRNA star (miRNA*) products. The mature miRNA is incorporated into a R

nesized proteins into the endoplasmic reticulum and reroutes them to the cytosol for proteasom

s its N-terminal domain, maybe through a neuronal-specific cofactor. Ativates BCL2 expression

ncing that preceeds resumption of meiosis, oocyte survival and normal zygotic genome activati

otein. [provided by RefSeq, Jul 2008]

RNA and antisense miRNA star (miRNA*) products. The mature miRNA is incorporated into a R

cytoplasmic DICER to generate mature miRNAs (PubMed:26027739, PubMed:26748718). The

air (NER) synthesis following UV irradiation (PubMed:20227374).

ription start site of genes and has been linked to activation of nuclear hormone receptors; SIRT

n a stem/progenitor to a postmitotic chromatin remodeling mechanism occurs as neurons exit th

ↄ300 to the sites of RNA polymerase II-blocking lesions (PubMed:16916636). Plays an importa

tor receptor (EGFR) on dual threonine residues, which leads to the suppression of epidermal g

aps in duplex DNA and specifically to nicks in duplex DNA.
d to the CX3 complex and seems to involve GEN1 during mitotic cell cycle progression. Part of
259151). Also acts by elongating ubiquitin chains initiated by the E2 enzyme UBE2D1/UBCH5

obin mRNA and commit it to the splicing pathway. The phosphorylated form (by SRPK2) is requ

lated. A correlated decrease in protein level occurs at M/G1 phase, probably due to its degrada

or components: the central clock, residing in the suprachiasmatic nucleus (SCN) of the brain, a

to cell adhesion in brain. Ensures the proper localization of GRIN2B (subunit 2B of the NMDA r

in embryonic development (By similarity). Involved in sexual development, acting as activator [illegible]

[illegible]dation of a set of translationally active cytokine-induced inflammation-related mRNAs, such as

[illegible] supercoils DNA, and plays an important role in kinetochore assembly. CENPT has a fundame[illegible]

[illegible]RNA and antisense miRNA star (miRNA*) products. The mature miRNA is incorporated into a [illegible]

[illegible]nbly of PP2A phosphatase with the PPP2R5B-AKT1 complex leading to dephosphorylation of [illegible]

[illegible]RNA and antisense miRNA star (miRNA*) products. The mature miRNA is incorporated into a [illegible]

[illegible]exes, it is more abundant in embryonic stem cells and plays a major role in forming H3K27me3[illegible]

[illegible]Also plays a key role in deuterosome-mediated centriole amplification in multiciliated that can g[illegible]

[illegible]RNA and antisense miRNA star (miRNA*) products. The mature miRNA is incorporated into a [illegible]

[illegible]creases TNF-alpha production in response to LPS. Seems to be involved in the induction of infl[illegible]

[illegible]atin regions, thereby playing a central role in the establishment of constitutive heterochromatin [illegible]

and in regulating mitochondrial fatty acid oxidation (By similarity). Plays an important role in bo[illegible]

› remodeling. Acts as a docking site for several chromatin remodeling enzymes (e.g. PML at th‹

xidase is the component of the respiratory chain that catalyzes the reduction of oxygen to wate

›r components: the central clock, residing in the suprachiasmatic nucleus (SCN) of the brain, aı

ı are activated through phosphorylation and further phosphorylate additional targets. Plays a r‹

›r components: the central clock, residing in the suprachiasmatic nucleus (SCN) of the brain, aı

ear and could be very weak (PubMed:28516954). May play a role in the promotion of late stage

C-3' RNA consensus sequence.

ved in transcriptional regulation by KLF4. Isoform 3 and isoform 4 can bind to a EPHA8 promot

PubMed:29917077). ATP5MD is a minor subunit of the mitochondrial membrane ATP synthase

PDPN (PubMed:18215137). After ligand activation, signals via sequential activation of SRC and

RNA and antisense miRNA star (miRNA*) products. The mature miRNA is incorporated into a R

ed in T-cell proliferation and effector function. In dendritic cells involved in endosome-to-membr

ed:10773016, PubMed:10075644, PubMed:9872744, PubMed:24305054). Signaling inhibits ac

RNA and antisense miRNA star (miRNA*) products. The mature miRNA is incorporated into a R

ition of phospholipids (phospholipase A2 activity) (PubMed:7794891, PubMed:8619991, PubM

th a minimum acyl chain length of 14 carbons (By similarity). Does not transport docosahexaen

ɾectly involved in protein transport from the Golgi apparatus to the plasma membrane (By simila

ɔr components: the central clock, residing in the suprachiasmatic nucleus (SCN) of the brain, aı

ıaling (By similarity). The cilium length response creates a negative feedback loop whereby flui

trusion in mature osteoclasts (By similarity). Regulates insulin sensitivity through calcium/calmo
cells. Plays important roles in mediating Src family kinase signals for the completion of cytokine

ɔ thought to regulate the transcriptional activity of other receptors, such as androgen, progester

e-stranded tail length of approximately 15 nt (flap substrates) or 10 nt length single-stranded ga

g with the interaction of STIL with CENPJ (PubMed:25385835). Plays a role in the regulation o

Part of the complex F(1) domain and of the central stalk which is part of the complex rotary eler

matin DNA via its DACHbox-N domain (By similarity).

RNA and antisense miRNA star (miRNA*) products. The mature miRNA is incorporated into a F

bound form, thereby promoting autophagosome maturation (PubMed:27103069). The C9orf72-
sis and differentiate into neurons, npBAF complexes which contain ACTL6A/BAF53A and PHF

ppase-mediated translocation of phosphatidylethanolamine (PE) at the plasma membrane. Act

RNA and antisense miRNA star (miRNA*) products. The mature miRNA is incorporated into a F

RNA and antisense miRNA star (miRNA*) products. The mature miRNA is incorporated into a F

RNA and antisense miRNA star (miRNA*) products. The mature miRNA is incorporated into a F

7, PubMed:19111657, PubMed:19589784). May contribute to ubiquitination and degradation of

ıg to its transcription start site. May play a role in ocular development and astrocyte function (B

tal and neurogenetic diseases such as microcephaly, macrocephaly, autism, schizophrenia, cc

ɟ:14559847, PubMed:12865317, PubMed:15805301, PubMed:15041462, PubMed:18577768,

ŘNA and antisense miRNA star (miRNA*) products. The mature miRNA is incorporated into a F

c trauma.
of calcium pumps and may regulate sarcoplasmic reticulum calcium uptake in myocytes. May a
ınt role in the regulation of DNA replication by stabilizing the licensing factor CDT1 (PubMed:27

ication licensing (PubMed:20129055). Inhibits the transcriptional activity of a subset of Hox pro

ŘNA and antisense miRNA star (miRNA*) products. The mature miRNA is incorporated into a F
rence PLP > PNP > PMP (PubMed:14522954).

of ADR1B. Also regulates AMPA-type glutamate receptor (AMPAR) immobilization at postsyna

ning and morphogenesis and may be involved in the early development of diencephalic subdiv

RNA and antisense miRNA star (miRNA*) products. The mature miRNA is incorporated into a R

as the sulfane donor (PubMed:24981631).

shelterin complex (telosome) components.

e involved in N-glycosylation of APP (amyloid-beta precursor protein). Can modulate gamma-s
RNA and antisense miRNA star (miRNA*) products. The mature miRNA is incorporated into a R
RNA and antisense miRNA star (miRNA*) products. The mature miRNA is incorporated into a R

so acts as a regulator of autophagy: following cellular senescence, able to induce autophagy.

h leads to release of the repressor complex. At the same time, there is increased recruitment o

ct (PubMed:29856954). Enhances Notch signaling pathway in a non-cell-autonomous manner v
tochondrial membrane potential and mitochondrial respiration (PubMed:20453889). Upon INS

-TRPV1 complex.
onia to efficiently accomplish the meiotic prophase.
bMed:20176812). Positively regulates transcription of RRP1B (PubMed:20040599).

ACNA1B as pore-forming subunit (PubMed:8107964).

ws them to respond to BMP4 in a paracrine and/or autocrine fashion. BMP4 signaling in the m[illegible]

xidase is the component of the respiratory chain that catalyzes the reduction of oxygen to wate

ous tissues has been associated with the proliferation and metastasis of tumor cells. [provided

imination of bound antigens (PubMed:20176268, PubMed:22158414). The antigen binding site

ectal, entorhino-hippocampal and hippocamposeptal pathways. Together with EFNA5 plays als
ces. May play a role in regulation of protein translation by phosphorylating EEF1A1, leading to
poptotic activities of BCL2L11 by sequestering it to microtubules. Upon apoptotic stimuli the B[illegible]

ɘrol on nascent APOE-containing lipoproteins secreted from glia and influences cerebral spinal

ubMed:14593112). May be involved in apoptosis (PubMed:14593112).

e differentiation; P-TEFB complex interacts with MYOD1; this tripartite complex promotes the t
ɛ, nick, or gap flaps of distinct sequences and lengths; and DNA ligase I (LIG1) on long-patch b

ɘvelopment and regulates, for instance, lymphoblast proliferation (PubMed:23178126).
in the immunological synapse and directly inhibits T-cell activation (By similarity). May inhibit a

d oxalate. Functions in multiple exchange modes involving pairs of these anions, which include

ptor tyrosine kinase including EPHA4, EPHA3 and EPHB4. Together with EPHB4 plays a centı

ALS3 by inhibiting NFKB1 (By similarity).

RNA and antisense miRNA star (miRNA*) products. The mature miRNA is incorporated into a F

the GDP-bound to the GTP-bound form (PubMed:7479768). Functions as an important negati

kines secretion (By similarity). Enhances CD4+ T-cell differentiation and proliferation (PubMed
rane components. Clathrin-associated adaptor protein (AP) complexes which can bind directly
ie cleavage of peptides bound to major histocompatibility complex class II molecules of antiger

acrophage activation and polyamine metabolism (By similarity). In fetal dendritic cells may play

kidney, and therefore may provide immediate immunological protection against reactivating infe
eton and cell motility. Plays a critical role in TNF-induced angiogenesis, and implicated in the s
endently of heterodimerization with survival-promoting BCL2 and BCL2L1 through induction of
containing mRNAs, and hence promotes ARE-mediated mRNA deadenylation and decay proce

ed in Schwann cell migration during peripheral nerve regeneration (By similarity).

ner (PubMed:17287217). Recruits CTNNB1 to caveolar membranes and may regulate CTNNB

m for signal transduction by assembling, via its cytoplasmic domain, protein complexes contair
les to MHC class II takes place. Serves as cell surface receptor for the cytokine MIF.

sity.
lar lumen (By similarity). These effects are mediated by recruitment and activation of the Par p

ton, regulates formation of membrane ruffles, cell adhesion and cell migration. Plays a role in l

homeostasis and repair. Upon epithelial CXADR-binding, JAML induces downstream cell sigr
nt signaling (PubMed:20227366). Inhibits HDAC6 and thereby promotes acetylation of alpha-t
f smooth muscle myosin phosphatase (SMPP1M) via phosphorylation of PPP1R12A; the inhib
f the genome. Together with RELA, acts as a coactivator to enhance NF-kappa-B-mediated tra

s in fibroblasts; acts as a negative regulator of the RhoA activation pathway. In platelets, functi
alateral inner ear efferent growth cones at the midline and of retinal ganglion cell axons to the

ally with TLR4 and probably TLR2 in inflammatory responses and modulates TLR3 signaling. H
PubMed:24371721). Degrade also gelatin, heat-denatured type I collagen, but not native colla

nay function as a decoy receptor for VEGFC and/or VEGFD and play an important role as a ne
CTNND1 (delta-catenin). Regulates cytoskeletal remodeling by phosphorylating several protei

plays a role in various processes such as endothelial cell survival during acidification by preve

a local cysteine supply and contributes to maintain intracellular GSH level. It is part of the cell
'ubMed:23238346). Contributes to the generation of inhibitory postsynaptic currents (PubMed:2

pendent Rho signaling also regulates protein phosphatese 2A activation causing dephosphory

estin-1/ARRB1. Phosphorylation of ARRB1 by GRK5 inhibits G-protein independent MAPK1/M
ters: acts by inhibiting the activity of the ubiquitin-conjugating enzyme UBE2N/Ubc13, leading t

nal programs. Its differential recruitment to chromatin is dependent on distribution of histone H

2 and this binding is essential for IGF2 signaling (PubMed:28873464).

shifts to more positive voltages. The inward rectification is mainly due to the blockage of outwa

similarity). Ligand for P4HB; the interaction retains P4HB at the cell surface of Th2 T-helper cel

thylated TAF10, a member of the transcription factor IID (TFIID) complex, which induces releas

rm the predominate functional GAG-binding site.||Lactoferroxins A, B and C have opioid antag

it PIK3R1, PLCG1, SRC, GRB2, STAT3 or the adapter GAB1. Recruitment of these downstrea

s and confers resistance to transcriptional repressors (PubMed:25417107, PubMed:27105114,

components: the central clock, residing in the suprachiasmatic nucleus (SCN) of the brain, an

ediate the antiviral effect via the classical RNase L-dependent pathway or an alternative antivi

ı polymerization and cell migration. Additionally, phosphorylates TNNI3/troponin I to modulate c
a) of the C-terminal glycine of the peptidylglycine substrate (PubMed:12699694). The second s
th and invasion of malignant melanoma cells (e.g. pseudomyxoma peritonei (PMP) cell line) (P

ıilic interaction with CD177 plays a role in transendothelial migration of neutrophils (PubMed:17
or both. In cooperation with ABCB4 may be involved in establishing integrity of the canalicular

632, PubMed:18162525). Inhibits the cleavage of MEPE by CTSB/cathepsin B thus preventing

erebroside and phosphatidyl ethanolamine (PubMed:9132017). Plays an important role in HDL

products that are not contiguous in the parental protein (PubMed:27049119). Acts as a major c

ylation of insulin receptor (IR) and insulin receptor substrate 1 (IRS-1), phosphorylation of prote

t of the pituitary gland and the olfactory bulb. Functions as tyrosine phosphatase (PubMed:852

equired for anchorage-independent proliferation of transformed cells (PubMed:19306925). Dur

scavenging extracellular LPL. Probably required in adipocytes for the formation of specialized

chemotactic factor that attracts lymphocytes and, slightly, neutrophils, but not monocytes (Pub

ıotactic for T-cells and monocytes and not for neutrophils. The membrane-bound form promote

ent transcription.

vth cone collapse in response to NGF (PubMed:22155786).

s during inflammation (By similarity). Plays a redundant role with SOX4 and SOX11 in cell surv

specific role of each SRC kinase is very difficult. SRC appears to be one of the primary kinase

ːIP6. Plays a role in cytokinesis through KIF14 interaction (By similarity).

logy and autophagy (PubMed:31036939).

ɔonse to LTBR stimulation. Inhibits TRAF2-mediated activation of NF-kappa-B. Down-regulates

ane microdomains that mediate membrane trafficking and signal transduction. They are implica

tiation.

May play a role in TFRC-mediated iron uptake (PubMed:20188707). In concert with FAM89B/L

complexes and subsequently prevents premature joining of the 40S and 60S ribosomal subun

PML bodies in the nucleus (PubMed:24128730, PubMed:20168092). May regulate the activatio

CNS) (By similarity). May play a role in the formation of typhoid toxin transport intermediates du

upling of PTPN12 to CD2. Also has a role in innate immunity and the inflammatory response. F

conversion probably implicating MON1A/B, and via binding SNAREs and SNARE complexes to

serving the epigenetic landscape (PubMed:28402439). Required for chromatin targeting and m
ubstrate specificity may be influenced by its subcellular location. Sulfates GlcNAc residues at te

activation by PPP3CA (PubMed:18218901).
nerve terminals. May function as a regulator of vesicle transport, through interactions with the

ation (By similarity).
IKBKG or TRAF6 in response to interleukin-1-beta (IL1B) stimulation or upon DNA damage (Pu

nediates cell signaling pathways involved in cellular functions such as cell adhesion and migrat

MALP-2), soluble tuberculosis factor (STF), phenol-soluble modulin (PSM) and B.burgdorferi ou

of endomembrane damage.
ggering RHOA activation leading to cell migration increase and invasiveness (PubMed:1704699
iclear export. Positively regulates the association of constitutive transport element (CTE)-conta

imer-mediated transcriptional activation of the core clock component PER2 (By similarity).

:ylglycerol (By similarity).

elomeric DNA, but not to a telomerase RNA template component (TER) (PubMed:12676087, P

developing brain (By similarity). Plays a role in RHOA activation and subsequent changes of tl
ement membrane during embryogenesis, and for normal embryonic epicardium and heart morp

nscription to occur, blocking early-stage virus replication in dendritic and other myeloid cells (P
unction does not rely on its G-protein coupled receptor (GPCR) structure but instead on its com

on (PubMed:18270267). Binds to phosphoinositides with preference for PI(3,4)P2 and PI(3,4,5
to autophagosomes and delivered to lysosomes for degradation (PubMed:20505359, PubMed:

caspase activation and degeneration of both neuronal cell bodies (via caspase-3) and axons (v

najor constituent of the ASC pyroptosome which forms upon potassium depletion and rapidly re

he nucleoplasm where it can inhibit MDM2 and allow p53/TP53 activation (PubMed:24120868,

HIF1A activity. May regulate insulin signaling cascade. May play a role in apoptosis, bone resor

osis factor TNF. When bound to PKB, it inhibits it probably by decreasing PKB level of phospho

plex by WASL, and thereby plays a role in the reorganization of the F-actin cytoskeleton. Binds

y sensitize epithelial cells to apoptosis specifically triggered by the death ligand TRAIL. May ha

uces the epsilon-cleavage of APP CTF-alpha, leading to a low production of AICD.

oblasts and thymocytes, but not in hepatocytes (By similarity). Required for mTORC1-mediate
s to escape degradation resulting in their translocation to the nucleus, heterodimerization with H

the stability of CDKN1A transcripts induced by p53/TP53. Also acts as a mRNA splicing factor

ɔreading, migration and sprouting of primary brain microvascular endothelial cells (MVECs). Als

recruited to the endoplasmic reticulum-plasma membrane contact sites (EPCS) which is media

ation by increasing the catalytic turnover without increasing the affinity of deubiquitinating enzy

phosphorylate psicosamines and ribulosamines (PubMed:14633848).

n. Enhances the inhibitory activity of SMAD7 and reduces the transcriptional activity of SMAD2

ɔmpromised BTB dimers is required for the differentiation and survival of neural crest and neur

ıosphorylation of MFN2 (PubMed:23620051). Activates PRKN in 2 steps: (1) by mediating pho

clearance of the protein from the cell surface by clathrin-dependent endocytosis. WNK4 appe

ßy similarity).
ear import (PubMed:19015240, PubMed:26609676).||[Isoform 2]: Functions as transcription re

ly (PB) in response to stress (PubMed:21964062).

shown to enhance the induction of cytotoxic T-cells and selectively stimulates interferon gamm
transcription factor that specifically activates the expression of the CD40 receptor and its ligan
ı79411).

exclusion of PIAS3 from the nucleus (PubMed:20516148).

e reticulophagy receptor TEX264, participates in the remodeling of subdomains of the endopla
possibly regulating CCDC88B-mediated lytic granule transport to MTOC during cell killing (Pub

724).

AURKA removal from centrosomes in prophase (PubMed:23649807). Also, regulates spindle

cell activation. CD47 binding prevents maturation of immature dendritic cells and inhibits cytoki

itol 3,4,5-trisphosphate (PubMed:30420721, PubMed:28882892). Plays an important role in MI

tion of ERK, JNK and NF-kappa-B pathways, inhibiting MAP3K3-mediated interleukin-8 produc

:16940153, PubMed:18945876, PubMed:20207939, PubMed:20089856). Saturated long-chain

it plays a central role in the formation and the maintenance of the neuromuscular junction (NM

n of WASF3 is critical for the stimulation of lamellipodia formation and cell migration. Involved in
n mediated by the nuclear hormone receptors PPARG and RARA (PubMed:22351778).

tion and activation of the energy sensor protein kinase AMPK (PubMed:24767988, PubMed:26
be related to the absence of a catalytically active form of the mitochondrial isozyme. The incre

milarity). May be involved in the regulation of MYC activity and the control cell proliferation (Pul

SBs). Its ability to specifically bind modified histones and chromatin modifying enzymes such as

ed phosphorylation. Interaction with overexpressed HADH2 leads to oxidative stress and neuro

ymes, and leading to HIF1A hydroxylation and subsequent proteasomal degradation (PubMed:2

on-independent deposition of histone H3.3 in pericentric DNA repeats outside S-phase and telo

the survival and differentiation of selected neuronal populations of the peripheral and central r

n important role in targeting the monocarboxylate transporters SLC16A1, SLC16A3, SLC16A8,

deficiency virus type 2 (HIV-2), simian immunodeficiency viruses (SIVs), equine infectious anem

nd GM-CSF promoters (Probable). Essential for the development of normal hematopoiesis (Pu

on. May prevent premature elimination of red blood cells. May be involved in membrane perme

d the subventricular zone of the lateral ventricles. Required during thymocyte development. Pr

nner by preventing the activation of cyclin B1/CDC2 complexes. Binds to BCL6 and down-regu

y other chaperones, such as HSPA8/HSC70 (PubMed:11123922). Does not refold proteins by

ot to bind to F-actin when assembled in the complex suggesting a different linkage between ac

IA replication checkpoint independently of Okazaki fragments processing. Possesses different

be involved in the promotion of lymphatic endothelial cells adhesion, migration and tube forma

in a cell type-specific manner. The corepressing function towards thyroid hormone receptor be

ts flaps from equilibrating into structurs that lead to duplications and deletions. Also possesses
ing molecules diacylglycerol and inositol 1,4,5-trisphosphate. Phosphorylation of FRS2 triggers

tion of endothelial cells, proliferation of some types of cancer cells, but does not promote prolife

:L7, CCL11, CCL13, CCL14, CCL17, CXCL5, CXCL6, IL8/CXCL8, CXCL11, GRO, RANTES, M

unctions as a recycling molecular chaperone which facilitates the secretions of tropoelastin and

ysosomal enzymes (PubMed:28453791, PubMed:28541286). Facilitates also the acidification (

iate the development of insulin resistance by regulating activation of transcription factors. Regu

NA-binding complexes and for activation of immunoglobulin heavy-chain transcription upon B-ly
complex composed of two proteins, the trifunctional enzyme subunit alpha/HADHA described h
l of two proteins, the trifunctional enzyme subunit alpha/HADHA carries the 2,3-enoyl-CoA hydr
ed from the complex and CREBBP is recruited, which facilitates transcriptional activation. Deac

e element (HRE) of target gene promoters (By similarity). Activation requires recruitment of tran
Med:30530481, PubMed:19177349, PubMed:24395804, PubMed:26758806). A number of HLA
743181).||Allele B*08:01: Presents to CD8-positive T cells viral epitopes derived from EBV/HH

As and long non-coding RNAs (lncRNAs) via a mechanism named 'm(6)A-switch', facilitating b

egulates the release of 5-hydroxytryptamine, dopamine and acetylcholine in the brain, and ther
tine kinase E-box enhancer. Regulates the circadian clock by repressing the transcriptional acti

ctive IFNB1 receptor by itself and activate a signaling cascade that does not involve activation c

RC2 complex and inhibits trimethylation of 'Lys-27' of histone H3 (H3K27me3) by the PRC2 co
omodimer has very low potassium channel activity, when expressed in heterologous systems, a

MK1 on a threonine residue located in its activation loop (PubMed:10436159). LIMK1 subsequ

PubMed:10395745, PubMed:22614033, PubMed:30140003). Via its role in the biosynthesis of

repair, tumor invasion, inflammation, and atherosclerotic plaque rupture. As well as degrading

pears to be involved in a very early step of clathrin-mediated endocytosis in polarized epithelial

kappa-B sites that they can bind with distinguishable affinity and specificity. Different dimer cor
regulates RANKL-induced osteoclast differentiation (PubMed:29149593). Positively regulates

l migration and tumor cell invasion. Promotes fibroblast migration and proliferation, and thereby

erase but not acetylcholinesterase. Seems to be mediated by a specific receptor (By similarity)

irus-1 (HHV-1) replication by sequestering the viral E3 ubiquitin-protein ligase ICP0 in the cytop

f clock information to metabolic pathways regulated by PER2.
owing mitochondrial depolarization, dephosphorylates DNM1L inducing DNM1L translocation to
DHD1 (By similarity). The primase subunit of the polymerase alpha complex initiates DNA synth
probably by indirectly activating myosin. Beta non-catalytic subunit acts as a scaffold on which t

orylating and activating RAF1, which mediates the activation of the MAPK/ERK signaling casca

e side chains (By similarity).

Seq, Mar 2010]
y members. This activity requires the integrity of the catalytic site, although it is unclear whether
s of galacto-cerebroside sulfate by arylsulfatase A (EC 3.1.6.8), GM1 gangliosides by beta-gala
eticulum to cytosol and is involved in calcium homeostasis (PubMed:16959576). Is a regulator

ylinositol 3-phosphate and inositol 1,3,4,5-tetrakisphosphate with order of substrate preference

and placenta development. Required for embryonic angiogenesis, normal cardiomyocyte migra

d (PubMed:19615464). Shows N-acyltransferase activity, catalyzing the calcium-independent tr
generation of ssDNA. Component of the BRCA1-RBBP8 complex that regulates CHEK1 activa

entiation of 4 of the 5 islet cell types during endocrine pancreas development, with the exceptio

histone H3 at 'Ser-10', which results in the subsequent transcriptional activation of several imm

ne and tryptophan (PubMed:9751058, PubMed:11557028, PubMed:11311135, PubMed:11564
ısmic Ca(2+) levels rapidly return to baseline. Required for normal embryonic heart developme

nplex (nBAF complex). During neural development a switch from a stem/progenitor to a postmit

(ISRE) to activate the transcription of IFN-stimulated genes (ISG), which drive the cell in an ar
pamine (PubMed:7779757, PubMed:11006110).

ted intermediate fragment called Notch extracellular truncation (NEXT) (PubMed:24226769). P

receptor complex composed of 2 TGFBR1 and 2 TGFBR2 molecules symmetrically bound to t
ulation of cell migration by targeting CDC42BPB to the leading edge of migrating cells (PubMe

PubMed:12524540). However, this activity is inhibited when the interaction with PPP1R13B/AS

factor recruitment, or activation or repression by disruption of binding sites or conformational D

mplicated in the regulation of a large spectrum signaling pathway (PubMed:16352599, PubMec

ptor-associated protein (RAP). Binds dimers of beta 2-glycoprotein I and may be involved in the

ubMed:11825900). Modulates the functional properties of KCNA5 (By similarity). Enhances KC
l viral entry. Also implicated in cell adhesion and control of cell growth and migration. Plays a ke

igh dephosphorylation of these bioactive lipid mediators produces new bioactive compounds ar
hondrial RNA and processing of non-coding RNA imported from the cytosol into mitochondria (

ı (PubMed:19759537, PubMed:21478859). Also mediates PARsylation of BLZF1 and CASC3,

ı. May play a role in cartilage remodeling. May be proteolytically processed, during sperm epidi

.

›its neuronal migration and activates the RhoA pathway by coupling to GNA13 and possibly GN

ases to APP cleavage sites.

n conjunction with the ESCRT machinery also appears to function in topologically equivalent m
ion (By similarity). The NCOR1-HDAC3 complex regulates the circadian expression of the core
RTF-A-dependent gene transcription. Does not activate SRF:MKL1/MRTF-A through RhoA (Pu

487, PubMed:11359771). Its binding to ligand-activated beta-1 adrenergic receptor ADRB1 lea

press critical genes for neural stem cell fate such as SOX2, EOMES and ROBO2 (By similarity

ir downstream signaling pathways (PubMed:10769033, PubMed:12221124). Plays a role as a

regulation of ESR1 expression in breast cancer. Serves as a corepressor of RARA and causes

filaments. Phosphorylates LIMK1, a kinase that also inhibits the activity of cofilin. Phosphorylat

similarity).

iated viral entry and VSV G protein-mediated viral entry. Induces cell cycle arrest and mediates

al activation of CREB1 (By similarity). May play a role in neurite growth. Isoform 3 may promote
s in the relocalization of mTORC1 to the lysosomes and its subsequent activation by the GTPa
heart rate (By similarity).

tivation stimulated by APP C-terminal intracellular fragment (AICD), most probably by competin

ivity is regulated by RAC1. Regulator of epithelial morphogenesis. As component of the WAVE
rmatoproteasome, a form of the proteasome specifically found in testis: binds to acetylated hist

he GAIT complex interacts with m7G cap-bound eIF4G at or near the eIF3-binding site and blo
of TMCC1, leading to recruitment of the endoplasmic reticulum to endosome tubules for fission
ed for cytokinesis (PubMed:15800069). Plays a role in epithelial cell junctions formation (PubM

cle arrest in G(0) (PubMed:22020328, PubMed:22323446). SCF(FBXL2) also mediates PIK3R2

so binds dimethylated at 'Lys-36' (H3K36me2). Isoform 1 and isoform 2 inhibit transcription from

se (RPS6KB1 or RPS6KB2) (PubMed:18498745). The Cul7-RING(FBXW8) complex also medi

ycle commitment (PubMed:29875408). As APC inhibitor, prevents the degradation of APC sub

CTPBP1 is not essential (By similarity). Plays an important role in the specification and differe

plex (PubMed:27602518). Involved in random X inactivation mediated by Xist RNA: acts by bin

ment to TNFR1 (PubMed:25681446). Prevents also the activation of NF-kappa-B by associati

suppress loss of contact inhibition elicited by activated oncogenes such as MYC (PubMed:15[illegible]

o the 43S PIC and scanning of the mRNA for AUG recognition. The eIF-3 complex is also requ
ton; probably by indirectly activating myosin. Gamma non-catalytic subunit mediates binding to

MYO5B to NPC1L1, and thus facilitates cholesterol uptake (By similarity).
Med:23303788). Binds preferentially to DNA containing cytidine-phosphate-guanosine (CpG) di

eoblastogenesis in mesenchymal stem cells (MSCs). Negative regulator of the canonical Wnt/b

vhich together ensure the dynamics and stability of the telomere.
involved in the regulation of blood pressure through the inactivation of angiotensin II and/or the

ate target genes involved in formation of various organs, including muscle, kidney, gonad, gang
clear export is thought to be conferred by an asymmetric distribution of the GTP- and GDP-bou

of mammalian cell membranes (Probable). Globotriaosylceramide/globoside Gb3Cer in blood a
ortant for regulation of endometrium cell proliferation. Important for normal prenatal and perina

e CLNS1A that controls the assembly of the core snRNP. Dissociation by the SMN complex of

255466). Acts as 3'-5' exodeoxyribonuclease for double-strand breaks resection and efficient h

viral RNA. Can also limit the replication of hepatitis C virus (HCV), West Nile virus (WNV), Chil

ytoplasmic domain of cargo proteins predominantly via VPS35; however, these interactions see
l in regulation of neurite outgrowth, and, reconstituted to liposomes, predomiminantly transport

GlcNAc but not alpha- or beta-benzyl GalNAc.
sponses to DNA damage, cellular stress and hypoxia by protecting CDKN1A against degradati

ity). Has also a modest lysophosphatidylinositol acyltransferase (LPIAT) activity, converts lysop

components: the central clock, residing in the suprachiasmatic nucleus (SCN) of the brain, an
, drives the retrieval and recycling of NxxY-motif-containing cargo proteins by coupling to SNX
nd -III complexes. ESCRT-III proteins mostly dissociate from the invaginating membrane before

ly regulates necroptosis by reducing RIPK3 expression (PubMed:29883609). Mediates 'Lys-63

egatively regulating the secretion of type I and type III collagens (PubMed:28939891). This calc

cells self-renewal (By similarity).

l phosphorylates ODF2 in vitro (PubMed:23400999). Plays a role in cell cycle progression, spe

ı of SEC23A, which promotes the transport and secretion of cartilage matrix proteins, and poss

des with an acetylated N-terminal Pro residue (PubMed:29632410).

resses the activity of the circadian transcriptional activator: CLOCK-ARNTL/BMAL1 heterodime

ably by repressing genes promoting cytokinesis and antagonizing action of classical E2F protei

led:22745828).

retion: acts by mediating efflux of calcium from mitochondrion, thereby affecting cytoplasmic ca

ent proteasomal degradation of the transcription factor HBP1 (PubMed:29911972). Acts as a n

EGFR2. Modulates endothelial cell migration in an integrin-dependent manner implicating integ

ion, without promoting KLF7 ubiquitination (By similarity).

). The MCM8-MCM9 complex is dispensable for DNA replication and S phase progression (Pu

d for membrane tubulation.
duced acrosome reaction. May play a role in cell growth. Modulates the liver regeneration in co

zzled FZD8 and LRP6. May negatively regulate the TGF-beta pathway (PubMed:21727895, Pu

ıbule assembly in axons (Probable).
icrotubule (MT) anchoring to the centrosomes (PubMed:28659385).

ɔnse is triggered by ADP-D-glycero-beta-D-manno-heptose (ADP-Heptose), a potent PAMP pre

o functions in the AP-2-independent endocytosis of the LDL receptor.

mian foamy virus independent of prior ectodomain shedding by furin or furin-like proprotein cor

er for GALK1 (PubMed:12753898). Also active on D-glucose although shows a preference for

unbranched filaments. Contributes to cell motility by controlling actin dynamics. May promote tl

ppa-B and FADD respectively (PubMed:12471095, PubMed:12539043, PubMed:14739303). P

ı-independent mechanism that works prior to the proviral integration, together exert efficient an

ıclude GALNS, ARSA, STS and ARSE (PubMed:12757706, PubMed:15907468, PubMed:1565

ɔmes to the trans-Golgi network. Required for the structural integrity of the Golgi complex. Play

2S1/eIF-2-alpha phosphorylation in response to stress converts EIF2S1/eIF-2-alpha in a globa

a negative feedback loop to destabilize Notch 1 intracellular domain (NICD) and downregulate

). Additionally, may play a role in protein biosynthesis by modifying the translation machinery (
RNA and antisense miRNA star (miRNA*) products. The mature miRNA is incorporated into a R

ary. Within the receptor complex, type-2 receptors (ACVR2A and/or ACVR2B) act as a primary

06, PubMed:24945728). Contributes to the formation and maturation of the immunological syn

d for normal cell cycle progression from G2 to mitosis. Required for appropriate cell cycle arrest
o ssDNA in tailed duplexes and replication fork structures. May play a role in the extension step

ctivation. In activated T-cells, inhibits PLCG1 activation and calcium mobilization upon restimula

RALAR2, while the predominant TIMM9-TIMM10 70 kDa complex mediates the import of much

ctin capping activity when associated with ABI1, thereby regulating actin-based motility process

and for efficient repair of DNA double strand breaks (DSBs) specifically when modified by C-ter

close proximity (PubMed:7797075, PubMed:11909973, PubMed:19522541, PubMed:18413230

transcriptional coactivators, thereby controlling muscle terminal differentiation (By similarity). In
Interacts with tRNA-Tyr. Stimulates the transcription of the human neurotropic virus JCV.

lays a critical role in both the early and late phases of the IFNA/B gene induction (PubMed:168
MA1 channel opener dehydrosoyasaponin I (DHS-1) triterpene glycoside and for external bindi

phase and for cell division. Plays a role in terminally differentiated hair cells development of the

MCMBP.

pterin. Acts by mediating the incorporation of 2 sulfur atoms from thiocarboxylated MOCS2A int

nal clustering of sodium channels at heminodes; not required for the formation of mature node

nay be involved in Ig class switch recombination (CSR) and/or Ig variable region somatic hyperr
ative pre-mRNA splicing. Associates to spliced mRNA within 60 nt upstream of the 5'-splice site

the closed conformation thus preventing double-stranded DNA to enter the active site cleft. Th
red. Associated with the PA200 or PA28, the 20S proteasome mediates ubiquitin-independent
LP-BER proceeds; endonuclease FEN1 cleavage activity on substrates with double, nick, or g

ase a master regulator of the DNA damage response (PubMed:24332808). It is required for the
djacent normal tissues has been observed, although no correlation between the level of expres

p-regulates Na(+) channels: SCNN1A/ENAC, SCN5A and ASIC1/ACCN2, K(+) channels: KCN
correct loading of SASS6 and CENPJ to the base of the procentriole to initiate procentriole as
1. Recognizes CDKN1A in association with CCNE1 or CCNE2 and CDK2. Promotes ubiquitina
ation of negative charges due to aspartate and Na(+) symport (PubMed:8857541, PubMed:266
9872227). Through binding of the fusogenic protein syncytin-1/ERVW-1 may mediate trophobla
sion components called SNAREs (Soluble NSF Attachment Protein REceptors) at presynaptic p

781166, PubMed:28076346). Is also a component of the minor U12 spliceosome (PubMed:15
erm tail.

proximity and results in final fusion. Participates in the calcium-dependent regulation of acrosor
rgeted to histone H3 via its interaction with RB1 and is involved in many processes, such as re

by TCF7 and CTNNB1.May also act as feedback transcriptional repressor of CTNNB1 and TC

(groEL-2) act via this protein to stimulate NF-kappa-B expression (PubMed:15809303). In com
unomodulating pentapeptide.||TP and TP5 may play a role in T-cell development and function.
NA 3'-OH attacks the covalent intermediate to expel the active-site tyrosine and restore the DN

20221403, PubMed:21775632, PubMed:27193233, PubMed:23644599, PubMed:25815583). A

ed:30071723). May also recognize and bind histone H3 succinylated at 'Lys-122' (H3K122succ
t of L-DOPA across the blood-brain barrier (By similarity). May act as the major transporter of t

r phosphatase activity allows both the hydrolysis and the cellular uptake of these bioactive lipid

neckpoint in response to genotoxic stress (PubMed:17141802, PubMed:17296725). TIMELESS

ne stability via cooperation with CALM1 and CETN2 (PubMed:16760425).

eading to reduced dephosphorylation of myosin MLC2 by myosin PP1. May be involved in DNA
nutrient and hormonal signals. May be involved in telomere length regulation.

in tandem to mediate ubiquitination of target proteins, ARIH1 mediating addition of the first ubic
08065). Promotes deacetylation of CTTN, leading to actin polymerization, promotion of autoph

h MYLIP-dependent ubiquitination of LDLR, VLDLR and LRP8 (PubMed:19481530). Interplays

to be the major enzyme contributing to LPCAT activity in the liver. Lysophospholipid acyltransf
ıroid lineages (By similarity). Contributes to the maintenance of steady-state ERBB3 levels by r

ay contribute to cancer (PubMed:14767476).

ndle and centrosomes (PubMed:18361916, PubMed:21402792). Involved in centriole duplicati
e release (By similarity).

l:24035200). Pol-delta3 and Pol-delta4 are characterized by the absence or the presence of P
le in centriole replication suggests a possible role in tumorigenesis, centrosome aberrations be

lication initiation, regulating DNA replication initiation (PubMed:10438470, PubMed:19187766,

by mediating AMPylation of HSPA5/BiP at 'Thr-518', thereby inactivating it (By similarity). In res
s thought to be involved in mRNA degradation by activating the decapping step in the 5'-to-3' n

nay be involved in Ig class switch recombination (CSR) and/or Ig variable region somatic hyperr

endent manner, and has anti-apoptotic activity (By similarity).

ATM at 'Ser-25'. Together with TP53BP1 regulates ATM association. Proposed to recruit PAGR

ed nuclear (TAN) lines which are bound to F-actin cables and couple the nucleus to retrograde

nay be involved in Ig class switch recombination (CSR) and/or Ig variable region somatic hyperr
cancer cells, but not in other cancer cells, via a caspase-2 mediated pathway that involves mit

nes such as CXCL1, CXCL8/IL8 and IL6 (PubMed:16785495, PubMed:17911633, PubMed:18
nportant role in paracellular Na(+) transport in the intestine and in Na(+) homeostasis. Requirec

ytochrome P450 proteins (PubMed:22292588).

vents easy recognition by sequence homology. This gene encodes a 28S subunit protein that h

vents easy recognition by sequence homology. This gene encodes a 39S subunit protein. [prov

position of adenine (N(6)-methyladenosine) (PubMed:30017583). N(6)-methyladenosine (m6
may play an essential role to ensure accurate mitotic chromosome condensation in neuron st

dulate the activity of proteasomes through association with PSMC3. Acts as a tissue specific c

vents easy recognition by sequence homology. This gene encodes a 28S subunit protein that

merging the MT-CO1/COX1 and MT-CO2/COX2 assembly lines (PubMed:29381136).

arsenite resistance but rather modulates arsenic sensitivity. Independently of its activity on mi

he early development of the kidney. May be involved in the regulation of ureteric bud initiation (
n and NF- Kappa-B through the RIG-I pathway (By similarity).

the activation of caspase-9, which then accelerates apoptosis by activating other caspases.

naling. Together with NDFIP1, limits the cytokine signaling and expansion of effector Th2 T-ce

led:26297806).

)124). Required for the recruitment of CEP295 to the proximal end of new-born centrioles at the

3. May inhibit STIM1-mediated Ca(2+) influx.
sion repair machinery. Not involved in processing oxidative damage.

hagy and for the cytoplasm to vacuole transport (Cvt). Preferred substrate is MAP1LC3A. Also [illegible]

RefSeq, Jul 2008]

omes and spindle apparatus during the cell cycle (PubMed:20596670).

d VLCFAs of different chain lengths that are involved in multiple biological processes as precurs

aposi's sarcoma-associated herpesvirus (KSHV) intronless mRNAs and infectious virus produc

s is involved in transcription regulation of downstream switch regions at the immunoglobulin he

ion.

B1 and is involved in many processes, such as cell cycle regulation, transcriptional repression

e CLNS1A that controls the assembly of the core snRNP. Dissociation by the SMN complex of
e CLNS1A that controls the assembly of the core snRNP. Dissociation by the SMN complex of

d:16046545). Negatively regulates canonical Wnt signaling; at least in part, cooperates with NI

n the GalNAc-beta-3-GlcNAc-beta-terminus is linked to the 4-position of O-mannose, suggestin

ı preference for 5'-flap structures, and promotes symmetrical cleavage of static and migrating H

sion, but is dispensable for cartwheel removal or centriole disengagement (PubMed:25131205)

affinity interactor RBM39 (PubMed:31686031, PubMed:31819272). Aryl sulfonamide anticance

ynthesized proteins into the endoplasmic reticulum and reroutes them to the cytosol for proteas

)12]

s. Binds to the E-box consensus sequence 5'-CANNTG-3' (By similarity).

)Med:27541860).
ı regulating protein levels of ANK1 isoform Mu17 probably implicating CUL3-dependent protea

:essive movement of microspheres along a microtubule in a depolymerization-coupled manner
oligomerization (PubMed:19289083). Affinity for microtubules is synergistically enhanced in the

ıdrial fission factor MFF, thereby promoting mitochondrial fragmentation and contributing to the

ulating expression and subcellular location of disheveled proteins. Stabilizes protein levels of J

ocessing via its association with pre-mRNA 3'-end processing factors, establishing a link betwe

bly and, potentially, ciliar re-assembly in cells that have already disassembled their cilia ensurir

f the first strand is rate limiting, while second strand cleavage is rapid. Largely monomeric, dim

rs including T-cell receptor (TCR), CD28 and ITGAL; proposed to be implicated in T cell prolifer

RNA and antisense miRNA star (miRNA*) products. The mature miRNA is incorporated into a R

RNA and antisense miRNA star (miRNA*) products. The mature miRNA is incorporated into a R
us to the cytoplasm where GTP hydrolysis releases Ran from importin. The directionality of nuc

uired for paracellular chloride transport in the kidney (By similarity).
nal proliferation. Shows strong GAP (GTPase activation) activity towards CDC42 and RAC1 and

E3 ligases TRAF6, TRAF2 and BIRC2, and E2 ubiquitin-conjugating enzymes UBE2N and UB
s proteins. Subsequently, phosphorylates the STATs proteins once they are recruited to the rec

NDC80 complex to track depolymerizing microtubules (PubMed:23085020). Plays a role in chro

lpha, liberating TPX2 from importin-alpha, allowing TPX2 to activates AURKA kinase and stimu

state of macrophages (PubMed:31978345). Enables a purine nucleotide cycle between adenos
3 genes and are involved in transcription regulation of these genes. Isoform 2 does not activate

iquitin-dependent protein catabolic process which controls CDKN1B degradation, resulting in p
ed:24939902). Acts as a loading platform to recruit DDR proteins that allow completion of DNA

RNA and antisense miRNA star (miRNA*) products. The mature miRNA is incorporated into a R

e center spindle by associating with polymerized microtubules (PubMed:20826784). Involved in

ptosis but not CASP9 itself (PubMed:12754298). May be involved in protein transport, membra

ion intermembrane space (in vivo) (PubMed:26071602). Mediates cell survival by inhibiting acti

asomes can also induce pyroptosis, an inflammatory form of programmed cell death (PubMed:

d localization at the cell periphery. Associated with the cytosolic face of lysosomes, the BORC

ably independent of BIRC4 inactivation which seems to occur in the cytoplasm. The BIRC4-XA

e interactions with G-proteins. May be involved in transduction and intercellular transmission o

RNA and antisense miRNA star (miRNA*) products. The mature miRNA is incorporated into a R

duced centrosome polarization and directional migration in aortic endothelial cells.
450 reductase (CPR; NADPH-ferrihemoprotein reductase) (PubMed:20149798, PubMed:8619

ptosis, and is important for immune self-tolerance. Acts downstream of several immune recepto
035, PubMed:19733258, PubMed:26091040). Activates RALA; this leads to the activation of ph

nd the radial migration of cortical neurons from the ventricular zone toward the superficial layer

d:23274085). May have an alkylhydroperoxide reductase activity born by the N-terminal domai
it does not affect the migration of smooth muscle cells or fibroblasts (PubMed:15467828, PubM
KL, DOK1 or ARHGAP35. Adhesion-dependent phosphorylation of ARHGAP35 promotes its as
(PubMed:24766809, PubMed:24766810). Its role in tRNA splicing and maturation is required

h.

RIF and TLR4- TICAM1/TRIF signaling pathways (PubMed:27881733).

stress, via TORC1 (PubMed:24947615). May positively regulate the transcription by NFE2L2

RNA-induced silencing complex (RISC), which recognizes target mRNAs through imperfect bas

iated by co-chaperones. The co-chaperones have been shown to not only regulate different ste

RNA-induced silencing complex (RISC), which recognizes target mRNAs through imperfect bas

roper cell cycle regulation of DNA replication (PubMed:16861906, PubMed:16949367, PubMed

iated by co-chaperones. The co-chaperones have been shown to not only regulate different ste

le as an oncogene promoting tumor growth and progression by positively regulating translation

he cellular signaling molecules diacylglycerol and inositol 1,4,5-trisphosphate. Phosphorylation
teracts with PARP1 and mediates MRE11-dependent DNA end resection during replication fork
:9106657).

RNA-induced silencing complex (RISC), which recognizes target mRNAs through imperfect bas

ness by inducing cell migration and invasion through its interaction with the actin-binding protei

Med:14506133, PubMed:19793056, PubMed:27804176). Upon DNA-binding, promotes DNA b

led:27499292, PubMed:26878239). May serve as an epigenetic mark that propagates centrom

icrotubule tracks that are detyrosinated (PubMed:25908662). Acts as a processive bi-directiona
cts embryonic stem cells toward a cardiac lineage. Involved in the regulation of DNA synthesis

Med:20389281, PubMed:21300290, PubMed:23721412). During EMT, involved with LOXL2 ir

ntral pair apparatus of motile cilia.

RNA-induced silencing complex (RISC), which recognizes target mRNAs through imperfect bas

ecies (ROS), hyperosmotic stress, UV irradiation and hypoxia. Involved in DNA damage respo

RNA-induced silencing complex (RISC), which recognizes target mRNAs through imperfect bas

1A. In podocytes, may play a role in the regulation of actin dynamics and/or foot process cytoa
:10873465, PubMed:12509299, PubMed:12547395, PubMed:19941824, PubMed:20028083, F
S100B after myocardial infarction may play a role in myocyte apoptosis by activating ERK1/2
nvolved in the control of the directional transport of ITGB1 in mesenchymal cells by phosphory

uct sphingosine 1-phosphate, stimulates TRAF2 E3 ubiquitin ligase activity, and promotes activ

adian component PER1 (PubMed:28602820). Inhibits PER1 by repressing the CLOCK-ARNTL/

0513427). Dephosphorylates RPS6KB1 and is involved in regulation of cap-dependent translat

h as trabeculation of the developing heart. Isoform 10 may play a role in motor and sensory ne

t genes and can bind to DNA as either monomers or homodimers, depending on the nature of

ly telomerase-synthesized 3' overhangs and to terminate telomerase action implicating the ass

mediated by type 1 interferon (By similarity). Regulates NOD2-induced pro-inflammatory cytoki

APGEF2 and the Ras inhibitor SYNGAP1, promoting their activity. Also regulates synaptic plas
oxic response. Required for HIF1A mRNA stability independent of poly(A) tail length regulation
. Has an inhibitory effect on cytokine production in response to T-cell receptor/CD3 cross-linkin

ubMed:20347428). In complex with CENPT, CENPW and CENPX (CENP-T-W-S-X heterotetra

expression the J and/or S elements in MHC II promoter (PubMed:7969177). Acts as a negative

' reducing its autoinhibitory phosphorylation at Ser-308 thereby increasing its catalytic activity (I

romoter of hypoxia-inducible target genes and hence inhibits HRE-driven transcriptional activat

ıerase reaction. Possesses an intrinsic strand annealing activity.

gulation. May be responsible for the liver-specific activity of enhancer II, probably in combinatic

r and DNA damage response pathways. Mediates ubiquitination and subsequent proteasome c

ependent manner (PubMed:29301984). Acts as a positive regulator of monocyte migration and

r cytoplasmic polyadenylation of mRNAs involved in carbohydrate metabolism, including the glu

l and proliferation in response to BCR stimulation, efficient IgG1 antibody responses to T-cell-d
ckpoint and survival in response to ionizing radiation (PubMed:19238419, PubMed:22132193).

nt regulator of innate immune signaling via regulation of Toll-like receptors (TLRs), Nodlike rece

RNA-induced silencing complex (RISC), which recognizes target mRNAs through imperfect bas
osomal degradation; Lys-33-linked is involved in kinase modification; Lys-48-linked is involved

MYT1, KIZ, PPP1R12A/MYPT1, PRC1, RACGAP1/CYK4, SGO1, STAG2/SA2, TEX14, TOPO
llular chromosome bridges and accumulation of DNA damage: phosphorylates CHMP4C, leadi
skeleton in regulating glucose metabolism (By similarity). Iso acts as a key regulator of chond

of the circadian clock: in the input component, is involved in modulating the magnitude of photi
pendent on class II bHLH factors and namely modulates the differentiation program initiated by

uired for genotoxic stress-induced cell death in breast cancer cells.
IFIT3 sequesters COPS5 in the cytoplasm, thereby increasing nuclear CDKN1B/p27 protein le

;/SCF and KIT promote activation of PLCG1, leading to the production of the cellular signaling

RNA-induced silencing complex (RISC), which recognizes target mRNAs through imperfect bas
3 and stimulates their capacity to induce apoptosis and regulate cell cycle. In response to doub

ɔf MAP kinases, but has no kinase activity (PubMed:15299019, PubMed:26455797).
:hore localization of PLK1 and CENPF. May play a role in the tension sensing mechanism of th

ıtribute to the internal structure of mitotic chromosomes (By similarity). May play a role in chrom

ıful cell division (PubMed:23035123). May be involved in UV-induced apoptosis via its interacti

ɔn of BCAR1. May be involved both in adult synaptic function and plasticity and in brain develo

ɛnt alternative splicing into isoform 3 is required in mechanosensory hair cells in the inner ear fo
8856, PubMed:11705987, PubMed:16260596, PubMed:12944386, PubMed:14751237). The U

the cytokine dimer results in the phosphorylation and the activation of TGFBR1 by the constitut

on of sperm chromatin. Involved in obesity resistance through regulation of metabolic genes su
:tivation of STAT family members STAT1, STAT3, STAT5A and STAT5B. Activation of PLCG1

nant-negative regulator of C/EBP-induced transcription: dimerizes with members of the C/EBP

n is enhanced in the presence of NDFIP1 (PubMed:25631046). Plays a role in dendrite formati

ates the TLR2 signaling pathway to activate specifically the downstream p38 and JNK MAP kin

roxymethylcytosine (5hmC), suggesting that it acts as a specific reader of 5hmC.

LA transcriptional activity in response to TNF and upon glucocorticoid, associates in the cytopl

RNA-induced silencing complex (RISC), which recognizes target mRNAs through imperfect bas
romatin where it recruits different chromatin modifiers required for this chromatin replication. Al

RNA-induced silencing complex (RISC), which recognizes target mRNAs through imperfect bas

)). The interaction with PDCD1/PD-1 inhibits cytotoxic T lymphocytes (CTLs) effector function (
, PubMed:24627487). Also involved in down-regulation of the androgen receptor (AR), enhanci
RNA-induced silencing complex (RISC), which recognizes target mRNAs through imperfect bas

eletal growth, matrix homeostasis and growth plate function (By similarity).

enobiotics such as tazarotenic acid (PubMed:26937021).

ity by blocking granule exocytosis by mediating homophilic binding to adjacent cells, allowing ir

ole in DNA repair by HR. Plays a role in regulating mitochondrial DNA copy number under con

its adenylate cyclase activity, leading to decreased intracellular cAMP levels (By similarity). The

RNA-induced silencing complex (RISC), which recognizes target mRNAs through imperfect bas

n of heat shock response, seems to play a positive feedback with HSF1 to modulate heat-shoc

ed in regulation of apoptosis. May be involved in protecting chondrocytes from apoptotic death

or epithelial fusion during palate development by regulating activation of TGF-beta-3 (TGFB3)

RNA-induced silencing complex (RISC), which recognizes target mRNAs through imperfect bas
by DNASE1 and DNASE1L3 is required to prevent formation of clots that obstruct blood vessel
ng the complex to target sites (By similarity). Inhibits ubiquitination and subsequent degradatior

from mitotic arrest. May play a role for tumor suppression.
nsuring proper chromosome segregation and binding to BUB3 is essential for this function. Can

meric enrichment of AUKRB in prometaphase.

n of two conserved methyltransferase motifs. Functions as a transcriptional corepressor by ass

bMed:15485867). Involved in the regulation of liver cancer cells self-renewal (PubMed:259857

PCM1 (PubMed:29973724). Does not mediate disassembly of all membraneless organelles: d
esting that it may also act as a regulator of smooth muscle cell differentiation and proliferation.

ent (ISRE) in their promoters. Its target genes for transcriptional activation activity include: gene

ssing via its association with pre-mRNA 3'-end processing factors, establishing a link between

te differentiation (PubMed:12925201). Involved in lipid raft organization and CD1D localization

undetectable in vitro, or very low (PubMed:21397848). In addition, cardiolipin is almost exclusi

ome family members, but it is not yet clear if it represents a distinct pathway or if it can be integ

ys seem to involve interactions with G-proteins. May be involved in transduction and intercellul

RNA-induced silencing complex (RISC), which recognizes target mRNAs through imperfect bas

asmic retention of TFEB and TFE3 (PubMed:31672913). Together with FLCN, regulates autop

3, PubMed:27332895). The flexibility of the SAH domain is proposed to allow AURKB/C to follo
1 chorionic somatomammotropin-B gene enhancer.

egion.

s-860'. Participates in the ETV6-mediated repression. Probably plays a role in cell proliferation.

various RNA species. Binds to the U8 snoRNA; metal is not required for RNA-binding. May pla

for example neural stem/progenitor cells and tumor cells, suggesting a role in replication-assoc

ith the highest activity toward 1-octadecanoyl-2-(5Z,8Z,11Z,14Z-eicosatetraenoyl)-sn-glycerol t
ıs, including ARHGEF2, BORA, BRCA1, CDC25B, DLGP5, HDAC6, KIF2A, LATS2, NDEL1, P

with MutS alpha regulates binding similar to a molecular switch: mismatched DNA provokes AD
1177, PubMed:16373578, PubMed:22942274, PubMed:26859324, PubMed:27226593). This c

ote the survival of cells with a compromised HR repair pathway, thereby preventing genomic ha

ı hepatocytes, inhibits growth hormone signaling (By similarity).

ı the nucleus in regulating the expression of various cytokines (PubMed:15793005). May regul:

ⲅNA-induced silencing complex (RISC), which recognizes target mRNAs through imperfect bas

In response to LPS, modulates inflammatory responses by playing a key role on the regulatior

agonizing action of classical E2F proteins (E2F1, E2F2 and/or E2F3). Required for placental de
ⲅNA-induced silencing complex (RISC), which recognizes target mRNAs through imperfect bas
ed:23817040). Plays a role in promoting protection against cell death during hypoxia by decrea

led:12867411, PubMed:15465040, PubMed:18511070).
ss ZBTB7B transcription factor during cytotoxic (CD8+) T cell development. They bind to RUNX

MST2 and STK4/MST1 activation and promotes cell-cycle exit and terminal differentiation in de

ration. Regulates cortical neuronal migration by mediating NTRK2/TRKB anterograde axonal tr

notes brown adipocyte differentiation (By similarity). Inhibitor of WNT signaling (PubMed:20028

CLNS1A from the trapped Sm proteins and their transfer to an SMN-Sm complex triggers the

classes of DNA response elements within the promoter of its target genes and can bind to DN

hosphorylation of NDC80. Involved in regulation of mitotic checkpoint protein complex via phos

eprogramming (PubMed:28740264).

RNA-induced silencing complex (RISC), which recognizes target mRNAs through imperfect bas

required for the increased expression of the thermogenic and anti-inflammatory gene progra

merization with one of the small Maf proteins and binding to ARE elements of cytoprotective ta

by assembling into the HDP-RNP complex, a complex that serves as a platform for IRF3 phos

tion of hepatocytes after partial hepatectomy. Mediates responses to ischemia and hypoxia; re[illegible]

nesis, involved in loading DNA polymerase POLE at the sites of local damage (PubMed:20227[illegible]

f AMPA receptors (AMPARs) in response to synaptic activity: this endocytic pathway maintains

vates EPHA2 by inducing tyrosine phosphorylation which leads to its internalization and degrad

FANCG, FOS, GFAP, GOLGA2/GM130, GRASP1, UBE2A/hHR6A, HIST1H1 proteins/histone

ceramide 1-phosphate/C1P (PubMed:9705349, PubMed:9607309, PubMed:27694435). Also a

soform Alpha. Exerts a dominant negative effect on isoform Alpha trans-repression mechanism

ions in signaling downstream of integrin and collagen receptors, immune receptors, G-protein c

the base of the ciliary membrane. Required for proper BBSome complex assembly and its cilia
trafficking of ATG9A upon activation of autophagy. May regulate the recruitment of ATG9A-AI

A damage sites via PARP9 binding to ribosylated PARP1 (PubMed:23230272). Subsequent P
dependent transcription.
with various co-chaperone proteins or complexes, that act as adapters, simultaneously able to

). Plays a role in the formation of the ciliary vesicle (CV), an early step in cilium biogenesis. Pr

PubMed:11743006). Although inserts the correct base, may cause base transitions and transve

t signaling between antigen presenting cells and lymphocytes. In response to ligation of TNFSF

ceptors subunits BMPR1A, BMPR1B, BMPR2 and ACVR2A, leading to the activation of SMAD
t express chemokine receptors CCR7 and CCR9, by reducing the availability of CCL19, CCL2

totic neurons (By similarity).

rmediates of the riboflavin pathway with by-products arising from other metabolic pathways suc

R of diverse inflammatory mRNAs (such as ceruplasmin), suppressing their translation. Interfe

targets single-stranded DNA and does not deaminate double-stranded DNA or single- or doub

-D1 and CDKN1C/p57Kip2 specifically in quiescent cells. Involved in regulation of the Wnt sig

sponsible for detecting the Picornaviridae family members such as encephalomyocarditis virus

pathway possibly by directly interacting with the posphatidylinositol 3-kinase regulatory subuni

meability. CLDN1 is required to prevent the paracellular diffusion of small molecules through tig

of isomers, with each of them potentially having a specialized function. Induces T-cell prolifera

lyze nitric oxide (NO) production via the reduction of nitrite to NO with NADH or aldehyde as el

:9705271, PubMed:9651580). The complex may also be required for DNA damage signaling vi

ıvolved in neuroblastoma G1/S phase cell cycle progression and cell proliferation inhibition by s

K-related kinases TBK1 and IKBKE which in turn phosphorylate the interferon regulatory factor
ed:24824780).

ria delivered to the cytoplasm (PubMed:26300263). Acts by binding cyclic dinucleotides: recogr

By similarity).

ɛstin involvement appears to vary significantly depending on the receptor, agonist and cell type.

. May also be involved in the differentiation of the fibroblast-like cells in the superficial layer of r

ıat the inhibition of phosphorylation may be the result of sumoylation of STAT1 'Lys-703' (PubM
c dystrophy pathophysiology (DM).
s, binds to the cytokine receptor WSX-1/TCCR. Another important role of IL-27 is its antitumor
ıia virus (MoMLV) and xenotropic MuLV-related virus (XMRV), filoviridae: ebola virus (EBOV) a

ʼnstream partner IRF3 to produce IFN-beta. Plays a neuroprotective role in the eye and optic ne

ssion of the immune checkpoint protein CD274 in ARNTL/BMAL1-deficient macrophages (By si

ɔn chromatin and accelerate DNA ligation during non-homologous end-joining (NHEJ) (PubMec

osome process.

534318). Required for the exosomal release of SDCBP, CD63 and syndecan (PubMed:226604
ılpha-dystroglycan (DAG1) (By similarity).

ass switch mediated by TLR9. Involved in the antigen presentation function of B-cells. Involved

PARP1-dependent PARP9-DTX3L-mediated ubiquitination promotes the rapid and specific re

tion by influencing the assembly and activity of the PRKCZ-SQSTM1-TRAF6 multiprotein signa

clobutane pyrimidine dimers (CPD), 6-4 photoproducts (6-4 PP), apurinic sites and short misma

otic cells, platelet aggregation, cytoskeleton reorganization and engulfment. Functions in the ret

ALX binds and induces tyrosine phosphorylation of PI3-kinase subunits PIK3R1, PIK3R2 and F
controlled by various mechanisms of post-translational modification and subcellular compartm

ocrine regulator in fine-tuning trophoblast invasion generated by the trophoblast itself. The rece

.1 and ITSN1. Involved in endocytosis of cystic fibrosis transmembrane conductance regulator/

phosphatidic acid, another bioactive lipid, through the phosphorylation of 1-alkyl-2-acetyl glyce
ytoplasmic dynein-dynactin motor complexes; SYNE1 and SYNE2 may act redundantly. Requir
ptors in the sarcoplasmic reticulum, regulates the membrane potential. In cochlea cells, its nun

d insulin sensitivity (Probable). Inhibits proliferation, migration, and tubule formation of endothe

ect via the classical RNASEL-dependent pathway or an alternative antiviral pathway independe
coactivator complex. Participates in sex determination and early gonad development by stimul
ntiviral pathway independent of RNase L. Displays antiviral activity against Chikungunya virus (

kappa-B (PubMed:12077347). May play a role in the development of neuromuscular junction (P

the base of the ciliary membrane. The BBSome complex, together with the LTZL1, controls SM

mplexes (nBAF). The npBAF complex is essential for the self-renewal/proliferative capacity of

ne mitochondrial outer membrane regulates the opening of a pore in the mitochondrial double m
ctivity. Component of the ternary complex, cyclin D1/CDK4/CDKN1B, required for nuclear trans

(MMCAT) and cone-rod dystrophy in human patients. [provided by RefSeq, May 2016]

arity). Disrupts insulin signaling by binding directly to Akt kinases and blocking their activation (
eath. Inhibits activation of caspases. Appears to regulate cell death by blocking the voltage-dep

n is mediated by association with SELPLG and dependent on phosphorylation by SRC-family k

slocation of lysosomal proteins from the cytoplasm to the mitochondrial matrix. May function as

s on the function of the cysteine desulfurase complex NFS1-LYRM4/ISD11, which serves as th

activating immune/hematopoietic cell receptor systems. Acts as a negative regulator of myeloic
ed to induce autophagy as an early stress response to small double-stranded RNA and at later

rectly plays a role in Toll-like receptor (TLR) signaling: ability to inhibit TLR-mediated NF-kappa

ed:27281216).||Plays a role in the TP53-regulated cellular response to DNA damage probably b

n which transformation of interstitial fibroblasts into myofibroblasts plus collagen deposition occ

ites of inflammation.

s to be hormone-sensitive. Regulator of chondrogenesis and osteogenesis and inhibits early h
robably via the regulation of cytoskeletal rearrangements. In the nervous system it has been sh

lpha-2-macroglobulin receptor (PubMed:26142438).

ncludes TAK1, TAB2, and TAB3). Activation of the MAP3K7/TAK1 complex by autophosphory

elements (5'-CACGTG-3') found within the promoters of its target genes (PubMed:15560782). I

d:12075506, PubMed:15317753, PubMed:18611980). Also prevents fat infiltration of muscle a

ubMed:10753840).

tivity. Component of the ternary complex, cyclin D2/CDK4/CDKN1B, required for nuclear trans

d exclusion of exon 5 of the NMDA receptor R1 pre-mRNA. Involved in the apoB RNA editing a

xonic enhancer (By similarity). Can regulate alternative splicing of neurexins NRXN1-3 in the la

targets single-stranded DNA and does not deaminate double-stranded DNA or single- or doub

d by human adenovirus E3-19K glycoprotein, which binds the TAP complex and acts as a tapa

egulates protein synthesis by phosphorylating key regulators of mRNA translation and ribosom

(PubMed:28978524). Acts as a receptor for extracellular ubiquitin; leading to enhanced intrac

c, Rap and Rho GTPases activity. Plays an important role in the development of the nervous s

nsported to the Golgi, where it is cleaved in a site-specific manner by resident proteases S1P/I

ylates LAT, which recruits numerous signaling molecules to form the LAT signalosome. The LA

ıbMed:23269243). During inflammation, limits the activity of inflammatory caspases CASP1, CA

gatively regulates IFN-beta production post-pathogen recognition by polyubiquitin-mediated de

ptosis enhancing preferentially the DNA binding and transactivation of p53/TP53 on cell-cycle

activation of transcriptional programs associated with oncogene and proto-oncogene mediated
ation by acting a regulator of immune response in skin: promotes Th1/Th17-dominated immune

tes large amplitude ACh-evoked currents through alpha-4:beta-2 nAChRs. Is involved in regula

medium-chain dicarboxylic fatty acids which are essential regulators of all fatty acid oxidation p

L31RA (PubMed:15194700). Acts as a regulator of inflammatory response by regulating differe
ıs, such as stimulated growth factor receptors, lysosomal enzymes and lipids. The MVB pathwa

:he process by which immature dendritic cells differentiate into fully competent antigen-presenti
ɔf longitudinally projecting axons (By similarity).

ment. Rotation of the central stalk against the surrounding alpha(3)beta(3) subunits leads to hy

RNA-induced silencing complex (RISC), which recognizes target mRNAs through imperfect bas
nal degradation (PubMed:26565908).

and protects neuronal cells from apoptosis (via the N-terminal domain). Induces neuronal proc
on (By similarity).

RNA-induced silencing complex (RISC), which recognizes target mRNAs through imperfect bas

heme-bound DGCR8 dimer binds pri-miRNAs as a cooperative trimer (of dimers) and is active

7 thereby acts as a transcription repressor (PubMed:22722849). Moreover, H3K18 hypoacetyl

ne cell cycle and become committed to their adult state. The transition from proliferating neural

nt role in regulating the choice of the DNA double-strand breaks (DSBs) repair pathway and G2

rowth factor (EGF)-induced MAPK8/JNK1 activation and subsequent JUN phosphorylation. Ph

a PALB2-scaffolded HR complex containing BRCA2 and RAD51C and which is thought to play
in vitro; it is however unclear whether UBE2D1/UBCH5 acts as an E2 enzyme for the APC/C ir

uired for cellular apoptosis in response to cisplatin treatment.

ation.

nd the peripheral clocks that are present in nearly every tissue and organ system. Both the cen

receptor) to neuronal postsynaptic density and may function in localizing synaptic vesicles at sy

of NR5A1 expression (PubMed:19361780).

IL6 and IL12B, during the early phase of inflammation (PubMed:26320658). Prevents aberrant

ental role in kinetochore assembly and function. It is one of the inner kinetochore proteins, with
RNA-induced silencing complex (RISC), which recognizes target mRNAs through imperfect bas

AKT1. Phosphorylates: PTPN1, SRSF1 and SRSF3. Regulates the alternative splicing of tissue
RNA-induced silencing complex (RISC), which recognizes target mRNAs through imperfect bas

, which is required for embryonic stem cell identity and proper differentiation. The PRC2/EED-E
generate more than 100 centrioles (By similarity). Overexpression of CEP152 can drive amplific
RNA-induced silencing complex (RISC), which recognizes target mRNAs through imperfect bas

lammatory genes activated through TLR/IL-1 receptor signaling. May promote apoptosis (By si
in these regions. KMT5C is targeted to histone H3 via its interaction with RB1 family proteins (F

ody energy homeostasis (By similarity). Contributes to the biosynthesis of membrane phospholi

e MHC-I locus) and also by recruiting corepressors (HDACs) or coactivators (HATs) directly to

r. Electrons originating from reduced cytochrome c in the intermembrane space (IMS) are trans

nd the peripheral clocks that are present in nearly every tissue and organ system. Both the cen

ole in myoblast differentiation and also in the down-regulation of cyclin D1 in response to hypox

nd the peripheral clocks that are present in nearly every tissue and organ system. Both the cen

s of autophagy, possibly autophagosome-lysosome fusion and/or lysosomal exocytosis in neur

ter sequence containing the DNA motif 5'-CGGTCA-3'; in cooperation with a PBX protein (such

required for dimerization of the ATP synthase complex and as such regulates ATP synthesis i

d SYK tyrosine kinases leading to activation of PLCG2 (PubMed:18955485).
RNA-induced silencing complex (RISC), which recognizes target mRNAs through imperfect bas

rane recycling of major histocompatibility complex (MHC) class II probably involving retromer a

denylate cyclase, stimulates phospholipase A2, activates potassium channels, inactivates volta

RNA-induced silencing complex (RISC), which recognizes target mRNAs through imperfect bas

ed:9425121, PubMed:10358058, PubMed:17472963, PubMed:18451993). Selectively hydroly

oic acid in unesterified fatty acid (By similarity). Specifically required for blood-brain barrier for

arity).

nd the peripheral clocks that are present in nearly every tissue and organ system. Both the cen

d shear-mediated deflection of the primary cilium, which decreases intracellular cAMP, leads to

dulin signaling pathway by regulating AKT1 activation and NOS3 activation in endothelial cells
esis via RAB11A.

one and mineralocorticoid receptors, that can bind to its GRE mimic region. Multiple functions

apped DNA substrates of a partial duplex DNA structure for helicase loading and translocation a

ıf DNA virus-mediated innate immune response by assembling into the HDP-RNP complex, a c

ment. Rotation of the central stalk against the surrounding alpha(3)beta(3) subunits leads to hy

ٰRNA-induced silencing complex (RISC), which recognizes target mRNAs through imperfect bas

SMCR8 complex also acts as a regulator of autophagy initiation by interacting with the ATG1/U
=10/BAF45A, are exchanged for homologous alternative ACTL6B/BAF53B and DPF1/BAF45B

:s as aminophospholipid translocase at the plasma membrane in neuronal cells.

ٰRNA-induced silencing complex (RISC), which recognizes target mRNAs through imperfect bas

ٰRNA-induced silencing complex (RISC), which recognizes target mRNAs through imperfect bas

ٰRNA-induced silencing complex (RISC), which recognizes target mRNAs through imperfect bas

 BRCA1 (PubMed:19887602). In vitro able to promote polyubiquitination using all 7 ubiquitin Ly

y similarity).

ɔgnitive disability, congenital heart disease, neuroblastoma, and congenital kidney and urinary t

PubMed:19965576, PubMed:20972997, PubMed:10681376). Catalyzes the hydroxylation of ca

RNA-induced silencing complex (RISC), which recognizes target mRNAs through imperfect bas

also phosphorylate FXYD1/PLM which is able to induce chloride currents. May also play a role
7296872).

ɔteins, enrolling them in cell proliferative control (PubMed:22615398).

RNA-induced silencing complex (RISC), which recognizes target mRNAs through imperfect bas

aptic density keeping the channels in an activated state in the presence of glutamate and preve

isions (By similarity).

RNA-induced silencing complex (RISC), which recognizes target mRNAs through imperfect bas

ecretase cleavage of APP by enhancing endoprotelysis of PSEN1.
RNA-induced silencing complex (RISC), which recognizes target mRNAs through imperfect bas
RNA-induced silencing complex (RISC), which recognizes target mRNAs through imperfect bas

f CREBBP to the promoter by a CREST-dependent mechanism, which leads to transcriptional

via direct interaction with NOTCH2 (PubMed:29856954). Also promotes Notch signaling pathwa
or IGF1 stimulation regulates cell growth and proliferation by controlling mitochondrial DNA rep

esenchyme, in turn, triggers epithelial outgrowth and augments MSX2 expression, which cause

r. Electrons originating from reduced cytochrome c in the intermembrane space (IMS) are trans

by RefSeq, Mar 2015]

e is formed by the variable domain of one heavy chain, together with that of its associated light

so a role in synaptic plasticity in adult brain through regulation of synaptogenesis. In addition to
increase translation efficiency. May also participate in respiratory regulation.
CL2L11-DYNLL1 complex dissociates from cytoplasmic dynein and translocates to mitochondri

fluid (CSF) APOE- and APOA1 levels. Together with APOE and the cholesterol transporter AE

ranscriptional activity of MYOD1 through its CDK9-mediated phosphorylation and binds the chr
ase excision repair substrates. The 9-1-1 complex is necessary for the recruitment of RHNO1 t

ntigen-specific T-cell activation in synergy with PDCD1/PD-1, possibly by acting as a corecepto

chloride-bicarbonate, chloride-oxalate, oxalate-formate, oxalate-sulfate and chloride-formate e

ral role in heart morphogenesis and angiogenesis through regulation of cell adhesion and cell r

RNA-induced silencing complex (RISC), which recognizes target mRNAs through imperfect bas

ve regulator of neuronal RAC1 activity (By similarity). Regulates macrophage functions such as

:20959412). Acts as a positive modulator of adenosine receptors ADORA1 and ADORA2A, by
to both the clathrin lattice and to the lipid and protein components of membranes are considere
presenting cells. May have a role in angiogenesis and promote cholesterol crystallization. May

a role in promoting immune suppression and T cell TNF-alpha production during gestation (Pu

ections or viral reinfection (By similarity). Binds specifically to the PRDI element in the promote
signaling of TEK and FLT1 receptors, 2 important receptor families essential for angiogenesis. I
mitochondrial outer membrane permeabilization, in a BAX- and BAK1-independent manner, in
esses (PubMed:25106868). Binds to 3'-UTR ARE of numerous mRNAs (PubMed:20506496, Pu

ʒ1-mediated signaling through the Wnt pathway (By similarity). Negatively regulates TGFB1-me

ning receptor kinases and membrane proteases (PubMed:18757307, PubMed:23589287). Suc

olarity complex and RAP1B (PubMed:20332120). Required for activation of PRKCZ and for the

lipoprotein clearance.

naling events in gamma-delta T-cells through PI3-kinase and MAP kinases. It results in prolifera
ubulin and stabilization of microtubules (PubMed:19893491). Plays a role in the regulation of m
ition of SMPP1M functions to enhance muscle responsiveness to Ca(2+) and promote a contra
inscriptional activation. Acts as a positive transcriptional regulator of cyclin CCND2 expression.

ons as a regulator of internal calcium mobilization across the dense tubular system that affects
optic disk. In addition to axon guidance, also regulates dendritic spines development and matu

Has a protective role in establishing the endothelial barrier; the activity involves coagulation fact
gen type I and IV, vibronectin, tenascin, laminin, fibronectin, fibrin or casein (PubMed:9065413

gative regulator of VEGFC-mediated lymphangiogenesis and angiogenesis. Binding of vascula
ns including the actin regulator WAS and the microtubule-associated proteins MAP2 and MAP

nting apoptosis, optimal cytokine signaling during human natural killer cell development, hepati

antioxidant defense mechanism. Isoform 3 seems to be inactive.
25445488).

lation of its target proteins (PubMed:15525651, PubMed:17565996). Promotes tumor cell invas

APK3 signaling downstream of 5HT4-receptors. Phosphorylation of HDAC5, a repressor of my
o stabilization of KDM4A and subsequent histone H3 'Lys-9' (H3K9) demethylation (By similarit

3 methylated at 'Lys-5' (H3K4me2) in estrogen-regulated genes. Involved in the development c

ard current by internal magnesium.

ls, increasing disulfide reductase activity at the plasma membrane, altering the plasma membra

se of TAF10 from promoters, leading to inhibition of TFIID-dependent transcription (PubMed:2

onist activity. Lactoferroxin A shows preference for mu-receptors, while lactoferroxin B and C h

m effectors by MET leads to the activation of several signaling cascades including the RAS-EF

PubMed:27545619). Recognizes and binds histone H3 crotonylated at 'Lys-9' (H3K9cr), and w

d the peripheral clocks that are present in nearly every tissue and organ system. Both the cent

ral pathway independent of RNase L. The secreted form displays antiviral effect against vesicu

:alcium sensitivity and relaxation kinetics of thin myofilaments. May also be involved in early ne
step, catalyzed by the peptidylglycine amidoglycolate lyase (PAL) domain, is the zinc-depender
ubMed:24705027).

7580308). Homophilic ligation of PECAM1 prevents macrophage-mediated phagocytosis of nei
membrane thus protecting hepatocytes from bile salts. Together with TMEM30A is involved in

MEPE degradation (PubMed:12220505).

remodeling which involves modulating the size and composition of HDL (PubMed:29883800).

:omponent of interferon gamma-induced sensitivity. Plays a key role in apoptosis via the degra

ein kinase B and glycogen synthase kinase-3 and insulin induced stimulation of glucose uptake

4829). Mediates dephosphorylation of NTRK1, NTRK2 and NTRK3 (By similarity). Plays a role

ing mitosis, supports the stabilization and elongation of the intracellular bridge between dividin

storage vesicles containing the glucose transporter SLC2A4/GLUT4 (GLUT4 storage vesicles,

Med:9038201, PubMed:11352563). Involved in the recruitment of both the proinflammatory IL

es adhesion of those leukocytes to endothelial cells. May play a role in regulating leukocyte adh

ival of developing tissues such as the neural tube, branchial arches and somites, thereby contr

s activated following engagement of receptors and plays a role in the activation of other protei

; proteolytic processing of NFKB2, and thereby inhibits non-canonical activation of NF-kappa-B

ated in many fundamental cellular processes, including growth, differentiation, migration, morph

.RAP25 mediates the targeting of LIMK1 to the lamellipodium resulting in its activation and sub

iits prior to initiation (PubMed:17581632). The eIF-3 complex specifically targets and initiates tr

n of NFKB1 by TNF-alpha, nerve growth factor (NGF) and interleukin-1. May play a role in titin/

uring Salmonella enterica serovar Typhi (S.Typhi) epithelial cell infection (PubMed:22042847).

Recruited to inflammasomes by MEFV. Induces formation of pyroptosomes, large supramolecu

mediate tethering and docking events during SNARE-mediated membrane fusion. The CORV

naximal enzymatic activity of Polycomb repressive complex 2 (PRC2); acts as a positive regula
erminal, non-reducing ends of oligosaccharide chains.

JNK-signaling components and motor proteins. Functions as an anti-apoptotic protein and who

ubMed:25861989). Promotes UBP10-induced deubiquitination of TRAF6 in response to DNA d

tion, tissue organization, and the regulation of the immune response (PubMed:12432078, PubM

ıter surface protein A lipoprotein (OspA-L) cooperatively with TLR2 (PubMed:11441107). In co

96, PubMed:21376833). Interaction with CD44 promotes directional cell migration in epithelial a
ining mRNA with large polyribosomes and translation initiation. According to some authors, is r

'ubMed:12699629).||Plays a role in nonsense-mediated mRNA decay (PubMed:18974281, Pub

he actin cytoskeleton (PubMed:12183458). Plays a role in axon guidance, invasive growth and
phogenesis.

ubMed:19525956, PubMed:21613998, PubMed:21720370, PubMed:23602554, PubMed:2360
nbined capacity to interact with RECK extracellularly and recruit the Dishevelled scaffolding pro

)P3 (PubMed:16143324). Also binds 25-hydroxycholesterol and cholesterol (PubMed:1742819
:28561066). Involved in an early step of the formation of preautophagosomal structures (PubM

via caspase-6). Negatively regulates oligodendrocyte survival, maturation and myelination. Play

ecruits and activates caspase-1. In innate immune response believed to act as an integral adap

PubMed:27829214). It may also positively regulate the function of p53/TP53 in cell cycle arres

ption and lipolysaccharide-mediated activation of NFKB. May produce superoxide in the nucleu

orylation.

s to membranes enriched in phosphatidylinositol 4,5-bisphosphate and promotes membrane tu

ave a role in the survival of glioma cells.

d defense against viral protein synthesis and virus replication (By similarity). Inhibits neuronal c
HIF1B, and increased expression of hypoxy-inducible genes. EGLN1 is the most important isoz

. Specifically regulates the expression of FGFR2-IIIb, an epithelial cell-specific isoform of FGFI

so induces the retraction of MVECs lamellipodia and filopodia in a ROCK pathway-dependent r

ated by the GRAM domain (By similarity). At the EPCS, the sterol-binding VASt/ASTER domain

mes for the substrate (PubMed:19075014, PubMed:27373336). In complex with USP12, acts a

!. Coexpression of SMURF2 with SMAD1 results in considerable decrease in steady-state level

onal cells (By similarity). The SCF(FBXL17) complex mediates ubiquitination and degradation [illegible]

sphorylation at 'Ser-65' of PRKN and (2) mediating phosphorylation of ubiquitin, converting PR[illegible]

ars to act as a molecular switch that can vary the balance between NaCl reabsorption and K(+)[illegible]

pressor; isoform 2 has lower transcription repressor activity than isoform 1 and isoform 3.||[Iso[illegible]

a production in the presence of T-cell receptor signaling.
d CD40L/CD154, two cell surface molecules on lymphocytes that are critical for antigen-depen[illegible]

smic reticulum into autophagosomes upon nutrient stress, which then fuse with lysosomes for [illegible]
Med:25762780).

orientation during mitosis (PubMed:23649807).

ine production by mature dendritic cells.

LKL-mediated necroptosis via its role in the biosynthesis of inositol pentakisphosphate (InsP5)

tion.

ı ceramides and unsaturated very long-chain ceramides are also good substrates, whereas sat

J) and directs key events in postsynaptic differentiation. Component of the AGRN-LRP4 recept

n the regulation of cell adhesion and motility through phosphorylation of key regulators of these

980435). Plays a protective role in the cellular response to oxidative stress (PubMed:1913089

eased exposure to acetaldehyde in individuals with the catalytically inactive form may also conf

›Med:8782822). Has actin bundling activity and stabilizes actin filaments against depolymeriza

› KAT5/TIP60, probably explains its transcription activation activity. Functions in association wi

toxicity. Also binds GPC1 in lipid rafts.||Appicans elicit adhesion of neural cells to the extracellu

28296633).
›meres, and the in vitro remodeling of H3.3-containing nucleosomes. Its heterochromatin targe

ıervous systems. Participates in axonal growth, pathfinding and in the modulation of dendritic g

SLC16A11 and SLC16A12 to the plasma membrane (PubMed:21778275). Plays pivotal roles
ıia virus (EIAV), feline immunodeficiency virus (FIV), prototype foamy virus (PFV), Mason-Pfize

ıbMed:17431401). Acts synergistically with ELF4 to transactivate the IL-3 promoter and with El
ability changes induced following virus infection.

›motes the production of newborn neurons, probably by modulating G1 length. Promotes, at le
ılates BCL6-induced transcriptional repression. Binds to E2F1 and MYC and blocks their transc

itself (PubMed:11123922). Binding to cell surface receptors triggers internalization of the chap

tin and adherens junctions components. The homodimeric form may regulate actin filament as

enzymatic activities, such as single-stranded DNA (ssDNA)-dependent ATPase, 5'-3' helicase

ation. When overexpressed, enhanced cell proliferation, a process inhibited by GPC3. Acts als

ta/THRB involves at least in part the inhibition of THRB binding to triiodothyronine response el

5'-3' exonuclease activity on nicked or gapped double-stranded DNA, and exhibits RNase H a
recruitment of GRB2, GAB1, PIK3R1 and SOS1, and mediates activation of RAS, MAPK1/EF

eration of normal fibroblasts (in vitro). Has very high affinity for VEGFA and relatively low protei

/ICP-1, TARC and also for the malaria parasites P.vivax and P.knowlesi. May regulate chemok

d its assembly into elastic fibers.

of lysosomes, causing degradation of mature CTSD by CTSB (PubMed:28073925). In addition

ulates protein synthesis by controlling the activity of initiation factor 2B (EIF2BE/EIF2B5) in the

ymphocyte activation.
here carries the 2,3-enoyl-CoA hydratase and the 3-hydroxyacyl-CoA dehydrogenase activities
ratase and the 3-hydroxyacyl-CoA dehydrogenase activities, while the trifunctional enzyme sub
etylates TSHZ3 and regulates its transcriptional repressor activity. Deacetylates 'Lys-310' in RE

nscriptional coactivators such as CREBBP and EP300. Activity is enhanced by interaction with
A-A*01:01-restricted peptides carry a post-translational modification with oxidation and N-termi
V-4 EBNA3 (QAKWRLQTL), eliciting cytotoxic T cell response.||Allele B*13:02: Presents multip

inding of HNRNPC, leading to regulation of mRNA splicing (PubMed:25719671).

reby affects neural activity, nociceptive processing, pain perception, mood and behavior. Beside
ivator activity of the CLOCK-ARNTL/BMAL1 heterodimer.

of the JAK-STAT pathway (By similarity).

mplex, thereby playing a key role in differentiation of embryonic stem cells and normal develop
and can function as weakly inward rectifying potassium channel (PubMed:8605869, PubMed:8

ently phosphorylates and inactivates the actin binding/depolymerizing factors cofilin-1/CFL1, c

complex N-glycans, plays an important role in the activation of cellular signaling pathways, reo

extracellular matrix proteins, can also act on several nonmatrix proteins such as big endothelia

cells (PubMed:11447109). May act as a regulator of F-actin dynamics (By similarity). As part o

mbinations act as transcriptional activators or repressors, respectively. NF-kappa-B is controlle
self-renewal of liver cancer cells (PubMed:25985737).

y contributes to cutaneous wound healing.

.

plasm. Isoform PML-6 shows restriction activity towards human cytomegalovirus (HCMV) and i

the mitochondrion (PubMed:18838687). Dephosphorylates heat shock protein HSPB1 (By sir
nesis by oligomerising short RNA primers on both leading and lagging strands (PubMed:17893
the AMPK complex assembles, via its C-terminus that bridges alpha (PRKAA1 or PRKAA2) an

ade, and/or by up-regulating CDKN1A, which facilitates active cyclin-dependent kinase (CDK) c

TGF-beta proteins are themselves degraded. By acting on TGF-beta signaling, may regulate
ctosidase (EC 3.2.1.23) and globotriaosylceramide by alpha-galactosidase A (EC 3.2.1.22). S
of mitochondrion-endoplasmic reticulum membrane tethering and modulates calcium ions shut

in vitro PtdIns(3,4,5)P3 > PtdIns(3,4)P2 > PtdIns3P > Ins(1,3,4,5)P4 (PubMed:26504226, Pub

ation and proliferation, and normal heart development. Regulates axon growth and neuronal ce

ansfer of a fatty acyl group at the sn-1 position of phosphatidylcholine (PC) and other glycerop
ition and controls cell cycle G2/M checkpoints on DNA damage (PubMed:10764811, PubMed:1

n of pancreatic PP (polypeptide-producing) cells. Regulates transcription by forming a heterodi

ediate-early genes (PubMed:9770464, PubMed:10436156). In response to mitogenic stimulati

694, PubMed:12117417, PubMed:12225859, PubMed:25998567, PubMed:30867591). The co
nt and the onset of heart contractions.

totic chromatin remodeling mechanism occurs as neurons exit the cell cycle and become comn

itiviral state. In response to type II IFN (IFN-gamma), STAT1 is tyrosine- and serine-phosphory

lays a role in the proteolytic processing of ACE2 (PubMed:24227843). Plays a role in hemosta

he cytokine dimer results in the phosphorylation and the activation of TGFRB1 by the constituti
d:21240187). Plays an important role in podosome formation and associated function, thus reg

PP1 or TP53BP2/ASPP2 is displaced by PPP1R13L/iASPP (PubMed:12524540). In cooperati

NA changes. Its activity is regulated by transcription factors and cytoplasmic proteins that have

d:17069755). Mediates estrogen action in various target organs (PubMed:22452784). Mediates

e suppression of platelet aggregation in the vasculature. Highly expressed in the initial segment

NB2 channel activity (By similarity). Binds NADPH and has NADPH-dependent aldoketoreduct
ey role in the antiproliferative action of IFN-gamma either by inhibiting the ERK activation or by

nd may regulate signal transduction in different cellular processes (Probable). Indirectly regulat
PubMed:28730546, PubMed:30184494). Participates as well in degradation of mitochondrion-a

followed by recruitment of RNF146 and subsequent ubiquitination (PubMed:21478859). Media

idymal maturation and the acrosome reaction. May play a role in sperm-egg binding through its

ɴA12 (PubMed:22238662). Plays a role in the maintenance of hematopoietic stem cells and/or

ıembrane fission events, such as the terminal stages of cytokinesis and enveloped virus buddi[illegible]
[illegible] clock gene ARTNL/BMAL1 and the genes involved in lipid metabolism in the liver (By similarit[illegible]
ıbMed:24440334).

ıds to the Ras activation through the G(s)-alpha signaling pathway. Involved in the cAMP-induc

[illegible]). Plays an essential role in the development of lymphoid tissue-inducer (LTi) cells, a subset n[illegible]

modulator of TGF-beta-signaling by restricting substrate specificity of RNF111 (By similarity). F

[illegible] its deacetylation (PubMed:28167758). In association with RARA, plays a role in the repressio

tes integrin beta5/ITGB5 and thus regulates cell motility. Phosphorylates ARHGEF2 and activa

; apoptosis by caspase activation and in p53-independent manner.

e neurite elongation, while isoform 1 may promoter neurite branching.
se RHEB (PubMed:20381137, PubMed:25936802). Involved in the RCC1/Ran-GTPase pathwa

ng with AICD for APBB1-binding. May modulate calcium-mediated postsynaptic signals (By sim

1 complex, required for BDNF-NTRK2 endocytic trafficking and signaling from early endosome
ones and promotes degradation of histones, thereby participating actively to the exchange of h

ocks the recruitment of the 43S ribosomal complex. Involved in methylation of rRNA.
(PubMed:30220460). Endosome membrane fission of early and late endosomes is essential t
ed:22891260).

2 ubiquitination and proteasomal degradation thereby regulating phosphatidylinositol 3-kinase s

n an HSV-tk promoter.

ates ubiquitination of MAP4K1/HPK1: recognizes and binds autophosphorylated MAP4K1/HPK

strates at multiple levels: by interacting with APC and blocking access of APC substrates to the

ntiation of lung epithelium. Acts cooperatively with FOXP4 to regulate lung secretory epithelial c

ding Xist RNA and recruiting the WMM complex, which mediates m6A methylation, leading to t

ng with CUL1 and thus inhibiting NF-kappa-B inhibitor alpha/NFKBIA degradation that remains

)29197). Represses hypoxia inducible factor's (HIF) activity by interacting with HIF prolyl hydrox

uired for disassembly and recycling of post-termination ribosomal complexes and subsequently
AMP, ADP and ATP, leading to activate or inhibit AMPK: AMP-binding results in allosteric activ

nucleotides over CpH (H=A, T, and C), hemimethylated-CpG and hemimethylated-hydroxymeth

beta-catenin signaling pathway. Binds to and phosphorylates TCF7L2/TCF4 and LEF1, promoti

e generation of bradykinin in the kidney.

glia, olfactory epithelium and cranial skeleton. Plays a role in several important steps of muscle
nd forms of Ran between the cytoplasm and nucleus. Its compartmentalized phosphorylation c

nd tissue cell membranes is the antigen Pk of blood histogroup P (PubMed:10747952).
tal lung development (By similarity).

CLNS1A from the trapped Sm proteins and their transfer to an SMN-Sm complex triggers the

omologous recombination (PubMed:20603073, PubMed:26807646). Plays a key role in contro

kungunya virus (CHIKV), herpes simplex virus type 1 (HHV-1) and human adenovirus (PubMec

em to be of low affinity and retromer SNX proteins may also contribute to cargo selectivity thus
s phosphatidylserine (PS) and to a lesser extent phosphatidylethanolamine (PE). The ATP8A1

on, and thereby plays a role in stress-induced cell cycle arrest (PubMed:23375502). Also acts

phosphatidylinositol (LPI) into phosphatidylinositol (By similarity).

d the peripheral clocks that are present in nearly every tissue and organ system. Both the cent
17, a cargo essential for the homeostatic maintenance of numerous cell surface proteins assoc
the ILV is released. The ESCRT machinery also functions in topologically equivalent membra

'-linked ubiquitination of RIPK1 (PubMed:29883609).

cium-binding protein also transiently associates with immature PCSK6 and regulates its secreti

cifically in the G1/S checkpoint (PubMed:25123532). Reduces neuronal cell survival (PubMed:

sibly that of ER biogenesis-related genes (By similarity). In a neuroblastoma cell line, protects c

er by competing for the binding to E-box elements (5'-CACGTG-3') found within the promoters o

ns (E2F1, E2F2 and/or E2F3). Required for placental development by promoting polyploidizatio

lcium responses (PubMed:23056385). Required for store-operated Ca(2+) entry (SOCE) and C

negative regulator of the canonical Wnt signaling pathway through preventing ubiquitination of b

grin ITGA5:ITGB1 and to a lesser extent ITGAV:ITGB3 and ITGAV:ITGB5 (By similarity). May n

bMed:23401855). However, may play a non-essential for DNA replication: may be involved in t

ooperation with nuclear PKC. Overexpression up-regulates the Erk signaling pathway and prolif

ıbMed:21909076, PubMed:22615920). Acts as a key regulator of angiogenesis by controlling v

esent in all Gram-negative and some Gram-positive bacteria: ADP-Heptose is recognized by Al

nvertase (PC)-mediated cleavage proteolysis (PubMed:23132852). May also have the ability to

galactose over glucose (PubMed:12753898).

ne rapid formation of a branched actin network by first nucleating new mother filaments and the

Phosphorylation by TBK1 on the pLxIS motif leads to recruitment and subsequent activation of t

tiretroviral effects in infected target cells. Selectively targets single-stranded DNA and does not

;7036).

's a role in the maturation of phagosomes that engulf pathogens, such as S.aureus and M.tube

l protein synthesis inhibitor, leading to a global attenuation of cap-dependent translation, and tl

the Notch signal, preventing expansion of the Notch signal into the anterior somite domain (By

PubMed:29915238). Acts as Fe(2+) and 2-oxoglutarate-dependent monoxygenase, catalyzing
RNA-induced silencing complex (RISC), which recognizes target mRNAs through imperfect bas

/ activin receptors whereas the type-1 receptors like ACVR1B act as downstream transducers c

napse via its interactions with CD6 (PubMed:15294938, PubMed:16352806). Mediates homotyp

ts after ionizing irradiation in both the S-phase and the G2 phase of the cell cycle. Involved in tr
) after strand invasion at replication-dependent DNA double-strand breaks; together with PALB

ation and promotes anergy. In B-cells, acts by ubiquitinating SYK and promoting its proteasoma

ı more proteins. Probably necessary for normal neurologic development.

: capping activity is auto-inhibited and inhibition is relieved upon ABI1 interaction. Also shows a

rminal phosphorylation.

), PubMed:19965638, PubMed:20123072). Involved in V(D)J recombination by acting as a cofa

adults, involved in glucose metabolism in adipose tissue, skeletal muscle and liver (Probable).

46591, PubMed:16979567, PubMed:20049431). Found in an inactive form in the cytoplasm of
ng of the agonist hormone 17-beta-estradiol (E2). Increases the binding activity of charybdotox

: cochlea and induces cells apoptosis.

to precursor Z to generate a dithiolene group.

s with normal sodium channel clusters. Required, together with GLDN, for maintaining NFASC

mutation (SHM) by targeting AICDA deamination activity to transcribed dsDNA substrates. In th
:s. Component of the PSAP complex which binds RNA in a sequence-independent manner anc

ne RPB4-RPB7 subcomplex binds single-stranded DNA and RNA (By similarity). Binds RNA.
protein degradation. This type of proteolysis is required in several pathways including spermatc
gap flaps of distinct sequences and lengths; and DNA ligase I (LIG1) on long-patch base excisi

: recruitment of the DNA double-strand break repair factors RAD51 and RAD52 to chromatin, ir
sion and the severity of the disease has been found. As is typical for genes encoding ribosoma

IJ1/ROMK1, KCNA1-5, KCNQ1-5 and KCNE1, epithelial Ca(2+) channels: TRPV5 and TRPV6
sembly (PubMed:22020124).
ation and destruction of CDH1 in a CK1-Dependent Manner, thereby regulating cell migration.||
590923). Plays an important role in L-glutamate and L-aspartate reabsorption in renal tubuli (Pu
asts syncytialization, the spontaneous fusion of their plasma membranes, an essential process
plasma membrane in conjunction with cysteine string protein-alpha/DNAJC5 (PubMed:2079828

146077). As part of the U7 snRNP it is involved in histone 3'-end processing (PubMed:129753

nal exocytosis in sperm (PubMed:23091057). Plays also an important role in the exocytosis of
epression of MYOD1-stimulated differentiation, regulation of the control switch for exiting the ce

F7L2 target genes.

plex with TLR6, promotes sterile inflammation in monocytes/macrophages in response to oxidi
TP5 is an immunomodulating pentapeptide.
A phosphodiester backbone. As an essential component of the RMI complex it is involved in ch

Acts as an 3'-untranslated region (UTR) ARE mRNA-binding adapter protein to communicate si

); additional evidences are however required to confirm this result in vivo (PubMed:29463709).
yrosine in fibroblasts (Probable). May mediate blood-to-retina L-leucine transport across the inr

mediators from the milieu, regulating signal transduction in different cellular processes (PubMe

S promotes TIPIN nuclear localization (PubMed:17141802, PubMed:17296725). Involved in cel

damage response: phosphorylates p53/TP53 at 'Ser-15' and 'Ser-392' and is recruited to the C

quitin on CRLs targets (PubMed:27565346). The functional specificity of the E3 ubiquitin-protei
agosome-lysosome fusion and completion of autophagy (PubMed:30538141). Involved in the I

s functionally with RORA for the regulation of genes involved in liver metabolism (By similarity).

erases (LPLATs) catalyze the reacylation step of the phospholipid remodeling pathway also kn
mediating its growth factor-independent degradation. Involved in the degradation of the inhibito

on. Required for CDK5RAP2, CEP152, WDR62 and CEP63 centrosomal localization and prom

OLD4. They exhibit differences in catalytic activity. Most notably, Pol-delta3 shows higher proof
ing frequently observed in tumors. Also involved in deuterosome-mediated centriole amplificati

PubMed:20129055, PubMed:24065767). Specificity of the HBO1 complexes is determined by

sponse to endoplasmic reticulum stress, acts as a phosphodiesterase by mediating removal of
mRNA decay pathway (Probable).

mutation (SHM) by targeting AICDA deamination activity to transcribed dsDNA substrates. In th

:1 to sites of DNA damage and the PAGR1:PAXIP1 complex is required for cell survival in resp

actin flow during actin-dependent nuclear movement. May be involved in nucleus-centrosome a

nutation (SHM) by targeting AICDA deamination activity to transcribed dsDNA substrates. In th
ochondrial membrane permeabilization but does not require other caspases. May also act as a

684971).
d for normal Na(+)-dependent intestinal nutrient uptake.

nas been called mitochondrial ribosomal protein S35 in the literature. [provided by RefSeq, Jul 2

vided by RefSeq, Jul 2008]

A) DNA is significantly enriched in exonic regions and is associated with gene transcriptional ac
em cells, ultimately affecting neuron pool and cortex size (By similarity). Seems to have lineage

activator of hormone-dependent transcription mediated by nuclear receptors.

elongs to the ribosomal protein S18P family. The encoded protein is one of three that has sigr

RNAs, necessary and sufficient to promote neural stem cell self-renewal. Does so by directly b

(By similarity).

lls by promoting degradation of JAK1, probably by ITCH- and NEDD4L-mediated ubiquitination

e centriolar microtubule wall during early S phase in a PLK4-dependent manner (PubMed:271[illegible]

acts as an autocatalytic E2-like enzyme, catalyzing the conjugation of ATG12 to itself, ATG12 [illegible]

sors of membrane lipids and lipid mediators.

tion. Plays a role in apoptosis negative control involved in brain development.

eavy-chain (Igh) locus independently of the MLL2/MLL3 complex (By similarity). Involved in both

and regulation of telomere length. May participate in regulation of higher-order chromatin orga

CLNS1A from the trapped Sm proteins and their transfer to an SMN-Sm complex triggers the
CLNS1A from the trapped Sm proteins and their transfer to an SMN-Sm complex triggers the

PHP4 in this function (PubMed:22654112).

g that this disaccharide serves as the substrate recognition motif.

ɫolliday junctions (HJs). Resolves HJs by generating two pairs of ligatable, nicked duplex produ

). Binds to and stabilizes centriolar microtubule (PubMed:27185865).

r drugs also promote ubiquitination and degradation of RBM23 and PRPF39 (PubMed:316938

somal degradation (By similarity). By controlling the steady-state expression of the IGF1R recep

somal degradation (PubMed:22573887).

· (PubMed:19289083). Affinity for microtubules is synergistically enhanced in the presence of tl
presence of the ndc-80 complex and may allow the ndc-80 complex to track depolymerizing n

development of obesity (By similarity).

ADE1 and promotes its translocation to the nucleus leading to cooperative inhibition of canoni

een pre-tRNA splicing and pre-mRNA 3'-end formation, suggesting that the endonuclease subu

ng the completion of cilium removal in the later stages of the cell cycle (PubMed:26290419).

erizes on the HJ and the first nick occurs upon dimerization at the junction (PubMed:26578604

ration and effector function. In dendritic cells involved in endosome-to-membrane recycling of r

RNA-induced silencing complex (RISC), which recognizes target mRNAs through imperfect bas

RNA-induced silencing complex (RISC), which recognizes target mRNAs through imperfect bas
lear import is thought to be conferred by an asymmetric distribution of the GTP- and GDP-bou

d less towards RHOA. Essential for the early stages of embryogenesis. May play a role in regu

E2D3. In cooperation with TAX1BP1 promotes ubiquitination of UBE2N and proteasomal degr
ceptor. Phosphorylated STATs then form homodimer or heterodimers and translocate to the nu

omosome congression and is essential for the end-on attachment of the kinetochores to spindle

ulates local microtubule nucleation (PubMed:26165940).

sine and inosine monophosphate and adenylosuccinate that prevents cytoplasmic acidification
HIV-1 gene expression. Isoform 2 and isoform 3 may be involved in apoptosis.

ositive regulation of cyclins, including CCNE1, CCND1 and CCNB1 (PubMed:24854087). Durir
replication after DNA damage and promote postreplication repair: Monoubiquitinated PCNA le

RNA-induced silencing complex (RISC), which recognizes target mRNAs through imperfect bas

the recruitment of CPC to centromeres during early mitosis via association with histone H3 ph

ane trafficking, or cell signaling during hematopoietic maturation (PubMed:10995579).

ivation of caspase-9 which prevents induction of apoptosis (PubMed:15735003).

22665479, PubMed:17418785). May be activated by muramyl dipeptide (MDP), a fragment of l

complex may recruit ARL8B and couple lysosomes to microtubule plus-end-directed kinesin m

F1 complex mediates down-regulation of BIRC5/survivin; the process requires the E3 ligase ac

f polarity information during tissue morphogenesis and/or in differentiated tissues.

RNA-induced silencing complex (RISC), which recognizes target mRNAs through imperfect bas

637).

ors, including the B-cell receptor, CD79A, CD79B, CD5, CD19, CD22, FCER1, FCGR2, FCGR
nospholipase C (PLC) and the formation of inositol 1,4,5-trisphosphate (PubMed:19306925). S

of the neocortex. Involved in cerebral cortex growth (By similarity). Acts as an oncogene for pr

n of the protein (By similarity). Was originally reported to contribute to oxidative stress resistan[illegible]
Med:16488400, PubMed:16707096).
ssociation with RASA1, resulting in recruitment of ARHGAP35 to the cell periphery where it inh
for cerebellar development (PubMed:24766809, PubMed:24766810). Component of the pre-m

of genes involved in the response to oxidative stress by facilitating the SQSTM1-mediated aut[illegible]

e pairing with the miRNA and most commonly results in translational inhibition or destabilizatio

eps of the ATPase cycle, but they also have an individual specificity such that one co-chaperon
e pairing with the miRNA and most commonly results in translational inhibition or destabilizatio

l:17085480). CDKN1A/p21(CIP1) degradation during S phase or following UV irradiation is ess

eps of the ATPase cycle, but they also have an individual specificity such that one co-chaperon

of t-plasminogen activator/PLAT (PubMed:22138752). Stimulates proliferation of melanocytes

of FRS2 triggers recruitment of GRB2, GAB1, PIK3R1 and SOS1, and mediates activation of l
k recovery (PubMed:23966166). Contributes to genomic stability by preventing telomere dysfur

e pairing with the miRNA and most commonly results in translational inhibition or destabilizatio

n EZR. Affects EZR-dependent signaling events, leading to increased activities of the MAPK a

ɔending (PubMed:7957066, PubMed:14506133). Acts as a transcriptional coactivator (PubMed

ıere identity through replication and cell division (PubMed:15475964, PubMed:15282608, PubM

al tracker of dynamic microtubule tips; after chromosomes have congressed, continues to play
and hence cell cycle progression, via its C-terminus. Has a potential role regulating skeletal my

ı negatively regulating pericentromeric heterochromatin transcription (By similarity). SNAI1 recr

e pairing with the miRNA and most commonly results in translational inhibition or destabilizatio

nse and G1/S transition checkpoint by phosphorylating CDC25A, p53/TP53 and p73/TP73. Ph

e pairing with the miRNA and most commonly results in translational inhibition or destabilizatio

rchitecture (By similarity). In the course of podocyte injury, shuttles into the nucleus and acts a
PubMed:20798892). Has only a low DNA repair activity by itself which is stimulated by RAD23E
and p53/TP53 signaling (By similarity). Receptor for amyloid beta peptide. Contributes to the tr
lating vimentin (VIM), an intermediate filament (IF) protein. In epithelial cells, associates with a

ration of NF-kappa-B in response to TNF signaling leading to IL17 secretion (PubMed:2057721

/BMAL1 heterodimer mediated transcriptional activation of PER1. The heterodimer ARNT:AHR

:ion (PubMed:21986499). Inhibits cancer cell proliferation and may act as a tumor suppressor.

ɿuron development. Binds to ERBB4 (PubMed:10867024, PubMed:7902537). Binds to ERBB3

the response element. Binds as a monomer to a response element composed of the consensu

ociation with the ACD:POT1 complex thus interfering with its telomerase stimulation activity. Th

ne secretion and autophagy (PubMed:23991106).

ticity independently of kinase activity, via its interaction with NSF that disrupts the interaction be
.
g; the oral intake of apelin in the colostrum and the milk might therefore modulate immune resp

amer), involved in the formation of a functional kinetochore outer plate, which is essential for ki

e regulator of cell proliferation by specifically activating expression of ARF, a tumor suppressor

By similarity).

tion (PubMed:16126907, PubMed:17998805, PubMed:19694616, PubMed:20416395). May ac

on with other hepatocyte transcription factors. Key regulator of cholesterol 7-alpha-hydroxylase

degradation of DYRK2 in nucleus. Ubiquitinates IGF1R and SNAI1 and promotes them to prote

a negative regulator of monocyte adhesion via the LYN kinase. Stimulates migration of monoc

ucose transporter SLC2A1/GLUT1 (PubMed:28383716). Plays a role in replication-dependent

lependent antigens and for normal induction of germinal center B-cells. May also be involved ir
Associates with chromatin to the CDC25B promoter (PubMed:22132193).

eptors (NLRs) and RIG-I like receptors (RLRs), collectively referred to as pattern recognition re

e pairing with the miRNA and most commonly results in translational inhibition or destabilizatio
in protein degradation via the proteasome; Lys-63-linked is involved in endocytosis, DNA-dama

ORS, p73/TP73, TPT1, WEE1 and HNRNPU. Plays a key role in centrosome functions and the
ng to retain abscission-competent VPS4 (VPS4A and/or VPS4B) at the midbody ring until absc
rogenic commitment of skeletal progenitor cells in response to lipid availability: when lipids leve

ic entrainment and in the output component, contributes to the regulation of a variety of liver clo
the pro-endocrine factor NEUROG3 (By similarity). During myogenesis, may play a role during

vels. Upregulates CDKN1A/p21 by downregulating MYC, a repressor of CDKN1A/p21. Can neg

molecules diacylglycerol and inositol 1,4,5-trisphosphate. KITLG/SCF acts synergistically with [illegible]

e pairing with the miRNA and most commonly results in translational inhibition or destabilizatio[illegible]

le-strand DNA breaks, promotes p53/TP53 phosphorylation on 'Ser-46' and subsequent apopt[illegible]

e spindle-assembly checkpoint by regulating PLK1 kinetochore affinity. Isoform 3 plays a role i

ıatin organization (PubMed:24867636). It is however unclear whether it plays a direct role in ch

on with PRPF19; however, these results need additional evidences (PubMed:24718257).

pment. Activates AKT1 by phosphorylating it on 'Tyr-176'. Phosphorylates AR on 'Tyr-267' and

or derepression of neuronal genes and hearing (By similarity).||Transcriptional repressor which
JV-DDB complex preferentially binds to cyclobutane pyrimidine dimers (CPD), 6-4 photoproduc

tively active TGFBR2. Activated TGFBR1 phosphorylates SMAD2 which dissociates from the r

ıch as PPARA and UCP1.
leads to the production of the cellular signaling molecules diacylglycerol and inositol 1,4,5-trisp

family, impairs their association with C/EBP binding sites in the promoter regions, and inhibits

ion by melanocytes (PubMed:23999003). Involved in the regulation of TOR signaling (PubMed

ases and promote the polarization of macrophages toward the pro-inflammatory M1 phenotype

asm with the glucocorticoid receptor NR3C1 and contributes to RELA inhibition and repression

e pairing with the miRNA and most commonly results in translational inhibition or destabilizatio
so localizes to euchromatic regions where it negatively regulates transcription possibly by impa

e pairing with the miRNA and most commonly results in translational inhibition or destabilizatio

By similarity). The blockage of the PDCD1-mediated pathway results in the reversal of the exha
ing ubiquitination and proteasome-mediated degradation of AR, probably by recruiting NEDD4
e pairing with the miRNA and most commonly results in translational inhibition or destabilizatio

ıteraction with and phosphorylation by LCK and interaction with the TCR/CD3 complex which r

ditions of oxidative stress in the presence of RAD51C and XRCC3. Also involved in interstrand

inactive GDP-bound form prevents the association of RGS14 with centrosomes and is require

e pairing with the miRNA and most commonly results in translational inhibition or destabilizatio

k downstream gene expression (PubMed:17603013). Plays a role in the regulation of hemato

by associating with cytochrome C and interfering with apoptosome assembly.||NAD-dependen

(By similarity).

e pairing with the miRNA and most commonly results in translational inhibition or destabilizatio
ls and cause organ damage following inflammation (By similarity).
ı of TP53, and thereby plays a role in regulating transcription of TP53 target genes (PubMed:1

ı regulate chromosome segregation in a kinetochore-independent manner. Can phosphorylate

ociating with ZHX1. Required for DUX4 silencing in somatic cells (PubMed:27153398).

37).

isassembly of P-body and nucleolus is not regulated by DYRK3 (PubMed:29973724). Dissoluti
Represses the cytomegalovirus enhancer.

s involved in anti-viral response, such as IFN-alpha/beta, DDX58/RIG-I, TNFSF10/TRAIL, OAS

pre-tRNA splicing and pre-mRNA 3'-end formation, suggesting that the endonuclease subunits

on thymocytes and antigen-presenting cells, which plays an important role in natural killer T-ce

ively found on the inner mitochondrial membrane, while PLD6 localizes to the outer mitochondi

rated in the canonical pathway, as PKC seems to be required for Wnt-mediated inactivation of

ar transmission of polarity information during tissue morphogenesis and/or in differentiated tiss

e pairing with the miRNA and most commonly results in translational inhibition or destabilizatio

hagy: following phosphorylation by ULK1, interacts with GABARAP and promotes autophagy ([illegible]

w substrates on dynamic microtubules while ensuring CPC docking to static chromatin (By sim

. Overexpression induces multinucleated cells, suggesting that it is required to accomplish norr

ay a role in the regulation of snoRNAs and mRNAs degradation. Acts also as a phosphatase; h

:iated DNA repair.

:he main diacylglycerol intermediate within the phosphatidylinositol turnover cycle (PubMed:197
'ARD3, PPP1R2, PLK1, RASSF1, TACC3, p53/TP53 and TPX2. Regulates KIF2A tubulin depo

)P-->ATP exchange, resulting in a discernible conformational transition that converts MutS alph
:omplex transformation is initiated by abstraction of hydrogen at carbon 13 (with S-stereochemi

avoc by resolving unrepaired lesions (By similarity). The polymerase acts by binding directly the

ate the expression of numerous cytokines, such as TNF, CCL2, CCL5, CXCL1, IL1A and IL10

e pairing with the miRNA and most commonly results in translational inhibition or destabilizatio

of TLR4 signaling and in the nuclear translocation of RELA/NF-kappa-B p65 and the secretio

evelopment by promoting polyploidization of trophoblast giant cells. Also involved in DNA dama
e pairing with the miRNA and most commonly results in translational inhibition or destabilizatio
sing mitochondria ROS levels in a HK2-dependent manner through a mechanism that is indep

-binding sequence within the ZBTB7B locus acting as transcriptional silencer and allowing for

eveloping epithelial tissues. Plays a role in centrosome disjunction by regulating the localizatio

ransport during brain development (By similarity). Acts as an adapter that bridges the interactio

3982).

assembly of core snRNPs and their transport to the nucleus.

A as either monomers or homodimers, depending on the nature of the response element. Bind

sphorylation of CDC20 and MAD2L1. Plays an active role in chromatin condensation during the

e pairing with the miRNA and most commonly results in translational inhibition or destabilizatio

ıs in fat. Required for some cold-induced thermogenic responses, suggesting a role in metabol

ırget genes (PubMed:19489739, PubMed:29590092). The NFE2L2/NRF2 pathway is also activ

ɔhorylation and subsequent innate immune response activation through the cGAS-STING pathv

gulates the expression of proteins such as IL1B and CXCL2 that are involved in inflammatory p

374).

levels of surface AMPARs in response to chronic changes in neuronal activity through synapti

lation. Acts as a negative regulator in the tumorigenesis of gliomas by down-regulating EPHA2

H1, HMGA1, HIVEP3/KRC, LMNA, LMNB, LMNC, LBR, LATS1, MAP1B, MAP4, MARCKS, M

acts on N-oleoyl ethanolamine phosphate/N-(9Z-octadecenoyl)-ethanolamine phosphate, a pot

(PubMed:20484466).||[Isoform Alpha-B]: More effective than isoform Alpha in transcriptional a

coupled receptors (GPCR), cytokine, chemokine and growth factor receptors, and mediates res

ary localization.
P2-containing vesicles to autophagic membranes (PubMed:24603492).

ARP1-dependent PARP9/BAL1-DTX3L-mediated ubiquitination promotes the rapid and specifi

interact with the specific client and the central chaperone itself (PubMed:29127155). Recruitm

oposed to be required for the fusion of distal appendage vesicles (DAVs) to form the CV by rec

ersions depending upon the context. May play a role in hypermutation at immunoglobulin genes

F14/LIGHT, delivers costimulatory signals to T cells, promoting cell proliferation and effector fu

'1-SMAD5-SMAD8 complex. The regulation of chondrogenic differentiation is inhibited by NOG

1, and CCL25 through internalization. Negatively regulates CXCR3-induced chemotaxis. Regul

:h as glycolysis. Typical potent antigenic metabolites are 5-(2-oxoethylideneamino)-6-D-ribitylar

:ron-gamma can therefore redirect, in specific cells, the EPRS1 function from protein synthesis

ɔle-stranded RNA. Exhibits antiviral activity against simian immunodeficiency virus (SIV), hepat

naling pathway probably by association with TCF7L2 and preventing TCF7L2 and CTNNB1 as:

; (EMCV) and mengo encephalomyocarditis virus (ENMG). Can also detect other viruses such :

t p85 (PIK3R1/PIK3R2) and disrupting the association between PIK3R1/PIK3R2 and the catal

ght junctions in the epidermis and is required for the normal barrier function of the skin. Require

ation.

ectron donor. May play a role in adipogenesis.

a activation of the ATM kinase (PubMed:15064416). In telomeres the MRN complex may modu

stimulating ubiquitination of NF-kappa-B subunit RELA and NF-kappa-B degradation in a COM

s IRF3 and IRF7, activating transcription of antiviral immunological genes including the IFN-alp

nizes and binds cyclic di-GMP (c-di-GMP), a second messenger produced by bacteria, and cyc

. Internalized arrestin-receptor complexes traffic to intracellular endosomes, where they remain

nandibular condylar cartilage into chondrocytes.

Med:29858569). Mono-ADP-ribosylates STAT6; enhancing STAT6-dependent transcription (Pu

activity as well as its antiangiogenic activity with activation of production of antiangiogenic che
nd marburg virus (MARV), togaviridae: sindbis virus (SINV) and Ross river virus (RRV). Specifi

erve. May act by regulating membrane trafficking and cellular morphogenesis via a complex tha

milarity).

d:21211721).

13).

I in B-cell chemotaxis in response to CXCL13 and sphingosine 1-phosphate (S1P). Required fo

cruitment of 53BP1/TP53BP1, UIMC1/RAP80, and BRCA1 to DNA damage sites (PubMed:23

aling complex. Functions as an HDAC-dependent corepressor for a subset of GFI1 target gene

atches (PubMed:15448697, PubMed:16260596, PubMed:16407242, PubMed:16940174). Also

tinal pigment epithelium (RPE) as a regulator of rod outer segments fragments phagocytosis. P

PIK3R3; but also GRB2, PLCG1, LCK and PTPN11. Other downstream substrate candidates fo
entalization as well as by interactions with other cofactors or corepressors. NF-kappa-B comple

ptor is also essential for normal gonadotropin-released hormone physiology and for puberty. Th

CFTR. Involved in endocytosis of megalin/LRP2 lipoprotein receptor during embryonal develop

rol (PubMed:22627129).
ed for centrosome migration to the apical cell surface during early ciliogenesis. May be involve
nber and kinetic properties partly determine the characteristic frequency of each hair cell and th

lial cells and reduces vascular leakage (PubMed:14583458, PubMed:17068295). Upon heterol

nt of RNASEL (PubMed:21142819). In addition, it may also play a role in other cellular process
ating transcription activation of SRY. Plays a role in controlling left-right patterning during embr
CHIKV), Dengue virus, Sindbis virus (SINV) and Semliki forest virus (SFV).

'ubMed:26924529). May play a role in late myogenic differentiation (By similarity).

MO ciliary trafficking and contributes to the sonic hedgehog (SHH) pathway regulation. Require

the multipotent neural stem cells. The nBAF complex along with CREST plays a role regulating

nembrane in order to mediate the translocation of lysosomal proteins from the cytoplasm to the
location and activity of the cyclin D-CDK4 complex. Exhibits transcriptional corepressor activity

(By similarity). May bind directly to and mask the 'Thr-308' phosphorylation site in AKT1 (By sim
endent anion channel (VDAC) by binding to it and preventing the release of the caspase activa

kinases. Interacts with HIV-1 matrix protein and is packaged into virions and overexpression ca

a tumor suppressor.

e sulfur donor for cluster synthesis, the iron-binding protein frataxin as the putative iron donor,

d cell proliferation/survival and chemotaxis, mast cell degranulation, immune cells homeostasis
stages of prolonged stress to activate caspase-dependent proteolytic cleavage of BECN1 to te

a-B activation and cytokine production is probably a consequence of its role in TGF-beta-1 sign

by cooperating with TP53 (PubMed:16897187, PubMed:18223688).

curs. The CUB domain has mitogenic activity in coronary artery smooth muscle cells, suggestin

ealing of bone fractures. Positively regulates PDPK1 kinase activity by stimulating its dissociati
nown to control the growth initiation of astrocytic processes in culture and to participate in trans

lation results in the induction and expression of NF-kappa-B and MAPK-responsive inflammato

Negatively regulates its own expression and the expression of DBP and BHLHE41/DEC2 (Pub

ınd bone marrow, helping to maintain the volume and strength of skeletal muscle and bone (Pu

location and activity of the cyclin D-CDK4 complex (By similarity).

ıctivity. Increases COX2 mRNA stability and inhibits COX2 mRNA translation in epithelial cells

minin G-like domain 6 containing the evolutionary conserved neurexin alternative spliced segm

ɔle-stranded RNA. Exhibits antiviral activity against simian immunodeficiency virus (SIV), hepat

ısin inhibitor, preventing MHC class I/TAP association. Expression of TAP1 is down-regulated t

e synthesis. mTORC1 phosphorylates EIF4EBP1 and releases it from inhibiting the elongation

:ellular calcium ions and reduced cellular cAMP levels (PubMed:20228059). Binds bacterial lipo

ystem controlling different steps of axonal guidance including the establishment of the corticos

MBTPS1 and S2P/MBTPS2. The released N-terminal cytosolic domain is translocated to the nı

AT signalosome propagates signal branching to three major signaling pathways, the calcium, th

ASP4 and CASP5 by suppressing their caspase-recruitment domain (CARD) oligomerization a

gradation of IRF3. Mediates the ubiquitin-mediated proteasomal degradation of IgG1 heavy ch

arrest target genes over proapoptotic target genes. May also regulate the ubiquitination and st

growth induction, tumor suppressor mediated growth arrest and replicative senescence, apopt
environment contributing to the development of basaloid skin tumors (By similarity). May act a

ation of the nAChR pentameric assembly in the endoplasmic reticulum. Shifts stoichiometry fror

athways (By similarity). Also involved in the degradation of long-chain dicarboxylic acids throug

entiation of naive CD4(+) T-cells into T-helper Th17 or regulatory T-cells (Treg): deacetylation a
ay appears to require the sequential function of ESCRT-O, -I,-II and -III complexes. ESCRT-III

ing cells that initiate T-cell responses. Mediates mitotic non-disjunction and chromosome instab

drolysis of ATP in three separate catalytic sites on the beta subunits (By similarity).

e pairing with the miRNA and most commonly results in translational inhibition or destabilizatio

cess outgrowth and the coordinate expression of genes encoding synaptic proteins. Exerts its r

e pairing with the miRNA and most commonly results in translational inhibition or destabilizatio

e in triggering pri-miRNA cleavage, whereas the heme-free DGCR8 monomer binds pri-miRNA

ation has been reported as a marker of malignancy in various cancers and seems to maintain

stem/progenitor cells to postmitotic neurons requires a switch in subunit composition of the np

2/M checkpoint activation; DNA-dependent ATPase activity is essential for this function (PubM[illegible]

osphorylates RIN1, inducing RIN1 binding to 14-3-3 proteins YWHAB, YWHAE and YWHAZ a

a role in DNA repair by HR. Plays a role in regulating mitochondrial DNA copy number under [illegible]
vivo. Also involved in ubiquitination and subsequent degradation of VHL, resulting in an accur

ntral and peripheral clocks can be reset by environmental cues, also known as Zeitgebers (Ger[illegible]

ynapses where it is recruited by beta-catenin and cadherin. Required to localize Kir2 channels,

: T-cell-mediated immune reaction by degradation of multiple mRNAs controlling T-cell activatic

most further proteins binding downstream. Required for normal chromosome organization and
e pairing with the miRNA and most commonly results in translational inhibition or destabilizatio

e factor (F3) pre-mRNA in endothelial cells. Phosphorylates PAGE4 at several serine and threo
e pairing with the miRNA and most commonly results in translational inhibition or destabilizatio

EZH2 complex may also serve as a recruiting platform for DNA methyltransferases, thereby link
cation of centrioles (PubMed:20852615).
e pairing with the miRNA and most commonly results in translational inhibition or destabilizatio

milarity). Involved in the induction of T helper 17 cells (Th17) differentiation upon recognition of
RB1, RBL1 and RBL2) (By similarity). Facilitates TP53BP1 foci formation upon DNA damage ar

pids, cholesterol esters and triglycerides (By similarity).

promoters and enhancers. Modulates genes that are essential in the maturation of the immune

sferred via the dinuclear copper A center (CU(A)) of subunit 2 and heme A of subunit 1 to the a

ntral and peripheral clocks can be reset by environmental cues, also known as Zeitgebers (Gerr

xia in adrenal cells suggesting MAPK12 may inhibit cell proliferation while promoting differentiat

ntral and peripheral clocks can be reset by environmental cues, also known as Zeitgebers (Gerr

roblastoma cells (PubMed:23801752, PubMed:29968769). May play a role in homologous reco

as PBX2) is proposed to be involved in the transcriptional activation of EPHA8 in the developi

n the mitochondria (PubMed:21345788, PubMed:29917077).

e pairing with the miRNA and most commonly results in translational inhibition or destabilizatio

nd subsequently allowing antigen sampling, loading and presentation during T-cell activation. Ir

ge-dependent calcium-channels and modulates inositol phospholipid hydrolysis (PubMed:1007

e pairing with the miRNA and most commonly results in translational inhibition or destabilizatio

zes sn-2 arachidonoyl group from membrane phospholipids, providing the precursor for eicosa

nation and function, probably by mediating lipid transport (By similarity). Not required for centra

ıtral and peripheral clocks can be reset by environmental cues, also known as Zeitgebers (Gerı

ɔ cilium shortening and thus decreases flow-induced signaling (By similarity). May be an ion-ch

(PubMed:29104511).

have been associated with this transcript, including cellular growth arrest and apoptosis. It has

along DNA in a 5' to 3' direction (PubMed:18499658, PubMed:22102414). The helicase activity

complex that serves as a platform for IRF3 phosphorylation and subsequent innate immune res

drolysis of ATP in three separate catalytic sites on the beta subunits (By similarity).

e pairing with the miRNA and most commonly results in translational inhibition or destabilizatio

JLK1 kinase complex and modulating its protein kinase activity (PubMed:27617292). Positively
or DPF3/BAF45C subunits in neuron-specific complexes (nBAF). The npBAF complex is esse

e pairing with the miRNA and most commonly results in translational inhibition or destabilizatio

e pairing with the miRNA and most commonly results in translational inhibition or destabilizatio

e pairing with the miRNA and most commonly results in translational inhibition or destabilizatio

ys residues, but may prefer 'Lys-11'-, 'Lys-27'-, 'Lys-48'- and 'Lys-63'-linked polyubiquitination (

tract anomalies. Altered expression of some gene family members is associated with several ty

arbon-hydrogen bonds. Exhibits high catalytic activity for the formation of hydroxyestrogens fro

e pairing with the miRNA and most commonly results in translational inhibition or destabilizatio

in synaptic plasticity.

e pairing with the miRNA and most commonly results in translational inhibition or destabilizatio

enting synaptic depression.

e pairing with the miRNA and most commonly results in translational inhibition or destabilizatio

e pairing with the miRNA and most commonly results in translational inhibition or destabilizatio
e pairing with the miRNA and most commonly results in translational inhibition or destabilizatio

activation. The CREST-BRG1 complex also binds to the NR2B promoter, and activity-depende

ay in a cell-autonomous manner through inhibition of cis DLL1-NOTCH2 interactions, which pro
plication and transcription, the ratio of mitochondria-to nuclear-encoded components of the elec

es the mammary mesenchyme to inhibit hair follicle formation within the nipple sheath (By simil

sferred via the dinuclear copper A center (CU(A)) of subunit 2 and heme A of subunit 1 to the a

chain. Thus, each immunoglobulin has two antigen binding sites with remarkable affinity for a p

its function in the nervous system, the interaction of EPHA5 with EFNA5 mediates communica

ia and sequesters BCL2 thus neutralizing its antiapoptotic activity.

3CA1, plays a key role in the maturation of glial-derived, nascent lipoproteins. Required for rem

romatin of promoters and enhancers of muscle-specific genes; this event correlates with hyper[illegible]
to sites of double-stranded breaks (DSB) occurring during the S phase. RAD9A possesses 3'->

or for PDCD1/PD-1 (By similarity). Negatively regulates the proliferation, activation, effector fun

exchange. Apical membrane chloride-bicarbonate exchanger that mediates luminal chloride abs

nigration. EPHB4-mediated forward signaling controls cellular repulsion and segregation from I

e pairing with the miRNA and most commonly results in translational inhibition or destabilizatio

s CSF-1 directed motility and phagocytosis through the modulation of RAC1 activity (By similar

enhancing their ligand affinity via conformational change (PubMed:23193172). Stimulates plas
ed to be the major clathrin adaptors contributing the CCV formation. AP-2 also serves as a carg
y have a role in amino acid transport by acting as binding partner of amino acid transporter SL(

ubMed:28614294). Regulates RPS6KB1 signaling, which promotes endothelial cell senescence

r of the beta-interferon gene (PubMed:1851123). Drives the maturation of B-lymphocytes into I
Required for the phosphorylation and activation of STAT3, a transcription factor involved in cell
response to inhibition of ERAD-proteasome degradation system, resulting in cytochrome c rele
ubMed:25106868, PubMed:14981510). Promotes ARE-containing mRNA decay of the low-den

ediated activation of SMAD2/3 by mediating the internalization of TGFBR1 from membrane raft

h effectors include PKN2, the RhoGTPases RAC1 and RHOA, Rho-kinases and phospholipas

localization of phosphorylated PRKCZ, PARD3, TIAM1 and RAP1B to the cell junction (PubM

ation and production of cytokines and growth factors by T-cells that in turn stimulate epithelial ti
microtubule dynamics, and thereby contributes to the regulation of cell proliferation, cell polariza
actile state. Phosphorylates MYL12B in non-muscle cells leading to reorganization of actin cyto
. Binds to the cyclin CCND2 promoter region. Associates with chromatin at the NF-kappa-B pro

platelet granule secretion pathways and aggregation. Also required for the formation of a dive
ration and stimulates the formation of excitatory synapses. Upon activation by EFNB1, abolish

tor X. Regulates endothelial cell barrier integrity during neutrophil extravasation, probably follov
;, PubMed:2172980, PubMed:7923219, PubMed:10347120, PubMed:10455171, PubMed:1237

ır growth factors to isoform 1 or isoform 2 leads to the activation of several signaling cascades;
T. Promotes cell survival by phosphorylating AGAP2/PIKE-A and preventing its apoptotic cleava

c regeneration, gonadotropin-releasing hormone neuron survival and migration, platelet activat

sion and metastasis by activating RhoA/ROCK signaling pathway and up-regulating proinflamm

/ocyte enhancer factor 2 (MEF2) leading to nuclear export of HDAC5 and allowing MEF2-medi
:y). Promotes cholesterol efflux by acting as a transcription coactivator (PubMed:19481530). Ac

›f multiple endoderm-derived organ systems such as liver, pancreas, lung and prostate; FOXA1

ane redox state and enhancing cell migration (PubMed:21670307). Ligand for CD44; the intera

5959397). LOXL2-mediated deamination of TAF10 results in transcriptional repression of gene

nave somewhat higher degrees of preference for kappa-receptors than for mu-receptors.||Lacto

RK, PI3 kinase-AKT, or PLCgamma-PKC. The RAS-ERK activation is associated with the morp

vith slightly lower affinity histone H3 crotonylated at 'Lys-18' (H3K18cr) (PubMed:27105114). Al

ral and peripheral clocks can be reset by environmental cues, also known as Zeitgebers (Germ

lar stomatitis virus (VSV), herpes simplex virus type 2 (HSV-2), and encephalomyocarditis virus

euronal development.
nt cleavage of the N-C-alpha bond, producing the alpha-amidated peptide and glyoxylate (PubM

ighboring viable leukocytes by transmitting a detachment signal (PubMed:12110892). Promote
uptake of the synthetic drug alkylphospholipid perifosine. Involved in the microvillus formation i

Also plays a key role in the uptake of cholesterol from peripheral cells and tissues that is subse

dation of the apoptotic inhibitor MCL1. May be involved in the inflammatory response pathway

(By similarity).

in down-regulation of signaling cascades that lead to the activation of Akt and MAP kinases (B

g cells. Cooperates with EXOC2 to recruit other components of the exocyst to the early midbod

, or GSVs). These vesicles provide a stable pool of SLC2A4 and confer increased responsiven

17 producing helper T-cells (Th17) and the regulatory T-cells (Treg) to sites of inflammation. R

hesion and migration processes at the endothelium (PubMed:9024663, PubMed:9177350).

ributing to organogenesis (By similarity).

n tyrosine kinase (PTK) families. Receptor clustering or dimerization leads to recruitment of SR

·. Promotes ubiquitination and proteasomal degradation of MAP3K14.

ıogenesis, cell-to-cell and cell-to-matrix interactions. They are required for instance in the prop

sequent phosphorylation of CFL1 which is important for lamellipodial F-actin regulation (By sim

ranslation of a subset of mRNAs involved in cell proliferation, including cell cycling, differentiatic

/TTN downstream signaling in muscle cells. May regulate signaling cascades through ubiquitina

ılar structures composed of oligomerized PYCARD dimers which form prior to inflammatory apo

/ET complex is proposed to function as a Rab5 effector to mediate early endosome fusion prob

tor of PRC2 activity by bridging the pre-existing histone H3K27me3 and newly recruited PRC2

ɔse level seems to influence the beta-cell death or survival response. Acts as a scaffold protein

amage (PubMed:25861989). May control negatively TRAF2-mediated NF-kappa-B activation s

Med:20881139). Plays a role in integrin-mediated cell adhesion through BCAR1-CRK-RAPGEF

mplex with TLR4, promotes sterile inflammation in monocytes/macrophages in response to oxi

and tumor cells (PubMed:20962267). In lymph nodes (LNs), controls fibroblastic reticular cells (
not involved in the nucleocytoplasmic export of unspliced (CTE)-containing RNA species accor

ɔMed:19060897, PubMed:20930030, PubMed:17053788). Is thought to provide a link to the m[illegible]

cell migration (PubMed:15184888). May modulate the activity of RAC1 and CDC42 (By similar[illegible]

[illegible]1106, PubMed:23364794, PubMed:25038827, PubMed:26101257, PubMed:22056990, PubM[illegible]
tein intracellularly (PubMed:30026314). Binds to the glycosaminoglycans heparin, heparin sulf[illegible]

[illegible]3).
ed:20505359, PubMed:28561066). Binds and is activated by phosphatidylinositol 3-phosphate

ys a role in signaling cascades triggered by stimulation of T-cell receptors, in the adaptive immu

oter in the assembly of the inflammasome which activates caspase-1 leading to processing and

st and apoptosis through direct interaction, preventing its MDM2-dependent ubiquitin-mediated

us and play a role in regulating gene expression upon cell stimulation. Isoform 3 is not function

bulation. Has lower affinity for membranes enriched in phosphatidylinositol 3-phosphate.

lifferentiation and neurite outgrowth mediated by NGF via its effect on mTORC1 activity. Requi
zyme under normoxia and, through regulating the stability of HIF1, involved in various hypoxia-i

R2. Plays a role in myogenic differentiation.

manner (By similarity).||[Isoform B]: Mainly function in endothelial cells and vascular smooth mu

binds to the cholesterol in the PM and facilitates its transfer from the PM to ER (By similarity).

as a potential tumor suppressor by positively regulating PHLPP1 stability (PubMed:24145035).

l of SMAD1 protein and a smaller decrease of SMAD2 level. Negatively regulates TGFB1-induc

of BACH1 (PubMed:24035498, PubMed:30190310). The SCF(FBXL17) complex is also involve

KN to its fully-active form (PubMed:24660806, PubMed:24751536, PubMed:24784582, PubMe

secretion to maintain integrated homeostasis. Phosphorylates NEDD4L. Acts as a scaffold to

form 3]: Functions as transcription repressor; its activity is marginally lower than that of isoform

dent-B-cell development (PubMed:11268217). Binds to A/T-rich promoters (PubMed:1126821

endoplasmic reticulum turnover (PubMed:31006538, PubMed:31006537).

and inositol hexakisphosphate (InsP6). Binding of these highly phosphorylated inositol phosph

urated very long-chain ceramides and short-chain ceramides are poor substrates (PubMed:200

tor complex that induces the phosphorylation and activation of MUSK. The activation of MUSK

e processes such as BCAR1, CRK, CRKL, DOK1, EFS or NEDD9. Phosphorylates multiple rec

5, PubMed:23474458, PubMed:26980435).
er greater susceptibility to many types of cancer. This gene encodes a mitochondrial isoform, w

tion in vitro (PubMed:28893863).

th TSHZ3, SET and HDAC factors as a transcriptional repressor, that inhibits the expression of

ılar matrix and may regulate neurite outgrowth in the brain.||Functions as a cell surface recepto

ting is proposed to involve a combinatorial readout of histone H3 modifications (specifically me

growth and morphology. Major regulator of synaptic transmission and plasticity at adult synapse

in spermatogenesis, embryo implantation, neural network formation and tumor progression. St
er monkey virus (MPMV), human T-cell leukemia virus type 1 (HTLV-1), Rous sarcoma virus (R

.F2 to transactivate the BLK promoter (PubMed:10207087, PubMed:14970218). Inhibits KAT6

ast in astrocytes, changes in patterns of gene expression, changes in the actin cytoskeleton in
criptional activator activity but has no effect on MYC transcriptional repression. Binds to TOP1/T

erone-client complex and subsequent lysosomal or proteasomal degradation (PubMed:215057

sembly and inhibit actin branching by competing with the Arp2/3 complex for binding to actin fil

and endonuclease activities. While the ATPase and endonuclease activities are well-defined a

as a serine exopeptidase with a dipeptidyl peptidase activity that regulates various physiologi

ements (TREs) by NR2F6. Inhibits NFATC transcription factor DNA binding and subsequently i

ctivity. Also involved in replication and repair of rDNA and in repairing mitochondrial DNA.
K2, MAPK3/ERK1 and the MAP kinase signaling pathway, as well as of the AKT1 signaling pa

n kinase activity; may function as a negative regulator of VEGFA signaling by limiting the amou

ine bioavailability and, consequently, leukocyte recruitment through two distinct mechanisms: v

, functions as wound-related growth factor that acts directly on dermal fibroblasts and endothel

same manner as glycogen synthase. In Wnt signaling, GSK3B forms a multimeric complex witl

while the trifunctional enzyme subunit beta/HADHB bears the 3-ketoacyl-CoA thiolase activity
ɔunit beta/HADHB described here bears the 3-ketoacyl-CoA thiolase activity (PubMed:8135828
ΞLA and thereby inhibits the transcriptional activity of NF-kappa-B. Deacetylates NR1D2 and ak

both, NCOA1 or NCOA2. Interaction with redox regulatory protein APEX seems to activate CT.
ıal acetylation being the most frequent (PubMed:25880248). Fails to present highly immunoge
ɔle HIV-1 epitopes derived from gag (RQANFLGKI, GQMREPRGSDI), nef (RQDILDLWI), gag-

ɜs, plays a role in vasoconstriction of cerebral arteries.

ıment. In cardiac cells, it is required to repress expression of cyclin-D1 (CCND1) by activating n
978667, PubMed:15820677, PubMed:21653227, PubMed:22431633, PubMed:23169818, Pub

ofilin-2/CFL2 and destrin/DSTN, thereby preventing the cleavage of filamentous actin (F-actin),

rganization of the actin cytoskeleton, cell-cell adhesion and cell migration. MGAT5-dependent

ıl 1 and beta-type CGRP promoting vasoconstriction. Also cleaves KISS at a Gly-|-Leu bond. A

f the DISP complex, may regulate the association of septins with actin and thereby regulate the

d by various mechanisms of post-translational modification and subcellular compartmentalizati

nfluenza A virus strains PR8(H1N1) and ST364(H3N2). Sumoylated isoform PML-4 and isofor

nilarity). Dephosphorylates and activates transcription factor NFATC1 (PubMed:19154138). In
144). These primers are initially extended by the polymerase alpha catalytic subunit and subse
d gamma subunits (PRKAG1, PRKAG2 or PRKAG3).

omplex formation in glioma cells. In intestinal cells stimulated by the phorbol ester PMA, can tr

many physiological processes, including retinal angiogenesis and neuronal survival and matura
aposin-B forms a solubilizing complex with the substrates of the sphingolipid hydrolases.||Sapo
tling between ER and mitochondria (PubMed:21285369).

Med:16824732). The lipid phosphatase activity is critical for its tumor suppressor function. Ant

ell migration, axon branching and synapse formation; required for normal development of the n

hospholipids to the primary amine of phosphatidylethanolamine (PE), forming N-acylphosphati
10910365, PubMed:15485915, PubMed:16581787, PubMed:16818604, PubMed:17965729, Pu

imer with another RFX protein and binding to the X-box in the promoter of target genes (PubMe

on (EGF and PMA), phosphorylates and activates NR4A1/NUR77 and ETV1/ER81 transcriptio

omplexes with SLC7A6 and SLC7A7 mediate uptake of dibasic amino acids (PubMed:9829974

nitted to their adult state. The transition from proliferating neural stem/progenitor cells to postm

lated (PubMed:26479788). It then forms a homodimer termed IFN-gamma-activated factor (GA

sis through shedding of GP1BA, the platelet glycoprotein Ib alpha chain (By similarity). Mediate

ively active TGFBR2. Activated TGFBR1 phosphorylates SMAD2 which dissociates from the re
ulating cell adhesion and matrix remodeling (PubMed:20930113). With TJP2 and TJP3, partici

on with mitochondrial PPIF is involved in activating oxidative stress-induced necrosis; the functi

been shown to abrogate or completely inhibit YY1-mediated activation or repression. For exar

the ubiquitination and subsequent proteasomal degradation of ZFHX3 (PubMed:22452784).

t of the epididymis, where it affects the functional expression of clusterin and phospholipid hydr

ase activity (By similarity). Has broad substrate specificity and can catalyze the reduction of me
arresting cell growth in G1 phase in a p53-dependent manner. Acts as a positive regulator of c

es, for instance, cell cycle G1/S phase transition through its phospholipid phosphatase activity
associated cytosolic rRNAs (PubMed:30385512).

tes PARsylation of TERF1, thereby contributing to the regulation of telomere length (PubMed:1

disintegrin domain.

leukemia stem cells in bone marrow niche (By similarity). Plays a critical role in cancer progres

ng (HIV-1 and other lentiviruses). VPS4A/B are required for the exosomal release of SDCBP, C
y).

ced Ras and Erk1/2 signaling pathway that leads to sustained inhibition of long term melanoger

ecessary for the formation of secondary lymphoid organs: peripheral lymph nodes and Peyer's

unctions as a DNA-binding transcription factor (PubMed:12095676). Is a potent regulator of th

n of microRNA-10a and thereby in the inflammatory response (PubMed:28167758).

tes the downstream target RHOA that plays a role in the regulation of assembly of focal adhes

ay (PubMed:9394008). May play a direct role in a TNF-alpha signaling pathway leading to indu

ilarity).

ːs (By similarity). May act as an invasion suppressor in cancers.
ıistones during spermatogenesis. Also involved in DNA damage response in somatic cells, by p

o separate regions destined for lysosomal degradation from carriers to be recycled to the plasn

signaling and autophagy (PubMed:23604317). PCYT1A monoubiquitination by SCF(FBXL2) ar

〈1, leading to its degradation, thereby affecting cell proliferation and differentiation (PubMed:24

e D-box coreceptor, formed by FZR1 and ANAPC10; by suppressing ubiquitin ligation and chai

cell fate and regeneration by restricting the goblet cell lineage program; the function may involv

target YTHDC1 reader on Xist RNA and promoting transcription repression activity of Xist (Pub

bound to NF-kappa-B (PubMed:28152074). Interacts with the BRCA1-BARD1 heterodimer and

xylase 2 (EGLN1) (PubMed:15897452). Can enhance apoptosis induced by serum starvation i

prevents premature joining of the 40S and 60S ribosomal subunits prior to initiation (PubMed:
vation of alpha catalytic subunit (PRKAA1 or PRKAA2) both by inducing phosphorylation and p

nyl-CpG (PubMed:29276034).

ng the dissociation of the TCF7L2/LEF1/beta-catenin complex from DNA, as well as the ubiqui

development. Controls the genesis of hypaxial myogenic progenitors in the dermomyotome by
cycle may also contribute to the directionality of export. Binds strongly to m7G-capped U1 and U

assembly of core snRNPs and their transport to the nucleus.

lling the initial steps of chromosomal break repair, it is recruited to chromatin in a damage-depe

d:26735137, PubMed:27974568). Binds nucleic acids with a higher affinity for ssRNA and ssDN

questioning the classical function of the CSC. The SNX-BAR retromer mediates retrograde tra
:TMEM30A flippase complex seems to play a role in regulation of cell migration probably involv

in the nucleus: together with SPIRE1 and SPIRE2, promotes assembly of nuclear actin filamer

ral and peripheral clocks can be reset by environmental cues, also known as Zeitgebers (Germ
ciated with processes that include cell migration, cell adhesion, nutrient supply and cell signalin
ne fission events, such as the terminal stages of cytokinesis and the budding of enveloped viru

on (PubMed:16433634).

15009667). Plays a role in energy homeostasis by regulating satiety and metabolic rate (By sin

ells from ER stress-induced death (PubMed:17178827). In vitro activates transcription of targe

of its target genes (PubMed:25083013). Negatively regulates its own expression and the expre

on of trophoblast giant cells. Acts as a promoter of sprouting angiogenesis, possibly by acting a

Ca(2+) release-activated Ca(2+) (CRAC) channel regulation: sodium transport by SLC8B1 lead

beta-catenin CTNNB1 by the beta-catenin destruction complex, thus negatively regulating CTN

negatively regulate the activity of homotrimeric non-collagenous domain 1 (PubMed:11257123).

the activation of the prereplicative complex (pre-RC) during G(1) phase by recruiting CDC6 to th

feration.

ascular stability and pruning: acts by activating the non-canonical Wnt signaling pathway in end

_PK1, which phosphorylates TIFA at Thr-9, leading to TIFA homooligomerization and subseque

serve as a shedding protease for subsequent intramembrane proteolysis by SPPL2A and SPF

en activating Arp2/3 to branch off these filaments. The p53/TP53-cofactor and actin activator a

the transcription factor IRF3 to induce expression of type I interferon and exert a potent immun

t deaminate double-stranded DNA or single- or double-stranded RNA. Exhibits antiviral activity

rculosis.

hus to a reduced overall utilization of amino acids, while concomitantly initiating the preferentia

/ similarity).

(S)-stereospecific hydroxylation at C-3 of 'Lys-22' of DRG1 and 'Lys-21' of DRG2 translation fa
e pairing with the miRNA and most commonly results in translational inhibition or destabilizatio

of activin signals. Activin binds to type-2 receptor at the plasma membrane and activates its se

pic interactions with cells that express ALCAM (PubMed:15496415, PubMed:16352806). Requi

ranscriptional regulation of P21 in response to DNA damage. Required for FANCD2 targeting t
2 is involved in both POLH localization at collapsed replication forks and DNA polymerization a

al degradation. Slightly promotes SRC ubiquitination. May be involved in EGFR ubiquitination a

actin bundling activity when associated with BAIAP2, enhancing BAIAP2-dependent membrane

actor of the RAG complex: acts by stimulating cleavage and RAG protein binding at the 23 bp s

Acts as a ligand for integrin which is required for IGF2 signaling (PubMed:28873464).

uninfected cells and following viral infection, double-stranded RNA (dsRNA), or toll-like recept
xin (CTX) toxin to KCNMA1 peptide blocker by increasing the CTX association rate and decrea

and sodium channel clusters at mature nodes of Ranvier.

ıe cytoplasm, the RNA exosome complex is involved in general mRNA turnover and specifically

d is proposed to be recruited to the EJC prior to or during the splicing process and to regulate s

ɔgenesis (20S-PA200 complex) or generation of a subset of MHC class I-presented antigenic p

on repair substrates (PubMed:15314187, PubMed:15556996, PubMed:15871698). The 9-1-1 c

ı response to DNA damage. Also recruits to sites of DNA damage proteins like XPA and XPG t
ıl proteins, there are multiple processed pseudogenes of this gene dispersed through the geno

, chloride channels: BSND, CLCN2 and CFTR, glutamate transporters: SLC1A3/EAAT1, SLC1

Through the ubiquitin-mediated proteasomal degradation of hepatitis C virus non-structural pro
ıbMed:21123949). Plays a redundant role in the rapid removal of released glutamate from the
in placental development (PubMed:10708449, PubMed:23492904).
32). This chaperone activity is important to sustain normal SNARE-complex assembly during ag

19).

hormones such as insulin or glucagon-like peptide 1 (GLP-1) (By similarity).
ıll cycle and entering differentiation, repression by the PML-RARA fusion protein, BMP-induced

ized low-density lipoprotein (oxLDL) or amyloid-beta 42. In this context, the initial signal is provi

ıromosome separation and the processing of homologous recombination intermediates to limit

gnaling events to the mRNA decay machinery (PubMed:15687258, PubMed:23644599). Recrı

Plays a key role in histone variant H2AZ1/H2A.Z deposition into specific chromatin regions: re
ner blood-retinal barrier (By similarity). Can mediate the transport of thyroid hormones triiodothy

ed:10962286, PubMed:12909631, PubMed:15461590, PubMed:17379599). It is for instance es

l survival after DNA damage or replication stress by promoting DNA repair (PubMed:17141802

CDKN1A/WAF1 promoter to participate in transcription activation by p53/TP53. May also act as

n ligase complexes depends on the variable substrate recognition components. As a compone
MTA1-mediated epigenetic regulation of ESR1 expression in breast cancer (PubMed:24413532

own as the Lands cycle.
r of apoptosis BIRC6 and thus is an important regulator of cell death by promoting apoptosis. A

notes the centrosomal localization of CDK2 (PubMed:26297806). In non-mitotic cells, upon stre

freading activity than Pol-delta4 (PubMed:19074196, PubMed:20334433). Although both Pol-de
ion in multiciliated that can generate more than 100 centrioles. Also involved in trophoblast diffe

the scaffold subunit: complexes containing BRPF scaffold (BRPF1, BRD1/BRPF2 or BRPF3) c

ATP (de-AMPylation) from HSPA5/BiP at 'Thr-518', leading to restore HSPA5/BiP activity (By s

ne cytoplasm, the RNA exosome complex is involved in general mRNA turnover and specifically

onse to DNA damage; the function is probably independent of MLL-containing histone methyltr

attachment. During interkinetic nuclear migration (INM) at G2 phase and nuclear migration in n

ne cytoplasm, the RNA exosome complex is involved in general mRNA turnover and specifically
n inhibitor of cyclin A-associated kinase. Also acts a component of the CENPA-CAD (nucleosc

2008]

ctivation (PubMed:30017583). May also play a role in the modulation of arsenic-induced toxicity
e-specific role in T-cell development (PubMed:14532007).

nificant sequence similarity to bacterial S18 proteins. The primary sequences of the three huma

binding SOX2 promoter and positively regulating its transcription (By similarity).

(By similarity).

35865). May be involved in the control of centriolar-microtubule growth by acting as a regulator

conjugation to ATG3 playing a role in mitochondrial homeostasis but not in autophagy. ATG7 (

n estrogen receptor-regulated gene transcription and estrogen-stimulated G1/S cell-cycle trans

nization during spermatogenesis. Recruited by the large PER complex to the E-box elements c

assembly of core snRNPs and their transport to the nucleus.
assembly of core snRNPs and their transport to the nucleus.

ıcts. Interacts with the structure-specific ERCC4-ERCC1 endonuclease and promotes the cleav

91, PubMed:31626998, PubMed:31686031).||Substrate-recognition component of the DCX(DC

ɔtor, indirectly regulates the insulin-like growth factor receptor signaling pathway (PubMed:266[illegible]

ne ndc-80 complex and may allow the ndc-80 complex to track depolymerizing microtubules (P
nicrotubules (PubMed:23085020).

cal Wnt signaling (PubMed:21498478, PubMed:22654112). Acts as negative regulator of the hi

nits function in multiple RNA-processing events.

·). Efficiently cleaves both single and double HJs contained within large recombination intermed

najor histocompatibility complex (MHC) class II probably involving retromer and subsequently a

e pairing with the miRNA and most commonly results in translational inhibition or destabilizatio

e pairing with the miRNA and most commonly results in translational inhibition or destabilizatio
nd forms of Ran between the cytoplasm and nucleus.

lating cortical activity through RHOA during cytokinesis. May participate in the regulation of sul

adation of UBE2N and UBE2D3. Upon TNF stimulation, deubiquitinates 'Lys-63'-polyubiquitin c
icleus to activate gene transcription. For example, upon IL2R activation by IL2, JAK1 and JAK3

microtubules (PubMed:25743205, PubMed:23891108).

and balances the cytoplasmic-mitochondrial redox interface (PubMed:31978345). The purine r

ng late neurogenesis, regulates the cerebellar, cerebral cortex and olfactory bulb development
eads to recruitment of translesion (TLS) polymerases, while 'Lys-63'-linked polyubiquitination of

e pairing with the miRNA and most commonly results in translational inhibition or destabilizatio

osphorylated at 'Thr-3' (H3pT3) during mitosis (PubMed:20929775). The complex with RAN pl

bacterial peptidoglycan, in a NOD2-dependent manner (PubMed:18511561). Contrary to its mo

otor (PubMed:25898167). May play a role in cell proliferation (PubMed:15381421).

ctivity of BIRC4. Seems to be involved in cellular sensitivity to the proapoptotic actions of TRAIL

e pairing with the miRNA and most commonly results in translational inhibition or destabilizatio

1A, TLR2 and TLR4. Plays a role in the inflammatory response to bacterial lipopolysaccharide.
ignaling mediates activation of down-stream MAP kinases (By similarity). Contributes to the reg

romoting bladder cancer cells proliferation, apoptosis inhibition and carcinogenic progression (P

ce by reducing PRDX1 (PubMed:15105503). However, this could not be confirmed (By similari

ibits RHO. Phosphorylates multiple receptor tyrosine kinases like PDGFRB and other substrate

RNA cleavage complex II (CF-II), which seems to be required for mRNA 3'-end formation. Also

phagic degradation of KEAP1 (PubMed:23274085). May also mediate TP53 inhibition of TOR

n of the target mRNA. The RefSeq represents the predicted microRNA stem-loop. [provided by

e may promote folding of a substrate while another may promote degradation. The affinity for

n of the target mRNA. The RefSeq represents the predicted microRNA stem-loop. [provided by

sential to control replication licensing (PubMed:18794348, PubMed:19332548). KMT5A degrad

ie may promote folding of a substrate while another may promote degradation. The affinity for p

s (PubMed:27857118). In contrast to CPEB1 and CPEB3, does not play role in synaptic plastici

RAS, MAPK1/ERK2, MAPK3/ERK1 and the MAP kinase signaling pathway, as well as of the A
nction (PubMed:23776040).||May play a role in uric acid excretion.

n of the target mRNA. The RefSeq represents the predicted microRNA stem-loop. [provided by

nd PI3K pathways in cancer cells.

l:26565916). Stimulates Indian hedgehog (Ihh)-induced target gene expression mediated by th

Med:26878239, PubMed:20739937, PubMed:21478274). Required for recruitment and assemb

an active role at kinetochores, enhancing their links with dynamic microtubule ends (PubMed:2
yogenesis and in cell differentiation in embryogenesis. Involved in dendritic cell regulation of T-

uits LOXL2 to pericentromeric regions to oxidize histone H3 and repress transcription which le

n of the target mRNA. The RefSeq represents the predicted microRNA stem-loop. [provided by

osphorylates p53/TP53 in response to reactive oxygen species (ROS), thereby promoting p53/

n of the target mRNA. The RefSeq represents the predicted microRNA stem-loop. [provided by

s a transcription regulator that represses WT1-dependent transcription regulation, thereby tran
3 and RAD23A. Has a preference to bind DNA containing a short single-stranded segment but
anslocation of amyloid-beta peptide (ABPP) across the cell membrane from the extracellular to
nd phosphorylates keratin-8 (KRT8), which induces targeting of desmoplakin at desmosomes a

4). In response to TNF and in parallel to NF-kappa-B activation, negatively regulates RANTES

R binds to core DNA sequence 5'-TGCGTG-3' within the dioxin response element (DRE) of targ

Dephosphorylation of PRKCA and PRKCB leads to their destabilization and degradation. Deph

(PubMed:20682778). Acts as a ligand for integrins and binds (via EGF domain) to integrins IT

us half-site motif 5'-[A/G]GGTCA-3' preceded by an A/T-rich 5' sequence (RevRE), or as a hom

he CST complex is also proposed to be involved in fill-in synthesis of the telomeric C-strand pro

etween NSF and the GRIA2 subunit of AMPARs, leading to a rapid rundown of AMPAR-mediat

ponses in neonates (By similarity). Plays a role in early coronary blood vessels formation (By si

netochore-microtubule attachment and faithful mitotic progression (PubMed:19620631). As a c

isoform of CDKN2A (PubMed:24382891).

:t as a tumor suppressor and inhibits malignant cell transformation (PubMed:17998805).||[Isofo

gene (CYP7A) expression in liver. May also contribute to the regulation of pancreas-specific g

easomal degradation (PubMed:12821780, PubMed:15053880, PubMed:15195100, PubMed:15

cytes and T-lymphocytes through its receptors, CXCR4 and ACKR3, and decreases monocyte a

histone mRNA degradation, probably through terminal uridylation of mature histone mRNAs. M

n neurogenesis and in the development of cortical architecture (By similarity). Isoforms that lack

ceptors (PRRs). Protects cells from spontaneous formation of the ripoptosome, a large multi-pr

n of the target mRNA. The RefSeq represents the predicted microRNA stem-loop. [provided by
age responses as well as in signaling processes leading to activation of the transcription factor

assembly of bipolar spindles by phosphorylating KIZ, NEDD1 and NINL. NEDD1 phosphorylati
cission checkpoint signaling is terminated at late cytokinesis (PubMed:22422861, PubMed:248
els are low, translocates to the nucleus and promotes expression of SOX9, which induces chon

ock-controlled genes involved in lipid metabolism.
the transition of myoblasts from the proliferative phase to the differentiation phase (By similari

gatively regulate the apoptotic effects of IFIT2.

other cytokines, probably interleukins.

n of the target mRNA. The RefSeq represents the predicted microRNA stem-loop. [provided by

osis. Acts as a tumor suppressor by inducing cell death by an autophagy and caspase-depend

n maintaining centriole cohesion involved in controlling spindle pole integrity. Involved in centro

ıromatin organization or whether it is an indirect consequence of its function in maintaining mito

'Tyr-363' thereby promoting its recruitment to androgen-responsive enhancers (AREs). Phosph

binds neuron-restrictive silencer element (NRSE) and represses neuronal gene transcription in
ts (6-4 PP), apurinic sites and short mismatches (PubMed:10882109, PubMed:11278856, Pub

eceptor and interacts with SMAD4. The SMAD2-SMAD4 complex is subsequently translocated

phosphate. KIT signaling is modulated by protein phosphatases, and by rapid internalization an

the expression of C/EBP regulated genes (By similarity). Positively regulates the transcription c

:27694961). Ubiquitinates and regulates protein levels of NTRK1 once this one is activated by

e (PubMed:28471450). It may also play a role in the regulation of inflammation induced by high

of inflammatory gene expression (PubMed:12628924, PubMed:18511904). In skeletal myobla

n of the target mRNA. The RefSeq represents the predicted microRNA stem-loop. [provided by
acting DNA methylation and histone modifications. Has E3 ubiquitin-protein ligase activity by m

n of the target mRNA. The RefSeq represents the predicted microRNA stem-loop. [provided by

austed T-cell phenotype and the normalization of the anti-tumor response, providing a rationale
(PubMed:18703514).
n of the target mRNA. The RefSeq represents the predicted microRNA stem-loop. [provided by

ecruits PTPN6 resulting in dephosphorylation of CD247 and ZAP70 (PubMed:18424730). Also

cross-link repair (PubMed:26253028).

ed for the translocation of RGS14 from the cytoplasm to the plasma membrane. Required for n

n of the target mRNA. The RefSeq represents the predicted microRNA stem-loop. [provided by

poiesis even if the mechanisms are unknown (By similarity). In cancers such as thyroid or lung

t protein deacetylase that links transcriptional regulation directly to intracellular energetics and

n of the target mRNA. The RefSeq represents the predicted microRNA stem-loop. [provided by

9098711). May also regulate the ubiquitin-mediated proteasomal degradation of other proteins

BUB3. The BUB1-BUB3 complex plays a role in the inhibition of APC/C when spindle-assembl

on of membraneless organelles at the onset of mitosis is also required to release mitotic regula

S1/2, PIAS1/GBP, EIF2AK2/PKR and RSAD2/viperin; antibacterial response, such as NOS2/IN

function in multiple RNA-processing events. Isoform 2 is responsible for processing a yet unkr

ll development and activation (By similarity). Plays a role in phagocytosis of apoptotic cells by r

rial membrane, facing the cytosol (PubMed:21397848). Has been shown to be a backbone-nor

GSK-3 kinase. Both pathways seem to involve interactions with G-proteins. May be involved ir

ues. Coreceptor along with RYK of Wnt proteins, such as WNT1.

n of the target mRNA. The RefSeq represents the predicted microRNA stem-loop. [provided by

PubMed:25126726). In addition to its role in mTORC1 signaling, also acts as a co-chaperone c

ıilarity). Activates AURKB and AURKC (PubMed:27332895). Required for localization of CBX5

nal mitosis.

ıydrolyzes the non-canonical purine nucleotides inosine diphosphate (IDP) and deoxyinosine d

744926, PubMed:22108654, PubMed:23274426). Can also phosphorylate diacylglycerol substr
olymerase activity. Required for normal axon formation. Plays a role in microtubule remodeling

ıa into a sliding clamp capable of hydrolysis-independent diffusion along the DNA backbone. T
istry), followed by insertion of molecular O2 to form the endoperoxide bridge between carbon 9

e 2 ends of resected double-strand breaks, allowing microhomologous sequences in the overha

(PubMed:10200294, PubMed:15793005).

n of the target mRNA. The RefSeq represents the predicted microRNA stem-loop. [provided by

n of pro- and anti-inflammatory cytokines. Also functions as a negative regulator of innate immu

age response: up-regulated by p53/TP53 following genotoxic stress and acts as a downstream
n of the target mRNA. The RefSeq represents the predicted microRNA stem-loop. [provided by
endent of its fructose-bisphosphatase activity (PubMed:23185017). In response to cardiac dam

cytotoxic T cell differentiation.

of NEK2 to centrosomes, and its ability to phosphorylate CROCC and CEP250. In conjunction

n between NTRK2/TRKB and KLC1 and drives NTRK2/TRKB axonal but not dendritic anterogr

s as a monomer to a response element composed of the consensus half-site motif 5'-[A/G]GG

e first meiotic division through phosphorylation of HMGA2. Phosphorylates: PPP1CC; SGO1; N

n of the target mRNA. The RefSeq represents the predicted microRNA stem-loop. [provided by

ic adaptations to cold temperatures (By similarity).

vated in response to selective autophagy: autophagy promotes interaction between KEAP1 and

way (PubMed:28712728).

processes and development of tissue damage after ischemia. Regulates biosynthesis of luteiniz

c scaling, thereby contributing to neuronal homeostasis. Acts as a postsynaptic mediator of act

and FAK. Can evoke collapse of embryonic neuronal growth cone and regulates dendritic spin

CM2, MCM4, MKLP1, MYB, NEFH, NFIC, NPC/nuclear pore complex, PITPNM1/NIR2, NPM1,

ential physiological compound (PubMed:9607309). Has both an extracellular and an intracellul

ıctivation, but not repression activity.||[Isoform Alpha-C1]: Has transcriptional activation activity.

;ponses to cellular stress. Forms multisubunit signaling complexes with SRC and SRC family r

c recruitment of 53BP1/TP53BP1, UIMC1/RAP80, and BRCA1 to DNA damage sites (PubMed

ıent of ATP and co-chaperone followed by client protein forms a functional chaperone. After the

:ruiting SNARE complex component SNAP29. Is required for recruitment of transition zone pro

; (PubMed:11376341, PubMed:14734526). Forms a Schiff base with 5'-deoxyribose phosphate

ınctions (PubMed:10754304). Interacts with CD160 on NK cells, enhancing IFNG production an

(PubMed:26643732). Also involved in the induction of adipogenesis from mesenchymal stem
ates T-cell development in the thymus.

minouracil (5-OE-RU) and 5-(2-oxopropylideneamino)-6-D-ribitylaminouracil (5-OP-RU), produc

to translation inhibition (PubMed:15479637, PubMed:23071094). Also functions as an effector

titis B virus (HBV), herpes simplex virus 1 (HHV-1) and Epstein-Barr virus (EBV) and may inhib

sociation with promoters of TCF-responsive genes. Seems to repress transcription from E2F1

as dengue virus (DENV), west Nile virus (WNV), and reovirus. Also involved in antiviral signalir

ytic subunit p110 (PIK3CA/PIK3CB/PIK3CD) and reducing PIK3R1/PIK3R2 activation. Via its re

ed for normal water homeostasis and to prevent excessive water loss through the skin, probabl

ulate t-loop formation (PubMed:10888888).

MD1- and actin-dependent manner (PubMed:10564580, PubMed:28604741). May play a role i

ha and IFN-beta interferons (PubMed:28469175, PubMed:31006531). Ligands include 5'-triph

lic GMP-AMP (cGAMP), a messenger produced by CGAS in response to DNA virus in the cyto

uncoupled from G-proteins. Two different modes of arrestin-mediated internalization occur. Cl

ıbMed:27796300). In macrophages, positively regulates MRC1 expression in response to IL4 s

mokines.
ically targets the multiply spliced but not unspliced or singly spliced HIV-1 mRNAs for degradat

at contains Rab8 and hungtingtin (HD). Mediates the interaction of Rab8 with the probable GTI

r proliferation, signaling and cytokine production of naive, effector and memory T-cells. Requir

230272). By monoubiquitinating histone H2B H2BC9/H2BJ and thereby promoting chromatin re

s. Acts as a transcriptional corepressor for SNAI1 and SNAI2/SLUG-dependent repression of E

functions as a component of numerous distinct DCX (DDB1-CUL4-X-box) E3 ubiquitin-protein

Plays also an important role in inhibition of Toll-like receptors (TLRs)-mediated innate immune r

r AXL are CBL, NCK2, SOCS1 and TNS2. Recruitment of GRB2 and phosphatidylinositol 3 ki
exes are held in the cytoplasm in an inactive state complexed with members of the NF-kappa-B

ne hypothalamic KiSS1/GPR54 system is a pivotal factor in central regulation of the gonadotro

ment. Required for recycling of the TGF-beta receptor. Involved in CFTR trafficking to the late

d in nuclear remodeling during sperm head formation in spermatogenenis; a probable SUN3:S
nereby helps to establish a tonotopic map. Kinetics of KCNMA1 channels are determined by alt

logous expression, inhibits the adhesion of endothelial cell to the extracellular matrix (ECM), ar

ses such as apoptosis, cell growth, differentiation and gene regulation (PubMed:21142819). Ma
yogenesis; potentiates transcriptional activation of NODAL-mediated gene transcription in the I

d for proper BBSome complex assembly and its ciliary localization.

g the activity of genes essential for dendrite growth (By similarity). Muscle-specific component c

e mitochondrial matrix.
with INSM1 on the NEUROD1 and INS promoters in a cell cycle-independent manner.

nilarity). Interacts with the NF-kappa-B transactivator p65 RELA and inhibits its phosphorylation
ator, CYC1, from the mitochondrial membrane. Also acts as a regulator of G2 checkpoint and p

n inhibit viral replication. May regulate matrix nuclear localization, both nuclear import of PIC (F

and the electron transfer chain comprised of ferredoxin reductase and ferredoxin, which receiv

, integrin alpha-IIb/beta-3 signaling in platelets and JNK signaling in B-cells. Regulates prolifera
erminate autophagy and promote apoptosis (PubMed:26263979). Might play a central role in th

aling (PubMed:23545260).

ıg a role beyond the maintenance of the latency of the PDGF domain. In the nucleus, PDGFC s

on from the 14-3-3 protein YWHAQ which acts as a negative regulator.
mitter release at synapses formed between cultured sympathetic ganglion cells. Critical particip

ory genes, thereby leading to an innate immune response in the infected cell. Restricts infectior

Med:14672706). Acts as a corepressor of RXR and the RXR-LXR heterodimers and represses

ıbMed:10587585). Required for cardiac homeostasis (PubMed:10580070, PubMed:12927431,

after radiation injury (By similarity). Modulates the cellular apoptosis program by regulating CO

ıent 4 (AS4) involved in neurexin selective targeting to postsynaptic partners such as neuroligin

itis B virus (HBV) and human T-cell leukemia virus type 1 (HTLV-1) and may inhibit the mobility

oy human Epstein-Barr virus vIL-10 protein, thereby affecting the transport of peptides into the

initiation factor 4E (eiF4E). mTORC1 phosphorylates and activates S6K1 at 'Thr-389', which tl

ɔpolysaccharide (LPS) et mediates LPS-induced inflammatory response, including TNF secreti

pinal projections. May also control the segregation of motor and sensory axons during neurom

ucleus to effect transcription of specific target genes. Plays a critical role in bone formation thro

ne mitogen-activated protein kinase (MAPK) kinase and the nuclear factor NF-kappa-B (NF-kB)

nd enzymatic activation (PubMed:30692621). When secreted, promotes the proliferation of bet

nain, which is linked to the VCP-mediated ER-associated degradation (ERAD) pathway. Promot

ability of CDKN1A promoting DNA damage-induced cell cycle arrest. Also plays a role in mega

tosis, and DNA repair (PubMed:14966270). The NuA4 complex ATPase and helicase activities
as an autoantigen in the immunopathogenesis of psoriasis, with certain peptide regions being a

m high sensitivity alpha-4(2):beta-2(3) to low sensitivity alpha-4(3):beta-2(2) nAChR (By similar

gh peroxisomal beta-oxidation (PubMed:15060085).

ınd oxidation of lysine residues by LOXL3, leads to disrupt STAT3 dimerization and inhibit its tr
proteins mostly dissociate from the invaginating membrane before the ILV is released (PubMe

oility, in long-term in vitro culture and cancers, by abbreviating mitotic phase and impairing the

n of the target mRNA. The RefSeq represents the predicted microRNA stem-loop. [provided by

najor developmental effects in somatosensory neurons and in brainstem nuclei involved in mo

n of the target mRNA. The RefSeq represents the predicted microRNA stem-loop. [provided by

s as a dimer and is much less active. Both double-stranded and single-stranded regions of a p

the transformed phenotype of cancer cells (PubMed:22722849). Also able to mediate deacetyl

BAF and nBAF complexes. As neural progenitors exit mitosis and differentiate into neurons, n

ed:25820262). Regulates the DNA repair pathway choice by inhibiting non-homologous end joi

nd increased competition with RAF1 for binding to GTP-bound form of Ras proteins (NRAS, HI

conditions of oxidative stress in the presence of RAD51 and RAD51C.
nulation of HIF1A (PubMed:16819549). In vitro able to promote polyubiquitination using all 7 ul

nan for 'timegivers'). The predominant Zeitgeber for the central clock is light, which is sensed b

GABA transporter (SLC6A12) and EGFR/ERBB1, ERBB2, ERBB3 and ERBB4 to the basolate

ɔn, such as those encoding cytokines (IL6 and IL2), cell surface receptors (ICOS, TNFRSF4 an

normal progress through mitosis.
n of the target mRNA. The RefSeq represents the predicted microRNA stem-loop. [provided by

ɔnine residues and this phosphorylation attenuates the ability of PAGE4 to potentiate the transc
n of the target mRNA. The RefSeq represents the predicted microRNA stem-loop. [provided by

king two epigenetic repression systems. Genes repressed by the PRC2/EED-EZH2 complex inc

n of the target mRNA. The RefSeq represents the predicted microRNA stem-loop. [provided by

antigen by T cell antigen receptor (TCR) (By similarity).
nd proficient non-homologous end-joining (NHEJ)-directed DNA repair by catalyzing the di- anc

ͤ T-cell CD8SP from thymocytes. Required for the switching of fetal globin species, and beta- a

ıctive site in subunit 1, a binuclear center (BNC) formed by heme A3 and copper B (CU(B)). Th

nan for 'timegivers'). The predominant Zeitgeber for the central clock is light, which is sensed b

tion. Phosphorylates DLG1. Following osmotic shock, MAPK12 in the cell nucleus increases its

nan for 'timegivers'). The predominant Zeitgeber for the central clock is light, which is sensed b

ımbination (PubMed:21247901). May promote DNA mismatch repair (PubMed:26221039).

ng midbrain. May be involved in regulation of myeloid differentiation. Can bind to the DNA sequ

n of the target mRNA. The RefSeq represents the predicted microRNA stem-loop. [provided by

nvolved in Arp2/3 complex-dependent actin assembly driving Salmonella typhimurium invasion

75644). Calcium is required for high affinity binding to GABA (By similarity). Plays a critical role

n of the target mRNA. The RefSeq represents the predicted microRNA stem-loop. [provided by

noid biosynthesis via the cyclooxygenase pathway (PubMed:18451993, PubMed:7794891, Pub

al nervous system vascular morphogenesis (By similarity). Acts as a transporter for tunicamycin

man for 'timegivers'). The predominant Zeitgeber for the central clock is light, which is sensed b

annel regulator. Involved in adhesive protein-protein and protein-carbohydrate interactions.

also been identified as a potential tumor suppressor, with its down-regulation associated with [illegible]

is capable of displacing duplex regions up to 100 bp, which can be extended up to 500 bp by

ponse activation through the cGAS-STING pathway (PubMed:28712728).

n of the target mRNA. The RefSeq represents the predicted microRNA stem-loop. [provided by

regulates initiation of autophagy by regulating the RAB1A-dependent trafficking of the ATG1/U
ntial for the self-renewal/proliferative capacity of the multipotent neural stem cells. The nBAF co

n of the target mRNA. The RefSeq represents the predicted microRNA stem-loop. [provided by

n of the target mRNA. The RefSeq represents the predicted microRNA stem-loop. [provided by

n of the target mRNA. The RefSeq represents the predicted microRNA stem-loop. [provided by

PubMed:20061386).

ypes of cancer. This gene family contains numerous pseudogenes. [provided by RefSeq, May 2

m estrone (E1) and 17beta-estradiol (E2), namely 2-hydroxy E1 and E2, as well as D-ring hydr

n of the target mRNA. The RefSeq represents the predicted microRNA stem-loop. [provided by

n of the target mRNA. The RefSeq represents the predicted microRNA stem-loop. [provided by

n of the target mRNA. The RefSeq represents the predicted microRNA stem-loop. [provided by

n of the target mRNA. The RefSeq represents the predicted microRNA stem-loop. [provided by
n of the target mRNA. The RefSeq represents the predicted microRNA stem-loop. [provided by

ent induction of NR2B expression involves a release of HDAC1 and recruitment of CREBBP (B

omotes neuronal differentiation (By similarity).
ctron transport chain resulting in control of mitochondrial ROS production (PubMed:20453889,

arity). Promotes colon cancer cell migration and invasion in an integrin alpha-6/beta-1-depende

active site in subunit 1, a binuclear center (BNC) formed by heme A3 and copper B (CU(B)). Th

particular antigen. The variable domains are assembled by a process called V-(D)-J rearranger

tion between pancreatic islet cells to regulate glucose-stimulated insulin secretion (By similarity

ıodeling high-density lipoprotein particles into their spherical forms (PubMed:10722751). Cataly

phosphorylation of the CTD domain of RNA pol II (By similarity). In addition, enhances MYOD1
·5' double stranded DNA exonuclease activity. Its phosphorylation by PRKCD may be required

ction and homeostasis of both CD8(+) and CD4(+) T-cells (PubMed:7805750, PubMed:86471[illegible]

sorption and bicarbonate secretion by the small intestinal brush border membrane and contribu

EFNB2-expressing cells. May play a role in constraining the orientation of longitudinally projecti

n of the target mRNA. The RefSeq represents the predicted microRNA stem-loop. [provided by

ity).

sminogen activation (PubMed:15016824). Plays a role in male fertility (PubMed:21919946, Pub
go receptor to selectively sort the membrane proteins involved in receptor-mediated endocytos
C6A19 and regulating its activity (By similarity).

e and inflammation and implicates NOS3/eNOS dysfunction (PubMed:22928666). Can inhibit e

g secreting cells (PubMed:12626569). Associates with the transcriptional repressor ZNF683 to
differentiation. Also involved in interleukin-6 (IL6) induced differentiation. Plays also a role in p
ease (PubMed:27076518). In response to DNA damage, mediates intrinsic apoptotic process i
sity lipoprotein (LDL) receptor (LDLR) mRNA in response to phorbol 12-myristate 13-acetate (

s leading to its subsequent degradation (PubMed:25893292).

e C that coordinate signaling pathways promoting calcium mobilization and actin-mediated cytc

led:20332120).

issues repair.
ation, cell migration, and angiogenesis (PubMed:18222923, PubMed:20194890). Required for r
skeleton. Isoform 2 can phosphorylate myosin, PPP1R12A and MYL12B. Overexpression lead
omoter region via association with RELA. Binds to poly(A) RNA. May be involved in 3'-end cleav

rse set of cell protrusions, such as filopodia and lamellipodia, necessary for platelet cell spread
es the ARHGEF15-mediated negative regulation on excitatory synapse formation. Controls oth

ving proteolytic cleavage by PRTN3 (PubMed:23202369). Proposed to have a bronchoprotecti
'6466, PubMed:16223769, PubMed:16651416, PubMed:18095711). Also has dipeptidyl peptid

isoform 2 seems to be less efficient in signal transduction, because it has a truncated C-termi
age. Participates in signal transduction pathways that regulate the integrity of the glomerular sl

ion, or regulation of thrombotic responses.

atory cytokine production (PubMed:23762476, PubMed:16787920, PubMed:16705036, PubM

ated transcription. Phosphorylation of TP53/p53, a crucial tumor suppressor, inhibits TP53/p53
cts as a regulator of B-cell development by inhibiting UBE2N/Ubc13, thereby restricting the acti

1 and FOXA2 seem to have at least in part redundant roles (By similarity). Modulates the trans

ıction enhances binding of SMAD3 to the FOXP3 promoter, leading to up-regulation of FOXP3

s required for embryonic stem cell pluripotency including POU5F1/OCT4, NANOG, KLF4 and S

otransferrin is a major iron-binding and multifunctional protein found in exocrine fluids such as b

ıhogenetic effects while PI3K/AKT coordinates prosurvival effects. During embryonic developm

so recognizes and binds histone H3 acetylated and butyrylated at 'Lys-9' (H3K9ac and H3K9bu

ıan for 'timegivers'). The predominant Zeitgeber for the central clock is light, which is sensed by

s (EMCV) and stimulates the alternative antiviral pathway independent of RNase L.

Med:12699694). Similarly, catalyzes the two-step conversion of an N-fatty acylglycine to a prim

s macrophage-mediated phagocytosis of apoptotic leukocytes by tethering them to the phagoc
n polarized epithelial cells; the function seems to be independent from its flippase activity. Req

equently transported to the liver for degradation and excretion (PubMed:21736953). Two distinc

. In cancer cells, substitution of isoform 1 (E2) by isoform 2 (E1) results in immunoproteasome

By similarity). Down-regulates TLR9-mediated activation of NF-kappa-B, as well as production c

ly (PubMed:18756269).

ess to insulin. May also mediate transport from the endoplasmic reticulum to the Golgi.

equired for optimal migration of thymic natural regulatory T cells (nTregs) and DN1 early thymo

C to the receptor complexes where it phosphorylates the tyrosine residues within the receptor c

er development and functioning of the nervous system. As an example of their role in signal tra

ıilarity). Triggers the formation of an extrusion apical actin ring required for epithelial extrusion c

ɔn and apoptosis, and uses different modes of RNA stem-loop binding to exert either translatio

ation. Adapter that mediates the interaction between TRAF6 and CYLD (By similarity). May be

ɔptosis. Binding to MEFV allows MEFV to bind to PYCARD and facilitates pyroptosome formati

ɔably in specific endosome subpopulations (PubMed:25266290). Functions predominantly in A

on neighboring nucleosomes (PubMed:22009739). Acts as a corepressor for REST by facilitat

ı that coordinates with SH3RF1 in organizing different components of the JNK pathway, includi

signaled by CD40, TNFR1 and TNFR2.

1 signaling and activation of the small GTPase RAP1 (PubMed:12432078). Promotes cell mig

idized low-density lipoprotein (oxLDL) or amyloid-beta 42. In this context, the initial signal is pro

(FRCs) adhesion to the extracellular matrix (ECM) and contraction of the actomyosin by mainta
ding to (PubMed:22253824). RNA-binding protein that plays a role in the regulation of alternati

RNA degradation machinery as it has endonuclease activity required to initiate NMD, and to se

rity).

ed:24336198, PubMed:28229507, PubMed:26294762, PubMed:26431200). Likewise, suppres
ate, chondroitin sulfate and dermatan sulfate (PubMed:16982628).

(PtdIns3P) forming on membranes of the endoplasmic reticulum upon activation of the upstre

une response and in the regulation of T-cell differentiation and proliferation. Negatively regulate

d secretion of proinflammatory cytokines. The function as activating adapter in different types o

proteasomal degradation (PubMed:22522597). Originally identified as a tumor suppressor, it m

al. Isoform 5 and isoform 6 display reduced activity.

red for normal neuron migration during embryonic brain development. Plays a role in neuronal
nfluenced processes such as angiogenesis in retinal and cardiac functionality. Target proteins

uscle cells, is also involved in immune system regulation (Probable). Modulator of vascular rem

In case of infection by Herpesvirus saimiri, may play a role in vesicular transport or membrane

ced epithelial-mesenchymal transition and myofibroblast differentiation (PubMed:30696809).

ed in the regulation of the hedgehog/smoothened (Hh) signaling pathway by mediating the ubic

ed:25527291). Required for ubiquinone reduction by mitochondrial complex I by mediating pho

inhibit SLC4A4 as well as CFTR activities and surface expression, recruits STK39 which media

1.

7). It is unclear how it can both act as a microtubule organizer and as a transcription factor; adc

ıates to MLKL mediates the release of an N-terminal auto-inhibitory region, leading to activatior

)89856). Also hydrolyzes dihydroceramides to produce dihydrosphingosine (PubMed:2020793

in myotubes induces the formation of NMJ by regulating different processes including the trans

:eptor tyrosine kinases and more particularly promotes endocytosis of EGFR, facilitates the forr

vhich has a low Km for acetaldehydes, and is localized in mitochondrial matrix. Alternative splic

F CASP4. Associates with chromatin in a region surrounding the CASP4 transcriptional start site

or and performs physiological functions on the surface of neurons relevant to neurite growth, ne

thylation states of H3K9 and H3K4) and association with CBX5. Involved in maintaining telome

es in many regions of the CNS. The versatility of BDNF is emphasized by its contribution to a ra

imulates adjacent fibroblasts to produce matrix metalloproteinases (MMPS). Seems to be a rec
RSV) and murine leukemia virus (MLV), flavivirideae: hepatitis C virus (HCV), filoviridae: ebola v

B-dependent transcriptional activation (By similarity). Involved in lineage commitment of immat

cluding loss of stress fibers, and enhanced motility during cell differentiation. Prevents myeloid
TOPOI and stimulates its activity. This complex binds to rRNA gene promoters and may play a

'92). Protects cells against apoptosis and against cytolysis by complement (PubMed:2780565)

aments. May play a crucial role in cell differentiation.

ınd play a key role in Okazaki fragments processing and DSB repair, the 5'-3' DNA helicase act

cal processes by cleaving peptides in the circulation, including many chemokines, mitogenic gr

its transcriptional activity. Acts as transcriptional repressor of IL-17 expression in Th-17 differer

ıthway. Promotes phosphorylation of SHC1, STAT1 and PTPN11/SHP2. In the nucleus, enhan

ınt of free VEGFA and preventing its binding to KDR. Likewise, isoforms lacking a transmembra

vhen expressed in endothelial cells, it sustains the abluminal to luminal transcytosis of tissue-d

lial cells to promote division, migration and the formation of capillary-like tubule structures (By s

h APC, AXIN1 and CTNNB1/beta-catenin and phosphorylates the N-terminus of CTNNB1 lead

(PubMed:8135828, PubMed:29915090, PubMed:30850536). Independently of the subunit beta
;, PubMed:29915090, PubMed:30850536).
progates the effect of KAT5-mediated relieving of NR1D2 transcription repression activity. Com

AD and potentiates activation by NCOA1 and CREBBP. Involved in the axonal distribution and
nic peptides from the EBV latent antigens (PubMed:18779413).||Allele A*02:01: A major allele
pol (RQYDQILIE, GQGQWTYQI) and rev (LQLPPLERL), all having in common a Gln residue a

nethylation of 'Lys-9' of histone H3 (H3K9me) by the GLP1/EHMT1 and G9a/EHMT2 histone m
Med:25001086). Channel activity is modulated by activation of serotonin receptors (By similari

and stabilizing the actin cytoskeleton (PubMed:11832213, PubMed:15660133, PubMed:1623

EGFR N-glycosylation enhances the interaction between EGFR and LGALS3 and thereby prev

ppears to have a role in myocardial cell death pathways. Contributes to myocardial oxidative st

actin cytoskeleton (PubMed:29467281). May play a role in transporting DAB2 from the plasm

on as well as by interactions with other cofactors or corepressors. NF-kappa-B complexes are

n PML-12 show antiviral activity against encephalomyocarditis virus (EMCV) by promoting nucl

response to increased Ca(2+) levels, regulates NFAT-mediated transcription probably by deph
quently transferred to polymerase delta and polymerase epsilon for processive synthesis on th

igger a cell cycle arrest program which is associated with the accumulation of the hyper-phosp

ation during development. Intracellularly, degrades TSC2, leading to the activation of TSC2 dov
sin-D is a specific sphingomyelin phosphodiesterase activator (EC 3.1.4.12).||Saposins are spe

agonizes the PI3K-AKT/PKB signaling pathway by dephosphorylating phosphoinositides and th

ervous system. Plays a role in osteogenesis and differentiation of osteoblasts. Functions in inte

dylethanolamine (NAPE), which serves as precursor for N-acylethanolamines (NAEs) (PubMec
ubMed:19202191, PubMed:19759395, PubMed:20064462, PubMed:20829486). During immur

ed:20148032). Represses transcription of MAP1A in non-neuronal cells but not in neuronal cell

n factors and the cofactor CREBBP (PubMed:16223362). Upon insulin-derived signal, acts ind

-, PubMed:10903140). The complexes function as amino acid exchangers (PubMed:11557028

itotic neurons requires a switch in subunit composition of the npBAF and nBAF complexes. As

AF), migrates into the nucleus and binds to the IFN gamma activated sequence (GAS) to drive

es the proteolytic cleavage of LAG3, leading to release the secreted form of LAG3 (By similarity

eceptor and interacts with SMAD4. The SMAD2-SMAD4 complex is subsequently translocated
ipates in the junctional retention and stability of the transcription factor DBPA, but is not involve

ion is largely independent of transcription. Induces the transcription of long intergenic non-codi

nple, it acts as a repressor in absence of adenovirus E1A protein but as an activator in its pres

roperoxide glutathione peroxidase (PHGPx), two proteins required for sperm maturation. May a

ethylglyoxal, 9,10-phenanthrenequinone, prostaglandin J2, 4-nitrobenzaldehyde, 4-nitroacetoph
osteoblast differentiation.

(By similarity).

1739745). Involved in centrosome maturation during prometaphase by mediating PARsylation

ssion by inhibiting VEGFA production threreby inhibiting angiogenesis through a signaling pathw

CD63 and syndecan (PubMed:22660413).

hesis by reducing dendrite extension and melanin synthesis. Provides also inhibitory signals for

patches. Acts as a developmental checkpoint and regulates thymocyte positive selection towa

e RB1 pathway through induction of RB1 expression (PubMed:14533007). Plays a crucial role

ions and actin stress fibers. Stimulates cell survival by phosphorylating the BCL2 antagonist of

uction of cell death (PubMed:8995684).

ɔromoting degradation of histones following DNA double-strand breaks.

na membrane (PubMed:30220460). Required for normal cell proliferation, cell migration, and n

nd subsequent degradation regulates synthesis of phosphatidylcholine, which is utilized for forn

·362026). Associated component of the 3M complex, suggesting that it mediates some of 3M c

n elongation by APC by preventing the UBE2C and UBE2S activities (PubMed:23708605, Pub

'e regulation of AGR2. Essential transcriptional regulator of B-cell development. Involved in reg

Med:27602518). Functions in the regulation of alternative or illicit splicing, possibly by regulatir

d regulates its activity. Specifically binds 'Lys-6'-linked polyubiquitin chains. Interaction with autc

n mammary epithelial cell line HC11 (By similarity).

17581632). The eIF-3 complex specifically targets and initiates translation of a subset of mRNA
preventing dephosphorylation of catalytic subunits. ADP also stimulates phosphorylation, withou

tination and subsequent proteolysis of LEF1. Together these effects inhibit the transcriptional a

/ transactivating PAX3 and the delamination and migration of the hypaxial precursors from the
J5 small nuclear RNAs (snRNAs) in a sequence-unspecific manner and phosphorylation-indep

endent manner and functionally interacts with the MRN complex to accelerate resection throug

NA than for dsDNA (PubMed:27974568).||Isoform 4 does not inhibit programmed ribosomal fra

nsport of cargo proteins from endosomes to the trans-Golgi network (TGN) and is involved in e
ving flippase-mediated translocation of phosphatidylethanolamine (PE) at the plasma membrar

nts in response to DNA damage in order to facilitate movement of chromatin and repair factors

nan for 'timegivers'). The predominant Zeitgeber for the central clock is light, which is sensed by
g (PubMed:28892079). Involved in copper-dependent ATP7A trafficking between the trans-Go
uses (HIV-1 and other lentiviruses). ESCRT-III proteins are believed to mediate the necessary v

nilarity). Promotes adipogenesis by activating JNK1 and inhibiting the p38MAPK pathway, and

t genes via direct binding to the CRE site (PubMed:17178827).

ssion of DBP and BHLHE41/DEC2. Acts as a corepressor of RXR and the RXR-LXR heterodin

is a transcription activator: associates with HIF1A, recognizes and binds the VEGFA promoter,

is to promote calcium-shuttling that modulates mitochondrial redox status, thereby regulating S

NB1 degradation (PubMed:27098453). Serves as a scaffold to coordinate PI3K/AKT pathway-

.||Probably plays a major role in determining the retinal structure as well as in the closure of the

he origin recognition complex (ORC) (PubMed:15684404). Probably by regulating HR, plays a

dothelial cells (By similarity) (PubMed:21727895, PubMed:21909076, PubMed:22615920). Car

ent activation of proinflammatory NF-kappa-B signaling (PubMed:30111836).

PL2B of the envelope glycoprotein gp130 (PubMed:23132852). Plays a role in the regulation of

ctivities are regulated via its subcellular location (By similarity).

ity against invading pathogens (PubMed:25636800). Component of a multi-helicase-TICAM1 c

also against hepatitis B virus (HBV), equine infectious anemia virus (EIAV), xenotropic MuLV-r

l translation of ISR-specific mRNAs, such as the transcriptional activator ATF4, and hence allo[illegible]

ıctors (TRAFAC), promoting their interaction with ribonucleic acids (RNA) (PubMed:29915238).
n of the target mRNA. The RefSeq represents the predicted microRNA stem-loop. [provided by

rine-threonine kinase. The activated receptor type-2 then phosphorylates and activates the typ[illegible]

ired for normal hematopoietic stem cell engraftment in the bone marrow (PubMed:24740813). [illegible]

ɔ sites of DNA damage. May function as a transcriptional regulator. Inhibits lipid synthesis by b
ıctivity. In concert with NPM1, regulates centrosome duplication. Interacts with the TREX-2 con

ınd internalization. May be functionally coupled with the E2 ubiquitin-protein ligase UB2D3. In a

ǝ extensions and promoting filopodial protrusions. Involved in the regulation of processes such

spacer of conserved recombination signal sequences (RSS) (By similarity). Proposed to be invo

ɔr (TLR) signaling, is phosphorylated by IKBKE and TBK1 kinases (PubMed:22394562, PubMe
sing the dissociation rate.

/ degrades inherently unstable mRNAs containing AU-rich elements (AREs) within their 3' untra
specific excision of introns in specific transcription subsets. Involved in the establishment and m

›eptides (20S-PA28 complex).
complex is necessary for the recruitment of RHNO1 to sites of double-stranded breaks (DSB) c

that are involved in nucleotide excision repair and is required for this mechanism of DNA repair
ome. Alternatively spliced transcript variants encoding different isoforms have been found for th

A2 /EAAT2, SLC1A1/EAAT3, SLC1A6/EAAT4 and SLC1A7/EAAT5, amino acid transporters: S

tein 5A, has an antiviral activity towards that virus.
synaptic cleft, which is essential for terminating the postsynaptic action of glutamate (By simila

ging (PubMed:20798282). Plays also a role in the regulation of the dopamine neurotransmissio

repression, repression of switch recombination to IgA and regulation of telomere length. Comp

ided by oxLDL- or amyloid-beta 42-binding to CD36. This event induces the formation of a hete

DNA crossover formation in cells. Has DNA decatenation activity (PubMed:30057030). It is rec

uits deadenylase CNOT7 (and probably the CCR4-NOT complex) via association with CNOT1,

cognizes and binds H3K14ac and H3K27ac on the promoters of actively transcribed genes and
yronine (T3) and thyroxine (T4) across the cell membrane (PubMed:11564694, PubMed:12225

ssential for the extracellular hydrolysis of S1P and subsequent conversion into intracellular S1P

2, PubMed:17296725, PubMed:26344098, PubMed:30356214). In response to double-strand b

s a tumor malignancy-associated factor by promoting tumor invasion and metastasis under reg

nt of the CSA complex promotes the ubiquitination of ERCC6 resulting in proteasomal degrada
2). In addition to its protein deacetylase activity, plays a key role in the degradation of misfolded

\cts also as a PRKN modifier that accelerates its degradation, resulting in a reduction of PRKN

ss induction, inhibits mammalian target of rapamycin complex 1 (mTORC1) association and re

elta3 and Pol-delta4 process Okazaki fragments in vitro, Pol-delta3 may also be better suited t
erentiation by phosphorylating HAND1, leading to disrupt the interaction between HAND1 and I

direct KAT7/HBO1 specificity towards H3K14ac, while complexes containing JADE (JADE1, JA

similarity). Although it is able to AMPylate RhoA, Rac and Cdc42 Rho GTPases in vitro, Rho G

/ degrades inherently unstable mRNAs containing AU-rich elements (AREs) within their 3' untra

ransferase (HMT) complexes. However, this function has been questioned (By similarity). Prom

eural progenitors its LINC complex association with SUN1/2 and probable association with cyto

y degrades inherently unstable mRNAs containing AU-rich elements (AREs) within their 3' untra
ome distal) complex, a complex recruited to centromeres which is involved in assembly of kinet

/ by mediating the conversion of monomethylarsonous acid (3+) into the less toxic dimethylarsc

ın mitochondrial S18 proteins are no more closely related to each other than they are to the prc

of tubulin release (PubMed:27306797).

E1-like enzyme) facilitates this reaction by forming an E1-E2 complex with ATG3. Promotes pri

ition (PubMed:19039327). Acts as transcriptional cofactor for nuclear hormone receptors. Inhib

›f the circadian target genes such as PER2 itself or PER1, contributes to the conversion of loca

vage of bubble structures. Interacts with the structure-specific MUS81-EME1 endonuclease and

CAF15) complex, a cullin-4-RING E3 ubiquitin-protein ligase complex that mediates ubiquitinatio

)92333).

ubMed:23085020). In the complex, it mediates the interaction with microtubules (PubMed:1928

ippo pathway by association with LATS1 and modifying LATS1-dependent phosphorylation anc

diates. Exhibits a weak sequence preference for incision between two G residues that reside in

allowing antigen sampling, loading and presentation during T-cell activation. Involved in Arp2/3

n of the target mRNA. The RefSeq represents the predicted microRNA stem-loop. [provided by

n of the target mRNA. The RefSeq represents the predicted microRNA stem-loop. [provided by

fate transport in male germ cells.

chains on RIPK1 and catalyzes the formation of 'Lys-48'-polyubiquitin chains. This leads to RIP
3 molecules bind to IL2R beta (IL2RB) and gamma chain (IL2RG) subunits inducing the tyrosin

nucleotide cycle consumes aspartate and releases fumarate in a manner involving fatty acid ox

through regulation of apoptosis, cell proliferation and cell division (By similarity). Also is require
f PCNA is involved in error-free pathway and employs recombination mechanisms to synthesiz

n of the target mRNA. The RefSeq represents the predicted microRNA stem-loop. [provided by

ays a role in mitotic spindle formation by serving as a physical scaffold to help deliver the RAN

ouse ortholog, not activated by Bacillus anthracis lethal toxin (PubMed:19651869). It is unclear

_. May be a tumor suppressor by mediating apoptosis resistance of cancer cells.

n of the target mRNA. The RefSeq represents the predicted microRNA stem-loop. [provided by

Mediates the responses to cytokines and growth factors in hematopoietic progenitors, platelet[illegible]
[illegible]gulation of cell shape. Promotes Rho-dependent reorganization of the actin cytoskeleton in neu[illegible]

[illegible]PubMed:17409436).

ty).

es which are involved in endocytosis regulation such as
phosphorylates the 5'-terminus of exogenously introduced short interfering RNAs (siRNA

C1 signaling upon genotoxic stress (PubMed:18692468). Has an alkylhydroperoxide reductase

RefSeq, Sep 2009]

polypeptides is regulated
RefSeq, Sep 2009]

ation is also important for a proper regulation of mechanisms such as TGF-beta signaling, cell

polypeptides is regulated

ity, learning and memory (By similarity).

KT1 signaling pathway. Plays a role in the regu

RefSeq, Sep 2009]

e transcription factor GLI2, and hence regulates endochondral ossification (By similarity). Acts

ly of kinetochore proteins, and as a consequence required for progress through mitosis, chrom

23955301). Suppresses chromosome congression in NDC80-depleted cells and co
cell immunity against chlamydia.

ads to release of heterochromatin component CBX5/HP1A, enabling chromatin reor

/ RefSeq, Sep 2009]

TP53-mediated apoptosis. Phosphorylates CHEK2 in response to DNA damage

/ RefSeq, Sep 2009]

slating changes in slit diaphragm str
not to damaged oligonucleotides (PubMed:10734143, PubMed:19609301, PubMed:20649465)
) the intracellular space in cortical neurons.
and regulates cell-cell contact. Phosp

induction through p38 MAPK signaling pathway (PubMed:23935096). In

et gene promoters and activates their transcription (PubMed:28602820).

ıosphorylates RAF1 inhibiting its kinase activity (PubMed:2

GAV:ITGB3 or ITGA6:ITGB4. Its binding to integrins and su

ıodimer to a direct repeat of the core motif spaced by two nuclegotides (Rev

obably implicating recruitment and activation of DNA po

ted current that occludes long term depression. Required for procentri

imilarity). Mediates myocardial contractility

component of MHF and CENP-T-W-S-X complexes, binds DNA and bends it to form a nucleos

rm 5]: Attenuates the ability of transcription factor H

enes and play important roles in embryonic deve

;632057, PubMed:16337594, PubMed:17290220, PubMed:19098711, PubMed:19219073, Pub

adherence to surfaces coated with ICAM-1, a ligand for beta-2 integr

lay play a role in sister chromatid cohesion (PubMed:18172165

‹ the repressor domain are more active than isoform

rotein complex that has the capability to ki

/ RefSeq, Sep 2009]
NF-kappa-B. Linear

ion promotes subsequent targeting of the gamma-tubulin ring complex (gTuRC) to the centrosc
14515). AURKB phosphorylates the CPC complex subunits BIRC5/survivin,
ıdrogenic commitment and suppresses fatty acid oxidation (By similarity). Acts

ty). Positively regulates HAMP transcription

/ RefSeq, Sep 2009]
ent mechanism. Can reduce cell migration by regulating the expressi

›meric enrichment of AUKRB in prometaphase.

›tic chromosomes dispersed (Probable).

ıorylates WWOX on 'Tyr-287'. Phosphorylates MCF2, thereby e

ı non-neuronal cells (PubMed:123995
›Med:11705987, PubMed:16260596, PubMed:12944386). Also functions as the substrate reco

to the nucleus where it modulates the transcription of the TGF-beta-regulated

ıd degradation of the receptor. Activated KIT promotes phosphorylation

›f TRIB3, IL6, IL8, IL23, TNFRSF10B/DR5, PPP1R15A/GADD34,

NGF (PubMed:27445338).

glucose through the PKB/AKT signaling pathway (PubMed:29168081). Also i

ısts is required for phosphorylation of RELA at 'Ser-276' during oxidative stress

/ RefSeq, Sep 2009]
ediating the ubiquitination of target

/ RefSeq, Sep 2009]

e for cancer immunotherapy (By similarity).

/ RefSeq, Sep 2009]

inhibits T cell proliferation and cytokine production through inhi

ormal cytokinesis during mitosis (PubMed:17635935). Required for cortical

/ RefSeq, Sep 2009]

cancer, it has been described as promoter of cell proliferatio

participates in the coordinat

/ RefSeq, Sep 2009]

like FANK1 to regulate apoptosis (PubMed:14765135, PubMed:

ly checkpoint is activated and inhibits the ubiquitin ligase activity of

ators, such as ZNF207, from liquid-unmixed organelles where they a

IOS; anti-proliferative response, such as p53/TP

nown RNA substrate. The complex co

macrophages (By similarity). Macrophage phagocytosis is stimulated by APO

n-specific, single strand-specific nuclease

n transduction and intercellular transmission of polarity information during ti

/ RefSeq, Sep 2009]

›f HSP90AA1/Hsp90: inhibits the ATPase activity of HSP90AA1/Hsp90, leading to activat

to mitotic centromeres (PubMed:21346195). Controls the kinetochore localization o

iphosphate (dITP) as well as guanosine diphosphate (GDP), deoxyguanosine diphosphate (dG

rates with a linoleoyl acyl chain at the sn-2 position but much less effi
during neurite extension. Important for microtubule formation and/or stabiliza

his transition is crucial for mismatch repair. MutS alpha may als
and 11 that defines prostaglandins. The insertion of a second molecule of O2 (bis-oxygenase

angs to form base pairs. It then extends each strand from the base-paired region using the o

/ RefSeq, Sep 2009]

une response via inhibition of AKT1 signaling pathway by forming a complex

effector of p53/TP53-dependent repression by medi
/ RefSeq, Sep 2009]
nage stress, mediates p53-induced inhibition of myocyte mitophagy through ROS

n with STK3/MST2, activates the transcriptional activity of

rade transport, which is essential for subsequent BDNF-trig

TCA-3' preceded by an A/T-rich 5' sequence (RevRE), or as a homodimer to a d

ECAB3 and NPM1. Essential for localization of MAD2L1 t

/ RefSeq, Sep 2009]

[illegible] SQSTM1/p62 and subsequent inactivation of the BCR(KEAP1) complex, leading to NFE2L2/[illegible]

zing hormone (LHB) in the pituitary (By similarity). Regulates t

tivity-dependent synapse elimination in the developin

ie morphogenesis.

, NCL, NUCKS1, NPM1/numatrin, ORC1, PRKAR2A, EEF1E1/p18, EIF3F/p47, p53/TP53, NO

ar phosphatase activity, allowing the hydrolysis and the cellular uptake of these b

.||[Isoform Alpha-C2]: Has transcriptional activation activity.||[Isofo

nembers upon activation; this leads to the pho

l:23230272, PubMed:28525742). In response to DNA damage, PARP9-DTX3L complex is requ

e completion of the chaperoning process, properl

teins CEP290, RPGRIP1L, TMEM67 and B9D2, and of IFT20 foll

e at abasic sites, but does not have any lyase activity, preventing the release of the 5'-de

nd anti-tumor immune response (PubMed:23761635). In the context of bacterial i

cells. This mechanism acts through the growth factor r

:ts of condensation of 5-amino-6-D-ribityaminouracil (5-A-RU) with glyoxal or meth

r of the mTORC1 signaling pathway by promoting, through SLC27A1, th

it the mobility of LTR and non-LT

and ATOH1 which involves ARID1A, indicative for the participation of a distinct SWI

ıg in response to viruses containing a dsDN

ɘgulation of the PI3K-AKT-mTOR pathway, controls cell proliferation,

y via an indirect effect on the expression levels

in tumor development (PubMed:10564580).

osphorylated ssRNAs and dsRNAs but also short dsRNAs (<1 kb in l

osol (PubMed:21947006, PubMed:23258412, PubMed:23707065, PubMed:23722158, PubMed

ass A receptors, like ADRB2, OPRM1, ENDRA

stimulation by promoting STAT6 phosphorylation (PubMed:27796300). Mono-ADP-ribosylates F

ion. Isoform 1 is a more potent viral inhibitor t

Pase-activating protein TBC1D17 during Rab8-mediate

ed for T-cell receptor (TCR) signaling. Mediat

emodeling, positively regulates STAT1-dependent interferon-stimulated gene transcription and

E-cadherin transcription. Acts as a hyp

ligase complexes which mediate the ubiquitination and subsequent proteasomal degradation [illegible]

esponse by activating STAT1, which selectively induc

ıase regulatory subunits by AXL leads to the down
[illegible] inhibitor (I-kappa-B) family. In a convention

pic axis at puberty and in adulthood

endosome. Involved in several receptor-mediated signali

YNE1/KASH1 LINC complex may tether spermatid nuclei to posterior cy
ternative splicing, phosphorylati

ıd inhibits the reorganization of the actin cytoskeleton, formatio

ay act as a negative regulator of lactation, stopping lactation
eft lateral plate mesoderm (LPM). Plays

of the BAF complex, a multiprotein complex involved in transcriptional a

ı and thus its transcriptional activation activity (P
rogression to cytokinesis during mitosis.

'reintegration complex) and export of GAG polyprotein and viral

ve their electrons from NADH (By

ation of osteoclast precursors, macrophage programming, p
ne regulation of mRNA turnover (PubMed:11585831). Cleave

seems to have additional function.

pant in signaling sequences that

n by N-tropic murine leukemia virus (

the ligand-induced RXRA and NR1H3/LXRA transactivation activity (PubMed:19786558). Ma

PubMed:18611980, PubMed:23666920).||Prelamin-A/C can accelerate smooth muscle cell se

X2-mediated prostaglandin E2 (PGE2) expressio

s and LRRTM family members (PubMed:26

y of LTR and non-LTR retrotransp

endoplasmic reticulum and subsequent peptide loadi

nen promotes protein synthesis by phosphorylating PDCD4 and targeting it

on by monocytes (PubMed:11276205). Involved in hematopoiesis and in cardiac ventricular

uscular circuit development. In addition to its role i

ɔugh the transcription of COL1A1, and possibly COL

) pathways, leading to the mobilization of transcription factors tha

ta-cells via its protease inhibitory function (PubMed:

tes IRF8 ubiquitination, which enhanced the ability of IRF8 to stimulate cytokin

karyocytes differentiation.

seem to be, at least in part, contributed by the association of
ı major target for aut

ity). In vitro modulates alpha-3:beta-4-containing nAChR

ranscription activity (PubMed:28065600). Involved in cell cycle regulation by inducing
d:12860994, PubMed:18209100). The ESCRT machinery also func

kinetochore localization of MAD2L1 during the pro

/ RefSeq, Sep 2009]

tor control. Stimulates the binding a

/ RefSeq, Sep 2009]

ri-miRNA are required for its binding (PubMed:15531877, PubMed:15574589, PubMed:155891

ation of histone H3 at 'Lys-36' (H3K36Ac) in the context of nucleosomes (PubMed:3065

BAF complexes which contain ACTL6A/BAF53A and PHF10/BAF45A, a

ning (NHEJ), thereby promoting the homologous recombination (HR)-mediat

RAS and KRAS). Acts downstream of the heterotrimeric G-protein beta/g

biquitin Lys residues, except 'Lys-48'-linked polyubiquitination (Pu

by retina and signals directly to the S

eral membrane of epithelial cells. May increase the amplitude of ASIC

ıd TNFR2) and transcription factor (REL) (By similarity). Inh

/ RefSeq, Sep 2009]

criptional activator activity of JUN (PubMed:28289210).
/ RefSeq, Sep 2009]

clude HOXC8, HOXA9, MYT1, CDKN2A and retinoic acid target genes. EZH2 can also methyla

/ RefSeq, Sep 2009]

d trimethylation of 'Lys-20' of histone H4 (PubMed:28114273). Ma

ınd gamma-globin genes regulation during erythroid differe

e BNC reduces molecular oxygen to 2 water molecules using 4

›y retina and signals directly to the S

; association with nuclear DLG1, thereby causing dissocia

›y retina and signals directly to the S

ɹence 5'-TGACAG-3'in the activator ACT sequence of the D

/ RefSeq, Sep 2009]

independent of ruffling. Involved in th

in the fine-tuning of inhibitory synaptic transmission (PubMed:9844003). Pre-synaptic GABA re

/ RefSeq, Sep 2009]

bMed:9425121, PubMed:10358058, PubMed:17472963). In an alternative pathway of eicosano

, an inhibitor of asparagine-linke

›y retina and signals directly to the S

cancer in multiple d

the replication protein A (RPA) or the cohesion CTF18-replication f

/ RefSeq, Sep 2009]

JLK1 kinase complex to the phagophore which leads to autophagosome formation (PubMe
omplex along with CREST plays a role regulating the activity of gen

/ RefSeq, Sep 2009]

/ RefSeq, Sep 2009]

/ RefSeq, Sep 2009]

2013]

roxylated E1 and E2 at the C15-alpha and C16-alpha positions (PubMed:11555828, PubMed:1

/ RefSeq, Sep 2009]

/ RefSeq, Sep 2009]

/ RefSeq, Sep 2009]

/ RefSeq, Sep 2009]
/ RefSeq, Sep 2009]

y similarity).

PubMed:17596519). Participates in dendritic cell endocytosis

ent manner through activation of Rac1.||Osteostatin is a potent inhib

e BNC reduces molecular oxygen to 2 water molecules unsing 4

ment and can then be subjected to somatic hypermutations whic

y).

yzes the hydrolysis of 1-O-alkyl-2-acetyl-sn-glycero-3-

-dependent transcription through interaction with PKN1
for the formation of the 9-1-1 complex.

35, PubMed:20421648). Also mediates immune tolerance:

ites to intracellular pH regulation in the duodenal

ing axons.

/ RefSeq, Sep 2009]

›Med:26166670). Plays a protective role in early postimplantation emb
is. AP-2 seems to play a role in the recycli

endothelial autophagy independently of its enzymatic activ

chromatin at gene promoter regions
›rogramming adap
n a TP53-dependent manner (PubMed:15102863). Plays a role in granulosa cell apoptosi
PMA) treatment in a p38 MAPK-dependent manner (PubMed:25106868). Positively regulates e

ɔskeleton reorganization essential for cell migration and adhesion (PubMed

ıormal cell cycle progress and normal cytokinesis (PubMed:17495026, Pu
ls to condensation of actin stress fibers into thick bundles. Involv
vage and polyadenylation of pre-mRNAs. Component of the tRNA-sp

lling, motility and migration. Acts as
ıer aspects of development including angiogenes

ve role in airway epithelium, but also shown to compromi
lase activity, exhibiting the ability to hydrolyze the prolyl bond two residues from the N-terminus

nus and therefore lacks several phosphorylation sites. Mediate
it diaphragm (an essential part of the glomeru

ed:27084452). Inhibits CDH1-mediated cell adhesion in process independent from Rho activat

-mediated apoptosis. Phosphorylation of ST13 regulates internalizat
vation of Toll-like receptors (TLRs) an

criptional activity of nuclear hormone rec

expression and increased induced regulatory T (iTreg) cell stabilit

SOX2 (By similarity). Involved in epithelial to mesenchymal transit

breast milk and mucosal secretions

ent, MET signaling plays a role in gastrulation, develop

u, respectively), but with lower affinity than crotonylated histone H3 (PubMed:25417107, PubM

y retina and signals directly to the S

ary fatty acid amide and glyoxylate (By similarity).

ytic cells; PECAM1-mediated detachment signal appears to be disable
uired for the preservation of coc

ct forms of PLTP exist in plasma: an active form that can t

deficiency. Required for the differentiation of

of TNF, interferon alpha and interferon beta (PubMed:

ocyte progenitor cells (By similarity). C-terminal processed forms

cytoplasmic domains. Plays an impo

ınsduction, they regulate the leptin receptor/LEPR in th

ɔf apoptotic cells (PubMed:29162624).

nal activation or repression (PubMed:25849773).||D

involved in cell differentiation, apoptosis, immune response and regulation of K(+)

ion. Regulates endocytosis and cell migration in neutrophils.

PPL1-containing endosomes and in degradative but not recycling trafficking

ing histone-lysine N-methyltransferase EHMT2 recruitment and H3K9 dimethylat

ng RAC1 or RAC2, MAP3K11/MLK3 or MAP3K7/TA

ration and invasion through the extracellular matrix (PubMed

›vided by oxLDL- or amyloid-beta 42-binding to CD36. Thi

ıining ERM proteins (EZR; MSN and RDX) and MYL9 activation through associ
ve splicing and influences mRNA splice site

erve as an adapter for UPF1 to protein phosphatase 2A (PP2A), thereby triggering UPF1 depho

ses LINE-1 retrotransposon activity (PubMed:24035396, PubMed:29610582, PubMed:242173

am ULK1 and PI3 kinases (PubMed:28561066). Once activated, WIPI2 recruits at phagopho

es T-cell responses and the release of cytokines

f inflammasomes is mediated by the pyrin and CARD domains and

nay also play a role in cell proliferation and apoptosis by positively regul

cell death.
are preferentially recognized via a LXXLAP motif.

odeling, promotes the migration of endotheli

fusion events necessary for transport to lysosomes. Induces lysosomal vesicle forma

uitination and degradation of SUFU, allowing the release of GLI1 from SUFU for p

sphorylation of complex I subunit NDUFA10 (By similarity).

ates the inhibition (By similarity).

litional evidences are requi

ı of the kinase. Essential for activated phospho-MLKL to oligomerize and local

9, PubMed:20628055). It is the ceramidase that controls the levels of circulating sphi

scription of specific genes and the

mation of neuromuscular synapses through MUSK, inhibits PDGFR

cing results in multiple transcript

e(s).

euronal adhesion and axonogenesis. In

ere structural integrity in embryonic stem cells which pr

ange of adaptive neuronal responses including long-term potentiation (LTP

ceptor for oligomannosidic glycans. In vitro, promotes outgrowth of astro
virus (EBOV) and marburg virus (M

ure T cell precursors. CBF complexes repress ZBTB7B transcription facto

differentiation by
role in rRNA transcription and/or maturation. Intera

. Intracellular forms interact with ubiquitin and SCF (SKP1-CUL1-F-box protein) E3 ubiquitin-pr

tivity is subject to debate. According to various reports, th

rowth factors, neuropeptides and pe

ntiated CD4(+) T cells and may be involved in induct

nces RPS6KA1 and CREB1 activity and contributes to the regulation of trans

ane domain, such as isoform

erived chemokines and their subsequent presentation to circulating l

similarity). Also promotes epithelial cel

ing to its degradation mediated by ubiquitin/proteasomes. Phosphory

a, the trifunctional enzyme subunit alpha/HADHA also has a monolysocardiolip

nponent of a RCOR/GFI/KDM1A/HDAC complex that suppre

transport of mitochondria in neurons during hypoxia.
in human populations, presents immunodominant viral epitopes derived from IAV M/matrix pro
at position 2 and mainly hydrophobic amino acids Leu, Ile or Val at the C-terminus. Associated

ıethyltransferases. Also acts as a transcriptional repress

ty). Heterodimeric channels containing KCNK1 and KCNK2 have much higher activity, a

0460, PubMed:23633677). In this way LIMK1 regulates several actin-dependent biological pr

ents rapid EGFR endocytosis and prolongs EGFR signaling. Required for

tress by regulating the activity of GSK3beta

a membrane to specific cellular targets (B

held in the cytoplasm in an inactive s

ear sequestration of viral polymerase (P3D-POL) with

osphorylating NFAT and promoting its nuclear translocation (PubMed:26248042). Dephosphor
e lagging and leading strand, respectively (By similarity). In the primase co

horylated growth-suppressive form of RB1 and i

vnstream targets.
ecific low-molecular mass non-enzymic proteins, they part

hereby modulating cell cycle progression and ce

egrin signal transduction, but also in signaling do

d:19615464, PubMed:22605381, PubMed:22825852). Promotes keratinocyte differentia
noglobulin heavy chain class-switch recombination, promotes microhomology-mediated alterna

ls (PubMed:12411430).

irectly on the transcription regulation of several genes by phosphorylating GSK3B at 'Ser-9'

, PubMed:10903140, PubMed:12117417, PubMed:12225859, PubMed:30867591). Required f

neural progenitors exit mitosis and differentiate into neurons, npBAF

the expression of the target genes, inducing a cellular anti

y).

to the nucleus where it modulates the transcription of the TGF-beta-regulated g
ed in its shuttling to the nucleus (By simila

ng RNA p21 (lincRNA-p21) and lincRNA-Mkln1. Lin

ence. Acts synergistically

ılso function as an endocytic receptor. Not re

ıenone and 4-oxo-trans-2-nonenal (in vitro) (By similarity).

of HEPACAM2/MIKI (PubMed:22864114). May also regulate vesicle trafficking and modulate

way mediated by PRKCA (PubMed:16757564, PubMed:2172458

cell proliferation of melanoma cells and promotes their apoptosis in a cAMP-inde

rd T cell lineage commitment. Required

in muscular differentiation (PubMed:12163359). Plays an indispensable

cell death BAD. Alternativel

ormal formation of lamellipodia (By similarity). Required for

nation of membranes and of pulmonary surfactant (By similarity).

:omplex functions (PubMed:24793695).

Med:23708001, PubMed:16921029). Plays a role in genome integrity preservation by coordina

ulation of cardiac muscle cell prolife

ig m6A methylation (PubMed:16129689). Inhibits pre-mRNA splicing (PubMed:2104

oubiquitinated BRCA1 leads to the inhibition of the E3 ubiquiti

As involved in cell proliferation, including cell cyc
ut stimulating already phosphorylated catalyt

activation of canonical Wnt/beta-catenin target genes. Negative regul

ventral lip to the limb buds through the
endent manner (By similarity). Plays also a role in the biogenesis of U3

h its 3'-5' exonuclease activity, which efficiently processes double-stranded D

meshifting (-1PRF). Does not bind to ribosomes.

ɛndosome-to-plasma membrane transport for cargo protein recycling.
ne. Required for the formation of the ATP8A2, ATP8B1 and ATP8B2 P-

after DNA damage (PubMed:26287480). Protects cells again

y retina and signals directly to the S
lgi network and vesicles in the cell periphery; the function i
vesicle extrusion and/or membrane fission activities, possi

triggers apoptosis by activating the JNK1 pathway (By similarity). Phosphorylates mTORC1 co

ners and represses the ligand-induced RXRA/B/G, NR1H3/LXRA, NR1H4 and VDR transactiva

which is different from canonical E2 recog

OCE activity (PubMed:28219928). Involved in B-lymphocyte chemotax

driven cell growth and migration (PubMed:26895380). Protects cells from oxidative stress-induc

neural tube.

key role during gametogenesis (By similarity). Stabilizes MCM9 protein (PubMed:23401855, Pu

ı also amplify Wnt signaling pathway independently of LGR4-6 receptors

cellular glycosylation processes (PubMed:25354954). Required to link T-cell antigen rec

omplex that acts as a cytoplasmic sensor of viral double-stranded RNA (dsRNA) and pl

elated virus (XMRV) and s

wing ATF4-mediated reprogramm

.
/ RefSeq, Sep 2009]

e-1 receptor such as ACVR1B. Once activated, the

Mediates attachment of dendritic cells onto endothelial cells via homotypic in

inding to inactive phosphory
nplex (transcription and export complex 2) subunits PCID2 and SEM1,

ssociation with CBL, required for proper fe

as axonal filopodia growth, stereocilia

olved in the innate immune response to nucleic acids by acting as a promis

ed:25636800, PubMed:27302953). This induces a conformational change, leading to its

anslated regions, and in RNA surveillance pathways, preventing translation
naintenance of epithelia cell-cell adhesion. Potential

occurring during the S phase (PubMed:21659603). Isoform 1 possesses 3'->5' double

· (PubMed:7697716). Plays also a role in base excision repair (BER
is gene. [provided by RefSeq, Mar 2

SLC1A5/ASCT2, SLC38A1/SN1 and SLC6A19, creatine transporter: SLC6A8,

rity). Contributes to glutathione biosynthesis and protection against oxid

n by associating with the dopamine transporter (DAT1) and thereby mo

ponent of the eNoSC (energy-depe

erodimer of TLR4 and TLR6, which is rapidly internalized and triggers inflamma

quired for mtDNA decatenation and segregation after

and hence promotes ARE-mediated mRNA deadenylation (PubMed:23644599). Functions als

d recruits NuA4-related complex to deposit H2AZ1/H2A.Z (PubMed:29437725). H2AZ
5859). When associated with LAPTM4B, the heterodimer formed by SLC3A2 and SLC7A5 is re

P (PubMed:17379599). Involved in the regulation of inflammation, platelets activation,

reaks (DSBs), accumulates at DNA damage sites and promotes homologous recombination r

ulation and phosphorylation by AKT1. Suppresses Fas-induced apoptos

ation. Recruits the E2 ubiquitin-conjugating enzyme CDC34 to the complex and brings it
d proteins: when misfolded proteins are too abundant to be d

l activity, influencing the balan

ecruits the mTORC1 component RPTOR to stress granules (SGs), thereby preventing mTORC1

o fulfill this task, exhibiting near-absence of strand displacement activity
MDFIC and activate HAND1. Phosphorylates

ADE2 and JADE3) scaffold direct KAT7/HBO1 specificity towards histone H4 (PubMed:1918776

TPases do not constitute physiological substrates (PubMed:

anslated regions, and in RNA surveillance pathways, preventing translation

notes ubiquitination of PCNA following UV irradiation and may regulate re

oplasmic dynein-dynactin motor complexes functions to pull the

anslated regions, and in RNA surveillance pathways, preventing translation
ochore proteins, mitotic progression a

onic acid (PubMed:21193388, PubMed:25997655). It however only plays a limited

okaryotic S18 proteins. Pseudogenes correspondin

imary ciliogenesis by removing OFD1 from centriolar satellites via the auto

›its the induction properties of several steroid receptors suc

ıl chromatin to a heterochromatin

d promotes the cleavage of 3'-flap and replication fork-li

on and degradation of target proteins (PubMed:16949367, PubMed:31452512). The DCX(DCA

39083, PubMed:23085020).

d localization of WWTR1/TAZ (PubMed:21555462).

a T-rich region of DNA (PubMed:28049850). Has also e

complex-dependent actin assembly driving Sa

/ RefSeq, Sep 2009]

/ RefSeq, Sep 2009]

'K1 proteasomal degradation and consequently termination of the TNF- or LP
e phosphorylation of both receptor subunits on th

idation and ATP-citrate lyase activity (PubMed:31978345). Participates in pattern

ed for chromosome congression and alignment durin
e across the lesion (PubMed:24695737).

/ RefSeq, Sep 2009]

effector molecule TPX2 to microtubules (PubMed:18591255). May counterac

whether isoform 2 is involved in inflammasome formation. It is not cleaved within the F

/ RefSeq, Sep 2009]

s, erythrocytes, and in mature myeloid cells,
ıronal cells and neurite retraction (PubMed:26091040). Promotes

e acti

cycle progression, DNA repair and cell

also as a transcriptional core

ıosome segregation and cytok

). This feature is prop

ome-like stru

›Med:19837670,

ome, an important step for

gnition module for the DCX (DDB2-CUL4

GDP

activity) yield

NR

NO/p54NRB, PAPOLA, PLEC/plectin, RB1, TPPP,

uired for efficient non-homologou

l:26229117, Pu

PARP

thus STA

of target proteins (PubMed:1473946

nescenc

I61,

eceptor

oid biosynthes

4559847, PubMed:12865317, PubMed:1

early adipogenesis by prom

; of

:ion (PubMed:11976333, PubMed:1

e

›sphorylatio

94). Not able to restrict in

o

tein 1 (GILGFVFTL),
with succe

ylates an

tive end joining (A

or targeting of SLC7A5 and SLC7A8 to the plasma membra

the subcellular

ating DNA replication

mpl

atio

ced a

ubMed

o by recruiting components of the cytop

ecruited to

1 hyper

66, P

\F15) complex act
