# Supplementary material for: Transcriptomic profiling and pathway analysis of cultured human lung microvascular endothelial cells following ionizing radiation exposure
Source: Sci Rep. 2021 Dec 20;11:24214. doi: 10.1038/s41598-021-03636-7 (PMC8688546; doi:10.1038/s41598-021-03636-7)
Supplement: Supplementary file 2 — Supplementary Information 2. [file 41598_2021_3636_MOESM2_ESM.pdf]

EndMT focused heatmaps

EndMT	2 hr	4 hr	6 hr	8 hr	24 hr	Name
PLAU	0.655	0.743	1.531	1.733	4.057	Plasminogen Activator, Urokinase
BMP2	0.568	0.699	0.78	0.88	3.549	Bone Morphogenetic Protein 2
CXCR4	-0.328	0.669	1.207	1.198	3.156	C-X-C Motif Chemokine Receptor 4
PLAT	0.286	0.133	0.307	0.919	3.139	Plasminogen Activator, Tissue Type
SELE	1.765	1.732	0.2	-0.316	2.629	Selectin E
ICAM1	0.116	1.137	0.956	0.963	2.611	Intercellular Adhesion Molecule 1
LAMC2	0.301	0.138	0.436	0.755	2.528	Laminin Subunit Gamma 2
ACTA2	0.589	1.186	1.511	1.996	2.44	Actin Alpha 2, Smooth Muscle
JUP	0.184	0.078	0.302	0.906	2.285	Junction Plakoglobin
CDH11	0.058	0.173	0.157	0.536	2.215	Cadherin 11
SMAD3	0.35	0.642	0.889	0.778	1.727	SMAD Family Member 3
ETS1	0.681	0.683	0.691	0.738	1.723	ETS Proto-Oncogene 1, Transcription Factor
NID2	0.549	0.329	0.038	0.325	1.658	Nidogen 2
SOX7	0.015	0.482	0.322	0.495	1.643	SRY-Box Transcription Factor 7
MMP14	0.146	0.245	0.376	0.702	1.439	Matrix Metallopeptidase 14
ZNF703	0.299	-0.096	0.079	0.434	1.358	Zinc Finger Protein 703
DAB2	0.268	0.172	0.338	0.612	1.317	DAB Adaptor Protein 2
CXADR	-0.415	-0.526	-0.075	-0.033	1.303	CXADR Ig-Like Cell Adhesion Molecule
NT5E	0.158	0.006	0.086	0.525	1.245	5'-Nucleotidase Ecto
FAP	0.212	-0.016	0.023	0.274	1.235	Fibroblast Activation Protein Alpha
CDH2	0.466	0.151	-0.159	0.274	1.23	Cadherin 2
FZD8	0.611	-0.308	-0.472	-0.175	1.151	Frizzled Class Receptor 8
CDH5	0.296	0.123	0.162	0.555	1.134	Cadherin 5
PECAM1	0.173	-0.033	-0.028	0.422	1.117	Platelet And Endothelial Cell Adhesion Molecule 1
VIM	0.342	0.131	0.061	0.442	1.098	Vimentin
RGCC	0.21	-0.079	0.149	-0.071	1.078	Regulator Of Cell Cycle
CAV1	-0.221	0.049	0.249	0.554	1.076	Caveolin 1
CLDN5	-0.447	-0.406	-0.143	0.22	1.07	Claudin 5
TWIST2	0.002	-0.308	-0.21	0.097	0.991	Twist Family BHLH Transcription Factor 2
COL5A2	0.165	0.155	0.097	0.521	0.914	Collagen Type V Alpha 2 Chain
SOX18	0.578	-0.377	-0.394	-0.286	0.904	SRY-Box Transcription Factor 18
DDR2	-0.259	-0.089	0.141	0.004	0.877	Discoidin Domain Receptor Tyrosine Kinase 2
BCL9L	0.22	-0.204	0.229	0.47	0.817	BCL9 Like
CD44	0.327	0.121	-0.009	0.348	0.817	CD44 Molecule (Indian Blood Group)
ICAM2	-0.259	-0.171	0.195	0.628	0.803	Intercellular Adhesion Molecule 2
SPOCK1	0.332	0.159	-0.02	0.379	0.801	SPARC (Osteonectin), Cwcv And Kazal Like Domains Proteoglycan 1
CD34	-0.042	0.042	0.015	0.261	0.73	CD34 Molecule
PRKCA	0.484	0.01	0.109	0.498	0.695	Protein Kinase C Alpha
PTK2	0.209	-0.065	0.171	0.521	0.688	Protein Tyrosine Kinase 2
PLAUR	0.124	-0.07	0.018	0.291	0.634	Plasminogen Activator, Urokinase Receptor
DLL4	-1.421	-0.079	-0.042	0.022	0.448	Delta Like Canonical Notch Ligand 4
TGFBR1	0.838	0.422	0.084	0.017	0.349	Transforming Growth Factor Beta Receptor 1
SNAI1	0.589	0.052	0.166	0.33	0.156	Snail Family Transcriptional Repressor 1
PIK3R3	0.795	-0.05	-0.054	0.308	0.147	Phosphoinositide-3-Kinase Regulatory Subunit 3
HEY1	1.199	-0.237	-0.721	-0.516	-0.154	Hes Related Family BHLH Transcription Factor With YRPW Motif 1
FGF2	1.11	0.858	0.306	0.435	-0.171	Fibroblast Growth Factor 2
GATA2	0.703	-0.471	-0.245	-0.038	-0.682	GATA Binding Protein 2
CTGF	0.08	0.015	-0.302	-0.351	-0.983	Cellular Communication Network Factor 2
POSTN	0.212	0.188	-0.047	0.307	-1.011	Periostin
TAGLN	0.268	0.042	0.149	-0.09	-1.029	Transgelin
EGFL7	-0.533	-0.363	-0.366	-0.746	-1.174	EGF Like Domain Multiple 7
FLT1	0.478	-0.075	-0.503	-0.318	-1.467	Fms Related Receptor Tyrosine Kinase 1
HEY2	1.175	-0.446	-0.329	0.153	-1.524	Hes Related Family BHLH Transcription Factor With YRPW Motif 2
BMP4	-0.653	-0.086	-0.581	-0.489	-2.128	Bone Morphogenetic Protein 4
IGFBP3	-0.076	-0.008	-0.263	-0.119	-3.927	Insulin Like Growth Factor Binding Protein 3
IGFBP1	-0.049	-0.109	-0.561	-0.532	-4.25	Insulin Like Growth Factor Binding Protein 1

Supplemental Table 2: Data used for the generation of focused heatmaps

Cell cycle heatmaps

**Cell cycle**	**2 hr**	**4 hr**	**6 hr**	**8 hr**	**24 hr**	**Name**
CDKN2B	0.673	0.852	0.679	0.885	2.224	Cyclin Dependent Kinase Inhibitor 2B
CCND2	-0.489	0.35	0.967	0.829	2.169	Cyclin D2
CDKN1A	2.376	2.121	1.81	1.717	2.078	Cyclin Dependent Kinase Inhibitor 1A
MDM2	2.096	1.75	1.922	1.397	1.909	MDM2 Proto-Oncogene
GADD45A	1.233	1.076	1.153	0.88	1.778	Growth Arrest And DNA Damage Inducible Alpha
SMAD3	0.35	0.642	0.889	0.778	1.727	SMAD Family Member 3
CCNA1	-0.14	-0.322	-0.009	0.526	0.932	Cyclin A1
CCND1	0.182	0.036	0.269	0.607	0.925	Cyclin D1
GSK3B	0.301	0.177	0.169	0.422	0.762	Glycogen Synthase Kinase 3 Beta
TFDP2	0.182	0.011	-0.198	0.043	0.655	Transcription Factor Dp-2
HDAC1	0.241	-0.041	-0.027	0.089	0.612	Histone Deacetylase 1
CDK6	0.442	0.3	0.078	-0.058	0.605	Cyclin Dependent Kinase 6
ABL1	0.452	-0.128	0.075	0.424	0.59	ABL Proto-Oncogene 1, Non-Receptor Tyrosine Kinase
YWHAQ	0.278	0.103	-0.003	0.28	0.446	Tyrosine 3-Monooxygenase/Tryptophan 5-Monooxygenase Activation Protein Theta
RBL2	-0.215	-0.142	0.09	0.169	0.443	RB Transcriptional Corepressor Like 2
PTTG1	-0.427	-0.578	-0.465	-0.255	0.147	PTTG1 Regulator Of Sister Chromatid Separation, Securin
CCNB1	-0.946	-1.877	-1.593	-0.76	-0.194	Cyclin B1
BUB1	-0.659	-1.189	-1.047	-0.361	-0.228	BUB1 Mitotic Checkpoint Serine/Threonine Kinase
PCNA	0.474	0.599	0.35	-0.004	-0.305	Proliferating Cell Nuclear Antigen
RBL1	0.429	0.244	0.032	0.082	-0.307	RB Transcriptional Corepressor Like 1
CCNB2	-0.564	-1.02	-1.08	-0.587	-0.315	Cyclin B2
TTK	-0.795	-0.904	-0.851	-0.191	-0.319	TTK Protein Kinase
CDC14A	-0.01	0.315	-0.54	-0.833	-0.401	Cell Division Cycle 14A
CDC25B	-0.455	-0.642	-0.403	-0.207	-0.433	Cell Division Cycle 25B
MCM7	0.471	0.088	-0.221	-0.182	-0.443	Minichromosome Maintenance Complex Component 7
CCNH	-0.108	-0.048	-0.111	0.008	-0.469	Cyclin H
MCM3	0.445	-0.035	-0.519	-0.464	-0.506	Minichromosome Maintenance Complex Component 3
CDK2	0.229	0.169	-0.208	-0.292	-0.532	Cyclin Dependent Kinase 2
E2F4	0.194	-0.21	-0.217	-0.217	-0.534	E2F Transcription Factor 4
BUB1B	-0.606	-0.783	-0.771	-0.304	-0.575	BUB1 Mitotic Checkpoint Serine/Threonine Kinase B
WEE1	-0.493	-0.498	-0.554	-0.643	-0.618	WEE1 G2 Checkpoint Kinase
MCM4	0.538	0.034	-0.495	-0.496	-0.635	Minichromosome Maintenance Complex Component 4
PLK1	-1.146	-2.204	-1.641	-0.69	-0.644	Polo Like Kinase 1
CDC20	-0.911	-1.817	-1.549	-0.721	-0.673	Cell Division Cycle 20
CDK1	-0.58	-0.475	-0.509	-0.371	-0.709	Cyclin Dependent Kinase 1
CDKN1B	-0.677	-0.178	-0.237	-0.16	-0.719	Cyclin Dependent Kinase Inhibitor 1B
CCNA2	-0.504	-0.78	-0.736	-0.267	-0.755	Cyclin A2
MCM6	0.583	0.067	-0.575	-0.527	-0.765	Minichromosome Maintenance Complex Component 6
MCM5	0.449	0.034	-0.409	-0.269	-0.797	Minichromosome Maintenance Complex Component 5
SKP2	-0.319	-0.282	-0.331	-0.302	-0.812	S-Phase Kinase Associated Protein 2
CDC25A	0.825	-0.142	-0.686	-0.819	-0.874	Cell Division Cycle 25A
DBF4	-0.809	-0.754	-0.654	-0.706	-1.007	DBF4 Zinc Finger
ORC6	0.22	-0.2	-0.572	-0.876	-1.053	Origin Recognition Complex Subunit 6
CDC6	0.76	0.119	-0.487	-0.553	-1.115	Cell Division Cycle 6
PKMYT1	0.144	0.15	-0.198	-0.356	-1.116	Protein Kinase, Membrane Associated Tyrosine/Threonine 1
CDC45	0.187	0.107	-0.297	-0.526	-1.255	Cell Division Cycle 45
CDC7	-0.231	-0.2	-0.22	-0.412	-1.261	Cell Division Cycle 7
CCNE1	0.606	-0.068	-0.611	-1.211	-1.268	Cyclin E1
CDKN2D	-0.131	-0.501	-0.056	0.151	-1.294	Cyclin Dependent Kinase Inhibitor 2D
CDKN2C	-0.494	-0.742	-0.661	-0.237	-1.402	Cyclin Dependent Kinase Inhibitor 2C
ORC1	0.767	0.053	-0.699	-0.621	-1.408	Origin Recognition Complex Subunit 1
ESPL1	-0.963	-0.673	-0.507	-0.599	-1.448	Extra Spindle Pole Bodies Like 1, Separase
E2F1	0.526	0.049	-0.852	-1.497	-1.559	E2F Transcription Factor 1
E2F2	0.741	-0.278	-0.795	-1.422	-1.942	E2F Transcription Factor 2
CCNE2	0.858	0.194	-0.632	-1.459	-1.955	Cyclin E2

Apoptosis focused heatmaps

**Apoptosis**	**2 hr**	**4 hr**	**6 hr**	**8 hr**	**24 hr**	**Name**
APAF1	0.385	0.292	0.25	0.253	0.704	Apoptotic Peptidase Activating Factor 1
BAX	0.229	0.339	0.427	0.561	0.477	BCL2 Associated X, Apoptosis Regulator
BBC3	2.09	1.701	1.827	1.627	2.389	BCL2 Binding Component 3
BCL2A1	-0.106	-0.257	0.089	0.339	1.801	BCL2 Related Protein A1
BCL2L1	0.49	0.528	0.68	1.035	1.091	BCL2 Like 1
BCL2L11	0.161	0.661	0.383	0.283	2.781	BCL2 Like 11
BIRC3	0.633	2.139	1.267	0.358	2.021	Baculoviral IAP Repeat Containing 3
BIRC5	-0.528	-0.629	-0.731	-0.558	-0.465	Baculoviral IAP Repeat Containing 5
BNIP3L	0.211	0.322	0.589	0.994	1.679	BCL2 Interacting Protein 3 Like
BOK	0.194	0.013	0.223	0.45	0.971	BCL2 Family Apoptosis Regulator BOK
CAPN2	0.179	0.065	0.218	0.549	1.275	Calpain 2
CASP1	-0.067	0.361	0.956	1.02	2.191	Caspase 1
CASP10	-0.109	0.349	0.312	0.334	0.577	Caspase 10
CASP2	0.422	-0.032	-0.217	-0.069	-0.066	Caspase 2
CASP3	0.098	0.271	0.344	0.542	0.909	Caspase 3
CASP6	0.081	-0.071	-0.07	0.248	0.687	Caspase 6
CASP7	-0.045	0.311	0.608	0.652	1.356	Caspase 7
CTSB	0.246	0.054	0.004	0.405	0.971	Cathepsin B
CTSF	-0.063	-0.001	-0.077	0.067	0.745	Cathepsin F
CTSK	0.175	0.331	0.277	-0.022	1.581	Cathepsin K
CTSO	0.271	0.342	0.219	0.537	1.147	Cathepsin O
CTSS	0.063	0.339	0.41	0.72	2.041	Cathepsin S
CTSZ	0.222	0.065	0.014	0.463	0.755	Cathepsin Z
DAB2IP	0.55	-0.14	-0.063	0.09	0.222	DAB2 Interacting Protein
ERN1	-0.352	0.484	0.365	-0.057	0.072	Endoplasmic Reticulum To Nucleus Signaling 1
FAS	0.871	1.51	1.348	0.91	1.263	Fas Cell Surface Death Receptor
GADD45A	1.233	1.076	1.153	0.88	1.778	Growth Arrest And DNA Damage Inducible Alpha
HELLS	0.074	0.357	-0.262	-0.368	-0.982	Helicase, Lymphoid Specific
IKBKB	-0.246	0.115	0.393	0.069	0.376	Inhibitor Of Nuclear Factor Kappa B Kinase Subunit Beta
IRF1	0.798	1.008	0.237	0.132	1.986	Interferon Regulatory Factor 1
IRF6	0.73	-0.035	-0.809	-0.98	-2.681	Interferon Regulatory Factor 6
ITPR3	-0.115	-0.192	0.305	-0.011	0.712	Inositol 1,4,5-Trisphosphate Receptor Type 3
JUN	-1.228	0.261	0.284	0.323	1.119	Jun Proto-Oncogene, AP-1 Transcription Factor Subunit
MAP3K5	0.412	-0.123	-0.204	0.073	0.009	Mitogen-Activated Protein Kinase Kinase Kinase 5
MDM2	2.096	1.75	1.922	1.397	1.909	MDM2 Proto-Oncogene
NFKBIA	1.116	0.659	0.216	0.081	0.853	NFKB Inhibitor Alpha
NFKBIE	0.458	0.669	0.246	0.033	0.738	NFKB Inhibitor Epsilon
PARP3	-0.116	0.155	0.246	0.119	0.546	Poly(ADP-Ribose) Polymerase Family Member 3
PARP4	0.233	-0.003	0.207	0.588	0.924	Poly(ADP-Ribose) Polymerase Family Member 4
PIDD1	-0.072	0.509	0.455	-0.274	-0.081	P53-Induced Death Domain Protein 1
PIK3CD	-0.223	0.604	0.742	0.654	1.672	Phosphatidylinositol-4,5-Bisphosphate 3-Kinase Catalytic Subunit Delta
PIK3R3	0.795	-0.05	-0.054	0.308	0.147	Phosphoinositide-3-Kinase Regulatory Subunit 3
PMAIP1	0.575	0.715	0.526	0.14	1.243	Phorbol-12-Myristate-13-Acetate-Induced Protein 1
RELB	0.213	1.85	1.101	0.575	1.811	RELB Proto-Oncogene, NF-KB Subunit
TNFRSF10B	0.268	0.646	0.424	0.098	0.79	TNF Receptor Superfamily Member 10b
TNFRSF10C	0.313	1.014	1.167	1.334	1.729	TNF Receptor Superfamily Member 10c
TNFRSF1B	0.105	-0.373	-0.357	-0.217	-1.109	TNF Receptor Superfamily Member 1B
TNFRSF21	0.384	0.236	0.274	0.495	1.893	TNF Receptor Superfamily Member 21
TNFSF10	-0.457	-0.028	0.174	0.223	2.081	TNF Superfamily Member 10
TP53	0.15	0.235	0.221	0.448	0.695	Tumor Protein P53
TRAF1	-0.272	1.614	1.3	0.337	2.844	TNF Receptor Associated Factor 1
TRAF3	0.167	0.429	0.202	0.116	0.686	TNF Receptor Associated Factor 3
TUBA1A	0.18	-0.151	-0.214	0.164	0.686	Tubulin Alpha 1a

DNA Damage focused heatmaps

DNA damage	2 hr	4 hr	6 hr	8 hr	24 hr	Name
ABL1	0.452	-0.128	0.075	0.424	0.59	ABL Proto-Oncogene 1, Non-Receptor Tyrosine Kinase
APAF1	0.385	0.292	0.25	0.253	0.704	Apoptotic Peptidase Activating Factor 1
ATRIP	-0.064	0.133	-0.112	-0.277	-0.86	ATR Interacting Protein
BAX	0.229	0.339	0.427	0.561	0.477	BCL2 Associated X, Apoptosis Regulator
BBC3	2.09	1.701	1.827	1.627	2.389	BCL2 Binding Component 3
BRCA1	0.028	0.14	0.021	0.027	-0.579	BRCA1 DNA Repair Associated
BRCA2	0.082	0.052	-0.186	-0.309	-0.823	BRCA2 DNA repair associated
CASP3	0.098	0.271	0.344	0.542	0.909	Caspase 3
CCNB1	-0.946	-1.877	-1.593	-0.76	-0.194	Cyclin B1
CCNB2	-0.564	-1.02	-1.08	-0.587	-0.315	Cyclin B2
CCND1	0.182	0.036	0.269	0.607	0.925	Cyclin D1
CCND2	-0.489	0.35	0.967	0.829	2.169	Cyclin D2
CCNE1	0.606	-0.068	-0.611	-1.211	-1.268	Cyclin E1
CCNE2	0.858	0.194	-0.632	-1.459	-1.955	Cyclin E2
CDC25A	0.825	-0.142	-0.686	-0.819	-0.874	Cell Division Cycle 25A
CDK1	-0.58	-0.475	-0.509	-0.371	-0.709	Cyclin Dependent Kinase 1
CDK2	0.229	0.169	-0.208	-0.292	-0.532	Cyclin Dependent Kinase 2
CDK6	0.442	0.3	0.078	-0.058	0.605	Cyclin Dependent Kinase 6
CDKN1A	2.376	2.121	1.81	1.717	2.078	Cyclin Dependent Kinase Inhibitor 1A
CDKN1B	-0.677	-0.178	-0.237	-0.16	-0.719	Cyclin Dependent Kinase Inhibitor 1B
DDB1	0.463	0.259	0.231	0.648	0.635	Damage Specific DNA Binding Protein 1
DDB2	0.652	1.191	1.211	1.142	1.125	Damage Specific DNA Binding Protein 2
E2F1	0.526	0.049	-0.852	-1.497	-1.559	E2F Transcription Factor 1
ERCC6	-0.397	-0.15	-0.389	-0.599	-1.726	ERCC Excision Repair 6, Chromatin Remodeling Factor
FANCD2	-0.135	0.114	0.084	-0.014	-0.573	FA Complementation Group D2
FANCA	-0.387	0.035	-0.073	-0.766	-1.489	FA Complementation Group A
FANCB	-0.122	-0.093	-0.481	-0.77	-1.134	FA Complementation Group B
FANCE	-0.069	-0.247	-0.371	-0.569	-1.044	FA Complementation Group E
FANCF	-0.555	-0.014	0.164	0.405	0.551	FA Complementation Group F
FANCG	-0.08	-0.08	-0.213	-0.408	-0.943	FA Complementation Group G
FANCI	0.084	0.21	0.135	0.229	-0.602	FA Complementation Group I
FANCL	-0.222	-0.007	-0.128	-0.277	-0.338	FA Complementation Group L
FANCM	-0.19	-0.157	-0.382	-0.341	-0.53	FA Complementation Group M
FAS	0.871	1.51	1.348	0.91	1.263	Fas Cell Surface Death Receptor
GADD45A	1.233	1.076	1.153	0.88	1.778	Growth Arrest And DNA Damage Inducible Alpha
H2AFX	-0.526	-0.741	-0.723	-0.366	-0.877	H2A.X Variant Histone
MDM2	2.096	1.75	1.922	1.397	1.909	MDM2 Proto-Oncogene
PIDD1	-0.072	0.509	0.455	-0.274	-0.081	P53-Induced Death Domain Protein 1
PMAIP1	0.575	0.715	0.526	0.14	1.243	Phorbol-12-Myristate-13-Acetate-Induced Protein 1
PML	-0.006	0.198	0.288	0.391	0.86	PML Nuclear Body Scaffold
RAD1	0.078	-0.003	-0.23	-0.342	-0.658	RAD1 Checkpoint DNA Exonuclease
RAD51	0.573	0.08	-0.372	-0.194	-0.586	RAD51 Recombinase
RAD52	-0.9	0.006	0.162	-0.537	-0.59	RAD52 Homolog, DNA Repair Protein
RAD9A	-0.424	-0.21	-0.281	-1.161	-1.309	RAD9 Checkpoint Clamp Component A
RPA2	0.199	0.025	-0.217	-0.114	-0.448	Replication Protein A2
RRM2B	0.606	1.02	1.008	1.006	1.278	Ribonucleotide Reductase Regulatory TP53 Inducible Subunit M2B
TP53	0.15	0.235	0.221	0.448	0.695	Tumor Protein P53
SESN1	1.545	1.359	1.31	1.108	1.605	Sestrin 1
TNFRSF10B	0.268	0.646	0.424	0.098	0.79	TNF Receptor Superfamily Member 10b

Senescence focused heatmaps

Senescence	2 hr	4 hr	6 hr	8 hr	24 hr	Name
IL1A	0.258	0.519	1.256	1.02	3.087	Interleukin 1 Alpha
NFATC2	0.762	0.812	1.642	1.81	2.853	Nuclear Factor Of Activated T Cells 2
CDKN2B	0.673	0.852	0.679	0.885	2.224	Cyclin Dependent Kinase Inhibitor 2B
CCND2	-0.489	0.35	0.967	0.829	2.169	Cyclin D2
CDKN1A	2.376	2.121	1.81	1.717	2.078	Cyclin Dependent Kinase Inhibitor 1A
MDM2	2.096	1.75	1.922	1.397	1.909	MDM2 Proto-Oncogene
GADD45A	1.233	1.076	1.153	0.88	1.778	Growth Arrest And DNA Damage Inducible Alpha
SMAD3	0.35	0.642	0.889	0.778	1.727	SMAD Family Member 3
ETS1	0.681	0.683	0.691	0.738	1.723	ETS Proto-Oncogene 1, Transcription Factor
PIK3CD	-0.223	0.604	0.742	0.654	1.672	Phosphatidylinositol-4,5-Bisphosphate 3-Kinase Catalytic Subunit Delta
CAPN2	0.179	0.065	0.218	0.549	1.275	Calpain 2
TRAF3IP2	-0.336	-0.164	0.344	0.873	1.162	TRAF3 Interacting Protein 2
SQSTM1	0.163	0.662	0.472	0.484	1.064	Sequestosome 1
MAP2K3	0.474	0.289	0.361	0.606	1.049	Mitogen-Activated Protein Kinase Kinase 3
CCNA1	-0.14	-0.322	-0.009	0.526	0.932	Cyclin A1
CCND1	0.182	0.036	0.269	0.607	0.925	Cyclin D1
FOXO1	0.586	0.567	0.406	0.49	0.888	Forkhead Box O1
TGFBR2	-0.134	-0.081	0.064	0.292	0.815	Transforming Growth Factor Beta Receptor 2
HLA-A	0.24	0.136	0.034	0.398	0.799	Major Histocompatibility Complex, Class I, A
PPP3CA	0.244	-0.113	-0.152	0.092	0.781	Protein Phosphatase 3 Catalytic Subunit Alpha
ITPR3	-0.115	-0.192	0.305	-0.011	0.712	Inositol 1,4,5-Trisphosphate Receptor Type 3
ZFP36L2	-0.183	0.393	0.014	-0.018	0.7	ZFP36 Ring Finger Protein Like 2
CDK6	0.442	0.3	0.078	-0.058	0.605	Cyclin Dependent Kinase 6
PTEN	0.157	0.241	0.099	0.303	0.6	Phosphatase And Tensin Homolog
RBL2	-0.215	-0.142	0.09	0.169	0.443	RB Transcriptional Corepressor Like 2
PPP1CB	0.227	0.117	0.125	0.36	0.44	Protein Phosphatase 1 Catalytic Subunit Beta
ZFP36L1	-0.386	0.272	0.159	0.134	0.379	ZFP36 Ring Finger Protein Like 1
TGFBR1	0.838	0.422	0.084	0.017	0.349	Transforming Growth Factor Beta Receptor 1
PIK3R3	0.795	-0.05	-0.054	0.308	0.147	Phosphoinositide-3-Kinase Regulatory Subunit 3
SIRT1	0.708	0.084	-0.089	0.055	0.058	Sirtuin 1
CCNB1	-0.946	-1.877	-1.593	-0.76	-0.194	Cyclin B1
LIN54	0.41	0.053	-0.195	-0.052	-0.295	Lin-54 DREAM MuvB Core Complex Component
RBL1	0.429	0.244	0.032	0.082	-0.307	RB Transcriptional Corepressor Like 1
CCNB2	-0.564	-1.02	-1.08	-0.587	-0.315	Cyclin B2
MAPK11	-0.242	0.172	0.156	-0.327	-0.329	Mitogen-Activated Protein Kinase 11
MAPK14	0.147	-0.232	-0.432	-0.257	-0.446	Mitogen-Activated Protein Kinase 14
CDK2	0.229	0.169	-0.208	-0.292	-0.532	Cyclin Dependent Kinase 2
E2F4	0.194	-0.21	-0.217	-0.217	-0.534	E2F Transcription Factor 4
RAD1	0.078	-0.003	-0.23	-0.342	-0.658	RAD1 Checkpoint DNA Exonuclease
CDK1	-0.58	-0.475	-0.509	-0.371	-0.709	Cyclin Dependent Kinase 1
LIN52	-0.1	-0.239	-0.467	-0.45	-0.744	Lin-52 DREAM MuvB Core Complex Component
CCNA2	-0.504	-0.78	-0.736	-0.267	-0.755	Cyclin A2
MAPK12	-0.209	-0.107	-0.207	-0.7	-0.784	Mitogen-Activated Protein Kinase 12
CDC25A	0.825	-0.142	-0.686	-0.819	-0.874	Cell Division Cycle 25A
CCNE1	0.606	-0.068	-0.611	-1.211	-1.268	Cyclin E1
RAD9A	-0.424	-0.21	-0.281	-1.161	-1.309	RAD9 Checkpoint Clamp Component A
E2F1	0.526	0.049	-0.852	-1.497	-1.559	E2F Transcription Factor 1
E2F2	0.741	-0.278	-0.795	-1.422	-1.942	E2F Transcription Factor 2
CCNE2	0.858	0.194	-0.632	-1.459	-1.955	Cyclin E2
IGFBP3	-0.076	-0.008	-0.263	-0.119	-3.927	Insulin Like Growth Factor Binding Protein 3

Inflammatory response focused heatmaps

Inflammator	2 hr	4 hr	6 hr	8 hr	24 hr	Name
TNFSF15	-0.336	0.56	1.691	2.056	5.46	TNF Superfamily Member 15
LTB	-0.335	-0.08	0.314	-0.081	4.73	Lymphotoxin Beta
TNFRSF4	-0.87	0.619	1.209	0.905	3.413	TNF Receptor Superfamily Member 4
CXCR4	-0.328	0.669	1.207	1.198	3.156	C-X-C Motif Chemokine Receptor 4
IL1A	0.258	0.519	1.256	1.02	3.087	Interleukin 1 Alpha
FGF16	-0.12	-0.09	0.293	0.444	2.89	Fibroblast Growth Factor 16
LGALS9	0.146	0.208	0.164	0.546	2.621	Galectin 9
CCL2	-0.31	1.245	0.886	0.285	2.573	C-C Motif Chemokine Ligand 2
CXCL5	0.026	0.853	0.856	0.919	2.548	C-X-C Motif Chemokine Ligand 5
IL32	0.221	0.52	0.757	0.823	2.378	Interleukin 32
SDC1	0.418	1.19	1.271	1.696	2.248	Syndecan 1
IL4I1	-0.032	0.639	0.856	1.265	2.175	Interleukin 4 Induced 1
UBE2L6	0.151	0.088	0.325	0.744	2.148	Ubiquitin Conjugating Enzyme E2 L6
TNFSF10	-0.457	-0.028	0.174	0.223	2.081	TNF Superfamily Member 10
LIF	1.374	1.709	1.827	0.972	2.039	LIF Interleukin 6 Family Cytokine
BIRC3	0.633	2.139	1.267	0.358	2.021	Baculoviral IAP Repeat Containing 3
TNFSF9	2.12	2.236	1.847	1.801	1.995	TNF Superfamily Member 9
PSMB9	0.065	0.433	0.738	1.047	1.989	Proteasome 20S Subunit Beta 9
CSF1	0.517	1.239	0.384	-0.155	1.945	Colony Stimulating Factor 1
TNFRSF21	0.384	0.236	0.274	0.495	1.893	TNF Receptor Superfamily Member 21
TNFSF4	-0.075	0.605	0.874	0.867	1.852	TNF Superfamily Member 4
IRAK2	0.414	0.909	0.684	0.616	1.847	Interleukin 1 Receptor Associated Kinase 2
TNFRSF10C	0.313	1.014	1.167	1.334	1.729	TNF Receptor Superfamily Member 10c
IL12A	0.544	1.478	1.564	0.608	1.6	Interleukin 12A
IL15	-0.049	0.746	0.866	0.542	1.459	Interleukin 15
UBA7	-0.206	0.146	0.505	0.205	1.343	Ubiquitin Like Modifier Activating Enzyme 7
KSR2	-0.12	-0.429	0.466	0.455	1.32	Kinase Suppressor Of Ras 2
TNFSF18	-0.978	0.252	0.425	0.086	1.224	TNF Superfamily Member 18
MMP1	0.188	0.008	-0.019	0.382	1.212	Matrix Metallopeptidase 1
TNFRSF14	-0.287	0.599	1.079	0.845	1.149	TNF Receptor Superfamily Member 14
VIM	0.342	0.131	0.061	0.442	1.098	Vimentin
SQSTM1	0.163	0.662	0.472	0.484	1.064	Sequestosome 1
RALA	0.354	-0.169	-0.301	-0.111	1.055	RAS Like Proto-Oncogene A
CXCL3	0.966	0.711	-0.194	-0.468	0.981	C-X-C Motif Chemokine Ligand 3
GBP3	-0.246	0.092	0.457	0.628	0.973	Guanylate Binding Protein 3
CSF3	-1.789	-0.501	0.25	-0.337	0.392	Colony Stimulating Factor 3
FGF2	1.11	0.858	0.306	0.435	-0.171	Fibroblast Growth Factor 2
CXCL8	-0.937	0.012	-0.628	-1.346	-0.567	C-X-C Motif Chemokine Ligand 8
IL11	1.145	0.329	-0.282	-0.618	-0.61	Interleukin 11
TNFRSF11A	0.151	-0.005	-0.228	-0.326	-1.091	TNF Receptor Superfamily Member 11a
TNFRSF1B	0.105	-0.373	-0.357	-0.217	-1.109	TNF Receptor Superfamily Member 1B
IL21R	1.534	0.253	-0.048	0.011	-1.148	Interleukin 21 Receptor
PTGS2	2.054	0.236	-0.45	-0.748	-1.219	Prostaglandin-Endoperoxide Synthase 2
IL18	-0.09	0.102	-0.466	-0.288	-1.287	Interleukin 18
RPS6KA5	0.836	-0.031	-0.089	-0.295	-1.319	Ribosomal Protein S6 Kinase A5
EGR1	-0.777	-0.185	-1.158	-1.788	-1.733	Early Growth Response 1
MAOA	0.196	-0.101	-0.418	-0.229	-2.023	Monoamine Oxidase A
IL1RL1	0.252	0.076	-0.4	-0.477	-2.633	Interleukin 1 Receptor Like 1
IRF6	0.73	-0.035	-0.809	-0.98	-2.681	Interferon Regulatory Factor 6
CXCL12	0.749	0.03	-0.782	-1	-3.244	C-X-C Motif Chemokine Ligand 12
